# Interventional Endoscopic Ultrasound in Gastroenterology: A Comprehensive Bibliometric Analysis (2001–2024)

**DOI:** 10.3390/clinpract16080153

**Published:** 2026-08-20

**Authors:** Koji Takahashi, Noa Minami, Kohei Horie, Taiga Sudo, Nana Yamada, Terunao Iwanaga, Takafumi Sakuma, Hidehiro Kamezaki

**Affiliations:** 1 Department of Gastroenterology, Eastern Chiba Medical Center, 3–6–2 Okayamadai, Togane 283-8686, Japan; 2 Department of General Medical Science, Graduate School of Medicine, Chiba University, 1–8–1 Inohana, Chiba 260-8670, Japan

**Keywords:** interventional endoscopic ultrasound, therapeutic EUS, EUS-guided biliary drainage, lumen-apposing metal stent, bibliometric analysis

## Abstract

**Background/Objectives**: Interventional endoscopic ultrasound (I-EUS) has evolved from a diagnostic imaging modality into a transformative therapeutic platform encompassing biliary drainage, pancreatic interventions, luminal bypass, pain management, and ablative therapies. Despite the rapid proliferation of I-EUS research, a comprehensive bibliometric analysis characterizing the global intellectual architecture of this field is lacking. **Methods**: A Web of Science Core Collection search identified 4340 records, of which 2237 were included (2001–2024), analyzed with R bibliometrix (version 5.0) and VOSviewer (version 1.6.20). **Results**: Publication output demonstrated three distinct phases with pronounced acceleration from 2017. The United States led global output (*n* = 625, 27.9%), followed by Japan (*n* = 435, 19.4%) and Italy (*n* = 166, 7.42%). At the continental level, Asia collectively produced the largest share of output (*n* = 884, 39.5% of the total corpus), exceeding the Americas (*n* = 687, 30.7%) and Europe (*n* = 492, 22.0%). *Gastrointestinal Endoscopy* was the most productive journal (*n* = 173). Tokyo Medical University was the most productive institution (*n* = 93, 4.16%). Four thematic clusters were identified: EUS-guided biliary and pancreatic ductal drainage; diagnostic, ablative, and injection EUS for pancreatic tumors; LAMS-enabled luminal bypass and gallbladder drainage; and pancreatic fluid collections and necrotizing pancreatitis. Temporal overlay confirmed EUS-guided gastroenterostomy, EUS-guided gallbladder drainage, and EUS-guided radiofrequency ablation as the leading research frontiers. Annual output showed no discernible contraction during the COVID-19 pandemic period (2020–2021). **Conclusions**: The United States led global I-EUS research output with higher rates of international collaboration compared with East Asian nations, although Asia as a continent generated the largest aggregate volume of publications. EUS-guided biliary drainage and pancreatic fluid collection management constitute the established, high-volume core of the field, while EUS-guided gastroenterostomy and novel ablative technologies represent the most dynamic investigative frontiers.

## 1. Introduction

Endoscopic ultrasound (EUS) has undergone a paradigm transformation over the past two and a half decades, evolving from a purely diagnostic imaging modality into a multidimensional interventional platform capable of accessing, draining, ablating, and bypassing structures throughout the gastrointestinal tract and adjacent organs [1,2]. This evolution began in 2001, when Giovannini et al. reported the first EUS-guided bilioduodenal anastomosis for biliary decompression [1], establishing the proof-of-concept for transluminal EUS-guided therapy and inaugurating what is now recognized as the era of interventional EUS (I-EUS). Subsequent technical milestones—including early descriptions of EUS-guided hepaticogastrostomy and the development of the first dedicated transmural drainage devices—progressively expanded the clinical scope of this modality [3,4].

The contemporary spectrum of I-EUS encompasses: EUS-guided biliary drainage (EUS-BD), including choledochoduodenostomy (EUS-CDS), hepaticogastrostomy (EUS-HGS), hepaticojejunostomy (EUS-HJS), and rendezvous procedures (EUS-RV); EUS-guided gallbladder drainage (EUS-GBD); EUS-guided gastroenterostomy (EUS-GE) for gastric outlet obstruction (GOO); EUS-guided pancreatic duct drainage (EUS-PD); transmural drainage of pancreatic fluid collections and walled-off necrosis (WON); EUS-guided celiac plexus neurolysis (EUS-CPN) and ganglia neurolysis (EUS-CGN); EUS-guided radiofrequency ablation (EUS-RFA) of pancreatic neoplasms; fine-needle injection of antitumor agents; and transmural drainage of abdominal abscesses [2,5]. Each modality has evolved from technical proof-of-concept to progressively consolidated randomized controlled trial (RCT) evidence, driven by the clinical urgency of the conditions it addresses.

The clinical demand for I-EUS is shaped by malignancies of considerable global epidemiological burden. Pancreatic adenocarcinoma, cholangiocarcinoma, and periampullary tumors collectively account for over 1 million deaths annually worldwide [6], with the majority of patients presenting at an unresectable stage requiring effective biliary or gastric decompression. Malignant GOO, affecting 10–20% of patients with advanced pancreatic or periampullary malignancy, carries severe nutritional and symptomatic consequences; EUS-GE has emerged as a technically favorable and physiologically superior alternative to both duodenal stenting and surgical gastrojejunostomy [7,8]. In the domain of inflammatory pancreatic disease, the paradigm shift from open surgical necrosectomy to the endoscopic step-up approach for infected necrotizing pancreatitis—validated by landmark PANTER and TENSION RCTs [9,10]—has positioned EUS-guided transmural drainage as a cornerstone of modern critical pancreatology. The lumen-apposing metal stent (LAMS), providing a stable, wide-bore conduit for transmural access, has been the enabling technology across multiple I-EUS applications, unifying the drainage of pancreatic pseudocysts, WON, gallbladder, and gastroenteric anastomosis within a single interventional paradigm [11]. The clinical consolidation of this platform has been formalized by a recent international expert consensus, developed through a modified Delphi process, providing recommendations for the safe use of LAMS across both on-label and off-label indications [12].

Given the rapid proliferation of procedural innovations, multicenter RCT evidence, and international guideline revisions across all I-EUS domains, a macroscopic quantitative perspective on the global intellectual structure of the field is essential for identifying consolidated evidence domains, detecting emerging research themes, and delineating future investigative priorities. Bibliometric analysis—a validated scientific mapping methodology quantifying research output, citation networks, and thematic clusters—is well-suited to this purpose [13,14]. While bibliometric analyses have been reported for individual I-EUS sub-domains, a comprehensive bibliometric analysis encompassing the full spectrum of interventional EUS applications from 2001 to 2024 has not previously been published.

The present study aims to: (1) characterize the temporal trajectory of annual publication output; (2) identify the most productive countries, continents, institutions, and source journals; and (3) map the intellectual structure and thematic evolution of the field using keyword co-occurrence network analysis.

## 2. Materials and Methods

### 2.1. Data Source and Search Strategy

This bibliometric analysis utilized the Web of Science (WoS) Core Collection (SCIE) [15], searched for publications spanning 1 January 2001 to 31 December 2024, and was executed on 9 May 2026. The start date of 2001 corresponds to the first reported EUS-guided choledochoduodenostomy by Giovannini et al. [1], the seminal technical milestone inaugurating the I-EUS era. The Topic field (TS) search encompassed the title, abstract, and author keywords, using a compound Boolean string combining I-EUS procedure-specific acronyms (EUS-BD, EUS-GBD, EUS-GE, EUS-PD, EUS-HGS, EUS-CDS, EUS-AG, EUS-CPN, EUS-CPB, EUS-CGN, EUS-RFA, EUS-FNI, EUS-FNT, LAMS, choledochoduodenostomy, hepaticogastrostomy, interventional EUS, therapeutic EUS) with proximity-operator constructs linking “endoscopic ultrasound” or equivalent terms with drainage, ablation, neurolysis, and anastomosis descriptors. A Web of Science Category filter restricted retrieval to Gastroenterology & Hepatology, Surgery, and Oncology. A NOT clause excluded known non-endoscopic medical contexts. The search string was constructed and cross-checked by two gastroenterologists with dedicated I-EUS practice (K.T. and H.K.) and was iteratively refined by verifying that all landmark I-EUS publications known a priori to the investigators (references [1,3,4,7,8,9,10,11]) were successfully retrieved. The complete Web of Science search query used was as follows:

TS = ((“EUS-BD” OR “EUS-GBD” OR “EUS-GE” OR “EUS-PD” OR “EUS-HGS” OR “EUS-CDS” OR “EUS-AG” OR “EUS-CPN” OR “EUS-CPB” OR “EUS-CGN” OR “EUS-RFA” OR “EUS-FNI” OR “EUS-FNT” OR “lumen-apposing metal stent*” OR “LAMS” OR “choledochoduodenostom*” OR “hepaticogastrostom*” OR “interventional endoscopic ultrasound*” OR “therapeutic endoscopic ultrasound*” OR “interventional EUS” OR “therapeutic EUS”) OR ((“endoscopic ultrasound*” OR “endoscopic ultrasonograph*” OR “endosonograph*” OR “echoendoscop*” OR “echo-endoscop*”) AND (“guided biliary drain*” OR “guided gallbladder drain*” OR “guided gastroenterostom*” OR “guided pancreatic duct drain*” OR “guided celiac plexus” OR “guided coeliac plexus” OR “guided celiac gangli*” OR “guided neurolysis” OR “guided ablat*” OR “guided ethanol inject*” OR “guided fiducial” OR “guided brachytherap*” OR “guided vascular” OR “guided portal vein” OR “guided anastomosis” OR “guided antegr*” OR “guided rendezvous”)) OR ((“endoscopic ultrasound*” OR “endoscopic ultrasonograph*” OR “echoendoscop*” OR “echo-endoscop*”) AND (“walled-off necrosis” OR “pancreatic pseudocyst*” OR “pancreatic fluid collection*” OR “pancreatic abscess*”))) AND WC = (“Gastroenterology & Hepatology” OR “Surgery” OR “Oncology”) NOT TS = (“lipid-associated macrophage*” OR “lipid associated macrophage*” OR “adipose-associated macrophage*” OR “foam cell*” OR “macrophage polariz*” OR “tumor-associated macrophage*” OR “lymphoma-associated macrophage*” OR “lamellipodia*” OR “lamellipodium” OR “Latin American Screening” OR “Los Angeles Motor Scale”).

### 2.2. Inclusion and Exclusion Criteria

**Figure 1:**
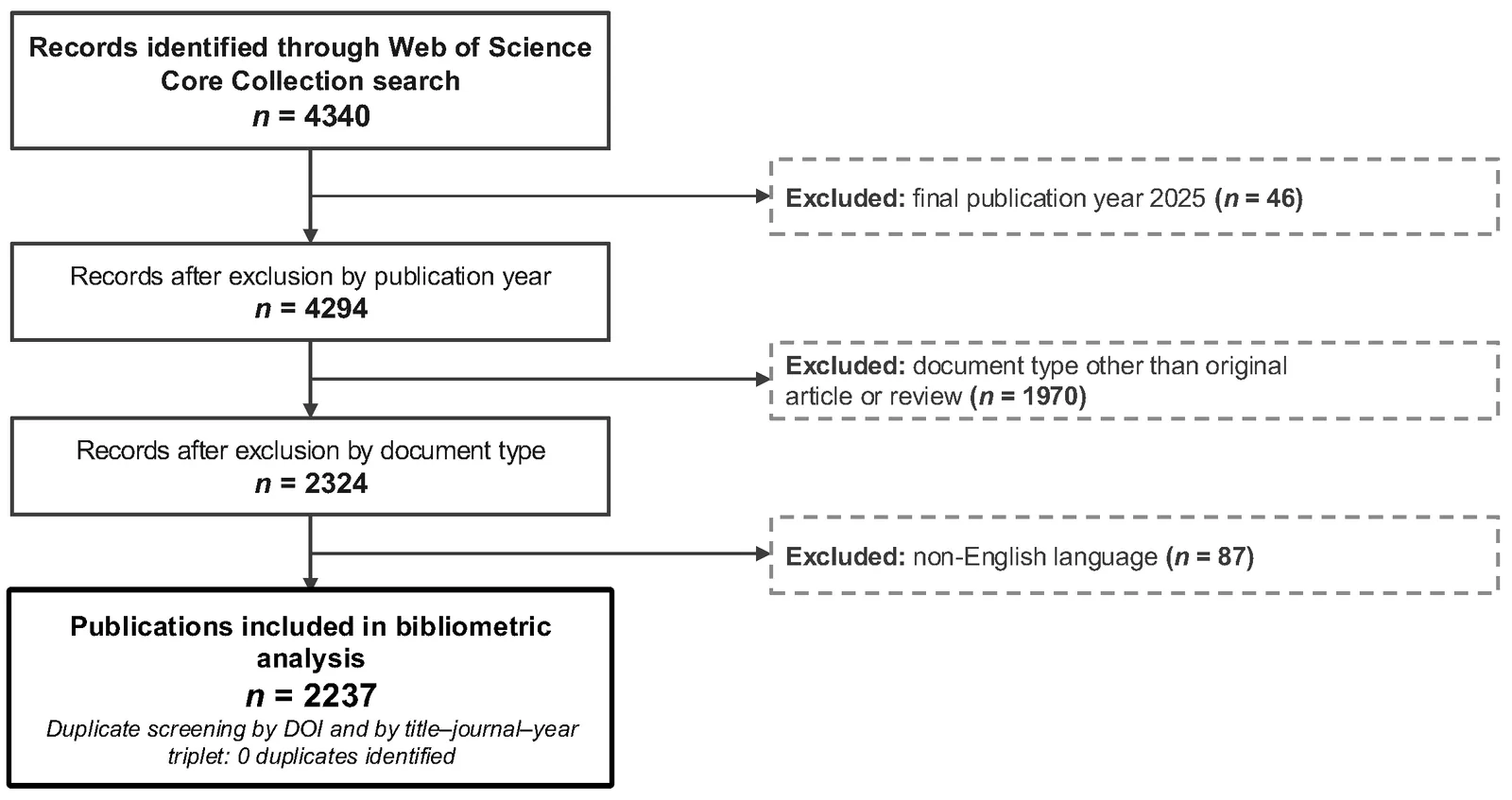
Flow diagram of record identification and selection. Search executed 9 May 2026; publication window 1 January 2001 to 31 December 2024. Conference abstracts, letters, editorials, and book chapters were excluded as document types. Retrieval was confined to a single database, so duplicate records could not arise by construction; screening by DOI and by title–journal–year triplet nonetheless confirmed that none was present.

Inclusion criteria were: (1) English language; (2) document type restricted to original articles or review articles; and (3) final publication date from 1 January 2001 to 31 December 2024. Conference abstracts, letters, editorials, and book chapters were excluded. Records with a final publication year of 2025 (*n* = 46) were excluded to ensure bibliometric stability across complete annual publication cycles. The inclusion and exclusion criteria applied at each stage of record selection, together with the number of records removed at each stage, are summarized in Figure 1.

### 2.3. Data Processing and Record Selection

The initial search retrieved 4340 documents. After excluding 2025 records (*n* = 46), non-original/review articles (*n* = 1970), and non-English records (*n* = 87), a final dataset of 2237 publications was analyzed. The complete selection process is depicted schematically in Figure 1.

Because retrieval was confined to a single bibliographic database (WoS Core Collection), no cross-database record merging was performed and duplicate records could not arise by construction. Nonetheless, the exported dataset was screened for duplicate Digital Object Identifiers (DOIs) and for identical title–journal–year triplets using R; no duplicate records were detected. Records lacking a DOI (predominantly early-period publications) were additionally screened manually by title.

### 2.4. Bibliometric Analysis and Visualization

Bibliometric analysis—including annual production trends and country-, continent-, institutional-, and journal-level analyses—was performed using the R package bibliometrix (version 5.0) [13]. Countries were assigned based on the corresponding author’s affiliation. Single-country publications (SCPs) were defined as those with all authors sharing the same country affiliation; multiple-country publications (MCPs) indicated international co-authorship.

Continental aggregation was performed by summing the national article counts of the 21 most productive countries and regions listed in Table 1 according to standard United Nations geographic regions; Turkey was assigned to Asia in accordance with the United Nations M49 standard. Because this aggregation is restricted to the top 21 contributing nations, which together account for 2098 of 2237 records (93.8%), the resulting continental totals represent a lower bound rather than an exhaustive continental census.

Institutional analysis was performed on the corresponding author’s affiliation field. Because institutional names are indexed inconsistently in WoS (variant abbreviations, departmental prefixes, and separate indexing of universities and their affiliated teaching hospitals), affiliation strings were manually consolidated prior to ranking. Specifically, university records were merged with the records of their integrated academic medical centers where the two constitute a single research entity (Harvard University with Harvard University Medical Affiliates; Johns Hopkins University with Johns Hopkins Medicine; Catholic University of the Sacred Heart with IRCCS Policlinico Gemelli; University of North Carolina with University of North Carolina at Chapel Hill; Cornell University with Weill Cornell Medicine; University of Ulsan with Asan Medical Center). Consolidation decisions were made independently by two investigators (K.T. and T.Sa.) and reconciled by discussion.

Keyword co-occurrence network analysis was performed using VOSviewer (version 1.6.20; Leiden University) [14]. Author keywords were extracted from all 2237 included documents. A total of 3923 unique author keywords were identified.

Author keywords in the raw export exhibited substantial redundancy arising from three sources: (i) singular/plural variants (e.g., “pseudocysts” vs. “pseudocyst”); (ii) hyphenation and spacing variants together with expanded-versus-abbreviated forms of identical concepts (e.g., “lumen-apposing metal stent,” “lumen apposing metal stents,” and “LAMS”); and (iii) synonymous procedural designations (e.g., “endoscopic ultrasonography,” “endosonography,” and “EUS”). To resolve this redundancy, a custom VOSviewer thesaurus file was constructed in the standard two-column “label/replace by” format, mapping each variant onto a single canonical term. The thesaurus comprised 106 mapping entries and reduced the keyword pool from 3923 to 3821 unique terms. The complete thesaurus is provided in Supplementary File S2. Thesaurus construction was performed by one investigator (K.T.) and independently verified by a second (N.Y.); no disagreements requiring adjudication arose, as all mappings were between orthographic or abbreviational variants of a single concept rather than between distinct clinical entities.

Applying a minimum co-occurrence threshold of 15 initially yielded 165 candidate keywords. Each term was independently assessed for exclusion against six prespecified categories, and after reconciliation of inter-rater disagreements (detailed below), 123 terms were excluded and 42 were retained for network construction. The categories were applied in the order listed, and terms satisfying more than one category were assigned to the lowest-numbered applicable category: (i) terms intrinsic to the search strategy itself, which are present in a large proportion of the corpus by construction and therefore carry no discriminative value (*n* = 27; e.g., “eus,” “ercp,” “endoscopy,” “ultrasound,” “drainage,” “transmural drainage,” “stent placement,” “ablation”); (ii) study-design and publication-type descriptors, which denote methodology rather than clinical theme (*n* = 14; e.g., “multicenter,” “randomized-trial,” “meta-analysis,” “case series,” “consensus,” “guidelines,” “feasibility,” “learning-curve”); (iii) generic outcome and non-specific clinical descriptors applied indiscriminately across sub-domains (*n* = 33; e.g., “outcomes,” “safety,” “efficacy,” “complications,” “management,” “therapy,” “risk,” “quality-of-life,” “survival,” “mortality,” “follow-up”); (iv) anatomical and organ-level terms lacking procedural specificity and common to multiple clusters (*n* = 21; e.g., “pancreas,” “liver,” “gallbladder,” “bile duct,” “biliary tract,” “stricture,” “pain”); (v) terms denoting non-EUS comparator or adjacent procedures, which appear in the corpus principally as control arms within outcome studies rather than as I-EUS research themes (*n* = 18; e.g., “cholecystectomy,” “surgical gastrojejunostomy,” “roux-en-y gastric bypass,” “radiotherapy,” “gemcitabine”); (vi) truncated, malformed, or topically unrelated index artifacts (*n* = 10; e.g., “long,” “edge,” “up approach,” “videos,” “ct,” “spinal-cord infarction”).

Conversely, terms denoting a specific anatomical target, disease entity, device, or procedure were retained irrespective of frequency; thus “walled-off necrosis,” “gastric outlet obstruction,” and “pancreatic fluid collection” were retained while the broader terms “necrosis,” “obstruction,” and “duct” were excluded. Exclusion was performed independently by two gastroenterologists (K.T. and T.I.), each blinded to the other’s assignments; observed inter-rater agreement was 96.4% (159 of 165 terms). The six discordant terms were resolved by adjudication with a third investigator (H.K.); this adjudication returned “gastric varices” and “pancreatic cysts” to the network as specific disease entities, while the remaining four discordant terms were assigned to exclusion. Thus, of the 165 candidate keywords, 123 were excluded and 42 were retained for network construction. The full list of excluded terms, annotated by category with the rationale for each category, is provided in Supplementary File S3. The network was visualized in cluster view, temporal overlay view, and density view. The dataset of the 2237 records included in the bibliometric analysis is provided in Supplementary File S1, the custom thesaurus used for keyword unification in Supplementary File S2, and the full list of excluded generic terms in Supplementary File S3.

## 3. Results

### 3.1. Publication Output and Global Contributions

**Figure 2:**
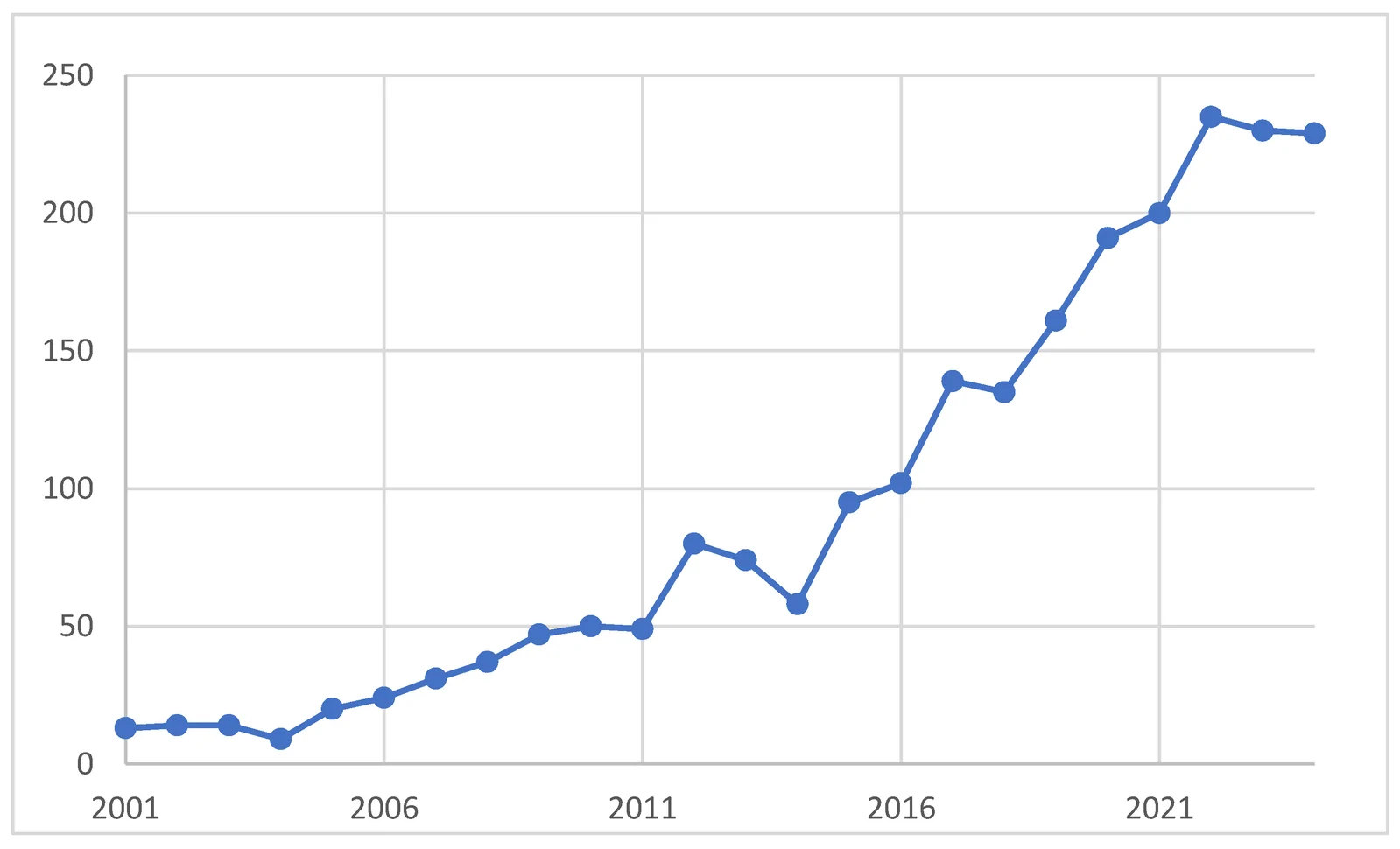
Annual publication output in interventional endoscopic ultrasound, 2001–2024 (*n* = 2237). The *x*-axis denotes the calendar year of publication; the *y*-axis denotes the number of articles published per year. Three phases are delimited: Phase 1 (2001–2009), sparse and stable; Phase 2 (2010–2016), moderate acceleration; Phase 3 (2017–2024), steep and sustained growth.

Analysis of 2237 publications demonstrated three phases of annual output (Figure 2). Phase 1 (2001–2009) was characterized by sparse and relatively stable output, reflecting the nascent, proof-of-concept nature of I-EUS during this period, in which individual case series and small institutional experiences dominated the literature. Phase 2 (2010–2016) showed moderate acceleration, coinciding with early LAMS development, growing RCT evidence for EUS-BD, and initial consolidation of the endoscopic step-up approach for necrotizing pancreatitis following the landmark PANTER trial [9]. Phase 3 (2017–2024) was marked by a steep and sustained increase, driven by the global dissemination of EUS-GE, publication of multiple pivotal RCTs, and accelerating international guideline endorsement. Notably, annual output exhibited no contraction during the COVID-19 pandemic period, remaining continuous with the preceding Phase 3 growth curve (Figure 2); this is examined in Section 4.2.

Table 1 shows the top 21 countries and regions by article output (2001–2024). The United States led global output (*n* = 625, 27.9%; MCP = 17.0%), followed by Japan (*n* = 435, 19.4%; SCP = 91.3%), Italy (*n* = 166, 7.42%; MCP = 42.2%), China (*n* = 152, 6.79%; SCP = 83.6%), and India (*n* = 151, 6.75%; SCP = 84.1%) (Table 1). The United States and Japan collectively accounted for 47.4% of total output (*n* = 1060/2237). Among the top five contributing nations, Italy exhibited the highest MCP rate (42.2%), consistent with active participation in ESGE guideline development networks. Across all contributing nations, Switzerland showed the highest MCP rate (78.6%), followed by Belgium (45.7%), Italy (42.2%), and Canada (40.0%).

Aggregation of the 21 most productive countries by continent revealed a pattern not evident from national rankings alone. Asia constituted the single largest contributing continent (*n* = 884, 39.5% of the total corpus; Japan, China, India, South Korea, Thailand, and Turkey), exceeding the Americas (*n* = 687, 30.7%; the United States, Brazil, and Canada) and Europe (*n* = 492, 22.0%; Italy, France, Spain, Germany, the United Kingdom, Belgium, Romania, Portugal, the Netherlands, and Switzerland). Oceania (*n* = 21, 0.94%) and Africa (*n* = 14, 0.63%) contributed marginally.

### 3.2. Most Productive Institutions

Table 2 shows the 11 most productive institutions (2001–2024). Tokyo Medical University was the leading contributing institution (*n* = 93, 4.16%), followed by the University of Ulsan including Asan Medical Center (*n* = 83, 3.71%) and Johns Hopkins University including Johns Hopkins Medicine (*n* = 79, 3.53%). The 11 institutions listed collectively accounted for 754 articles (33.7% of total output). Their national distribution—five institutions in the United States, three in Japan, and one each in South Korea, India, and Italy—mirrors the country-level ranking in Table 1 and identifies the specific high-volume academic centers underlying each nation’s output. Notably, all 11 are tertiary referral centers with dedicated therapeutic endoscopy programs, a concentration whose interpretive implications are addressed in Section 4.1.

### 3.3. Most Productive Journals

Table 3 shows the top 10 most productive source journals (2001–2024). *Gastrointestinal Endoscopy* was the most productive publication venue (*n* = 173, 7.73%), followed by *Endoscopic Ultrasound* (*n* = 138, 6.17%) and *Endoscopy International Open* (*n* = 137, 6.12%) (Table 3). The emergence of *Endoscopic Ultrasound* as the second-ranked journal—a specialty journal established specifically to serve the I-EUS community—reflects the sub-specialty maturation of the field. The ten most productive journals collectively accounted for 1056 articles (47.2% of total output).

With respect to journal quality and accessibility, nine of the ten leading venues are indexed in the first or second Journal Citation Reports quartile of the Gastroenterology & Hepatology or Surgery categories, indicating that I-EUS research is predominantly published in established, well-cited journals rather than in low-visibility outlets. Five of the ten (*Endoscopic Ultrasound*, *Endoscopy International Open*, *Clinical Endoscopy*, *World Journal of Gastroenterology*, and *World Journal of Gastrointestinal Endoscopy*) operate under a fully open-access model, while the remaining five are hybrid subscription journals offering optional open access. The publication economics of these two models differ substantially: fully open-access venues in this field levy article processing charges (APCs) typically in the range of approximately USD 1000–3000, whereas optional open access in hybrid journals commonly exceeds USD 4000 per article. This asymmetry has interpretive consequences for the geographic distribution described in Section 3.1 and is discussed in Section 4.1.

### 3.4. Knowledge Structure: Keyword Co-Occurrence Network Analysis

**Figure 3:**
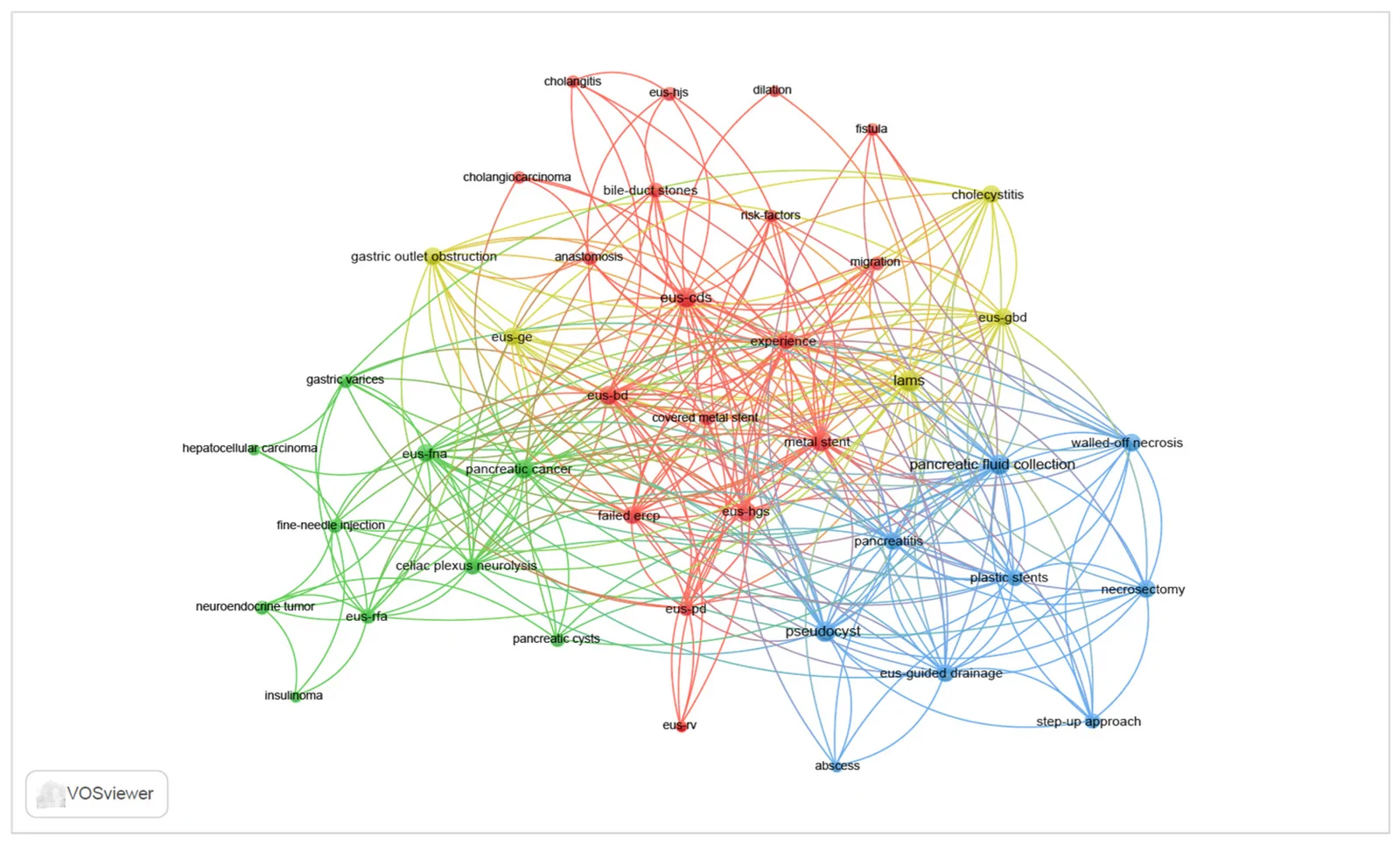
Keyword co-occurrence network of interventional EUS research, 2001–2024—Cluster view. Network constructed from 2237 publications in VOSviewer 1.6.20 (42 keywords retained; see Section 2.4 for thesaurus and exclusion procedures). Node diameter is proportional to keyword frequency; edge thickness to co-occurrence strength. Four modularity-based clusters: Cluster 1 (red), EUS-guided biliary and pancreatic ductal drainage; Cluster 2 (green), diagnostic, ablative, and injection EUS for pancreatic tumors; Cluster 3 (yellow), LAMS-enabled luminal bypass and gallbladder drainage; Cluster 4 (blue), pancreatic fluid collections and necrotizing pancreatitis. Abbreviations are defined in the Abbreviations list.

Keyword co-occurrence network analysis identified four thematic clusters, illustrated in the cluster view (Figure 3). Cluster 1 (red), the largest and most central cluster, encompasses EUS-guided biliary and pancreatic ductal drainage, with dominant nodes including “eus-bd,” “eus-cds,” “eus-hgs,” “eus-hjs,” “eus-pd,” “eus-rv,” “failed ercp,” and “covered metal stent,” reflecting the field’s historical and volumetric core; the co-localization of “eus-pd” within this cluster rather than as a separate domain reflects the shared transluminal-access principle underlying biliary and pancreatic ductal drainage. Cluster 2 (green) groups diagnostic, ablative, and injection-based EUS directed at pancreatic and adjacent tumors, comprising “pancreatic cancer,” “eus-fna,” “eus-rfa,” “fine-needle injection,” “celiac plexus neurolysis,” “neuroendocrine tumor,” and “insulinoma,” spanning both the field’s oldest pain-management stream and its most recently emergent oncological ablation technologies. Cluster 3 (yellow) captures LAMS-enabled luminal bypass and gallbladder drainage, including “lams,” “eus-ge,” “eus-gbd,” “gastric outlet obstruction,” “anastomosis,” and “cholecystitis,” defining the most structurally novel domain in the corpus; the position of “lams” within this cluster while bordering the pancreatic-drainage cluster reflects the platform’s dual role across luminal bypass and pancreatic fluid collection drainage. Cluster 4 (blue) represents pancreatic fluid collections and necrotizing pancreatitis, anchored by “pancreatic fluid collection,” “walled-off necrosis,” “pseudocyst,” “necrosectomy,” and “step-up approach.” Node size is proportional to cumulative keyword frequency, with “eus-bd,” “walled-off necrosis,” and “lams” forming among the largest nodes, consistent with their status as the most extensively published topics in the corpus.

### 3.5. Thematic Evolution: Overlay Visualization

**Figure 4:**
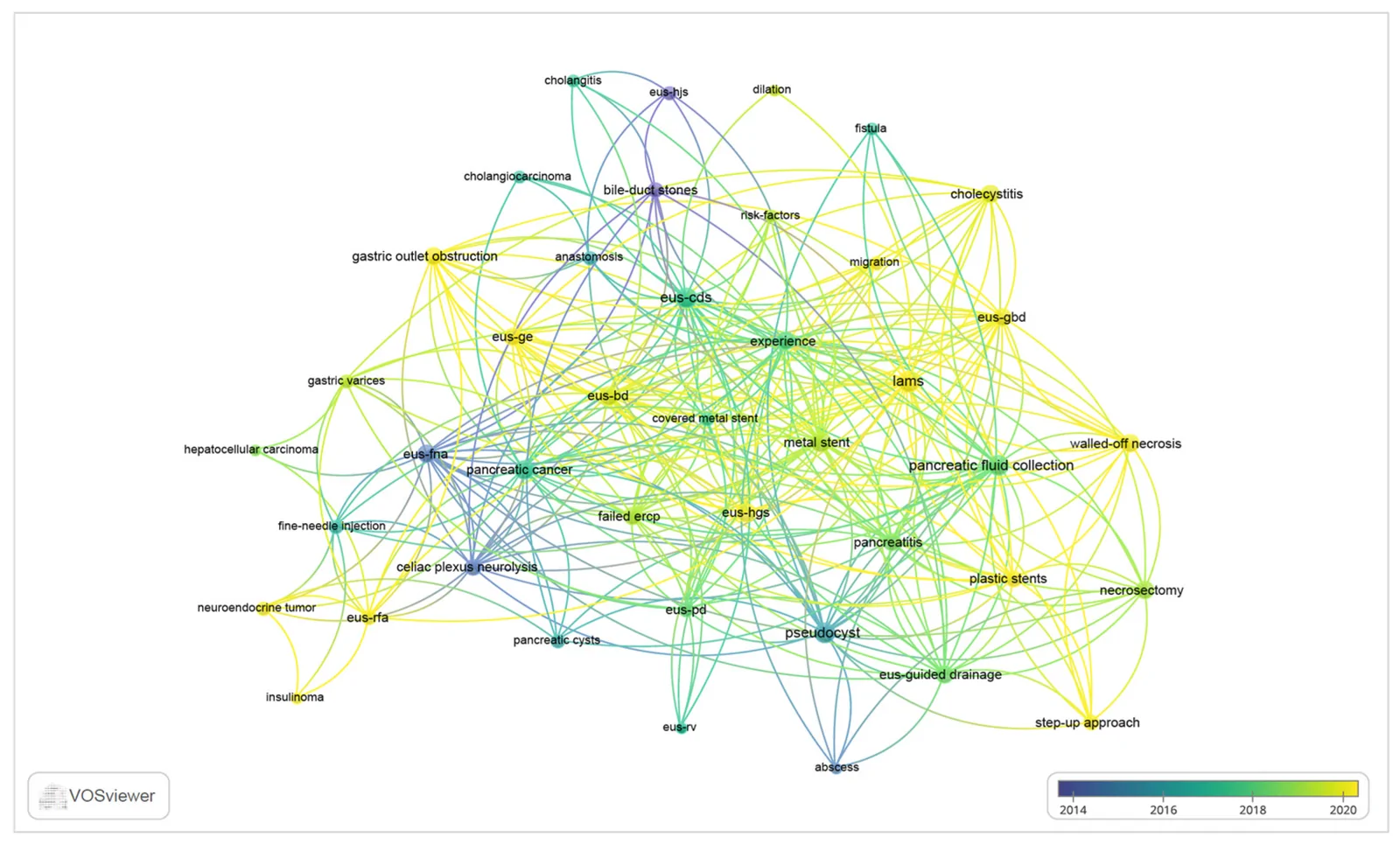
Keyword co-occurrence network—Temporal overlay view. The network of Figure 3 with each node colored by the mean publication year of documents containing that keyword (dark blue, ~2012–2014; yellow-green, ~2020–2022). Dark-blue nodes denote foundational research streams; intermediate-colored nodes the consolidation phase (~2015–2018); yellow-green nodes the current research frontiers (~2019–2022). Node diameter is as in Figure 3. Abbreviations are defined in the Abbreviations list.

The temporal overlay view (Figure 4) maps thematic research evolution, with node colors ranging from dark blue (mean publication year approximately 2012–2014) to yellow-green (approximately 2020–2022), providing a chronological stratification of the field’s intellectual trajectory that complements the static cluster topology of Figure 3. The transition from dark-blue to yellow-green nodes across the network encodes the sequential maturation of I-EUS research domains: from early salvage drainage procedures through endoscopic management of pancreatic necrosis to contemporary luminal bypass and ablative frontiers. Terms in the deepest blue—“pseudocyst,” “celiac plexus neurolysis,” “eus-fna,” “eus-cds,” and “failed ercp”—reflect the earliest research streams of the field, with mean publication years approximately 2012–2015, indicating that these topics dominated the literature during the foundational period. Terms of intermediate recency (~2015–2018) include “eus-bd,” “eus-hgs,” “walled-off necrosis,” “step-up approach,” and “necrosectomy,” consistent with the maturation of EUS-BD as a clinical standard and the consolidation of endoscopic necrosectomy protocols during this period. The most recently active terms (yellow-green, ~2019–2022)—“eus-ge,” “eus-gbd,” “eus-rfa,” “lams,” “anastomosis,” and “gastric outlet obstruction”—confirm EUS-guided luminal bypass and ablative technologies as the temporal frontier of the field. Notably, “lams” appears as an intermediate-to-recent node straddling the boundary between the luminal-bypass cluster (Cluster 3) and the pancreatic fluid collection cluster (Cluster 4), reflecting its progressive expansion from pancreatic pseudocyst drainage into the newer GE and GBD applications and underscoring LAMS as the enabling technology that has catalyzed the most recent phase of I-EUS growth. The spatial separation between the deepest-blue nodes (biliary salvage, pseudocyst) and the yellow-green frontier nodes (GE, GBD, RFA) recapitulates the entire 24-year developmental arc of the field within a single network plane.

### 3.6. The Density View

**Figure 5:**
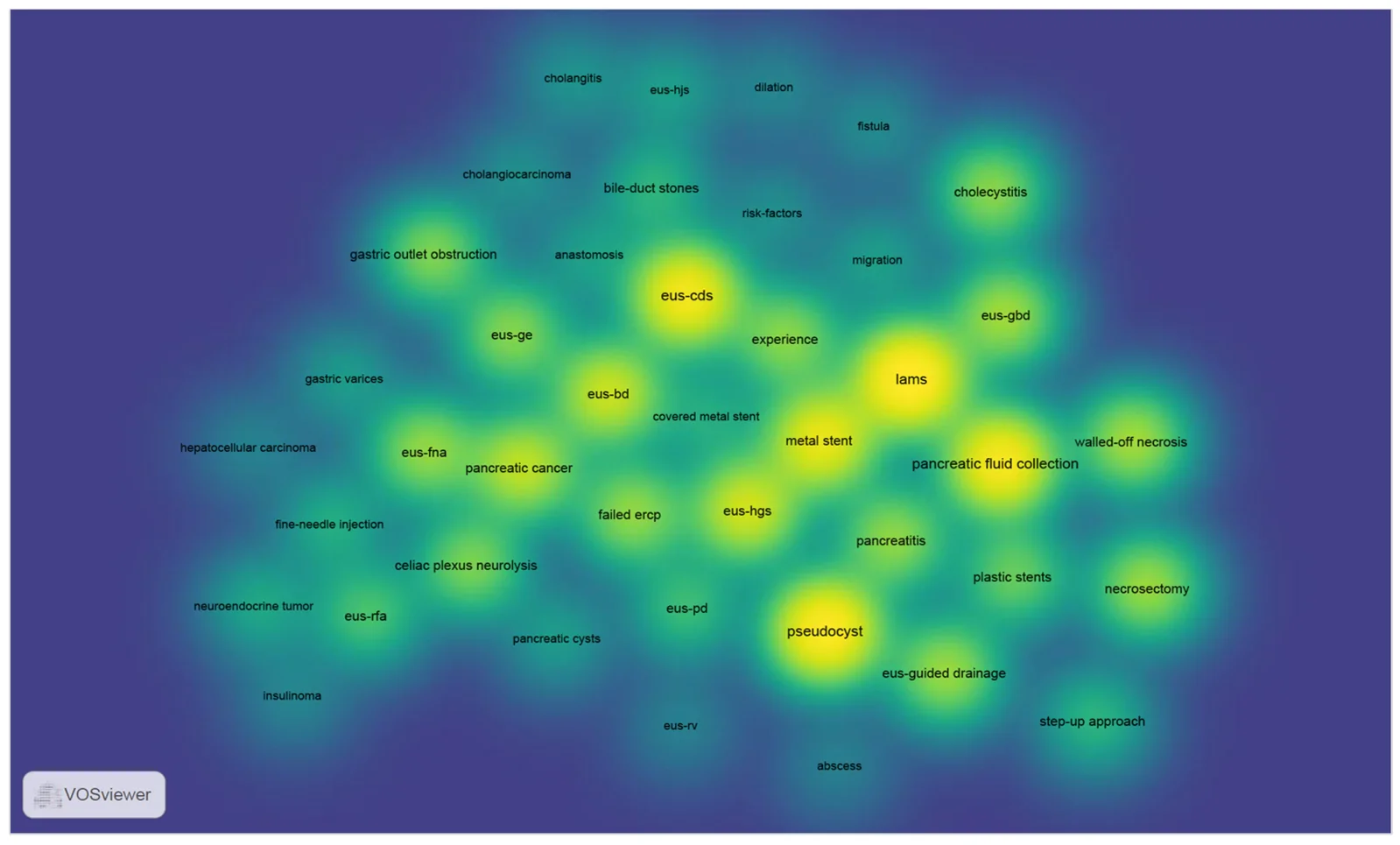
Keyword co-occurrence network of interventional EUS research, 2001–2024—Density view. The network of Figure 3 rendered in density mode. Yellow-red shading indicates regions of highest co-occurrence density; blue-purple shading indicates lower density. Abbreviations are defined in the Abbreviations list.

The density view (Figure 5) complements the cluster and temporal overlay visualizations by mapping the relative publication intensity across the same keyword network. The highest-density centroids are localized within the “eus-bd”–“eus-cds”–“eus-hgs” continuum (Cluster 1) and the “walled-off necrosis”–“pseudocyst” node pair (Cluster 4), confirming EUS-guided biliary drainage and pancreatic fluid collection management as the most prolifically studied research themes in this corpus. Intermediate-density regions correspond to the “lams” node and the surrounding luminal bypass cluster (Cluster 3, yellow), consistent with the intermediate-to-recent temporal positioning of these terms observed in Figure 4. The lowest-density nodes—including “eus-rfa,” “eus-pd,” and “insulinoma”—reflect their status as emerging or highly specialized research themes with comparatively limited but rapidly growing publication volume and notably correspond to the most recently dated nodes in Figure 4, confirming that low current density does not imply low future trajectory for these frontiers.

## 4. Discussion

### 4.1. General Trends, Geographic Distribution, and Bibliometric Bias

The accelerating growth in annual publication output from 2017 onward reflects the convergence of three concurrent forces: the global dissemination of LAMS-enabled transmural procedures; the maturation of EUS-BD from rescue technique to prospectively validated first-line alternative, supported by multicenter randomized controlled trials [16,17] and subsequently by further randomized evidence [18,19] and by meta-analysis [20]; and the parallel consolidation of endoscopic step-up management for necrotizing pancreatitis as the dominant treatment paradigm following the TENSION Lancet trial [10]. The acceleration from Phase 2 to Phase 3 coincides precisely with the commercial introduction and worldwide adoption of LAMS platforms that enabled technically reliable EUS-GE and EUS-GBD, generating an entirely new publication stratum [7,8].

The United States’ leadership in global output (*n* = 625, 27.9%) with an MCP rate of 17.0% departs markedly from the pattern observed in the parallel domain of malignant biliary obstruction endoscopy, where Japan occupies the dominant position with a higher single-country publication rate. This structural divergence reflects the multidisciplinary and clinically diverse nature of I-EUS, which encompasses pain management, pancreatic necrosis management, and luminal bypass procedures that have generated substantial academic output from high-volume North American and European centers conducting international multicenter trials. Japan retains a strong second-place position (*n* = 435, 19.4%) reflecting its pre-eminence in biliary endoscopy and ERCP-adjacent practice, but its SCP rate (91.3%) signals predominantly self-contained national research networks—a structural constraint on the international visibility of Japanese evidence in Western guideline development processes, including ESGE and ASGE.

The continental aggregation presented in Section 3.1 reveals a dimension of the global research landscape that national rankings alone obscure. Although the United States is by a wide margin the most productive single nation, Asia is the most productive continent (*n* = 884, 39.5%), exceeding both the Americas (*n* = 687, 30.7%) and Europe (*n* = 492, 22.0%). This inversion between national and continental leadership has a structural explanation: Asian output is distributed across several largely independent national programs—Japan, China, India, and South Korea each contributing more than 100 articles—whereas output in the Americas is concentrated almost entirely within a single country. Crucially, the four leading Asian contributors also carry the highest SCP rates in the corpus (Japan 91.3%, South Korea 88.9%, India 84.1%, China 83.6%), whereas the major European contributors show uniformly higher international co-authorship. Asia’s aggregate volume advantage therefore does not translate into proportionate influence over guideline development, because its output is fragmented into nationally self-contained networks. Given the high incidence of pancreatobiliary malignancy across East and South Asia, the region generating the largest disease burden and research volume is nonetheless underrepresented in the consensus processes that shape global practice. Establishing pan-Asian multicenter trial consortia, analogous to the European networks underpinning Italy’s and Belgium’s high MCP rates, is a concrete strategic priority.

A further consideration relevant to interpreting geographic distribution concerns publication economics. As noted in Section 3.3, half of the ten leading venues in this corpus are fully open-access journals levying APCs, and hybrid journals typically charge higher fees for optional open access. Because APCs are borne by authors or their institutions, differential access to publication funding may shape which centers and which nations are able to publish in the highest-visibility venues, independently of scientific merit. This constitutes a structural bias in bibliometric analysis that is rarely acknowledged and that plausibly disadvantages investigators in lower-resource settings. It offers one partial explanation for the marked concentration of output within a small number of well-resourced academic systems, with Africa (*n* = 14, 0.63%) and Latin America (Brazil, *n* = 32, 1.43%) contributing minimally despite substantial regional pancreatobiliary disease burden. We emphasize that this is one contributing factor among several—differences in procedural volume, echoendoscope and LAMS availability, and dedicated research infrastructure are likely to be more consequential—and that the present data permit no formal apportionment among them.

The institutional analysis (Table 2) identifies the specific centers underlying national output patterns and reinforces the interpretation above. Three of the eleven leading institutions are Japanese and one is Korean, consistent with East Asian pre-eminence in biliary and pancreatic endoscopy; five are North American, reflecting the concentration of multicenter trial leadership in United States academic centers; and the sole European entry—Catholic University of the Sacred Heart with IRCCS Policlinico Gemelli—corresponds precisely to the network identified below as driving Italy’s exceptional MCP rate. That the eleven leading institutions account for one-third of the entire corpus (33.7%) quantifies the extent to which I-EUS evidence is generated by a small number of tertiary referral centers with dedicated therapeutic endoscopy infrastructure, high procedural volume, and established research programs. This concentration is itself a finding with implications for external validity: outcomes reported from these centers may not be reproducible in lower-volume community settings, a consideration directly relevant to the skill-dissemination priorities outlined in Section 4.8. The methodological caveats attending institutional name standardization are detailed in the note to Table 2 and in Section 4.7.

Italy’s exceptional MCP rate (42.2%) among the top five contributing nations reflects the deep integration of Italian academic endoscopy centers—particularly the Catholic University/Policlinico Gemelli network and the San Raffaele consortium in Milan—into European multicenter trial networks and ESGE guideline working groups. Belgium’s high MCP rate (45.7%)—exceeded only by Switzerland, whose very small output (*n* = 14) renders its rate unstable—similarly reflects the Université Libre de Bruxelles/Erasme Hospital network’s established role in European endoscopy research coordination.

Several sources of systematic bias require acknowledgment. Restriction to the WoS Core Collection introduces English-language and high-impact-journal bias, potentially underrepresenting significant work published in Japanese, Korean, or Chinese specialty journals—a limitation particularly relevant given the Asia-led nature of biliary EUS. A high-volume center publication bias is operative: academic outputs predominantly originate from tertiary referral centers with dedicated I-EUS infrastructure, which systematically underrepresents community endoscopy practice. Potential self-citation inflation, particularly relevant for the small cohort of key opinion leaders who anchor the I-EUS literature, warrants acknowledgment when interpreting citation metrics. These and further limitations are systematically enumerated in Section 4.7.

### 4.2. Publication Output During the COVID-19 Pandemic

The COVID-19 pandemic imposed severe and well-documented constraints on gastrointestinal endoscopy practice worldwide from 2020–2021, including the deferral of elective procedures, restrictions on aerosol-generating interventions, redeployment of endoscopy personnel, and suspension of prospective trial enrollment. It might therefore be anticipated that annual I-EUS publication output would exhibit a corresponding contraction. Figure 2 shows that it did not: the growth trajectory across 2020–2021 remained continuous with the preceding Phase 3 curve, with no inflection attributable to the pandemic period.

Three explanations, not mutually exclusive, account for this apparent discordance. First, and most importantly, publication date lags procedural date by a substantial and variable interval—typically 12 to 36 months from the completion of data collection to final publication, encompassing analysis, manuscript preparation, peer review, and production. Articles appearing from 2020–2021 therefore predominantly report procedures performed from 2018–2020, and the bibliometric signal of any pandemic-related reduction in procedural volume would be expected to manifest from 2022–2023 rather than contemporaneously. Second, I-EUS procedures are disproportionately performed for urgent or semi-urgent indications—malignant biliary obstruction, infected walled-off necrosis, acute cholecystitis in patients unfit for surgery, and malignant gastric outlet obstruction—which were prioritized for continuation under most pandemic triage protocols and were consequently less affected than screening or elective diagnostic endoscopy. Third, the reduction in clinical service commitments during pandemic periods plausibly increased the time available to investigators for retrospective analysis and manuscript preparation, a phenomenon documented across multiple medical specialties from 2020–2021.

A modest attenuation of growth is discernible in the most recent years of the series. Because this study did not extract the procedural dates of individual studies—only their publication dates—we cannot determine with confidence whether this attenuation reflects the delayed bibliometric echo of pandemic-era reductions in procedural volume and trial enrollment, a natural plateau following the saturation of the LAMS-driven publication surge, or the incomplete indexing of recent records at the time of data extraction. Distinguishing among these explanations would require linkage of publication records to study-period metadata, which is not systematically available in bibliographic databases and would necessitate full-text review of the entire corpus. We therefore present this observation descriptively and refrain from causal inference. Formal analysis of the pandemic’s effect on I-EUS research productivity—ideally through interrupted time-series methods applied to procedure-date rather than publication-date data—represents a discrete and worthwhile subject for future investigation.

### 4.3. Thematic Shift 1: EUS-BD—From Biliary Rescue to First-Line Therapy

The dominance of Cluster 1 (EUS-Guided Biliary and Pancreatic Ductal Drainage) as the most central thematic domain is historically grounded in the foundational 2001 paper by Giovannini et al. [1] and reflects the trajectory of EUS-BD from a rescue salvage technique for failed ERCP to a prospectively validated first-line alternative to ERCP in selected patients. The early literature—anchored by the temporal overlay’s deepest-blue nodes “eus-cds” and “failed ercp”—reflects this originating salvage context, while subsequent case series [3,4], a systematic review [21], and cohort analyses of long-term outcomes and adverse-event predictors [22] together established the reproducibility of EUS-BD beyond pioneer centers.

The most consequential bibliometric shift within Cluster 1 is the normalization of EUS-BD as a first-line approach, formalized by the ESGE guideline of Van der Merwe et al. (2022) [5]. This guideline arrived after pivotal RCTs by Paik et al. [16] and Bang et al. [17] demonstrated non-inferiority of primary EUS-BD to ERCP for distal malignant biliary obstruction with a more favorable pancreatitis profile and was further supported by the systematic review of Sharaiha et al. [23] confirming EUS-BD’s superiority to percutaneous biliary drainage as ERCP rescue.

Randomized evidence published after the study period has continued to refine rather than settle this question, with the two largest trials reaching different conclusions. The international DRA-MBO trial found EUS-guided choledochoduodenostomy and ERCP comparable in efficacy and safety as the initial approach, but with significantly lower risks of reintervention, postprocedure pancreatitis, and tumor ingrowth or overgrowth, and a shorter hospital stay, in the EUS-guided arm [18]. The multicenter ELEMENT trial, by contrast, found EUS-CDS not superior to ERCP in stent function, positioning it as a complementary, exchangeable first-line modality rather than a replacement [19]. The characterization of EUS-BD as a first-line option should therefore be read as procedure- and endpoint-dependent—parity in clinical success with advantages on specific secondary endpoints, not uniform superiority. Because both trials postdate the 2024 corpus boundary, neither contributes to the network topology reported here, but their appearance indicates that the established, high-volume core of Cluster 1 remains an actively contested domain. The ongoing evolution of EUS-HGS and EUS-HJS as the preferred routes in patients with surgically altered anatomy is captured by the more recent mean publication years of “eus-hgs” and “eus-rv” nodes in the temporal overlay (Figure 4), defining the current investigative edge of the biliary drainage cluster.

### 4.4. Thematic Shift 2: Pancreatic Necrosis—The Endoscopic Step-Up Paradigm

Cluster 4 (Pancreatic Fluid Collections and Necrotizing Pancreatitis) reflects one of the most dramatic management paradigm shifts in modern gastroenterology: the transition from open surgical necrosectomy to an endoscopy-led step-up approach for infected WON [9,10,24]. The endoscopic foundation of this shift was established early: EUS-guided transmural drainage of pancreatic fluid collections, first described by Giovannini et al. [25], was subsequently shown to be superior or complementary to conventional non-EUS transmural drainage in a prospective comparison [26] and a randomized controlled trial [27], establishing endosonographic guidance as the procedural standard for transmural access. The foundational PANTER RCT (van Santvoort et al. 2010) [9] demonstrated the superiority of minimally invasive approaches over open necrosectomy. The landmark TENSION RCT (van Brunschot et al. 2018) [10], published in *The Lancet*, subsequently confirmed non-inferiority of endoscopic over surgical step-up, with a significantly lower new-onset organ failure rate in the endoscopic arm—definitively establishing the endoscopic step-up approach as the dominant management paradigm. This trial evidence was rapidly translated into practice guidance: the ESGE multidisciplinary guideline on the endoscopic management of acute necrotizing pancreatitis [28] and the subsequent AGA clinical practice update [29] both positioned endoscopic transmural drainage as the preferred initial intervention, illustrating the short interval between randomized evidence and guideline endorsement that characterizes this cluster.

The co-occurrence of “lams” with “walled-off necrosis” at the boundary between the luminal-bypass cluster (Cluster 3) and the pancreatic fluid collection cluster (Cluster 4) confirms the LAMS–WON interface as a structurally pivotal intersection in the field’s intellectual topology, where Itoi et al. [11] provided the foundational LAMS validation dataset. This was followed by large multicenter experience confirming the feasibility of LAMS for peripancreatic fluid collections and necrosis [30] and by comparative studies evaluating LAMS against fully covered self-expanding metal stents and plastic stents for WON drainage [31]. Subsequent RCT-level evidence—including Bang et al.’s trial demonstrating non-superiority of LAMS over plastic stents for WON drainage [32]—has refined LAMS selection criteria. The accumulated experience across these indications has since been synthesized into an international expert consensus on the safe use of LAMS, developed through a modified Delphi process and encompassing peripancreatic fluid collections, EUS-BD, EUS-GBD, EUS-GE, and temporary gastric access [12]. That a single consensus now spans all five domains mirrors the topology recovered by our network analysis, in which “lams” occupies the interface between the luminal-bypass and pancreatic fluid collection clusters. The temporal overlay reveals “step-up approach” and “necrosectomy” as intermediate-recency nodes (~2016–2018), consistent with the active RCT generation, long-term follow-up reporting [33], and evidence syntheses [34] of this period. This suggests that the fundamental drainage-versus-necrosectomy question has approached resolution, while more nuanced questions of stent type, timing, and multistep protocols continue to generate literature.

### 4.5. Thematic Shift 3: LAMS and EUS-GE as the Structural Research Frontier

Cluster 3 (LAMS-Enabled Luminal Bypass and Gallbladder Drainage) represents the most structurally novel domain in this bibliometric corpus, defined by the convergence of LAMS technology with EUS-GE and EUS-GBD. EUS-GE—the endoscopic creation of a transmural gastroenteric anastomosis using a LAMS for malignant or benign GOO—was first described by Khashab et al. in 2015 [7] and subsequently validated in the multicenter DRA-GOO randomized trial by Teoh et al., which found that EUS-GE reduced the frequency of reintervention, improved stent patency, and produced better patient-reported eating habits than duodenal stenting [35]. That trial was retracted and subsequently corrected and republished in 2025; both the original and the corrected, republished versions are cited here [35], and the findings relied upon are those of the corrected version. Retrospective multicenter series by Khashab et al. [36] and Chen et al. [37] further delineated the technical approaches and outcomes of EUS-GE across institutions with varying expertise levels. The temporal overlay confirms “eus-ge,” “gastric outlet obstruction,” and “anastomosis” among the most recently active terms (~2020–2022), defining the acute edge of the research frontier.

The randomized evidence base has broadened further since the close of the study period. The ENDURO trial, conducted across twelve Dutch academic and teaching hospitals, compared endoscopic with surgical gastroenterostomy and found the endoscopic approach superior for time to resumption of solid oral intake and non-inferior for persistent or recurrent obstructive symptoms requiring reintervention, leading its investigators to conclude that endoscopic gastroenterostomy should be the preferred palliative treatment for malignant gastric outlet obstruction [38]. Notably for the training considerations discussed in Section 4.8, ENDURO restricted participation to centers that had performed at least twenty LAMS placements of any indication and at least ten endoscopic gastroenterostomies, with formally approved competence—an explicit operationalization of the case-volume thresholds that this literature has otherwise left implicit.

EUS-GBD—transmural LAMS-based drainage of the gallbladder for acute cholecystitis in patients unfit for cholecystectomy—has been incorporated into international guidance on the management of acute cholecystitis, including the Tokyo Guidelines 2018 and the WSES guidelines, which address the positioning of gallbladder drainage relative to cholecystectomy [39,40], and has generated a rapidly growing body of comparative evidence against percutaneous cholecystostomy. The DRAC 1 RCT by Teoh et al. [41] demonstrated the superiority of EUS-GBD over percutaneous cholecystostomy in very high-risk surgical patients, providing the first prospective randomized evidence for this technique. The co-localization of “eus-gbd” and “cholecystitis” within Cluster 3 confirms EUS-GBD’s bibliometric proximity to the LAMS–GE framework, sharing enabling technology, procedural principle, and overlapping patient populations. The compression of the interval between technical innovation and consensus standardization is striking in this cluster: EUS-GE progressed from first clinical description [7] to inclusion in the international LAMS consensus [12] within approximately eight years, a markedly shorter interval than the two decades separating the first EUS-BD report [1] from its formalization as a first-line option in the 2022 ESGE guideline [5]. Both procedures remain in the RCT consolidation phase, with continued high per-year citation accumulation anticipated as long-term comparative data mature.

### 4.6. Thematic Shift 4: Therapeutic and Ablative EUS as an Emerging Frontier, and Pediatric Applications

Cluster 2 (Diagnostic, Ablative, and Injection EUS for Pancreatic Tumors) encompasses both the field’s oldest research stream—EUS-CPN, with roots in the foundational paper by Wiersema and Wiersema (1996) [42] and the prospective series by Gress et al. (2001) [43]—and its most recently emergent technologies, including EUS-RFA and fine-needle injection. The yellow-green temporal positioning of “eus-rfa” (~2021–2022) reflects the rapid proliferation of single-institution and early multicenter feasibility data for thermal ablation of pancreatic insulinomas [44], non-functioning neuroendocrine tumors, and cystic neoplasms, consistent with the broader expansion of I-EUS beyond luminal access into parenchymal oncological therapy. The trajectory implied by that temporal positioning has since been borne out: a prospective international multicenter study enrolling patients with both functional and non-functional pancreatic neuroendocrine tumors over a 43-month period, with adverse-event rate as its primary endpoint, reported EUS-RFA to be safe and effective across both tumor types [45]. The progression from the single-center series that dominate the corpus to prospective multicenter evaluation illustrates how a node identified as a temporal frontier in a bibliometric map can be expected to mature and provides external validation that the overlay analysis identified a genuinely ascendant rather than merely fashionable research theme.

EUS-guided pancreatic duct drainage (EUS-PD) did not form a separate cluster but co-localized within Cluster 1 (red), alongside the biliary-access nodes, reflecting the shared transluminal-access principle. It nonetheless remains a highly specialized domain whose evidence base comprises predominantly case series and small cohort studies [46]. The modest bibliometric footprint of “eus-pd” relative to other nodes is consistent with the limited number of centers with sufficient expertise and case volume to generate systematic prospective evidence and anticipates substantial future growth as procedural training and dedicated device development progress.

A domain conspicuously absent from the network topology deserves comment. No pediatric keyword—“pediatric,” “children,” or any age-specific descriptor—met the co-occurrence threshold of 15, so pediatric applications form no identifiable node in any of the three visualizations. This absence is substantive rather than an artifact of the search strategy. Pediatric I-EUS is constrained by the rarity in children of the malignant pancreatobiliary indications that drive adult volume, by echoendoscope diameter relative to pediatric luminal caliber, by the absence of LAMS and accessories dimensioned for pediatric anatomy, and by the concentration of pediatric therapeutic endoscopy in few specialized centers. The published experience accordingly comprises case reports and small series, chiefly transmural drainage of posttraumatic or postpancreatitis pseudocysts and, less often, EUS-CPN for chronic pancreatitis pain. A systematic review and meta-analysis of endoscopic drainage for pediatric pancreatic fluid collections found the approach safe and effective, while noting that study design and small sample size preclude firm conclusions [47]—a caveat applying to the wider pediatric evidence base. Such reports are further attenuated by our Web of Science Category filter (Gastroenterology & Hepatology, Surgery, Oncology), which excluded records indexed principally under Pediatrics. Readers should therefore interpret this analysis as characterizing adult I-EUS research, and a dedicated pediatric review employing age-specific terminology and category filters would be a valuable complement, particularly as device miniaturization progresses.

### 4.7. Strengths and Limitations

The strengths of this analysis merit brief statement. First, to our knowledge this is the first bibliometric analysis encompassing the full spectrum of interventional EUS applications rather than an individual sub-domain, permitting direct comparison of the relative maturity and trajectory of biliary, pancreatic, luminal-bypass, and ablative research streams within a single analytical frame. Second, the 24-year observation window spans the field’s entire history from the index publication of 2001, allowing the complete developmental arc to be reconstructed. Third, the corpus is large (*n* = 2237) and was assembled using a search strategy that combines procedure-specific acronyms with proximity-operator constructs and category filtering, reported here in full to permit exact replication. Fourth, keyword processing was performed using a documented thesaurus and prespecified, categorized exclusion criteria applied by independent raters with formal agreement measurement, and both the thesaurus and the annotated exclusion list are provided as Supplementary Files—a level of methodological transparency uncommon in bibliometric reporting. Fifth, the triangulation of cluster, temporal-overlay, and density visualizations of a single network permits established and emerging domains to be distinguished from one another rather than conflated within a single static map.

Several limitations must be weighed against these strengths.

Database and language restriction. Retrieval was confined to the WoS Core Collection, and non-English records were excluded. Neither Scopus, Embase, nor PubMed was searched. This introduces two distinct biases. The first is coverage bias: journals not indexed in the Core Collection are invisible to the analysis regardless of their clinical influence. The second is language bias, which is not uniformly distributed across the field: because Japan, China, and South Korea are among the leading contributors and each maintains substantial national-language specialty literature, the exclusion of non-English records disproportionately attenuates Asian output. The continental analysis in Section 3.1—which already places Asia first—should therefore be read as conservative, and the true Asian share of global I-EUS scholarship is likely greater than the 39.5% reported here.

Corresponding-author attribution. Country and institutional counts were assigned by the corresponding author’s affiliation, a standard bibliometric convention that nonetheless systematically undercounts centers contributing substantively to multicenter trials without holding corresponding authorship. Given that multicenter collaboration is a defining feature of contemporary I-EUS evidence generation, this limitation is more consequential in this field than in single-center-dominated specialties, and Table 2 should accordingly be read as a ranking of publication leadership rather than of total research contribution.

Institutional name standardization. As detailed in the note to Table 2, institutional affiliations are indexed heterogeneously in WoS. Manual consolidation was applied, but residual misclassification cannot be excluded, and adjacent ranks separated by few articles should not be treated as meaningfully distinct.

Self-citation. Self-citation rates were not quantified. Reliable corpus-scale computation would require author-level disambiguation—complicated here by inconsistent name formatting, common East Asian surnames, and missing author identifiers in older records—beyond the scope of this study. We note without quantification that I-EUS is anchored by a small, densely interconnected cohort of key opinion leaders and that citation-derived metrics should be interpreted with this in mind.

Author-keyword dependence. Keyword co-occurrence analysis depends on author-assigned keywords, whose indexing conventions vary across journals and have shifted over the 24-year window. The applied thesaurus and exclusion list reduce network noise but introduce a degree of analyst-dependent judgment, which we have sought to constrain and document through the prespecified categories, independent dual rating, and full disclosure described in Section 2.4.

Threshold effects. The minimum co-occurrence threshold of 15 necessarily excludes genuinely novel topics that have not yet accumulated sufficient publication volume. Emerging areas—including artificial-intelligence-assisted EUS, EUS-guided portal-vein sampling, and pediatric applications as discussed in Section 4.6—are therefore underrepresented in the network by construction, and their absence should not be read as evidence of absent research activity.

Absence of patient-level data. Bibliometric analysis operates exclusively on bibliographic metadata: authorship, affiliation, journal, keywords, and citation linkages. It does not extract clinical content and consequently cannot report the number of patients treated at each contributing center, procedural success rates, or adverse-event frequencies. Such quantities are obtainable only through systematic review with full-text data extraction—a methodologically distinct undertaking that would additionally require reconciling overlapping patient cohorts reported across multiple publications from the same center, a well-recognized source of double-counting in this literature. The present study should therefore be understood as mapping the structure of the research enterprise, not as summarizing its clinical outcomes; the two approaches are complementary rather than substitutable.

Absence of individual-level research metrics. We deliberately refrained from computing author-level indices such as the h-index. Beyond the practical obstacle of author-record retrieval across several thousand investigators, such analysis is inappropriate on two grounds: institution-level metrics cannot be meaningfully anonymized, since the leading investigators at each of the eleven centers in Table 2 are readily identifiable to any reader familiar with the field; and bibliometric indices are widely acknowledged to be unsuited to evaluating individual researchers, as reflected in the San Francisco Declaration on Research Assessment and the Leiden Manifesto. The unit of analysis here is accordingly the publication, not the investigator.

Citation of postcorpus literature. Several randomized trials and prospective multicenter studies published after 31 December 2024 are cited in the Discussion [18,19,38,45,48] to situate the bibliometric findings within the current state of the evidence. These publications form no part of the analyzed corpus and contribute nothing to the network topology, cluster structure, or citation metrics reported in the Results. They are cited solely to indicate whether the research trajectories identified by the temporal overlay have subsequently been borne out, and readers should not infer from their presence that the analytic window extends beyond 2024.

Descriptive and non-causal design. Finally, as is inherent to the methodology, this analysis identifies temporal associations between keyword trends and research trajectories but cannot establish causal relationships between bibliometric signals and the evolution of clinical practice. The chronological correspondences noted throughout the Discussion—between LAMS commercialization and Phase 3 acceleration, for instance—are interpretive rather than inferential.

### 4.8. Research Priorities and Future Directions

These bibliometric findings highlight five clinical research priorities.

First, consolidation and generalization of the EUS-GE evidence base beyond expert centers. The randomized comparisons the field required have now largely been delivered—against duodenal stenting [35] and against surgical gastroenterostomy [38]—so the outstanding question is no longer whether EUS-GE is efficacious but whether its reported outcomes are reproducible outside the high-volume institutions that generated them. ENDURO’s eligibility requirement of at least twenty prior LAMS placements and ten prior endoscopic gastroenterostomies per participating center [38] quantifies the expertise threshold at which those results were obtained; trials enrolling intermediate-volume centers, with standardized patient-centered endpoints, are the necessary next step [36,49].

Second, extension of EUS-RFA evidence from prospective multicenter cohorts to randomized comparison against surgical resection. The transition from single-institution feasibility series to prospective multicenter evaluation has been accomplished [45], establishing safety and effectiveness in both functional and non-functional pancreatic neuroendocrine tumors. What remains absent is randomized evidence against the established alternative of surgical resection in patients who are operative candidates, together with long-term recurrence data adequate to support a curative-intent claim [44].

Third, international standardization of EUS-GBD patient selection criteria and technical protocols to enable equitable skill dissemination beyond pioneer centers [41], for which the recent international LAMS consensus [12] provides a foundation on which indication-specific protocols can be built.

Fourth, development of structured EUS-PD training frameworks with appropriate case volume and performance benchmarks to expand access to this high-complexity procedure.

Fifth—and following directly from the geographic analysis presented in Section 3.1 and Section 4.1—the establishment of pan-Asian collaborative trial infrastructure. The finding that Asia generates the largest continental research volume while simultaneously exhibiting the highest single-country publication rates identifies a specific and remediable structural inefficiency: substantial regional evidence is being produced in a form that limits its integration into international consensus processes. Multinational Asian consortia, modeled on the European networks that underpin the high international co-authorship rates observed for Italy and Belgium, would convert this volume into proportionate influence over global practice standards—an objective of particular importance given the region’s pancreatobiliary disease burden.

These priorities carry direct implications for training. The concentration of one-third of the corpus within eleven tertiary centers (Table 2) indicates that I-EUS expertise, and the evidence generated from it, remains geographically and institutionally narrow. Three training-related needs follow. First, procedures approaching evidentiary maturity—EUS-BD and transmural drainage of pancreatic fluid collections, which occupy the most heavily published core of the network—now require structured competency frameworks with defined case-volume thresholds and validated performance metrics, so that dissemination to intermediate-volume centers proceeds without erosion of the outcomes established in pioneer institutions. Second, procedures at the temporal frontier—EUS-GE, EUS-GBD, and EUS-RFA—require simulation-based and proctored training pathways developed in parallel with, rather than subsequent to, the ongoing RCT programs, since the historical pattern in this field has been for technique dissemination to outpace formal training infrastructure. Third, the low network density of EUS-PD despite its clinical necessity in patients with failed transpapillary access argues for regional referral networks concentrating these cases at designated centers, rather than for broad dissemination of a procedure whose case volume at any individual institution is insufficient to establish or maintain competence.

As LAMS platform diversity expands and artificial intelligence-assisted EUS image interpretation enters clinical evaluation—with systematic review of pancreatic imaging confirming that convolutional neural network approaches now achieve substantial accuracy in lesion detection, segmentation, and benign-versus-malignant differentiation, while remaining predominantly at the stage of retrospective validation rather than prospective clinical deployment [48]—bibliometric surveillance of this field will serve as a valuable instrument for monitoring the translation of investigational innovation into practice-changing evidence.

## 5. Conclusions

This bibliometric analysis maps the global intellectual architecture of interventional EUS across a 24-year corpus. The United States led national output with the highest rate of international collaboration, whereas Asia was the most productive continent, its influence limited by fragmentation into domestic networks. Eleven tertiary institutions accounted for one-third of the corpus, and annual output showed no contraction during the COVID-19 pandemic. EUS-guided biliary drainage and pancreatic fluid collection management constitute the established, high-volume core of the field, while EUS-guided gastroenterostomy, gallbladder drainage, and radiofrequency ablation represent its most active frontiers. Strategic priorities include multicenter trials extending EUS-GE and EUS-GBD beyond pioneer centers, pan-Asian collaborative consortia, structured training for EUS-RFA and EUS-PD, and continued bibliometric surveillance as AI-assisted imaging and next-generation LAMS platforms mature.

## Figures and Tables

**Table 1:** Top 21 countries and regions by article output (2001–2024).

Country	Articles	Articles %	SCPs	MCPs	MCP %	Continent
USA	625	27.9	519	106	17.0	Americas
Japan	435	19.4	397	38	8.74	Asia
Italy	166	7.42	96	70	42.2	Europe
China	152	6.79	127	25	16.4	Asia
India	151	6.75	127	24	15.9	Asia
South Korea	108	4.83	96	12	11.1	Asia
France	82	3.67	60	22	26.8	Europe
Spain	52	2.32	45	7	13.5	Europe
Germany	44	1.97	35	9	20.5	Europe
United Kingdom	42	1.88	28	14	33.3	Europe
Belgium	35	1.56	19	16	45.7	Europe
Brazil	32	1.43	22	10	31.3	Americas
Canada	30	1.34	18	12	40.0	Americas
Romania	22	0.983	15	7	31.8	Europe
Australia	21	0.939	20	1	4.76	Oceania
Thailand	21	0.939	17	4	19.0	Asia
Portugal	20	0.894	16	4	20.0	Europe
Turkey	17	0.760	15	2	11.8	Asia
Netherlands	15	0.671	11	4	26.7	Europe
Egypt	14	0.626	13	1	7.14	Africa
Switzerland	14	0.626	3	11	78.6	Europe

SCPs, single-country publications (all authors share the same country affiliation); MCPs, multiple-country publications (authors from two or more countries). MCP %, percentage of MCPs among total national output. USA, United States of America. Continental assignment follows the United Nations M49 standard; Turkey is classified as Asia. The 21 countries listed account for 2098 of 2237 records (93.8%); continental totals therefore represent a lower bound.

**Table 2:** Top 11 most productive institutions (2001–2024).

Affiliation	Articles	Articles %	Country
Tokyo Medical University	93	4.16	Japan
University of Ulsan	83	3.71	South Korea
Johns Hopkins University	79	3.53	USA
Harvard University	72	3.22	USA
Osaka Medical and Pharmaceutical University	67	3.00	Japan
Mayo Clinic	63	2.82	USA
Cornell University	61	2.73	USA
Postgraduate Institute of Medical Education and Research	61	2.73	India
University of North Carolina	61	2.73	USA
Catholic University of the Sacred Heart	57	2.55	Italy
University of Tokyo	57	2.55	Japan

Article counts reflect records indexed by the Web of Science Core Collection under the corresponding author’s institutional affiliation. Institutional records were consolidated to account for the separate indexing of universities and their integrated academic medical centers (Harvard University with Harvard University Medical Affiliates; Johns Hopkins University with Johns Hopkins Medicine; Catholic University of the Sacred Heart with IRCCS Policlinico Gemelli; University of North Carolina with University of North Carolina at Chapel Hill; Cornell University with Weill Cornell Medicine; University of Ulsan with Asan Medical Center).

**Table 3:** Top 10 most productive source journals (2001–2024).

Sources	Articles	Articles %	2024 Journal Impact Factor	2024 Journal Citation Indicator	2024 Total Citations	2024 JIF Quartile	Access Model
*Gastrointestinal Endoscopy*	173	7.73	7.5	1.57	24,376	Q1	Hybrid
*Endoscopic Ultrasound*	138	6.17	5.4	0.91	1616	Q1	Full OA
*Endoscopy International Open*	137	6.12	2.3	0.78	3972	Q2	Full OA
*Digestive Endoscopy*	134	5.99	4.7	1.38	5093	Q1	Hybrid
*Endoscopy*	100	4.47	12.8	3.26	13,742	Q1	Hybrid
*Surgical Endoscopy*	95	4.25	2.7	1.22	29,419	Q2	Hybrid
*Clinical Endoscopy*	80	3.58	2.3	0.46	2076	Q2	Full OA
*World Journal of Gastroenterology*	74	3.31	5.4	0.91	50,565	Q1	Full OA
*World Journal of Gastrointestinal Endoscopy*	68	3.04	1.8	0.33	1684	Q3	Full OA
*Journal of Hepato-Biliary-Pancreatic Sciences*	57	2.55	2.8	1.00	5180	Q2	Hybrid

JIF, journal impact factor. Percentages were calculated using the total analyzed corpus (*n* = 2237) as the denominator. Quartile refers to the 2024 Journal Citation Reports ranking within the journal’s primary Web of Science category (Gastroenterology & Hepatology or Surgery).

## Data Availability

The data presented in this study are available in the Supplementary Materials. Supplementary File S1 contains the bibliographic records of the 2237 publications included in the analysis, exported from the Web of Science Core Collection; redistribution of these records is subject to the terms of use of Clarivate Analytics.

## Supplementary Materials

The following supporting information can be downloaded at: https://www.mdpi.com/article/10.3390/clinpract16080153/s1. Supplementary File S1: Dataset of the 2237 records included in the bibliometric analysis; Supplementary File S2: The custom thesaurus used for keyword unification; Supplementary File S3: The full list of excluded terms, annotated by exclusion category.

## Abbreviations

The following abbreviations are used in this manuscript:

EUS	Endoscopic ultrasound
I-EUS	Interventional endoscopic ultrasound
ERCP	Endoscopic retrograde cholangiopancreatography
LAMS	Lumen-apposing metal stent
EUS-BD	EUS-guided biliary drainage
EUS-CDS	EUS-guided choledochoduodenostomy
EUS-AG	EUS-guided antegrade treatment / stenting
EUS-HGS	EUS-guided hepaticogastrostomy
EUS-HJS	EUS-guided hepaticojejunostomy
EUS-RV	EUS-guided rendezvous
EUS-GBD	EUS-guided gallbladder drainage
EUS-GE	EUS-guided gastroenterostomy
EUS-PD	EUS-guided pancreatic duct drainage
EUS-CPN	EUS-guided celiac plexus neurolysis
EUS-CPB	EUS-guided celiac plexus block
EUS-CGN	EUS-guided celiac ganglia neurolysis
EUS-RFA	EUS-guided radiofrequency ablation
EUS-FNI	EUS-guided fine-needle injection
EUS-FNT	EUS-guided fine-needle tattooing
EUS-FNA	EUS-guided fine-needle aspiration
GOO	Gastric outlet obstruction
WON	Walled-off necrosis
RCT	Randomized controlled trial
WoS	Web of Science
SCIE	Science Citation Index Expanded
SCP	Single-country publication
MCP	Multiple-country publication
JIF	Journal impact factor
JCI	Journal citation indicator
APC	Article processing charge
OA	Open access
ESGE	European Society of Gastrointestinal Endoscopy
ASGE	American Society for Gastrointestinal Endoscopy
AGA	American Gastroenterological Association
WSES	World Society of Emergency Surgery
AI	Artificial intelligence
