# Supplementary material for: Interventional Endoscopic Ultrasound in Gastroenterology: A Comprehensive Bibliometric Analysis (2001–2024)
Source: Clin Pract. 2026 Aug 20;16(8):153. doi: 10.3390/clinpract16080153 (PMC13511046; doi:10.3390/clinpract16080153)
Supplement: Supplementary file 1 [file clinpract-16-00153-s001.zip › Supplementary File S1.pdf]

Supplementary File S1. Dataset of the 2,237 records included in the bibliometric analysis

The final analysed corpus retrieved from the Web of Science Core Collection (SCIE) using the full Boolean query reproduced in Section 2.1, executed on 9 May 2026 for the publication window 1 January 2001 to 31 December 2024. These 2,237 records remained after the inclusion and exclusion criteria described in Section 2.2 were applied to the 4,340 records initially identified. Bibliographic fields exported from Web of Science are provided for each record.

PT J
AU Khan, R
Law, R
AF Khan, Rishad
Law, Ryan
TI Therapeutic Endoscopic Ultrasound and Endoscopic Ultrasound-Endoscopic
Retrograde Cholangiopancreatography Interventions
SO GASTROENTEROLOGY CLINICS OF NORTH AMERICA
LA English
DT Article
DE Endoscopic ultrasound; Biliary obstruction; Pancreatic fluid collection;
Gastric outlet obstruction; Lumen-apposing metal stent
ID GUIDED GALLBLADDER DRAINAGE; APPOSING-METAL STENT; LONG-TERM OUTCOMES;
PANCREATIC FLUID COLLECTIONS; DISTAL BILIARY OBSTRUCTION; INTERNATIONAL
MULTICENTER; PERCUTANEOUS DRAINAGE; ACUTE CHOLECYSTITIS;
EUROPEAN-SOCIETY; MANAGEMENT
AB Therapeutic EUS is an innovative and rapidly developing field that allows for internal access to pancreaticobiliary and adjacent luminal structures. While these procedures are demanding and require significant expertise, they can have excellent clinical outcomes and provide valuable treatment options for patients.
C1 [Khan, Rishad] Univ Toronto, Dept Med, Div Gastroenterol & Hepatol, Toronto, ON, Canada.
[Law, Ryan] Mayo Clin, Dept Med, Div Gastroenterol, 200 1st St Southwest, Rochester, MN 55905 USA.
C3 University of Toronto; Mayo Clinic
RP Law, R (corresponding author), Mayo Clin, 200 1st St Southwest, Rochester, MN 55905 USA.
EM dr.ryanlaw@gmail.com
RI Khan, Rishad/I-4961-2019
CR Amato A, 2021, ENDOSCOPY, V53, P1037, DOI 10.1055/a-1324-7919
Anderloni A, 2019, GASTROINTEST ENDOSC, V89, P69, DOI [10.1016/j.gie.2018.04.1746, 10.1016/j.gie.2018.08.047]
Armellini E, 2023, WORLD J GASTROENTERO, V29, P3341, DOI 10.3748/wjg.v29.i21.3341
Arvanitakis M, 2018, ENDOSCOPY, V50, P524, DOI 10.1055/a-0588-5365
Bang JY, 2024, ENDOSCOPY, V56, P184, DOI 10.1055/a-2169-0362
Bang JY, 2020, DIGEST ENDOSC, V32, P298, DOI 10.1111/den.13470
Bang JY, 2019, GUT, V68, P1200, DOI 10.1136/gutjnl-2017-315335
Bang JY, 2018, GASTROINTEST ENDOSC, V88, P9, DOI 10.1016/j.gie.2018.03.012
Bang JY, 2018, ANN SURG, V267, P561, DOI 10.1097/SLA.0000000000002082
Baron TH, 2007, GASTROINTEST ENDOSC, V65, P735, DOI 10.1016/j.gie.2006.07.041

Basiliya K, 2021, ENDOSCOPY, V53, P266, DOI 10.1055/a-1200-0199
Berríos SI, 2017, JAMA SURG, V152, P784, DOI 10.1001/jamasurg.2017.0904
Bhasin DK, 2013, PANCREATOLOGY, V13, P250, DOI 10.1016/j.pan.2013.02.011
Boregowda U, 2023, DIAGNOSTICS, V13, DOI 10.3390/diagnostics13040657
Boxhoorn L, 2021, NEW ENGL J MED, V385, P1372, DOI 10.1056/NEJMoa2100826
Boxhoorn L, 2021, ENDOSCOPY, V53, P136, DOI 10.1055/a-1198-7501
Bronswijk M, 2021, GASTROINTEST ENDOSC, V94, P526, DOI 10.1016/j.gie.2021.04.006
Bronswijk Michiel, 2020, VideoGIE, V5, P442, DOI [10.1016/j.vgie.2020.06.012, 10.1016/j.vgie.2020.06.012]
Carbajo AY, 2020, J CLIN GASTROENTEROL, V54, P1, DOI 10.1097/MCG.0000000000001262
Chan SM, 2017, GASTROINTEST ENDOSC, V85, P1225, DOI 10.1016/j.gie.2016.10.014
Chandan S, 2020, ENDOSC INT OPEN, V08, pE1664, DOI 10.1055/a-1236-3350
Chandrasekhara V, 2021, CLIN GASTROENTEROL H, V19, P2192, DOI 10.1016/j.cgh.2021.05.005
Chandrasekhara V, 2020, ENDOSC INT OPEN, V08, pE1639, DOI 10.1055/a-1243-0092
Chen YI, 2023, GASTROENTEROLOGY, V165, P1249, DOI 10.1053/j.gastro.2023.07.024
Chen Yen-I, 2023, VideoGIE, V8, P500, DOI [10.1016/j.vgie.2023.07.014, 10.1016/j.vgie.2023.07.014]
Chen YI, 2017, GASTROINTEST ENDOSC, V85, P170, DOI 10.1016/j.gie.2016.07.031
Cho SH, 2024, J GASTROEN HEPATOL, V39, P552, DOI 10.1111/jgh.16442
Cho SH, 2020, GASTROINTEST ENDOSC, V91, P543, DOI 10.1016/j.gie.2019.09.042
Choi JH, 2014, ENDOSCOPY, V46, P656, DOI 10.1055/s-0034-1365720
De Cassan C, 2017, ENDOSC ULTRASOUND, V6, P329, DOI 10.4103/2303-9027.209869
Dhindsa BS, 2020, ENDOSC INT OPEN, V8, pE163, DOI 10.1055/a-1067-4411
Dollhopf M, 2017, GASTROINTEST ENDOSC, V86, P636, DOI 10.1016/j.gie.2017.02.027
François E, 2002, GASTROINTEST ENDOSC, V56, P128, DOI 10.1067/mge.2002.125547
Fujii LL, 2013, GASTROINTEST ENDOSC, V78, P854, DOI 10.1016/j.gie.2013.05.016
Garcia-Sumalla A, 2021, SURG ENDOSC, V35, P4873, DOI 10.1007/s00464-021-08435-9
Ghandour B, 2022, GASTROINTEST ENDOSC, V95, P80, DOI 10.1016/j.gie.2021.07.023
Hayat U, 2024, GASTROINTEST ENDOSC, V99, DOI 10.1016/j.gie.2023.10.043
Hedjoudje A, 2019, UNITED EUR GASTROENT, V7, P60, DOI 10.1177/2050640618808147
Hemerly MC, 2023, SURG ENDOSC, V37, P2421, DOI 10.1007/s00464-022-09712-x
Ichkhanian Y, 2020, GASTROINTEST ENDOSC, V91, P1188, DOI 10.1016/j.gie.2019.12.043
Ikeuchi N, 2015, GASTROINTEST ENDOSC, V82, P166, DOI 10.1016/j.gie.2015.01.010
Imai H, 2016, GASTROINTEST ENDOSC, V84, P147, DOI 10.1016/j.gie.2015.12.024
Imoto A, 2020, CLIN ENDOSC, V53, P525, DOI 10.5946/ce.2020.173
Iqbal U, 2020, ENDOSC ULTRASOUND, V9, P16, DOI 10.4103/eus.eus_70_19
Irani S, 2015, GASTROINTEST ENDOSC, V82, P1110, DOI 10.1016/j.gie.2015.05.045
Itoi T, 2013, GASTROINTEST ENDOSC, V78, P934, DOI 10.1016/j.gie.2013.09.025
Itonaga M, 2019, DIGEST ENDOSC, V31, P291, DOI 10.1111/den.13300
James TW, 2020, GASTROINTEST ENDOSC, V91, P537, DOI 10.1016/j.gie.2019.11.017
James TW, 2019, ENDOSC INT OPEN, V7, pE1099, DOI 10.1055/a-0965-6662
Jearth V, 2024, ANN GASTROENTEROL, V37, P1, DOI 10.20524/aog.2023.0854
Jeurnink SM, 2010, GASTROINTEST ENDOSC, V71, P490, DOI 10.1016/j.gie.2009.09.042
Kamal F, 2023, ENDOSC ULTRASOUND, V12, P8, DOI 10.4103/EUS-D-21-00206

Kamata K, 2017, WORLD J GASTROENTERO, V23, P661, DOI 10.3748/wjg.v23.i4.661
Kawakubo K, 2014, J HEPATO-BIL-PAN SCI, V21, P328, DOI 10.1002/jhbp.27
Khashab MA, 2015, GASTROINTEST ENDOSC, V81, P1234, DOI 10.1016/j.gie.2014.12.053
Klair JS, 2023, J CLIN GASTROENTEROL, V57, P211, DOI 10.1097/MCG.0000000000001543
Krafft MR, 2019, DIGEST DIS SCI, V64, P1770, DOI 10.1007/s10620-019-05495-9
Krishnamoorthi R, 2020, SURG ENDOSC, V34, P1904, DOI 10.1007/s00464-020-07409-7
Kwan V, 2007, GASTROINTEST ENDOSC, V66, P582, DOI 10.1016/j.gie.2007.02.065
Ladd AM, 2020, WORLD J GASTRO ENDOS, V12, P149, DOI 10.4253/wjge.v12.i5.149
Law R, 2016, CLIN GASTROENTEROL H, V14, P476, DOI 10.1016/j.cgh.2015.10.026
Lee TH, 2016, CLIN GASTROENTEROL H, V14, P1011, DOI 10.1016/j.cgh.2015.12.032
Manno M, 2020, ENDOSCOPY, V52, pE144, DOI 10.1055/a-1032-8218
Marrache MK, 2021, GASTROINTEST ENDOSC, V93, P34, DOI 10.1016/j.gie.2020.06.057
Marshall GT, 1996, ARCH SURG-CHICAGO, V131, P278
Miller C, 2023, ENDOSC INT OPEN, V11, pE660, DOI 10.1055/a-2098-2570
Miller CS, 2020, ENDOSCOPY, V52, P151, DOI 10.1055/a-0991-7530
Miyano A, 2018, J GASTROINTEST SURG, V22, P1312, DOI 10.1007/s11605-018-3758-1
Mori Y, 2018, J HEPATO-BIL-PAN SCI, V25, P87, DOI 10.1002/jhbp.504
Osman Karim T, 2023, IGIE, V2, P324, DOI [10.1016/j.igie.2023.07.003, 10.1016/j.igie.2023.07.003]
Perez-Miranda M, 2018, ENDOSC ULTRASOUND, V7, P79, DOI 10.4103/eus.eus_5_18
Pisano M, 2020, WORLD J EMERG SURG, V15, DOI 10.1186/s13017-020-00336-x
Ramouz A, 2022, SURG ENDOSC, V36, P3708, DOI 10.1007/s00464-022-09137-6
Rana SS, 2021, ANN GASTROENTEROL, V34, P142, DOI 10.20524/aog.2020.0568
Runge TM, 2021, ENDOSCOPY, V53, P611, DOI 10.1055/a-1254-3942
Sharaiha RZ, 2017, GASTROINTEST ENDOSC, V85, P904, DOI 10.1016/j.gie.2016.12.023
Shinn B, 2021, GASTROINTEST ENDOSC, V94, P727, DOI 10.1016/j.gie.2021.04.022
Siddiqui A, 2019, SURG ENDOSC, V33, P1260, DOI 10.1007/s00464-018-6406-7
Szakó L, 2020, PANCREATOLOGY, V20, P132, DOI 10.1016/j.pan.2019.10.006
Teoh AY, 2019, ENDOSC INT OPEN, V7, pE964, DOI 10.1055/a-0915-2098
Teoh AYB, 2020, GUT, V69, P1085, DOI 10.1136/gutjnl-2019-319996
Teoh AYB, 2023, GASTROENTEROLOGY, V165, P473, DOI 10.1053/j.gastro.2023.04.016
Teoh AYB, 2021, GASTROINTEST ENDOSC, V93, P577, DOI 10.1016/j.gie.2020.06.066
Teoh AYB, 2014, GASTROINTEST ENDOSC, V80, P1171, DOI 10.1016/j.gie.2014.03.038
Tsuchiya T, 2018, GASTROINTEST ENDOSC, V87, P1138, DOI 10.1016/j.gie.2017.08.017
Tsuchiya T, 2016, DIGEST ENDOSC, V28, P96, DOI 10.1111/den.12611
Tyberg A, 2023, CLIN TRANSL GASTROEN, V14, DOI 10.14309/ctg.0000000000000593
Tyberg A, 2016, GASTROINTEST ENDOSC, V83, P254, DOI 10.1016/j.gie.2015.08.003
Umeda J, 2015, GASTROINTEST ENDOSC, V82, P390, DOI 10.1016/j.gie.2015.02.041
van der Merwe SW, 2022, ENDOSCOPY, V54, P185, DOI 10.1055/a-1717-1391
van Santvoort HC, 2011, GASTROENTEROLOGY, V141, P1254, DOI 10.1053/j.gastro.2011.06.073
van Wanrooij RLJ, 2022, ENDOSCOPY, V54, P310, DOI 10.1055/a-1738-6780
Vanek P, 2023, GASTROINTEST ENDOSC, V97, P1070, DOI 10.1016/j.gie.2022.12.026
Veitch AM, 2021, ENDOSCOPY, V53, P947, DOI 10.1055/a-1547-2282

Venkatesan AM, 2010, J VASC INTERV RADIOL, V21, P1611, DOI 10.1016/j.jvir.2010.07.018
Wiersema MJ, 1996, GASTROINTEST ENDOSC, V44, P614, DOI 10.1016/S0016-5107(96)70022-6
Yamamoto Y, 2020, ENDOSC ULTRASOUND, V9, P110, DOI 10.4103/eus.eus_68_19
Yamazaki H, 2024, DEN OPEN, V4, DOI 10.1002/deo2.274
Yang MJ, 2020, DIGEST DIS, V38, P542, DOI 10.1159/000505644
NR 99
TC 0
Z9 0
U1 0
U2 1
PU W B SAUNDERS CO-ELSEVIER INC
PI PHILADELPHIA
PA 1600 JOHN F KENNEDY BOULEVARD, STE 1800, PHILADELPHIA, PA 19103-2899 USA
SN 0889-8553
EI 1558-1942
J9 GASTROENTEROL CLIN N
JI Gastroenterol. Clin. North Am.
PD DEC
PY 2024
VL 53
IS 4
BP 683
EP 707
DI 10.1016/j.gtc.2024.08.016
EA NOV 2024
PG 25
WC Gastroenterology & Hepatology
WE Science Citation Index Expanded (SCI-EXPANDED)
SC Gastroenterology & Hepatology
GA L4J7M
UT WOS:001350403900001
PM 39489582
DA 2026-05-09
ER

PT J
AU Mussetto, A
Fugazza, A
Fuccio, L
Triossi, O
Repici, A
Anderloni, A

AF Mussetto, Alessandro
Fugazza, Alessandro
Fuccio, Lorenzo
Triossi, Omero
Repici, Alessandro
Anderloni, Andrea
TI Current uses and outcomes of lumen-apposing metal stents
SO ANNALS OF GASTROENTEROLOGY
LA English
DT Review
DE Lumen-apposing metal stents; pancreatic fluid collections; endoscopic ultrasound-guided biliary drainage; endoscopic ultrasound-guided gastroenterostomy
ID PANCREATIC FLUID COLLECTIONS; EUS-GUIDED DRAINAGE; DISTAL BILIARY OBSTRUCTION; WALLED-OFF NECROSIS; GALLBLADDER DRAINAGE; DELIVERY-SYSTEM; MULTICENTER; CHOLEDOCHODUODENOSTOMY; GASTROJEJUNOSTOMY; EXPERIENCE
AB The lumen-apposing metal stent (LAMS) is one of the revolutionary devices recently developed for gastrointestinal endoscopy. This device has a saddle-shaped design and large lumen. It was originally designed for drainage of transmural pancreatic fluid collection and in the last few years it has been used extensively for that indication. More recently, other in- and off-label indications have been proposed. Several types of LAMS are available, with or without an electrocautery-enhanced delivery system. In the current review we discuss the state of the art with regard to LAMS and their indications, usage, and outcomes.
C1 [Mussetto, Alessandro; Triossi, Omero] S Maria Croci Hosp, Gastroenterol Unit, Ravenna, Italy.
[Fugazza, Alessandro; Repici, Alessandro; Anderloni, Andrea] Humanitas Res Hosp, Div Gastroenterol, Digest Endoscopy Unit, Milan, Italy.
[Fuccio, Lorenzo] Univ Bologna, S Orsola Malpighi Hosp, Dept Clin Med, Bologna, Italy.
C3 IRCCS Azienda Ospedaliero-Universitaria di Bologna; University of Bologna
RP Mussetto, A (corresponding author), Osped Santa Maria Croci, Viale Randi 5, I-48100 Ravenna, Italy.
EM alessandromussetto@gmail.com
RI Anderloni, Andrea/ABE-9139-2020; Mussetto, Alessandro/GVU-0673-2022; Fugazza, Alessandro/ABG-9381-2021; Repici, Alessandro/HFH-8162-2022; Fuccio, Lorenzo/A-2557-2010
OI Anderloni, Andrea/0000-0002-1021-0031; Mussetto, Alessandro/0000-0001-8140-4223; Fugazza, Alessandro/0000-0003-0485-4903; Repici, Alessandro/0000-0002-1621-6450; Fuccio, Lorenzo/0000-0001-8618-2447
CR Anderloni A, 2016, ENDOSCOPY, V48, pE321, DOI 10.1055/s-0042-117425
Bang JY, 2017, GUT, V66, P2054, DOI 10.1136/gutjnl-2016-312812
Binmoeller KF, 2011, ENDOSCOPY, V43, P337, DOI 10.1055/s-0030-1256127
Dollhopf M, 2017, GASTROINTEST ENDOSC, V86, P636, DOI 10.1016/j.gie.2017.02.027
Han D, 2017, J CLIN GASTROENTEROL

Imai H, 2016, GASTROINTEST ENDOSC, V84, P147, DOI 10.1016/j.gie.2015.12.024
Irani S, 2017, CLIN GASTROENTEROL H, V15, P738, DOI 10.1016/j.cgh.2016.12.021
Irani S, 2015, GASTROINTEST ENDOSC, V82, P1110, DOI 10.1016/j.gie.2015.05.045
Itoi T, 2014, GASTROINTEST ENDOSC, V79, P715, DOI 10.1016/j.gie.2013.11.021
Khashab MA, 2017, ENDOSC INT OPEN, V5, pE275, DOI 10.1055/s-0043-101695
Kunda R, 2016, SURG ENDOSC, V30, P5002, DOI 10.1007/s00464-016-4845-6
Lang GD, 2018, GASTROINTEST ENDOSC, V87, P150, DOI 10.1016/j.gie.2017.06.029
Law ST, 2018, SURG ENDOSC, V32, P2448, DOI 10.1007/s00464-017-5946-6
Mudireddy PR, 2018, GASTROINTEST ENDOSC, V87, P1256, DOI 10.1016/j.gie.2017.08.011
Ngamruengphong S, 2017, ENDOSCOPY, V49, P549, DOI 10.1055/s-0043-105072
Rinninella E, 2015, GASTROINTEST ENDOSC, V82, P1039, DOI 10.1016/j.gie.2015.04.006
Samuelson AL, 2012, GASTROENTEROL CLIN N, V41, P47, DOI 10.1016/j.gtc.2011.12.007
Shah RJ, 2015, CLIN GASTROENTEROL H, V13, P747, DOI 10.1016/j.cgh.2014.09.047
Sharaiha RZ, 2016, CLIN GASTROENTEROL H, V14, P1797, DOI 10.1016/j.cgh.2016.05.011
Siddiqui AA, 2017, GASTROINTEST ENDOSC, V85, P758, DOI 10.1016/j.gie.2016.08.014
Siddiqui AA, 2016, GASTROINTEST ENDOSC, V83, P699, DOI 10.1016/j.gie.2015.10.020
Teoh AYB, 2017, ENDOSCOPY, V49, P130, DOI 10.1055/s-0042-119036
Teoh AYB, 2014, GASTROINTEST ENDOSC, V80, P1171, DOI 10.1016/j.gie.2014.03.038
Tsuchiya T, 2018, GASTROINTEST ENDOSC, V87, P1138, DOI 10.1016/j.gie.2017.08.017
Tyberg A, 2016, ENDOSC INT OPEN, V4, pE276, DOI 10.1055/s-0042-101789
Walter D, 2016, GUT, V65, P6, DOI 10.1136/gutjnl-2015-309925
Walter D, 2015, ENDOSCOPY, V47, P63, DOI 10.1055/s-0034-1378113
Yang D, 2017, ENDOSCOPY, V49, P327, DOI 10.1055/s-0042-122779
Yoo J, 2017, ENDOSC ULTRASOUND, V6, P131, DOI 10.4103/2303-9027.204814
NR 29
TC 85
Z9 94
U1 0
U2 2
PU HELLENIC SOC GASTROENTEROLOGY
PI ATHENS
PA DEMOKRATIAS AVE 67, ATHENS, 15451, GREECE
SN 1108-7471
EI 1792-7463
J9 ANN GASTROENTEROL
JI Ann. Gastroenterol.
PY 2018
VL 31
IS 5
BP 535
EP 540
DI 10.20524/aog.2018.0287

PG 6
WC Gastroenterology & Hepatology
WE Emerging Sources Citation Index (ESCI)
SC Gastroenterology & Hepatology
GA GR6QI
UT WOS:000442781700002
PM 30174389
OA Green Submitted, gold
DA 2026-05-09
ER

PT J
AU Jearth, V
Sundaram, S
Kale, A
Sachan, A
Rana, SS
AF Jearth, Vaneet
Sundaram, Sridhar
Kale, Aditya
Sachan, Anurag
Rana, Surinder Singh
TI Current paradigm of endoscopic ultrasound in biliary and pancreatic duct
drainage: an update
SO ANNALS OF GASTROENTEROLOGY
LA English
DT Review
DE Endosonography; pancreatic duct; gallbladder; self-expanding metal
stent; lumen-apposing metal stent
ID EXPANDABLE METAL STENT; GUIDED GALLBLADDER DRAINAGE; SURGICALLY ALTERED
ANATOMY; FAILED ERCP; PERCUTANEOUS CHOLECYSTOSTOMY; INTERNATIONAL
MULTICENTER; BILE-DUCT; EUS; CHOLEDOCHODUODENOSTOMY; OBSTRUCTION
AB Endoscopic retrograde cholangiopancreatography (ERCP) is the workhorse for biliary and pancreatic ductal interventions. Despite advances in both endoscopes and accessories for ERCP, it still has limitations in the presence of altered anatomy, luminal obstruction hindering access to the papilla, and proximal duct obstructions by tight stricture, calculi or intraductal growth. Endoscopic ultrasound-guided biliary drainage (EUS-BD) and EUS-guided pancreatic duct drainage (EUS-PDD) have expanded the rescue procedures after failed ERCP. This review discusses the techniques and results of various EUS-BD procedures, as well as EUS-PDD.
C1 [Jearth, Vaneet; Sundaram, Sridhar; Sachan, Anurag; Rana, Surinder Singh] PGIMER, Dept Gastroenterol, Chandigarh, India.
[Kale, Aditya] Tata Mem Hosp, Dept Digest Dis & Clin Nutr, Mumbai, Maharashtra, India.
[Rana, Surinder Singh] PGIMER, Dept Gastroenterol, Chandigarh 160012, India.

C3 Post Graduate Institute of Medical Education & Research (PGIMER),
Chandigarh; Tata Memorial Centre (TMC); Tata Memorial Hospital; Post
Graduate Institute of Medical Education & Research (PGIMER), Chandigarh
RP Rana, SS (corresponding author), PGIMER, Dept Gastroenterol, Chandigarh 160012, India.
EM drsurinderrana@yahoo.co.in
RI S, Sridhar/R-6416-2017; Sachan, Anurag/AAI-6504-2021; Kale,
Aditya/KHW-7676-2024
CR Abdelqader A, 2022, DIGEST DIS SCI, V67, P1649, DOI 10.1007/s10620-022-07423-w
ADAMS DB, 1994, ANN SURG, V219, P481, DOI 10.1097/00000658-199405000-00006
Ahmed O, 2018, SURG ENDOSC, V32, P1627, DOI 10.1007/s00464-018-6041-3
Anderloni A, 2019, GASTROINTEST ENDOSC, V89, P69, DOI [10.1016/j.gie.2018.04.1746, 10.1016/j.gie.2018.08.047]
Artifon ELA, 2012, J CLIN GASTROENTEROL, V46, P768, DOI 10.1097/MCG.0b013e31825f264c
Artifon ELA, 2011, J PANCREAS, V12, P610
Attasaranya S, 2012, GASTROENT RES PRACT, V2012, DOI 10.1155/2012/680753
Bhurwal A, 2021, PANCREATOLOGY, V21, P990, DOI 10.1016/j.pan.2021.03.021
Bories E, 2007, ENDOSCOPY, V39, P287, DOI 10.1055/s-2007-966212
Burmester E, 2003, GASTROINTEST ENDOSC, V57, P246, DOI 10.1067/mge.2003.85
Cahen DL, 2007, NEW ENGL J MED, V356, P676, DOI 10.1056/NEJMoa060610
Chandan S, 2020, ENDOSC INT OPEN, V08, pE1664, DOI 10.1055/a-1236-3350
Chen YI, 2017, GASTROINTEST ENDOSC, V85, P170, DOI 10.1016/j.gie.2016.07.031
Choi JH, 2014, ENDOSCOPY, V46, P656, DOI 10.1055/s-0034-1365720
Coté GA, 2012, CLIN GASTROENTEROL H, V10, P920, DOI 10.1016/j.cgh.2012.02.019
Dalal A, 2020, DIGEST ENDOSC, V32, P409, DOI 10.1111/den.13504
Dalsania Raj, 2020, Curr Gastroenterol Rep, V22, P34, DOI [10.1007/s11894-020-00772-4, 10.1007/s11894-020-00772-4]
Sanz MD, 2021, SURG ENDOSC, V35, P6754, DOI 10.1007/s00464-020-08179-y
Dhir V, 2013, UNITED EUR GASTROENT, V1, P103, DOI 10.1177/2050640613480145
Dhir V, 2012, GASTROINTEST ENDOSC, V75, P354, DOI 10.1016/j.gie.2011.07.075
Doyle JB, 2023, J CLIN MED, V12, DOI 10.3390/jcm12072736
El Chafic AH, 2019, DIGEST DIS SCI, V64, P3321, DOI 10.1007/s10620-019-05688-2
Fujii LL, 2013, GASTROINTEST ENDOSC, V78, P851
Giovannini M, 2001, ENDOSCOPY, V33, P898, DOI 10.1055/s-2001-17324
Giri S, 2023, GASTROINTEST ENDOSC, V98, DOI 10.1016/j.gie.2023.06.055
GOBIEN RP, 1983, AM J ROENTGENOL, V141, P795, DOI 10.2214/ajr.141.4.795
Hara K, 2013, ENDOSCOPY, V45, P392, DOI 10.1055/s-0032-1326076
Hara K, 2011, AM J GASTROENTEROL, V106, P1239, DOI 10.1038/ajg.2011.84
Honjo M, 2018, ENDOSC ULTRASOUND, V7, P376, DOI 10.4103/eus.eus_2_18
Hosmer A, 2018, ENDOSC INT OPEN, V6, pE127, DOI 10.1055/s-0043-123188
Imoto A, 2020, CLIN ENDOSC, V53, P525, DOI 10.5946/ce.2020.173
Irani S, 2017, CLIN GASTROENTEROL H, V15, P738, DOI 10.1016/j.cgh.2016.12.021
Issa D, 2021, ENDOSCOPY, V53, P827, DOI 10.1055/a-1259-0349

Iwashita T, 2012, ENDOSCOPY, V44, P60, DOI 10.1055/s-0030-1256871
Iwashita T, 2020, J HEPATO-BIL-PAN SCI, V27, P968, DOI 10.1002/jhbp.823
Iwashita T, 2016, GASTROINTEST ENDOSC, V83, P394, DOI 10.1016/j.gie.2015.04.043
Iwashita T, 2014, CLIN J GASTROENTEROL, V7, P94, DOI 10.1007/s12328-014-0467-5
Iwashita T, 2013, DIGEST DIS SCI, V58, P2417, DOI 10.1007/s10620-013-2645-6
Jacques J, 2019, ENDOSCOPY, V51, P540, DOI 10.1055/a-0735-9137
Kahaleh M, 2006, GASTROINTEST ENDOSC, V64, P52, DOI 10.1016/j.gie.2006.01.063
Kawakubo K, 2014, J HEPATO-BIL-PAN SCI, V21, P328, DOI 10.1002/jhbp.27
Khashab MA, 2016, ENDOSC INT OPEN, V4, pE1322, DOI 10.1055/s-0042-110790
Khashab MA, 2016, ENDOSC INT OPEN, V4, pE175, DOI 10.1055/s-0041-109083
Khashab MA, 2013, GASTROINTEST ENDOSC, V78, P734, DOI 10.1016/j.gie.2013.05.013
Kogure H, 2021, DIGEST ENDOSC, V33, P441, DOI 10.1111/den.13746
Komaki T, 2011, PANCREATOLOGY, V11, P47, DOI 10.1159/000323508
Kongkam P, 2021, ENDOSCOPY, V53, P55, DOI 10.1055/a-1195-8197
Krafft MR, 2019, DIGEST DIS SCI, V64, P1770, DOI 10.1007/s10620-019-05495-9
Law R, 2016, CLIN GASTROENTEROL H, V14, P476, DOI 10.1016/j.cgh.2015.10.026
Le Bian AZ, 2018, J GASTROINTEST SURG, V22, P2021, DOI 10.1007/s11605-018-3859-x
Li JS, 2022, FRONT SURG, V9, DOI 10.3389/fsurg.2022.811005
Mallery S, 2004, GASTROINTEST ENDOSC, V59, P100, DOI 10.1016/S0016-5107(03)02300-9
Maranki J, 2009, ENDOSCOPY, V41, P532, DOI 10.1055/s-0029-1214712
Matsubara S, 2022, J CLIN MED, V11, DOI 10.3390/jcm11061591
Matsumoto S, 2020, DIGEST ENDOSC, V32, P957, DOI 10.1111/den.13620
Minaga K, 2017, SURG ENDOSC, V31, P4764, DOI 10.1007/s00464-017-5553-6
Mukai S, 2019, GASTROINTEST ENDOSC, V89, P399, DOI 10.1016/j.gie.2018.07.030
Nakai Y, 2019, DIGEST ENDOSC, V31, P65, DOI 10.1111/den.13333
Nakai Y, 2019, CLIN ENDOSC, V52, P212, DOI 10.5946/ce.2018.188
Nakai Y, 2019, SAUDI J GASTROENTERO, V25, P210, DOI 10.4103/sjg.SJG_474_18
Nakai Y, 2017, ENDOSCOPY, V49, P983, DOI 10.1055/s-0043-113444
Nguyen-Tang T, 2010, ENDOSCOPY, V42, P232, DOI 10.1055/s-0029-1243858
Ogura T, 2021, DEN OPEN, V1, DOI 10.1002/deo2.8
Ogura T, 2021, ENDOSCOPY, V53, P369, DOI 10.1055/a-1199-5418
Ogura T, 2016, ENDOSCOPY, V48, P156, DOI 10.1055/s-0034-1392859
Ogura T, 2015, WORLD J GASTROENTERO, V21, P820, DOI 10.3748/wjg.v21.i3.820
Oh D, 2020, J GASTROEN HEPATOL, V35, P994, DOI 10.1111/jgh.14897
Oh D, 2017, THER ADV GASTROENTER, V10, P42, DOI 10.1177/1756283X16671671
Oh D, 2016, GASTROINTEST ENDOSC, V83, P366, DOI 10.1016/j.gie.2015.07.015
Park DH, 2013, GASTROINTEST ENDOSC, V78, P91, DOI 10.1016/j.gie.2013.01.042
Park DH, 2012, GASTROINTEST ENDOSC, V75, P692, DOI 10.1016/j.gie.2011.04.013
Park DH, 2011, GASTROINTEST ENDOSC, V74, P1276, DOI 10.1016/j.gie.2011.07.054
Park SJ, 2013, GASTROINTEST ENDOSC, V78, P374, DOI 10.1016/j.gie.2013.04.183
Poincloux L, 2015, ENDOSCOPY, V47, P794, DOI [10.1055/s-0035-1545399, 10.1055/s-0034-1391988]
Rai P, 2018, ENDOSC INT OPEN, V6, pE67, DOI 10.1055/s-0043-120664

Rana SS, 2021, ANN GASTROENTEROL, V34, P142, DOI 10.20524/aog.2020.0568
Rana SS, 2022, J DIG ENDOSC, V13, P82, DOI 10.1055/s-0042-1743519
Rana SS, 2020, ENDOSC ULTRASOUND, V9, P274, DOI 10.4103/eus.eus_46_20
Rana SS, 2019, PANCREATOLOGY, V19, P608, DOI 10.1016/j.pan.2019.05.454
Rerknimitr R, 2013, J GASTROEN HEPATOL, V28, P593, DOI 10.1111/jgh.12128
Salerno R, 2019, WORLD J GASTRO ENDOS, V11, P354, DOI 10.4253/wjge.v11.i5.354
Shah JN, 2012, GASTROINTEST ENDOSC, V75, P56, DOI 10.1016/j.gie.2011.08.032
Sundaram S, 2023, WORLD J GASTRO ENDOS, V15, P122, DOI 10.4253/wjge.v15.i3.122
Sundaram S, 2023, DIGEST DIS SCI, V68, P1551, DOI 10.1007/s10620-022-07655-w
Sundaram S, 2021, ENDOSC ULTRASOUND, V10, P154, DOI 10.4103/EUS-D-21-00004
Teh JL, 2023, J CLIN MED, V12, DOI 10.3390/jcm12041626
Teoh AYB, 2023, GASTROENTEROLOGY, V165, P473, DOI 10.1053/j.gastro.2023.04.016
Tsuchiya T, 2016, DIGEST ENDOSC, V28, P96, DOI 10.1111/den.12611
Tyberg Amy, 2023, J Clin Gastroenterol, V57, P962, DOI 10.1097/MCG.0000000000001795
Tyberg A, 2020, ENDOSC ULTRASOUND, V9, P175, DOI 10.4103/eus.eus_3_20
Tyberg A, 2018, J CLIN GASTROENTEROL, V52, P79, DOI 10.1097/MCG.0000000000000786
Uchida D, 2018, CAN J GASTROENTEROL, V2018, DOI 10.1155/2018/8216109
Uemura RS, 2018, J CLIN GASTROENTEROL, V52, P123, DOI 10.1097/MCG.0000000000000948
van der Merwe SW, 2022, ENDOSCOPY, V54, P185, DOI 10.1055/a-1717-1391
Vanella G, 2023, DIGEST ENDOSC, V35, P377, DOI 10.1111/den.14445
Vila JJ, 2012, GASTROINTEST ENDOSC, V76, P1133, DOI 10.1016/j.gie.2012.08.001
Wiersema MJ, 1996, GASTROINTEST ENDOSC, V43, P102, DOI 10.1016/S0016-5107(06)80108-2
Will U, 2015, WORLD J GASTROENTERO, V21, P13140, DOI 10.3748/wjg.v21.i46.13140
NR 98
TC 5
Z9 5
U1 2
U2 10
PU HELLENIC SOC GASTROENTEROLOGY
PI ATHENS
PA DEMOKRATIAS AVE 67, ATHENS, 15451, GREECE
SN 1108-7471
EI 1792-7463
J9 ANN GASTROENTEROL
JI Ann. Gastroenterol.
PY 2024
VL 37
IS 1
BP 1
EP 14
DI 10.20524/aog.2023.0854
EA DEC 2024

PG 14
WC Gastroenterology & Hepatology
WE Emerging Sources Citation Index (ESCI)
SC Gastroenterology & Hepatology
GA IE7O9
UT WOS:001136322700001
PM 38223246
OA Green Submitted, gold
DA 2026-05-09
ER

PT J
AU Radlinski, MJ
Strand, DS
Shami, VM
AF Radlinski, Mark J.
Strand, Daniel S.
Shami, Vanessa M.
TI Evolution of interventional endoscopic ultrasound
SO GASTROENTEROLOGY REPORT
LA English
DT Review
DE endoscopic ultrasound; interventional endoscopic ultrasound; biliary
drainage; gastrojejunostomy; pancreatic pseudocyst drainage
ID APPOSING METAL STENT; PRESSURE-GRADIENT MEASUREMENT; EUS-GUIDED
GASTROJEJUNOSTOMY; GASTRIC OUTLET OBSTRUCTION; TRANSMURAL DRAINAGE;
BILIARY DRAINAGE; LAPAROSCOPIC GASTROJEJUNOSTOMY; PANCREATIC
PSEUDOCYSTS; GALLBLADDER DRAINAGE; CLINICAL-EXPERIENCE
AB Endoscopic ultrasound (EUS) has become an indispensable modality for the assessment of the gastrointestinal tract and adjacent structures since its origin in the 1980s. Following the development of the linear echoendoscope, EUS has evolved from a purely diagnostic modality to a sophisticated tool for intervention, with numerous luminal, pancreaticobiliary, and hepatic applications. Broadly, these applications may be subdivided into three categories: transluminal drainage or access procedures, injection therapy, and EUS-guided liver interventions. Transluminal drainage or access procedures include management of pancreatic fluid collection, EUS-guided biliary drainage, EUS-guided bile duct drainage, EUS-guided pancreatic duct drainage, and enteral anastomosis formation. Injection therapies include therapeutic EUS-guided injections for management of malignancies accessible by EUS. EUS-guided liver applications include EUS-guided liver biopsy, EUS-guided portal pressure gradient measurement, and EUS-guided vascular therapies. In this review, we discuss the origins of each of these EUS applications, evolution of techniques leading to the current status, and future directions of EUS-guided interventional therapy.
C1 [Radlinski, Mark J.; Strand, Daniel S.; Shami, Vanessa M.] Univ Virginia, Div Gastroenterol & Hepatol, Charlottesville, VA USA.
[Shami, Vanessa M.] Univ Virginia Hlth Syst, Div Gastroenterol & Hepatol, Charlottesville, VA 22908 USA.

C3 University of Virginia; University of Virginia; University of Virginia
(UVA) Health System
RP Shami, VM (corresponding author), Univ Virginia Hlth Syst, Div Gastroenterol & Hepatol, Charlottesville, VA 22908 USA.
EM VMS4E@uvahealth.org
RI Strand, Daniel/GNP-4891-2022; Shami, Vanessa/LCD-7308-2024
OI Strand, Daniel/0000-0001-5573-6291;
CR Attili F, 2015, ENDOSCOPY, V47, pE619, DOI 10.1055/s-0034-1393669
Bang JY, 2021, CLIN GASTROENTEROL H, V19, P1275, DOI 10.1016/j.cgh.2020.07.022
Bang JY, 2019, GUT, V68, P1200, DOI 10.1136/gutjnl-2017-315335
Bang JY, 2017, DIGEST ENDOSC, V29, P83, DOI 10.1111/den.12681
Baron TH, 1996, GASTROENTEROLOGY, V111, P755, DOI 10.1053/gast.1996.v111.pm8780582
Barthet M, 2015, GASTROINTEST ENDOSC, V81, P215, DOI 10.1016/j.gie.2014.09.039
Basiliya K, 2021, ENDOSCOPY, V53, P266, DOI 10.1055/a-1200-0199
Bick BL, 2019, SURG ENDOSC, V33, P1837, DOI 10.1007/s00464-018-6462-z
Binmoeller KF, 2011, ENDOSCOPY, V43, P337, DOI 10.1055/s-0030-1256127
BINMOELLER KF, 1995, GASTROINTEST ENDOSC, V42, P219, DOI 10.1016/S0016-5107(95)70095-1
BORIE D, 1994, PRESSE MED, V23, P1064
Bronswijk M, 2021, GASTROINTEST ENDOSC, V94, P526, DOI 10.1016/j.gie.2021.04.006
Ching-Companioni RA, 2019, ENDOSCOPY, V51, P1059, DOI 10.1055/a-0956-6922
Choi JH, 2014, ENDOSCOPY, V46, P656, DOI 10.1055/s-0034-1365720
DeWitt J, 2009, GASTROINTEST ENDOSC, V69, P535, DOI 10.1016/j.gie.2008.09.056
Di Mitri R, 2022, SURG ENDOSC, V36, P4553, DOI 10.1007/s00464-021-08808-0
DIMAGNO EP, 1980, LANCET, V1, P629
El Chafic AH, 2019, DIGEST DIS SCI, V64, P3321, DOI 10.1007/s10620-019-05688-2
Falque A, 2021, ENDOSC INT OPEN, V09, pE934, DOI 10.1055/a-1302-1484
François E, 2002, GASTROINTEST ENDOSC, V56, P128, DOI 10.1067/mge.2002.125547
Fujii-Lau LL, 2014, ENDOSCOPY, V46, pE654, DOI 10.1055/s-0034-1390845
Gan SI, 2005, GASTROINTEST ENDOSC, V61, P746, DOI 10.1016/S0016-5107(05)00320-2
Gupta K, 2014, J CLIN GASTROENTEROL, V48, P80, DOI 10.1097/MCG.0b013e31828c6822
Henry Z, 2021, CLIN GASTROENTEROL H, V19, P1098, DOI 10.1016/j.cgh.2021.01.027
Huang JY, 2017, GASTROINTEST ENDOSC, V85, P996, DOI 10.1016/j.gie.2016.09.026
Irani S, 2015, GASTROINTEST ENDOSC, V82, P1110, DOI 10.1016/j.gie.2015.05.045
Itoi T, 2016, GASTROINTEST ENDOSC, V83, P461, DOI 10.1016/j.gie.2015.08.030
Itoi T, 2013, DIGEST ENDOSC, V25, P241, DOI 10.1111/den.12048
Jeurnink SM, 2010, GASTROINTEST ENDOSC, V71, P490, DOI 10.1016/j.gie.2009.09.042
Kahaleh M, 2006, ENDOSCOPY, V38, P355, DOI 10.1055/s-2006-925249
Karstensen JG, 2023, GUT, V72, P1167, DOI 10.1136/gutjnl-2022-328225
Kedia P, 2015, ENDOSCOPY, V47, P159, DOI 10.1055/s-0034-1390771
Khashab MA, 2017, ENDOSC INT OPEN, V5, pE275, DOI 10.1055/s-0043-101695
Khashab MA, 2015, GASTROINTEST ENDOSC, V82, P932, DOI 10.1016/j.gie.2015.06.017
Kouanda A, 2021, SURG ENDOSC, V35, P7058, DOI 10.1007/s00464-020-08221-z

KOZAREK RA, 1985, GASTROINTEST ENDOSC, V31, P322, DOI 10.1016/S0016-5107(85)72215-8
Krafft MR, 2019, DIGEST DIS SCI, V64, P1770, DOI 10.1007/s10620-019-05495-9
Kunda R, 2016, SURG ENDOSC, V30, P5002, DOI 10.1007/s00464-016-4845-6
Lahoti S, 2000, GASTROINTEST ENDOSC, V51, P331, DOI 10.1016/S0016-5107(00)70363-4
Lopes CV, 2007, SCAND J GASTROENTERO, V42, P524, DOI 10.1080/00365520601065093
Mehta S, 2006, SURG ENDOSC, V20, P239, DOI 10.1007/s00464-005-0130-9
Minata MK, 2016, ENDOSC INT OPEN, V4, pE1158, DOI 10.1055/s-0042-115935
Mohan BP, 2019, Gastrointest Endosc, V89
Moyer MT, 2017, GASTROENTEROLOGY, V153, P1295, DOI 10.1053/j.gastro.2017.08.009
Mukai S, 2015, ENDOSCOPY, V47, P47, DOI 10.1055/s-0034-1377966
Nennstiel S, 2015, J CLIN GASTROENTEROL, V49, P764, DOI 10.1097/MCG.0000000000000275
Paik Yong-Han, 2008, Korean J Hepatol, V14, P136, DOI 10.3350/kjhep.2008.14.2.136
Park DH, 2009, ENDOSCOPY, V41, P842, DOI 10.1055/s-0029-1215133
Pawa R, 2022, DIGEST ENDOSC, V34, P1234, DOI 10.1111/den.14266
Peery AF, 2022, GASTROENTEROLOGY, V162, P621, DOI 10.1053/j.gastro.2021.10.017
Perez-Miranda M, 2017, J CLIN GASTROENTEROL, V51, P896, DOI 10.1097/MCG.0000000000000887
Pizzicannella M, 2020, ENDOSC ULTRASOUND, V9, P45, DOI 10.4103/eus.eus_55_19
Prakash S, 2022, ENDOSCOPY, V54, P52, DOI 10.1055/a-1376-2394
Puga M, 2018, ENDOSCOPY, V50, P1022, DOI 10.1055/a-0582-9127
Robles-Medranda C, 2020, ENDOSCOPY, V52, P268, DOI 10.1055/a-1123-9054
ROGERS BHG, 1975, GASTROINTEST ENDOSC, V21, P133
SAHEL J, 1987, PANCREAS, V2, P447, DOI 10.1097/00006676-198707000-00012
Seifert H, 2000, LANCET, V356, P653, DOI 10.1016/S0140-6736(00)02611-8
Shami Vanessa M, 2007, Gastrointest Endosc Clin N Am, V17, P581, DOI 10.1016/j.giec.2007.05.015
Sharaiha RZ, 2017, GASTROINTEST ENDOSC, V85, P904, DOI 10.1016/j.gie.2016.12.023
Shimamura Y, 2017, CLIN ENDOSC, V50, P112, DOI 10.5946/ce.2017.046
Shimura T, 2009, J GASTROEN HEPATOL, V24, P1358, DOI 10.1111/j.1440-1746.2009.05857.x
Siddiqui UD, 2018, GASTROENTEROLOGY, V154, P1911, DOI 10.1053/j.gastro.2017.12.046
STROHM WD, 1980, ENDOSCOPY, V12, P241, DOI 10.1055/s-2007-1021752
Suk KT, 2014, CLIN MOL HEPATOL, V20, P6, DOI 10.3350/cmh.2014.20.1.6
Talreja JP, 2008, GASTROINTEST ENDOSC, V68, P1199, DOI 10.1016/j.gie.2008.06.015
Teoh AYB, 2020, GUT, V69, P1085, DOI 10.1136/gutjnl-2019-319996
Thompson CC, 2014, GASTROINTEST ENDOSC, V80, P517, DOI 10.1016/j.gie.2014.06.011
Trikudanathan G, 2018, AM J GASTROENTEROL, V113, P1550, DOI 10.1038/s41395-018-0232-3
Tyberg A, 2016, ENDOSC INT OPEN, V4, pE276, DOI 10.1055/s-0042-101789
Tyberg A, 2016, GASTROINTEST ENDOSC, V83, P254, DOI 10.1016/j.gie.2015.08.003
Tyberg A, 2015, GASTROINTEST ENDOSC, V81, P1011, DOI 10.1016/j.gie.2014.10.018
Vanek P, 2023, GASTROINTEST ENDOSC, V97, P1070, DOI 10.1016/j.gie.2022.12.026
Wadhawan M, 2006, DIGEST DIS SCI, V51, P2264, DOI 10.1007/s10620-006-9310-2
Walter D, 2016, GUT, V65, P6, DOI 10.1136/gutjnl-2015-309925
Wiersema MJ, 1996, GASTROINTEST ENDOSC, V44, P614, DOI 10.1016/S0016-5107(96)70022-6
Wiersema MJ, 2001, GASTROINTEST ENDOSC, V53, P811, DOI 10.1067/mge.2001.113272

Williams EJ, 2012, ENDOSCOPY, V44, P674, DOI 10.1055/s-0032-1309345
Yang D, 2018, ENDOSC INT OPEN, V6, pE1267, DOI 10.1055/a-0732-502
Zhang W, 2021, GASTROINTEST ENDOSC, V93, P565, DOI 10.1016/j.gie.2020.06.065
NR 80
TC 15
Z9 23
U1 1
U2 11
PU OXFORD UNIV PRESS
PI OXFORD
PA GREAT CLARENDON ST, OXFORD OX2 6DP, ENGLAND
SN 2052-0034
J9 GASTROENTEROL REP
JI Gastroenterol. Rep.
PD DEC 30
PY 2023
VL 11
AR goad038
DI 10.1093/gastro/goad038
PG 9
WC Gastroenterology & Hepatology
WE Science Citation Index Expanded (SCI-EXPANDED)
SC Gastroenterology & Hepatology
GA L2UI9
UT WOS:001021857200001
PM 37398926
OA Green Submitted, gold
DA 2026-05-09
ER

PT J
AU Ryozawa, S
Fujita, N
Irisawa, A
Hirooka, Y
Mine, T
AF Ryozawa, Shomei
Fujita, Naotaka
Irisawa, Atsushi
Hirooka, Yoshiki
Mine, Tetsuya
TI Current status of interventional endoscopic ultrasound

SO DIGESTIVE ENDOSCOPY
LA English
DT Review
DE biliary drainage; endoscopic ultrasound; gastrojejunostomy;
interventional; pancreatic pseudocyst
ID APPOSING METAL STENT; GUIDED BILIARY DRAINAGE; PANCREATIC-FLUID
COLLECTIONS; GASTRIC FUNDAL VARICES; GALLBLADDER DRAINAGE; ACUTE
CHOLECYSTITIS; CYANOACRYLATE GLUE; FAILED ERCP; MULTICENTER; EXPERIENCE

AB Endoscopic ultrasound (EUS) is being used increasingly in the management of pancreatic fluid collection, biliary and pancreatic duct drainage in cases of failed endoscopic retrograde cholangiopancreatography, drainage of the gallbladder, and other conditions. The role of interventional EUS is rapidly expanding and new interventions are continuously emerging. The development of devices could be a major breakthrough in the field of interventional EUS. New devices would enable the expansion of its role even further and prompt its widespread use in clinical practice. This review focuses on the current status of interventional EUS, especially highlighting the topics that are presently drawing the interest of endoscopists.

C1 [Ryozawa, Shomei] Saitama Med Univ, Int Med Ctr, Dept Gastroenterol, 1397-1 Yamane, Hidaka, Saitama 3501298, Japan.
[Fujita, Naotaka] Miyagi Hlth Check Up Plaza, Sendai, Miyagi, Japan.
[Irisawa, Atsushi] Fukushima Med Univ, Dept Gastroenterol, Aizu Med Ctr, Aizu Wakamatsu, Fukushima, Japan.
[Hirooka, Yoshiki] Nagoya Univ Hosp, Dept Endoscopy, Nagoya, Aichi, Japan.
[Mine, Tetsuya] Tokai Univ, Dept Gastroenterol, Sch Med, Isehara, Kanagawa, Japan.
C3 Saitama Medical University; Nagoya University; Tokai University
RP Ryozawa, S (corresponding author), Saitama Med Univ, Int Med Ctr, Dept Gastroenterol, 1397-1 Yamane, Hidaka, Saitama 3501298, Japan.
EM ryozawa@saitama-med.ac.jp
RI Hirooka, Yoshiki/I-7351-2014

CR Antillon MR, 2006, GASTROINTEST ENDOSC, V63, P797, DOI 10.1016/j.gie.2005.10.025
Banks PA, 2013, GUT, V62, P102, DOI 10.1136/gutjnl-2012-302779
Baron TH, 2007, GASTROINTEST ENDOSC, V65, P735, DOI 10.1016/j.gie.2006.07.041
Bhat YM, 2016, GASTROINTEST ENDOSC, V83, P1164, DOI 10.1016/j.gie.2015.09.040
Binmoeller KF, 2011, GASTROINTEST ENDOSC, V74, P1019, DOI 10.1016/j.gie.2011.06.030
Bollen FL, 2008, BRIT J SURG, V95, P6, DOI 10.1002/bjs.6010
Chan S M., 2016, Gastrointest Endosc
Ergun M, 2011, ENDOSCOPY, V43, P518, DOI 10.1055/s-0030-1256333
Fritscher-Ravens A, 2002, GASTROINTEST ENDOSC, V56, P737, DOI 10.1067/mge.2002.129084
Fujii LL, 2013, GASTROINTEST ENDOSC, V78, P854, DOI 10.1016/j.gie.2013.05.016
Fujii-Lau LL, 2016, SURG ENDOSC, V30, P1396, DOI 10.1007/s00464-015-4342-3
Gardner TB, 2011, GASTROINTEST ENDOSC, V73, P718, DOI 10.1016/j.gie.2010.10.053
Ge N, 2016, BMC GASTROENTEROL, V16, DOI 10.1186/s12876-016-0420-9
Hara K, 2016, WORLD J GASTROENTERO, V22, P1297, DOI 10.3748/wjg.v22.i3.1297
Holt BA, 2016, GASTROINTEST ENDOSC, V83, P160, DOI 10.1016/j.gie.2015.06.019
Isayama H, 2016, J GASTROEN HEPATOL, V31, P1546, DOI 10.1111/jgh.13394
Itoi T, 2016, GUT, V65, P193, DOI 10.1136/gutjnl-2015-310348

Itoi T, 2015, ENDOSCOPY, V47, P462, DOI 10.1055/s-0034-1391083
Itoi T, 2013, GASTROINTEST ENDOSC, V78, P934, DOI 10.1016/j.gie.2013.09.025
Itoi T, 2012, GASTROINTEST ENDOSC, V75, P870, DOI 10.1016/j.gie.2011.10.020
Iwashita T, 2014, CLIN J GASTROENTEROL, V7, P94, DOI 10.1007/s12328-014-0467-5
Jang JW, 2012, GASTROENTEROLOGY, V142, P805, DOI 10.1053/j.gastro.2011.12.051
Kamata K, 2010, ENDOSCOPY, V42, pE331, DOI 10.1055/s-0030-1255941
Kawakubo K, 2014, J HEPATO-BIL-PAN SCI, V21, P328, DOI 10.1002/jhbp.27
Lakhtakia S, 2016, ENDOSCOPY, V48, pE180, DOI 10.1055/s-0042-106966
Law R, 2016, CLIN GASTROENTEROL H, V14, P476, DOI 10.1016/j.cgh.2015.10.026
Martins FP, 2010, ENDOSCOPY, V42, pE126, DOI 10.1055/s-0029-1243911
Park DH, 2013, GASTROINTEST ENDOSC, V78, P91, DOI 10.1016/j.gie.2013.01.042
Poincloux L, 2015, ENDOSCOPY, V47, P794, DOI [10.1055/s-0035-1545399, 10.1055/s-0034-1391988]
Prachayakul V, 2011, ENDOSCOPY, V43, pE213, DOI 10.1055/s-0030-1256395
Romero-Castro R, 2010, ENDOSCOPY, V42, pE35, DOI 10.1055/s-0029-1215261
Romero-Castro R, 2013, GASTROINTEST ENDOSC, V78, P711, DOI 10.1016/j.gie.2013.05.009
Seewald S, 2009, GASTROINTEST ENDOSC, V69, pS13, DOI 10.1016/j.gie.2008.10.061
Seifert H, 2009, GUT, V58, P1260, DOI 10.1136/gut.2008.163733
Shah JN, 2012, GASTROINTEST ENDOSC, V75, P56, DOI 10.1016/j.gie.2011.08.032
Siripun A, 2015, WORLD J GASTRO ENDOS, V7, P283, DOI 10.4253/wjge.v7.i3.283
Takagi W, 2016, THER ADV GASTROENTER, V9, P19, DOI 10.1177/1756283X15609285
Teoh AYB, 2014, GASTROINTEST ENDOSC, V80, P1171, DOI 10.1016/j.gie.2014.03.038
Turner BG, 2013, ENDOSCOPY, V45, pE114, DOI 10.1055/s-0032-1325968
van Santvoort HC, 2010, NEW ENGL J MED, V362, P1491, DOI 10.1056/NEJMoa0908821
Varadarajulu S, 2011, J GASTROEN HEPATOL, V26, P1504, DOI 10.1111/j.1440-1746.2011.06771.x
Vila JJ, 2012, GASTROINTEST ENDOSC, V76, P1133, DOI 10.1016/j.gie.2012.08.001
Walter D, 2016, GUT, V65, P6, DOI 10.1136/gutjnl-2015-309925
Walter D, 2015, ENDOSCOPY, V47, P63, DOI 10.1055/s-0034-1378113
Will U, 2011, ULTRASCHALL MED, V32, P176, DOI 10.1055/s-0031-1273347
Yasuda I, 2013, ENDOSCOPY, V45, P627, DOI 10.1055/s-0033-1344027
NR 46
TC 30
Z9 31
U1 0
U2 5
PU WILEY
PI HOBOKEN
PA 111 RIVER ST, HOBOKEN 07030-5774, NJ USA
SN 0915-5635
EI 1443-1661
J9 DIGEST ENDOSC
JI Dig. Endosc.
PD JUL

PY 2017
VL 29
IS 5
BP 559
EP 566
DI 10.1111/den.12872
PG 8
WC Gastroenterology & Hepatology; Surgery
WE Science Citation Index Expanded (SCI-EXPANDED)
SC Gastroenterology & Hepatology; Surgery
GA EZ4YR
UT WOS:000404718900003
PM 28317208
OA Bronze
DA 2026-05-09
ER

PT J
AU McCarty, TR
Thompson, CC
AF McCarty, Thomas R.
Thompson, Christopher C.
TI Lumen Apposition: A Changing Landscape in Therapeutic Endoscopy
SO DIGESTIVE DISEASES AND SCIENCES
LA English
DT Review
DE Lumen apposing metal stents (LAMS); Advanced endoscopy; Endoscopic
surgery; Endoscopic ultrasound
ID GUIDED GALLBLADDER DRAINAGE; Y GASTRIC BYPASS; PANCREATIC FLUID
COLLECTIONS; DIRECTED TRANSGASTRIC ERCP; METAL STENTS; PLASTIC STENTS;
MANAGEMENT; GASTROJEJUNOSTOMY; MULTICENTER; EFFICACY
AB The concept of endoscopic lumen apposition has seen a dramatic shift in the last several decades. Early natural orifice trans-luminal endoscopic surgery (NOTES) concepts have transformed into specialized lumen-apposing metal stents (LAMSs) and delivery devices, which provide endoscopists a minimally invasive alternative to surgical intervention. These LAMSs have become the bedrock of therapeutic endoscopy and provide treatment for a wide spectrum of gastrointestinal disorders. In this review, we summarize the changing landscape of therapeutic endoscopy by highlighting the use of LAMS and future directions as well as alternative devices to achieve lumen apposition.
C1 [McCarty, Thomas R.; Thompson, Christopher C.] Harvard Med Sch, Brigham & Womens Hosp, Div Gastroenterol Hepatol & Endoscopy, 75 Francis St, Boston, MA 02115 USA.
C3 Harvard University; Harvard University Medical Affiliates; Brigham &
Women's Hospital; Harvard Medical School
RP Thompson, CC (corresponding author), Harvard Med Sch, Brigham & Womens Hosp, Div Gastroenterol Hepatol &

Endoscopy, 75 Francis St, Boston, MA 02115 USA.
EM cthompson@hms.harvard.edu
CR Adler DG, 2019, AM J GASTROENTEROL, V114, P700, DOI 10.14309/ajg.0000000000000067
Adler DG, 2017, AM J GASTROENTEROL, V112, P516, DOI 10.1038/ajg.2016.566
Ahmad W, 2020, SCAND J GASTROENTERO, V55, P242, DOI 10.1080/00365521.2019.1710246
Bakkaloglu H, 2006, WORLD J GASTROENTERO, V12, P7179, DOI 10.3748/wjg.v12.i44.7179
Bakker OJ, 2012, JAMA-J AM MED ASSOC, V307, P1053, DOI 10.1001/jama.2012.276
Bang JY, 2019, GASTROENTEROLOGY, V156, P1027, DOI 10.1053/j.gastro.2018.11.031
Bang JY, 2019, GUT, V68, P1200, DOI 10.1136/gutjnl-2017-315335
Bergström M, 2006, GASTROINTEST ENDOSC, V63, P307, DOI 10.1016/j.gie.2005.09.035
Binmoeller KF, 2011, ENDOSCOPY, V43, P337, DOI 10.1055/s-0030-1256127
Bukhari M, 2018, GASTROINTEST ENDOSC, V88, P486, DOI 10.1016/j.gie.2018.04.2356
Chan JHY, 2018, CLIN ENDOSC, V51, P150, DOI 10.5946/ce.2017.125
Chandrasekhara V, 2020, ENDOSC INT OPEN, V08, pE1639, DOI 10.1055/a-1243-0092
Chaudhary S, 2016, ENDOSC ULTRASOUND, V5, P281, DOI 10.4103/2303-9027.191605
Chen YI, 2018, ENDOSC INT OPEN, V6, pE780, DOI 10.1055/a-0611-5082
Chowdhury SD, 2016, ENDOSCOPY, V48, pE263, DOI 10.1055/s-0042-112971
Sanz MD, 2021, SURG ENDOSC, V35, P6754, DOI 10.1007/s00464-020-08179-y
Dubroff J, 2019, PRACT GASTROENTEROL, V43, P34
Farias GFA, 2019, MEDICINE, V98, DOI 10.1097/MD.0000000000014255
Fritscher-Ravens A, 2003, GASTROINTEST ENDOSC, V58, P585
Garg PK, 2020, SURG ENDOSC, V34, P1157, DOI 10.1007/s00464-019-06866-z
Ge Phillip S, 2019, VideoGIE, V4, P172, DOI [10.1016/j.vgie.2018.08.001, 10.1016/j.vgie.2018.08.001]
Ge PS, 2019, SURG ENDOSC, V33, P3404, DOI 10.1007/s00464-018-06636-3
Ge PS, 2016, GASTROENTEROL CLIN N, V45, P9, DOI 10.1016/j.gtc.2015.10.003
Gornals JB, 2013, SURG ENDOSC, V27, P1428, DOI 10.1007/s00464-012-2591-y
Graves CE, 2017, J AM COLL SURGEONS, V225, P676, DOI 10.1016/j.jamcollsurg.2017.07.1062
Guo JT, 2018, ENDOSC ULTRASOUND, V7, P356, DOI 10.4103/eus.eus_53_18
Hakim S., 2020, J CLIN GASTROENTEROL
Hakim Seifeldin, 2020, VideoGIE, V5, P364, DOI [10.1016/j.vgie.2020.04.017, 10.1016/j.vgie.2020.04.017]
Hammad T, 2018, DIGEST DIS SCI, V63, P289, DOI 10.1007/s10620-017-4851-0
Han D, 2018, J CLIN GASTROENTEROL, V52, P835, DOI 10.1097/MCG.0000000000000934
Irani S, 2017, GASTROINTEST ENDOSC, V85, P1285, DOI 10.1016/j.gie.2016.08.028
Irani S, 2015, GASTROINTEST ENDOSC, V82, P1110, DOI 10.1016/j.gie.2015.05.045
Itoi T, 2017, DIGEST ENDOSC, V29, P495, DOI 10.1111/den.12794
Itoi T, 2014, GASTROINTEST ENDOSC, V79, P715, DOI 10.1016/j.gie.2013.11.021
Itoi T, 2012, GASTROINTEST ENDOSC, V75, P870, DOI 10.1016/j.gie.2011.10.020
Kalloo AN, 2004, GASTROINTEST ENDOSC, V60, P114, DOI 10.1016/S0016-5107(04)01309-4
Kantsevoy SV, 2005, GASTROINTEST ENDOSC, V62, P287, DOI 10.1016/S0016-5107(05)01565-8
Kayal A, 2021, SURG ENDOSC, V35, P2698, DOI 10.1007/s00464-020-07699-x
Kedia P, 2019, J CLIN GASTROENTEROL, V53, P304, DOI 10.1097/MCG.0000000000001037
Kedia P, 2015, GASTROINTEST ENDOSC, V81, P223, DOI 10.1016/j.gie.2014.04.007

Kedia P, 2014, GASTROENTEROLOGY, V147, P566, DOI 10.1053/j.gastro.2014.05.045
Khashab MA, 2017, ENDOSC INT OPEN, V5, pE275, DOI 10.1055/s-0043-101695
Krafft MR, 2019, ENDOSC INT OPEN, V7, pE1231, DOI 10.1055/a-0915-2192
Kunda R, 2016, SURG ENDOSC, V30, P5002, DOI 10.1007/s00464-016-4845-6
Machytka E, 2017, GASTROINTEST ENDOSC, V86, P904, DOI 10.1016/j.gie.2017.07.009
Marrache MK, 2020, GASTROINTEST ENDOSC
Alcalá FM, 2015, ENDOSCOPY, V47, pE413, DOI 10.1055/s-0034-1392676
McCarty TR, 2020, GASTROINTEST ENDOSC, V91, pAB92, DOI 10.1016/j.gie.2020.03.855
McCarty TR, 2020, GASTROINTEST ENDOSC, V91, pAB301, DOI 10.1016/j.gie.2020.03.2112
McCarty Thomas R, 2019, Endosc Int Open, V7, pE1474, DOI [10.1055/a-0996-8178, 10.1055/a-0996-8178]
McCarty TR, 2018, GASTROINTEST ENDOSC, V87, pAB231, DOI 10.1016/j.gie.2018.04.1514
McCarty TR, 2020, IN PRESS
Melki Gabriel, 2020, Middle East J Dig Dis, V12, P206, DOI [10.34172/mejdd.2020.183, 10.34172/mejdd.2020.183]
Mudireddy PR, 2018, GASTROINTEST ENDOSC, V87, P1256, DOI 10.1016/j.gie.2017.08.011
Muneer Asif, 2008, Expert Rev Gastroenterol Hepatol, V2, P155, DOI 10.1586/17474124.2.2.155
Mussetto A, 2018, ANN GASTROENTEROL, V31, P535, DOI 10.20524/aog.2018.0287
Ngamruengphong S, 2017, ENDOSCOPY, V49, P549, DOI 10.1055/s-0043-105072
Perez-Miranda M, 2017, J CLIN GASTROENTEROL, V51, P896, DOI 10.1097/MCG.0000000000000887
Pinson R, 2017, ACG CASE REP J, V4, DOI 10.14309/crj.2017.20
Podboy A, 2021, GASTROINTEST ENDOSC, V93, P797, DOI 10.1016/j.gie.2020.09.040
Poincloux L, 2017, ENDOSCOPY, V49, P484, DOI 10.1055/s-0042-122011
Powers PC, 2019, ENDOSC ULTRASOUND, V8, P194, DOI 10.4103/eus.eus_59_18
Priyanka P, 2019, CASE REP GASTROINTES, V2019, DOI 10.1155/2019/7656950
Rodrigues-Pinto E, 2016, GASTROENTEROLOGY, V151, P30, DOI 10.1053/j.gastro.2016.04.039
Ryou M, 2016, SURG ENDOSC, V30, P4533, DOI 10.1007/s00464-016-4789-x
Ryou M, 2016, GASTROINTEST ENDOSC, V83, P821, DOI 10.1016/j.gie.2015.10.023
Samuelson AL, 2012, GASTROENTEROL CLIN N, V41, P47, DOI 10.1016/j.gtc.2011.12.007
Sánchez-Luna SA, 2020, DIGEST ENDOSC, V32, P1117, DOI 10.1111/den.13822
Santos-Fernandez J, 2017, WORLD J GASTRO ENDOS, V9, P571, DOI 10.4253/wjge.v9.i12.571
Saumoy Monica, 2018, Gastrointest Endosc Clin N Am, V28, P197, DOI [10.1016/j.giec.2017.11.007, 10.1016/j.giec.2017.11.007]
Saumoy M, 2017, GASTROINTEST ENDOSC, V85, P1253, DOI 10.1016/j.gie.2017.03.008
Saunders R, 2019, SURG ENDOSC, V33, P1412, DOI 10.1007/s00464-018-6416-5
Schulman AR, 2018, GASTROINTEST ENDOSC, V87, P890, DOI 10.1016/j.gie.2017.09.032
Shah RJ, 2013, GASTROINTEST ENDOSC, V77, P593, DOI 10.1016/j.gie.2012.10.015
Shaikh SN, 2010, WORLD J GASTRO SURG, V2, P210, DOI 10.4240/wjgs.v2.i6.210
Sharma P, 2020, WORLD J GASTROENTERO, V26, P2715, DOI 10.3748/wjg.v26.i21.2715
Sharma SS, 2016, INDIAN J GASTROENTER, V35, P40, DOI 10.1007/s12664-016-0624-5
Siddiqui AA, 2016, GASTROINTEST ENDOSC, V83, P699, DOI 10.1016/j.gie.2015.10.020
Skinner M, 2014, ENDOSCOPY, V46, P560, DOI 10.1055/s-0034-1365698
Teoh AYB, 2020, GUT, V69, P1085, DOI 10.1136/gutjnl-2019-319996
Teoh AY, 2016, GASTROINTEST ENDOSC, V84, P62, DOI 10.1016/j.gie.2015.11.041

Teoh AYB, 2021, GASTROINTEST ENDOSC, V93, P577, DOI 10.1016/j.gie.2020.06.066
Teoh AYB, 2020, DIGEST ENDOSC, V32, P391, DOI 10.1111/den.13494
Trindade AJ, 2018, ENDOSCOPY, V50, pE210, DOI 10.1055/a-0624-1140
Tsuchiya T, 2018, GASTROINTEST ENDOSC, V87, P1138, DOI 10.1016/j.gie.2017.08.017
Turkeltaub Joshua A, 2019, Curr Treat Options Gastroenterol, V17, P187, DOI [10.1007/s11938-019-00232-7, 10.1007/s11938-019-00232-7]
Uchima H, 2016, GASTROINTEST ENDOSC, V83, P251, DOI 10.1016/j.gie.2015.07.008
US Food and Drug Administration (FDA), 2013, APPR AXIOS STENT DEL
Varadarajulu S, 2013, GASTROENTEROLOGY, V145, P583, DOI 10.1053/j.gastro.2013.05.046
Varadarajulu S, 2011, J GASTROEN HEPATOL, V26, P1504, DOI 10.1111/j.1440-1746.2011.06771.x
Walter D, 2016, GUT, V65, P6, DOI 10.1136/gutjnl-2015-309925
Wang TJ, 2019, SURG ENDOSC, V33, P2024, DOI 10.1007/s00464-019-06715-z
Yang D, 2017, ENDOSCOPY, V49, P327, DOI 10.1055/s-0042-122779
Yang JL, 2019, ENDOSCOPY, V51, P1035, DOI 10.1055/a-0759-1353
Yang J, 2018, GASTROINTEST ENDOSC, V87, pAB105, DOI 10.1016/j.gie.2018.04.129
NR 95
TC 4
Z9 4
U1 0
U2 4
PU SPRINGER
PI DORDRECHT
PA VAN GODEWIJCKSTRAAT 30, 3311 GZ DORDRECHT, NETHERLANDS
SN 0163-2116
EI 1573-2568
J9 DIGEST DIS SCI
JI Dig. Dis. Sci.
PD MAY
PY 2022
VL 67
IS 5
SI SI
BP 1660
EP 1673
DI 10.1007/s10620-022-07426-7
EA APR 2022
PG 14
WC Gastroenterology & Hepatology
WE Science Citation Index Expanded (SCI-EXPANDED)
SC Gastroenterology & Hepatology
GA 1N7UO
UT WOS:000782889900001

PM 35430698
DA 2026-05-09
ER

PT J
AU Saumoy, M
Kahaleh, M
AF Saumoy, Monica
Kahaleh, Michel
TI Safety and Complications of Interventional Endoscopic Ultrasound
SO CLINICAL ENDOSCOPY
LA English
DT Review
DE Endosonography; Therapeutics; Pseudocyst; EUS-BD; EUS-GLB
ID GUIDED BILIARY DRAINAGE; PANCREATIC FLUID COLLECTIONS; GALLBLADDER DRAINAGE; RANDOMIZED-TRIAL; EUS; EFFICACY; ULTRASONOGRAPHY; ERCP; PSEUDOCYST; EXPERIENCE
AB Endoscopic ultrasound (EUS) has become an essential tool for the diagnostic and therapeutic intervention of gastrointestinal diseases. Beyond the drainage of fluid collections, it enables decompression of inaccessible bile and pancreatic ducts, the gallbladder, and the creation of anastomosis within the gastrointestinal tract using fully lumen-apposing stents. This review explored the safety and efficacy of these novel procedures and discussed the training pathway that is necessary to perform them efficiently and safely.
C1 [Saumoy, Monica; Kahaleh, Michel] Weill Cornell Med Coll, Dept Gastroenterol, 1305 York Ave,4th Floor, New York, NY 10021 USA.
C3 Cornell University; Weill Cornell Medicine
RP Kahaleh, M (corresponding author), Weill Cornell Med Coll, Dept Gastroenterol, 1305 York Ave,4th Floor, New York, NY 10021 USA.
EM mkahaleh@gmail.com
RI ; Kahaleh, Michel/A-9408-2011
OI Saumoy, Monica/0000-0003-2780-1206;
CR Alvarez-Sanchez MV, 2014, SURG ENDOSC, V28, P712, DOI 10.1007/s00464-013-3260-5
Anderloni A, 2016, SURG ENDOSC, V30, P5200, DOI 10.1007/s00464-016-4894-x
Dollhopf M, 2017, GASTROINTEST ENDOSC, V86, P636, DOI 10.1016/j.gie.2017.02.027
Feurer ME, 2016, BEST PRACT RES CL GA, V30, P397, DOI 10.1016/j.bpg.2016.04.005
Fujii LL, 2013, GASTROINTEST ENDOSC, V78, P854, DOI 10.1016/j.gie.2013.05.016
Hara K, 2016, WORLD J GASTROENTERO, V22, P1297, DOI 10.3748/wjg.v22.i3.1297
Isayama H, 2016, J GASTROEN HEPATOL, V31, P1546, DOI 10.1111/jgh.13394
Jorgensen J, 2016, GASTROINTEST ENDOSC, V83, P279, DOI 10.1016/j.gie.2015.11.006
Kahaleh M, 2006, ENDOSCOPY, V38, P355, DOI 10.1055/s-2006-925249
Kahaleh M, 2007, GASTROINTEST ENDOSC, V65, P224, DOI 10.1016/j.gie.2006.05.008
Kahaleh M, 2013, WORLD J GASTROENTERO, V19, P1372, DOI 10.3748/wjg.v19.i9.1372
Kawakubo K, 2014, J HEPATO-BIL-PAN SCI, V21, P328, DOI 10.1002/jhbp.27

Kedia P, 2015, GASTROINTEST ENDOSC, V82, P1031, DOI 10.1016/j.gie.2015.03.1912
Khan MA, 2017, GASTROINTEST ENDOSC, V85, P76, DOI 10.1016/j.gie.2016.06.032
Khan MA, 2016, DIGEST DIS SCI, V61, P684, DOI 10.1007/s10620-015-3933-0
Lakhtakia S, 2016, BEST PRACT RES CL GA, V30, P807, DOI 10.1016/j.bpg.2016.10.008
Park DH, 2009, ENDOSCOPY, V41, P842, DOI 10.1055/s-0029-1215133
Perez-Miranda Manuel, 2012, Gastrointest Endosc Clin N Am, V22, P491, DOI 10.1016/j.giec.2012.05.004
Rosenthal LS, 2008, GASTROINTEST ENDOSC, V68, P1150, DOI 10.1016/j.gie.2008.09.020
Sadik R, 2011, WORLD J GASTROENTERO, V17, P499, DOI 10.3748/wjg.v17.i4.499
Shah RJ, 2015, CLIN GASTROENTEROL H, V13, P747, DOI 10.1016/j.cgh.2014.09.047
Siddiqui AA, 2016, GASTROINTEST ENDOSC, V83, P699, DOI 10.1016/j.gie.2015.10.020
Teoh AYB, 2017, ENDOSCOPY, V49, P130, DOI 10.1055/s-0042-119036
Tyberg A, 2018, J CLIN GASTROENTEROL, V52, P79, DOI 10.1097/MCG.0000000000000786
Tyberg A, 2017, GASTROINTEST ENDOSC, V85, P164, DOI 10.1016/j.gie.2016.07.030
Varadarajulu S, 2013, GASTROENTEROLOGY, V145, P583, DOI 10.1053/j.gastro.2013.05.046
Varadarajulu S, 2011, J GASTROINTEST SURG, V15, P2080, DOI 10.1007/s11605-011-1621-8
Vilmann AS, 2015, WORLD J GASTROENTERO, V21, P11842, DOI 10.3748/wjg.v21.i41.11842
NR 28
TC 14
Z9 15
U1 0
U2 3
PU KOREAN SOC GASTROINTESTINAL ENDOSCOPY
PI SEOUL
PA 2003 LG PALACE, 165-8 DONGGYO-DONG, MAPO-GU, SEOUL, 121-754, SOUTH KOREA
SN 2234-2400
EI 2234-2443
J9 CLIN ENDOSC
JI Clin. Endosc.
PD MAY
PY 2018
VL 51
IS 3
BP 235
EP 238
DI 10.5946/ce.2017.081
PG 4
WC Gastroenterology & Hepatology
WE Emerging Sources Citation Index (ESCI)
SC Gastroenterology & Hepatology
GA GI0TJ
UT WOS:000434082100007
PM 28719966

OA Green Submitted, gold
DA 2026-05-09
ER

PT J
AU Sundaram, S
Giri, S
Binmoeller, K
AF Sundaram, Sridhar
Giri, Suprabhat
Binmoeller, Kenneth
TI Lumen-apposing metal stents: A primer on indications and technical tips
SO INDIAN JOURNAL OF GASTROENTEROLOGY
LA English
DT Review
DE Lumen-apposing metal stents; LAMS; Troubleshooting
ID EUS-GUIDED GASTROENTEROSTOMY; GASTRIC OUTLET OBSTRUCTION; GALLBLADDER DRAINAGE; MANAGEMENT; PLACEMENT; GASTROJEJUNOSTOMY; EFFICACY; SAFETY
AB Lumen-apposing metal stents have ushered a new frontier for interventional endoscopic ultrasound. Initial use for the drainage of pancreatic fluid collections has rapidly expanded to the bile duct, gallbladder and small bowel. Intra-luminal applications for short strictures have also emerged. Electrocautery enhancement has made the stent delivery speedy. While the advent of lumen-apposing metal stents (LAMS) has brought a paradigm shift in the practice of interventional endoscopic ultrasound, their use is associated with certain technicalities that any advanced endosonologist should know. Understanding indications for LAMS is critical and their use in appropriate settings. Troubleshooting in tricky situations is always a challenge and the background considerations for their use include knowing the target organ, cautery and puncture technique, managing partially opened LAMS and also managing maldeployment. In this review, we discuss the intricacies and technical tips for the use of lumen-apposing metal stents.
C1 [Sundaram, Sridhar] Tata Mem Hosp, Dept Digest Dis & Clin Nutr, Dr E Borges Rd, Mumbai 400012, India.
[Giri, Suprabhat] Kalinga Inst Med Sci, Dept Gastroenterol, Bhubaneswar 751024, India.
[Binmoeller, Kenneth] Calif Pacific Med Ctr, Intervent Endoscopy Serv, San Francisco, CA USA.
C3 Tata Memorial Centre (TMC); Tata Memorial Hospital; Kalinga Institute of Industrial Technology (KIIT); California Pacific Medical Center
RP Sundaram, S (corresponding author), Tata Mem Hosp, Dept Digest Dis & Clin Nutr, Dr E Borges Rd, Mumbai 400012, India.
EM drsridharsundaram@gmail.com
RI GIRI, SUPRABHAT/AFO-1762-2022; S, Sridhar/R-6416-2017
CR Abbas A, 2022, GASTROINTEST ENDOSC, V95, P682, DOI 10.1016/j.gie.2021.10.030
Anderloni A, 2017, ENDOSC INT OPEN, V5, pE25, DOI 10.1055/s-0042-122009
Armellini E, 2023, WORLD J GASTROENTERO, V29, P3341, DOI 10.3748/wjg.v29.i21.3341
Bang JY, 2023, GASTROENTEROLOGY, V164, pS1572
Bang JY, 2022, DIGEST ENDOSC, V34, P612, DOI 10.1111/den.14099
Bang JY, 2019, GUT, V68, P1200, DOI 10.1136/gutjnl-2017-315335

Bhenswala P, 2021, J CLIN GASTROENTEROL, V55, P641, DOI 10.1097/MCG.0000000000001566
Binda C, 2023, GASTROINTEST ENDOSC, V98, P765, DOI 10.1016/j.gie.2023.06.054
Binmoeller KF, 2012, ENDOSCOPY, V44, P499, DOI 10.1055/s-0032-1309382
Binmoeller KF, 2011, ENDOSCOPY, V43, P337, DOI 10.1055/s-0030-1256127
Binmoeller KF, 2020, TECH INNOVAT GASTROI, V22, P3, DOI 10.1016/j.tgie.2019.150653
Boxhoorn L, 2023, GUT, V72, P66, DOI 10.1136/gutjnl-2021-325632
Chandan S, 2021, ENDOSC INT OPEN, V09, pE496, DOI 10.1055/a-1341-0788
Chen YI, 2023, GASTROENTEROLOGY, V165, P1249, DOI 10.1053/j.gastro.2023.07.024
Chen Yen-I, 2023, VideoGIE, V8, P500, DOI [10.1016/j.vgie.2023.07.014, 10.1016/j.vgie.2023.07.014]
Cope C, 1999, J VASC INTERV RADIOL, V10, P1379, DOI 10.1016/S1051-0443(99)70248-4
Sanz MD, 2021, SURG ENDOSC, V35, P6754, DOI 10.1007/s00464-020-08179-y
Dhaliwal AJ, 2019, GASTROINTEST ENDOSC, V89, pAB210, DOI 10.1016/j.gie.2019.03.185
Fabbri C, 2022, SURG ENDOSC, V36, P7974, DOI 10.1007/s00464-022-09339-y
Food and Drug Administration Department of Health and Human Services, 2017, I7696EN10817 FAO, P1
Garcia-Sumalla A, 2023, GASTROINTEST ENDOSC, V97, pAB865
Ghandour B, 2022, GASTROINTEST ENDOSC, V95, P80, DOI 10.1016/j.gie.2021.07.023
Giovannini M, 2001, ENDOSCOPY, V33, P898, DOI 10.1055/s-2001-17324
Giri S, 2023, THER ADV GASTROINTES, V16, DOI 10.1177/26317745231199364
Giri S, 2023, ANN GASTROENTEROL, DOI 10.20524/aog.2023.0819
Giri S, 2023, GASTROINTEST ENDOSC, V98, DOI 10.1016/j.gie.2023.06.055
Gkolfakis P, 2022, LIFE-BASEL, V12, DOI 10.3390/life12101646
Grover D, 2023, WORLD J GASTRO ENDOS, V15, DOI 10.4253/wjge.v15.i9.574
Hemerly MC, 2023, SURG ENDOSC, V37, P2421, DOI 10.1007/s00464-022-09712-x
Itoi T, 2013, J HEPATO-BIL-PAN SCI, V20, P403, DOI 10.1007/s00534-012-0551-5
Jena A, 2023, EXPERT REV GASTROENT, V17, P825, DOI 10.1080/17474124.2023.2242243
Krishnamoorthi R, 2020, SURG ENDOSC, V34, P1904, DOI 10.1007/s00464-020-07409-7
Kumar A, 2022, ENDOSC INT OPEN, V10, pE448, DOI 10.1055/a-1765-4035
Lyu YX, 2021, PANCREAS, V50, P571, DOI 10.1097/MPA.0000000000001798
Mangiavillano B, 2024, ENDOSCOPY, V56, P249, DOI 10.1055/a-2219-3179
Mangiavillano B, 2022, GASTROINTEST ENDOSC, V95, P115, DOI 10.1016/j.gie.2021.07.021
Monino L, 2023, ENDOSCOPY, V55, P991, DOI 10.1055/a-2119-7529
Ngamruengphong S, 2017, ENDOSCOPY, V49, P549, DOI 10.1055/s-0043-105072
Ogura T, 2021, DEN OPEN, V1, DOI 10.1002/deo2.8
Pawa R, 2022, DIGEST ENDOSC, V34, P1234, DOI 10.1111/den.14266
Rana SS, 2021, ANN GASTROENTEROL, V34, P142, DOI 10.20524/aog.2020.0568
Rimbas M, 2021, ENDOSC INT OPEN, V09, pE1801, DOI 10.1055/a-1526-1208
Shah JM, 2023, ENDOSCOPY, V55, pE416, DOI 10.1055/a-2008-0431
Siddiqui A, 2021, ANN GASTROENTEROL, V34, P273, DOI 10.20524/aog.2021.0570
Song TJ, 2020, TECH INNOVAT GASTROI, V22, P9, DOI 10.1016/j.tgie.2019.150637
Stefanovic S, 2021, WORLD J GASTRO SURG, V13, P620, DOI 10.4240/wjgs.v13.i7.620
Sundaram S, 2023, GASTROINTEST ENDOSC, V97, pAB881
Teoh A, 2023, GASTROINTEST ENDOSC, V97, pAB882

Teoh AY, 2016, GASTROINTEST ENDOSC, V84, P62, DOI 10.1016/j.gie.2015.11.041
Teoh AYB, 2023, GASTROENTEROLOGY, V165, P473, DOI 10.1053/j.gastro.2023.04.016
Vanek P, 2023, GASTROINTEST ENDOSC, V97, P1070, DOI 10.1016/j.gie.2022.12.026
Vanella G, 2023, DIGEST ENDOSC, V35, P377, DOI 10.1111/den.14445
Vedantam S, 2023, CLIN ENDOSC, V56, P298, DOI 10.5946/ce.2022.299
Westerveld Donevan, 2022, VideoGIE, V7, P109, DOI 10.1016/j.vgie.2021.12.007
Yoo HW, 2021, ENDOSCOPY, V53, P922, DOI 10.1055/a-1301-1526
Zhai YQ, 2022, GASTROINTEST ENDOSC, V96, P522, DOI 10.1016/j.gie.2022.04.014
NR 56
TC 9
Z9 10
U1 0
U2 0
PU SPRINGER INDIA
PI NEW DELHI
PA 7TH FLOOR, VIJAYA BUILDING, 17, BARAKHAMBA ROAD, NEW DELHI, 110 001, INDIA
SN 0254-8860
EI 0975-0711
J9 INDIAN J GASTROENTER
JI Indian J. Gastroenterol.
PD OCT
PY 2024
VL 43
IS 5
BP 886
EP 895
DI 10.1007/s12664-024-01562-w
EA MAY 2024
PG 10
WC Gastroenterology & Hepatology
WE Emerging Sources Citation Index (ESCI)
SC Gastroenterology & Hepatology
GA J6W5A
UT WOS:001216088500002
PM 38717660
DA 2026-05-09
ER

PT J
AU Chen, B
Johal, R

Abdulsamad, M
AF Chen, Bing
Johal, Riya
Abdulsamad, Molham
TI Therapeutic Endoscopy to the Rescue: EUS Gallbladder Drainage and ESD of
a Giant Duodenal Polyp
SO ACG CASE REPORTS JOURNAL
LA English
DT Article
DE endoscopic ultrasound-guided gallbladder drainage; endoscopic submucosal
dissection; duodenal polyp
AB The role of endoscopy in the management of traditionally surgical conditions continues to evolve. Endoscopic techniques, including endoscopic mucosal resection and more recently endoscopic submucosal dissection, which remove cancerous and precancerous lesions in the gastrointestinal tract, continue to grow in use. In addition, therapeutic endoscopic ultrasound has evolved to include the management of patients with acute cholecystitis. Here, we present an 83-year-old man with acute calculus cholecystitis, who was considered ineligible for cholecystectomy and had a giant duodenal polyp that obstructed the visualization of the duodenal lumen, preventing the successful placement of lumen-apposing metal stents. Volume reduction of a duodenal polyp was performed through epinephrine injection to assist in the successful endoscopic ultrasound-guided gallbladder drainage. Subsequently, the patient underwent elective endoscopic mucosal resection to remove the giant duodenal polyp.
C1 [Chen, Bing; Johal, Riya; Abdulsamad, Molham] Geisinger Med Ctr, Dept Gastroenterol & Hepatol, Danville, PA 17822 USA.
C3 Geisinger Medical Center
RP Chen, B (corresponding author), Geisinger Med Ctr, Dept Gastroenterol & Hepatol, Danville, PA 17822 USA.
EM chenbingalex@gmail.com; riyajohal@gmail.com; mabdulsamad@geisinger.edu
OI Chen, Bing/0000-0002-6058-9529
CR Baron TH, 2007, GASTROINTEST ENDOSC, V65, P735, DOI 10.1016/j.gie.2006.07.041
Boregowda U, 2023, DIAGNOSTICS, V13, DOI 10.3390/diagnostics13040657
Hogan RB, 2007, GASTROINTEST ENDOSC, V66, P1018, DOI 10.1016/j.gie.2007.03.1078
Hoteya S, 2017, DIGESTION, V95, P36, DOI 10.1159/000452363
Irani SS, 2023, CLIN GASTROENTEROL H, V21, P1141, DOI 10.1016/j.cgh.2022.12.039
Ling C, 2019, GASTROINTEST ENDOSC, V89, pAB292, DOI 10.1016/j.gie.2019.03.1176
Mohan BP, 2020, ENDOSCOPY, V52, P96, DOI 10.1055/a-1020-3932
Okada K, 2011, AM J GASTROENTEROL, V106, P357, DOI 10.1038/ajg.2010.422
Vanbiervliet G, 2021, ENDOSCOPY, V53, P522, DOI 10.1055/a-1442-2395
Yahagi N, 2018, GASTROINTEST ENDOSC, V88, P676, DOI 10.1016/j.gie.2018.05.002
NR 10
TC 0
Z9 0
U1 0
U2 0
PU LIPPINCOTT WILLIAMS & WILKINS

PI PHILADELPHIA
PA TWO COMMERCE SQ, 2001 MARKET ST, PHILADELPHIA, PA 19103 USA
EI 2326-3253
J9 ACG CASE REP J
JI ACG Case Rep. J.
PD DEC
PY 2023
VL 10
IS 12
AR e01243
DI 10.14309/crj.0000000000001243
PG 4
WC Gastroenterology & Hepatology
WE Emerging Sources Citation Index (ESCI)
SC Gastroenterology & Hepatology
GA CW9G4
UT WOS:001128382800001
PM 38130478
OA Green Submitted, gold
DA 2026-05-09
ER

PT J
AU Madhu, D
Dhir, V
AF Madhu, Deepak
Dhir, Vinay
TI Endoscopic ultrasound-guided biliary interventions
SO INDIAN JOURNAL OF GASTROENTEROLOGY
LA English
DT Review
DE Endoscopic ultrasound-guided biliary drainage; Endoscopic
ultrasound-guided hepaticogastrostomy; Endoscopic ultrasound-guided
choledochoduodenostomy; Endoscopic ultrasound-guided gallbladder
drainage; Interventional endoscopic ultrasound; Malignant biliary
obstruction
ID TRANSMURAL GALLBLADDER DRAINAGE; APPOSING METAL STENT; LEARNING-CURVE;
RETROGRADE CHOLANGIOPANCREATOGRAPHY; INTERNATIONAL MULTICENTER; PRECUT
PAPILLOTOMY; ADVERSE EVENTS; OBSTRUCTION; CHOLEDOCHODUODENOSTOMY; ACCESS
AB Endoscopic ultrasound (EUS)-guided biliary drainage (EUS-BD) includes EUS-guided hepaticogastrostomy (EUS-HGS), EUS-guided choledochoduodenostomy (EUS-CDS), EUS-guided gallbladder drainage (EUS-GBD), EUS-guided antegrade stenting (EUS-AG) and EUS-guided rendezvous (EUS-RV). While EUS-HGS, EUS-CDS and EUS-GBD are

transluminal drainage procedures, EUS-AG is a traspapillary drainage procedure and EUS-RV is a procedure intended to facilitate endoscopic retrograde cholangio pancreatography (ERCP) in instances of failed cannulation. These procedures were initially developed as options for endoscopic salvage of failed ERCP, but have evolved to become first-line interventions also for select indications over time as the technique and expertise improved. Several randomised controlled trials have demonstrated EUS-BD, especially EUS-CDS has similar or even better outcomes as compared to ERCP in malignant biliary obstruction. However, widespread adoption of these modalities is limited by the availability of expertise, steep learning curve, lack of standardization of techniques and cost. In this review, we aim to provide an overview of various EUS-BD procedures including the indications, accessories, technique, outcomes and follow-up of each of these procedures.
C1 [Madhu, Deepak] Caritas Hosp, Dept Gastroenterol, Kottayam 686630, India.
[Dhir, Vinay] SL Raheja Hosp, Inst Digest & Liver Care, Mumbai 400016, India.
RP Dhir, V (corresponding author), SL Raheja Hosp, Inst Digest & Liver Care, Mumbai 400016, India.
EM vinaydhir@gmail.com
RI ; Madhu, Deepak/ABE-3321-2020
OI Dhir, Vinay/0000-0001-6338-6762;
CR Amato A, 2021, ENDOSCOPY, V53, P1037, DOI 10.1055/a-1324-7919
Baars JE, 2018, ENDOSC ULTRASOUND, V7, P4, DOI 10.4103/eus.eus_105_17
Bang JY, 2018, GASTROINTEST ENDOSC, V88, P9, DOI 10.1016/j.gie.2018.03.012
Berry R, 2019, WORLD J GASTRO ENDOS, V11, P5, DOI 10.4253/wjge.v11.i1.5
Binda C, 2021, ENDOSCOPY, V53, pP427
Binmoeller KF, 2011, ENDOSCOPY, V43, P337, DOI 10.1055/s-0030-1256127
Boregowda U, 2023, DIAGNOSTICS, V13, DOI 10.3390/diagnostics13040657
Chang JI, 2019, ENDOSC INT OPEN, V7, pE655, DOI 10.1055/a-0826-4309
Chen YI, 2023, GASTROENTEROLOGY, V165, P1249, DOI 10.1053/j.gastro.2023.07.024
Choudhury A, 2022, ENDOSCOPY, V54, P6
Dhir V, 2022, ENDOSC INT OPEN, V10, pE634, DOI 10.1055/a-1784-3978
Dhir V, 2014, DIGEST ENDOSC, V26, P430, DOI 10.1111/den.12153
Dhir V, 2012, GASTROINTEST ENDOSC, V75, P354, DOI 10.1016/j.gie.2011.07.075
Giovannini M, 2001, ENDOSCOPY, V33, P898, DOI 10.1055/s-2001-17324
Giovannini M, 2012, GASTROINTEST ENDOSC, V75, P441
Giri S, 2023, GASTROINTEST ENDOSC, V98, DOI 10.1016/j.gie.2023.06.055
Guilmoteau T, 2024, MEDICINA-LITHUANIA, V60, DOI 10.3390/medicina60020220
Hirashita T, 2015, Surgical Science, V06, P35, DOI [10.4236/ss.2015.62006, 10.4236/ss.2015.62006, DOI 10.4236/SS.2015.62006]
Imai H, 2016, GASTROINTEST ENDOSC, V84, P147, DOI 10.1016/j.gie.2015.12.024
Irani SS, 2023, CLIN GASTROENTEROL H, V21, P1141, DOI 10.1016/j.cgh.2022.12.039
Isayama H, 2019, J HEPATO-BIL-PAN SCI, V26, P249, DOI 10.1002/jhbp.631
Ishiwatari H, 2021, SURG ENDOSC, V35, P6836, DOI 10.1007/s00464-020-08189-w
Issa D, 2021, ENDOSCOPY, V53, P827, DOI 10.1055/a-1259-0349
Itonaga M, 2021, MEDICINE, V100, DOI 10.1097/MD.0000000000025268
Iwashita T, 2014, CLIN J GASTROENTEROL, V7, P94, DOI 10.1007/s12328-014-0467-5
Jacques J, 2020, GASTROINTEST ENDOSC, V92, P134, DOI 10.1016/j.gie.2020.01.055

James TW, 2019, ENDOSC INT OPEN, V7, pE600, DOI 10.1055/a-0867-9599
Kan M, 2019, INT J GASTROINT INT, V8, P87, DOI 10.18528/ijgii180040
Kawakubo K, 2014, J HEPATO-BIL-PAN SCI, V21, P328, DOI 10.1002/jhbp.27
Khashab MA, 2016, ENDOSC INT OPEN, V4, pE175, DOI 10.1055/s-0041-109083
Ki ELL, 2019, ENDOSC ULTRASOUND, V8, pS17, DOI 10.4103/eus.eus_50_19
Kitagawa K, 2023, SCAND J GASTROENTERO, V58, P296, DOI 10.1080/00365521.2022.2118557
Klair JS, 2023, J CLIN GASTROENTEROL, V57, P211, DOI 10.1097/MCG.0000000000001543
Kongkam P, 2021, ENDOSCOPY, V53, P55, DOI 10.1055/a-1195-8197
Kongkam P, 2019, DIGEST ENDOSC, V31, P50, DOI 10.1111/den.13371
Lambin T.B.J., 2021, Endoscopy, V53, pOP94
Law R, 2016, CLIN GASTROENTEROL H, V14, P476, DOI 10.1016/j.cgh.2015.10.026
Matsubara S, 2022, J CLIN MED, V11, DOI 10.3390/jcm11061591
Matsumoto S, 2020, DIGEST ENDOSC, V32, P957, DOI 10.1111/den.13620
Miutescu B, 2024, DIGEST ENDOSC, V36, P59, DOI 10.1111/den.14667
Miyano A, 2018, J GASTROINTEST SURG, V22, P1312, DOI 10.1007/s11605-018-3758-1
Mohan BP, 2019, ENDOSC ULTRASOUND, V8, P241, DOI 10.4103/eus.eus_63_18
Mukai S, 2023, EXPERT REV GASTROENT, V17, P1197, DOI 10.1080/17474124.2023.2293813
Ogura T, 2021, DEN OPEN, V1, DOI 10.1002/deo2.8
Ogura T, 2021, GUT LIVER, V15, P196
Ogura T, 2015, ENDOSCOPY, V47, pE583, DOI 10.1055/s-0034-1393381
Ogura T, 2015, ENDOSCOPY, V47, P72, DOI 10.1055/s-0034-1378111
Oh D, 2017, THER ADV GASTROENTER, V10, P42, DOI 10.1177/1756283X16671671
Paik WH, 2018, AM J GASTROENTEROL, V113, P987, DOI 10.1038/s41395-018-0122-8
Paleti S, 2019, GASTROINTEST ENDOSC, V89, pAB308, DOI 10.1016/j.gie.2019.03.1212
Park DH, 2011, GASTROINTEST ENDOSC, V74, P1276, DOI 10.1016/j.gie.2011.07.054
Park JK, 2018, GASTROINTEST ENDOSC, V88, P277, DOI 10.1016/j.gie.2018.03.015
Poincloux L, 2015, ENDOSCOPY, V47, P794, DOI [10.1055/s-0035-1545399, 10.1055/s-0034-1391988]
Rai P, 2023, J DIG ENDOSC, V14, P30, DOI 10.1055/s-0043-1761591
Rai P, 2018, ENDOSC INT OPEN, V6, pE67, DOI 10.1055/s-0043-120664
Salerno R, 2019, WORLD J GASTRO ENDOS, V11, P354, DOI 10.4253/wjge.v11.i5.354
Sekine M, 2023, DEN OPEN, V3, DOI 10.1002/deo2.201
Shah RH, 2021, J DIG ENDOSC, V12, P229, DOI 10.1055/s-0041-1741514
Tang R, 2023, GASTROINTEST ENDOSC, V97, pAB932
Teoh AYB, 2023, GASTROENTEROLOGY, V165, P473, DOI 10.1053/j.gastro.2023.04.016
Tsuchiya T, 2016, DIGEST ENDOSC, V28, P96, DOI 10.1111/den.12611
Tyberg A, 2022, ENDOSC ULTRASOUND, V11, P38, DOI 10.4103/EUS-D-21-00006
Tyberg A, 2020, ENDOSC ULTRASOUND, V9, P392, DOI 10.4103/eus.eus_42_20
Uchida D, 2018, J MED ULTRASON, V45, P399, DOI 10.1007/s10396-017-0846-x
van der Merwe SW, 2022, ENDOSCOPY, V54, P185, DOI 10.1055/a-1717-1391
Vargas-Madrigal J, 2024, ENDOSCOPY, V56, P864, DOI 10.1055/a-2340-7432
Yamamoto Y, 2020, ENDOSC ULTRASOUND, V9, P110, DOI 10.4103/eus.eus_68_19
Yamazaki H, 2024, DEN OPEN, V4, DOI 10.1002/deo2.274

Yoon SB, 2024, DIGEST ENDOSC, V36, P129, DOI 10.1111/den.14636
Zhou X, 2023, FRONT ONCOL, V13, DOI 10.3389/fonc.2023.1247763
NR 70
TC 2
Z9 2
U1 0
U2 2
PU SPRINGER INDIA
PI NEW DELHI
PA 7TH FLOOR, VIJAYA BUILDING, 17, BARAKHAMBA ROAD, NEW DELHI, 110 001,
INDIA
SN 0254-8860
EI 0975-0711
J9 INDIAN J GASTROENTER
JI Indian J. Gastroenterol.
PD OCT
PY 2024
VL 43
IS 5
BP 943
EP 953
DI 10.1007/s12664-024-01680-5
EA SEP 2024
PG 11
WC Gastroenterology & Hepatology
WE Emerging Sources Citation Index (ESCI)
SC Gastroenterology & Hepatology
GA J6W5A
UT WOS:001310450000005
PM 39259450
DA 2026-05-09
ER

PT J
AU Fabbri, C
Scalvini, D
Paolo, G
Binda, C
Mauro, A
Coluccio, C
Mazza, S
Trebbi, M

Viera, FT
Anderloni, A
AF Fabbri, Carlo
Scalvini, Davide
Paolo, Giuffrida
Binda, Cecilia
Mauro, Aurelio
Coluccio, Chiara
Mazza, Stefano
Trebbi, Margherita
Viera, Francesca Torello
Anderloni, Andrea
TI Complications and management of interventional endoscopic ultrasound: A
critical review
SO BEST PRACTICE & RESEARCH CLINICAL GASTROENTEROLOGY
LA English
DT Review
DE EUS-complications; Interventional-EUS; Biliary drainages;
Choledochoduodenostomy; Hepaticogastrostomy; EUS-Guided gallbladder
drainage; PFC; EUS-Gastroenterostomy
ID APPOSING-METAL STENT; PANCREATIC FLUID COLLECTIONS; GUIDED GALLBLADDER
DRAINAGE; GASTRIC OUTLET OBSTRUCTION; DIRECTED TRANSGASTRIC ERCP; DISTAL
BILIARY OBSTRUCTION; CELIAC PLEXUS NEUROLYSIS; LONG-TERM OUTCOMES;
NECROTIZING PANCREATITIS; PERCUTANEOUS CHOLECYSTOSTOMY
AB In the last decades, Endoscopic ultrasound (EUS) has rapidly grown and evolved from being mainly a diagnostic procedure, to being an interventional and therapeutic tool in several pathological clinical scenarios. With the progressive growth in technical expertise and dedicated devices, interventional endoscopic ultrasound procedures (IEUSP) have shown high rates of technical and clinical success, together with a relatively safe profile. However, the description and the standardization of different and specific types of adverse events (AEs) are still scarce in literature, and, consequently, even less the management of AEs. The aim of this study is to critical review and to describe AEs related to each of the main IEUSP, and to provide an overview on the possible management strategies of endoscopic complications. Future studies and guidelines are surely required to reach a better standardization of different AEs and their best management.
C1 [Fabbri, Carlo; Paolo, Giuffrida; Binda, Cecilia; Coluccio, Chiara; Trebbi, Margherita] AUSL Romagna, Forli Cesena Hosp, Gastroenterol & Digest Endoscopy Unit, Forli, Italy.
[Scalvini, Davide; Mauro, Aurelio; Mazza, Stefano; Viera, Francesca Torello; Anderloni, Andrea] Fdn IRCCS Policlin San Matteo, Gastroenterol & Digest Endoscopy Unit, Pavia, Italy.
[Scalvini, Davide] Univ Pavia, Dept Internal Med & Therapeut, Pavia, Italy.
C3 AUSL della Romagna; IRCCS Fondazione San Matteo; University of Pavia
RP Paolo, G (corresponding author), AUSL Romagna, Forli Cesena Hosp, Gastroenterol & Digest Endoscopy Unit, Forli, Italy.
EM paologiuffrida1@gmail.com
RI Binda, Cecilia/ABH-1588-2020; Scalvini, Davide/KHX-0744-2024; Giuffrida,

Paolo/AER-7895-2022

OI Scalvini, Davide/0009-0002-7051-5432; Giuffrida,

Paolo/0000-0002-4628-1130

CR Ahmed HM, 2009, ENDOSCOPY, V41, pE218, DOI 10.1055/s-0029-1214941

Amato A, 2021, ENDOSCOPY, V53, P1037, DOI 10.1055/a-1324-7919

Anderloni A, 2023, ENDOSC ULTRASOUND, V12, P59, DOI 10.4103/EUS-D-22-00023

Anderloni A, 2019, GASTROINTEST ENDOSC, V89, P69, DOI [10.1016/j.gie.2018.04.1746, 10.1016/j.gie.2018.08.047]

Anderloni A, 2017, ENDOSC INT OPEN, V5, pE25, DOI 10.1055/s-0042-122009

Anderloni A, 2016, SURG ENDOSC, V30, P5200, DOI 10.1007/s00464-016-4894-x

Armellini E, 2023, WORLD J GASTROENTERO, V29, P3341, DOI 10.3748/wjg.v29.i21.3341

Arvanitakis M, 2018, ENDOSCOPY, V50, P524, DOI 10.1055/a-0588-5365

Auriemma Francesco, 2018, Am J Gastroenterol, V113, P1582, DOI [10.1038/s41395-018-0266-6, 10.1038/s41395-018-0266-6]

Bakker OJ, 2012, JAMA-J AM MED ASSOC, V307, P1053, DOI 10.1001/jama.2012.276

Ban T, 2018, DIGEST ENDOSC, V30, P682, DOI 10.1111/den.13179

Bang JY, 2020, GUT, V69, P1379, DOI 10.1136/gutjnl-2019-320539

Bang JY, 2019, GUT, V68, P1200, DOI 10.1136/gutjnl-2017-315335

Barthet M, 2019, ENDOSCOPY, V51, P836, DOI 10.1055/a-0824-7067

Basha J, 2021, ENDOSC INT OPEN, V09, pE1918, DOI 10.1055/a-1642-7892

Bazarbashi AN, 2024, GASTROINTEST ENDOSC, V99, P31, DOI 10.1016/j.gie.2023.07.043

Bejjani M, 2022, ENDOSCOPY, V54, P680, DOI 10.1055/a-1654-6914

Biedermann Julia, 2020, VideoGIE, V5, P37, DOI [10.1016/j.vgie.2019.09.002, 10.1016/j.vgie.2019.09.002]

Binda Cecilia, 2024, VideoGIE, V9, P182, DOI [10.1016/j.vgie.2023.12.002, 10.1016/j.vgie.2023.12.002]

Binda C, 2024, MINERVA SURG, V79, P183, DOI 10.23736/S2724-5691.23.10132-8

Binda C, 2023, GASTROINTEST ENDOSC, V98, P765, DOI 10.1016/j.gie.2023.06.054

Binda C, 2023, MEDICINA-LITHUANIA, V59, DOI 10.3390/medicina59040750

Binda C, 2023, ENDOSCOPY, V55, pE484, DOI 10.1055/a-2024-9832

Binda C, 2022, ENDOSC ULTRASOUND, V11, P426, DOI 10.4103/EUS-D-21-00083

Bonnot B, 2014, AM J GASTROENTEROL, V109, P607, DOI 10.1038/ajg.2013.473

Boregowda U, 2023, DIAGNOSTICS, V13, DOI 10.3390/diagnostics13040657

Bronswijk M, 2021, GASTROINTEST ENDOSC, V94, P526, DOI 10.1016/j.gie.2021.04.006

Bukhari M, 2018, GASTROINTEST ENDOSC, V88, P486, DOI 10.1016/j.gie.2018.04.2356

Chan SM, 2017, GASTROINTEST ENDOSC, V85, P1225, DOI 10.1016/j.gie.2016.10.014

Chandrasekhara V, 2020, ENDOSC INT OPEN, V08, pE1639, DOI 10.1055/a-1243-0092

Chen YI, 2023, GASTROENTEROLOGY, V165, P1249, DOI 10.1053/j.gastro.2023.07.024

Chen YI, 2018, GASTROINTEST ENDOSC, V87, P1215, DOI 10.1016/j.gie.2017.07.030

Chen YI, 2017, SURG ENDOSC, V31, P2946, DOI 10.1007/s00464-016-5311-1

Cho SH, 2020, GASTROINTEST ENDOSC, V91, P543, DOI 10.1016/j.gie.2019.09.042

Cotton PB, 2010, GASTROINTEST ENDOSC, V71, P446, DOI 10.1016/j.gie.2009.10.027

Crinò SF, 2023, CLIN GASTROENTEROL H, V21, P2834, DOI 10.1016/j.cgh.2023.02.022

Crismale James F, 2016, VideoGIE, V1, P61, DOI [10.1016/j.vgie.2016.09.002, 10.1016/j.vgie.2016.09.002]

Cucchetti A, 2022, GASTROINTEST ENDOSC, V95, P399, DOI 10.1016/j.gie.2021.09.028
De Cassan C, 2017, ENDOSC ULTRASOUND, V6, P329, DOI 10.4103/2303-9027.209869
DeSimone ML, 2017, WORLD J GASTRO ENDOS, V9, P456, DOI 10.4253/wjge.v9.i9.456
DeWitt JM, 2021, CLIN GASTROENTEROL H, V19, P24, DOI 10.1016/j.cgh.2020.09.029
Di Mitri R, 2022, SURG ENDOSC, V36, P4553, DOI 10.1007/s00464-021-08808-0
Dollhopf M, 2017, GASTROINTEST ENDOSC, V86, P636, DOI 10.1016/j.gie.2017.02.027
El Chafic AH, 2019, DIGEST DIS SCI, V64, P3321, DOI 10.1007/s10620-019-05688-2
El Sayed G, 2021, THER ADV GASTROENTER, V14, DOI 10.1177/17562848211042171
Everett SM, 2023, ENDOSCOPY, V55, P952, DOI 10.1055/a-2133-3365
Fabbri C, 2022, SURG ENDOSC, V36, P7974, DOI 10.1007/s00464-022-09339-y
Fabbri C, 2015, GASTROINTEST ENDOSC, V82, P585, DOI 10.1016/j.gie.2015.04.035
Facciorusso A, 2022, DIGEST ENDOSC, V34, P1459, DOI 10.1111/den.14354
Facciorusso A, 2018, ANN TRANSL MED, V6, DOI 10.21037/atm.2018.03.36
Frassei JD, 2021, EINSTEIN-SAO PAULO, V19, DOI [10.31744/einstein_journal/2021ai5778, 10.31744/einstein_journal/2021AI5778]
Fugazza A, 2022, GASTROINTEST ENDOSC, V95, P896, DOI 10.1016/j.gie.2021.12.032
Fugazza A, 2022, HEPATOB PANCREAT DIS, V21, P234, DOI 10.1016/j.hbpd.2022.03.003
Fugazza A, 2022, DIGEST LIVER DIS, V54, P529, DOI 10.1016/j.dld.2021.07.010
Fugazza A, 2020, GASTROINTEST ENDOSC, V91, P574, DOI 10.1016/j.gie.2019.11.021
Garcia-Sumalla A, 2021, SURG ENDOSC, V35, P4873, DOI 10.1007/s00464-021-08435-9
Ge N, 2016, BMC GASTROENTEROL, V16, DOI 10.1186/s12876-016-0420-9
Ge PS, 2019, SURG ENDOSC, V33, P3404, DOI 10.1007/s00464-018-06636-3
Ghandour B, 2022, GASTROINTEST ENDOSC, V95, P80, DOI 10.1016/j.gie.2021.07.023
Ginestet C, 2022, SURG ENDOSC, V36, P3365, DOI 10.1007/s00464-021-08653-1
Giovannini M, 2004, BEST PRACT RES CL GA, V18, P183, DOI 10.1016/S1521-6918(03)00103-3
Giri S, 2023, GASTROINTEST ENDOSC, V98, DOI 10.1016/j.gie.2023.06.055
Gralnek IM, 2022, ENDOSCOPY, V54, P1094, DOI 10.1055/a-1939-4887
Gralnek IM, 2021, ENDOSCOPY, V53, P300, DOI 10.1055/a-1369-5274
Gress F, 2001, AM J GASTROENTEROL, V96, P409
Guzmán-Calderón E, 2022, J HEPATO-BIL-PAN SCI, V29, P198, DOI 10.1002/jhbp.1008
Hajifathalian K, 2022, GASTROINTEST ENDOSC, V95, P703, DOI 10.1016/j.gie.2021.11.037
Han D, 2018, J CLIN GASTROENTEROL, V52, P835, DOI 10.1097/MCG.0000000000000934
Hedjoudje A, 2019, UNITED EUR GASTROENT, V7, P60, DOI 10.1177/2050640618808147
Hemerly MC, 2023, SURG ENDOSC, V37, P2421, DOI 10.1007/s00464-022-09712-x
Iqbal U, 2020, ENDOSC ULTRASOUND, V9, P16, DOI 10.4103/eus.eus_70_19
Irani Shayan, 2020, VideoGIE, V5, P48, DOI [10.1016/j.vgie.2019.10.007, 10.1016/j.vgie.2019.10.007]
Irani SS, 2023, ANN SURG, V278, pE556, DOI 10.1097/SLA.0000000000005784
Ishii S, 2023, ENDOSC ULTRASOUND, V12, P266, DOI 10.4103/EUS-D-22-00104
Jacques J, 2019, ENDOSCOPY, V51, P540, DOI 10.1055/a-0735-9137
James TW, 2019, OBES SURG, V29, P451, DOI 10.1007/s11695-018-3531-2
Kamal F, 2023, ENDOSC ULTRASOUND, V12, P8, DOI 10.4103/EUS-D-21-00206
Keane MG, 2023, GASTROINTEST ENDOSC, V97, P291, DOI 10.1016/j.gie.2022.10.004

Kedia P, 2019, J CLIN GASTROENTEROL, V53, P304, DOI 10.1097/MCG.0000000000001037
Kedia P, 2014, GASTROENTEROLOGY, V147, P566, DOI 10.1053/j.gastro.2014.05.045
Kinnersley P, 2013, COCHRANE DB SYST REV, DOI 10.1002/14651858.CD009445.pub2
Kitamura H, 2022, J HEPATO-BIL-PAN SCI, V29, P404, DOI 10.1002/jhbp.1059
Krishnamoorthi R, 2022, ENDOSC INT OPEN, V10, pE874, DOI 10.1055/a-1794-0635
Kunda R, 2016, SURG ENDOSC, V30, P5002, DOI 10.1007/s00464-016-4845-6
Lakhtakia S, 2016, BEST PRACT RES CL GA, V30, P807, DOI 10.1016/j.bpg.2016.10.008
LeBlanc JK, 2011, GASTROINTEST ENDOSC, V74, P1300, DOI 10.1016/j.gie.2011.07.073
Lera ME, 2019, ENDOSCOPY, V51, pE77, DOI 10.1055/a-0820-1456
Li JS, 2023, MINIM INVASIV THER, V32, P285, DOI 10.1080/13645706.2023.2221336
Ligresti D, 2018, ENDOSCOPY, V50, P822, DOI 10.1055/a-0624-2050
Lisotti A, 2022, MINERVA GASTROENTERO, V68, P154, DOI 10.23736/S2724-5985.21.02854-3
Majmudar K, 2016, GASTROINTEST ENDOSC, V84, P853, DOI 10.1016/j.gie.2016.06.007
Mandarino FV, 2024, GASTROINTEST ENDOSC, V99, P914, DOI 10.1016/j.gie.2023.12.023
Mangiavillano B, 2024, DIGEST ENDOSC, V36, P351, DOI 10.1111/den.14606
Mangiavillano B, 2022, GASTROINTEST ENDOSC, V95, P115, DOI 10.1016/j.gie.2021.07.021
Manno M, 2020, ENDOSCOPY, V52, pE144, DOI 10.1055/a-1032-8218
McCarty Thomas R, 2019, Endosc Int Open, V7, pE1474, DOI [10.1055/a-0996-8178, 10.1055/a-0996-8178]
Miller C, 2023, ENDOSC INT OPEN, V11, pE660, DOI 10.1055/a-2098-2570
Miura F, 2018, J HEPATO-BIL-PAN SCI, V25, P31, DOI 10.1002/jhbp.509
Mohan BP, 2019, J CLIN GASTROENTEROL, V53, P243, DOI 10.1097/MCG.0000000000001167
Monino L, 2023, ENDOSCOPY, V55, P991, DOI 10.1055/a-2119-7529
Muscatiello N, 2006, ENDOSCOPY, V38, P858, DOI 10.1055/s-2006-925382
Mutignani M, 2023, ENDOSC INT OPEN, V11, pE394, DOI 10.1055/a-2032-3077
Mutignani M, 2015, ENDOSCOPY, V47, pE350, DOI 10.1055/s-0034-1392424
Nakai Y, 2020, GASTROINTEST ENDOSC, V92, P623, DOI 10.1016/j.gie.2020.03.3856
Ngamruengphong S, 2017, ENDOSCOPY, V49, P549, DOI 10.1055/s-0043-105072
Ogura T, 2021, DEN OPEN, V1, DOI 10.1002/deo2.8
Oleas R, 2019, THER ADV GASTROINTES, V12, DOI 10.1177/2631774519878282
Paik WH, 2019, ENDOSC ULTRASOUND, V8, pS44, DOI 10.4103/eus.eus_51_19
Pan MX, 2019, ENERGIES, V12, DOI [10.3390/en12173284, 10.20524/aog.2021.0650]
Perez-Miranda M, 2018, ENDOSC ULTRASOUND, V7, P79, DOI 10.4103/eus.eus_5_18
Perez-Miranda M, 2017, J CLIN GASTROENTEROL, V51, P896, DOI 10.1097/MCG.0000000000000887
Prakash S, 2022, ENDOSCOPY, V54, P52, DOI 10.1055/a-1376-2394
Rana SS, 2019, ANN GASTROENTEROL, V32, P441, DOI 10.20524/aog.2019.0404
Rickman OB, 2004, MAYO CLIN PROC, V79, P1455, DOI 10.4065/79.11.1455
Robles-Medranda C, 2019, DIGEST ENDOSC, V31, P283, DOI 10.1111/den.13305
Rodrigues-Pinto E, 2016, GASTROINTEST ENDOSC, V83, P853, DOI 10.1016/j.gie.2015.11.017
Runge TM, 2021, ENDOSCOPY, V53, P611, DOI 10.1055/a-1254-3942
Saftoiu A, 2013, BMC GASTROENTEROL, V13, DOI 10.1186/1471-230X-13-93
Samanta J, 2023, LIVER INT, V43, P1783, DOI 10.1111/liv.15630
Shinn B, 2021, GASTROINTEST ENDOSC, V94, P727, DOI 10.1016/j.gie.2021.04.022

Sidhu R, 2024, GUT, V73, P219, DOI 10.1136/gutjnl-2023-330396
Spadaccini M, 2022, MEDICINA-LITHUANIA, V58, DOI 10.3390/medicina58030331
Storm AC, 2016, GASTROINTEST ENDOSC, V83, P1037, DOI 10.1016/j.gie.2015.11.004
Tarantino I, 2014, ENDOSCOPY, V46, pE71, DOI 10.1055/s-0033-1359164
Teoh AYB, 2018, GUT, V67, P1209, DOI 10.1136/gutjnl-2017-314341
Teoh AYB, 2023, GASTROENTEROLOGY, V165, P473, DOI 10.1053/j.gastro.2023.04.016
Teoh AYB, 2017, ENDOSCOPY, V49, P130, DOI 10.1055/s-0042-119036
Tsuchiya T, 2018, GASTROINTEST ENDOSC, V87, P1138, DOI 10.1016/j.gie.2017.08.017
Tyberg A, 2016, ENDOSC INT OPEN, V4, pE276, DOI 10.1055/s-0042-101789
Tyberg A, 2016, WORLD J GASTROENTERO, V22, P2256, DOI 10.3748/wjg.v22.i7.2256
van Brunschot S, 2014, SURG ENDOSC, V28, P1425, DOI 10.1007/s00464-013-3382-9
van der Merwe SW, 2022, ENDOSCOPY, V54, P185, DOI 10.1055/a-1717-1391
van Wanrooij RLJ, 2022, ENDOSCOPY, V54, P1023, DOI 10.1055/a-1782-7568
van Wanrooij RLJ, 2022, ENDOSCOPY, V54, P310, DOI 10.1055/a-1738-6780
Vanderschueren E, 2023, SEMIN LIVER DIS, V43, P176, DOI 10.1055/s-0043-1769009
Vanek P, 2023, GASTROINTEST ENDOSC, V97, P1070, DOI 10.1016/j.gie.2022.12.026
Vanella G, 2023, DIGEST ENDOSC, V35, P377, DOI 10.1111/den.14445
Vanella Giuseppe, 2022, VideoGIE, V7, P120, DOI [10.1016/j.vgie.2021.10.002, 10.1016/j.vgie.2021.10.002]
Varadarajulu S, 2013, GASTROENTEROLOGY, V145, P583, DOI 10.1053/j.gastro.2013.05.046
Varadarajulu S, 2011, J GASTROEN HEPATOL, V26, P1504, DOI 10.1111/j.1440-1746.2011.06771.x
Venkatachalapathy SV, 2018, ENDOSC INT OPEN, V6, pE259, DOI 10.1055/s-0043-125362
Voermans RP, 2015, J HEPATO-BIL-PAN SCI, V22, P20, DOI 10.1002/jhbp.180
von Renteln D, 2010, GASTROINTEST ENDOSC, V72, P1289, DOI 10.1016/j.gie.2010.07.033
Wang SP, 2016, ENDOSCOPY, V48, pE28, DOI 10.1055/s-0042-100454
Wang TJ, 2019, SURG ENDOSC, V33, P2024, DOI 10.1007/s00464-019-06715-z
Wiechowska-Kozlowska A, 2012, GASTROENT RES PRACT, V2012, DOI 10.1155/2012/503098
Wiersema MJ, 1996, GASTROINTEST ENDOSC, V44, P656, DOI 10.1016/S0016-5107(96)70047-0
Wong T, 2020, LOCAL REG ANESTH, V13, P1, DOI 10.2147/LRA.S181458
Wyse JM, 2011, J CLIN ONCOL, V29, P3541, DOI 10.1200/JCO.2010.32.2750
Yamazaki H, 2024, DEN OPEN, V4, DOI 10.1002/deo2.274
Yang D, 2018, ENDOSC INT OPEN, V6, pE1267, DOI 10.1055/a-0732-502
Yang MJ, 2018, J GASTROEN HEPATOL, V33, P772, DOI 10.1111/jgh.14060
Yasuda I, 2013, ENDOSCOPY, V45, P627, DOI 10.1055/s-0033-1344027
NR 153
TC 7
Z9 7
U1 1
U2 6
PU ELSEVIER SCI LTD
PI London
PA 125 London Wall, London, ENGLAND
SN 1521-6918

EI 1532-1916
J9 BEST PRACT RES CL GA
JI Best Pract. Res. Clin. Gastroenterol.
PD MAR
PY 2024
VL 69
AR 101888
DI 10.1016/j.bpg.2024.101888
EA MAY 2024
PG 12
WC Gastroenterology & Hepatology
WE Science Citation Index Expanded (SCI-EXPANDED)
SC Gastroenterology & Hepatology
GA TM5T8
UT WOS:001241701000001
PM 38749575
DA 2026-05-09
ER

PT J
AU Issa, D
Irani, S
Law, R
Shah, S
Bhalla, S
Mahadev, S
Hajifathalian, K
Sampath, K
Mukewar, S
Carr-Locke, DL
Khashab, MA
Sharaiha, RZ
AF Issa, Danny
Irani, Shayan
Law, Ryan
Shah, Shawn
Bhalla, Sean
Mahadev, Srihari
Hajifathalian, Kaveh
Sampath, Kartik
Mukewar, Saurabh
Carr-Locke, David L.

Khashab, Mouen A.
Sharaiha, Reem Z.
TI Endoscopic ultrasound-guided gallbladder drainage as a rescue therapy
for unresectable malignant biliary obstruction: a multicenter experience
SO ENDOSCOPY
LA English
DT Article
ID STENT
AB Background Endoscopic retrograde cholangiopancreatography (ERCP) is often unsuccessful in patients with duodenal stenosis or malignant ampullary infiltration. While endoscopic ultrasound-guided biliary drainage (EUS-BD) has been proposed as an alternative, EUS-guided gallbladder drainage (EUS-GBD) is an attractive option when both approaches fail. We aimed to assess the effectiveness and safety of EUS-GBD as rescue therapy for malignant distal bile duct obstruction.

Methods A multicenter retrospective study was performed on patients with unresectable malignant distal bile duct obstruction who underwent EUS-GBD between 2014 and 2019 after unsuccessful ERCP and EUS-BD. Clinical success was defined as a decrease in serum bilirubin of >50% within 2 weeks.

Results 28 patients were included, with a lumen-apposing metal stent used in 26 (93%) and a self-expandable metal stent in two (7%). The technical success rate was 100%. The clinical success rate was 93%, with an improvement in bilirubin (7.3 [SD 5.4] pre-procedure vs. 2.8 [SD 1.1] post-procedure; P =0.001). Delayed adverse events included food impaction of the stent (n=3), with a further two patients developing cholecystitis and bleeding.

Conclusion This study demonstrates the feasibility of gallbladder drainage to relieve malignant distal bile duct obstruction in patients with failed ERCP and EUS-BD.
C1 [Issa, Danny; Shah, Shawn; Mahadev, Srihari; Hajifathalian, Kaveh; Sampath, Kartik; Mukewar, Saurabh; Carr-Locke, David L.; Sharaiha, Reem Z.] New York Presbyterian Hosp, Div Gastroenterol & Hepatol, New York, NY USA.
[Issa, Danny; Shah, Shawn; Mahadev, Srihari; Hajifathalian, Kaveh; Sampath, Kartik; Mukewar, Saurabh; Carr-Locke, David L.; Sharaiha, Reem Z.] Weill Cornell Med Ctr, New York, NY USA.
[Irani, Shayan; Bhalla, Sean] Virginia Mason Med Ctr, Div Gastroenterol & Hepatol, Seattle, WA 98101 USA.
[Law, Ryan] Univ Michigan, Div Gastroenterol & Hepatol, Ann Arbor, MI 48109 USA.
[Khashab, Mouen A.] Johns Hopkins Univ Hosp, Div Gastroenterol & Hepatol, Baltimore, MD 21287 USA.
C3 Cornell University; Weill Cornell Medicine; NewYork-Presbyterian
Hospital; Cornell University; Weill Cornell Medicine; Weill Cornell
Medical Center; Virginia Mason Medical Center; University of Michigan
System; University of Michigan; Johns Hopkins University; Johns Hopkins
Medicine
RP Sharaiha, RZ (corresponding author), Weill Cornell Med Coll, Div Gastroenterol & Hepatol, 1305 York Ave Fourth Floor, New York, NY 10021 USA.
EM rzs9001@med.cornell.edu
RI Hajifathalian, Kaveh/AAX-6179-2021; khashab, mouen/F-6643-2017
OI Issa, Danny/0000-0002-0343-1397
CR Bishay K, 2019, ENDOSCOPY, V51, P950, DOI 10.1055/a-0901-7343
Chang JI, 2019, ENDOSC INT OPEN, V7, pE655, DOI 10.1055/a-0826-4309
Chung YH, 2012, J CLIN GASTROENTEROL, V46, P216, DOI 10.1097/MCG.0b013e3182274375
Cotton PB, 2010, GASTROINTEST ENDOSC, V71, P446, DOI 10.1016/j.gie.2009.10.027

Das A, 2000, Cancer Control, V7, P452
Imai H, 2016, GASTROINTEST ENDOSC, V84, P147, DOI 10.1016/j.gie.2015.12.024
Itoi T, 2013, DIGEST ENDOSC, V25, P137, DOI 10.1111/den.12084
Kedia P, 2015, GASTROINTEST ENDOSC, V82, P1031, DOI 10.1016/j.gie.2015.03.1912
Luk SWY, 2019, ENDOSCOPY, V51, P722, DOI 10.1055/a-0929-6603
McKay A, 2012, SURG ENDOSC, V26, P1343, DOI 10.1007/s00464-011-2035-0
Nakai Y, 2016, GASTROINTEST ENDOSC, V83, P1302, DOI 10.1016/j.gie.2016.01.015
Paik WH, 2018, AM J GASTROENTEROL, V113, P987, DOI 10.1038/s41395-018-0122-8
Park JK, 2018, GASTROINTEST ENDOSC, V88, P277, DOI 10.1016/j.gie.2018.03.015
SPEER AG, 1987, LANCET, V2, P57, DOI 10.1016/S0140-6736(87)92733-4
Tuca Albert, 2012, Cancer Manag Res, V4, P159, DOI 10.2147/CMAR.S29297
NR 15
TC 54
Z9 61
U1 0
U2 3
PU GEORG THIEME VERLAG KG
PI STUTTGART
PA Oswald-Hesse-Strasse 50, D-70469 STUTTGART, GERMANY
SN 0013-726X
EI 1438-8812
J9 ENDOSCOPY
JI Endoscopy
PD AUG
PY 2021
VL 53
IS 8
BP 827
EP 831
DI 10.1055/a-1259-0349
EA SEP 2020
PG 5
WC Gastroenterology & Hepatology; Surgery
WE Science Citation Index Expanded (SCI-EXPANDED)
SC Gastroenterology & Hepatology; Surgery
GA TV4TM
UT WOS:000599330400001
PM 32898918
DA 2026-05-09
ER

PT J

AU Mukai, S
Itoi, T
Tsuchiya, T
Ishii, K
Tanaka, R
Tonozuka, R
Furuichi, Y
Joyama, E
Miyazawa, H
Sofuni, A
AF Mukai, Shuntaro
Itoi, Takao
Tsuchiya, Takayoshi
Ishii, Kentaro
Tanaka, Reina
Tonozuka, Ryosuke
Furuichi, Yoshihiro
Joyama, Eri
Miyazawa, Hideaki
Sofuni, Atsushi
TI Experimental study of a physician-controlled electrocautery-enhanced
delivery system incorporating a newly developed lumen-apposing metal
stent for interventional endoscopic ultrasound (with videos)
SO JOURNAL OF HEPATO-BILIARY-PANCREATIC SCIENCES
LA English
DT Article
DE animal; drainage; endosonography; models; stents
ID PANCREATIC FLUID COLLECTIONS; EUS-GUIDED DRAINAGE; GALLBLADDER DRAINAGE;
CLINICAL-EVALUATION; NECROSIS
AB Background/Purpose Although the lumen-apposing metal stent (LAMS) is useful for interventional endoscopic ultrasound (EUS) procedures, there has been some concern about the potential for stent-induced adverse events because of the high lumen-apposing force. A newly designed LAMS with less lumen-apposing force has been developed for use with a physician-controlled electrocautery-enhanced delivery system. The aim of this animal study was to evaluate the feasibility of performing interventional EUS using this newly designed LAMS system. Methods Endoscopic ultrasound-guided cystogastrostomy was performed using the novel LAMS three times in a wet simulation model. EUS-guided gastroenterostomy and EUS-guided gallbladder drainage were then performed using the system in four pigs. Results The LAMS was successfully placed in all three EUS-guided cystogastrostomy procedures using the wet simulation model and in all four EUS-guided gastroenterostomy and gallbladder drainage procedures in the animal model. In the 3 weeks following the procedure, eating behavior was normal in all animals and there were no adverse events. The stents remained patent during this time and were removed without difficulty. The fistula was mature in all cases and a standard upper gastrointestinal endoscope was easily advanced via the fistula to observe the afferent and efferent loops or the lumen of the gallbladder. Necropsy confirmed complete adhesion between the stomach and the wall of the jejunum or gallbladder.

Conclusions Our study findings demonstrate the feasibility of this new LAMS system and its potential clinical value for interventional EUS.
C1 [Mukai, Shuntaro; Itoi, Takao; Tsuchiya, Takayoshi; Ishii, Kentaro; Tanaka, Reina; Tonozuka, Ryosuke; Furuichi, Yoshihiro; Sofuni, Atsushi] Tokyo Med Univ, Dept Gastroenterol & Hepatol, Tokyo, Japan.
[Joyama, Eri] Tokyo Med Univ, Dept Int Med Care, Tokyo, Japan.
[Miyazawa, Hideaki] Tokyo Kamata Med Ctr, Dept Gastroenterol, Tokyo, Japan.
C3 Tokyo Medical University; Tokyo Medical University
RP Itoi, T (corresponding author), Tokyo Med Univ, Dept Gastroenterol & Hepatol, Shinjuku Ku, 6-7-1 Nishishinjuku, Tokyo 1600023, Japan.
EM itoi@tokyo-med.ac.jp
RI Mukai, Shuntaro/AAD-4278-2020; Ishii, Kentaro/AAW-1590-2020; Furuichi, Yoshihiro/AAS-4980-2020
OI Mukai, Shuntaro/0000-0001-5737-620X;
CR Bang JY, 2019, DIGEST ENDOSC, V31, P619, DOI 10.1111/den.13428
Bang JY, 2019, GUT, V68, P1200, DOI 10.1136/gutjnl-2017-315335
Binmoeller KF, 2011, ENDOSCOPY, V43, P337, DOI 10.1055/s-0030-1256127
Brimhall B, 2018, CLIN GASTROENTEROL H, V16, P1521, DOI 10.1016/j.cgh.2018.02.021
Chandran S, 2015, GASTROINTEST ENDOSC, V81, P127, DOI 10.1016/j.gie.2014.06.025
Chen YI, 2018, GASTROINTEST ENDOSC, V87, P1215, DOI 10.1016/j.gie.2017.07.030
Fabbri C, 2015, GASTROINTEST ENDOSC, V82, P585, DOI 10.1016/j.gie.2015.04.035
Itoi T, 2017, DIGEST ENDOSC, V29, P495, DOI 10.1111/den.12794
Itoi T, 2016, GUT, V65, P193, DOI 10.1136/gutjnl-2015-310348
Itoi T, 2013, GASTROINTEST ENDOSC, V78, P934, DOI 10.1016/j.gie.2013.09.025
Itoi T, 2012, GASTROINTEST ENDOSC, V75, P870, DOI 10.1016/j.gie.2011.10.020
Lakhtakia Sundeep, 2018, VideoGIE, V3, P284, DOI [10.1016/j.vgie.2018.07.001, 10.1016/j.vgie.2018.07.001]
Ligresti D, 2018, ENDOSCOPY, V50, P822, DOI 10.1055/a-0624-2050
Mukai S, 2017, GASTROINTEST ENDOSC, V86, P203, DOI [10.1016/j.gie.2016.07.056, 10.1016/j.gie.2016.11.025, 10.1016/j.gie.2016.07.056]
Mukai S, 2015, ENDOSC ULTRASOUND, V4, P120, DOI 10.4103/2303-9027.156738
Mukai S, 2015, ENDOSCOPY, V47, P47, DOI 10.1055/s-0034-1377966
Rinninella E, 2015, GASTROINTEST ENDOSC, V82, P1039, DOI 10.1016/j.gie.2015.04.006
Sanchez-Ocana R, 2017, GASTROINTEST ENDOSC, V86, P229, DOI 10.1016/j.gie.2017.03.023
Siddiqui AA, 2016, GASTROINTEST ENDOSC, V83, P699, DOI 10.1016/j.gie.2015.10.020
Song TJ, 2019, GASTROINTEST ENDOSC, V90, P507, DOI 10.1016/j.gie.2019.05.033
Teoh AY, 2016, GASTROINTEST ENDOSC, V84, P62, DOI 10.1016/j.gie.2015.11.041
Teoh AYB, 2020, DIGEST ENDOSC, V32, P391, DOI 10.1111/den.13494
Tsuchiya T, 2018, GASTROINTEST ENDOSC, V87, P1138, DOI 10.1016/j.gie.2017.08.017
Walter D, 2016, GUT, V65, P6, DOI 10.1136/gutjnl-2015-309925
NR 24
TC 9
Z9 10
U1 0

U2 0
PU WILEY
PI HOBOKEN
PA 111 RIVER ST, HOBOKEN 07030-5774, NJ USA
SN 1868-6974
EI 1868-6982
J9 J HEPATO-BIL-PAN SCI
JI J. Hepato-Biliary-Pancreat. Sci.
PD JUL
PY 2022
VL 29
IS 7
BP 817
EP 824
DI 10.1002/jhbp.1113
EA JAN 2022
PG 8
WC Gastroenterology & Hepatology; Surgery
WE Science Citation Index Expanded (SCI-EXPANDED)
SC Gastroenterology & Hepatology; Surgery
GA 3C2FN
UT WOS:000746646000001
PM 35030302
DA 2026-05-09
ER

PT J
AU Ogawa, T
Kanno, Y
Koshita, S
Kusunose, H
Sakai, T
Yonamine, K
Miyamoto, K
Kozakai, F
Okano, H
Anan, H
Hosokawa, K
Ito, K
AF Ogawa, Takahisa
Kanno, Yoshihide
Koshita, Shinsuke

Kusunose, Hiroaki
Sakai, Toshitaka
Yonamine, Keisuke
Miyamoto, Kazuaki
Kozakai, Fumisato
Okano, Haruka
Anan, Hideyuki
Hosokawa, Kento
Ito, Kei
TI Prospective feasibility study on the efficacy and safety of a novel spiral dilator for endoscopic ultrasound-guided drainage
SO DEN OPEN
LA English
DT Article
DE endoscopic ultrasound-guided biliary drainage; endoscopic ultrasound-guided gallbladder drainage; endoscopic ultrasound-guided hepaticogastrostomy; endoscopic ultrasound-guided pancreatic duct drainage; Tornus ES
ID PANCREATIC-DUCT DRAINAGE; BILIARY DRAINAGE
AB Objectives: A difficult step in endoscopic ultrasound (EUS)-guided drainage procedures is dilation of the puncture tract before stent deployment. The efficacy and safety of a novel spiral dilator, Tornus ES, for EUS-guided drainage were investigated in this study.

Methods: This study was conducted as a prospective, single-arm, observational study at Sendai City Medical center. Dilation of the puncture tract using a spiral dilator was attempted for all EUS-guided drainage cases. The primary outcome was the technical success rate which was defined as successful stent placement in the puncture tract. Secondary outcomes were the success rate of dilation using a spiral dilator, procedure time, and adverse events related to the procedures.

Results: A total of 10 patients were enrolled between January and March 2022. Seven patients underwent EUS-guided biliary drainage (hepaticogastrostomy for six and hepaticojejunostomy for one), and the remaining three patients underwent EUS-guided gallbladder drainage. The technical success rate and the success rate of dilation using a spiral dilator were both 100%. The mean procedure time was 27 min. No adverse events related to the procedure occurred in all cases.

Conclusions: Dilation of the puncture tract using a spiral dilator was effective and safe and might make it easier to perform EUS-guided drainage.
C1 [Ogawa, Takahisa; Kanno, Yoshihide; Koshita, Shinsuke; Kusunose, Hiroaki; Sakai, Toshitaka; Yonamine, Keisuke; Miyamoto, Kazuaki; Kozakai, Fumisato; Okano, Haruka; Anan, Hideyuki; Hosokawa, Kento; Ito, Kei] Sendai City Med Ctr, Dept Gastroenterol, 5-22-1 Tsurugaya,Miyagino Ku, Sendai, Miyagi 9830824, Japan.
RP Ogawa, T (corresponding author), Sendai City Med Ctr, Dept Gastroenterol, 5-22-1 Tsurugaya,Miyagino Ku, Sendai, Miyagi 9830824, Japan.
EM t-ogawa@openhp.or.jp
RI Miyamoto, Kazuaki/OLR-0799-2025
OI Koshita, Shinsuke/0000-0001-6880-5532
CR Amano M, 2017, J GASTROEN HEPATOL, V32, P716, DOI 10.1111/jgh.13489
Bang JY, 2018, GASTROINTEST ENDOSC, V88, P9, DOI 10.1016/j.gie.2018.03.012

Cotton PB, 2010, GASTROINTEST ENDOSC, V71, P446, DOI 10.1016/j.gie.2009.10.027
Hara K, 2022, J HEPATO-BIL-PAN SCI, V29, pE91, DOI 10.1002/jhbp.1130
Hedjoudje A, 2019, UNITED EUR GASTROENT, V7, P60, DOI 10.1177/2050640618808147
Honjo M, 2018, ENDOSC ULTRASOUND, V7, P376, DOI 10.4103/eus.eus_2_18
Kanno Yoshihide, 2020, VideoGIE, V5, P355, DOI [10.1016/j.vgie.2020.04.003, 10.1016/j.vgie.2020.04.003]
Kanno Y, 2017, WORLD J GASTRO ENDOS, V9, P304, DOI 10.4253/wjge.v9.i7.304
Khan MA, 2016, DIGEST DIS SCI, V61, P684, DOI 10.1007/s10620-015-3933-0
Kozakai F, 2022, DIGEST ENDOSC, V34, pE66, DOI 10.1111/den.14258
Kozakai F, 2019, CLIN ENDOSC, V52, P262, DOI 10.5946/ce.2018.183
Mohan BP, 2019, J CLIN GASTROENTEROL, V53, P243, DOI 10.1097/MCG.0000000000001167
Ogura T, 2019, J HEPATO-BIL-PAN SCI, V26, pE3, DOI 10.1002/jhbp.609
OKEN MM, 1982, AM J CLIN ONCOL-CANC, V5, P649, DOI 10.1097/00000421-198212000-00014
Paik WH, 2018, AM J GASTROENTEROL, V113, P987, DOI 10.1038/s41395-018-0122-8
Park JK, 2018, GASTROINTEST ENDOSC, V88, P277, DOI 10.1016/j.gie.2018.03.015
Pausawasdi N, 2021, J MED ULTRASOUND, V29, P250, DOI 10.4103/JMU.JMU_148_20
Sakai T, 2022, PANCREATOLOGY, V22, P58, DOI 10.1016/j.pan.2021.10.006
Vila JJ, 2012, GASTROINTEST ENDOSC, V76, P1133, DOI 10.1016/j.gie.2012.08.001
Wang KX, 2016, GASTROINTEST ENDOSC, V83, P1218, DOI 10.1016/j.gie.2015.10.033
NR 20
TC 9
Z9 8
U1 0
U2 4
PU WILEY
PI HOBOKEN
PA 111 RIVER ST, HOBOKEN 07030-5774, NJ USA
SN 2692-4609
J9 DEN OPEN
JI DEN Open
PD APR
PY 2023
VL 3
IS 1
AR e170
DI 10.1002/deo2.170
PG 6
WC Gastroenterology & Hepatology
WE Emerging Sources Citation Index (ESCI)
SC Gastroenterology & Hepatology
GA W4OQ6
UT WOS:001091438100047
PM 36262217

OA Green Submitted, gold
DA 2026-05-09
ER

PT J
AU Dharan, M
AF Dharan, Murali
TI Stent occlusion in endoscopic ultrasound-guided gallbladder drainage
from bleeding mitigated by double pigtail plastic stent deployment
within lumen apposing metal stent
SO INTERNATIONAL JOURNAL OF GASTROINTESTINAL INTERVENTION
LA English
DT Article
DE Cholecystitis; Endoscopic ultrasound; Gallbladder drainage
AB Double pigtail plastic stents (DPTPS) are routinely placed with the lumen apposing metal stent (LAMS) during endoscopic ultrasound-guided gallbladder drainage. Several reasons are postulated for this practice. This case report highlights a previously unreported benefit from use of DPTPS within LAMS. Copyright (C) 2021, Society of Gastrointestinal Intervention.
C1 [Dharan, Murali] Univ Connecticut, Ctr Hlth, Dept Gastroenterol & Hepatol, 263 Farmington Ave, Farmington, CT 06030 USA.
C3 University of Connecticut
RP Dharan, M (corresponding author), Univ Connecticut, Ctr Hlth, Dept Gastroenterol & Hepatol, 263 Farmington Ave, Farmington, CT 06030 USA.
EM dharan@uchc.edu
OI Dharan, Murali/0000-0002-9764-0068
CR Adler DG, 2019, AM J GASTROENTEROL, V114, P700, DOI 10.14309/ajg.0000000000000067
Gornals JB, 2016, DIGEST ENDOSC, V28, P100, DOI 10.1111/den.12548
James TW, 2019, ENDOSC ULTRASOUND, V8, pS28, DOI 10.4103/eus.eus_41_19
Lisotti A, 2020, ENDOSCOPY, V52, P517, DOI [10.1055/a-1065-1678, 10.1055/a-1157-6615, 10.1055/a-1065-1678]
Manta R, 2018, EUR J GASTROEN HEPAT, V30, P695, DOI 10.1097/MEG.0000000000001112
Perez-Miranda M, 2018, ENDOSC ULTRASOUND, V7, P79, DOI 10.4103/eus.eus_5_18
Saumoy M, 2018, ENDOSC ULTRASOUND, V7, P97, DOI 10.4103/eus.eus_9_18
Siddiqui A, 2019, SURG ENDOSC, V33, P1260, DOI 10.1007/s00464-018-6406-7
Teoh AYB, 2017, ENDOSCOPY, V49, P130, DOI 10.1055/s-0042-119036
NR 9
TC 1
Z9 1
U1 0
U2 0
PU SOC GASTROINTESTINAL INTERVENTION
PI SEOUL
PA 12, HAKDONG-RO 31, GANGNAM-GU, SEOUL, 00000, SOUTH KOREA

SN 2636-0004
EI 2636-0012
J9 INT J GASTROINT INT
JI Int. J. Gastrointest. Interv.
PD OCT
PY 2021
VL 10
IS 4
BP 189
EP 191
DI 10.18528/ijgii210005
PG 3
WC Gastroenterology & Hepatology
WE Emerging Sources Citation Index (ESCI)
SC Gastroenterology & Hepatology
GA XG7ZV
UT WOS:000724968800008
OA Green Submitted, gold
DA 2026-05-09
ER

PT J
AU Anderloni, A
Troncone, E
Fugazza, A
Cappello, A
Blanco, GD
Monteleone, G
Repici, A
AF Anderloni, Andrea
Troncone, Edoardo
Fugazza, Alessandro
Cappello, Annalisa
Blanco, Giovanna Del Vecchio
Monteleone, Giovanni
Repici, Alessandro
TI Lumen-apposing metal stents for malignant biliary obstruction: Is this
the ultimate horizon of our experience?
SO WORLD JOURNAL OF GASTROENTEROLOGY
LA English
DT Review
DE Interventional endoscopic ultrasonography; Endoscopic

ultrasonography-guided biliary drainage; Endoscopic
ultrasonography-guided choledocho-duodenostomy; Biliary metal stent;
Lumen-apposing metal stent
ID EUS-GUIDED CHOLEDOCHODUODENOSTOMY; PANCREATIC FLUID COLLECTIONS;
GALLBLADDER DRAINAGE; PLASTIC STENTS; RISK-FACTORS; PALLIATION;
PLACEMENT; CATHETER; OUTCOMES; ADENOCARCINOMA
AB In the last years, endoscopic ultrasonography (EUS) has evolved from a purely diagnostic technique to a more and more complex interventional procedure, with the possibility to perform several type of therapeutic interventions. Among these, EUS-guided biliary drainage (BD) is gaining popularity as a therapeutic approach after failed endoscopic retrograde cholangiopancreatography in distal malignant biliary obstruction (MBO), due to the avoidance of external drainage, a lower rate of adverse events and re-interventions, and lower costs compared to percutaneous trans-hepatic BD. Initially, devices created for luminal procedures (e.g., luminal biliary stents) have been adapted to the new trans-luminal EUS-guided interventions, with predictable shortcomings in technical success, outcome and adverse events. More recently, new metal stents specifically designed for transluminal drainage, namely lumen-apposing metal stents (LAMS), have been made available for EUS-guided procedures. An electrocautery enhanced delivery system (EC-LAMS), which allows direct access of the delivery system to the target lumen, has subsequently simplified the classic multi-step procedure of EUS-guided drainages. EUS-BD using LAMS and EC-LAMS has been demonstrated effective and safe, and currently seems one of the most performing techniques for EUS-BD. In this Review, we summarize the evolution of the EUS-BD in distal MBO, focusing on the novelty of LAMS and analyzing the unresolved questions about the possible role of EUS as the first therapeutic option to achieve BD in this setting of patients.
C1 [Anderloni, Andrea; Fugazza, Alessandro; Cappello, Annalisa; Repici, Alessandro] Humanitas Res Hosp, Div Gastroenterol, Digest Endoscopy Unit, Via Manzoni 56, I-20089 Milan, Italy.
[Troncone, Edoardo; Blanco, Giovanna Del Vecchio; Monteleone, Giovanni] Univ Roma Tor Vergata, Dept Syst Med, I-00133 Rome, Italy.
[Repici, Alessandro] Humanitas Univ, Div Gastroenterol, Digest Endoscopy Unit, I-20089 Milan, Italy.
C3 University of Rome Tor Vergata; Humanitas University
RP Anderloni, A (corresponding author), Humanitas Res Hosp, Div Gastroenterol, Digest Endoscopy Unit, Via Manzoni 56, I-20089 Milan, Italy.
EM andrea.anderloni@humanitas.it
RI Fugazza, Alessandro/ABG-9381-2021; Monteleone, Giovanni/AAE-1418-2021;
Troncone, Edoardo/AHD-9276-2022; Anderloni, Andrea/ABE-9139-2020;
Repici, Alessandro/HFH-8162-2022
OI Fugazza, Alessandro/0000-0003-0485-4903; Cappello,
Annalisa/0000-0002-9248-9549; Troncone, Edoardo/0000-0003-4520-6865;
Anderloni, Andrea/0000-0002-1021-0031; Repici,
Alessandro/0000-0002-1621-6450
CR Adams MA, 2012, J GASTROINTEST ONCOL, V3, P309, DOI 10.3978/j.issn.2078-6891.2011.050
Anderloni A, 2018, ANN GASTROENTEROL, V31, P735, DOI 10.20524/aog.2018.0299
Anderloni A, 2019, GASTROINTEST ENDOSC, V89, P69, DOI [10.1016/j.gie.2018.04.1746, 10.1016/j.gie.2018.08.047]
Anderloni A, 2018, AM J GASTROENTEROL, V113, P644, DOI 10.1038/s41395-018-0040-9
Anderloni A, 2016, SURG ENDOSC, V30, P5200, DOI 10.1007/s00464-016-4894-x

Anderloni A, 2016, ENDOSCOPY, V48, pE321, DOI 10.1055/s-0042-117425
Artifon ELA, 2006, AM J GASTROENTEROL, V101, P2031, DOI 10.1111/j.1572-0241.2006.00764.x
Bang JY, 2018, GASTROINTEST ENDOSC, V88, P9, DOI 10.1016/j.gie.2018.03.012
Binmoeller KF, 2011, ENDOSCOPY, V43, P337, DOI 10.1055/s-0030-1256127
Bliss LA, 2016, HPB, V18, P671, DOI 10.1016/j.hpb.2016.05.008
Born P, 1998, SCAND J GASTROENTERO, V33, P544
Boulay Brian R, 2018, Gastrointest Endosc Clin N Am, V28, P171, DOI [10.1016/j.giec.2017.11.005, 10.1016/j.giec.2017.11.005]
Dollhopf M, 2017, GASTROINTEST ENDOSC, V86, P636, DOI 10.1016/j.gie.2017.02.027
Dumonceau JM, 2018, ENDOSCOPY, V50, P910, DOI 10.1055/a-0659-9864
Enochsson L, 2010, GASTROINTEST ENDOSC, V72, P1175, DOI 10.1016/j.gie.2010.07.047
Fabbri C, 2019, DIGEST LIVER DIS, V51, pE126, DOI 10.1016/S1590-8658(19)30236-1
Giovannini M, 2001, ENDOSCOPY, V33, P898, DOI 10.1055/s-2001-17324
Glessing BR, 2015, GASTROINTEST ENDOSC, V81, P1019, DOI 10.1016/j.gie.2014.09.061
GUNTHER RW, 1988, CARDIOVASC INTER RAD, V11, P65, DOI 10.1007/BF02577061
Guo JT, 2018, ENDOSC ULTRASOUND, V7, P356, DOI 10.4103/eus.eus_53_18
Huguier M, 1993, Eur J Surg Oncol, V19, P342
Imai H, 2016, GASTROINTEST ENDOSC, V84, P147, DOI 10.1016/j.gie.2015.12.024
Itoi T, 2014, GASTROINTEST ENDOSC, V79, P715, DOI 10.1016/j.gie.2013.11.021
Itoi T, 2012, GASTROINTEST ENDOSC, V75, P870, DOI 10.1016/j.gie.2011.10.020
Iwashita T, 2012, ENDOSCOPY, V44, P60, DOI 10.1055/s-0030-1256871
Jacques J, 2019, ENDOSCOPY, V51, P540, DOI 10.1055/a-0735-9137
Kneuertz PJ, 2011, J GASTROINTEST SURG, V15, P1917, DOI 10.1007/s11605-011-1665-9
Krokidis M, 2010, CARDIOVASC INTER RAD, V33, P97, DOI 10.1007/s00270-009-9604-9
Kunda R, 2016, SURG ENDOSC, V30, P5002, DOI 10.1007/s00464-016-4845-6
Law Ryan, 2018, Gastrointest Endosc Clin N Am, V28, P187, DOI [10.1016/j.giec.2017.11.006, 10.1016/j.giec.2017.11.006]
Maire F, 2006, AM J GASTROENTEROL, V101, P735, DOI 10.1111/j.1572-0241.2006.00559.x
Maple JT, 2017, GASTROINTEST ENDOSC, V85, P465, DOI 10.1016/j.gie.2016.11.021
Masci E, 2003, ENDOSCOPY, V35, P830
MOLNAR W, 1974, AM J ROENTGENOL, V122, P356, DOI 10.2214/ajr.122.2.356
MUELLER PR, 1982, AM J ROENTGENOL, V138, P17, DOI 10.2214/ajr.138.1.17
Mussetto A, 2018, ANN GASTROENTEROL, V31, P535, DOI 10.20524/aog.2018.0287
Nam K, 2018, ENDOSC ULTRASOUND, V7, P48, DOI 10.4103/eus.eus_100_17
Nennstiel S, 2019, DIGEST DIS, V37, P77, DOI 10.1159/000493120
Nennstiel S, 2015, J CLIN GASTROENTEROL, V49, P764, DOI 10.1097/MCG.0000000000000275
Paik WH, 2018, AM J GASTROENTEROL, V113, P987, DOI 10.1038/s41395-018-0122-8
Park JK, 2018, GASTROINTEST ENDOSC, V88, P277, DOI 10.1016/j.gie.2018.03.015
PEREIRAS RV, 1978, ANN INTERN MED, V89, P589, DOI 10.7326/0003-4819-89-5-589
Rinninella E, 2015, GASTROINTEST ENDOSC, V82, P1039, DOI 10.1016/j.gie.2015.04.006
ROSEMURGY AS, 1989, AM SURGEON, V55, P55
Sarwar A, 2019, RADIOLOGY, V290, P722, DOI 10.1148/radiol.2018180279

Shah RJ, 2015, CLIN GASTROENTEROL H, V13, P747, DOI 10.1016/j.cgh.2014.09.047
Sharaiha RZ, 2017, GASTROINTEST ENDOSC, V85, P904, DOI 10.1016/j.gie.2016.12.023
Sharaiha RZ, 2016, SURG ENDOSC, V30, P5500, DOI 10.1007/s00464-016-4913-y
Siddiqui AA, 2017, GASTROINTEST ENDOSC, V85, P758, DOI 10.1016/j.gie.2016.08.014
Siddiqui AA, 2016, GASTROINTEST ENDOSC, V83, P699, DOI 10.1016/j.gie.2015.10.020
SINGH SM, 1989, SURG CLIN N AM, V69, P599
Spadaccini M, 2019, AM J GASTROENTEROL, V114, P174, DOI 10.1038/s41395-018-0371-6
Stark A, 2015, SEMIN ONCOL, V42, P163, DOI 10.1053/j.seminoncol.2014.12.014
Stier Matthew W, 2018, Gastrointest Endosc Clin N Am, V28, P207, DOI [10.1016/j.giec.2017.11.008, 10.1016/j.giec.2017.11.008]
Teoh AYB, 2018, GUT, V67, P1209, DOI 10.1136/gutjnl-2017-314341
Thornton RH, 2011, AM J ROENTGENOL, V197, pW514, DOI 10.2214/AJR.10.6069
Tringali A, 2018, ENDOSCOPY, V50, P631, DOI 10.1055/s-0043-125062
Tsuchiya T, 2018, GASTROINTEST ENDOSC, V87, P1138, DOI 10.1016/j.gie.2017.08.017
Uemura RS, 2018, J CLIN GASTROENTEROL, V52, P123, DOI 10.1097/MCG.0000000000000948
van der Gaag NA, 2010, NEW ENGL J MED, V362, P129, DOI 10.1056/NEJMoa0903230
Walter D, 2016, GUT, V65, P6, DOI 10.1136/gutjnl-2015-309925
Walter D, 2015, ENDOSCOPY, V47, P63, DOI 10.1055/s-0034-1378113
Wang KX, 2016, GASTROINTEST ENDOSC, V83, P1218, DOI 10.1016/j.gie.2015.10.033
Wang P, 2009, AM J GASTROENTEROL, V104, P31, DOI 10.1038/ajg.2008.5
NR 64
TC 31
Z9 35
U1 0
U2 1
PU BAISHIDENG PUBLISHING GROUP INC
PI PLEASANTON
PA 8226 REGENCY DR, PLEASANTON, CA 94588 USA
SN 1007-9327
EI 2219-2840
J9 WORLD J GASTROENTERO
JI World J. Gastroenterol.
PD AUG 7
PY 2019
VL 25
IS 29
BP 3857
EP 3869
DI 10.3748/wjg.v25.i29.3857
PG 13
WC Gastroenterology & Hepatology
WE Science Citation Index Expanded (SCI-EXPANDED)

SC Gastroenterology & Hepatology
GA IN8XW
UT WOS:000478964100004
PM 31413524
OA Green Submitted, hybrid
DA 2026-05-09
ER

PT J
AU Kawai, A
Kikuyama, M
Enokida, K
Kawaguchi, S
Shirane, N
Terada, S
AF Kawai, Asami
Kikuyama, Masataka
Enokida, Kohei
Kawaguchi, Shinya
Shirane, Naofumi
Terada, Shuzo
TI A case of pancreatic cancer with severe vomiting treated by endoscopic
ultrasound-guided celiac ganglia neurolysis
SO CLINICAL JOURNAL OF GASTROENTEROLOGY
LA English
DT Article
DE Celiac ganglion; Ethanol; Gastrointestinal obstruction; Brain
metastasis; EUS-CGN
ID NUCLEUS-TRACTUS-SOLITARIUS; PLEXUS NEUROLYSIS; NAUSEA; EMESIS;
VISUALIZATION; TRANSMISSION; MECHANISMS; BLOCK; PAIN
AB A 50-year-old man with advanced pancreatic cancer was admitted for intractable severe vomiting 5-6 times a day, continuing over a week. He had been treated for advanced pancreatic cancer with chemotherapy for 6 months, and had undergone self-expandable metalic stent placement for obstructive jaundice due to the pancreatic cancer 4 months before admission. No abnormal findings suggesting gastrointestinal obstruction or brain metastasis were revealed on diagnostic imaging. We performed endoscopic ultrasound-guided celiac ganglia neurolysis twice by injecting ethanol into the celiac ganglion. After the treatments, the vomiting disappeared, and his eating habits gradually returned to normal. The patient died 7 months after treatment due to the advanced pancreatic cancer without recurrence of the vomiting.
C1 [Kawai, Asami; Enokida, Kohei; Kawaguchi, Shinya; Shirane, Naofumi; Terada, Shuzo] Shizuoka Prefectural Gen Hosp, Dept Gastroenterol, Shizuoka, Japan.
[Kikuyama, Masataka] Komagome Hosp, Tokyo Metropolitan Canc & Infect Dis, Dept Gastroenterol, Bunkyo Ku, 3-18-22 Honkomagome, Tokyo 1138677, Japan.
C3 Shizuoka General Hospital; Tokyo Metropolitan Cancer & Infectious

Diseases Center Komagome Hospital
RP Kikuyama, M (corresponding author), Komagome Hosp, Tokyo Metropolitan Canc & Infect Dis, Dept Gastroenterol, Bunkyo Ku, 3-18-22 Honkomagome, Tokyo 1138677, Japan.
EM kikuyama110@yahoo.co.jp
RI Kawaguchi, Shinya/ORK-1948-2025
CR ANDRESEN MC, 1990, AM J PHYSIOL, V259, pH1307, DOI 10.1152/ajpheart.1990.259.4.H1307
ANDRESEN MC, 1994, ANNU REV PHYSIOL, V56, P93, DOI 10.1146/annurev.physiol.56.1.93
Andrews PLR, 2006, AUTON NEUROSCI-BASIC, V125, P100, DOI 10.1016/j.autneu.2006.01.008
ANDREWS PLR, 1990, CAN J PHYSIOL PHARM, V68, P325, DOI 10.1139/y90-047
Ascunce G, 2011, GASTROINTEST ENDOSC, V73, P267, DOI 10.1016/j.gie.2010.10.029
Babic T, 2014, EUR J PHARMACOL, V722, P38, DOI 10.1016/j.ejphar.2013.08.047
Baptista V, 2005, BRAIN RES, V1052, P139, DOI 10.1016/j.brainres.2005.05.073
Blackshaw LA, 2007, NEUROGASTROENT MOTIL, V19, P1, DOI 10.1111/j.1365-2982.2006.00871.x
Darmani NA, 2004, EUR J PHARMACOL, V488, P201, DOI 10.1016/j.ejphar.2004.02.018
Doi S, 2013, ENDOSCOPY, V45, P362, DOI 10.1055/s-0032-1326225
Endo T, 2000, TOXICOLOGY, V153, P189, DOI 10.1016/S0300-483X(00)00314-0
Faigel DO, 1996, AM J GASTROENTEROL, V91, P1675
Gleeson FC, 2007, ENDOSCOPY, V39, P620, DOI 10.1055/s-2007-966337
Gunaratnam NT, 2001, GASTROINTEST ENDOSC, V54, P316, DOI 10.1067/mge.2001.117515
Ha Tae-In, 2008, Korean Journal of Internal Medicine, V23, P5, DOI 10.3904/kjim.2008.23.1.5
Hasler WL, 2017, AUTON NEUROSCI-BASIC, V202, P28, DOI 10.1016/j.autneu.2016.08.001
Horn CC, 2008, APPETITE, V50, P430, DOI 10.1016/j.appet.2007.09.015
Kappis M, 1914, Verh. Dtsch. Ges. Chir, V43, P87
LANG IM, 1986, GASTROENTEROLOGY, V90, P40, DOI 10.1016/0016-5085(86)90072-7
LANG IM, 1993, AM J PHYSIOL, V265, pG963, DOI 10.1152/ajpgi.1993.265.5.G963
Levy MJ, 2008, AM J GASTROENTEROL, V103, P98, DOI 10.1111/j.1572-0241.2007.01607.x
MILLER AD, 1990, J APPL PHYSIOL, V69, P21, DOI 10.1152/jappl.1990.69.1.21
Puli SR, 2009, DIGEST DIS SCI, V54, P2330, DOI 10.1007/s10620-008-0651-x
Sahai AV, 2009, AM J GASTROENTEROL, V104, P326, DOI 10.1038/ajg.2008.64
Travagli RA, 2006, ANNU REV PHYSIOL, V68, P279, DOI 10.1146/annurev.physiol.68.040504.094635
WANG SC, 1951, AM J PHYSIOL, V164, P520, DOI 10.1152/ajplegacy.1951.164.2.520
WARD EM, 1979, ANESTH ANALG, V58, P461
Wiersema MJ, 1996, GASTROINTEST ENDOSC, V44, P656, DOI 10.1016/S0016-5107(96)70047-0
Wood GJ, 2007, JAMA-J AM MED ASSOC, V298, P1196, DOI 10.1001/jama.298.10.1196
NR 29
TC 0
Z9 0
U1 1
U2 2
PU SPRINGER JAPAN KK
PI TOKYO
PA CHIYODA FIRST BLDG EAST, 3-8-1 NISHI-KANDA, CHIYODA-KU, TOKYO, 101-0065,

JAPAN
SN 1865-7257
EI 1865-7265
J9 CLIN J GASTROENTEROL
JI Clin. J. Gastroenterol.
PD OCT
PY 2017
VL 10
IS 5
BP 464
EP 468
DI 10.1007/s12328-017-0761-0
PG 5
WC Gastroenterology & Hepatology
WE Emerging Sources Citation Index (ESCI)
SC Gastroenterology & Hepatology
GA FK3DG
UT WOS:000413364600012
PM 28815450
DA 2026-05-09
ER

PT J
AU Itonaga, M
Kitano, M
Ashida, R
AF Itonaga, Masahiro
Kitano, Masayuki
Ashida, Reiko
TI Development of devices for interventional endoscopic ultrasound for the
management of pancreatobiliary diseases
SO DIGESTIVE ENDOSCOPY
LA English
DT Review
DE endoscopic device; EUS-guided biliary drainage (EUS-BD); interventional
EUS; lumen-apposing metal stent (LAMS); self-expandable metal stent
(SEMS)
ID GUIDED BILIARY DRAINAGE; APPOSING METAL STENT; THIN DELIVERY-SYSTEM;
LONG-TERM OUTCOMES; GALLBLADDER DRAINAGE; FLUID COLLECTIONS;
ELECTROCAUTERY DILATOR; PHOTODYNAMIC THERAPY; HEPATICOGASTROSTOMY;
OBSTRUCTION
AB A variety of devices have been developed for interventional endoscopic ultrasound (EUS). EUS-guided drainage of the

bile duct and pancreatic duct, as well as fluid collection adjacent to the gastrointestinal tract, is performed by a procedure involving puncture, guidewire manipulation, tract dilation, and stent placement. Devices specialized for interventional EUS are being developed for each step of the procedure. Mechanical dilators such as bougie, balloon, and electrocautery dilators are used for tract dilation. Various types of plastic stents, self-expandable metal stents, and lumen-apposing metal stents specifically designed for interventional EUS are now available, including one-step devices developed to improve the efficacy and safety of interventional EUS. In addition, radiofrequency ablation and the placement of fiducial markers and radioactive seeds under EUS guidance are now becoming established for pancreatic neoplasms. Such development of specialized devices has expanded the indications for interventional EUS, increased the success rate, and lowered the adverse event rate.
C1 [Itonaga, Masahiro; Kitano, Masayuki; Ashida, Reiko] Wakayama Med Univ, Dept Internal Med 2, 811-1 Kimiidera, Wakayama, Wakayama 6410012, Japan.
C3 Wakayama Medical University
RP Kitano, M (corresponding author), Wakayama Med Univ, Dept Internal Med 2, 811-1 Kimiidera, Wakayama, Wakayama 6410012, Japan.
EM kitano@wakayama-med.ac.jp
RI Itonaga, Masahiro/AAK-6905-2021
CR Arvanitakis M, 2018, ENDOSCOPY, V50, P524, DOI 10.1055/a-0588-5365
ASGE Technology Committee, 2017, VideoGIE, V2, P35, DOI [10.1016/j.vgie.2016.12.001, 10.1016/j.vgie.2016.12.001]
Ashida R, 2020, WORLD J GASTRO ONCOL, V12, P768, DOI 10.4251/wjgo.v12.i7.768
Bang JY, 2016, DIGEST ENDOSC, V28, P103, DOI 10.1111/den.12557
Bang JY, 2015, DIGEST ENDOSC, V27, P486, DOI 10.1111/den.12418
Bhurwal A, 2021, PANCREATOLOGY, V21, P990, DOI 10.1016/j.pan.2021.03.021
Bhutani MS, 2020, ENDOSC ULTRASOUND, V9, P24, DOI 10.4103/eus.eus_44_19
Binmoeller KF, 2011, ENDOSCOPY, V43, P337, DOI 10.1055/s-0030-1256127
Bronswijk M, 2021, GASTROINTEST ENDOSC, V94, P526, DOI 10.1016/j.gie.2021.04.006
Bronswijk Michiel, 2020, VideoGIE, V5, P442, DOI [10.1016/j.vgie.2020.06.012, 10.1016/j.vgie.2020.06.012]
Cazacu Irina M, 2020, Curr Gastroenterol Rep, V22, P27, DOI [10.1007/s11894-020-00767-1, 10.1007/s11894-020-00767-1]
Chin JYL, 2020, ENDOSC INT OPEN, V08, pE1633, DOI 10.1055/a-1236-3217
Choi JH, 2015, ENDOSCOPY, V47, P1035, DOI 10.1055/s-0034-1392150
Coronel Emmanuel, 2019, VideoGIE, V4, P403, DOI [10.1016/j.vgie.2019.05.007, 10.1016/j.vgie.2019.05.007]
Coronel E, 2019, GASTROINTEST ENDOSC, V89, P659, DOI 10.1016/j.gie.2018.10.047
Sanz MD, 2021, SURG ENDOSC, V35, P6754, DOI 10.1007/s00464-020-08179-y
De Cassan C, 2017, ENDOSC ULTRASOUND, V6, P329, DOI 10.4103/2303-9027.209869
Dhir V, 2017, DIGEST ENDOSC, V29, P472, DOI 10.1111/den.12818
Hanada Y, 2021, GASTROINTEST ENDOSC, V94, P179, DOI 10.1016/j.gie.2021.02.027
Honjo M, 2018, ENDOSC ULTRASOUND, V7, P376, DOI 10.4103/eus.eus_2_18
Huh G, 2020, SCI REP-UK, V10, DOI 10.1038/s41598-020-67374-y
Ichkhanian Yervant, 2020, VideoGIE, V5, P260, DOI [10.1016/j.vgie.2020.02.005, 10.1016/j.vgie.2020.02.005]
Irani S, 2015, GASTROINTEST ENDOSC, V82, P1110, DOI 10.1016/j.gie.2015.05.045
Isayama H, 2019, J HEPATO-BIL-PAN SCI, V26, P249, DOI 10.1002/jhbp.631

Ishiwatari H, 2022, J HEPATO-BIL-PAN SCI, V29, P703, DOI 10.1002/jhbp.1118
Itoi T, 2017, DIGEST ENDOSC, V29, P495, DOI 10.1111/den.12794
Itoi T, 2016, GUT, V65, P193, DOI 10.1136/gutjnl-2015-310348
Itoi T, 2010, GASTROINTEST ENDOSC, V71, P1038, DOI 10.1016/j.gie.2010.01.026
Itonaga M., 2021, ENDOSC ULTRASOUND, DOI [10.4103/EUS-D-21-00073, DOI 10.4103/EUS]
Itonaga M, 2019, DIGEST ENDOSC, V31, P291, DOI 10.1111/den.13300
Iwashita T, 2022, J HEPATO-BIL-PAN SCI, V29, P810, DOI 10.1002/jhbp.1021
Jang JW, 2012, GASTROENTEROLOGY, V142, P805, DOI 10.1053/j.gastro.2011.12.051
Kahaleh M, 2016, DIGEST LIVER DIS, V48, P1054, DOI 10.1016/j.dld.2016.05.021
Kanno Yoshihide, 2020, VideoGIE, V5, P355, DOI [10.1016/j.vgie.2020.04.003, 10.1016/j.vgie.2020.04.003]
Kawakami H, 2017, ENDOSCOPY, V49, pE264, DOI 10.1055/s-0043-116014
Khashab MA, 2016, ENDOSC INT OPEN, V4, pE175, DOI 10.1055/s-0041-109083
Khashab MA, 2015, GASTROINTEST ENDOSC, V82, P932, DOI 10.1016/j.gie.2015.06.017
Khashab MA, 2015, DIGEST DIS SCI, V60, P557, DOI 10.1007/s10620-014-3300-6
Kim SH, 2021, CLIN ENDOSC, V54, P314, DOI 10.5946/ce.2021.102
Koga T, 2022, DEN OPEN, V2, DOI 10.1002/deo2.56
Kozakai F, 2022, DIGEST ENDOSC, V34, pE66, DOI 10.1111/den.14258
Kuraoka Naosuke, 2020, Endosc Int Open, V8, pE861, DOI [10.1055/a-1161-8488, 10.1055/a-1161-8488]
Lakhtakia S, 2017, DIGEST ENDOSC, V29, P486, DOI 10.1111/den.12833
Lee TH, 2014, WORLD J GASTROENTERO, V20, P5859, DOI 10.3748/wjg.v20.i19.5859
Mangiavillano B, 2022, GASTROINTEST ENDOSC, V95, P115, DOI 10.1016/j.gie.2021.07.021
Mangiavillano B, 2016, WORLD J GASTROINTEST, V8, P143, DOI 10.4253/wjge.v8.i3.143
Matsubara S, 2021, DIGEST ENDOSC, V33, pE119, DOI 10.1111/den.14012
Matsumoto S, 2020, DIGEST ENDOSC, V32, P957, DOI 10.1111/den.13620
Matsunami Y, 2018, ENDOSC INT OPEN, V6, pE505, DOI 10.1055/s-0044-101753
McCarty TR, 2022, DIGEST DIS SCI, V67, P1660, DOI 10.1007/s10620-022-07426-7
Minaga K, 2018, J MED ULTRASON, V45, P391, DOI 10.1007/s10396-017-0850-1
Minaga K, 2018, DIGEST ENDOSC, V30, P38, DOI 10.1111/den.12910
Mukai S, 2022, J HEPATO-BIL-PAN SCI, V29, P817, DOI 10.1002/jhbp.1113
Mukai S, 2017, GASTROINTEST ENDOSC, V86, P203, DOI [10.1016/j.gie.2016.07.056, 10.1016/j.gie.2016.11.025, 10.1016/j.gie.2016.07.056]
Mussetto A, 2018, ANN GASTROENTEROL, V31, P535, DOI 10.20524/aog.2018.0287
Naidu J, 2022, ENDOSCOPY, V54, P75, DOI 10.1055/a-1353-0941
Nakai Y, 2020, GASTROINTEST ENDOSC, V92, P623, DOI 10.1016/j.gie.2020.03.3856
Nakai Y, 2021, DIGEST DIS SCI, V66, P1540, DOI 10.1007/s10620-020-06345-9
Nakai Y, 2017, ENDOSCOPY, V49, P983, DOI 10.1055/s-0043-113444
Ogura T, 2021, DIGEST ENDOSC, V33, P1188, DOI 10.1111/den.14077
Ogura T, 2022, J HEPATO-BIL-PAN SCI, V29, pE88, DOI 10.1002/jhbp.904
Ogura T, 2020, ENDOSC ULTRASOUND, V9, P267, DOI 10.4103/eus.eus_38_20
Ogura T, 2019, ENDOSC INT OPEN, V7, pE1652, DOI 10.1055/a-0961-7890
Ogura T, 2018, DIGEST ENDOSC, V30, P252, DOI 10.1111/den.12976
Ogura T, 2015, J GASTROEN HEPATOL, V30, P1748, DOI 10.1111/jgh.13021

Oh D, 2020, J GASTROEN HEPATOL, V35, P994, DOI 10.1111/jgh.14897
Oh D, 2016, GASTROINTEST ENDOSC, V83, P366, DOI 10.1016/j.gie.2015.07.015
Okuzono T, 2022, J HEPATO-BIL-PAN SCI, V29, P825, DOI 10.1002/jhbp.1139
Paik WH, 2018, AM J GASTROENTEROL, V113, P987, DOI 10.1038/s41395-018-0122-8
Ryou M, 2020, GASTROINTEST ENDOSC, V91, P178, DOI 10.1016/j.gie.2019.07.035
Song TJ, 2019, GASTROINTEST ENDOSC, V90, P507, DOI 10.1016/j.gie.2019.05.033
Song TJ, 2014, GASTROINTEST ENDOSC, V80, P707, DOI 10.1016/j.gie.2014.05.327
Takagi W, 2016, THER ADV GASTROENTER, V9, P19, DOI 10.1177/1756283X15609285
Teoh AYB, 2021, DIGEST ENDOSC, V33, P1139, DOI 10.1111/den.13911
Tsuchiya T, 2018, GASTROINTEST ENDOSC, V87, P1138, DOI 10.1016/j.gie.2017.08.017
Umeda J, 2015, GASTROINTEST ENDOSC, V82, P390, DOI 10.1016/j.gie.2015.02.041
van Wanrooij RLJ, 2022, ENDOSCOPY, V54, P310, DOI 10.1055/a-1738-6780
Varadarajulu S, 2008, GASTROINTEST ENDOSC, V68, P656, DOI 10.1016/j.gie.2008.03.1091
Wang JL, 2021, ANN PALLIAT MED, V10, P1842, DOI 10.21037/apm-20-1468
Yamamoto N, 2013, GASTROINTEST ENDOSC, V77, P809, DOI 10.1016/j.gie.2013.01.009
Yamamura M, 2022, ENDOSC INT OPEN, V10, pE209, DOI 10.1055/a-1729-0048
Yamashita Y, 2021, GASTROINTEST ENDOSC, V94, P999, DOI 10.1016/j.gie.2021.06.014
Zhang K, 2018, GASTROINTEST ENDOSC, V88, P957, DOI 10.1016/j.gie.2018.07.019
Zhou XZ, 2021, J CLIN GASTROENTEROL, V55, P652, DOI 10.1097/MCG.0000000000001539
NR 84
TC 17
Z9 18
U1 0
U2 4
PU WILEY
PI HOBOKEN
PA 111 RIVER ST, HOBOKEN 07030-5774, NJ USA
SN 0915-5635
EI 1443-1661
J9 DIGEST ENDOSC
JI Dig. Endosc.
PD MAR
PY 2023
VL 35
IS 3
BP 302
EP 313
DI 10.1111/den.14428
EA OCT 2022
PG 12
WC Gastroenterology & Hepatology; Surgery
WE Science Citation Index Expanded (SCI-EXPANDED)

SC Gastroenterology & Hepatology; Surgery
GA D9BR8
UT WOS:000865786200001
PM 36052861
OA Bronze
DA 2026-05-09
ER

PT J
AU Law, R
Baron, TH
AF Law, Ryan
Baron, Todd H.
TI Endoscopic ultrasound-guided biliary interventions: an update on recent developments
SO CURRENT OPINION IN GASTROENTEROLOGY
LA English
DT Review
DE biliary rendezvous; endoscopic ultrasound-guided biliary drainage; internal biliary drainage; lumen-apposing metal stent
ID APPOSING METAL STENT; GALLBLADDER DRAINAGE; FAILED ERCP; OBSTRUCTION; CHOLEDOCHODUODENOSTOMY
AB Purpose of reviewEndoscopic ultrasound (EUS)-guided biliary drainage procedures can be utilized in patients with biliary obstruction and incomplete drainage with prior interventions, in those whose ampulla is inaccessible (i.e. from surgically altered anatomy and malignant luminal obstruction), or in those who failed bile duct cannulation during endoscopic retrograde cholangiopancreatography. Improved techniques and accessories have streamlined these procedures; however, further investigation is required. The purpose of this review is to provide the highlights of the most recent literature on EUS-guided biliary drainage.Recent findingsRecent literature has demonstrated the effectiveness and safety of EUS-guided gallbladder drainage, both as a primary intervention in patients with cholecystitis who are unfit for urgent surgical intervention and as a secondary intervention to internalize biliary drainage after initial placement of a cholecystostomy catheter. EUS-guided bile duct drainage can be performed using various techniques. The optimal technique requires further study and may vary by clinical scenario.SummaryThe role of EUS-guided biliary drainage continues to evolve. The most recent literature has attempted to clarify the optimal strategies and indications for employing the various EUS-guided biliary drainage techniques.
C1 [Law, Ryan] Univ Michigan, Div Gastroenterol, Ann Arbor, MI 48109 USA.
[Baron, Todd H.] Univ N Carolina, Div Gastroenterol & Hepatol, 41041 Bioinformat Blvd,CB 7080, Chapel Hill, NC 27599 USA.
C3 University of Michigan System; University of Michigan; University of North Carolina; University of North Carolina Chapel Hill
RP Baron, TH (corresponding author), Univ N Carolina, Div Gastroenterol & Hepatol, 41041 Bioinformat Blvd,CB 7080, Chapel Hill, NC 27599 USA.
EM todd_baron@med.unc.edu

CR Artifon ELA, 2015, ENDOSC ULTRASOUND, V4, P235, DOI 10.4103/2303-9027.163010
Artifon ELA, 2015, GASTROINTEST ENDOSC, V81, P950, DOI 10.1016/j.gie.2014.09.047
Baron TH, 2015, NEW ENGL J MED, V373, P357, DOI 10.1056/NEJMra1411372
Brückner S, 2015, ENDOSCOPY, V47, P858, DOI 10.1055/s-0034-1392104
de la Serna-Higuera C, 2013, GASTROINTEST ENDOSC, V77, P303, DOI 10.1016/j.gie.2012.09.021
Dhir V, 2015, GASTROINTEST ENDOSC, V81, P913, DOI 10.1016/j.gie.2014.09.054
Irani S, 2015, GASTROINTEST ENDOSC, V82, P1110, DOI 10.1016/j.gie.2015.05.045
Itoi T, 2010, GASTROINTEST ENDOSC, V71, P1038, DOI 10.1016/j.gie.2010.01.026
Khan MA, 2015, DIG DIS SCI
Khashab MA, 2015, DIGEST DIS SCI, V60, P557, DOI 10.1007/s10620-014-3300-6
Law R, 2015, CLIN GASTROENTEROL H
Moon JH, 2014, GASTROINTEST ENDOSC, V79, P990, DOI 10.1016/j.gie.2014.02.015
Ogura T, 2015, ENDOSCOPY
Peñas-Herrero I, 2015, J HEPATO-BIL-PAN SCI, V22, P35, DOI 10.1002/jhbp.182
Poincloux L, 2015, ENDOSCOPY, V47, P794, DOI [10.1055/s-0035-1545399, 10.1055/s-0034-1391988]
Walter D, 2016, GUT, V65, P6, DOI 10.1136/gutjnl-2015-309925
Wang K, 2015, GASTROINTEST ENDOSC
NR 17
TC 22
Z9 22
U1 0
U2 1
PU LIPPINCOTT WILLIAMS & WILKINS
PI PHILADELPHIA
PA TWO COMMERCE SQ, 2001 MARKET ST, PHILADELPHIA, PA 19103 USA
SN 0267-1379
EI 1531-7056
J9 CURR OPIN GASTROEN
JI Curr. Opin. Gastroenterol.
PD MAY
PY 2016
VL 32
IS 3
BP 232
EP 237
DI 10.1097/MOG.0000000000000255
PG 6
WC Gastroenterology & Hepatology
WE Science Citation Index Expanded (SCI-EXPANDED)
SC Gastroenterology & Hepatology
GA DK2TQ
UT WOS:000374767700014

PM 26959514
DA 2026-05-09
ER

PT J
AU Paduano, D
Facciorusso, A
De Marco, A
Ofosu, A
Auriemma, F
Calabrese, F
Tarantino, I
Franchellucci, G
Lisotti, A
Fusaroli, P
Repici, A
Mangiavillano, B
AF Paduano, Danilo
Facciorusso, Antonio
De Marco, Alessandro
Ofosu, Andrew
Auriemma, Francesco
Calabrese, Federica
Tarantino, Ilaria
Franchellucci, Gianluca
Lisotti, Andrea
Fusaroli, Pietro
Repici, Alessandro
Mangiavillano, Benedetto
TI Endoscopic Ultrasound Guided Biliary Drainage in Malignant Distal
Biliary Obstruction
SO CANCERS
LA English
DT Review
DE MBO; EUS; LAMS; EUS-BD; cancer; stent
ID APPOSING METAL STENT; INTERVENTIONAL EUS PROCEDURES; PANCREATIC FLUID
COLLECTION; GALLBLADDER DRAINAGE; MANAGEMENT; EFFICACY; SAFETY; ERCP;
MULTICENTER; EXPERIENCE
AB Simple Summary EUS-guided biliary drainage (EUS-BD) has proven effective in the palliation of malignant biliary obstructions. There are several methods for obtaining EUS-guided biliary drainage when endoscopic retrograde cholangiopancreatography (ERCP) fails. To date, EUS-BD, along with the well-established role of percutaneous transhepatic biliary drainage (PTBD) and surgical hepaticojejunostomy, has been demonstrated as a viable palliative

treatment option in these patients. There are no specific guidelines with regard to the optimal drainage method. In this review, we compare all these techniques, demonstrating that EUS-BD in expert hands represents a minimally invasive and effective method in the palliative treatment of patients with malignant biliary obstruction. Malignant biliary obstruction (MBO) is a challenging medical problem that often negatively impacts the patient's quality of life (QoL), postoperative complications, and survival rates. Endoscopic approaches to biliary drainage are generally performed by ERCP or, in selected cases, with a percutaneous transhepatic biliary drainage (PTBD). Recent advances in therapeutic endoscopic ultrasound (EUS) allow drainage where previous methods have failed. EUS has evolved from a purely diagnostic technique to one that allows a therapeutic approach in the event of ERCP failure in distal MBO. Moreover, the introduction of dedicated accessories and prostheses for EUS-guided transmural biliary drainage (EUS-BD) made these procedures more successful with regard to technical success, clinical outcomes and reduction of adverse events (AEs). Finally, lumen-apposing metal stents (LAMS) have improved the therapeutic role of the EUS. Subsequently, the electrocautery enhanced tip of the LAMS (EC-LAMS) allows a direct access of the delivery system to the target lumen, thereby simplifying and reducing the EUS-BD procedure time. EUS-BD using LAMS and EC-LAMS has proven effective and safe with a low rate of AEs. This review aims to evaluate biliary drainage techniques in malignant obstruction, focusing on the role of EUS biliary drainage by LAMS.
C1 [Paduano, Danilo; Auriemma, Francesco; Calabrese, Federica; Mangiavillano, Benedetto] Humanitas Mater Domini, Gastrointestinal Endoscopy Unit, I-21053 Castellanza, Italy.

[Facciorusso, Antonio] Univ Foggia, Dept Surg & Med Sci, Gastroenterol Unit, I-71122 Foggia, Italy.

[Facciorusso, Antonio] Univ Hosp Verona, Pancreas Inst, Dept Med, Gastroenterol & Digest Endoscopy Unit, I-37100 Verona, Italy.

[De Marco, Alessandro; Franchellucci, Gianluca; Repici, Alessandro] IRCCS, Endoscopy Unit, Humanitas Clin & Res Ctr, I-20089 Rozzano, Italy.

[Ofosu, Andrew] Univ Cincinnati, Div Gastroenterol & Hepatol, Cincinnati, OH 45201 USA.

[Tarantino, Ilaria] IRCCS ISMETT, Dept Diagnost & Therapeut Serv, Endoscopy Serv, I-90100 Palermo, Italy.

[Lisotti, Andrea; Fusaroli, Pietro] Univ Bologna, Hosp Imola, Gastroenterol Unit, I-40121 Bologna, Italy.

[Repici, Alessandro; Mangiavillano, Benedetto] Humanitas Univ, Dept Biomed Sci, I-20089 Rozzano, Italy.

C3 University of Foggia; University of Verona; Azienda Ospedaliera
Universitaria Integrata Verona; IRCCS Humanitas Research Hospital;
University System of Ohio; University of Cincinnati; IRCCS Istituto
Mediterraneo per i Trapianti e Terapie ad Alta Specializzazione
(ISMETT); University of Bologna; Azienda USL di Imola; IRCCS Humanitas
Research Hospital; Humanitas University

RP Paduano, D; Mangiavillano, B (corresponding author), Humanitas Mater Domini, Gastrointestinal Endoscopy Unit, I-21053 Castellanza, Italy.; Mangiavillano, B (corresponding author), Humanitas Univ, Dept Biomed Sci, I-20089 Rozzano, Italy.

EM danilo.paduano@libero.it; bennymangiavillano@gmail.com

RI Facciorusso, Antonio/AAA-5966-2019; Lisotti, Andrea/L-6762-2019; Repici,
Alessandro/HFH-8162-2022; TARANTINO, ILARIA/AFS-0726-2022; Fusaroli,
Pietro/AAC-2182-2020

OI Lisotti, Andrea/0000-0002-7724-7402; Repici,
Alessandro/0000-0002-1621-6450; TARANTINO, ILARIA/0000-0001-8791-3395;
Franchellucci, Gianluca/0000-0002-3440-4981; PADUANO,

DANILO/0000-0003-2732-0840
CR Aadam AA, 2019, J SURG ONCOL, V120, P57, DOI 10.1002/jso.25483
Adler DG, 2005, GASTROINTEST ENDOSC, V62, P1, DOI 10.1016/j.gie.2005.04.015
Anderloni A, 2019, GASTROINTEST ENDOSC, V89, P69, DOI [10.1016/j.gie.2018.04.1746, 10.1016/j.gie.2018.08.047]
Anderloni A, 2017, ENDOSC INT OPEN, V5, pE25, DOI 10.1055/s-0042-122009
Anderloni A, 2016, SURG ENDOSC, V30, P5200, DOI 10.1007/s00464-016-4894-x
Bang JY, 2019, DIGEST ENDOSC, V31, P619, DOI 10.1111/den.13428
Bang JY, 2017, GUT, V66, P2054, DOI 10.1136/gutjnl-2016-312812
Bhenswala P, 2021, J CLIN GASTROENTEROL, V55, P641, DOI 10.1097/MCG.0000000000001566
Bories E, 2007, ENDOSCOPY, V39, P287, DOI 10.1055/s-2007-966212
Boulay BR, 2016, WORLD J GASTRO ONCOL, V8, P498, DOI 10.4251/wjgo.v8.i6.498
Chandran S, 2015, GASTROINTEST ENDOSC, V81, P127, DOI 10.1016/j.gie.2014.06.025
Pu LZCT, 2016, GASTROENT RES PRACT, V2016, DOI 10.1155/2016/3296801
Conio M, 2018, GASTROINTEST ENDOSC, V88, P283, DOI 10.1016/j.gie.2018.03.029
De Bie C, 2022, SURG ENDOSC, V36, P2393, DOI 10.1007/s00464-021-08520-z
DeSimone ML, 2017, WORLD J GASTRO ENDOS, V9, P456, DOI 10.4253/wjge.v9.i9.456
Dhir V, 2017, DIGEST ENDOSC, V29, P472, DOI 10.1111/den.12818
Di Mitri R, 2022, SURG ENDOSC, V36, P4553, DOI 10.1007/s00464-021-08808-0
Dumonceau JM, 2018, ENDOSCOPY, V50, P910, DOI 10.1055/a-0659-9864
El Chafic AH, 2019, DIGEST DIS SCI, V64, P3321, DOI 10.1007/s10620-019-05688-2
Fabbri C, 2015, GASTROINTEST ENDOSC, V82, P585, DOI 10.1016/j.gie.2015.04.035
Facciorusso A, 2022, CANCERS, V14, DOI 10.3390/cancers14133291
Fung Brian M, 2021, Eur Med J Hepatol, V9, P73, DOI 10.33590/emjhepatol/21-00005
Gajjar B, 2020, J INVEST MED HIGH IM, V8, DOI 10.1177/2324709620965800
Giovannini M, 2001, ENDOSCOPY, V33, P898, DOI 10.1055/s-2001-17324
Han SY, 2019, SCI REP-UK, V9, DOI 10.1038/s41598-019-52993-x
Inamdar S, 2016, JAMA ONCOL, V2, P112, DOI 10.1001/jamaoncol.2015.3670
Jagielski M, 2021, BMC GASTROENTEROL, V21, DOI 10.1186/s12876-021-01798-2
Jain D, 2018, DIGESTION, V98, P1, DOI 10.1159/000487185
James TW, 2019, ENDOSC ULTRASOUND, V8, pS28, DOI 10.4103/eus.eus_41_19
Garcia-Alonso FJ, 2018, ENDOSCOPY, V50, P386, DOI 10.1055/a-0581-9040
Jo SJ, 2023, J HEPATO-BIL-PAN SCI, V30, P263, DOI 10.1002/jhbp.1180
Khashab MA, 2015, GASTROINTEST ENDOSC, V82, P932, DOI 10.1016/j.gie.2015.06.017
Khoo S, 2020, ENDOSC ULTRASOUND, V9, P369, DOI 10.4103/eus.eus_59_20
Kunda R, 2016, SURG ENDOSC, V30, P5002, DOI 10.1007/s00464-016-4845-6
Lee TH, 2016, CLIN GASTROENTEROL H, V14, P1011, DOI 10.1016/j.cgh.2015.12.032
Leng Jian-Jun, 2014, World J Surg Oncol, V12, P272
Mangiavillano B, 2022, GASTROINTEST ENDOSC, V95, P115, DOI 10.1016/j.gie.2021.07.021
Mangiavillano B, 2022, ENDOSCOPY, V54, pE421, DOI 10.1055/a-1583-8947
Mangiavillano B, 2020, ENDOSC INT OPEN, V08, pE1315, DOI 10.1055/a-1221-4578
Mangiavillano B, 2020, EUR J GASTROEN HEPAT, V32, P1279, DOI 10.1097/MEG.0000000000001752

Moole H, 2017, MEDICINE, V96, DOI 10.1097/MD.0000000000005154
Nabi Z, 2018, GASTROINTEST ENDOSC, V88, P18, DOI 10.1016/j.gie.2018.04.2355
NAPOLEON B, 2022, ENDOSC ULTRASOUND, V11, P487, DOI DOI 10.4103/EUS-D-22-00014
Nennstiel S, 2015, J CLIN GASTROENTEROL, V49, P764, DOI 10.1097/MCG.0000000000000275
Ogura T, 2021, DEN OPEN, V1, DOI 10.1002/deo2.8
Ogura T, 2019, DIGEST ENDOSC, V31, P55, DOI 10.1111/den.13334
Ogura T, 2015, WORLD J GASTROENTERO, V21, P820, DOI 10.3748/wjg.v21.i3.820
Paduano D, 2022, ENDOSCOPY, V54, pE918, DOI 10.1055/a-1866-3459
Paik WH, 2019, ENDOSC ULTRASOUND, V8, pS44, DOI 10.4103/eus.eus_51_19
Paik WH, 2018, AM J GASTROENTEROL, V113, P987, DOI 10.1038/s41395-018-0122-8
Rana SS, 2021, ANN GASTROENTEROL, V34, P142, DOI 10.20524/aog.2020.0568
Rinninella E, 2015, GASTROINTEST ENDOSC, V82, P1039, DOI 10.1016/j.gie.2015.04.006
Salerno R, 2019, WORLD J GASTRO ENDOS, V11, P354, DOI 10.4253/wjge.v11.i5.354
Sharaiha RZ, 2017, GASTROINTEST ENDOSC, V85, P904, DOI 10.1016/j.gie.2016.12.023
Siddiqui AA, 2016, GASTROINTEST ENDOSC, V83, P699, DOI 10.1016/j.gie.2015.10.020
SMITH AC, 1994, LANCET, V344, P1655, DOI 10.1016/S0140-6736(94)90455-3
Stier Matthew W, 2018, Gastrointest Endosc Clin N Am, V28, P207, DOI [10.1016/j.giec.2017.11.008, 10.1016/j.giec.2017.11.008]
Téllez-Avila FI, 2018, SURG LAPARO ENDO PER, V28, P183, DOI 10.1097/SLE.0000000000000528
Teoh AYB, 2018, GUT, V67, P1209, DOI 10.1136/gutjnl-2017-314341
Teoh AYB, 2014, GASTROINTEST ENDOSC, V80, P1171, DOI 10.1016/j.gie.2014.03.038
Tharian B, 2016, GASTROINTEST ENDOSC, V83, P460, DOI 10.1016/j.gie.2015.08.016
Tsuchiya T, 2018, GASTROINTEST ENDOSC, V87, P1138, DOI 10.1016/j.gie.2017.08.017
Tyberg A, 2016, ENDOSC INT OPEN, V4, pE276, DOI 10.1055/s-0042-101789
van der Merwe SW, 2022, ENDOSCOPY, V54, P185, DOI 10.1055/a-1717-1391
van Wanrooij RLJ, 2022, ENDOSCOPY, V54, P310, DOI 10.1055/a-1738-6780
Walter D, 2016, GUT, V65, P6, DOI 10.1136/gutjnl-2015-309925
Yang D, 2018, ENDOSC INT OPEN, V6, pE1267, DOI 10.1055/a-0732-502
Yi H, 2022, FRONT MED-LAUSANNE, V9, DOI 10.3389/fmed.2022.1002031
NR 68
TC 16
Z9 21
U1 1
U2 7
PU MDPI
PI BASEL
PA ST ALBAN-ANLAGE 66, CH-4052 BASEL, SWITZERLAND
EI 2072-6694
J9 CANCERS
JI Cancers
PD JAN
PY 2023

VL 15
IS 2
AR 490
DI 10.3390/cancers15020490
PG 14
WC Oncology
WE Science Citation Index Expanded (SCI-EXPANDED)
SC Oncology
GA 7Y2BY
UT WOS:000914692700001
PM 36672438
OA Green Submitted, gold
DA 2026-05-09
ER

PT J
AU Kumbhari, V
Peñas, I
Tieu, AH
De la Serna-Higuera, C
Juneja, M
Maufa, F
Ngamruengphong, S
El-Zein, M
Haddad, N
Krishnan, S
Gonzalez, S
Renny, P
Saxena, P
Howard, L
DiMaio, C
Buscaglia, J
Perez-Miranda, M
Khashab, MA
AF Kumbhari, Vivek
Penas, Irene
Tieu, Alan H.
De la Serna-Higuera, Carlos
Juneja, Manie
Maufa, Fuad
Ngamruengphong, Saowanee
El-Zein, Mohamad H.

Haddad, Nadim
Krishnan, Sandeep
Gonzalez, Susana
Renny, Peter V.
Saxena, Payal
Howard, Linda
DiMaio, Christopher J.
Buscaglia, Jonathan M.
Perez-Miranda, Manuel
Khashab, Mouen A.

TI Interventional EUS Using a Flexible 19-Gauge Needle: An International Multicenter Experience in 162 Patients

SO DIGESTIVE DISEASES AND SCIENCES

LA English

DT Article

DE Flexible 19-G needle; Drainage; Endosonography; Biliary drainage; Pancreatic fluid collection; Therapeutic endoscopic ultrasound; Interventional endoscopic ultrasound; Angulated

ID GUIDED BILIARY DRAINAGE; CELIAC PLEXUS NEUROLYSIS; FAILED ERCP; ASPIRATION NEEDLES; RANDOMIZED-TRIAL; OBSTRUCTION; CHOLEDOCHODUODENOSTOMY; PERFORMANCE; 19G; FNA

AB To facilitate access for interventional EUS, flexible (nitinol) 19-G needles have been introduced to permit needle puncture even when the echoendoscope is in an angulated position, such as in the second part of the duodenum. The aims of the study were to evaluate the performance of a flexible 19-G needle during interventional EUS procedures and compare outcomes when the echoendoscope was in the straight versus angulated position.

A retrospective review was undertaken of 162 consecutive patients that underwent a variety of interventional EUS procedures with a flexible 19-G needle across five centers. Patients were subdivided into categories (straight or angulated) depending on the echoendoscope position used for obtaining access to the area of interest (Fig. 1).

In the entire cohort, needle-specific technical success was achieved in 93.2 %, procedural success in 85.2 %, and overall clinical success in 76.5 % of cases at a mean follow-up of 3.1 months. Needle-specific technical success was similar between the straight and angulated cohorts (94.0 vs. 91.2 %, p = 0.74). Procedural success (86.7 vs. 77.2 %, p = 0.05), and rate of clinical success was similar between the cohorts (83.3 vs. 86.4 %, p = 0.79), respectively. Overall adverse events were noted in 14.2 % of patients with no difference between the straight and angulated cohorts (p = 0.48).

This study demonstrates equivalent technical success, clinical success and safety of using a flexible 19-G needle in straight and angulated endoscope positions for interventional EUS. Therefore, a flexible needle may be considered where an angulated echoendoscope position is encountered.

C1 [Kumbhari, Vivek; Tieu, Alan H.; Ngamruengphong, Saowanee; El-Zein, Mohamad H.; Saxena, Payal; Howard, Linda; Khashab, Mouen A.] Johns Hopkins Univ Hosp, Div Gastroenterol & Hepatol, Dept Med, 1800 Orleans St,Suite 7125 B, Baltimore, MD 21205 USA.

[Penas, Irene; De la Serna-Higuera, Carlos; Perez-Miranda, Manuel] Hosp Univ Rio Hortega, Dept Gastroenterol, Endoscopy Unit, Valladolid, Spain.

[Juneja, Manie; Maufa, Fuad; Haddad, Nadim] Georgetown Univ Med Ctr, Div Gastroenterol & Hepatol, Washington,

DC USA.
[Krishnan, Sandeep; Gonzalez, Susana; DiMaio, Christopher J.] Icahn Sch Med Mt Sinai, Div Gastroenterol, New York, NY 10029 USA.
[Renny, Peter V.; Buscaglia, Jonathan M.] SUNY Stony Brook, Sch Med, Dept Gastroenterol & Hepatol, Stony Brook, NY 11794 USA.
C3 Johns Hopkins University; Johns Hopkins Medicine; Hospital del Rio
Hortega; Georgetown University; Icahn School of Medicine at Mount Sinai;
State University of New York (SUNY) System; Stony Brook University
RP Tieu, AH (corresponding author), Johns Hopkins Univ Hosp, Div Gastroenterol & Hepatol, Dept Med, 1800 Orleans St,Suite 7125 B, Baltimore, MD 21205 USA.
EM alan.tieu@jhmi.edu; mkhasha1@jhmi.edu
RI khashab, mouen/F-6643-2017; Ngamruengphong, Saowanee/P-5094-2019;
Howard, Linda/B-2463-2009
OI Peñas Herrero, Irene/0009-0007-9986-8107
FU Olympus Australia; Cook Medical; Pentax Medical
FX Jonathan M. Bucaglia is a consultant for Boston Scientific. Payal Saxena
is a consultant for Boston Scientific and has received consulting fees
from Olympus Australia, Cook Medical, Pentax Medical and research
support from Cook Medical. Mouen A. Khashab is a consultant for Boston
Scientific and Olympus America and has received research support from
Cook Medical. All other authors have no relevant disclosures.
CR Artifon ELA, 2015, GASTROINTEST ENDOSC, V81, P950, DOI 10.1016/j.gie.2014.09.047
Artifon ELA, 2012, J CLIN GASTROENTEROL, V46, P768, DOI 10.1097/MCG.0b013e31825f264c
Bang JY, 2013, GASTROINTEST ENDOSC, V77, P739, DOI 10.1016/j.gie.2012.11.029
Chavalitdhamrong D, 2013, GUT LIVER, V7, P756, DOI 10.5009/gnl.2013.7.6.756
Choi JH, 2015, DIGEST ENDOSC, V27, P1, DOI 10.1111/den.12386
Cotton PB, 2010, GASTROINTEST ENDOSC, V71, P446, DOI 10.1016/j.gie.2009.10.027
Doi S, 2013, ENDOSCOPY, V45, P362, DOI 10.1055/s-0032-1326225
Holt B, 2015, J HEPATO-BIL-PAN SCI, V22, P12, DOI 10.1002/jhbp.150
Ishiwatari H, 2015, GASTROINTEST ENDOSC, V81, P479, DOI 10.1016/j.gie.2014.10.031
Itoi T, 2015, ENDOSCOPY, V47, P462, DOI 10.1055/s-0034-1391083
Kahaleh M, 2015, WORLD J GASTROENTERO, V21, P726, DOI 10.3748/wjg.v21.i3.726
Katanuma A, 2015, J HEPATO-BIL-PAN SCI, V22, P379, DOI 10.1002/jhbp.201
Khashab MA, 2015, DIGEST DIS SCI, V60, P557, DOI 10.1007/s10620-014-3300-6
Khashab MA, 2013, GASTROINTEST ENDOSC, V78, P102, DOI 10.1016/j.gie.2013.03.004
LeBlanc JK, 2011, GASTROINTEST ENDOSC, V74, P1300, DOI 10.1016/j.gie.2011.07.073
Musumba C, 2014, ENDOSCOPY, V46, P690, DOI 10.1055/s-0034-1365424
Ogura T, 2015, ENDOSCOPY, V47, P72, DOI 10.1055/s-0034-1378111
Park DH, 2013, GASTROINTEST ENDOSC, V78, P91, DOI 10.1016/j.gie.2013.01.042
Ramesh J, 2015, PANCREAS, V44, P128, DOI 10.1097/MPA.0000000000000217
Romero-Castro R, 2013, GASTROINTEST ENDOSC, V78, P711, DOI 10.1016/j.gie.2013.05.009
Song TJ, 2010, AM J GASTROENTEROL, V105, P1739, DOI 10.1038/ajg.2010.108

Storm AC, 2014, GASTROINTEST ENDOSC, V80, P164, DOI 10.1016/j.gie.2014.04.005
Tyberg A, 2015, GASTROENTEROLOGY, V148, P506, DOI 10.1053/j.gastro.2014.12.029
Varadarajulu S, 2013, GASTROENTEROLOGY, V145, P583, DOI 10.1053/j.gastro.2013.05.046
Varadarajulu S, 2012, GASTROINTEST ENDOSC, V76, P336, DOI 10.1016/j.gie.2012.04.455
Varadarajulu S, 2011, J GASTROINTEST SURG, V15, P2080, DOI 10.1007/s11605-011-1621-8
Varadarajulu S, 2011, J GASTROEN HEPATOL, V26, P1504, DOI 10.1111/j.1440-1746.2011.06771.x
NR 27
TC 6
Z9 7
U1 0
U2 2
PU SPRINGER
PI DORDRECHT
PA VAN GODEWIJCKSTRAAT 30, 3311 GZ DORDRECHT, NETHERLANDS
SN 0163-2116
EI 1573-2568
J9 DIGEST DIS SCI
JI Dig. Dis. Sci.
PD DEC
PY 2016
VL 61
IS 12
BP 3552
EP 3559
DI 10.1007/s10620-016-4327-7
PG 8
WC Gastroenterology & Hepatology
WE Science Citation Index Expanded (SCI-EXPANDED)
SC Gastroenterology & Hepatology
GA EC6DT
UT WOS:000388227400026
PM 27714511
DA 2026-05-09
ER

PT J
AU Lesmana, CRA
Mandasari, BKD
AF Lesmana, Cosmas Rinaldi Adithya
Mandasari, Baiq Kirana Dyah
TI The new era of endoscopic ultrasound in biliary disorders
SO CLINICAL JOURNAL OF GASTROENTEROLOGY

LA English

DT Review

DE Biliary obstruction; Portal hypertension; Endoscopic ultrasound (EUS);
EUS-guided biliary drainage (EUS-BD); EUS-guided gallbladder drainage
(EUS-GBD)

ID GUIDED GALLBLADDER DRAINAGE; METAL STENT; PANCREATIC PSEUDOCYST; EUS;
ULTRASONOGRAPHY; SAFETY; PLACEMENT; EFFICACY; ACCESS

AB Biliary obstruction is one of challenging biliary disorders in gastroenterology field, where this long-standing condition can also lead to portal hypertension and multi-disciplinary teamwork is usually needed to manage this problem. Biliary drainage is the primary management to prevent prolonged cholestasis. Biliary system with its thin-walled and tubular structure sometimes makes the diagnosis and therapeutic not easy to approach. Over the past 3 decades, numerous new and modern diagnostic and therapeutic modalities have been developed to manage the complex biliary problems. It is well known that endoscopic retrograde cholangiopancreatography (ERCP), percutaneous transhepatic biliary drainage (PTBD), and surgical procedure are common procedures in managing biliary disorders. However, surgical therapy and ERCP are not always easy to perform due to several contraindications. Because of difficulty in performing these procedures or unavailability of these procedures in the facility, PTBD, as the primary non-surgical procedure of choice, has been popular due to its easy technique. Endoscopic ultrasound (EUS) has evolved significantly not only as a diagnostic tool for identification and staging, but also for interventional approaches, especially in management of biliary malignancy. Recently, EUS-guided biliary drainage (EUS-BD) and EUS-guided gallbladder drainage (EUS-GBD) are developed for managing biliary disorders. Whether EUS can be useful for managing biliary obstruction as a primary procedure is still controversial. Hence, a large number of further studies are required to validate.

C1 [Lesmana, Cosmas Rinaldi Adithya; Mandasari, Baiq Kirana Dyah] Univ Indonesia, Med Fac, Dr Cipto Mangunkusumo Natl Gen Hosp, Dept Internal Med,Div Hepatobiliary, JL Diponegoro 71, Jakarta 10430, Dki Jakarta, Indonesia.
[Lesmana, Cosmas Rinaldi Adithya] Medistra Hosp, Digest Dis & GI Oncol Ctr, Jakarta, Indonesia.

C3 University of Indonesia; Medistra Hospital

RP Lesmana, CRA (corresponding author), Univ Indonesia, Med Fac, Dr Cipto Mangunkusumo Natl Gen Hosp, Dept Internal Med,Div Hepatobiliary, JL Diponegoro 71, Jakarta 10430, Dki Jakarta, Indonesia.; Lesmana, CRA (corresponding author), Medistra Hosp, Digest Dis & GI Oncol Ctr, Jakarta, Indonesia.

EM medicaldr2001id@yahoo.com

CR Artifon ELA, 2012, J PANCREAS, V13, P7
Bapaye A, 2013, UNITED EUR GASTROENT, V1, P285, DOI 10.1177/2050640613490928
Campos S, 2019, ENDOSC INT OPEN, V7, pE1262, DOI 10.1055/a-0958-2183
Cheriyan Danny, 2015, Gastroenterol Hepatol (N Y), V11, P467
de la Serna-Higuera C, 2013, GASTROINTEST ENDOSC, V77, P303, DOI 10.1016/j.gie.2012.09.021
DeWitt J, 2018, GASTROENTEROLOGY, V155, P237, DOI 10.1053/j.gastro.2018.04.002
Dhir V, 2013, UNITED EUR GASTROENT, V1, P103, DOI 10.1177/2050640613480145
Dhir V, 2014, DIGEST ENDOSC, V26, P430, DOI 10.1111/den.12153
DIMAGNO EP, 1980, LANCET, V1, P629
DIMAGNO EP, 1982, GASTROENTEROLOGY, V83, P824
DiMagno EP, 2016, DIGEST DIS SCI, V61, P342, DOI 10.1007/s10620-015-3999-8
Giovannini M, 2001, ENDOSCOPY, V33, P898, DOI 10.1055/s-2001-17324
Giovannini M, 2012, GASTROENT RES PRACT, V2012, DOI 10.1155/2012/348719

Hedjoudje A, 2019, UNITED EUR GASTROENT, V7, P60, DOI 10.1177/2050640618808147
Imai H, 2016, GASTROINTEST ENDOSC, V84, P147, DOI 10.1016/j.gie.2015.12.024
Imaoka H, 2019, J CLIN MED, V8, DOI 10.3390/jcm8081173
Itoi T, 2018, ENDOSC ULTRASOUND, V7, P76, DOI 10.4103/eus.eus_4_18
Itoi T, 2012, GASTROINTEST ENDOSC, V75, P870, DOI 10.1016/j.gie.2011.10.020
Iwashita T, 2012, ENDOSCOPY, V44, P60, DOI 10.1055/s-0030-1256871
Iwashita T, 2014, CLIN J GASTROENTEROL, V7, P94, DOI 10.1007/s12328-014-0467-5
James TW, 2019, ENDOSC ULTRASOUND, V8, pS28, DOI 10.4103/eus.eus_41_19
Jang JW, 2012, GASTROENTEROLOGY, V142, P805, DOI 10.1053/j.gastro.2011.12.051
Jang JW, 2011, GASTROINTEST ENDOSC, V74, P176, DOI 10.1016/j.gie.2011.03.1120
Jenssen C, 2012, WORLD J GASTROENTERO, V18, P4659, DOI 10.3748/wjg.v18.i34.4659
Khan MA, 2017, GASTROINTEST ENDOSC, V85, P76, DOI 10.1016/j.gie.2016.06.032
Kim TH, 2020, PLOS ONE, V15, DOI 10.1371/journal.pone.0240219
KIMMEY MB, 1989, GASTROENTEROLOGY, V96, P433, DOI 10.1016/0016-5085(89)91568-0
Lesmana C Rinaldi A, 2015, Case Rep Gastroenterol, V9, P206, DOI [10.1159/000431308, 10.1159/000431308]
McGrath K., 2006, DIS GALLBLADDER BILE, P97, DOI [10.1002/9780470986981.ch5, DOI 10.1002/9780470986981.CH5]
Merkle EM., 2006, DIS GALLBLADDER BILE, P71
Patel N, 2015, FRONTLINE GASTROENTE, V6, P101, DOI 10.1136/flgastro-2014-100448
Paumgartner G, 2015, HARRISON PRINCIPLE I, P2076
ROSCH T, 1992, GASTROENTEROLOGY, V102, P188
ROSCH T, 1992, NEW ENGL J MED, V326, P1721, DOI 10.1056/NEJM199206253262601
Shaffer EA, 2005, 1 PRINCIPLES GASTROE, V5th, P460
Sharaiha RZ, 2017, GASTROINTEST ENDOSC, V85, P904, DOI 10.1016/j.gie.2016.12.023
Sharma M, 2014, J CLIN EXP HEPATOL, V4, pS53, DOI 10.1016/j.jceh.2013.08.015
Sivak MV, 2006, GUT, V55, P1061, DOI 10.1136/gut.2005.086371
Song TJ, 2014, GASTROINTEST ENDOSC, V80, P707, DOI 10.1016/j.gie.2014.05.327
Umeda J, 2015, J GASTROENTEROL, V50, P940, DOI 10.1007/s00535-015-1096-6
Varadarajulu S, 2013, GASTROENTEROLOGY, V145, P583, DOI 10.1053/j.gastro.2013.05.046
VILMANN P, 1992, GASTROINTEST ENDOSC, V38, P172, DOI 10.1016/S0016-5107(92)70385-X
Washington K., 2006, DIS GALLBLADDER BILE, P21, DOI [10.1002/9780470986981.ch2, DOI 10.1002/9780470986981.CH2]
Widmer J, 2014, DIGEST ENDOSC, V26, P525, DOI 10.1111/den.12221
Xu MM, 2016, THER ADV GASTROENTER, V9, P138, DOI 10.1177/1756283X15618178
NR 45
TC 1
Z9 1
U1 0
U2 4
PU SPRINGER JAPAN KK
PI TOKYO
PA SHIROYAMA TRUST TOWER 5F, 4-3-1 TORANOMON, MINATO-KU, TOKYO, 105-6005,

JAPAN
SN 1865-7257
EI 1865-7265
J9 CLIN J GASTROENTEROL
JI Clin. J. Gastroenterol.
PD AUG
PY 2021
VL 14
IS 4
BP 923
EP 931
DI 10.1007/s12328-021-01419-1
EA APR 2021
PG 9
WC Gastroenterology & Hepatology
WE Emerging Sources Citation Index (ESCI)
SC Gastroenterology & Hepatology
GA TR1DP
UT WOS:000643188600001
PM 33895970
DA 2026-05-09
ER

PT J
AU Lariño-Noia, J
Fernández, RM
Novo, MP
García, DD
Iglesias-García, J
Castiñeira, AQ
Pérez, EV
Dominguez-Muñoz, JE
AF Larino-Noia, Jose
Mejuto Fernandez, Rafael
Paz Novo, Manuel
de la Iglesia Garcia, Daniel
Iglesias-Garcia, Julio
Quiroga Castineira, Adriano
Varo Perez, Evaristo
Enrique Dominguez-Munoz, Juan
TI Emergent endoscopic ultrasound-guided cholecystoduodenostomy does not
prevent R0 resection in a pancreaticoduodenectomy for pancreatic cancer

SO CLINICAL JOURNAL OF GASTROENTEROLOGY

LA English

DT Article

DE Endosonography; Gallbladder drainage; Lumen apposing metal stents;
Pancreatic cancer; Pancreaticoduodenectomy

ID EUS; DRAINAGE

AB Endoscopic ultrasound-guided biliary drainage, mainly choledochoduodenostomy, is commonly used as rescue therapy after Endoscopic Retrograde Cholangiopancreatography (ERCP) in malignant distal biliary obstruction due to un-resectable pancreatic cancer. An alternative when the cystic duct is patent and choledochoduodenostomy is not feasible is performing an Endoscopic Ultrasound-gallbladder drainage. The advent of the Lumen Apposing Metal Stents (LAMS) has shortened and simplified this procedure. However, many concerns exist about the performance of these procedures with metal stents preoperatively in resectable tumors. The evidence about Endoscopic Ultrasound-gallbladder drainage before surgery of pancreatic cancer is scarce. An emergent Endoscopic Ultrasound (EUS)-gallbladder drainage (cholecysto-duodenostomy) was performed due to acute cholangitis in the setting of a resectable pancreatic tumor. Surgery after neoadjuvant therapy was done three months later. A complete resection of tumor was feasible. EUS-guided gallbladder drainage using LAMS does not preclude performing a complete resection of a pancreatic head tumor.

C1 [Larino-Noia, Jose; Mejuto Fernandez, Rafael; de la Iglesia Garcia, Daniel; Iglesias-Garcia, Julio; Quiroga Castineira, Adriano; Enrique Dominguez-Munoz, Juan] Univ Hosp Santiago de Compostela, Hlth Res Inst Santiago IDIS, Dept Gastroenterol & Hepatol, La Coruna 15895, Spain.

[Paz Novo, Manuel; Varo Perez, Evaristo] Univ Hosp Santiago de Compostela, Hlth Res Inst Santiago IDIS, Liver Transplant Unit, Surg Dept, La Coruna 15895, Spain.

C3 Complexo Hospitalario Universitario de Santiago de Compostela; Complexo
Hospitalario Universitario de Santiago de Compostela

RP Lariño-Noia, J (corresponding author), Univ Hosp Santiago de Compostela, Hlth Res Inst Santiago IDIS, Dept Gastroenterol & Hepatol, La Coruna 15895, Spain.

EM joselarnoi@outlook.es

RI Dominguez-Muñoz, Enrique/Q-8922-2017; de la Iglesia,
Daniel/AAF-8955-2021

OI Dominguez-Muñoz, Enrique/0000-0001-6133-538X; LARIÑO,
JOSE/0000-0003-4892-6702; de la Iglesia, Daniel/0000-0002-1826-2282

CR Baars JE, 2018, ENDOSC ULTRASOUND, V7, P4, DOI 10.4103/eus.eus_105_17
Baron TH, 2015, NEW ENGL J MED, V373, P357, DOI 10.1056/NEJMra1411372
Chin JYL, 2020, ENDOSC INT OPEN, V08, pE1633, DOI 10.1055/a-1236-3217
Fugazza A, 2022, DIGEST LIVER DIS, V54, P529, DOI 10.1016/j.dld.2021.07.010
Imai H, 2016, GASTROINTEST ENDOSC, V84, P147, DOI 10.1016/j.gie.2015.12.024
Jacques J, 2020, GASTROINTEST ENDOSC, V92, P134, DOI 10.1016/j.gie.2020.01.055
Jin Z, 2020, DIGEST ENDOSC, V32, P16, DOI 10.1111/den.13456
Luk SWY, 2019, ENDOSCOPY, V51, P722, DOI 10.1055/a-0929-6603
Nakai Y, 2017, DIGEST ENDOSC, V29, P218, DOI 10.1111/den.12752
Teoh AYB, 2020, GUT, V69, P1085, DOI 10.1136/gutjnl-2019-319996
Wada K, 2007, J HEPATO-BILIARY-PAN, V14, P52, DOI 10.1007/s00534-006-1156-7

NR 11

TC 4
Z9 5
U1 0
U2 0
PU SPRINGER JAPAN KK
PI TOKYO
PA SHIROYAMA TRUST TOWER 5F, 4-3-1 TORANOMON, MINATO-KU, TOKYO, 105-6005,
JAPAN
SN 1865-7257
EI 1865-7265
J9 CLIN J GASTROENTEROL
JI Clin. J. Gastroenterol.
PD FEB
PY 2022
VL 15
IS 1
BP 263
EP 267
DI 10.1007/s12328-021-01540-1
EA OCT 2021
PG 5
WC Gastroenterology & Hepatology
WE Emerging Sources Citation Index (ESCI)
SC Gastroenterology & Hepatology
GA ZD0ZX
UT WOS:000710834300001
PM 34694600
DA 2026-05-09
ER

PT J
AU Shah, SL
Perez-Miranda, M
Kahaleh, M
Tyberg, A
AF Shah, Shawn L.
Perez-Miranda, Manuel
Kahaleh, Michel
Tyberg, Amy
TI Updates in Therapeutic Endoscopic Ultrasonography
SO JOURNAL OF CLINICAL GASTROENTEROLOGY
LA English

DT Review
DE therapeutic endoscopic ultrasound; biliary duct drainage; pancreatic
duct drainage; EUS-guided gallbladder drainage; EUS-guided
gastroenterostomy; EUS-directed transgastric ERCP
ID GUIDED GALLBLADDER DRAINAGE; GASTRIC OUTLET OBSTRUCTION; PANCREATIC-DUCT
DRAINAGE; APPOSING METAL STENT; FAILED ERCP; RETROGRADE
CHOLANGIOPANCREATOGRAPHY; INTERNATIONAL MULTICENTER; ACUTE
CHOLECYSTITIS; BILIARY DRAINAGE; BYPASS PATIENTS
AB The last decade has seen a dramatic rise in the possibilities of therapeutic endoscopic ultrasound (EUS). From EUS fine needle aspiration of cancerous lesion to pseudocyst drainage, it has now not only replaced some of the percutaneous techniques but has permitted to bypass all together laparoscopic approach for patient with altered anatomy or malignant gastric outlet obstruction. This review will emphasize the novel therapeutic EUS procedures added to our arsenal.
C1 [Shah, Shawn L.] New York Presbyterian Hosp, Weill Cornell Med, Dept Gastroenterol, New York, NY USA.
[Perez-Miranda, Manuel] Hosp Univ Rio Hortega, Valladolid, Spain.
[Kahaleh, Michel; Tyberg, Amy] Robert Wood Johnson Med Ctr, Dept Gastroenterol, New Brunswick, NJ USA.
C3 Cornell University; Weill Cornell Medicine; NewYork-Presbyterian
Hospital; Hospital del Rio Hortega; Rutgers University System; Rutgers
University New Brunswick; Rutgers University Biomedical & Health
Sciences
RP Kahaleh, M (corresponding author), Robert Wood Johnson Univ Hosp, New Brunswick, NJ 08901 USA.
EM mkahaleh@gmail.com
RI Kahaleh, Michel/A-9408-2011
CR Anderloni A, 2016, SURG ENDOSC, V30, P5200, DOI 10.1007/s00464-016-4894-x
[Anonymous], 2018, J CLIN GASTROENTEROL
Barkay O, 2010, GASTROINTEST ENDOSC, V71, P1166, DOI 10.1016/j.gie.2009.10.048
Brauer BC, 2009, GASTROINTEST ENDOSC, V70, P471, DOI 10.1016/j.gie.2008.12.233
Chen YI, 2017, GASTROINTEST ENDOSC, V85, P170, DOI 10.1016/j.gie.2016.07.031
Choi JH, 2014, ENDOSCOPY, V46, P656, DOI 10.1055/s-0034-1365720
Chopita N, 2005, ENDOSCOPY, V37, P313, DOI 10.1055/s-2005-861358
Dhir V, 2017, DIGEST ENDOSC, V29, P472, DOI 10.1111/den.12818
DIMAGNO EP, 1982, GASTROENTEROLOGY, V83, P824
Dollhopf M, 2017, GASTROINTEST ENDOSC, V86, P636, DOI 10.1016/j.gie.2017.02.027
Elton E, 1998, GASTROINTEST ENDOSC, V47, P62, DOI 10.1016/S0016-5107(98)70300-1
Ergun M, 2011, ENDOSCOPY, V43, P518, DOI 10.1055/s-0030-1256333
François E, 2002, GASTROINTEST ENDOSC, V56, P128, DOI 10.1067/mge.2002.125547
Fujii LL, 2013, GASTROINTEST ENDOSC, V78, P854, DOI 10.1016/j.gie.2013.05.016
Fujii-Lau LL, 2015, J HEPATO-BIL-PAN SCI, V22, P51, DOI 10.1002/jhbp.187
Itoi T, 2016, GUT, V65, P193, DOI 10.1136/gutjnl-2015-310348
Jang JW, 2012, GASTROENTEROLOGY, V142, P805, DOI 10.1053/j.gastro.2011.12.051
Kahaleh M, 2003, GASTROINTEST ENDOSC, V58, P919, DOI 10.1016/S0016-5107(03)02297-1
Kahaleh M, 2007, GASTROINTEST ENDOSC, V65, P224, DOI 10.1016/j.gie.2006.05.008
Kahaleh M, 2016, DIGEST LIVER DIS, V48, P1054, DOI 10.1016/j.dld.2016.05.021

Kedia P, 2015, GASTROINTEST ENDOSC, V81, P223, DOI 10.1016/j.gie.2014.04.007
Khashab MA, 2017, ENDOSC INT OPEN, V5, pE275, DOI 10.1055/s-0043-101695
Kinney T P, 2005, Minerva Gastroenterol Dietol, V51, P265
Kurihara T, 2013, DIGEST ENDOSC, V25, P109, DOI 10.1111/den.12100
Liao WC, 2017, GASTROINTEST ENDOSC, V85, P295, DOI 10.1016/j.gie.2016.09.037
Mallery S, 2004, GASTROINTEST ENDOSC, V59, P100, DOI 10.1016/S0016-5107(03)02300-9
Maple JT, 2017, GASTROINTEST ENDOSC, V85, P465, DOI 10.1016/j.gie.2016.11.021
Medina-Franco H, 2007, AM SURGEON, V73, P871
Ogura T, 2016, ENDOSCOPY, V48, P156, DOI 10.1055/s-0034-1392859
Oh HC, 2007, ENDOSCOPY, V39, P731, DOI 10.1055/s-2007-966577
Perez-Miranda M, 2017, J CLIN GASTROENTEROL, V51, P896, DOI 10.1097/MCG.0000000000000887
Phillips MS, 2008, J GASTROINTEST SURG, V12, P2045, DOI 10.1007/s11605-008-0598-4
Poincloux L, 2015, ENDOSCOPY, V47, P794, DOI [10.1055/s-0035-1545399, 10.1055/s-0034-1391988]
Saleem A, 2012, J GASTROINTEST SURG, V16, P203, DOI 10.1007/s11605-011-1760-y
Schreiner MA, 2012, GASTROINTEST ENDOSC, V75, P748, DOI 10.1016/j.gie.2011.11.019
Shah JN, 2012, GASTROINTEST ENDOSC, V75, P56, DOI 10.1016/j.gie.2011.08.032
Skinner M, 2014, ENDOSCOPY, V46, P560, DOI 10.1055/s-0034-1365698
Teoh AYB, 2017, ENDOSCOPY, V49, P130, DOI 10.1055/s-0042-119036
Tessier G, 2007, GASTROINTEST ENDOSC, V65, P233, DOI 10.1016/j.gie.2006.06.029
Tyberg A, 2018, J CLIN GASTROENTEROL, V52, P79, DOI 10.1097/MCG.0000000000000786
Tyberg A, 2017, CLIN ENDOSC, V50, P185, DOI 10.5946/ce.2016.030
Tyberg A, 2017, GASTROINTEST ENDOSC, V85, P164, DOI 10.1016/j.gie.2016.07.030
Tyberg A, 2016, GASTROINTEST ENDOSC, V84, P941, DOI 10.1016/j.gie.2016.05.035
Tyberg A, 2016, ENDOSC INT OPEN, V4, pE276, DOI 10.1055/s-0042-101789
Uemura RS, 2018, J CLIN GASTROENTEROL, V52, P123, DOI 10.1097/MCG.0000000000000948
Vila JJ, 2012, GASTROINTEST ENDOSC, V76, P1133, DOI 10.1016/j.gie.2012.08.001
Wallace MB, 2017, GASTROENTEROLOGY, V153, P364, DOI 10.1053/j.gastro.2017.06.014
Walter D, 2016, GUT, V65, P6, DOI 10.1136/gutjnl-2015-309925
Wang KX, 2016, GASTROINTEST ENDOSC, V83, P1218, DOI 10.1016/j.gie.2015.10.033
Will U, 2007, J HEPATO-BILIARY-PAN, V14, P377, DOI 10.1007/s00534-006-1139-8
Will U, 2015, WORLD J GASTROENTERO, V21, P13140, DOI 10.3748/wjg.v21.i46.13140
NR 51
TC 5
Z9 8
U1 0
U2 7
PU LIPPINCOTT WILLIAMS & WILKINS
PI PHILADELPHIA
PA TWO COMMERCE SQ, 2001 MARKET ST, PHILADELPHIA, PA 19103 USA
SN 0192-0790
EI 1539-2031
J9 J CLIN GASTROENTEROL

JI J. Clin. Gastroenterol.
PD OCT
PY 2018
VL 52
IS 9
BP 765
EP 772
DI 10.1097/MCG.0000000000001104
PG 8
WC Gastroenterology & Hepatology
WE Science Citation Index Expanded (SCI-EXPANDED)
SC Gastroenterology & Hepatology
GA GV0LW
UT WOS:000445753800003
PM 30106838
DA 2026-05-09
ER

PT J
AU Fugazza, A
Khalaf, K
Pawlak, KM
Spadaccini, M
Colombo, M
Andreozzi, M
Carrara, S
Giacchetto, M
Ferrari, C
Binda, C
Mangiavillano, B
Anderloni, A
Repici, A
AF Fugazza, Alessandro
Khalaf, Kareem
Pawlak, Katarzyna M.
Spadaccini, Marco
Colombo, Matteo
Andreozzi, Marta
Carrara, Silvia
Giacchetto, Marco
Ferrari, Chiara
Binda, Cecilia

Mangiavillano, Benedetto
Anderloni, Andrea
Repici, Alessandro
TI Use of endoscopic ultrasound-guided gallbladder drainage as a rescue approach in cases of unsuccessful biliary drainage
SO WORLD JOURNAL OF GASTROENTEROLOGY
LA English
DT Review
DE Endoscopic ultrasound-guided gallbladder drainage; Gallbladder drainage; Acute cholecystitis; Malignant obstruction; Interventional endoscopic ultrasound; Lumen-apposing metal stents
ID OBSTRUCTION; STENT; ERCP
AB This narrative review provides an overview of the utilization of endoscopic ultrasound-guided gallbladder drainage (EUS-GBD) as a salvage approach in cases of unsuccessful conventional management. EUS-GBD is a minimally invasive and effective technique for drainage in patients with acute cholecystitis with high risk of surgery. The procedure has demonstrated impressive technical and clinical success rates with low rates of adverse events, making it a safe and effective option for appropriate candidates. Furthermore, EUS-GBD can also serve as a rescue option for patients who have failed endoscopic retrograde cholangiopancreatography or EUS biliary drainage for relief of jaundice in malignant biliary stricture. However, patient selection is critical for the success of EUS-GBD, and proper patient selection and risk assessment are important to ensure the safety and efficacy of the procedure. As the field continues to evolve and mature, ongoing research will further refine our understanding of the benefits and limitations of EUS-GBD, ultimately leading to improved outcomes for patients.
C1 [Fugazza, Alessandro; Spadaccini, Marco; Colombo, Matteo; Andreozzi, Marta; Carrara, Silvia; Giacchetto, Marco; Repici, Alessandro] Human Res Hosp IRCCS, Div Gastroenterol & Digest Endoscopy, Via Manzoni 56, I-20089 Rozzano, Milano, Italy.
[Khalaf, Kareem; Pawlak, Katarzyna M.] Univ Toronto, St Michaels Hosp, Div Gastroenterol, Toronto, ON M5B 1W8, Canada.
[Ferrari, Chiara] Human Res Hosp IRCCS, Dept Anesthesia, I-20089 Rozzano, Milano, Italy.
[Binda, Cecilia] Forli Cesena Hosp, Gastroenterol & Digest Endoscopy Unit, I-47121 Romagna, Italy.
[Mangiavillano, Benedetto] Gastrointestinal Endoscopy Unit, Human Mater Domini, I-21100 Castellanza, Italy.
[Anderloni, Andrea] Fdn IRCCS Policlin San Matteo, Gastroenterol & Digest Endoscopy Unit, I-27100 Pavia, Italy.
C3 University of Toronto; Saint Michaels Hospital Toronto; IRCCS Fondazione San Matteo
RP Spadaccini, M (corresponding author), Human Res Hosp IRCCS, Div Gastroenterol & Digest Endoscopy, Via Manzoni 56, I-20089 Rozzano, Milano, Italy.
EM marcospadaccini9@gmail.com
RI Carrara, Silvia/AAC-5298-2022; Ferrari, Chiara/LZI-5669-2025; Fugazza, Alessandro/ABG-9381-2021; Aneozzi, Marta/LGZ-4173-2024; Pawlak, Katarzyna M/AAY-7967-2021; Colombo, Matteo/N-9849-2013; Khalaf, Kareem/NFT-3571-2025; Repici, Alessandro/HFH-8162-2022; Binda, Cecilia/ABH-1588-2020; Spadaccini, Marco/HOH-7613-2023
OI Carrara, Silvia/0000-0003-4206-9463; Ferrari,

Chiara/0009-0008-0779-0945; Fugazza, Alessandro/0000-0003-0485-4903;
Aneozzi, Marta/0009-0003-5271-278X; Pawlak, Katarzyna
M/0000-0002-0771-1177; Colombo, Matteo/0000-0003-0715-8233; Khalaf,
Kareem/0000-0002-5534-7533; Repici, Alessandro/0000-0002-1621-6450;
CR Binda C, 2023, GASTROINTEST ENDOSC, V98, P765, DOI 10.1016/j.gie.2023.06.054
Boregowda U., 2023, Diagnostics (Basel), V13
Chang JI, 2019, ENDOSC INT OPEN, V7, pE655, DOI 10.1055/a-0826-4309
de la Serna-Higuera C, 2013, GASTROINTEST ENDOSC, V77, P303, DOI 10.1016/j.gie.2012.09.021
Facciorusso A, 2022, CANCERS, V14, DOI 10.3390/cancers14133291
Flynn D, 2021, ENDOSC ULTRASOUND, V10, P381, DOI 10.4103/EUS-D-21-00040
Fugazza A, 2022, GASTROINTEST ENDOSC, V95, P896, DOI 10.1016/j.gie.2021.12.032
Fugazza A, 2022, DIGEST LIVER DIS, V54, P529, DOI 10.1016/j.dld.2021.07.010
Fugazza A, 2020, CLIN EXP GASTROENTER, V13, P193, DOI 10.2147/CEG.S203626
Hathorn KE, 2019, ENDOSC INT OPEN, V7, pE1432, DOI 10.1055/a-0990-9488
Imai H, 2016, GASTROINTEST ENDOSC, V84, P147, DOI 10.1016/j.gie.2015.12.024
Irani S, 2017, CLIN GASTROENTEROL H, V15, P738, DOI 10.1016/j.cgh.2016.12.021
Issa D, 2021, ENDOSCOPY, V53, P827, DOI 10.1055/a-1259-0349
Jain D, 2018, CLIN ENDOSC, V51, P450, DOI 10.5946/ce.2018.024
James TW, 2019, ENDOSC ULTRASOUND, V8, pS28, DOI 10.4103/eus.eus_41_19
Jang JW, 2012, GASTROENTEROLOGY, V142, P805, DOI 10.1053/j.gastro.2011.12.051
Kamal F, 2023, ENDOSC ULTRASOUND, V12, P8, DOI 10.4103/EUS-D-21-00206
Kedia P, 2015, GASTROINTEST ENDOSC, V82, P1031, DOI 10.1016/j.gie.2015.03.1912
Khashab MA, 2015, DIGEST DIS SCI, V60, P557, DOI 10.1007/s10620-014-3300-6
Kurihara H, 2022, MEDICINA-LITHUANIA, V58, DOI 10.3390/medicina58111647
Mangiavillano B, 2024, DIGEST ENDOSC, V36, P351, DOI 10.1111/den.14606
Paduano D, 2023, CANCERS, V15, DOI 10.3390/cancers15020490
Paik WH, 2018, AM J GASTROENTEROL, V113, P987, DOI 10.1038/s41395-018-0122-8
Perez-Miranda M, 2018, ENDOSC ULTRASOUND, V7, P79, DOI 10.4103/eus.eus_5_18
Posner H, 2020, TRANSL GASTROENT HEP, V5, DOI 10.21037/tgh.2019.12.20
Raiter A, 2022, ENDOSCOPY, V54, pE55, DOI 10.1055/a-1352-2468
Sharaiha RZ, 2017, GASTROINTEST ENDOSC, V85, P904, DOI 10.1016/j.gie.2016.12.023
Sharaiha RZ, 2016, SURG ENDOSC, V30, P5500, DOI 10.1007/s00464-016-4913-y
Teh JL, 2022, BEST PRACT RES CL GA, V60, DOI 10.1016/j.bpg.2022.101806
Teoh AYB, 2017, ENDOSCOPY, V49, P130, DOI 10.1055/s-0042-119036
Tyberg A, 2018, J CLIN GASTROENTEROL, V52, P79, DOI 10.1097/MCG.0000000000000786
van der Merwe SW, 2022, ENDOSCOPY, V54, P185, DOI 10.1055/a-1717-1391
Widmer J, 2014, DIGEST ENDOSC, V26, P599, DOI 10.1111/den.12163
NR 33
TC 6
Z9 7
U1 0
U2 0

PU BAISHIDENG PUBLISHING GROUP INC
PI PLEASANTON
PA 7041 Koll Center Parkway, Suite 160, PLEASANTON, CA, UNITED STATES
SN 1007-9327
EI 2219-2840
J9 WORLD J GASTROENTERO
JI World J. Gastroenterol.
PD JAN 7
PY 2024
VL 30
IS 1
DI 10.3748/wjg.v30.i1.70
PG 10
WC Gastroenterology & Hepatology
WE Science Citation Index Expanded (SCI-EXPANDED)
SC Gastroenterology & Hepatology
GA HC6G9
UT WOS:001157325400004
PM 38293324
OA Green Submitted, hybrid
DA 2026-05-09
ER

PT J
AU Jang, DK
Lee, DW
Kim, SH
Cho, KB
Lakhtakia, S
AF Jang, Dong Kee
Lee, Dong Wook
Kim, Seong-Hun
Cho, Kwang Bum
Lakhtakia, Sundeep
TI Advances in self-expandable metal stents for endoscopic
ultrasound-guided interventions
SO CLINICAL ENDOSCOPY
LA English
DT Review
DE Drainage; Endosonography; Self expandable metallic stents
ID GALLBLADDER DRAINAGE
AB Endoscopic ultrasound (EUS)-guided interventions have evolved rapidly in recent years, with dedicated metal stents

playing a crucial role in this process. Specifically, the invention of biflanged short metal-covered stents, including lumen-apposing metal stents (LAMS), and modifications in a variety of tubular self-expandable metal stents (SEMS), have led to innovations in EUS-guided interventions. LAMS or non- LAMS stents are commonly used in the EUS-guided drainage of pancreatic fluid collections, especially in cases of walled-off necrosis. Additionally, LAMS is commonly considered for drainage of the EUS-guided gallbladder or dilated common bile duct and EUS-guided gastroenterostomy. Fully or partially covered tubular SEMS with several new designs are being considered for EUS-guided biliary drainage. This review focuses on advances in SEMS for EUS-guided interventions and discusses related research results.
C1 [Jang, Dong Kee] Seoul Natl Univ, Dept Internal Med, Seoul Metropolitan Govt Boramae Med Ctr, Coll Med, Seoul, South Korea.
[Lee, Dong Wook] Kyungpook Natl Univ, Sch Med, Dept Internal Med, Daegu, South Korea.
[Kim, Seong-Hun] Jeonbuk Natl Univ Hosp, Dept Internal Med, Jeonju, South Korea.
[Cho, Kwang Bum] Keimyung Univ, Dept Internal Med, Sch Med, Daegu, South Korea.
[Lakhtakia, Sundeep] Asian Inst Gastroenterol, Hyderabad, India.
C3 Seoul National University (SNU); Kyungpook National University (KNU);
Jeonbuk National University; Jeonbuk National University Hospital;
Keimyung University
RP Lakhtakia, S (corresponding author), AIG Hosp, Asian Inst Gastroenterol, Dept Med Gastroenterol, Mindspace Rd, Hyderabad 500034, Telangana, India.; Cho, KB (corresponding author), Keimyung Univ, Dongsan Hosp, Sch Med, Dept Internal Med, 1035 Dalgubeol Daero, Daegu 42601, South Korea.
EM chokb@dsmc.or.kr; drsundeeplakhtakia@gmail.com
RI Lakhtakia, Sundeep/H-3607-2019
OI Lakhtakia, Sundeep/0000-0001-7562-8060; Kim,
Seong-Hun/0000-0002-7592-8060; Lee, Dong Wook/0000-0002-1029-9064; Jang,
Dong Kee/0000-0001-6642-6635
CR Abbas A, 2022, ENDOSCOPY, V54, P671, DOI 10.1055/a-1708-0037
Amato A, 2021, ENDOSCOPY, V53, P1037, DOI 10.1055/a-1324-7919
Arvanitakis M, 2018, ENDOSCOPY, V50, P524, DOI 10.1055/a-0588-5365
Bang JY, 2019, DIGEST ENDOSC, V31, P619, DOI 10.1111/den.13428
Bang JY, 2019, GUT, V68, P1200, DOI 10.1136/gutjnl-2017-315335
Bazerbachi F, 2018, GASTROINTEST ENDOSC, V87, P30, DOI 10.1016/j.gie.2017.08.025
Binmoeller KF, 2011, ENDOSCOPY, V43, P337, DOI 10.1055/s-0030-1256127
Canakis A, 2023, GASTROINTEST ENDOSC, V98, P348, DOI 10.1016/j.gie.2023.03.022
Chan SM, 2017, GASTROINTEST ENDOSC, V85, P1225, DOI 10.1016/j.gie.2016.10.014
Cho DH, 2017, GASTROINTEST ENDOSC, V85, P1067, DOI 10.1016/j.gie.2016.09.010
De Cassan C, 2017, ENDOSC ULTRASOUND, V6, P329, DOI 10.4103/2303-9027.209869
Higa Jennifer T, 2019, Curr Treat Options Gastroenterol, V17, P357, DOI [10.1007/s11938-019-00243-4, 10.1007/s11938-019-00243-4]
Huh G, 2020, SCI REP-UK, V10, DOI 10.1038/s41598-020-67374-y
Irani SS, 2023, CLIN GASTROENTEROL H, V21, P1141, DOI 10.1016/j.cgh.2022.12.039
Ishii S, 2023, ENDOSC ULTRASOUND, V12, P266, DOI 10.4103/EUS-D-22-00104
Jang JW, 2012, GASTROENTEROLOGY, V142, P805, DOI 10.1053/j.gastro.2011.12.051
Karstensen JG, 2023, GUT, V72, P1167, DOI 10.1136/gutjnl-2022-328225

Kedia P, 2015, ENDOSCOPY, V47, P159, DOI 10.1055/s-0034-1390771
Mangiavillano B, 2022, GASTROINTEST ENDOSC, V95, P115, DOI 10.1016/j.gie.2021.07.021
Misra SP, 2009, GASTROINTEST ENDOSC, V70, P317, DOI 10.1016/j.gie.2008.12.054
Ogura T, 2021, DEN OPEN, V1, DOI 10.1002/deo2.8
Oh CH, 2021, CLIN ENDOSC, V54, P505
Ribas PHBV, 2022, CUREUS J MED SCIENCE, V14, DOI 10.7759/cureus.31526
Rinninella E, 2015, GASTROINTEST ENDOSC, V82, P1039, DOI 10.1016/j.gie.2015.04.006
Sentürk H, 2024, CLIN ENDOSC, V57, P209, DOI 10.5946/ce.2023.022
Shibuki T, 2023, CLIN ENDOSC, V56, P802, DOI 10.5946/ce.2022.211
Small AJ, 2018, ENDOSC ULTRASOUND, V7, P89, DOI 10.4103/eus.eus_8_18
So H, 2021, J HEPATO-BIL-PAN SCI, V28, P1130, DOI 10.1002/jhbp.1011
Song TJ, 2019, GASTROINTEST ENDOSC, V90, P507, DOI 10.1016/j.gie.2019.05.033
van Wanrooij RLJ, 2022, ENDOSCOPY, V54, P310, DOI 10.1055/a-1738-6780
Vanek P, 2023, GASTROINTEST ENDOSC, V97, P1070, DOI 10.1016/j.gie.2022.12.026
Vedantam S, 2023, CLIN ENDOSC, V56, P298, DOI 10.5946/ce.2022.299
Walter D, 2016, GUT, V65, P6, DOI 10.1136/gutjnl-2015-309925
Yoo HW, 2022, ENDOSCOPY, V54, P384, DOI 10.1055/a-1518-7039
Yoo HW, 2021, ENDOSCOPY, V53, P922, DOI 10.1055/a-1301-1526
Zhang LY, 2022, ENDOSCOPY, V54, P706, DOI 10.1055/a-1682-7095
NR 36
TC 7
Z9 8
U1 0
U2 0
PU KOREAN SOC GASTROINTESTINAL ENDOSCOPY
PI SEOUL
PA 2003 LG PALACE, 165-8 DONGGYO-DONG, MAPO-GU, SEOUL, 121-754, SOUTH KOREA
SN 2234-2400
EI 2234-2443
J9 CLIN ENDOSC
JI Clin. Endosc.
PD SEP
PY 2024
VL 57
IS 5
BP 588
EP 594
DI 10.5946/ce.2023.169
EA JUL 2024
PG 7
WC Gastroenterology & Hepatology
WE Emerging Sources Citation Index (ESCI)

SC Gastroenterology & Hepatology
GA P1E7V
UT WOS:001266078300001
PM 38978397
OA Green Submitted, gold
DA 2026-05-09
ER

PT J
AU Lakhtakia, S
AF Lakhtakia, Sundeep
TI Complications of diagnostic and therapeutic Endoscopic Ultrasound
SO BEST PRACTICE & RESEARCH CLINICAL GASTROENTEROLOGY
LA English
DT Article
DE Complications of EUS; EUS guided FNA; EUS guided drainage of pancreatic
fluid collection; EUS guided biliary drainage; EUS guided PD drainage
ID GUIDED BILIARY DRAINAGE; FINE-NEEDLE-ASPIRATION; PANCREATIC-FLUID
COLLECTIONS; EXPANDABLE METAL STENTS; THROUGH-THE-SCOPE; TRANSMURAL
DRAINAGE; PRELIMINARY EXPERIENCE; TRANSLUMINAL DRAINAGE;
CLINICAL-EVALUATION; DILATION TECHNIQUE
AB Endoscopic Ultrasound (EUS) provides the unique opportunity to visualize, interrogate and intervene gastrointestinal (GI) luminal, mural or peri-luminal structures and pathology with negligible adverse effects. Diagnostic, upper GI and rectal EUS is feasible, extremely safe, and efficacious. Most EUS guided interventions are safe, effective and minimally invasive, compared to peers in the percutaneous radiological or surgical procedures.

As with any endoscopic procedure, EUS and its guided interventions may be accompanied by adverse events. EUS related complications are generally infrequent in expert hands, and mainly include bleeding and perforation. However, the nature and severity of adverse events associated with each EUS guided procedure are unique. Hence, it is paramount for endosonographer to have sufficient knowledge of the indications, techniques, and potential risks involved before contemplating any given procedure.

Most common intervention with EUS is transmural fine needle aspiration (FNA), which is an extremely safe procedure. EUS guided drainage procedures are rapidly evolving with newer devices and methods being employed. Among them, EUS guided drainage of pancreatic fluid collection-pseudocyst or walled off necrosis (WON), has largely replaced other methods (surgical, percutaneous or non-EUS endoscopic) with acceptable complications. Currently, dedicated metal stents are more widely used compared to plastic stents for drainage of PFC, especially WON. EUS has made a definite impact in biliary access and drainage of obstructed biliary system, in patients where ERCP has failed or is technically not possible, closely competing with percutaneous biliary drainage. In spite of some complications, recent improvement in devices for bilio-enteric fistula creation and stent designs, has added to its safety and efficacy. EUS guided pancreatic duct drainage remains the most challenging of EUS guided interventions where in-roads are being made. (C) 2016 Elsevier Ltd. All rights reserved.
C1 [Lakhtakia, Sundeep] Asian Inst Gastroenterol, 6-3-661 Somajiguda, Hyderabad 500082, Telangana, India.
RP Lakhtakia, S (corresponding author), Asian Inst Gastroenterol, 6-3-661 Somajiguda, Hyderabad 500082, Telangana,

India.
EM drsundeeplakhtakia@gmail.com
RI Lakhtakia, Sundeep/H-3607-2019
OI Lakhtakia, Sundeep/0000-0001-7562-8060
CR Affi A, 2001, GASTROINTEST ENDOSC, V53, P221, DOI 10.1067/mge.2001.111391
Akbar A, 2012, GASTROINTEST ENDOSC, V76, P1060, DOI 10.1016/j.gie.2012.07.014
Anderson MA, 2009, GASTROINTEST ENDOSC, V70, P1060, DOI 10.1016/j.gie.2009.09.040
Antillon MR, 2006, GASTROINTEST ENDOSC, V63, P797, DOI 10.1016/j.gie.2005.10.025
Attam R, 2014, GASTROINTEST ENDOSC, V80, P312, DOI 10.1016/j.gie.2014.02.013
Azar RR, 2006, GASTROINTEST ENDOSC, V63, P688, DOI 10.1016/j.gie.2005.10.032
Banerjee S, 2008, GASTROINTEST ENDOSC, V67, P791, DOI 10.1016/j.gie.2008.02.068
Bapaye A, 2015, DIGEST ENDOSC, V27, P499, DOI 10.1111/den.12421
Barawi M, 2001, GASTROINTEST ENDOSC, V53, P189, DOI 10.1067/mge.2001.108966
Barkay O, 2010, GASTROINTEST ENDOSC, V71, P1166, DOI 10.1016/j.gie.2009.10.048
Baron TH, 2002, GASTROINTEST ENDOSC, V56, P7, DOI 10.1067/mge.2002.125106
Barthet M, 2008, GASTROINTEST ENDOSC, V67, P245, DOI 10.1016/j.gie.2007.06.014
BINMOELLER KF, 1995, GASTROINTEST ENDOSC, V42, P219, DOI 10.1016/S0016-5107(95)70095-1
Bonnot B, 2014, AM J GASTROENTEROL, V109, P607, DOI 10.1038/ajg.2013.473
Boustière C, 2011, ENDOSCOPY, V43, P445, DOI 10.1055/s-0030-1256317
Brückner S, 2015, ENDOSCOPY, V47, P858, DOI 10.1055/s-0034-1392104
CATALANO MF, 1995, GASTROINTEST ENDOSC, V41, P535, DOI 10.1016/S0016-5107(95)70186-9
Chak A, 1997, GASTROINTEST ENDOSC, V45, P291, DOI 10.1016/S0016-5107(97)70272-4
Chandran S, 2015, GASTROINTEST ENDOSC, V81, P127, DOI 10.1016/j.gie.2014.06.025
Chang KJ, 1997, GASTROINTEST ENDOSC, V45, P387, DOI 10.1016/S0016-5107(97)70149-4
Chen HY, 2002, GASTROINTEST ENDOSC, V56, P594, DOI 10.1067/mge.2002.128103
Das A, 2001, GASTROINTEST ENDOSC
Dhir V, 2015, GASTROINTEST ENDOSC, V81, P913, DOI 10.1016/j.gie.2014.09.054
ELOUBEIDI MA, AM J GASTROENTEROL, V98, P2663, DOI DOI 10.1111/J.1572-0241.2003.08666.X
Ergun M, 2011, ENDOSCOPY, V43, P518, DOI 10.1055/s-0030-1256333
Fabbri C, 2012, ENDOSCOPY, V44, P429, DOI 10.1055/s-0031-1291624
Fabbri C, 2011, ENDOSCOPY, V43, P438, DOI 10.1055/s-0030-1256097
FOCKENS P, 1994, GASTROINTEST ENDOSC, V40, P226, DOI 10.1016/S0016-5107(94)70173-3
Fujii LL, 2013, GASTROINTEST ENDOSC, V78, P851
Gardner TB, 2011, GASTROINTEST ENDOSC, V73, P718, DOI 10.1016/j.gie.2010.10.053
Gimeno-Garcia AZ, 2012, ENDOSCOPY S2, V44, P2012
Giovannini M, 2001, ENDOSCOPY, V33, P898, DOI 10.1055/s-2001-17324
Giovannini M, 2001, ENDOSCOPY, V33, P473, DOI 10.1055/s-2001-14967
Gress F, 2002, GASTROINTEST ENDOSC, V56, P864, DOI 10.1067/mge.2002.129602
Gress F, 2001, AM J GASTROENTEROL, V96, P409
Gress FG, 1997, GASTROINTEST ENDOSC, V45, P243, DOI 10.1016/S0016-5107(97)70266-9
Grossman M, 2012, GASTROINTEST ENDOSC, V75, P1294
Hara K, 2016, WORLD J GASTROENTERO, V22, P1297, DOI 10.3748/wjg.v22.i3.1297

Hirota WK, 2003, GASTROINTEST ENDOSC, V58, P475, DOI 10.1067/S0016-5107(03)01883-2
Hoffman BJ, 2002, GASTROINTEST ENDOSC, V56, pS26, DOI 10.1067/mge.2002.127759
Hookey LC, 2006, GASTROINTEST ENDOSC, V63, P635, DOI 10.1016/j.gie.2005.06.028
Isayama H, 2016, J GASTROENTEROL HEPA
Itoi T, 2013, J HEPATO-BIL-PAN SCI, V20, P403, DOI 10.1007/s00534-012-0551-5
Itoi T, 2012, GASTROINTEST ENDOSC, V75, P870, DOI 10.1016/j.gie.2011.10.020
Jacobson BC, 2002, PANCREATOLOGY, V2, P26, DOI 10.1159/000049444
Jacobson BC, 2003, GASTROINTEST ENDOSC, V57, P251, DOI 10.1067/mge.2003.86
Janssen J, 2004, GASTROINTEST ENDOSC, V59, P339, DOI 10.1016/S0016-5107(03)02707-X
Johnson MD, 2009, J CLIN GASTROENTEROL, V43, P586, DOI 10.1097/MCG.0b013e31817440be
Kahaleh M, 2006, ENDOSCOPY, V38, P355, DOI 10.1055/s-2006-925249
Kahaleh M, 2014, GASTROINTEST ENDOSC, V79, pAB305
Kahaleh M, 2007, GASTROINTEST ENDOSC, V65, P224, DOI 10.1016/j.gie.2006.05.008
KALLIMANIS GE, 1995, GASTROINTEST ENDOSC, V41, P540, DOI 10.1016/S0016-5107(95)70187-7
Katanuma A, 2013, DIGEST DIS SCI, V58, P2093, DOI 10.1007/s10620-013-2590-4
Kawakami H., 2015, ENDOSCOPY, P164
Kawakubo K, 2014, J HEPATO-BIL-PAN SCI, V21, P328, DOI 10.1002/jhbp.27
Khan MA, 2015, DIG DIS SCI
Khashab MA, 2013, GASTROINTEST ENDOSC, V78, P734, DOI 10.1016/j.gie.2013.05.013
Lakhtakia S, 2010, GASTROINTEST ENDOSC, V71, pAB136, DOI 10.1016/j.gie.2010.03.122
Lakhtakia S, 2016, ENDOSCOPY, V48, pE180, DOI 10.1055/s-0042-106966
Lalueza A, 2011, ENDOSCOPY S2, V43, P9
Lee TH, 2014, WORLD J GASTROENTERO, V20, P5859, DOI 10.3748/wjg.v20.i19.5859
Levy MJ, 2008, AM J GASTROENTEROL, V103, P98, DOI 10.1111/j.1572-0241.2007.01607.x
Levy MJ, 2007, CLIN GASTROENTEROL H, V5, P684, DOI 10.1016/j.cgh.2007.02.029
Levy MJ, 2003, GASTROINTEST ENDOSC, V57, P672, DOI 10.1067/mge.2003.204
Lopes CV, 2007, SCAND J GASTROENTERO, V42, P524, DOI 10.1080/00365520601065093
Martins FP, 2010, ENDOSCOPY, V42, pE126, DOI 10.1055/s-0029-1243911
Menzel J, 1999, ENDOSCOPY, V31, P291
Minaga K, 2016, GASTROINTEST ENDOSC, V83, P1039, DOI 10.1016/j.gie.2015.10.044
Mortensen MB, 2005, ENDOSCOPY, V37, P146, DOI 10.1055/s-2005-861142
Mukai S, 2015, ENDOSC ULTRASOUND, V4, P120, DOI 10.4103/2303-9027.156738
O'Toole D, 2001, GASTROINTEST ENDOSC, V53, P470, DOI 10.1067/mge.2001.112839
O'Toole TM, 2009, ENDOSCOPY, V41, P593, DOI 10.1055/s-0029-1214868
Okuno N, 2015, ENDOSCOPY, V47, pE311, DOI 10.1055/s-0034-1392314
Paik WH, 2014, WORLD J GASTROENTERO, V20, P5051, DOI 10.3748/wjg.v20.i17.5051
Park DH, 2009, ENDOSCOPY, V41, P842, DOI 10.1055/s-0029-1215133
Pfau PR, 2000, AM J GASTROENTEROL, V95, P2813, DOI 10.1016/S0002-9270(00)01101-1
Poincloux L, 2015, ENDOSCOPY, V47, P794, DOI [10.1055/s-0035-1545399, 10.1055/s-0034-1391988]
Polkowski M, 2012, ENDOSCOPY, V44, P190, DOI 10.1055/s-0031-1291543
Prachayakul V, 2011, ENDOSCOPY, V43, pE213, DOI 10.1055/s-0030-1256395
Prachayakul Varayu, 2012, J Interv Gastroenterol, V2, P202

Rimbas M, 2015, GASTROINTEST ENDOSC, V82, P405, DOI 10.1016/j.gie.2015.03.1907
Ryan AG, 2002, ENDOSCOPY, V34, P838, DOI 10.1055/s-2002-34262
Sadik R, 2011, WORLD J GASTROENTERO, V17, P499, DOI 10.3748/wjg.v17.i4.499
Sarkaria S, 2014, J CLIN GASTROENTEROL, V48, P145, DOI 10.1097/MCG.0b013e3182972219
Schwartz DA, 2002, GASTROINTEST ENDOSC, V56, P100, DOI 10.1067/mge.2002.125233
Seifert H, 2009, GUT, V58, P1260, DOI 10.1136/gut.2008.163733
Shah JN, 2012, GASTROINTEST ENDOSC, V75, P56, DOI 10.1016/j.gie.2011.08.032
Shah RJ, 2015, CLIN GASTROENTEROL H, V13, P747, DOI 10.1016/j.cgh.2014.09.047
Song TJ, 2014, GASTROINTEST ENDOSC, V80, P707, DOI 10.1016/j.gie.2014.05.327
Tessier G, 2007, GASTROINTEST ENDOSC, V65, P233, DOI 10.1016/j.gie.2006.06.029
Ushijima T, 2016, GASTROINTEST ENDOSC
Van Dam J., 1993, Cancer
Varadarajulu S, 2004, GASTROINTEST ENDOSC, V60, P631, DOI 10.1016/S0016-5107(04)01891-7
Varadarajulu S, 2013, GASTROENTEROLOGY, P145
Varadarajulu S, 2008, GASTROINTEST ENDOSC, V68, P1102, DOI 10.1016/j.gie.2008.04.028
Varadarajulu S, 2008, GASTROINTEST ENDOSC, V68, P656, DOI 10.1016/j.gie.2008.03.1091
Varadarajulu S, 2007, GASTROINTEST ENDOSC, V66, P1107, DOI 10.1016/j.gie.2007.03.1027
Varadarajulu S, 2011, J GASTROINTEST SURG, V15, P2080, DOI 10.1007/s11605-011-1621-8
Varadarajulu S, 2011, J GASTROEN HEPATOL, V26, P1504, DOI 10.1111/j.1440-1746.2011.06771.x
Voss M, 2000, GUT, V46, P244, DOI 10.1136/gut.46.2.244
Wallace MB, 2000, GASTROINTEST ENDOSC, V51, P309, DOI 10.1016/S0016-5107(00)70360-9
Walter D, 2015, ENDOSCOPY, V47, P63, DOI 10.1055/s-0034-1378113
Wang KX, 2011, GASTROINTEST ENDOSC, V73, P283, DOI 10.1016/j.gie.2010.10.045
Wiechowska-Kozlowska A, 2012, GASTROENTEROL RES PR, V2012
Wiersema MJ, 1996, GASTROINTEST ENDOSC, V43, P102, DOI 10.1016/S0016-5107(06)80108-2
Wildi SM, 2003, GASTROINTEST ENDOSC, V58, P362, DOI 10.1016/S0016-5107(03)00009-9
Will U, 2006, WORLD J GASTROENTERO, V12, P4175, DOI 10.3748/wjg.v12.i26.4175
Williams DB, 1999, GUT, V44, P720, DOI 10.1136/gut.44.5.720
Yasuda I, 2013, ENDOSCOPY, V45, P627, DOI 10.1055/s-0033-1344027
NR 109
TC 37
Z9 38
U1 0
U2 7
PU ELSEVIER SCI LTD
PI OXFORD
PA THE BOULEVARD, LANGFORD LANE, KIDLINGTON, OXFORD OX5 1GB, OXON, ENGLAND
SN 1521-6918
EI 1532-1916
J9 BEST PRACT RES CL GA
JI Best Pract. Res. Clin. Gastroenterol.
PD OCT

PY 2016
VL 30
IS 5
BP 807
EP 823
DI 10.1016/j.bpg.2016.10.008
PG 17
WC Gastroenterology & Hepatology
WE Science Citation Index Expanded (SCI-EXPANDED)
SC Gastroenterology & Hepatology
GA EG0MR
UT WOS:000390727400012
PM 27931638
DA 2026-05-09
ER

PT J
AU Jo, SJ
Moon, JH
Lee, YN
Park, JK
Lee, TH
Park, SH
Park, SI
Jeong, S
Lee, DH
AF Jo, Seok Jung
Moon, Jong Ho
Lee, Yun Nah
Park, Jae Keun
Lee, Tae Hoon
Park, Sang-Heum
Park, Se Ik
Jeong, Seok
Lee, Don Haeng
TI A novel bipolar electrocautery-enhanced delivery system with a
lumen-apposing metal stent for EUS-guided drainage: A porcine study
SO JOURNAL OF HEPATO-BILIARY-PANCREATIC SCIENCES
LA English
DT Article
DE endoscopic ultrasound; gallbladder; self-expandable metallic stents
ID PANCREATIC-FLUID COLLECTIONS; FEASIBILITY

AB Background An electrocautery-enhanced delivery system with a lumen-apposing metal stent (LAMS) is available for one-step endoscopic ultrasound-guided transmural drainage (EUS-TD). Bipolar electrosurgery has several potential clinical advantages, including reduced collateral thermal damage, enhanced hemostasis, and no requirement for a return electrode plate. In this study, we compared the technical feasibility and safety of a newly developed bipolar electrocautery-enhanced delivery system with a conventional delivery system for EUS-TD using a LAMS in a porcine model. Method Ten days before the study, 12 mini pigs underwent common bile duct ligation for EUS-guided gallbladder drainage. Transenteric puncture was performed, followed by placement of a guidewire. In six pigs, a bipolar electrocautery-enhanced delivery system with LAMS (Hot SPAXUS) was inserted over the guidewire and advanced into the gallbladder, without prior dilation of the tract, by applying a bipolar cut current. In the remaining six pigs, a conventional delivery system with LAMS (Cold SPAXUS) was inserted after tract dilatation using a cystotome. The stent was removed after 4 weeks. Results In all pigs, the stent was successfully inserted and deployed in the gallbladder without adverse events. The mean procedure time of EUS-TD was significantly lower in the Hot SPAXUS group than that of the Cold SPAXUS group (mean +/- standard deviation: 188.7 +/- 5.2 vs 449.5 +/- 97.5 s, P = .0019). Stent migration was not observed, and all stents were removed successfully. Conclusions Endoscopic ultrasound-guided transmural drainage using a bipolar electrocautery-enhanced LAMS is feasible for reducing the procedure time while maintaining the high success rate and safety of conventional LAMS.
C1 [Jo, Seok Jung; Moon, Jong Ho; Lee, Yun Nah; Park, Jae Keun] SoonChunHyang Univ, Digest Dis Ctr & Res Inst, Dept Internal Med, Sch Med, 170 Jomaru Ro, Bucheon 14584, South Korea.
[Lee, Tae Hoon; Park, Sang-Heum] SoonChunHyang Univ, Digest Dis Ctr & Res Inst, Dept Internal Med, Sch Med, Cheonan, South Korea.
[Park, Se Ik] Taewoong Med, Ilsan, South Korea.
[Jeong, Seok; Lee, Don Haeng] Inha Univ, Dept Internal Med, Sch Med, Incheon, South Korea.
C3 Soonchunhyang University; Soonchunhyang University; Inha University
RP Moon, JH (corresponding author), SoonChunHyang Univ, Digest Dis Ctr & Res Inst, Dept Internal Med, Sch Med, 170 Jomaru Ro, Bucheon 14584, South Korea.
EM jhmoon@schmc.ac.kr
RI Lee, Tae-Hoon/AAR-3708-2021; Lee, Yun/J-1260-2014; Jeong, Seok/C-9987-2011
OI Moon, Jong Ho/0000-0002-3946-9944;
FU SoonChunHyang University
FX SoonChunHyang University
CR Dollhopf M, 2017, GASTROINTEST ENDOSC, V86, P636, DOI 10.1016/j.gie.2017.02.027
Hookey LC, 2006, GASTROINTEST ENDOSC, V63, P635, DOI 10.1016/j.gie.2005.06.028
Jang JW, 2011, GASTROINTEST ENDOSC, V74, P176, DOI 10.1016/j.gie.2011.03.1120
Kunda R, 2016, SURG ENDOSC, V30, P5002, DOI 10.1007/s00464-016-4845-6
Lakhtakia Sundeep, 2018, VideoGIE, V3, P284, DOI [10.1016/j.vgie.2018.07.001, 10.1016/j.vgie.2018.07.001]
Moon JH, 2014, GASTROINTEST ENDOSC, V79, P990, DOI 10.1016/j.gie.2014.02.015
Rinninella E, 2015, GASTROINTEST ENDOSC, V82, P1039, DOI 10.1016/j.gie.2015.04.006
SAVIDES TJ, 1995, GASTROINTEST ENDOSC, V42, P573, DOI 10.1016/S0016-5107(95)70013-7
Song TJ, 2019, GASTROINTEST ENDOSC, V90, P507, DOI 10.1016/j.gie.2019.05.033
Sutton PA, 2010, BRIT J SURG, V97, P428, DOI 10.1002/bjs.6901
Talreja JP, 2008, GASTROINTEST ENDOSC, V68, P1199, DOI 10.1016/j.gie.2008.06.015

Walter D, 2015, ENDOSCOPY, V47, P63, DOI 10.1055/s-0034-1378113
NR 12
TC 5
Z9 6
U1 0
U2 3
PU WILEY
PI HOBOKEN
PA 111 RIVER ST, HOBOKEN 07030-5774, NJ USA
SN 1868-6974
EI 1868-6982
J9 J HEPATO-BIL-PAN SCI
JI J. Hepato-Biliary-Pancreat. Sci.
PD FEB
PY 2023
VL 30
IS 2
BP 263
EP 268
DI 10.1002/jhbp.1180
EA JUN 2022
PG 6
WC Gastroenterology & Hepatology; Surgery
WE Science Citation Index Expanded (SCI-EXPANDED)
SC Gastroenterology & Hepatology; Surgery
GA 8Z5RH
UT WOS:000806298600001
PM 35583204
DA 2026-05-09
ER

PT J
AU Ogura, T
Ohama, H
Higuchi, K
AF Ogura, Takeshi
Ohama, Hideko
Higuchi, Kazuhide
TI Endoscopic Ultrasound-Guided Pancreatic Transmural Stenting and
Transmural Intervention
SO CLINICAL ENDOSCOPY
LA English

DT Review

DE Endoscopic retrograde cholangiopancreatography; Endoscopic ultrasound;
Endoscopic ultrasound-guided pancreatic duct drainage; Pancreas;
Pancreatic duct stricture

ID DUCT DRAINAGE; EUS; PANCREATOGRAPHY; MULTICENTER; OUTCOMES; SAFETY

AB Endoscopic ultrasound (EUS)-guided pancreatic access is an emergent method that can be divided into the two main techniques of EUS-guided rendezvous and pancreatic transmural stenting (PTS). While many reports have described EUS-guided procedures, the indications, technical tips, clinical effects, and safety of EUS-guided pancreatic duct drainage (EUS-PD) remain controversial. This review describes the current status of and problems associated with EUS-PD, particularly PTS. We reviewed clinical data derived from a total of 334 patients. Rates of technical and clinical success ranged from 63% to 100% and 76% to 100%, respectively. In contrast, the rate of procedure-related adverse events was high at 26.7% (89/334). The most frequent adverse events comprised abdominal pain (n=38), acute pancreatitis (n=15), bleeding (n=9), and issues associated with pancreatic juice leakage such as perigastric fluid, pancreatic fluid collection, or pancreatic juice leaks (n=8). In conclusion, indications for EUS-PTS are limited, as is the evidence of its viability, due to the scarcity of expert operators. Despite improvements made to various devices, EUS-PTS remains technically challenging. Therefore, a long-term, large-scale, multicenter study is required to establish this technique as a

C1 [Ogura, Takeshi; Ohama, Hideko; Higuchi, Kazuhide] Osaka Med Coll, Dept Internal Med 2, 2-7 Daigakuchou, Takatsuki, Osaka 5698686, Japan.

C3 Osaka Medical & Pharmaceutical University

RP Ogura, T (corresponding author), Osaka Med Coll, Dept Internal Med 2, 2-7 Daigakuchou, Takatsuki, Osaka 5698686, Japan.

EM oguratakeshi0411@yahoo.co.jp

RI Ogura, Takeshi/AAU-5643-2021; Ohama, Hideko/HJH-2482-2023

CR Ali UA, 2015, COCHRANE DB SYST REV, DOI 10.1002/14651858.CD007884.pub3
Amano M, 2017, J GASTROEN HEPATOL, V32, P716, DOI 10.1111/jgh.13489
Bataille L, 2002, GASTROINTEST ENDOSC, V55, P740, DOI 10.1067/mge.2002.123621
Chen YI, 2018, GASTROINTEST ENDOSC, V88, P267, DOI 10.1016/j.gie.2018.03.021
Chen YI, 2017, GASTROINTEST ENDOSC, V85, P170, DOI 10.1016/j.gie.2016.07.031
François E, 2002, GASTROINTEST ENDOSC, V56, P128, DOI 10.1067/mge.2002.125547
Fujii LL, 2013, GASTROINTEST ENDOSC, V78, P854, DOI 10.1016/j.gie.2013.05.016
Fujii-Lau LL, 2015, J HEPATO-BIL-PAN SCI, V22, P51, DOI 10.1002/jhbp.187
HARADA N, 1995, ENDOSCOPY, V27, P612, DOI 10.1055/s-2007-1005769
Honjo M, 2018, ENDOSC ULTRASOUND, V7, P376, DOI 10.4103/eus.eus_2_18
Itoi T, 2017, DIGEST ENDOSC, V29, P495, DOI 10.1111/den.12794
Itoi T, 2013, DIGEST ENDOSC, V25, P241, DOI 10.1111/den.12048
James TW, 2018, ENDOSC INT OPEN, V6, pE735, DOI 10.1055/a-0607-2484
Kahaleh M, 2003, GASTROINTEST ENDOSC, V58, P919, DOI 10.1016/S0016-5107(03)02297-1
Krafft MR, 2019, DIGEST DIS SCI, V64, P1770, DOI 10.1007/s10620-019-05495-9
Kunda R, 2016, SURG ENDOSC, V30, P5002, DOI 10.1007/s00464-016-4845-6
Matsunami Y, 2018, ENDOSC INT OPEN, V6, pE505, DOI 10.1055/s-0044-101753
Mizandari M, 2017, CARDIOVASC INTER RAD, V40, P1911, DOI 10.1007/s00270-017-1727-9
Moon SH, 2010, GASTROINTEST ENDOSC, V72, P86, DOI 10.1016/j.gie.2010.01.063

Nakai Y, 2019, SAUDI J GASTROENTERO, V25, P210, DOI 10.4103/sjg.SJG_474_18
Ogura T, 2019, J HEPATO-BIL-PAN SCI, V26, pE3, DOI 10.1002/jhbp.609
Oh D, 2016, GASTROINTEST ENDOSC, V83, P366, DOI 10.1016/j.gie.2015.07.015
Tessier G, 2007, GASTROINTEST ENDOSC, V65, P233, DOI 10.1016/j.gie.2006.06.029
Tyberg A, 2017, GASTROINTEST ENDOSC, V85, P164, DOI 10.1016/j.gie.2016.07.030
Will U, 2015, WORLD J GASTROENTERO, V21, P13140, DOI 10.3748/wjg.v21.i46.13140
NR 25
TC 18
Z9 22
U1 0
U2 1
PU KOREAN SOC GASTROINTESTINAL ENDOSCOPY
PI SEOUL
PA 2003 LG PALACE, 165-8 DONGGYO-DONG, MAPO-GU, SEOUL, 121-754, SOUTH KOREA
SN 2234-2400
EI 2234-2443
J9 CLIN ENDOSC
JI Clin. Endosc.
PY 2020
VL 53
IS 4
BP 429
EP 435
DI 10.5946/ce.2019.130
PG 7
WC Gastroenterology & Hepatology
WE Emerging Sources Citation Index (ESCI)
SC Gastroenterology & Hepatology
GA MV9WT
UT WOS:000556699600010
PM 31771320
OA Green Submitted, gold
DA 2026-05-09
ER

PT J
AU Koutlas, NJ
Pawa, R
AF Koutlas, Nicholas J.
Pawa, Rishi
TI Cholecystocolonic fistula following endoscopic ultrasound-guided
gallbladder drainage for stump cholecystitis

SO CLINICAL JOURNAL OF GASTROENTEROLOGY
LA English
DT Article
DE Cholecystocolonic fistula; Endoscopic ultrasound-guided gallbladder
drainage; Lumen apposing metal stent; Cholecystitis
ID MANAGEMENT
AB Cholecystocolonic fistulas are a rare sequela of gallstone disease. Presenting symptoms are variable but a triad of chronic diarrhea, vitamin K malabsorption, and pneumobilia has been proposed. If untreated, recurrent biliary sepsis can occur with substantial morbidity and mortality. Definitive management is surgical although endoscopic treatment has been described in nonsurgical patients. We present a case of a cholecystocolonic fistula following transgastric endoscopic ultrasound-guided gallbladder drainage with a lumen-apposing metal stent for stump cholecystitis. The patient's presenting symptom was diarrhea. Upper endoscopy and cholecystoscopy 4 weeks following gallbladder drainage revealed a cholecystocolonic fistula. The cholecystogastric tract was closed through the scope clips. The patient had no episodes of cholangitis and had a patent biliary tree with a prior biliary sphincterotomy so clinical observation was chosen. Colonoscopy 1 month later confirmed the closure of the fistula and the patient had a resolution of diarrhea. Our case highlights a novel adverse event of endoscopic ultrasound-guided gallbladder drainage caused by direct pressure of the lumen apposing metal and double pigtail stents on an already inflamed gallbladder wall. Endoscopic therapies that aid in transcapillary biliary drainage are viable alternatives to surgery and can result in fistula closure.
C1 [Koutlas, Nicholas J.; Pawa, Rishi] Wake Forest Sch Med, Sect Gastroenterol, Dept Med, Med Ctr Blvd, Winston Salem, NC 27101 USA.
C3 Wake Forest University
RP Pawa, R (corresponding author), Wake Forest Sch Med, Sect Gastroenterol, Dept Med, Med Ctr Blvd, Winston Salem, NC 27101 USA.
EM rpawa@wakehealth.edu
OI Pawa, Rishi/0000-0001-6452-2359
CR Abbasi SUN, 2019, CUREUS J MED SCIENCE, V11, DOI 10.7759/cureus.4874
Angrisani L, 2001, SURG ENDOSC-ULTRAS, V15, P1038, DOI 10.1007/s004640000317
CAROLIBOSC FX, 1990, GASTROEN CLIN BIOL, V14, P767
Fujitani K, 2001, SURG TODAY, V31, P740, DOI 10.1007/s005950170083
GLENN F, 1981, SURG GYNECOL OBSTET, V153, P527
GOLDBERG RI, 1988, GASTROINTEST ENDOSC, V34, P55, DOI 10.1016/S0016-5107(88)71232-8
Kobayashi K, 2022, MEDICINE, V101, DOI 10.1097/MD.0000000000029680
Mori Y, 2018, J HEPATO-BIL-PAN SCI, V25, P87, DOI 10.1002/jhbp.504
Okidi R, 2021, CLIN CASE REP, V9, DOI 10.1002/ccr3.4510
Saumoy M, 2021, GASTROINTEST ENDOSC, V94, P671, DOI 10.1016/j.gie.2021.05.031
Savvidou S, 2009, WORLD J GASTROENTERO, V15, P4077, DOI 10.3748/wjg.15.4077
Wang WK, 2006, WORLD J GASTROENTERO, V12, P772, DOI 10.3748/wjg.v12.i5.772
Yokoe M, 2018, J HEPATO-BIL-PAN SCI, V25, P41, DOI 10.1002/jhbp.515
NR 13
TC 1
Z9 2
U1 0

U2 0
PU SPRINGER JAPAN KK
PI TOKYO
PA SHIROYAMA TRUST TOWER 5F, 4-3-1 TORANOMON, MINATO-KU, TOKYO, 105-6005,
JAPAN
SN 1865-7257
EI 1865-7265
J9 CLIN J GASTROENTEROL
JI Clin. J. Gastroenterol.
PD FEB
PY 2023
VL 16
IS 1
BP 116
EP 120
DI 10.1007/s12328-022-01726-1
EA OCT 2022
PG 5
WC Gastroenterology & Hepatology
WE Emerging Sources Citation Index (ESCI)
SC Gastroenterology & Hepatology
GA 8Q0HN
UT WOS:000876101500001
PM 36287350
DA 2026-05-09
ER

PT J
AU Teoh, AYB
Dhir, V
Kida, M
Yasuda, I
Jin, ZD
Seo, DW
Almadi, M
Ang, TL
Hara, K
Hilmi, I
Itoi, T
Lakhtakia, S
Matsuda, K
Pausawasdi, N

Puri, R
Tang, RS
Wang, HP
Yang, AM
Hawes, R
Varadarajulu, S
Yasuda, K
Ho, LKY
AF Teoh, Anthony Y. B.
Dhir, Vinay
Kida, Mitsuhiro
Yasuda, Ichiro
Jin, Zhen Dong
Seo, Dong Wan
Almadi, Majid
Ang, Tiing Leong
Hara, Kazuo
Hilmi, Ida
Itoi, Takao
Lakhtakia, Sundeep
Matsuda, Koji
Pausawasdi, Nonthalee
Puri, Rajesh
Tang, Raymond S.
Wang, Hsiu-Po
Yang, Ai Ming
Hawes, Robert
Varadarajulu, Shyam
Yasuda, Kenjiro
Ho, Lawrence Khek Yu
TI Consensus guidelines on the optimal management in interventional EUS
procedures: results from the Asian EUS group RAND/UCLA expert panel
SO GUT
LA English
DT Article
ID CELIAC PLEXUS NEUROLYSIS; PANCREATIC-FLUID COLLECTIONS; GUIDED BILIARY
DRAINAGE; ACUTE NECROTIZING PANCREATITIS; PROSPECTIVE-RANDOMIZED-TRIAL;
APPOSING METAL STENT; ENDOSCOPIC RETROGRADE PANCREATOGRAPHY; GASTRIC
OUTLET OBSTRUCTION; EARLY ANTIBIOTIC-TREATMENT; ENTEROSCOPY-ASSISTED
ERCP
AB Objectives Interventional endoscopic ultrasonography (EUS) procedures are gaining popularity and the most commonly performed procedures include EUS-guided drainage of pancreatic pseudocyst, EUS-guided biliary drainage, EUS-guided

pancreatic duct drainage and EUS-guided celiac plexus ablation. The aim of this paper is to formulate a set of practice guidelines addressing various aspects of the above procedures.

Methods Formulation of the guidelines was based on the best scientific evidence available. The RAND/UCLA appropriateness methodology (RAM) was used. Panellists recruited comprised experts in surgery, interventional EUS, interventional radiology and oncology from 11 countries. Between June 2014 and October 2016, the panellists met in meetings to discuss and vote on the clinical scenarios for each of the interventional EUS procedures in question.

Results A total of 15 statements on EUS-guided drainage of pancreatic pseudocyst, 15 statements on EUS-guided biliary drainage, 12 statements on EUS-guided pancreatic duct drainage and 14 statements on EUS-guided celiac plexus ablation were formulated. The statements addressed the indications for the procedures, technical aspects, pre- and post-procedural management, management of complications, and competency and training in the procedures. All statements except one were found to be appropriate. Randomised studies to address clinical questions in a number of aspects of the procedures are urgently required.

Conclusions The current guidelines on interventional EUS procedures are the first published by an endoscopic society. These guidelines provide an in-depth review of the current evidence and standardise the management of the procedures.
C1 [Teoh, Anthony Y. B.] Chinese Univ Hong Kong, Prince Wales Hosp, Dept Surg, Shatin, Hong Kong, Peoples R China.

[Dhir, Vinay] Baldota Inst Digest Sci, Mumbai, Maharashtra, India.

[Kida, Mitsuhiro] Kitasato Univ Hosp, Dept Gastroenterol, Sagamihara, Kanagawa, Japan.

[Yasuda, Ichiro] Teikyo Univ, Dept Gastroenterol, Mizonokuchi Hosp, Kawasaki, Kanagawa, Japan.

[Jin, Zhen Dong] Second Mil Med Univ, Changhai Hosp, Dept Gastroenterol, Shanghai, Peoples R China.

[Seo, Dong Wan] Asan Med Ctr, Dept Gastroenterol, Seoul, South Korea.

[Almadi, Majid] King Saud Univ, King Khalid Univ Hosp, Riyadh, Saudi Arabia.

[Ang, Tiing Leong] Changi Gen Hosp, Dept Gastroenterol & Hepatol, Singapore, Singapore.

[Hara, Kazuo] Aichi Canc Ctr Hosp, Dept Gastroenterol, Nagoya, Aichi, Japan.

[Hilmi, Ida] Univ Malaya, Dept Med, Kuala Lumpur, Malaysia.

[Itoi, Takao] Tokyo Med Univ, Dept Gastroenterol, Tokyo, Japan.

[Lakhtakia, Sundeep] Asian Inst Gastroenterol, Dept Gastroenterol, Hyderabad, Telangana, India.

[Matsuda, Koji] St Marianna Univ, Yokohama City Seibu Hosp, Sch Med, Kawasaki, Kanagawa, Japan.

[Pausawasdi, Nonthalee] Mahidol Univ, Siriraj Hosp, Fac Med, Dept Internal Med, Bangkok, Thailand.

[Puri, Rajesh] The Medicity, Inst Digest & Hepatobiliary Sci Medanta, Dept Gastroenterol, Gurgaon, India.

[Tang, Raymond S.] Chinese Univ Hong Kong, Prince Wales Hosp, Inst Digest Dis, Hong Kong, Hong Kong, Peoples R China.

[Wang, Hsiu-Po] Natl Taiwan Univ, Coll Med & Hosp, Dept Internal Med, Taipei, Taiwan.

[Yang, Ai Ming] Chinese Acad Med Sci, Peking Union Med Coll, Peking Union Med Coll Hosp, Dept Gastroenterol, Beijing, Peoples R China.

[Hawes, Robert; Varadarajulu, Shyam] Florida Hosp, Ctr Intervent Endoscopy, Orlando, FL USA.

[Yasuda, Kenjiro] Kyoto Second Red Cross Hosp, Dept Gastroenterol, Kyoto, Japan.

[Ho, Lawrence Khek Yu] Natl Univ Singapore, Dept Med, Singapore, Singapore.

C3 Prince of Wales Hospital Hong Kong; Chinese University of Hong Kong;
Kitasato University; Teikyo University; Naval Medical University;
University of Ulsan; Asan Medical Center; King Saud University; King
Khalid University Hospital; Changi General Hospital; Aichi Cancer
Center; Universiti Malaya; Tokyo Medical University; St Marianna

University; Mahidol University; Prince of Wales Hospital Hong Kong;
Chinese University of Hong Kong; National Taiwan University; Chinese
Academy of Medical Sciences - Peking Union Medical College; Peking Union
Medical College; Peking Union Medical College Hospital; Adventist Health
Services; AdventHealth; National University of Singapore
RP Teoh, AYB (corresponding author), Chinese Univ Hong Kong, Prince Wales Hosp, Dept Surg, Shatin, Hong Kong, Peoples R China.
EM anthonyteoh@surgery.cuhk.edu.hk
RI Almadi, Majid/KIA-2074-2024; Hilmi, Ida/AAN-7129-2020; Yasuda,
Ichiro/IUP-0131-2023; Teoh, Anthony/L-4796-2016; Lakhtakia,
Sundeep/H-3607-2019; Tang, Raymond Shing-Yan/AED-4572-2022
OI Almadi, Majid/0000-0001-9917-2758; Hilmi, Ida/0000-0001-7091-0032;
Yasuda, Ichiro/0000-0002-6888-0310; Yang, Aiming/0000-0002-2907-7904;
jin, zhendong/0000-0002-9358-7351; Tang, Raymond
Shing-Yan/0000-0002-2433-8036
CR ADAMS DB, 1993, J SURG RES, V54, P150, DOI 10.1006/jsre.1993.1024
ADAMS DB, 1992, ANN SURG, V215, P571, DOI 10.1097/00000658-199206000-00003
Ahmed HM, 2009, ENDOSCOPY, V41, pE218, DOI 10.1055/s-0029-1214941
Alvarez-Sanchez MV, 2014, SURG ENDOSC, V28, P712, DOI 10.1007/s00464-013-3260-5
Andriulli A, 2007, AM J GASTROENTEROL, V102, P1781, DOI 10.1111/j.1572-0241.2007.01279.x
Ang TL, 2017, DIGEST ENDOSC, V29, P463, DOI 10.1111/den.12797
[Anonymous], EIO
[Anonymous], J SUPPORT ONCOL
[Anonymous], GASTROENTEROL RES PR
Arcidiacono PG, 2011, COCHRANE DB SYST REV, DOI 10.1002/14651858.CD007519.pub2
Artifon ELA, 2015, ENDOSC ULTRASOUND, V4, P235, DOI 10.4103/2303-9027.163010
Artifon ELA, 2015, GASTROINTEST ENDOSC, V81, P950, DOI 10.1016/j.gie.2014.09.047
Artifon ELA, 2012, J CLIN GASTROENTEROL, V46, P768, DOI 10.1097/MCG.0b013e31825f264c
Arvanitakis M, 2007, GASTROINTEST ENDOSC, V65, P609, DOI 10.1016/j.gie.2006.06.083
Ascunce G, 2011, GASTROINTEST ENDOSC, V73, P267, DOI 10.1016/j.gie.2010.10.029
Attili F, 2015, ENDOSCOPY, V47, pE619, DOI 10.1055/s-0034-1393669
Bang JY, 2017, GUT, V66, P2054, DOI 10.1136/gutjnl-2016-312812
Bang JY, 2015, DIGEST ENDOSC, V27, P486, DOI 10.1111/den.12418
Bang JY, 2014, SURG ENDOSC, V28, P2877, DOI 10.1007/s00464-014-3541-7
Banks PA, 2013, GUT, V62, P102, DOI 10.1136/gutjnl-2012-302779
Baron TH, 2015, ENDOSC ULTRASOUND, V4, P34, DOI 10.4103/2303-9027.151326
Berzosa M, 2012, ENDOSCOPY, V44, P543, DOI 10.1055/s-0031-1291710
Bhasin DK, 2006, AM J GASTROENTEROL, V101, P1780, DOI 10.1111/j.1572-0241.2006.00644.x
Bhasin DK, 2010, SURG ENDOSC, V24, P1085, DOI 10.1007/s00464-009-0732-8
BOENDER J, 1995, AM J GASTROENTEROL, V90, P233
Bracher GA, 1999, GASTROINTEST ENDOSC, V49, P710, DOI 10.1016/S0016-5107(99)70287-7
BRADLEY EL, 1979, AM J SURG, V137, P135, DOI 10.1016/0002-9610(79)90024-2

Brand M, 2010, COCHRANE DB SYST REV, DOI 10.1002/14651858.CD007345.pub2
Brennan PM, 2006, DIGEST SURG, V23, P250, DOI 10.1159/000095398
Broder MS, 2000, OBSTET GYNECOL, V95, P199, DOI 10.1016/S0029-7844(99)00519-0
Cahen D, 2005, ENDOSCOPY, V37, P977, DOI 10.1055/s-2005-870336
Caraceni A, 1996, CANCER-AM CANCER SOC, V78, P639
Catenacci DVT, 2015, GASTROENTEROLOGY, V149, P1794, DOI 10.1053/j.gastro.2015.08.050
Chan Shannon Melissa, 2015, Curr Treat Options Gastroenterol, V13, P171, DOI [10.1007/s11938-015-0047-x, 10.1007/s11938-015-0047-x]
Chapman CG, 2016, CLIN ENDOSC, V49, P161, DOI 10.5946/ce.2016.011
Chen YI, 2017, GASTROINTEST ENDOSC, V85, P170, DOI 10.1016/j.gie.2016.07.031
Cui ML, 2014, DIGEST DIS SCI, V59, P1055, DOI 10.1007/s10620-013-2967-4
Delcenserie R, 1996, PANCREAS, V13, P198
Dellinger EP, 2007, ANN SURG, V245, P674, DOI 10.1097/01.sla.0000250414.09255.84
Dhir V, 2015, GASTROINTEST ENDOSC, V82, P718
Dhir V, 2017, DIGEST ENDOSC, V29, P472, DOI 10.1111/den.12818
Dhir Vinay, 2011, J Interv Gastroenterol, V1, P153
Dhir V, 2015, GASTROINTEST ENDOSC, V82, P650, DOI 10.1016/j.gie.2015.01.061
Dhir V, 2015, GASTROINTEST ENDOSC, V81, P440, DOI 10.1016/j.gie.2014.09.011
Diculescu Mircea, 2005, Rom J Gastroenterol, V14, P129
Dixon M, 2013, ANN SURG ONCOL, V20, P533, DOI 10.1245/s10434-012-2584-z
Doi S, 2013, ENDOSCOPY, V45, P362, DOI 10.1055/s-0032-1326225
Fabbri C, 2012, ENDOSCOPY, V44, P429, DOI 10.1055/s-0031-1291624
Fitch K., 2001, The RAND/UCLA Appropriateness Method User's Manual
François E, 2002, GASTROINTEST ENDOSC, V56, P128, DOI 10.1067/mge.2002.125547
Freeman ML, 2003, GASTROINTEST ENDOSC, V58, P41, DOI 10.1067/mge.2003.292
Fujii L, 2012, ENDOSCOPY, V44, pE265, DOI 10.1055/s-0032-1309708
Fujii LL, 2013, GASTROINTEST ENDOSC, V78, P854, DOI 10.1016/j.gie.2013.05.016
Fujii-Lau LL, 2015, J HEPATO-BIL-PAN SCI, V22, P51, DOI 10.1002/jhbp.187
Fusaroli P, 2016, ULTRASCHALL MED, V37, P408, DOI 10.1055/s-0035-1553742
Gimeno-García AZ, 2012, ENDOSCOPY, V44, pE267, DOI 10.1055/s-0032-1309709
Goodman KA, 2016, PRACT RADIAT ONCOL, V6, P166, DOI 10.1016/j.prro.2015.11.014
Gore ME, 2012, EUR J CANCER, V48, P1038, DOI 10.1016/j.ejca.2012.02.058
Gornals JB, 2013, SURG ENDOSC, V27, P1428, DOI 10.1007/s00464-012-2591-y
Gress F, 1999, AM J GASTROENTEROL, V94, P900
Gress F, 2001, AM J GASTROENTEROL, V96, P409
GRESS F, 1997, GASTROINTEST ENDOSC, V45, pAB173
GRIMM H, 1992, GASTROINTEST ENDOSC, V38, P170, DOI 10.1016/S0016-5107(92)70384-8
Gunaratnam NT, 2001, GASTROINTEST ENDOSC, V54, P316, DOI 10.1067/mge.2001.117515
Gupta K, 2014, J CLIN GASTROENTEROL, V48, P80, DOI 10.1097/MCG.0b013e31828c6822
Gupta Ruchi, 2007, MedGenMed, V9, P8
Gurusamy KS, 2016, COCHRANE DB SYST REV, V4, DOI DOI 10.1002/14651858.CD011392.PUB2
Guyatt GH, 2011, J CLIN EPIDEMIOL, V64, P380, DOI 10.1016/j.jclinepi.2010.09.011

Harada N, 1997, Gastrointest Endosc Clin N Am, V7, P237
Harewood GC, 2003, GASTROINTEST ENDOSC, V58, P230, DOI 10.1067/mge.2003.346
Hawes RH, 2009, GASTROINTEST ENDOSC, V69, pS1, DOI 10.1016/j.gie.2008.11.013
Heider R, 1999, ANN SURG, V229, P781, DOI 10.1097/00000658-199906000-00004
Helton S, 2001, J GASTROINTEST SURG, V5, P118
Hemingway H, 2001, HEART, V85, P672, DOI 10.1136/heart.85.6.672
HIGUCHI S, 1987, LANCET, V1, P629
Inamdar S, 2015, GASTROINTEST ENDOSC, V82, P9, DOI 10.1016/j.gie.2015.02.013
Irani S, 2017, CLIN GASTROENTEROL H, V15, P738, DOI 10.1016/j.cgh.2016.12.021
Isenmann R, 2004, GASTROENTEROLOGY, V126, P997, DOI 10.1053/j.gastro.2003.12.050
Ishiwatari H, 2015, GASTROINTEST ENDOSC, V81, P479, DOI 10.1016/j.gie.2014.10.031
Ishiwatari H, 2014, WORLD J GASTROENTERO, V20, P10512, DOI 10.3748/wjg.v20.i30.10512
Itoi T, 2016, GUT, V65, P193, DOI 10.1136/gutjnl-2015-310348
Itoi T, 2015, ENDOSCOPY, V47, P462, DOI 10.1055/s-0034-1391083
Itoi T, 2013, DIGEST ENDOSC, V25, P241, DOI 10.1111/den.12048
Itoi T, 2012, GASTROINTEST ENDOSC, V75, P870, DOI 10.1016/j.gie.2011.10.020
Itoi T, 2009, DIGEST ENDOSC, V21, pS53, DOI 10.1111/j.1443-1661.2009.00851.x
Iwata K, 2011, DIGEST ENDOSC, V23, P140, DOI 10.1111/j.1443-1661.2010.01046.x
Jang HY, 2013, CLIN ENDOSC, V46, P306, DOI 10.5946/ce.2013.46.3.306
Jang JW, 2016, PANCREATOLOGY, V16, P958, DOI 10.1016/j.pan.2016.09.009
Kahaleh M, 2006, ENDOSCOPY, V38, P355, DOI 10.1055/s-2006-925249
Kahaleh M, 2003, GASTROINTEST ENDOSC, V58, P919, DOI 10.1016/S0016-5107(03)02297-1
Kahaleh M, 2013, WORLD J GASTROENTERO, V19, P1372, DOI 10.3748/wjg.v19.i9.1372
KAHAN JP, 1994, MED CARE, V32, P357, DOI 10.1097/00005650-199404000-00004
Kaufman M, 2010, J CLIN GASTROENTEROL, V44, P127, DOI 10.1097/MCG.0b013e3181bb854d
Khan MA, 2016, DIGEST DIS SCI, V61, P684, DOI 10.1007/s10620-015-3933-0
Khashab MA, 2016, ENDOSC INT OPEN, V4, pE175, DOI 10.1055/s-0041-109083
Khashab MA, 2015, GASTROINTEST ENDOSC, V82, P745, DOI 10.1016/j.gie.2015.05.017
KOURTESIS G, 1990, AM SURGEON, V56, P796
KOZAREK RA, 1991, GASTROENTEROLOGY, V100, P1362
Kumbhari V, 2016, DIGEST DIS SCI, V61, P3552, DOI 10.1007/s10620-016-4327-7
Kunda R, 2016, SURG ENDOSC, V30, P5002, DOI 10.1007/s00464-016-4845-6
Lakhtakia S, 2017, GASTROINTEST ENDOSC, V85, P1243, DOI 10.1016/j.gie.2016.10.037
Lakhtakia S, 2016, GASTROINTEST ENDOSC, V83, P234, DOI 10.1016/j.gie.2015.08.085
Lalueza A, 2011, ENDOSCOPY, V43, pE9, DOI 10.1055/s-0030-1255804
LANG EK, 1991, SOUTHERN MED J, V84, P55, DOI 10.1097/00007611-199101000-00014
Lau ST, 2001, AM J SURG, V181, P411, DOI 10.1016/S0002-9610(01)00606-7
Lavu H, 2015, J AM COLL SURGEONS, V220, P497, DOI 10.1016/j.jamcollsurg.2014.12.013
Leblanc JK, 2013, DIAGN THER ENDOSC, V2013, P1
LeBlanc JK, 2011, GASTROINTEST ENDOSC, V74, P1300, DOI 10.1016/j.gie.2011.07.073
Lee TH, 2016, CLIN GASTROENTEROL H, V14, P1011, DOI 10.1016/j.cgh.2015.12.032
Lee TH, 2014, WORLD J GASTROENTERO, V20, P5859, DOI 10.3748/wjg.v20.i19.5859

Levy M, 2006, AM J GASTROENTEROL, V101, P1787, DOI 10.1111/j.1572-0241.2006.00685.x
Levy MJ, 2008, AM J GASTROENTEROL, V103, P98, DOI 10.1111/j.1572-0241.2007.01607.x
Loeve US, 2013, GASTROINTEST ENDOSC, V77, P151, DOI 10.1016/j.gie.2012.03.005
LONDON NJM, 1989, GUT, V30, P397, DOI 10.1136/gut.30.3.397
Mahajan RJ, 2002, GASTROINTEST ENDOSC, V55, pAB101
Maule WF, 1993, PANCREAS BIOL PATHOB
Melman L, 2009, SURG ENDOSC, V23, P267, DOI 10.1007/s00464-008-0196-2
Minaga K, 2017, GASTROINTEST ENDOSC, V85, P263, DOI 10.1016/j.gie.2016.03.016
Moon JH, 2014, GASTROINTEST ENDOSC, V79, P990, DOI 10.1016/j.gie.2014.02.015
Morgan DE, 1997, RADIOLOGY, V203, P773, DOI 10.1148/radiology.203.3.9169703
Morton JM, 2005, J GASTROINTEST SURG, V9, P15, DOI 10.1016/j.gassur.2004.10.005
Nobre Moura Renata, 2015, Rev. gastroenterol. Perú, V35, P333
Muscatiello N, 2006, ENDOSCOPY, V38, P858, DOI 10.1055/s-2006-925382
Nagels W, 2013, PAIN MED, V14, P1140, DOI 10.1111/pme.12176
NEOPTOLEMOS JP, 1993, BRIT J SURG, V80, P94, DOI 10.1002/bjs.1800800131
Nordback I, 2001, J GASTROINTEST SURG, V5, P113, DOI 10.1016/S1091-255X(01)80021-4
O'Toole TM, 2009, ENDOSCOPY, V41, P593, DOI 10.1055/s-0029-1214868
Ogura T, 2015, ENDOSCOPY, V47, P72, DOI 10.1055/s-0034-1378111
Oh D, 2016, GASTROINTEST ENDOSC, V83, P366, DOI 10.1016/j.gie.2015.07.015
Park DH, 2009, ENDOSCOPY, V41, P842, DOI 10.1055/s-0029-1215133
Park DH, 2015, J GASTROEN HEPATOL, V30, P1461, DOI 10.1111/jgh.13027
Park DH, 2011, GASTROINTEST ENDOSC, V74, P1276, DOI 10.1016/j.gie.2011.07.054
Parodi A, 2010, GASTROINTEST ENDOSC, V72, P881, DOI 10.1016/j.gie.2010.04.006
PEDERZOLI P, 1993, SURG GYNECOL OBSTET, V176, P480
Penn DE, 2012, GASTROINTEST ENDOSC, V76, P679, DOI 10.1016/j.gie.2012.04.457
Puli SR, 2009, DIGEST DIS SCI, V54, P2330, DOI 10.1007/s10620-008-0651-x
Puri R, 2012, J GASTROEN HEPATOL, V27, P722, DOI 10.1111/j.1440-1746.2012.07089.x
Rana SS, 2010, J GASTROEN HEPATOL, V25, P1087, DOI 10.1111/j.1440-1746.2009.06172.x
Rinninella E, 2015, GASTROINTEST ENDOSC, V82, P1039, DOI 10.1016/j.gie.2015.04.006
Rudmik L, 2016, INT FORUM ALLERGY RH, V6, P557, DOI 10.1002/alr.21769
Sahai AV, 2009, AM J GASTROENTEROL, V104, P326, DOI 10.1038/ajg.2008.64
SAINIO V, 1995, LANCET, V346, P663, DOI 10.1016/S0140-6736(95)92280-6
Sakamoto H., 2006, DIGEST ENDOSC, V18, P206
Sakamoto H, 2010, AM J GASTROENTEROL, V105, P2599, DOI 10.1038/ajg.2010.339
Sauer B, 2010, GASTROINTEST ENDOSC, V71, P432, DOI 10.1016/j.gie.2009.05.026
Schreiner MA, 2012, GASTROINTEST ENDOSC, V75, P748, DOI 10.1016/j.gie.2011.11.019
Seicean A, 2013, J GASTROINTEST LIVER, V22, P59
Seicean A, 2011, J GASTROINTEST LIVER, V20, P39
Shah RJ, 2013, GASTROINTEST ENDOSC, V77, P593, DOI 10.1016/j.gie.2012.10.015
Sharaiha RZ, 2016, J CLIN GASTROENTEROL, V50, P388, DOI 10.1097/MCG.0000000000000336
Shekelle PG, 1998, INT J TECHNOL ASSESS, V14, P707, DOI 10.1017/S0266462300012022
Shekelle PG, 2001, J CLIN EPIDEMIOL, V54, P1004, DOI 10.1016/S0895-4356(01)00365-1

Shimatani M, 2016, AM J GASTROENTEROL, V111, P1750, DOI 10.1038/ajg.2016.420
Siddiqui AA, 2016, GASTROINTEST ENDOSC, V83, P699, DOI 10.1016/j.gie.2015.10.020
Siddiqui AA, 2013, GASTROINTEST ENDOSC, V78, P589, DOI 10.1016/j.gie.2013.03.1337
Song TJ, 2016, GASTROINTEST ENDOSC, V83, P440, DOI 10.1016/j.gie.2015.08.048
Talreja JP, 2008, GASTROINTEST ENDOSC, V68, P1199, DOI 10.1016/j.gie.2008.06.015
Tanaka T, 2013, PANCREATOLOGY, V13, P621, DOI 10.1016/j.pan.2013.08.006
Telford JJ, 2002, GASTROINTEST ENDOSC, V56, P18, DOI 10.1067/mge.2002.125107
Téllez-Avila FI, 2013, ENDOSC ULTRASOUND, V2, P153, DOI [10.4103/2303-9027.117664, 10.7178/eus.06.007]
Teoh AY, 2016, GASTROINTEST ENDOSC, V84, P62, DOI 10.1016/j.gie.2015.11.041
Teoh AYB, 2017, ENDOSCOPY, V49, P130, DOI 10.1055/s-0042-119036
Teoh AYB, 2016, WORLD J GASTRO ENDOS, V8, P310, DOI 10.4253/wjge.v8.i6.310
Teoh AYB, 2015, ENDOSC INT OPEN, V3, pE130, DOI 10.1055/s-0034-1390890
Teoh AYB, 2014, GASTROINTEST ENDOSC, V80, P1171, DOI 10.1016/j.gie.2014.03.038
Tessier G, 2007, GASTROINTEST ENDOSC, V65, P233, DOI 10.1016/j.gie.2006.06.029
Topa L, 2006, GASTROINTEST ENDOSC, V64, P460, DOI 10.1016/j.gie.2006.04.009
Tran QN, 2006, J SUPPORT ONCOL, V4, P463
Tran Quy N H, 2006, J Support Oncol, V4, P460
Trevino JM, 2010, J GASTROEN HEPATOL, V25, P526, DOI 10.1111/j.1440-1746.2009.06109.x
Tyberg A, 2018, J CLIN GASTROENTEROL, V52, P79, DOI 10.1097/MCG.0000000000000786
Tyberg A, 2017, GASTROINTEST ENDOSC, V85, P164, DOI 10.1016/j.gie.2016.07.030
Varadarajulu S, 2005, GASTROINTEST ENDOSC, V61, P568, DOI 10.1016/S0016-5107(04)02832-9
Varadarajulu S, 2008, GASTROINTEST ENDOSC, V68, P1102, DOI 10.1016/j.gie.2008.04.028
Varadarajulu S, 2008, GASTROINTEST ENDOSC, V68, P656, DOI 10.1016/j.gie.2008.03.1091
Varadarajulu S, 2008, GASTROINTEST ENDOSC, V68, P649, DOI 10.1016/j.gie.2008.02.057
Varadarajulu S, 2013, GASTROENTEROLOGY, V145, P583, DOI 10.1053/j.gastro.2013.05.046
Vazquez-Sequeiros E, 2016, GASTROINTEST ENDOSC, V84, P450, DOI 10.1016/j.gie.2016.02.044
Villatoro E, 2010, COCHRANE DB SYST REV, DOI 10.1002/14651858.CD002941.pub3
Wang KX, 2016, GASTROINTEST ENDOSC, V83, P1218, DOI 10.1016/j.gie.2015.10.033
Wang MH, 2015, DIGEST ENDOSC, V27, P687, DOI 10.1111/den.12458
Weilert F, 2012, ENDOSCOPY, V44, P780, DOI 10.1055/s-0032-1309839
Wiersema MJ, 1996, GASTROINTEST ENDOSC, V44, P656, DOI 10.1016/S0016-5107(96)70047-0
Will U, 2005, ENDOSCOPY, V37, P171, DOI 10.1055/s-2004-826151
Wyse JM, 2011, J CLIN ONCOL, V29, P3541, DOI 10.1200/JCO.2010.32.2750
Wyse JM, 2009, GASTROINTEST ENDOSC, V69, pAB326, DOI 10.1016/j.gie.2009.03.935
Yane K, 2017, ENDOSCOPY, V49, P69, DOI 10.1055/s-0042-118301
Yasuda I, 2017, DIGEST ENDOSC, V29, P455, DOI 10.1111/den.12824
YEO CJ, 1990, SURG GYNECOL OBSTET, V170, P411
Yuan HC, 2015, PANCREAS, V44, P570, DOI 10.1097/MPA.0000000000000307
Zhong W, 2014, PAIN PRACT, V14, P43, DOI 10.1111/papr.12083
NR 191
TC 167
Z9 195

U1 0
U2 15
PU BMJ PUBLISHING GROUP
PI LONDON
PA BRITISH MED ASSOC HOUSE, TAVISTOCK SQUARE, LONDON WC1H 9JR, ENGLAND
SN 0017-5749
EI 1468-3288
J9 GUT
JI Gut
PD JUL
PY 2018
VL 67
IS 7
BP 1209
EP 1228
DI 10.1136/gutjnl-2017-314341
PG 20
WC Gastroenterology & Hepatology
WE Science Citation Index Expanded (SCI-EXPANDED)
SC Gastroenterology & Hepatology
GA GM3VI
UT WOS:000438038300004
PM 29463614
OA Bronze
DA 2026-05-09
ER

PT J
AU Simons-Linares, CR
   Chahal, P
AF Simons-Linares, Carlos Roberto
   Chahal, Prabhleen
TI Advances in Interventional Endoscopic Ultrasound (EUS) A Technical
   Review
SO JOURNAL OF CLINICAL GASTROENTEROLOGY
LA English
DT Review
DE endoscopic ultrasound; fine needle aspiration; lumen-apposing metal
   stent; interventional EUS; gallbladder drainage; EUS-guided pancreatic
   fluid collection drainage; EUS-guided gastroenterostomy
ID FINE-NEEDLE-ASPIRATION; GUIDED LIVER-BIOPSY; APPOSING METAL STENT;
   TRANSHEPATIC GALLBLADDER DRAINAGE; TRANSMURAL DRAINAGE; DIAGNOSTIC

YIELD; CORE NEEDLE; BILIARY; MANAGEMENT; GASTROENTEROSTOMY

AB Endoscopic ultrasound (EUS) has become the therapeutic intervention of choice for multiple diseases and continues to evolve rapidly. Its increasing use has allowed the development and adaptation of multiple, revolutionary devices and tools. Currently, there is paucity of randomized clinical trials evaluating multiple EUS-guided interventions and the vast majority of published data is heterogenous. However, the available literature on EUS-guided therapeutic interventions continues to expand and demonstrate its safety, efficacy and cost effectiveness in carefully selected patients when performed by expert endosonographers. The future of interventional EUS appears to be bright!

C1 [Simons-Linares, Carlos Roberto; Chahal, Prabhleen] Cleveland Clin Fdn, Digest Dis & Surg Inst, Dept Gastroenterol & Hepatol, 9500 Euclid Ave, Cleveland, OH 44195 USA.

C3 Cleveland Clinic Foundation

RP Chahal, P (corresponding author), 9500 Euclid Ave, Cleveland, OH 44195 USA.

EM chahalp@ccf.org

RI Simons-Linares, C./O-6069-2018

CR Abdelfatah MM, 2018, GASTROINTEST ENDOSC, V87, P495, DOI 10.1016/j.gie.2017.08.033

Akahoshi K, 2014, J GASTROINTEST LIVER, V23, P405, DOI 10.15403/jgld.2014.1121.234.eug

Akbar A, 2012, GASTROINTEST ENDOSC, V76, P1060, DOI 10.1016/j.gie.2012.07.014

Arena M, 2017, MINERVA MED, V108, P547, DOI 10.23736/S0026-4806.17.05327-7

Arora Zubin, 2017, VideoGIE, V2, P6, DOI [10.1016/j.vgie.2016.10.004, 10.1016/j.vgie.2016.10.004]

Artifon ELA, 2015, ENDOSC ULTRASOUND, V4, P235, DOI 10.4103/2303-9027.163010

Bang JY, 2019, GUT, V68, P1200, DOI 10.1136/gutjnl-2017-315335

Bang JY, 2018, GASTROINTEST ENDOSC, V88, P9, DOI 10.1016/j.gie.2018.03.012

Baron TH, 2015, NEW ENGL J MED, V373, P357, DOI 10.1056/NEJMra1411372

Baron TH, 2014, CURR OPIN GASTROEN, V30, P453, DOI 10.1097/MOG.0000000000000100

Baron TH, 2014, SURG CLIN N AM, V94, P395, DOI 10.1016/j.suc.2013.12.005

Bhattacharya A, 2018, ABDOM RADIOL, V43, P364, DOI 10.1007/s00261-017-1347-5

Binmoeller KF, 2012, ENDOSCOPY, V44, P499, DOI 10.1055/s-0032-1309382

Brimhall B, 2018, CLIN GASTROENTEROL H, V16, P1521, DOI 10.1016/j.cgh.2018.02.021

Bukhari M, 2018, GASTROINTEST ENDOSC, V88, P486, DOI 10.1016/j.gie.2018.04.2356

Catenacci DVT, 2015, GASTROENTEROLOGY, V149, P1794, DOI 10.1053/j.gastro.2015.08.050

Chan JHY, 2018, ENDOSC ULTRASOUND, V7, P85, DOI 10.4103/eus.eus_7_18

Chapman CG, 2018, ENDOSC ULTRASOUND, V7, P240, DOI 10.4103/eus.eus_28_18

Chen YI, 2018, ENDOSC INT OPEN, V6, pE780, DOI 10.1055/a-0611-5082

Chen YI, 2018, ENDOSC INT OPEN, V6, pE363, DOI 10.1055/s-0043-123468

Chen YI, 2017, SURG ENDOSC, V31, P2946, DOI 10.1007/s00464-016-5311-1

Choi JH, 2015, ENDOSCOPY, V47, P1035, DOI 10.1055/s-0034-1392150

Coronel E, 2018, ENDOSCOPY, V50, P285, DOI 10.1055/s-0043-122597

de la Serna-Higuera C, 2013, GASTROINTEST ENDOSC, V77, P303, DOI 10.1016/j.gie.2012.09.021

Debol SM, 2003, DIAGN CYTOPATHOL, V28, P316, DOI 10.1002/dc.10294

DeWitt J, 2009, GASTROINTEST ENDOSC, V69, P535, DOI 10.1016/j.gie.2008.09.056

Diehl DL, 2015, ENDOSC INT OPEN, V3, pE210, DOI 10.1055/s-0034-1391412

DIMAGNO EP, 1980, LANCET, V1, P629

Dollhopf M, 2017, GASTROINTEST ENDOSC, V86, P636, DOI 10.1016/j.gie.2017.02.027

Ergun M, 2011, ENDOSCOPY, V43, P518, DOI 10.1055/s-0030-1256333
Facciorusso A, 2019, GASTROINTEST ENDOSC, V90, P893, DOI 10.1016/j.gie.2019.07.009
Faigel D, 2014, Endosc Ultrasound, V3, pS1
Faigel DO, 2016, GASTROINTEST ENDOSC, V83, P444, DOI 10.1016/j.gie.2015.08.064
Franco MC, 2017, ENDOSCOPY, V49, pE177, DOI 10.1055/s-0043-109425
Fujii LL, 2013, GASTROINTEST ENDOSC, V78, P854, DOI 10.1016/j.gie.2013.05.016
Fujii-Lau LL, 2014, ENDOSCOPY, V46, pE654, DOI 10.1055/s-0034-1390845
Fujii-Lau LL, 2015, J HEPATO-BIL-PAN SCI, V22, P51, DOI 10.1002/jhbp.187
Giday SA, 2008, GASTROINTEST ENDOSC, V67, P338, DOI 10.1016/j.gie.2007.08.037
Giday SA, 2007, GASTROINTEST ENDOSC, V66, P814, DOI 10.1016/j.gie.2007.05.056
Giovannini M, 2001, ENDOSCOPY, V33, P898, DOI 10.1055/s-2001-17324
Goldberg SN, 1999, GASTROINTEST ENDOSC, V50, P392, DOI 10.1053/ge.1999.v50.98847
Gor N, 2014, GASTROINTEST ENDOSC, V79, P170, DOI 10.1016/j.gie.2013.06.031
Gress F, 2000, AM J GASTROENTEROL, V95, P961
Guedes HG, 2018, CLINICS, V73, DOI 10.6061/clinics/2018/e261
Huang JY, 2017, GASTROINTEST ENDOSC, V85, P996, DOI 10.1016/j.gie.2016.09.026
Irani S, 2017, CURR OPIN GASTROEN, V33, P320, DOI 10.1097/MOG.0000000000000389
Irani S, 2017, GASTROINTEST ENDOSC, V85, P1285, DOI 10.1016/j.gie.2016.08.028
Irani S, 2017, CLIN GASTROENTEROL H, V15, P738, DOI 10.1016/j.cgh.2016.12.021
Irani S, 2015, GASTROINTEST ENDOSC, V82, P1110, DOI 10.1016/j.gie.2015.05.045
Itoi T, 2016, GUT, V65, P193, DOI 10.1136/gutjnl-2015-310348
Jang JW, 2012, GASTROENTEROLOGY, V142, P805, DOI 10.1053/j.gastro.2011.12.051
Kedia P, 2019, J CLIN GASTROENTEROL, V53, P304, DOI 10.1097/MCG.0000000000001037
Kedia P, 2014, GASTROENTEROLOGY, V147, P566, DOI 10.1053/j.gastro.2014.05.045
Khan MA, 2017, ENDOSC INT OPEN, V5, pE363, DOI 10.1055/s-0043-101693
Khan MA, 2017, GASTROINTEST ENDOSC, V85, P76, DOI 10.1016/j.gie.2016.06.032
Khan MA, 2016, DIGEST DIS SCI, V61, P684, DOI 10.1007/s10620-015-3933-0
Khashab MA, 2017, ENDOSC INT OPEN, V5, pE275, DOI 10.1055/s-0043-101695
Khashab MA, 2015, GASTROINTEST ENDOSC, V82, P932, DOI 10.1016/j.gie.2015.06.017
Kim GH, 2014, SCAND J GASTROENTERO, V49, P347, DOI 10.3109/00365521.2013.867361
Law Ryan, 2017, Gastrointest Endosc Clin N Am, V27, P715, DOI [10.1016/j.giec.2017.06.007, 10.1016/j.giec.2017.06.007]
Law Ryan, 2018, Gastrointest Endosc Clin N Am, V28, P187, DOI [10.1016/j.giec.2017.11.006, 10.1016/j.giec.2017.11.006]
Law R, 2017, ENDOSCOPY, V49, pE309, DOI 10.1055/s-0043-119351
Law R, 2016, CURR OPIN GASTROEN, V32, P232, DOI 10.1097/MOG.0000000000000255
Lennon AM, 2015, GASTROENTEROLOGY, V149, P825, DOI 10.1053/j.gastro.2015.05.062
Lévy P, 2018, VISC MED, V34, P192, DOI 10.1159/000489242
Mangiavillano B, 2016, WORLD J GASTROINTEST, V8, P143, DOI 10.4253/wjge.v8.i3.143
Mankaney G, 2017, GASTROENTEROLOGY, V153, P353, DOI 10.1053/j.gastro.2017.05.059
Mohan BP, 2020, ENDOSCOPY, V52, P96, DOI 10.1055/a-1020-3932
Mohan BP, 2019, GASTROINTEST ENDOSC, V89, P238, DOI [10.1016/j.gie.2018.10.018,

10.1016/j.gie.2018.10.036]
Muddana V, 2017, ENDOSC ULTRASOUND, V6, P278, DOI 10.4103/2303-9027.190920
Nelson DB, 1999, GASTROINTEST ENDOSC, V50, P928
Ngamruengphong S, 2017, ENDOSCOPY, V49, P549, DOI 10.1055/s-0043-105072
Nieto J, 2018, GASTROINTEST ENDOSC, V87, P469, DOI 10.1016/j.gie.2017.05.013
Noh DH, 2018, SCAND J GASTROENTERO, V53, P94, DOI 10.1080/00365521.2017.1390597
Paik WH, 2018, AM J GASTROENTEROL, V113, P987, DOI 10.1038/s41395-018-0122-8
Palazzo M, 2018, GASTROINTEST ENDOSC, V87, P1481, DOI 10.1016/j.gie.2017.12.033
Parekh PJ, 2015, ENDOSC ULTRASOUND, V4, P85, DOI 10.4103/2303-9027.156711
Park DH, 2015, J GASTROEN HEPATOL, V30, P1461, DOI 10.1111/jgh.13027
Pineda JJ, 2016, GASTROINTEST ENDOSC, V83, P360, DOI 10.1016/j.gie.2015.08.025
Rodrigues-Pinto E, 2016, GASTROINTEST ENDOSC, V84, P1040, DOI 10.1016/j.gie.2016.06.034
Rodrigues-Pinto E, 2016, EXPERT REV MED DEVIC, V13, P793, DOI 10.1080/17434440.2016.1222898
Rodrigues-Pinto E, 2016, ENDOSC INT OPEN, V4, pE506, DOI 10.1055/s-0041-110952
Rombaoa C, 2018, GASTROINTEST ENDOSC, V87, pAB458, DOI 10.1016/j.gie.2018.04.2001
Samarasena JB, 2018, ENDOSC ULTRASOUND, V7, P257, DOI 10.4103/eus.eus_35_18
Saumoy M, 2018, ENDOSC ULTRASOUND, V7, P97, DOI 10.4103/eus.eus_9_18
Saunders R, 2019, SURG ENDOSC, V33, P1412, DOI 10.1007/s00464-018-6416-5
Sey MSL, 2016, GASTROINTEST ENDOSC, V83, P347, DOI 10.1016/j.gie.2015.08.012
Shah ND, 2018, J HEPATO-BIL-PAN SCI, V25, P171, DOI 10.1002/jhbp.528
Shah ND, 2017, CLIN GASTROENTEROL H, V15, P784, DOI 10.1016/j.cgh.2017.01.011
Sharma M, 2018, WORLD J GASTRO ENDOS, V10, P283, DOI 10.4253/wjge.v10.i10.283
Siddiqui AA, 2014, DIGEST DIS SCI, V59, P687, DOI 10.1007/s10620-013-2945-x
Siddiqui UD, 2018, GASTROENTEROLOGY, V154, P1911, DOI 10.1053/j.gastro.2017.12.046
Simons-Linares CR, 2018, OBES SURG, V28, P3301, DOI 10.1007/s11695-018-3422-6
Spadaccini M, 2019, AM J GASTROENTEROL, V114, P174, DOI 10.1038/s41395-018-0371-6
Stavropoulos SN, 2012, GASTROINTEST ENDOSC, V75, P310, DOI 10.1016/j.gie.2011.09.043
Tanaka M, 2017, PANCREATOLOGY, V17, P738, DOI 10.1016/j.pan.2017.07.007
Teoh AYB, 2017, ENDOSCOPY, V49, P130, DOI 10.1055/s-0042-119036
Tessier G, 2007, GASTROINTEST ENDOSC, V65, P233, DOI 10.1016/j.gie.2006.06.029
Tyberg A, 2018, J CLIN GASTROENTEROL, V52, P79, DOI 10.1097/MCG.0000000000000786
Tyberg A, 2017, ENDOSCOPY, V49, pE252, DOI 10.1055/s-0043-115886
Varadarajulu S, 2008, GASTROINTEST ENDOSC, V68, P1102, DOI 10.1016/j.gie.2008.04.028
Vege SS, 2015, GASTROENTEROLOGY, V148, P819, DOI 10.1053/j.gastro.2015.01.015
Walter D, 2016, GUT, V65, P6, DOI 10.1136/gutjnl-2015-309925
Wang KX, 2016, GASTROINTEST ENDOSC, V83, P1218, DOI 10.1016/j.gie.2015.10.033
WIERSEMA MJ, 1994, GASTROINTEST ENDOSC, V40, P700, DOI 10.1016/S0016-5107(94)70113-X
NR 105
TC 13
Z9 13
U1 1
U2 3

PU LIPPINCOTT WILLIAMS & WILKINS
PI PHILADELPHIA
PA TWO COMMERCE SQ, 2001 MARKET ST, PHILADELPHIA, PA 19103 USA
SN 0192-0790
EI 1539-2031
J9 J CLIN GASTROENTEROL
JI J. Clin. Gastroenterol.
PD AUG
PY 2020
VL 54
IS 7
BP 579
EP 590
DI 10.1097/MCG.0000000000001337
PG 12
WC Gastroenterology & Hepatology
WE Science Citation Index Expanded (SCI-EXPANDED)
SC Gastroenterology & Hepatology
GA NE7VK
UT WOS:000562811300002
PM 32301834
DA 2026-05-09
ER

PT J
AU Takasawa, O
Fujita, N
Noda, Y
Kobayashi, G
Ito, K
Horaguchi, J
Obana, T
AF Takasawa, Osamu
Fujita, Naotaka
Noda, Yutaka
Kobayashi, Go
Ito, Kei
Horaguchi, Jun
Obana, Takashi
TI ENDOSONOGRAPHY-GUIDED GALLBLADDER DRAINAGE FOR ACUTE CHOLECYSTITIS FOLLOWING COVERED METAL STENT DEPLOYMENT
SO DIGESTIVE ENDOSCOPY

LA English
DT Article
DE acute cholecystitis; endosonography-guided biliary drainage;
endosonography-guided gallbladder drainage; gallbladder drainage;
interventional endoscopic ultrasound
ID DELAYED LAPAROSCOPIC CHOLECYSTECTOMY; HIGH-RISK PATIENTS; BILIARY
DRAINAGE; BILE-DUCT; PERCUTANEOUS CHOLECYSTOSTOMY; ENDOSCOPIC
SPHINCTEROTOMY; CHOLANGIOGRAPHY; OBSTRUCTION
AB Endosonography-guided biliary drainage (ESBD) is gaining acceptance as an effective treatment for obstructive jaundice. Only a few reports on the application of this technique to the gallbladder (endosonography-guided gallbladder drainage [ESGBD]) have been published in the literature. In order to relieve acute cholecystitis which developed in a patient with unresectable malignant biliary obstruction after deployment of a covered metal stent (CMS), we applied this technique. ESGBD was carried out by using an electronic curved linear array echoendoscope. After visualization of the gallbladder and determination of the puncture route, a needle knife papillotome was advanced with electrocautery to pierce the gastric and gallbladder walls. Under the guidance of a guidewire inserted through the needle sheath into the gallbladder, a 7.2 Fr, 30 cm-long, single pigtail plastic tube was placed to bridge the gallbladder and the stomach. No complications relevant to the procedure were encountered. ESGBD was quite effective in ameliorating the patient's acute cholecystitis and the drainage tube was removed after 10 days without sequelae. Acute cholecystitis following CMS deployment is considered to be a good indication for ESGBD.
C1 [Takasawa, Osamu] Sendai City Med Ctr, Dept Gastroenterol, Miyagino Ku, Sendai, Miyagi 9830824, Japan.
RP Takasawa, O (corresponding author), Sendai City Med Ctr, Dept Gastroenterol, Miyagino Ku, 5-22-1 Tsurugaya, Sendai, Miyagi 9830824, Japan.
EM taka1@pm.highway.ne.jp
CR Ang TL, 2007, J PANCREAS, V8, P438
ASSOULINE Y, 1993, GASTROEN CLIN BIOL, V17, P251
Bakkaloglu H, 2006, WORLD J GASTROENTERO, V12, P7179, DOI 10.3748/wjg.v12.i44.7179
Baron TH, 2002, GASTROINTEST ENDOSC, V56, P753, DOI 10.1067/mge.2002.129217
Baron TH, 2007, GASTROINTEST ENDOSC, V65, P735, DOI 10.1016/j.gie.2006.07.041
Bories E, 2007, ENDOSCOPY, V39, P287, DOI 10.1055/s-2007-966212
Burmester E, 2003, GASTROINTEST ENDOSC, V57, P246, DOI 10.1067/mge.2003.85
Conway JD, 2005, GASTROINTEST ENDOSC, V61, P32, DOI 10.1016/S0016-5107(04)02445-9
FERETIS C, 1993, ENDOSCOPY, V25, P392, DOI 10.1055/s-2007-1010347
Fujita N, 2008, J GASTROENTEROL, V43, P637, DOI 10.1007/s00535-008-2204-7
Fujita N, 2008, DIGEST ENDOSC, V20, P55, DOI 10.1111/j.1443-1661.2008.00782.x
Fujita N, 2007, WORLD J GASTROENTERO, V13, P5512, DOI 10.3748/wjg.v13.i41.5512
Giovannini M, 2003, ENDOSCOPY, V35, P1076, DOI 10.1055/s-2003-44596
Giovannini M, 2001, ENDOSCOPY, V33, P898, DOI 10.1055/s-2001-17324
Ito K, 2004, AM J ROENTGENOL, V183, P193, DOI 10.2214/ajr.183.1.1830193
Kahaleh M, 2006, GASTROINTEST ENDOSC, V64, P52, DOI 10.1016/j.gie.2006.01.063
Kahaleh M, 2005, GASTROINTEST ENDOSC, V61, P307, DOI 10.1016/S0016-5107(04)02585-4
Kim KH, 2000, AM J SURG, V179, P111, DOI 10.1016/S0002-9610(00)00247-6
Kjaer DW, 2007, ENDOSCOPY, V39, P304, DOI 10.1055/s-2007-966335

Kwan V, 2007, GASTROINTEST ENDOSC, V66, P582, DOI 10.1016/j.gie.2007.02.065
Lai R, 2005, ENDOSCOPY, V37, P487, DOI 10.1055/s-2005-861250
Lee SS, 2007, GASTROINTEST ENDOSC, V66, P1008, DOI 10.1016/j.gie.2007.03.1080
LENRIOT JP, 1993, GASTROEN CLIN BIOL, V17, P244
Mallery S, 2004, GASTROINTEST ENDOSC, V59, P100, DOI 10.1016/S0016-5107(03)02300-9
MARI M, 2001, GASTROINTEST ENDOSC, V54, P511
Püspök A, 2005, AM J GASTROENTEROL, V100, P1743, DOI 10.1111/j.1572-0241.2005.41806.x
Schlenker C, 2006, AM J GASTROENTEROL, V101, P278, DOI 10.1111/j.1572-0241.2006.00403.x
Spira RM, 2002, AM J SURG, V183, P62, DOI 10.1016/S0002-9610(01)00849-2
Takasawa O, 2006, WORLD J GASTROENTERO, V12, P7299, DOI 10.3748/wjg.v12.i45.7299
Will U, 2007, ENDOSCOPY, V39, P292, DOI 10.1055/s-2007-966215
NR 30
TC 18
Z9 19
U1 0
U2 1
PU WILEY
PI HOBOKEN
PA 111 RIVER ST, HOBOKEN 07030-5774, NJ USA
SN 0915-5635
EI 1443-1661
J9 DIGEST ENDOSC
JI Dig. Endosc.
PD JAN
PY 2009
VL 21
IS 1
BP 43
EP 47
DI 10.1111/j.1443-1661.2008.00822.x
PG 5
WC Gastroenterology & Hepatology; Surgery
WE Science Citation Index Expanded (SCI-EXPANDED)
SC Gastroenterology & Hepatology; Surgery
GA 387OZ
UT WOS:000261962800010
PM 19691802
DA 2026-05-09
ER

PT J
AU Ki, ELL

Napoleon, B
AF Ki, En-Ling Leung
Napoleon, Bertrand
TI Endoscopic ultrasound-guided biliary drainage: A change in paradigm?
SO WORLD JOURNAL OF GASTROINTESTINAL ENDOSCOPY
LA English
DT Review
DE Endoscopic ultrasound-guided biliary drainage; Endoscopic ultrasound-guided choledochoduodenostomy; Endoscopic ultrasound-guided hepaticogastrostomy; Lumen-apposing metal stents; Electrocautery-enhanced lumen-apposing metal stents
ID RETROGRADE CHOLANGIOPANCREATOGRAPHY; INTERNATIONAL MULTICENTER; PERCUTANEOUS DRAINAGE; STENT PLACEMENT; ERCP FAILS; OBSTRUCTION; CHOLEDOCHODUODENOSTOMY; ULTRASONOGRAPHY; HEPATICOGASTROSTOMY; GUIDELINES
AB Endoscopic ultrasound-guided biliary drainage (EUS-BD) has been developed as an alternative means of biliary drainage for malignant biliary obstruction (MBO). Compared to percutaneous transhepatic biliary drainage, EUS-BD offers effective internal drainage in a single session in the event of failed endoscopic retrograde cholangiopancreatography and has fewer adverse events (AE). In choosing which technique to use for EUS-BD, a combination of factors appears to be important in decision-making; technical expertise, the risk of AE, and anatomy. With the advent of novel all-in-one EUS-BD specific devices enabling simpler and safer techniques, as well as the growing experience and training of endosonographers, EUS-BD may potentially become a first-line technique in biliary drainage for MBO.
C1 [Ki, En-Ling Leung; Napoleon, Bertrand] Jean Mermoz Private Hosp, Dept Gastroenterol, 55 Ave Jean Mermoz, F-69008 Lyon, France.
RP Napoleon, B (corresponding author), Jean Mermoz Private Hosp, Dept Gastroenterol, 55 Ave Jean Mermoz, F-69008 Lyon, France.
EM dr.napoleon@wonadoo.fr
CR Alvarez-Sanchez MV, 2014, SURG ENDOSC, V28, P712, DOI 10.1007/s00464-013-3260-5
Anderloni A, 2016, ENDOSCOPY, V48, pE321, DOI 10.1055/s-0042-117425
Ardengh JC, 2018, WORLD J GASTRO ENDOS, V10, P99, DOI 10.4253/wjge.v10.i5.99
Artifon ELA, 2012, J CLIN GASTROENTEROL, V46, P768, DOI 10.1097/MCG.0b013e31825f264c
Bang JY, 2018, GASTROINTEST ENDOSC, V88, P9, DOI 10.1016/j.gie.2018.03.012
Baniya R, 2017, CLIN EXP GASTROENTER, V10, P67, DOI 10.2147/CEG.S132004
Dhir V, 2015, GASTROINTEST ENDOSC, V81, P913, DOI 10.1016/j.gie.2014.09.054
Dhir V, 2014, DIGEST ENDOSC, V26, P430, DOI 10.1111/den.12153
Dumonceau JM, 2018, ENDOSCOPY, V50, P910, DOI 10.1055/a-0659-9864
Fabbri C, 2014, WORLD J GASTROENTERO, V20, P8424, DOI 10.3748/wjg.v20.i26.8424
Giovannini M, 2001, ENDOSCOPY, V33, P898, DOI 10.1055/s-2001-17324
Holt BA, 2016, GASTROINTEST ENDOSC, V83, P160, DOI 10.1016/j.gie.2015.06.019
Jacques J, 2019, ENDOSCOPY, V51, P540, DOI 10.1055/a-0735-9137
Jacques J, 2019, ENDOSCOPY, V51, P40, DOI 10.1055/a-0628-6639
Jain D, 2018, DIGESTION, V98, P1, DOI 10.1159/000487185
Kahaleh M, 2013, WORLD J GASTROENTERO, V19, P1372, DOI 10.3748/wjg.v19.i9.1372

Kawakubo K, 2016, ENDOSCOPY, V48, P164, DOI 10.1055/s-0034-1393179
Khan MA, 2016, DIGEST DIS SCI, V61, P684, DOI 10.1007/s10620-015-3933-0
Khashab MA, 2016, ENDOSC INT OPEN, V4, pE487, DOI 10.1055/s-0042-102648
Kunda R, 2016, SURG ENDOSC, V30, P5002, DOI 10.1007/s00464-016-4845-6
Lee TH, 2016, CLIN GASTROENTEROL H, V14, P1011, DOI 10.1016/j.cgh.2015.12.032
Minaga K, 2018, DIGEST ENDOSC, V30, P38, DOI 10.1111/den.12910
Moole H, 2017, MEDICINE, V96, DOI 10.1097/MD.0000000000005154
Mukai S, 2017, J HEPATO-BIL-PAN SCI, V24, P537, DOI 10.1002/jhbp.496
Mussetto A, 2018, ANN GASTROENTEROL, V31, P535, DOI 10.20524/aog.2018.0287
Nakai Y, 2019, CLIN ENDOSC, V52, P220
Nakai Y, 2019, ENDOSC ULTRASOUND, V8, P111, DOI 10.4103/eus.eus_17_18
Nam K, 2018, ENDOSC ULTRASOUND, V7, P48, DOI 10.4103/eus.eus_100_17
Oh D, 2017, THER ADV GASTROENTER, V10, P42, DOI 10.1177/1756283X16671671
Paik WH, 2018, AM J GASTROENTEROL, V113, P987, DOI 10.1038/s41395-018-0122-8
Park JK, 2018, GASTROINTEST ENDOSC, V88, P277, DOI 10.1016/j.gie.2018.03.015
Sharaiha RZ, 2017, GASTROINTEST ENDOSC, V85, P904, DOI 10.1016/j.gie.2016.12.023
Teoh AYB, 2018, GUT, V67, P1209, DOI 10.1136/gutjnl-2017-314341
Uemura RS, 2018, J CLIN GASTROENTEROL, V52, P123, DOI 10.1097/MCG.0000000000000948
Wang KX, 2016, GASTROINTEST ENDOSC, V83, P1218, DOI 10.1016/j.gie.2015.10.033
Yamao K, 2018, GASTROINTEST ENDOSC, V88, P66, DOI 10.1016/j.gie.2018.01.021
NR 36
TC 12
Z9 13
U1 0
U2 1
PU BAISHIDENG PUBLISHING GROUP INC
PI PLEASANTON
PA 8226 REGENCY DR, PLEASANTON, CA 94588 USA
SN 1948-5190
J9 WORLD J GASTRO ENDOS
JI World J. Gastrointest. Endosc.
PD MAY 16
PY 2019
VL 11
IS 5
BP 345
EP 353
DI 10.4253/wjge.v11.i5.345
PG 9
WC Gastroenterology & Hepatology
WE Emerging Sources Citation Index (ESCI)
SC Gastroenterology & Hepatology

GA IC3WS
UT WOS:000470894200003
PM 31205595
OA Green Accepted, hybrid
DA 2026-05-09
ER

PT J
AU Dhir, V
Paramasivam, RK
Lazaro, JC
Maydeo, A
AF Dhir, Vinay
Paramasivam, Rajesh Kumar
Lazaro, Josef Carlo
Maydeo, Amit
TI The role of therapeutic endoscopic ultrasound now and for the future
SO EXPERT REVIEW OF GASTROENTEROLOGY & HEPATOLOGY
LA English
DT Review
DE biliary drainage; endoscopy; endosonography; pseudocyst; therapeutic
ID GUIDED BILIARY DRAINAGE; CELIAC PLEXUS NEUROLYSIS; PANCREATIC FLUID
COLLECTIONS; COVERED METAL STENT; TERM-FOLLOW-UP; TRANSMURAL DRAINAGE;
GALLBLADDER DRAINAGE; ETHANOL ABLATION; CONTROLLED-TRIAL;
SINGLE-OPERATOR
AB Therapeutic endoscopic ultrasound (EUS) became possible after the advent of the linear echoendoscope and the EUS guided fine needle aspiration. Over the past two decades, the indications for therapeutic EUS have expanded and evidence regarding its utility has been steadily accumulating. Randomized studies have shown EUS to be effective for cancer pain relief (celiac plexus neurolysis), pancreatic fluid collection drainage, and biliary drainage. Prospective studies have shown EUS-guided biliary drainage to be safe and effective in patients with failed ERCP. There is evidence to suggest that EUS is effective for pancreatic duct drainage, gallbladder drainage, and drainage of pelvic collections. EUS may also be useful for targeted cancer treatment via brachytherapy, radiofrequency ablation, or injection therapy. Therapeutic EUS is likely to play an increasingly important role in endoscopic therapy of gastrointestinal diseases in the near future.
C1 [Dhir, Vinay; Paramasivam, Rajesh Kumar; Lazaro, Josef Carlo; Maydeo, Amit] Global Hosp, Baldota Inst Digest Sci, Dept Endoscopy & Endosonog, Bombay 400012, Maharashtra, India.
RP Dhir, V (corresponding author), Global Hosp, Baldota Inst Digest Sci, Dept Endoscopy & Endosonog, Bombay 400012, Maharashtra, India.
EM vinaydhir@gmail.com
CR Artifon ELA, 2012, J CLIN GASTROENTEROL, V46, P768, DOI 10.1097/MCG.0b013e31825f264c
Ascunce G, 2011, GASTROINTEST ENDOSC, V73, P267, DOI 10.1016/j.gie.2010.10.029
Ascunce G, 2010, SURG ENDOSC, V24, P1447, DOI 10.1007/s00464-009-0798-3
Bakker OJ, 2012, JAMA-J AM MED ASSOC, V307, P1053, DOI 10.1001/jama.2012.276

Bang JY, 2013, CLIN ENDOSC, V46, P500, DOI 10.5946/ce.2013.46.5.500
Barkay O, 2010, GASTROINTEST ENDOSC, V71, P1166, DOI 10.1016/j.gie.2009.10.048
Chaves DM, 2012, ENDOSCOPY, V44, pE99, DOI 10.1055/s-0030-1256740
Daldoul S, 2012, J VISC SURG, V149, pE172, DOI 10.1016/j.jviscsurg.2012.03.008
de Paulo GA, 2006, GASTROINTEST ENDOSC, V63, P396, DOI 10.1016/j.gie.2005.10.039
Deprez PH, 2009, BEST PRACT RES CL GA, V23, P623, DOI 10.1016/j.bpg.2009.07.001
DeWitt John, 2012, Gastrointest Endosc Clin N Am, V22, P291, DOI 10.1016/j.giec.2012.04.001
DeWitt J, 2010, GASTROINTEST ENDOSC, V72, P862, DOI 10.1016/j.gie.2010.02.039
DeWitt J, 2009, GASTROINTEST ENDOSC, V70, P710, DOI 10.1016/j.gie.2009.03.1173
Dhir Vinay, 2011, J Interv Gastroenterol, V1, P153
Dhir V, 2014, DIGEST ENDOSC, V26, P430, DOI 10.1111/den.12153
Dhir V, 2012, GASTROINTEST ENDOSC, V75, P354, DOI 10.1016/j.gie.2011.07.075
Doi S, 2013, ENDOSCOPY, V45, P362, DOI 10.1055/s-0032-1326225
Draganov PV, 2013, DIGEST ENDOSC, V25, P615, DOI 10.1111/den.12046
Ergun M, 2011, ENDOSCOPY, V43, P518, DOI 10.1055/s-0030-1256333
Fritscher-Ravens A, 2004, GASTROINTEST ENDOSC, V59, P89, DOI 10.1016/S0016-5107(03)02355-1
Fritscher-Ravens A, 2002, GASTROINTEST ENDOSC, V56, P737, DOI 10.1067/mge.2002.129084
Fritscher-Ravens A, 2006, MINIM INVASIV THER, V15, P366, DOI 10.1080/13645700601040834
Fujii LL, 2013, GASTROINTEST ENDOSC, V78, P854, DOI 10.1016/j.gie.2013.05.016
Ge N, 2013, GASTROENT RES PRACT, V2013, DOI 10.1155/2013/785483
Gornals JB, 2013, SURG ENDOSC, V27, P1428, DOI 10.1007/s00464-012-2591-y
Gupta K, 2014, J CLIN GASTROENTEROL, V48, P80, DOI 10.1097/MCG.0b013e31828c6822
Hara K, 2013, ENDOSCOPY, V45, P392, DOI 10.1055/s-0032-1326076
Hara K, 2011, AM J GASTROENTEROL, V106, P1239, DOI 10.1038/ajg.2011.84
Itoi T, 2013, GASTROINTEST ENDOSC, V78, P934, DOI 10.1016/j.gie.2013.09.025
Itoi T, 2013, DIGEST ENDOSC, V25, P241, DOI 10.1111/den.12048
Itoi Takao, 2012, Gastrointest Endosc Clin N Am, V22, P371, DOI 10.1016/j.giec.2012.04.015
Itoi T, 2011, DIGEST ENDOSC, V23, P51, DOI 10.1111/j.1443-1661.2011.01141.x
Itoi T, 2010, GASTROINTEST ENDOSC, V71, P1038, DOI 10.1016/j.gie.2010.01.026
Iwashita T, 2012, ENDOSCOPY, V44, P60, DOI 10.1055/s-0030-1256871
Iwashita T, 2013, DIGEST DIS SCI, V58, P2417, DOI 10.1007/s10620-013-2645-6
Iwata K, 2011, DIGEST ENDOSC, V23, P140, DOI 10.1111/j.1443-1661.2010.01046.x
Jang JW, 2012, GASTROENTEROLOGY, V142, P805, DOI 10.1053/j.gastro.2011.12.051
Jin Z, 2008, ENDOSCOPY, V40, P314, DOI 10.1055/s-2007-995476
Jürgensen C, 2012, SURG ENDOSC, V26, P1359, DOI 10.1007/s00464-011-2039-9
Kawakubo K, 2014, J HEPATO-BIL-PAN SCI, V21, P328, DOI 10.1002/jhbp.27
Kawakubo K, 2013, SURG ENDOSC, V27, P3437, DOI 10.1007/s00464-013-2896-5
Kedia P, 2013, CLIN ENDOSC, V46, P543, DOI 10.5946/ce.2013.46.5.543
Khashab MA, 2013, GASTROINTEST ENDOSC, V78, P734, DOI 10.1016/j.gie.2013.05.013
Kim TH, 2012, WORLD J GASTROENTERO, V18, P2526, DOI 10.3748/wjg.v18.i20.2526
Komaki T, 2011, PANCREATOLOGY, V11, P47, DOI 10.1159/000323508
LeBlanc JK, 2011, GASTROINTEST ENDOSC, V74, P1300, DOI 10.1016/j.gie.2011.07.073

LeBlanc JK, 2009, GASTROINTEST ENDOSC, V69, P835, DOI 10.1016/j.gie.2008.05.071
Levy MJ, 2008, AM J GASTROENTEROL, V103, P352, DOI 10.1111/j.1572-0241.2007.01616.x
Mavrogenis G, 2013, PANCREATOLOGY, V13, P316, DOI 10.1016/j.pan.2013.04.004
Nici AJ, 2012, DIGEST ENDOSC, V24, P185, DOI 10.1111/j.1443-1661.2011.01202.x
Noh SH, 2010, GASTROINTEST ENDOSC, V71, P1314, DOI 10.1016/j.gie.2009.12.045
O'Toole TM, 2009, ENDOSCOPY, V41, P593, DOI 10.1055/s-0029-1214868
Oh HC, 2011, GASTROENTEROLOGY, V140, P172, DOI 10.1053/j.gastro.2010.10.001
Pai M, 2013, GASTROINTEST ENDOSC, V77, pAB351
Panamonta N, 2012, EUR J GASTROEN HEPAT, V24, P1355, DOI 10.1097/MEG.0b013e32835871eb
Park DH, 2009, ENDOSCOPY, V41, P842, DOI 10.1055/s-0029-1215133
Park DH, 2013, GASTROINTEST ENDOSC, V78, P91, DOI 10.1016/j.gie.2013.01.042
Park DH, 2011, GASTROINTEST ENDOSC, V74, P1276, DOI 10.1016/j.gie.2011.07.054
Park NH, 2011, J CLIN ULTRASOUND, V39, P441, DOI 10.1002/jcu.20861
Prachayakul V, 2013, WORLD J GASTROENTERO, V19, P4758, DOI 10.3748/wjg.v19.i29.4758
Puli SR, 2009, DIGEST DIS SCI, V54, P2330, DOI 10.1007/s10620-008-0651-x
Ramesh J, 2013, J GASTROEN HEPATOL, V28, P620, DOI 10.1111/jgh.12081
Rana SS, 2011, INDIAN J GASTROENTER, V30, P280, DOI 10.1007/s12664-011-0139-z
Rische S, 2013, SCAND J GASTROENTERO, V48, P231, DOI 10.3109/00365521.2012.752029
Roberts KJ, 2012, HPB, V14, P489, DOI 10.1111/j.1477-2574.2012.00470.x
Romero-Castro R, 2013, GASTROINTEST ENDOSC, V78, P711, DOI 10.1016/j.gie.2013.05.009
Sadik R, 2011, WORLD J GASTROENTERO, V17, P499, DOI 10.3748/wjg.v17.i4.499
Sahai AV, 2009, AM J GASTROENTEROL, V104, P326, DOI 10.1038/ajg.2008.64
Sakamoto H, 2010, AM J GASTROENTEROL, V105, P2599, DOI 10.1038/ajg.2010.339
Seifert H, 2009, GUT, V58, P1260, DOI 10.1136/gut.2008.163733
Shah JN, 2012, GASTROINTEST ENDOSC, V75, P56, DOI 10.1016/j.gie.2011.08.032
Shami VM, 2008, GASTROINTEST ENDOSC, V67, P136, DOI 10.1016/j.gie.2007.07.040
Shirley LA, 2013, GASTROENT RES PRACT, V2013, DOI [10.1155/2013/207129, DOI 10.1155/2013/207129]
Sun S, 2006, ENDOSCOPY, V38, P399, DOI 10.1055/s-2006-925253
Suzuki Rei, 2013, Diagn Ther Endosc, V2013, P157581, DOI 10.1155/2013/157581
Tessier G, 2007, GASTROINTEST ENDOSC, V65, P233, DOI 10.1016/j.gie.2006.06.029
Trevino JM, 2008, GASTROINTEST ENDOSC, V68, P1215, DOI 10.1016/j.gie.2008.07.016
Varadarajulu S, 2010, ENDOSCOPY, V42, P423, DOI 10.1055/s-0029-1243989
Varadarajulu S, 2013, GASTROENTEROLOGY, V145, P583, DOI 10.1053/j.gastro.2013.05.046
Varadarajulu S, 2011, J GASTROEN HEPATOL, V26, P1504, DOI 10.1111/j.1440-1746.2011.06771.x
Varadarajulu S, 2009, GASTROINTEST ENDOSC, V70, P1121, DOI 10.1016/j.gie.2009.08.034
Weilert F, 2012, ENDOSCOPY, V44, P780, DOI 10.1055/s-0032-1309839
Widmer J, 2013, DIG ENDOSC
Wyse JM, 2011, J CLIN ONCOL, V29, P3541, DOI 10.1200/JCO.2010.32.2750
Yamamoto N, 2013, GASTROINTEST ENDOSC, V77, P809, DOI 10.1016/j.gie.2013.01.009
Yasuda I, 2013, ENDOSCOPY, V45, P627, DOI 10.1055/s-0033-1344027
Zhang WY, 2013, WORLD J GASTROENTERO, V19, P3397, DOI 10.3748/wjg.v19.i22.3397
NR 87

TC 7
Z9 7
U1 0
U2 7
PU TAYLOR & FRANCIS LTD
PI ABINGDON
PA 2-4 PARK SQUARE, MILTON PARK, ABINGDON OR14 4RN, OXON, ENGLAND
SN 1747-4124
EI 1747-4132
J9 EXPERT REV GASTROENT
JI Expert Rev. Gastroenterol. Hepatol.
PD SEP
PY 2014
VL 8
IS 7
BP 775
EP 791
DI 10.1586/17474124.2014.917953
PG 17
WC Gastroenterology & Hepatology
WE Science Citation Index Expanded (SCI-EXPANDED)
SC Gastroenterology & Hepatology
GA AN8CM
UT WOS:000340829500007
PM 24830540
DA 2026-05-09
ER

PT J
AU Bang, JY
Varadarajulu, S
AF Bang, Ji Young
Varadarajulu, Shyam
TI Lumen-apposing metal stents for endoscopic ultrasound-guided drainage of
pancreatic fluid collections
SO TECHNIQUES AND INNOVATIONS IN GASTROINTESTINAL ENDOSCOPY
LA English
DT Article
DE Lumen-apposing metal stent; Pancreatic fluid collections; Walled-off
necrosis; AXIOS stent; SPAXUS stent
AB Lumen-apposing metal stents (LAMS) integrated in a single-step delivery platform are ideally suited for the endoscopic ultrasound-guided drainage of pancreatic fluid collections. Given their unique ability to approximate the capsule of the fluid

collection to the adjoining gastric or duodenal wall, the potential for leak or perforation is minimal. Additionally, the wide stent lumen not only facilitates faster drainage of cyst contents but also enables other therapeutic interventions such as endoscopic debridement in patients with infected necrotizing pancreatitis. A keen awareness of the technical design of LAMS and its clinical effectiveness is important to minimize adverse events and maximize treatment outcomes. This review outlines the technical approach and clinical outcomes when LAMS are used for the endoscopic drainage of pancreatic fluid collections. (c) 2019 Elsevier Inc. All rights reserved.

C1 [Bang, Ji Young; Varadarajulu, Shyam] AdventHlth Orlando, Ctr Intervent Endoscopy, Orlando, FL 32803 USA.

C3 Adventist Health Services; AdventHealth; (AdventHealth) Central Florida

Division; Central Florida Hospital - South; AdventHealth Orlando

RP Varadarajulu, S (corresponding author), AdventHlth Orlando, Ctr Intervent Endoscopy, Orlando, FL 32803 USA.

EM svaradarajulu@uab.edu

CR Bang JY, 2019, GUT, V68, P1200, DOI 10.1136/gutjnl-2017-315335

Bang JY, 2017, GUT, V66, P2054, DOI 10.1136/gutjnl-2016-312812

Bang JY, 2017, DIGEST ENDOSC, V29, P83, DOI 10.1111/den.12681

Brimhall B, 2018, CLIN GASTROENTEROL H, V16, P1521, DOI 10.1016/j.cgh.2018.02.021

Chen YI, 2019, ENDOSC INT OPEN, V7, pE347, DOI 10.1055/a-0828-7630

Chen YI, 2018, ENDOSC INT OPEN, V6, pE780, DOI 10.1055/a-0611-5082

Holt BA, 2014, GASTROINTEST ENDOSC, V80, P1199, DOI 10.1016/j.gie.2014.07.044

Manrai M, 2018, ANN SURG, V267, P357, DOI 10.1097/SLA.0000000000002065

Patra PS, 2014, BRIT J SURG, V101, P1721, DOI 10.1002/bjs.9666

Puga M, 2018, ENDOSCOPY, V50, P1022, DOI 10.1055/a-0582-9127

Shah RJ, 2015, CLIN GASTROENTEROL H, V13, P747, DOI 10.1016/j.cgh.2014.09.047

Sharaiha RZ, 2016, CLIN GASTROENTEROL H, V14, P1797, DOI 10.1016/j.cgh.2016.05.011

Song TJ, 2019, GASTROINTEST ENDOSC

Walter D, 2015, ENDOSCOPY, V47, P63, DOI 10.1055/s-0034-1378113

Yang JL, 2019, ENDOSCOPY, V51, P1035, DOI 10.1055/a-0759-1353

NR 15

TC 2

Z9 3

U1 0

U2 0

PU ELSEVIER SCIENCE INC

PI NEW YORK

PA STE 800, 230 PARK AVE, NEW YORK, NY 10169 USA

SN 2666-5107

EI 2590-0307

J9 TECH INNOVAT GASTROI

JI Tech. Innovation Gastrointes. Endoscopy

PD JAN

PY 2020

VL 22

IS 1

SI SI
BP 14
EP 18
DI 10.1016/j.tgie.2019.150635
PG 5
WC Gastroenterology & Hepatology
WE Emerging Sources Citation Index (ESCI)
SC Gastroenterology & Hepatology
GA VK9JD
UT WOS:000768800500005
DA 2026-05-09
ER

PT J
AU Dhir, V
Isayama, H
Itoi, T
Almadi, M
Siripun, A
Teoh, AYB
Ho, KY
AF Dhir, Vinay
Isayama, Hiroyuki
Itoi, Takao
Almadi, Majid
Siripun, Aroon
Teoh, Anthony Y. B.
Ho, Khek Yu
TI Endoscopic ultrasonography-guided biliary and pancreatic duct
interventions
SO DIGESTIVE ENDOSCOPY
LA English
DT Review
DE biliary drainage; chronic pancreatitis; endoscopic ultrasound; ERCP
ID FAILED ERCP; GALLBLADDER DRAINAGE; STENT PLACEMENT; METAL STENT;
INTERNATIONAL MULTICENTER; ACUTE CHOLECYSTITIS; PRIME-TIME; EUS;
OBSTRUCTION; ACCESS
AB Drainage of obstructed bile duct and pancreatic duct under endoscopic ultrasonography (EUS) guidance has evolved into viable techniques suitable for patients with failed endoscopic retrograde cholangiopancreatography (ERCP) and/or altered surgical anatomy. One of the major advantages of EUS guidance is the possibility of multiple access points depending upon patient and ductal anatomy. Unlike ERCP, an approachable papilla is not a requisite for successful EUS-guided biliary or pancreatic ductal drainage. Moreover, as the access is away from the papilla, there is the possibility of

reduced pancreatitis. A variety of procedures have become available for EUS-guided drainage, and it is important to develop standard terminology and procedural details. EUS-specific stents, including lumen-apposing metal stents have recently become available, and are likely to impact the outcomes of these procedures. Available data show a high success rate and acceptable adverse event rate for EUS-guided biliary drainage. Success rate appears to be low for pancreatic duct drainage because of a variety of reasons. Outcomes of EUS-guided biliary drainage appear equivalent to percutaneous drainage and ERCP. EUS-guided gallbladder drainage appears promising for patients requiring gallbladder drainage but unfit for surgery. Further large controlled studies are needed to evaluate the exact role of these procedures.
C1 [Dhir, Vinay] Global Hosp, Baldota Inst Digest Sci, Mumbai 400012, Maharashtra, India.
[Isayama, Hiroyuki] Univ Tokyo, Grad Sch Med, Tokyo, Japan.
[Itoi, Takao] Tokyo Med Univ, Tokyo, Japan.
[Almadi, Majid] King Saud Univ, King Khalid Univ Hosp, Riyadh, Saudi Arabia.
[Siripun, Aroon] Bangkok Hosp, Bangkok, Thailand.
[Teoh, Anthony Y. B.] Chinese Univ Hong Kong, Prince Wales Hosp, Hong Kong, Hong Kong, Peoples R China.
[Ho, Khek Yu] Natl Univ Hlth Syst, Singapore, Singapore.
C3 University of Tokyo; Tokyo Medical University; King Saud University;
King Khalid University Hospital; Chinese University of Hong Kong; Prince
of Wales Hospital Hong Kong; National University of Singapore
RP Dhir, V (corresponding author), Global Hosp, Baldota Inst Digest Sci, Mumbai 400012, Maharashtra, India.
EM vinaydhir@gmail.com
RI Almadi, Majid/KIA-2074-2024; Teoh, Anthony/L-4796-2016; Isayama,
Hiroyuki/AER-9916-2022
OI Almadi, Majid/0000-0001-9917-2758;
CR Artifon ELA, 2012, J CLIN GASTROENTEROL, V46, P768, DOI 10.1097/MCG.0b013e31825f264c
Bataille L, 2002, GASTROINTEST ENDOSC, V55, P740, DOI 10.1067/mge.2002.123621
Bories E, 2008, ENDOSCOPY S1, V40, pA55
Cho DH, 2017, GASTROINTEST ENDOSC, V85, P1067, DOI 10.1016/j.gie.2016.09.010
Choi JH, 2014, ENDOSCOPY, V46, P656, DOI 10.1055/s-0034-1365720
Dhir V, 2015, GASTROINTEST ENDOSC, V81, P913, DOI 10.1016/j.gie.2014.09.054
Dhir V, 2013, UNITED EUR GASTROENT, V1, P103, DOI 10.1177/2050640613480145
Dhir V, 2014, DIGEST ENDOSC, V26, P430, DOI 10.1111/den.12153
Dhir V, 2012, GASTROINTEST ENDOSC, V75, P354, DOI 10.1016/j.gie.2011.07.075
Giovannini M, 2013, GASTROINTEST ENDOSC, V78, P865, DOI 10.1016/j.gie.2013.10.019
Gupta K, 2014, J CLIN GASTROENTEROL, V48, P80, DOI 10.1097/MCG.0b013e31828c6822
Hara K, 2013, ENDOSCOPY, V45, P392, DOI 10.1055/s-0032-1326076
Itoi T, 2015, ENDOSCOPY, V47, P462, DOI 10.1055/s-0034-1391083
Itoi T, 2014, J HEPATO-BIL-PAN SCI, V21, pE4, DOI 10.1002/jhbp.43
Itoi T, 2013, DIGEST ENDOSC, V25, P241, DOI 10.1111/den.12048
Itoi T, 2010, J HEPATO-BIL-PAN SCI, V17, P611, DOI 10.1007/s00534-009-0196-1
Iwashita T, 2012, ENDOSCOPY, V44, P60, DOI 10.1055/s-0030-1256871
Iwashita T, 2016, GASTROINTEST ENDOSC, V83, P394, DOI 10.1016/j.gie.2015.04.043
Jang JW, 2012, GASTROENTEROLOGY, V142, P805, DOI 10.1053/j.gastro.2011.12.051
Kahaleh M, 2006, GASTROINTEST ENDOSC, V64, P52, DOI 10.1016/j.gie.2006.01.063

Kahaleh M, 2007, GASTROINTEST ENDOSC, V65, P224, DOI 10.1016/j.gie.2006.05.008
Kahaleh M, 2016, DIGEST LIVER DIS, V48, P1054, DOI 10.1016/j.dld.2016.05.021
Kawakubo K, 2016, ENDOSCOPY, V48, P164, DOI 10.1055/s-0034-1393179
Kawakubo K, 2014, J HEPATO-BIL-PAN SCI, V21, P328, DOI 10.1002/jhbp.27
Khan MA, 2016, DIGEST DIS SCI, V61, P684, DOI 10.1007/s10620-015-3933-0
Khashab MA, 2016, ENDOSC INT OPEN, V4, pE487, DOI 10.1055/s-0042-102648
Khashab MA, 2016, ENDOSC INT OPEN, V4, pE175, DOI 10.1055/s-0041-109083
Kunda R, 2016, SURG ENDOSC, V30, P5002, DOI 10.1007/s00464-016-4845-6
Lee TH, 2016, CLIN GASTROENTEROL H, V14, P1011, DOI 10.1016/j.cgh.2015.12.032
Liao WC, 2017, GASTROINTEST ENDOSC, V85, P295, DOI 10.1016/j.gie.2016.09.037
Mallery S, 2004, GASTROINTEST ENDOSC, V59, P100, DOI 10.1016/S0016-5107(03)02300-9
Maranki J, 2009, ENDOSCOPY, V41, P532, DOI 10.1055/s-0029-1214712
Martins FP, 2010, ENDOSCOPY, V42, pE126, DOI 10.1055/s-0029-1243911
Minaga K, 2017, GASTROINTEST ENDOSC, V85, P263, DOI 10.1016/j.gie.2016.03.016
Nakai Y, 2016, ENDOSCOPY, V48, P1125, DOI 10.1055/s-0042-116595
Park DH, 2013, GASTROINTEST ENDOSC, V78, P91, DOI 10.1016/j.gie.2013.01.042
Park DH, 2011, GASTROINTEST ENDOSC, V74, P1276, DOI 10.1016/j.gie.2011.07.054
Poincloux L, 2015, ENDOSCOPY, V47, P794, DOI [10.1055/s-0035-1545399, 10.1055/s-0034-1391988]
Shah JN, 2012, GASTROINTEST ENDOSC, V75, P56, DOI 10.1016/j.gie.2011.08.032
Tessier G, 2007, GASTROINTEST ENDOSC, V65, P233, DOI 10.1016/j.gie.2006.06.029
Tyberg A, 2016, GASTROINTEST ENDOSC, V84, P941, DOI 10.1016/j.gie.2016.05.035
Varadarajulu S, 2005, GASTROINTEST ENDOSC, V61, P568, DOI 10.1016/S0016-5107(04)02832-9
Vila JJ, 2012, GASTROINTEST ENDOSC, V76, P1133, DOI 10.1016/j.gie.2012.08.001
Walter D, 2016, GUT, V65, P6, DOI 10.1136/gutjnl-2015-309925
Will U, 2015, WORLD J GASTROENTERO, V21, P13140, DOI 10.3748/wjg.v21.i46.13140
NR 45
TC 92
Z9 110
U1 0
U2 4
PU WILEY
PI HOBOKEN
PA 111 RIVER ST, HOBOKEN 07030-5774, NJ USA
SN 0915-5635
EI 1443-1661
J9 DIGEST ENDOSC
JI Dig. Endosc.
PD MAY
PY 2017
VL 29
IS 4
SI SI

BP 472
EP 485
DI 10.1111/den.12818
PG 14
WC Gastroenterology & Hepatology; Surgery
WE Science Citation Index Expanded (SCI-EXPANDED)
SC Gastroenterology & Hepatology; Surgery
GA EU6PN
UT WOS:000401156000006
PM 28118509
OA Bronze
DA 2026-05-09
ER

PT J
AU Fugazza, A
Colombo, M
Repici, A
Anderloni, A
AF Fugazza, Alessandro
Colombo, Matteo
Repici, Alessandro
Anderloni, Andrea
TI Endoscopic Ultrasound-Guided Gallbladder Drainage: Current Perspectives
SO CLINICAL AND EXPERIMENTAL GASTROENTEROLOGY
LA English
DT Review
DE endoscopic ultrasound-guided gallbladder drainage; lumen-apposing metal
stent; acute cholecystitis; percutaneous gallbladder drainage;
cholecystostomy
ID APPOSING METAL STENT; PERCUTANEOUS CHOLECYSTOSTOMY; ACUTE CHOLECYSTITIS;
BILIARY OBSTRUCTION; MANAGEMENT; PLACEMENT; OUTCOMES
AB According to the recently updated Tokyo Guidelines, laparoscopic cholecystectomy still represents the gold standard for the treatment of acute cholecystitis. However, fragile patients, due to comorbidities or poor clinical conditions, have a high surgical risk. In such cases, percutaneous or endoscopic gallbladder drainage is considered the treatment of choice. In particular, endoscopic ultrasound-guided gallbladder drainage with the placement of specifically designed stents is now considered an alternative option. In addition, the opening of an access door to the lumen of the gallbladder could offer new opportunities for the endoscopic treatment of gallbladder diseases. The purpose of this review is to provide an update on the latest available evidence in the literature regarding the endoscopic ultrasound-guided gallbladder drainage.
C1 [Fugazza, Alessandro; Colombo, Matteo; Repici, Alessandro; Anderloni, Andrea] IRCCS, Humanitas Clin & Res Ctr, Digest Endoscopy Unit, Dept Gastroenterol, Rozzano, MI, Italy.
[Colombo, Matteo; Repici, Alessandro] Humanitas Univ, Dept Biomed Sci, Milan, Italy.

C3 IRCCS Humanitas Research Hospital; Humanitas University
RP Anderloni, A (corresponding author), Humanitas Res Hosp, Div Gastroenterol, Digest Endoscopy Unit, Rozzano, Italy.
EM andrea.anderloni@humanitas.it
RI Repici, Alessandro/HFH-8162-2022; Anderloni, Andrea/ABE-9139-2020;
Colombo, Matteo/N-9849-2013; Fugazza, Alessandro/ABG-9381-2021
OI Repici, Alessandro/0000-0002-1621-6450; Anderloni,
Andrea/0000-0002-1021-0031; Colombo, Matteo/0000-0003-0715-8233;
Fugazza, Alessandro/0000-0003-0485-4903
CR Anderloni A, 2019, WORLD J GASTROENTERO, V25, P3857, DOI 10.3748/wjg.v25.i29.3857
Anderloni A, 2019, GASTROINTEST ENDOSC, V89, P69, DOI [10.1016/j.gie.2018.04.1746, 10.1016/j.gie.2018.08.047]
Anderloni A, 2016, SURG ENDOSC, V30, P5200, DOI 10.1007/s00464-016-4894-x
Chan SM, 2017, GASTROINTEST ENDOSC, V85, P1225, DOI 10.1016/j.gie.2016.10.014
Chen YY, 2011, ENDOSCOPY, V43, pE88, DOI 10.1055/s-0030-1255983
Choi JH, 2014, ENDOSCOPY, V46, P656, DOI 10.1055/s-0034-1365720
FERETIS CB, 1990, GASTROINTEST ENDOSC, V36, P523, DOI 10.1016/S0016-5107(90)71134-0
Ge N, 2016, BMC GASTROENTEROL, V16, DOI 10.1186/s12876-016-0420-9
Imai H, 2016, GASTROINTEST ENDOSC, V84, P147, DOI 10.1016/j.gie.2015.12.024
Irani S, 2017, CLIN GASTROENTEROL H, V15, P738, DOI 10.1016/j.cgh.2016.12.021
Itoi T, 2008, GASTROINTEST ENDOSC, V68, P455, DOI 10.1016/j.gie.2008.02.052
Itoi T, 2010, GASTROINTEST ENDOSC, V71, P1038, DOI 10.1016/j.gie.2010.01.026
Jain D, 2018, CLIN ENDOSC, V51, P450, DOI 10.5946/ce.2018.024
Jang JW, 2012, GASTROENTEROLOGY, V142, P805, DOI 10.1053/j.gastro.2011.12.051
Kedia P, 2015, GASTROINTEST ENDOSC, V82, P1031, DOI 10.1016/j.gie.2015.03.1912
Kwan V, 2007, GASTROINTEST ENDOSC, V66, P582, DOI 10.1016/j.gie.2007.02.065
Lee TH, 2011, ENDOSCOPY, V43, P702, DOI 10.1055/s-0030-1256226
Luk SWY, 2019, ENDOSCOPY, V51, P722, DOI 10.1055/a-0929-6603
Maekawa S, 2013, BMC GASTROENTEROL, V13, DOI 10.1186/1471-230X-13-65
McCarthy ST, 2015, DIGEST DIS SCI, V60, P2516, DOI 10.1007/s10620-014-3371-4
MCGAHAN JP, 1989, RADIOLOGY, V173, P481, DOI 10.1148/radiology.173.2.2678261
Minaga K, 2019, DIGEST ENDOSC, V31, P180, DOI 10.1111/den.13242
Mori Y, 2018, J HEPATO-BIL-PAN SCI, V25, P87, DOI 10.1002/jhbp.504
Mussetto A, 2018, ANN GASTROENTEROL, V31, P535, DOI 10.20524/aog.2018.0287
Oh D, 2019, GASTROINTEST ENDOSC, V89, P289, DOI 10.1016/j.gie.2018.08.052
Okamoto K, 2018, J HEPATO-BIL-PAN SCI, V25, P55, DOI 10.1002/jhbp.516
Perez-Miranda M, 2018, ENDOSC ULTRASOUND, V7, P79, DOI 10.4103/eus.eus_5_18
Song TJ, 2010, GASTROINTEST ENDOSC, V71, P634, DOI 10.1016/j.gie.2009.11.024
Sugiyama M, 1998, WORLD J SURG, V22, P459, DOI 10.1007/s002689900416
Teoh AY, 2019, ENDOSC INT OPEN, V7, pE964, DOI 10.1055/a-0915-2098
Teoh AYB, 2017, ENDOSCOPY, V49, P130, DOI 10.1055/s-0042-119036
Tian L, 2018, ENDOSCOPY, V50, pE307, DOI 10.1055/a-0631-7970
Yuste RT, 2019, ANN GASTROENTEROL, V32, P514, DOI 10.20524/aog.2019.0395

Tyberg A, 2018, J CLIN GASTROENTEROL, V52, P79, DOI 10.1097/MCG.0000000000000786
Walter D, 2016, GUT, V65, P6, DOI 10.1136/gutjnl-2015-309925
Winbladh A, 2009, HPB, V11, P183, DOI 10.1111/j.1477-2574.2009.00052.x
NR 36
TC 23
Z9 24
U1 0
U2 3
PU DOVE MEDICAL PRESS LTD
PI ALBANY
PA PO BOX 300-008, ALBANY, AUCKLAND 0752, NEW ZEALAND
SN 1178-7023
J9 CLIN EXP GASTROENTER
JI Clin. Exp. Gastroenterol.
PY 2020
VL 13
BP 193
EP 201
DI 10.2147/CEG.S203626
PG 9
WC Gastroenterology & Hepatology
WE Emerging Sources Citation Index (ESCI)
SC Gastroenterology & Hepatology
GA LQ8UK
UT WOS:000535273500001
PM 32523368
OA Green Submitted, gold
DA 2026-05-09
ER

PT J
AU Krishnamoorthi, R
Irani, S
AF Krishnamoorthi, Rajesh
Irani, Shayan
TI Endoscopic ultrasonography-guided gallbladder drainage using
lumen-apposing metal stents
SO TECHNIQUES AND INNOVATIONS IN GASTROINTESTINAL ENDOSCOPY
LA English
DT Article
DE Acute cholecystitis; Endoscopic ultrasound; Lumen-apposing metal stents;
EUS-guided gallbladder drainage; Cholecystostomy

AB Laparoscopic cholecystectomy is the standard of care for treatment of acute cholecystitis. When patients are deemed poor candidates for surgery, percutaneous cholecystostomy was historically performed for decompression of the gallbladder. Endoscopic transpapillary gallbladder drainage via endoscopic retrograde cholangiopancreatography (and endoscopic ultrasound (EUS) guided transmural gallbladder drainage (EUS-GBD) are 2 alternative techniques for gallbladder drainage. Over the last decade, there has been substantial progress in EUS-GBD technique, including use of covered self-expanding metal stents and lumen-apposing metal stents (LAMS). The unique design of LAMS results in apposition of gallbladder to duodenum/stomach and reduces the risk of adverse events. Recent studies comparing the outcomes of EUS-GBD using LAMS with percutaneous cholecystostomy and endoscopic transpapillary gallbladder drainage have reported results favoring EUS-GBD because of lower rates of adverse events. Furthermore, the use of LAMS for gallbladder drainage offers new opportunities for endoscopic management of gallbladder diseases through cholecystoscopy. Further studies are needed to assess optimal duration of stenting and safety of long-term stenting. (c) 2019 Published by Elsevier Inc.
C1 [Krishnamoorthi, Rajesh; Irani, Shayan] Virginia Mason Med Ctr, Digest Dis Inst, Seattle, WA 98101 USA.
C3 Virginia Mason Medical Center
RP Krishnamoorthi, R (corresponding author), Virginia Mason Med Ctr, Digest Dis Inst, Seattle, WA 98101 USA.
EM rajesh.krishnamoorthi@virginiamason.org
RI krishnamoorthi, rajesh/AAG-4265-2020
CR Anderloni A, 2019, GASTROINTEST ENDOSC, V89, P69, DOI [10.1016/j.gie.2018.04.1746, 10.1016/j.gie.2018.08.047]
Anderloni A, 2016, SURG ENDOSC, V30, P5200, DOI 10.1007/s00464-016-4894-x
Anderloni AA, 2018, GASTROINTEST ENDOSC, V87, pAB347
Baron TH, 2007, GASTROINTEST ENDOSC, V65, P735, DOI 10.1016/j.gie.2006.07.041
Baron TH, 2015, NEW ENGL J MED, V373, P357, DOI 10.1056/NEJMra1411372
Chan JHY, 2018, CLIN ENDOSC, V51, P150, DOI 10.5946/ce.2017.125
Chan SM, 2017, GASTROINTEST ENDOSC, V85, P1225, DOI 10.1016/j.gie.2016.10.014
Corral JE, 2019, SURG ENDOSC
Dollhopf M, 2017, GASTROINTEST ENDOSC, V86, P636, DOI 10.1016/j.gie.2017.02.027
Ge N, 2016, BMC GASTROENTEROL, V16, DOI 10.1186/s12876-016-0420-9
Higa JT, 2019, GASTROINTEST ENDOSC, V90, P483, DOI 10.1016/j.gie.2019.04.238
Imai H, 2016, GASTROINTEST ENDOSC, V84, P147, DOI 10.1016/j.gie.2015.12.024
Irani S, 2017, CLIN GASTROENTEROL H, V15, P738, DOI 10.1016/j.cgh.2016.12.021
Jang JW, 2012, GASTROENTEROLOGY, V142, P805, DOI 10.1053/j.gastro.2011.12.051
Garcia-Alonso FJ, 2018, ENDOSCOPY, V50, P386, DOI 10.1055/a-0581-9040
Kedia P, 2015, GASTROINTEST ENDOSC, V82, P1031, DOI 10.1016/j.gie.2015.03.1912
Krishnamoorthi R, 2019, SURG CLIN N AM, V99, P369, DOI 10.1016/j.suc.2018.12.002
Kunda R, 2016, SURG ENDOSC, V30, P5002, DOI 10.1007/s00464-016-4845-6
Law R, 2016, CLIN GASTROENTEROL H, V14, P476, DOI 10.1016/j.cgh.2015.10.026
Luk SWY, 2019, ENDOSCOPY, V51, P722, DOI 10.1055/a-0929-6603
MCGAHAN JP, 1989, RADIOLOGY, V173, P481, DOI 10.1148/radiology.173.2.2678261
Mohan BP, 2019, ENDOSC ULTRASOUND, V8, P241, DOI 10.4103/eus.eus_63_18
Mussetto A, 2018, ANN GASTROENTEROL, V31, P535, DOI 10.20524/aog.2018.0287
Nakai Y, 2014, DIGEST ENDOSC, V26, P86, DOI 10.1111/den.12152
Oh D, 2019, GASTROINTEST ENDOSC, V89, P289, DOI 10.1016/j.gie.2018.08.052

Siddiqui UD, 2018, GASTROENTEROLOGY, V154, P1911, DOI 10.1053/j.gastro.2017.12.046
Teoh AY, 2018, GASTROINTEST ENDOSC, V87, pAB333, DOI 10.1016/j.gie.2018.04.1716
Teoh AYB, 2014, ENDOSCOPY, V46, pE13, DOI 10.1055/s-0033-1359139
Teoh AYB, 2017, ENDOSCOPY, V49, P130, DOI 10.1055/s-0042-119036
Tsuyuguchi T, 2013, J HEPATO-BIL-PAN SCI, V20, P81, DOI 10.1007/s00534-012-0570-2
Tyberg A, 2018, J CLIN GASTROENTEROL, V52, P79, DOI 10.1097/MCG.0000000000000786
Walter D, 2016, GUT, V65, P6, DOI 10.1136/gutjnl-2015-309925
NR 32
TC 1
Z9 1
U1 0
U2 0
PU ELSEVIER SCIENCE INC
PI NEW YORK
PA STE 800, 230 PARK AVE, NEW YORK, NY 10169 USA
SN 2666-5107
EI 2590-0307
J9 TECH INNOVAT GASTROI
JI Tech. Innovation Gastrointes. Endoscopy
PD JAN
PY 2020
VL 22
IS 1
SI SI
BP 19
EP 23
DI 10.1016/j.tgie.2019.150652
PG 5
WC Gastroenterology & Hepatology
WE Emerging Sources Citation Index (ESCI)
SC Gastroenterology & Hepatology
GA VK9JD
UT WOS:000768800500006
DA 2026-05-09
ER

PT J
AU Lau, K
Rimba, M
Tripodi, G
Larghi, A
AF Lau, Kar

Rimba, Mihai
Tripodi, Giulia
Larghi, Alberto
TI Endoscopic Ultrasound Biliary Drainage in Pancreatic Cancer
SO JOURNAL OF GASTROINTESTINAL AND LIVER DISEASES
LA English
DT Review
DE pancreatic cancer; endoscopic ultrasound; biliary drainage; lumen
apposing metal stents
ID EUS-GUIDED CHOLEDOCHODUODENOSTOMY; INTERNATIONAL MULTICENTER;
OBSTRUCTION; ERCP; HEPATICOGASTROSTOMY; PALLIATION; EXPERIENCE
AB Endoscopic ultrasound (EUS) guided biliary drainage (BD) is an accepted salvage procedure in patients with distal malignant biliary obstruction (DMBO) when endoscopic retrograde cholangiopancreatography (ERCP) is unsuccessful. The potential advantages of EUS-BD include gastric or duodenal biliary access, utilization of novel biliary stents and stent placement away from the area of stenosis, resulting in longer stent patency. These features make EUS-BD very appealing as a primary procedure for biliary drainage. There is a growing body of evidence supporting the utilization of EUS as a primary drainage procedure instead of ERCP, with comparable outcomes.
C1 [Lau, Kar] Royal Stoke Univ Hosp, Dept Gastroenterol, Stoke On Trent, Staffs, England.
[Rimba, Mihai] Colentina Clin Hosp, Gastroenterol Dept, Bucharest, Romania.
[Rimba, Mihai] Carol Davila Univ Med, Dept Internal Med, Bucharest, Romania.
[Tripodi, Giulia; Larghi, Alberto] Fdn Policlin Univ A Gemelli IRCCS, Digest Endoscopy Unit, Rome, Italy.
C3 Royal Stoke University Hospital; Carol Davila University of Medicine &
Pharmacy; Catholic University of the Sacred Heart; IRCCS Policlinico
Gemelli
RP Larghi, A (corresponding author), Fdn Policlin Univ A Gemelli IRCCS, Digest Endoscopy Unit, Rome, Italy.
EM alberto.larghi@yahoo.it
RI Larghi, Alberto/AAC-7448-2022
CR Amato A, 2021, ENDOSCOPY, V53, P1037, DOI 10.1055/a-1324-7919
[Anonymous], 2022, Cancer Facts Figures 2021
Asadi H, 2016, CLIN RADIOL, V71, DOI 10.1016/j.crad.2016.05.013
Bang JY, 2018, GASTROINTEST ENDOSC, V88, P9, DOI 10.1016/j.gie.2018.03.012
Fabbri C, 2019, J GASTROINTEST LIVER, V28, P125, DOI 10.15403/jgld.2014.1121.281.eus
Frazzoni L, 2018, GASTROINTEST ENDOSC, V88, P571, DOI 10.1016/j.gie.2018.04.2335
Garcia-Sumalla A, 2022, TRIALS, V23, DOI 10.1186/s13063-022-06138-7
Garcia-Sumalla A, 2021, SURG ENDOSC, V35, P4873, DOI 10.1007/s00464-021-08435-9
Gaujoux S, 2021, HPB, V23, P154, DOI 10.1016/j.hpb.2020.06.001
Giovannini M, 2001, ENDOSCOPY, V33, P898, DOI 10.1055/s-2001-17324
Guo JT, 2016, GASTROENT RES PRACT, V2016, DOI 10.1155/2016/9469472
Han SY, 2019, SCI REP-UK, V9, DOI 10.1038/s41598-019-52993-x
Hayat U, 2022, ENDOSC ULTRASOUND, V11, P4, DOI 10.4103/EUS-D-21-00009
Hedjoudje A, 2019, UNITED EUR GASTROENT, V7, P60, DOI 10.1177/2050640618808147
Heedman PA, 2018, SCAND J SURG, V107, P48, DOI 10.1177/1457496917731192

Imai H, 2016, GASTROINTEST ENDOSC, V84, P147, DOI 10.1016/j.gie.2015.12.024
Issa D, 2021, ENDOSCOPY, V53, P827, DOI 10.1055/a-1259-0349
Itonaga M, 2023, DIGEST ENDOSC, V35, P302, DOI 10.1111/den.14428
Kedia P, 2013, CLIN ENDOSC, V46, P543, DOI 10.5946/ce.2013.46.5.543
Khan MA, 2016, DIGEST DIS SCI, V61, P684, DOI 10.1007/s10620-015-3933-0
Khashab MA, 2016, ENDOSC INT OPEN, V4, pE487, DOI 10.1055/s-0042-102648
Kim R, 2010, J GASTROINTEST SURG, V14, P1159, DOI 10.1007/s11605-010-1213-z
Koga T, 2022, DEN OPEN, V2, DOI 10.1002/deo2.56
Kunda R, 2016, SURG ENDOSC, V30, P5002, DOI 10.1007/s00464-016-4845-6
Larghi A, 2019, ENDOSC ULTRASOUND, V8, P217, DOI 10.4103/eus.eus_61_18
Lee TH, 2016, CLIN GASTROENTEROL H, V14, P1011, DOI 10.1016/j.cgh.2015.12.032
Li JS, 2022, FRONT SURG, V9, DOI 10.3389/fsurg.2022.811005
Makris GC, 2021, CARDIOVASC INTER RAD, V44, P1625, DOI 10.1007/s00270-021-02888-0
Minaga K, 2022, DIGEST ENDOSC, V34, P79, DOI 10.1111/den.14149
Moole H, 2016, CAN J GASTROENTEROL, V2016, DOI 10.1155/2016/4726078
Nakai Y, 2020, J GASTROEN HEPATOL, V35, P967, DOI 10.1111/jgh.14955
Ogura T, 2021, DEN OPEN, V1, DOI 10.1002/deo2.8
Ogura T, 2015, ENDOSCOPY, V47, pE583, DOI 10.1055/s-0034-1393381
Paik WH, 2018, AM J GASTROENTEROL, V113, P987, DOI 10.1038/s41395-018-0122-8
Park JK, 2018, GASTROINTEST ENDOSC, V88, P277, DOI 10.1016/j.gie.2018.03.015
Poincloux L, 2015, ENDOSCOPY, V47, P794, DOI [10.1055/s-0035-1545399, 10.1055/s-0034-1391988]
Principe DR, 2021, FRONT ONCOL, V11, DOI 10.3389/fonc.2021.688377
Rimbas M, 2021, ENDOSC INT OPEN, V09, pE1801, DOI 10.1055/a-1526-1208
Rimbas M, 2021, ENDOSCOPY, V53, P873, DOI 10.1055/a-1299-1495
Ryou M, 2020, GASTROINTEST ENDOSC, V91, P178, DOI 10.1016/j.gie.2019.07.035
Sharaiha RZ, 2017, GASTROINTEST ENDOSC, V85, P904, DOI 10.1016/j.gie.2016.12.023
Sut M, 2010, J PALLIAT MED, V13, P1311, DOI 10.1089/jpm.2010.0205
Tyberg Amy, 2023, J Clin Gastroenterol, V57, P962, DOI 10.1097/MCG.0000000000001795
Tyberg A, 2022, ENDOSC ULTRASOUND, V11, P38, DOI 10.4103/EUS-D-21-00006
Tyberg A, 2021, GASTROINTEST ENDOSC, V93, pAB266, DOI 10.1016/j.gie.2021.03.568
Uberoi R, 2012, CARDIOVASC INTER RAD, V35, P127, DOI 10.1007/s00270-011-0103-4
Uemura RS, 2018, J CLIN GASTROENTEROL, V52, P123, DOI 10.1097/MCG.0000000000000948
van der Merwe SW, 2022, ENDOSCOPY, V54, P185, DOI 10.1055/a-1717-1391
Versteijne E, 2022, J CLIN ONCOL, V40, P1220, DOI 10.1200/JCO.21.02233
Vila JJ, 2012, GASTROINTEST ENDOSC, V76, P1133, DOI 10.1016/j.gie.2012.08.001
Wang KX, 2016, GASTROINTEST ENDOSC, V83, P1218, DOI 10.1016/j.gie.2015.10.033
Zeuge Ulf, 2014, Case Rep Gastroenterol, V8, P358, DOI 10.1159/000369298
Zhao X, 2022, CLINICS, V77
NR 53
TC 2
Z9 2
U1 0

U2 2
PU MEDICAL UNIV PRESS
PI CLUJ-NAPOCA
PA 3RD MEDICAL CLINIC, STR CROITORILOR NO 19-21, CLUJ-NAPOCA, 400162,
ROMANIA
SN 1841-8724
EI 1842-1121
J9 J GASTROINTEST LIVER
JI J. Gastrointest. Liver Dis.
PD DEC
PY 2023
VL 32
IS 4
BP 545
EP 553
DI 10.15403/jgld-4922
PG 9
WC Gastroenterology & Hepatology
WE Science Citation Index Expanded (SCI-EXPANDED)
SC Gastroenterology & Hepatology
GA HF8Q8
UT WOS:001158173500018
PM 38147611
OA gold
DA 2026-05-09
ER

PT J
AU Ahmed, O
Ogura, T
Eldahrouty, A
Khalaf, H
Mohammed, E
Okasha, H
Sameer, A
Abdelaal, U
Higuchi, K
AF Ahmed, Omar
Ogura, Takeshi
Eldahrouty, Ali
Khalaf, Hanaa
Mohammed, Ehab

Okasha, Hussein
Sameer, Ayat
Abdelaal, Usama
Higuchi, Kazuhide
TI Endoscopic ultrasound-guided gallbladder drainage: Results of long-term follow-up
SO SAUDI JOURNAL OF GASTROENTEROLOGY
LA English
DT Article
DE Cholecystitis; endoscopic ultrasond-guided biliary drainage; endoscopic ultrasound-; guided gallbladder drainage; endoscopic
ID DELAYED LAPAROSCOPIC CHOLECYSTECTOMY; EXPANDABLE METAL STENT; HIGH-RISK PATIENTS; ACUTE CHOLECYSTITIS; PERCUTANEOUS CHOLECYSTOSTOMY; PLACEMENT; OUTCOMES; REMOVAL; SAFETY
AB Background/Aim: Recently, endoscopic ultrasound-guided gallbladder drainage (EUS-GBD) has emerged using a self-expandable metallic stent (SEMS). The aim of the study was to evaluate the long-term outcomes of this procedure. In addition, the efficacy and safety of EUS-GBD with SEMS were assessed.

Patients and Methods: Thirteen consecutive patients who underwent EUS-GBD for acute cholecystitis between February 2014 and September 2016 were included in this retrospective study. EUS-GBD was performed under the guidance of EUS and fluoroscopy, through puncturing the gallbladder with a needle, inserting a guidewire, dilating the puncture hole, and placing a SEMS.

Results: The rates of technical success, functional success, and adverse events were 100%, 92.3% and 7.7%, respectively. The median procedure time was 26.9 min (range 19-42 min). The median follow-up time was 240 days (range 14-945 days) and during this follow-up period recurrence of cholecystitis was observed in one patient (7.7%).

Conclusion: EUS-GBD with a SEMS is a possible alternative treatment for acute cholecystitis in high surgical risk patients. Long-term outcomes after EUS-GBD were promising.
C1 [Ahmed, Omar; Ogura, Takeshi; Higuchi, Kazuhide] Osaka Med Coll, Dept Internal Med, Osaka, Japan.

[Ahmed, Omar; Eldahrouty, Ali; Khalaf, Hanaa; Mohammed, Ehab; Sameer, Ayat] Minia Univ Hosp, Dept Trop Med, Al Minya, Egypt.

[Okasha, Hussein] Cairo Univ, Dept Internal Med, Cairo, Egypt.

[Abdelaal, Usama] Sohag Univ Hosp, Dept Internal Med, Sohag, Egypt.

C3 Osaka Medical & Pharmaceutical University; Egyptian Knowledge Bank (EKB); Minia University; Egyptian Knowledge Bank (EKB); Cairo University; Egyptian Knowledge Bank (EKB); Sohag University

RP Ogura, T (corresponding author), Osaka Med Coll, Dept Internal Med, 1-1 Daigakuchou, Takatsuki, Osaka 4648681, Japan.

EM oguratakeshi0411@yahoo.co.jp

RI Ogura, Takeshi/AAU-5643-2021
CR Anderloni A, 2016, SURG ENDOSC, V30, P5200, DOI 10.1007/s00464-016-4894-x
Choi JH, 2015, DIGEST ENDOSC, V27, P1, DOI 10.1111/den.12386
Choi JH, 2014, ENDOSCOPY, V46, P656, DOI 10.1055/s-0034-1365720
Cotton PB, 2010, GASTROINTEST ENDOSC, V71, P446, DOI 10.1016/j.gie.2009.10.027

De la Serna-Higuera C, 2013, GASTROINTEST ENDOSC, V61, P601
Dollhopf M, 2017, GASTROINTEST ENDOSC, V86, P636, DOI 10.1016/j.gie.2017.02.027
Fitz-Henry J, 2011, ANN ROY COLL SURG, V93, P185, DOI 10.1308/rcsann.2011.93.3.185a
Ito K, 2004, AM J ROENTGENOL, V183, P193, DOI 10.2214/ajr.183.1.1830193
Itoi T, 2012, GASTROINTEST ENDOSC, V75, P870, DOI 10.1016/j.gie.2011.10.020
Itoi T, 2011, J HEPATO-BIL-PAN SCI, V18, P282, DOI 10.1007/s00534-010-0310-4
Itoi T, 2010, GASTROINTEST ENDOSC, V71, P1038, DOI 10.1016/j.gie.2010.01.026
Jang JW, 2012, GASTROENTEROLOGY, V142, P805, DOI 10.1053/j.gastro.2011.12.051
Jang JW, 2011, GASTROINTEST ENDOSC, V74, P176, DOI 10.1016/j.gie.2011.03.1120
Kamata K, 2017, WORLD J GASTROENTERO, V23, P661, DOI 10.3748/wjg.v23.i4.661
Kimura Y, 2013, J HEPATO-BIL-PAN SCI, V20, P8, DOI 10.1007/s00534-012-0564-0
Kiviniemi H, 1998, INT SURG, V83, P299
Lo CM, 1998, ANN SURG, V227, P461, DOI 10.1097/00000658-199804000-00001
MCGAHAN JP, 1989, RADIOLOGY, V173, P481, DOI 10.1148/radiology.173.2.2678261
McKay A, 2012, SURG ENDOSC, V26, P1343, DOI 10.1007/s00464-011-2035-0
Mönkemüller K, 2013, ENDOSCOPY, V45, pE164, DOI 10.1055/s-0032-1325776
Ogura T, 2014, ENDOSCOPY, V46, pE75, DOI 10.1055/s-0033-1359135
Peñas-Herrero I, 2015, J HEPATO-BIL-PAN SCI, V22, P35, DOI 10.1002/jhbp.182
Shayan I, 2015, GASTROINTEST ENDOSC, V82, P1110
Song TJ, 2010, GASTROINTEST ENDOSC, V71, P634, DOI 10.1016/j.gie.2009.11.024
Spira RM, 2002, AM J SURG, V183, P62, DOI 10.1016/S0002-9610(01)00849-2
Takagi W, 2016, THER ADV GASTROENTER, V9, P19, DOI 10.1177/1756283X15609285
Turner BG, 2013, ENDOSCOPY, V45, pE114, DOI 10.1055/s-0032-1325968
Walter D, 2016, GUT, V65, P6, DOI 10.1136/gutjnl-2015-309925
Yi NJ, 2006, MINIM INVASIV THER, V15, P159, DOI 10.1080/13645700600760044
NR 29
TC 16
Z9 19
U1 0
U2 0
PU MEDKNOW PUBLICATIONS & MEDIA PVT LTD
PI MUMBAI
PA B-9, KANARA BUSINESS CENTRE, OFF LINK RD, GHAKTOPAR-E, MUMBAI, 400075, INDIA
SN 1319-3767
EI 1998-4049
J9 SAUDI J GASTROENTERO
JI Saudi J. Gastroenterol.
PD MAY-JUN
PY 2018
VL 24
IS 3

BP 183
EP 188
DI 10.4103/sjg.SJG_506_17
PG 6
WC Gastroenterology & Hepatology
WE Science Citation Index Expanded (SCI-EXPANDED)
SC Gastroenterology & Hepatology
GA GH2BP
UT WOS:000433206900008
PM 29652030
OA Green Submitted, gold
DA 2026-05-09
ER

PT J
AU Rajadurai, A
Pu, LZCT
Cameron, R
Tagkalidis, P
Holt, B
Bassan, M
Gupta, S
Croagh, D
Swan, M
Saxena, P
Efthymiou, M
Vaughan, R
Chandran, S
AF Rajadurai, Anton
Pu, Leonardo Zorron Cheng Tao
Cameron, Rees
Tagkalidis, Peter
Holt, Bronte
Bassan, Milan
Gupta, Saurabh
Croagh, Daniel
Swan, Michael
Saxena, Payal
Efthymiou, Marios
Vaughan, Rhys
Chandran, Sujievvan
CA ANZEC

TI Endoscopic ultrasound-guided gallbladder and bile duct drainage with lumen apposing metal stent: A large multicenter cohort (with videos)

SO JOURNAL OF GASTROENTEROLOGY AND HEPATOLOGY

LA English

DT Article

DE biliary drainage; cholecystitis; endoscopic ultrasound; gallbladder drainage; jaundice; lumen-apposing metal stents; malignant biliary obstruction

ID LONG-TERM OUTCOMES; BILIARY OBSTRUCTION; PLACEMENT

AB Background and Aim Cholecystectomy and endoscopic retrograde cholangiopancreatography are the gold standard for managing acute cholecystitis and malignant biliary obstruction, respectively. Recent advances in therapeutic endoscopic ultrasound (EUS) have provided alternatives for managing patients in whom these approaches fail, namely, EUS-guided gallbladder drainage (EUS-GB) and EUS-guided bile duct drainage (EUS-BD). We aimed to assess the technical and clinical success of these techniques in the largest multicenter cohort published to date. Methods A retrospective, multicenter, observational study involving 17 centers across Australia and New Zealand was conducted. All patients who had EUS-GB or EUS-BD performed in a participating center using a lumen apposing metal stent between 2016 and 2020 were included. Primary outcome was technical success, defined as intra-procedural successful drainage. Secondary outcomes included clinical success and 30-day mortality. Results One hundred and fifteen patients underwent EUS-GB (n = 49) or EUS-BD (n = 66). EUS-GB was technically successful in 47 (95.9%) while EUS-BD was successful in 60 (90.9%). All failed cases were due to maldeployment of the distal flange outside of the targeted lumen. Clinical success of EUS-GB was achieved in 39 (79.6%). No patients required subsequent cholecystectomy. Clinical success of EUS-BD was achieved in 52 (78.8 %). Thirty-day mortality was 14.3% for EUS-GB and 12.1% for EUS-BD. Conclusions EUS-guided gallbladder drainage and EUS-BD are promising alternatives for managing nonsurgical candidates with cholecystitis and malignant biliary obstruction following failed endoscopic retrograde pancreatography. Both techniques delivered high technical success with acceptable clinical success. Further research is needed to investigate the gap between technical and clinical success.

C1 [Rajadurai, Anton; Pu, Leonardo Zorron Cheng Tao; Cameron, Rees; Tagkalidis, Peter; Holt, Bronte; Bassan, Milan; Gupta, Saurabh; Croagh, Daniel; Swan, Michael; Saxena, Payal; Efthymiou, Marios; Vaughan, Rhys; Chandran, Sujievvan] Austin Hlth, Dept Gastroenterol & Hepatol, 8th Floor Harold Stokes Bldg,145 Studley Rd, Heidelberg, Vic 3084, Australia.

C3 Florey Institute of Neuroscience & Mental Health; Howard Florey Institute Affiliates; Austin Research Institute

RP Pu, LZCT (corresponding author), Austin Hlth, Dept Gastroenterol & Hepatol, 8th Floor Harold Stokes Bldg,145 Studley Rd, Heidelberg, Vic 3084, Australia.

EM leo.zorronchengtaopu@austin.org.au

RI Vaughan, Rhys/D-3954-2017; Gupta, Saurabh/KIB-1858-2024; Zorron Cheng Tao Pu, Leonardo/N-6103-2016

OI Holt, Bronte/0000-0002-1387-6525; Gupta, Saurabh/0009-0008-2128-6550;

CR de Lima SLA, 2015, INT J HEPATOBILIARY, V5, P35, DOI 10.5348/ijhpd-2015-32-CR-7
Alvino DML, 2017, J GASTROINTEST SURG, V21, P761, DOI 10.1007/s11605-017-3375-4
Anderloni A, 2018, ANN GASTROENTEROL, V31, P735, DOI 10.20524/aog.2018.0299
Artifon ELA, 2006, AM J GASTROENTEROL, V101, P2031, DOI 10.1111/j.1572-0241.2006.00764.x
Baron TH, 2007, GASTROINTEST ENDOSC, V65, P735, DOI 10.1016/j.gie.2006.07.041
Pu LZCT, 2016, GASTROENT RES PRACT, V2016, DOI 10.1155/2016/3296801

Coelen RJS, 2018, LANCET GASTROENTEROL, V3, P681, DOI 10.1016/S2468-1253(18)30234-6
Hathorn KE, 2019, ENDOSC INT OPEN, V7, pE1432, DOI 10.1055/a-0990-9488
Itoi T, 2012, GASTROINTEST ENDOSC, V75, P870, DOI 10.1016/j.gie.2011.10.020
Kunda R, 2016, SURG ENDOSC, V30, P5002, DOI 10.1007/s00464-016-4845-6
Loozen CS, 2018, BMJ-BRIT MED J, V363, DOI 10.1136/bmj.k3965
Minaga K, 2019, DIGEST ENDOSC, V31, P575, DOI 10.1111/den.13406
Nikfarjam M, 2014, ANZ J SURG, V84, P943, DOI 10.1111/ans.12313
Patil R, 2016, ANN GASTROENTEROL, V29, P162, DOI 10.20524/aog.2016.0001
Rinninella E, 2015, GASTROINTEST ENDOSC, V82, P1039, DOI 10.1016/j.gie.2015.04.006
Teoh AYB, 2020, GUT, V69, P1085, DOI 10.1136/gutjnl-2019-319996
Tsuchiya T, 2018, GASTROINTEST ENDOSC, V87, P1138, DOI 10.1016/j.gie.2017.08.017
NR 17
TC 7
Z9 7
U1 0
U2 1
PU WILEY
PI HOBOKEN
PA 111 RIVER ST, HOBOKEN 07030-5774, NJ USA
SN 0815-9319
EI 1440-1746
J9 J GASTROEN HEPATOL
JI J. Gastroenterol. Hepatol.
PD JAN
PY 2022
VL 37
IS 1
SI SI
BP 179
EP 189
DI 10.1111/jgh.15688
EA OCT 2021
PG 11
WC Gastroenterology & Hepatology
WE Science Citation Index Expanded (SCI-EXPANDED)
SC Gastroenterology & Hepatology
GA YK9QA
UT WOS:000704561600001
PM 34562319
DA 2026-05-09
ER

PT J
AU Anderloni, A
Fugazza, A
Maroni, L
Troncone, E
Milani, O
Cappello, A
Alkandari, A
Repici, A
AF Anderloni, Andrea
Fugazza, Alessandro
Maroni, Luca
Troncone, Edoardo
Milani, Olivia
Cappello, Annalisa
Alkandari, Asma
Repici, Alessandro
TI Endoscopic ultrasound-guided gallbladder drainage by transduodenal
lumen-apposing metal stent in a patient with Roux-en-Y reconstruction
SO ANNALS OF GASTROENTEROLOGY
LA English
DT Article
DE Endoscopic gallbladder drainage; endoscopic ultrasound; altered anatomy;
lumen-apposing metal stent
ID PERCUTANEOUS CHOLECYSTOSTOMY; OUTCOMES
AB Endoscopic ultrasound-guided gallbladder drainage (EUS-GBD) is increasingly recognized as an effective and safe treatment option in patients with acute cholecystitis (AC) who are deemed unfit for cholecystectomy. Recent studies suggest that an endoscopic approach to biliary decompression may reduce patient discomfort and procedure-related costs compared with the standard percutaneous transhepatic GBD, while maintaining high rates of technical and clinical success. Here we present a case of a EUS-GBD in an elderly patient with AC and with altered anatomy due to a previous partial gastrectomy with Roux-en-Y reconstruction for gastric cancer. The linear array echoendoscope was carefully advanced within the afferent limb until EUS gallbladder visualization was achieved. Transmural GBD was then performed by placement of an electrocautery-enhanced lumen-apposing metal stent. If alternative treatments are precluded, EUS-GBD in patients with AC and altered anatomy is a feasible and effective therapeutic option when performed by experienced endoscopists.
C1 [Anderloni, Andrea; Fugazza, Alessandro; Maroni, Luca; Troncone, Edoardo; Cappello, Annalisa; Repici, Alessandro] Humanitas Res Hosp, Div Gastroenterol, Digest Endoscopy Unit, Via Manzoni 56, I-20089 Rozzano, MI, Italy.
[Maroni, Luca] Univ Politecn Marche, Dept Gastroenterol & Hepatol, Ancona, AN, Italy.
[Milani, Olivia] Osped Maggiore Policlin, IRCCS Fdn Ca Granda, Dept Internal Med, Milan, MI, Italy.
[Alkandari, Asma] Alamiri Hosp, Thanyan Alglian Ctr Gastroenterol & Hematol, Kuwait, Kuwait.
[Repici, Alessandro] Humanitas Univ, Rozzano, MI, Italy.
[Troncone, Edoardo] Univ Rome Tor Vegrata, Dept Syst Med, Rome, Italy.
C3 IRCCS Humanitas Research Hospital; Marche Polytechnic University; IRCCS

Ca Granda Ospedale Maggiore Policlinico; IRCCS Humanitas Research
Hospital; Humanitas University
RP Anderloni, A (corresponding author), Humanitas Res Hosp, Div Gastroenterol, Digest Endoscopy Unit, Via Manzoni 56, I-20089 Rozzano, MI, Italy.
EM andrea.anderloni@humanitas.it
RI Repici, Alessandro/HFH-8162-2022; Fugazza, Alessandro/ABG-9381-2021;
Anderloni, Andrea/ABE-9139-2020; Troncone, Edoardo/AHD-9276-2022;
MARONI, LUCA/A-8153-2011
OI Repici, Alessandro/0000-0002-1621-6450; Cappello,
Annalisa/0000-0002-9248-9549; Fugazza, Alessandro/0000-0003-0485-4903;
Anderloni, Andrea/0000-0002-1021-0031; Troncone,
Edoardo/0000-0003-4520-6865; MARONI, LUCA/0000-0003-4707-7374
CR Anderloni A, 2017, ENDOSC INT OPEN, V5, pE1100, DOI 10.1055/s-0043-118828
Anderloni A, 2016, SURG ENDOSC, V30, P5200, DOI 10.1007/s00464-016-4894-x
Baron TH, 2015, NEW ENGL J MED, V373, P357, DOI 10.1056/NEJMra1411372
Dollhopf M, 2017, GASTROINTEST ENDOSC, V86, P636, DOI 10.1016/j.gie.2017.02.027
Irani S, 2017, CLIN GASTROENTEROL H, V15, P738, DOI 10.1016/j.cgh.2016.12.021
Okamoto K, 2018, J HEPATO-BIL-PAN SCI, V25, P55, DOI 10.1002/jhbp.516
Saumoy M, 2018, ENDOSC ULTRASOUND, V7, P97, DOI 10.4103/eus.eus_9_18
Teoh AYB, 2017, ENDOSCOPY, V49, P130, DOI 10.1055/s-0042-119036
Tyberg A, 2018, J CLIN GASTROENTEROL, V52, P79, DOI 10.1097/MCG.0000000000000786
Yeo CSW, 2016, J HEPATO-BIL-PAN SCI, V23, P65, DOI 10.1002/jhbp.304
NR 10
TC 10
Z9 10
U1 0
U2 0
PU HELLENIC SOC GASTROENTEROLOGY
PI ATHENS
PA DEMOKRATIAS AVE 67, ATHENS, 15451, GREECE
SN 1108-7471
EI 1792-7463
J9 ANN GASTROENTEROL
JI Ann. Gastroenterol.
PY 2019
VL 32
IS 5
BP 522
EP 524
DI 10.20524/aog.2019.0384
PG 3
WC Gastroenterology & Hepatology

WE Emerging Sources Citation Index (ESCI)
SC Gastroenterology & Hepatology
GA IU1BT
UT WOS:000483312500014
PM 31474800
OA gold
DA 2026-05-09
ER

PT J
AU Nakaji, S
Hirata, N
Mikata, R
Kobayashi, M
Shiratori, T
Ogasawara, S
Ooka, Y
Tsuyuguchi, T
Yamaguchi, T
Yokosuka, O
AF Nakaji, So
Hirata, Nobuto
Mikata, Rintaro
Kobayashi, Masayoshi
Shiratori, Toshiyasu
Ogasawara, Sadahisa
Ooka, Yoshihiko
Tsuyuguchi, Toshio
Yamaguchi, Taketo
Yokosuka, Osamu
TI Clinical outcomes of endoscopic ultrasound-guided ethanol injection for
hepatocellular carcinoma in the caudate lobe
SO ENDOSCOPY INTERNATIONAL OPEN
LA English
DT Article
ID PERCUTANEOUS RADIOFREQUENCY ABLATION; CHEMOEMBOLIZATION; LIVER
AB Background and study aims: Accurately puncturing hepatocellular carcinomas (HCC) that arise from the caudate lobe is generally considered to be technically difficult. We conducted a retrospective study to evaluate the feasibility and safety (the therapeutic outcomes and adverse events) of endoscopic ultrasound (EUS)-guided ethanol injection as a novel treatment for HCC in the caudate lobe.

Patients and methods: Twelve patients with early-stage HCC of the caudate lobe that were treated with EUS-guided ethanol injection at two tertiary referral centers were reviewed retrospectively. To evaluate the therapeutic effect of the

treatment, a local control curve and an overall survival curve were constructed using the Kaplan-Meier method.

Results: The mean follow-up duration was 31.0 months. The 1-year local control rate was 80.2%, and recurrent lesions developed in 2 cases (after 3 and 9 months, respectively). The overall survival rate was 91.7%, 75.0%, and 53.3% at 1, 2, and 3 years, respectively. Concerning procedure-related adverse events (AEs), 2 patients suffered episodes of fever lasting a few days; however, no serious AEs occurred.

Conclusions: EUS-guided ethanol injection could be a useful treatment for early-stage HCC in the caudate lobe because of its simplicity and reduced invasiveness.
C1 [Nakaji, So; Hirata, Nobuto; Kobayashi, Masayoshi; Shiratori, Toshiyasu] Kameda Med Ctr, Dept Gastroenterol, Kamogawa, Japan.

[Mikata, Rintaro; Ogasawara, Sadahisa; Ooka, Yoshihiko; Tsuyuguchi, Toshio; Yokosuka, Osamu] Chiba Univ, Grad Sch Med, Dept Gastroenterol & Nephrol, Chiba, Japan.

[Yamaguchi, Taketo] Chiba Canc Ctr, Dept Gastroenterol, Chiba, Japan.

C3 Chiba University; Chiba Cancer Center

RP Nakaji, S (corresponding author), 929 Higashi Cho, Kamogawa City, Chiba 2968602, Japan.

EM nakajisou1978@yahoo.co.jp

RI Ogasawara, Sadahisa/GRF-7061-2022
CR Di Matteo F, 2011, GASTROINTEST ENDOSC, V73, P632, DOI 10.1016/j.gie.2010.08.019

Fu Y, 2015, INT J CLIN EXP MED, V8, P10388

Günter E, 2003, GASTROINTEST ENDOSC, V57, P113, DOI 10.1067/mge.2003.39

Hatanaka T, 2015, ACTA GASTRO-ENT BELG, V78, P267

Jiang K, 2013, EJSO-EUR J SURG ONC, V39, P1236, DOI 10.1016/j.ejso.2013.08.002

Jürgensen C, 2006, GASTROINTEST ENDOSC, V63, P1059, DOI 10.1016/j.gie.2005.10.034

Kanda Y, 2013, BONE MARROW TRANSPL, V48, P452, DOI 10.1038/bmt.2012.244

Kariyama K, 2011, AM J ROENTGENOL, V197, pW571, DOI 10.2214/AJR.11.6893

Kim HC, 2010, RADIOLOGY, V257, P278, DOI 10.1148/radiol.10100105

Nakaji S, 2012, Endoscopy, V44 Suppl 2 UCTN, pE380, DOI 10.1055/s-0032-1309918

Nakaji S, 2015, ENDOSCOPY, V47, pE558, DOI 10.1055/s-0034-1393394

Nishigaki Y, 2013, HEPATOL RES, V43, P467, DOI 10.1111/j.1872-034X.2012.01095.x

Sun S, 2006, ENDOSCOPY, V38, P399, DOI 10.1055/s-2006-925253

Tanaka S, 2005, AM J SURG, V190, P451, DOI 10.1016/j.amjsurg.2004.12.005
NR 14

TC 14

Z9 16

U1 0

U2 3

PU GEORG THIEME VERLAG KG

PI STUTTGART

PA RUDIGERSTR 14, D-70469 STUTTGART, GERMANY

SN 2364-3722

EI 2196-9736

J9 ENDOSC INT OPEN

JI Endosc. Int. Open

PD OCT
PY 2016
VL 4
IS 10
BP E1111
EP E1115
DI 10.1055/s-0042-116146
PG 5
WC Gastroenterology & Hepatology; Surgery
WE Emerging Sources Citation Index (ESCI)
SC Gastroenterology & Hepatology; Surgery
GA EA0XQ
UT WOS:000386313200017
PM 27747288
OA Green Submitted, gold
DA 2026-05-09
ER

PT J
AU Sakamoto, H
Kitano, M
Komaki, T
Imai, H
Kamata, K
Kudo, M
AF Sakamoto, Hiroki
Kitano, Masayuki
Komaki, Takamitsu
Imai, Hajime
Kamata, Ken
Kudo, Masatoshi
TI Endoscopic Ultrasound-Guided Neurolysis in Pancreatic Cancer
SO PANCREATOLOGY
LA English
DT Article
DE Abdominal pain; Celiac plexus neurolysis; Endoscopic ultrasound;
EUS-guided broad plexus neurolysis; EUS-guided celiac ganglia
neurolysis; Pancreatic cancer
ID CELIAC PLEXUS BLOCK; EUS; PAIN; GANGLIA; NEEDLE; COMPLICATIONS
AB Abdominal pain in patients with pancreatic cancer is a common symptom that is often difficult to manage. Opioids are frequently used in an attempt to mitigate pain; however, side effects may develop. Celiac plexus neurolysis (CPN) affords effective pain control in patients with pancreatic cancer and is not associated with opioid side effects. Endoscopic

ultrasound (EUS)-guided CPN has demonstrated safety and efficacy due to real-time imaging and anterior access to the celiac plexus from the posterior gastric wall, thereby avoiding complications related to the puncture of spinal nerves, arteries and the diaphragm, and is now practiced widely. Furthermore, two new techniques of EUS-guided neurolysis for abdominal pain management in pancreatic cancer patients have recently been developed. The first technique is EUS-guided celiac ganglia neurolysis (EUS-CGN) in which EUS facilitates CGN by enabling direct injection into the individual celiac ganglion, and the second technique is EUS-guided broad plexus neurolysis (EUS-BPN) which extends over the superior mesenteric artery. This review provides evidence for the efficacy of EUS-CPN. Particular attention is paid to the two new techniques of EUS-guided neurolysis, EUS-CGN and EUS-BPN. Copyright (C) 2011 S. Karger AG, Basel and IAP
C1 [Sakamoto, Hiroki; Kitano, Masayuki; Komaki, Takamitsu; Imai, Hajime; Kamata, Ken; Kudo, Masatoshi] Kinki Univ, Dept Gastroenterol & Hepatol, Sch Med, Osakasayama, Japan.
C3 Kindai University (Kinki University)
RP Sakamoto, H (corresponding author), Kinki Univ, Div Gastroenterol & Hepatol, Dept Internal Med, Sch Med, 377-2 Ohnohigashi, Osaka 5898511, Japan.
EM hiroki.sakamoto@nifty.com
RI Kudo, Masatoshi/AAA-9744-2019
OI Kudo, Masatoshi/0000-0002-4102-3474
FU Grants-in-Aid for Scientific Research [22590764] Funding Source: KAKEN
CR Abdalla EK, 1999, J GASTROINTEST SURG, V3, P668, DOI 10.1016/S1091-255X(99)80091-2
[Anonymous], 1990, Cancer pain relief and palliative care: report of a WHO expert committee [meeting held in Geneva from 3 to 10 July 1989]
BRESCIA FJ, 1992, J CLIN ONCOL, V10, P149, DOI 10.1200/JCO.1992.10.1.149
Brescia Frank J, 2004, Cancer Control, V11, P39
Caratozzolo M, 1997, SURG ENDOSC-ULTRAS, V11, P239, DOI 10.1007/s004649900334
Ceyhan GO, 2008, BEST PRACT RES CL GA, V22, P31, DOI 10.1016/j.bpg.2007.10.016
Collins D, 2006, ENDOSCOPY, V38, P935, DOI 10.1055/s-2006-944734
DAVIES DD, 1993, J ROY SOC MED, V86, P264
DeCicco M, 1997, ANESTHESIOLOGY, V87, P1301, DOI 10.1097/00000542-199712000-00007
EISENBERG E, 1995, ANESTH ANALG, V80, P290, DOI 10.1097/00000539-199502000-00015
EISENBERG E, 1995, ANESTH ANALG, V1, P213
Gleeson FC, 2007, ENDOSCOPY, V39, P620, DOI 10.1055/s-2007-966337
Gress F, 1999, AM J GASTROENTEROL, V94, P900
GRESS F, 1997, GASTROINTEST ENDOSC, V45, pAB173
Gunaratnam NT, 2001, GASTROINTEST ENDOSC, V54, P316, DOI 10.1067/mge.2001.117515
Ha Tae-In, 2008, Korean Journal of Internal Medicine, V23, P5, DOI 10.3904/kjim.2008.23.1.5
Hoffman BJ, 2002, GASTROINTEST ENDOSC, V56, pS26, DOI 10.1067/mge.2002.127759
Jacobson BC, 2005, GASTROINTEST ENDOSC, V61, P8, DOI 10.1016/S0016-5107(04)02393-4
Kappis M, 1914, Verh. Dtsch. Ges. Chir, V43, P87
Levy M, 2006, AM J GASTROENTEROL, V101, P1787, DOI 10.1111/j.1572-0241.2006.00685.x
Levy MJ, 2008, AM J GASTROENTEROL, V103, P98, DOI 10.1111/j.1572-0241.2007.01607.x
LILLEMOE KD, 1993, ANN SURG, V217, P447, DOI 10.1097/00000658-199305010-00004
MATAMALA AM, 1989, BRIT J ANAESTH, V62, P637, DOI 10.1093/bja/62.6.637
Montero Matamala A, 1988, PAIN, V34, P285

Sahai AV, 2009, AM J GASTROENTEROL, V104, P326, DOI 10.1038/ajg.2008.64
Sakamoto H., 2006, DIGEST ENDOSC, V18, P206
Sakamoto H, 2010, AM J GASTROENTEROL, V105, P2599, DOI 10.1038/ajg.2010.339
Sakamoto H, 2009, J GASTROENTEROL, V44, P432, DOI 10.1007/s00535-009-0004-3
Sakamoto H, 2009, J GASTROEN HEPATOL, V24, P384, DOI 10.1111/j.1440-1746.2008.05636.x
Schmulewitz N, 2003, ENDOSCOPY, V35, pS49
VENTAFRIDDA V, 1987, CANCER-AM CANCER SOC, V59, P850, DOI 10.1002/1097-0142(19870215)59:4<850::AID-CNCR2820590432>3.0.CO;2-1
Wang PJ, 2006, ABDOM IMAGING, V31, P710, DOI 10.1007/s00261-006-9153-5
Wiersema M, 1996, GASTROINTEST ENDOSC, V43, P565
NR 33
TC 27
Z9 28
U1 0
U2 11
PU KARGER
PI BASEL
PA ALLSCHWILERSTRASSE 10, CH-4009 BASEL, SWITZERLAND
SN 1424-3903
J9 PANCREATOLOGY
JI Pancreatology
PY 2011
VL 11
SU 2
BP 52
EP 58
DI 10.1159/000323513
PG 7
WC Gastroenterology & Hepatology
WE Science Citation Index Expanded (SCI-EXPANDED)
SC Gastroenterology & Hepatology
GA 872YH
UT WOS:000298846900010
PM 21471704
DA 2026-05-09
ER

PT J
AU Bhutani, MS
Arora, A
AF Bhutani, Manoop S.
Arora, Ayush

TI New developments in endoscopic ultrasound-guided therapies
SO ENDOSCOPIC ULTRASOUND
LA English
DT Review
DE Ablation; biliary drainage; chemoablation; cystic pancreatic tumors;
Digestive Disease Week (DDW); endoscopic ultrasound (EUS); EUS-guided
biliary drainage; fiducials; gastric varices (GVs); pancreatitis;
pancreatic adenocarcinoma; pancreatic cysts; pancreatic neuroendocrine
tumor (PNET); pancreatic pseudocyst; pancreatic tumors; pseudocyst;
radiofrequency; walled-off necrosis
ID MANAGEMENT
AB Endoscopic ultrasound (EUS) has opened new horizons in minimally invasive therapies for diverse gastrointestinal pathologies. Digestive Disease Weak 2015 held in Washington, DC., USA featured exciting research articles on EUS-guided therapeutic procedures. EUS-guided biliary drainage has been attempted and described for many years. There seems to be a lot of interest among various international groups to compare this technique with other alternatives in terms of efficacy and safety. Similarly, EUS-guided pancreatic drainage of cysts and fluid collections continues to evolve with new stents and devices being developed specifically for deployment under endosonographic guidance. EUS-guided ablation of cystic pancreatic tumors is innovative but not always effective. Combining alcohol ablation with injecting chemotherapeutic agents may improve long-term results regarding efficacy. Similarly, for solid pancreatic tumors there appears to be ongoing interest and continuing efforts in injecting different chemotherapeutic or ablative agents, delivering fiducials for radiation guidance and even attempting ablation with radiofrequency. Gastric variceal treatment and EUS-guided anastomoses also continue to be investigated. This review article is focused on the recent developments in EUS-guided therapies presented at Digestive Disease Week (DDW) 2015.
C1 [Bhutani, Manoop S.; Arora, Ayush] Univ Texas MD Anderson Canc Ctr, Dept Gastroenterol Hepatol & Nutr, Houston, TX 77030 USA.
C3 University of Texas System; UTMD Anderson Cancer Center
RP Bhutani, MS (corresponding author), Univ Texas MD Anderson Canc Ctr, 1515 Holcombe Blvd,Unit 1466, Houston, TX 77030 USA.
EM Manoop.Bhutani@mdanderson.org
CR Ardengh JC, 2015, GASTROINTEST ENDOSC, V81, pAB200, DOI 10.1016/j.gie.2015.03.174
Atar M, 2015, GASTROINTEST ENDOSC, V81, pAB555, DOI 10.1016/j.gie.2015.03.1816
Banerjee N, 2015, GASTROENTEROLOGY, V148, pS, DOI 10.1016/s0016-5085(15)32627-5
Bang JY, 2015, GASTROINTEST ENDOSC, V81, pAB437, DOI 10.1016/j.gie.2015.03.815
Bhat YM, 2015, GASTROINTEST ENDOSC, V81, pAB124, DOI 10.1016/j.gie.2015.03.028
Bill J, 2015, GASTROINTEST ENDOSC, V81, pAB531, DOI 10.1016/j.gie.2015.03.1806
Cho MK, 2015, GASTROINTEST ENDOSC, V81, pAB184, DOI 10.1016/j.gie.2015.03.1274
Dhir V, 2015, GASTROINTEST ENDOSC, V82, P650, DOI 10.1016/j.gie.2015.01.061
Dhir VK, 2015, GASTROINTEST ENDOSC, V81, pAB424, DOI 10.1016/j.gie.2015.03.1615
Dollhopf M, 2015, GASTROINTEST ENDOSC, V81, pAB450, DOI 10.1016/j.gie.2015.03.848
El Zein MH, 2015, GASTROINTEST ENDOSC, V81, pAB535, DOI 10.1016/j.gie.2015.03.1077
Ge N, 2014, J TRANSL INTERN MED, V2, P104, DOI 10.4103/2224-4018.141829
Giovannini M, 2015, GASTROINTEST ENDOSC, V81, pAB174, DOI 10.1016/j.gie.2015.03.1926

Holt BA, 2015, GASTROINTEST ENDOSC, V81, pAB131, DOI 10.1016/j.gie.2015.03.045
Iwashita T, 2015, GASTROINTEST ENDOSC, V81, pAB422, DOI 10.1016/j.gie.2015.03.1610
Kandula M, 2015, GASTROENTEROLOGY, V148, pS520
Khan MA, 2015, GASTROINTEST ENDOSC, V81, pAB421, DOI 10.1016/j.gie.2015.03.1606
Khashab MA, 2015, GASTROINTEST ENDOSC, V82, P745, DOI 10.1016/j.gie.2015.05.017
Khashab MA, 2015, GASTROINTEST ENDOSC, V81, P81, DOI 10.1016/j.gie.2014.08.008
Larghi A, 2015, GASTROINTEST ENDOSC, V81, pAB427, DOI 10.1016/j.gie.2015.03.1623
Luna OB, 2015, GASTROINTEST ENDOSC, V81, pAB424, DOI 10.1016/j.gie.2015.03.1614
Luz LP, 2014, ENDOSC ULTRASOUND, V3, P213, DOI 10.4103/2303-9027.144515
Mohamadnejad M, 2015, GASTROINTEST ENDOSC, V81, pAB440, DOI 10.1016/j.gie.2015.03.825
Moyer MT, 2015, GASTROINTEST ENDOSC, V81, pAB114, DOI 10.1016/j.gie.2015.03.010
Nakai Y, 2015, GASTROINTEST ENDOS S, V81, pAB423
Nakai Y, 2015, GASTROINTEST ENDO S1, V81, pAB423
Nieto J, 2015, GASTROINTEST ENDOSC, V81, pAB543, DOI 10.1016/j.gie.2015.03.1097
Nishimura M, 2015, GASTROINTEST ENDOSC, V81, pAB440, DOI 10.1016/j.gie.2015.03.823
Park DH, 2015, GASTROINTEST ENDOSC, V81, pAB130, DOI 10.1016/j.gie.2015.03.041
Park DH, 2015, GASTROINTEST ENDOSC, V81, pAB174, DOI 10.1016/j.gie.2015.03.1927
Senturk H, 2015, GASTROINTEST ENDOSC, V81, pAB423, DOI 10.1016/j.gie.2015.03.1612
Siddiqui A, 2015, GASTROINTEST ENDOSC, V81, pAB131, DOI 10.1016/j.gie.2015.03.044
Song TJ, 2015, GASTROINTEST ENDOSC, V81, pAB536, DOI 10.1016/j.gie.2015.03.1080
Teoh AY, 2015, GASTROINTEST ENDOSC, V81, pAB533, DOI 10.1016/j.gie.2015.03.1810
Vilmann AS, 2015, WORLD J GASTROENTERO, V21, P11842, DOI 10.3748/wjg.v21.i41.11842
Weilert F, 2015, GASTROINTEST ENDOSC, V81, pAB534, DOI 10.1016/j.gie.2015.03.1074
NR 36
TC 10
Z9 10
U1 0
U2 2
PU MEDKNOW PUBLICATIONS & MEDIA PVT LTD
PI MUMBAI
PA B-9, KANARA BUSINESS CENTRE, OFF LINK RD, GHAKTOPAR-E, MUMBAI, 400075, INDIA
SN 2303-9027
EI 2226-7190
J9 ENDOSC ULTRASOUND
JI Endosc. Ultrasound
PD OCT-DEC
PY 2015
VL 4
IS 4
BP 304
EP 311

DI 10.4103/2303-9027.170419
PG 8
WC Gastroenterology & Hepatology
WE Science Citation Index Expanded (SCI-EXPANDED)
SC Gastroenterology & Hepatology
GA CX5WB
UT WOS:000365772100007
PM 26643698
DA 2026-05-09
ER

PT J
AU Jain, D
Bhandari, BS
Agrawal, N
Singhal, S
AF Jain, Deepanshu
Bhandari, Bharat Singh
Agrawal, Nikhil
Singhal, Shashideep
TI Endoscopic Ultrasound-Guided Gallbladder Drainage Using a Lumen-Apposing
Metal Stent for Acute Cholecystitis: A Systematic Review
SO CLINICAL ENDOSCOPY
LA English
DT Review
DE Acute cholecystitis; Endoscopic ultrasound; Lumen-apposing metal stent
ID VIDEO
AB Surgery remains the standard treatment for acute cholecystitis except in high-risk candidates where percutaneous transhepatic gallbladder drainage (PT-GBD), endoscopic transpapillary cystic duct stenting (ET-CDS), and endoscopic ultrasound-guided gallbladder drainage (EUS-GBD) are potential choices. PT-GBD is contraindicated in patients with coagulopathy or ascites and is not preferred by patients owing to aesthetic reasons. ET-CDS is successful only if the cystic duct can be visualized and cannulated. For 189 patients who underwent EUS-GBD via insertion of a lumen-apposing metal stent (LAMS), the composite technical success rate was 95.2%, which increased to 96.8% when LAMS was combined with co-axial self-expandable metal stent (SEMS). The composite clinical success rate was 96.7%. We observed a small risk of recurrent cholecystitis (5.1%), gastrointestinal bleeding (2.6%) and stent migration (1.1%). Cautery enhanced LAMS significantly decreases the stent deployment time compared to non-cautery enhanced LAMS. Prophylactic placement of a pigtail stent or SEMS through the LAMS avoids re-interventions, particularly in patients, where it is intended to remain in situ indefinitely. Limited evidence suggests that the efficacy of EUS-GBD via LAMS is comparable to that of PT-GBD with the former showing better results in postoperative pain, length of hospitalization, and need for antibiotics. EUS-GBD via LAMS is a safe and efficacious option when performed by experts.
C1 [Jain, Deepanshu] Einstein Healthcare Network, Div Gastroenterol & Hepatol, Dept Digest Dis & Transplantat, Internal Med, Philadelphia, PA USA.

[Bhandari, Bharat Singh] St Vincent Hosp, Dept Internal Med, Worcester, MA 01604 USA.
[Agrawal, Nikhil] SUNY Buffalo, Dept Internal Med, Buffalo, NY USA.
[Singhal, Shashideep] Univ Texas Hlth Sci Ctr Houston, Div Gastroenterol Hepatol & Nutr, 6431 Fannin,MSB 4-234, Houston, TX 77030 USA.
C3 State University of New York (SUNY) System; University at Buffalo, SUNY;
University of Texas System; University of Texas Health Science Center
Houston
RP Singhal, S (corresponding author), Univ Texas Hlth Sci Ctr Houston, Div Gastroenterol Hepatol & Nutr, 6431 Fannin,MSB 4-234, Houston, TX 77030 USA.
EM sdsinghal@gmail.com
RI Bhandari, Bharat/JDC-2002-2023; Agrawal, Nikhil/IAR-6131-2023
CR Binmoeller KF, 2011, ENDOSCOPY, V43, P337, DOI 10.1055/s-0030-1256127
de la Serna-Higuera C, 2013, GASTROINTEST ENDOSC, V77, P303, DOI 10.1016/j.gie.2012.09.021
Dollhopf M, 2017, GASTROINTEST ENDOSC, V86, P636, DOI 10.1016/j.gie.2017.02.027
FRIEDMAN GD, 1989, J CLIN EPIDEMIOL, V42, P127, DOI 10.1016/0895-4356(89)90086-3
Garden OJ, 2002, PRINCIPLES PRACTICE
Hassler KR, 2017, GALLBLADDER CHOLECYS
Irani S, 2017, CLIN GASTROENTEROL H, V15, P738, DOI 10.1016/j.cgh.2016.12.021
Irani S, 2015, GASTROINTEST ENDOSC, V82, P1110, DOI 10.1016/j.gie.2015.05.045
Itoi T, 2008, GASTROINTEST ENDOSC, V68, P455, DOI 10.1016/j.gie.2008.02.052
Itoi T, 2014, GASTROINTEST ENDOSC, V79, P547, DOI 10.1016/j.gie.2013.12.010
Itoi T, 2013, DIGEST ENDOSC, V25, P137, DOI 10.1111/den.12084
Kumta NA, 2016, ENDOSCOPY, V48, pE327, DOI 10.1055/s-0042-117634
Law R, 2016, CLIN GASTROENTEROL H, V14, P476, DOI 10.1016/j.cgh.2015.10.026
Tharian B, 2016, GASTROINTEST ENDOSC, V83, P460, DOI 10.1016/j.gie.2015.08.016
Walter D, 2016, GUT, V65, P6, DOI 10.1136/gutjnl-2015-309925
Widmer J, 2015, CLIN ENDOSC, V48, P411, DOI 10.5946/ce.2015.48.5.411
Zacks SL, 2002, AM J GASTROENTEROL, V97, P334
NR 17
TC 49
Z9 59
U1 0
U2 2
PU KOREAN SOC GASTROINTESTINAL ENDOSCOPY
PI SEOUL
PA 2003 LG PALACE, 165-8 DONGGYO-DONG, MAPO-GU, SEOUL, 121-754, SOUTH KOREA
SN 2234-2400
EI 2234-2443
J9 CLIN ENDOSC
JI Clin. Endosc.
PD SEP
PY 2018

VL 51
IS 5
BP 450
EP 462
DI 10.5946/ce.2018.024
PG 13
WC Gastroenterology & Hepatology
WE Emerging Sources Citation Index (ESCI)
SC Gastroenterology & Hepatology
GA GV3AT
UT WOS:000445965100011
PM 29852730
OA Green Submitted, gold
DA 2026-05-09
ER

PT J
AU Jain, D
Shah, M
Patel, U
Sharma, A
Singhal, S
AF Jain, Deepanshu
Shah, Manan
Patel, Upen
Sharma, Abhinav
Singhal, Shashideep
TI Endoscopic Ultrasound Guided Choledocho-Enterostomy by Using Lumen
Apposing Metal Stent in Patients with Failed Endoscopic Retrograde
Cholangiopancreatography: A Literature Review
SO DIGESTION
LA English
DT Review
DE Endoscopic ultrasound; Bile drainage; Obstructive jaundice; Lumen
apposing metal stent
ID MALIGNANT BILIARY OBSTRUCTION; DUODENAL OBSTRUCTION;
CHOLEDOCHODUODENOSTOMY; DRAINAGE; ERCP; PLACEMENT;
CHOLEDOCHOJEJUNOSTOMY; ANASTOMOSIS; STATISTICS
AB Background: Endoscopic retrograde cholangiopancreatography (ERCP) has been the cornerstone in the management of malignant jaundice to provide symptomatic relief and/or to allow chemotherapy. Difficult biliary cannulation or the presence of concomitant duodenal obstruction may lead to its failure. Method: An extensive English literature search was done via PubMed and Google Scholar to identify 13 peer-reviewed original articles. Results: Of 92 patients, 98.9% (91/92)

successfully underwent EUS-guided biliary drainage by placement of a lumen-apposing metal stent (LAMS) across extrahepatic biliary tree and small bowel. Although technically challenging, a similar technical success was noted for patients with limited dilation of extrahepatic biliary tree (less than 1 cm) and altered gastrointestinal anatomy (Roux-en-Y, Whipple, or Billroth II). Of 91 patients, 98.9% (90/91) had clinical success. Procedure complications included perforation (2/92; 2.2%) and bleeding (1/92; 1.1%). Stent complications included obstruction (7/91; 7.7%), and migration (1/91; 1.1%) with all responding to endoscopic debridement of food or tumor, placement of plastic pigtail stent through the LAMS, or replacement with a new stent. No procedure-related mortality was noted. Conclusion: In the hands of an expert, EUS-guided biliary drainage with the use of LAMS is an efficacious and safe option for patients with distal malignant biliary stricture, not amenable to ERCP. (c) 2018 S. Karger AG, Basel
C1 [Jain, Deepanshu] Einstein Healthcare Network, Dept Digest Dis & Transplantat, Div Gastroenterol & Hepatol, Philadelphia, PA 19141 USA.

[Shah, Manan; Patel, Upen; Sharma, Abhinav; Singhal, Shashideep] Univ Texas Hlth Sci Ctr Houston, Div Gastroenterol Hepatol & Nutr, 6431 Fannin,MSB 4-234, Houston, TX 77030 USA.

C3 University of Texas System; University of Texas Health Science Center Houston

RP Singhal, S (corresponding author), Univ Texas Hlth Sci Ctr Houston, Div Gastroenterol Hepatol & Nutr, 6431 Fannin,MSB 4-234, Houston, TX 77030 USA.

EM sdsinghal@gmail.com
CR Abraham NS, 2002, GASTROINTEST ENDOSC, V56, P835, DOI 10.1067/mge.2002.129868
Akhan O, 2002, EUR J RADIOL, V43, P229, DOI 10.1016/S0720-048X(02)00158-4
Anderloni A, 2016, ENDOSCOPY, V48, pE321, DOI 10.1055/s-0042-117425
Artifon ELA, 2015, ENDOSC ULTRASOUND, V4, P235, DOI 10.4103/2303-9027.163010
Attili F, 2015, ENDOSCOPY, V47, pE619, DOI 10.1055/s-0034-1393669
Binmoeller KF, 2011, ENDOSCOPY, V43, P337, DOI 10.1055/s-0030-1256127
French JB, 2016, CLIN J GASTROENTEROL, V9, P79, DOI 10.1007/s12328-016-0634-y
Giovannini M, 2001, ENDOSCOPY, V33, P898, DOI 10.1055/s-2001-17324
Glessing BR, 2015, GASTROINTEST ENDOSC, V81, P1019, DOI 10.1016/j.gie.2014.09.061
Gornals JB, 2016, DIGEST ENDOSC, V28, P100, DOI 10.1111/den.12548
Itoi T, 2015, GASTROINTEST ENDOSC, V81, P1259, DOI 10.1016/j.gie.2014.08.033
Itoi T, 2014, GASTROINTEST ENDOSC, V79, P715, DOI 10.1016/j.gie.2013.11.021
Itoi T, 2012, GASTROINTEST ENDOSC, V75, P870, DOI 10.1016/j.gie.2011.10.020
Kedia P, 2015, GASTROINTEST ENDOSC, V82, P560, DOI 10.1016/j.gie.2015.03.1913
Kunda R, 2016, SURG ENDOSC, V30, P5002, DOI 10.1007/s00464-016-4845-6
Miller KD, 2016, CA-CANCER J CLIN, V66, P271, DOI 10.3322/caac.21349
Mukai S, 2016, GASTROINTEST ENDOSC, V83, P832, DOI 10.1016/j.gie.2015.09.047
Park DH, 2011, GASTROINTEST ENDOSC, V74, P1276, DOI 10.1016/j.gie.2011.07.054
Perez-Miranda M, 2014, GASTROINTEST ENDOSC, V80, P339, DOI 10.1016/j.gie.2014.04.030
Prachayakul V, 2015, WORLD J GASTRO ENDOS, V7, P37, DOI 10.4253/wjge.v7.i1.37
RING EJ, 1984, AM J ROENTGENOL, V142, P31, DOI 10.2214/ajr.142.1.31
Sejpal DV, 2015, GASTROINTEST ENDOSC, V82, P170, DOI 10.1016/j.gie.2015.02.002
Siegel RL, 2020, CA-CANCER J CLIN, V70, P7, DOI 10.3322/caac.21590
Tsuchiya T, 2017, GASTROINTEST ENDOSC

Tyberg A, 2015, ENDOSCOPY, V47, pE342, DOI 10.1055/s-0034-1392318
NR 25
TC 23
Z9 23
U1 0
U2 3
PU KARGER
PI BASEL
PA ALLSCHWILERSTRASSE 10, CH-4009 BASEL, SWITZERLAND
SN 0012-2823
EI 1421-9867
J9 DIGESTION
JI Digestion
PY 2018
VL 98
IS 1
BP 1
EP 10
DI 10.1159/000487185
PG 10
WC Gastroenterology & Hepatology
WE Science Citation Index Expanded (SCI-EXPANDED)
SC Gastroenterology & Hepatology
GA GK4LA
UT WOS:000436129800001
PM 29672294
OA Bronze
DA 2026-05-09
ER

PT J
AU Artifon, ELA
Ferreira, FC
Otoch, JP
Rasslan, S
Itoi, T
Perez-Miranda, M
AF Artifon, Everson L. A.
Ferreira, Flavio Coelho
Otoch, Jose Pinhata
Rasslan, Samir
Itoi, Takao

Perez-Miranda, Manuel
TI EUS-Guided Biliary Drainage: A Review Article
SO JOURNAL OF THE PANCREAS
LA English
DT Review
DE Biliary Tract; Drainage; Endoscopy; Gastrointestinal; Endosonography;
Jaundice; Obstructive
ID UNRESECTABLE PANCREATIC-CANCER; COVERED METAL STENT; ENDOSCOPIC
ULTRASOUND; INTERVENTIONAL EUS; CHOLEDOCHODUODENOSTOMY; OBSTRUCTION;
HEPATICOGASTROSTOMY; PLACEMENT; CHOLANGIOPANCREATOGRAPHY;
CHOLANGIOGRAPHY
AB To demonstrate a comprehensive review of published articles regarding EUS-guided biliary drainage. Methods Review of studies regarding EUS-guided biliary drainage including case reports, case series and previous reviews. Results EUS-guided hepaticogastrostomy, choledochoduodenostomy and choledochoantrostomy are advanced procedures on biliary and pancreatic endoscopy and together make up the echo-guided biliary drainage. Hepaticogastrostomy is indicated in cases of hilar obstruction, while the procedure of choice is choledochoduodenostomy in distal lesions. Both procedures must be done only after unsuccessful ERCP. The indication of these procedures must be made under a multidisciplinary view while sharing information with the patient or legal guardian. Conclusion Hepaticogastrostomy and choledochoduodenostomy are feasible when performed by endoscopists with expertise in biliopancreatic endoscopy and advanced echo-endoscopy and should be performed currently under rigorous protocol in educational institutions.
C1 [Artifon, Everson L. A.; Ferreira, Flavio Coelho; Otoch, Jose Pinhata; Rasslan, Samir] Univ Sao Paulo, Sao Paulo, Brazil.
[Itoi, Takao] Tokyo Med Univ Hosp, Tokyo, Japan.
[Perez-Miranda, Manuel] Hosp Univ Rio Hortega, Valladolid, Spain.
C3 Universidade de Sao Paulo; Tokyo Medical University; Hospital del Rio
Hortega
RP Artifon, ELA (corresponding author), Univ Sao Paulo, Med Sch, Av Dr Eneas Carvalho Aguiar,255 Cerqueira Cesar, BR-0540300 Sao Paulo, SP, Brazil.
EM eartifon@hotmail.com
RI Ferreira, Flavio Coelho/K-7958-2017
OI Ferreira, Flavio Coelho/0000-0002-8857-2438
CR Ang TL, 2007, J PANCREAS, V8, P438
Artifon ELA, 2007, CLINICS, V62, P799, DOI 10.1590/S1807-59322007000600023
Artifon ELA, 2011, GASTROINTEST ENDOSC, V73, P1317, DOI 10.1016/j.gie.2010.10.041
Bories E, 2007, ENDOSCOPY, V39, P287, DOI 10.1055/s-2007-966212
Bories E, 2008, ENDOSCOPY S1, V40, pA55
Brauer BC, 2009, GASTROINTEST ENDOSC, V70, P471, DOI 10.1016/j.gie.2008.12.233
Burmester E, 2003, GASTROINTEST ENDOSC, V57, P246, DOI 10.1067/mge.2003.85
Chopin-Laly X, 2009, SURGERY, V145, P123, DOI 10.1016/j.surg.2008.07.019
Eum J, 2010, GASTROINTEST ENDOSC, V72, P1279, DOI 10.1016/j.gie.2010.07.026
Fogel EL, 2001, ENDOSCOPY, V33, P31, DOI 10.1055/s-2001-11186
Fujita N, 2008, J GASTROENTEROL, V43, P637, DOI 10.1007/s00535-008-2204-7

Fujita N, 2007, WORLD J GASTROENTERO, V13, P5512, DOI 10.3748/wjg.v13.i41.5512
Fujita N, 2009, DIGEST ENDOSC, V21, P48, DOI 10.1111/j.1443-1661.2008.00821.x
Giovannini M, 2006, ENDOSCOPY, V38, pS93, DOI 10.1055/s-2006-946665
Giovannini M, 2004, BEST PRACT RES CL GA, V18, P183, DOI 10.1016/S1521-6918(03)00103-3
Giovannini M, 2003, ENDOSCOPY, V35, P1076, DOI 10.1055/s-2003-44596
Giovannini M, 2001, ENDOSCOPY, V33, P898, DOI 10.1055/s-2001-17324
Giovannini M, 2007, TECH GASTROINTEST EN, V9, P32, DOI 10.1016/j.tgie.2006.11.013
GRIMM H, 1992, GASTROINTEST ENDOSC, V38, P170, DOI 10.1016/S0016-5107(92)70384-8
Gupta K, 2007, REV GASTROENTEROL DI, V7, P22
Hanada K, 2009, DIGEST ENDOSC, V21, pS75, DOI 10.1111/j.1443-1661.2009.00855.x
Hara K, 2011, AM J GASTROENTEROL, V106, P1239, DOI 10.1038/ajg.2011.84
Horaguchi J, 2009, DIGEST ENDOSC, V21, P239, DOI 10.1111/j.1443-1661.2009.00899.x
Iglesias-García J, 2008, REV ESP ENFERM DIG, V100, P236, DOI 10.4321/s1130-01082008000400008
Itoi T, 2008, WORLD J GASTROENTERO, V14, P6078, DOI 10.3748/wjg.14.6078
Iwamuro M, 2010, DIGEST ENDOSC, V22, P236, DOI 10.1111/j.1443-1661.2010.00997.x
Kahaleh M, 2006, GASTROINTEST ENDOSC, V64, P52, DOI 10.1016/j.gie.2006.01.063
Kim YS, 2010, ENDOSCOPY, V42, P496, DOI 10.1055/s-0029-1244082
Komaki T, 2011, PANCREATOLOGY, V11, P47, DOI 10.1159/000323508
LAI ECS, 1992, NEW ENGL J MED, V326, P1582, DOI 10.1056/NEJM199206113262401
Maranki J, 2009, ENDOSCOPY, V41, P532, DOI 10.1055/s-0029-1214712
Martins FP, 2010, ENDOSCOPY, V42, pE126, DOI 10.1055/s-0029-1243911
Nguyen-Tang T, 2010, ENDOSCOPY, V42, P232, DOI 10.1055/s-0029-1243858
Park DH, 2010, GASTROINTEST ENDOSC, V71, P413, DOI 10.1016/j.gie.2009.10.015
Park DH, 2009, AM J GASTROENTEROL, V104, P2168, DOI 10.1038/ajg.2009.254
Perez-Miranda M, 2010, WORLD J GASTRO ENDOS, V2, P212, DOI 10.4253/wjge.v2.i6.212
Perez-Miranda M, 2010, GASTROINTEST ENDOSC, V71, pAB136, DOI 10.1016/j.gie.2010.03.119
Püspök A, 2005, AM J GASTROENTEROL, V100, P1743, DOI 10.1111/j.1572-0241.2005.41806.x
Siddiqui AA, 2011, SURG ENDOSC, V25, P549, DOI 10.1007/s00464-010-1216-6
SMITH AC, 1994, LANCET, V344, P1655, DOI 10.1016/S0140-6736(94)90455-3
Talreja JP, 2008, GASTROINTEST ENDOSC, V68, P1199, DOI 10.1016/j.gie.2008.06.015
Tarantino I, 2008, ENDOSCOPY, V40, P336, DOI 10.1055/s-2007-995455
Wiersema MJ, 1996, GASTROINTEST ENDOSC, V43, P102, DOI 10.1016/S0016-5107(06)80108-2
Will U, 2007, ENDOSCOPY, V39, P292, DOI 10.1055/s-2007-966215
Wilson JA, 2010, GASTROINTEST ENDOSC, V72, P947, DOI 10.1016/j.gie.2010.07.016
Yamao K, 2008, ENDOSCOPY, V40, P340, DOI 10.1055/s-2007-995485
Yamao K, 2006, GASTROINTEST ENDOSC, V64, P663, DOI 10.1016/j.gie.2006.07.003
NR 47
TC 23
Z9 28
U1 0
U2 0
PU E S BURIONI RICERCHE BIBLIOGRAFICHE

PI GENOVA
PA CORSO FIRENZE 41-2, GENOVA, 16136, ITALY
SN 1590-8577
J9 J PANCREAS
JI J. Pancreas
PD JAN
PY 2012
VL 13
IS 1
BP 7
EP 17
PG 11
WC Gastroenterology & Hepatology
WE Emerging Sources Citation Index (ESCI)
SC Gastroenterology & Hepatology
GA V05AG
UT WOS:000213844900002
PM 22233941
DA 2026-05-09
ER

PT J
AU Okuno, N
Hara, K
Mizuno, N
Kuwahara, T
Iwaya, H
Tajika, M
Tanaka, T
Ishihara, M
Hirayama, Y
Onishi, S
Toriyama, K
Ito, A
Kuraoka, N
Matsumoto, S
Obata, M
Yasuda, M
Kurita, Y
Tanaka, H
Niwa, Y
AF Okuno, Nozomi

Hara, Kazuo
Mizuno, Nobumasa
Kuwahara, Takamichi
Iwaya, Hiromichi
Tajika, Masahiro
Tanaka, Tsutomu
Ishihara, Makoto
Hirayama, Yutaka
Onishi, Sachiyo
Toriyama, Kazuhiro
Ito, Ayako
Kuraoka, Naosuke
Matsumoto, Shimpei
Obata, Masahiro
Yasuda, Muneji
Kurita, Yusuke
Tanaka, Hiroki
Niwa, Yasumasa
TI Infectious peritonitis after endoscopic ultrasound-guided biliary
drainage in a patient with ascites
SO INTERNATIONAL JOURNAL OF GASTROINTESTINAL INTERVENTION
LA English
DT Article
DE Endoscopic ultrasonography; Endoscopic ultrasound-guided biliary
drainage; Endoscopic ultrasound-guided choledochoduodenostomy;
Endoscopic ultrasound-guided hepaticogastrostomy; Interventional
endoscopic ultrasound
AB A Summary of Event: Bacterial, mycotic peritonitis and Candida fungemia developed in a patient with moderate ascites who had undergone endoscopic ultrasound-guided biliary drainage (EUS-BD). Antibiotics and antifungal agent were administered and ascites drainage was performed. Although the infection improved, the patient's general condition gradually deteriorated due to aggravation of the primary cancer and he died.

Teaching Point: This is the first report to describe infectious peritonitis after EUS-BD. Ascites carries the potential risk of severe complications. As such, in patients with ascites, endoscopic retrograde cholangiopancreatography (ERCP) is typically preferred over EUS-BD or percutaneous drainage to prevent bile leakage. However, ERCP may not be possible in some patients with tumor invasion of the duodenum or with surgically altered anatomy. Thus, in patients with ascites who require EUS-BD, we recommend inserting the drainage tube percutaneously and draining the ascites before and after the intervention in order to prevent severe infection. Copyright (C) 2018, Society of Gastrointestinal Intervention. All rights reserved.
C1 [Okuno, Nozomi; Hara, Kazuo; Mizuno, Nobumasa; Kuwahara, Takamichi; Iwaya, Hiromichi; Toriyama, Kazuhiro; Ito, Ayako; Kuraoka, Naosuke; Matsumoto, Shimpei; Obata, Masahiro; Yasuda, Muneji; Kurita, Yusuke; Tanaka, Hiroki] Aichi Canc Ctr Hosp, Dept Gastroenterol, Nagoya, Aichi, Japan.

[Tajika, Masahiro; Tanaka, Tsutomu; Ishihara, Makoto; Hirayama, Yutaka; Onishi, Sachiyo] Aichi Canc Ctr Hosp, Dept

Endoscopy, Nagoya, Aichi, Japan.
[Niwa, Yasumasa] Aichi Canc Ctr Hosp, Nagoya, Aichi, Japan.
C3 Aichi Cancer Center; Aichi Cancer Center; Aichi Cancer Center
RP Hara, K (corresponding author), Aichi Canc Ctr Hosp, Dept Gastroenterol, Chikusa Ku, 1-1 Kanokoden, Nagoya, Aichi 4648681, Japan.
EM khara@aichi-cc.jp
RI Kurita, Yusuke/GPK-7068-2022; Okuno, Nozomi/AHE-2009-2022
OI Okuno, Nozomi/0000-0001-6376-687X
CR Anderloni A, 2017, ENDOSC INT OPEN, V5, pE1100, DOI 10.1055/s-0043-118828
Giovannini M, 2001, ENDOSCOPY, V33, P898, DOI 10.1055/s-2001-17324
Hara K, 2013, ENDOSCOPY, V45, P392, DOI 10.1055/s-0032-1326076
Hara K, 2016, WORLD J GASTROENTERO, V22, P1297, DOI 10.3748/wjg.v22.i3.1297
Kawakubo K, 2014, J HEPATO-BIL-PAN SCI, V21, P328, DOI 10.1002/jhbp.27
Martins FP, 2010, ENDOSCOPY, V42, pE126, DOI 10.1055/s-0029-1243911
Okuno N, 2015, ENDOSCOPY, V47, pE311, DOI 10.1055/s-0034-1392314
Park DH, 2009, AM J GASTROENTEROL, V104, P2168, DOI 10.1038/ajg.2009.254
NR 8
TC 7
Z9 7
U1 0
U2 1
PU SOC GASTROINTESTINAL INTERVENTION
PI SEOUL
PA 12, HAKDONG-RO 31, GANGNAM-GU, SEOUL, 00000, SOUTH KOREA
SN 2636-0004
EI 2636-0012
J9 INT J GASTROINT INT
JI Int. J. Gastrointest. Interv.
PD APR
PY 2018
VL 7
IS 1
BP 40
EP 43
DI 10.18528/gii180006
PG 4
WC Gastroenterology & Hepatology
WE Emerging Sources Citation Index (ESCI)
SC Gastroenterology & Hepatology
GA VJ0HG
UT WOS:000521643300010
OA Green Submitted, gold

DA 2026-05-09
ER

PT J
AU Duarte-Chavez, R
Kahaleh, M
AF Duarte-Chavez, Rodrigo
Kahaleh, Michel
TI Therapeutic endoscopic ultrasound
SO TRANSLATIONAL GASTROENTEROLOGY AND HEPATOLOGY
LA English
DT Review
DE Gallbladder drainage; biliary drainage; pancreatic duct drainage; gastroenterostomy (GE); endoscopic ultrasound-directed transgastric endoscopic retrograde cholangiopancreatography procedure (EDGE procedure)
ID GUIDED BILIARY DRAINAGE; APPOSING METAL STENT; PANCREATIC-DUCT DRAINAGE; LONG-TERM OUTCOMES; TRANSHEPATIC GALLBLADDER DRAINAGE; MULTICENTER COMPARATIVE TRIAL; FAILED ERCP; RETROGRADE CHOLANGIOPANCREATOGRAPHY; SURGICAL GASTROJEJUNOSTOMY; INTERNATIONAL MULTICENTER
AB Endoscopic ultrasound (EUS) has been continuously evolving for the past three decades and has become widely used for both diagnostic and therapeutic purposes. The efficacy of therapeutic EUS (TEUS) has proven to be superior and better tolerated than conventional percutaneous or surgical techniques. TEUS has allowed the performance of multiple procedures including gallbladder, pancreatic duct and biliary drainage as well as gastrointestinal anastomoses. TEUS procedures generally require the following critical steps: needle access, guidewire placement, fistula creation and stent deployment. The indications and contraindication for TEUS procedures vary with different procedures but common contraindications include hemodynamic instability, severe coagulopathy unable to be reversed, large volume ascites or the inability to obtain access to the target site. Proficiency and high volume in endoscopic retrograde cholangiopancreatography (ERCP) and diagnostic EUS procedures are required for training in TEUS. The complexity of the cases performed can be seen as a pyramid with drainage of pancreatic fluid collections at the base, pancreaticobiliary decompression in the middle, and creation of digestive anastomosis at the top. The mastery of each level is crucial prior to reaching the next level of complexity. TEUS has been incorporated in our arsenal and is impacting on a daily basis the way we offer minimally invasive therapy.
C1 [Duarte-Chavez, Rodrigo] St Lukes Univ Hlth Network, Dept Internal Med, Bethlehem, PA USA.
[Kahaleh, Michel] Rutgers Robert Wood Johnson Med Sch, Dept Internal Med, New Brunswick, NJ 08901 USA.
C3 Rutgers University System; Rutgers University New Brunswick; Rutgers University Biomedical & Health Sciences
RP Kahaleh, M (corresponding author), Rutgers Robert Wood Johnson Med Sch, Dept Internal Med, Med, New Brunswick, NJ 08901 USA.; Kahaleh, M (corresponding author), Rutgers Robert Wood Johnson Med Sch, Dept Internal Med, Gastroenterol, New Brunswick, NJ 08901 USA.; Kahaleh, M (corresponding author), Rutgers Robert Wood Johnson Med Sch, Dept Internal Med, Endoscopy, New Brunswick, NJ 08901 USA.
EM mkahaleh@gmail.com
RI Kahaleh, Michel/A-9408-2011

CR Amateau SK, 2018, OBES SURG, V28, P1445, DOI 10.1007/s11695-018-3171-6
Bang JY, 2018, GASTROINTEST ENDOSC, V88, P9, DOI 10.1016/j.gie.2018.03.012
Baron TH, 2007, GASTROINTEST ENDOSC, V65, P735, DOI 10.1016/j.gie.2006.07.041
Baron TH, 2015, NEW ENGL J MED, V373, P357, DOI 10.1056/NEJMra1411372
Bataille L, 2002, GASTROINTEST ENDOSC, V55, P740, DOI 10.1067/mge.2002.123621
Binmoeller KF, 2012, ENDOSCOPY, V44, P499, DOI 10.1055/s-0032-1309382
Binmoeller KF, 2011, ENDOSCOPY, V43, P337, DOI 10.1055/s-0030-1256127
Bukhari M, 2018, GASTROINTEST ENDOSC, V88, P486, DOI 10.1016/j.gie.2018.04.2356
Burmester E, 2003, GASTROINTEST ENDOSC, V57, P246, DOI 10.1067/mge.2003.85
Chen YI, 2018, GASTROINTEST ENDOSC, V87, P1215, DOI 10.1016/j.gie.2017.07.030
Chen YI, 2017, GASTROINTEST ENDOSC, V85, P170, DOI 10.1016/j.gie.2016.07.031
Chiang AL, 2018, GASTROINTEST ENDOSC, V87, pAB70, DOI 10.1016/j.gie.2018.04.058
Cho DH, 2017, GASTROINTEST ENDOSC, V85, P1067, DOI 10.1016/j.gie.2016.09.010
Choi JH, 2014, ENDOSCOPY, V46, P656, DOI 10.1055/s-0034-1365720
Cirocchi R, 2017, SURG-J R COLL SURG E, V15, P379, DOI 10.1016/j.surge.2017.05.004
Dhindsa BS, 2020, ENDOSC INT OPEN, V8, pE163, DOI 10.1055/a-1067-4411
DIMAGNO EP, 1980, LANCET, V1, P629
Dollhopf M, 2017, GASTROINTEST ENDOSC, V86, P636, DOI 10.1016/j.gie.2017.02.027
Duarte-Chavez R, 2020, GASTROINTEST ENDOSC, V91, pAB308, DOI 10.1016/j.gie.2020.03.2126
Dumonceau JM, 1999, GASTROINTEST ENDOSC, V50, P274, DOI 10.1016/S0016-5107(99)70240-3
Ergun M, 2011, ENDOSCOPY, V43, P518, DOI 10.1055/s-0030-1256333
François E, 2002, GASTROINTEST ENDOSC, V56, P128, DOI 10.1067/mge.2002.125547
Fujii LL, 2013, GASTROINTEST ENDOSC, V78, P854, DOI 10.1016/j.gie.2013.05.016
Fujii-Lau LL, 2015, J HEPATO-BIL-PAN SCI, V22, P51, DOI 10.1002/jhbp.187
Ge PS, 2019, SURG ENDOSC, V33, P3404, DOI 10.1007/s00464-018-06636-3
GHATTAS G, 1992, GASTROINTEST ENDOSC, V38, P590, DOI 10.1016/S0016-5107(92)70524-0
Giovannini M, 2003, ENDOSCOPY, V35, P1076, DOI 10.1055/s-2003-44596
Giovannini M, 2001, ENDOSCOPY, V33, P898, DOI 10.1055/s-2001-17324
Giovannini M, 2017, ENDOSC ULTRASOUND, V6, P281, DOI 10.4103/eus.eus_86_17
GLENN F, 1977, ANN SURG, V185, P185, DOI 10.1097/00000658-197702000-00009
HARADA N, 1995, ENDOSCOPY, V27, P612, DOI 10.1055/s-2007-1005769
Higa JT, 2019, GASTROINTEST ENDOSC, V90, P483, DOI 10.1016/j.gie.2019.04.238
Hindryckx P, 2019, WORLD J GASTRO ENDOS, V11, P103, DOI 10.4253/wjge.v11.i2.103
Ichkhanian Y., 2020, Gastrointest Endosc, V91
Irani S, 2017, CLIN GASTROENTEROL H, V15, P738, DOI 10.1016/j.cgh.2016.12.021
Itoi T, 2017, DIGEST ENDOSC, V29, P495, DOI 10.1111/den.12794
Itoi T, 2016, GUT, V65, P193, DOI 10.1136/gutjnl-2015-310348
Itoi T, 2013, GASTROINTEST ENDOSC, V78, P934, DOI 10.1016/j.gie.2013.09.025
James HJ, 2019, ENDOSCOPY, V51, P1051, DOI 10.1055/a-0938-3918
James TW, 2019, OBES SURG, V29, P451, DOI 10.1007/s11695-018-3531-2
Jang JW, 2012, GASTROENTEROLOGY, V142, P805, DOI 10.1053/j.gastro.2011.12.051
Kahaleh M, 2004, GASTROINTEST ENDOSC, V60, P138, DOI 10.1016/S0016-5107(04)01528-7

Kahaleh M, 2005, GASTROINTEST ENDOSC, V61, P307, DOI 10.1016/S0016-5107(04)02585-4
Kahaleh M, 2016, DIGEST LIVER DIS, V48, P1054, DOI 10.1016/j.dld.2016.05.021
Kedia P, 2014, GASTROENTEROLOGY, V147, P566, DOI 10.1053/j.gastro.2014.05.045
Kedia P, 2013, CLIN ENDOSC, V46, P543, DOI 10.5946/ce.2013.46.5.543
Kerdsirichairat T, 2018, GASTROINTEST ENDOSC, V87, pAB320, DOI 10.1016/j.gie.2018.04.1692
Khan MA, 2016, DIGEST DIS SCI, V61, P684, DOI 10.1007/s10620-015-3933-0
Khashab MA, 2017, ENDOSC INT OPEN, V5, pE275, DOI 10.1055/s-0043-101695
Khashab MA, 2016, ENDOSC INT OPEN, V4, pE175, DOI 10.1055/s-0041-109083
KIMMEY MB, 1990, GASTROINTEST ENDOSC, V36, pS40, DOI 10.1016/S0016-5107(90)71014-0
Kunda R, 2016, SURG ENDOSC, V30, P5002, DOI 10.1007/s00464-016-4845-6
Lajin M, 2019, VIDEOGIE, V5, P75
Lee TH, 2016, CLIN GASTROENTEROL H, V14, P1011, DOI 10.1016/j.cgh.2015.12.032
Mai HD, 2018, CLIN J GASTROENTEROL, V11, P282, DOI 10.1007/s12328-018-0837-5
Matsunami Y, 2018, ENDOSC INT OPEN, V6, pE505, DOI 10.1055/s-0044-101753
Mayumi T, 2018, J HEPATO-BIL-PAN SCI, V25, P96, DOI 10.1002/jhbp.519
Minaga K, 2017, SURG ENDOSC, V31, P4764, DOI 10.1007/s00464-017-5553-6
Mohan BP, 2020, ENDOSCOPY, V52, P96, DOI 10.1055/a-1020-3932
Mori Y, 2018, J HEPATO-BIL-PAN SCI, V25, P87, DOI 10.1002/jhbp.504
Nakai Y, 2016, ENDOSCOPY, V48, P1125, DOI 10.1055/s-0042-116595
Oh D, 2019, GASTROINTEST ENDOSC, V89, P289, DOI 10.1016/j.gie.2018.08.052
Oh D, 2016, GASTROINTEST ENDOSC, V83, P366, DOI 10.1016/j.gie.2015.07.015
Park DH, 2011, GASTROINTEST ENDOSC, V74, P1276, DOI 10.1016/j.gie.2011.07.054
Perez-Miranda M, 2018, ENDOSC ULTRASOUND, V7, P79, DOI 10.4103/eus.eus_5_18
Perez-Miranda M, 2017, J CLIN GASTROENTEROL, V51, P896, DOI 10.1097/MCG.0000000000000887
Poincloux L, 2015, ENDOSCOPY, V47, P794, DOI [10.1055/s-0035-1545399, 10.1055/s-0034-1391988]
Saumoy M, 2019, J CLIN GASTROENTEROL, V53, P231, DOI 10.1097/MCG.0000000000001036
Saumoy Monica, 2017, Gastrointest Endosc Clin N Am, V27, P749, DOI [10.1016/j.giec.2017.06.012, 10.1016/j.giec.2017.06.012]
Seamon MJ, 2007, J TRAUMA, V62, P829, DOI 10.1097/TA.0b013e318033a790
Shah JN, 2012, GASTROINTEST ENDOSC, V75, P56, DOI 10.1016/j.gie.2011.08.032
Shah SL, 2018, J CLIN GASTROENTEROL, V52, P765, DOI 10.1097/MCG.0000000000001104
Sharaiha RZ, 2017, GASTROINTEST ENDOSC, V85, P904, DOI 10.1016/j.gie.2016.12.023
Sharaiha RZ, 2016, SURG ENDOSC, V30, P5500, DOI 10.1007/s00464-016-4913-y
SHORVON PJ, 1985, GUT, V26, P1373, DOI 10.1136/gut.26.12.1373
Siddiqui A, 2019, SURG ENDOSC, V33, P1260, DOI 10.1007/s00464-018-6406-7
Sooklal Shelini, 2019, VideoGIE, V4, P530, DOI [10.1016/j.vgie.2019.07.015, 10.1016/j.vgie.2019.07.015]
Sportes A, 2017, THER ADV GASTROENTER, V10, P483, DOI 10.1177/1756283X17702096
Teoh AYB, 2020, GUT, V69, P1085, DOI 10.1136/gutjnl-2019-319996
Teoh AYB, 2017, ENDOSCOPY, V49, P130, DOI 10.1055/s-0042-119036
Tessier G, 2007, GASTROINTEST ENDOSC, V65, P233, DOI 10.1016/j.gie.2006.06.029
Tringali A, 2019, ANN GASTROENTEROL, V32, P330, DOI 10.20524/aog.2019.0390
Tyberg A, 2020, J CLIN GASTROENTEROL, V54, P569, DOI 10.1097/MCG.0000000000001326

Tyberg A, 2020, ENDOSC INT OPEN, V8, pE92, DOI 10.1055/a-1005-6602
Tyberg A, 2018, J CLIN GASTROENTEROL, V52, P79, DOI 10.1097/MCG.0000000000000786
Tyberg A, 2017, CLIN ENDOSC, V50, P185, DOI 10.5946/ce.2016.030
Tyberg A, 2017, GASTROINTEST ENDOSC, V85, P164, DOI 10.1016/j.gie.2016.07.030
Tyberg A, 2016, ENDOSC INT OPEN, V4, pE276, DOI 10.1055/s-0042-101789
Uemura RS, 2018, J CLIN GASTROENTEROL, V52, P123, DOI 10.1097/MCG.0000000000000948
Walter D, 2016, GUT, V65, P6, DOI 10.1136/gutjnl-2015-309925
Wang TJ, 2019, SURG ENDOSC, V33, P2024, DOI 10.1007/s00464-019-06715-z
Widmer JL, 2019, GASTROINTEST ENDOSC, V89, pAB291, DOI 10.1016/j.gie.2019.03.1175
Wiersema MJ, 1996, GASTROINTEST ENDOSC, V43, P102, DOI 10.1016/S0016-5107(06)80108-2
Will U, 2015, WORLD J GASTROENTERO, V21, P13140, DOI 10.3748/wjg.v21.i46.13140
Xu MM, 2020, CLIN ENDOSC, V53, P94, DOI 10.5946/ce.2018.196
NR 95
TC 6
Z9 7
U1 0
U2 5
PU AME PUBLISHING COMPANY
PI SHATIN
PA FLAT-RM C 16F, KINGS WING PLAZA 1, NO 3 KWAN ST, SHATIN, HONG KONG
00000, PEOPLES R CHINA
EI 2415-1289
J9 TRANSL GASTROENT HEP
JI Transl. Gastroenterol. Hepatol.
PD APR
PY 2022
VL 7
AR 20
DI 10.21037/tgh-2020-12
PG 14
WC Gastroenterology & Hepatology
WE Emerging Sources Citation Index (ESCI)
SC Gastroenterology & Hepatology
GA 0Z5NX
UT WOS:000791125900005
PM 35548470
OA Green Submitted, gold
DA 2026-05-09
ER

PT J
AU Voiosu, TA

Rimbas, M
Larghi, A
AF Voiosu, Theodor Alexandru
Rimbas, Mihai
Larghi, Alberto
TI The Role of Lumen Apposing Metal Stents in the Palliation of Distal
Malignant Biliary Distal Obstruction
SO CANCERS
LA English
DT Review
DE lumen apposing metal stents (LAMS); biliary obstruction; endoscopic
ultrasonography
ID EUS-GUIDED CHOLEDOCHODUODENOSTOMY; GALLBLADDER DRAINAGE; FAILED ERCP;
HEPATICOGASTROSTOMY; MANAGEMENT; DELIVERY; TRIAL
AB Malignant biliary obstruction (DMBO) has been traditionally managed by endoscopic retrograde cholangiopancreatography (ERCP). In the case of ERC failure, percutaneous transhepatic biliary drainage (PT-BD) has been widely utilized as a salvage procedure. However, over the last decade, endoscopic ultrasound-guided biliary drainage (EUS-BD) has gained increasing popularity, especially after the advent of electrocautery-enhanced lumen apposing metal stent devices (EC-LAMSs) which enable a one-step procedure, granting prevention of biliary leakage and minimizing occurrence of adverse events (AEs). In parallel, increasing evidence suggests a possible role of EUS-BD in the management of DMBO as a primary palliative drainage modality. In the current paper, we aim to review all the available evidence on the role of EUS-BD performed with EC-LAMSs and discuss salient technical aspects of this type of procedure.
C1 [Voiosu, Theodor Alexandru; Rimbas, Mihai] Carol Davila Fac Med, Bucharest 021155, Romania.
[Voiosu, Theodor Alexandru; Rimbas, Mihai] Colentina Clin Hosp, Gastroenterol Dept, Bucharest 020125, Romania.
[Larghi, Alberto] Catholic Univ, Fdn Policlin Univ A Gemelli, Sci Inst Res Hospitalizat & Hlth Care, Digest Endoscopy Unit, I-00168 Rome, Italy.
C3 Catholic University of the Sacred Heart; IRCCS Policlinico Gemelli
RP Larghi, A (corresponding author), Catholic Univ, Fdn Policlin Univ A Gemelli, Sci Inst Res Hospitalizat & Hlth Care, Digest Endoscopy Unit, I-00168 Rome, Italy.
EM theodor.voiosu@gmail.com; mrimbas@gmail.com; alberto.larghi@yahoo.it
RI Rimbas, Mihai/AAF-5521-2020; Larghi, Alberto/AAC-7448-2022; Voiosu,
Theodor/C-2445-2012
CR Amato A, 2021, ENDOSCOPY, V53, P1037, DOI 10.1055/a-1324-7919
Anderloni A, 2019, WORLD J GASTROENTERO, V25, P3857, DOI 10.3748/wjg.v25.i29.3857
[Anonymous], 2022, Cancer Facts Figures 2021
Attili F, 2015, ENDOSCOPY, V47, pE619, DOI 10.1055/s-0034-1393669
Bang JY, 2017, GUT, V66, P2054, DOI 10.1136/gutjnl-2016-312812
bostonscientific.com, About Us
Chen YI, 2019, TRIALS, V20, DOI 10.1186/s13063-019-3918-y
Sanz MD, 2021, SURG ENDOSC, V35, P6754, DOI 10.1007/s00464-020-08179-y
Di Mitri R, 2022, SURG ENDOSC, V36, P4553, DOI 10.1007/s00464-021-08808-0
Fabbri C, 2019, DIGEST LIVER DIS, V51, pE126, DOI 10.1016/S1590-8658(19)30236-1

Facciorusso A, 2022, CANCERS, V14, DOI 10.3390/cancers14133291
Fugazza A, 2022, GASTROINTEST ENDOSC, V95, P896, DOI 10.1016/j.gie.2021.12.032
Garcia-Sumalla A, 2022, TRIALS, V23, DOI 10.1186/s13063-022-06106-1
Garcia-Sumalla A, 2021, SURG ENDOSC, V35, P4873, DOI 10.1007/s00464-021-08435-9
Geyl S, 2023, ENDOSC ULTRASOUND, V12, P237, DOI 10.4103/EUS-D-22-00120
Giovannini M, 2001, ENDOSCOPY, V33, P898, DOI 10.1055/s-2001-17324
Hassan Z, 2022, WORLD J GASTROENTERO, V28, P3514, DOI 10.3748/wjg.v28.i27.3514
Hayat U, 2022, ENDOSC ULTRASOUND, V11, P4, DOI 10.4103/EUS-D-21-00009
Imai H, 2016, GASTROINTEST ENDOSC, V84, P147, DOI 10.1016/j.gie.2015.12.024
Issa D, 2021, ENDOSCOPY, V53, P827, DOI 10.1055/a-1259-0349
Jo SJ, 2023, J HEPATO-BIL-PAN SCI, V30, P263, DOI 10.1002/jhbp.1180
Kakked G, 2020, ENDOSC ULTRASOUND, V9, P298, DOI 10.4103/eus.eus_10_20
Kamal F, 2023, ENDOSC ULTRASOUND, V12, P8, DOI 10.4103/EUS-D-21-00206
Khan MA, 2016, DIGEST DIS SCI, V61, P684, DOI 10.1007/s10620-015-3933-0
Kim R, 2010, J GASTROINTEST SURG, V14, P1159, DOI 10.1007/s11605-010-1213-z
Krishnamoorthi R, 2020, SURG ENDOSC, V34, P2866, DOI 10.1007/s00464-020-07484-w
Larghi A, 2019, ENDOSC ULTRASOUND, V8, P217, DOI 10.4103/eus.eus_61_18
Li JS, 2022, FRONT SURG, V9, DOI 10.3389/fsurg.2022.811005
O'Brien S, 2020, SURG ENDOSC, V34, P1186, DOI 10.1007/s00464-019-06871-2
Principe DR, 2021, FRONT ONCOL, V11, DOI 10.3389/fonc.2021.688377
Rimbas M, 2021, ENDOSC INT OPEN, V09, pE1801, DOI 10.1055/a-1526-1208
Robles-Medranda C, 2023, GASTROINTEST ENDOSC, V97, P445, DOI 10.1016/j.gie.2022.10.037
Schaefer M, 2019, ENDOSCOPY, V51, pE219, DOI 10.1055/a-0885-9196
Schmitz D, 2022, PLOS ONE, V17, DOI 10.1371/journal.pone.0275029
Shah JM, 2023, ENDOSCOPY, V55, pE416, DOI 10.1055/a-2008-0431
Tanisaka Y, 2023, SCAND J GASTROENTERO, V58, P107, DOI 10.1080/00365521.2022.2111228
Teoh AYB, 2014, GASTROINTEST ENDOSC, V80, P1171, DOI 10.1016/j.gie.2014.03.038
Tyberg Amy, 2023, J Clin Gastroenterol, V57, P962, DOI 10.1097/MCG.0000000000001795
Tyberg A, 2022, ENDOSC ULTRASOUND, V11, P38, DOI 10.4103/EUS-D-21-00006
Uemura RS, 2018, J CLIN GASTROENTEROL, V52, P123, DOI 10.1097/MCG.0000000000000948
van der Merwe SW, 2022, ENDOSCOPY, V54, P185, DOI 10.1055/a-1717-1391
Varadarajulu S, 2008, GASTROINTEST ENDOSC, V67, P1180, DOI 10.1016/j.gie.2008.02.005
Versteijne E, 2022, J CLIN ONCOL, V40, P1220, DOI 10.1200/JCO.21.02233
Voiosu T., GASTROINTEST ENDOSC, P2023
Wang KX, 2016, GASTROINTEST ENDOSC, V83, P1218, DOI 10.1016/j.gie.2015.10.033
Yoo J, 2017, ENDOSC ULTRASOUND, V6, P131, DOI 10.4103/2303-9027.204814
Zhang Linda Y, 2022, Gastrointest Endosc Clin N Am, V32, P563, DOI [10.1016/j.giec.2022.02.001, 10.1016/j.giec.2022.02.001]
NR 47
TC 2
Z9 2
U1 0

U2 3
PU MDPI
PI BASEL
PA ST ALBAN-ANLAGE 66, CH-4052 BASEL, SWITZERLAND
EI 2072-6694
J9 CANCERS
JI Cancers
PD MAY 12
PY 2023
VL 15
IS 10
AR 2730
DI 10.3390/cancers15102730
PG 10
WC Oncology
WE Science Citation Index Expanded (SCI-EXPANDED)
SC Oncology
GA H7XQ9
UT WOS:000998051500001
PM 37345067
OA Green Submitted, gold
DA 2026-05-09
ER

PT J
AU Kadah, A
Khoury, T
Mari, A
Mahamid, M
Sbeit, W
AF Kadah, Anas
Khoury, Tawfik
Mari, Amir
Mahamid, Mahmud
Sbeit, Wisam
TI Lumen-apposing metal stents in interventional endoscopy: a
state-of-the-art review with focus on technical and clinical successes
and complications
SO EUROPEAN JOURNAL OF GASTROENTEROLOGY & HEPATOLOGY
LA English
DT Review
DE complications; clinical; lumens apposing metal stents; safety; success;

technical

ID PANCREATIC FLUID COLLECTIONS; EUS-GUIDED DRAINAGE; MALIGNANT BILIARY OBSTRUCTION; GALLBLADDER DRAINAGE; PLASTIC STENTS; DOUBLE-PIGTAIL; DUODENAL OBSTRUCTION; PSEUDOCYST DRAINAGE; DUCT DRAINAGE; CHOLEDOCHODUODENOSTOMY

AB Endoscopic transmural drainage is considered the initial treatment option for pancreatic fluid collections. Recently, lumen-apposing metal stents have been introduced for pancreatic fluid collections drainage under endoscopic ultrasound guidance, in addition to evolving data in bile duct and gallbladder drainage. We aimed to perform systematic review with reporting pooled data analysis on technical success, clinical success and complications rate of endoscopic ultrasound-guided lumen-apposing metal stents uses. A MEDLINE/PubMed and EMBASE search for all studies on lumen-apposing metal stents uses in pancreatic, biliary and other indications was conducted. Data regarding safety, complications and yield were extracted and included in the final pooled analysis. Overall, 19 articles dealing with pancreatic fluid collections drainage, 18 articles reporting on bile duct drainage and 19 articles relevant to gallbladder drainage were identified. Technical and clinical successes in all disease conditions were high and reached more than 95%. Complications rate in pancreatic fluid collections, bile duct and gallbladder drainages were 12%, 11.2% and 9.8%, respectively. Endoscopic ultrasound-guided lumen-apposing metal stents is technically feasible with very high success rate and acceptable complications rate.

C1 [Kadah, Anas; Khoury, Tawfik; Sbeit, Wisam] Galilee Med Ctr, Dept Gastroenterol, IL-22100 Nahariyya, Israel.

[Kadah, Anas; Khoury, Tawfik; Mari, Amir; Mahamid, Mahmud; Sbeit, Wisam] Bar Ilan Univ, Fac Med Galilee, Safed, Israel.

[Khoury, Tawfik; Mari, Amir; Mahamid, Mahmud] Nazareth Hosp, Gastroenterol & Endoscopy United, EMMS, Nazareth, Israel.

C3 Western Galilee Hospital; Bar Ilan University

RP Khoury, T (corresponding author), Galilee Med Ctr, Dept Gastroenterol, IL-22100 Nahariyya, Israel.

EM tawfikkhoury1@hotmail.com

CR Abu Dayyeh BK, 2018, GASTROINTEST ENDOSC, V87, P141, DOI 10.1016/j.gie.2017.04.032

Aburajab M, 2018, GASTROINTEST ENDOSC, V87, P1248, DOI 10.1016/j.gie.2017.11.033

Alali A, 2017, CLIN ENDOSC, V50, P117, DOI 10.5946/ce.2017.045

Anderloni A, 2018, ANN GASTROENTEROL, V31, P735, DOI 10.20524/aog.2018.0299

Anderloni A, 2016, ENDOSCOPY, V48, pE321, DOI 10.1055/s-0042-117425

Ang TL, 2016, ENDOSC ULTRASOUND, V5, P320, DOI 10.4103/2303-9027.191659

Attili F, 2015, ENDOSCOPY, V47, pE619, DOI 10.1055/s-0034-1393669

Bang JY, 2017, GUT, V66, P2054, DOI 10.1136/gutjnl-2016-312812

Banks PA, 2013, GUT, V62, P102, DOI 10.1136/gutjnl-2012-302779

Bapaye A, 2015, DIGEST ENDOSC, V27, P499, DOI 10.1111/den.12421

Binmoeller KF, 2011, ENDOSCOPY, V43, P337, DOI 10.1055/s-0030-1256127

Chandran S, 2015, GASTROINTEST ENDOSC, V81, P127, DOI 10.1016/j.gie.2014.06.025

Cho DH, 2019, SURG ENDOSC, V33, P2135, DOI 10.1007/s00464-018-6485-5

de la Serna-Higuera C, 2013, GASTROINTEST ENDOSC, V77, P303, DOI 10.1016/j.gie.2012.09.021

DeSimone ML, 2017, WORLD J GASTRO ENDOS, V9, P456, DOI 10.4253/wjge.v9.i9.456

Dhir V, 2015, GASTROINTEST ENDOSC, V82, P650, DOI 10.1016/j.gie.2015.01.061

Dollhopf M, 2017, GASTROINTEST ENDOSC, V86, P636, DOI 10.1016/j.gie.2017.02.027

French JB, 2016, CLIN J GASTROENTEROL, V9, P79, DOI 10.1007/s12328-016-0634-y

Ge N, 2017, J TRANSL INTERN MED, V5, P213, DOI 10.1515/jtim-2017-0036
GLENN F, 1977, ANN SURG, V185, P185, DOI 10.1097/00000658-197702000-00009
Glessing BR, 2015, GASTROINTEST ENDOSC, V81, P1019, DOI 10.1016/j.gie.2014.09.061
Gornals JB, 2016, DIGEST ENDOSC, V28, P100, DOI 10.1111/den.12548
Gornals JB, 2013, SURG ENDOSC, V27, P1428, DOI 10.1007/s00464-012-2591-y
Hara K, 2013, ENDOSCOPY, V45, P392, DOI 10.1055/s-0032-1326076
Huggett MT, 2015, ENDOSCOPY, V47, P929, DOI 10.1055/s-0034-1392413
Irani S, 2017, CLIN GASTROENTEROL H, V15, P738, DOI 10.1016/j.cgh.2016.12.021
Irani S, 2015, GASTROINTEST ENDOSC, V82, P1110, DOI 10.1016/j.gie.2015.05.045
Itoi T, 2015, GASTROINTEST ENDOSC, V81, P1259, DOI 10.1016/j.gie.2014.08.033
Itoi T, 2014, GASTROINTEST ENDOSC, V79, P715, DOI 10.1016/j.gie.2013.11.021
Itoi T, 2013, DIGEST ENDOSC, V25, P137, DOI 10.1111/den.12084
Itoi T, 2013, J HEPATO-BIL-PAN SCI, V20, P403, DOI 10.1007/s00534-012-0551-5
Itoi T, 2012, GASTROINTEST ENDOSC, V75, P870, DOI 10.1016/j.gie.2011.10.020
Jacques J, 2019, ENDOSCOPY, V51, P540, DOI 10.1055/a-0735-9137
Jang JW, 2011, GASTROINTEST ENDOSC, V74, P176, DOI 10.1016/j.gie.2011.03.1120
Kumar M, 2018, ACG CASE REP J, V5, DOI 10.14309/crj.2018.41
Kunda R, 2016, SURG ENDOSC, V30, P5002, DOI 10.1007/s00464-016-4845-6
Lakhtakia S, 2016, ENDOSCOPY, V48, pE180, DOI 10.1055/s-0042-106966
Law R, 2016, CLIN GASTROENTEROL H, V14, P476, DOI 10.1016/j.cgh.2015.10.026
Ligresti D, 2019, ENDOSCOPY, V51, P94, DOI 10.1055/a-0754-1847
McKay A, 2012, SURG ENDOSC, V26, P1343, DOI 10.1007/s00464-011-2035-0
Mönkemüller K, 2013, ENDOSCOPY, V45, pE164, DOI 10.1055/s-0032-1325776
Moon JH, 2014, GASTROINTEST ENDOSC, V79, P990, DOI 10.1016/j.gie.2014.02.015
Mudireddy PR, 2018, GASTROINTEST ENDOSC, V87, P1256, DOI 10.1016/j.gie.2017.08.011
Mukai S, 2016, GASTROINTEST ENDOSC, V83, P832, DOI 10.1016/j.gie.2015.09.047
Mukai S, 2015, ENDOSC ULTRASOUND, V4, P120, DOI 10.4103/2303-9027.156738
Ngamruengphong S, 2017, ENDOSCOPY, V49, P549, DOI 10.1055/s-0043-105072
Perez-Miranda M, 2014, GASTROINTEST ENDOSC, V80, P339, DOI 10.1016/j.gie.2014.04.030
Püspök A, 2005, AM J GASTROENTEROL, V100, P1743, DOI 10.1111/j.1572-0241.2005.41806.x
Rinninella E, 2015, GASTROINTEST ENDOSC, V82, P1039, DOI 10.1016/j.gie.2015.04.006
Sandru V, 2018, TURK J GASTROENTEROL, V29, P511, DOI 10.5152/tjg.2018.18095
Sejpal DV, 2015, GASTROINTEST ENDOSC, V82, P170, DOI 10.1016/j.gie.2015.02.002
Shah RJ, 2015, CLIN GASTROENTEROL H, V13, P747, DOI 10.1016/j.cgh.2014.09.047
Sharaiha RZ, 2016, CLIN GASTROENTEROL H, V14, P1797, DOI 10.1016/j.cgh.2016.05.011
Siddiqui A, 2019, SURG ENDOSC, V33, P1260, DOI 10.1007/s00464-018-6406-7
Siddiqui AA, 2017, GASTROINTEST ENDOSC, V85, P758, DOI 10.1016/j.gie.2016.08.014
Siddiqui AA, 2016, GASTROINTEST ENDOSC, V83, P699, DOI 10.1016/j.gie.2015.10.020
Siddiqui AA, 2011, SURG ENDOSC, V25, P549, DOI 10.1007/s00464-010-1216-6
Suarez Alejandro L, 2018, VideoGIE, V3, P364, DOI [10.1016/j.vgie.2018.08.016, 10.1016/j.vgie.2018.08.016]
Teoh AYB, 2014, ENDOSCOPY, V46, pE13, DOI 10.1055/s-0033-1359139
Teoh AYB, 2014, GASTROINTEST ENDOSC, V80, P1171, DOI 10.1016/j.gie.2014.03.038

Tsuchiya T, 2018, GASTROINTEST ENDOSC, V87, P1138, DOI 10.1016/j.gie.2017.08.017
Turner BG, 2013, ENDOSCOPY, V45, pE114, DOI 10.1055/s-0032-1325968
Tyberg A, 2016, ENDOSC INT OPEN, V4, pE276, DOI 10.1055/s-0042-101789
Tyberg A, 2015, ENDOSCOPY, V47, pE342, DOI 10.1055/s-0034-1392318
Varadarajulu S, 2013, GASTROENTEROLOGY, V145, P583, DOI 10.1053/j.gastro.2013.05.046
Vazquez-Sequeiros E, 2016, GASTROINTEST ENDOSC, V84, P450, DOI 10.1016/j.gie.2016.02.044
Venkatachalapathy SV, 2018, ENDOSC INT OPEN, V6, pE259, DOI 10.1055/s-0043-125362
Vila JJ, 2012, GASTROINTEST ENDOSC, V76, P1133, DOI 10.1016/j.gie.2012.08.001
Walter D, 2016, GUT, V65, P6, DOI 10.1136/gutjnl-2015-309925
Walter D, 2015, ENDOSCOPY, V47, P63, DOI 10.1055/s-0034-1378113
Yamamoto N, 2013, GASTROINTEST ENDOSC, V77, P809, DOI 10.1016/j.gie.2013.01.009
Yamao K, 2008, ENDOSCOPY, V40, P340, DOI 10.1055/s-2007-995485
Yang D, 2017, ENDOSCOPY, V49, P327, DOI 10.1055/s-0042-122779
Yoo J, 2017, ENDOSC ULTRASOUND, V6, P131, DOI 10.4103/2303-9027.204814
Zhu HY, 2018, TRIALS, V19, DOI 10.1186/s13063-018-2901-3
NR 75
TC 4
Z9 6
U1 0
U2 3
PU LIPPINCOTT WILLIAMS & WILKINS
PI PHILADELPHIA
PA TWO COMMERCE SQ, 2001 MARKET ST, PHILADELPHIA, PA 19103 USA
SN 0954-691X
EI 1473-5687
J9 EUR J GASTROEN HEPAT
JI Eur. J. Gastroenterol. Hepatol.
PD JAN
PY 2020
VL 32
IS 1
BP 1
EP 9
DI 10.1097/MEG.0000000000001571
PG 9
WC Gastroenterology & Hepatology
WE Science Citation Index Expanded (SCI-EXPANDED)
SC Gastroenterology & Hepatology
GA NC6TW
UT WOS:000561350800001
PM 31651655
DA 2026-05-09

ER

PT J
AU Ogura, T
Nishioka, N
Yamada, M
Yamada, T
Higuchi, K
AF Ogura, Takeshi
Nishioka, Nobu
Yamada, Masanori
Yamada, Tadahiro
Higuchi, Kazuhide
TI EUS-Guided Gallbladder Drainage Using an Improved Self-Expandable
Covered Metal Stent with Anti-Stent Migration System (with Video)
SO DIGESTIVE DISEASES
LA English
DT Article
DE Endoscopic ultrasound; Endoscopic ultrasound-guided biliary drainage;
Endoscopic ultrasound-guided gallbladder drainage; Eendoscopic
retrograde cholangiopancreatography
AB Background: Recently, lumen-apposing metal stents (LAMS) have been developed as novel devices for not only endoscopic ultrasound (EUS)-guided pancreatic fluid drainage but also for EUS-guided gallbladder drainage (GBD). Although LAMS might be clinically impactful, these stents have not yet become available as EUS-GBD stents in all countries, including Japan. Instead, improved metal stents (NEO stents) have become available. The aim of this pilot study was to evaluate the feasibility and safety of EUS-GBD using the improved metal stent with an anti-stent migration system for acute cholecystitis (AC). Method: The gallbladder was punctured using a 19-G needle, and a 0.025-inch guidewire was inserted into the gallbladder. After fistula dilation, stent deployment was performed from the gallbladder to the duodenum. Finally, a plastic 7-Fr pigtail stent was placed to prevent stent migration. Result: EUS-GBD using NEO stents with the anti-stent migration system was attempted in 12 consecutive patients with AC as a complication of other conditions. EUS-GBD was successfully performed in all patients without any adverse events, and clinical success was achieved in all patients. Median procedure time was 19.0 min (range, 19-24 min). In 4 patients, stent removal was successfully performed after 3 months. Stent migration during follow-up (median, 189 days) was not seen in any of the patients. Conclusion: Although this study has several limitations such as small number of patients and short follow-up period, the results show that NEO stents might be suitable as EUS-GBD stents in selected patients. Our results should be validated by a larger prospective, randomized study.
C1 [Ogura, Takeshi; Nishioka, Nobu; Yamada, Masanori; Yamada, Tadahiro; Higuchi, Kazuhide] Osaka Med Coll, Dept Internal Med 2, 2-7 Daigakuchou, Osaka 5698686, Japan.
C3 Osaka Medical & Pharmaceutical University
RP Ogura, T (corresponding author), Osaka Med Coll, Dept Internal Med 2, 2-7 Daigakuchou, Osaka 5698686, Japan.
EM oguratakeshi0411@yahoo.co.jp
RI Ogura, Takeshi/AAU-5643-2021

CR Choi JH, 2014, ENDOSCOPY, V46, P656, DOI 10.1055/s-0034-1365720
de la Serna-Higuera C, 2013, GASTROINTEST ENDOSC, V77, P303, DOI 10.1016/j.gie.2012.09.021
Dollhopf M, 2017, GASTROINTEST ENDOSC, V86, P636, DOI 10.1016/j.gie.2017.02.027
Irani S, 2015, GASTROINTEST ENDOSC, V82, P1110, DOI 10.1016/j.gie.2015.05.045
Jang JW, 2012, GASTROENTEROLOGY, V142, P805, DOI 10.1053/j.gastro.2011.12.051
Jang JW, 2011, GASTROINTEST ENDOSC, V74, P176, DOI 10.1016/j.gie.2011.03.1120
Kahaleh M, 2016, DIGEST LIVER DIS, V48, P1054, DOI 10.1016/j.dld.2016.05.021
Kamata K, 2017, WORLD J GASTROENTERO, V23, P661, DOI 10.3748/wjg.v23.i4.661
Kedia P, 2015, GASTROINTEST ENDOSC, V82, P1031, DOI 10.1016/j.gie.2015.03.1912
Khan MA, 2017, GASTROINTEST ENDOSC, V85, P76, DOI 10.1016/j.gie.2016.06.032
Okamoto K, 2018, J HEPATO-BIL-PAN SCI, V25, P55, DOI 10.1002/jhbp.516
Song TJ, 2010, GASTROINTEST ENDOSC, V71, P634, DOI 10.1016/j.gie.2009.11.024
Takagi W, 2016, THER ADV GASTROENTER, V9, P19, DOI 10.1177/1756283X15609285
Tyberg A, 2018, J CLIN GASTROENTEROL, V52, P79, DOI 10.1097/MCG.0000000000000786
Walter D, 2016, GUT, V65, P6, DOI 10.1136/gutjnl-2015-309925
NR 15
TC 6
Z9 7
U1 0
U2 2
PU KARGER
PI BASEL
PA ALLSCHWILERSTRASSE 10, CH-4009 BASEL, SWITZERLAND
SN 0257-2753
EI 1421-9875
J9 DIGEST DIS
JI Dig. Dis.
PD MAR
PY 2021
VL 39
IS 2
BP 150
EP 155
DI 10.1159/000509215
PG 6
WC Gastroenterology & Hepatology
WE Science Citation Index Expanded (SCI-EXPANDED)
SC Gastroenterology & Hepatology
GA QY6NM
UT WOS:000630154200007
PM 32512575
DA 2026-05-09

ER

PT J
AU Mcdonald, NM
Bilal, M
Azeem, N
Amateau, SK
AF Mcdonald, Nicholas M.
Bilal, Mohammad
Azeem, Nabeel
Amateau, Stuart K.
TI Practice Patterns of Endoscopists Performing Endoscopic Gallbladder
Drainage
SO TECHNIQUES AND INNOVATIONS IN GASTROINTESTINAL ENDOSCOPY
LA English
DT Article
DE Gallbladder drainage; Transpapillary drainage; Endoscopic
ultrasound-guided gallbladder drainage; Lumen apposing metal stent
ID ACUTE CHOLECYSTITIS; STENT PLACEMENT; MANAGEMENT; THERAPY; LONG
AB BACKGROUND AND AIMS: The standard of care for treatment of patients with acute cholecystitis is cholecystectomy. However, many patients are unfit for surgery due to substantial medical comorbidities, or surgery is technically challenging due to anatomic considerations. Options for patients who are not surgical candidates include percutaneous cholecystostomy tube placement by interventional radiology, endoscopic gallbladder drainage (EGBD), or conservative management. Over the last decade, techniques of EGBD have been employed for temporary or definitive gallbladder drainage in patients with acute cholecystitis who are not surgical candidates. Despite growing interest and clinical use of EGBD, little is known about practice patterns of EGBD. Our aim was to survey endoscopists to evaluate the practice patterns and technical considerations regarding EGBD. METHODS: An 18-item survey was distributed to all members of the American Society of Gastrointestinal Endoscopy. Each response was included in the final analysis. Descriptive statistics were calculated using frequencies and percentages. RESULTS: Responses were received from 217 endoscopists. Of these, 178 perform endoscopic ultrasound-guided gallbladder draining and 178 perform endoscopic transpapillary gallbladder drainage. The preferred approach for EGBD was endoscopic ultrasound-guided gallbladder drainage in 58.8%, endoscopic retrograde cholangiopancreatography (ERCP)-guided transpapillary gallbladder stent placement in 30.2%, and no preference in 11%. CONCLUSION: Despite growing interest in EGBD, significant heterogeneity in practice patterns exists. Further study is needed to better understand these differences and provide data for future guidelines.
C1 [Mcdonald, Nicholas M.; Bilal, Mohammad; Azeem, Nabeel; Amateau, Stuart K.] Univ Minnesota, Div Gastroenterol Hepatol & Nutr, Minneapolis, MN USA.
[Mcdonald, Nicholas M.] Univ Minnesota, Div Gastroenterol Hepatol & Nutr, 500 SE Harvard St,MMC 36, Minneapolis, MN 55454 USA.
C3 University of Minnesota System; University of Minnesota Twin Cities;
University of Minnesota System; University of Minnesota Twin Cities
RP Mcdonald, NM (corresponding author), Univ Minnesota, Div Gastroenterol Hepatol & Nutr, 500 SE Harvard St,MMC

36, Minneapolis, MN 55454 USA.

EM Mcdon520@umn.edu

OI McDonald, Nicholas/0000-0003-3226-1718

CR Avila P, 2020, GASTROINTEST ENDOSC, V91, P568, DOI 10.1016/j.gie.2019.11.013

Barnhart BJ, 2021, EVAL HEALTH PROF, V44, P245, DOI 10.1177/01632787211019445

Baron TH, 2007, GASTROINTEST ENDOSC, V65, P735, DOI 10.1016/j.gie.2006.07.041

Boregowda Umesha, 2018, World J Gastrointest Pharmacol Ther, V9, P47, DOI [10.4292/wjgpt.v9.i6.47, 10.4292/wjgpt.v9.i6.47]

Chan WPW, 2021, J DIGEST DIS, V22, P463, DOI 10.1111/1751-2980.13031

Cook DA, 2016, J MED INTERNET RES, V18, DOI 10.2196/jmir.6318

Gallaher JR, 2022, JAMA-J AM MED ASSOC, V327, P965, DOI 10.1001/jama.2022.2350

Glessing BR, 2015, DIGEST DIS SCI, V60, P3817, DOI 10.1007/s10620-015-3814-6

Gomes CA, 2017, WORLD J GASTRO SURG, V9, P118, DOI 10.4240/wjgs.v9.i5.118

James TW, 2019, ENDOSC ULTRASOUND, V8, pS28, DOI 10.4103/eus.eus_41_19

Janssen ERI, 2020, SURG INFECT, V21, P428, DOI 10.1089/sur.2019.261

Kedia P, 2015, GASTROINTEST ENDOSC, V82, P1031, DOI 10.1016/j.gie.2015.03.1912

Khan MA, 2017, GASTROINTEST ENDOSC, V85, P76, DOI 10.1016/j.gie.2016.06.032

Liang Peter S, 2014, J Clin Gastroenterol, V48, pe93, DOI 10.1097/MCG.0000000000000095

McCarthy ST, 2015, DIGEST DIS SCI, V60, P2516, DOI 10.1007/s10620-014-3371-4

McGillicuddy EA, 2012, BRIT J SURG, V99, P1254, DOI 10.1002/bjs.8836

Mukai S, 2017, J HEPATO-BIL-PAN SCI, V24, P537, DOI 10.1002/jhbp.496

Mutignani M, 2009, ENDOSCOPY, V41, P539, DOI 10.1055/s-0029-1214727

Pannala R, 2008, Minerva Gastroenterol Dietol, V54, P107

Singh M, 2013, GASTROINTEST ENDOSC, V78, P689, DOI 10.1016/j.gie.2013.05.002

Sofuni A, 2019, ENDOSC INT OPEN, V7, pE1115, DOI 10.1055/a-0838-5456

Storm AC, 2021, GASTROINTEST ENDOSC, V94, P742, DOI 10.1016/j.gie.2021.03.025

Teoh AYB, 2020, GUT, V69, P1085, DOI 10.1136/gutjnl-2019-319996

Vozzo Catherine Frakes, 2020, VideoGIE, V5, P500, DOI [10.1016/j.vgie.2020.07.009, 10.1016/j.vgie.2020.07.009]

NR 24

TC 1

Z9 1

U1 0

U2 1

PU ELSEVIER SCIENCE INC

PI NEW YORK

PA STE 800, 230 PARK AVE, NEW YORK, NY 10169 USA

SN 2666-5107

EI 2590-0307

J9 TECH INNOVAT GASTROI

JI Tech. Innovation Gastrointes. Endoscopy

PY 2023

VL 25

IS 3
BP 221
EP 227
DI 10.1016/j.tige.2023.03.005
EA JUL 2023
PG 7
WC Gastroenterology & Hepatology
WE Emerging Sources Citation Index (ESCI)
SC Gastroenterology & Hepatology
GA IH4I1
UT WOS:001165419400001
DA 2026-05-09
ER

PT J
AU Salerno, R
Davies, SEC
Mezzina, N
Ardizzone, S
AF Salerno, Raffaele
Davies, Sophia Elizabeth Campbell
Mezzina, Nicola
Ardizzone, Sandro
TI Comprehensive review on EUS-guided biliary drainage
SO WORLD JOURNAL OF GASTROINTESTINAL ENDOSCOPY
LA English
DT Review
DE Endoscopic ultrasonography-guided biliary drainage; EUS; Percutaneous transhepatic biliary drainage; Endoscopic ultrasonography-guided hepatogastric anastomosis; Endoscopic ultrasonography-guided antegrade stent placement; Endoscopic ultrasonography-guided choledochoduodenostomy; Endoscopic ultrasonography-guided transgallbladder; Endoscopic ultrasonography-guided rendezvous
ID APPOSING METAL STENT; PERCUTANEOUS DRAINAGE; FAILED ERCP; MALIGNANT OBSTRUCTION; GALLBLADDER DRAINAGE; CHOLEDOCHODUODENOSTOMY; HEPATICOGASTROSTOMY; COMPLICATIONS; ALGORITHM; ACCESS
AB Feasibility of endoscopic retrograde cholangiopancreatography (ERCP) for biliary drainage is not always applicable due to anatomical alterations or to inability to access the papilla. Percutaneous transhepatic biliary drainage has always been considered the only alternative for this indication. However, endoscopic ultrasonography-guided biliary drainage represents a valid option to replace percutaneous transhepatic biliary drainage when ERCP fails. According to the access site to the biliary tree, two kinds of approaches may be described: the intrahepatic and the extrahepatic. Endoscopic ultrasonography-guided rendezvous transpapillary drainage is performed where the second portion of the duodenum is easily reached but

conventional ERCP fails. The recent introduction of self-expandable metal stents and lumen-apposing metal stents has improved this field. However, the role of the latter is still controversial. Echoendoscopic transmural biliary drainage can be challenging with potential severe adverse events. Therefore, trained endoscopists, in both ERCP and endoscopic ultrasonography are needed with surgical and radiological backup.
C1 [Salerno, Raffaele; Mezzina, Nicola; Ardizzone, Sandro] Univ Milan, Gastroenterol & Digest Endoscopy Unit, ASST Fatebenefratelli Sacco, Dept Biochem & Clin Sci L Sacco, Corso Portz Nuova 23, I-20100 Milan, Italy.

[Davies, Sophia Elizabeth Campbell] ASST Fatebenefratelli Sacco, Hosp Pharm, Piazza Principessa Clotilde, I-20121 Milan, Italy.

C3 University of Milan

RP Salerno, R (corresponding author), Univ Milan, Gastroenterol & Digest Endoscopy Unit, ASST Fatebenefratelli Sacco, Dept Biochem & Clin Sci L Sacco, Corso Portz Nuova 23, I-20100 Milan, Italy.

EM raffaele.salerno@asst-fbf-sacco.it

RI Ardizzone, Sandro/J-3251-2017
CR Anderloni A, 2019, GASTROINTEST ENDOSC, V89, P69, DOI [10.1016/j.gie.2018.04.1746, 10.1016/j.gie.2018.08.047]

Artifon ELA, 2015, GASTROINTEST ENDOSC, V81, P950, DOI 10.1016/j.gie.2014.09.047

Artifon ELA, 2012, J CLIN GASTROENTEROL, V46, P768, DOI 10.1097/MCG.0b013e31825f264c

Bapaye A, 2013, UNITED EUR GASTROENT, V1, P285, DOI 10.1177/2050640613490928

Bill J, 2015, GASTROINTEST ENDOSC, V81, pAB531, DOI 10.1016/j.gie.2015.03.1806

Coté GA, 2012, CLIN GASTROENTEROL H, V10, P920, DOI 10.1016/j.cgh.2012.02.019

Giovannini M, 2001, ENDOSCOPY, V33, P898, DOI 10.1055/s-2001-17324

Giovannini M, 2015, GASTROINTEST ENDOSC, V81, pAB174, DOI 10.1016/j.gie.2015.03.1926

Guo JT, 2018, ENDOSC ULTRASOUND, V7, P356, DOI 10.4103/eus.eus_53_18

Hara K, 2016, WORLD J GASTROENTERO, V22, P1297, DOI 10.3748/wjg.v22.i3.1297

Holt BA, 2016, GASTROINTEST ENDOSC, V83, P160, DOI 10.1016/j.gie.2015.06.019

Imai H, 2016, GASTROINTEST ENDOSC, V84, P147, DOI 10.1016/j.gie.2015.12.024

Itoi T, 2013, DIGEST ENDOSC, V25, P137, DOI 10.1111/den.12084

Iwashita T, 2012, ENDOSCOPY, V44, P60, DOI 10.1055/s-0030-1256871

Iwashita T, 2016, GASTROINTEST ENDOSC, V83, P394, DOI 10.1016/j.gie.2015.04.043

Jang JW, 2012, GASTROENTEROLOGY, V142, P805, DOI 10.1053/j.gastro.2011.12.051

Kahaleh M, 2013, WORLD J GASTROENTERO, V19, P1372, DOI 10.3748/wjg.v19.i9.1372

Kalva NR, 2018, CAN J GASTROENTEROL, V2018, DOI 10.1155/2018/7070961

Kawakubo K, 2016, ENDOSCOPY, V48, P164, DOI 10.1055/s-0034-1393179

Khan MA, 2016, DIGEST DIS SCI, V61, P684, DOI 10.1007/s10620-015-3933-0

Khashab MA, 2016, ENDOSC INT OPEN, V4, pE175, DOI 10.1055/s-0041-109083

Khashab MA, 2015, DIGEST DIS SCI, V60, P557, DOI 10.1007/s10620-014-3300-6

Lee TH, 2016, CLIN GASTROENTEROL H, V14, P1011, DOI 10.1016/j.cgh.2015.12.032

Nakai Y, 2019, ENDOSC ULTRASOUND, V8, P111, DOI 10.4103/eus.eus_17_18

Nennstiel S, 2015, J CLIN GASTROENTEROL, V49, P764, DOI 10.1097/MCG.0000000000000275

Oh D, 2017, THER ADV GASTROENTER, V10, P42, DOI 10.1177/1756283X16671671

Park DH, 2013, GASTROINTEST ENDOSC, V78, P91, DOI 10.1016/j.gie.2013.01.042

Savides TJ, 2009, GASTROINTEST ENDOSC, V69, pS3, DOI 10.1016/j.gie.2008.10.060

Sharaiha RZ, 2017, GASTROINTEST ENDOSC, V85, P904, DOI 10.1016/j.gie.2016.12.023
Sharaiha RZ, 2016, SURG ENDOSC, V30, P5500, DOI 10.1007/s00464-016-4913-y
Song TJ, 2014, GASTROINTEST ENDOSC, V80, P707, DOI 10.1016/j.gie.2014.05.327
Sportes A, 2016, GASTROINTEST ENDOSC, V83, pAB522, DOI 10.1016/j.gie.2016.03.775
Torres-Ruiz MF, 2016, GASTROINTEST ENDOSC, V83, pAB356, DOI 10.1016/j.gie.2016.03.910
Tsuchiya T, 2018, GASTROINTEST ENDOSC, V87, P1138, DOI 10.1016/j.gie.2017.08.017
Tyberg A, 2016, GASTROINTEST ENDOSC, V84, P941, DOI 10.1016/j.gie.2016.05.035
Wang KX, 2016, GASTROINTEST ENDOSC, V83, P1218, DOI 10.1016/j.gie.2015.10.033
Wiersema MJ, 1996, GASTROINTEST ENDOSC, V43, P102, DOI 10.1016/S0016-5107(06)80108-2
Yarmohammadi H, 2016, CHIN CLIN ONCOL, V5, DOI 10.21037/cco.2016.10.07
NR 38
TC 35
Z9 45
U1 0
U2 3
PU BAISHIDENG PUBLISHING GROUP INC
PI PLEASANTON
PA 8226 REGENCY DR, PLEASANTON, CA 94588 USA
SN 1948-5190
J9 WORLD J GASTRO ENDOS
JI World J. Gastrointest. Endosc.
PD MAY 16
PY 2019
VL 11
IS 5
BP 354
EP 364
DI 10.4253/wjge.v11.i5.354
PG 11
WC Gastroenterology & Hepatology
WE Emerging Sources Citation Index (ESCI)
SC Gastroenterology & Hepatology
GA IC3WS
UT WOS:000470894200004
PM 31205596
OA Green Submitted, hybrid
DA 2026-05-09
ER

PT J
AU McDonagh, P
Awadelkarim, B

Leeds, JS
Nayar, MK
Oppong, KW
AF McDonagh, Padraic
Awadelkarim, Bidour
Leeds, John S.
Nayar, Manu K.
Oppong, Kofi W.
TI Endoscopic Ultrasound-Guided Gallbladder Drainage for Malignant Biliary Obstruction: A Systematic Review
SO CANCERS
LA English
DT Review
DE endoscopic ultrasound-guided gallbladder drainage; malignant biliary obstruction
ID ACUTE CHOLECYSTITIS; STENT; PALLIATION; MANAGEMENT
AB Background: Endoscopic ultrasound-guided gallbladder drainage (EUS-GBD) is a rescue technique for patients with malignant biliary obstruction who fail conventional treatment with ERCP or EUS-guided biliary drainage. The technique has been successfully employed in the management of acute cholecystitis in patients not fit for surgery. However, the evidence for its use in malignant obstruction is less robust. This review article aims to evaluate the data available at present to better understand the safety and efficacy of EUS-guided gallbladder drainage. Methods: A detailed literature review was conducted and several databases were searched for any studies relating to EUS-GBD in malignant biliary obstruction. Pooled rates with 95% confidence intervals were calculated for clinical success and adverse events. Results: Our search identified 298 studies related to EUS-GBD. The final analysis included 7 studies with 136 patients. The pooled rate of clinical success (95% CI) was 85% (78-90%, I-2: 0%). The pooled rate of adverse events (95% CI) was 13% (7-19%, I-2: 0%). Adverse events included: peritonitis, bleeding, bile leakage, stent migration, and stent occlusion. No deaths directly related to the procedure were reported; however, in some of the studies, deaths occurred due to disease progression. Conclusion: This review supports the use of EUS-guided gallbladder drainage as a rescue option for patients who have failed conventional measures.
C1 [McDonagh, Padraic; Awadelkarim, Bidour; Leeds, John S.; Nayar, Manu K.; Oppong, Kofi W.] Freeman Rd Hosp, Newcastle Tyne Hosp NHS Fdn Trust, HPB Med, Newcastle Upon Tyne NE7 7DN, England.
[Leeds, John S.] Newcastle Univ, Populat Hlth Sci Inst, Newcastle Upon Tyne NE1 7RU, England.
[Oppong, Kofi W.] Newcastle Univ, Translat & Clin Res Inst, Newcastle Upon Tyne NE1 7RU, England.
C3 Newcastle Freeman Hospital; Newcastle Upon Tyne Hospitals NHS Foundation Trust; Newcastle University - UK; Newcastle University - UK; Newcastle University - UK
RP Oppong, KW (corresponding author), Freeman Rd Hosp, Newcastle Tyne Hosp NHS Fdn Trust, HPB Med, Newcastle Upon Tyne NE7 7DN, England.
EM padraic.mcdonagh@nhs.net; bidour.awadelkarim@nhs.net; j.leeds@nhs.net; manu.nayar@nhs.net; kofi.oppong@nhs.net
RI ; Oppong, Kofi/R-7648-2018; Nayar, Manu/AAO-1414-2021
OI Leeds, John/0000-0002-5140-6225;

CR Anderloni A, 2019, GASTROINTEST ENDOSC, V89, P69, DOI [10.1016/j.gie.2018.04.1746, 10.1016/j.gie.2018.08.047]
Anderloni A, 2016, SURG ENDOSC, V30, P5200, DOI 10.1007/s00464-016-4894-x
Balik E, 2013, J ONCOL, V2013, DOI 10.1155/2013/201681
Baron TH, 2007, GASTROINTEST ENDOSC, V65, P735, DOI 10.1016/j.gie.2006.07.041
Binda C., 2021, ENDOSCOPY, V53, peP427
Chang JI, 2019, ENDOSC INT OPEN, V7, pE655, DOI 10.1055/a-0826-4309
Pu LZCT, 2016, GASTROENT RES PRACT, V2016, DOI 10.1155/2016/3296801
Chin JYL, 2020, ENDOSC INT OPEN, V08, pE1633, DOI 10.1055/a-1236-3217
Dhindsa BS, 2020, ENDOSC ULTRASOUND, V9, P101, DOI 10.4103/eus.eus_80_19
Fabbri C, 2022, SURG ENDOSC, V36, P7974, DOI 10.1007/s00464-022-09339-y
Ginestet C, 2022, SURG ENDOSC, V36, P3365, DOI 10.1007/s00464-021-08653-1
Hayat U, 2022, ENDOSC ULTRASOUND, V11, P4, DOI 10.4103/EUS-D-21-00009
Higa Jennifer T, 2019, Curr Treat Options Gastroenterol, V17, P357, DOI [10.1007/s11938-019-00243-4, 10.1007/s11938-019-00243-4]
Imai H, 2016, GASTROINTEST ENDOSC, V84, P147, DOI 10.1016/j.gie.2015.12.024
Irani SS, 2023, CLIN GASTROENTEROL H, V21, P1141, DOI 10.1016/j.cgh.2022.12.039
Issa D, 2021, ENDOSCOPY, V53, P827, DOI 10.1055/a-1259-0349
Khan MA, 2017, GASTROINTEST ENDOSC, V85, P76, DOI 10.1016/j.gie.2016.06.032
Lambin T.B.J., 2021, Endoscopy, V53, pOP94
Lorenz JM, 2016, SEMIN INTERVENT RAD, V33, P259, DOI 10.1055/s-0036-1592330
Maire F, 2013, J VISC SURG, V150, pS27, DOI 10.1016/j.jviscsurg.2013.03.005
McInnes MDF, 2018, JAMA-J AM MED ASSOC, V319, P388, DOI 10.1001/jama.2017.19163
McKay A, 2012, SURG ENDOSC, V26, P1343, DOI 10.1007/s00464-011-2035-0
Moss AC, 2006, COCHRANE DB SYST REV, DOI 10.1002/14651858.CD004200.pub4
Mussetto A, 2018, ANN GASTROENTEROL, V31, P535, DOI 10.20524/aog.2018.0287
O'Reilly D, 2018, PANCREATOLOGY, V18, P962, DOI 10.1016/j.pan.2018.09.012
On W, 2022, GASTROINTEST ENDOSC, V95, P432, DOI 10.1016/j.gie.2021.09.040
Paleti S, 2019, GASTROINTEST ENDOSC, V89, pAB308, DOI 10.1016/j.gie.2019.03.1212
Paranandi B, 2016, ENDOSCOPY, V48, pE163, DOI 10.1055/s-0042-106578
Park JK, 2018, GASTROINTEST ENDOSC, V88, P277, DOI 10.1016/j.gie.2018.03.015
SINGH SM, 1989, SURG CLIN N AM, V69, P599
SPEER AG, 1987, LANCET, V2, P57, DOI 10.1016/S0140-6736(87)92733-4
Teoh AYB, 2020, GUT, V69, P1085, DOI 10.1136/gutjnl-2019-319996
Tyberg A, 2022, GASTROINTEST ENDOSC, V95, pAB520
Udd M, 2010, WORLD J GASTRO ENDOS, V2, P97, DOI 10.4253/wjge.v2.i3.97
van Wanrooij RLJ, 2022, ENDOSCOPY, V54, P310, DOI 10.1055/a-1738-6780
Zhang K, 2018, GASTROINTEST ENDOSC, V88, P957, DOI 10.1016/j.gie.2018.07.019
NR 36
TC 9
Z9 10
U1 0

U2 2
PU MDPI
PI BASEL
PA ST ALBAN-ANLAGE 66, CH-4052 BASEL, SWITZERLAND
EI 2072-6694
J9 CANCERS
JI Cancers
PD MAY 30
PY 2023
VL 15
IS 11
AR 2988
DI 10.3390/cancers15112988
PG 11
WC Oncology
WE Science Citation Index Expanded (SCI-EXPANDED)
SC Oncology
GA I9KI7
UT WOS:001005892600001
PM 37296955
OA Green Submitted, gold
DA 2026-05-09
ER

PT J
AU Rimbas, M
Tripodi, G
Rizzatti, G
Larghi, A
AF Rimbas, Mihai
Tripodi, Giulia
Rizzatti, Gianenrico
Larghi, Alberto
TI Endoscopic ultrasound in the management of acute cholecystitis:
Practical review
SO DIGESTIVE ENDOSCOPY
LA English
DT Review
DE cholecystectomy; endoscopic ultrasound; EUS; EUS-GBD; gallbladder
drainage
ID GUIDED GALLBLADDER DRAINAGE; LONG-TERM OUTCOMES; PERCUTANEOUS
CHOLECYSTOSTOMY; CONVERSION; THERAPY; PATIENT; STENT; TIME

AB Endoscopic ultrasound-guided gallbladder drainage (EUS-GBD) has emerged over the last years as an alternative procedure to percutaneous drainage (PT)-GBD in patients with acute cholecystitis (AC) at high surgical risk. This process has been driven by the advent of lumen-apposing metal stents (LAMS) with electrocautery-enhanced capability, which has rendered the drainage procedure easier to accomplish and safer. Studies and meta-analyses have proven the superiority of EUS-GBD over PT-GBD in high-surgical-risk patients with AC. Little evidence exists in the same setting that EUS-GBD compares equally with laparoscopic cholecystectomy (LC). Moreover, EUS-GBD might theoretically have a possible role in patients at high surgical risk with an indication to undergo cholecystectomy or with a high probability of conversion from LC to open cholecystectomy. Properly designed studies are needed to better clarify the role of EUS-GBD in these patient populations.
C1 [Rimbas, Mihai] Colentina Clin Hosp, Gastroenterol Dept, Bucharest, Romania.
[Rimbas, Mihai] Carol Davila Univ Med, Clin Internal Med, Bucharest, Romania.
[Tripodi, Giulia; Rizzatti, Gianenrico; Larghi, Alberto] Fdn Policlin Univ A Gemelli, Digest Endoscopy Unit, IRCCS, Largo A Gemelli 8, I-00168 Rome, Italy.
C3 Carol Davila University of Medicine & Pharmacy; Catholic University of the Sacred Heart; IRCCS Policlinico Gemelli
RP Larghi, A (corresponding author), Fdn Policlin Univ A Gemelli, Digest Endoscopy Unit, IRCCS, Largo A Gemelli 8, I-00168 Rome, Italy.
EM alberto.larghi@yahoo.it
RI Larghi, Alberto/AAC-7448-2022; Rizzatti, Gianenrico/B-1406-2019; Rimbas, Mihai/AAF-5521-2020
CR Andalib I, 2019, ENDOSCOPY, V51, pE370, DOI 10.1055/a-0929-3218
Anderloni A, 2018, ANN GASTROENTEROL, V31, P735, DOI 10.20524/aog.2018.0299
Anderloni A, 2017, ENDOSC INT OPEN, V5, pE1100, DOI 10.1055/s-0043-118828
Anderloni A, 2016, SURG ENDOSC, V30, P5200, DOI 10.1007/s00464-016-4894-x
Atar E, 2014, CLIN RADIOL, V69, pE247, DOI 10.1016/j.crad.2014.01.012
Baron TH, 2007, GASTROINTEST ENDOSC, V65, P735, DOI 10.1016/j.gie.2006.07.041
Baron TH, 2015, NEW ENGL J MED, V373, P357, DOI 10.1056/NEJMra1411372
Binmoeller KF, 2011, ENDOSCOPY, V43, P337, DOI 10.1055/s-0030-1256127
Chantarojanasiri Tanyaporn, 2017, Saudi J Gastroenterol, V23, P318, DOI 10.4103/sjg.SJG_115_17
Choi JH, 2014, ENDOSCOPY, V46, P656, DOI 10.1055/s-0034-1365720
Crinò SF, 2019, J GASTROINTEST LIVER, V28, P337
Dimou FM, 2017, J AM COLL SURGEONS, V224, P502, DOI 10.1016/j.jamcollsurg.2016.12.021
Goonawardena J, 2015, AM J SURG, V210, P492, DOI 10.1016/j.amjsurg.2015.04.003
Huang HJ, 2022, UPDATES SURG, V74, P55, DOI 10.1007/s13304-021-01081-9
Jang JW, 2012, GASTROENTEROLOGY, V142, P805, DOI 10.1053/j.gastro.2011.12.051
Kamata K, 2017, WORLD J GASTROENTERO, V23, P661, DOI 10.3748/wjg.v23.i4.661
Khan MA, 2017, GASTROINTEST ENDOSC, V85, P76, DOI 10.1016/j.gie.2016.06.032
KOZAREK RA, 1984, J CLIN GASTROENTEROL, V6, P37
Krishnamoorthi R, 2020, SURG ENDOSC, V34, P1904, DOI 10.1007/s00464-020-07409-7
Law R, 2016, CLIN GASTROENTEROL H, V14, P476, DOI 10.1016/j.cgh.2015.10.026
Lee TH, 2011, ENDOSCOPY, V43, P702, DOI 10.1055/s-0030-1256226
Loozen CS, 2018, BMJ-BRIT MED J, V363, DOI 10.1136/bmj.k3965

Luk SWY, 2019, ENDOSCOPY, V51, P722, DOI 10.1055/a-0929-6603
McKay A, 2012, SURG ENDOSC, V26, P1343, DOI 10.1007/s00464-011-2035-0
Mohan BP, 2020, ENDOSCOPY, V52, P96, DOI 10.1055/a-1020-3932
Mohan BP, 2019, ENDOSC ULTRASOUND, V8, P241, DOI 10.4103/eus.eus_63_18
Mori Y, 2018, J HEPATO-BIL-PAN SCI, V25, P87, DOI 10.1002/jhbp.504
Rimbas M, 2021, GASTROINTEST ENDOSC, V94, P419, DOI 10.1016/j.gie.2021.03.933
Sanjay P, 2013, HPB, V15, P511, DOI 10.1111/j.1477-2574.2012.00610.x
Saumoy M, 2021, GASTROINTEST ENDOSC, V94, P671, DOI 10.1016/j.gie.2021.05.031
Saumoy M, 2019, J CLIN GASTROENTEROL, V53, P231, DOI 10.1097/MCG.0000000000001036
Shah AA, 2019, AM J SURG, V217, P732, DOI 10.1016/j.amjsurg.2018.12.038
Tarantino I, 2016, GASTROINTEST ENDOSC, V83, P1284, DOI 10.1016/j.gie.2015.12.022
Teoh AYB, 2020, GUT, V69, P1085, DOI 10.1136/gutjnl-2019-319996
Teoh AYB, 2021, GASTROINTEST ENDOSC, V93, P577, DOI 10.1016/j.gie.2020.06.066
Teoh AYB, 2019, ENDOSC ULTRASOUND, V8, pS40, DOI 10.4103/eus.eus_49_19
Teoh AYB, 2017, ENDOSCOPY, V49, P130, DOI 10.1055/s-0042-119036
Teoh AYB, 2014, GASTROINTEST ENDOSC, V80, P1171, DOI 10.1016/j.gie.2014.03.038
Yuste RT, 2020, DIGEST ENDOSC, V32, P608, DOI 10.1111/den.13562
Tyberg A, 2018, J CLIN GASTROENTEROL, V52, P79, DOI 10.1097/MCG.0000000000000786
van der Merwe SW, 2022, ENDOSCOPY, V54, P185, DOI 10.1055/a-1717-1391
van Wanrooij RLJ, 2022, ENDOSCOPY, V54, P310, DOI 10.1055/a-1738-6780
Veitch AM, 2021, ENDOSCOPY, V53, P947, DOI 10.1055/a-1547-2282
Weischbillig-Meunier K, 2005, SURG ENDOSC, V19, P1256, DOI 10.1007/s00464-004-2248-6
Widmer J, 2014, DIGEST ENDOSC, V26, P599, DOI 10.1111/den.12163
NR 45
TC 3
Z9 4
U1 0
U2 0
PU WILEY
PI HOBOKEN
PA 111 RIVER ST, HOBOKEN 07030-5774, NJ USA
SN 0915-5635
EI 1443-1661
J9 DIGEST ENDOSC
JI Dig. Endosc.
PD NOV
PY 2023
VL 35
IS 7
BP 809
EP 818
DI 10.1111/den.14605

EA JUN 2023
PG 10
WC Gastroenterology & Hepatology; Surgery
WE Science Citation Index Expanded (SCI-EXPANDED)
SC Gastroenterology & Hepatology; Surgery
GA LF2F7
UT WOS:001013302300001
PM 37253177
OA Green Submitted, Bronze
DA 2026-05-09
ER

PT J
AU Sobani, ZA
Ling, CA
Rustagi, T
AF Sobani, Zain A.
Ling, Christina
Rustagi, Tarun
TI Endoscopic Ultrasound-Guided Gallbladder Drainage
SO DIGESTIVE DISEASES AND SCIENCES
LA English
DT Review
DE Acute cholecystitis; Transmural; High-risk patients; Endoscopic
gallbladder drainage; Lumen-apposing metal stents; LAMS; Endoscopic
ultrasound (EUS)
ID APPOSING METAL STENT; CONFOCAL LASER ENDOMICROSCOPY; GALL-BLADDER
DRAINAGE; LONG-TERM OUTCOMES; ACUTE CHOLECYSTITIS
AB The mainstay of management of acute cholecystitis is surgical. Despite the advances in anesthesia and laparoscopic surgery, there is a significant pool of patients that are not candidates for surgery given their significant comorbidities and limited functional reserve. Historically percutaneous gallbladder drainage has been utilized to temporize these patients. Recently, endoscopic approaches are being explored with transpapillary and transmural drainage. In this article, we discuss the background, current status, technical challenges, adverse events, and outcomes of endoscopic ultrasound-guided gallbladder drainage for management of acute cholecystitis.
C1 [Sobani, Zain A.; Ling, Christina; Rustagi, Tarun] Univ New Mexico, Dept Internal Med, Div Gastroenterol & Hepatol, MSC10 5550,1, Albuquerque, NM 87131 USA.
C3 University of New Mexico
RP Rustagi, T (corresponding author), Univ New Mexico, Dept Internal Med, Div Gastroenterol & Hepatol, MSC10 5550,1, Albuquerque, NM 87131 USA.
EM tarunrustagi06@gmail.com
CR Ahmed O, 2018, SAUDI J GASTROENTERO, V24, P183, DOI 10.4103/sjg.SJG_506_17
Anderloni A, 2017, ENDOSC INT OPEN, V5, pE1100, DOI 10.1055/s-0043-118828

Bundy J, 2018, CARDIOVASC INTER RAD, V41, P928, DOI 10.1007/s00270-018-1884-5
Chan EOT, 2020, ENDOSCOPY
Chan JHY, 2018, CLIN ENDOSC, V51, P150, DOI 10.5946/ce.2017.125
Chan SM, 2017, GASTROINTEST ENDOSC, V85, P1225, DOI 10.1016/j.gie.2016.10.014
Chantarojanasiri Tanyaporn, 2017, Saudi J Gastroenterol, V23, P318, DOI 10.4103/sjg.SJG_115_17
Cho SH, 2020, GASTROINTEST ENDOSC, V91, P543, DOI 10.1016/j.gie.2019.09.042
Choi JH, 2014, ENDOSCOPY, V46, P656, DOI 10.1055/s-0034-1365720
de la Serna-Higuera C, 2013, GASTROINTEST ENDOSC, V77, P303, DOI 10.1016/j.gie.2012.09.021
Dollhopf M, 2017, GASTROINTEST ENDOSC, V86, P636, DOI 10.1016/j.gie.2017.02.027
Fugazza A, 2016, BIOMED RES INT, V2016, DOI 10.1155/2016/4638683
Gulati R, 2021, J HEPATO-BIL-PAN SCI, V28, pe8, DOI 10.1002/jhbp.752
Han D, 2018, J CLIN GASTROENTEROL, V52, P835, DOI 10.1097/MCG.0000000000000934
Higa JT, 2019, GASTROINTEST ENDOSC, V90, P483, DOI 10.1016/j.gie.2019.04.238
Imai H, 2016, GASTROINTEST ENDOSC, V84, P147, DOI 10.1016/j.gie.2015.12.024
Irani S, 2017, CLIN GASTROENTEROL H, V15, P738, DOI 10.1016/j.cgh.2016.12.021
Irani S, 2015, GASTROINTEST ENDOSC, V82, P1110, DOI 10.1016/j.gie.2015.05.045
Itoi T, 2012, GASTROINTEST ENDOSC, V75, P870, DOI 10.1016/j.gie.2011.10.020
Itoi T, 2010, GASTROINTEST ENDOSC, V71, P1038, DOI 10.1016/j.gie.2010.01.026
Jain D, 2018, CLIN ENDOSC, V51, P450, DOI 10.5946/ce.2018.024
Kalva NR, 2018, CAN J GASTROENTEROL, V2018, DOI 10.1155/2018/7070961
Kamata K, 2017, WORLD J GASTROENTERO, V23, P661, DOI 10.3748/wjg.v23.i4.661
Khan MA, 2017, GASTROINTEST ENDOSC, V85, P76, DOI 10.1016/j.gie.2016.06.032
Krishnamoorthi R, 2020, SURG ENDOSC, V34, P1904, DOI 10.1007/s00464-020-07409-7
Kwan V, 2007, GASTROINTEST ENDOSC, V66, P582, DOI 10.1016/j.gie.2007.02.065
Law Ryan, 2018, Gastrointest Endosc Clin N Am, V28, P187, DOI [10.1016/j.giec.2017.11.006, 10.1016/j.giec.2017.11.006]
Law R, 2016, CLIN GASTROENTEROL H, V14, P476, DOI 10.1016/j.cgh.2015.10.026
Ling C, 2019, GASTROINTEST ENDOSC, V89, pAB292, DOI 10.1016/j.gie.2019.03.1176
Lisotti A, 2019, ENDOSCOPY
Manta R, 2018, EUR J GASTROEN HEPAT, V30, P695, DOI 10.1097/MEG.0000000000001112
Minaga K, 2019, DIGEST ENDOSC, V31, P180, DOI 10.1111/den.13242
Mohan BP, 2020, ENDOSCOPY, V52, P96, DOI 10.1055/a-1020-3932
Nakai Y, 2014, DIGEST ENDOSC, V26, P86, DOI 10.1111/den.12152
Oh D, 2019, GASTROINTEST ENDOSC, V89, P289, DOI 10.1016/j.gie.2018.08.052
Paleti S, 2019, GASTROINTEST ENDOSC, V89, pAB308, DOI 10.1016/j.gie.2019.03.1212
Rustagi T, 2016, GASTROINTEST ENDOSC, V84, P528, DOI 10.1016/j.gie.2016.03.1505
Sánchez-Luna SA, 2020, ACG CASE REP J, V7, DOI 10.14309/crj.0000000000000368
Saumoy M, 2019, J CLIN GASTROENTEROL, V53, P231, DOI 10.1097/MCG.0000000000001036
Sharma V, 2015, WORLD J GASTRO ENDOS, V7, P628, DOI 10.4253/wjge.v7.i6.628
Siddiqui A, 2019, SURG ENDOSC, V33, P1260, DOI 10.1007/s00464-018-6406-7
Sobani ZA, 2021, DIGEST DIS SCI, V66, P1425, DOI 10.1007/s10620-020-06422-z
Teoh AYB, 2020, GUT, V69, P1085, DOI 10.1136/gutjnl-2019-319996

Teoh AYB, 2014, ENDOSCOPY, V46, pE13, DOI 10.1055/s-0033-1359139
Teoh AYB, 2014, GASTROINTEST ENDOSC, V80, P1171, DOI 10.1016/j.gie.2014.03.038
Teoh AYB, 2020, GUT
Yuste RT, 2019, ANN GASTROENTEROL, V32, P514, DOI 10.20524/aog.2019.0395
Walter D, 2016, GUT, V65, P6, DOI 10.1136/gutjnl-2015-309925
Zhang K, 2018, GASTROINTEST ENDOSC, V88, P957, DOI 10.1016/j.gie.2018.07.019
NR 49
TC 16
Z9 18
U1 0
U2 1
PU SPRINGER
PI DORDRECHT
PA VAN GODEWIJCKSTRAAT 30, 3311 GZ DORDRECHT, NETHERLANDS
SN 0163-2116
EI 1573-2568
J9 DIGEST DIS SCI
JI Dig. Dis. Sci.
PD JUL
PY 2021
VL 66
IS 7
BP 2154
EP 2161
DI 10.1007/s10620-020-06520-y
EA AUG 2020
PG 8
WC Gastroenterology & Hepatology
WE Science Citation Index Expanded (SCI-EXPANDED)
SC Gastroenterology & Hepatology
GA SZ9GA
UT WOS:000555778300002
PM 32749635
DA 2026-05-09
ER

PT J
AU Sportes, A
Airinei, G
Kamel, R
Raynaud, JJ
Benamouzig, R

AF Sportes, Adrien
Airinei, Gheoghe
Kamel, Ralph
Raynaud, Jean Jacques
Benamouzig, Robert
TI Endoscopic ultrasound-guided choledochoduodenostomy with a
lumen-apposing metal stent through an uncovered metal duodenal stent
SO ENDOSCOPY INTERNATIONAL OPEN
LA English
DT Article
ID HEPATICOGASTROSTOMY; DRAINAGE; ERCP
AB Background and study aims Endoscopic retrograde cholangiopancreatography (ERCP) in patients with a preexisting duodenal stent is particularly challenging and has a low success rate. Endoscopic ultrasound (EUS)-guided biliary drainage (EUS-BD) has been increasingly used as an alternative to percutaneous transhepatic biliary drainage after failed ERCP. EUS-guided choledochoduodenostomy (EUS-CD) and EUS-guided hepaticogastrostomy (EUS-HGS) have been reported to have similar efficacity. Recently, a novel dedicated fully-covered lumen-apposing metal stent (LAMS) has been developed for EUS-CD (Hot AXIOS; Boston Scientific, Massachusetts, United States). It seems that this new device decreases the morbidity of EUS-CD. We present a case in which EUS-CD with LAMS through an uncovered metal duodenal stent was used successfully.
C1 [Sportes, Adrien; Airinei, Gheoghe; Kamel, Ralph; Raynaud, Jean Jacques; Benamouzig, Robert] Hop Avicenne, Dept Gastroenterol, Bobigny, France.
C3 Universite Paris 13; Assistance Publique Hopitaux Paris (APHP); Hopital
Universitaire Avicenne - APHP
RP Sportes, A (corresponding author), Hop Avicenne, AP HP, Gastroenterol Unit, 125 Rue Stalingrad, F-93000 Bobigny, France.
EM adrien.sportes@free.fr
RI BENAMOUZIG, ROBERT/W-8067-2019
OI BENAMOUZIG, ROBERT/0000-0003-1952-6830
CR Khashab MA, 2014, ENDOSCOPY, V46, P251, DOI 10.1055/s-0033-1359214
Kunda R, 2016, SURG ENDOSC, V30, P5002, DOI 10.1007/s00464-016-4845-6
Sharaiha RZ, 2017, GASTROINTEST ENDOSC, V85, P904, DOI 10.1016/j.gie.2016.12.023
Sportes A, 2017, THER ADV GASTROENTER, V10, P483, DOI 10.1177/1756283X17702096
Uemura RS, 2018, J CLIN GASTROENTEROL, V52, P123, DOI 10.1097/MCG.0000000000000948
NR 5
TC 7
Z9 8
U1 0
U2 1
PU GEORG THIEME VERLAG KG
PI STUTTGART
PA RUDIGERSTR 14, D-70469 STUTTGART, GERMANY
SN 2364-3722

EI 2196-9736
J9 ENDOSC INT OPEN
JI Endosc. Int. Open
PD DEC
PY 2018
VL 6
IS 12
BP E1395
EP E1397
DI 10.1055/a-0735-9254
PG 3
WC Gastroenterology & Hepatology; Surgery
WE Emerging Sources Citation Index (ESCI)
SC Gastroenterology & Hepatology; Surgery
GA HB5EX
UT WOS:000451082700001
PM 30505931
OA Green Submitted, gold
DA 2026-05-09
ER

PT J
AU Canakis, A
Baron, TH
AF Canakis, Andrew
Baron, Todd H. H.
TI Therapeutic Endoscopic Ultrasound: Current Indications and Future
Perspectives
SO GE PORTUGUESE JOURNAL OF GASTROENTEROLOGY
LA English
DT Review
DE Endoscopic ultrasound; Biliary drainage; Hepaticogastrostomy;
Choledochoduodenostomy; Pancreatic ductal drainage; Necrotic fluid
collections; Pancreatic cyst ablation; Lumen apposing metal stents
ID GUIDED BILIARY DRAINAGE; TRANSHEPATIC GALLBLADDER DRAINAGE; PANCREATIC
CYSTIC NEOPLASMS; APPOSING METAL STENTS; GASTRIC VARICES; INTERNATIONAL
MULTICENTER; RETROGRADE CHOLANGIOPANCREATOGRAPHY; CYANOACRYLATE
INJECTION; ACUTE CHOLECYSTITIS; SIMILAR EFFICACIES
AB The transcendence of endoscopic ultrasound (EUS) from diagnostic to therapeutic tool has revolutionized management options in the field of gastroenterology. Through EUS-guided methods, pancreaticobiliary obstruction can now be utilized as an alternative to surgical and percutaneous approaches. This modality also allows for gallbladder drainage in patients who are not ideal operative candidates. By utilizing its unique imaging capabilities, EUS also allows for drainage access

points in cases of gastric outlet obstruction as well as windows to ablate pancreatic cystic lesions. As technical progress continues to evolve, interventional gastroenterology continues to push the envelope of minimally invasive therapeutic procedures in a multidisciplinary setting. In this comprehensive review, we set out to describe current indications and innovations through EUS.

C1 [Canakis, Andrew] Univ Maryland, Div Gastroenterol & Hepatol, Sch Med, Baltimore, MD USA.
[Baron, Todd H. H.] Univ North Carolina, Div Gastroenterol & Hepatol, Chapel Hill, NC 27599 USA.
C3 University System of Maryland; University of Maryland Baltimore;
University of North Carolina; University of North Carolina Chapel Hill
RP Baron, TH (corresponding author), Univ North Carolina, Div Gastroenterol & Hepatol, Chapel Hill, NC 27599 USA.
EM todd_baron@med.unc.edu
OI Canakis, Andrew/0000-0002-6646-6693
CR Ali UA, 2015, COCHRANE DB SYST REV, DOI 10.1002/14651858.CD007884.pub3
Amano M, 2017, J GASTROEN HEPATOL, V32, P716, DOI 10.1111/jgh.13489
Anderloni A, 2016, SURG ENDOSC, V30, P5200, DOI 10.1007/s00464-016-4894-x
Andriulli A, 2007, AM J GASTROENTEROL, V102, P1781, DOI 10.1111/j.1572-0241.2007.01279.x
Artifon ELA, 2015, GASTROINTEST ENDOSC, V81, P950, DOI 10.1016/j.gie.2014.09.047
Arvanitakis M, 2018, ENDOSCOPY, V50, P524, DOI 10.1055/a-0588-5365
Attasaranya S, 2012, GASTROENT RES PRACT, V2012, DOI 10.1155/2012/680753
Attila T, 2019, EUR J GASTROEN HEPAT, V31, P1, DOI 10.1097/MEG.0000000000001297
Bang JY, 2018, GASTROINTEST ENDOSC, V88, P9, DOI 10.1016/j.gie.2018.03.012
Baniya R, 2017, CLIN EXP GASTROENTER, V10, P67, DOI 10.2147/CEG.S132004
Baron TH, 2007, GASTROINTEST ENDOSC, V65, P735, DOI 10.1016/j.gie.2006.07.041
Baron TH, 2021, NEW ENGL J MED, V385, P1433, DOI 10.1056/NEJMe2110313
Baron TH, 2020, GASTROENTEROLOGY, V158, P67, DOI 10.1053/j.gastro.2019.07.064
Barthet M, 2019, ENDOSCOPY, V51, P836, DOI 10.1055/a-0824-7067
Basiliya K, 2021, ENDOSCOPY, V53, P266, DOI 10.1055/a-1200-0199
Bazarbashi AN, 2020, CLIN TRANSL GASTROEN, V11
Bazarbashi AN, 2020, ENDOSC INT OPEN, V8, pE221, DOI 10.1055/a-1027-6708
Bhat YM, 2016, GASTROINTEST ENDOSC, V83, P1164, DOI 10.1016/j.gie.2015.09.040
Bispo M, 2020, GE PORT J GASTROENT, V27, P410, DOI 10.1159/000507895
Boxhoorn L, 2021, NEW ENGL J MED, V385, P1372, DOI 10.1056/NEJMoa2100826
Bronswijk Michiel, 2020, VideoGIE, V5, P442, DOI [10.1016/j.vgie.2020.06.012, 10.1016/j.vgie.2020.06.012]
Canakis A, 2022, J HEPATO-BIL-PAN SCI, V29, P941, DOI 10.1002/jhbp.1055
Canakis A, 2022, DIGEST DIS SCI, V67, P1573, DOI 10.1007/s10620-021-07084-1
Canakis A, 2020, BMJ OPEN GASTROENTER, V7, DOI 10.1136/bmjgast-2020-000428
Canakis A, 2020, GASTROINTEST ENDOSC, V91, P520, DOI 10.1016/j.gie.2019.09.037
Carbajo AY, 2020, J CLIN GASTROENTEROL, V54, P1, DOI 10.1097/MCG.0000000000001262
Chandrasekhara V, 2021, CLIN GASTROENTEROL H, V19, P2192, DOI 10.1016/j.cgh.2021.05.005
Chantarojanasiri T, 2018, ENDOSC INT OPEN, V6, pE1398, DOI 10.1055/a-0751-2698
Chen YI, 2018, GASTROINTEST ENDOSC, V87, P1215, DOI 10.1016/j.gie.2017.07.030
Chen YI, 2018, ENDOSC INT OPEN, V6, pE363, DOI 10.1055/s-0043-123468
Chen YI, 2017, SURG ENDOSC, V31, P2946, DOI 10.1007/s00464-016-5311-1

Chen YI, 2017, GASTROINTEST ENDOSC, V85, P170, DOI 10.1016/j.gie.2016.07.031
Cho DH, 2017, GASTROINTEST ENDOSC, V85, P1067, DOI 10.1016/j.gie.2016.09.010
Dalal A, 2020, DIGEST ENDOSC, V32, P409, DOI 10.1111/den.13504
de Mestral C, 2013, J TRAUMA ACUTE CARE, V74, P26, DOI 10.1097/TA.0b013e3182788e4d
DeWitt JM, 2021, CLIN GASTROENTEROL H, V19, P24, DOI 10.1016/j.cgh.2020.09.029
DeWitt JM, 2014, ENDOSCOPY, V46, P457, DOI 10.1055/s-0034-1365496
Dumonceau JM, 2018, ENDOSCOPY, V50, P910, DOI 10.1055/a-0659-9864
Elta GH, 2018, AM J GASTROENTEROL, V113, P464, DOI 10.1038/ajg.2018.14
Frost JW, 2018, ENDOSC INT OPEN, V6, pE664, DOI 10.1055/a-0599-0440
Fujii-Lau LL, 2015, J HEPATO-BIL-PAN SCI, V22, P51, DOI 10.1002/jhbp.187
Garg R, 2022, ENDOSC ULTRASOUND, V11, P170, DOI 10.4103/EUS-D-21-00044
Ge PS, 2020, PANCREAS, V49, P236, DOI 10.1097/MPA.0000000000001476
Ge Phillip S, 2019, VideoGIE, V4, P154, DOI 10.1016/j.vgie.2018.08.004
Ge PS, 2019, SURG ENDOSC, V33, P3404, DOI 10.1007/s00464-018-06636-3
Giovannini M, 2001, ENDOSCOPY, V33, P898, DOI 10.1055/s-2001-17324
Gómez V, 2016, GASTROINTEST ENDOSC, V83, P914, DOI 10.1016/j.gie.2015.08.069
Gornals JB, 2016, ENDOSCOPY, V48, pE276, DOI 10.1055/s-0042-113186
Guo JT, 2016, GASTROENT RES PRACT, V2016, DOI 10.1155/2016/9469472
Halttunen J, 2014, SCAND J GASTROENTERO, V49, P752, DOI 10.3109/00365521.2014.894120
Hathorn KE, 2022, GASTROINTEST ENDOSC, V95, P443, DOI 10.1016/j.gie.2021.10.013
Hayat U, 2020, ENDOSC INT OPEN, V8, pE196, DOI 10.1055/a-1005-6573
Hemerly MC, 2023, SURG ENDOSC, V37, P2421, DOI 10.1007/s00464-022-09712-x
Holt BA, 2016, GASTROINTEST ENDOSC, V83, P160, DOI 10.1016/j.gie.2015.06.019
Irani Shayan, 2020, VideoGIE, V5, P48, DOI [10.1016/j.vgie.2019.10.007, 10.1016/j.vgie.2019.10.007]
Irani S, 2017, CLIN GASTROENTEROL H, V15, P738, DOI 10.1016/j.cgh.2016.12.021
James TW, 2019, ENDOSC INT OPEN, V7, pE1099, DOI 10.1055/a-0965-6662
James TW, 2018, ENDOSC INT OPEN, V6, pE735, DOI 10.1055/a-0607-2484
Jang JW, 2012, GASTROENTEROLOGY, V142, P805, DOI 10.1053/j.gastro.2011.12.051
Kakked G, 2020, ENDOSC ULTRASOUND, V9, P298, DOI 10.4103/eus.eus_10_20
Kawakubo K, 2014, J HEPATO-BIL-PAN SCI, V21, P328, DOI 10.1002/jhbp.27
Keane MG, 2020, ENDOSC INT OPEN, V08, pE1455, DOI 10.1055/a-1231-5011
Khan Z, 2021, ENDOSC ULTRASOUND, V10, P98, DOI 10.4103/eus.eus_67_20
Khashab MA, 2016, ENDOSC INT OPEN, V4, pE175, DOI 10.1055/s-0041-109083
Kim TH, 2012, WORLD J GASTROENTERO, V18, P2526, DOI 10.3748/wjg.v18.i20.2526
Krafft MR, 2019, DIGEST DIS SCI, V64, P1770, DOI 10.1007/s10620-019-05495-9
Law Ryan, 2018, Gastrointest Endosc Clin N Am, V28, P187, DOI [10.1016/j.giec.2017.11.006, 10.1016/j.giec.2017.11.006]
Lee TH, 2016, CLIN GASTROENTEROL H, V14, P1011, DOI 10.1016/j.cgh.2015.12.032
Lee YS, 2021, GUT LIVER, V15, P459, DOI 10.5009/gnl19304
Lee YT, 2000, GASTROINTEST ENDOSC, V52, P168, DOI 10.1067/mge.2000.107911
Li JS, 2022, FRONT SURG, V9, DOI 10.3389/fsurg.2022.811005
Lo GH, 2020, ENDOSCOPY, V52, P548, DOI 10.1055/a-1127-3170

Luk SWY, 2019, ENDOSCOPY, V51, P722, DOI 10.1055/a-0929-6603
Mao KJ, 2022, SURG LAPARO ENDO PER, V32, P124, DOI 10.1097/SLE.0000000000000992
McCarty TR, 2020, ENDOSC ULTRASOUND, V9, P6, DOI 10.4103/eus.eus_37_19
Miller CS, 2019, ENDOSC INT OPEN, V7, pE1563, DOI 10.1055/a-0998-8129
Minaga K, 2019, DIGEST ENDOSC, V31, P575, DOI 10.1111/den.13406
Mori Y, 2018, J HEPATO-BIL-PAN SCI, V25, P87, DOI 10.1002/jhbp.504
Moyer MT, 2017, GASTROENTEROLOGY, V153, P1295, DOI 10.1053/j.gastro.2017.08.009
Moyer MT., 2021, CHARM 2 CHEMOTHERAPY
Mukai S, 2017, J HEPATO-BIL-PAN SCI, V24, P537, DOI 10.1002/jhbp.496
Muthusamy VR, 2016, GASTROINTEST ENDOSC, V83, P481, DOI 10.1016/j.gie.2015.11.027
Nakai Y, 2020, J GASTROEN HEPATOL, V35, P967, DOI 10.1111/jgh.14955
Oblizajek N, 2020, GASTROINTEST ENDOSC, V91, P1303, DOI 10.1016/j.gie.2020.01.017
Ogura T, 2021, DEN OPEN, V1, DOI 10.1002/deo2.8
Ogura T, 2021, GUT LIVER, V15, P196
Ogura T, 2016, ENDOSCOPY, V48, P156, DOI 10.1055/s-0034-1392859
Oh D, 2017, THER ADV GASTROENTER, V10, P42, DOI 10.1177/1756283X16671671
Oh D, 2016, GASTROINTEST ENDOSC, V83, P366, DOI 10.1016/j.gie.2015.07.015
Oh HC, 2008, GASTROINTEST ENDOSC, V67, P636, DOI 10.1016/j.gie.2007.09.038
Oleinikov K, 2019, J CLIN ENDOCR METAB, V104, P2637, DOI 10.1210/jc.2019-00282
Pai M, 2015, WORLD J GASTRO SURG, V7, P52, DOI 10.4240/wjgs.v7.i4.52
Paik WH, 2018, AM J GASTROENTEROL, V113, P987, DOI 10.1038/s41395-018-0122-8
Park DH, 2015, J GASTROEN HEPATOL, V30, P1461, DOI 10.1111/jgh.13027
Park JK, 2018, GASTROINTEST ENDOSC, V88, P277, DOI 10.1016/j.gie.2018.03.015
Park JK, 2016, PANCREAS, V45, P889, DOI 10.1097/MPA.0000000000000567
Parsa N, 2020, ENDOSCOPY, V52, P211, DOI 10.1055/a-1096-3299
Peng CY, 2013, BMC GASTROENTEROL, V13, DOI 10.1186/1471-230X-13-147
Perez-Miranda M, 2017, J CLIN GASTROENTEROL, V51, P896, DOI 10.1097/MCG.0000000000000887
Poincloux L, 2015, ENDOSCOPY, V47, P794, DOI [10.1055/s-0035-1545399, 10.1055/s-0034-1391988]
Prachayakul V, 2013, WORLD J GASTROENTERO, V19, P4758, DOI 10.3748/wjg.v19.i29.4758
Ramai D, 2023, GASTROINTEST ENDOSC, V97, P415, DOI 10.1016/j.gie.2022.11.003
Rana SS, 2021, J GASTROINTEST SURG, V25, P2328, DOI 10.1007/s11605-021-04945-y
Robles-Medranda C, 2020, ENDOSCOPY, V52, P268, DOI 10.1055/a-1123-9054
Robles-Medranda C, 2019, DIGEST ENDOSC, V31, P283, DOI 10.1111/den.13305
Romero-Castro R, 2013, GASTROINTEST ENDOSC, V78, P711, DOI 10.1016/j.gie.2013.05.009
Saad WEA, 2010, J VASC INTERV RADIOL, V21, P789, DOI 10.1016/j.jvir.2010.01.012
Siddiqui AA, 2017, GASTROINTEST ENDOSC, V85, P758, DOI 10.1016/j.gie.2016.08.014
Sportes A, 2017, THER ADV GASTROENTER, V10, P483, DOI 10.1177/1756283X17702096
Takeno A, 2013, ANN SURG ONCOL, V20, P3527, DOI 10.1245/s10434-013-3033-3
Teoh AYB, 2020, GUT, V69, P1085, DOI 10.1136/gutjnl-2019-319996
Teoh AYB, 2018, GUT, V67, P1209, DOI 10.1136/gutjnl-2017-314341
Teoh AYB, 2021, GASTROINTEST ENDOSC, V93, P577, DOI 10.1016/j.gie.2020.06.066
Teoh AYB, 2019, ENDOSC INT OPEN, V7, pE1064, DOI 10.1055/a-0959-5870

Tessier G, 2007, GASTROINTEST ENDOSC, V65, P233, DOI 10.1016/j.gie.2006.06.029
Trikudanathan G, 2019, GASTROENTEROLOGY, V156, P1994, DOI 10.1053/j.gastro.2019.01.269
Trikudanathan G, 2018, AM J GASTROENTEROL, V113, P1550, DOI 10.1038/s41395-018-0232-3
Tyberg A, 2022, ENDOSC ULTRASOUND, V11, P38, DOI 10.4103/EUS-D-21-00006
Tyberg A, 2020, ENDOSC ULTRASOUND, V9, P392, DOI 10.4103/eus.eus_42_20
Tyberg A, 2020, ENDOSC ULTRASOUND, V9, P175, DOI 10.4103/eus.eus_3_20
Tyberg A, 2018, J CLIN GASTROENTEROL, V52, P79, DOI 10.1097/MCG.0000000000000786
Uemura RS, 2018, J CLIN GASTROENTEROL, V52, P123, DOI 10.1097/MCG.0000000000000948
van der Merwe SW, 2022, ENDOSCOPY, V54, P185, DOI 10.1055/a-1717-1391
van Santvoort HC, 2010, NEW ENGL J MED, V362, P1491, DOI 10.1056/NEJMoa0908821
van Wanrooij RLJ, 2022, ENDOSCOPY, V54, P1023, DOI 10.1055/a-1782-7568
van Wanrooij RLJ, 2022, ENDOSCOPY, V54, P310, DOI 10.1055/a-1738-6780
Wang XM, 2019, J INT MED RES, V47, P1802, DOI 10.1177/0300060519830207
Wani ZA, 2015, J RES MED SCI, V20, P1200, DOI 10.4103/1735-1995.172990
Will U, 2015, WORLD J GASTROENTERO, V21, P13140, DOI 10.3748/wjg.v21.i46.13140
NR 129
TC 11
Z9 12
U1 0
U2 1
PU KARGER
PI BASEL
PA ALLSCHWILERSTRASSE 10, CH-4009 BASEL, SWITZERLAND
SN 2341-4545
EI 2387-1954
J9 GE PORT J GASTROENT
JI GE Port. J. Gastroenterol.
PD SEP
PY 2023
VL 30
IS SUPPL 1
SU 1
BP 4
EP 18
DI 10.1159/000529089
EA FEB 2023
PG 15
WC Gastroenterology & Hepatology
WE Emerging Sources Citation Index (ESCI)
SC Gastroenterology & Hepatology
GA R6TT3
UT WOS:000935275400001

PM 37818395
OA Green Submitted, gold
DA 2026-05-09
ER

PT J
AU Oh, SY
Irani, S
Kozarek, RA
AF Oh, Stephen Y.
Irani, Shayan
Kozarek, Richard A.
TI What are the current and potential future roles for endoscopic ultrasound in the treatment of pancreatic cancer?
SO WORLD JOURNAL OF GASTROINTESTINAL ENDOSCOPY
LA English
DT Review
DE Endoscopic ultrasound; Pancreatic cancer; Palliation; Endoscopic ultrasound-guided celiac plexus neurolysis and block; Endoscopic ultrasound-guided biliary drainage; Endoscopic ultrasound-guided gastrojejunal anastomosis; Endoscopic ultrasound-guided antitumor therapy; Endoscopic ultrasound-guided fiducial placement; Endoscopic ultrasound-guided ablation
ID CELIAC PLEXUS NEUROLYSIS; GUIDED BILIARY DRAINAGE; MAGNETIC COMPRESSION ANASTOMOSIS; GROUP DOCUMENT EVALUATION; EXPANDABLE METAL STENT; SPINAL-CORD INFARCTION; FINE-NEEDLE INJECTION; PHOTODYNAMIC THERAPY; RADIOFREQUENCY ABLATION; STEREOTACTIC RADIOSURGERY
AB Pancreatic adenocarcinoma is the fourth leading cause of cancer-related death in the United States. Due to the aggressive tumor biology and late manifestations of the disease, long-term survival is extremely uncommon and the current 5-year survival rate is 7%. Over the last two decades, endoscopic ultrasound (EUS) has evolved from a diagnostic modality to a minimally invasive therapeutic alternative to radiologic procedures and surgery for pancreatic diseases. EUS-guided celiac plexus intervention is a useful adjunct to conventional analgesia for patients with pancreatic cancer. EUS-guided biliary drainage has emerged as a viable option in patients who have failed endoscopic retrograde cholangiopancreatography. Recently, the use of lumen-apposing metal stent to create gastrojejunal anastomosis under EUS and fluoroscopic guidance in patients with malignant gastric outlet obstruction has been reported. On the other hand, anti-tumor therapies delivered by EUS, such as the injection of anti-tumor agents, brachytherapy and ablations are still in the experimental stage without clear survival benefit. In this article, we provide updates on well-established EUS-guided interventions as well as novel techniques relevant to pancreatic cancer.
C1 [Oh, Stephen Y.; Irani, Shayan; Kozarek, Richard A.] Virginia Mason Med Ctr, Inst Digest Dis, 1100 9th Ave,MS C3-GAS, Seattle, WA 98101 USA.
C3 Virginia Mason Medical Center
RP Kozarek, RA (corresponding author), Virginia Mason Med Ctr, Inst Digest Dis, 1100 9th Ave,MS C3-GAS, Seattle, WA

98101 USA.
EM richard.kozarek@virginiamason.org
RI Kozarek, Richard/AAF-2695-2020
CR [Anonymous], HEAD NECK CANC CURRE
[Anonymous], 2013, J Interv Gastroenterol, DOI [DOI 10.7178/JIG.102, 10.7178/jig.102]
Arcidiacono PG, 2011, COCHRANE DB SYST REV, DOI 10.1002/14651858.CD007519.pub2
Arcidiacono PG, 2012, GASTROINTEST ENDOSC, V76, P1142, DOI 10.1016/j.gie.2012.08.006
Artifon ELA, 2012, J CLIN GASTROENTEROL, V46, P768, DOI 10.1097/MCG.0b013e31825f264c
Artifon ELA, 2011, J PANCREAS, V12, P610
Ascunce G, 2011, GASTROINTEST ENDOSC, V73, P267, DOI 10.1016/j.gie.2010.10.029
Bown SG, 2002, GUT, V50, P549, DOI 10.1136/gut.50.4.549
Carrara S, 2008, ENDOSCOPY, V40, P321, DOI 10.1055/s-2007-995595
Chan HH, 2004, GASTROINTEST ENDOSC, V59, P95, DOI 10.1016/S0016-5107(03)02361-7
Chang KJ, 2000, CANCER-AM CANCER SOC, V88, P1325, DOI 10.1002/(SICI)1097-0142(20000315)88:6<1325::AID-CNCR8>3.0.CO;2-T
CHATLANI PT, 1992, BJS-BRIT J SURG, V79, P786, DOI 10.1002/bjs.1800790826
Chopita N, 2005, ENDOSCOPY, V37, P313, DOI 10.1055/s-2005-861358
COPE C, 1995, J VASC INTERV RADIOL, V6, P539, DOI 10.1016/S1051-0443(95)71131-9
COPE C, 1995, J VASC INTERV RADIOL, V6, P546, DOI 10.1016/S1051-0443(95)71132-0
Dhir V, 2012, GASTROINTEST ENDOSC, V75, P354, DOI 10.1016/j.gie.2011.07.075
Didolkar MS, 2010, J GASTROINTEST SURG, V14, P1547, DOI 10.1007/s11605-010-1323-7
Doi S, 2013, ENDOSCOPY, V45, P362, DOI 10.1055/s-0032-1326225
Fabbri C, 2011, ENDOSCOPY, V43, P438, DOI 10.1055/s-0030-1256097
Fan BG, 2007, PANCREAS, V34, P385, DOI 10.1097/mpa.0b013e3180439c50
Fritscher-Ravens A, 2003, GASTROINTEST ENDOSC, V58, P585
Fritscher-Ravens A, 2002, GASTROINTEST ENDOSC, V56, P737, DOI 10.1067/mge.2002.129084
Fujii L, 2012, ENDOSCOPY, V44, pE265, DOI 10.1055/s-0032-1309708
Gavini H, 2015, J CLIN GASTROENTEROL, V49, P185, DOI 10.1097/MCG.0000000000000276
Gimeno-García AZ, 2012, ENDOSCOPY, V44, pE267, DOI 10.1055/s-0032-1309709
Gleeson FC, 2007, ENDOSCOPY, V39, P620, DOI 10.1055/s-2007-966337
Goldberg SN, 1999, GASTROINTEST ENDOSC, V50, P392, DOI 10.1053/ge.1999.v50.98847
Gunaratnam NT, 2001, GASTROINTEST ENDOSC, V54, P316, DOI 10.1067/mge.2001.117515
Hanna N, 2012, CANCER GENE THER, V19, P374, DOI 10.1038/cgt.2012.10
Hara K, 2011, AM J GASTROENTEROL, V106, P1239, DOI 10.1038/ajg.2011.84
Hecht JR, 2012, GASTROINTEST ENDOSC, V75, P332, DOI 10.1016/j.gie.2011.10.007
Hecht JR, 2003, CLIN CANCER RES, V9, P555
Herman JM, 2013, J CLIN ONCOL, V31, P886, DOI 10.1200/JCO.2012.44.7516
Hirooka Y, 2009, PANCREAS, V38, pE69, DOI 10.1097/MPA.0b013e318197a9e3
Huggett MT, 2014, BRIT J CANCER, V110, P1698, DOI 10.1038/bjc.2014.95
Hwang RF, 2008, CANCER RES, V68, P918, DOI 10.1158/0008-5472.CAN-07-5714
Ikeuchi N, 2015, GASTROINTEST ENDOSC, V82, P166, DOI 10.1016/j.gie.2015.01.010
Irisawa A, 2007, PANCREAS, V35, P189, DOI 10.1097/01.mpa.0000250141.25639.e9

Itoi T, 2005, ENDOSCOPY, V37, P1248, DOI 10.1055/s-2005-870269
Itoi T, 2015, J HEPATO-BIL-PAN SCI, V22, P3, DOI 10.1002/jhbp.148
Itoi T, 2013, GASTROINTEST ENDOSC, V78, P934, DOI 10.1016/j.gie.2013.09.025
Itoi T, 2013, DIGEST ENDOSC, V25, P137, DOI 10.1111/den.12084
Itoi T, 2009, GASTROINTEST ENDOSC, V69, pS8, DOI 10.1016/j.gie.2008.11.003
Iwashita T, 2012, ENDOSCOPY, V44, P60, DOI 10.1055/s-0030-1256871
Jang HY, 2013, CLIN ENDOSC, V46, P306, DOI 10.5946/ce.2013.46.3.306
Jang JW, 2012, GASTROENTEROLOGY, V142, P805, DOI 10.1053/j.gastro.2011.12.051
Jemal A, 2010, CA-CANCER J CLIN, V60, P277, DOI [10.3322/caac.20073, 10.3322/caac.21254]
Jin Z, 2008, ENDOSCOPY, V40, P314, DOI 10.1055/s-2007-995476
Jin Zhendong, 2012, Gastrointest Endosc Clin N Am, V22, P325, DOI 10.1016/j.giec.2012.04.012
John S, 2007, J SURG ONCOL, V96, P89, DOI 10.1002/jso.20764
Kahaleh M, 2006, GASTROINTEST ENDOSC, V64, P52, DOI 10.1016/j.gie.2006.01.063
Kaufman M, 2010, J CLIN GASTROENTEROL, V44, P127, DOI 10.1097/MCG.0b013e3181bb854d
Khashab MA, 2013, GASTROINTEST ENDOSC, V78, P102, DOI 10.1016/j.gie.2013.03.004
Khashab MA, 2012, GASTROINTEST ENDOSC, V76, P962, DOI 10.1016/j.gie.2012.07.006
Kim HJ, 2012, GASTROINTEST ENDOSC, V76, P1039, DOI 10.1016/j.gie.2012.07.015
Kim YS, 2010, ENDOSCOPY, V42, P496, DOI 10.1055/s-0029-1244082
Komaki T, 2011, PANCREATOLOGY, V11, P47, DOI 10.1159/000323508
Koong AC, 2005, INT J RADIAT ONCOL, V63, P320, DOI 10.1016/j.ijrobp.2005.07.002
LeBlanc JK, 2011, GASTROINTEST ENDOSC, V74, P1300, DOI 10.1016/j.gie.2011.07.073
Loeve US, 2013, GASTROINTEST ENDOSC, V77, P151, DOI 10.1016/j.gie.2012.03.005
Luz LP, 2014, WORLD J GASTROENTERO, V20, P7808, DOI 10.3748/wjg.v20.i24.7808
Maranki J, 2009, ENDOSCOPY, V41, P532, DOI 10.1055/s-0029-1214712
MCCAUGHAN JS, 1991, ARCH SURG-CHICAGO, V126, P111
McGreevy K, 2013, PAIN PRACT, V13, P89, DOI 10.1111/j.1533-2500.2012.00557.x
Mimuro A, 2003, GASTROINTEST ENDOSC, V58, P283, DOI 10.1067/mge.2003.354
Mittal MK, 2012, NEUROLOGY, V78, pE57, DOI 10.1212/WNL.0b013e318248df51
Nguyen-Tang T, 2010, ENDOSCOPY, V42, P232, DOI 10.1055/s-0029-1243858
Ogura T, 2015, WORLD J GASTROENTERO, V21, P1049, DOI 10.3748/wjg.v21.i4.1049
Olive KP, 2009, SCIENCE, V324, P1457, DOI 10.1126/science.1171362
Ortner MAEJ, 1998, GASTROENTEROLOGY, V114, P536, DOI 10.1016/S0016-5085(98)70537-2
Ortner MEJ, 2003, GASTROENTEROLOGY, V125, P1355, DOI 10.1016/j.gastro.2003.07.015
Park DH, 2013, GASTROINTEST ENDOSC, V78, P91, DOI 10.1016/j.gie.2013.01.042
Park DH, 2011, GASTROINTEST ENDOSC, V74, P1276, DOI 10.1016/j.gie.2011.07.054
Park WG, 2010, GASTROINTEST ENDOSC, V71, P513, DOI 10.1016/j.gie.2009.10.030
Pereira SP, 2007, EUR J GASTROEN HEPAT, V19, P479, DOI 10.1097/MEG.0b013e328013c0bd
Pishvaian AC, 2006, GASTROINTEST ENDOSC, V64, P412, DOI 10.1016/j.gie.2006.01.048
Puli SR, 2009, DIGEST DIS SCI, V54, P2330, DOI 10.1007/s10620-008-0651-x
Rwigema JCM, 2011, AM J CLIN ONCOL-CANC, V34, P63, DOI 10.1097/COC.0b013e3181d270b4
Sakamoto H, 2010, AM J GASTROENTEROL, V105, P2599, DOI 10.1038/ajg.2010.339
Sanders MK, 2010, GASTROINTEST ENDOSC, V71, P1178, DOI 10.1016/j.gie.2009.12.020

Savides TJ, 2009, GASTROINTEST ENDOSC, V69, pS3, DOI 10.1016/j.gie.2008.10.060
Shah JN, 2012, GASTROINTEST ENDOSC, V75, P56, DOI 10.1016/j.gie.2011.08.032
Sun HH, 2014, SCI REP-UK, V4, DOI 10.1038/srep06747
Sun S, 2006, ENDOSCOPY, V38, P399, DOI 10.1055/s-2006-925253
Tyberg A, 2015, GASTROINTEST ENDOSC, V81, P1011, DOI 10.1016/j.gie.2014.10.018
van Hooft JE, 2010, GASTROINTEST ENDOSC, V72, P530, DOI 10.1016/j.gie.2010.05.025
Vonlaufen A, 2008, CANCER RES, V68, P2085, DOI 10.1158/0008-5472.CAN-07-2477
Widmer J, 2014, DIGEST ENDOSC, V26, P599, DOI 10.1111/den.12163
Wiersema MJ, 1996, GASTROINTEST ENDOSC, V44, P656, DOI 10.1016/S0016-5107(96)70047-0
Witzigmann H, 2006, ANN SURG, V244, P230, DOI 10.1097/01.sla.0000217639.10331.47
Wolfsen HC, 2005, J CLIN GASTROENTEROL, V39, P653, DOI 10.1097/01.mcg.0000173930.60115.62
Wu YL, 2006, J SURG ONCOL, V94, P392, DOI 10.1002/jso.20580
Wyse JM, 2011, J CLIN ONCOL, V29, P3541, DOI 10.1200/JCO.2010.32.2750
Yan BM, 2007, AM J GASTROENTEROL, V102, P430, DOI 10.1111/j.1572-0241.2006.00967.x
Yeo Theresa Pluth, 2002, Curr Probl Cancer, V26, P176, DOI 10.1067/mcn.2002.129579
Zoepf T, 2005, AM J GASTROENTEROL, V100, P2426, DOI 10.1111/j.1572-0241.2005.00318.x
NR 96
TC 7
Z9 11
U1 0
U2 5
PU BAISHIDENG PUBLISHING GROUP INC
PI PLEASANTON
PA 7041 Koll Center Parkway, Suite 160, PLEASANTON, CA, UNITED STATES
SN 1948-5190
J9 WORLD J GASTRO ENDOS
JI World J. Gastrointest. Endosc.
PD APR 10
PY 2016
VL 8
IS 7
BP 319
EP 329
DI 10.4253/wjge.v8.i7.319
PG 11
WC Gastroenterology & Hepatology
WE Emerging Sources Citation Index (ESCI)
SC Gastroenterology & Hepatology
GA DI1WT
UT WOS:000373288400001
PM 27076870
OA hybrid

DA 2026-05-09
ER

PT J
AU Bazaga, S
García-Alonso, FJ
Tormo, JRA
Moreno, BM
Sanchiz, V
Gornals, JB
Loras, C
Terán, A
Vazquez-Sequeiros, E
Sanz, RP
Súbtil, JC
Pérez-Millan, A
Porta, FU
Vila, JJ
de la Serna-Higuera, C
Couto-Worner, I
Guarner-Argente, C
Perez-Miranda, M
AF Bazaga, Sergio
Garcia-Alonso, Francisco Javier
Tormo, Jose Ramon Aparicio
Moreno, Belen Martinez
Sanchiz, Vicente
Gornals, Joan B.
Loras, Carme
Teran, Alvaro
Vazquez-Sequeiros, Enrique
Sanz, Rafael Pedraza
Subtil, Jose Carlos
Perez-Millan, Antonio
Porta, Francisco Uceda
Vila, Juan J.
de la Serna-Higuera, Carlos
Couto-Worner, Ignacio
Guarner-Argente, Carlos
Perez-Miranda, Manuel
CA RNPAL
Natl Lumen Apposing Met Stent

TI Endoscopic ultrasound-guided gallbladder drainage with long-term lumen-apposing metal stent indwell: 1-year results from a prospective nationwide observational study

SO JOURNAL OF GASTROENTEROLOGY AND HEPATOLOGY

LA English

DT Article

DE endoscopic ultrasound; EUS-guided gallbladder drainage (EUS-GBD); lumen-apposing metal stent (LAMS); prospective nationwide observational study

ID 2018 DIAGNOSTIC-CRITERIA; PERCUTANEOUS CHOLECYSTOSTOMY; ACUTE CHOLECYSTITIS; MANAGEMENT; OUTCOMES; ERCP

AB Background and AimThis study aimed to determine safety and risk factors for adverse events (AEs) of endoscopic ultrasound-guided gallbladder drainage (EUS-GBD) with long-term indwell of lumen-apposing metal stents (LAMS).MethodsThis study is a multicenter prospective observational study on consecutive high surgical-risk patients requiring gallbladder drainage who underwent EUS-GBD with LAMS over 12 months. Centralized telephone follow-up interviews were conducted every 3 months for 1 year. Patients were censored at LAMS removal, cholecystectomy, or death. AE-free survival was determined using log-rank tests. Cumulative risks were estimated using life-table analysis.ResultsEighty-two patients were included (53.7% male, median [interquartile range] age of 84.6 [76.5-89.8] years, and 85.4% with acute cholecystitis). Technical success was achieved in 79 (96.3%), and clinical success in 73 (89%). No patient was lost to follow-up; 45 patients (54.9%) completed 1-year follow-up with in situ LAMS. Median (interquartile range) LAMS indwell time was 364 (47-367) days. Overall, 12 (14.6%) patients presented 14 AEs, including 5 (6.1%) recurrent biliary events (3 acute cholangitis, 1 mild acute pancreatitis, and 1 acute cholecystitis). Patients with pancreatobiliary malignancy had an increased risk of recurrent biliary events (33% vs 1.5%, P = 0.001). The overall 1-year cumulative risk of recurrent biliary events was 9.7% (4.1-21.8%). The 1-year risk of AEs and of severe AEs was 18.8% (11-31.2%) and 7.9% (3.3-18.2%), respectively. Pancreatobiliary malignancy was the single risk factor for recurrent biliary events; LAMS misdeployment was the strongest risk factor for AEs.ConclusionsLong-term LAMS indwell does not increase the risk of delayed AEs following EUS-GBD.

image

C1 [Bazaga, Sergio; Garcia-Alonso, Francisco Javier; de la Serna-Higuera, Carlos; Perez-Miranda, Manuel] Hosp Univ Rio Hortega, Dept Gastroenterol, Valladolid, Spain.

[Bazaga, Sergio; Guarner-Argente, Carlos] Univ Autonoma Barcelona, Hosp Santa Creu & St Pau, PhD Program, Barcelona, Spain.

[Guarner-Argente, Carlos] Univ Autonoma Barcelona, Hosp Santa Creu & St Pau, Dept Gastroenterol, Barcelona, Spain.

[Gornals, Joan B.] Univ Barcelona, Hosp Univ Bellvitge, Bellvitge Biomed Res Inst IDIBELL, Dept Digest Dis, Barcelona, Spain.

[Loras, Carme] Ctr Invest Biomed Red Enfermedades Hepat & Digest, Barcelona, Spain.

[Tormo, Jose Ramon Aparicio; Moreno, Belen Martinez] Hosp Gen Univ Alicante, Alicante, Spain.

[Porta, Francisco Uceda] Hosp Gen Univ Elche, Alicante, Spain.

[Sanchiz, Vicente] Hosp Clin Univ Valencia, Valencia, Spain.

[Loras, Carme] Hosp Univ Mutua Terrassa, Fundacio Recerca Mutua Terrassa, Dept Gastroenterol, Terrassa, Spain.

[Teran, Alvaro] Hosp Univ Marques Valdecilla, Santander, Spain.

[Vazquez-Sequeiros, Enrique] Hosp Ramon & Cajal, IRYCIS, Dept Gastroenterol & Hepatol, Endoscopy Unit, Madrid, Spain.
[Sanz, Rafael Pedraza] Hosp Gen Univ Castellon, Castellon De La Plana, Spain.
[Subtil, Jose Carlos] Univ Navarra, Univ Navarra Clin, Dept Gastroenterol, Endoscopy Unit, Pamplona, Spain.
[Vila, Juan J.] Complejo Hosp Navarra, Pamplona, Spain.
[Perez-Millan, Antonio] Hosp Gen Rio Carrion, Palencia, Spain.
[Couto-Worner, Ignacio] Complexo Hosp Univ A Coruna, La Coruna, Spain.
C3 Hospital del Rio Hortega; Autonomous University of Barcelona; Hospital of Santa Creu i Sant Pau; Hospital of Santa Creu i Sant Pau; Autonomous University of Barcelona; Institut d'Investigacio Biomedica de Bellvitge (IDIBELL); Bellvitge University Hospital; University of Barcelona; CIBER - Centro de Investigacion Biomedica en Red; CIBEREHD; General University Hospital of Alicante; Hospital Clinic Universitari de Valencia; Hospital Universitario Mutua Terrassa; Hospital Universitario Marques de Valdecilla (HUMV); Hospital Universitario Ramon y Cajal; University of Navarra; Servicio Navarro de Salud - Osasunbidea; Universidade da Coruna; Complejo Hospitalario Universitario A Coruna
RP Bazaga, S (corresponding author), Hosp Univ Rio Hortega, Dept Gastroenterol, C Dulzaina 2, Valladolid 47012, Spain.
EM sergio.bpr@gmail.com
RI ; García-Alonso, Francisco Javier/GOK-0879-2022; Bazaga, Sergio/PNG-0684-2026; Vila, Juan/E-9123-2010; Guarner-Argente, Carlos/M-7616-2019
OI gornals, joan b/0000-0001-8857-3556; Aparicio Tormo, Jose Ramón/0000-0003-2245-4006; perez millan, Antonio/0000-0003-1333-8900; García-Alonso, Francisco Javier/0000-0002-1425-8534; Guarner-Argente, Carlos/0000-0002-8180-1488; LORAS ALASTRUEY, CARME/0000-0002-4035-3316
FU Gerencia Regional de Salud, Junta de Castilla y Leon (Spain) [1992/A/19, INT_M_12_23]
FX This study was supported by the Gerencia Regional de Salud, Junta de Castilla y Leon (Spain), grant number 1992/A/19. Francisco Javier Garcia-Alonso was supported by the Gerencia Regional de Salud, Junta de Castilla y Leon (Spain), grant number INT_M_12_23.
CR Al-Jundi W, 2012, ANN ROY COLL SURG, V94, P99, DOI 10.1308/003588412X13171221501302
Bang JY, 2017, GUT, V66, P2054, DOI 10.1136/gutjnl-2016-312812
Cho JH, 2022, SURG ENDOSC, V36, P8950, DOI 10.1007/s00464-022-09346-z
Choi JH, 2014, ENDOSCOPY, V46, P656, DOI 10.1055/s-0034-1365720
Cotton PB, 2010, GASTROINTEST ENDOSC, V71, P446, DOI 10.1016/j.gie.2009.10.027
Dollhopf M, 2017, GASTROINTEST ENDOSC, V86, P636, DOI 10.1016/j.gie.2017.02.027
Fugazza A, 2022, GASTROINTEST ENDOSC, V95, P896, DOI 10.1016/j.gie.2021.12.032
Fugazza A, 2020, GASTROINTEST ENDOSC, V91, P574, DOI 10.1016/j.gie.2019.11.021
Fujii-Lau LL, 2016, SURG ENDOSC, V30, P1396, DOI 10.1007/s00464-015-4342-3
Ghandour B, 2022, GASTROINTEST ENDOSC, V95, P80, DOI 10.1016/j.gie.2021.07.023

Harris PA, 2009, J BIOMED INFORM, V42, P377, DOI 10.1016/j.jbi.2008.08.010
Higa JT, 2019, GASTROINTEST ENDOSC, V90, P483, DOI 10.1016/j.gie.2019.04.238
Inoue T, 2016, GASTROINTEST ENDOSC, V83, P905, DOI 10.1016/j.gie.2015.08.072
Irani S, 2017, CLIN GASTROENTEROL H, V15, P738, DOI 10.1016/j.cgh.2016.12.021
Irani SS, 2023, ANN SURG, V278, pE556, DOI 10.1097/SLA.0000000000005784
Itoi T, 2008, GASTROINTEST ENDOSC, V68, P455, DOI 10.1016/j.gie.2008.02.052
James TW, 2019, ENDOSC INT OPEN, V7, pE1099, DOI 10.1055/a-0965-6662
Jang JW, 2012, GASTROENTEROLOGY, V142, P805, DOI 10.1053/j.gastro.2011.12.051
Kiriyama S, 2018, J HEPATO-BIL-PAN SCI, V25, P17, DOI 10.1002/jhbp.512
Law R, 2016, CLIN GASTROENTEROL H, V14, P476, DOI 10.1016/j.cgh.2015.10.026
Lisotti A, 2022, SURG ENDOSC, V36, P569, DOI 10.1007/s00464-021-08318-z
Loozen CS, 2018, BMJ-BRIT MED J, V363, DOI 10.1136/bmj.k3965
Maruta A, 2021, J HEPATO-BIL-PAN SCI, V28, P1138, DOI 10.1002/jhbp.967
MEDDEV 2.7/3 rev 3 M 2015, 2015, 273 MEDDEV, P1
Miura F, 2018, J HEPATO-BIL-PAN SCI, V25, P31, DOI 10.1002/jhbp.509
Mori Y, 2018, J HEPATO-BIL-PAN SCI, V25, P87, DOI 10.1002/jhbp.504
Mussetto A, 2018, ANN GASTROENTEROL, V31, P535, DOI 10.20524/aog.2018.0287
Podboy A, 2021, GASTROINTEST ENDOSC, V93, P797, DOI 10.1016/j.gie.2020.09.040
Siddiqui A, 2019, SURG ENDOSC, V33, P1260, DOI 10.1007/s00464-018-6406-7
Tenner S, 2013, AM J GASTROENTEROL, V108, P1400, DOI 10.1038/ajg.2013.218
Teoh AY, 2019, ENDOSC INT OPEN, V7, pE964, DOI 10.1055/a-0915-2098
Teoh AYB, 2020, GUT, V69, P1085, DOI 10.1136/gutjnl-2019-319996
Teoh AYB, 2019, ENDOSC INT OPEN, V7, pE662, DOI 10.1055/a-0830-4675
Teoh AYB, 2017, ENDOSCOPY, V49, P130, DOI 10.1055/s-0042-119036
Yuste RT, 2019, ANN GASTROENTEROL, V32, P514, DOI 10.20524/aog.2019.0395
van der Merwe SW, 2022, ENDOSCOPY, V54, P185, DOI 10.1055/a-1717-1391
Walter D, 2016, GUT, V65, P6, DOI 10.1136/gutjnl-2015-309925
Yang D, 2018, ENDOSC INT OPEN, V6, pE1267, DOI 10.1055/a-0732-502
Yokoe M, 2018, J HEPATO-BIL-PAN SCI, V25, P41, DOI 10.1002/jhbp.515
NR 39
TC 16
Z9 19
U1 0
U2 8
PU WILEY
PI HOBOKEN
PA 111 RIVER ST, HOBOKEN 07030-5774, NJ USA
SN 0815-9319
EI 1440-1746
J9 J GASTROEN HEPATOL
JI J. Gastroenterol. Hepatol.
PD FEB

PY 2024
VL 39
IS 2
BP 360
EP 368
DI 10.1111/jgh.16392
EA NOV 2023
PG 9
WC Gastroenterology & Hepatology
WE Science Citation Index Expanded (SCI-EXPANDED)
SC Gastroenterology & Hepatology
GA HW9M9
UT WOS:001093179800001
PM 37920889
DA 2026-05-09
ER

PT J
AU Yang, MJ
Hwang, JC
Yoo, BM
Kim, JH
AF Yang, Min Jae
Hwang, Jae Chul
Yoo, Byung Moo
Kim, Jin Hong
TI Tips for Dealing with Common Beginner's Mistakes Made during Endoscopic
Ultrasound-Guided Gallbladder Drainage
SO DIGESTIVE DISEASES
LA English
DT Article
DE Stent misplacement; Endoscopic ultrasound; Endoscopic ultrasound-guided
gallbladder drainage; Acute cholecystitis; Technical failure
ID APPOSING METAL STENT; PERCUTANEOUS CHOLECYSTOSTOMY
AB Endoscopic ultrasound-guided gallbladder drainage (EUS-GBD) has been performed as an alternative to percutaneous drainage in surgically high-risk patients. Technical failures of EUS-GBD made by beginners are often attributed to failure of over-the-wire insertion of a fistula-dilating device or stent delivery system into the gallbladder, or stent misplacement in the final technical step. We herein report technical tips to prevent the failure of fistula dilation and provide tricks to avoid inward or outward stent misplacement.
C1 [Yang, Min Jae; Hwang, Jae Chul; Yoo, Byung Moo; Kim, Jin Hong] Ajou Univ, Dept Gastroenterol, Sch Med, Suwon, South Korea.
C3 Ajou University

RP Yoo, BM (corresponding author), Ajou Univ, Dept Gastroenterol, Sch Med, 164 Worldcup Ro, Suwon 16499, Gyeonggi Do, South Korea.
EM ybm6403@gmail.com; jinhkim@ajou.ac.kr
CR Choi JH, 2017, GASTROINTEST ENDOSC, V85, P357, DOI 10.1016/j.gie.2016.07.067
Choi JH, 2015, DIGEST ENDOSC, V27, P1, DOI 10.1111/den.12386
Choi JH, 2014, ENDOSCOPY, V46, P656, DOI 10.1055/s-0034-1365720
Irani S, 2017, CLIN GASTROENTEROL H, V15, P738, DOI 10.1016/j.cgh.2016.12.021
Jang JW, 2012, GASTROENTEROLOGY, V142, P805, DOI 10.1053/j.gastro.2011.12.051
Law R, 2016, CLIN GASTROENTEROL H, V14, P476, DOI 10.1016/j.cgh.2015.10.026
Ligresti D, 2018, ENDOSCOPY, V50, pE190, DOI 10.1055/a-0605-3198
Minaga K, 2019, DIGEST ENDOSC, V31, P180, DOI 10.1111/den.13242
Teoh AYB, 2017, ENDOSCOPY, V49, P130, DOI 10.1055/s-0042-119036
Tyberg A, 2018, J CLIN GASTROENTEROL, V52, P79, DOI 10.1097/MCG.0000000000000786
Yang MJ, 2018, J GASTROEN HEPATOL, V33, P772, DOI 10.1111/jgh.14060
NR 11
TC 8
Z9 9
U1 0
U2 2
PU KARGER
PI BASEL
PA ALLSCHWILERSTRASSE 10, CH-4009 BASEL, SWITZERLAND
SN 0257-2753
EI 1421-9875
J9 DIGEST DIS
JI Dig. Dis.
PD NOV
PY 2020
VL 38
IS 6
BP 542
EP 546
DI 10.1159/000505644
PG 5
WC Gastroenterology & Hepatology
WE Science Citation Index Expanded (SCI-EXPANDED)
SC Gastroenterology & Hepatology
GA PZ6MS
UT WOS:000612854600013
PM 32053818
OA Bronze
DA 2026-05-09

ER

PT J
AU Rana, SS
AF Rana, Surinder S.
TI Endoscopic ultrasound-guided gallbladder drainage: a technical review
SO ANNALS OF GASTROENTEROLOGY
LA English
DT Review
DE Stent; endosonography; magnetic resonance cholangiopancreatography;
endoscopic retrograde cholangiopancreatography; lumen-apposing metal
stent
ID ACUTE CHOLECYSTITIS; METAL STENT; INTERVENTIONS; FEASIBILITY; EFFICACY
AB Endoscopic ultrasound-guided gallbladder drainage (EUS-GBD) has emerged as a safe and effective minimally invasive alternative to both percutaneous and endoscopic transpapillary GBD in patients with acute cholecystitis. Over the last few years, the technique, as well as the indications for EUS-GBD have been gradually evolving, and the procedure has become simpler and safer as the accepted indications have expanded. The development of lumen-apposing metal stents (LAMS) has allowed us to realize the dream of creating endoscopic gastrointestinal anastomoses, and has thus paved the way for a safer EUS-GBD. Single step EUS-guided LAMS delivery systems have obviated the use of other endoscopic accessories and thus made EUS-GBD simpler and safer. However, EUS-GBD can be associated with potentially serious complications, and therefore should be performed by expert interventional endosonologists at centers with surgical and radiological back up. EUS-GBD is a relatively new procedure still in its infancy, but continued improvement in EUS accessories and dedicated stents will make this procedure safer and also expand its current indications. This review focuses on the technical aspects, including procedural details, as well as the complications of EUS-GBD.
C1 [Rana, Surinder S.] Postgrad Inst Med Educ & Res PGIMER, Dept Gastroenterol, Chandigarh 160012, India.
C3 Post Graduate Institute of Medical Education & Research (PGIMER),
Chandigarh
RP Rana, SS (corresponding author), Postgrad Inst Med Educ & Res PGIMER, Dept Gastroenterol, Chandigarh 160012, India.
EM drsurinderrana@yahoo.co.in
CR Adler DG, 2018, ENDOSC ULTRASOUND, V7, P1, DOI 10.4103/eus.eus_3_18
Anderloni A, 2017, ENDOSC INT OPEN, V5, pE1100, DOI 10.1055/s-0043-118828
Anderloni A, 2016, SURG ENDOSC, V30, P5200, DOI 10.1007/s00464-016-4894-x
[Anonymous], 2019, INTERVENTIONAL ENDOS, DOI DOI 10.1016/B978-0-12-811913-6.00002-3
Boregowda Umesha, 2018, World J Gastrointest Pharmacol Ther, V9, P47, DOI [10.4292/wjgpt.v9.i6.47, 10.4292/wjgpt.v9.i6.47]
Bruno MJ, 2020, ENDOSC INT OPEN, V08, pE1161, DOI 10.1055/a-1135-8953
Chan JHY, 2018, CLIN ENDOSC, V51, P150, DOI 10.5946/ce.2017.125
Chan SM, 2017, GASTROINTEST ENDOSC, V85, P1225, DOI 10.1016/j.gie.2016.10.014
Crinò SF, 2019, J GASTROINTEST LIVER, V28, P337
Guo JT, 2017, ENDOSC ULTRASOUND, V6, P285, DOI 10.4103/eus.eus_85_17
Iino C, 2018, ENDOSC INT OPEN, V6, pE594, DOI 10.1055/s-0044-102091

Imai H, 2016, GASTROINTEST ENDOSC, V84, P147, DOI 10.1016/j.gie.2015.12.024
Itoi T, 2012, GASTROINTEST ENDOSC, V75, P870, DOI 10.1016/j.gie.2011.10.020
Itoi T, 2011, J HEPATO-BIL-PAN SCI, V18, P282, DOI 10.1007/s00534-010-0310-4
Jain D, 2018, CLIN ENDOSC, V51, P450, DOI 10.5946/ce.2018.024
James TW, 2019, ENDOSC ULTRASOUND, V8, pS28, DOI 10.4103/eus.eus_41_19
Jones MW, 2020, ANATOMY ABDOMEN PELV
Kalva NR, 2018, CAN J GASTROENTEROL, V2018, DOI 10.1155/2018/7070961
Kamata K, 2017, WORLD J GASTROENTERO, V23, P661, DOI 10.3748/wjg.v23.i4.661
Kim JJ, 2019, J CLIN GASTROENTEROL, V53, P392, DOI 10.1097/MCG.0000000000000890
Krishnamoorthi R, 2020, SURG ENDOSC, V34, P1904, DOI 10.1007/s00464-020-07409-7
Law Ryan, 2018, Gastrointest Endosc Clin N Am, V28, P187, DOI [10.1016/j.giec.2017.11.006, 10.1016/j.giec.2017.11.006]
Luk SWY, 2019, ENDOSCOPY, V51, P722, DOI 10.1055/a-0929-6603
Mahadevan V, 2014, SURGERY OXFORD
Manta R, 2017, ENDOSC INT OPEN, V5, pE1111, DOI 10.1055/s-0043-118659
Maruta A, 2020, J HEPATO-BIL-PAN SCI, V27, P429, DOI 10.1002/jhbp.744
Ogawa O, 2008, CAN J GASTROENTEROL, V22, P681, DOI 10.1155/2008/702516
Ogura T, 2019, DIGEST ENDOSC, V31, P55, DOI 10.1111/den.13334
Okamoto K, 2018, J HEPATO-BIL-PAN SCI, V25, P55, DOI 10.1002/jhbp.516
Perez-Miranda M, 2018, ENDOSC ULTRASOUND, V7, P79, DOI 10.4103/eus.eus_5_18
Perisetti A, 2020, GASTROINTEST ENDOSC, V91, P961, DOI 10.1016/j.gie.2019.12.008
Posner H, 2020, TRANSL GASTROENT HEP, V5, DOI 10.21037/tgh.2019.12.20
Rana SS, 2019, ANN GASTROENTEROL, V32, P441, DOI 10.20524/aog.2019.0404
Rana SS, 2020, ANN GASTROENTEROL, V33, P426, DOI 10.20524/aog.2020.0499
Sagami R, 2020, ANN GASTROENTEROL, V33, P391, DOI 10.20524/aog.2020.0496
Saumoy M, 2019, J CLIN GASTROENTEROL, V53, P231, DOI 10.1097/MCG.0000000000001036
Sharma V, 2015, WORLD J GASTRO ENDOS, V7, P628, DOI 10.4253/wjge.v7.i6.628
Sobani ZA, 2021, DIGEST DIS SCI, V66, P2154, DOI 10.1007/s10620-020-06520-y
Teoh AY, 2019, ENDOSC INT OPEN, V7, pE964, DOI 10.1055/a-0915-2098
Teoh AYB, 2020, GUT, V69, P1085, DOI 10.1136/gutjnl-2019-319996
Teoh AYB, 2020, J GASTROEN HEPATOL, V35, P1288, DOI 10.1111/jgh.15099
Teoh AYB, 2020, ENDOSC INT OPEN, V8, pE97, DOI 10.1055/a-1036-6337
Teoh AYB, 2019, ENDOSC ULTRASOUND, V8, pS40, DOI 10.4103/eus.eus_49_19
Widmer J, 2014, DIGEST ENDOSC, V26, P525, DOI 10.1111/den.12221
Xu MM, 2016, THER ADV GASTROENTER, V9, P138, DOI 10.1177/1756283X15618178
Yang MJ, 2020, DIGEST DIS, V38, P542, DOI 10.1159/000505644
Zou YP, 2007, HEPATOB PANCREAT DIS, V6, P199
NR 47
TC 26
Z9 32
U1 0
U2 0

PU HELLENIC SOC GASTROENTEROLOGY
PI ATHENS
PA DEMOKRATIAS AVE 67, ATHENS, 15451, GREECE
SN 1108-7471
EI 1792-7463
J9 ANN GASTROENTEROL
JI Ann. Gastroenterol.
PY 2021
VL 34
IS 2
BP 142
EP 148
DI 10.20524/aog.2020.0568
PG 7
WC Gastroenterology & Hepatology
WE Emerging Sources Citation Index (ESCI)
SC Gastroenterology & Hepatology
GA QT7NO
UT WOS:000626776600003
PM 33654351
DA 2026-05-09
ER

PT J
AU Nakano, R
Shiomi, H
Ota, S
Iijima, H
AF Nakano, Ryota
Shiomi, Hideyuki
Ota, Shogo
Iijima, Hiroko
TI Endoscopic ultrasound-guided celiac plexus neurolysis for managing
abdominal pain related with advanced cancer
SO INTERNATIONAL JOURNAL OF GASTROINTESTINAL INTERVENTION
LA English
DT Review
DE Chronic pancreatitis; Endoscopic ultrasound-guided celiac ganglia
neurolysis; Endoscopic ultrasound-guided celiac plexus neurolysis;
Pancreatic cancer
ID UNRESECTABLE PANCREATIC-CANCER; SPINAL-CORD INFARCTION; GANGLIA
NEUROLYSIS; BLOCK; EFFICACY; MANAGEMENT; RELIEF; COMPLICATION;

MULTICENTER; TOMOGRAPHY

AB Cancer associated abdominal pain, as typified by pancreatic cancer, has conventionally been treated with narcotic drugs, but often the severe abdominal pain is difficult to control. Endoscopic ultrasound-guided celiac plexus neurolysis (EUS-CPN) is commonly performed for abdominal pain that is difficult to control. EUS-CPN can be performed more safely and reliably than other conventional procedures on the celiac plexus, and good outcomes of pain relief have been reported. Although a variety of endoscopic techniques are available for EUS-CPN, the choice varies among institutions, and evidence on the efficacy and safety of each procedure is limited. In this review, we summarize the indications for treatment, specific endoscopic techniques, therapeutic efficacy for pain relief, and complications from previous reports on EUS-CPN.

Copyright (C) 2022, Society of Gastrointestinal Intervention.

C1 [Nakano, Ryota; Shiomi, Hideyuki; Ota, Shogo; Iijima, Hiroko] Ilyogo Med Univ, Dept Internal Med, Div Gastroenterol & Hepatobiliary Pancreatol, 1-1 Mukogawa Cho, Nishinomiya, Hyogo 6638501, Japan.

RP Shiomi, H (corresponding author), Ilyogo Med Univ, Dept Internal Med, Div Gastroenterol & Hepatobiliary Pancreatol, 1-1 Mukogawa Cho, Nishinomiya, Hyogo 6638501, Japan.

EM hideshio0403@gmail.com

OI Iijima, Hiroko/0000-0002-5841-8827; Nakano, Ryota/0000-0002-1844-2865;
Ota, Shogo/0000-0001-5171-1847

CR Ascunce G, 2011, GASTROINTEST ENDOSC, V73, P267, DOI 10.1016/j.gie.2010.10.029
Asif AA, 2021, J COMMUNITY HOSP INT, V11, P536, DOI 10.1080/20009666.2021.1929049
Bang JY, 2019, GASTROINTEST ENDOSC, V89, P58, DOI 10.1016/j.gie.2018.08.005
Bhatnagar S, 2014, PAIN PRACT, V14, pE63, DOI 10.1111/papr.12107
Caraceni A, 1996, CANCER-AM CANCER SOC, V78, P639
Das A, 2000, Cancer Control, V7, P452
Doi S, 2013, ENDOSCOPY, V45, P362, DOI 10.1055/s-0032-1326225
EISENBERG E, 1995, ANESTH ANALG, V80, P290, DOI 10.1097/00000539-199502000-00015
Facciorusso A, 2020, ENDOSC ULTRASOUND, V9, P238, DOI 10.4103/eus.eus_24_20
Fujii L, 2012, ENDOSCOPY, V44, pE265, DOI 10.1055/s-0032-1309708
Gerke H, 2006, GASTROINTEST ENDOSC, V64, P35, DOI 10.1016/j.gie.2006.01.071
Gimeno-García AZ, 2012, ENDOSCOPY, V44, pE267, DOI 10.1055/s-0032-1309709
Gress F, 1999, AM J GASTROENTEROL, V94, P900
Gress F, 2001, AM J GASTROENTEROL, V96, P409
Gunaratnam NT, 2001, GASTROINTEST ENDOSC, V54, P316, DOI 10.1067/mge.2001.117515
Han CQ, 2021, WORLD J GASTROENTERO, V27, P69, DOI 10.3748/wjg.v27.i1.69
Hao SJ, 2014, SURG LAPARO ENDO PER, V24, P31, DOI 10.1097/SLE.0000000000000032
Ishiwatari H, 2014, WORLD J GASTROENTERO, V20, P10512, DOI 10.3748/wjg.v20.i30.10512
Iwata K, 2011, DIGEST ENDOSC, V23, P140, DOI 10.1111/j.1443-1661.2010.01046.x
Jang HY, 2013, CLIN ENDOSC, V46, P306, DOI 10.5946/ce.2013.46.3.306
Kanno Y, 2020, GASTROINTEST ENDOSC, V92, P120, DOI 10.1016/j.gie.2020.01.011
Kaufman M, 2010, J CLIN GASTROENTEROL, V44, P127, DOI 10.1097/MCG.0b013e3181bb854d
Koulouris AI, 2021, PANCREATOLOGY, V21, P434, DOI 10.1016/j.pan.2020.12.016
LeBlanc JK, 2011, GASTROINTEST ENDOSC, V74, P1300, DOI 10.1016/j.gie.2011.07.073
LeBlanc JK, 2009, GASTROINTEST ENDOSC, V69, P835, DOI 10.1016/j.gie.2008.05.071
Levy M, 2006, AM J GASTROENTEROL, V101, P1787, DOI 10.1111/j.1572-0241.2006.00685.x

Levy MJ, 2008, AM J GASTROENTEROL, V103, P98, DOI 10.1111/j.1572-0241.2007.01607.x
Levy MJ, 2019, CLIN GASTROENTEROL H, V17, P728, DOI 10.1016/j.cgh.2018.08.040
Li M, 2021, MEDICINE, V100, DOI 10.1097/MD.0000000000027103
Mekaroonkamol P, 2015, J PANCREAS, V16, P33, DOI 10.6092/1590-8577/2890
Minaga K, 2016, THER ADV GASTROENTER, V9, P483, DOI 10.1177/1756283X16644248
Mittal MK, 2012, NEUROLOGY, V78, pE57, DOI 10.1212/WNL.0b013e318248df51
Nobre Moura Renata, 2015, Rev. gastroenterol. Perú, V35, P333
Nagels W, 2013, PAIN MED, V14, P1140, DOI 10.1111/pme.12176
O'Toole TM, 2009, ENDOSCOPY, V41, P593, DOI 10.1055/s-0029-1214868
Puli SR, 2009, DIGEST DIS SCI, V54, P2330, DOI 10.1007/s10620-008-0651-x
Sahai AV, 2009, AM J GASTROENTEROL, V104, P326, DOI 10.1038/ajg.2008.64
Sakamoto H, 2010, AM J GASTROENTEROL, V105, P2599, DOI 10.1038/ajg.2010.339
Saleh AAG, 2021, J GASTROINTEST CANC, V52, P682, DOI 10.1007/s12029-020-00449-1
Seicean A, 2013, J GASTROINTEST LIVER, V22, P59
Siegel RL, 2016, CA-CANCER J CLIN, V66, P7, DOI 10.3322/caac.21332
Téllez-Avila FI, 2013, ENDOSC ULTRASOUND, V2, P153, DOI [10.4103/2303-9027.117664, 10.7178/eus.06.007]
Teoh AYB, 2018, GUT, V67, P1209, DOI 10.1136/gutjnl-2017-314341
Wiechowska-Kozlowska A, 2012, GASTROENT RES PRACT, V2012, DOI 10.1155/2012/503098
Wiersema MJ, 1996, GASTROINTEST ENDOSC, V44, P656, DOI 10.1016/S0016-5107(96)70047-0
Wong GY, 2004, JAMA-J AM MED ASSOC, V291, P1092, DOI 10.1001/jama.291.9.1092
Wray CJ, 2005, GASTROENTEROLOGY, V128, P1626, DOI 10.1053/j.gastro.2005.03.035
Wyse JM, 2011, J CLIN ONCOL, V29, P3541, DOI 10.1200/JCO.2010.32.2750
Zhang CL, 2008, DIGEST DIS SCI, V53, P856, DOI 10.1007/s10620-007-9905-2
Zhao YC, 2021, ANN PALLIAT MED, V10, P1755, DOI 10.21037/apm-20-1158
NR 50
TC 1
Z9 1
U1 0
U2 4
PU SOC GASTROINTESTINAL INTERVENTION
PI SEOUL
PA 12, HAKDONG-RO 31, GANGNAM-GU, SEOUL, 00000, SOUTH KOREA
SN 2636-0004
EI 2636-0012
J9 INT J GASTROINT INT
JI Int. J. Gastrointest. Interv.
PD JUL
PY 2022
VL 11
IS 3
BP 126
EP 131

DI 10.18528/ijgii220026
PG 6
WC Gastroenterology & Hepatology
WE Emerging Sources Citation Index (ESCI)
SC Gastroenterology & Hepatology
GA 3L8CE
UT WOS:000834989400007
OA Green Submitted, gold
DA 2026-05-09
ER

PT J
AU Peñas-Herrero, I
de la Serna-Higuera, C
Perez-Miranda, M
AF Penas-Herrero, Irene
de la Serna-Higuera, Carlos
Perez-Miranda, Manuel
TI Endoscopic ultrasound-guided gallbladder drainage for the management of
acute cholecystitis (with video)
SO JOURNAL OF HEPATO-BILIARY-PANCREATIC SCIENCES
LA English
DT Article
DE Acute cholecystitis; Endoscopic drainage; Endoscopic ultrasound-guided
drainage; Endoscopic ultrasound-guided gallbladder drainage;
Lumen-apposing metal stents; Metal stent; Nasobiliary drainage;
Self-expandable metal stents
ID SINGLE-STEP PLACEMENT; APPOSING METAL STENT; PERCUTANEOUS
CHOLECYSTOSTOMY; PANCREATIC-CANCER; CHOLECYSTOGASTROSTOMY;
CHOLECYSTECTOMY; FEASIBILITY; ANASTOMOSIS
AB Endoscopic ultrasound-guided gallbladder drainage (EUS-GBD) has been introduced as an alternative to percutaneous transhepatic gallbladder drainage for the treatment of acute cholecystitis in non-surgical candidates. A systematic review of the English language literature through PubMed search until June 2014 was conducted. One hundred and fifty-five patients with acute cholecystitis treated with EUS-GBD in eight studies and 12 case reports, and two patients with EUS-GBD for other causes were identified. Overall, technical success was obtained in 153 patients (97.45%) and clinical success in 150 (99.34%) patients with acute cholecystitis. Adverse events developed in less than 8% of patients, all of them managed conservatively. EUS-GBD has been performed with plastic stents, nasobiliary drainage tubes, standard or modified tubular self-expandable metal stents (SEMS) and lumen-apposing metal stents (LAMS) by different authors with apparently similar outcomes. No comparison studies between stent types for EUS-GBD have been reported. EUS-GBD is a promising novel alternative intervention for the treatment of acute cholecystitis in high surgical risk patients. Feasibility, safety and efficacy in published studies from expert centers are very high compared to currently available alternatives. Further studies are needed to establish the safety and long-term outcomes of this procedure in other practice settings before EUS-GBD can be

widely disseminated.

C1 [Penas-Herrero, Irene; de la Serna-Higuera, Carlos; Perez-Miranda, Manuel] Hosp Univ Rio Hortega, Dept Gastroenterol & Hepatol, Valladolid 47012, Spain.
C3 Hospital del Rio Hortega
RP Perez-Miranda, M (corresponding author), Hosp Univ Rio Hortega, Dept Gastroenterol & Hepatol, Dulzaina 2, Valladolid 47012, Spain.
EM mperezmiranda@saludcastillayleon.es
OI Peñas Herrero, Irene/0009-0007-9986-8107

CR Akhan O, 2002, EUR J RADIOL, V43, P229, DOI 10.1016/S0720-048X(02)00158-4
Baron TH, 2007, GASTROINTEST ENDOSC, V65, P735, DOI 10.1016/j.gie.2006.07.041
Binmoeller KF, 2011, ENDOSCOPY, V43, P337, DOI 10.1055/s-0030-1256127
Súbtil JC, 2010, WORLD J GASTRO ENDOS, V2, P203, DOI 10.4253/wjge.v2.i6.203
Chang L, 2000, AM J SURG, V180, P198, DOI 10.1016/S0002-9610(00)00476-1
Choi JH, 2014, ENDOSCOPY, V46, P656, DOI 10.1055/s-0034-1365720
Davis CA, 1999, ARCH SURG-CHICAGO, V134, P727, DOI 10.1001/archsurg.134.7.727
de la Serna-Higuera C, 2013, GASTROINTEST ENDOSC, V77, P303, DOI 10.1016/j.gie.2012.09.021
Hasan Muhammad K, 2013, Gastrointest Endosc Clin N Am, V23, P453, DOI 10.1016/j.giec.2012.12.010
Hatzidakis AA, 2002, EUR RADIOL, V12, P1778, DOI 10.1007/s00330-001-1247-4
Hikichi Takuto, 2007, Fukushima J Med Sci, V53, P11
Ito K, 2004, AM J ROENTGENOL, V183, P193, DOI 10.2214/ajr.183.1.1830193
Itoi T, 2013, DIGEST ENDOSC, V25, P137, DOI 10.1111/den.12084
Itoi T, 2012, GASTROINTEST ENDOSC, V75, P870, DOI 10.1016/j.gie.2011.10.020
Itoi T, 2011, J HEPATO-BIL-PAN SCI, V18, P282, DOI 10.1007/s00534-010-0310-4
Itoi T, 2010, GASTROINTEST ENDOSC, V71, P1038, DOI 10.1016/j.gie.2010.01.026
Jang JW, 2012, GASTROENTEROLOGY, V142, P805, DOI 10.1053/j.gastro.2011.12.051
Jang JW, 2011, GASTROINTEST ENDOSC, V74, P176, DOI 10.1016/j.gie.2011.03.1120
Kamata K, 2009, ENDOSCOPY, V41, pE315, DOI 10.1055/s-0029-1215258
Kedia P, 2015, GASTROINTEST ENDOSC, V81, P228, DOI 10.1016/j.gie.2014.04.054
Kiviniemi H, 1998, INT SURG, V83, P299
Kwan V, 2007, GASTROINTEST ENDOSC, V66, P582, DOI 10.1016/j.gie.2007.02.065
LEE MJ, 1991, AM J ROENTGENOL, V156, P1163, DOI 10.2214/ajr.156.6.2028859
Lee SS, 2007, GASTROINTEST ENDOSC, V66, P1008, DOI 10.1016/j.gie.2007.03.1080
LO LD, 1995, J VASC INTERV RADIOL, V6, P629, DOI 10.1016/S1051-0443(95)71150-2
MCGAHAN JP, 1989, RADIOLOGY, V173, P481, DOI 10.1148/radiology.173.2.2678261
Miura F, 2013, J HEPATO-BIL-PAN SCI, V20, P47, DOI 10.1007/s00534-012-0563-1
Mönkemüller K, 2013, ENDOSCOPY, V45, pE164, DOI 10.1055/s-0032-1325776
Moon JH, 2014, GASTROINTEST ENDOSC, V79, P990, DOI 10.1016/j.gie.2014.02.015
Ogura T, 2014, ENDOSCOPY, V46, pE75, DOI 10.1055/s-0033-1359135
Park PO, 2005, GASTROINTEST ENDOSC, V61, P601, DOI 10.1016/S0016-5107(04)02774-9
Perez-Miranda M, 2010, WORLD J GASTRO ENDOS, V2, P212, DOI 10.4253/wjge.v2.i6.212
Song TJ, 2010, GASTROINTEST ENDOSC, V71, P634, DOI 10.1016/j.gie.2009.11.024
Sugiyama M, 1998, WORLD J SURG, V22, P459, DOI 10.1007/s002689900416

Takasawa O, 2009, DIGEST ENDOSC, V21, P43, DOI 10.1111/j.1443-1661.2008.00822.x
Talreja JP, 2008, GASTROINTEST ENDOSC, V68, P1199, DOI 10.1016/j.gie.2008.06.015
Teoh AYB, 2014, ENDOSCOPY, V46, pE13, DOI 10.1055/s-0033-1359139
Teoh AYB, 2014, GASTROINTEST ENDOSC, V80, P1171, DOI 10.1016/j.gie.2014.03.038
Tsuyuguchi T, 2013, J HEPATO-BIL-PAN SCI, V20, P81, DOI 10.1007/s00534-012-0570-2
Widmer J, 2014, DIGEST ENDOSC, V26, P525, DOI 10.1111/den.12221
Widmer J, 2014, DIGEST ENDOSC, V26, P599, DOI 10.1111/den.12163
NR 41
TC 51
Z9 67
U1 0
U2 10
PU WILEY
PI HOBOKEN
PA 111 RIVER ST, HOBOKEN 07030-5774, NJ USA
SN 1868-6974
EI 1868-6982
J9 J HEPATO-BIL-PAN SCI
JI J. Hepato-Biliary-Pancreat. Sci.
PD JAN
PY 2015
VL 22
IS 1
BP 35
EP 43
DI 10.1002/jhbp.182
PG 9
WC Gastroenterology & Hepatology; Surgery
WE Science Citation Index Expanded (SCI-EXPANDED)
SC Gastroenterology & Hepatology; Surgery
GA AW9MS
UT WOS:000346582200009
PM 25392972
OA Bronze
DA 2026-05-09
ER

PT J
AU Manta, R
Mutignani, M
Galloro, G
Conigliaro, R

Zullo, A
AF Manta, Raffaele
Mutignani, Massimiliano
Galloro, Giuseppe
Conigliaro, Rita
Zullo, Angelo
TI Endoscopic ultrasound-guided gallbladder drainage for acute cholecystitis with a lumen-apposing metal stent: a systematic review of case series
SO EUROPEAN JOURNAL OF GASTROENTEROLOGY & HEPATOLOGY
LA English
DT Review
DE cholecystitis; drainage; endoscopic ultrasound; lumen-apposing metal stent
ID PERCUTANEOUS CHOLECYSTOSTOMY; VIDEOS; CHOLECYSTECTOMY; MANAGEMENT
AB Management of patients with acute cholecystitis unfit for surgery is challenging. Recently, endoscopic ultrasound (EUS)-guided gallbladder drainage with a lumen-apposing metal stent (LAMS) has been introduced for these patients. We performed a systematic review and pooled-data analysis in this field. A comprehensive review of case series on gallbladder drainage with EUS-guided LAMS placement was performed. Only case series with at least five patients were considered. The rates of technical success, clinical success, and adverse events were computed. Overall, nine case series with a total of 226 patients were identified. The stent was positioned successfully in 215 cases [95.1%, 95% confidence interval (CI)=92.3-98]. Clinical success was achieved in 207 patients, corresponding to a 91.6% (95% CI=88-95.2) rate at intention-to-treat analysis and 96.3% (95% CI=93.7-99) at per-protocol analysis. A total of 24 (10.6%) adverse events occurred, including 11 (4.9%) cases during the procedure, and 13 (5.7%) observed at follow-up (median=6 months; range: 2-12 months). A surgical approach was required in only 25% of patients with a major adverse event. No case of procedure-related death was reported. EUS-guided LAMS placement for gallbladder drainage in patients with acute cholecystitis not suitable for surgery is highly successful and acceptably safe.
C1 [Manta, Raffaele; Conigliaro, Rita] New St Agostino Estense Civil Hosp, Gastroenterol Unit, Modena, Italy.
[Mutignani, Massimiliano] Osped Niguarda Ca Granda, Dept Diagnost & Intervent Digest Endoscopy, Milan, Italy.
[Galloro, Giuseppe] Federico II Univ Naples, Unit Surg Digest Endoscopy, Dept Clin Med & Surg, Naples, Italy.
[Zullo, Angelo] Nuovo Regina Margherita Hosp, Dept Gastroenterol & Digest Endoscopy, Via Emilio Morosini 30, I-00153 Rome, Italy.
C3 IRCCS Ca Granda Ospedale Maggiore Policlinico; Ospedale Niguarda Ca' Granda; University of Naples Federico II; Poliambulatorio Nuovo Regina Margherita
RP Zullo, A (corresponding author), Nuovo Regina Margherita Hosp, Dept Gastroenterol & Digest Endoscopy, Via Emilio Morosini 30, I-00153 Rome, Italy.
EM angelozullo66@yahoo.it
RI Zullo, Angelo/AAV-7092-2020
CR Anderloni A, 2016, SURG ENDOSC, V30, P5200, DOI 10.1007/s00464-016-4894-x
Bakkaloglu H, 2006, WORLD J GASTROENTERO, V12, P7179, DOI 10.3748/wjg.v12.i44.7179
Baron TH, 2015, GASTROINTEST ENDOSC, V82, P1037, DOI 10.1016/j.gie.2015.04.016

Coccolini F, 2015, INT J SURG, V18, P196, DOI 10.1016/j.ijsu.2015.04.083
de la Serna-Higuera C, 2013, GASTROINTEST ENDOSC, V77, P303, DOI 10.1016/j.gie.2012.09.021
Dollhopf M, 2017, GASTROINTEST ENDOSC, V86, P636, DOI 10.1016/j.gie.2017.02.027
Fabbri C, 2014, WORLD J GASTROENTERO, V20, P8424, DOI 10.3748/wjg.v20.i26.8424
Irani S, 2015, GASTROINTEST ENDOSC, V82, P1110, DOI 10.1016/j.gie.2015.05.045
Itoi T, 2012, GASTROINTEST ENDOSC, V75, P870, DOI 10.1016/j.gie.2011.10.020
Kahaleh M, 2016, DIGEST LIVER DIS, V48, P1054, DOI 10.1016/j.dld.2016.05.021
Kedia P, 2015, GASTROINTEST ENDOSC, V82, P1031, DOI 10.1016/j.gie.2015.03.1912
Khan MA, 2017, GASTROINTEST ENDOSC, V85, P76, DOI 10.1016/j.gie.2016.06.032
Kwan V, 2007, GASTROINTEST ENDOSC, V66, P582, DOI 10.1016/j.gie.2007.02.065
Law R, 2016, CLIN GASTROENTEROL H, V14, P476, DOI 10.1016/j.cgh.2015.10.026
Manta R, 2017, ENDOSC INT OPEN, V5, pE1111, DOI 10.1055/s-0043-118659
Moon JH, 2014, GASTROINTEST ENDOSC, V79, P990, DOI 10.1016/j.gie.2014.02.015
Mutignani M, 2009, ENDOSCOPY, V41, P539, DOI 10.1055/s-0029-1214727
Patil R, 2016, ANN GASTROENTEROL, V29, P162, DOI 10.20524/aog.2016.0001
Peñas-Herrero I, 2015, J HEPATO-BIL-PAN SCI, V22, P35, DOI 10.1002/jhbp.182
Teoh AYB, 2017, ENDOSCOPY, V49, P130, DOI 10.1055/s-0042-119036
Venara A, 2014, J VISC SURG, V151, P435, DOI 10.1016/j.jviscsurg.2014.06.003
Walter D, 2016, GUT, V65, P6, DOI 10.1136/gutjnl-2015-309925
Widmer J, 2014, DIGEST ENDOSC, V26, P525, DOI 10.1111/den.12221
NR 23
TC 28
Z9 32
U1 0
U2 5
PU LIPPINCOTT WILLIAMS & WILKINS
PI PHILADELPHIA
PA TWO COMMERCE SQ, 2001 MARKET ST, PHILADELPHIA, PA 19103 USA
SN 0954-691X
EI 1473-5687
J9 EUR J GASTROEN HEPAT
JI Eur. J. Gastroenterol. Hepatol.
PD JUL
PY 2018
VL 30
IS 7
BP 695
EP 698
DI 10.1097/MEG.0000000000001112
PG 4
WC Gastroenterology & Hepatology
WE Science Citation Index Expanded (SCI-EXPANDED)

SC Gastroenterology & Hepatology
GA GI5PV
UT WOS:000434424000001
PM 29578866
DA 2026-05-09
ER

PT J
AU Maulahela, H
Annisa, NG
Fauzi, A
Renaldi, K
Abdullah, M
Simadibrata, M
Makmun, D
Syam, AF
AF Maulahela, Hasan
Annisa, Nagita Gianty
Fauzi, Achmad
Renaldi, Kaka
Abdullah, Murdani
Simadibrata, Marcellus
Makmun, Dadang
Syam, Ari Fahrial
TI Role of interventional endoscopic ultrasound in a developing country
SO CLINICAL ENDOSCOPY
LA English
DT Article
DE Drainage; Endoscopic ultrasound-guided fine needle aspiration;
Endosonography; Gastrointestinal endosocopy
ID PANCREATIC FLUID COLLECTIONS; GUIDED BILIARY DRAINAGE; PERCUTANEOUS
DRAINAGE
AB Background/Aims: Endoscopic ultrasound (EUS) has become an essential diagnostic and therapeutic tool. EUS was introduced in 2013 in Indonesia and is considered relatively new. This study aimed to describe the current role of interventional EUS at our hospital as a part of the Indonesian tertiary health center experience.

Methods: This retrospective study included all patients who underwent interventional EUS (n=94) at our center between January 2015 and December 2020. Patient characteristics, technical success, clinical success, and adverse events associated with each type of interventional EUS procedure were evaluated.

Results: Altogether, 94 interventional EUS procedures were performed at our center between 2015 and 2020 including 75 cases of EUS-guided biliary drainage (EUS-BD), 14 cases of EUS-guided pancreatic fluid drainage, and 5 cases of EUS-guided celiac plexus neurolysis. The technical and clinical success rates of EUS-BD were 98.6% and 52%, respectively. The technical success rate was 100% for both EUS-guided pancreatic fluid drainage and EUS-guided celiac plexus neurolysis.

The adverse event rates were 10.6% and 7.1% for EUS-BD and EUS-guided pancreatic fluid drainage, respectively.

Conclusions: EUS is an effective and safe tool for the treatment of gastrointestinal and biliary diseases. It has a low rate of adverse events, even in developing countries.
C1 [Maulahela, Hasan; Fauzi, Achmad; Renaldi, Kaka; Abdullah, Murdani; Simadibrata, Marcellus; Makmun, Dadang; Syam, Ari Fahrial] Univ Indonesia, Div Gastroenterol, Dept Internal Med, Fac Med,Cipto Mangunkusumo Natl Gen Hosp, Jl Pangeran Diponegoro 71,RW-5, Jakarta 10430, Indonesia.

[Annisa, Nagita Gianty] Univ Indonesia, Dept Internal Med, Fac Med, Jakarta, Indonesia.

C3 University of Indonesia; University of Indonesia

RP Maulahela, H (corresponding author), Univ Indonesia, Div Gastroenterol, Dept Internal Med, Fac Med,Cipto Mangunkusumo Natl Gen Hosp, Jl Pangeran Diponegoro 71,RW-5, Jakarta 10430, Indonesia.

EM hasan.maulahela@yahoo.com

RI Syam, Ari Fahrial/ABI-4994-2020; Renaldi, Kaka/KIC-1270-2024; Fauzi, Achmad/LFV-7269-2024

OI Syam, Ari Fahrial/0000-0003-0041-3553; maulahela, hasan/0000-0002-0396-4433; Renaldi, Kaka/0000-0002-1092-9644; Makmun, Dadang/0000-0002-9055-8678;
CR Baars JE, 2018, ENDOSC ULTRASOUND, V7, P4, DOI 10.4103/eus.eus_105_17

Friedberg SR, 2017, WORLD J GASTRO ENDOS, V9, P499, DOI 10.4253/wjge.v9.i10.499

Hao SJ, 2017, WORLD J GASTRO ENDOS, V9, P486, DOI 10.4253/wjge.v9.i9.486

Kanno Y, 2019, J GASTROINTEST CANC, V50, P469, DOI 10.1007/s12029-018-0096-1

Kato Shin, 2013, Diagn Ther Endosc, V2013, P924291, DOI 10.1155/2013/924291

Kaufman M, 2010, J CLIN GASTROENTEROL, V44, P127, DOI 10.1097/MCG.0b013e3181bb854d

Kawakubo K, 2014, J HEPATO-BIL-PAN SCI, V21, P328, DOI 10.1002/jhbp.27

Keane MG, 2016, SURG ENDOSC, V30, P3730, DOI 10.1007/s00464-015-4668-x

Kumta NA, 2019, DIGEST LIVER DIS, V51, P1557, DOI 10.1016/j.dld.2019.05.033

Makmun Dadang, 2017, Diagn Ther Endosc, V2017, P4856276, DOI [10.1155/2017/4856276, 10.1155/2017/4856276]

Paik WH, 2018, AM J GASTROENTEROL, V113, P987, DOI 10.1038/s41395-018-0122-8

Park SW, 2020, J CLIN MED, V9, DOI 10.3390/jcm9113595

Puli SR, 2009, DIGEST DIS SCI, V54, P2330, DOI 10.1007/s10620-008-0651-x

Ramírez-Luna MA, 2011, ENDOSCOPY, V43, P826, DOI 10.1055/s-0030-1256406

Saumoy M, 2018, CLIN ENDOSC, V51, P235, DOI 10.5946/ce.2017.081

Sharma V, 2015, WORLD J GASTRO ENDOS, V7, P628, DOI 10.4253/wjge.v7.i6.628

Xie LT, 2019, MED SCI MONITOR, V25, P5785, DOI 10.12659/MSM.915193
NR 17

TC 1

Z9 1

U1 0

U2 0

PU KOREAN SOC GASTROINTESTINAL ENDOSCOPY

PI SEOUL

PA 2003 LG PALACE, 165-8 DONGGYO-DONG, MAPO-GU, SEOUL, 121-754, SOUTH KOREA

SN 2234-2400

EI 2234-2443
J9 CLIN ENDOSC
JI Clin. Endosc.
PD JAN
PY 2023
VL 56
IS 1
BP 100
EP 106
DI 10.5946/ce.2022.058
PG 7
WC Gastroenterology & Hepatology
WE Emerging Sources Citation Index (ESCI)
SC Gastroenterology & Hepatology
GA FU8W0
UT WOS:001148469300009
PM 36646497
OA Green Submitted, gold
DA 2026-05-09
ER

PT J
AU Kamata, K
Kinoshita, M
Kinoshita, I
Imai, H
Ogura, T
Matsumoto, H
Minaga, K
Chiba, Y
Takenaka, M
Kudo, M
Kitano, M
AF Kamata, Ken
Kinoshita, Makiko
Kinoshita, Ikuharu
Imai, Hajime
Ogura, Takeshi
Matsumoto, Hisakazu
Minaga, Kosuke
Chiba, Yasutaka
Takenaka, Mamoru

Kudo, Masatoshi
Kitano, Masayuki
TI Efficacy of EUS-guided celiac plexus neurolysis in combination with
EUS-guided celiac ganglia neurolysis for pancreatic cancer-associated
pain: a multicenter prospective trial
SO INTERNATIONAL JOURNAL OF CLINICAL ONCOLOGY
LA English
DT Article
DE Cancer-associated pain; Celiac ganglia neurolysis; Celiac plexus
neurolysis; EUS; Pancreatic cancer
ID ABDOMINAL-PAIN; INJECTION; BLOCK
AB Objectives This study evaluated the efficacy of endoscopic ultrasound-guided celiac plexus neurolysis (EUS-CPN) in combination with EUS-guided celiac ganglia neurolysis (EUS-CGN) for pancreatic cancer-associated pain. Methods This multicenter prospective trial was registered in the University Hospital Medical Information Network (UMIN000031228). Fifty-one consecutive patients with pancreatic cancer-associated pain who presented at one of five Japanese referral centers between February 2018 and March 2021 were enrolled. EUS-CGN was added in cases of visible celiac ganglia. The primary endpoint was effectiveness, defined as a decrease in the numerical rating scale (NRS) by >= 3 points. NRS data were prospectively acquired at 1 week after the procedure to evaluate its effectiveness and the extent of pain relief. Results The technical success rates of EUS-CPN and EUS-CGN were 100% and 80.4%, respectively. The overall efficacy rate was 82.4% [90% confidence interval (CI) 71.2-90.5, P < 0.0001]. The complete pain relief rate was 27.4%. The adverse events rate was 15.7%. The average pain relief period was 72 days. The efficacy rate was higher in the EUS-CPN plus EUS-CGN group than in the EUS-CPN alone group. EUS-CPN plus EUS-CGN was superior to EUS-CPN alone for achieving complete pain relief (P = 0.045). EUS-CGN did not improve the average length of the pain relief period. Conclusions EUS-CPN combined with EUS-CGN is safe, feasible, and effective for pain relief in patients with pancreatic cancer. The patients who received additional EUS-CGN had a better short-term response.
C1 [Kamata, Ken; Takenaka, Mamoru; Kudo, Masatoshi] Kindai Univ, Dept Gastroenterol & Hepatol, Fac Med, Osaka, Japan.
[Kinoshita, Makiko; Kinoshita, Ikuharu] Natl Hosp Org Minami Wakayama Med Ctr, Dept Gastroenterol, Wakayama, Japan.
[Imai, Hajime] Okanami Gen Hosp, Dept Gastroenterol, Iga, Mie, Japan.
[Ogura, Takeshi] Osaka Med Coll, Dept Internal Med 2, Osaka, Japan.
[Matsumoto, Hisakazu; Minaga, Kosuke] Japanese Red Cross Wakayama Med Ctr, Dept Gastroenterol, Wakayama, Japan.
[Chiba, Yasutaka] Kindai Univ Hosp, Clin Res Ctr, Osaka, Japan.
[Kitano, Masayuki] Wakayama Med Univ, Dept Internal Med 2, Sch Med, Wakayama, Japan.
C3 Kindai University (Kinki University); Osaka Medical & Pharmaceutical
University; Wakayama Medical University
RP Kamata, K (corresponding author), Kindai Univ, Dept Gastroenterol & Hepatol, Fac Med, Osaka, Japan.
EM ky11@leto.eonet.ne.jp
RI Kudo, Masatoshi/AAA-9744-2019; Ogura, Takeshi/AAU-5643-2021; Minaga,
Kosuke/AAD-2107-2020
CR Ascunce G, 2011, GASTROINTEST ENDOSC, V73, P267, DOI 10.1016/j.gie.2010.10.029

Doi S, 2013, ENDOSCOPY, V45, P362, DOI 10.1055/s-0032-1326225
EISENBERG E, 1995, ANESTH ANALG, V80, P290, DOI 10.1097/00000539-199502000-00015
Faigel DO, 1996, AM J GASTROENTEROL, V91, P1675
Gunaratnam NT, 2001, GASTROINTEST ENDOSC, V54, P316, DOI 10.1067/mge.2001.117515
Iwata K, 2011, DIGEST ENDOSC, V23, P140, DOI 10.1111/j.1443-1661.2010.01046.x
Kanno Y, 2020, GASTROINTEST ENDOSC, V92, P120, DOI 10.1016/j.gie.2020.01.011
Kappelle WFW, 2017, GASTROINTEST ENDOSC, V86, P655, DOI 10.1016/j.gie.2017.01.041
Kappis M, 1914, Verh. Dtsch. Ges. Chir, V43, P87
Leblanc JK., 2013, DIAGN THER ENDOSC, V2013, DOI 10.1155/2013/327036
LeBlanc JK, 2011, GASTROINTEST ENDOSC, V74, P1300, DOI 10.1016/j.gie.2011.07.073
Levy M, 2006, AM J GASTROENTEROL, V101, P1787, DOI 10.1111/j.1572-0241.2006.00685.x
Levy MJ, 2019, CLIN GASTROENTEROL H, V17, P728, DOI 10.1016/j.cgh.2018.08.040
Minaga K, 2018, CANCERS, V10, DOI 10.3390/cancers10020050
Minaga K, 2016, THER ADV GASTROENTER, V9, P483, DOI 10.1177/1756283X16644248
Sahai AV, 2009, AM J GASTROENTEROL, V104, P326, DOI 10.1038/ajg.2008.64
Sakamoto H., 2006, DIGEST ENDOSC, V18, P206
Sakamoto H, 2010, AM J GASTROENTEROL, V105, P2599, DOI 10.1038/ajg.2010.339
Téllez-Avila FI, 2013, ENDOSC ULTRASOUND, V2, P153, DOI [10.4103/2303-9027.117664, 10.7178/eus.06.007]
Wiersema MJ, 1996, GASTROINTEST ENDOSC, V44, P656, DOI 10.1016/S0016-5107(96)70047-0
Wyse JM, 2011, J CLIN ONCOL, V29, P3541, DOI 10.1200/JCO.2010.32.2750
NR 21
TC 9
Z9 13
U1 0
U2 4
PU SPRINGER JAPAN KK
PI TOKYO
PA SHIROYAMA TRUST TOWER 5F, 4-3-1 TORANOMON, MINATO-KU, TOKYO, 105-6005,
JAPAN
SN 1341-9625
EI 1437-7772
J9 INT J CLIN ONCOL
JI Int. J. Clin. Oncol.
PD JUL
PY 2022
VL 27
IS 7
SI SI
BP 1196
EP 1201
DI 10.1007/s10147-022-02160-6
EA APR 2022

PG 6
WC Oncology
WE Science Citation Index Expanded (SCI-EXPANDED)
SC Oncology
GA 2G8IU
UT WOS:000781699100002
PM 35412211
DA 2026-05-09
ER

PT J
AU van der Merwe, SW
van Wanrooij, RLJ
Bronswijk, M
Everett, S
Lakhtakia, S
Rimbas, M
Hucl, T
Kunda, R
Badaoui, A
Law, R
Arcidiacono, PG
Larghi, A
Giovannini, M
Khashab, MA
Binmoeller, KF
Barthet, M
Perez-Miranda, M
van Hooft, JE
AF van der Merwe, Schalk W.
van Wanrooij, Roy L. J.
Bronswijk, Michiel
Everett, Simon
Lakhtakia, Sundeep
Rimbas, Mihai
Hucl, Tomas
Kunda, Rastislav
Badaoui, Abdenor
Law, Ryan
Arcidiacono, Paolo G.
Larghi, Alberto
Giovannini, Marc

Khashab, Mouen A.
Binmoeller, Kenneth F.
Barthet, Marc
Perez-Miranda, Manuel
van Hooft, Jeanin E.
TI Therapeutic endoscopic ultrasound: European Society of Gastrointestinal Endoscopy (ESGE) Guideline
SO ENDOSCOPY
LA English
DT Article
ID MALIGNANT BILIARY OBSTRUCTION; PANCREATIC-DUCT DRAINAGE; LONG-TERM OUTCOMES; COVERED METAL STENT; MULTICENTER COMPARATIVE TRIAL; GASTRIC OUTLET OBSTRUCTION; GALLBLADDER DRAINAGE; INTERNATIONAL MULTICENTER; FAILED ERCP; RETROGRADE CHOLANGIOPANCREATOGRAPHY
AB Main Recommendations 1 ESGE recommends the use of endoscopic ultrasound-guided biliary drainage (EUS-BD) over percutaneous transhepatic biliary drainage (PTBD) after failed endoscopic retrograde cholangiopancreatography (ERCP) in malignant distal biliary obstruction when local expertise is available. Strong recommendation, moderate quality evidence. 2 ESGE suggests EUS-BD with hepaticogastrostomy only for malignant inoperable hilar biliary obstruction with a dilated left hepatic duct when inadequately drained by ERCP and/or PTBD in high volume expert centers. Weak recommendation, moderate quality evidence. 3 ESGE recommends that EUS-guided pancreatic duct (PD) drainage should only be considered in symptomatic patients with an obstructed PD when retrograde endoscopic intervention fails or is not possible. Strong recommendation, low quality evidence. 4 ESGE recommends rendezvous EUS techniques over transmural PD drainage in patients with favorable anatomy owing to its lower rate of adverse events. Strong recommendation, low quality evidence. 5 ESGE recommends that, in patients at high surgical risk, EUS-guided gallbladder drainage (GBD) should be favored over percutaneous gallbladder drainage where both techniques are available, owing to the lower rates of adverse events and need for re-interventions in EUS-GBD. Strong recommendation, high quality of evidence. 6 ESGE recommends EUS-guided gastroenterostomy (EUS-GE), in an expert setting, for malignant gastric outlet obstruction, as an alternative to enteral stenting or surgery. Strong recommendation, low quality evidence. 7 ESGE recommends that EUS-GE may be considered in the management of afferent loop syndrome, especially in the setting of malignancy or in poor surgical candidates. Strong recommendation, low quality evidence. 8 ESGE suggests that endoscopic ultrasound-directed transgastric ERCP (EDGE) can be offered, in expert centers, to patients with a Roux-en-Y gastric bypass following multidisciplinary decision-making, with the aim of overcoming the invasiveness of laparoscopy-assisted ERCP and the limitations of enteroscopy-assisted ERCP. Weak recommendation, low quality evidence.
C1 [van der Merwe, Schalk W.; Bronswijk, Michiel] Univ Hosp Leuven, Dept Gastroenterol & Hepatol, Leuven, Belgium.
[van Wanrooij, Roy L. J.] Vrije Univ Amsterdam, Amsterdam UMC, AGEM Inst, Dept Gastroenterol & Hepatol, Amsterdam, Netherlands.
[Bronswijk, Michiel] Imelda Gen Hosp, Dept Gastroenterol & Hepatol, Bonheiden, Belgium.
[Everett, Simon] Leeds Teaching Hosp NHS Trust, Dept Gastroenterol & Hepatol, Leeds, W Yorkshire, England.
[Lakhtakia, Sundeep] Asian Inst Gastroenterol Hosp, Dept Med Gastroenterol, Hyderabad, India.
[Rimbas, Mihai] Colentina Clin Hosp, Dept Gastroenterol, Bucharest, Romania.
[Hucl, Tomas] Inst Clin & Expt Med, Dept Gastroenterol & Hepatol, Prague, Czech Republic.
[Kunda, Rastislav] Vrije Univ Brussel, Univ Ziekenhuis Brussel, Dept Surg, Dept Gastroenterol & Hepatol, Brussels,

Belgium.
[Kunda, Rastislav] Vrije Univ Brussel, Univ Ziekenhuis Brussel, Dept Adv Intervent Endoscopy, Brussels, Belgium.
[Badaoui, Abdenor] Univ Catholic Louvain, CHU UCL Namur, Dept Gastroenterol & Hepatol, Yvoir, Belgium.
[Law, Ryan] Mayo Clin, Dept Gastroenterol & Hepatol, Rochester, MN 55905 USA.
[Arcidiacono, Paolo G.] IRCCS San Raffaele Sci Inst, Pancreatobiliary Endoscopy & Endosonog Div, Milan, Italy.
[Arcidiacono, Paolo G.] Univ Cattolica Sacro Cuore, Fdn Policlin Univ A Gemelli IRCCS, Digest Endoscopy Unit, Rome, Italy.
[Larghi, Alberto] Catholic Univ, Ctr Endoscop Res Therapeut & Training CERTT, Rome, Italy.
[Giovannini, Marc] Paoli Calmettes Inst, Dept Gastrointestinal Dis, Marseille, France.
[Khashab, Mouen A.] Johns Hopkins Univ Hosp, Div Gastroenterol & Hepatol, Baltimore, MD 21287 USA.
[Binmoeller, Kenneth F.] Calif Pacific Med Ctr, Intervent Endoscopy Serv, San Francisco, CA USA.
[Barthet, Marc] Aix Marseille Univ, Hop Nord, AP HP, Dept Gastroenterol, Marseille, France.
[Perez-Miranda, Manuel] Hosp Univ Rio Hortega, Dept Gastroenterol, Valladolid, Spain.
[van Hooft, Jeanin E.] Leiden Univ, Med Ctr, Dept Gastroenterol & Hepatol, Leiden, Netherlands.
C3 KU Leuven; University Hospital Leuven; University of Amsterdam; Vrije Universiteit Amsterdam; Imeldaziekenhuis; University of Leeds; Institute for Clinical & Experimental Medicine (IKEM); University Hospital Brussels; Vrije Universiteit Brussel; University Hospital Brussels; Vrije Universiteit Brussel; Universite Catholique Louvain; Mayo Clinic; Vita-Salute San Raffaele University; IRCCS Ospedale San Raffaele; Catholic University of the Sacred Heart; IRCCS Policlinico Gemelli; Catholic University of the Sacred Heart; IRCCS Policlinico Gemelli; UNICANCER; Institut Paoli-Calmette (IPC); Johns Hopkins University; Johns Hopkins Medicine; California Pacific Medical Center; Aix-Marseille Universite; Assistance Publique-Hopitaux de Marseille; Assistance Publique Hopitaux Paris (APHP); Hospital del Rio Hortega; Leiden University; Leiden University Medical Center (LUMC); Leiden University - Excl LUMC
RP van der Merwe, SW (corresponding author), Univ Hosp Leuven, Dept Gastroenterol & Hepatol, Leuven, Belgium.
EM Schalk.vandermerwe@uzleuven.be
RI ; van Hooft, Jeanin/AAT-3600-2020; Arcidiacono, Paolo Giorgio/K-2122-2018; Larghi, Alberto/AAC-7448-2022; Lakhtakia, Sundeep/H-3607-2019; Rimbas, Mihai/AAF-5521-2020; Kunda, Rastislav/N-1775-2019; khashab, mouen/F-6643-2017
OI Bronswijk, Michiel/0000-0003-2039-5022; van Wanrooij, Roy L.J/0000-0003-0477-4532; Badaoui, Abdenor/0009-0002-1202-7967; Van der Merwe, Schalk/0000-0002-9891-2686; van Hooft, Jeanin/0000-0002-4424-0079; Arcidiacono, Paolo Giorgio/0000-0001-6692-7720; Larghi, Alberto/0000-0001-7131-3686;
CR Ahmed O, 2018, SURG ENDOSC, V32, P1627, DOI 10.1007/s00464-018-6041-3
Ali UA, 2015, COCHRANE DB SYST REV, DOI 10.1002/14651858.CD007884.pub3
Amano M, 2017, J GASTROEN HEPATOL, V32, P716, DOI 10.1111/jgh.13489

Anderloni A, 2019, ENDOSCOPY, V51, pE239, DOI 10.1055/a-0889-7051
Anderloni A, 2016, SURG ENDOSC, V30, P5200, DOI 10.1007/s00464-016-4894-x
Antonelli G, 2020, DIGEST LIVER DIS, V52, P1294, DOI 10.1016/j.dld.2020.04.021
Artifon ELA, 2015, GASTROINTEST ENDOSC, V81, P950, DOI 10.1016/j.gie.2014.09.047
Artifon ELA, 2012, J CLIN GASTROENTEROL, V46, P768, DOI 10.1097/MCG.0b013e31825f264c
Arvanitakis M, 2018, ENDOSCOPY, V50, P524, DOI 10.1055/a-0588-5365
Attam R, 2015, ENDOSCOPY, V47, P647, DOI 10.1055/s-0034-1391124
Bang JY, 2018, GASTROINTEST ENDOSC, V88, P9, DOI 10.1016/j.gie.2018.03.012
Bapaye A, 2013, UNITED EUR GASTROENT, V1, P285, DOI 10.1177/2050640613490928
Basiliya K, 2021, ENDOSCOPY, V53, P266, DOI 10.1055/a-1200-0199
Bill JG, 2022, SURG ENDOSC, V36, P1362, DOI 10.1007/s00464-021-08418-w
Brauer BC, 2009, GASTROINTEST ENDOSC, V70, P471, DOI 10.1016/j.gie.2008.12.233
Bronswijk M, 2021, GASTROINTEST ENDOSC, V94, P526, DOI 10.1016/j.gie.2021.04.006
Bronswijk Michiel, 2020, VideoGIE, V5, P442, DOI [10.1016/j.vgie.2020.06.012, 10.1016/j.vgie.2020.06.012]
Bronswijk M, 2021, ENDOSCOPY, V53, P204, DOI 10.1055/a-1190-3228
Bukhari M, 2018, GASTROINTEST ENDOSC, V88, P486, DOI 10.1016/j.gie.2018.04.2356
Chahal P, 2006, ENDOSCOPY, V38, P1241, DOI 10.1055/s-2006-945003
Chang JI, 2019, ENDOSC INT OPEN, V7, pE655, DOI 10.1055/a-0826-4309
Chavan Radhika, 2020, VideoGIE, V5, P64, DOI [10.1016/j.vgie.2019.10.003, 10.1016/j.vgie.2019.10.003]
Chen YI, 2018, GASTROINTEST ENDOSC, V87, P1215, DOI 10.1016/j.gie.2017.07.030
Chen YI, 2018, ENDOSC INT OPEN, V6, pE363, DOI 10.1055/s-0043-123468
Chen YI, 2017, SURG ENDOSC, V31, P2946, DOI 10.1007/s00464-016-5311-1
Chen YI, 2017, GASTROINTEST ENDOSC, V85, P170, DOI 10.1016/j.gie.2016.07.031
Chin JYL, 2020, ENDOSC INT OPEN, V08, pE1633, DOI 10.1055/a-1236-3217
Cho DH, 2017, GASTROINTEST ENDOSC, V85, P1067, DOI 10.1016/j.gie.2016.09.010
Choi JH, 2014, ENDOSCOPY, V46, P656, DOI 10.1055/s-0034-1365720
Coelen RJS, 2018, LANCET GASTROENTEROL, V3, P681, DOI 10.1016/S2468-1253(18)30234-6
Colan-Hernandez J, 2017, SURG ENDOSC, V31, P3711, DOI 10.1007/s00464-016-5410-z
Cotton PB, 2010, GASTROINTEST ENDOSC, V71, P446, DOI 10.1016/j.gie.2009.10.027
Sanz MD, 2021, SURG ENDOSC, V35, P6754, DOI 10.1007/s00464-020-08179-y
De Bie C, 2022, SURG ENDOSC, V36, P2393, DOI 10.1007/s00464-021-08520-z
Dhindsa BS, 2020, ENDOSC INT OPEN, V8, pE163, DOI 10.1055/a-1067-4411
Dhir V, 2015, GASTROINTEST ENDOSC, V81, P913, DOI 10.1016/j.gie.2014.09.054
Dhir V, 2013, UNITED EUR GASTROENT, V1, P103, DOI 10.1177/2050640613480145
Dhir V, 2012, GASTROINTEST ENDOSC, V75, P354, DOI 10.1016/j.gie.2011.07.075
Dumonceau JM, 2012, ENDOSCOPY, V44, P277, DOI 10.1055/s-0031-1291633
Dumonceau JM, 2018, ENDOSCOPY, V50, P910, DOI 10.1055/a-0659-9864
El Bacha H, 2020, ACG CASE REP J, V7, DOI 10.14309/crj.0000000000000442
El Chafic AH, 2019, DIGEST DIS SCI, V64, P3321, DOI 10.1007/s10620-019-05688-2
Fan WT, 2022, MINIM INVASIV THER, V31, P159, DOI 10.1080/13645706.2020.1792500
Fiori E, 2004, ANTICANCER RES, V24, P269
François E, 2002, GASTROINTEST ENDOSC, V56, P128, DOI 10.1067/mge.2002.125547

Fujii LL, 2013, GASTROINTEST ENDOSC, V78, P854, DOI 10.1016/j.gie.2013.05.016
Garcia-Sumalla A, 2021, SURG ENDOSC, V35, P4873, DOI 10.1007/s00464-021-08435-9
Gaujoux S, 2021, HPB, V23, P154, DOI 10.1016/j.hpb.2020.06.001
Ge PS, 2019, SURG ENDOSC, V33, P3404, DOI 10.1007/s00464-018-06636-3
Godat S, 2017, ENDOSC ULTRASOUND, V6, P181, DOI 10.4103/2303-9027.208174
Guo JT, 2016, GASTROENT RES PRACT, V2016, DOI 10.1155/2016/9469472
Gupta K, 2014, J CLIN GASTROENTEROL, V48, P80, DOI 10.1097/MCG.0b013e31828c6822
Gutierrez OIB, 2018, ENDOSCOPY, V50, P891, DOI 10.1055/s-0044-102254
Guyatt GH, 2011, J CLIN EPIDEMIOL, V64, P1311, DOI [10.1016/j.jclinepi.2011.03.017, 10.1016/j.jclinepi.2011.06.004]
Han SY, 2019, SCI REP-UK, V9, DOI 10.1038/s41598-019-52993-x
Hassan C, 2020, ENDOSCOPY, V52, P123, DOI 10.1055/a-1067-4657
Hedjoudje A, 2019, UNITED EUR GASTROENT, V7, P60, DOI 10.1177/2050640618808147
Honjo M, 2018, ENDOSC ULTRASOUND, V7, P376, DOI 10.4103/eus.eus_2_18
Hosmer A, 2018, ENDOSC INT OPEN, V6, pE127, DOI 10.1055/s-0043-123188
Hu JL, 2020, SCAND J GASTROENTERO, V55, P865, DOI 10.1080/00365521.2020.1778077
Imai H, 2016, GASTROINTEST ENDOSC, V84, P147, DOI 10.1016/j.gie.2015.12.024
Iqbal U, 2020, ENDOSC ULTRASOUND, V9, P16, DOI 10.4103/eus.eus_70_19
Issa D, 2021, ENDOSCOPY, V53, P827, DOI 10.1055/a-1259-0349
Itoi T, 2017, DIGEST ENDOSC, V29, P495, DOI 10.1111/den.12794
Itoi T, 2013, DIGEST ENDOSC, V25, P241, DOI 10.1111/den.12048
Iwashita T, 2012, ENDOSCOPY, V44, P60, DOI 10.1055/s-0030-1256871
Iwashita T, 2017, DIGEST ENDOSC, V29, P362, DOI 10.1111/den.12800
Iwashita T, 2016, GASTROINTEST ENDOSC, V83, P394, DOI 10.1016/j.gie.2015.04.043
Iwashita T, 2013, DIGEST DIS SCI, V58, P2417, DOI 10.1007/s10620-013-2645-6
Jacques J, 2020, GASTROINTEST ENDOSC, V92, P134, DOI 10.1016/j.gie.2020.01.055
Jacques J, 2019, ENDOSCOPY, V51, P540, DOI 10.1055/a-0735-9137
James TW, 2019, ENDOSC ULTRASOUND, V8, pS28, DOI 10.4103/eus.eus_41_19
James TW, 2020, GASTROINTEST ENDOSC, V91, P537, DOI 10.1016/j.gie.2019.11.017
James TW, 2018, GASTROINTEST ENDOSC, V88, P547, DOI 10.1016/j.gie.2018.04.2353
Jang JW, 2012, GASTROENTEROLOGY, V142, P805, DOI 10.1053/j.gastro.2011.12.051
García-Alonso FJ, 2021, ENDOSCOPY, V53, P691, DOI 10.1055/a-1266-7592
Jeurnink SM, 2010, GASTROINTEST ENDOSC, V71, P490, DOI 10.1016/j.gie.2009.09.042
Jin Z, 2020, DIGEST ENDOSC, V32, P16, DOI 10.1111/den.13456
Jovani M, 2021, GASTROINTEST ENDOSC, V93, P1088, DOI 10.1016/j.gie.2020.09.041
Jue TL, 2021, GASTROINTEST ENDOSC, V93, P309, DOI 10.1016/j.gie.2020.07.063
Kahaleh M, 2006, GASTROINTEST ENDOSC, V64, P52, DOI 10.1016/j.gie.2006.01.063
Kahaleh M, 2007, GASTROINTEST ENDOSC, V65, P224, DOI 10.1016/j.gie.2006.05.008
Kahaleh M, 2013, WORLD J GASTROENTERO, V19, P1372, DOI 10.3748/wjg.v19.i9.1372
Kalva NR, 2018, CAN J GASTROENTEROL, V2018, DOI 10.1155/2018/7070961
Kamata K, 2017, WORLD J GASTROENTERO, V23, P661, DOI 10.3748/wjg.v23.i4.661
Kanno Y, 2019, J GASTROINTEST CANC, V50, P469, DOI 10.1007/s12029-018-0096-1

Kastelijn JB, 2021, CLIN ENDOSC, V54, P603, DOI 10.5946/ce.2020.201
Kastelijn JB, 2020, ENDOSC INT OPEN, V08, pE1194, DOI 10.1055/a-1214-5659
Kawakubo K, 2014, J HEPATO-BIL-PAN SCI, V21, P328, DOI 10.1002/jhbp.27
Kedia P, 2019, J CLIN GASTROENTEROL, V53, P304, DOI 10.1097/MCG.0000000000001037
Kerdsirichairat T, 2019, ENDOSC INT OPEN, V7, pE144, DOI 10.1055/a-0799-9939
Khan MA, 2016, DIGEST DIS SCI, V61, P684, DOI 10.1007/s10620-015-3933-0
Khan Z, 2021, ENDOSC ULTRASOUND, V10, P98, DOI 10.4103/eus.eus_67_20
Khashab Mouen A, 2019, Gastroenterol Hepatol (N Y), V15, P323
Khashab MA, 2017, ENDOSC INT OPEN, V5, pE275, DOI 10.1055/s-0043-101695
Khashab MA, 2016, ENDOSC INT OPEN, V4, pE487, DOI 10.1055/s-0042-102648
Khashab MA, 2016, ENDOSC INT OPEN, V4, pE175, DOI 10.1055/s-0041-109083
Khashab MA, 2015, DIGEST DIS SCI, V60, P557, DOI 10.1007/s10620-014-3300-6
Khashab MA, 2013, GASTROINTEST ENDOSC, V78, P734, DOI 10.1016/j.gie.2013.05.013
Kim TH, 2012, WORLD J GASTROENTERO, V18, P2526, DOI 10.3748/wjg.v18.i20.2526
Kiriukova M, 2020, FRONT MED-LAUSANNE, V7, DOI 10.3389/fmed.2020.00495
Kochhar Gursimran S, 2020, Endosc Int Open, V8, pE877, DOI [10.1055/a-1164-6282, 10.1055/a-1164-6282]
Kongkam P, 2021, ENDOSCOPY, V53, P55, DOI 10.1055/a-1195-8197
Kongkam P, 2019, DIGEST ENDOSC, V31, P50, DOI 10.1111/den.13371
Kouanda A, 2021, SURG ENDOSC, V35, P7058, DOI 10.1007/s00464-020-08221-z
KOZAREK RA, 1990, GASTROINTEST ENDOSC, V36, P558, DOI 10.1016/S0016-5107(90)71163-7
Krafft MR, 2020, GASTROINTEST ENDOSC, V92, P1055, DOI 10.1016/j.gie.2020.04.061
Krafft MR, 2019, DIGEST DIS SCI, V64, P1770, DOI 10.1007/s10620-019-05495-9
Krishnamoorthi R, 2020, GASTROINTEST ENDOSC, V92, P1067, DOI 10.1016/j.gie.2020.05.053
Krishnamoorthi R, 2020, SURG ENDOSC, V34, P1904, DOI 10.1007/s00464-020-07409-7
Kunda R, 2016, SURG ENDOSC, V30, P5002, DOI 10.1007/s00464-016-4845-6
Kuraoka Naosuke, 2020, Endosc Int Open, V8, pE861, DOI [10.1055/a-1161-8488, 10.1055/a-1161-8488]
Lee TH, 2016, CLIN GASTROENTEROL H, V14, P1011, DOI 10.1016/j.cgh.2015.12.032
Luk SWY, 2019, ENDOSCOPY, V51, P722, DOI 10.1055/a-0929-6603
Ma KW, 2020, J GASTROEN HEPATOL, V35, P1753, DOI 10.1111/jgh.15089
Mangas-Sanjuan C, 2020, ENDOSCOPY, V52, pE47, DOI 10.1055/a-0991-7763
Marrache MK, 2021, GASTROINTEST ENDOSC, V93, P34, DOI 10.1016/j.gie.2020.06.057
Martínez B, 2019, ENDOSC INT OPEN, V7, pE1038, DOI 10.1055/a-0918-5931
McCarty Thomas R, 2019, Endosc Int Open, V7, pE1474, DOI [10.1055/a-0996-8178, 10.1055/a-0996-8178]
Mehta S, 2006, SURG ENDOSC, V20, P239, DOI 10.1007/s00464-005-0130-9
Minaga K, 2020, THER ADV GASTROENTER, V13, DOI 10.1177/1756284820930964
Minaga K, 2019, DIGEST ENDOSC, V31, P575, DOI 10.1111/den.13406
Minaga K, 2017, SURG ENDOSC, V31, P4764, DOI 10.1007/s00464-017-5553-6
Minata MK, 2016, ENDOSC INT OPEN, V4, pE1158, DOI 10.1055/s-0042-115935
Miyano A, 2018, J GASTROINTEST SURG, V22, P1312, DOI 10.1007/s11605-018-3758-1
Mohan BP, 2019, J CLIN GASTROENTEROL, V53, P243, DOI 10.1097/MCG.0000000000001167
Moole H, 2016, CAN J GASTROENTEROL, V2016, DOI 10.1155/2016/4726078
Mori Y, 2018, J HEPATO-BIL-PAN SCI, V25, P87, DOI 10.1002/jhbp.504

Moryoussef F, 2017, THER ADV GASTROENTER, V10, P537, DOI 10.1177/1756283X17702614
Mukai S, 2019, CURR OPIN GASTROEN, V35, P408, DOI 10.1097/MOG.0000000000000565
Mukai S, 2019, GASTROINTEST ENDOSC, V89, P399, DOI 10.1016/j.gie.2018.07.030
Mukai S, 2017, J HEPATO-BIL-PAN SCI, V24, P537, DOI 10.1002/jhbp.496
Nakai Y, 2020, GASTROINTEST ENDOSC, V92, P623, DOI 10.1016/j.gie.2020.03.3856
Nakai Y, 2019, CLIN ENDOSC, V52, P212, DOI 10.5946/ce.2018.188
Nakai Y, 2019, ENDOSC ULTRASOUND, V8, P111, DOI 10.4103/eus.eus_17_18
Nakai Y, 2016, ENDOSCOPY, V48, P1125, DOI 10.1055/s-0042-116595
Nennstiel S, 2015, J CLIN GASTROENTEROL, V49, P764, DOI 10.1097/MCG.0000000000000275
Ogura T, 2019, ENDOSC ULTRASOUND, V8, P398, DOI 10.4103/eus.eus_45_19
Ogura T, 2018, DIGEST ENDOSC, V30, P252, DOI 10.1111/den.12976
Ogura T, 2016, ENDOSCOPY, V48, P156, DOI 10.1055/s-0034-1392859
Oh D, 2017, THER ADV GASTROENTER, V10, P42, DOI 10.1177/1756283X16671671
Oh D, 2016, GASTROINTEST ENDOSC, V83, P366, DOI 10.1016/j.gie.2015.07.015
Okuno N, 2017, INTERNAL MED, V56, P3135, DOI 10.2169/internalmedicine.8677-16
Paik WH, 2018, AM J GASTROENTEROL, V113, P987, DOI 10.1038/s41395-018-0122-8
Park DH, 2015, J GASTROEN HEPATOL, V30, P1461, DOI 10.1111/jgh.13027
Park DH, 2011, GASTROINTEST ENDOSC, V74, P1276, DOI 10.1016/j.gie.2011.07.054
Park JK, 2018, GASTROINTEST ENDOSC, V88, P277, DOI 10.1016/j.gie.2018.03.015
Park SJ, 2013, GASTROINTEST ENDOSC, V78, P374, DOI 10.1016/j.gie.2013.04.183
Park SW, 2022, DIGEST ENDOSC, V34, P439, DOI 10.1111/den.14083
Perez-Miranda M, 2018, ENDOSC ULTRASOUND, V7, P79, DOI 10.4103/eus.eus_5_18
Perez-Miranda M, 2017, J CLIN GASTROENTEROL, V51, P896, DOI 10.1097/MCG.0000000000000887
Pizzicannella M, 2020, ENDOSC ULTRASOUND, V9, P45, DOI 10.4103/eus.eus_55_19
Poincloux L, 2015, ENDOSCOPY, V47, P794, DOI [10.1055/s-0035-1545399, 10.1055/s-0034-1391988]
Prachayakul V, 2013, WORLD J GASTROENTERO, V19, P4758, DOI 10.3748/wjg.v19.i29.4758
Prakash S, 2022, ENDOSCOPY, V54, P52, DOI 10.1055/a-1376-2394
Rai P, 2018, ENDOSC INT OPEN, V6, pE67, DOI 10.1055/s-0043-120664
Rodrigues-Pinto E, 2016, CLIN GASTROENTEROL H, V14, P633, DOI 10.1016/j.cgh.2015.11.010
Runge TM, 2021, ENDOSCOPY, V53, P611, DOI 10.1055/a-1254-3942
Saumoy M, 2018, ENDOSC ULTRASOUND, V7, P97, DOI 10.4103/eus.eus_9_18
Shah JN, 2012, GASTROINTEST ENDOSC, V75, P56, DOI 10.1016/j.gie.2011.08.032
Sharaiha RZ, 2017, GASTROINTEST ENDOSC, V85, P904, DOI 10.1016/j.gie.2016.12.023
Sharaiha RZ, 2016, SURG ENDOSC, V30, P5500, DOI 10.1007/s00464-016-4913-y
Shimamura Y, 2017, CLIN ENDOSC, V50, P112, DOI 10.5946/ce.2017.046
Shiomi H, 2018, DIGEST DIS SCI, V63, P787, DOI 10.1007/s10620-018-4908-8
Siddiqui A, 2019, SURG ENDOSC, V33, P1260, DOI 10.1007/s00464-018-6406-7
Siddiqui UD, 2018, GASTROENTEROLOGY, V154, P1911, DOI 10.1053/j.gastro.2017.12.046
Skinner M, 2014, ENDOSCOPY, V46, P560, DOI 10.1055/s-0034-1365698
Sobani ZA, 2020, AM J GASTROENTEROL, V115, P634, DOI 10.14309/ajg.0000000000000567
SOLT J, 1984, GASTROINTEST ENDOSC, V30, P359, DOI 10.1016/S0016-5107(84)72456-4
Sportes A, 2017, THER ADV GASTROENTER, V10, P483, DOI 10.1177/1756283X17702096

Sundaram S, 2021, ENDOSC ULTRASOUND, V10, P154, DOI 10.4103/EUS-D-21-00004
Swan MP, 2011, WORLD J GASTROENTERO, V17, P4993, DOI 10.3748/wjg.v17.i45.4993
Taibi A, 2020, DIGEST ENDOSC, V32, P816, DOI 10.1111/den.13645
Taunk P, 2015, ENDOSCOPY, V47, pE395, DOI 10.1055/s-0034-1392564
Teoh AYB, 2020, GUT, V69, P1085, DOI 10.1136/gutjnl-2019-319996
Teoh AYB, 2018, GUT, V67, P1209, DOI 10.1136/gutjnl-2017-314341
Teoh AYB, 2019, ENDOSC ULTRASOUND, V8, pS40, DOI 10.4103/eus.eus_49_19
Tessier G, 2007, GASTROINTEST ENDOSC, V65, P233, DOI 10.1016/j.gie.2006.06.029
Tonnesen CJ, 2020, ENDOSCOPY, V52, P654, DOI 10.1055/a-1139-9313
Tonozuka R, 2020, CLIN ENDOSC, V53, P510, DOI 10.5946/ce.2020.151
Tringali A, 2019, ANN GASTROENTEROL, V32, P330, DOI 10.20524/aog.2019.0390
Tyberg A, 2017, GASTROINTEST ENDOSC, V85, P164, DOI 10.1016/j.gie.2016.07.030
Tyberg A, 2016, ENDOSC INT OPEN, V4, pE276, DOI 10.1055/s-0042-101789
Uemura RS, 2018, J CLIN GASTROENTEROL, V52, P123, DOI 10.1097/MCG.0000000000000948
Vanella G, 2020, ENDOSC INT OPEN, V08, pE1782, DOI 10.1055/a-1264-7511
Vila JJ, 2012, GASTROINTEST ENDOSC, V76, P1133, DOI 10.1016/j.gie.2012.08.001
Walter D, 2016, GUT, V65, P6, DOI 10.1136/gutjnl-2015-309925
Wang KX, 2016, GASTROINTEST ENDOSC, V83, P1218, DOI 10.1016/j.gie.2015.10.033
Wang TJ, 2019, SURG ENDOSC, V33, P2024, DOI 10.1007/s00464-019-06715-z
Widmer Jessica, 2013, Gastrointest Endosc Clin N Am, V23, P847, DOI 10.1016/j.giec.2013.06.011
Xu GF, 2020, ENDOSC INT OPEN, V08, pE1690, DOI 10.1055/a-1221-9656
Xu MM, 2020, CLIN ENDOSC, V53, P94, DOI 10.5946/ce.2018.196
NR 192
TC 372
Z9 427
U1 5
U2 30
PU GEORG THIEME VERLAG KG
PI STUTTGART
PA Oswald-Hesse-Strasse 50, D-70469 STUTTGART, GERMANY
SN 0013-726X
EI 1438-8812
J9 ENDOSCOPY
JI Endoscopy
PD FEB
PY 2022
VL 54
IS 02
BP 185
EP 205
DI 10.1055/a-1717-1391
EA DEC 2021

PG 21
WC Gastroenterology & Hepatology; Surgery
WE Science Citation Index Expanded (SCI-EXPANDED)
SC Gastroenterology & Hepatology; Surgery
GA YW4LO
UT WOS:000732987600001
PM 34937098
OA Green Submitted, Green Accepted, Green Published, Bronze
DA 2026-05-09
ER

PT J
AU Saumoy, M
Tyberg, A
Brown, E
Eachempati, SR
Lieberman, M
Afaneh, C
Kunda, R
Cosgrove, N
Siddiqui, A
Gaidhane, M
Kahaleh, M
AF Saumoy, Monica
Tyberg, Amy
Brown, Elizabeth
Eachempati, Soumitra R.
Lieberman, Michael
Afaneh, Cheguevara
Kunda, Rastislav
Cosgrove, Natalie
Siddiqui, Ali
Gaidhane, Monica
Kahaleh, Michel
TI Successful Cholecystectomy After Endoscopic Ultrasound Gallbladder
Drainage Compared With Percutaneous Cholecystostomy, Can it Be Done?
SO JOURNAL OF CLINICAL GASTROENTEROLOGY
LA English
DT Article
DE endoscopic ultrasound-guided gallbladder drainage; EGBD; lumen apposing
metal stent; LAMS; percutaneous gallbladder drainage; PTGD;
cholecystectomy; gallbladder

ID LAPAROSCOPIC CHOLECYSTECTOMY; ACUTE CHOLECYSTITIS; MANAGEMENT

AB Background: Endoscopic ultrasound-guided gallbladder drainage (EGBD) with a lumen apposing metal stent is becoming a widely accepted alternative to percutaneous gallbladder drainage (PTGD) for patients who are not candidates for cholecystectomy (CCY). In some patients, medical comorbidites can improve, allowing them to undergo CCY. We compare feasibility and outcomes of interval CCY after EGBD versus PTGD. Methods: We conducted a multicentered international cohort study of patients who underwent EGBD or PTGD and then underwent interval CCY. Baseline patient demographics, procedural details, and follow-up data were recorded and compared. Results: In total, 34 patients were included. Thirteen patients underwent EGBD followed by CCY (mean age, 53.77 +/- 17.27, 46.15% male), and 21 patients underwent PTGD followed by CCY (mean age, 62.14 +/- 13.06, 61.9% male). There was no statistically significant difference in mean Charlson Comorbidity Index (P=0.12) or etiology of cholecystitis (P=0.85) between the 2 groups. All patients had a technically successful CCY. There was no difference between rates of open versus laparoscopic CCY (P=1). In addition, there was no difference in postsurgical adverse events (P=0.23). Conclusions: Surgical CCY after EGBD with lumen apposing metal stent is safe and feasible for the management of cholecystitis. If patient's underlying medical conditions improve, previous EUS-GLB drainage should not preclude patients from undergoing CCY as part of standard of care.

C1 [Saumoy, Monica; Brown, Elizabeth; Eachempati, Soumitra R.; Lieberman, Michael; Afaneh, Cheguevara] Weill Cornell Med Ctr, New York, NY USA.

[Tyberg, Amy; Gaidhane, Monica; Kahaleh, Michel] Rutgers Robert Wood Johnson, New Brunswick, NJ USA.

[Cosgrove, Natalie; Siddiqui, Ali] Thomas Jefferson, Dept Gastroenterol, Philadelphia, PA USA.

[Kunda, Rastislav] Aarhus Univ Hosp, Dept Gastroenterol, Aarhus, Denmark.

C3 Cornell University; Weill Cornell Medical Center; Weill Cornell
Medicine; Rutgers University System; Rutgers University New Brunswick;
Rutgers University Biomedical & Health Sciences; Thomas Jefferson
University; Aarhus University

RP Kahaleh, M (corresponding author), Rutgers State Univ, Dept Med, Rutgers Robert Wood Johnson Med Sch, Robert Wood Johnson Univ Hosp, 1 RWJ Pl,MEB 464, New Brunswick, NJ 08901 USA.

EM mkahaleh@gmail.com

RI Kunda, Rastislav/N-1775-2019; Kahaleh, Michel/A-9408-2011; Gaidhane,
Monica/B-9414-2009

OI Saumoy, Monica/0000-0003-2780-1206; Gaidhane, Monica/0000-0001-6773-7080

CR Alvino DML, 2017, J GASTROINTEST SURG, V21, P761, DOI 10.1007/s11605-017-3375-4

Baron TH, 2007, GASTROINTEST ENDOSC, V65, P735, DOI 10.1016/j.gie.2006.07.041

Cherng N, 2012, J AM COLL SURGEONS, V214, P196, DOI 10.1016/j.jamcollsurg.2011.11.005

Davis CA, 1999, ARCH SURG-CHICAGO, V134, P727, DOI 10.1001/archsurg.134.7.727

Davis CA, 1999, ARCH SURG-CHICAGO, V134, P731, DOI [10.1001/archsurg.134.7.727, DOI 10.1001/ARCHSURG.134.7.727]

de la Serna-Higuera C, 2013, GASTROINTEST ENDOSC, V77, P303, DOI 10.1016/j.gie.2012.09.021

ELYADERANI MK, 1983, J CLIN GASTROENTEROL, V5, P277

Gurusamy K, 2010, BRIT J SURG, V97, P141, DOI 10.1002/bjs.6870

Gurusamy KS, 2013, COCHRANE DB SYST REV, DOI 10.1002/14651858.CD007088.pub2

Hsieh YC, 2012, J GASTROINTEST SURG, V16, P1860, DOI 10.1007/s11605-012-1965-8

Hultman CS, 1996, AM SURGEON, V62, P263

Jang JW, 2012, GASTROENTEROLOGY, V142, P805, DOI 10.1053/j.gastro.2011.12.051
Jung Bo-Hyun, 2017, Ann Hepatobiliary Pancreat Surg, V21, P21, DOI [10.14701/ahbps.2017.21.1.21, 10.14701/ahbps.2017.21.1.21]
Kedia P, 2015, GASTROINTEST ENDOSC, V82, P1031, DOI 10.1016/j.gie.2015.03.1912
Khan MA, 2017, GASTROINTEST ENDOSC, V85, P76, DOI 10.1016/j.gie.2016.06.032
Khasawneh MA, 2015, J TRAUMA ACUTE CARE, V78, P100, DOI 10.1097/TA.0000000000000498
Livingston EH, 2004, AM J SURG, V188, P205, DOI 10.1016/j.amjsurg.2004.06.013
Loftus TJ, 2017, J TRAUMA ACUTE CARE, V82, P351, DOI 10.1097/TA.0000000000001315
McKay A, 2012, SURG ENDOSC, V26, P1343, DOI 10.1007/s00464-011-2035-0
Patterson EJ, 1996, SURG ENDOSC, V10, P1185, DOI 10.1007/s004649900275
Teoh AYB, 2017, ENDOSCOPY, V49, P130, DOI 10.1055/s-0042-119036
TEPLICK SK, 1991, GASTROINTEST RADIOL, V16, P154, DOI 10.1007/BF01887332
Thornton DJA, 2004, ANN ROY COLL SURG, V86, P87, DOI 10.1308/003588404322827446
Tyberg A, 2018, J CLIN GASTROENTEROL, V52, P79, DOI 10.1097/MCG.0000000000000786
Walter D, 2016, GUT, V65, P6, DOI 10.1136/gutjnl-2015-309925
Widmer J, 2015, CLIN ENDOSC, V48, P411, DOI 10.5946/ce.2015.48.5.411
NR 26
TC 69
Z9 76
U1 1
U2 7
PU LIPPINCOTT WILLIAMS & WILKINS
PI PHILADELPHIA
PA TWO COMMERCE SQ, 2001 MARKET ST, PHILADELPHIA, PA 19103 USA
SN 0192-0790
EI 1539-2031
J9 J CLIN GASTROENTEROL
JI J. Clin. Gastroenterol.
PD MAR
PY 2019
VL 53
IS 3
BP 231
EP 235
DI 10.1097/MCG.0000000000001036
PG 5
WC Gastroenterology & Hepatology
WE Science Citation Index Expanded (SCI-EXPANDED)
SC Gastroenterology & Hepatology
GA HM1II
UT WOS:000459203600017
PM 29697498

DA 2026-05-09
ER

PT J
AU Khan, S
Chandran, S
Chin, J
Karim, S
Mangira, D
Nasr, M
Ermerak, G
Trinh, A
Kia, CYH
Mules, T
Zad, M
Ang, TL
Johns, E
Tee, D
Kaul, A
Ratanachu-Ek, T
Jirathan-Opas, J
Fisher, L
Cameron, R
Welch, C
Lim, G
Metz, AJ
Moss, A
Bassan, M
Saxena, P
Kaffes, A
St John, A
Hourigan, LF
Tagkalidis, P
Weilert, F
Vaughan, R
Devereaux, B
AF Khan, Saad
Chandran, Sujievvan
Chin, Jerry
Karim, Shwan
Mangira, Dileep
Nasr, Mohamad

Ermerak, Goktug
Trinh, Andrew
Kia, Christopher Y. H.
Mules, Thomas
Zad, Mohammadali
Ang, Tiing Leong
Johns, Estella
Tee, Derrick
Kaul, Abha
Ratanachu-Ek, Thawee
Jirathan-Opas, Jirat
Fisher, Leon
Cameron, Rees
Welch, Christine
Lim, Gary
Metz, Andrew J.
Moss, Alan
Bassan, Milan
Saxena, Payal
Kaffes, Arthur
St John, Andrew
Hourigan, Luke F.
Tagkalidis, Peter
Weilert, Frank
Vaughan, Rhys
Devereaux, Benedict
TI Drainage of pancreatic fluid collections using a lumen-apposing metal stent with an electrocautery-enhanced delivery system
SO JOURNAL OF GASTROENTEROLOGY AND HEPATOLOGY
LA English
DT Article
DE endoscopic ultrasound; lumen-apposing metal stents; pancreatic fluid collections; pancreatitis
ID EUS-GUIDED DRAINAGE; ENDOSCOPIC ULTRASOUND; PLASTIC STENTS; NECROSECTOMY; MULTICENTER; MANAGEMENT; EFFICACY; EXPERIENCE; NECROSIS; SAFETY
AB Background and Aim Our aim was to evaluate the efficacy and safety of a lumen-apposing metal stent with an electrocautery-enhanced delivery system (EDS-LAMS) for endoscopic ultrasound (EUS)-guided drainage of pancreatic fluid collections (PFCs) in regular clinical practice. Methods A retrospective and subsequent prospective analysis was undertaken of all patients who underwent EUS-guided drainage of their PFCs using the EDS-LAMS at 17 tertiary therapeutic endoscopy centers. Results Two hundred eight cases of EDS-LAMS deployment were attempted in 202 patients (mean age 52.9 years) at time of evaluation. Ninety-seven patients had pancreatic pseudocysts (PPs), 75 walled-off

pancreatic necrosis (WOPN), 10 acute peripancreatic fluid collections (APFCs), 6 acute necrotic collections (ANCs), and 14 postoperative collections (POCs). Procedural technical success was achieved in 202/208 cases (97.1%). Maldeployment occurred in 7/208 cases (3.4%). Clinical success was achieved in 142/160 (88.8%) patients (PP 90%, WOPN 85.2%, APFC 100%, ANC 75%, POC 100%). Delayed adverse events included stent migration in 15/202 (7.4%), stent occlusion and infection in 16/202 (7.9%), major bleeding in 4/202 (2%), and buried EDS-LAMS in 2/202 (1%). PFC recurrence occurred in 13/142 (9.2%) patients; 9/202 (4.5%) required surgical or radiological intervention for PFC management after EDS-LAMS insertion. Conclusions This large international multicenter study evaluating the EDS-LAMS for drainage of PFCs in routine clinical practice suggests that the EDS-LAMS are safe and effective for drainage of all types of PFCs; however, further endoscopic therapy is often required for WOPN. Major bleeding was a rare complication in our cohort.
C1 [Khan, Saad; Kia, Christopher Y. H.; Devereaux, Benedict] Royal Brisbane & Womens Hosp, Dept Gastroenterol, Level 9,Ned Hanlon Bldg, Herston, Qld 4029, Australia.

[Khan, Saad; Chandran, Sujievvan; Vaughan, Rhys] Princess Alexandra Hosp, Dept Gastroenterol & Hepatol, Herston, Qld, Australia.

[Chin, Jerry; Weilert, Frank] Univ Queensland, Sch Med, Brisbane, Qld, Australia.

[Karim, Shwan; St John, Andrew; Hourigan, Luke F.] Univ Queensland, Gallipoli Med Res Inst, Sch Med, Greenslopes Private Hosp, Brisbane, Qld, Australia.

[Mangira, Dileep; Moss, Alan; Tagkalidis, Peter] Townsville Hosp, Dept Gastroenterol, Townsville, Qld, Australia.

[Nasr, Mohamad; Saxena, Payal; Kaffes, Arthur] Toowoomba Hosp, Dept Med, Toowoomba, Qld, Australia.

[Ermerak, Goktug; Bassan, Milan] Austin Hlth, Dept Gastroenterol, Toowoomba, Qld, Australia.

[Trinh, Andrew; Metz, Andrew J.; Tagkalidis, Peter] Western Hlth, Dept Gastroenterol, Toowoomba, Qld, Australia.

[Mules, Thomas; Lim, Gary] Royal Melbourne Hosp, Dept Gastroenterol, Melbourne, Vic, Australia.

[Zad, Mohammadali; Welch, Christine] Peninsula Hlth, Dept Gastroenterol, Melbourne, Vic, Australia.

[Ang, Tiing Leong] Liverpool Hosp, Dept Gastroenterol & Hepatol, Liverpool, Australia.

[Johns, Estella; Cameron, Rees] Univ New South Wales, South Western Sydney Clin Sch, Sydney, NSW, Australia.

[Tee, Derrick] Univ Sydney, Fac Med, Sydney, NSW, Australia.

[Kaul, Abha; Fisher, Leon] Lyell McEwin Hosp, Dept Gastroenterol, Adelaide, SA, Australia.

[Ratanachu-Ek, Thawee] Waikato Dist Hlth Board, Waikato Hosp, Dept Gastroenterol, Hamilton, New Zealand.

[Jirathan-Opas, Jirat] Canterbury Dist Hlth Board, Christchurch Hosp, Dept Gastroenterol, Christchurch, New Zealand.

[Bassan, Milan] Capital & Coast Dist Hlth Board, Wellington Hosp, Dept Gastroenterol, Wellington, New Zealand.

[Saxena, Payal; Kaffes, Arthur] Changi Gen Hosp, Dept Gastroenterol & Hepatol, Singapore, Singapore.

[St John, Andrew] Rajavithi Hosp, Dept Gastroenterol, Bangkok, Thailand.

[St John, Andrew; Hourigan, Luke F.; Devereaux, Benedict] Hatyai Hosp, Dept Gastroenterol, Hat Yai, Songkhla Provin, Thailand.

[Vaughan, Rhys] Univ Melbourne, Austin Hlth, Dept Med, Melbourne, Vic, Australia.

C3 Royal Brisbane & Women's Hospital; Princess Alexandra Hospital;
University of Queensland; University of Queensland; Greenslopes Private
Hospital; Queensland Health; Townsville Hospital; Queensland Health;
Toowoomba Hospital; Florey Institute of Neuroscience & Mental Health;
Howard Florey Institute Affiliates; Melbourne Health; Royal Melbourne
Hospital; Liverpool Hospital; University of New South Wales Sydney;
University of Sydney; Lyell McEwin Hospital; Waikato Hospital;
Christchurch Hospital New Zealand; Changi General Hospital; Rajavithi

Hospital; Prince of Songkla University; Florey Institute of Neuroscience
& Mental Health; Howard Florey Institute Affiliates; Austin Research
Institute; University of Melbourne
RP Devereaux, B (corresponding author), Royal Brisbane & Womens Hosp, Dept Gastroenterol, Level 9,Ned Hanlon Bldg, Herston, Qld 4029, Australia.
EM bdevereaux@ddq.net.au
RI ; Vaughan, Rhys/D-3954-2017; St John, Andrew/N-4564-2013; Nasr,
Mohamed/GPX-8673-2022
OI Tee, Derrick/0000-0003-0088-024X; Mules, Thomas/0000-0002-3943-9198;
CR Akshintala VS, 2014, GASTROINTEST ENDOSC, V79, P921, DOI 10.1016/j.gie.2013.10.032
Bakker OJ, 2012, JAMA-J AM MED ASSOC, V307, P1053, DOI 10.1001/jama.2012.276
Bang JY, 2019, GUT, V68, P1200, DOI 10.1136/gutjnl-2017-315335
Banks PA, 2013, GUT, V62, P102, DOI 10.1136/gutjnl-2012-302779
Bazerbachi F, 2018, GASTROINTEST ENDOSC, V87, P30, DOI 10.1016/j.gie.2017.08.025
Chandran S, 2015, GASTROINTEST ENDOSC, V81, P127, DOI 10.1016/j.gie.2014.06.025
Hammad T, 2018, DIGEST DIS SCI, V63, P289, DOI 10.1007/s10620-017-4851-0
Hookey LC, 2006, GASTROINTEST ENDOSC, V63, P635, DOI 10.1016/j.gie.2005.06.028
Lang GD, 2018, GASTROINTEST ENDOSC, V87, P150, DOI 10.1016/j.gie.2017.06.029
Mohan BP, 2019, ENDOSC ULTRASOUND, V8, P82, DOI 10.4103/eus.eus_7_19
Nealon WH, 2002, ANN SURG, V235, P751, DOI 10.1097/00000658-200206000-00001
Puga M, 2018, ENDOSCOPY, V50, P1022, DOI 10.1055/a-0582-9127
Rinninella E, 2015, GASTROINTEST ENDOSC, V82, P1039, DOI 10.1016/j.gie.2015.04.006
Seifert H, 2009, GUT, V58, P1260, DOI 10.1136/gut.2008.163733
Shah RJ, 2015, CLIN GASTROENTEROL H, V13, P747, DOI 10.1016/j.cgh.2014.09.047
Sharaiha RZ, 2016, CLIN GASTROENTEROL H, V14, P1797, DOI 10.1016/j.cgh.2016.05.011
Siddiqui AA, 2016, GASTROINTEST ENDOSC, V83, P699, DOI 10.1016/j.gie.2015.10.020
Singhal S, 2013, CLIN ENDOSC, V46, P506, DOI 10.5946/ce.2013.46.5.506
Talreja JP, 2008, GASTROINTEST ENDOSC, V68, P1199, DOI 10.1016/j.gie.2008.06.015
Tilara A, 2014, J AM COLL SURGEONS, V218, P33, DOI 10.1016/j.jamcollsurg.2013.09.001
Tsiotos G G, 1999, Curr Gastroenterol Rep, V1, P139
van Santvoort HC, 2010, NEW ENGL J MED, V362, P1491, DOI 10.1056/NEJMoa0908821
Venkatachalapathy SV, 2018, ENDOSC INT OPEN, V6, pE259, DOI 10.1055/s-0043-125362
Walter D, 2015, ENDOSCOPY, V47, P63, DOI 10.1055/s-0034-1378113
NR 24
TC 25
Z9 26
U1 0
U2 2
PU WILEY
PI HOBOKEN
PA 111 RIVER ST, HOBOKEN 07030-5774, NJ USA
SN 0815-9319

EI 1440-1746
J9 J GASTROEN HEPATOL
JI J. Gastroenterol. Hepatol.
PD DEC
PY 2021
VL 36
IS 12
BP 3395
EP 3401
DI 10.1111/jgh.15658
EA AUG 2021
PG 7
WC Gastroenterology & Hepatology
WE Science Citation Index Expanded (SCI-EXPANDED)
SC Gastroenterology & Hepatology
GA XQ7AE
UT WOS:000686391000001
PM 34370869
DA 2026-05-09
ER

PT J
AU El Chafic, A
Romero, RV
Shah, JN
AF El Chafic, Abdul
Romero, Ricardo V.
Shah, Janak N.
TI Interventional endoscopic ultrasound and advanced endoscopic retrograde cholangiopancreatography access techniques
SO TECHNIQUES IN GASTROINTESTINAL ENDOSCOPY
LA English
DT Review
DE ERC cannulation; Precut papillotomy; EUS-guided pseudocystdrainage; EUS-guided rendezvous; EUS-guided biliarydrainage; EUS-guided angiotherapy
ID GUIDED BILIARY DRAINAGE; PANCREATIC-FLUID COLLECTIONS; PROSPECTIVE RANDOMIZED-TRIAL; NEEDLE-KNIFE PAPILLOTOMY; CUMULATIVE SUM ANALYSIS; APPOSING METAL STENT; WALLED-OFF NECROSIS; TRANSMURAL DRAINAGE; PRECUT PAPILLOTOMY; FAILED ERCP
AB Advanced endoscopic retrograde cholangiopancreatography (ERCP) ductal access techniques require a higher level of skill compared to standard ERCP access maneuvers. These techniques are used in cases of standard ERCP access failures

and include dual-wire techniques and precut papillotomy. Interventional endoscopic ultrasound (EUS) procedures requiring a higher level of technical skill as compared to more basic EUS procedures (eg, diagnostic EUS and EUS-fine needle aspiration) include EUS-guided drainage of pancreatic fluid collections, EUS-guided pancreaticobiliary ductal access and drainage, and EUS-guided vascular interventions. In this article, we review techniques and outcomes of advanced ERCP access techniques and of interventional EUS procedures. Issues related to training in these techniques, assessing competency in these procedures, and incorporating these highly complex and higher-risk procedures into endoscopic practice are also discussed. (C) 2017 Elsevier Inc. All rights reserved.
C1 [El Chafic, Abdul; Romero, Ricardo V.; Shah, Janak N.] Ochsner Med Ctr, Dept Gastroenterol & Hepatol, Sect Adv Endoscopy, 1514 Jefferson Hwy, New Orleans, LA 70121 USA.
C3 Ochsner Health System
RP Shah, JN (corresponding author), Ochsner Med Ctr, Dept Gastroenterol & Hepatol, Sect Adv Endoscopy, 1514 Jefferson Hwy, New Orleans, LA 70121 USA.
EM janak.shah@gmail.com
CR Abu-Ramda EM, 2005, J CLIN GASTROENTEROL, V39, P717, DOI 10.1097/01.mcg.0000173928.82986.56
Adler DG, 2015, AM J GASTROENTEROL, V110, P91, DOI 10.1038/ajg.2014.386
Angsuwatcharakon P, 2012, J GASTROEN HEPATOL, V27, P356, DOI 10.1111/j.1440-1746.2011.06927.x
Arvanitakis M, 2007, GASTROINTEST ENDOSC, V65, P609, DOI 10.1016/j.gie.2006.06.083
Bailey AA, 2010, GASTROINTEST ENDOSC, V71, P266, DOI 10.1016/j.gie.2009.09.024
Bakker OJ, 2012, JAMA-J AM MED ASSOC, V307, P1053, DOI 10.1001/jama.2012.276
Bang JY, 2015, DIGEST ENDOSC, V27, P486, DOI 10.1111/den.12418
Banks PA, 2013, GUT, V62, P102, DOI 10.1136/gutjnl-2012-302779
Barkay O, 2010, GASTROINTEST ENDOSC, V71, P1166, DOI 10.1016/j.gie.2009.10.048
Baron TH, 2015, ENDOSC ULTRASOUND, V4, P34, DOI 10.4103/2303-9027.151326
Besselink M, 2013, PANCREATOLOGY, V13, pE1, DOI 10.1016/j.pan.2013.07.063
Bhat YM, 2016, GASTROINTEST ENDOSC, V83, P1164, DOI 10.1016/j.gie.2015.09.040
Binmoeller KF, 2015, J HEPATO-BIL-PAN SCI, V22, P44, DOI 10.1002/jhbp.183
Catalano MF, 2004, GASTROINTEST ENDOSC, V60, P557, DOI 10.1016/S0016-5107(04)01877-2
Cheng LF, 2010, CLIN GASTROENTEROL H, V8, P760, DOI 10.1016/j.cgh.2010.05.019
Consiglieri CF, 2016, GASTROINTEST ENDOSC, V84, P152, DOI 10.1016/j.gie.2015.12.013
COTTON PB, 1989, GASTROINTEST ENDOSC, V35, P578, DOI 10.1016/S0016-5107(89)72921-7
Cotton PB, 2015, GASTROINTEST ENDOSC, V81, P1343, DOI 10.1016/j.gie.2015.02.026
de Paulo GA, 2006, GASTROINTEST ENDOSC, V63, P396, DOI 10.1016/j.gie.2005.10.039
Dhir V, 2015, GASTROINTEST ENDOSC, V81, P440, DOI 10.1016/j.gie.2014.09.011
Dhir V, 2012, GASTROINTEST ENDOSC, V75, P354, DOI 10.1016/j.gie.2011.07.075
DiMaio CJ, 2012, GASTROINTEST ENDOSC, V76, P476, DOI 10.1016/j.gie.2012.04.437
Fabbri C, 2014, WORLD J GASTROENTERO, V20, P8424, DOI 10.3748/wjg.v20.i26.8424
FOUTCH PG, 1995, GASTROINTEST ENDOSC, V41, P25, DOI 10.1016/S0016-5107(95)70272-5
Freeman ML, 2001, GASTROINTEST ENDOSC, V54, P425, DOI 10.1067/mge.2001.117550
Giovannini M, 2001, ENDOSCOPY, V33, P898, DOI 10.1055/s-2001-17324
Harewood GC, 2002, AM J GASTROENTEROL, V97, P1708
Holt BA, 2016, GASTROINTEST ENDOSC, V83, P160, DOI 10.1016/j.gie.2015.06.019
Itoi T, 2012, GASTROINTEST ENDOSC, V75, P870, DOI 10.1016/j.gie.2011.10.020

Iwashita T, 2016, GASTROINTEST ENDOSC, V83, P394, DOI 10.1016/j.gie.2015.04.043
Kahaleh M, 2013, WORLD J GASTROENTERO, V19, P1372, DOI 10.3748/wjg.v19.i9.1372
Kasmin FE, 1996, GASTROINTEST ENDOSC, V44, P48, DOI 10.1016/S0016-5107(96)70228-6
Katsinelos P, 2012, DIGEST DIS SCI, V57, P3286, DOI 10.1007/s10620-012-2271-8
Kawakubo K, 2014, J HEPATO-BIL-PAN SCI, V21, P328, DOI 10.1002/jhbp.27
Khashab MA, 2016, ENDOSC INT OPEN, V4, pE175, DOI 10.1055/s-0041-109083
Kumar N, 2014, PANCREAS, V43, P1334, DOI 10.1097/MPA.0000000000000213
Kunda R, 2016, SURG ENDOSC, V30, P5002, DOI 10.1007/s00464-016-4845-6
Lahoti S, 2000, GASTROINTEST ENDOSC, V51, P331, DOI 10.1016/S0016-5107(00)70363-4
Lee A, 2017, ENDOSCOPY, V49, P146, DOI 10.1055/s-0042-120995
Lee TH, 2011, DIGEST DIS SCI, V56, P1896, DOI 10.1007/s10620-010-1483-z
Levy MJ, 2008, GASTROINTEST ENDOSC, V67, P572, DOI 10.1016/j.gie.2007.06.063
Levy MJ, 2008, AM J GASTROENTEROL, V103, P352, DOI 10.1111/j.1572-0241.2007.01616.x
Liao WC, 2017, GASTROINTEST ENDOSC, V85, P295, DOI 10.1016/j.gie.2016.09.037
Mallery S, 2004, GASTROINTEST ENDOSC, V59, P100, DOI 10.1016/S0016-5107(03)02300-9
Maple JT, 2017, GASTROINTEST ENDOSC, V85, P465, DOI 10.1016/j.gie.2016.11.021
Mavrogiannis C, 1999, GASTROINTEST ENDOSC, V50, P334, DOI 10.1053/ge.1999.v50.98593
Mukai S, 2015, ENDOSCOPY, V47, P47, DOI 10.1055/s-0034-1377966
Muthusamy VR, 2016, GASTROINTEST ENDOSC, V83, P481, DOI 10.1016/j.gie.2015.11.027
NEOPTOLEMOS JP, 1993, BRIT J SURG, V80, P94, DOI 10.1002/bjs.1800800131
Parihar V, 2017, EUR J GASTROEN HEPAT, V29, P371, DOI 10.1097/MEG.0000000000000818
Park DH, 2009, ENDOSCOPY, V41, P842, DOI 10.1055/s-0029-1215133
Park DH, 2013, GASTROINTEST ENDOSC, V78, P91, DOI 10.1016/j.gie.2013.01.042
Park DH, 2011, GASTROINTEST ENDOSC, V74, P1276, DOI 10.1016/j.gie.2011.07.054
Robbins D, 2014, GASTROINTEST ENDOSC, V79, P1007, DOI 10.1016/j.gie.2014.01.004
Robison LS, 2007, WORLD J GASTROENTERO, V13, P2183, DOI 10.3748/wjg.v13.i15.2183
Rollhauser C, 1998, ENDOSCOPY, V30, P691, DOI 10.1055/s-2007-1001390
Romero-Castro R, 2007, GASTROINTEST ENDOSC, V66, P402, DOI 10.1016/j.gie.2007.03.008
Romero-Castro R, 2013, GASTROINTEST ENDOSC, V78, P711, DOI 10.1016/j.gie.2013.05.009
Schutz Stephen M, 2011, Gastroenterol Hepatol (N Y), V7, P674
Shah JN, 2012, GASTROINTEST ENDOSC, V75, P56, DOI 10.1016/j.gie.2011.08.032
Shah RJ, 2015, CLIN GASTROENTEROL H, V13, P747, DOI 10.1016/j.cgh.2014.09.047
Shahidi N, 2015, GASTROINTEST ENDOSC, V81, P1337, DOI 10.1016/j.gie.2014.12.054
Sharaiha R, 2013, GASTROINTEST ENDOSC, V78, P638, DOI 10.1016/j.gie.2013.05.034
Sharaiha RZ, 2015, GASTROINTEST ENDOSC, V82, P822, DOI 10.1016/j.gie.2015.02.035
Shrode CW, 2013, DIGEST LIVER DIS, V45, P129, DOI 10.1016/j.dld.2012.08.026
Siddiqui AA, 2017, GASTROINTEST ENDOSC, V85, P758, DOI 10.1016/j.gie.2016.08.014
Siddiqui AA, 2016, GASTROINTEST ENDOSC, V83, P699, DOI 10.1016/j.gie.2015.10.020
Storm AC, 2014, GASTROINTEST ENDOSC, V80, P164, DOI 10.1016/j.gie.2014.04.005
Tang SJ, 2005, ENDOSCOPY, V37, P58, DOI 10.1055/s-2004-826077
Testoni PA, 2016, ENDOSCOPY, V48, P657, DOI 10.1055/s-0042-108641
Trevino JM, 2010, J GASTROEN HEPATOL, V25, P526, DOI 10.1111/j.1440-1746.2009.06109.x

Varadarajulu S, 2008, GASTROINTEST ENDOSC, V68, P1102, DOI 10.1016/j.gie.2008.04.028
Vila JJ, 2012, GASTROINTEST ENDOSC, V76, P1133, DOI 10.1016/j.gie.2012.08.001
Walter D, 2015, ENDOSCOPY, V47, P63, DOI 10.1055/s-0034-1378113
Wani S, 2017, CLIN GASTROENTEROL H
Wani S, 2015, CLIN GASTROENTEROL H, V13, P1318, DOI 10.1016/j.cgh.2014.11.008
Wani S, 2013, GASTROINTEST ENDOSC, V77, P558, DOI 10.1016/j.gie.2012.10.012
Weilert F, 2014, SURG ENDOSC, V28, P3193, DOI 10.1007/s00464-014-3588-5
Weilert F, 2012, GASTROINTEST ENDOSC, V76, P915, DOI 10.1016/j.gie.2011.09.027
Yoo YW, 2013, WORLD J GASTROENTERO, V19, P108, DOI 10.3748/wjg.v19.i1.108
NR 80
TC 0
Z9 0
U1 0
U2 0
PU ELSEVIER SCIENCE INC
PI NEW YORK
PA 360 PARK AVE SOUTH, NEW YORK, NY 10010-1710 USA
SN 1096-2883
EI 1558-5050
J9 TECH GASTROINTEST EN
JI Tech. Gastrointest. Endosc.
PD JUL
PY 2017
VL 19
IS 3
BP 143
EP 150
DI 10.1016/j.tgie.2017.08.004
PG 8
WC Gastroenterology & Hepatology
WE Emerging Sources Citation Index (ESCI)
SC Gastroenterology & Hepatology
GA FP5EM
UT WOS:000417642800006
DA 2026-05-09
ER

PT J
AU Yuste, RT
Garcia-Alonso, FJ
Sanchez-Ocana, R
Roman, MC

Herrero, IP
Carbajo, AY
Sanz, MD
Cuadrado, NM
Higuera, CD
Perez-Miranda, M
AF Torres Yuste, Raul
Javier Garcia-Alonso, Francisco
Sanchez-Ocana, Ramon
Cimavilla Roman, Marta
Penas Herrero, Irene
Yaiza Carbajo, Ana
De Benito Sanz, Marina
Mora Cuadrado, Natalia
De la Serna Higuera, Carlos
Perez-Miranda, Manuel
TI Safety and efficacy of endoscopic ultrasound-guided gallbladder drainage combined with endoscopic retrograde cholangiopancreatography in the same session
SO DIGESTIVE ENDOSCOPY
LA English
DT Article
DE acute cholecystitis; endoscopic retrograde cholangiopancreatography; endoscopic ultrasound; gallstone; retrospective studies
ID APPOSING METAL STENT; LONG-TERM OUTCOMES; ACUTE CHOLECYSTITIS; CHOLECYSTECTOMY; CHOLECYSTOSTOMY
AB Background and Aim Endoscopic ultrasound-guided gallbladder drainage (EUS-GBD) is an emerging option for acute cholecystitis in non-surgical candidates. Combining endoscopic retrograde cholangiopancreatography (ERCP) for common bile duct stones with EUS-GBD in a single session might become a non-surgical management strategy to comprehensively treat gallstone disease in selected patients. Methods Single-center retrospective cohort study comparing outcomes between EUS-GBD alone (group A) and single-session ERCP combined with EUS-GBD (group B). Consecutive patients who underwent EUS-GBD with a lumen-apposing metal stent (LAMS) between June 2011 and August 2018 were analyzed. Exclusion criteria were subjects included in randomized clinical trials, patients who had had ERCP within 5 days of EUS-GBD, patients in whom ERCP or EUS-GBD was carried out for salvage of one or the other procedure, and patients who underwent concurrent EUS-guided biliary drainage. Results One hundred and nine consecutive patients underwent EUS-GBD with LAMS during the study period. Seventy-one patients satisfied the inclusion criteria and 34 patients were in group A and 37 in group B. Baseline characteristics were similar in both groups. There were no significant differences in technical (97.1% vs 97.3%; P = 0.19) and clinical success rates (88.2% vs 94.6%; P = 0.42) of EUS-GBD in group A versus group B. Rate of adverse events was similar in both groups, five (14.7%) in group A versus five (13.5%) in group B. Conclusions Single-session EUS-GBD combined with ERCP has comparable rates of technical and clinical success to EUS-GBD alone. A combined EUS-GBD and ERCP procedure does not appear to increase adverse events and makes possible comprehensive treatment of gallstone disease by purely endoscopic means.

C1 [Torres Yuste, Raul; Javier Garcia-Alonso, Francisco; Sanchez-Ocana, Ramon; Cimavilla Roman, Marta; Penas Herrero, Irene; Yaiza Carbajo, Ana; De Benito Sanz, Marina; Mora Cuadrado, Natalia; De la Serna Higuera, Carlos; Perez-Miranda, Manuel] Univ Hosp Rio Hortega, Gastroenterol Dept, Calle Dulzaina 2, Valladolid 47012, Spain.
C3 Hospital del Rio Hortega
RP Yuste, RT (corresponding author), Univ Hosp Rio Hortega, Gastroenterol Dept, Calle Dulzaina 2, Valladolid 47012, Spain.
EM tyraul89@gmail.com
RI ; García-Alonso, Francisco Javier/GOK-0879-2022
OI Peñas Herrero, Irene/0009-0007-9986-8107; García-Alonso, Francisco Javier/0000-0002-1425-8534; Torres Yuste, Raul/0000-0003-4240-4110
FU Gerencia Regional de Salud
FX THIS STUDY WAS funded by the Gerencia Regional de Salud.
CR Binmoeller KF, 2011, ENDOSCOPY, V43, P337, DOI 10.1055/s-0030-1256127
Chan JHY, 2018, CLIN ENDOSC, V51, P150, DOI 10.5946/ce.2017.125
Choi JH, 2014, ENDOSCOPY, V46, P656, DOI 10.1055/s-0034-1365720
Cotton PB, 2010, GASTROINTEST ENDOSC, V71, P446, DOI 10.1016/j.gie.2009.10.027
de la Serna-Higuera C, 2013, GASTROINTEST ENDOSC, V77, P303, DOI 10.1016/j.gie.2012.09.021
Doi S, 2018, DIGEST ENDOSC, V30, P501, DOI 10.1111/den.13028
Dollhopf M, 2017, GASTROINTEST ENDOSC, V86, P636, DOI 10.1016/j.gie.2017.02.027
Gornals JB, 2016, GASTROENT HEPAT-BARC, V39, P627, DOI 10.1016/j.gastrohep.2015.12.008
Itoi T, 2008, GASTROINTEST ENDOSC, V68, P455, DOI 10.1016/j.gie.2008.02.052
Jang JW, 2012, GASTROENTEROLOGY, V142, P805, DOI 10.1053/j.gastro.2011.12.051
García-Alonso FJ, 2015, DIGEST DIS SCI, V60, P1770, DOI 10.1007/s10620-014-3497-4
Kahaleh M, 2016, DIGEST LIVER DIS, V48, P1054, DOI 10.1016/j.dld.2016.05.021
Khan MA, 2017, GASTROINTEST ENDOSC, V85, P76, DOI 10.1016/j.gie.2016.06.032
Luzón-Solanas L, 2018, REV ESP ENFERM DIG, V110, P217, DOI [10.17235/reed.2017.5262/2017, 10.17235/reed.2018.5262/2017]
Manta R, 2018, EUR J GASTROEN HEPAT, V30, P695, DOI 10.1097/MEG.0000000000001112
Mutignani M, 2009, ENDOSCOPY, V41, P539, DOI 10.1055/s-0029-1214727
Nakai Y, 2017, DIGEST ENDOSC, V29, P218, DOI 10.1111/den.12752
Peñas-Herrero I, 2015, J HEPATO-BIL-PAN SCI, V22, P35, DOI 10.1002/jhbp.182
Peng WK, 2005, BJS-BRIT J SURG, V92, P1241, DOI 10.1002/bjs.4955
Perez-Miranda Castillo M, 2015, THESIS, P118
Reinders JSK, 2010, GASTROENTEROLOGY, V138, P2315, DOI 10.1053/j.gastro.2010.02.052
Riall TS, 2010, J AM COLL SURGEONS, V210, P668, DOI 10.1016/j.jamcollsurg.2009.12.031
Saumoy M, 2018, ENDOSC ULTRASOUND, V7, P97, DOI 10.4103/eus.eus_9_18
Siddiqui A, 2019, SURG ENDOSC, V33, P1260, DOI 10.1007/s00464-018-6406-7
Staub J, 2018, GASTROINTEST ENDOSC, V87, P1499, DOI 10.1016/j.gie.2018.01.040
Teoh AY, 2019, ENDOSC INT OPEN, V7, pE964, DOI 10.1055/a-0915-2098
Teoh AYB, 2017, ENDOSCOPY, V49, P130, DOI 10.1055/s-0042-119036
Yuste RT, 2019, ANN GASTROENTEROL, V32, P514, DOI 10.20524/aog.2019.0395
Tsuyuguchi T, 2013, J HEPATO-BIL-PAN SCI, V20, P81, DOI 10.1007/s00534-012-0570-2

Walter D, 2016, GUT, V65, P6, DOI 10.1136/gutjnl-2015-309925
Walter D, 2015, ENDOSCOPY, V47, P63, DOI 10.1055/s-0034-1378113
Winbladh A, 2009, HPB, V11, P183, DOI 10.1111/j.1477-2574.2009.00052.x
Yamashita Y, 2013, J HEPATO-BIL-PAN SCI, V20, P89, DOI 10.1007/s00534-012-0567-x
NR 33
TC 22
Z9 24
U1 0
U2 1
PU WILEY
PI HOBOKEN
PA 111 RIVER ST, HOBOKEN 07030-5774, NJ USA
SN 0915-5635
EI 1443-1661
J9 DIGEST ENDOSC
JI Dig. Endosc.
PD MAY
PY 2020
VL 32
IS 4
BP 608
EP 615
DI 10.1111/den.13562
EA NOV 2019
PG 8
WC Gastroenterology & Hepatology; Surgery
WE Science Citation Index Expanded (SCI-EXPANDED)
SC Gastroenterology & Hepatology; Surgery
GA LJ6VO
UT WOS:000498141200001
PM 31608503
DA 2026-05-09
ER

PT J
AU Lesmana, CRA
Paramitha, MS
Gani, RA
AF Lesmana, Cosmas Rinaldi Adithya
Paramitha, Maria Satya
Gani, Rino Alvani
TI Therapeutic interventional endoscopic ultrasound in pancreato-biliary

disorders: Does it really replace the surgical/percutaneous approach?
SO WORLD JOURNAL OF GASTROINTESTINAL SURGERY
LA English
DT Review
DE Pancreato-biliary; Endoscopic ultrasound; Percutaneous approach;
Surgical approach; Biliary drainage; Pancreatic fluid collection
ID APPOSING METAL STENTS; PERCUTANEOUS DRAINAGE; PLASTIC STENTS; FAILED
ERCP; EUS; EFFICACY; GASTROJEJUNOSTOMY; OBSTRUCTION; SAFETY
AB Pancreato-biliary disorders are still incredibly challenging in the field of gastroenterology, as they would sometimes require multi-approach interventional procedures. Recently, therapeutic interventional endoscopic ultrasound (EUS) has emerged as a potential alternative to surgical or percutaneous approaches. Unfortunately, considering the high cost of EUS, lack of facility and expertise, most gastroenterologists still often refer cases to undergo surgical interventions without contemplating the possibility of utilizing EUS first. EUS-guided biliary drainage has become one of the best choices for establishing access to biliary system, given the clear visualization of pancreas, gallbladder, and common bile duct. Although there are still only a few studies which directly compare EUS-guided and surgical approaches for biliary drainage, current evidence demonstrated the superiority of EUS-guided approach in terms of adverse events and re-intervention rates, with similarly high technical and clinical success rates compared to percutaneous and surgical approaches, especially in patients with history of failed endoscopic retrograde cholangiopancreatography attempt. Comparable success rates with shorter length of hospital stay between endoscopic and surgical approaches have also been exhibited for pancreatic pseudocysts and walled-off necrosis. Recent findings about the progress of EUS approach in gastroenterostomy/jejunostomy also indicated a promising potential of EUS, as a less invasive approach, for managing gastric outlet obstruction.
C1 [Lesmana, Cosmas Rinaldi Adithya; Paramitha, Maria Satya; Gani, Rino Alvani] Univ Indonesia, Dr Cipto Mangunkusumo Natl Gen Hosp, Hepatobiliary Div, Dept Internal Med,Med Fac, Jakarta 10430, Indonesia.
[Lesmana, Cosmas Rinaldi Adithya] Medistra Hosp, Digest Dis & GI Oncol Ctr, Jakarta 12950, Indonesia.
C3 University of Indonesia; Medistra Hospital
RP Lesmana, CRA (corresponding author), Dr Cipto Mangunkusumo Natl Gen Hosp, Hepatobiliary Div, Dept Internal Med, Jl Diponegoro 71, Jakarta 10430, Indonesia.
EM medicaldr2001id@yahoo.com
CR Anderloni A, 2016, SURG ENDOSC, V30, P5200, DOI 10.1007/s00464-016-4894-x
Artifon ELA, 2015, ENDOSC ULTRASOUND, V4, P235, DOI 10.4103/2303-9027.163010
Bang JY, 2019, GUT, V68, P1200, DOI 10.1136/gutjnl-2017-315335
Binmoeller KF, 2011, ENDOSCOPY, V43, P337, DOI 10.1055/s-0030-1256127
Chen YI, 2019, ENDOSC INT OPEN, V7, pE347, DOI 10.1055/a-0828-7630
Chen YI, 2018, ENDOSC INT OPEN, V6, pE780, DOI 10.1055/a-0611-5082
DeWitt JM, 2021, CLIN GASTROENTEROL H, V19, P24, DOI 10.1016/j.cgh.2020.09.029
El Chafic AH, 2019, DIGEST DIS SCI, V64, P3321, DOI 10.1007/s10620-019-05688-2
Farias GFA, 2019, MEDICINE, V98, DOI 10.1097/MD.0000000000014255
Ge PS, 2020, PANCREAS, V49, P236, DOI 10.1097/MPA.0000000000001476
Han SY, 2019, SCI REP-UK, V9, DOI 10.1038/s41598-019-52993-x
Iqbal U, 2020, ENDOSC ULTRASOUND, V9, P16, DOI 10.4103/eus.eus_70_19
Isayama H, 2013, J HEPATO-BIL-PAN SCI, V20, P413, DOI 10.1007/s00534-012-0577-8
Jayaraj M., 2019, AM J GASTROENTEROL, V114, pS510, DOI 10.14309/01.ajg.0000593064.39353.39

Kalva NR, 2018, CAN J GASTROENTEROL, V2018, DOI 10.1155/2018/7070961
Khan MA, 2017, GASTROINTEST ENDOSC, V85, P76, DOI 10.1016/j.gie.2016.06.032
Khashab MA, 2017, ENDOSC INT OPEN, V5, pE275, DOI 10.1055/s-0043-101695
Khashab MA, 2015, DIGEST DIS SCI, V60, P557, DOI 10.1007/s10620-014-3300-6
Kouanda A, 2021, SURG ENDOSC, V35, P7058, DOI 10.1007/s00464-020-08221-z
Lesmana C, 2019, CASE REP GASTROENTER, V13, P385, DOI 10.1159/000502835
Lesmana CRA., 2018, DIG SYST, V2, P1, DOI DOI 10.15761/DSJ.1000120
Luk SWY, 2019, ENDOSCOPY, V51, P722, DOI 10.1055/a-0929-6603
Mohan BP, 2019, ENDOSC ULTRASOUND, V8, P82, DOI 10.4103/eus.eus_7_19
Moole H, 2017, MEDICINE, V96, DOI 10.1097/MD.0000000000005154
Okuno N, 2017, INTERNAL MED, V56, P3135, DOI 10.2169/internalmedicine.8677-16
Perez-Miranda M, 2017, J CLIN GASTROENTEROL, V51, P896, DOI 10.1097/MCG.0000000000000887
Poincloux L, 2015, ENDOSCOPY, V47, P794, DOI [10.1055/s-0035-1545399, 10.1055/s-0034-1391988]
Puga M, 2018, ENDOSCOPY, V50, P1022, DOI 10.1055/a-0582-9127
Salerno R, 2019, WORLD J GASTRO ENDOS, V11, P354, DOI 10.4253/wjge.v11.i5.354
Sharaiha RZ, 2017, GASTROINTEST ENDOSC, V85, P904, DOI 10.1016/j.gie.2016.12.023
Simons-Linares CR, 2020, J CLIN GASTROENTEROL, V54, P579, DOI 10.1097/MCG.0000000000001337
Szakó L, 2020, PANCREATOLOGY, V20, P132, DOI 10.1016/j.pan.2019.10.006
Téllez-Avila FI, 2018, SURG LAPARO ENDO PER, V28, P183, DOI 10.1097/SLE.0000000000000528
Troncone E, 2020, WORLD J GASTROENTERO, V26, P1847, DOI 10.3748/wjg.v26.i16.1847
Tyberg A, 2016, GASTROINTEST ENDOSC, V84, P941, DOI 10.1016/j.gie.2016.05.035
Vanbrugghe C, 2019, J VISC SURG, V156, P30, DOI 10.1016/j.jviscsurg.2018.07.009
Varadarajulu S, 2013, GASTROENTEROLOGY, V145, P583, DOI 10.1053/j.gastro.2013.05.046
Yarmohammadi H, 2016, CHIN CLIN ONCOL, V5, DOI 10.21037/cco.2016.10.07
NR 38
TC 6
Z9 6
U1 1
U2 6
PU BAISHIDENG PUBLISHING GROUP INC
PI PLEASANTON
PA 7041 Koll Center Parkway, Suite 160, PLEASANTON, CA, UNITED STATES
SN 1948-9366
J9 WORLD J GASTRO SURG
JI World J. Gastrointest. Surg.
PD JUN 27
PY 2021
VL 13
IS 6
BP 537
EP 547
DI 10.4240/wjgs.v13.i6.537

PG 11
WC Gastroenterology & Hepatology; Surgery
WE Science Citation Index Expanded (SCI-EXPANDED)
SC Gastroenterology & Hepatology; Surgery
GA TC7TE
UT WOS:000668841400003
PM 34194612
OA Green Accepted, gold
DA 2026-05-09
ER

PT J
AU Peng, ZX
Chen, FF
Tang, W
Zeng, X
Du, HJ
Pi, RX
Liu, HM
Lu, XX
AF Peng, Zu-Xiang
Chen, Fang-Fang
Tang, Wen
Zeng, Xu
Du, Hong-Juan
Pi, Ru-Xian
Liu, Hong-Ming
Lu, Xiao-Xiao
TI Endoscopic-ultrasound-guided biliary drainage with placement of
electrocautery-enhanced lumen-apposing metal stent for palliation of
malignant biliary obstruction: Updated meta-analysis
SO WORLD JOURNAL OF GASTROINTESTINAL SURGERY
LA English
DT Article
DE Biliary obstruction; Biliary drainage; Electrocautery-enhanced
lumen-apposing metal stents; Endoscopic ultrasound; Endoscopic
retrograde cholangiopancreatography failure
ID RETROGRADE CHOLANGIOPANCREATOGRAPHY; CHOLEDOCHODUODENOSTOMY;
MULTICENTER; PANCREATICODUODENECTOMY; EXPERIENCE; FAILS
AB BACKGROUND Endoscopic ultrasound-guided biliary drainage using electrocautery-enhanced (ECE) delivery of lumen-apposing metal stent (LAMS) is gradually being recognized as a viable palliative technique for malignant biliary obstruction after endoscopic retrograde cholangiopancreatography (ERCP) failure. However, most of the studies that have

assessed its efficacy and safety were small and heterogeneous. Prior meta-analyses of six or fewer studies that were published 2 years ago were therefore underpowered to yield convincing evidence. AIM To update the efficacy and safety of ECE-LAMS for treatment of biliary obstruction after ERCP failure. METHODS We searched PubMed, EMBASE, and Scopus databases from the inception of the ECE technique to May 13, 2022. Primary outcome measure was pooled technical success rate, and secondary outcomes were pooled rates of clinical success, reintervention, and adverse events. Meta-analysis was performed using a random-effects model following Freeman-Tukey double-arcsine transformation in R software (version 4.1.3). RESULTS Fourteen eligible studies involving 620 participants were ultimately included. The pooled rate of technical success was 96.7%, and clinical success was 91.0%. Adverse events were reported in 17.5% of patients. Overall reintervention rate was 7.3%. Subgroup analyses showed results were generally consistent. CONCLUSION ECE-LAMS has favorable success with acceptable adverse events in relieving biliary obstruction when ERCP is impossible. The consistency of results across most subgroups suggested that this is a generalizable approach.
C1 [Peng, Zu-Xiang; Tang, Wen; Pi, Ru-Xian; Liu, Hong-Ming] Army Med Univ, Daping Hosp, Dept Hepatobiliary Surg, Chongqing 400042, Peoples R China.

[Chen, Fang-Fang; Du, Hong-Juan] Chongqing Univ, Chongqing Gen Hosp, Dept Oncol, Chongqing 401147, Peoples R China.

[Zeng, Xu] Peoples Hosp Fenggang Cty, Dept Gastroenterol & Hepatobiliary Surg, Guizhou 564200, Guizhou, Peoples R China.

[Lu, Xiao-Xiao] Chongqing Univ, Canc Hosp, Hepatobiliary & Pancreat Tumor Ctr, Chongqing 400030, Peoples R China.

[Lu, Xiao-Xiao] Chongqing Univ Canc Hosp, Hepatobiliary & Pancreat Tumor Ctr, 181 Hanyu Rd, Chongqing 400030, Peoples R China.

C3 Army Medical University; Chongqing University; Chongqing University

RP Lu, XX (corresponding author), Chongqing Univ Canc Hosp, Hepatobiliary & Pancreat Tumor Ctr, 181 Hanyu Rd, Chongqing 400030, Peoples R China.

EM 377522589@qq.com

RI Lu, Xiaoxiao/J-1014-2019
CR Amato A, 2021, ENDOSCOPY, V53, P1037, DOI 10.1055/a-1324-7919

Anderloni A, 2019, WORLD J GASTROENTERO, V25, P3857, DOI 10.3748/wjg.v25.i29.3857

Anderloni A, 2019, GASTROINTEST ENDOSC, V89, P69, DOI [10.1016/j.gie.2018.04.1746, 10.1016/j.gie.2018.08.047]

Artifon ELA, 2006, AM J GASTROENTEROL, V101, P2031, DOI 10.1111/j.1572-0241.2006.00764.x

Baniya R, 2017, CLIN EXP GASTROENTER, V10, P67, DOI 10.2147/CEG.S132004

Barendregt JJ, 2013, J EPIDEMIOL COMMUN H, V67, P974, DOI 10.1136/jech-2013-203104

Bliss LA, 2016, HPB, V18, P671, DOI 10.1016/j.hpb.2016.05.008

Chin JYL, 2020, ENDOSC INT OPEN, V08, pE1633, DOI 10.1055/a-1236-3217

Cotton PB, 2010, GASTROINTEST ENDOSC, V71, P446, DOI 10.1016/j.gie.2009.10.027

Darnell EP, 2021, AM J SURG, V221, P134, DOI 10.1016/j.amjsurg.2020.07.025

DerSimonian R, 2015, CONTEMP CLIN TRIALS, V45, P139, DOI 10.1016/j.cct.2015.09.002

Di Mitri R, 2022, SURG ENDOSC, V36, P4553, DOI 10.1007/s00464-021-08808-0

Dumonceau JM, 2012, ENDOSCOPY, V44, P277, DOI 10.1055/s-0031-1291633

Ekkelenkamp VE, 2015, ENDOSCOPY, V47, P504, DOI 10.1055/s-0034-1391231

El Chafic AH, 2019, DIGEST DIS SCI, V64, P3321, DOI 10.1007/s10620-019-05688-2

Garcia-Sumalla A, 2022, TRIALS, V23, DOI 10.1186/s13063-022-06106-1
Garcia-Sumalla A, 2021, SURG ENDOSC, V35, P4873, DOI 10.1007/s00464-021-08435-9
Gaujoux S, 2021, HPB, V23, P154, DOI 10.1016/j.hpb.2020.06.001
Ginestet C, 2022, SURG ENDOSC, V36, P3365, DOI 10.1007/s00464-021-08653-1
Hindryckx P, 2021, SURG ENDOSC, V35, P6013, DOI 10.1007/s00464-020-08090-6
Horaguchi J, 2009, DIGEST ENDOSC, V21, P239, DOI 10.1111/j.1443-1661.2009.00899.x
Jacques J, 2020, GASTROINTEST ENDOSC, V92, P134, DOI 10.1016/j.gie.2020.01.055
Jacques J, 2019, ENDOSCOPY, V51, P540, DOI 10.1055/a-0735-9137
Krishnamoorthi R, 2020, SURG ENDOSC, V34, P2866, DOI 10.1007/s00464-020-07484-w
Kunda R, 2016, SURG ENDOSC, V30, P5002, DOI 10.1007/s00464-016-4845-6
Law RJ, 2021, GASTROINTEST ENDOSC, V94, P457, DOI 10.1016/j.gie.2021.05.020
Mohan BP, 2019, J CLIN GASTROENTEROL, V53, P243, DOI 10.1097/MCG.0000000000001167
Munn Z, 2015, INT J EVID-BASED HEA, V13, P147, DOI 10.1097/XEB.0000000000000054
Nam K, 2018, ENDOSC ULTRASOUND, V7, P48, DOI 10.4103/eus.eus_100_17
Nennstiel S, 2015, J CLIN GASTROENTEROL, V49, P764, DOI 10.1097/MCG.0000000000000275
On W, 2022, GASTROINTEST ENDOSC, V95, P432, DOI 10.1016/j.gie.2021.09.040
Parasa S, 2020, JAMA NETW OPEN, V3, DOI 10.1001/jamanetworkopen.2020.11335
Peng ZX, 2021, CAN J GASTROENTEROL, V2021, DOI 10.1155/2021/6696950
Poincloux L, 2015, ENDOSCOPY, V47, P794, DOI [10.1055/s-0035-1545399, 10.1055/s-0034-1391988]
Püspök A, 2005, AM J GASTROENTEROL, V100, P1743, DOI 10.1111/j.1572-0241.2005.41806.x
Rees J, 2020, BMJ OPEN, V10, DOI 10.1136/bmjopen-2019-033576
Salerno R, 2019, WORLD J GASTRO ENDOS, V11, P354, DOI 10.4253/wjge.v11.i5.354
Sharaiha RZ, 2017, GASTROINTEST ENDOSC, V85, P904, DOI 10.1016/j.gie.2016.12.023
Slim K, 2003, ANZ J SURG, V73, P712, DOI 10.1046/j.1445-2197.2003.02748.x
Stark A, 2015, SEMIN ONCOL, V42, P163, DOI 10.1053/j.seminoncol.2014.12.014
Tarantino I, 2021, ENDOSC INT OPEN, V09, pE110, DOI 10.1055/a-1313-6850
Tsuchiya T, 2018, GASTROINTEST ENDOSC, V87, P1138, DOI 10.1016/j.gie.2017.08.017
Tyberg A, 2020, ENDOSC ULTRASOUND, V9, P392, DOI 10.4103/eus.eus_42_20
Venkatachalapathy SV, 2021, GASTROINTEST ENDOSC, V94, P321, DOI 10.1016/j.gie.2021.01.029
Vila JJ, 2012, GASTROINTEST ENDOSC, V76, P1133, DOI 10.1016/j.gie.2012.08.001
Yoo HW, 2021, ENDOSCOPY, V53, P922, DOI 10.1055/a-1301-1526
NR 46
TC 8
Z9 9
U1 0
U2 1
PU BAISHIDENG PUBLISHING GROUP INC
PI PLEASANTON
PA 7041 Koll Center Parkway, Suite 160, PLEASANTON, CA, UNITED STATES
SN 1948-9366
J9 WORLD J GASTRO SURG
JI World J. Gastrointest. Surg.

PD MAR 27
PY 2024
VL 16
IS 3
DI 10.4240/wjgs.v16.i3.907
PG 15
WC Gastroenterology & Hepatology; Surgery
WE Science Citation Index Expanded (SCI-EXPANDED)
SC Gastroenterology & Hepatology; Surgery
GA NC5I3
UT WOS:001198258900021
PM 38577086
OA Green Submitted, gold
DA 2026-05-09
ER

PT J
AU Lyu, YX
  Li, T
  Wang, B
  Cheng, YX
  Chen, L
  Zhao, SC
AF Lyu, Yunxiao
  Li, Ting
  Wang, Bin
  Cheng, Yunxiao
  Chen, Liang
  Zhao, Sicong
TI Comparison Between Lumen-Apposing Metal Stents and Plastic Stents in
  Endoscopic Ultrasound-Guided Drainage of Pancreatic Fluid Collection A
  Meta-analysis and Systematic Review
SO PANCREAS
LA English
DT Review
DE endoscopic ultrasound; pancreatic fluid collections; systematic review;
  meta-analysis; LAMS; lumen-apposing metal stent; DPPS; double-pigtail
  plastic stent; EUS; endoscopic ultrasound; PFC; pancreatic fluid
  collection; WON; walled-off necrosis; PP; pancreatic pseudocyst; DEN;
  direct endoscopic necrosectomy; OR; odds ratio; CI; confidence interval
ID DOUBLE-PIGTAIL STENTS; MANAGEMENT; EFFICACY; SHIFT
AB Objectives

This study aimed to explore efficacy and safety between LAMSs (lumen-apposing metal stents) and DPPSs (double-pigtail plastic stents) in endoscopic ultrasound-guided drainage for pancreatic fluid collections. Methods

Electronic databases were searched to identify relevant studies published until July 20, 2020. Results

Fifteen studies were identified in this study. Endoscopic ultrasound-guided drainage with LAMS has higher clinical success (90.01% vs 82.56%) (odds ratio [OR], 2.44; 95% confidence interval [CI], 1.79-3.33; P < 0.00001), less recurrence (OR, 0.44; 95% CI, 0.29-0.68; P = 0.0002), and fewer additional interventions (OR, 0.34; 95% CI, 0.211-0.55; P < 0.001). There was no significant difference between LAMS and DPPS in technical success (97.45% vs 97.38%) (OR, 0.92; 95% CI, 0.50-1.70; P = 0.80), adverse events (OR, 0.92; 95% CI, 0.41-2.09; P = 0.84), stent-related adverse events (OR, 0.78; 95% CI, 0.39-1.54; P = 0.47), and bleeding (OR, 1.47; 95% CI, 0.57-3.28; P = 0.42). Lumen-apposing metal stents have slightly more perforations (OR, 7.10; 95% CI, 1.22-41.30; P = 0.03) in studies of walled-off necrosis. Conclusions

Lumen-apposing metal stents have the advantage of higher clinical success, less recurrence, and fewer additional interventions. However, LAMS may increase perforation for walled-off necrosis.
C1 [Lyu, Yunxiao; Wang, Bin; Cheng, Yunxiao; Chen, Liang; Zhao, Sicong] Dongyang Peoples Hosp, Dept Hepatobiliary Surg, Dongyang, Peoples R China.

[Li, Ting] Dongyang Peoples Hosp, Dept Personnel, Dongyang, Peoples R China.

RP Lyu, YX (corresponding author), Dongyang Peoples Hosp, Dept Hepatobiliary Surg, Dongyang, Peoples R China.

EM lvyunxiao1986@gmail.com
FU Health Commission of Zhejiang Province [2019ky761]

FX This study was supported by a grant (2019ky761) from the Health
Commission of Zhejiang Province.
CR Bang JY, 2019, DIGEST ENDOSC, V31, P619, DOI 10.1111/den.13428
Bang JY, 2019, GUT, V68, P1200, DOI 10.1136/gutjnl-2017-315335
Bang JY, 2017, DIGEST ENDOSC, V29, P83, DOI 10.1111/den.12681
Banks PA, 2013, GUT, V62, P102, DOI 10.1136/gutjnl-2012-302779
Bapaye A, 2017, DIGEST ENDOSC, V29, P104, DOI 10.1111/den.12704
Baron TH, 1996, GASTROENTEROLOGY, V111, P755, DOI 10.1053/gast.1996.v111.pm8780582
Brimhall B, 2018, CLIN GASTROENTEROL H, V16, P1521, DOI 10.1016/j.cgh.2018.02.021
Chen YI, 2019, ENDOSC INT OPEN, V7, pE347, DOI 10.1055/a-0828-7630
Elmunzer BJ, 2018, CLIN GASTROENTEROL H, V16, P1851, DOI 10.1016/j.cgh.2018.03.021
Fabbri C, 2012, WORLD J GASTRO ENDOS, V4, P479, DOI 10.4253/wjge.v4.i11.479
Fasullo M, 2018, CLIN EXP GASTROENTER, V11, P249, DOI 10.2147/CEG.S167736
Ge N, 2017, J TRANSL INTERN MED, V5, P213, DOI 10.1515/jtim-2017-0036
Ge PS, 2020, PANCREAS, V49, P236, DOI 10.1097/MPA.0000000000001476
Hammad T, 2018, DIGEST DIS SCI, V63, P289, DOI 10.1007/s10620-017-4851-0
Jadad AR, 1996, CONTROL CLIN TRIALS, V17, P1, DOI 10.1016/0197-2456(95)00134-4
Kayal A, 2021, SURG ENDOSC, V35, P2698, DOI 10.1007/s00464-020-07699-x
Lang GD, 2018, GASTROINTEST ENDOSC, V87, P150, DOI 10.1016/j.gie.2017.06.029
Moher D, 2010, INT J SURG, V8, P336, DOI 10.1016/j.ijsu.2010.02.007
Muthusamy VR, 2016, GASTROINTEST ENDOSC, V83, P481, DOI 10.1016/j.gie.2015.11.027
Park CH, 2020, J GASTROEN HEPATOL, V35, P941, DOI 10.1111/jgh.14960
Park CH, 2018, GASTROINTEST ENDOSC, V87, P1040, DOI 10.1016/j.gie.2017.09.021
Poornachandra KS, 2011, J CLIN GASTROENTEROL, V45, P159, DOI 10.1097/MCG.0b013e3181dd9d14

Rinninella E, 2015, GASTROINTEST ENDOSC, V82, P1039, DOI 10.1016/j.gie.2015.04.006
Shin HC, 2019, CLIN ENDOSC, V52, P353
Siddiqui AA, 2017, GASTROINTEST ENDOSC, V85, P758, DOI 10.1016/j.gie.2016.08.014
Stang A, 2010, EUR J EPIDEMIOL, V25, P603, DOI 10.1007/s10654-010-9491-z
Tan SL, 2020, GASTROENT RES PRACT, V2020, DOI 10.1155/2020/4952721
Theerasuwipakorn N, 2019, J TRANSL INTERN MED, V7, P170, DOI 10.2478/jtim-2019-0032
Tyberg A, 2016, GASTROINTEST ENDOSC, V84, P458, DOI 10.1016/j.gie.2016.04.038
Tyberg A, 2016, WORLD J GASTROENTERO, V22, P2256, DOI 10.3748/wjg.v22.i7.2256
Wang ZJ, 2019, J GASTROEN HEPATOL, V34, P791, DOI 10.1111/jgh.14557
Yang D, 2018, ENDOSC INT OPEN, V6, pE1267, DOI 10.1055/a-0732-502
Yang JL, 2019, ENDOSCOPY, V51, P1035, DOI 10.1055/a-0759-1353
Zhu HY, 2020, J GASTROEN HEPATOL, V35, P2103, DOI 10.1111/jgh.15086
NR 34
TC 23
Z9 26
U1 1
U2 7
PU LIPPINCOTT WILLIAMS & WILKINS
PI PHILADELPHIA
PA TWO COMMERCE SQ, 2001 MARKET ST, PHILADELPHIA, PA 19103 USA
SN 0885-3177
EI 1536-4828
J9 PANCREAS
JI Pancreas
PD APR
PY 2021
VL 50
IS 4
BP 571
EP 578
DI 10.1097/MPA.0000000000001798
PG 8
WC Gastroenterology & Hepatology
WE Science Citation Index Expanded (SCI-EXPANDED)
SC Gastroenterology & Hepatology
GA RZ3UM
UT WOS:000648522600017
PM 33939671
DA 2026-05-09
ER

PT J

AU Magahis, PT
Westerveld, D
Simons, M
Mahadev, S
AF Magahis, Patrick Tiongco
Westerveld, Donevan
Simons, Malorie
Mahadev, SriHari
TI A surprising culprit for delayed gastrointestinal bleeding after endoscopic ultrasound-guided cholecystoduodenostomy: the double-pigtail stent
SO CLINICAL JOURNAL OF GASTROENTEROLOGY
LA English
DT Article
DE Endoscopic-guided gallbladder drainage; Cholecystoduodenostomy; Cholecystitis; LAMS; Double-pigtail stent
ID APPOSING METAL STENT; GALLBLADDER DRAINAGE
AB Endoscopic ultrasound-guided gallbladder drainage using a lumen-apposing metal stent has emerged as an accepted option for the treatment of acute cholecystitis in patients unfit for surgery. While metal stents carry a risk of intra- and post-procedural bleeding, the coaxial placement of a double-pigtail stents through lumen-apposing metal stents has been proposed to lower the bleeding risk by preventing tissue abrasion against the stent flanges. We present a case of an 83 year-old male who had previously undergone uncomplicated endoscopic ultrasound-guided cholecystoduodenostomy with this technique. Six months later, he presented with upper gastrointestinal bleeding due to a duodenal pressure ulcer from the coaxial 10-Fr double-pigtail stent originally employed to prevent such bleeding. The 10-Fr stent was replaced with two 7-Fr stents whose increased flexibility and distribution of pressure across multiple points of contact with the duodenal wall was theorized to reduce the likelihood of erosion or perforation. Following the procedure, the patient's clinical course improved significantly with complete resolution of his symptoms of choledocholithiasis and cholecystitis. While 10-Fr double-pigtail stents are generally preferred for this indication due to their stiffness that reduces out-migration, use of more flexible 7-Fr stents may be advisable in thin-walled structures such as the duodenum.
C1 [Magahis, Patrick Tiongco] Cornell Univ, Joan & Sanford Weill Med Coll 1, New York, NY USA.
[Westerveld, Donevan; Simons, Malorie; Mahadev, SriHari] Weill Cornell Med, Div Gastroenterol & Hepatol, New York Presbyterian Hosp, 1283 York Ave,9Th Floor, New York, NY 10065 USA.
C3 Cornell University; Cornell University; Weill Cornell Medicine; NewYork-Presbyterian Hospital
RP Mahadev, S (corresponding author), Weill Cornell Med, Div Gastroenterol & Hepatol, New York Presbyterian Hosp, 1283 York Ave,9Th Floor, New York, NY 10065 USA.
EM srm9005@med.cornell.edu
OI Magahis, Patrick/0000-0002-2083-3398
CR Aburajab M, 2018, GASTROINTEST ENDOSC, V87, P1248, DOI 10.1016/j.gie.2017.11.033
Anderloni A, 2016, SURG ENDOSC, V30, P5200, DOI 10.1007/s00464-016-4894-x
Cho DH, 2019, SURG ENDOSC, V33, P2135, DOI 10.1007/s00464-018-6485-5
Choi JH, 2017, GASTROINTEST ENDOSC, V85, P357, DOI 10.1016/j.gie.2016.07.067

Dollhopf M, 2017, GASTROINTEST ENDOSC, V86, P636, DOI 10.1016/j.gie.2017.02.027
Gornals JB, 2016, SURG ENDOSC, V30, P2592, DOI 10.1007/s00464-015-4505-2
James TW, 2019, ENDOSC ULTRASOUND, V8, pS28, DOI 10.4103/eus.eus_41_19
Jamwal KD, 2018, J CLIN TRANSL HEPATO, V6, P35, DOI 10.14218/JCTH.2017.00018
Jang JW, 2012, GASTROENTEROLOGY, V142, P805, DOI 10.1053/j.gastro.2011.12.051
Kim JJ, 2019, J CLIN GASTROENTEROL, V53, P392, DOI 10.1097/MCG.0000000000000890
Lisotti A, 2022, SURG ENDOSC, V36, P569, DOI 10.1007/s00464-021-08318-z
Puga M, 2018, ENDOSCOPY, V50, P1022, DOI 10.1055/a-0582-9127
Walter D, 2016, GUT, V65, P6, DOI 10.1136/gutjnl-2015-309925
NR 13
TC 2
Z9 2
U1 0
U2 0
PU SPRINGER JAPAN KK
PI TOKYO
PA SHIROYAMA TRUST TOWER 5F, 4-3-1 TORANOMON, MINATO-KU, TOKYO, 105-6005, JAPAN
SN 1865-7257
EI 1865-7265
J9 CLIN J GASTROENTEROL
JI Clin. J. Gastroenterol.
PD APR
PY 2023
VL 16
IS 2
BP 279
EP 282
DI 10.1007/s12328-022-01749-8
EA DEC 2022
PG 4
WC Gastroenterology & Hepatology
WE Emerging Sources Citation Index (ESCI)
SC Gastroenterology & Hepatology
GA C3OO7
UT WOS:000901591000002
PM 36538256
OA Green Accepted, Bronze
DA 2026-05-09
ER

PT J

AU Fujii, Y
Kato, H
Himei, H
Ueta, E
Ogawa, T
Terasawa, H
Yamazaki, T
Matsumoto, K
Horiguchi, S
Tsutsumi, K
Okada, H
AF Fujii, Yuki
Kato, Hironari
Himei, Hitomi
Ueta, Eijiro
Ogawa, Taiji
Terasawa, Hiroyuki
Yamazaki, Tatsuhiro
Matsumoto, Kazuyuki
Horiguchi, Shigeru
Tsutsumi, Koichiro
Okada, Hiroyuki
TI Double guidewire technique stabilization procedure for endoscopic ultrasound-guided hepaticogastrostomy involving modifying the guidewire angle at the insertion site
SO SURGICAL ENDOSCOPY AND OTHER INTERVENTIONAL TECHNIQUES
LA English
DT Article
DE Double guidewire; Endoscopic ultrasound-guided hepaticogastrostomy; Interventions; Endoscopic ultrasound; Biliary drainage
ID BILIARY DRAINAGE; PERCUTANEOUS DRAINAGE; PANCREATIC PSEUDOCYST; OBSTRUCTION; EXPERIENCE; STENTS; ACCESS
AB Background and aims Endoscopic ultrasonography-guided hepaticogastrostomy (EUS-HGS) is often performed using a single guidewire (SGW), but the efficacy of the double guidewire (DGW) technique during endoscopic ultrasonography-guided biliary drainage has been reported. We evaluated the efficacy of the DGW technique for EUS-HGS, focusing on the guidewire angle at the insertion site. Methods This retrospective cohort study included consecutive patients who underwent EUS-HGS between April 2012 and March 2021. We measured the guidewire angle at the insertion site using still fluoroscopic imaging. We compared the clinical outcomes of EUS-HGS with the DGW and SGW techniques. The factors associated with successful cannula insertion, need for additional fistula dilation and adverse event rate were assessed by a logistic regression multivariable analysis. Results The DGW group showed higher technical ($p = 0.020$) and clinical success rates ($p = 0.016$) than the SGW group, which showed more adverse events ($p = 0.017$) than the DGW group. Successful cannula insertion was associated with a guidewire angle > 137 degrees and an uneven double-lumen cannula. The DGW

technique made the guidewire angle obtuse at the insertion site ($p < 0.0001$). A guidewire angle <= 137 degrees (OR, 35.6; 95% CI, 1.70-744; p = 0.0045) and intrahepatic bile duct diameter of the puncture site <= 3.0 mm (OR, 14.4; 95% CI, 1.37-152; p = 0.0056) were risk factors for needing additional fistula dilation in a multivariate analysis, and additional dilation was a significant predictive factor for adverse events (OR, 8.3; 95% CI, 0.9-77; p = 0.026). Conclusions The DGW technique can modify the guidewire angle at the insertion site and facilitate stent deployment with few adverse events.
C1 [Fujii, Yuki; Kato, Hironari; Himei, Hitomi; Ueta, Eijiro; Ogawa, Taiji; Terasawa, Hiroyuki; Yamazaki, Tatsuhiro; Matsumoto, Kazuyuki; Horiguchi, Shigeru; Tsutsumi, Koichiro; Okada, Hiroyuki] Okayama Univ, Dept Gastroenterol & Hepatol, Dent & Pharmaceut Sci, Grad Sch Med,Kita Ku, 2-5-1 Shikata Cho, Okayama, Okayama 7008558, Japan.
C3 Okayama University
RP Fujii, Y (corresponding author), Okayama Univ, Dept Gastroenterol & Hepatol, Dent & Pharmaceut Sci, Grad Sch Med,Kita Ku, 2-5-1 Shikata Cho, Okayama, Okayama 7008558, Japan.
EM pmug1j9r@okayama-u.ac.jp
RI Tsutsumi, Koichiro/MGU-6876-2025
CR Adler DG, 2018, GASTROINTEST ENDOSC, V87, P617, DOI 10.1016/j.gie.2017.08.007
Ang TL, 2008, GASTROINTEST ENDOSC, V68, P192, DOI 10.1016/j.gie.2007.11.018
Chandrasekhara V, 2020, ENDOSC INT OPEN, V08, pE1639, DOI 10.1055/a-1243-0092
Cotton PB, 2010, GASTROINTEST ENDOSC, V71, P446, DOI 10.1016/j.gie.2009.10.027
Fogel EL, 2001, ENDOSCOPY, V33, P31, DOI 10.1055/s-2001-11186
Freeman ML, 2005, GASTROINTEST ENDOSC, V61, P112, DOI 10.1016/S0016-5107(04)02463-0
Giovannini M, 2001, ENDOSCOPY, V33, P898, DOI 10.1055/s-2001-17324
Hathorn KE, 2019, ENDOSC INT OPEN, V7, pE1432, DOI 10.1055/a-0990-9488
Hedjoudje A, 2019, UNITED EUR GASTROENT, V7, P60, DOI 10.1177/2050640618808147
Isayama H, 2019, J HEPATO-BIL-PAN SCI, V26, P249, DOI 10.1002/jhbp.631
Ishiwatari H, 2019, ENDOSCOPY, V51, pE321, DOI 10.1055/a-0915-1917
Itoi T, 2009, DIGEST ENDOSC, V21, pS53, DOI 10.1111/j.1443-1661.2009.00851.x
Kawakubo K, 2014, J HEPATO-BIL-PAN SCI, V21, P328, DOI 10.1002/jhbp.27
Lee TH, 2016, CLIN GASTROENTEROL H, V14, P1011, DOI 10.1016/j.cgh.2015.12.032
Manta R, 2018, EUR J GASTROEN HEPAT, V30, P695, DOI 10.1097/MEG.0000000000001112
Miller CS, 2019, ENDOSC INT OPEN, V7, pE1563, DOI 10.1055/a-0998-8129
Nakai Y, 2021, DIGEST DIS SCI, V66, P1540, DOI 10.1007/s10620-020-06345-9
Ogura T, 2021, ENDOSCOPY, V53, P369, DOI 10.1055/a-1199-5418
Oh D, 2017, THER ADV GASTROENTER, V10, P42, DOI 10.1177/1756283X16671671
Poincloux L, 2015, ENDOSCOPY, V47, P794, DOI [10.1055/s-0035-1545399, 10.1055/s-0034-1391988]
Prachayakul Varayu, 2012, J Interv Gastroenterol, V2, P202
Shiomi H, 2019, DIGEST ENDOSC, V31, pe50, DOI 10.1111/den.13328
van Delden OM, 2008, EUR RADIOL, V18, P448, DOI 10.1007/s00330-007-0796-6
Vila JJ, 2012, GASTROINTEST ENDOSC, V76, P1133, DOI 10.1016/j.gie.2012.08.001
Wang KX, 2016, GASTROINTEST ENDOSC, V83, P1218, DOI 10.1016/j.gie.2015.10.033
Yamamoto Y, 2020, ENDOSC ULTRASOUND, V9, P110, DOI 10.4103/eus.eus_68_19
NR 26
TC 20
Z9 21

U1 0
U2 1
PU SPRINGER
PI NEW YORK
PA ONE NEW YORK PLAZA, SUITE 4600, NEW YORK, NY, UNITED STATES
SN 0930-2794
EI 1432-2218
J9 SURG ENDOSC
JI Surg. Endosc.
PD DEC
PY 2022
VL 36
IS 12
BP 8981
EP 8991
DI 10.1007/s00464-022-09350-3
EA AUG 2022
PG 11
WC Surgery
WE Science Citation Index Expanded (SCI-EXPANDED)
SC Surgery
GA 6D1PZ
UT WOS:000836103500006
PM 35927355
DA 2026-05-09
ER

PT J
AU Andalib, I
Dawod, E
Kahaleh, M
AF Andalib, Iman
Dawod, Enad
Kahaleh, Michel
TI Modern Management of Pancreatic Fluid Collections
SO JOURNAL OF CLINICAL GASTROENTEROLOGY
LA English
DT Review
DE pseudocyst; pancreatic necrosis; pancreatic fluid collection; endoscopic
necrosectomy; lumen-apposing metal stent
ID PERCUTANEOUS CATHETER DRAINAGE; ACUTE NECROTIZING PANCREATITIS;
ULTRASOUND-GUIDED DRAINAGE; APPOSING METAL STENT; WALLED-OFF NECROSIS;

ENDOSCOPIC ULTRASOUND; TRANSMURAL DRAINAGE; PSEUDOCYST DRAINAGE;
SURGICAL DRAINAGE; UP APPROACH
AB The last decade has seen dramatic shift in paradigm in the management of pancreatic fluid collections with the rise of endoscopic therapy over radiologic or surgical management. Endosonographic drainage is now considered the gold standard therapy for pancreatic pseudocyst. Infected pancreatic necroses are being offered endoscopic necrosectomy that has been facilitated by the arrival on the market of large diameter lumen-apposing metal stent. Severe pancreatitis or failure to thrive should receive enteral nutrition while pancreatic ductal disruption or strictures are best treated by pancreatic stenting.
C1 [Andalib, Iman; Dawod, Enad; Kahaleh, Michel] Weill Cornell Med Coll, Dept Gastroenterol & Hepatol, New York, NY 10021 USA.
C3 Cornell University; Weill Cornell Medicine
RP Kahaleh, M (corresponding author), Weill Cornell Med Coll, 1305 York Ave,4th Floor, New York, NY 10021 USA.
EM mkahaleh@gmail.com
RI Kahaleh, Michel/A-9408-2011
FU Boston Scientific; Olympus; Xlumena Inc.; Gore
FX M.K. has received grant support from Boston Scientific, Olympus, Xlumena
Inc. and Gore. He is a consultant for Boston Scientific and Olympus. All
remaining authors have no conflicts of interest to disclose
CR ADAMS DB, 1992, ANN SURG, V215, P571, DOI 10.1097/00000658-199206000-00003
Adamson GD, 2003, SURG ENDOSC, V17
Aghdassi Alexander A, 2006, HPB (Oxford), V8, P432, DOI 10.1080/13651820600748012
Aghdassi A, 2008, PANCREAS, V36, P105, DOI 10.1097/MPA.0b013e31815a8887
Akshintala VS, 2014, GASTROINTEST ENDOSC, V79, P921, DOI 10.1016/j.gie.2013.10.032
Ang TL, 2016, ENDOSC ULTRASOUND, V5, P320, DOI 10.4103/2303-9027.191659
[Anonymous], ARCH SURG
[Anonymous], GUT
Antillon MR, 2006, GASTROINTEST ENDOSC, V63, P797, DOI 10.1016/j.gie.2005.10.025
Bakker OJ, 2012, JAMA-J AM MED ASSOC, V307, P1053, DOI 10.1001/jama.2012.276
Bang JY, 2017, DIGEST ENDOSC, V29, P83, DOI 10.1111/den.12681
Bang JY, 2014, CLIN ENDOSC, V47, P429, DOI 10.5946/ce.2014.47.5.429
Banks PA, 2013, GUT, V62, P102, DOI 10.1136/gutjnl-2012-302779
Bapaye A, 2017, DIGEST ENDOSC, V29, P104, DOI 10.1111/den.12704
Baron TH, 1996, GASTROENTEROLOGY, V111, P755, DOI 10.1053/gast.1996.v111.pm8780582
Bergman S, 2007, SURG CLIN N AM, V87, P1447, DOI 10.1016/j.suc.2007.09.003
BINMOELLER KF, 1995, GASTROINTEST ENDOSC, V42, P219, DOI 10.1016/S0016-5107(95)70095-1
Bruennler T, 2008, WORLD J GASTROENTERO, V14, P725, DOI 10.3748/wjg.14.725
Chang YC, 2006, DIGEST DIS SCI, V51, P1388, DOI 10.1007/s10620-006-9112-6
Chang YS, 2013, CRIT CARE, V17, DOI 10.1186/cc12790
Cirocchi R, 2013, SURG LAPARO ENDO PER, V23, P8, DOI 10.1097/SLE.0b013e3182754bca
Fisher Jessica M, 2013, Gastrointest Endosc Clin N Am, V23, P787, DOI 10.1016/j.giec.2013.06.013
Ge PS, 2016, GASTROENTEROL CLIN N, V45, P9, DOI 10.1016/j.gtc.2015.10.003
Gornals JB, 2016, SURG ENDOSC, V30, P2592, DOI 10.1007/s00464-015-4505-2
Heider R, 1999, ANN SURG, V229, P781, DOI 10.1097/00000658-199906000-00004

Holt BA, 2015, GASTROINTEST ENDOSC, V81, P804, DOI 10.1016/j.gie.2014.12.026
Iso Y, 2013, SURG LAPARO ENDO PER, V23, P330, DOI 10.1097/SLE.0b013e31828e3675
Kahaleh M, 2006, ENDOSCOPY, V38, P355, DOI 10.1055/s-2006-925249
KOZAREK RA, 1990, GASTROINTEST ENDOSC, V36, P93, DOI 10.1016/S0016-5107(90)70958-3
Lakhtakia S, 2017, GASTROINTEST ENDOSC, V85, P1243, DOI 10.1016/j.gie.2016.10.037
LANG EK, 1991, SOUTHERN MED J, V84, P55, DOI 10.1097/00007611-199101000-00014
Lau ST, 2001, AM J SURG, V181, P411, DOI 10.1016/S0002-9610(01)00606-7
Lee JK, 2007, PANCREAS, V34, P399, DOI 10.1097/MPA.0b013e318043c0b1
Li JY, 2013, PLOS ONE, V8, DOI 10.1371/journal.pone.0064926
Libera E D, 2000, HPB Surg, V11, P333
Lin H, 2014, DIGEST DIS SCI, V59, P59
Mortelé KJ, 2009, AM J ROENTGENOL, V192, P110, DOI 10.2214/AJR.08.1116
Morton JM, 2005, J GASTROINTEST SURG, V9, P15, DOI 10.1016/j.gassur.2004.10.005
Mukai S, 2015, ENDOSCOPY, V47, P47, DOI 10.1055/s-0034-1377966
Muthusamy VR, 2016, GASTROINTEST ENDOSC, V83, P481, DOI 10.1016/j.gie.2015.11.027
Nealon WH, 2009, J AM COLL SURGEONS, V208, P790, DOI 10.1016/j.jamcollsurg.2008.12.027
NEOPTOLEMOS JP, 1993, BRIT J SURG, V80, P94, DOI 10.1002/bjs.1800800131
Parekh D, 2006, ARCH SURG-CHICAGO, V141, P895, DOI 10.1001/archsurg.141.9.895
Park DH, 2009, ENDOSCOPY, V41, P842, DOI 10.1055/s-0029-1215133
Parks RW, 2000, ANN ROY COLL SURG, V82, P383
Penn DE, 2012, GASTROINTEST ENDOSC, V76, P679, DOI 10.1016/j.gie.2012.04.457
Rana SS, 2014, ANN GASTROENTEROL, V27, P258
Raraty MGT, 2010, ANN SURG, V251, P787, DOI 10.1097/SLA.0b013e3181d96c53
Rinninella E, 2015, GASTROINTEST ENDOSC, V82, P1039, DOI 10.1016/j.gie.2015.04.006
Seewald S, 2005, GASTROINTEST ENDOSC, V62, P92, DOI 10.1016/S0016-5107(05)00541-9
Seifert H, 2009, GUT, V58, P1260, DOI 10.1136/gut.2008.163733
Shah RJ, 2015, CLIN GASTROENTEROL H, V13, P747, DOI 10.1016/j.cgh.2014.09.047
Sharaiha RZ, 2016, CLIN GASTROENTEROL H, V14, P1797, DOI 10.1016/j.cgh.2016.05.011
Sharaiha RZ, 2015, GASTROINTEST ENDOSC, V82, P822, DOI 10.1016/j.gie.2015.02.035
Siddiqui AA, 2016, GASTROINTEST ENDOSC, V83, P699, DOI 10.1016/j.gie.2015.10.020
Singh N, 2012, PANCREAS, V41, P153, DOI 10.1097/MPA.0b013e318221c4a8
Smith MT, 1996, GASTROINTEST ENDOSC, V44, P268, DOI 10.1016/S0016-5107(96)70163-3
Talreja JP, 2008, GASTROINTEST ENDOSC, V68, P1199, DOI 10.1016/j.gie.2008.06.015
Teoh AYB, 2016, WORLD J GASTRO ENDOS, V8, P310, DOI 10.4253/wjge.v8.i6.310
Thompson CC, 2016, PANCREATOLOGY, V16, P66, DOI 10.1016/j.pan.2015.12.001
Trevino JM, 2010, J GASTROEN HEPATOL, V25, P526, DOI 10.1111/j.1440-1746.2009.06109.x
Tyberg A, 2016, WORLD J GASTROENTERO, V22, P2256, DOI 10.3748/wjg.v22.i7.2256
van Baal MC, 2011, BRIT J SURG, V98, P18, DOI 10.1002/bjs.7304
van Grinsven J, 2016, NAT REV GASTRO HEPAT, V13, P306, DOI 10.1038/nrgastro.2016.23
van Santvoort HC, 2007, WORLD J SURG, V31, P1635, DOI 10.1007/s00268-007-9083-6
van Santvoort HC, 2011, GASTROENTEROLOGY, V141, P1254, DOI 10.1053/j.gastro.2011.06.073
van Santvoort HC, 2010, NEW ENGL J MED, V362, P1491, DOI 10.1056/NEJMoa0908821

Varadarajulu S, 2005, GASTROINTEST ENDOSC, V61, P568, DOI 10.1016/S0016-5107(04)02832-9
Varadarajulu S, 2008, GASTROINTEST ENDOSC, V68, P1102, DOI 10.1016/j.gie.2008.04.028
Varadarajulu S, 2013, GASTROENTEROLOGY, V145, P583, DOI 10.1053/j.gastro.2013.05.046
Varadarajulu S, 2011, J GASTROINTEST SURG, V15, P2080, DOI 10.1007/s11605-011-1621-8
Voermans RP, 2015, J HEPATO-BIL-PAN SCI, V22, P20, DOI 10.1002/jhbp.180
Walter D, 2015, ENDOSCOPY, V47, P63, DOI 10.1055/s-0034-1378113
WARSHAW AL, 1985, ANN SURG, V202, P720, DOI 10.1097/00000658-198512000-00010
NR 74
TC 18
Z9 22
U1 0
U2 14
PU LIPPINCOTT WILLIAMS & WILKINS
PI PHILADELPHIA
PA TWO COMMERCE SQ, 2001 MARKET ST, PHILADELPHIA, PA 19103 USA
SN 0192-0790
EI 1539-2031
J9 J CLIN GASTROENTEROL
JI J. Clin. Gastroenterol.
PD FEB
PY 2018
VL 52
IS 2
BP 97
EP 104
DI 10.1097/MCG.0000000000000940
PG 8
WC Gastroenterology & Hepatology
WE Science Citation Index Expanded (SCI-EXPANDED)
SC Gastroenterology & Hepatology
GA FT9ED
UT WOS:000423453900003
PM 29095421
DA 2026-05-09
ER

PT J
AU Antunes, PB
Leal, T
Gonçalves, R
Gonçalves, B
AF Antunes, Pedro Bernardes

Leal, Tiago
Goncalves, Raquel
Goncalves, Bruno
TI EUS-Guided Gastroenterostomy for the Management of Malignant Gastric
Outlet Obstruction: A Single-Center Initial Experience
SO GE PORTUGUESE JOURNAL OF GASTROENTEROLOGY
LA English
DT Article
DE Gastric outlet obstruction; Gastroenterostomy; Lumen-apposing metal
stent; Therapeutic endoscopic ultrasound
C1 [Antunes, Pedro Bernardes; Leal, Tiago; Goncalves, Raquel; Goncalves, Bruno] Hosp Braga, Gastroenterol Dept, Braga, Portugal.
C3 Hospital de Braga
RP Antunes, PB (corresponding author), Hosp Braga, Gastroenterol Dept, Braga, Portugal.
EM pedrobba@gmail.com
RI ; Gonçalves, Raquel/KDO-3279-2024
OI Leal, Tiago/0000-0002-0604-2290; Gonçalves, Raquel/0000-0002-5930-8611
CR Carbajo AY, 2020, J CLIN GASTROENTEROL, V54, P1, DOI 10.1097/MCG.0000000000001262
Iqbal U, 2020, ENDOSC ULTRASOUND, V9, P16, DOI 10.4103/eus.eus_70_19
Khashab MA, 2015, GASTROINTEST ENDOSC, V81, P1234, DOI 10.1016/j.gie.2014.12.053
Tendler DA, 2002, AM J GASTROENTEROL, V97, P4
van der Merwe SW, 2022, ENDOSCOPY, V54, P185, DOI 10.1055/a-1717-1391
NR 5
TC 0
Z9 0
U1 0
U2 0
PU KARGER
PI BASEL
PA ALLSCHWILERSTRASSE 10, CH-4009 BASEL, SWITZERLAND
SN 2341-4545
EI 2387-1954
J9 GE PORT J GASTROENT
JI GE Port. J. Gastroenterol.
PD SEP
PY 2023
VL 30
IS SUPPL 1
SU 1
BP 61
EP 64
DI 10.1159/000527015

EA OCT 2022
PG 4
WC Gastroenterology & Hepatology
WE Emerging Sources Citation Index (ESCI)
SC Gastroenterology & Hepatology
GA R6TT3
UT WOS:000878747100001
PM 37818396
OA Green Submitted, gold
DA 2026-05-09
ER

PT J
AU Metelli, F
Pezzoli, A
Fabbri, C
Pizzo, E
Arena, R
Cifalà, V
Simone, L
Solimando, R
Merighi, A
AF Metelli, Flavio
Pezzoli, Alessandro
Fabbri, Carlo
Pizzo, Elena
Arena, Rosario
Cifala, Viviana
Simone, Loredana
Solimando, Riccardo
Merighi, Alberto
TI Combined Endoscopic Stent-In-Stent Placement by Lumen-Apposing Metal
Stents Through Self-Expanding Metal Stents for Simultaneous Malignant
Biliary and Duodenal Obstruction
SO ACG CASE REPORTS JOURNAL
LA English
DT Article
ID DRAINAGE; PALLIATION; ERCP; HEAD
AB The palliation of simultaneous biliary and duodenal obstruction in patients with advanced pancreatic cancer is a clinically and technically challenging scenario. Endoscopic procedures are a valid alternative to surgical or percutaneous transhepatic biliary drainage. The availability of self-expanding metal stents (SEMSs) and lumen-apposing metal stents (LAMS) have expanded therapeutic options. We describe a case in which biliary and duodenal obstructions were treated

successfully with the combined use of SEMS and LAMS devices. Endoscopic ultrasound-guided biliary drainage with the use of new LAMS and a duodenal SEMS can be a valid option in expert hands as a palliative and minimally invasive treatment for gastric outlet and biliary obstruction.
C1 [Metelli, Flavio; Pezzoli, Alessandro; Arena, Rosario; Cifala, Viviana; Simone, Loredana; Solimando, Riccardo; Merighi, Alberto] Univ Hosp, Dept Gastroenterol & GI Endoscopy, Ferrara, Italy.
[Fabbri, Carlo] Dept Gastroenterol & GI Endoscopy, Forli Cesena, Italy.
[Pizzo, Elena] UCL, Dept Hlth Econ, London, England.
C3 University of Ferrara; Arcispedale Sant'Anna; University of London; University College London
RP Pezzoli, A (corresponding author), Univ Hosp, Dept Gastroenterol & GI Endoscopy, Ferrara, Italy.
EM a.pezzoli@ospfe.it
RI Solimando, Riccardo/IQT-4288-2023; Fabbri, Carlo/AAC-4467-2022; Arena, Rosario/IQT-8453-2023
OI Arena, Rosario/0000-0003-1140-2374
CR De Davide Laurence, 2019, VideoGIE, V4, P131, DOI [10.1016/j.vgie.2018.11.004, 10.1016/j.vgie.2018.11.004]
Dormann A, 2004, ENDOSCOPY, V36, P543, DOI 10.1055/s-2004-814434
Hammad T., 2016, ACG 2016 ANN SCI M O
Katsinelos P, 2010, SURG LAPARO ENDO PER, V20, P410, DOI 10.1097/SLE.0b013e3182001f26
Khan MA, 2016, DIGEST DIS SCI, V61, P684, DOI 10.1007/s10620-015-3933-0
Khashab MA, 2014, ENDOSCOPY, V46, P251, DOI 10.1055/s-0033-1359214
Kunda R, 2016, SURG ENDOSC, V30, P5002, DOI 10.1007/s00464-016-4845-6
Lisotti A, 2022, SURG ENDOSC, V36, P569, DOI 10.1007/s00464-021-08318-z
Maire F, 2006, AM J GASTROENTEROL, V101, P735, DOI 10.1111/j.1572-0241.2006.00559.x
Mutignani M, 2007, ENDOSCOPY, V39, P440, DOI 10.1055/s-2007-966327
Rimbas Mihai, 2017, Gastrointest Endosc Clin N Am, V27, P681, DOI [10.1016/j.giec.2017.06.006, 10.1016/j.giec.2017.06.006]
Rinninella E, 2015, GASTROINTEST ENDOSC, V82, P1039, DOI 10.1016/j.gie.2015.04.006
Sharaiha RZ, 2017, GASTROINTEST ENDOSC, V85, P904, DOI 10.1016/j.gie.2016.12.023
SHEPHERD HA, 1988, BRIT J SURG, V75, P1166, DOI 10.1002/bjs.1800751207
SMITH AC, 1994, LANCET, V344, P1655, DOI 10.1016/S0140-6736(94)90455-3
Sportes A, 2018, ENDOSC INT OPEN, V6, pE1395, DOI 10.1055/a-0735-9254
Sportes A, 2017, THER ADV GASTROENTER, V10, P483, DOI 10.1177/1756283X17702096
Téllez-Avila FI, 2018, SURG LAPARO ENDO PER, V28, P183, DOI 10.1097/SLE.0000000000000528
van Hooft JE, 2009, GASTROINTEST ENDOSC, V69, P1059, DOI 10.1016/j.gie.2008.07.026
NR 19
TC 0
Z9 0
U1 0
U2 0
PU LIPPINCOTT WILLIAMS & WILKINS
PI PHILADELPHIA
PA TWO COMMERCE SQ, 2001 MARKET ST, PHILADELPHIA, PA 19103 USA

EI 2326-3253
J9 ACG CASE REP J
JI ACG Case Rep. J.
PD NOV
PY 2021
VL 8
IS 11
AR e00704
DI 10.14309/crj.0000000000000704
PG 4
WC Gastroenterology & Hepatology
WE Emerging Sources Citation Index (ESCI)
SC Gastroenterology & Hepatology
GA XB7WM
UT WOS:000721535500001
PM 34841002
OA Green Submitted, gold
DA 2026-05-09
ER

PT J
AU Auriemma, F
Fugazza, A
Colombo, M
Spadaccini, M
Repici, A
Anderloni, A
AF Auriemma, Francesco
Fugazza, Alessandro
Colombo, Matteo
Spadaccini, Marco
Repici, Alessandro
Anderloni, Andrea
TI Safety issues in endoscopy ultrasound-guided interventions using lumen
apposing metal stents
SO MINERVA GASTROENTEROLOGY
LA English
DT Review
DE Gallbladder emptying; Ultrasonography; Choledochostomy; Stents; Safety
ID PANCREATIC FLUID COLLECTIONS; GALLBLADDER DRAINAGE; MALIGNANT BILIARY;
NECROTIZING PANCREATITIS; MANAGEMENT; MULTICENTER; FEASIBILITY;
OBSTRUCTION; NECROSIS; GASTROENTEROSTOMY

AB Lumen-apposing metal stents (LAMS) are recently introduced devices that allow to create anastomoses. The indications for LAMS placement are increasing and currently these stents can be used in multiple clinical situations such as endoscopic drainage of pancreatic fluid collections (PFCs), endoscopic ultrasound-guided choledochoduodenostomy (EUSCDS), endoscopic ultrasound gallbladder drainage (EUS-GBD), endoscopic ultrasound gastrojejunostomy (EUS-GJ). Since their introduction, they have revealed to allow a higher rate of technical and clinical success, and potentially lower risk of adverse events (AEs) compared with previously available devices. Despite ongoing innovation, any advanced intervention carries risks of AEs. These AEs may occur during the deployment or can be delayed because of the consequent effects of the procedure and/or stent itself. This review represents an overview of current indications, technical and clinical results, with an extensive focus on safety issues related to placement and on long-term indwelling related ones. (Cite this article as: Auriemma F, Fugazza A, Colombo M, Spadaccini M, Repici A, Anderloni A. Safety issues in endoscopy ultrasound-guided interventions using lumen apposing metal stents. Minerva Gastroenterol 2022;68:177-85. DOI: 10.23736/ S2724-5985.21.02862-X)
C1 [Auriemma, Francesco] Humanitas Mater Domini, Digest Endoscopy Unit, Castellanza, Varese, Italy.
[Fugazza, Alessandro; Colombo, Matteo; Spadaccini, Marco; Repici, Alessandro; Anderloni, Andrea] Humanitas Clin & Res Ctr IRCCS, Div Gastroenterol, Digest Endoscopy Unit, Milan, Italy.
[Fugazza, Alessandro; Colombo, Matteo; Spadaccini, Marco; Repici, Alessandro; Anderloni, Andrea] Humanitas Univ, Dept Biomed Sci, Milan, Italy.
C3 Humanitas University
RP Anderloni, A (corresponding author), Humanitas Res Hosp, Div Gastroenterol, Digest Endoscopy Unit, Rozzano, MI, Italy.
EM andrea.anderloni@humanitas.it
RI Fugazza, Alessandro/ABG-9381-2021; Repici, Alessandro/HFH-8162-2022;
Colombo, Matteo/N-9849-2013; Spadaccini, Marco/HOH-7613-2023; Anderloni, Andrea/ABE-9139-2020
OI Fugazza, Alessandro/0000-0003-0485-4903; Repici, Alessandro/0000-0002-1621-6450; Colombo, Matteo/0000-0003-0715-8233; Spadaccini, Marco/0000-0003-3909-9012;
CR Amateau SK, 2018, OBES SURG, V28, P1445, DOI 10.1007/s11695-018-3171-6
Amateau Stuart K, 2018, Gastrointest Endosc Clin N Am, V28, P219, DOI [10.1016/j.giec.2017.11.009, 10.1016/j.giec.2017.11.009]
Amato A, 2021, ENDOSCOPY, V53, P1037, DOI 10.1055/a-1324-7919
Anderloni A, 2021, SURG ENDOSC, V35, P1741, DOI 10.1007/s00464-020-07567-8
Anderloni A, 2019, WORLD J GASTROENTERO, V25, P3857, DOI 10.3748/wjg.v25.i29.3857
Anderloni A, 2018, ANN GASTROENTEROL, V31, P735, DOI 10.20524/aog.2018.0299
Anderloni A, 2019, GASTROINTEST ENDOSC, V89, P69, DOI [10.1016/j.gie.2018.04.1746, 10.1016/j.gie.2018.08.047]
Anderloni A, 2017, ENDOSC INT OPEN, V5, pE1100, DOI 10.1055/s-0043-118828
Anderloni A, 2016, SURG ENDOSC, V30, P5200, DOI 10.1007/s00464-016-4894-x
Anderloni A, 2016, ENDOSCOPY, V48, pE321, DOI 10.1055/s-0042-117425
Arvanitakis M, 2018, ENDOSCOPY, V50, P524, DOI 10.1055/a-0588-5365
Auriemma Francesco, 2018, Am J Gastroenterol, V113, P1582, DOI [10.1038/s41395-018-0266-6, 10.1038/s41395-018-0266-6]

Bang JY, 2017, GUT, V66, P2054, DOI 10.1136/gutjnl-2016-312812
Bang JY, 2019, DIGEST ENDOSC
Bapaye A, 2015, DIGEST ENDOSC, V27, P499, DOI 10.1111/den.12421
Chandrasekhara V, 2020, ENDOSC INT OPEN, V08, pE1639, DOI 10.1055/a-1243-0092
Cotton PB, 2010, GASTROINTEST ENDOSC, V71, P446, DOI 10.1016/j.gie.2009.10.027
De Angelis CG, 2019, SAUDI J GASTROENTERO, V25, P335, DOI 10.4103/sjg.SJG_166_19
Dhir V, 2018, ENDOSCOPY, V50, P22, DOI [10.1055/s-0043-118592, 10.1055/s-0043-123575]
Dollhopf M, 2017, GASTROINTEST ENDOSC, V86, P636, DOI 10.1016/j.gie.2017.02.027
Fugazza A, 2021, DIGEST LIVER DIS, V53, P8, DOI 10.1016/j.dld.2020.09.014
Fugazza A, 2020, ENDOSC INT OPEN, V08, pE1435, DOI 10.1055/a-1221-5183
Fugazza A, 2020, AM J GASTROENTEROL, V115, P811, DOI 10.14309/ajg.0000000000000685
Fugazza A, 2020, CLIN EXP GASTROENTER, V13, P193, DOI 10.2147/CEG.S203626
Fugazza A, 2020, ENDOSCOPY, V52, pE430, DOI 10.1055/a-1158-8749
Fugazza A, 2020, GASTROINTEST ENDOSC, V91, P574, DOI 10.1016/j.gie.2019.11.021
Fusaroli P, 2015, ENDOSC ULTRASOUND, V4, P336, DOI 10.4103/2303-9027.170427
Fusaroli P, 2012, DIGEST DIS SCI, V57, P602, DOI 10.1007/s10620-011-1961-y
Gulati R, 2021, ENDOSCOPY, V53, P72, DOI 10.1055/a-1192-3219
Guo JT, 2018, ENDOSC ULTRASOUND, V7, P356, DOI 10.4103/eus.eus_53_18
Guo JT, 2017, ENDOSC ULTRASOUND, V6, P285, DOI 10.4103/eus.eus_85_17
Han D, 2018, J CLIN GASTROENTEROL, V52, P835, DOI 10.1097/MCG.0000000000000934
Issa D, 2021, ENDOSCOPY, V53, P827, DOI 10.1055/a-1259-0349
Garcia-Alonso FJ, 2018, ENDOSCOPY, V50, P386, DOI 10.1055/a-0581-9040
Kamata K, 2018, DIGEST ENDOSC, V30, P98, DOI 10.1111/den.12900
Khashab MA, 2017, ENDOSC INT OPEN, V5, pE275, DOI 10.1055/s-0043-101695
Krishnamoorthi R, 2020, SURG ENDOSC, V34, P2866, DOI 10.1007/s00464-020-07484-w
Ligresti Dario, 2020, VideoGIE, V5, P154, DOI [10.1016/j.vgie.2019.12.011, 10.1016/j.vgie.2019.12.011]
Lisotti A, 2020, ENDOSCOPY, V52, P517, DOI [10.1055/a-1065-1678, 10.1055/a-1157-6615, 10.1055/a-1065-1678]
Mangiavillano B, 2020, AM J GASTROENTEROL, V115, P1572, DOI 10.14309/ajg.0000000000000740
Mussetto A, 2018, ANN GASTROENTEROL, V31, P535, DOI 10.20524/aog.2018.0287
Ngamruengphong S, 2017, ENDOSCOPY, V49, P549, DOI 10.1055/s-0043-105072
Paik WH, 2018, AM J GASTROENTEROL, V113, P987, DOI 10.1038/s41395-018-0122-8
Parsa N, 2020, ENDOSCOPY, V52, P211, DOI 10.1055/a-1096-3299
Sagami R, 2020, ANN GASTROENTEROL, V33, P391, DOI 10.20524/aog.2020.0496
Samuelson AL, 2012, GASTROENTEROL CLIN N, V41, P47, DOI 10.1016/j.gtc.2011.12.007
Sharaiha RZ, 2016, CLIN GASTROENTEROL H, V14, P1797, DOI 10.1016/j.cgh.2016.05.011
Siddiqui AA, 2016, GASTROINTEST ENDOSC, V83, P699, DOI 10.1016/j.gie.2015.10.020
Teoh AYB, 2020, GUT, V69, P1085, DOI 10.1136/gutjnl-2019-319996
Teoh AYB, 2021, GASTROINTEST ENDOSC, V93, P577, DOI 10.1016/j.gie.2020.06.066
Yuste RT, 2019, ANN GASTROENTEROL, V32, P514, DOI 10.20524/aog.2019.0395
Troncone E, 2020, WORLD J GASTROENTERO, V26, P1847, DOI 10.3748/wjg.v26.i16.1847
Tyberg A, 2016, ENDOSC INT OPEN, V4, pE276, DOI 10.1055/s-0042-101789
van Brunschot S, 2014, SURG ENDOSC, V28, P1425, DOI 10.1007/s00464-013-3382-9

Vozzo Catherine Frakes, 2020, VideoGIE, V5, P500, DOI [10.1016/j.vgie.2020.07.009, 10.1016/j.vgie.2020.07.009]
Walter D, 2015, ENDOSCOPY, V47, P63, DOI 10.1055/s-0034-1378113
NR 56
TC 4
Z9 4
U1 0
U2 0
PU EDIZIONI MINERVA MEDICA
PI TURIN
PA CORSO BRAMANTE 83-85 INT JOURNALS DEPT., 10126 TURIN, ITALY
SN 2724-5985
EI 2724-5365
J9 MINERVA GASTROENTERO
JI Minerva Gastroenterol.
PD JUN
PY 2022
VL 68
IS 2
BP 177
EP 185
DI 10.23736/S2724-5985.21.02862-X
PG 9
WC Gastroenterology & Hepatology
WE Science Citation Index Expanded (SCI-EXPANDED)
SC Gastroenterology & Hepatology
GA 2P1CA
UT WOS:000819487200006
PM 33871222
DA 2026-05-09
ER

PT J
AU Dollhopf, M
Larghi, A
Will, U
Rimbas, M
Anderloni, A
Sanchez-Yague, A
Teoh, AYB
Kunda, R
AF Dollhopf, Markus
Larghi, Alberto

Will, Uwe
Rimbas, Mihai
Anderloni, Andrea
Sanchez-Yague, Andres
Teoh, Anthony Yuen Bun
Kunda, Rastislav
TI EUS-guided gallbladder drainage in patients with acute cholecystitis and
high surgical risk using an electrocautery-enhanced lumen-apposing metal
stent device
SO GASTROINTESTINAL ENDOSCOPY
LA English
DT Article
ID PANCREATIC FLUID COLLECTIONS; ENDOSCOPIC ULTRASOUND; DELIVERY-SYSTEM;
PERCUTANEOUS CHOLECYSTOSTOMY; OUTCOMES; CHOLECYSTECTOMY; FEASIBILITY;
PLACEMENT; VIDEOS
AB Background and Aims: In high-risk surgical patients, the treatment of choice of acute cholecystitis is percutaneous transhepatic gallbladder drainage (PTGBD). Recently, a novel endoscopic device containing a lumen-apposing metal stent with an electrocautery (ECE-LAMS) on the tip has been developed.

Methods: High-risk surgical patients with acute cholecystitis who underwent EUS-guided gallbladder drainage (EUS-GBD) with the novel device were retrospectively retrieved from 7 tertiary care referral centers. Main endpoints were technical and clinical success rates, rate of procedural adverse events, and short-and long-term adverse events.

Results: Seventy-five patients (mean age, 75 +/- 11 years; 36 men) underwent EUS-GBD. The procedure was technically and clinically successful in 98.7% and 95.9% of cases, respectively. Three patients without resolution of cholecystitis died, and 2 patients had procedure-related adverse events: 1 perforation requiring surgery and 1 major bleeding resolved conservatively. The mean follow-up for the entire cohort was 201 +/- 226 days. Seven patients (9.6%) died within the first 30 days; 50 patients (71.4%) were alive at the last date of follow-up. Short-and long-term adverse events occurred in 6 patients: 3 had recurrent cholecystitis, 2 had migration of the stent, and 1 developed Bouveret syndrome, all managed nonsurgically. Overall, 8 adverse events (10.7%) occurred in the entire cohort of patients.

Conclusions: The novel ECE-LAMS for high-risk surgical patients with acute cholecystitis is safe, with a high technical and clinical success rate. Future multicenter studies comparing EUS-GBD versus PTGBD are warranted to determine which procedure is safer and clinically more effective for patients with high surgical risk acute cholecystitis.
C1 [Dollhopf, Markus] Klinikum Neuperlach, Dept Gastroenterol, Munich, Germany.
[Larghi, Alberto; Rimbas, Mihai] Catholic Univ, Digest Endoscopy Unit, Rome, Italy.
[Will, Uwe] Municipal Hosp, Dept Gastroenterol, Gera, Germany.
[Rimbas, Mihai] Carol Davila Univ Med, Colentina Clin Hosp, Dept Gastroenterol, Bucharest, Romania.
[Rimbas, Mihai] Carol Davila Univ Med, Colentina Clin Hosp, Dept Internal Med, Bucharest, Romania.
[Anderloni, Andrea] Humanitas Res Hosp, Dept Gastroenterol, Digest Endoscopy Unit, Rozzano, Italy.
[Sanchez-Yague, Andres] Hosp Costa del Sol, Endoscopy Unit, Marbella, Spain.
[Teoh, Anthony Yuen Bun] Prince Wales Hosp, Dept Surg, Shatin, Hong Kong, Peoples R China.
[Kunda, Rastislav] L Aarhus Univ Hosp, Dept Surg Gastroenterol, Aarhus, Denmark.
C3 Munchen Klinik; Catholic University of the Sacred Heart; IRCCS
Policlinico Gemelli; Carol Davila University of Medicine & Pharmacy;

Carol Davila University of Medicine & Pharmacy; IRCCS Humanitas Research Hospital; Prince of Wales Hospital Hong Kong; Aarhus University
RP Dollhopf, M (corresponding author), Klinikum Neuperlach, Klin Gastroenterol & Hepatol, Oskar Maria Graf Ring 51, D-81737 Munich, Germany.
RI Rimbas, Mihai/AAF-5521-2020; Kunda, Rastislav/N-1775-2019; Anderloni, Andrea/ABE-9139-2020; Larghi, Alberto/AAC-7448-2022; Teoh, Anthony/L-4796-2016
OI Anderloni, Andrea/0000-0002-1021-0031; Larghi, Alberto/0000-0001-7131-3686;
CR Anderloni A, 2017, ENDOSC INT OPEN, V5, pE25, DOI 10.1055/s-0042-122009
Anderloni A, 2016, SURG ENDOSC, V30, P5200, DOI 10.1007/s00464-016-4894-x
Attili F, 2015, ENDOSCOPY, V47, pE619, DOI 10.1055/s-0034-1393669
Bang JY, 2016, GUT, V0, P1
Baron TH, 2015, N ENGL J MED, V373, P357
Baron TH, 2007, GASTROINTEST ENDOSC, V65, P735, DOI 10.1016/j.gie.2006.07.041
Binmoeller KF, 2011, ENDOSCOPY, V43, P337, DOI 10.1055/s-0030-1256127
Súbtil JC, 2010, WORLD J GASTRO ENDOS, V2, P203, DOI 10.4253/wjge.v2.i6.203
Choi JH, 2014, ENDOSCOPY, V46, P656, DOI 10.1055/s-0034-1365720
Fuccio Lorenzo, 2014, Curr Treat Options Gastroenterol, V12, P183, DOI 10.1007/s11938-014-0015-x
Ge N, 2016, BMC GASTROENTEROL, V16, DOI 10.1186/s12876-016-0420-9
Irani S, 2015, GASTROINTEST ENDOSC, V82, P1110, DOI 10.1016/j.gie.2015.05.045
Itoi T, 2012, GASTROINTEST ENDOSC, V75, P870, DOI 10.1016/j.gie.2011.10.020
Jang JW, 2012, GASTROENTEROLOGY, V142, P805, DOI 10.1053/j.gastro.2011.12.051
Jang JW, 2011, GASTROINTEST ENDOSC, V74, P176, DOI 10.1016/j.gie.2011.03.1120
Kamata K, 2009, ENDOSCOPY, V41, pE315, DOI 10.1055/s-0029-1215258
Kunda R, 2016, SURG ENDOSC, V30, P5002, DOI 10.1007/s00464-016-4845-6
Kwan V, 2007, GASTROINTEST ENDOSC, V66, P582, DOI 10.1016/j.gie.2007.02.065
Larghi A, 2016, AM J GASTROENTEROL, V111, P1516, DOI 10.1038/ajg.2016.258
Lee SS, 2007, GASTROINTEST ENDOSC, V66, P1008, DOI 10.1016/j.gie.2007.03.1080
Lee TH, 2011, ENDOSCOPY, V43, P702, DOI 10.1055/s-0030-1256226
Peñas-Herrero I, 2015, J HEPATO-BIL-PAN SCI, V22, P35, DOI 10.1002/jhbp.182
Rinninella E, 2015, GASTROINTEST ENDOSC, V82, P1039, DOI 10.1016/j.gie.2015.04.006
Sanjay P, 2013, HPB, V15, P511, DOI 10.1111/j.1477-2574.2012.00610.x
Teoh AYB, 2017, ENDOSCOPY, V49, P130, DOI 10.1055/s-0042-119036
Teoh AYB, 2014, GASTROINTEST ENDOSC, V80, P1171, DOI 10.1016/j.gie.2014.03.038
Tsuyuguchi T, 2013, J HEPATO-BIL-PAN SCI, V20, P81, DOI 10.1007/s00534-012-0570-2
Walter D, 2016, GUT, V65, P6, DOI 10.1136/gutjnl-2015-309925
Walter D, 2015, ENDOSCOPY, V47, P63, DOI 10.1055/s-0034-1378113
Wang AY, 2009, ENDOSCOPY, V41, P781, DOI 10.1055/s-0029-1215050
Winbladh A, 2009, HPB, V11, P183, DOI 10.1111/j.1477-2574.2009.00052.x
Xu MM, 2016, THER ADV GASTROENTER, V9, P138, DOI 10.1177/1756283X15618178
Yokoe M, 2013, J HEPATO-BIL-PAN SCI, V20, P35, DOI 10.1007/s00534-012-0568-9

NR 33
TC 137
Z9 159
U1 0
U2 7
PU MOSBY-ELSEVIER
PI NEW YORK
PA 360 PARK AVENUE SOUTH, NEW YORK, NY 10010-1710 USA
SN 0016-5107
EI 1097-6779
J9 GASTROINTEST ENDOSC
JI Gastrointest. Endosc.
PD OCT
PY 2017
VL 86
IS 4
BP 636
EP 643
DI 10.1016/j.gie.2017.02.027
PG 8
WC Gastroenterology & Hepatology
WE Science Citation Index Expanded (SCI-EXPANDED)
SC Gastroenterology & Hepatology
GA FG7SN
UT WOS:000410624300011
PM 28259594
DA 2026-05-09
ER

PT J
AU Ge, PS
Young, JY
Jirapinyo, P
Dong, W
Ryou, M
Thompson, CC
AF Ge, Phillip S.
Young, Joyce Y.
Jirapinyo, Pichamol
Dong, William
Ryou, Marvin
Thompson, Christopher C.

TI Comparative Study Evaluating Lumen Apposing Metal Stents Versus Double
Pigtail Plastic Stents for Treatment of Walled-Off Necrosis

SO PANCREAS
LA English
DT Article
DE lumen apposing metal stent; LAMS; cystoenterostomy; necrosectomy;
walled-off necrosis; therapeutic endoscopic ultrasound
ID PANCREATIC-FLUID COLLECTIONS; EUS-GUIDED DRAINAGE; STEP-UP APPROACH;
INFECTED NECROTIZING PANCREATITIS; ENDOSCOPIC DRAINAGE;
CLINICAL-OUTCOMES; NECROSECTOMY; MANAGEMENT; PSEUDOCYSTS; EFFICACY

AB Objectives

Lumen-apposing metal stents (LAMSs) are increasingly used for direct endoscopic necrosectomy (DEN) in the management of walled-off necrosis (WON). We compared LAMS and traditional cystoenterostomy in the management of WON.

Methods

Retrospective analysis of patients who underwent DEN for management of WON was performed. Primary outcome was rate of WON resolution. Secondary outcomes included technical and clinical success, time and number of procedures until resolution, requirement for alternative therapy, recurrence, and adverse events.

Results

One hundred twelve patients underwent DEN with LAMS (n = 34) or traditional cystoenterostomy (n = 78). Mean WON size was 90.2 x 60.1 mm, and 61.8% had infected necrosis. Overall WON resolution was similar between LAMS and traditional cystoenterostomy (94.1% vs 92.1%, P = 0.510), with similar number of procedures until resolution (1.5 vs 1.5, P = 0.871). The LAMSs were associated with faster resolution (86.9 vs 133.6 days, P = 0.038), lower recurrence (6.3% vs 23.1%, P = 0.032), and decreased requirement for surgery (0% vs 12.8%, P = 0.031) compared with traditional cystoenterostomy, but higher adverse event rates (41.2% vs 7.7%, P < 0.001).

Conclusions

Despite higher adverse event rates, initial LAMS cystoenterostomy for management of WON results in faster resolution, lower recurrence, and decreased requirement for surgery.

C1 [Ge, Phillip S.; Young, Joyce Y.; Jirapinyo, Pichamol; Dong, William; Ryou, Marvin; Thompson, Christopher C.] Harvard Med Sch, Dev Endoscopy Lab, Boston, MA 02115 USA.

[Ge, Phillip S.; Jirapinyo, Pichamol; Ryou, Marvin; Thompson, Christopher C.] Brigham & Womens Hosp, Div Gastroenterol Hepatol & Endoscopy, 75 Francis St, Boston, MA 02115 USA.

C3 Harvard University; Harvard Medical School; Harvard University; Harvard
University Medical Affiliates; Brigham & Women's Hospital

RP Thompson, CC (corresponding author), Brigham & Womens Hosp, Div Gastroenterol Hepatol & Endoscopy, Dev Endoscopy, 75 Francis St, Boston, MA 02115 USA.

EM ccthompson@bwh.harvard.edu

FU National Institute of Diabetes and Digestive and Kidney Diseases
[P30DK034854] Funding Source: NIH RePORTER; NIDDK NIH HHS [P30 DK034854,
T32 DK007533] Funding Source: Medline

CR Abu Dayyeh BK, 2018, GASTROINTEST ENDOSC, V87, P141, DOI 10.1016/j.gie.2017.04.032
Bakker OJ, 2012, JAMA-J AM MED ASSOC, V307, P1053, DOI 10.1001/jama.2012.276

Banks PA, 2013, GUT, V62, P102, DOI 10.1136/gutjnl-2012-302779
Baron TH, 2002, GASTROINTEST ENDOSC, V56, P7, DOI 10.1067/mge.2002.125106
Bazerbachi F, 2018, GASTROINTEST ENDOSC, V87, P30, DOI 10.1016/j.gie.2017.08.025
Ge PS, 2016, GASTROENTEROL CLIN N, V45, P9, DOI 10.1016/j.gtc.2015.10.003
Gluck M, 2010, CLIN GASTROENTEROL H, V8, P1083, DOI 10.1016/j.cgh.2010.09.010
Gornals JB, 2013, SURG ENDOSC, V27, P1428, DOI 10.1007/s00464-012-2591-y
Hookey LC, 2006, GASTROINTEST ENDOSC, V63, P635, DOI 10.1016/j.gie.2005.06.028
Itoi T, 2012, GASTROINTEST ENDOSC, V75, P870, DOI 10.1016/j.gie.2011.10.020
Khan MA, 2019, J CLIN GASTROENTEROL, V53, P81, DOI 10.1097/MCG.0000000000001141
Kumar N, 2014, PANCREAS, V43, P1334, DOI 10.1097/MPA.0000000000000213
Lang GD, 2018, GASTROINTEST ENDOSC, V87, P150, DOI 10.1016/j.gie.2017.06.029
Rinninella E, 2015, GASTROINTEST ENDOSC, V82, P1039, DOI 10.1016/j.gie.2015.04.006
Shah RJ, 2015, CLIN GASTROENTEROL H, V13, P747, DOI 10.1016/j.cgh.2014.09.047
Sharaiha RZ, 2016, CLIN GASTROENTEROL H, V14, P1797, DOI 10.1016/j.cgh.2016.05.011
Sharaiha RZ, 2015, GASTROINTEST ENDOSC, V82, P822, DOI 10.1016/j.gie.2015.02.035
Siddiqui AA, 2017, GASTROINTEST ENDOSC, V85, P758, DOI 10.1016/j.gie.2016.08.014
Siddiqui AA, 2016, GASTROINTEST ENDOSC, V83, P699, DOI 10.1016/j.gie.2015.10.020
Talreja JP, 2008, GASTROINTEST ENDOSC, V68, P1199, DOI 10.1016/j.gie.2008.06.015
Thompson CC, 2016, PANCREATOLOGY, V16, P66, DOI 10.1016/j.pan.2015.12.001
van Brunschot S, 2018, LANCET, V391, P51, DOI 10.1016/S0140-6736(17)32404-2
van Santvoort HC, 2010, NEW ENGL J MED, V362, P1491, DOI 10.1056/NEJMoa0908821
Varadarajulu S, 2011, AM SURGEON, V77, P1650
Varadarajulu S, 2011, GASTROINTEST ENDOSC, V74, P74, DOI 10.1016/j.gie.2011.03.1122
Vazquez-Sequeiros E, 2016, GASTROINTEST ENDOSC, V84, P450, DOI 10.1016/j.gie.2016.02.044
Walter D, 2015, ENDOSCOPY, V47, P63, DOI 10.1055/s-0034-1378113
NR 27
TC 25
Z9 29
U1 0
U2 3
PU LIPPINCOTT WILLIAMS & WILKINS
PI PHILADELPHIA
PA TWO COMMERCE SQ, 2001 MARKET ST, PHILADELPHIA, PA 19103 USA
SN 0885-3177
EI 1536-4828
J9 PANCREAS
JI Pancreas
PD FEB
PY 2020
VL 49
IS 2
BP 236

EP 241
DI 10.1097/MPA.0000000000001476
PG 6
WC Gastroenterology & Hepatology
WE Science Citation Index Expanded (SCI-EXPANDED)
SC Gastroenterology & Hepatology
GA KP9ZH
UT WOS:000516589700014
PM 31972728
OA Green Submitted
DA 2026-05-09
ER

PT J
AU Ogura, T
Higuchi, K
AF Ogura, Takeshi
Higuchi, Kazuhide
TI Endoscopic Ultrasound-Guided Hepaticogastrostomy: Technical Review and Tips to Prevent Adverse Events
SO GUT AND LIVER
LA English
DT Review
DE Endoscopic retrograde cholangiopancreatography; Endoscopic ultrasound-guided biliary drainage; Drainage; Endoscopic ultrasound
ID MALIGNANT BILIARY OBSTRUCTION; PANCREATIC-DUCT DRAINAGE; STENT MIGRATION; METAL STENT; FAILED ERCP; ELECTROCAUTERY DILATOR; CHOLEDOCHODUODENOSTOMY; REINTERVENTION; RESCUE; LONG
AB Most patients who require biliary drainage can be treated by endoscopic retrograde cholangiopancreatography (ERCP)-guided procedures. However, ERCP can be challenging in patients with complications, such as malignant duodenal obstruction, or a surgically-altered anatomy, such as a Roux-en-Y anastomosis, which prevent advancement of the duodenoscope into the ampulla of Vater. Recently, endoscopic ultrasound (EUS)-guided biliary drainage via transhepatic or transduodenal approaches has emerged as an alternative means of biliary drainage. Typically, EUS-guided gallbladder drainage or choledochoduodenostomy can be performed via both approaches, as can EUS-guided hepaticogastrostomy (HGS). EUS-HGS, because of its transgastric approach, can be performed in patients with malignant duodenal obstruction. Technical tips for EUS-HGS have reached maturity due to device and technical developments. Although the technical success rates of EUS-HGS are high, the rate of adverse events is not low, with stent migration still being reported despite many preventive efforts. In this review, we described technical tips for EUS-HGS related to bile duct puncture, guidewire insertion, fistula dilation, and stent deployment, along with a literature review. Additionally, we provided technical tips to improve the technical success of EUS-HGS.
C1 [Ogura, Takeshi; Higuchi, Kazuhide] Osaka Med Coll, Dept Internal Med 2, Osaka, Japan.
C3 Osaka Medical & Pharmaceutical University

RP Ogura, T (corresponding author), Osaka Med Coll, Dept Internal Med 2, Osaka, Japan.

EM oguratakeshi0411@yahoo.co.jp

RI Ogura, Takeshi/AAU-5643-2021

CR Adler DG, 2018, GASTROINTEST ENDOSC, V87, P617, DOI 10.1016/j.gie.2017.08.007

Amano M, 2017, J GASTROEN HEPATOL, V32, P716, DOI 10.1111/jgh.13489

Artifon ELA, 2015, GASTROINTEST ENDOSC, V81, P950, DOI 10.1016/j.gie.2014.09.047

Bories E, 2007, ENDOSCOPY, V39, P287, DOI 10.1055/s-2007-966212

Cho DH, 2017, GASTROINTEST ENDOSC, V85, P1067, DOI 10.1016/j.gie.2016.09.010

Fogel EL, 2001, ENDOSCOPY, V33, P31, DOI 10.1055/s-2001-11186

Hamada T, 2013, DIGEST ENDOSC, V25, P340, DOI 10.1111/den.12035

Honjo M, 2018, ENDOSC ULTRASOUND, V7, P376, DOI 10.4103/eus.eus_2_18

De Moura DTH, 2016, GASTROINTEST ENDOSC, V83, P656, DOI 10.1016/j.gie.2015.09.005

Kamata K, 2017, ARAB J GASTROENTEROL, V18, P120, DOI 10.1016/j.ajg.2017.05.016

Kaneko J, 2020, ENDOSCOPY, V52, P519, DOI 10.1055/a-1065-1726

Kanno Y, 2019, J GASTROINTEST CANC, V50, P469, DOI 10.1007/s12029-018-0096-1

Kawakubo K, 2014, J HEPATO-BIL-PAN SCI, V21, P328, DOI 10.1002/jhbp.27

Khashab MA, 2016, ENDOSC INT OPEN, V4, pE175, DOI 10.1055/s-0041-109083

Martins FP, 2010, ENDOSCOPY, V42, pE126, DOI 10.1055/s-0029-1243911

Minaga K, 2019, DIGEST ENDOSC, V31, P575, DOI 10.1111/den.13406

Minaga K, 2017, SURG ENDOSC, V31, P4764, DOI 10.1007/s00464-017-5553-6

Minaga K, 2017, GASTROINTEST ENDOSC, V85, P263, DOI 10.1016/j.gie.2016.03.016

Minaga K, 2016, ENDOSCOPY, V48, pE369, DOI 10.1055/s-0042-117714

Miyano A, 2018, J GASTROINTEST SURG, V22, P1312, DOI 10.1007/s11605-018-3758-1

Moryoussef F, 2017, THER ADV GASTROENTER, V10, P537, DOI 10.1177/1756283X17702614

Nakai Y, 2016, ENDOSCOPY, V48, P1125, DOI 10.1055/s-0042-116595

Ogura T, 2020, J GASTROEN HEPATOL, V35, P707, DOI 10.1111/jgh.14895

Ogura T, 2019, ENDOSC INT OPEN, V7, pE1652, DOI 10.1055/a-0961-7890

Ogura T, 2019, DIGEST LIVER DIS, V51, P247, DOI 10.1016/j.dld.2018.09.015

Ogura T, 2018, DIGEST ENDOSC, V30, P252, DOI 10.1111/den.12976

Ogura T, 2017, J GASTROEN HEPATOL, V32, P163, DOI 10.1111/jgh.13437

Ogura T, 2016, WORLD J GASTROENTERO, V22, P3945, DOI 10.3748/wjg.v22.i15.3945

Ogura T, 2016, ENDOSCOPY, V48, P156, DOI 10.1055/s-0034-1392859

Ogura T, 2015, J GASTROEN HEPATOL, V30, P1748, DOI 10.1111/jgh.13021

Ogura T, 2015, ENDOSCOPY, V47, pE583, DOI 10.1055/s-0034-1393381

Oh D, 2017, THER ADV GASTROENTER, V10, P42, DOI 10.1177/1756283X16671671

Okuno N, 2018, J GASTROEN HEPATOL, V33, P1413, DOI 10.1111/jgh.14112

Paik WH, 2018, AM J GASTROENTEROL, V113, P987, DOI 10.1038/s41395-018-0122-8

Paik WH, 2017, ENDOSCOPY, V49, P544, DOI 10.1055/s-0043-102388

Paik WH, 2014, WORLD J GASTROENTERO, V20, P5051, DOI 10.3748/wjg.v20.i17.5051

Park DH, 2015, J GASTROEN HEPATOL, V30, P1461, DOI 10.1111/jgh.13027

Park DH, 2013, GASTROINTEST ENDOSC, V78, P91, DOI 10.1016/j.gie.2013.01.042

Park DH, 2011, GASTROINTEST ENDOSC, V74, P1276, DOI 10.1016/j.gie.2011.07.054

Poincloux L, 2015, ENDOSCOPY, V47, P794, DOI [10.1055/s-0035-1545399, 10.1055/s-0034-1391988]
Ryou M, 2020, GASTROINTEST ENDOSC, V91, P178, DOI 10.1016/j.gie.2019.07.035
Shimatani M, 2009, ENDOSCOPY, V41, P849, DOI 10.1055/s-0029-1215108
Shimatani M, 2017, J GASTROEN HEPATOL, V32, P1348, DOI 10.1111/jgh.13713
Sportes A, 2017, THER ADV GASTROENTER, V10, P483, DOI 10.1177/1756283X17702096
Tonozuka R, 2016, GASTROINTEST ENDOSC, V84, P206, DOI 10.1016/j.gie.2016.03.020
Umeda J, 2015, GASTROINTEST ENDOSC, V82, P390, DOI 10.1016/j.gie.2015.02.041
Vila JJ, 2012, GASTROINTEST ENDOSC, V76, P1133, DOI 10.1016/j.gie.2012.08.001
Williams EJ, 2007, GUT, V56, P821, DOI 10.1136/gut.2006.097543
Yamada A, 2019, DIGEST ENDOSC, V31, P86, DOI 10.1111/den.13261
Yang MJ, 2018, J GASTROEN HEPATOL, V33, P772, DOI 10.1111/jgh.14060
NR 50
TC 70
Z9 76
U1 1
U2 11
PU EDITORIAL OFFICE GUT & LIVER
PI SEOUL
PA 305 LOTTE GOLD ROSE II, 890-59, DAECHI 4-DONG, GANGNAM-GU, SEOUL,
135-839, SOUTH KOREA
SN 1976-2283
EI 2005-1212
J9 GUT LIVER
JI Gut Liver
PD MAR
PY 2021
VL 15
IS 2
BP 196
EP 205
DI 10.5009/gnl20096
PG 10
WC Gastroenterology & Hepatology
WE Science Citation Index Expanded (SCI-EXPANDED)
SC Gastroenterology & Hepatology
GA QX4QR
UT WOS:000629332600005
PM 32694240
OA Green Submitted, gold
DA 2026-05-09
ER

PT J
AU Kamata, K
Takenaka, M
Kitano, M
Omoto, S
Miyata, T
Minaga, K
Yamao, K
Imai, H
Sakurai, T
Watanabe, T
Nishida, N
Kudo, M
AF Kamata, Ken
Takenaka, Mamoru
Kitano, Masayuki
Omoto, Shunsuke
Miyata, Takeshi
Minaga, Kosuke
Yamao, Kentaro
Imai, Hajime
Sakurai, Toshiharu
Watanabe, Tomohiro
Nishida, Naoshi
Kudo, Masatoshi
TI Endoscopic ultrasound-guided gallbladder drainage for acute
cholecystitis: Long-term outcomes after removal of a self-expandable
metal stent
SO WORLD JOURNAL OF GASTROENTEROLOGY
LA English
DT Article
DE Endoscopic ultrasound-guided gallbladder drainage; Cholecystitis;
Endoscopic ultrasound-guided biliary drainage
ID PERCUTANEOUS CHOLECYSTOSTOMY; CHOLECYSTECTOMY; FEASIBILITY; ASPIRATION;
PLACEMENT; VIDEOS; RISK
AB AIM
To assess the long-term outcomes of this procedure after removal of self-expandable metal stent (SEMS). The efficacy and safety of endoscopic ultrasound-guided gallbladder drainage (EUS-GBD) with SEMS were also assessed.
METHODS
Between January 2010 and April 2015, 12 patients with acute calculous cholecystitis, who were deemed unsuitable for cholecystectomy, underwent EUS-GBD with a SEMS. EUS-GBD was performed under the guidance of EUS and fluoroscopy, by puncturing the gallbladder with a needle, inserting a guidewire, dilating the puncture hole, and placing a

SEMS. The SEMS was removed and/or replaced with a 7-Fr plastic pigtail stent after cholecystitis improved. The technical and clinical success rates, adverse event rate, and recurrence rate were all measured.

RESULTS

The rates of technical success, clinical success, and adverse events were 100%, 100%, and 0%, respectively. After cholecystitis improved, the SEMS was removed without replacement in eight patients, whereas it was replaced with a 7-Fr pigtail stent in four patients. Recurrence was seen in one patient (8.3%) who did not receive a replacement pigtail stent. The median follow-up period after EUS-GBD was 304 d (78-1492).

CONCLUSION

EUS-GBD with a SEMS is a possible alternative treatment for acute cholecystitis. Long-term outcomes after removal of the SEMS were excellent. Removal of the SEMS at 4-wk after SEMS placement and improvement of symptoms might avoid migration of the stent and recurrence of cholecystitis due to food impaction.
C1 [Kamata, Ken; Takenaka, Mamoru; Kitano, Masayuki; Omoto, Shunsuke; Miyata, Takeshi; Minaga, Kosuke; Yamao, Kentaro; Imai, Hajime; Sakurai, Toshiharu; Watanabe, Tomohiro; Nishida, Naoshi; Kudo, Masatoshi] Kindai Univ, Fac Med, Dept Gastroenterol & Hepatol, 377-2 Ohno Higashi, Osaka 5898511, Japan.

C3 Kindai University (Kinki University)

RP Takenaka, M (corresponding author), Kindai Univ, Fac Med, Dept Gastroenterol & Hepatol, 377-2 Ohno Higashi, Osaka 5898511, Japan.

EM mamoxyo45@gmail.com

RI Watanabe, Tomohiro/ABA-4712-2021; Minaga, Kosuke/AAD-2107-2020; Nishida, Naoshi/AFV-2598-2022; Kudo, Masatoshi/AAA-9744-2019

OI Watanabe, Tomohiro/0000-0002-6175-8064;
FU Japan Society for the Promotion of Science; Japanese Foundation for the Research and Promotion of Endoscopy [22590764, 25461035]; Grants-in-Aid for Scientific Research [25461035, 22590764] Funding Source: KAKEN

FX Supported by the Japan Society for the Promotion of Science and the Japanese Foundation for the Research and Promotion of Endoscopy No. 22590764 and No. 25461035.
CR Anderloni A, 2016, SURG ENDOSC, V30, P5200, DOI 10.1007/s00464-016-4894-x

Baron TH, 2007, GASTROINTEST ENDOSC, V65, P735, DOI 10.1016/j.gie.2006.07.041

Súbtil JC, 2010, WORLD J GASTRO ENDOS, V2, P203, DOI 10.4253/wjge.v2.i6.203

Choi JH, 2014, ENDOSCOPY, V46, P656, DOI 10.1055/s-0034-1365720

Chopra S, 2001, AM J ROENTGENOL, V176, P1025, DOI 10.2214/ajr.176.4.1761025

de la Serna-Higuera C, 2013, GASTROINTEST ENDOSC, V77, P303, DOI 10.1016/j.gie.2012.09.021

DUBOIS F, 1990, ANN SURG, V211, P60, DOI 10.1097/00000658-199001000-00010

Ito K, 2004, AM J ROENTGENOL, V183, P193, DOI 10.2214/ajr.183.1.1830193

Itoi T, 2012, GASTROINTEST ENDOSC, V75, P870, DOI 10.1016/j.gie.2011.10.020

Itoi T, 2011, J HEPATO-BIL-PAN SCI, V18, P282, DOI 10.1007/s00534-010-0310-4

Jang JW, 2012, GASTROENTEROLOGY, V142, P805, DOI 10.1053/j.gastro.2011.12.051

Jang JW, 2011, GASTROINTEST ENDOSC, V74, P176, DOI 10.1016/j.gie.2011.03.1120

Kamata K, 2009, ENDOSCOPY, V41, pE315, DOI 10.1055/s-0029-1215258

Lee SS, 2007, GASTROINTEST ENDOSC, V66, P1008, DOI 10.1016/j.gie.2007.03.1080

Minaga K, 2016, DIG ENDOSC

Moon JH, 2014, GASTROINTEST ENDOSC, V79, P990, DOI 10.1016/j.gie.2014.02.015
Ogura T, 2014, ENDOSCOPY, V46, pE75, DOI 10.1055/s-0033-1359135
REDDICK E J, 1989, Surgical Endoscopy, V3, P131, DOI 10.1007/BF00591357
Song TJ, 2010, GASTROINTEST ENDOSC, V71, P634, DOI 10.1016/j.gie.2009.11.024
Takasawa O, 2009, DIGEST ENDOSC, V21, P43, DOI 10.1111/j.1443-1661.2008.00822.x
Teoh AYB, 2014, GASTROINTEST ENDOSC, V80, P1171, DOI 10.1016/j.gie.2014.03.038
Walter D, 2016, GUT, V65, P6, DOI 10.1136/gutjnl-2015-309925
Wang KX, 2016, GASTROINTEST ENDOSC, V83, P1218, DOI 10.1016/j.gie.2015.10.033
NR 23
TC 50
Z9 56
U1 0
U2 2
PU BAISHIDENG PUBLISHING GROUP INC
PI PLEASANTON
PA 8226 REGENCY DR, PLEASANTON, CA 94588 USA
SN 1007-9327
EI 2219-2840
J9 WORLD J GASTROENTERO
JI World J. Gastroenterol.
PD JAN 28
PY 2017
VL 23
IS 4
BP 661
EP 667
DI 10.3748/wjg.v23.i4.661
PG 7
WC Gastroenterology & Hepatology
WE Science Citation Index Expanded (SCI-EXPANDED)
SC Gastroenterology & Hepatology
GA EI1JH
UT WOS:000392232700011
PM 28216973
OA Green Submitted, hybrid
DA 2026-05-09
ER

PT J
AU Kahaleh, M
Artifon, ELA
Perez-Miranda, M

Gaidhane, M
Rondon, C
Itoi, T
Giovannini, M
AF Kahaleh, Michel
Artifon, Everson L. A.
Perez-Miranda, Manuel
Gaidhane, Monica
Rondon, Carlos
Itoi, Takao
Giovannini, Marc
TI Endoscopic ultrasonography guided drainage: Summary of consortium
meeting, May 21, 2012, San Diego, California
SO WORLD JOURNAL OF GASTROENTEROLOGY
LA English
DT Review
DE Endoscopic ultrasound; Endoscopic ultrasound-guided biliary drainage;
Consortium; Biliary drainage; Pancreatic drainage; Endoscopic
ultrasound-guided
ID Y GASTRIC BYPASS; MALIGNANT BILIARY OBSTRUCTION; GASTROSTOMY TUBE
PLACEMENT; EXPANDABLE METAL STENT; GI TRACT SURGERY; BILE-DUCT ACCESS;
GALLBLADDER DRAINAGE; ACUTE CHOLECYSTITIS; INTERVENTIONAL EUS;
PANCREATIC-CANCER
AB Endoscopic retrograde cholangiopancreatography (ERCP) is the preferred procedure for biliary and pancreatic drainage. While ERCP is successful in about 95% of cases, a small subset of cases are unsuccessful due to altered anatomy, peri-ampullary pathology, or malignant obstruction. Endoscopic ultrasound-guided drainage is a promising technique for biliary, pancreatic and recently gallbladder decompression, which provides multiple advantages over percutaneous or surgical biliary drainage. Multiple retrospective and some prospective studies have shown endoscopic ultrasound-guided drainage to be safe and effective. Based on the currently reported literature, regardless of the approach, the cumulative success rate is 84%-93% with an overall complication rate of 16%-35%. endoscopic ultrasound-guided drainage seems a viable therapeutic modality for failed conventional drainage when performed by highly skilled advanced endoscopists at tertiary centers with expertise in both echo-endoscopy and therapeutic endoscopy
C1 [Kahaleh, Michel; Gaidhane, Monica; Rondon, Carlos] Weill Cornell Med Ctr, Dept Med, Div Gastroenterol & Hepatol, New York, NY 10021 USA.
[Artifon, Everson L. A.] Univ Sao Paulo, Sch Med, Dept Surg, BR-05403000 Sao Paulo, Brazil.
[Perez-Miranda, Manuel] Hosp Univ Rio Hortega, Endoscopy Unit, Valladolid 47012, Spain.
[Itoi, Takao] Tokyo Med Univ, Dept Gastroenterol & Hematol, Tokyo 1600023, Japan.
[Giovannini, Marc] Paoli Calmettes Inst, Dept Gastroenterol, F-13273 Marseille, France.
C3 Cornell University; Weill Cornell Medical Center; Weill Cornell
Medicine; Universidade de Sao Paulo; Hospital del Rio Hortega; Tokyo
Medical University; UNICANCER; Institut Paoli-Calmette (IPC)
RP Kahaleh, M (corresponding author), Weill Cornell Med Coll, Dept Med, Div Gastroenterol & Hepatol, 1305 York

Ave,4th Floor, New York, NY 10021 USA.
EM mkahaleh@gmail.com
RI M, Gaidhane/B-9414-2009; Kahaleh, Michel/A-9408-2011; Gaidhane, Monica/B-9414-2009
OI Kahaleh, Michel/0000-0003-0836-6114; Gaidhane, Monica/0000-0001-6773-7080
CR Ang TL, 2007, J PANCREAS, V8, P438
Artifon EL, 2012, GASTROINTEST ENDOSC, V75, P162
Artifon ELA, 2012, J CLIN GASTROENTEROL, V46, P768, DOI 10.1097/MCG.0b013e31825f264c
Artifon ELA, 2011, J PANCREAS, V12, P610
Artifon ELA, 2010, J PANCREAS, V11, P597
Ashida R, 2009, J HEPATO-BILIARY-PAN, V16, P592, DOI 10.1007/s00534-009-0129-z
Attam R, 2011, GASTROINTEST ENDOSC, V74, P677, DOI 10.1016/j.gie.2011.05.018
Attasaranya S, 2012, GASTROENT RES PRACT, V2012, DOI 10.1155/2012/680753
Bapaye A, 2012, Endoscopy, V44 Suppl 2 UCTN, pE435, DOI 10.1055/s-0032-1325894
Barkay O, 2010, GASTROINTEST ENDOSC, V71, P1166, DOI 10.1016/j.gie.2009.10.048
Baron TH, 1998, GASTROINTEST ENDOSC, V48, P640, DOI 10.1016/S0016-5107(98)70052-5
Baron TH, 2008, GASTROINTEST ENDOSC, V68, P189, DOI 10.1016/j.gie.2007.11.016
Baron TH, 2007, GASTROINTEST ENDOSC, V65, P735, DOI 10.1016/j.gie.2006.07.041
Baron TH, 2012, GASTROINTEST ENDOSC, V76, P641, DOI 10.1016/j.gie.2012.05.027
Baron TH, 2012, GASTROINTEST ENDOSC, V75, P1258, DOI 10.1016/j.gie.2012.02.026
Belletrutti PJ, 2010, J PANCREAS, V11, P234
Bertin PM, 2011, SURG ENDOSC, V25, P2592, DOI 10.1007/s00464-011-1593-5
Bories E, 2007, ENDOSCOPY, V39, P287, DOI 10.1055/s-2007-966212
Brauer BC, 2009, GASTROINTEST ENDOSC, V70, P471, DOI 10.1016/j.gie.2008.12.233
Burmester E, 2003, GASTROINTEST ENDOSC, V57, P246, DOI 10.1067/mge.2003.85
Súbtil JC, 2010, WORLD J GASTRO ENDOS, V2, P203, DOI 10.4253/wjge.v2.i6.203
Choi EK, 2013, SURG ENDOSC, V27, P2894, DOI 10.1007/s00464-013-2850-6
Chopin-Laly X, 2009, SURGERY, V145, P123, DOI 10.1016/j.surg.2008.07.019
Costamagna G, 2010, GASTROINTEST ENDOSC, V72, P551, DOI 10.1016/j.gie.2010.04.052
de la Serna-Higuera C, 2013, GASTROINTEST ENDOSC, V77, P303, DOI 10.1016/j.gie.2012.09.021
Dhir V, 2012, GASTROINTEST ENDOSC, V75, P354, DOI 10.1016/j.gie.2011.07.075
Ergun M, 2011, ENDOSCOPY, V43, P518, DOI 10.1055/s-0030-1256333
Fabbri C, 2011, ENDOSCOPY, V43, P438, DOI 10.1055/s-0030-1256097
Feitoza AB, 2001, GASTROINTEST ENDOSC, V54, P743, DOI 10.1067/mge.2001.120169
Feitoza AB, 2002, GASTROINTEST ENDOSC, V55, P75, DOI 10.1067/mge.2002.120385
FRAZEE RC, 1989, MAYO CLIN PROC, V64, P163, DOI 10.1016/S0025-6196(12)65670-5
Fujita N, 2007, WORLD J GASTROENTERO, V13, P5512, DOI 10.3748/wjg.v13.i41.5512
Fujita N, 2009, DIGEST ENDOSC, V21, P48, DOI 10.1111/j.1443-1661.2008.00821.x
Giovannini M, 2006, ENDOSCOPY, V38, pS93, DOI 10.1055/s-2006-946665
Giovannini M, 2003, ENDOSCOPY, V35, P1076, DOI 10.1055/s-2003-44596
Giovannini M, 2001, ENDOSCOPY, V33, P898, DOI 10.1055/s-2001-17324

Gupta K, 2014, J CLIN GASTROENTEROL, V48, P80, DOI 10.1097/MCG.0b013e31828c6822
Hanada K, 2009, DIGEST ENDOSC, V21, pS75, DOI 10.1111/j.1443-1661.2009.00855.x
Hara K, 2013, ENDOSCOPY, V45, P392, DOI 10.1055/s-0032-1326076
Hara K, 2011, AM J GASTROENTEROL, V106, P1239, DOI 10.1038/ajg.2011.84
Henry WA, 2012, GASTROINTEST ENDOSC, V76, P1065, DOI 10.1016/j.gie.2011.09.046
Hirota M, 2007, J HEPATO-BILIARY-PAN, V14, P78, DOI 10.1007/s00534-006-1159-4
Horaguchi J, 2009, DIGEST ENDOSC, V21, P239, DOI 10.1111/j.1443-1661.2009.00899.x
HOUGHTON PWJ, 1985, BRIT J SURG, V72, P220, DOI 10.1002/bjs.1800720327
Irisawa A, 2009, J HEPATO-BILIARY-PAN, V16, P598, DOI 10.1007/s00534-009-0131-5
Ito K, 2010, DIGEST ENDOSC, V22, pS132, DOI 10.1111/j.1443-1661.2010.00971.x
Itoi T, 2008, WORLD J GASTROENTERO, V14, P6078, DOI 10.3748/wjg.14.6078
Itoi T, 2008, GASTROINTEST ENDOSC, V68, P455, DOI 10.1016/j.gie.2008.02.052
Itoi T, 2013, DIGEST ENDOSC, V25, P142, DOI 10.1111/den.12065
Itoi T, 2012, GASTROINTEST ENDOSC, V75, P870, DOI 10.1016/j.gie.2011.10.020
Itoi T, 2011, J HEPATO-BIL-PAN SCI, V18, P282, DOI 10.1007/s00534-010-0310-4
Itoi T, 2010, GASTROINTEST ENDOSC, V71, P1038, DOI 10.1016/j.gie.2010.01.026
Itoi T, 2009, GASTROINTEST ENDOSC, V69, pS8, DOI 10.1016/j.gie.2008.11.003
Iwamuro M, 2010, DIGEST ENDOSC, V22, P236, DOI 10.1111/j.1443-1661.2010.00997.x
Iwashita T, 2012, ENDOSCOPY, V44, P60, DOI 10.1055/s-0030-1256871
Iwashita T, 2013, DIGEST DIS SCI, V58, P2417, DOI 10.1007/s10620-013-2645-6
Jang JW, 2012, GASTROENTEROLOGY, V142, P805, DOI 10.1053/j.gastro.2011.12.051
Jang JW, 2011, GASTROINTEST ENDOSC, V74, P176, DOI 10.1016/j.gie.2011.03.1120
Jürgensen C, 2013, ENDOSCOPY, V45, pE40, DOI [10.1055/s0032-1325891, 10.1055/s-0032-1325891]
Kahaleh M, 2006, GASTROINTEST ENDOSC, V64, P52, DOI 10.1016/j.gie.2006.01.063
Kahaleh M, 2004, GASTROINTEST ENDOSC, V60, P138, DOI 10.1016/S0016-5107(04)01528-7
Kahaleh M, 2005, GASTROINTEST ENDOSC, V61, P307, DOI 10.1016/S0016-5107(04)02585-4
Kahaleh M, 2013, GASTROINTEST ENDOS S, V77, pAB127, DOI DOI 10.1016/J.GIE.2013.04.021
Kahaleh M, 2013, WORLD J GASTROENTERO, V19, P1372, DOI 10.3748/wjg.v19.i9.1372
Kamata K, 2009, ENDOSCOPY, V41, pE315, DOI 10.1055/s-0029-1215258
Katanuma A, 2012, DIGEST ENDOSC, V24, P34, DOI 10.1111/j.1443-1661.2012.01256.x
Kawakubo K, 2012, GUT LIVER, V6, P399, DOI 10.5009/gnl.2012.6.3.399
Kedia P, 2013, CLIN ENDOSC, V46, P543, DOI 10.5946/ce.2013.46.5.543
Khashab MA, 2012, GASTROINTEST ENDOSC, V76, P209, DOI 10.1016/j.gie.2012.03.170
Kim TH, 2012, WORLD J GASTROENTERO, V18, P2526, DOI 10.3748/wjg.v18.i20.2526
Kim YS, 2010, ENDOSCOPY, V42, P496, DOI 10.1055/s-0029-1244082
Komaki T, 2011, PANCREATOLOGY, V11, P47, DOI 10.1159/000323508
Kwan V, 2007, GASTROINTEST ENDOSC, V66, P582, DOI 10.1016/j.gie.2007.02.065
Lai R, 2005, ENDOSCOPY, V37, P487, DOI 10.1055/s-2005-861250
Lee SS, 2007, GASTROINTEST ENDOSC, V66, P1008, DOI 10.1016/j.gie.2007.03.1080
Ma J, 2011, ENDOSCOPY, V43, pE102, DOI 10.1055/s-0030-1256104
Madan A, 2011, ENDOSCOPY, V43, pE73, DOI 10.1055/s-0030-1256038
Mallery S, 2004, GASTROINTEST ENDOSC, V59, P100, DOI 10.1016/S0016-5107(03)02300-9

Maranki J, 2009, ENDOSCOPY, V41, P532, DOI 10.1055/s-0029-1214712
MARINONE MG, 1991, HAEMATOLOGICA, V76, P519
Martinez J, 2006, SURG ENDOSC, V20, P1548, DOI 10.1007/s00464-005-0267-6
Matsumoto K, 2013, GASTROINTEST ENDOSC, V77, P132, DOI 10.1016/j.gie.2012.09.016
Mergener K, 2003, GASTROINTEST ENDOSC, V58, P461, DOI 10.1067/S0016-5107(03)00032-4
Miura F, 2013, J HEPATO-BIL-PAN SCI, V20, P47, DOI 10.1007/s00534-012-0563-1
Nahrwold DL., 1997, Textbook of Surgery, P1126
Nguyen-Tang T, 2010, ENDOSCOPY, V42, P232, DOI 10.1055/s-0029-1243858
Pannala R, 2011, GASTROINTEST ENDOSC, V74, P295, DOI 10.1016/j.gie.2011.04.029
Park DH, 2013, GASTROINTEST ENDOSC, V78, P91, DOI 10.1016/j.gie.2013.01.042
Park DH, 2012, GASTROINTEST ENDOSC, V75, P692, DOI 10.1016/j.gie.2011.04.013
Park DH, 2011, GASTROINTEST ENDOSC, V74, P1276, DOI 10.1016/j.gie.2011.07.054
Park SJ, 2013, GASTROINTEST ENDOSC, V78, P374, DOI 10.1016/j.gie.2013.04.183
Perez-Miranda M, 2011, GASTROINTEST ENDOSC, V73, pAB126, DOI [10.1016/j.gie.2011.03.050, DOI 10.1016/J.GIE.2011.03.050]
Perez-Miranda M, 2007, GASTROINTEST ENDOSC, V65, pAB123, DOI 10.1016/j.gie.2007.03.122
Perez-Miranda Manuel, 2012, Gastrointest Endosc Clin N Am, V22, P491, DOI 10.1016/j.giec.2012.05.004
Pimentel RR, 2004, GASTROINTEST ENDOSC, V59, P325, DOI 10.1016/S0016-5107(03)02549-5
Prachayakul V, 2011, ENDOSCOPY, V43, pE109, DOI 10.1055/s-0030-1256140
Püspök A, 2005, AM J GASTROENTEROL, V100, P1743, DOI 10.1111/j.1572-0241.2005.41806.x
Ramírez-Luna MA, 2011, ENDOSCOPY, V43, P826, DOI 10.1055/s-0030-1256406
Sarkaria S, 2013, GUT LIVER, V7, P129, DOI 10.5009/gnl.2013.7.2.129
Schreiner MA, 2012, GASTROINTEST ENDOSC, V75, P748, DOI 10.1016/j.gie.2011.11.019
Shah JN, 2012, GASTROINTEST ENDOSC, V75, P56, DOI 10.1016/j.gie.2011.08.032
Shah RJ, 2013, GASTROINTEST ENDOSC, V77, P593, DOI 10.1016/j.gie.2012.10.015
Shear W, 2012, ENDOSCOPY, V44, pE58, DOI 10.1055/s-0031-1291562
Siddiqui AA, 2011, SURG ENDOSC, V25, P549, DOI 10.1007/s00464-010-1216-6
Song TJ, 2012, WORLD J GASTROENTERO, V18, P4435, DOI 10.3748/wjg.v18.i32.4435
Song TJ, 2010, GASTROINTEST ENDOSC, V71, P634, DOI 10.1016/j.gie.2009.11.024
Takada T, 2013, J HEPATO-BIL-PAN SCI, V20, P1, DOI 10.1007/s00534-012-0566-y
Takasawa O, 2009, DIGEST ENDOSC, V21, P43, DOI 10.1111/j.1443-1661.2008.00822.x
Tarantino I, 2008, ENDOSCOPY, V40, P336, DOI 10.1055/s-2007-995455
Tekola B, 2011, DIGEST DIS SCI, V56, P3364, DOI 10.1007/s10620-011-1743-6
Tessier G, 2007, GASTROINTEST ENDOSC, V65, P233, DOI 10.1016/j.gie.2006.06.029
Tsuyuguchi T, 2013, J HEPATO-BIL-PAN SCI, V20, P81, DOI 10.1007/s00534-012-0570-2
Weilert F, 2011, ENDOSCOPY, V43, P1105, DOI 10.1055/s-0030-1256961
Wiersema MJ, 1996, GASTROINTEST ENDOSC, V43, P102, DOI 10.1016/S0016-5107(06)80108-2
Will U, 2007, ENDOSCOPY, V39, P292, DOI 10.1055/s-2007-966215
Yamao K, 2008, ENDOSCOPY, V40, P340, DOI 10.1055/s-2007-995485
Yamao K, 2006, GASTROINTEST ENDOSC, V64, P663, DOI 10.1016/j.gie.2006.07.003
Yokoe M, 2012, J HEPATO-BIL-PAN SCI, V19, P578, DOI 10.1007/s00534-012-0548-0
NR 118

TC 14
Z9 19
U1 1
U2 8
PU BAISHIDENG PUBLISHING GROUP INC
PI PLEASANTON
PA 8226 REGENCY DR, PLEASANTON, CA 94588 USA
SN 1007-9327
EI 2219-2840
J9 WORLD J GASTROENTERO
JI World J. Gastroenterol.
PD JAN 21
PY 2015
VL 21
IS 3
BP 726
EP 741
DI 10.3748/wjg.v21.i3.726
PG 16
WC Gastroenterology & Hepatology
WE Science Citation Index Expanded (SCI-EXPANDED)
SC Gastroenterology & Hepatology
GA AZ7TA
UT WOS:000348419400002
PM 25624708
OA Green Submitted, Bronze
DA 2026-05-09
ER

PT J
AU Staub, J
Adler, DG
AF Staub, Judith
Adler, Douglas G.
TI Endoscopic Ultrasound-Guided Gallbladder Drainage
SO PRACTICAL GASTROENTEROLOGY
LA English
DT Article
ID PERCUTANEOUS CHOLECYSTOSTOMY; ACUTE CHOLECYSTITIS; METAL STENT;
MANAGEMENT; OUTCOMES
C1 [Staub, Judith; Adler, Douglas G.] Utah Sch Med, Div Gastroenterol & Hepatol, Salt Lake City, UT 84132 USA.
RP Staub, J (corresponding author), Utah Sch Med, Div Gastroenterol & Hepatol, Salt Lake City, UT 84132 USA.

CR Anderloni A, 2017, ENDOSC INT OPEN, V5, pE1100, DOI 10.1055/s-0043-118828
Borzellino G, 2008, SURG ENDOSC, V22, P8, DOI 10.1007/s00464-007-9511-6
Chan JHY, 2018, CLIN ENDOSC, V51, P150, DOI 10.5946/ce.2017.125
Chan SM, 2017, GASTROINTEST ENDOSC, V85, P1225, DOI 10.1016/j.gie.2016.10.014
Choi JH, 2014, ENDOSCOPY, V46, P656, DOI 10.1055/s-0034-1365720
Dollhopf M, 2017, GASTROINTEST ENDOSC, V86, P636, DOI 10.1016/j.gie.2017.02.027
Gurusamy KS, 2013, COCHRANE DB SYST REV, DOI 10.1002/14651858.CD007088.pub2
Itoi T, 2010, GASTROINTEST ENDOSC, V71, P1038, DOI 10.1016/j.gie.2010.01.026
Leveau P, 2008, SCAND J GASTROENTERO, V43, P593, DOI 10.1080/00365520701851673
MCGAHAN JP, 1989, RADIOLOGY, V173, P481, DOI 10.1148/radiology.173.2.2678261
Sanjay P, 2013, HPB, V15, P511, DOI 10.1111/j.1477-2574.2012.00610.x
Teoh AYB, 2017, ENDOSCOPY, V49, P130, DOI 10.1055/s-0042-119036
Tyberg A, 2018, J CLIN GASTROENTEROL, V52, P79, DOI 10.1097/MCG.0000000000000786
Walter D, 2016, GUT, V65, P6, DOI 10.1136/gutjnl-2015-309925
NR 14
TC 0
Z9 0
U1 0
U2 0
PU SHUGAR PUBLISHING INC
PI WESTHAMPTON BEACH
PA 32 MILL ROAD, WESTHAMPTON BEACH, NY 11978 USA
SN 0277-4208
J9 PRACT GASTROENTEROL
JI Pract. Gasterontol.
PD APR
PY 2018
VL 42
IS 4
BP 32
EP 39
PG 5
WC Gastroenterology & Hepatology
WE Emerging Sources Citation Index (ESCI)
SC Gastroenterology & Hepatology
GA JB1AL
UT WOS:000488291500002
DA 2026-05-09
ER

PT J
AU Teh, JL

Rimbas, M
Larghi, A
Teoh, AYB
AF Teh, J. L.
Rimbas, Mihai
Larghi, Alberto
Teoh, Anthony Yuen Bun
TI Endoscopic ultrasound in the management of acute cholecystitis
SO BEST PRACTICE & RESEARCH CLINICAL GASTROENTEROLOGY
LA English
DT Article
DE Acute cholecystitis; Lumen apposing metal stents; Endoscopic gallbladder drainage
ID GUIDED GALLBLADDER DRAINAGE; APPOSING METAL STENT; PERCUTANEOUS CHOLECYSTOSTOMY; EFFICACY; FEASIBILITY; OUTCOMES; SAFETY
AB Endoscopic ultrasound-guided gallbladder drainage (EUS-GBD) is increasingly utilised for patients with acute cholecystitis who are high-risk candidates for surgery. The technique to perform EUS-GBD has evolved and matured over the last two decades since EUS-GBD was first described in 2007 using a nasobiliary catheter. Compared to percutaneous gallbladder drainage (PT-GBD), EUS-GBD offers benefits of shorter hospitalisation stay and lesser procedural pain and need for reintervention. The purpose of this review is to provide an updated review of the equipment and techniques available for EUS-GBD, outcomes of the procedure and how it compares against endoscopic transpapillary drainage (ET-GBD), PT-GBD and laparoscopic cholecystectomy. Controversies surrounding the procedure will also be discussed.
C1 [Teh, J. L.] Natl Univ Hlth Syst, Dept Surg, Juronghealth Campus, Singapore, Singapore.
[Rimbas, Mihai; Larghi, Alberto] Fdn Policlin Univ A Gemelli IRCCS, Digest Endoscopy Unit, Rome, Italy.
[Teoh, Anthony Yuen Bun] Chinese Univ Hong Kong, Prince Wales Hosp, Dept Surg, Hong Kong, Peoples R China.
C3 National University of Singapore; Catholic University of the Sacred Heart; IRCCS Policlinico Gemelli; Prince of Wales Hospital Hong Kong; Chinese University of Hong Kong
RP Teoh, AYB (corresponding author), Chinese Univ Hong Kong, Prince Wales Hosp, Dept Surg, Hong Kong, Peoples R China.
EM anthonyteoh@surgery.cuhk.edu.hk
RI Teoh, Anthony/L-4796-2016; Teh, Jun liang/MCK-1569-2025; Larghi, Alberto/AAC-7448-2022
OI Teoh, Anthony/0000-0002-1885-8035;
CR Ahmed O, 2018, SURG ENDOSC, V32, P1627, DOI 10.1007/s00464-018-6041-3
Anderloni A, 2019, ANN GASTROENTEROL, V32, P522, DOI 10.20524/aog.2019.0384
Anderloni A, 2016, SURG ENDOSC, V30, P5200, DOI 10.1007/s00464-016-4894-x
Chan SM, 2022, ENDOSC INT OPEN, V10, pE1073, DOI 10.1055/a-1819-8124
Chan SM, 2017, GASTROINTEST ENDOSC, V85, P1225, DOI 10.1016/j.gie.2016.10.014
Chantarojanasiri Tanyaporn, 2017, Saudi J Gastroenterol, V23, P318, DOI 10.4103/sjg.SJG_115_17
Cho DH, 2019, SURG ENDOSC, V33, P2135, DOI 10.1007/s00464-018-6485-5
Cho SH, 2020, GASTROINTEST ENDOSC, V91, P543, DOI 10.1016/j.gie.2019.09.042

Corral JE, 2019, SURG ENDOSC, V33, P3567, DOI 10.1007/s00464-019-07026-z
Cucchetti A, 2022, GASTROINTEST ENDOSC, V95, P399, DOI 10.1016/j.gie.2021.09.028
Cull JD, 2014, J GASTROINTEST SURG, V18, P328, DOI 10.1007/s11605-013-2341-z
de Mestral C, 2013, J TRAUMA ACUTE CARE, V74, P175, DOI 10.1097/TA.0b013e31827890e1
Di Mitri R, 2021, GASTROINTEST ENDOSC, V94, P1014, DOI 10.1016/j.gie.2021.05.025
Dollhopf M, 2017, GASTROINTEST ENDOSC, V86, P636, DOI 10.1016/j.gie.2017.02.027
Fabbri C, 2022, SURG ENDOSC, V36, P7974, DOI 10.1007/s00464-022-09339-y
Higa Jennifer T, 2019, Curr Treat Options Gastroenterol, V17, P357, DOI [10.1007/s11938-019-00243-4, 10.1007/s11938-019-00243-4]
Higa JT, 2019, GASTROINTEST ENDOSC, V90, P483, DOI 10.1016/j.gie.2019.04.238
Hultman CS, 1996, AM SURGEON, V62, P263
Imai H, 2016, GASTROINTEST ENDOSC, V84, P147, DOI 10.1016/j.gie.2015.12.024
Irani S, 2017, CLIN GASTROENTEROL H, V15, P738, DOI 10.1016/j.cgh.2016.12.021
Issa D, 2021, ENDOSCOPY, V53, P827, DOI 10.1055/a-1259-0349
Itoi T, 2012, GASTROINTEST ENDOSC, V75, P870, DOI 10.1016/j.gie.2011.10.020
Jandura DM, 2021, WORLD J GASTRO ENDOS, V13, P345, DOI 10.4253/wjge.v13.i8.345
Jang JW, 2012, GASTROENTEROLOGY, V142, P805, DOI 10.1053/j.gastro.2011.12.051
Kamata K, 2017, WORLD J GASTROENTERO, V23, P661, DOI 10.3748/wjg.v23.i4.661
Kanno Y, 2019, TURK J GASTROENTEROL, V30, P1055, DOI 10.5152/tjg.2019.181032
Krishnamoorthi R, 2020, SURG ENDOSC, V34, P1904, DOI 10.1007/s00464-020-07409-7
Law R, 2016, CLIN GASTROENTEROL H, V14, P476, DOI 10.1016/j.cgh.2015.10.026
Lee SS, 2007, GASTROINTEST ENDOSC, V66, P1008, DOI 10.1016/j.gie.2007.03.1080
Lisotti A, 2022, SURG ENDOSC, V36, P569, DOI 10.1007/s00464-021-08318-z
Luk SWY, 2019, ENDOSCOPY, V51, P722, DOI 10.1055/a-0929-6603
Lyu Y, 2021, J LAPAROENDOSC ADV S, V31, P1295
Lyu YX, 2021, J LAPAROENDOSC ADV S, V31, P1232, DOI 10.1089/lap.2020.0786
Mangiavillano B, 2022, GASTROINTEST ENDOSC, V95, P115, DOI 10.1016/j.gie.2021.07.021
Maruta A, 2022, INTERNAL MED
Minaga K, 2019, DIGEST ENDOSC, V31, P180, DOI 10.1111/den.13242
Mohan BP, 2020, ENDOSCOPY, V52, P96, DOI 10.1055/a-1020-3932
Moon JH, 2014, GASTROINTEST ENDOSC, V79, P990, DOI 10.1016/j.gie.2014.02.015
Mori Y, 2018, J HEPATO-BIL-PAN SCI, V25, P87, DOI 10.1002/jhbp.504
Nishiguchi K, 2021, ENDOSC ULTRASOUND, V10, P448, DOI 10.4103/EUS-D-20-00130
Okamoto K, 2018, J HEPATO-BIL-PAN SCI, V25, P55, DOI 10.1002/jhbp.516
Ong LWL, 2022, ENDOSCOPY, V54, pE800, DOI 10.1055/a-1806-1646
Pisano M, 2020, WORLD J EMERG SURG, V15, DOI 10.1186/s13017-020-00336-x
Podboy A, 2021, GASTROINTEST ENDOSC, V93, P797, DOI 10.1016/j.gie.2020.09.040
Rajadurai A, 2022, J GASTROEN HEPATOL, V37, P179, DOI 10.1111/jgh.15688
Sagami R, 2020, CLIN ENDOSC, V53, P176, DOI 10.5946/ce.2019.177
Saumoy M, 2019, J CLIN GASTROENTEROL, V53, P231, DOI 10.1097/MCG.0000000000001036
Siddiqui A, 2019, SURG ENDOSC, V33, P1260, DOI 10.1007/s00464-018-6406-7
Sugiyama M, 1998, WORLD J SURG, V22, P459, DOI 10.1007/s002689900416

Teoh AY, 2019, ENDOSC INT OPEN, V7, pE964, DOI 10.1055/a-0915-2098
Teoh AYB, 2020, GUT, V69, P1085, DOI 10.1136/gutjnl-2019-319996
Teoh AYB, 2021, DIGEST ENDOSC, V33, P1139, DOI 10.1111/den.13911
Teoh AYB, 2021, GASTROINTEST ENDOSC, V93, P577, DOI 10.1016/j.gie.2020.06.066
Teoh AYB, 2019, ENDOSC ULTRASOUND, V8, pS40, DOI 10.4103/eus.eus_49_19
Yuste RT, 2020, DIGEST ENDOSC, V32, P608, DOI 10.1111/den.13562
Yuste RT, 2019, ANN GASTROENTEROL, V32, P514, DOI 10.20524/aog.2019.0395
Tsuyuguchi T, 2013, J HEPATO-BIL-PAN SCI, V20, P81, DOI 10.1007/s00534-012-0570-2
van der Merwe SW, 2022, ENDOSCOPY, V54, P185, DOI 10.1055/a-1717-1391
van Wanrooij RLJ, 2022, ENDOSCOPY, V54, P310, DOI 10.1055/a-1738-6780
Vanella Giuseppe, 2022, VideoGIE, V7, P120, DOI [10.1016/j.vgie.2021.10.002, 10.1016/j.vgie.2021.10.002]
Villarroel M, 2021, ENDOSCOPY, V53, pE275, DOI 10.1055/a-1260-2830
Yang MJ, 2020, DIGEST DIS, V38, P542, DOI 10.1159/000505644
Yokoe M, 2018, J HEPATO-BIL-PAN SCI, V25, P41, DOI 10.1002/jhbp.515
Yoo HW, 2022, ENDOSCOPY, V54, P384, DOI 10.1055/a-1518-7039
NR 64
TC 5
Z9 6
U1 0
U2 3
PU ELSEVIER SCI LTD
PI London
PA 125 London Wall, London, ENGLAND
SN 1521-6918
EI 1532-1916
J9 BEST PRACT RES CL GA
JI Best Pract. Res. Clin. Gastroenterol.
PD SEP-DEC
PY 2022
VL 60
AR 101806
DI 10.1016/j.bpg.2022.101806
EA DEC 2022
PG 9
WC Gastroenterology & Hepatology
WE Science Citation Index Expanded (SCI-EXPANDED)
SC Gastroenterology & Hepatology
GA 7U9XF
UT WOS:000912476600001
PM 36577527
DA 2026-05-09
ER

PT J
AU Bang, JY
Varadarajulu, S
AF Bang, Ji Young
Varadarajulu, Shyam
TI Lumen-apposing metal stents for endoscopic ultrasonography-guided
interventions
SO DIGESTIVE ENDOSCOPY
LA English
DT Review
DE drainage; endoscopic ultrasound; interventional EUS; lumen-apposing
metal stent; pancreatic fluid collections
ID TRANSHEPATIC GALLBLADDER DRAINAGE; GASTROENTEROSTOMY
AB Lumen-apposing metal stents (LAMS) are integrated in a single-step delivery platform and can be deployed under endoscopic ultrasound-guidance for the drainage of intra-abdominal fluid collections, decompression of obstructed ductal systems, establishment of anastomosis or creation of fistulous tracts between organs that can facilitate further endoscopic interventions if required. A unique feature of LAMS is their ability to approximate the adjoining structures, thereby minimizing the potential for leak and perforation. Consequently, the innovation has advanced our ability to carry out complex therapeutic interventions and obviate the need for high-risk surgical procedures, particularly in morbid patients. However, a keen awareness of the technical design of LAMS and its implication on treatment effect is important to minimize adverse events and to improve clinical outcomes. This review outlines the technical approach to placement of LAMS, procedural challenges, adverse events, current clinical indications and emerging applications.
C1 [Bang, Ji Young; Varadarajulu, Shyam] AdventHlth Orlando, Ctr Intervent Endoscopy, 601 East Rollins St, Orlando, FL 32803 USA.
C3 Adventist Health Services; AdventHealth; (AdventHealth) Central Florida
Division; Central Florida Hospital - South; AdventHealth Orlando
RP Varadarajulu, S (corresponding author), AdventHlth Orlando, Ctr Intervent Endoscopy, 601 East Rollins St, Orlando, FL 32803 USA.
EM svaradarajulu@yahoo.com
CR Anderloni A, 2019, GASTROINTEST ENDOSC, V89, P69, DOI [10.1016/j.gie.2018.04.1746, 10.1016/j.gie.2018.08.047]
Ang TL, 2017, DIGEST ENDOSC, V29, P463, DOI 10.1111/den.12797
[Anonymous], GUT
Bang JY, 2018, ANN SURG, V267, P561, DOI 10.1097/SLA.0000000000002082
Bang JY, 2017, GUT, V66, P2054, DOI 10.1136/gutjnl-2016-312812
Bang JY, 2017, DIGEST ENDOSC, V29, P83, DOI 10.1111/den.12681
Brimhall B, 2018, CLIN GASTROENTEROL H, V16, P1521, DOI 10.1016/j.cgh.2018.02.021
Bukhari M, 2018, GASTROINTEST ENDOSC, V88, P486, DOI 10.1016/j.gie.2018.04.2356
Chen YI, 2018, ENDOSC INT OPEN, V6, pE780, DOI 10.1055/a-0611-5082
Chen YI, 2018, GASTROINTEST ENDOSC, V88, P267, DOI 10.1016/j.gie.2018.03.021
Chen YI, 2017, SURG ENDOSC, V31, P2946, DOI 10.1007/s00464-016-5311-1

Ge PS, 2019, SURG ENDOSC, V33, P3404, DOI 10.1007/s00464-018-06636-3
Irani S, 2017, GASTROINTEST ENDOSC, V85, P1285, DOI 10.1016/j.gie.2016.08.028
Irani S, 2017, CLIN GASTROENTEROL H, V15, P738, DOI 10.1016/j.cgh.2016.12.021
Itoi T, 2017, DIGEST ENDOSC, V29, P495, DOI 10.1111/den.12794
Itoi T, 2016, GUT, V65, P193, DOI 10.1136/gutjnl-2015-310348
Jacques J, 2018, ENDOSCOPY
Jang JW, 2012, GASTROENTEROLOGY, V142, P805, DOI 10.1053/j.gastro.2011.12.051
Khashab MA, 2015, GASTROINTEST ENDOSC, V82, P932, DOI 10.1016/j.gie.2015.06.017
Law R, 2016, CLIN GASTROENTEROL H, V14, P476, DOI 10.1016/j.cgh.2015.10.026
Manta R, 2018, EUR J GASTROEN HEPAT, V30, P695, DOI 10.1097/MEG.0000000000001112
Mukai S, 2015, ENDOSCOPY, V47, P47, DOI 10.1055/s-0034-1377966
Tsou YK, 2016, SCAND J GASTROENTERO, V51, P95, DOI 10.3109/00365521.2015.1056223
Tsuchiya T, 2018, GASTROINTEST ENDOSC, V87, P1138, DOI 10.1016/j.gie.2017.08.017
Varadarajulu S, 2013, GASTROENTEROLOGY, V145, P583, DOI 10.1053/j.gastro.2013.05.046
Yang JL, 2019, ENDOSCOPY, V51, P1035, DOI 10.1055/a-0759-1353
NR 26
TC 58
Z9 66
U1 1
U2 2
PU WILEY
PI HOBOKEN
PA 111 RIVER ST, HOBOKEN 07030-5774, NJ USA
SN 0915-5635
EI 1443-1661
J9 DIGEST ENDOSC
JI Dig. Endosc.
PD NOV
PY 2019
VL 31
IS 6
BP 619
EP 626
DI 10.1111/den.13428
PG 8
WC Gastroenterology & Hepatology; Surgery
WE Science Citation Index Expanded (SCI-EXPANDED)
SC Gastroenterology & Hepatology; Surgery
GA JK2XF
UT WOS:000494709200013
PM 31050068
DA 2026-05-09

ER

PT J
AU Ginestet, C
Sanglier, F
Hummel,
Rouchaud, A
Legros, R
Lepetit, H
Dahan, M
Carrier, P
Loustaud-Ratti,
Sautereau, D
Albouys, J
Jacques, J
Geyl, S
AF Ginestet, C.
Sanglier, F.
Hummel, V
Rouchaud, A.
Legros, R.
Lepetit, H.
Dahan, M.
Carrier, P.
Loustaud-Ratti, V
Sautereau, D.
Albouys, J.
Jacques, J.
Geyl, S.
TI EUS-guided biliary drainage with electrocautery-enhanced lumen-apposing metal stent placement should replace PTBD after ERCP failure in patients with distal tumoral biliary obstruction: a large real-life study
SO SURGICAL ENDOSCOPY AND OTHER INTERVENTIONAL TECHNIQUES
LA English
DT Article
DE ERCP failure; Malignant biliary obstruction; EUS-BD; Electrocautery-enhanced lumen-apposing metal stent; PTBD
ID PERCUTANEOUS DRAINAGE; FAILED ERCP; CHOLEDOCHODUODENOSTOMY; MULTICENTER
AB Aims In cases of malignant distal biliary obstruction, ERCP is the preferred technique for bile duct drainage. In case of failure, the alternative techniques are percutaneous transhepatic biliary drainage (PTBD) and more recently endoscopic ultrasound-guided biliary drainage. A new type of stent called the electrocautery-enhanced lumen-apposing metal stent (EC-LAMS) has been developed to enable the performance of biliary-enteric anastomosis under EUS-guidance in a single step,

without prior bile duct puncture or the need for a guidewire. The aim of our study was to compare the real-life efficacies of PTBD and EUS-BD with the EC-LAMS for cases of ERCP failure in patients with malignant biliary obstruction. Methods We performed a monocentric retrospective study comparing PTBD and EUS-BD with the use of electrocautery-enhanced lumen-apposing metal stent in the context of a malignant distal biliary obstruction after ERCP failure. Results 95 patients were included (50 in EUS-BD group and 45 in PTBD group). The main etiology of malignant obstruction was adenocarcinoma of the head of pancreas (85%). There was a significant difference in favor of endoscopic ultrasound-guided biliary drainage using electrocautery-enhanced lumen-apposing metal stent for the following criteria: clinical success: 89.3% vs. 45.5%; $p < 0.0001$; procedure-related adverse event rate: 2.12% vs. 22.7%; $p = 0.003$; duration of post-drainage hospitalization: 3.5 vs. 8.2 days; $p < 0.0001$, overall survival (median survival): 118.2 vs. 42 days; $p = 0.012$, overall cost of the strategy per patient: 5098 vs. 9363 euros; $p < 0.001$. Conclusion Our results are in favor of EUS-BD using electrocautery-enhanced lumen-apposing metal stent in case of ERCP failure for a distal tumor biliary obstruction. Operators performing ERCP for distal tumor biliary obstruction must learn this backup procedure because of its superiority over percutaneous transhepatic biliary drainage in terms of clinical success, safety, cost, and overall survival.
C1 [Ginestet, C.; Legros, R.; Lepetit, H.; Dahan, M.; Carrier, P.; Loustaud-Ratti, V; Sautereau, D.; Albouys, J.; Jacques, J.; Geyl, S.] CHU Dupuytren, CHU Limoges Hop Dupuytren, Dept HepatoGastro Enterol, Serv HepatoGastro Enterol, F-87042 Limoges, France.

[Sanglier, F.; Hummel, V; Rouchaud, A.] CHU Dupuytren, CHU Limoges Hop Dupuytren, Dept Radiol, Serv Radiol, F-87042 Limoges, France.

C3 CHU Limoges; CHU Limoges

RP Ginestet, C (corresponding author), CHU Dupuytren, CHU Limoges Hop Dupuytren, Dept HepatoGastro Enterol, Serv HepatoGastro Enterol, F-87042 Limoges, France.

EM claireginestet@hotmail.fr; florian.sanglier@chu-limoges.fr;
yhummel@gmail.com; aymeric.rouchaud@unilim.fr; rom1.legros@gmail.com;
hugo.lepetit@hotmail.fr; martindahan@hotmail.fr;
paul.carrier@chu-limoges.fr; veronique.loustaud-ratti@unilim.fr;
denis.sautereau@unilim.fr; jeremie.albouys@gmail.com;
jeremiejacques@gmail.com; sophiegeyl@hotmail.fr

RI Rouchaud, Aymeric/E-8667-2017

OI Rouchaud, Aymeric/0000-0003-0902-3375; Ginestet,
Claire/0000-0003-3096-3097
CR Anderloni A, 2019, GASTROINTEST ENDOSC, V89, P69, DOI [10.1016/j.gie.2018.04.1746, 10.1016/j.gie.2018.08.047]
Artifon ELA, 2012, J CLIN GASTROENTEROL, V46, P768, DOI 10.1097/MCG.0b013e31825f264c
Bapaye A, 2013, UNITED EUR GASTROENT, V1, P285, DOI 10.1177/2050640613490928
Bill JG, 2016, ENDOSC INT OPEN, V4, pE980, DOI 10.1055/s-0042-112584
Cotton PB, 2010, GASTROINTEST ENDOSC, V71, P446, DOI 10.1016/j.gie.2009.10.027
Dumonceau JM, 2018, ENDOSCOPY, V50, P910, DOI 10.1055/a-0659-9864
Ekkelenkamp VE, 2015, ENDOSCOPY, V47, P504, DOI 10.1055/s-0034-1391231
Giovannini M, 2001, ENDOSCOPY, V33, P898, DOI 10.1055/s-2001-17324
Jacques J, 2020, GASTROINTEST ENDOSC, V92, P134, DOI 10.1016/j.gie.2020.01.055
Jacques J, 2019, ENDOSCOPY, V51, P540, DOI 10.1055/a-0735-9137
Khashab MA, 2015, DIGEST DIS SCI, V60, P557, DOI 10.1007/s10620-014-3300-6

Krishnamoorthi R, 2020, SURG ENDOSC, V34, P2866, DOI 10.1007/s00464-020-07484-w
Kunda R, 2016, SURG ENDOSC, V30, P5002, DOI 10.1007/s00464-016-4845-6
Lee TH, 2016, CLIN GASTROENTEROL H, V14, P1011, DOI 10.1016/j.cgh.2015.12.032
Poincloux L, 2015, ENDOSCOPY, V47, P794, DOI [10.1055/s-0035-1545399, 10.1055/s-0034-1391988]
Sharaiha RZ, 2017, GASTROINTEST ENDOSC, V85, P904, DOI 10.1016/j.gie.2016.12.023
Sharaiha RZ, 2016, SURG ENDOSC, V30, P5500, DOI 10.1007/s00464-016-4913-y
Sportes A, 2017, THER ADV GASTROENTER, V10, P483, DOI 10.1177/1756283X17702096
Téllez-Avila FI, 2018, SURG LAPARO ENDO PER, V28, P183, DOI 10.1097/SLE.0000000000000528
Tsuchiya T, 2018, GASTROINTEST ENDOSC, V87, P1138, DOI 10.1016/j.gie.2017.08.017
Williams EJ, 2012, ENDOSCOPY, V44, P674, DOI 10.1055/s-0032-1309345
NR 21
TC 30
Z9 32
U1 0
U2 5
PU SPRINGER
PI NEW YORK
PA ONE NEW YORK PLAZA, SUITE 4600, NEW YORK, NY, UNITED STATES
SN 0930-2794
EI 1432-2218
J9 SURG ENDOSC
JI Surg. Endosc.
PD MAY
PY 2022
VL 36
IS 5
BP 3365
EP 3373
DI 10.1007/s00464-021-08653-1
EA OCT 2021
PG 9
WC Surgery
WE Science Citation Index Expanded (SCI-EXPANDED)
SC Surgery
GA 0M4BB
UT WOS:000703375600001
PM 34606007
DA 2026-05-09
ER

PT J
AU Khashab, MA

Varadarajulu, S
AF Khashab, Mouen A.
Varadarajulu, Shyam
TI Endoscopic ultrasonography as a therapeutic modality
SO CURRENT OPINION IN GASTROENTEROLOGY
LA English
DT Review
DE biliary drainage; endoscopic ultrasonography; endoscopic retrograde cholangiopancreatography; pseudocysts; walled-off pancreatic necrosis
ID GUIDED BILIARY DRAINAGE; PANCREATIC FLUID COLLECTIONS; EXPANDABLE METAL STENT; TRANSLUMINAL DRAINAGE; RANDOMIZED-TRIAL; FAILED ERCP; EUS; NECROSECTOMY; PLACEMENT; MULTICENTER
AB Purpose of review

Endoscopic ultrasonography (EUS) has taken on more of a therapeutic role in recent years. This review will focus on the therapeutic applications of EUS.

Recent findings

Multiple studies on the therapeutic applications of EUS have been published. EUS facilitates endoscopic drainage of pancreatic fluid collections (PFCs) including walled-off pancreatic necrosis, management of refractory gastrointestinal bleeding from gastric varix or vasculature by fine-needle injection and decompression of obstructive pancreatic or biliary ductal systems following failed access by standard endoscopic or radiological techniques.

Summary

The indications and role of therapeutic EUS have expanded rapidly in recent years. The procedures can be technically challenging, requiring expertise in both endosonography and endoscopic retrograde cholangiopancreatography. Refinement in echoendoscope design and dedicated accessories are required to further expand the applications of therapeutic EUS.
C1 [Khashab, Mouen A.] Johns Hopkins Med Inst, Dept Med, Div Gastroenterol & Hepatol, Baltimore, MD 21205 USA.
[Varadarajulu, Shyam] Univ Alabama Birmingham, Med Sch Birmingham, Div Gastroenterol & Hepatol, Birmingham, AL USA.
C3 Johns Hopkins University; Johns Hopkins Medicine; University of Alabama System; University of Alabama Birmingham
RP Khashab, MA (corresponding author), Johns Hopkins Univ Hosp, NCB Zayed Tower,1800 Orleans St,Suite 7125B, Baltimore, MD 21205 USA.
EM mkhasha1@jhmi.edu
RI khashab, mouen/F-6643-2017
CR Artifon ELA, 2011, J PANCREAS, V12, P610
Artifon ELA, 2011, GASTROINTEST ENDOSC, V73, P1317, DOI 10.1016/j.gie.2010.10.041
Bakker OJ, 2012, JAMA-J AM MED ASSOC, V307, P1053, DOI 10.1001/jama.2012.276
Binmoeller KF, 2011, GASTROINTEST ENDOSC, V74, P1019, DOI 10.1016/j.gie.2011.06.030
Binmoeller KF, 2011, J HEPATO-BIL-PAN SCI, V18, P319, DOI 10.1007/s00534-010-0358-1
Chavalitdhamrong D, 2012, WORLD J GASTROENTERO, V18, P491, DOI 10.3748/wjg.v18.i6.491
Chennat J, 2011, GASTROENTEROLOGY, V140, P1403, DOI 10.1053/j.gastro.2011.03.003
DiMaio CJ, 2011, PANCREAS, V40, P664, DOI 10.1097/MPA.0b013e3182128d06
Ergun M, 2011, ENDOSCOPY, V43, P518, DOI 10.1055/s-0030-1256333

Giovannini M, 2012, GASTROENT RES PRACT, V2012, DOI 10.1155/2012/348719
Gonzalez JM, 2012, ENDOSCOPY, V44, P539, DOI 10.1055/s-0031-1291609
Hara K, 2011, INTERNAL MED, V50, P991, DOI 10.2169/internalmedicine.50.4738
Henry WA, 2011, GASTROINTEST ENDOSC
Itoi T, 2012, GASTROINTEST ENDOSC, V75, P870, DOI 10.1016/j.gie.2011.10.020
Itoi T, 2011, J HEPATO-BIL-PAN SCI, V18, P664, DOI 10.1007/s00534-011-0410-9
Kahaleh M, 2007, GASTROINTEST ENDOSC, V65, P224, DOI 10.1016/j.gie.2006.05.008
Khashab M, SURG ENDOSC 1216
Khashab MA, 2010, ENDOSCOPY, V42, pE292, DOI 10.1055/s-0030-1255808
Khashab MA, 2012, GASTROINTEST ENDOSC, V76, P209, DOI 10.1016/j.gie.2012.03.170
Kikuyama M, 2011, GASTROINTEST ENDOSC, V73, P376, DOI 10.1016/j.gie.2010.10.015
Komaki T, 2011, PANCREATOLOGY, V11, P47, DOI 10.1159/000323508
Levy MJ, 2008, AM J GASTROENTEROL, V103, P352, DOI 10.1111/j.1572-0241.2007.01616.x
Nguyen-Tang T, 2010, ENDOSCOPY, V42, P232, DOI 10.1055/s-0029-1243858
Park DH, 2011, GASTROINTEST ENDOSC, V74, P1276, DOI 10.1016/j.gie.2011.07.054
PESSA ME, 1987, ANN SURG, V205, P389, DOI 10.1097/00000658-198704000-00008
Ramírez-Luna MA, 2011, ENDOSCOPY, V43, P826, DOI 10.1055/s-0030-1256406
Seifert H, 2009, GUT, V58, P1260, DOI 10.1136/gut.2008.163733
Shah JN, 2012, GASTROINTEST ENDOSC, V75, P56, DOI 10.1016/j.gie.2011.08.032
Shami Vanessa M, 2007, Gastrointest Endosc Clin N Am, V17, P581, DOI 10.1016/j.giec.2007.05.015
Shami VM, 2007, GASTROINTEST ENDOSC, V17, pvii
Sharaiha RZ, 2012, GASTROINTEST ENDOSC, V76, P227, DOI 10.1016/j.gie.2012.01.044
SMITH AC, 1994, LANCET, V344, P1655, DOI 10.1016/S0140-6736(94)90455-3
Tessier G, 2007, GASTROINTEST ENDOSC, V65, P233, DOI 10.1016/j.gie.2006.06.029
van Santvoort HC, 2010, NEW ENGL J MED, V362, P1491, DOI 10.1056/NEJMoa0908821
Varadarajulu S, 2008, GASTROINTEST ENDOSC, V68, P1102, DOI 10.1016/j.gie.2008.04.028
Varadarajulu S, 2011, AM SURGEON, V77, P1650
Varadarajulu S, 2011, GASTROINTEST ENDOSC, V74, P1408, DOI 10.1016/j.gie.2011.07.049
Varadarajulu S, 2011, GASTROINTEST ENDOSC, V74, P418, DOI 10.1016/j.gie.2011.03.1242
Varadarajulu S, 2011, GASTROINTEST ENDOSC, V74, P74, DOI 10.1016/j.gie.2011.03.1122
Varadarajulu S, 2009, GASTROINTEST ENDOSC, V69, pS200, DOI 10.1016/j.gie.2008.12.032
Voermans RP, 2011, GASTROINTEST ENDOSC, V74, P1285, DOI 10.1016/j.gie.2011.07.059
Weilert F, 2011, ENDOSCOPY, V43, P1105, DOI 10.1055/s-0030-1256961
Will Uwe, 2011, Therap Adv Gastroenterol, V4, P213, DOI 10.1177/1756283X10394232
NR 43
TC 21
Z9 21
U1 0
U2 4
PU LIPPINCOTT WILLIAMS & WILKINS
PI PHILADELPHIA
PA TWO COMMERCE SQ, 2001 MARKET ST, PHILADELPHIA, PA 19103 USA

SN 0267-1379
EI 1531-7056
J9 CURR OPIN GASTROEN
JI Curr. Opin. Gastroenterol.
PD SEP
PY 2012
VL 28
IS 5
BP 467
EP 476
DI 10.1097/MOG.0b013e32835672b4
PG 10
WC Gastroenterology & Hepatology
WE Science Citation Index Expanded (SCI-EXPANDED)
SC Gastroenterology & Hepatology
GA 991DI
UT WOS:000307681300008
PM 22885945
DA 2026-05-09
ER

PT J
AU Mangiavillano, B
Ramai, D
Kahaleh, M
Tyberg, A
Shahid, H
Sarkar, A
Samanta, J
Dhar, J
Bronswijk, M
Van der Merwe, S
Kouanda, A
Ji, H
Dai, SC
Deprez, P
Vargas-Madrigal, J
Vanella, G
Roberto, L
Arcidiacono, PG
Robles-Medranda, C
Vasquez, JA

Arevalo-Mora, M
Fugazza, A
Ko, C
Morris, J
Lisotti, A
Fusaroli, P
Dhaliwal, A
Mutignani, M
Forti, E
Cottone, I
Larghi, A
Rizzatti, G
Galasso, D
Barbera, C
Di Matteo, FM
Stigliano, S
Binda, C
Fabbri, C
Pham, KD
Di Mitri, R
Amata, M
Crinó, SF
Ofosu, A
De Luca, L
Al-Lehibi, A
Auriemma, F
Paduano, D
Calabrese, F
Gentile, C
Hassan, C
Repici, A
Facciorusso, A
AF Mangiavillano, Benedetto
Ramai, Daryl
Kahaleh, Michel
Tyberg, Amy
Shahid, Haroon
Sarkar, Avik
Samanta, Jayanta
Dhar, Jahnvi
Bronswijk, Michiel
Van der Merwe, Schalk

Kouanda, Abdul
Ji, Hyun
Dai, Sun-Chuan
Deprez, Pierre
Vargas-Madrigal, Jorge
Vanella, Giuseppe
Roberto, Leone
Arcidiacono, Paolo Giorgio
Robles-Medranda, Carlos
Vasquez, Juan Alcivar
Arevalo-Mora, Martha
Fugazza, Alessandro
Ko, Christopher
Morris, John
Lisotti, Andrea
Fusaroli, Pietro
Dhaliwal, Amaninder
Mutignani, Massimiliano
Forti, Edoardo
Cottone, Irene
Larghi, Alberto
Rizzatti, Gianenrico
Galasso, Domenico
Barbera, Carmelo
Di Matteo, Francesco Maria
Stigliano, Serena
Binda, Cecilia
Fabbri, Carlo
Pham, Khanh Do-Cong
Di Mitri, Roberto
Amata, Michele
Crino, Stefano Francesco
Ofosu, Andrew
De Luca, Luca
Al-Lehibi, Abed
Auriemma, Francesco
Paduano, Danilo
Calabrese, Federica
Gentile, Carmine
Hassan, Cesare
Repici, Alessandro
Facciorusso, Antonio

TI Outcomes of lumen apposing metal stent placement in patients with
surgically altered anatomy: Multicenter international experience
SO ENDOSCOPY INTERNATIONAL OPEN
LA English
DT Article
DE Endoscopy Lower GI Tract; Stenting; Endoscopy Small Bowel; Endoscopic
ultrasonography
ID EUS; EFFICACY; SAFETY; EDGE
AB Background and study aims Although outcomes of lumen-apposing metal stents (LAMS) placement in native anatomy have been reported, data on LAMS placement in surgically altered anatomy (SAA) are sparse. We aimed to assess outcomes of LAMS placement in patients with SAA for different indications. Patients and methods This was an international, multicenter, retrospective, observational study at 25 tertiary care centers through November 2023. Consecutive patients with SAA who underwent LAMS placement were included. The primary outcome was technical success defined as correct placement of LAMS. Secondary outcomes were clinical success and safety. Results Two hundred and seventy patients (125 males; average age 61 +/- 15 years) underwent LAMS placement with SAA. Procedures included EUS-directed transgastric ERCP (EDGE) and EUS-directed transenteric ERCP (EDEE) (n = 82), EUS-guided entero-enterostomy (n = 81), EUS-guided biliary drainage (n = 57), EUS-guided drainage of peri-pancreatic fluid collections (n = 48), and EUS-guided pancreaticogastrostomy (n = 2). Most cases utilized AXIOS stents (n = 255) compared with SPAXUS stents (n = 15). Overall, technical success was 98%, clinical success was 97%, and the adverse event (AE) rate was 12%. Using AGREE classification, five events were rated as Grade II, 21 events as Grade IIIa, and six events as IIIb. No difference in AEs were noted among stent types (P = 0.52). Conclusions This study shows that placement of LAMS is associated with high technical and clinical success rates in patients with SAA. However, the rate of AEs is noteworthy, and thus, these procedures should be performed by expert endoscopists at tertiary centers.
C1 [Mangiavillano, Benedetto] Ist Clin Mater Domini Casa Cura Privata SpA, Gastrointestinal Endoscopy, Via Gerenzano 2, I-21053 Castellanza, Italy.
[Ramai, Daryl; Ko, Christopher; Morris, John] Univ Utah Hlth, Gastroenterol & Hepatol, Salt Lake City, UT USA.
[Kahaleh, Michel] Fdn Intervent & Therapeut Endoscopy, New Brunswick, NJ USA.
[Tyberg, Amy; Shahid, Haroon; Sarkar, Avik] Hackensack Meridian Hosp, Div Gastroenterol, Hackensack, NJ USA.
[Samanta, Jayanta; Dhar, Jahnvi] Post Grad Inst Med Educ & Res, Gastroenterol, Chandigarh, India.
[Bronswijk, Michiel] Katholieke Univ Leuven, Univ Hosp Leuven, Gastroenterol & Hepatol, Leuven, Belgium.
[Van der Merwe, Schalk] Univ Pretoria, Dept Immunol, Hepatol & GI Res Lab, Pretoria, South Africa.
[Van der Merwe, Schalk] Katholieke Univ Leuven, Dept Hepatol & Biliopancreat Dis, Leuven, Belgium.
[Kouanda, Abdul] Univ Calif San Francisco, Gastroenterol, Med Ctr Parnassus, San Francisco, CA 94143 USA.
[Ji, Hyun] Univ Calif San Francisco, Gastroenterol, San Francisco, CA 94143 USA.
[Dai, Sun-Chuan] Univ Calif San Francisco, Med Ctr, Gastroenterol Div, San Francisco, CA USA.
[Deprez, Pierre] Clin Univ St Luc, Gastroenterol, Brussels, Belgium.
[Vargas-Madrigal, Jorge] Enrique Baltodano Briceno Hosp, Gastroenterol Dept, Liberia, Costa Rica.
[Vanella, Giuseppe] Univ Vita Salute San Raffaele, San Raffaele Sci Inst IRCCS, Pancreas Translat & Clin Res Ctr, San Raffaele Sci Inst,Pancreatobiliary Endoscopy, Milan, Italy.
[Roberto, Leone] Univ Vita Salute San Raffaele, Med, Milan, Italy.
[Arcidiacono, Paolo Giorgio] Univ Vita Salute San Raffaele, IRCCS San Raffaele Hosp, Gastroenterol & Gastrointestinal Endoscopy, Milan, Italy.

[Robles-Medranda, Carlos; Vasquez, Juan Alcivar] Inst Ecuatoriano Enfermedades Digest IECED, Gastroenterol, Guayaquil, Ecuador.

[Robles-Medranda, Carlos] Omni Hosp, Endoscopy, Guayaquil, Ecuador.

[Arevalo-Mora, Martha] Inst Ecuatoriano Enfermedades Digest, IECED, Guayaquil, Ecuador.

[Fugazza, Alessandro] Humanitas Res Hosp, Div Gastroenterol, Digest Endoscopy Unit, Rozzano, Italy.

[Lisotti, Andrea; Fusaroli, Pietro] Univ Bologna, Hosp Imola, Gastroenterol Unit, Imola, Italy.

[Dhaliwal, Amaninder] Univ Nebraska Med Ctr, Div Gastroenterol, Omaha, NE USA.

[Mutignani, Massimiliano] UOC Digest Endoscopy Unit, Surg Dept, Milan, Italy.

[Forti, Edoardo; Cottone, Irene] Osped Niguarda Ca Granda, Digest Endoscopy, Milan, Italy.

[Larghi, Alberto] Univ Cattolica Sacro Cuore, Digest Endoscopy Unit, Rome, Italy.

[Rizzatti, Gianenrico] Fdn Policlin Univ A Gemelli IRCCS, Digest Endoscopy Unit, Rome, Italy.

[Galasso, Domenico] Hop Riviera Chablais, Dept Med, Gastroenterol Unit, Rennaz, Switzerland.

[Barbera, Carmelo] Nuovo Osped Civile S Agostino Estense, Gastroenterol & Digest Endoscopy Unit, Baggiovara di Modena, MO, Italy.

[Barbera, Carmelo] Azienda Unidad Sanitaria Locale Modena, Gastroenterol & Digest Endoscopy Unit, Modena, Italy.

[Di Matteo, Francesco Maria] Campus Biomed Univ Hosp, Operat Endoscopy Dept, Rome, Italy.

[Stigliano, Serena] Campus Biomed Univ Hosp, Therapeut GI Endoscopy Unit, Rome, Italy.

[Binda, Cecilia] Morgagni Pierantoni Hosp, Unit Gastroenterol & Digest Endoscopy, Forli, Italy.

[Binda, Cecilia] Maurizio Bufalini Hosp, Unit Gastroenterol & Digest Endoscopy, Cesena, Italy.

[Fabbri, Carlo] Azienda Unita Sanitaria Locale Romagna, Forli Cesena Hosp, Digest Endoscopy & Gastroenterol Unit, Forli Cesena, Italy.

[Pham, Khanh Do-Cong] Haukeland Hosp, Med, Bergen, Norway.

[Pham, Khanh Do-Cong] Univ Bergen, Clin Med K1, Bergen, Norway.

[Di Mitri, Roberto] ARNAS Civ Di Cristina Benfratelli Hosp, Gastroenterol & Endoscopy Unit, Palermo, Italy.

[Amata, Michele] UPMC, IsMeTT, Gastroenterol, Palermo, Italy.

[Crino, Stefano Francesco] Univ Verona, Pancreas Inst, Gastroenterol & Digest Endoscopy Unit, Verona, Italy.

[Ofosu, Andrew] Brooklyn Hosp Ctr, Gastroenterol & Hepatol, Brooklyn, NY USA.

[De Luca, Luca] Azienda Osped Ospedali Riuniti Marche Nord, Gastroenterol & Digest Endoscopy, Pesaro, Italy.

[Al-Lehibi, Abed] King Fahad Med City, Gastroenterol & Hepatol, Riyadh, Saudi Arabia.

[Auriemma, Francesco] Univ Federico II, Gastroenterol, Naples, Italy.

[Auriemma, Francesco] Ist Clin Humanitas, Div Gastroenterol, Digest Endoscopy Unit, Rozzano, Italy.

[Paduano, Danilo] Humanitas Mater Domini, Gastrointestinal Endoscopy Unit, Castellanza, Italy.

[Calabrese, Federica; Gentile, Carmine] Humanitas Mater Domini, Gastrointestinal Endoscopy Unit, Castellanza, Varese, Italy.

[Hassan, Cesare] Humanitas Univ, Dept Biomed Sci, Milan, Italy.

[Hassan, Cesare] IRCCS Humanitas Clin & Res Ctr, Endoscopy Unit, Milan, Italy.

[Repici, Alessandro] Humanitas Univ, Dept Biomed Sci, Milan, Italy.

[Repici, Alessandro] IRCCS Humanitas Res Hosp, Digest Endoscopy Unit, Rozzano, Italy.

[Facciorusso, Antonio] Gastroenterol, Med Sci, Foggia, Italy.

C3 Utah System of Higher Education; University of Utah; Post Graduate
Institute of Medical Education & Research (PGIMER), Chandigarh; KU
Leuven; University Hospital Leuven; University of Pretoria; KU Leuven;

University of California System; University of California San Francisco;
University of California System; University of California San Francisco;
University of California System; University of California San Francisco;
Universite Catholique Louvain; Cliniques Universitaires Saint-Luc;
Vita-Salute San Raffaele University; IRCCS Ospedale San Raffaele;
Vita-Salute San Raffaele University; Vita-Salute San Raffaele
University; IRCCS Ospedale San Raffaele; IRCCS Humanitas Research
Hospital; Azienda USL di Imola; University of Bologna; University of
Nebraska System; University of Nebraska Medical Center; IRCCS Ca Granda
Ospedale Maggiore Policlinico; Ospedale Niguarda Ca' Granda; Catholic
University of the Sacred Heart; IRCCS Policlinico Gemelli; Catholic
University of the Sacred Heart; IRCCS Policlinico Gemelli; University
Campus Bio-Medico - Rome Italy; Fondazione Policlinico Universitario
Campus Bio-Medico; University Campus Bio-Medico - Rome Italy; Fondazione
Policlinico Universitario Campus Bio-Medico; Ospedale Maurizio Bufalini;
University of Bergen; Haukeland University Hospital; University of
Bergen; A.R.N.A.S. Ospedali Civico Di Cristina Benfratelli; IRCCS
Istituto Mediterraneo per i Trapianti e Terapie ad Alta Specializzazione
(ISMETT); University of Verona; Brooklyn Hospital Center; King Fahad
Medical City; University of Naples Federico II; IRCCS Humanitas Research
Hospital; Humanitas University; Humanitas University
RP Mangiavillano, B (corresponding author), Ist Clin Mater Domini Casa Cura Privata SpA, Gastrointestinal Endoscopy, Via Gerenzano 2, I-21053 Castellanza, Italy.
EM bennymangiavillano@gmail.com
RI Vanella, Giuseppe/LQI-8930-2024; Robles-Medranda, Carlos/AFY-9788-2022;
Amata, Michele/KPB-0825-2024; Dhaliwal, Amaninder/AAJ-3827-2021; Repici,
Alessandro/HFH-8162-2022; Stigliano, SERENA/AAC-1821-2022; Arcidiacono,
Paolo/K-2122-2018; Larghi, Alberto/AAC-7448-2022; Facciorusso,
Antonio/AAA-5966-2019; Binda, Cecilia/ABH-1588-2020; hassan,
cesare/H-2844-2012; Fusaroli, Pietro/AAC-2182-2020; Di Mitri,
Roberto/AAC-7452-2022; Rizzatti, Gianenrico/B-1406-2019; Fugazza,
Alessandro/ABG-9381-2021; Di Matteo, Francesco Maria/AAC-1076-2022;
Gentile, Carmine/G-6446-2017; Lisotti, Andrea/L-6762-2019; Kahaleh,
Michel/A-9408-2011; Cottone, Irene/NXY-3795-2025
OI Van der Merwe, Schalk/0000-0002-9891-2686; Alcívar-Vásquez,
Juan/0000-0002-1869-9230; Bronswijk, Michiel/0000-0003-2039-5022;
Arevalo-Mora, Martha/0000-0003-2561-8512; Mangiavillano,
Benedetto/0000-0003-0611-7448; Kahaleh, Michel/0000-0003-0836-6114;
CR Amato A, 2022, GUT, V71, P1050, DOI 10.1136/gutjnl-2022-326880
Bhenswala P, 2021, J CLIN GASTROENTEROL, V55, P641, DOI 10.1097/MCG.0000000000001566
Chowdhury SD, 2016, TECH GASTROINTEST EN, V18, P98, DOI 10.1016/j.tgie.2016.07.001
Crinò SF, 2019, J GASTROINTEST LIVER, V28, P337

Deliwala SS, 2023, SURG ENDOSC, V37, P4144, DOI 10.1007/s00464-023-09926-7
Facciorusso A, 2022, GASTROINTEST ENDOSC, V95, P1158, DOI 10.1016/j.gie.2021.12.006
Giri S, 2023, THER ADV GASTROINTES, V16, DOI 10.1177/26317745231199364
Hayat U, 2020, ENDOSC INT OPEN, V8, pE196, DOI 10.1055/a-1005-6573
Ichkhanian Y, 2023, CURR OPIN GASTROEN, V39, P348, DOI 10.1097/MOG.0000000000000965
Kedia P, 2014, GASTROENTEROLOGY, V147, P566, DOI 10.1053/j.gastro.2014.05.045
Koutlas NJ, 2023, DIAGNOSTICS, V13, DOI 10.3390/diagnostics13111933
Mangiavillano B, 2022, GASTROINTEST ENDOSC, V95, P115, DOI 10.1016/j.gie.2021.07.021
Nass KJ, 2022, GASTROINTEST ENDOSC, V95, P1078, DOI 10.1016/j.gie.2021.11.038
Runge TM, 2021, ENDOSCOPY, V53, P611, DOI 10.1055/a-1254-3942
Sharaiha RZ, 2017, GASTROINTEST ENDOSC, V85, P904, DOI 10.1016/j.gie.2016.12.023
Sharma P, 2020, WORLD J GASTROENTERO, V26, P2715, DOI 10.3748/wjg.v26.i21.2715
Spadaccini M, 2023, DIAGNOSTICS, V13, DOI 10.3390/diagnostics13243623
Tanisaka Y, 2023, SCAND J GASTROENTERO, V58, P107, DOI 10.1080/00365521.2022.2111228
Teoh AYB, 2020, GUT, V69, P1085, DOI 10.1136/gutjnl-2019-319996
Teoh AYB, 2024, LANCET GASTROENTEROL, V9, P124, DOI 10.1016/S2468-1253(23)00242-X
Tyberg A, 2020, J CLIN GASTROENTEROL, V54, P569, DOI 10.1097/MCG.0000000000001326
van der Merwe SW, 2022, ENDOSCOPY, V54, P185, DOI 10.1055/a-1717-1391
NR 22
TC 7
Z9 7
U1 0
U2 1
PU GEORG THIEME VERLAG KG
PI STUTTGART
PA Oswald-Hesse-Strasse 50, D-70469 STUTTGART, GERMANY
SN 2364-3722
EI 2196-9736
J9 ENDOSC INT OPEN
JI Endosc. Int. Open
PD OCT
PY 2024
VL 12
IS 10
BP E1143
EP E1149
DI 10.1055/a-2411-1814
PG 7
WC Gastroenterology & Hepatology; Surgery
WE Emerging Sources Citation Index (ESCI)
SC Gastroenterology & Hepatology; Surgery
GA J3X0C

UT WOS:001336414300002
PM 39398449
OA Green Submitted, gold
DA 2026-05-09
ER

PT J
AU Capurso, G
Rizzo, GEM
Coluccio, C
Crinò, SF
Cucchetti, A
Facciorusso, A
Hassan, C
Amato, A
Auriemma, F
Bertani, H
Binda, C
Cipolletta, F
Forti, E
Fugazza, A
Lisotti, A
Maida, M
Sinagra, E
Sbrancia, M
Spadaccini, M
Tacelli, M
Vanella, G
Anderloni, A
Fabbri, C
Tarantino, I
AF Capurso, Gabriele
Rizzo, Giacomo Emanuele Maria
Coluccio, Chiara
Crino, Stefano Francesco
Cucchetti, Alessandro
Facciorusso, Antonio
Hassan, Cesare
Amato, Arnaldo
Auriemma, Francesco
Bertani, Helga
Binda, Cecilia

Cipolletta, Fabio
Forti, Edoardo
Fugazza, Alessandro
Lisotti, Andrea
Maida, Marcello
Sinagra, Emanuele
Sbrancia, Monica
Spadaccini, Marco
Tacelli, Matteo
Vanella, Giuseppe
Anderloni, Andrea
Fabbri, Carlo
Tarantino, Ilaria
CA i-EUS working grp
TI The i-EUS consensus on the management of pancreatic fluid collections - Part 1
SO DIGESTIVE AND LIVER DISEASE
LA English
DT Article
DE Pancreatic fluid collections; Walled-off necrosis; Pseudocysts; Lumen apposing metal stent; Endoscopy; EUS; Consensus
ID APPOSING METAL STENTS; GUIDED TRANSMURAL DRAINAGE; WALLED-OFF NECROSIS; REVISED ATLANTA CLASSIFICATION; DUAL-MODALITY DRAINAGE; STEP-UP APPROACH; ENDOSCOPIC NECROSECTOMY; NECROTIZING PANCREATITIS; GASTROINTESTINAL ENDOSCOPY; RETROSPECTIVE-COHORT
AB Pancreatic fluid collections (PFCs), including pancreatic pseudocysts (PPs) and walled-off pancreatic necrosis (WON), are common complications of pancreatitis and pancreatic surgery. Historically, the treatment of these conditions has relied on surgical and radiological approaches; however, it has later shifted toward an endoscopy-based approach. With the development of dedicated lumen-apposing metal stents (LAMS), interventional Endoscopic Ultrasound (EUS)-guided procedures have become the standard approach for PFC drainage. However, there is still limited consensus on several aspects of the multidisciplinary management of PFCs. The interventional endoscopy and ultrasound (i-EUS) group is an Italian network of clinicians and scientists with special interest in biliopancreatic interventional endoscopy, especially interventional EUS. This manuscript describes the first part of the results of a consensus conference organized by i-EUS with the aim of providing evidence-based guidance on aspects such as indications for treating PFCs, the timing of intervention, and different technical strategies for managing patients with PFCs. (c) 2024 Editrice Gastroenterologica Italiana S.r.l. Published by Elsevier Ltd. All rights are reserved, including those for text and data mining, AI training, and similar technologies.
C1 [Capurso, Gabriele] Ist Sci San Raffaele, Pancreat Biliary Endoscopy & Endosonog Div, Pancreas Translat & Clin Res Ctr, Milan, Italy.
[Rizzo, Giacomo Emanuele Maria; Tarantino, Ilaria] IRCCS ISMETT, Dept Diagnost & Therapeut Serv, Endoscopy Serv, Palermo, Italy.
[Rizzo, Giacomo Emanuele Maria] Univ Palermo, Dept Precis Med Med Surg & Cit Care Area Me Pre CC, Palermo,

Italy.
[Coluccio, Chiara; Binda, Cecilia; Sbrancia, Monica; Fabbri, Carlo] AUSL Romagna, Forli Cesena Hosp, Gastroenterol & Digest Endoscopy Unit, Forli Cesena, Italy.
[Crino, Stefano Francesco] GB Rossi Univ Hosp, Pancreas Inst, Diagnost & Intervent Endoscopy Pancreas, I-37134 Verona, Italy.
[Cucchetti, Alessandro] Univ Bologna, Dept Med & Surg Sci, Alma Mater Studiorum, DIMEC, Bologna, Italy.
[Facciorusso, Antonio] Univ Foggia, Dept Med Sci, Gastroenterol Unit, Foggia, Italy.
[Hassan, Cesare; Spadaccini, Marco] Humanitas Univ, Dept Biomed Sci, Pieve Emanuele, Italy.
[Hassan, Cesare] IRCCS, Human Clin & Res Ctr, Endoscopy Unit, Rozzano, Italy.
[Amato, Arnaldo; Fugazza, Alessandro] ASST Lecco, Digest Endoscopy & Gastroenterol Dept, Lecce, Italy.
[Auriemma, Francesco] Humanitas Mater Domini, Gastrointestinal Endoscopy Unit, Castellanza, Italy.
[Bertani, Helga] Azienda Osped Univ Policlin Modena, Gastroenterol Endoscopia Digest, Modena, Italy.
[Cipolletta, Fabio] Osped Mare, ASL NA1 Ctr, Dept Gastroenterol, Naples, Italy.
[Forti, Edoardo] ASST Niguarda Hosp, Digest & Intervent Endoscopy Unit, Milan, Italy.
[Lisotti, Andrea] Univ Bologna, Hosp Imola, Gastroenterol Unit, Imola, Italy.
[Maida, Marcello] Univ Enna Kore, Umberto 1 Hosp, Dept Med & Surg, Gastroenterol Unit, Enna, Italy.
[Sinagra, Emanuele] Fdn Ist G Giglio, Gastroenterol & Endoscopy Unit, Cefalu, Italy.
[Tacelli, Matteo; Vanella, Giuseppe] San Raffaele Sci Inst IRCCS, Pancreato biliary Endoscopy & EUS Div, Milan, Italy.
[Anderloni, Andrea] IRCCS Fdn Policlin San Matteo, Gastroenterol & Digest Endoscopy Unit, Pavia, Italy.
C3 Vita-Salute San Raffaele University; IRCCS Ospedale San Raffaele; IRCCS Istituto Mediterraneo per i Trapianti e Terapie ad Alta Specializzazione (ISMETT); University of Palermo; AUSL della Romagna; University of Verona; Azienda Ospedaliera Universitaria Integrata Verona; University of Bologna; University of Foggia; Humanitas University; Universita di Modena e Reggio Emilia; Universita di Modena e Reggio Emilia Hospital; Azienda USL di Imola; University of Bologna; Universita Kore di ENNA; Vita-Salute San Raffaele University; IRCCS Ospedale San Raffaele; IRCCS Fondazione San Matteo
RP Coluccio, C (corresponding author), AUSL Romagna, Forli Cesena Hosp, Gastroenterol & Digest Endoscopy Unit, Forli Cesena, Italy.
EM colucciochiara@gmail.com
RI Maida, Marcello/I-4118-2019; Vanella, Giuseppe/LQI-8930-2024; Spadaccini, Marco/HOH-7613-2023; Tarantino, Ilaria/AFS-0726-2022; Binda, Cecilia/ABH-1588-2020; Facciorusso, Antonio/AAA-5966-2019; Lisotti, Andrea/L-6762-2019; Rizzo, Giacomo Emanuele Maria/IAR-5913-2023; hassan, cesare/H-2844-2012; Sinagra, Emanuele/C-9336-2014; Cucchetti, Alessandro/B-5194-2014; Capurso, Gabriele/AAB-1389-2019; Amato, Arnaldo/ABC-5688-2020; Crinó, Stefano Francesco/Q-3421-2019; Fugazza, Alessandro/ABG-9381-2021; Tacelli, Matteo/AAK-8808-2020; Bertani, Helga/AAF-9839-2019
OI Coluccio, Chiara/0000-0002-8791-7764; Rizzo, Giacomo Emanuele

Maria/0000-0001-9335-6740; hassan, cesare/0000-0001-7167-1459;
Cucchetti, Alessandro/0000-0001-5269-1964; Capurso,
Gabriele/0000-0002-0019-8753; Amato, Arnaldo/0000-0002-4397-4142; Crinó,
Stefano Francesco/0000-0003-4560-8741; Fugazza,
Alessandro/0000-0003-0485-4903; Tacelli, Matteo/0000-0003-2642-6694;
Bertani, Helga/0000-0003-0818-7167
FX Acknowledgements Gabriele Calarese for the support in the organization
of the Consensus Conference.
CR Abdallah M, 2022, GASTROINTEST ENDOSC, V95, P1150, DOI 10.1016/j.gie.2021.11.030
AbiMansour JP, 2024, GASTROINTEST ENDOSC, V99, P104, DOI 10.1016/j.gie.2023.09.005
Adler DG, 2019, ENDOSC ULTRASOUND, V8, P36, DOI 10.4103/eus.eus_30_17
Amato A, 2022, GUT, V71, P1050, DOI 10.1136/gutjnl-2022-326880
Anderloni A, 2021, SURG ENDOSC, V35, P1741, DOI 10.1007/s00464-020-07567-8
Arvanitakis M, 2004, GASTROENTEROLOGY, V126, P715, DOI 10.1053/j.gastro.2003.12.006
Arvanitakis M, 2018, ENDOSCOPY, V50, P524, DOI 10.1055/a-0588-5365
Atkins D, 2004, BMC HEALTH SERV RES, V4, DOI 10.1186/1472-6963-4-38
Auriemma F, 2022, MINERVA GASTROENTERO, V68, P177, DOI 10.23736/S2724-5985.21.02862-X
Auriemma Francesco, 2018, Am J Gastroenterol, V113, P1582, DOI [10.1038/s41395-018-0266-6, 10.1038/s41395-018-0266-6]
BALTHAZAR EJ, 1990, RADIOLOGY, V174, P331, DOI 10.1148/radiology.174.2.2296641
Bang JY, 2014, BRIT J SURG, V101, P1729, DOI 10.1002/bjs.9664
Bang JY, 2024, LANCET GASTROENTEROL, V9, P22, DOI 10.1016/S2468-1253(23)00331-X
Bang JY, 2024, ENDOSCOPY, V56, P184, DOI 10.1055/a-2169-0362
Bang JY, 2022, DIGEST ENDOSC, V34, P612, DOI 10.1111/den.14099
Bang JY, 2019, GASTROENTEROLOGY, V156, P1027, DOI 10.1053/j.gastro.2018.11.031
Bang JY, 2019, GUT, V68, P1200, DOI 10.1136/gutjnl-2017-315335
Bang JY, 2018, ANN SURG, V267, P561, DOI 10.1097/SLA.0000000000002082
Bang JY, 2017, GUT, V66, P2054, DOI 10.1136/gutjnl-2016-312812
Banks PA, 2013, GUT, V62, P102, DOI 10.1136/gutjnl-2012-302779
Baron TH, 2020, GASTROENTEROLOGY, V158, P67, DOI 10.1053/j.gastro.2019.07.064
Bennett Shelby, 2012, Semin Intervent Radiol, V29, P314, DOI 10.1055/s-0032-1330066
Beran A, 2023, GASTROENTEROL RES, V16, P59, DOI 10.14740/gr1601
Bhatia H, 2023, ABDOM RADIOL, V48, P2415, DOI 10.1007/s00261-023-03883-4
Binmoeller KF, 2011, ENDOSCOPY, V43, P337, DOI 10.1055/s-0030-1256127
Bomman S, 2023, SURG ENDOSC, V37, P902, DOI 10.1007/s00464-022-09561-8
Bonnot B, 2014, AM J GASTROENTEROL, V109, P607, DOI 10.1038/ajg.2013.473
Borens B, 2017, EUR RADIOL, V27, P1064, DOI 10.1007/s00330-016-4462-8
Boxhoorn L, 2021, NEW ENGL J MED, V385, P1372, DOI 10.1056/NEJMoa2100826
Boxhoorn L, 2023, GUT, V72, P66, DOI 10.1136/gutjnl-2021-325632
Caillol F, 2019, ENDOSC ULTRASOUND, V8, P91, DOI 10.4103/eus.eus_112_17
Chandrasekhara V, 2021, CLIN GASTROENTEROL H, V19, P2192, DOI 10.1016/j.cgh.2021.05.005
Chantarojanasiri T, 2018, ENDOSC INT OPEN, V6, pE1398, DOI 10.1055/a-0751-2698

Chavan R, 2022, ENDOSCOPY, V54, P861, DOI 10.1055/a-1747-3283
Cho J, 2020, CLIN TRANSL GASTROEN, V11, DOI 10.14309/ctg.0000000000000251
Cornberg M, 2019, J HEPATOL, V70, P5, DOI 10.1016/j.jhep.2018.10.011
Cosgrove N, 2022, J CLIN GASTROENTEROL, V56, P457, DOI 10.1097/MCG.0000000000001549
Denzer UW, 2016, Z GASTROENTEROL, V54, P1047, DOI 10.1055/s-0042-112032
Devière J, 2008, GASTROINTEST ENDOSC, V67, P680, DOI 10.1016/j.gie.2007.12.056
Dhillon Amit, 2021, J Can Assoc Gastroenterol, V4, P158, DOI [10.1093/jcag/gwaa023, 10.1093/jcag/gwaa023]
Dhir V, 2018, ENDOSCOPY, V50, P22, DOI [10.1055/s-0043-118592, 10.1055/s-0043-123575]
Donepudi S, 2013, WORLD J GASTRO ENDOS, V5, P359, DOI 10.4253/wjge.v5.i8.359
Facciorusso A, 2022, DIGEST ENDOSC, V34, P1459, DOI 10.1111/den.14354
Falk Vanessa, 2020, J Can Assoc Gastroenterol, V3, P26, DOI [10.1093/jcag/gwy049, 10.1093/jcag/gwy049]
Fugazza A, 2020, AM J GASTROENTEROL, V115, P811, DOI 10.14309/ajg.0000000000000685
Fugazza A, 2020, GASTROINTEST ENDOSC, V91, P574, DOI 10.1016/j.gie.2019.11.021
Ghazanfar H, 2023, CASE REP GASTROENTER, V17, P294, DOI 10.1159/000533617
Giri S, 2023, THER ADV GASTROINTES, V16, DOI 10.1177/26317745231199364
Gluck M, 2012, J GASTROINTEST SURG, V16, P248, DOI 10.1007/s11605-011-1759-4
Gornals JB, 2024, SURG ENDOSC, V38, P2142, DOI 10.1007/s00464-024-10699-w
Gulati R, 2021, ENDOSCOPY, V53, P72, DOI 10.1055/a-1192-3219
Guo JT, 2020, SURG ENDOSC, V34, P1177, DOI 10.1007/s00464-019-06870-3
Guo JT, 2017, ENDOSC ULTRASOUND, V6, P285, DOI 10.4103/eus.eus_85_17
Hollemans RA, 2019, GASTROENTEROLOGY, V156, P1016, DOI 10.1053/j.gastro.2018.10.045
Holmes I, 2022, GASTROINTEST ENDOSC, V95, P482, DOI 10.1016/j.gie.2021.10.015
Honta S, 2021, DIGEST ENDOSC, V33, pe8, DOI 10.1111/den.13861
Huggett MT, 2015, ENDOSCOPY, V47, P929, DOI 10.1055/s-0034-1392413
Iacono C, 2013, BRIT J SURG, V100, P873, DOI 10.1002/bjs.9136
Iannuzzi JP, 2022, GASTROENTEROLOGY, V162, P122, DOI 10.1053/j.gastro.2021.09.043
Islim F, 2014, ABDOM IMAGING, V39, P472, DOI 10.1007/s00261-014-0076-2
Jagielski M, 2022, SCI REP-UK, V12, DOI 10.1038/s41598-021-03924-2
Jürgensen C, 2019, GASTROINTEST ENDOSC, V89, P311, DOI 10.1016/j.gie.2018.08.046
Kamal A, 2015, ABDOM IMAGING, V40, P1608, DOI 10.1007/s00261-014-0303-x
Kapoor H, 2020, ABDOM RADIOL, V45, P1534, DOI 10.1007/s00261-019-02093-1
Karstensen JG, 2023, GUT, V72, P1167, DOI 10.1136/gutjnl-2022-328225
Khan S, 2021, J GASTROEN HEPATOL, V36, P3395, DOI 10.1111/jgh.15658
Kwon YM, 2013, SURG ENDOSC, V27, P2422, DOI 10.1007/s00464-012-2752-z
Ladd AM, 2020, WORLD J GASTRO ENDOS, V12, P149, DOI 10.4253/wjge.v12.i5.149
Lecesne R, 1999, RADIOLOGY, V211, P727, DOI 10.1148/radiology.211.3.r99jn08727
Maatman Thomas K, 2020, Can J Surg, V63, pE272, DOI 10.1503/cjs.009519
Memis A, 2002, EUR J RADIOL, V43, P219, DOI 10.1016/S0720-048X(02)00157-2
Mohan B, 2019, ENDOSC ULTRASOUND, V8, P298, DOI 10.4103/eus.eus_18_19
Morgan DE, 1997, RADIOLOGY, V203, P773, DOI 10.1148/radiology.203.3.9169703
Mouli VP, 2013, GASTROENTEROLOGY, V144, P333, DOI 10.1053/j.gastro.2012.10.004
Mudireddy PR, 2018, GASTROINTEST ENDOSC, V87, P1256, DOI 10.1016/j.gie.2017.08.011

Mukai S, 2015, ENDOSC ULTRASOUND, V4, P120, DOI 10.4103/2303-9027.156738
Mukai T, 2024, SURG ENDOSC, V38, P999, DOI 10.1007/s00464-023-10568-y
Nayar M, 2022, GUT, V71, P850, DOI 10.1136/gutjnl-2021-325812
Ning N, 2024, BMC GASTROENTEROL, V24, DOI 10.1186/s12876-024-03145-7
Oblizajek N, 2020, GASTROINTEST ENDOSC, V91, P1303, DOI 10.1016/j.gie.2020.01.017
Oh D, 2022, GASTROINTEST ENDOSC, V95, P735, DOI 10.1016/j.gie.2021.12.015
Okuno Mitsuru, 2019, VideoGIE, V4, P220, DOI [10.1016/j.vgie.2019.02.007, 10.1016/j.vgie.2019.02.007]
Papachristou GI, 2007, ANN SURG, V245, P943, DOI 10.1097/01.sla.0000254366.19366.69
Parsa N, 2020, ENDOSCOPY, V52, P211, DOI 10.1055/a-1096-3299
Patra PS, 2014, BRIT J SURG, V101, P1721, DOI 10.1002/bjs.9666
Pedrazzoli S, 2009, ANN SURG, V249, P97, DOI 10.1097/SLA.0b013e31819274fe
Pokras S, 2021, PANCREAS, V50, P330, DOI 10.1097/MPA.0000000000001757
Poropat G, 2015, COCHRANE DB SYST REV, DOI 10.1002/14651858.CD010605.pub2
Puga M, 2018, ENDOSCOPY, V50, P1022, DOI 10.1055/a-0582-9127
Puli SR, 2014, CAN J GASTROENTEROL, V28, P50, DOI 10.1155/2014/539783
Rahnemai-Azar AA, 2021, AM J ROENTGENOL, V217, P921, DOI 10.2214/AJR.20.25168
Ramai D, 2023, GASTROINTEST ENDOSC, V97, P415, DOI 10.1016/j.gie.2022.11.003
Rana SS, 2019, ANN GASTROENTEROL, V32, P441, DOI 10.20524/aog.2019.0404
Rana SS, 2017, PANCREATOLOGY, V17, P658, DOI 10.1016/j.pan.2017.08.002
Rana SS, 2020, ENDOSC ULTRASOUND, V9, P402, DOI 10.4103/eus.eus_65_20
Rana SS, 2014, ENDOSC ULTRASOUND, V3, P118, DOI 10.4103/2303-9027.131039
Rizzo GEM, 2023, ENDOSCOPY, V55, pE767, DOI 10.1055/a-2085-0109
Ross A, 2010, GASTROINTEST ENDOSC, V71, P79, DOI 10.1016/j.gie.2009.06.037
Ross AS, 2014, GASTROINTEST ENDOSC, V79, P929, DOI 10.1016/j.gie.2013.10.014
Seewald S, 2009, GASTROINTEST ENDOSC, V69, pS13, DOI 10.1016/j.gie.2008.10.061
Seifert H, 2009, GUT, V58, P1260, DOI 10.1136/gut.2008.163733
Sekikawa Z, 2019, J VASC INTERV RADIOL, V30, P124, DOI 10.1016/j.jvir.2018.05.025
Sharaiha RZ, 2016, CLIN GASTROENTEROL H, V14, P1797, DOI 10.1016/j.cgh.2016.05.011
Siddiqui A, 2021, ANN GASTROENTEROL, V34, P273, DOI 10.20524/aog.2021.0570
Siddiqui AA, 2016, GASTROINTEST ENDOSC, V83, P699, DOI 10.1016/j.gie.2015.10.020
Stassen PMC, 2022, GASTROINTEST ENDOSC, V95, P471, DOI 10.1016/j.gie.2021.09.025
Storm AC, 2020, GASTROINTEST ENDOSC, V91, P1085, DOI 10.1016/j.gie.2019.11.045
Tamura T, 2019, THER ADV GASTROENTER, V12, DOI 10.1177/1756284819884418
Teoh AYB, 2020, DIGEST ENDOSC, V32, P391, DOI 10.1111/den.13494
Thoeni RF, 2012, RADIOLOGY, V262, P751, DOI 10.1148/radiol.11110947
Tilara A, 2014, J AM COLL SURGEONS, V218, P33, DOI 10.1016/j.jamcollsurg.2013.09.001
Trikudanathan G, 2018, AM J GASTROENTEROL, V113, P1550, DOI 10.1038/s41395-018-0232-3
van Baal MC, 2011, BRIT J SURG, V98, P18, DOI 10.1002/bjs.7304
van Baal MC, 2014, SURGERY, V155, P442, DOI 10.1016/j.surg.2013.10.001
van Brunschot S, 2018, GUT, V67, P697, DOI 10.1136/gutjnl-2016-313341
van Brunschot S, 2018, LANCET, V391, P51, DOI 10.1016/S0140-6736(17)32404-2
van Brunschot S, 2014, SURG ENDOSC, V28, P1425, DOI 10.1007/s00464-013-3382-9

van der Merwe SW, 2022, ENDOSCOPY, V54, P185, DOI 10.1055/a-1717-1391
van Santvoort HC, 2011, GASTROENTEROLOGY, V141, P1254, DOI 10.1053/j.gastro.2011.06.073
Vanek P, 2023, GASTROINTEST ENDOSC, V97, P1070, DOI 10.1016/j.gie.2022.12.026
Varadarajulu S, 2011, J GASTROINTEST SURG, V15, P2080, DOI 10.1007/s11605-011-1621-8
Varadarajulu S, 2009, GASTROINTEST ENDOSC, V70, P1260, DOI 10.1016/j.gie.2009.06.007
Viremouneix L, 2007, J MAGN RESON IMAGING, V26, P331, DOI 10.1002/jmri.21037
Voermans RP, 2015, J HEPATO-BIL-PAN SCI, V22, P20, DOI 10.1002/jhbp.180
Walter D, 2015, ENDOSCOPY, V47, P63, DOI 10.1055/s-0034-1378113
Watanabe Y, 2017, WORLD J GASTROENTERO, V23, P7110, DOI 10.3748/wjg.v23.i39.7110
Willems P, 2024, ENDOSC INT OPEN, V12, pE317, DOI 10.1055/a-2226-0840
Wundsam HV, 2019, J LAPAROENDOSC ADV S, V29, P891, DOI 10.1089/lap.2018.0768
Yan L, 2019, ENDOSC ULTRASOUND, V8, P172, DOI 10.4103/eus.eus_108_17
Yang JLN, 2019, ENDOSCOPY, V51, P715, DOI 10.1055/a-0924-5591
Yasuda I, 2013, ENDOSCOPY, V45, P627, DOI 10.1055/s-0033-1344027
NR 131
TC 15
Z9 17
U1 1
U2 2
PU ELSEVIER SCIENCE INC
PI NEW YORK
PA STE 800, 230 PARK AVE, NEW YORK, NY 10169 USA
SN 1590-8658
EI 1878-3562
J9 DIGEST LIVER DIS
JI Dig. Liver Dis.
PD OCT
PY 2024
VL 56
IS 10
BP 1663
EP 1674
DI 10.1016/j.dld.2024.06.030
EA SEP 2024
PG 12
WC Gastroenterology & Hepatology
WE Science Citation Index Expanded (SCI-EXPANDED)
SC Gastroenterology & Hepatology
GA H7P3G
UT WOS:001325321300001
PM 39048418
OA hybrid

DA 2026-05-09
ER

PT J
AU Yoo, HW
Moon, JH
Jo, SJ
Lee, YN
Park, JK
Lee, TH
Cha, SW
Cho, YD
Park, SH
Park, SI
Jeong, S
Lee, DH
AF Yoo, Hae Won
Moon, Jong Ho
Jo, Seok Jung
Lee, Yun Nah
Park, Jae Keun
Lee, Tae Hoon
Cha, Sang-Woo
Cho, Young Deok
Park, Sang-Heum
Park, Se Ik
Jeong, Seok
Lee, Don Haeng
TI A novel electrocautery-enhanced delivery system for one-step endoscopic ultrasound-guided drainage of the gallbladder and bile duct using a lumen-apposing metal stent: a feasibility study
SO ENDOSCOPY
LA English
DT Article
AB Background The use of a lumen-apposing metal stent (LAMS) capable of one-step endoscopic ultrasound-guided transmural drainage (EUS-TD) can increase the effectiveness of the procedure. We evaluated the newly developed electrocautery-enhanced (EC) delivery system with a LAMS for one-step EUS-guided gallbladder drainage (EUS-GBD) or choledochoduodenostomy (EUS-CDS).

Methods In the animal experiment, an EC-LAMS was advanced into the gallbladder without prior tract dilation in four pigs. A conventional LAMS was inserted in another four pigs as a control group. After the animal experiment, 17 patients underwent EUS-TD using the EC-LAMS (EUS-GBD in 10 patients, EUS-CDS in 7). The primary outcome was the technical success rate.

Results In the animal study, the mean procedure time was significantly shorter in the EC-LAMS group than in the conventional LAMS group. In the human study, the overall technical success rate was 94.1%, with one EUS-GBD failure. The clinical success rate was 100%. The overall adverse event rate was 17.6%.

Conclusions One-step EUS-GBD or EUS-CDS using the novel EC-LAMS is a feasible approach that achieves a high success rate and maintains safety.
C1 [Yoo, Hae Won; Moon, Jong Ho; Jo, Seok Jung; Lee, Yun Nah; Park, Jae Keun] SoonChunHyang Univ, Digest Dis Ctr, Sch Med, 170 Jomaru Ro, Bucheon 14584, South Korea.

[Yoo, Hae Won; Moon, Jong Ho; Jo, Seok Jung; Lee, Yun Nah; Park, Jae Keun] SoonChunHyang Univ, Res Inst, Sch Med, Dept Internal Med, Bucheon, South Korea.

[Lee, Tae Hoon; Park, Sang-Heum] SoonChunHyang Univ, Digest Dis Ctr, Sch Med, Cheonan, South Korea.

[Lee, Tae Hoon; Park, Sang-Heum] SoonChunHyang Univ, Res Inst, Sch Med, Dept Internal Med, Cheonan, South Korea.

[Cha, Sang-Woo; Cho, Young Deok] SoonChunHyang Univ, Digest Dis Ctr, Sch Med, Seoul, South Korea.

[Cha, Sang-Woo; Cho, Young Deok] SoonChunHyang Univ, Res Inst, Sch Med, Dept Internal Med, Seoul, South Korea.

[Park, Se Ik] Taewoong Med, Dept Res & Dev, Ilsan, South Korea.

[Jeong, Seok; Lee, Don Haeng] Inha Univ, Digest Dis Ctr, Coll Med, Incheon, South Korea.

[Jeong, Seok; Lee, Don Haeng] Inha Univ, Res Inst, Coll Med, Dept Internal Med, Incheon, South Korea.

C3 Soonchunhyang University; Soonchunhyang University; Soonchunhyang University; Soonchunhyang University; Soonchunhyang University; Soonchunhyang University; Inha University; Inha University

RP Moon, JH (corresponding author), SoonChunHyang Univ, Digest Dis Ctr, Sch Med, 170 Jomaru Ro, Bucheon 14584, South Korea.; Moon, JH (corresponding author), SoonChunHyang Univ, Bucheon Hosp, Res Inst, 170 Jomaru Ro, Bucheon 14584, South Korea.

EM jhmoon@schmc.ac.kr

RI Lee, Yun/J-1260-2014; Lee, Tae-Hoon/AAR-3708-2021; Jeong, Seok/C-9987-2011

OI Yoo, Hae Won/0000-0001-5086-5385
FU National Center of Efficacy Evaluation for the Development of Health Products Targeting Digestive Disorders of South Korea; SoonChunHyang University Research Fund

FX We thank Kwang Seok Kim, Young Il Hong, and Byeong Yong Shin of Taewoong Medical Co. for their efforts in developing the Niti-S HOT SPAXUS. Prof. Jong Ho Moon is the inventor of the Niti-S HOT Spaxus. We thank the National Center of Efficacy Evaluation for the Development of Health Products Targeting Digestive Disorders of South Korea for the support for the animal study. We thank Do Hyeong Kim, VMD for his cooperation with the animal study. We thank Olympus Korea Co. Ltd. for providing the echoendoscope and unit. We also thank Sun Hwa Cho, RN, and the rest of the nursing staff for their support and assistance during this procedure. This work was also supported, in part, by the SoonChunHyang University Research Fund.
CR Anderloni A, 2018, ANN GASTROENTEROL, V31, P735, DOI 10.20524/aog.2018.0299

Anderloni A, 2019, GASTROINTEST ENDOSC, V89, P69, DOI [10.1016/j.gie.2018.04.1746, 10.1016/j.gie.2018.08.047]
Cotton PB, 2010, GASTROINTEST ENDOSC, V71, P446, DOI 10.1016/j.gie.2009.10.027
Dollhopf M, 2017, GASTROINTEST ENDOSC, V86, P636, DOI 10.1016/j.gie.2017.02.027
Doyle D J., 2020, American Society of Anesthesiologists Classification
El Chafic AH, 2019, DIGEST DIS SCI, V64, P3321, DOI 10.1007/s10620-019-05688-2
Itonaga M, 2019, DIGEST ENDOSC, V31, P291, DOI 10.1111/den.13300
Jacques J, 2019, ENDOSCOPY, V51, P540, DOI 10.1055/a-0735-9137
Jang JW, 2011, GASTROINTEST ENDOSC, V74, P176, DOI 10.1016/j.gie.2011.03.1120
Mangas-Sanjuan C, 2020, ENDOSCOPY, V52, pE47, DOI 10.1055/a-0991-7763
Moon JH, 2014, GASTROINTEST ENDOSC, V79, P990, DOI 10.1016/j.gie.2014.02.015
Paik WH, 2018, AM J GASTROENTEROL, V113, P987, DOI 10.1038/s41395-018-0122-8
Song TJ, 2019, GASTROINTEST ENDOSC, V90, P507, DOI 10.1016/j.gie.2019.05.033
Walter D, 2016, GUT, V65, P6, DOI 10.1136/gutjnl-2015-309925
Yokoe M, 2018, J HEPATO-BIL-PAN SCI, V25, P41, DOI 10.1002/jhbp.515
NR 15
TC 21
Z9 25
U1 0
U2 3
PU GEORG THIEME VERLAG KG
PI STUTTGART
PA Oswald-Hesse-Strasse 50, D-70469 STUTTGART, GERMANY
SN 0013-726X
EI 1438-8812
J9 ENDOSCOPY
JI Endoscopy
PD SEP
PY 2021
VL 53
IS 9
BP 922
EP 926
DI 10.1055/a-1301-1526
EA DEC 2020
PG 5
WC Gastroenterology & Hepatology; Surgery
WE Science Citation Index Expanded (SCI-EXPANDED)
SC Gastroenterology & Hepatology; Surgery
GA YN3PU
UT WOS:000599528400009
PM 33336343

DA 2026-05-09
ER

PT J
AU Manta, R
Torrisi, S
Castellani, D
Germani, U
Zito, FP
Morelli, O
Brunori, PM
Zullo, A
De Francesco, V
AF Manta, Raffaele
Torrisi, Sabrina
Castellani, Danilo
Germani, Ugo
Zito, Francesco P.
Morelli, Olivia
Brunori, Paolo M.
Zullo, Angelo
De Francesco, Vincenzo
TI Endoscopic ultrasonography-guided biliary drainage for malignant
jaundice by using electrocautery-enhanced lumen-apposing metal stents
(Hot-SPAXUS stent)
SO ANNALS OF GASTROENTEROLOGY
LA English
DT Article
DE Biliary drainage; obstructive jaundice; electrocautery-enhanced
lumen-apposing metal stents; endoscopic ultrasonography
ID OBSTRUCTION; ERCP
AB Background Biliary drainage with endoscopic retrograde cholangiopancreatography (ERCP) for tumoral jaundice fails in a certain percentage of patients. In these patients, endoscopic ultrasonography-guided lumen-apposing metal stents (LAMS) with electrocautery-enhanced (ECE) technology allows a single-step, radiation-free palliative treatment.Methods We reviewed the data of patients who underwent choledochoduodenostomy with placement of ECE-LAMS (Hot-SPAXUS stent) after ERCP failure in a single tertiary center. Technical and clinical success rates were calculated and adverse events recorded.Results Data of 15 patients (8 male, median age 72 years) were collected. The procedure was technically successful in all patients, whilst clinical success was achieved in 14 (93.3%) patients. One (6.7%) patient presented delayed bleeding treated endoscopically. At follow up, stent occlusion with recurrence of jaundice occurred in 2 (13.3%) patients, due to food impaction (n=1), or neoplastic ingrowth (n=1).Conclusion Our results suggest that the Hot-SPAXUS procedure is effective and safe for palliative treatment in patients with distal biliary malignant obstruction after failure of ERCP.
C1 [Manta, Raffaele; Torrisi, Sabrina; Castellani, Danilo; Germani, Ugo; Morelli, Olivia; Brunori, Paolo M.] Santa Maria

Misericordia Hosp, Digest Endoscopy Unit, Perugia, Italy.
[Zito, Francesco P.] AORN Cardarelli, Gastroenterol & Digest Endoscopy Unit, Naples, Italy.
[Zullo, Angelo] Nuovo Regina Margher Hosp, Gastroenterol, Rome, Italy.
[De Francesco, Vincenzo] Riuniti Hosp, Gastroenterol Unit, Foggia, Italy.
[Zullo, Angelo] Osped Nuovo Regina Margher, Via E Morosini,30, I-00153 Rome, Italy.
C3 Hospital Santa Maria della Misericordia; Poliambulatorio Nuovo Regina
Margherita
RP Zullo, A (corresponding author), Osped Nuovo Regina Margher, Via E Morosini,30, I-00153 Rome, Italy.
EM angelozullo66@yahoo.it
RI Zullo, Angelo/AAV-7092-2020; Zito, Francesco Paolo/AAS-3197-2021
OI Zito, Francesco Paolo/0000-0002-1084-3373
CR Anderloni A, 2019, WORLD J GASTROENTERO, V25, P3857, DOI 10.3748/wjg.v25.i29.3857
Anderloni A, 2019, GASTROINTEST ENDOSC, V89, P69, DOI [10.1016/j.gie.2018.04.1746, 10.1016/j.gie.2018.08.047]
Binmoeller KF, 2011, ENDOSCOPY, V43, P337, DOI 10.1055/s-0030-1256127
Dhir V, 2015, GASTROINTEST ENDOSC, V81, P913, DOI 10.1016/j.gie.2014.09.054
Di Mitri R, 2022, SURG ENDOSC, V36, P4553, DOI 10.1007/s00464-021-08808-0
Dumonceau JM, 2018, ENDOSCOPY, V50, P910, DOI 10.1055/a-0659-9864
Giovannini M, 2001, ENDOSCOPY, V33, P898, DOI 10.1055/s-2001-17324
Hakim S, 2021, J CLIN GASTROENTEROL, V55, pE56, DOI 10.1097/MCG.0000000000001453
Kunda R, 2016, SURG ENDOSC, V30, P5002, DOI 10.1007/s00464-016-4845-6
Lee TH, 2016, CLIN GASTROENTEROL H, V14, P1011, DOI 10.1016/j.cgh.2015.12.032
Mangiavillano B, 2021, DIGEST LIVER DIS, V53, P1203, DOI 10.1016/j.dld.2020.08.015
Mangiavillano B, 2019, WORLD J GASTRO SURG, V11, P53, DOI 10.4240/wjgs.v11.i2.53
Peng CY, 2013, BMC GASTROENTEROL, V13, DOI 10.1186/1471-230X-13-147
Sarwar A, 2019, RADIOLOGY, V290, P722, DOI 10.1148/radiol.2018180279
Stark A, 2015, SEMIN ONCOL, V42, P163, DOI 10.1053/j.seminoncol.2014.12.014
Tsuchiya T, 2018, GASTROINTEST ENDOSC, V87, P1138, DOI 10.1016/j.gie.2017.08.017
Wang KX, 2016, GASTROINTEST ENDOSC, V83, P1218, DOI 10.1016/j.gie.2015.10.033
Williams EJ, 2012, ENDOSCOPY, V44, P674, DOI 10.1055/s-0032-1309345
Yoo HW, 2021, ENDOSCOPY, V53, P922, DOI 10.1055/a-1301-1526
NR 19
TC 4
Z9 4
U1 0
U2 1
PU HELLENIC SOC GASTROENTEROLOGY
PI ATHENS
PA DEMOKRATIAS AVE 67, ATHENS, 15451, GREECE
SN 1108-7471
EI 1792-7463
J9 ANN GASTROENTEROL

JI Ann. Gastroenterol.
PY 2022
VL 35
IS 6
BP 663
EP 667
DI 10.20524/aog.2022.0743
EA OCT 2022
PG 5
WC Gastroenterology & Hepatology
WE Emerging Sources Citation Index (ESCI)
SC Gastroenterology & Hepatology
GA 8J8XK
UT WOS:000869907200001
PM 36406962
OA Green Submitted, gold
DA 2026-05-09
ER

PT J
AU Carbajo, AY
Vegas, FJB
García-Alonso, FJ
Cimavilla, M
Yuste, RT
Gil-Simón, P
de la Serna-Higuera, C
Pérez, GCF
Pérez-Miranda, M
AF Yaiza Carbajo, Ana
Brunie Vegas, Francisco Javier
Javier Garcia-Alonso, Francisco
Cimavilla, Marta
Torres Yuste, Raul
Gil-Simon, Paula
de la Serna-Higuera, Carlos
Fernandez Perez, Gabriel Carlos
Perez-Miranda, Manuel
TI Retrospective cohort study comparing endoscopic ultrasound-guided and
percutaneous drainage of upper abdominal abscesses
SO DIGESTIVE ENDOSCOPY
LA English

DT Article
DE abdominal abscess; drainage; interventional EUS; liver abscess;
therapeutic endoscopic ultrasound
ID LIVER-ABSCESS; TRANSMURAL DRAINAGE; METALLIC STENT; MANAGEMENT;
GUIDELINES; ADULTS
AB Background and Aim Endoscopic ultrasonography (EUS)-guided drainage (EUS-D) has become the standard treatment for peripancreatic fluid collections. Its use in other intra-abdominal abscesses has been reported, although there is limited evidence. Methods We carried out a single-center retrospective cohort study comparing percutaneous drainage (PCD) and EUS-D of upper abdominal abscesses between January 2012 and June 2017. Pancreatic fluid collections and liver transplant recipients were excluded. Primary endpoints were technical and clinical success rates. Results We included 18 EUS-D (nine hepatic and nine intraperitoneal abscesses) and 62 PCD. There were no differences regarding age, gender and etiology. Size was larger in the PCD group (80 vs 65.5 mm, P = 0.04) and perivesicular location was more frequent in the PCD group (24.2% vs 11.1%, P = 0.003). In the EUS-D group, metal stents were deployed in 16 (88.9%) subjects (eight lumen-apposing metal stents and eight self-expandable metal stents), coaxial double-pigtail plastic stents in six (33.3%) and lavage/debridement was carried out in five (27.8%). There were no significant differences in technical success (EUS-D: 88.9%, PCD: 96.8%, P = 0.22) or clinical success (EUS-D: 88.9%, PCD: 82.3%, P = 0.50), with no relapses in the EUS-D group and 10 (16.1%) in the PCD group (P = 0.11). There were four (22.2%) adverse events in the EUS-D group, none of them severe, and 13 (21%) in the PCD group (P = 0.91). Conclusions EUS-D is an alternative to PCD in the treatment of upper abdominal abscesses, reaching similar success, relapse and adverse events rates.
C1 [Yaiza Carbajo, Ana; Javier Garcia-Alonso, Francisco; Cimavilla, Marta; Torres Yuste, Raul; Gil-Simon, Paula; de la Serna-Higuera, Carlos; Perez-Miranda, Manuel] Hosp Univ Rio Hortega, Dept Gastroenterol, Valladolid, Spain.
[Brunie Vegas, Francisco Javier; Fernandez Perez, Gabriel Carlos] Hosp Univ Rio Hortega, Dept Radiol, Valladolid, Spain.
C3 Hospital del Rio Hortega; Hospital del Rio Hortega
RP Carbajo, AY (corresponding author), Calle Dulzaina 2, Valladolid 47012, Spain.
EM anaycarbajo@gmail.com
RI García-Alonso, Francisco Javier/GOK-0879-2022
OI García-Alonso, Francisco Javier/0000-0002-1425-8534; Torres Yuste,
Raul/0000-0003-4240-4110
CR ADAMS DB, 1992, ANN SURG, V215, P571, DOI 10.1097/00000658-199206000-00003
Ang TL, 2009, ENDOSCOPY, V41, pE21, DOI 10.1055/s-0028-1103468
[Anonymous], 2009, NIH PUBLICATION
Besselink M, 2013, PANCREATOLOGY, V13, pE1, DOI 10.1016/j.pan.2013.07.063
BRUNBUISSON C, 1995, JAMA-J AM MED ASSOC, V274, P968, DOI 10.1001/jama.274.12.968
Cotton PB, 2010, GASTROINTEST ENDOSC, V71, P446, DOI 10.1016/j.gie.2009.10.027
Donatelli G, 2018, SURG ENDOSC, V32, P2281, DOI 10.1007/s00464-017-5922-1
Gupta T, 2012, GASTROINTEST ENDOSC, V76, P1259, DOI 10.1016/j.gie.2012.07.037
Itoi T, 2011, DIGEST ENDOSC, V23, P158, DOI 10.1111/j.1443-1661.2011.01115.x
Jaffe TA, 2016, ABDOM RADIOL, V41, P629, DOI 10.1007/s00261-016-0649-3
Kawakami H, 2014, ENDOSCOPY, V46, pE982, DOI 10.1055/s-0034-1391125
Keohane John, 2011, J Interv Gastroenterol, V1, P139
Medrado BF, 2013, ENDOSCOPY, V45, pE331, DOI 10.1055/s-0033-1344128

Mudireddy PR, 2018, GASTROINTEST ENDOSC, V87, P1256, DOI 10.1016/j.gie.2017.08.011
Noh SH, 2010, GASTROINTEST ENDOSC, V71, P1314, DOI 10.1016/j.gie.2009.12.045
Ogura T, 2016, DIGEST DIS SCI, V61, P303, DOI 10.1007/s10620-015-3841-3
Robbins D, 2013, GASTROINTEST ENDOSC, V78, P941, DOI 10.1016/j.gie.2013.07.034
Seewald S, 2005, GASTROINTEST ENDOSC, V61, P495, DOI 10.1016/S0016-5107(04)02848-2
Solomkin JS, 2010, CLIN INFECT DIS, V50, P133, DOI [10.1086/649554, 10.1089/sur.2009.9930]
Tilara A, 2014, J AM COLL SURGEONS, V218, P33, DOI 10.1016/j.jamcollsurg.2013.09.001
Tonozuka R, 2015, GASTROINTEST ENDOSC, V81, P1463, DOI 10.1016/j.gie.2015.01.023
Varadarajulu S, 2011, GASTROINTEST ENDOSC, V74, P418, DOI 10.1016/j.gie.2011.03.1242
von Elm E, 2008, J CLIN EPIDEMIOL, V61, P344, DOI 10.2471/BLT.07.045120
NR 23
TC 29
Z9 35
U1 0
U2 0
PU WILEY
PI HOBOKEN
PA 111 RIVER ST, HOBOKEN 07030-5774, NJ USA
SN 0915-5635
EI 1443-1661
J9 DIGEST ENDOSC
JI Dig. Endosc.
PD JUL
PY 2019
VL 31
IS 4
BP 431
EP 438
DI 10.1111/den.13342
PG 8
WC Gastroenterology & Hepatology; Surgery
WE Science Citation Index Expanded (SCI-EXPANDED)
SC Gastroenterology & Hepatology; Surgery
GA IH2CK
UT WOS:000474302600016
PM 30629764
DA 2026-05-09
ER

PT J
AU Yoo, HW
Moon, JH

Lee, YN
Song, YH
Yang, JK
Lee, TH
Cha, SW
Cho, YD
Park, SH
AF Yoo, Hae Won
Moon, Jong Ho
Lee, Yun Nah
Song, Yeon Han
Yang, Jae Kook
Lee, Tae Hoon
Cha, Sang-Woo
Cho, Young Deok
Park, Sang-Heum
TI Peroral cholecystoscopy using a multibending ultraslim endoscope through a lumen-apposing metal stent for endoscopic ultrasound-guided gallbladder drainage: a feasibility study
SO ENDOSCOPY
LA English
DT Article
AB Background Endoscopic ultrasound-guided gallbladder drainage (EUS-GBD) using a lumen-apposing metal stent (LAMS) has rendered endoscopic gallbladder assessment through a stent possible, but an appropriate endoscope is required. We assessed the feasibility of peroral cholecystoscopy (POCC) using a multibending ultraslim endoscope passed through a LAMS after EUS-GBD.

Methods 14 patients with a LAMS for EUS-GBD who consecutively underwent POCC were included. POCC was performed using a multibending endoscope inserted through the LAMS. The primary outcome was the technical success rate, defined as complete endoscopic examination of the gallbladder from the orifice of the cystic duct to the fundus. The types of intervention and adverse events were recorded.

Results 17 POCCs were performed in 14 patients. Of the 17 POCCs, 15 (88.2 %) were technically successful. Narrow-band imaging endoscopy was performed in 12 procedures (70.6 %), and cholecystoscopy-guided target biopsies were obtained in six. Gallstone extraction was performed in two patients. After POCC, all LAMSs remained stable and no adverse events were observed.

Conclusions POCC using a multibending ultraslim endoscope can be effectively and safely performed through a LAMS after EUS-GBD.
C1 [Yoo, Hae Won; Moon, Jong Ho; Lee, Yun Nah; Song, Yeon Han] SoonChunHyang Univ, Digest Dis Ctr & Res Inst, Dept Internal Med, Sch Med, Bucheon, South Korea.

[Yang, Jae Kook; Lee, Tae Hoon; Park, Sang-Heum] SoonChunHyang Univ, Digest Dis Ctr & Res Inst, Dept Internal Med, Sch Med, Cheonan, South Korea.

[Cha, Sang-Woo; Cho, Young Deok] SoonChunHyang Univ, Digest Dis Ctr & Res Inst, Dept Internal Med, Sch Med, Seoul, South Korea.

C3 Soonchunhyang University; Soonchunhyang University; Soonchunhyang University
RP Moon, JH (corresponding author), SoonChunHyang Univ, Bucheon Hosp, Sch Med, Digest Dis Ctr & Res Inst, 170 Jomaru Ro, Bucheon 14584, South Korea.
EM jhmoon@schmc.ac.kr
RI ; Lee, Tae-Hoon/AAR-3708-2021; Lee, Yun/J-1260-2014
OI Moon, Jong Ho/0000-0002-3946-9944; Yoo, Hae Won/0000-0001-5086-5385;
FU SoonChunHyang University Research Fund
FX We thank Olympus Medical systems (Tokyo, Japan) for providing the multibending ultraslim endoscope. Research support for this study was provided in part by the SoonChunHyang University Research Fund. We also thank A Ri Song, RN, Song Ah Jeong, RN, Sun Hwa Cho, RN, and the rest of the nursing staff for their support and assistance with the procedure.
CR Barkay O, 2009, GASTROINTEST ENDOSC, V70, P1039, DOI 10.1016/j.gie.2009.03.033
Chan JHY, 2018, ENDOSC ULTRASOUND, V7, P85, DOI 10.4103/eus.eus_7_18
Chan SM, 2017, GASTROINTEST ENDOSC, V85, P1225, DOI 10.1016/j.gie.2016.10.014
Cotton PB, 2010, GASTROINTEST ENDOSC, V71, P446, DOI 10.1016/j.gie.2009.10.027
FUJITA R, 1989, ENDOSCOPY, V21, P378, DOI 10.1055/s-2007-1012996
Ge N, 2016, BMC GASTROENTEROL, V16, DOI 10.1186/s12876-016-0420-9
Gutkin E, 2012, GUT LIVER, V6, P136, DOI 10.5009/gnl.2012.6.1.136
INUI K, 1989, ENDOSCOPY, V21, P361, DOI 10.1055/s-2007-1012991
Itoi T, 2014, GASTROINTEST ENDOSC, V79, P547, DOI 10.1016/j.gie.2013.12.010
Jang JW, 2011, GASTROINTEST ENDOSC, V74, P176, DOI 10.1016/j.gie.2011.03.1120
Lee YN, 2020, GASTROINTEST ENDOSC, V91, P92, DOI 10.1016/j.gie.2019.08.007
Takahara N, 2012, ENDOSCOPY, V44, pE145, DOI 10.1055/s-0031-1291495
Teoh AYB, 2014, ENDOSCOPY, V46, pE13, DOI 10.1055/s-0033-1359139
Walter D, 2016, GUT, V65, P6, DOI 10.1136/gutjnl-2015-309925
Yoo HW, 2021, ENDOSCOPY, V53, P922, DOI 10.1055/a-1301-1526
NR 15
TC 9
Z9 10
U1 0
U2 4
PU GEORG THIEME VERLAG KG
PI STUTTGART
PA Oswald-Hesse-Strasse 50, D-70469 STUTTGART, GERMANY
SN 0013-726X
EI 1438-8812
J9 ENDOSCOPY
JI Endoscopy
PD APR
PY 2022

VL 54
IS 04
BP 384
EP 388
DI 10.1055/a-1518-7039
EA JUL 2021
PG 5
WC Gastroenterology & Hepatology; Surgery
WE Science Citation Index Expanded (SCI-EXPANDED)
SC Gastroenterology & Hepatology; Surgery
GA 0M0IV
UT WOS:000670032500001
PM 34229357
DA 2026-05-09
ER

PT J
AU Dietrich, CF
Arcidiacono, PG
Bhutani, MS
Braden, B
Burmester, E
Fusaroli, P
Hocke, M
Ignee, A
Jenssen, C
Al-Lehibi, A
Aljahdli, E
Napoleon, B
Rimbas, M
Vanella, G
AF Dietrich, Christoph Frank
Arcidiacono, Paolo Giorgio
Bhutani, Manoop S.
Braden, Barbara
Burmester, Eike
Fusaroli, Pietro
Hocke, Michael
Ignee, Andre
Jenssen, Christian
Al-Lehibi, Abed
Aljahdli, Emad

Napoleon, Bertrand
Rimbas, Mihai
Vanella, Giuseppe
TI Controversies in Endoscopic Ultrasound-Guided Biliary Drainage
SO CANCERS
LA English
DT Review
DE acute cholecystitis; endoscopic retrograde cholangiopancreatography;
endoscopic ultrasound; endoscopic; ultrasound-guided biliary drainage;
endoscopic ultrasound-guided gallbladder drainage; obstructive jaundice
ID PANCREATIC-DUCT DRAINAGE; APPOSING METAL STENT; MALIGNANT BILIARY;
FAILED ERCP; BILE-DUCT; RETROGRADE CHOLANGIOPANCREATOGRAPHY;
PERCUTANEOUS DRAINAGE; EUROPEAN-SOCIETY; PLASTIC STENT; OBSTRUCTION
AB Simple Summary In this review, first, the history of EUS-guided biliary drainage (EUS-BD) is summarized. In the following chapters controversies arising from various approaches and challenges in EUS-BD, EUS-guided gallbladder drainage (EUS-GBD) and alternatively performed procedures are discussed from different points of view on the background of the available evidence. In addition, for all topics arguments in favor and against the techniques are described and reflected. The topics include "Why do we need procedures other than ERCP?"; "Should EUS-BD and ERCP be performed by the same operator?"; rendezvous techniques, including "Should rendezvous be used first?" and "Which rendezvous route should be used?"; percutaneous transhepatic cholangiography and biliary drainage (PTBD); "Should PTBD and EUS-BD be performed by the same physician?"; "Do we need cystotomes?"; "Do we need bougies?"; "Are all EUS needles the same for EUS-BD?"; "Plastic or metal stents?"; and adverse events.Abstract In this 14th document in a series of papers entitled "Controversies in Endoscopic Ultrasound" we discuss various aspects of EUS-guided biliary drainage that are debated in the literature and in practice. Endoscopic retrograde cholangiography is still the reference technique for therapeutic biliary access, but EUS-guided techniques for biliary access and drainage have developed into safe and highly effective alternative options. However, EUS-guided biliary drainage techniques are technically demanding procedures for which few training models are currently available. Different access routes require modifications to the basic technique and specific instruments. In experienced hands, percutaneous transhepatic cholangiodrainage is also a good alternative. Therefore, in this paper, we compare arguments for different options of biliary drainage and different technical modifications.
C1 [Dietrich, Christoph Frank] Kliniken DAIM Hirslanden Beau Site, Dept Allgemeine Innere Med, CH-3013 Bern, Switzerland.
[Arcidiacono, Paolo Giorgio; Vanella, Giuseppe] Univ Vita Salute San Raffaele, IRCCS San Raffaele Sci Inst, Div Pancreatobiliary Endoscopy & Endosonog, I-20132 Milan, Italy.
[Bhutani, Manoop S.] Univ Texas MD Anderson Canc Ctr, Dept Gastroenterol Hepatol & Nutr, Houston, TX 77030 USA.
[Braden, Barbara] Univ Munster, Dept Med B, Albert Schweitzer Campus 1, D-48149 Munster, Germany.
[Burmester, Eike] Sana Kliniken Luebeck, Med Klin 2, D-23560 Lubeck, Germany.
[Fusaroli, Pietro] Univ Bologna, Hosp Imola, Dept Med & Surg Sci, Gastrointestinal Unit, I-40126 Bologna, Italy.
[Hocke, Michael] Helios Klinikum Meiningen, Dept Med, D-98617 Meiningen, Germany.
[Ignee, Andre] Klinikum Wurzburg Mitte, Standort Juliusspital, D-97074 Wurzburg, Germany.
[Jenssen, Christian] Krankenhaus Maerk Oderland, Med Dept, D-15441 Strausberg, Germany.
[Jenssen, Christian] Univ Brandenburg, Brandenburg Inst Clin Ultrasound Med, D-16816 Neuruppin, Germany.

[Al-Lehibi, Abed] King Fahad Med City, Dept Gastroenterol & Hepatol, Riyadh 11525, Saudi Arabia.
[Aljahdli, Emad] King Abdulaziz Univ, King Abdul Aziz Univ Hosp KAUH, Fac Med, Gastrointestinal Oncol Unit, Jeddah 22252, Saudi Arabia.
[Napoleon, Bertrand] Hop Prive J Mermoz Ramsay Gen Sante, F-69008 Lyon, France.
[Rimbas, Mihai] Carol Davila Univ Med, Colentina Clin Hosp, Dept Gastroenterol, Clin Internal Med, Bucharest 050474, Romania.
C3 Vita-Salute San Raffaele University; IRCCS Ospedale San Raffaele;
University of Texas System; UTMD Anderson Cancer Center; University of
Munster; Azienda USL di Imola; University of Bologna; Helios Kliniken;
King Fahad Medical City; King Abdulaziz University; Carol Davila
University of Medicine & Pharmacy
RP Dietrich, CF (corresponding author), Kliniken DAIM Hirslanden Beau Site, Dept Allgemeine Innere Med, CH-3013 Bern, Switzerland.
EM c.f.dietrich@googlemail.com; arcidiacono.paologiorgio@hsr.it;
manoop.bhutani@mdanderson.org; barbara.braden@ukmuenster.de;
burmester.buc@t-online.de; michaelhocke1@aol.com;
andre.ignee@kwm-klinikum.de; c.jenssen@khmol.de; aallehibi@kfmc.med.sa;
ealjahdli@gmail.com; dr.napoleon@wanadoo.fr; mrimbas@gmail.com;
vanella.giuseppe@hsr.it
RI ; Aljahdli, Emad/ISU-1755-2023; Rimbas, Mihai/AAF-5521-2020; Dietrich,
Christoph Frank/AFQ-4722-2022; Braden, Barbara/AAH-8693-2021; Fusaroli,
Pietro/AAC-2182-2020; Arcidiacono, Paolo Giorgio/K-2122-2018; Vanella,
Giuseppe/LQI-8930-2024
OI Ignee, André/0000-0002-2388-2945; Aljahdli, Emad/0000-0003-2786-8224;
Dietrich, Christoph Frank/0000-0001-6015-6347; Braden,
Barbara/0000-0002-8534-6873; Fusaroli, Pietro/0000-0002-4397-9314;
AlLehibi, Abed/0000-0002-4375-0384; Arcidiacono, Paolo
Giorgio/0000-0001-6692-7720; Jenssen, Christian/0000-0002-7008-6650;
CR Alvarez-Sanchez MV, 2014, SURG ENDOSC, V28, P712, DOI 10.1007/s00464-013-3260-5
Artifon ELA, 2015, GASTROINTEST ENDOSC, V81, P950, DOI 10.1016/j.gie.2014.09.047
Artifon ELA, 2012, J CLIN GASTROENTEROL, V46, P768, DOI 10.1097/MCG.0b013e31825f264c
Ba YJ, 2020, ENDOSC INT OPEN, V8, pE203, DOI 10.1055/a-0990-9114
Bang JY, 2018, GASTROINTEST ENDOSC, V88, P9, DOI 10.1016/j.gie.2018.03.012
Bapaye A, 2013, UNITED EUR GASTROENT, V1, P285, DOI 10.1177/2050640613490928
Baron TH, 2015, ENDOSC ULTRASOUND, V4, P34, DOI 10.4103/2303-9027.151326
Bories E, 2007, ENDOSCOPY, V39, P287, DOI 10.1055/s-2007-966212
Brauer BC, 2009, GASTROINTEST ENDOSC, V70, P471, DOI 10.1016/j.gie.2008.12.233
Burmester E, 2003, GASTROINTEST ENDOSC, V57, P246, DOI 10.1067/mge.2003.85
Chapman CG, 2016, CLIN ENDOSC, V49, P161, DOI 10.5946/ce.2016.011
CREMER M, 1990, ENDOSCOPY, V22, P76, DOI 10.1055/s-2007-1012797
Dhir V, 2017, DIGEST ENDOSC, V29, P472, DOI 10.1111/den.12818
Dhir V, 2012, GASTROINTEST ENDOSC, V75, P354, DOI 10.1016/j.gie.2011.07.075

Dietrich CF, 2022, ENDOSC ULTRASOUND, V11, P186, DOI 10.4103/EUS-D-21-00106
Dietrich CF, 2022, ENDOSC ULTRASOUND, V11, P27, DOI 10.4103/EUS-D-21-00102
Dollhopf M, 2017, GASTROINTEST ENDOSC, V86, P636, DOI 10.1016/j.gie.2017.02.027
Domagk D, 2018, UNITED EUR GASTROENT, V6, P1448, DOI 10.1177/2050640618808157
El Chafic AH, 2019, DIGEST DIS SCI, V64, P3321, DOI 10.1007/s10620-019-05688-2
Enochsson L, 2010, GASTROINTEST ENDOSC, V72, P1175, DOI 10.1016/j.gie.2010.07.047
Fabbri C, 2019, J GASTROINTEST LIVER, V28, P125, DOI 10.15403/jgld.2014.1121.281.eus
Fabbri C, 2014, WORLD J GASTROENTERO, V20, P8424, DOI 10.3748/wjg.v20.i26.8424
Fuldner Frank, 2021, Z Gastroenterol, V59, P933, DOI [10.1055/a-1540-7975, 10.1055/a-1540-7975]
Fusaroli P, 2016, ULTRASCHALL MED, V37, P408, DOI 10.1055/s-0035-1553742
Gaujoux S, 2021, HPB, V23, P154, DOI 10.1016/j.hpb.2020.06.001
Giovannini M, 2003, ENDOSCOPY, V35, P1076, DOI 10.1055/s-2003-44596
Giovannini M, 2001, ENDOSCOPY, V33, P898, DOI 10.1055/s-2001-17324
Giri S, 2023, GASTROINTEST ENDOSC, V98, DOI 10.1016/j.gie.2023.06.055
Guo JT, 2018, ENDOSC ULTRASOUND, V7, P356, DOI 10.4103/eus.eus_53_18
Gupta K, 2014, J CLIN GASTROENTEROL, V48, P80, DOI 10.1097/MCG.0b013e31828c6822
Hara K, 2011, AM J GASTROENTEROL, V106, P1239, DOI 10.1038/ajg.2011.84
Hayat U, 2024, GASTROINTEST ENDOSC, V99, DOI 10.1016/j.gie.2023.10.043
Hedjoudje A, 2019, UNITED EUR GASTROENT, V7, P60, DOI 10.1177/2050640618808147
Hindryckx P, 2019, WORLD J GASTRO ENDOS, V11, P103, DOI 10.4253/wjge.v11.i2.103
Honjo M, 2018, ENDOSC ULTRASOUND, V7, P376, DOI 10.4103/eus.eus_2_18
Horaguchi J, 2009, DIGEST ENDOSC, V21, P239, DOI 10.1111/j.1443-1661.2009.00899.x
Irani S, 2017, CLIN GASTROENTEROL H, V15, P738, DOI 10.1016/j.cgh.2016.12.021
Isayama H, 2013, J HEPATO-BIL-PAN SCI, V20, P413, DOI 10.1007/s00534-012-0577-8
Itoi T, 2016, GASTROINTEST ENDOSC, V83, P401, DOI 10.1016/j.gie.2015.05.049
Itoi T, 2014, J HEPATO-BIL-PAN SCI, V21, pE86, DOI 10.1002/jhbp.165
Itoi T, 2014, J HEPATO-BIL-PAN SCI, V21, pE4, DOI 10.1002/jhbp.43
Itonaga M, 2019, DIGEST ENDOSC, V31, P291, DOI 10.1111/den.13300
Iwashita T, 2012, ENDOSCOPY, V44, P60, DOI 10.1055/s-0030-1256871
Iwashita T, 2016, GASTROINTEST ENDOSC, V83, P394, DOI 10.1016/j.gie.2015.04.043
Iwashita T, 2014, CLIN J GASTROENTEROL, V7, P94, DOI 10.1007/s12328-014-0467-5
Kahaleh M, 2006, GASTROINTEST ENDOSC, V64, P52, DOI 10.1016/j.gie.2006.01.063
Kahaleh M, 2013, WORLD J GASTROENTERO, V19, P1372, DOI 10.3748/wjg.v19.i9.1372
Khan MA, 2016, DIGEST DIS SCI, V61, P684, DOI 10.1007/s10620-015-3933-0
Khashab MA, 2016, ENDOSC INT OPEN, V4, pE1322, DOI 10.1055/s-0042-110790
Khashab MA, 2016, ENDOSC INT OPEN, V4, pE175, DOI 10.1055/s-0041-109083
Khashab MA, 2015, DIGEST DIS SCI, V60, P557, DOI 10.1007/s10620-014-3300-6
Khashab MA, 2013, GASTROINTEST ENDOSC, V78, P734, DOI 10.1016/j.gie.2013.05.013
Kim TH, 2012, WORLD J GASTROENTERO, V18, P2526, DOI 10.3748/wjg.v18.i20.2526
Kim YS, 2010, ENDOSCOPY, V42, P496, DOI 10.1055/s-0029-1244082
Kunda R, 2016, SURG ENDOSC, V30, P5002, DOI 10.1007/s00464-016-4845-6
Mallery S, 2004, GASTROINTEST ENDOSC, V59, P100, DOI 10.1016/S0016-5107(03)02300-9

Maranki J, 2009, ENDOSCOPY, V41, P532, DOI 10.1055/s-0029-1214712
Marx M, 2022, ENDOSC ULTRASOUND, V11, P495, DOI 10.4103/EUS-D-21-00108
MCCUNE WS, 1968, ANN SURG, V167, P752, DOI 10.1097/00000658-196805000-00013
Miller CS, 2019, ENDOSC INT OPEN, V7, pE1563, DOI 10.1055/a-0998-8129
Oh HC, 2007, ENDOSCOPY, V39, P731, DOI 10.1055/s-2007-966577
Okuno N, 2017, INTERNAL MED, V56, P3135, DOI 10.2169/internalmedicine.8677-16
Paik WH, 2018, AM J GASTROENTEROL, V113, P987, DOI 10.1038/s41395-018-0122-8
Park DH, 2013, GASTROINTEST ENDOSC, V78, P91, DOI 10.1016/j.gie.2013.01.042
Park DH, 2011, GASTROINTEST ENDOSC, V74, P1276, DOI 10.1016/j.gie.2011.07.054
Park JK, 2018, GASTROINTEST ENDOSC, V88, P277, DOI 10.1016/j.gie.2018.03.015
Pavlides M, 2014, WORLD J GASTROENTERO, V20, P13153, DOI 10.3748/wjg.v20.i36.13153
Prachayakul V, 2015, WORLD J GASTROENTERO, V21, P2725, DOI 10.3748/wjg.v21.i9.2725
Püspök A, 2005, AM J GASTROENTEROL, V100, P1743, DOI 10.1111/j.1572-0241.2005.41806.x
Ragab KM, 2023, ACTA GASTRO-ENT BELG, V86, P26, DOI 10.51821/86.1.10828
Robbins D, 2014, GASTROINTEST ENDOSC, V80, P342, DOI 10.1016/j.gie.2014.03.010
Ryou M, 2020, GASTROINTEST ENDOSC, V91, P178, DOI 10.1016/j.gie.2019.07.035
Saad WEA, 2010, J VASC INTERV RADIOL, V21, P789, DOI 10.1016/j.jvir.2010.01.012
Salerno R, 2019, WORLD J GASTRO ENDOS, V11, P354, DOI 10.4253/wjge.v11.i5.354
Schmidt A, 2015, ENDOSCOPY, V47, P430, DOI 10.1055/s-0034-1391232
Song TJ, 2010, GASTROINTEST ENDOSC, V71, P634, DOI 10.1016/j.gie.2009.11.024
Tarantino I, 2008, ENDOSCOPY, V40, P336, DOI 10.1055/s-2007-995455
Teoh AYB, 2020, GUT, V69, P1085, DOI 10.1136/gutjnl-2019-319996
Teoh AYB, 2023, GASTROENTEROLOGY, V165, P473, DOI 10.1053/j.gastro.2023.04.016
Teoh AYB, 2021, GASTROINTEST ENDOSC, V93, P577, DOI 10.1016/j.gie.2020.06.066
Tse F, 2013, ENDOSCOPY, V45, P605, DOI 10.1055/s-0032-1326640
Tyberg A, 2020, ENDOSC ULTRASOUND, V9, P392, DOI 10.4103/eus.eus_42_20
Tyberg A, 2016, GASTROINTEST ENDOSC, V84, P941, DOI 10.1016/j.gie.2016.05.035
Umeda J, 2015, GASTROINTEST ENDOSC, V82, P390, DOI 10.1016/j.gie.2015.02.041
van Wanrooij RLJ, 2022, ENDOSCOPY, V54, P1023, DOI 10.1055/a-1782-7568
van Wanrooij RLJ, 2022, ENDOSCOPY, V54, P310, DOI 10.1055/a-1738-6780
Vanella G, 2020, ENDOSC INT OPEN, V08, pE1782, DOI 10.1055/a-1264-7511
Varadarajulu S, 2009, GASTROINTEST ENDOSC, V69, pS176, DOI 10.1016/j.gie.2008.12.024
Veitch AM, 2021, ENDOSCOPY, V53, P947, DOI 10.1055/a-1547-2282
Venkatachalapathy SV, 2021, GASTROINTEST ENDOSC, V94, P321, DOI 10.1016/j.gie.2021.01.029
Vila JJ, 2012, GASTROINTEST ENDOSC, V76, P1133, DOI 10.1016/j.gie.2012.08.001
Wang KX, 2016, GASTROINTEST ENDOSC, V83, P1218, DOI 10.1016/j.gie.2015.10.033
Wang L, 2019, WORLD J SURG ONCOL, V17, DOI 10.1186/s12957-019-1656-y
Wiersema MJ, 1996, GASTROINTEST ENDOSC, V43, P102, DOI 10.1016/S0016-5107(06)80108-2
Will U, 2015, ULTRASCHALL MED, V36, P276, DOI 10.1055/s-0034-1366557
Winbladh A, 2009, HPB, V11, P183, DOI 10.1111/j.1477-2574.2009.00052.x
Winick A B, 2001, Tech Vasc Interv Radiol, V4, P200, DOI 10.1016/S1089-2516(01)90026-5
Yamao K, 2008, ENDOSCOPY, V40, P340, DOI 10.1055/s-2007-995485

NR 98
TC 8
Z9 8
U1 1
U2 3
PU MDPI
PI BASEL
PA ST ALBAN-ANLAGE 66, CH-4052 BASEL, SWITZERLAND
EI 2072-6694
J9 CANCERS
JI Cancers
PD MAY
PY 2024
VL 16
IS 9
AR 1616
DI 10.3390/cancers16091616
PG 25
WC Oncology
WE Science Citation Index Expanded (SCI-EXPANDED)
SC Oncology
GA QH8X6
UT WOS:001220089100001
PM 38730570
OA Green Submitted, gold
DA 2026-05-09
ER

PT J
AU Vanella, G
Dell'Anna, G
Bronswijk, M
van Wanrooij, RLJ
Rizzatti, G
Gkolfakis, P
Larghi, A
van der Merwe, S
Arcidiacono, PG
AF Vanella, Giuseppe
Dell'Anna, Giuseppe
Bronswijk, Michiel
van Wanrooij, Roy L. J.

Rizzatti, Gianenrico
Gkolfakis, Paraskevas
Larghi, Alberto
van der Merwe, Schalk
Arcidiacono, Paolo Giorgio
TI Endoscopic ultrasound-guided biliary drainage and gastrointestinal anastomoses: the journey from promising innovations to standard of care
SO ANNALS OF GASTROENTEROLOGY
LA English
DT Review
DE Endoscopic ultrasound; gastric outlet obstruction; hepaticogastrostomy; choledochoduodenostomy; gastroenterostomy
ID GASTRIC OUTLET OBSTRUCTION; APPOSING METAL STENT; LONG-TERM OUTCOMES; MALIGNANT BILIARY; GALLBLADDER DRAINAGE; INTERNATIONAL MULTICENTER; PERCUTANEOUS DRAINAGE; TRACT OBSTRUCTION; RESCUE TREATMENT; LEARNING-CURVE
AB Biliary obstruction (BO) and gastric outlet obstruction (GOO) are frequent complications of pancreatobiliary and gastroduodenal neoplasia, which can severely impact oncological outcomes, patient survival and quality of life. Although endoscopic retrograde cholangiopancreatography (ERCP) remains the gold standard for biliary drainage, this may fail or be unfeasible because of duodenal/papillary infiltration or surgically-altered anatomy. Percutaneous transhepatic biliary drainage (PTBD) has been the standard rescue therapy in this setting, but is burdened by high morbidity and reduced quality of life. As for GOO, surgical gastroenterostomy and enteral stenting are limited by invasiveness and suboptimal long-term outcomes, respectively. Endoscopic ultrasound (EUS) has evolved from a diagnostic to a therapeutic modality, providing a safe and effective alternative for draining the pancreatobiliary tract into the stomach or duodenum. EUS-guided biliary drainage (EUS-BD) has already demonstrated similar efficacy, greater safety and fewer reinterventions compared to PTBD, and can be performed in the same session after ERCP failure. Further development of lumen apposing metal stents has paved the way towards the creation of EUS-guided anastomoses. EUS- guided gastroenterostomy (EUS-GE) is nowadays increasingly used to treat GOO, combining the minimal invasiveness of endoscopy with surgical-range efficacy. This review summarizes the technical details, current evidence and society recommendations contributing to EUS-BD and EUS-GE gaining ground in everyday practice or tertiary referral centers and becoming crucial in improving the multidisciplinary management of cancer-related symptoms.
C1 [Vanella, Giuseppe; Dell'Anna, Giuseppe; Arcidiacono, Paolo Giorgio] IRCCS San Raffaele Sci Inst & Univ, Milan, Italy.
[Bronswijk, Michiel; van der Merwe, Schalk] Univ Leuven, Univ Hosp Gasthuisberg, Leuven, Belgium.
[Bronswijk, Michiel] Imelda Gen Hosp, Bonheiden, Belgium.
[van Wanrooij, Roy L. J.] Vrije Univ, Amsterdam UMC, Amsterdam, Netherlands.
[Rizzatti, Gianenrico; Larghi, Alberto] Catholic Univ, Fdn Policlin Univ A Gemelli IRCCS, Rome, Italy.
[Gkolfakis, Paraskevas] Univ Libre Bruxelles, Erasme Univ Hosp, Brussels, Belgium.
C3 KU Leuven; University Hospital Leuven; Imeldaziekenhuis; Vrije Universiteit Amsterdam; University of Amsterdam; Catholic University of the Sacred Heart; IRCCS Policlinico Gemelli; Universite Libre de Bruxelles

RP Vanella, G (corresponding author), IRCCS San Raffaele Sci Inst & Univ, Pancretobiliary Endoscopy & Endosonog Div, Pancreas Clin & Translat Res Ctr, Via Olgettina 60, I-20132 Milan, Italy.
EM vanella.giuseppe@hsr.it
RI ; Rizzatti, Gianenrico/B-1406-2019; Gkolfakis, Paraskevas/I-8375-2017;
Arcidiacono, Paolo Giorgio/K-2122-2018; Dell'Anna,
Giuseppe/IQT-0773-2023; Larghi, Alberto/AAC-7448-2022; Vanella,
Giuseppe/LQI-8930-2024
OI van Wanrooij, Roy L.J/0000-0003-0477-4532; Bronswijk,
Michiel/0000-0003-2039-5022; Arcidiacono, Paolo
Giorgio/0000-0001-6692-7720; Dell'Anna, Giuseppe/0000-0002-9395-5468;
Van der Merwe, Schalk/0000-0002-9891-2686;
CR Ahmed O, 2021, CLIN GASTROENTEROL H, V19, P1780, DOI 10.1016/j.cgh.2021.03.046
Antonelli G, 2020, DIGEST LIVER DIS, V52, P1294, DOI 10.1016/j.dld.2020.04.021
Balik E, 2013, J ONCOL, V2013, DOI 10.1155/2013/201681
Balzano G, 2021, HPB, V23, P1095, DOI 10.1016/j.hpb.2020.11.006
Bang JY, 2018, GASTROINTEST ENDOSC, V88, P9, DOI 10.1016/j.gie.2018.03.012
Boghossian MB, 2021, LANGENBECK ARCH SURG, V406, P1803, DOI 10.1007/s00423-021-02215-8
Bronswijk M, 2022, ENDOSC INT OPEN, V10, pE294, DOI 10.1055/a-1781-6960
Bronswijk M, 2021, GASTROINTEST ENDOSC, V94, P526, DOI 10.1016/j.gie.2021.04.006
Bronswijk Michiel, 2020, VideoGIE, V5, P442, DOI [10.1016/j.vgie.2020.06.012, 10.1016/j.vgie.2020.06.012]
Bronswijk M, 2021, ENDOSCOPY, V53, P204, DOI 10.1055/a-1190-3228
Caillol F, 2019, ENDOSC ULTRASOUND, V8, P199, DOI 10.4103/eus.eus_64_18
Caillol F, 2019, UNITED EUR GASTROENT, V7, P52, DOI 10.1177/2050640618803812
Chang JI, 2019, ENDOSC INT OPEN, V7, pE655, DOI 10.1055/a-0826-4309
De Bie C, 2022, SURG ENDOSC, V36, P2393, DOI 10.1007/s00464-021-08520-z
Dumonceau JM, 2018, ENDOSCOPY, V50, P910, DOI 10.1055/a-0659-9864
Fabbri C, 2019, J GASTROINTEST LIVER, V28, P125, DOI 10.15403/jgld.2014.1121.281.eus
Fan WT, 2022, MINIM INVASIV THER, V31, P159, DOI 10.1080/13645706.2020.1792500
Fugazza A, 2022, GASTROINTEST ENDOSC, V95, P896, DOI 10.1016/j.gie.2021.12.032
Garcia-Sumalla Albert, 2021, VideoGIE, V6, P263, DOI [10.1016/j.vgie.2021.03.002, 10.1016/j.vgie.2021.03.002]
Gaujoux S, 2021, HPB, V23, P154, DOI 10.1016/j.hpb.2020.06.001
Ge PS, 2019, SURG ENDOSC, V33, P3404, DOI 10.1007/s00464-018-06636-3
Ghandour B, 2022, GASTROINTEST ENDOSC, V95, P80, DOI 10.1016/j.gie.2021.07.023
Gutierrez OIB, 2018, ENDOSCOPY, V50, P891, DOI 10.1055/s-0044-102254
Hamada T, 2018, SCAND J GASTROENTERO, V53, P46, DOI 10.1080/00365521.2017.1382567
Han SY, 2019, SCI REP-UK, V9, DOI 10.1038/s41598-019-52993-x
Hara K, 2016, WORLD J GASTROENTERO, V22, P1297, DOI 10.3748/wjg.v22.i3.1297
Hathorn KE, 2022, GASTROINTEST ENDOSC, V95, P443, DOI 10.1016/j.gie.2021.10.013
Hayat U, 2022, ENDOSC ULTRASOUND, V11, P4, DOI 10.4103/EUS-D-21-00009
Imai H, 2016, GASTROINTEST ENDOSC, V84, P147, DOI 10.1016/j.gie.2015.12.024
Inamdar S, 2016, JAMA ONCOL, V2, P112, DOI 10.1001/jamaoncol.2015.3670
Iqbal U, 2020, ENDOSC ULTRASOUND, V9, P16, DOI 10.4103/eus.eus_70_19

Ishiwatari H, 2022, J HEPATO-BIL-PAN SCI, V29, P703, DOI 10.1002/jhbp.1118
Issa D, 2021, ENDOSCOPY, V53, P827, DOI 10.1055/a-1259-0349
James TW, 2020, GASTROINTEST ENDOSC, V91, P537, DOI 10.1016/j.gie.2019.11.017
James TW, 2019, ENDOSC INT OPEN, V7, pE600, DOI 10.1055/a-0867-9599
James TW, 2019, OBES SURG, V29, P451, DOI 10.1007/s11695-018-3531-2
Jin Z, 2020, DIGEST ENDOSC, V32, P16, DOI 10.1111/den.13456
Jovani M, 2021, GASTROINTEST ENDOSC, V93, P1088, DOI 10.1016/j.gie.2020.09.041
Jue TL, 2021, GASTROINTEST ENDOSC, V93, P309, DOI 10.1016/j.gie.2020.07.063
Kakked G, 2020, ENDOSC ULTRASOUND, V9, P298, DOI 10.4103/eus.eus_10_20
Kedia P, 2015, ENDOSCOPY, V47, P159, DOI 10.1055/s-0034-1390771
Khara Harshit S, 2021, Curr Gastroenterol Rep, V23, P10, DOI [10.1007/s11894-021-00808-3, 10.1007/s11894-021-00808-3]
Khashab Mouen A, 2019, Endoscopy, V51, P1119, DOI 10.1055/a-0958-2323
Kitano M, 2013, AM J GASTROENTEROL, V108, P1713, DOI 10.1038/ajg.2013.305
Kongkam P, 2021, ENDOSCOPY, V53, P55, DOI 10.1055/a-1195-8197
Behera RK, 2021, ABDOM RADIOL, V46, P768, DOI 10.1007/s00261-020-02651-y
Law R, 2021, GASTROINTEST ENDOSC, V93, P534, DOI 10.1016/j.gie.2020.08.033
Law RJ, 2021, GASTROINTEST ENDOSC, V94, P457, DOI 10.1016/j.gie.2021.05.020
Lee TH, 2016, CLIN GASTROENTEROL H, V14, P1011, DOI 10.1016/j.cgh.2015.12.032
Lyu YX, 2021, DIGEST LIVER DIS, V53, P1247, DOI 10.1016/j.dld.2021.03.029
Mao KJ, 2022, SURG LAPARO ENDO PER, V32, P124, DOI 10.1097/SLE.0000000000000992
Marrache MK, 2021, GASTROINTEST ENDOSC, V93, P34, DOI 10.1016/j.gie.2020.06.057
Matsubara S, 2020, J CLIN MED, V9, DOI 10.3390/jcm9123879
McCarty Thomas R, 2019, Endosc Int Open, V7, pE1474, DOI [10.1055/a-0996-8178, 10.1055/a-0996-8178]
Minaga K, 2020, THER ADV GASTROENTER, V13, DOI 10.1177/1756284820930964
Minata MK, 2016, ENDOSC INT OPEN, V4, pE1158, DOI 10.1055/s-0042-115935
Mohan BP, 2019, J CLIN GASTROENTEROL, V53, P243, DOI 10.1097/MCG.0000000000001167
Nagem RG, 2012, HEPATOB PANCREAT DIS, V11, P630, DOI 10.1016/S1499-3872(12)60236-7
Nakai Y, 2020, GASTROINTEST ENDOSC, V92, P623, DOI 10.1016/j.gie.2020.03.3856
Nakai Y, 2017, DIGEST ENDOSC, V29, P16, DOI 10.1111/den.12729
Nakai Y, 2016, GASTROINTEST ENDOSC, V83, P1302, DOI 10.1016/j.gie.2016.01.015
Nennstiel S, 2015, J CLIN GASTROENTEROL, V49, P764, DOI 10.1097/MCG.0000000000000275
NIH U.S. National Library of Medicine ClinicalTrials.gov, EUS GUIDED CHOLEDOCH
Oh D, 2017, THER ADV GASTROENTER, V10, P42, DOI 10.1177/1756283X16671671
Olsson G, 2021, SCAND J GASTROENTERO, V56, P336, DOI 10.1080/00365521.2020.1867894
Paik WH, 2018, AM J GASTROENTEROL, V113, P987, DOI 10.1038/s41395-018-0122-8
Park JC, 2012, DIGEST LIVER DIS, V44, P999, DOI 10.1016/j.dld.2012.06.019
Pedersoli F, 2021, EUR RADIOL, V31, P3035, DOI 10.1007/s00330-020-07368-6
Potz BA, 2016, WORLD J GASTRO SURG, V8, P545, DOI 10.4240/wjgs.v8.i8.545
Prakash S, 2022, ENDOSCOPY, V54, P52, DOI 10.1055/a-1376-2394
Quinn PL, 2020, HPB, V22, pS89, DOI 10.1016/j.hpb.2020.04.570
Rimbas M, 2021, ENDOSC INT OPEN, V09, pE1801, DOI 10.1055/a-1526-1208

Seo DW, 2019, GASTROINTEST ENDOSC, V90, P602, DOI 10.1016/j.gie.2019.06.032
Sharaiha RZ, 2017, GASTROINTEST ENDOSC, V85, P904, DOI 10.1016/j.gie.2016.12.023
Tamura T, 2021, DIGEST ENDOSC, V33, P1170, DOI 10.1111/den.13926
Tonnesen CJ, 2020, ENDOSCOPY, V52, P654, DOI 10.1055/a-1139-9313
Tsuchiya T, 2018, GASTROINTEST ENDOSC, V87, P1138, DOI 10.1016/j.gie.2017.08.017
Turan AS, 2022, ABDOM RADIOL, V47, P3338, DOI 10.1007/s00261-021-03207-4
Tyberg A, 2022, ENDOSC ULTRASOUND, V11, P38, DOI 10.4103/EUS-D-21-00006
van der Gaag NA, 2010, NEW ENGL J MED, V362, P129, DOI 10.1056/NEJMoa0903230
van der Merwe SW, 2022, ENDOSCOPY, V54, P185, DOI 10.1055/a-1717-1391
van Halsema EE, 2015, WORLD J GASTROENTERO, V21, P12468, DOI 10.3748/wjg.v21.i43.12468
van Wanrooij RLJ, 2022, ENDOSCOPY, V54, P1023, DOI 10.1055/a-1782-7568
van Wanrooij RLJ, 2022, ENDOSCOPY, V54, P310, DOI 10.1055/a-1738-6780
Vanella Giuseppe, 2022, VideoGIE, V7, P120, DOI [10.1016/j.vgie.2021.10.002, 10.1016/j.vgie.2021.10.002]
Vanella G, 2022, ENDOSCOPY, V54, pE143, DOI 10.1055/a-1408-1180
Vanella G, 2020, ENDOSC INT OPEN, V08, pE1782, DOI 10.1055/a-1264-7511
Vanella G, 2022, AM J GASTROENTEROL, V117, P1192, DOI 10.14309/ajg.0000000000001730
Vienne A, 2010, GASTROINTEST ENDOSC, V72, P728, DOI 10.1016/j.gie.2010.06.040
Yamamoto K, 2018, ENDOSC ULTRASOUND, V7, P404, DOI 10.4103/eus.eus_51_18
Zulli C, 2020, ENDOSCOPY, V52, pE215, DOI 10.1055/a-1073-7317
NR 91
TC 21
Z9 22
U1 0
U2 4
PU HELLENIC SOC GASTROENTEROLOGY
PI ATHENS
PA DEMOKRATIAS AVE 67, ATHENS, 15451, GREECE
SN 1108-7471
EI 1792-7463
J9 ANN GASTROENTEROL
JI Ann. Gastroenterol.
PY 2022
VL 35
IS 5
BP 441
EP 451
DI 10.20524/aog.2022.0736
EA JUL 2022
PG 11
WC Gastroenterology & Hepatology
WE Emerging Sources Citation Index (ESCI)
SC Gastroenterology & Hepatology

GA CP3R3
UT WOS:000829089000001
PM 36061163
OA Green Submitted, Green Published, gold
DA 2026-05-09
ER

PT J
AU Pawa, R
Pleasant, T
Tom, C
Pawa, S
AF Pawa, Rishi
Pleasant, Troy
Tom, Chloe
Pawa, Swati
TI Endoscopic ultrasound-guided biliary drainage: Are we there yet?
SO WORLD JOURNAL OF GASTROINTESTINAL ENDOSCOPY
LA English
DT Review
DE Endoscopic ultrasound-guided biliary drainage; Endoscopic ultrasound-guided rendezvous; Endoscopic ultrasound-guided choledochoduodenostomy; Endoscopic ultrasound-guided hepaticogastrostomy; Endoscopic ultrasound-guided gallbladder drainage; Endoscopic ultrasound-directed transgastric endoscopic retrograde cholangiopancreatography
ID APPOSING METAL STENT; LONG-TERM OUTCOMES; SURGICALLY ALTERED ANATOMY; RETROGRADE CHOLANGIOPANCREATOGRAPHY; BILE-DUCT; PERCUTANEOUS DRAINAGE; PRECUT PAPILLOTOMY; LEARNING-CURVE; PLASTIC STENT; CHOLEDOCHODUODENOSTOMY
AB Endoscopic retrograde cholangiopancreatography (ERCP) is the mainstay procedure of choice for management of obstructive biliary disease. While ERCP is widely performed with high success rates, the procedure is not feasible in every patient such as cases of non-accessible papilla. In the setting of unsuccessful ERCP, endoscopic ultrasound-guided biliary drainage (EUS-BD) has become a promising alternative to surgical bypass and percutaneous biliary drainage (PTBD). A variety of different forms of EUS-BD have been described, allowing for both intrahepatic and extrahepatic approaches. Recent studies have reported high success rates utilizing EUS-BD for both transpapillary and transluminal drainage, with fewer adverse events when compared to PTBD. Advancements in novel technologies designed specifically for EUS-BD have led to increased success rates as well as improved safety profile for the procedure. The techniques of EUS-BD are yet to be fully standardized and are currently performed by highly trained advanced endoscopists. The aim of our review is to highlight the different EUS-guided interventions for achieving biliary drainage and to both assess the progress that has been made in the field as well as consider what the future may hold.
C1 [Pawa, Rishi; Pleasant, Troy; Pawa, Swati] Wake Forest Baptist Med Ctr, Sect Gastroenterol & Hepatol, Dept Internal

Med, Med Ctr Blvd, Winston Salem, NC 27157 USA.
[Tom, Chloe] Wake Forest Sch Med, Dept Med, Winston Salem, NC 27101 USA.
C3 Wake Forest University; Wake Forest Baptist Medical Center; Wake Forest University
RP Pawa, R (corresponding author), Wake Forest Baptist Med Ctr, Sect Gastroenterol & Hepatol, Dept Internal Med, Med Ctr Blvd, Winston Salem, NC 27157 USA.
EM rpawa@wakehealth.edu
CR Artifon ELA, 2012, ENDOSC ULTRASOUND, V1, P2, DOI 10.7178/eus.01.002
Balik E, 2013, J ONCOL, V2013, DOI 10.1155/2013/201681
Baniya R, 2017, CLIN EXP GASTROENTER, V10, P67, DOI 10.2147/CEG.S132004
Bapaye A, 2013, UNITED EUR GASTROENT, V1, P285, DOI 10.1177/2050640613490928
Baron TH, 2007, GASTROINTEST ENDOSC, V65, P735, DOI 10.1016/j.gie.2006.07.041
Binmoeller KF, 2011, ENDOSCOPY, V43, P337, DOI 10.1055/s-0030-1256127
Bukhari M, 2018, GASTROINTEST ENDOSC, V88, P486, DOI 10.1016/j.gie.2018.04.2356
Burmester E, 2003, GASTROINTEST ENDOSC, V57, P246, DOI 10.1067/mge.2003.85
Caillol F, 2019, ENDOSC ULTRASOUND, V8, pS50, DOI 10.4103/eus.eus_52_19
Cho DH, 2017, GASTROINTEST ENDOSC, V85, P1067, DOI 10.1016/j.gie.2016.09.010
Artifon ELD, 2019, ENDOSC ULTRASOUND, V8, pS72, DOI 10.4103/eus.eus_62_19
DeBenedet AT, 2013, AM J GASTROENTEROL, V108, P1696, DOI 10.1038/ajg.2013.217
Dhir V, 2013, UNITED EUR GASTROENT, V1, P103, DOI 10.1177/2050640613480145
Dhir V, 2012, GASTROINTEST ENDOSC, V75, P354, DOI 10.1016/j.gie.2011.07.075
Dorrell R, 2020, CASE REP GASTROINTES, V2020, DOI 10.1155/2020/8866899
Dorrell R, 2021, DIGEST ENDOSC, V33, P204, DOI 10.1111/den.13851
El Chafic AH, 2019, DIGEST DIS SCI, V64, P3321, DOI 10.1007/s10620-019-05688-2
Enochsson L, 2010, GASTROINTEST ENDOSC, V72, P1175, DOI 10.1016/j.gie.2010.07.047
Fabbri C, 2019, J GASTROINTEST LIVER, V28, P125, DOI 10.15403/jgld.2014.1121.281.eus
Giovannini M, 2001, ENDOSCOPY, V33, P898, DOI 10.1055/s-2001-17324
Giovannini M, 2019, ENDOSC ULTRASOUND, V8, pS35, DOI 10.4103/eus.eus_47_19
Hara K, 2013, ENDOSCOPY, V45, P392, DOI 10.1055/s-0032-1326076
Hara K, 2016, WORLD J GASTROENTERO, V22, P1297, DOI 10.3748/wjg.v22.i3.1297
Hara K, 2011, AM J GASTROENTEROL, V106, P1239, DOI 10.1038/ajg.2011.84
Hedjoudje A, 2019, UNITED EUR GASTROENT, V7, P60, DOI 10.1177/2050640618808147
Honjo M, 2018, ENDOSC ULTRASOUND, V7, P376, DOI 10.4103/eus.eus_2_18
Huang C, 2016, ENDOSC INT OPEN, V4, pE1107, DOI 10.1055/s-0042-115407
Imai H, 2017, ONCOLOGY-BASEL, V93, P69, DOI 10.1159/000481233
Imai H, 2016, GASTROINTEST ENDOSC, V84, P147, DOI 10.1016/j.gie.2015.12.024
Irani Shayan, 2018, VideoGIE, V3, P322, DOI [10.1016/j.vgie.2018.07.008, 10.1016/j.vgie.2018.07.008]
Issa D, 2021, ENDOSCOPY, V53, P827, DOI 10.1055/a-1259-0349
Itoi T, 2008, WORLD J GASTROENTERO, V14, P6078, DOI 10.3748/wjg.14.6078
Itoi T, 2014, GASTROINTEST ENDOSC, V79, P715, DOI 10.1016/j.gie.2013.11.021
Itoi T, 2013, DIGEST ENDOSC, V25, P137, DOI 10.1111/den.12084
Itoi T, 2011, J HEPATO-BIL-PAN SCI, V18, P664, DOI 10.1007/s00534-011-0410-9

Itoi T, 2011, J HEPATO-BIL-PAN SCI, V18, P282, DOI 10.1007/s00534-010-0310-4
Iwashita T, 2017, DIGEST ENDOSC, V29, P362, DOI 10.1111/den.12800
Iwashita T, 2016, J HEPATO-BIL-PAN SCI, V23, P227, DOI 10.1002/jhbp.329
Iwashita T, 2016, GASTROINTEST ENDOSC, V83, P394, DOI 10.1016/j.gie.2015.04.043
Jacques J, 2019, ENDOSCOPY, V51, P540, DOI 10.1055/a-0735-9137
Jain D, 2018, DIGESTION, V98, P1, DOI 10.1159/000487185
James TW, 2018, GASTROINTEST ENDOSC, V88, P547, DOI 10.1016/j.gie.2018.04.2353
Kedia P, 2019, J CLIN GASTROENTEROL, V53, P304, DOI 10.1097/MCG.0000000000001037
Kedia P, 2014, GASTROENTEROLOGY, V147, P566, DOI 10.1053/j.gastro.2014.05.045
Khashab MA, 2016, ENDOSC INT OPEN, V4, pE175, DOI 10.1055/s-0041-109083
Khashab MA, 2015, DIGEST DIS SCI, V60, P557, DOI 10.1007/s10620-014-3300-6
Ki ELL, 2019, ENDOSC ULTRASOUND, V8, pS17, DOI 10.4103/eus.eus_50_19
Krishnamoorthi R, 2020, SURG ENDOSC, V34, P2866, DOI 10.1007/s00464-020-07484-w
Lee A, 2017, ENDOSCOPY, V49, P146, DOI 10.1055/s-0042-120995
Lee TH, 2016, CLIN GASTROENTEROL H, V14, P1011, DOI 10.1016/j.cgh.2015.12.032
Mallery S, 2004, GASTROINTEST ENDOSC, V59, P100, DOI 10.1016/S0016-5107(03)02300-9
Martínez B, 2019, ENDOSC INT OPEN, V7, pE1038, DOI 10.1055/a-0918-5931
Martins FP, 2010, ENDOSCOPY, V42, pE126, DOI 10.1055/s-0029-1243911
Matsubara S, 2020, J CLIN MED, V9, DOI 10.3390/jcm9123879
Matsumoto S, 2020, DIGEST ENDOSC, V32, P957, DOI 10.1111/den.13620
Miyano A, 2018, J GASTROINTEST SURG, V22, P1312, DOI 10.1007/s11605-018-3758-1
Mohan BP, 2019, J CLIN GASTROENTEROL, V53, P243, DOI 10.1097/MCG.0000000000001167
Moole H, 2017, MEDICINE, V96, DOI 10.1097/MD.0000000000005154
Mussetto A, 2018, ANN GASTROENTEROL, V31, P535, DOI 10.20524/aog.2018.0287
Nakai Y, 2020, GASTROINTEST ENDOSC, V92, P623, DOI 10.1016/j.gie.2020.03.3856
Nakai Y, 2019, ENDOSC ULTRASOUND, V8, P111, DOI 10.4103/eus.eus_17_18
Nennstiel S, 2015, J CLIN GASTROENTEROL, V49, P764, DOI 10.1097/MCG.0000000000000275
Ogura T, 2021, GUT LIVER, V15, P196
Ogura T, 2018, DIGEST ENDOSC, V30, P252, DOI 10.1111/den.12976
Ogura T, 2016, WORLD J GASTROENTERO, V22, P3945, DOI 10.3748/wjg.v22.i15.3945
Ogura T, 2015, J GASTROEN HEPATOL, V30, P1748, DOI 10.1111/jgh.13021
Ogura T, 2015, ENDOSCOPY, V47, pE583, DOI 10.1055/s-0034-1393381
Ogura T, 2014, ENDOSCOPY, V46, pE32, DOI 10.1055/s-0033-1359133
Oh D, 2017, THER ADV GASTROENTER, V10, P42, DOI 10.1177/1756283X16671671
Okuno N, 2015, ENDOSCOPY, V47, pE311, DOI 10.1055/s-0034-1392314
Park DH, 2015, J GASTROEN HEPATOL, V30, P1461, DOI 10.1111/jgh.13027
Park DH, 2011, GASTROINTEST ENDOSC, V74, P1276, DOI 10.1016/j.gie.2011.07.054
Park SJ, 2013, GASTROINTEST ENDOSC, V78, P374, DOI 10.1016/j.gie.2013.04.183
Peng CY, 2013, BMC GASTROENTEROL, V13, DOI 10.1186/1471-230X-13-147
Rebello C, 2012, ENDOSC ULTRASOUND, V1, P156, DOI 10.7178/eus.03.007
Runge TM, 2021, ENDOSCOPY, V53, P611, DOI 10.1055/a-1254-3942
Ryou M, 2020, GASTROINTEST ENDOSC, V91, P178, DOI 10.1016/j.gie.2019.07.035

Sarkaria S, 2013, GUT LIVER, V7, P129, DOI 10.5009/gnl.2013.7.2.129
Sharaiha RZ, 2017, GASTROINTEST ENDOSC, V85, P904, DOI 10.1016/j.gie.2016.12.023
Sharma M, 2013, ENDOSC ULTRASOUND, V2, P171, DOI [10.4103/2303-9027.117679, 10.7178/eus.06.0013]
Sobani ZA, 2021, DIGEST DIS SCI, V66, P2154, DOI 10.1007/s10620-020-06520-y
Song TJ, 2012, WORLD J GASTROENTERO, V18, P4435, DOI 10.3748/wjg.v18.i32.4435
Song TJ, 2010, GASTROINTEST ENDOSC, V71, P634, DOI 10.1016/j.gie.2009.11.024
Tsuchiya T, 2018, GASTROINTEST ENDOSC, V87, P1138, DOI 10.1016/j.gie.2017.08.017
Tyberg A, 2020, ENDOSC ULTRASOUND, V9, P392, DOI 10.4103/eus.eus_42_20
Tyberg A, 2017, CLIN ENDOSC, V50, P185, DOI 10.5946/ce.2016.030
Uchida D, 2018, J MED ULTRASON, V45, P399, DOI 10.1007/s10396-017-0846-x
Uemura RS, 2018, J CLIN GASTROENTEROL, V52, P123, DOI 10.1097/MCG.0000000000000948
Umeda J, 2015, GASTROINTEST ENDOSC, V82, P390, DOI 10.1016/j.gie.2015.02.041
Ustundag Y, 2012, GASTROINTEST ENDOSC, V76, P466, DOI 10.1016/j.gie.2012.02.013
Wiersema MJ, 1996, GASTROINTEST ENDOSC, V43, P102, DOI 10.1016/S0016-5107(06)80108-2
Yamao K, 2006, GASTROINTEST ENDOSC, V64, P663, DOI 10.1016/j.gie.2006.07.003
NR 92
TC 9
Z9 12
U1 1
U2 7
PU BAISHIDENG PUBLISHING GROUP INC
PI PLEASANTON
PA 7041 Koll Center Parkway, Suite 160, PLEASANTON, CA, UNITED STATES
SN 1948-5190
J9 WORLD J GASTRO ENDOS
JI World J. Gastrointest. Endosc.
PD AUG 16
PY 2021
VL 13
IS 8
BP 302
EP 318
DI 10.4253/wjge.v13.i8.302
PG 17
WC Gastroenterology & Hepatology
WE Emerging Sources Citation Index (ESCI)
SC Gastroenterology & Hepatology
GA UM8KK
UT WOS:000693574600005
PM 34512878
OA Green Accepted, gold
DA 2026-05-09

ER

PT J
AU Cho, DH
Jo, SJ
Lee, JH
Song, TJ
Park, DH
Lee, SK
Kim, MH
Lee, SS
AF Cho, Dong Hui
Jo, Seok Jung
Lee, Jae Hoon
Song, Tae Jun
Park, Do Hyun
Lee, Sung Koo
Kim, Myung-Hwan
Lee, Sang Soo
TI Feasibility and safety of endoscopic ultrasound-guided gallbladder drainage using a newly designed lumen-apposing metal stent
SO SURGICAL ENDOSCOPY AND OTHER INTERVENTIONAL TECHNIQUES
LA English
DT Article
DE Endoscopic ultrasound; Cholecystitis; Gallbladder; Drainage; Metal stent
ID PERCUTANEOUS CHOLECYSTOSTOMY; ACUTE CHOLECYSTITIS; OUTCOMES
AB Background and aimsEndoscopic ultrasound-guided gallbladder drainage (EUS-GBD) is increasingly accepted as an effective treatment option in patients who require drainage for acute cholecystitis. A newly designed lumen-apposing metal stent (LAMS) has been introduced recently in this procedure. In this study, we evaluated the feasibility and safety of the newly designed LAMS in patients with acute cholecystitis who were unsuitable for cholecystectomy.MethodsBetween Mar 2017 and Oct 2017, 22 patients with acute cholecystitis who were unsuitable for cholecystectomy underwent EUS-GBD with the newly designed LAMS. We evaluated the technical and clinical success and the adverse event profiles.ResultsEUS-GBD with newly designed LAMS was technically and clinically successful in 21 of the 22 patients. EUS-GB stenting was performed at urgent setting in 17 patients, while 5 patients, who had undergone initial PTGBD, underwent EUS-GB stenting to remove PTGBD tube. The median procedure time was 11.5 (range 8.8-17.0) min. A late adverse event of stent occlusion developed in one patient. Stent migration was not observed during follow-up (median 318.0 days, range 39.0-398.0days) and cumulative stent patency rate at 1year was 95%.ConclusionEUS-GBD with newly designed LAMS is feasible and shows acceptable safety profiles for both the urgent drainage of acute cholecystitis and elective internalization following PTGBD in patients with high surgical risk.
C1 [Cho, Dong Hui; Jo, Seok Jung; Song, Tae Jun; Park, Do Hyun; Lee, Sung Koo; Kim, Myung-Hwan; Lee, Sang Soo] Univ Ulsan, Dept Gastroenterol, Asan Med Ctr, Coll Med, 88 Olymp Ro 43 Gil, Seoul 05505, South Korea.
[Lee, Jae Hoon] Univ Ulsan, Dept Surg, Asan Med Ctr, Coll Med, Seoul, South Korea.

C3 University of Ulsan; Asan Medical Center; University of Ulsan; Asan
Medical Center
RP Lee, SS (corresponding author), Univ Ulsan, Dept Gastroenterol, Asan Med Ctr, Coll Med, 88 Olymp Ro 43 Gil, Seoul 05505, South Korea.
EM ssleedr@amc.seoul.kr
CR Anderloni A, 2016, SURG ENDOSC, V30, P5200, DOI 10.1007/s00464-016-4894-x
Baron TH, 2007, GASTROINTEST ENDOSC, V65, P735, DOI 10.1016/j.gie.2006.07.041
Choi JH, 2017, GASTROINTEST ENDOSC, V85, P357, DOI 10.1016/j.gie.2016.07.067
Choi JH, 2014, ENDOSCOPY, V46, P656, DOI 10.1055/s-0034-1365720
de la Serna-Higuera C, 2013, GASTROINTEST ENDOSC, V77, P303, DOI 10.1016/j.gie.2012.09.021
Irani S, 2015, GASTROINTEST ENDOSC, V82, P1110, DOI 10.1016/j.gie.2015.05.045
Jamwal KD, 2018, J CLIN TRANSL HEPATO, V6, P35, DOI 10.14218/JCTH.2017.00018
Jang JW, 2012, GASTROENTEROLOGY, V142, P805, DOI 10.1053/j.gastro.2011.12.051
Jang JW, 2011, GASTROINTEST ENDOSC, V74, P176, DOI 10.1016/j.gie.2011.03.1120
Kim JJ, 2017, J CLIN GASTROENTEROL
Kunda R, 2016, SURG ENDOSC, V30, P5002, DOI 10.1007/s00464-016-4845-6
Law R, 2016, CLIN GASTROENTEROL H, V14, P476, DOI 10.1016/j.cgh.2015.10.026
Lee SS, 2007, GASTROINTEST ENDOSC, V66, P1008, DOI 10.1016/j.gie.2007.03.1080
Loozen CS, 2017, DIGEST SURG, V34, P371, DOI 10.1159/000455241
Moon JH, 2014, GASTROINTEST ENDOSC, V79, P990, DOI 10.1016/j.gie.2014.02.015
Okamoto K, 2013, J HEPATO-BIL-PAN SCI, V20, P55, DOI 10.1007/s00534-012-0562-2
Sanjay P, 2013, HPB, V15, P511, DOI 10.1111/j.1477-2574.2012.00610.x
Takada T, 2013, J HEPATO-BIL-PAN SCI, V20, P1, DOI 10.1007/s00534-012-0566-y
Tsuyuguchi T, 2013, J HEPATO-BIL-PAN SCI, V20, P81, DOI 10.1007/s00534-012-0570-2
Tyberg Amy., 2016, J CLIN GASTROENTEROL
Walter D, 2016, GUT, V65, P6, DOI 10.1136/gutjnl-2015-309925
Yokoe M, 2013, J HEPATO-BIL-PAN SCI, V20, P35, DOI 10.1007/s00534-012-0568-9
NR 22
TC 23
Z9 27
U1 0
U2 2
PU SPRINGER
PI NEW YORK
PA 233 SPRING ST, NEW YORK, NY 10013 USA
SN 0930-2794
EI 1432-2218
J9 SURG ENDOSC
JI Surg. Endosc.
PD JUL
PY 2019
VL 33

IS 7
BP 2135
EP 2141
DI 10.1007/s00464-018-6485-5
PG 7
WC Surgery
WE Science Citation Index Expanded (SCI-EXPANDED)
SC Surgery
GA ID4CC
UT WOS:000471622800011
PM 30334155
DA 2026-05-09
ER

PT J
AU Kappelle, WFW
Bleys, RLAW
van Wijck, AJM
Siersema, PD
Vleggaar, FP
AF Kappelle, Wouter F. W.
Bleys, Ronald L. A. W.
van Wijck, Albert J. M.
Siersema, Peter D.
Vleggaar, Frank P.
TI EUS-guided celiac ganglia neurolysis: a clinical and human cadaver study
SO GASTROINTESTINAL ENDOSCOPY
LA English
DT Article
ID UNRESECTABLE PANCREATIC-CANCER; ENDOSCOPIC ULTRASOUND; PLEXUS NEUROLYSIS; BLOCK; PAIN; CARCINOMA; EFFICACY; TRIAL
AB Background and Aims: There is little evidence that structures targeted during EUS-guided celiac ganglia neurolysis (EUS-CGN) are celiac ganglia and that selective ethanol injection into ganglia is feasible. We aimed to visualize celiac ganglia, confirm that these structures are ganglia, and visualize ethanol spread after EUS-CGN and EUS-guided celiac plexus neurolysis (EUS-CPN).

Methods: First, celiac ganglia were sought during 97 consecutive EUS procedures. Second, ganglia were identified in a prosected human cadaver by placing a linear echoendoscope next to the celiac trunk and removing the underlying tissue for histology. Finally, various EUS-CGN and EUS-CPN techniques were performed in human cadavers; EUS-CGN was performed with 1 mL ethanol in 1 ganglion, 1 mL per ganglion (both low volume), and 4 mL per ganglion (high volume). EUS-CPN was performed with a central (20 mL) and a bilateral (2*10 mL) approach. Transverse sections (75 mu m) were obtained and photographed to allow visualization of the spread of ethanol.

Results: A total of 204 ganglia were detected in 83 patients. Mean (+/- standard deviation) size of the long axis was 8.1

mm (+/- 7.4 mm). Histology of the removed region in the cadaver showed only nerve cell bodies. After low-volume EUS-CGN in cadavers, ethanol spread well beyond the targeted ganglion. After high-volume EUS-CGN in cadavers, a larger ethanol spread was seen, which also reached unidentified ganglia; the spread was comparable to the spread after EUS-CPN.

Conclusions: Specific EUS-CGN is not feasible because ethanol spreads well beyond the targeted ganglion. Unidentified celiac ganglia are better reached with high-volume EUS-CGN, and this would likely result in a more thorough neurolysis. High-volume EUS-CGN should be preferred to low-volume EUS-CGN.
C1 [Kappelle, Wouter F. W.; Siersema, Peter D.; Vleggaar, Frank P.] Univ Med Ctr Utrecht, Dept Gastroenterol & Hepatol, POB 85500, NL-3508 GA Utrecht, Netherlands.

[Bleys, Ronald L. A. W.] Univ Med Ctr Utrecht, Dept Anat, Utrecht, Netherlands.

[van Wijck, Albert J. M.] Univ Med Ctr Utrecht, Dept Anesthesiol, Utrecht, Netherlands.

[Siersema, Peter D.] Radboud Univ Nijmegen, Med Ctr, Dept Gastroenterol & Hepatol, Nijmegen, Netherlands.

C3 Utrecht University; Utrecht University Medical Center; Utrecht University; Utrecht University Medical Center; Utrecht University; Utrecht University Medical Center; Radboud University Nijmegen

RP Vleggaar, FP (corresponding author), Univ Med Ctr Utrecht, Dept Gastroenterol & Hepatol, POB 85500, NL-3508 GA Utrecht, Netherlands.

RI Bleys, Ronald/AFK-5277-2022; Siersema, Peter/V-1636-2019
CR Caraceni A, 1996, CANCER-AM CANCER SOC, V78, P639

Cherny N, 2001, J CLIN ONCOL, V19, P2542, DOI 10.1200/JCO.2001.19.9.2542

Doi S, 2013, ENDOSCOPY, V45, P362, DOI 10.1055/s-0032-1326225

EISENBERG E, 1995, ANESTH ANALG, V80, P290, DOI 10.1097/00000539-199502000-00015

Furness JB, 2014, ADV EXP MED BIOL, V817, P39, DOI 10.1007/978-1-4939-0897-4_3

Gleeson FC, 2007, ENDOSCOPY, V39, P620, DOI 10.1055/s-2007-966337

ISCHIA S, 1983, PAIN, V16, P333, DOI 10.1016/0304-3959(83)90148-3

ISCHIA S, 1992, ANESTHESIOLOGY, V76, P534, DOI 10.1097/00000542-199204000-00008

Kappis M, 1914, Verh. Dtsch. Ges. Chir, V43, P87

Kaufman M, 2010, J CLIN GASTROENTEROL, V44, P127, DOI 10.1097/MCG.0b013e3181bb854d

LeBlanc JK, 2011, GASTROINTEST ENDOSC, V74, P1300, DOI 10.1016/j.gie.2011.07.073

Levy MJ, 2008, AM J GASTROENTEROL, V103, P98, DOI 10.1111/j.1572-0241.2007.01607.x

Levy MJ, 2003, GASTROINTEST ENDOSC, V57, P923, DOI 10.1016/S0016-5107(03)70036-4

LIEBERMAN RP, 1990, RADIOLOGY, V175, P274, DOI 10.1148/radiology.175.1.2315494

Minaga K, 2016, THER ADV GASTROENTER, V9, P483, DOI 10.1177/1756283X16644248

Moayeri Nizar, 2008, Anesthesiology, V108, P299, DOI 10.1097/01.anes.0000299433.25179.70

Sahai AV, 2013, ENDOSCOPY, V45, P854, DOI 10.1055/s-0033-1344389

Sahai AV, 2009, AM J GASTROENTEROL, V104, P326, DOI 10.1038/ajg.2008.64

Téllez-Avila FI, 2013, ENDOSC ULTRASOUND, V2, P153, DOI [10.4103/2303-9027.117664, 10.7178/eus.06.007]

VANLEEUWEN MBM, 1990, STAIN TECHNOL, V65, P37, DOI 10.3109/10520299009105606

Wang KX, 2012, GASTROINTEST ENDOSC, V76, P945, DOI 10.1016/j.gie.2012.05.032

WARD EM, 1979, ANESTH ANALG, V58, P461

Wiersema MJ, 1996, GASTROINTEST ENDOSC, V44, P656, DOI 10.1016/S0016-5107(96)70047-0

Wong GY, 2004, JAMA-J AM MED ASSOC, V291, P1092, DOI 10.1001/jama.291.9.1092

Zhang XM, 2006, AM J ROENTGENOL, V186, P1520, DOI 10.2214/AJR.04.1765

NR 25
TC 19
Z9 24
U1 0
U2 0
PU MOSBY-ELSEVIER
PI NEW YORK
PA 360 PARK AVENUE SOUTH, NEW YORK, NY 10010-1710 USA
SN 0016-5107
EI 1097-6779
J9 GASTROINTEST ENDOSC
JI Gastrointest. Endosc.
PD OCT
PY 2017
VL 86
IS 4
BP 655
EP 663
DI 10.1016/j.gie.2017.01.041
PG 9
WC Gastroenterology & Hepatology
WE Science Citation Index Expanded (SCI-EXPANDED)
SC Gastroenterology & Hepatology
GA FG7SN
UT WOS:000410624300013
PM 28188723
DA 2026-05-09
ER

PT J
AU Srinivasan, I
Tang, SJ
Vilmann, AS
Menachery, J
Vilmann, P
AF Srinivasan, Indu
Tang, Shou-Jiang
Vilmann, Andreas S.
Menachery, John
Vilmann, Peter
TI Hepatic applications of endoscopic ultrasound: Current status and future
directions

SO WORLD JOURNAL OF GASTROENTEROLOGY
LA English
DT Review
DE Endoscopic ultrasound; Drainage; Liver; Intrahepatic lesion
ID FINE-NEEDLE-ASPIRATION; GUIDED BILIARY DRAINAGE; PORTAL-VEIN CATHETERIZATION; ESOPHAGEAL COLLATERAL VEINS; HEPATOCELLULAR-CARCINOMA; PORTOSYSTEMIC SHUNT; ABSCESS DRAINAGE; GASTRIC VARICES; LIVER-LESIONS; EUS
AB The diagnosis and staging of various gastrointestinal malignancies have been made possible with the use of endoscopic ultrasound, which is a relatively safe procedure. The field of endoscopic ultrasound is fast expanding due to advancements in therapeutic endoscopic ultrasound. Though various studies have established its role in gastrointestinal malignancies and pancreatic conditions, its potential in the field of hepatic lesions still remains vastly untapped. In this paper the authors attempt to review important and landmark trials, case series and case studies involving hepatic applications of endoscopic ultrasound, thus not only providing an overview of utilization of endoscopic ultrasound in various liver conditions but also speculating its future role.
C1 [Srinivasan, Indu; Tang, Shou-Jiang] Univ Mississippi, Med Ctr, Dept Med, Div Digest Dis, Jackson, MS 39216 USA.
[Vilmann, Andreas S.; Vilmann, Peter] Univ Copenhagen, Herlev Hosp, Dept Surg Gastroenterol, DK-2730 Herlev, Denmark.
[Menachery, John] Rajagiri Hosp Aluva, Dept Gastroenterol, Kerala 683112, India.
C3 University of Mississippi; University of Mississippi Medical Center; University of Copenhagen; Herlev & Gentofte Hospital
RP Vilmann, P (corresponding author), Univ Copenhagen, Herlev Hosp, Dept Surg Gastroenterol, Norregade 10, DK-2730 Herlev, Denmark.
EM peter.vilmann@regionh.dk
RI Vilmann, Peter/AAJ-8401-2020
CR Albanis Efsevia, 2006, Clin Liver Dis, V10, P821, DOI 10.1016/j.cld.2006.08.027
Alvarez-Sanchez MV, 2014, SURG ENDOSC, V28, P712, DOI 10.1007/s00464-013-3260-5
[Anonymous], J INTERV GASTROENTER
Awad SS, 2002, AM J SURG, V184, P601, DOI 10.1016/S0002-9610(02)01092-9
Barclay RL, 2002, GASTROINTEST ENDOSC, V55, P266, DOI 10.1067/mge.2002.120784
BEAN WJ, 1985, AM J ROENTGENOL, V144, P237, DOI 10.2214/ajr.144.2.237
Bedossa P, 2003, HEPATOLOGY, V38, P1449, DOI 10.1016/j.hep.2003.09.022
BERGAMINI TM, 1987, AM SURGEON, V53, P596
BERTEL CK, 1986, ARCH SURG-CHICAGO, V121, P554
BLOECHLE C, 1994, AM J GASTROENTEROL, V89, P1537
Bogstad J, 1997, ENDOSCOPY, V29, P322, DOI 10.1055/s-2007-1004198
Bories E, 2007, ENDOSCOPY, V39, P287, DOI 10.1055/s-2007-966212
Bureau C, 2007, LIVER INT, V27, P742, DOI 10.1111/j.1478-3231.2007.01522.x
Buscaglia JM, 2009, GASTROINTEST ENDOSC, V69, P941, DOI 10.1016/j.gie.2008.09.051
Cadranel JF, 2000, HEPATOLOGY, V32, P477, DOI 10.1053/jhep.2000.16602
CALETTI G, 1990, GASTROINTEST ENDOSC, V36, pS21, DOI 10.1016/S0016-5107(90)71011-5
Cattan P, 1999, GASTROINTEST ENDOSC, V49, P252, DOI 10.1016/S0016-5107(99)70497-9

CHANG KJ, 1994, GASTROINTEST ENDOSC, V40, P694, DOI 10.1016/S0016-5107(94)70112-1
Choi JH, 2014, GUT LIVER, V8, P88, DOI 10.5009/gnl.2014.8.1.88
Choudhuri G, 1996, HEPATO-GASTROENTEROL, V43, P1250
Chung Y. F. A., 2003, SMJ Singapore Medical Journal, V44, P299
Clark TWI, 2004, J VASC INTERV RADIOL, V15, P147, DOI 10.1097/01.RVI.0000109401.52762.56
CLARKE MP, 1989, SURGERY, V106, P849
Colombato L, 2007, J CLIN GASTROENTEROL, V41, pS344, DOI 10.1097/MCG.0b013e318157e500
Crowe DR, 2006, CANCER CYTOPATHOL, V108, P180, DOI 10.1002/cncr.21912
CURET P, 1984, WORLD J SURG, V8, P2, DOI 10.1007/BF01658356
de Paulo GA, 2006, GASTROINTEST ENDOSC, V63, P396, DOI 10.1016/j.gie.2005.10.039
DeWitt J, 2003, AM J GASTROENTEROL, V98, P1976, DOI 10.1016/S0002-9270(03)00549-5
Dhir V, 2012, GASTROINTEST ENDOSC, V75, P354, DOI 10.1016/j.gie.2011.07.075
Di Matteo F, 2011, GASTROINTEST ENDOSC, V73, P632, DOI 10.1016/j.gie.2010.08.019
Fritscher-Ravens A, 2000, GASTROINTEST ENDOSC, V52, P534, DOI 10.1067/mge.2000.109589
Giday SA, 2008, GASTROINTEST ENDOSC, V67, P338, DOI 10.1016/j.gie.2007.08.037
Giday SA, 2007, GASTROINTEST ENDOSC, V66, P814, DOI 10.1016/j.gie.2007.05.056
Giovannini M, 2003, ENDOSCOPY, V35, P1076, DOI 10.1055/s-2003-44596
Giovannini M, 2012, GASTROENT RES PRACT, V2012, DOI 10.1155/2012/348719
Glasbrenner B, 1999, ENDOSCOPY, V31, P712
Hara K, 2011, DIGEST ENDOSC, V23, P26, DOI 10.1111/j.1443-1661.2011.01137.x
Hemming AW, 2003, ANN SURG, V237, P686, DOI 10.1097/00000658-200305000-00011
Hollerbach S, 2003, ENDOSCOPY, V35, P743, DOI 10.1055/s-2003-41593
Hu YH, 2010, ENDOSCOPY, V42, pE256, DOI 10.1055/s-0030-1255653
Irisawa A, 2003, J GASTROEN HEPATOL, V18, P309, DOI 10.1046/j.1440-1746.2003.02956.x
Irisawa A, 2001, GASTROINTEST ENDOSC, V53, P77, DOI 10.1067/mge.2001.108479
Itoh A, 2006, RADIOLOGY, V239, P341, DOI 10.1148/radiol.2391041676
Itoi T, 2014, J HEPATO-BIL-PAN SCI, V21, pE86, DOI 10.1002/jhbp.165
Itoi T, 2010, J HEPATO-BIL-PAN SCI, V17, P611, DOI 10.1007/s00534-009-0196-1
Iwashita T, 2012, ENDOSCOPY, V44, P60, DOI 10.1055/s-0030-1256871
Jhala NC, 2003, AM J CLIN PATHOL, V120, P351, DOI 10.1309/MFRFJ0XYJLN8NVDP
Kahaleh M, 2004, GASTROINTEST ENDOSC, V60, P138, DOI 10.1016/S0016-5107(04)01528-7
Karam AR, 2011, J RADIOL CASE REP, V5, P19, DOI 10.3941/jrcr.v5i2.634
Kato K, 2008, LIVER INT, V28, P1264, DOI 10.1111/j.1478-3231.2008.01701.x
Kaul V, 2010, GASTROINTEST ENDOSC, V72, P1, DOI 10.1016/j.gie.2010.01.023
Kawakubo K, 2014, J HEPATO-BIL-PAN SCI, V21, P328, DOI 10.1002/jhbp.27
Kawakubo K, 2013, SURG ENDOSC, V27, P3437, DOI 10.1007/s00464-013-2896-5
Konishi Y, 2002, GASTROINTEST ENDOSC, V55, P197, DOI 10.1067/mge.2002.121338
Kuramochi A, 2007, J GASTROENTEROL, V42, P219, DOI 10.1007/s00535-006-1992-x
Lai L, 2004, GASTROINTEST ENDOSC, V59, P280, DOI 10.1016/S0016-5107(03)02544-6
Lee S, 2014, GASTROINTEST ENDOSC, V80, P1014, DOI 10.1016/j.gie.2014.03.037
Lee YT, 2002, ENDOSCOPY, V34, P391, DOI 10.1055/s-2002-25286
Liu CH, 2009, J VASC INTERV RADIOL, V20, P1059, DOI 10.1016/j.jvir.2009.04.062

Liu H, 2005, WORLD J GASTROENTERO, V11, P2051, DOI 10.3748/wjg.v11.i14.2051
Mallery S, 2004, GASTROINTEST ENDOSC, V59, P100, DOI 10.1016/S0016-5107(03)02300-9
Maranki J, 2009, ENDOSCOPY, V41, P532, DOI 10.1055/s-0029-1214712
Matthes K, 2005, ACTA GASTRO-ENT BELG, V68, P412
McGrath K, 2006, AM J GASTROENTEROL, V101, P1742, DOI 10.1111/j.1572-0241.2006.00665.x
McKiernan PJ, 2008, TRANSPLANTATION, V86, P1470, DOI 10.1097/TP.0b013e3181891d63
Nadan R, 2007, GASTROINTEST ENDOSC, V66, P823, DOI 10.1016/j.gie.2007.06.047
Nakaji S, 2012, Endoscopy, V44 Suppl 2 UCTN, pE380, DOI 10.1055/s-0032-1309918
Nguyen P, 1999, GASTROINTEST ENDOSC, V50, P357, DOI 10.1053/ge.1999.v50.97208
Noh SH, 2010, GASTROINTEST ENDOSC, V71, P1314, DOI 10.1016/j.gie.2009.12.045
Ogura T, 2014, J GASTROEN HEPATOL, V29, P1815, DOI 10.1111/jgh.12598
Ogura T, 2014, ENDOSCOPY, V46, P416, DOI 10.1055/s-0034-1365020
Ogura T, 2014, ENDOSCOPY, V46, pE32, DOI 10.1055/s-0033-1359133
Panpimanmas Sukij, 2011, J Med Assoc Thai, V94 Suppl 2, pS129
Park DH, 2013, GASTROINTEST ENDOSC, V78, P91, DOI 10.1016/j.gie.2013.01.042
Park DH, 2012, GASTROINTEST ENDOSC, V75, P692, DOI 10.1016/j.gie.2011.04.013
Park SJ, 2013, GASTROINTEST ENDOSC, V78, P374, DOI 10.1016/j.gie.2013.04.183
Prasad P, 2004, GASTROINTEST ENDOSC, V59, P49, DOI 10.1016/S0016-5107(03)02378-2
Püspök A, 2005, AM J GASTROENTEROL, V100, P1743, DOI 10.1111/j.1572-0241.2005.41806.x
RIFKIN MD, 1986, SCAND J GASTROENTERO, V21, P68, DOI 10.3109/00365528609091865
Rimbas M, 2009, Curr Health Sci J, V35, P225
Romero-Castro R, 2007, GASTROINTEST ENDOSC, V66, P402, DOI 10.1016/j.gie.2007.03.008
Romero-Castro R, 2013, GASTROINTEST ENDOSC, V78, P711, DOI 10.1016/j.gie.2013.05.009
ROSCH J, 1969, RADIOLOGY, V92, P1112
RYAN ME, 1991, GASTROINTEST ENDOSC, V37, P139, DOI 10.1016/S0016-5107(91)70671-8
Saftoiu A, 2008, GASTROINTEST ENDOSC, V68, P1086, DOI 10.1016/j.gie.2008.04.031
Saftoiu Adrian, 2006, J Gastrointestin Liver Dis, V15, P161
SAINI S, 1983, AM J ROENTGENOL, V141, P559, DOI 10.2214/ajr.141.3.559
Sandulescu Larisa, 2012, Curr Health Sci J, V38, P32
Sarkaria S, 2013, GUT LIVER, V7, P129, DOI 10.5009/gnl.2013.7.2.129
Sato T, 2009, HEPATOL RES, V39, P126, DOI 10.1111/j.1872-034X.2008.00415.x
Seewald S, 2005, GASTROINTEST ENDOSC, V61, P495, DOI 10.1016/S0016-5107(04)02848-2
Shah JN, 2012, GASTROINTEST ENDOSC, V75, P56, DOI 10.1016/j.gie.2011.08.032
Shin HJC, 2002, CANCER CYTOPATHOL, V96, P174, DOI 10.1002/cncr.10614
Singh P, 2009, J CLIN GASTROENTEROL, V43, P367, DOI 10.1097/MCG.0b013e318167b8cc
Singhal S, 2014, THER ADV GASTROENTER, V7, P93, DOI 10.1177/1756283X13506178
SOYER P, 1993, AM J ROENTGENOL, V160, P511, DOI 10.2214/ajr.160.3.8430544
Tazawa J, 1997, AM J GASTROENTEROL, V92, P271
tenBerge J, EUS
Tierney WM, 2007, GASTROINTEST ENDOSC, V66, P435, DOI 10.1016/j.gie.2007.05.028
Trakarnsanga A, 2010, ENDOSCOPY, V42, pE340, DOI 10.1055/s-0030-1255940
Tsutsumi M, 2007, INT J CLIN ONCOL, V12, P250, DOI 10.1007/s10147-007-0669-7

VILMANN P, 1992, GASTROINTEST ENDOSC, V38, P172, DOI 10.1016/S0016-5107(92)70385-X
Wells RG, 2005, J CLIN GASTROENTEROL, V39, pS158, DOI 10.1097/01.mcg.0000155516.02468.0f
Winick A B, 2001, Tech Vasc Interv Radiol, V4, P200, DOI 10.1016/S1089-2516(01)90026-5
Wong Florence, 2006, Ann Hepatol, V5, P5
YOSHIDA J, 1987, AM J GASTROENTEROL, V82, P448
NR 106
TC 8
Z9 11
U1 0
U2 1
PU BAISHIDENG PUBLISHING GROUP INC
PI PLEASANTON
PA 7041 Koll Center Parkway, Suite 160, PLEASANTON, CA, UNITED STATES
SN 1007-9327
EI 2219-2840
J9 WORLD J GASTROENTERO
JI World J. Gastroenterol.
PD NOV 28
PY 2015
VL 21
IS 44
BP 12544
EP 12557
DI 10.3748/wjg.v21.i44.12544
PG 14
WC Gastroenterology & Hepatology
WE Science Citation Index Expanded (SCI-EXPANDED)
SC Gastroenterology & Hepatology
GA CX8PP
UT WOS:000365966300003
PM 26640331
OA Green Submitted, Bronze
DA 2026-05-09
ER

PT J
AU Ang, TL
AF Ang, Tiing Leong
TI Endoscopic ultrasound: Moving from diagnostics to therapeutics
SO JOURNAL OF DIGESTIVE DISEASES
LA English
DT Review

DE diagnosis; drainage; endosonography; therapy
ID EUS-GUIDED DRAINAGE; FINE-NEEDLE-ASPIRATION; HELICAL
COMPUTED-TOMOGRAPHY; CELIAC PLEXUS NEUROLYSIS; DOUBLE-WIRE TECHNIQUE;
BILE-DUCT STONES; PANCREATIC PSEUDOCYSTS; TRANSMURAL DRAINAGE;
ESOPHAGEAL CANCER; GASTRIC-CANCER
AB Endoscopic ultrasound was initially introduced in the 1980s as a diagnostic modality using echoendoscopes with radial scanning that permitted reconstruction of cross-sectional images similar to computed tomography. The close proximity of the ultrasound transducer to the gastrointestinal wall and retroperitoneal structures allowed a detailed examination that is not possible with cross-sectional imaging such as computed tomography and magnetic resonance imaging. It proved to be highly accurate and useful in the staging of gastrointestinal malignancies, as well as in characterizing the nature of subepithelial lesions and disorders of the pancreaticobiliary system. The introduction of linear echoendoscopes facilitated fine needle aspiration because, with linear scanning, it was able to trace the path of the tip of the needle during the puncture process. In addition to being very useful for tissue acquisition for diagnostic purposes, the principles behind endoscopic ultrasound-guided fine needle aspiration paved the way for the development of therapeutic endoscopic ultrasound. Substances could now be delivered by endoscopic ultrasound into targeted areas, an example being an endoscopic ultrasound-guided celiac plexus block and neurolysis. In addition, the endoscopic ultrasound-guided puncture of fluid collections, abscesses and obstructed biliary and pancreatic ductal systems facilitated the passage of guidewires, thus allowing therapeutic drainage procedures to be performed using the Seldinger technique. This review summarizes the diagnostic capability of endoscopic ultrasound and then moves on to elaborate in detail its therapeutic capability and potential.
C1 Changi Gen Hosp, Div Gastroenterol, Dept Med, Singapore 529889, Singapore.
C3 Changi General Hospital
RP Ang, TL (corresponding author), Changi Gen Hosp, Div Gastroenterol, Dept Med, 2 Simei St 3, Singapore 529889, Singapore.
EM tiing_leong_ang@cgh.com.sg
OI Ang, Tiing Leong/0000-0001-9993-8549
CR Ahmad NA, 2001, J CLIN GASTROENTEROL, V32, P54, DOI 10.1097/00004836-200101000-00013
Ang TL, 2007, ALIMENT PHARM THER, V26, P1163, DOI 10.1111/j.1365-2036.2007.03463.x
Ang TL, 2007, RESP MED, V101, P1299, DOI 10.1016/j.rmed.2006.10.013
Ang TL, 2007, J PANCREAS, V8, P438
Ang Tiing Leong, 2006, Chin J Dig Dis, V7, P191, DOI 10.1111/j.1443-9573.2006.00270.x
Ang TL, 2008, GASTROINTEST ENDOSC, V68, P192, DOI 10.1016/j.gie.2007.11.018
Ang TL, 2007, ANN ACAD MED SINGAP, V36, P954
[Anonymous], 2002, AJCC CANC STAG MAN
Artifon ELA, 2007, GASTROINTEST ENDOSC, V66, P1201, DOI 10.1016/j.gie.2007.05.051
Aslanian H, 2005, GASTROINTEST ENDOSC, V62, P723, DOI 10.1016/j.gie.2005.06.048
Baillie J, 2004, GASTROINTEST ENDOSC, V60, P105, DOI 10.1016/S0016-5107(04)01532-9
Baron TH, 2002, GASTROINTEST ENDOSC, V56, P7, DOI 10.1067/mge.2002.125106
Bhandari S, 2004, GASTROINTEST ENDOSC, V59, P619, DOI 10.1016/S0016-5107(04)00169-5
BINMOELLER KF, 1995, GASTROINTEST ENDOSC, V42, P219, DOI 10.1016/S0016-5107(95)70095-1
Bories E, 2007, ENDOSCOPY, V39, P287, DOI 10.1055/s-2007-966212
BOTET JF, 1991, RADIOLOGY, V181, P419, DOI 10.1148/radiology.181.2.1924783

Catalano MF, 1999, GASTROINTEST ENDOSC, V50, P352, DOI 10.1053/ge.1999.v50.98154
Chang KJ, 2006, ENDOSCOPY, V38, pS88, DOI 10.1055/s-2006-946664
Chang KJ, 2000, CANCER-AM CANCER SOC, V88, P1325, DOI 10.1002/(SICI)1097-0142(20000315)88:6<1325::AID-CNCR8>3.0.CO;2-T
Charnley RM, 2006, ENDOSCOPY, V38, P925, DOI 10.1055/s-2006-944731
Collins D, 2006, ENDOSCOPY, V38, P935, DOI 10.1055/s-2006-944734
Eloubeidi MA, 2001, GASTROINTEST ENDOSC, V54, P714, DOI 10.1067/mge.2001.119873
Erickson RA, 2004, GASTROINTEST ENDOSC, V60, P267, DOI 10.1016/S0016-5107(04)01529-9
Farrell JJ, 2006, GASTROINTEST ENDOSC, V63, pAB93, DOI 10.1016/j.gie.2006.03.071
Fockens P, 1997, GASTROINTEST ENDOSC, V46, P412, DOI 10.1016/S0016-5107(97)70033-6
Gan SI, 2007, GASTROINTEST ENDOSC, V66, P425, DOI 10.1016/j.gie.2007.05.026
Giovannini M, 2001, ENDOSCOPY, V33, P898, DOI 10.1055/s-2001-17324
Giovannini M, 2001, ENDOSCOPY, V33, P473, DOI 10.1055/s-2001-14967
Goldberg SN, 1999, GASTROINTEST ENDOSC, V50, P392, DOI 10.1053/ge.1999.v50.98847
Gress F, 2001, AM J GASTROENTEROL, V96, P409
GRIMM H, 1991, GASTROINTEST ENDOSC, V37, pA279
Gunaratnam NT, 2001, GASTROINTEST ENDOSC, V54, P316, DOI 10.1067/mge.2001.117515
Günter E, 2003, GASTROINTEST ENDOSC, V57, P113, DOI 10.1067/mge.2003.39
Hecht JR, 2003, CLIN CANCER RES, V9, P555
Hookey LC, 2006, GASTROINTEST ENDOSC, V63, P635, DOI 10.1016/j.gie.2005.06.028
Jansen JM, 2007, GASTROINTEST ENDOSC, V66, P157, DOI 10.1016/j.gie.2007.02.016
Jürgensen C, 2006, GASTROINTEST ENDOSC, V63, P1059, DOI 10.1016/j.gie.2005.10.034
Kahaleh M, 2006, GASTROINTEST ENDOSC, V64, P52, DOI 10.1016/j.gie.2006.01.063
Kahaleh M, 2006, ENDOSCOPY, V38, P355, DOI 10.1055/s-2006-925249
Kahaleh M, 2007, GASTROINTEST ENDOSC, V65, P224, DOI 10.1016/j.gie.2006.05.008
Kida M, 1998, Endoscopy, V30 Suppl 1, pA64, DOI 10.1055/s-2007-1001474
Kuntz C, 1999, SEMIN SURG ONCOL, V17, P96, DOI 10.1002/(SICI)1098-2388(199909)17:2<96::AID-SSU3>3.3.CO;2-W
Kwan V, 2007, GASTROINTEST ENDOSC, V66, P582, DOI 10.1016/j.gie.2007.02.065
Lee DH, 2006, GASTROINTEST ENDOSC, V64, P631, DOI 10.1016/j.gie.2006.04.031
Lee SS, 2007, GASTROINTEST ENDOSC, V66, P1008, DOI 10.1016/j.gie.2007.03.1080
Legmann P, 1998, AM J ROENTGENOL, V170, P1315, DOI 10.2214/ajr.170.5.9574609
Matthes K, 2007, GASTROINTEST ENDOSC, V65, P448, DOI 10.1016/j.gie.2006.06.030
Micames CG, 2007, CHEST, V131, P539, DOI 10.1378/chest.06-1437
Mortensen MB, 2007, SURG ENDOSC, V21, P431, DOI 10.1007/s00464-006-9029-3
MULLER MF, 1994, RADIOLOGY, V190, P745
Murata Y, 2003, ENDOSCOPY, V35, P429, DOI 10.1055/s-2003-38774
Papachristou GI, 2007, ANN SURG, V245, P943, DOI 10.1097/01.sla.0000254366.19366.69
Park JJ, 2002, GASTROINTEST ENDOSC, V55, P256, DOI 10.1067/mge.2002.120887
Polkowski M, 2004, ENDOSCOPY, V36, P617, DOI 10.1055/s-2004-814522
Preston SR, 2003, BRIT J SURG, V90, P1220, DOI 10.1002/bjs.4268
Püspök A, 2005, AM J GASTROENTEROL, V100, P1743, DOI 10.1111/j.1572-0241.2005.41806.x

ROSCH T, 1992, DEUT MED WOCHENSCHR, V117, P563, DOI 10.1055/s-2008-1062347
ROSCH T, 1991, GASTROINTEST ENDOSC, V37, P347, DOI 10.1016/S0016-5107(91)70729-3
Rosch T, 1995, Gastrointest Endosc Clin N Am, V5, P537
Sahai AV, 1998, GASTROINTEST ENDOSC, V48, P18, DOI 10.1016/S0016-5107(98)70123-3
Sahai AV, 1999, GASTROINTEST ENDOSC, V49, P334, DOI 10.1016/S0016-5107(99)70010-6
Sangro B, 2004, J CLIN ONCOL, V22, P1389, DOI 10.1200/JCO.2004.04.059
Seewald S, 2007, Minerva Med, V98, P305
Seewald S, 2005, GASTROINTEST ENDOSC, V62, P92, DOI 10.1016/S0016-5107(05)00541-9
Seewald S, 2005, GASTROINTEST ENDOSC, V61, P495, DOI 10.1016/S0016-5107(04)02848-2
Seewald S, 2004, GASTROINTEST ENDOSC, V59, P463, DOI 10.1016/S0016-5107(03)02708-1
Seewald S, 2004, GASTROINTEST ENDOSC, V59, P578, DOI 10.1016/S0016-5107(03)02878-5
Seewald S, 2006, GASTROINTEST ENDOSC, V64, P805, DOI 10.1016/j.gie.2006.07.049
SNADY H, 1992, GASTROINTEST ENDOSC, V38, P27, DOI 10.1016/S0016-5107(92)70326-5
Soriano A, 2004, AM J GASTROENTEROL, V99, P492, DOI 10.1111/j.1572-0241.2004.04087.x
Sun S, 2007, ENDOSCOPY, V39, P530, DOI 10.1055/s-2007-966353
Sun S, 2006, ENDOSCOPY, V38, P399, DOI 10.1055/s-2006-925253
Sun SY, 2005, GASTROINTEST ENDOSC, V62, P775, DOI 10.1016/j.gie.2005.06.050
Tessier G, 2007, GASTROINTEST ENDOSC, V65, P233, DOI 10.1016/j.gie.2006.06.029
Varadarajulu S, 2007, GASTROINTEST ENDOSC, V66, P372, DOI 10.1016/j.gie.2007.02.054
Vilmann P, 1998, ENDOSCOPY, V30, P730, DOI 10.1055/s-2007-1001399
Vosoghi Mehrdad, 2002, MedGenMed, V4, P2
WANG HP, 2004, DIGESTIVE ENDOSCOP S, V16, pS157
Wehrmann T, 2005, GASTROINTEST ENDOSC, V62, P344, DOI 10.1016/j.gie.2005.03.001
Wiersema MJ, 1996, GASTROINTEST ENDOSC, V44, P656, DOI 10.1016/S0016-5107(96)70047-0
Wiersema MJ, 1996, GASTROINTEST ENDOSC, V43, P102, DOI 10.1016/S0016-5107(06)80108-2
Will U, 2007, ENDOSCOPY, V39, P292, DOI 10.1055/s-2007-966215
Yovino S, 2007, J GASTROINTEST SURG, V11, P36, DOI 10.1007/s11605-007-0110-6
YUSUF TE, 2008, GASTROINTEST ENDOSC
ZIEGLER K, 1993, GUT, V34, P604, DOI 10.1136/gut.34.5.604
NR 85
TC 5
Z9 10
U1 0
U2 3
PU WILEY-BLACKWELL
PI MALDEN
PA COMMERCE PLACE, 350 MAIN ST, MALDEN 02148, MA USA
SN 1751-2972
J9 J DIGEST DIS
JI J. Dig. Dis.
PD AUG
PY 2008

VL 9
IS 3
BP 117
EP 128
DI 10.1111/j.1751-2980.2008.00333.x
PG 12
WC Gastroenterology & Hepatology
WE Science Citation Index Expanded (SCI-EXPANDED)
SC Gastroenterology & Hepatology
GA 334YL
UT WOS:000258257600001
PM 18956589
OA Bronze
DA 2026-05-09
ER

PT J
AU Tarantino, I
Barresi, L
Fabbri, C
Traina, M
AF Tarantino, Ilaria
Barresi, Luca
Fabbri, Carlo
Traina, Mario
TI Endoscopic ultrasound guided biliary drainage
SO WORLD JOURNAL OF GASTROINTESTINAL ENDOSCOPY
LA English
DT Article
DE Interventional endoscopic ultrasonography; Endoscopic ultrasonography
drainage; Biliary drainage; Endoscopic retrograde
cholangio-pancreatography
AB Endoscopic retrograde cholangio-pancreatography is the most appropriate technique for treating common bile duct and pancreatic duct stenosis secondary to benign and malignant diseases. Even if the procedure is performed by skillful endoscopist, there are patients in whom endoscopic stent placement is not possible. Common causes of failure include complex peripapillary diverticula, prior surgery procedures, tumor involvement of the papilla, biliary sphincter stenosis, and impacted stones. Percutaneous trans-hepatic biliary drainage (PTBD) and surgical intervention carry morbidity and mortality. Recently endoscopic ultrasonography-guided biliary drainage has been reported as an alternative technique. Endoscopic ultrasonography-guided biliary drainage using either direct access or a rendezvous technique has attracted attention as an alternative procedure to PTBD, with a technical success between 75%-100% and with low complication rate. We have reviewed published data on EUS guided biliary drainage procedures with the aim of summarizing the efficacy and safety of this promising method. (C) 2012 Baishideng. All rights reserved.

C1 [Tarantino, Ilaria; Barresi, Luca; Traina, Mario] Univ Pittsburgh, Med Ctr, ISMETT Mediterranean Inst Transplantat & Adv Spe, Unit Gastroenterol & Digest Endoscopy, I-54000 Palermo, Italy.
[Fabbri, Carlo] AUSL Bologna Bellaria Maggiore Hosp, Unit Gastroenterol & Digest Endoscopy, I-40121 Palermo, Italy.
C3 AUSL di Bologna
RP Tarantino, I (corresponding author), Univ Pittsburgh, Med Ctr, ISMETT Mediterranean Inst Transplantat & Adv Spe, Unit Gastroenterol & Digest Endoscopy, I-54000 Palermo, Italy.
EM itarantino@ismett.edu
RI traina, mario/A-1309-2012; Fabbri, Carlo/AAC-4467-2022; Barresi, Luca/B-4622-2013; Tarantino, Ilaria/AFS-0726-2022
CR Ang TL, 2007, J PANCREAS, V8, P438
Artifon ELA, 2007, CLINICS, V62, P799, DOI 10.1590/S1807-59322007000600023
Artifon ELA, 2011, GASTROINTEST ENDOSC, V73, P1317, DOI 10.1016/j.gie.2010.10.041
Artifon ELA, 2010, J PANCREAS, V11, P597
Belletrutti PJ, 2010, J PANCREAS, V11, P234
Bories E, 2007, ENDOSCOPY, V39, P287, DOI 10.1055/s-2007-966212
Burmester E, 2003, GASTROINTEST ENDOSC, V57, P246, DOI 10.1067/mge.2003.85
Cortas GA, 1999, GASTROINTEST ENDOSC, V50, P775, DOI 10.1016/S0016-5107(99)70157-4
Fabbri C, 2011, ENDOSCOPY, V43, P438, DOI 10.1055/s-0030-1256097
FERRUCCI JT, 1980, RADIOLOGY, V135, P1
Fujita N, 2007, WORLD J GASTROENTERO, V13, P5512, DOI 10.3748/wjg.v13.i41.5512
Hanada K, 2009, DIGEST ENDOSC, V21, pS75, DOI 10.1111/j.1443-1661.2009.00855.x
Hara K, 2010, DIGEST ENDOSC, V22, P147, DOI 10.1111/j.1443-1661.2010.00944.x
Itoi T, 2010, J HEPATO-BIL-PAN SCI, V17, P611, DOI 10.1007/s00534-009-0196-1
Iwamuro M, 2010, DIGEST ENDOSC, V22, P236, DOI 10.1111/j.1443-1661.2010.00997.x
Kahaleh M, 2006, GASTROINTEST ENDOSC, V64, P52, DOI 10.1016/j.gie.2006.01.063
Kahaleh M, 2004, GASTROINTEST ENDOSC, V60, P138, DOI 10.1016/S0016-5107(04)01528-7
Kahaleh M, 2005, GASTROINTEST ENDOSC, V61, P307, DOI 10.1016/S0016-5107(04)02585-4
Kim YS, 2010, ENDOSCOPY, V42, P496, DOI 10.1055/s-0029-1244082
Lai LH, 2010, GASTROINTEST ENDOSC, V72, P186, DOI 10.1016/j.gie.2010.01.052
Lai R, 2005, ENDOSCOPY, V37, P487, DOI 10.1055/s-2005-861250
Mallery S, 2004, GASTROINTEST ENDOSC, V59, P100, DOI 10.1016/S0016-5107(03)02300-9
Maranki J, 2009, ENDOSCOPY, V41, P532, DOI 10.1055/s-0029-1214712
MARTIN DF, 1994, GUT, V35, P1011, DOI 10.1136/gut.35.8.1011
Martins FP, 2010, ENDOSCOPY, V42, pE126, DOI 10.1055/s-0029-1243911
Nguyen-Tang T, 2010, ENDOSCOPY, V42, P232, DOI 10.1055/s-0029-1243858
Park DH, 2010, GASTROINTEST ENDOSC, V71, P413, DOI 10.1016/j.gie.2009.10.015
Park DH, 2009, AM J GASTROENTEROL, V104, P2168, DOI 10.1038/ajg.2009.254
Ponchon T, 2002, ENDOSCOPY, V34, P29, DOI 10.1055/s-2002-19397
Püspök A, 2005, AM J GASTROENTEROL, V100, P1743, DOI 10.1111/j.1572-0241.2005.41806.x
Schöfl R, 2001, ENDOSCOPY, V33, P147
Siddiqui AA, 2011, SURG ENDOSC, V25, P549, DOI 10.1007/s00464-010-1216-6

Tarantino I, 2008, ENDOSCOPY, V40, P336, DOI 10.1055/s-2007-995455
VILMANN P, 1992, GASTROINTEST ENDOSC, V38, P172, DOI 10.1016/S0016-5107(92)70385-X
Wiersema MJ, 1996, GASTROINTEST ENDOSC, V43, P102, DOI 10.1016/S0016-5107(06)80108-2
Will U, 2007, ENDOSCOPY, V39, P292, DOI 10.1055/s-2007-966215
Will U, 2007, J HEPATO-BILIARY-PAN, V14, P377, DOI 10.1007/s00534-006-1139-8
Yamao K, 2008, ENDOSCOPY, V40, P340, DOI 10.1055/s-2007-995485
YAMAO K, 2006, SUIZO, V21, P353
Yamao K, 2006, GASTROINTEST ENDOSC, V64, P663, DOI 10.1016/j.gie.2006.07.003
NR 40
TC 15
Z9 16
U1 0
U2 1
PU BAISHIDENG PUBLISHING GROUP INC
PI PLEASANTON
PA 8226 REGENCY DR, PLEASANTON, CA 94588 USA
SN 1948-5190
J9 WORLD J GASTRO ENDOS
JI World J. Gastrointest. Endosc.
PD JUL 16
PY 2012
VL 4
IS 7
BP 306
EP 311
DI 10.4253/wjge.v4.i7.306
PG 6
WC Gastroenterology & Hepatology
WE Emerging Sources Citation Index (ESCI)
SC Gastroenterology & Hepatology
GA V5D6E
UT WOS:000219511500006
PM 22816011
OA Bronze
DA 2026-05-09
ER

PT J
AU Karagyozov, PI
Tishkov, I
Boeva, I
Draganov, K

AF Karagyozov, Petko Ivanov
Tishkov, Ivan
Boeva, Irina
Draganov, Kiril
TI Endoscopic ultrasound-guided biliary drainage-current status and future perspectives
SO WORLD JOURNAL OF GASTROINTESTINAL ENDOSCOPY
LA English
DT Review
DE Endoscopic ultrasound-guided biliary drainage; Malignant bile duct obstruction; Endoscopic ultrasound-guided hepaticogastrostomy; Endoscopic ultrasound-guided rendezvous technique; Endoscopic ultrasound-guided choledochoduodenostomy; Endoscopic ultrasound-guided antegrade stenting; Endoscopic retrograde cholangiopancreatography
ID RETROGRADE CHOLANGIOPANCREATOGRAPHY; OBSTRUCTION; ERCP; CHOLEDOCHODUODENOSTOMY; MANAGEMENT; COMPLICATIONS; PLACEMENT; EFFICACY; ACCESS; TRIAL
AB Endoscopic retrograde cholangiopancreatography (ERCP) with stenting is the treatment modality of choice for patients with benign and malignant bile duct obstruction. ERCP could fail in cases of duodenal obstruction, duodenal diverticulum, ampullary neoplastic infiltration or surgically altered anatomy. In these cases percutaneous biliary drainage (PTBD) is traditionally used as a rescue procedure but is related to high morbidity and mortality and lower quality of life. Endoscopic ultrasound-guided biliary drainage (EUS-BD) is a relatively new interventional procedure that arose due to the development of curvilinear echoendoscope and the various endoscopic devices. A large amount of data is already collected that proves its efficacy, safety and ability to replace PTBD in cases of ERCP failure. It is also possible that EUS-BD could be chosen as a first-line treatment option in some clinical scenarios in the near future. Several EUS-BD techniques are developed EUS-guided transmural stenting, antegrade stenting and rendezvous technique and can be personalized depending on the individual anatomy. EUS-BD is normally performed in the same session from the same endoscopist in case of ERCP failure. The lack of training, absence of enough dedicated devices and lack of standardization still makes EUS-BD a difficult and not very popular procedure, which is related to life-threatening adverse events. Developing training models, dedicated devices and guidelines hopefully will make EUS-BD easier, safer and well accepted in the future. This paper focuses on the technical aspects of the different EUS-BD procedures, available literature data, advantages, negative aspects and the future perspectives of these modalities.
C1 [Karagyozov, Petko Ivanov; Tishkov, Ivan; Boeva, Irina] Tokuda Hopsital, Acibadem City Clin, Clin Gastroenterol, Dept Intervent Gastroenterol, 51B N Vaptzarov Blvd, Sofia 1407, Bulgaria.
[Draganov, Kiril] Tokuda Hosp, Acibadem City Clin, Clin Liver Biliary Pancreat & Gen Surg, Sofia 1407, Bulgaria.
C3 Medical University Sofia
RP Karagyozov, PI (corresponding author), Tokuda Hopsital, Acibadem City Clin, Clin Gastroenterol, Dept Intervent Gastroenterol, 51B N Vaptzarov Blvd, Sofia 1407, Bulgaria.
EM petko.karagyozov@gmail.com
RI Draganov, Kiril/JCP-1124-2023; Karagyozov, Petko/AAA-9580-2019
OI Karagyozov, Petko/0000-0002-2297-547X
CR Andriulli A, 2007, AM J GASTROENTEROL, V102, P1781, DOI 10.1111/j.1572-0241.2007.01279.x

Artifon ELA, 2012, J PANCREAS, V13, P7

Baars JE, 2018, ENDOSC ULTRASOUND, V7, P4, DOI 10.4103/eus.eus_105_17

Bang JY, 2018, GASTROINTEST ENDOSC, V88, P9, DOI 10.1016/j.gie.2018.03.012

Chen YI, 2019, TRIALS, V20, DOI 10.1186/s13063-019-3918-y

Dhir V, 2017, ENDOSC INT OPEN, V5, pE1087, DOI 10.1055/s-0043-118097

Dhir V, 2015, GASTROINTEST ENDOSC, V81, P913, DOI 10.1016/j.gie.2014.09.054

El Chafic Abdul H, 2020, Curr Gastroenterol Rep, V22, P62, DOI [10.1007/s11894-020-00800-3, 10.1007/s11894-020-00800-3]

Giovannini M, 2001, ENDOSCOPY, V33, P898, DOI 10.1055/s-2001-17324

Giovannini M, 2012, GASTROENT RES PRACT, V2012, DOI 10.1155/2012/348719

Han SY, 2019, SCI REP-UK, V9, DOI 10.1038/s41598-019-52993-x

Hathorn KE, 2019, ENDOSC INT OPEN, V7, pE1432, DOI 10.1055/a-0990-9488

Itoi T, 2008, WORLD J GASTROENTERO, V14, P6078, DOI 10.3748/wjg.14.6078

Iwashita T, 2014, CLIN J GASTROENTEROL, V7, P94, DOI 10.1007/s12328-014-0467-5

Jin Z, 2020, DIGEST ENDOSC, V32, P16, DOI 10.1111/den.13456

Kakked G, 2020, ENDOSC ULTRASOUND, V9, P298, DOI 10.4103/eus.eus_10_20

Kawakubo K, 2016, ENDOSCOPY, V48, P164, DOI 10.1055/s-0034-1393179

Khan MA, 2016, DIGEST DIS SCI, V61, P684, DOI 10.1007/s10620-015-3933-0

Khashab MA, 2016, ENDOSC INT OPEN, V4, pE175, DOI 10.1055/s-0041-109083

Khashab MA, 2015, DIGEST DIS SCI, V60, P557, DOI 10.1007/s10620-014-3300-6

Logiudice FP, 2019, WORLD J GASTRO ENDOS, V11, P281, DOI 10.4253/wjge.v11.i4.281

Lou X, 2019, MINERVA MED, V110, P564, DOI 10.23736/S0026-4806.19.05981-0

Minaga K, 2020, THER ADV GASTROENTER, V13, DOI 10.1177/1756284820930964

Mukai S, 2019, ENDOSC ULTRASOUND, V8, pS7, DOI 10.4103/eus.eus_46_19

Nennstiel S, 2015, J CLIN GASTROENTEROL, V49, P764, DOI 10.1097/MCG.0000000000000275

Nguyen-Tang T, 2010, ENDOSCOPY, V42, P232, DOI 10.1055/s-0029-1243858

Nunes N, 2021, GE PORT J GASTROENT, V28, P179, DOI 10.1159/000510026

Oh D, 2017, THER ADV GASTROENTER, V10, P42, DOI 10.1177/1756283X16671671

Paik WH, 2018, AM J GASTROENTEROL, V113, P987, DOI 10.1038/s41395-018-0122-8

Paik WH, 2017, CLIN ENDOSC, V50, P104, DOI 10.5946/ce.2017.036

Park DH, 2015, J HEPATO-BIL-PAN SCI, V22, P664, DOI 10.1002/jhbp.271

Park JK, 2018, GASTROINTEST ENDOSC, V88, P277, DOI 10.1016/j.gie.2018.03.015

Peng CY, 2013, BMC GASTROENTEROL, V13, DOI 10.1186/1471-230X-13-147

Saad WEA, 2010, J VASC INTERV RADIOL, V21, P789, DOI 10.1016/j.jvir.2010.01.012

Salerno R, 2019, WORLD J GASTRO ENDOS, V11, P354, DOI 10.4253/wjge.v11.i5.354

Sarkaria S, 2013, GUT LIVER, V7, P129, DOI 10.5009/gnl.2013.7.2.129

Sharaiha RZ, 2017, GASTROINTEST ENDOSC, V85, P904, DOI 10.1016/j.gie.2016.12.023

Teoh AYB, 2018, GUT, V67, P1209, DOI 10.1136/gutjnl-2017-314341

NR 38

TC 10

Z9 11

U1 0

U2 7
PU BAISHIDENG PUBLISHING GROUP INC
PI PLEASANTON
PA 7041 Koll Center Parkway, Suite 160, PLEASANTON, CA, UNITED STATES
SN 1948-5190
J9 WORLD J GASTRO ENDOS
JI World J. Gastrointest. Endosc.
PD DEC 16
PY 2021
VL 13
IS 12
BP 607
EP 618
DI 10.4253/wjge.v13.i12.607
PG 12
WC Gastroenterology & Hepatology
WE Emerging Sources Citation Index (ESCI)
SC Gastroenterology & Hepatology
GA YJ6VR
UT WOS:000744670400003
PM 35070022
OA Green Accepted, gold
DA 2026-05-09
ER

PT J
AU Seicean, A
Pojoga, C
Rednic, V
Hagiu, C
Seicean, R
AF Seicean, Andrada
Pojoga, Cristina
Rednic, Voicu
Hagiu, Claudia
Seicean, Radu
TI Endoscopic ultrasound drainage of pancreatic fluid collections: do we
know enough about the best approach?
SO THERAPEUTIC ADVANCES IN GASTROENTEROLOGY
LA English
DT Review
DE endoscopic ultrasound; drainage; LAMS; pancreatic fluid collections;

plastic stents; pseudocysts; walled-off necrosis

ID WALLED-OFF NECROSIS; APPOSING METAL STENTS; EUS-GUIDED DRAINAGE; PIGTAIL PLASTIC STENTS; STEP-UP APPROACH; NECROTIZING PANCREATITIS; TRANSMURAL DRAINAGE; CLINICAL-OUTCOMES; EUROPEAN-SOCIETY; MANAGEMENT

AB Plain language summaryEndoscopic ultrasound drainage of pancreatic fluid collectionsThis review summarizes the current knowledge on the indications, techniques, outcomes, complications, and treatment-associated EUS drainage of pancreatic fluid collections. Special attention is paid to the practical approach in necrosectomy and postprocedural antibiotherapy, discontinuation of proton pump therapy, and timing of stent removal.

Pancreatic fluid collection often occurs as a local complication of acute pancreatitis, and drainage is indicated in symptomatic patients. The drainage may be surgical, percutaneous, or endoscopic ultrasound (EUS) guided. In symptomatic collections older than 4 weeks and localized in the upper abdomen, EUS-guided drainage is the first choice of treatment. Lumen-apposing metal stents are useful in cases of walled-off necrosis, facilitating access to the cavity; however, they do not reduce the number of necrosectomy sessions required. In most pancreatic pseudocysts requiring drainage, plastic stents remain the first choice of treatment. This review aimed to summarize the principles and techniques of step-up therapy of pancreatic fluid collections, including preprocedural and postprocedural assessment and practical approaches of drainage and necrosectomy, making available evidence more accessible to endoscopists aiming to train for this procedure. Successful and safe EUS drainage connotes early recognition and treatment of complications and the presence of a multidisciplinary team for optimal patient management. However, the best time for necrosectomy, modality of drainage method (lumen-apposing metal stents or plastic stents), and duration of antibiotherapy are still under evaluation.

C1 [Pojoga, Cristina] Babes Bolyai Univ, UBBMed, Cluj Napoca, Romania.

[Seicean, Andrada; Pojoga, Cristina; Rednic, Voicu; Hagiu, Claudia] Reg Inst Gastroenterol & Hepatol Prof Dr Octavian, Dept Gastroenterol, Cluj Napoca, Romania.

[Seicean, Andrada; Hagiu, Claudia] Iuliu Hatieganu Univ Med & Pharm, Cluj Napoca, Romania.

[Seicean, Radu] Iuliu Hatieganu Univ Med & Pharm, Dept Surg 1, Cluj Napoca, Romania.

C3 Babes Bolyai University from Cluj; Regional Institute of Gastroenterology & Hepatology; Iuliu Hatieganu University of Medicine & Pharmacy; Iuliu Hatieganu University of Medicine & Pharmacy

RP Pojoga, C (corresponding author), Babes Bolyai Univ, UBBMed, Cluj Napoca, Romania.; Pojoga, C (corresponding author), Reg Inst Gastroenterol & Hepatol Prof Dr Octavian, Dept Gastroenterol, Cluj Napoca, Romania.

EM cristinapojoga@yahoo.com

RI Seicean, Anada/C-4057-2011; Seicean, Radu/IUP-6041-2023; Pojoga, Cristina/JPK-8791-2023

OI Seicean, Anada/0000-0002-5809-1334;

CR Aburajab M, 2018, GASTROINTEST ENDOSC, V87, P1248, DOI 10.1016/j.gie.2017.11.033

Ahmad W, 2020, SCAND J GASTROENTERO, V55, P242, DOI 10.1080/00365521.2019.1710246

Ahmed A, 2016, DIGEST SURG, V33, P259, DOI 10.1159/000445005

Anderloni A, 2021, SURG ENDOSC, V35, P1741, DOI 10.1007/s00464-020-07567-8

Angadi S, 2021, PANCREATOLOGY, V21, P1291, DOI 10.1016/j.pan.2021.06.006

Arvanitakis M, 2018, ENDOSCOPY, V50, P524, DOI 10.1055/a-0588-5365

Bang JY, 2022, DIGEST ENDOSC, V34, P612, DOI 10.1111/den.14099

Bang JY, 2019, GASTROENTEROLOGY, V156, P1027, DOI 10.1053/j.gastro.2018.11.031

Bang JY, 2017, DIGEST ENDOSC, V29, P83, DOI 10.1111/den.12681

Banks PA, 2013, GUT, V62, P102, DOI 10.1136/gutjnl-2012-302779
Bapaye A, 2017, DIGEST ENDOSC, V29, P104, DOI 10.1111/den.12704
Baron TH., 2020, Gastroenterology, V75
Bazerbachi F, 2018, GASTROINTEST ENDOSC, V87, P30, DOI 10.1016/j.gie.2017.08.025
Bekkali N., 2017, Endosc Int Open, V05
Besselink Marc G H, 2006, BMC Surg, V6, P6, DOI 10.1186/1471-2482-6-6
Bhargava MV, 2022, DIGEST DIS SCI, V67, P4146, DOI 10.1007/s10620-021-07201-0
Bhargava V, 2021, SURGERY, V170, P1532, DOI 10.1016/j.surg.2021.05.028
Bhatia H, 2022, EXPERT REV GASTROENT, V16, P121, DOI 10.1080/17474124.2022.2031978
Binda C, 2020, EUR J GASTROEN HEPAT, V32, P1401, DOI 10.1097/MEG.0000000000001793
Boxhoorn L, 2021, NEW ENGL J MED, V385, P1372, DOI 10.1056/NEJMoa2100826
Boxhoorn L, 2023, GUT, V72, P66, DOI 10.1136/gutjnl-2021-325632
Brand M, 2022, WORLD J GASTRO SURG, V14, P799, DOI 10.4240/wjgs.v14.i8.799
Brimhall B, 2018, CLIN GASTROENTEROL H, V16, P1521, DOI 10.1016/j.cgh.2018.02.021
Chandrasekhara V., 2020, Endosc Int Open, V8
Chandrasekhara V, 2021, CLIN GASTROENTEROL H, V19, P2192, DOI 10.1016/j.cgh.2021.05.005
Chavan R, 2022, ENDOSCOPY, V54, P861, DOI 10.1055/a-1747-3283
Chen YI, 2018, ENDOSC INT OPEN, V6, pE780, DOI 10.1055/a-0611-5082
Chen YI., 2019, Endosc Int Open, V7
Chong E, 2021, BMC GASTROENTEROL, V21, DOI 10.1186/s12876-021-01663-2
Dhar J, 2022, ENDOSCOPY, V54, P728, DOI 10.1055/a-1823-0974
Dhir V, 2018, ENDOSCOPY, V50, P22, DOI [10.1055/s-0043-118592, 10.1055/s-0043-123575]
Ding L, 2022, J DIGEST DIS, V23, P174, DOI 10.1111/1751-2980.13083
Facciorusso A, 2022, DIGEST ENDOSC, V34, P1459, DOI 10.1111/den.14354
Fugazza A, 2020, GASTROINTEST ENDOSC, V91, P574, DOI 10.1016/j.gie.2019.11.021
Gardner TB, 2011, GASTROINTEST ENDOSC, V73, P718, DOI 10.1016/j.gie.2010.10.053
Ge PS, 2020, PANCREAS, V49, P236, DOI 10.1097/MPA.0000000000001476
Giovannini M, 2021, ENDOSC ULTRASOUND, V10, P317, DOI 10.4103/EUS-D-21-00205
Gkolfakis P, 2022, ANN GASTROENTEROL, V35, P654, DOI 10.20524/aog.2022.0753
Gkolfakis P, 2021, ENDOSCOPY, V53, P1141, DOI 10.1055/a-1319-5093
Guo JT, 2020, SURG ENDOSC, V34, P1177, DOI 10.1007/s00464-019-06870-3
Guo JT, 2017, ENDOSC ULTRASOUND, V6, P285, DOI 10.4103/eus.eus_85_17
Guzmán-Calderón E, 2022, J HEPATO-BIL-PAN SCI, V29, P198, DOI 10.1002/jhbp.1008
Hamada T, 2022, DIGEST ENDOSC, V34, P676, DOI 10.1111/den.14142
Hentschel V, 2022, ANTIBIOTICS-BASEL, V11, DOI 10.3390/antibiotics11030420
Holmes I, 2022, GASTROINTEST ENDOSC, V95, P482, DOI 10.1016/j.gie.2021.10.015
Isayama H, 2016, J GASTROEN HEPATOL, V31, P1546, DOI 10.1111/jgh.13394
Jagielski M, 2022, FRONT CELL INFECT MI, V12, DOI 10.3389/fcimb.2022.939138
Johnson G, 2021, ENDOSCOPY, V53, P1071, DOI 10.1055/a-1537-8999
Khan S, 2021, J GASTROEN HEPATOL, V36, P3395, DOI 10.1111/jgh.15658
Laique S, 2019, WORLD J GASTRO ENDOS, V11, P403, DOI 10.4253/wjge.v11.i6.403
Lariño-Noia J, 2021, SURG ENDOSC, V35, P644, DOI 10.1007/s00464-020-07428-4

Law RJ, 2021, GASTROINTEST ENDOSC, V94, P457, DOI 10.1016/j.gie.2021.05.020
Law ST, 2018, SURG ENDOSC, V32, P2448, DOI 10.1007/s00464-017-5946-6
Li J, 2021, ABDOM RADIOL, V46, P776, DOI 10.1007/s00261-020-02696-z
Liao Q, 2022, EUR J RADIOL, V154, DOI 10.1016/j.ejrad.2022.110419
Manrai M, 2018, ANN SURG, V267, P357, DOI 10.1097/SLA.0000000000002065
Messallam AA, 2021, AM J GASTROENTEROL, V116, P700, DOI 10.14309/ajg.0000000000000987
Mohan BP, 2022, GASTROINTEST ENDOSC, V95, P1060, DOI 10.1016/j.gie.2022.01.018
Mukai S, 2021, DIGEST ENDOSC, V33, P985, DOI 10.1111/den.14016
Mukai S, 2015, J GASTROENTEROL, V50, P211, DOI 10.1007/s00535-014-0957-8
Nakai Y, 2023, DEN OPEN, V3, DOI 10.1002/deo2.171
Onnekink AM, 2022, GASTROENTEROLOGY, V163, P712, DOI 10.1053/j.gastro.2022.05.015
Parsa N, 2020, ENDOSCOPY, V52, P211, DOI 10.1055/a-1096-3299
Patra PS, 2014, BRIT J SURG, V101, P1721, DOI 10.1002/bjs.9666
Pawar A., 2021, CLIN ENDOSC, V54, P909
Powers PC, 2019, ENDOSC ULTRASOUND, V8, P194, DOI 10.4103/eus.eus_59_18
Puga M, 2018, ENDOSCOPY, V50, P1022, DOI 10.1055/a-0582-9127
Ramai D, 2023, GASTROINTEST ENDOSC, V97, P5
Rana SS, 2022, ENDOSCOPY, V54, P920, DOI 10.1055/a-1857-5600
Rana SS, 2016, GASTROENTEROL REP, V4, P50, DOI 10.1093/gastro/gou088
Rana SS, 2020, ENDOSC ULTRASOUND, V9, P130, DOI 10.4103/eus.eus_8_20
Rana SS, 2020, PANCREATOLOGY, V20, P177, DOI 10.1016/j.pan.2019.12.011
Sahar N, 2018, J GASTROEN HEPATOL, V33, P1548, DOI 10.1111/jgh.14111
Samanta J, 2022, EXPERT REV GASTROENT, V16, P297, DOI 10.1080/17474124.2022.2047649
Seicean A, 2020, J GASTROINTEST LIVER, V29, P623, DOI 10.15403/jgld-3128
Shamah SP, 2022, ENDOSC ULTRASOUND, V11, P53, DOI 10.4103/EUS-D-21-00030
Siddiqui A, 2021, ANN GASTROENTEROL, V34, P273, DOI 10.20524/aog.2021.0570
Siddiqui AA, 2017, GASTROINTEST ENDOSC, V85, P758, DOI 10.1016/j.gie.2016.08.014
Siddiqui AA, 2013, GASTROINTEST ENDOSC, V78, P589, DOI 10.1016/j.gie.2013.03.1337
Stassen PMC., 2022, Gastrointest Endosc, V95
Staudenmann D, 2022, ENDOSC ULTRASOUND, V11, P147, DOI 10.4103/EUS-D-21-00054
Teoh AYB, 2018, GUT, V67, P1209, DOI 10.1136/gutjnl-2017-314341
Trikudanathan G, 2018, AM J GASTROENTEROL, V113, P1550, DOI 10.1038/s41395-018-0232-3
Tsujimae M, 2023, SURG ENDOSC, V37, P1096, DOI 10.1007/s00464-022-09610-2
van Brunschot S, 2018, LANCET, V391, P51, DOI 10.1016/S0140-6736(17)32404-2
van Santvoort HC, 2010, NEW ENGL J MED, V362, P1491, DOI 10.1056/NEJMoa0908821
van Wanrooij RLJ, 2022, ENDOSCOPY, V54, P310, DOI 10.1055/a-1738-6780
Varadarajulu S., 2013, Gastroenterology, V590
Varadarajulu S, 2008, GASTROINTEST ENDOSC, V68, P1102, DOI 10.1016/j.gie.2008.04.028
Varadarajulu S, 2011, J GASTROINTEST SURG, V15, P2080, DOI 10.1007/s11605-011-1621-8
Varadarajulu S, 2011, GASTROINTEST ENDOSC, V74, P74, DOI 10.1016/j.gie.2011.03.1122
Veitch AM, 2021, GUT, V70, P1611, DOI 10.1136/gutjnl-2021-325184
Yan L, 2019, ENDOSC ULTRASOUND, V8, P172, DOI 10.4103/eus.eus_108_17

Yao YX, 2019, MEDICINE, V98, DOI 10.1097/MD.0000000000014348
Yasuda I, 2013, ENDOSCOPY, V45, P627, DOI 10.1055/s-0033-1344027
Zhai YQ., 2022, Gastrointest Endosc, V529
Zhang LY, 2022, ENDOSCOPY, V54, P706, DOI 10.1055/a-1682-7095
NR 97
TC 5
Z9 7
U1 2
U2 4
PU SAGE PUBLICATIONS LTD
PI LONDON
PA 1 OLIVERS YARD, 55 CITY ROAD, LONDON EC1Y 1SP, ENGLAND
SN 1756-283X
EI 1756-2848
J9 THER ADV GASTROENTER
JI Ther. Adv. Gastroenterol.
PD JUL
PY 2023
VL 16
AR 17562848231180047
DI 10.1177/17562848231180047
PG 20
WC Gastroenterology & Hepatology
WE Science Citation Index Expanded (SCI-EXPANDED)
SC Gastroenterology & Hepatology
GA M0WU1
UT WOS:001027439200001
PM 37485492
OA Green Submitted, gold
DA 2026-05-09
ER

PT J
AU van Wanrooij, RLJ
Bronswijk, M
Kunda, R
Everett, SM
Lakhtakia, S
Rimbas, M
Hucl, T
Badaoui, A
Law, R

Arcidiacono, PG
Larghi, A
Giovannini, M
Khashab, MA
Binmoeller, KF
Barthet, M
Pérez-Miranda, M
van Hooft, JE
van der Merwe, SW
AF van Wanrooij, Roy L. J.
Bronswijk, Michiel
Kunda, Rastislav
Everett, Simon M.
Lakhtakia, Sundeep
Rimbas, Mihai
Hucl, Tomas
Badaoui, Abdenor
Law, Ryan
Arcidiacono, Paolo Giorgio
Larghi, Alberto
Giovannini, Marc
Khashab, Mouen A.
Binmoeller, Kenneth F.
Barthet, Marc
Perez-Miranda, Manuel
van Hooft, Jeanin E.
van der Merwe, Schalk W.
TI Therapeutic endoscopic ultrasound: European Society of Gastrointestinal Endoscopy (ESGE) Technical Review
SO ENDOSCOPY
LA English
DT Review
ID GUIDED GALLBLADDER DRAINAGE; DIRECTED TRANSGASTRIC ERCP; PANCREATIC-DUCT DRAINAGE; LUMEN-APPOSING STENT; LONG-TERM OUTCOMES; Y GASTRIC BYPASS; EXPANDABLE METAL STENT; BILIARY DRAINAGE; ADVERSE EVENTS; PERCUTANEOUS CHOLECYSTOSTOMY
AB Main Recommendations 1 ESGE recommends a prolonged course of a prophylactic broad-spectrum antibiotic in patients with ascites who are undergoing therapeutic endoscopic ultrasound (EUS) procedures. Strong recommendation, low quality evidence. 2 ESGE recommends placement of partially or fully covered self-expandable metal stents during EUS-guided hepaticogastrostomy for biliary drainage in malignant disease. Strong recommendation, moderate quality evidence. 3 ESGE recommends EUS-guided pancreatic duct (PD) drainage should only be performed in high volume expert centers, owing to the complexity of this technique and the high risk of adverse events. Strong recommendation, low quality

evidence. 4 ESGE recommends a stepwise approach to EUS-guided PD drainage in patients with favorable anatomy, starting with rendezvous-assisted endoscopic retrograde pancreatography (RV-ERP), followed by antegrade or transmural drainage only when RV-ERP fails or is not feasible. Strong recommendation, low quality evidence. 5 ESGE suggests performing transduodenal EUS-guided gallbladder drainage with a lumen-apposing metal stent (LAMS), rather than using the transgastric route, as this may reduce the risk of stent dysfunction. Weak recommendation, low quality evidence. 6 ESGE recommends using saline instillation for small-bowel distension during EUS-guided gastroenterostomy. Strong recommendation, low quality evidence. 7 ESGE recommends the use of saline instillation with a 19G needle and an electrocautery-enhanced LAMS for EUS-directed transgastric endoscopic retrograde cholangiopancreatography (EDGE) procedures. Strong recommendation, low quality evidence. 8 ESGE recommends the use of either 15- or 20-mm LAMSs for EDGE, with a preference for 20-mm LAMSs when considering a same-session ERCP. Strong recommendation, low quality evidence.
C1 [van Wanrooij, Roy L. J.] Vrije Univ Amsterdam, Amsterdam UMC, AGEM Inst, Dept Gastroenterol & Hepatol, Amsterdam, Netherlands.

[Bronswijk, Michiel] Imelda Gen Hosp, Dept Gastroenterol & Hepatol, Bonheiden, Belgium.

[Bronswijk, Michiel; van der Merwe, Schalk W.] Univ Hospitals Leuven, Dept Gastroenterol & Hepatol, Leuven, Belgium.

[Kunda, Rastislav] Vrije Univ Brussel, Dept Surg, Dept Gastroenterol & Hepatol, Brussels, Belgium.

[Kunda, Rastislav] Vrije Univ Brussel, Univ Ziekenhuis Brussel, Dept Adv Intervent Endoscopy, Brussels, Belgium.

[Everett, Simon M.] Leeds Teaching Hosp NHS Trust, Dept Gastroenterol & Hepatol, Leeds, W Yorkshire, England.

[Lakhtakia, Sundeep] Asian Inst Gastroenterol Hosp, Dept Med Gastroenterol, Hyderabad, India.

[Rimbas, Mihai] Colentina Clin Hosp, Gastroenterol Dept, Bucharest, Romania.

[Hucl, Tomas] Inst Clin & Expt Med, Dept Gastroenterol & Hepatol, Prague, Czech Republic.

[Badaoui, Abdenor] Univ Catholic Louvain, Dept Gastroenterol & Hepatol, CHU UCL Namur, Yvoir, Belgium.

[Law, Ryan] Mayo Clin, Dept Gastroenterol & Hepatol, Rochester, MN 55905 USA.

[Arcidiacono, Paolo Giorgio] IRCCS San Raffaele Sci Inst, Pancreatobil Endoscopy & Endosonog Div, Milan, Italy.

[Larghi, Alberto] Catholic Univ, Fdn Policlin Univ A Gemelli IRCCS, Univ Cattolica Sacro Cuore, Digest Endoscopy Unit, Rome, Italy.

[Larghi, Alberto] Catholic Univ, Ctr Endoscop Res Therapeut & Training, Rome, Italy.

[Giovannini, Marc] Paoli Calmettes Inst, Dept Gastrointestinal Dis, Marseille, France.

[Khashab, Mouen A.] Johns Hopkins Univ Hosp, Div Gastroenterol & Hepatol, Baltimore, MD USA.

[Binmoeller, Kenneth F.] Calif Pacific Med Ctr, Intervent Endoscopy Serv, San Francisco, CA USA.

[Barthet, Marc] Aix Marseille Univ, Hop Nord, APHM, Dept Gastroenterol, Marseille, France.

Hosp Univ Rio Hortega, Dept Gastroenterol, Valladolid, Spain.

Leiden Univ, Med Ctr, Dept Gastroenterol & Hepatol, Leiden, Netherlands.

C3 University of Amsterdam; Vrije Universiteit Amsterdam; Imeldaziekenhuis;
Vrije Universiteit Brussel; University Hospital Brussels; Vrije
Universiteit Brussel; University of Leeds; Institute for Clinical &
Experimental Medicine (IKEM); Universite Catholique Louvain; Mayo
Clinic; Vita-Salute San Raffaele University; IRCCS Ospedale San
Raffaele; Catholic University of the Sacred Heart; IRCCS Policlinico
Gemelli; Catholic University of the Sacred Heart; IRCCS Policlinico
Gemelli; UNICANCER; Institut Paoli-Calmette (IPC); Johns Hopkins

University; Johns Hopkins Medicine; California Pacific Medical Center;
Aix-Marseille Universite; Assistance Publique-Hopitaux de Marseille;
Hospital del Rio Hortega; Leiden University - Excl LUMC; Leiden
University; Leiden University Medical Center (LUMC)
RP van der Merwe, SW (corresponding author), Univ Hospitals Leuven, Dept Gastroenterol & Hepatol, Leuven, Belgium.
RI Kunda, Rastislav/N-1775-2019; Arcidiacono, Paolo Giorgio/K-2122-2018;
van Hooft, Jeanin/AAT-3600-2020; khashab, mouen/F-6643-2017; Larghi,
Alberto/AAC-7448-2022; Rimbas, Mihai/AAF-5521-2020
OI Van der Merwe, Schalk/0000-0002-9891-2686; Arcidiacono, Paolo
Giorgio/0000-0001-6692-7720; Bronswijk, Michiel/0000-0003-2039-5022; van
Hooft, Jeanin/0000-0002-4424-0079; Badaoui, Abdenor/0009-0002-1202-7967;
van Wanrooij, Roy L.J/0000-0003-0477-4532;
CR Adler DG, 2018, GASTROINTEST ENDOSC, V87, P617, DOI 10.1016/j.gie.2017.08.007
Ahmed O, 2018, SURG ENDOSC, V32, P1627, DOI 10.1007/s00464-018-6041-3
Amato A, 2021, ENDOSCOPY, V53, P1037, DOI 10.1055/a-1324-7919
Amin Sunil, 2017, Gastrointest Endosc Clin N Am, V27, P707, DOI [10.1016/j.giec.2017.06.009,
10.1016/j.giec.2017.06.009]
Anderloni A, 2017, ENDOSC INT OPEN, V5, pE1100, DOI 10.1055/s-0043-118828
Anderloni A, 2017, ENDOSC INT OPEN, V5, pE25, DOI 10.1055/s-0042-122009
Anderloni A, 2016, SURG ENDOSC, V30, P5200, DOI 10.1007/s00464-016-4894-x
Arvanitakis M, 2018, ENDOSCOPY, V50, P524, DOI 10.1055/a-0588-5365
Attam R, 2015, ENDOSCOPY, V47, P647, DOI 10.1055/s-0034-1391124
Attam R, 2011, GASTROINTEST ENDOSC, V74, P677, DOI 10.1016/j.gie.2011.05.018
Attardo S, 2019, ENDOSCOPY, V51, pE118, DOI 10.1055/a-0836-2447
Attasaranya S, 2012, GASTROENT RES PRACT, V2012, DOI 10.1155/2012/680753
Bang JY, 2018, GASTROINTEST ENDOSC, V88, P9, DOI 10.1016/j.gie.2018.03.012
Barthet M, 2015, GASTROINTEST ENDOSC, V81, P215, DOI 10.1016/j.gie.2014.09.039
Basiliya K, 2021, ENDOSCOPY, V53, P266, DOI 10.1055/a-1200-0199
Berríos SI, 2017, JAMA SURG, V152, P784, DOI 10.1001/jamasurg.2017.0904
Bronswijk M, 2021, GASTROINTEST ENDOSC, V94, P526, DOI 10.1016/j.gie.2021.04.006
Bronswijk Michiel, 2020, VideoGIE, V5, P442, DOI [10.1016/j.vgie.2020.06.012, 10.1016/j.vgie.2020.06.012]
Bukhari M, 2018, GASTROINTEST ENDOSC, V88, P486, DOI 10.1016/j.gie.2018.04.2356
Cahen DL, 2007, NEW ENGL J MED, V356, P676, DOI 10.1056/NEJMoa060610
Chan SM, 2017, GASTROINTEST ENDOSC, V85, P1225, DOI 10.1016/j.gie.2016.10.014
Chang JI, 2019, ENDOSC INT OPEN, V7, pE655, DOI 10.1055/a-0826-4309
Chantarojanasiri T, 2021, CLIN ENDOSC, V54, P301
Chavan Radhika, 2020, VideoGIE, V5, P64, DOI [10.1016/j.vgie.2019.10.003, 10.1016/j.vgie.2019.10.003]
Chen YI, 2018, GASTROINTEST ENDOSC, V87, P1215, DOI 10.1016/j.gie.2017.07.030
Chen YI, 2018, ENDOSC INT OPEN, V6, pE363, DOI 10.1055/s-0043-123468
Chen YI, 2017, SURG ENDOSC, V31, P2946, DOI 10.1007/s00464-016-5311-1
Chen YI, 2017, GASTROINTEST ENDOSC, V85, P170, DOI 10.1016/j.gie.2016.07.031
Cho SH, 2020, GASTROINTEST ENDOSC, V91, P543, DOI 10.1016/j.gie.2019.09.042

Choi JH, 2014, ENDOSCOPY, V46, P656, DOI 10.1055/s-0034-1365720
Crismale James F, 2016, VideoGIE, V1, P61, DOI [10.1016/j.vgie.2016.09.002, 10.1016/j.vgie.2016.09.002]
Sanz MD, 2021, SURG ENDOSC, V35, P6754, DOI 10.1007/s00464-020-08179-y
De Cassan C, 2017, ENDOSC ULTRASOUND, V6, P329, DOI 10.4103/2303-9027.209869
Dollhopf M, 2017, GASTROINTEST ENDOSC, V86, P636, DOI 10.1016/j.gie.2017.02.027
Duloy Anna, 2019, VideoGIE, V4, P508, DOI [10.1016/j.vgie.2019.07.012, 10.1016/j.vgie.2019.07.012]
Dumonceau JM, 2019, ENDOSCOPY, V51, P179, DOI 10.1055/a-0822-0832
François E, 2002, GASTROINTEST ENDOSC, V56, P128, DOI 10.1067/mge.2002.125547
Fujii LL, 2013, GASTROINTEST ENDOSC, V78, P854, DOI 10.1016/j.gie.2013.05.016
Garcia-Sumalla Albert, 2021, VideoGIE, V6, P263, DOI [10.1016/j.vgie.2021.03.002, 10.1016/j.vgie.2021.03.002]
Garcia-Sumalla A, 2021, SURG ENDOSC, V35, P4873, DOI 10.1007/s00464-021-08435-9
Ge N, 2016, BMC GASTROENTEROL, V16, DOI 10.1186/s12876-016-0420-9
Ge PS, 2019, SURG ENDOSC, V33, P3404, DOI 10.1007/s00464-018-06636-3
Ghandour B, 2022, GASTROINTEST ENDOSC, V95, P80, DOI 10.1016/j.gie.2021.07.023
Guyatt GH, 2011, J CLIN EPIDEMIOL, V64, P1311, DOI [10.1016/j.jclinepi.2011.03.017, 10.1016/j.jclinepi.2011.06.004]
Hassan C, 2020, ENDOSCOPY, V52, P123, DOI 10.1055/a-1067-4657
Higa JT, 2019, GASTROINTEST ENDOSC, V90, P483, DOI 10.1016/j.gie.2019.04.238
Hu JL, 2020, SCAND J GASTROENTERO, V55, P865, DOI 10.1080/00365521.2020.1778077
Ichkhanian Y, 2021, SURG ENDOSC, V35, P1755, DOI 10.1007/s00464-020-07570-z
Ichkhanian Yervant, 2020, VideoGIE, V5, P260, DOI [10.1016/j.vgie.2020.02.005, 10.1016/j.vgie.2020.02.005]
Ichkhanian Y, 2020, GASTROINTEST ENDOSC, V91, P1188, DOI 10.1016/j.gie.2019.12.043
Imai H, 2016, GASTROINTEST ENDOSC, V84, P147, DOI 10.1016/j.gie.2015.12.024
Iqbal U, 2020, ENDOSC ULTRASOUND, V9, P16, DOI 10.4103/eus.eus_70_19
Irani Shayan, 2020, VideoGIE, V5, P586, DOI [10.1016/j.vgie.2020.07.017, 10.1016/j.vgie.2020.07.017]
Irani Shayan, 2020, VideoGIE, V5, P125, DOI [10.1016/j.vgie.2019.12.005, 10.1016/j.vgie.2019.12.005]
Irani Shayan, 2018, VideoGIE, V3, P322, DOI [10.1016/j.vgie.2018.07.008, 10.1016/j.vgie.2018.07.008]
Irani S, 2015, GASTROINTEST ENDOSC, V82, P1110, DOI 10.1016/j.gie.2015.05.045
Issa D, 2021, ENDOSCOPY, V53, P827, DOI 10.1055/a-1259-0349
Itoi T, 2017, DIGEST ENDOSC, V29, P495, DOI 10.1111/den.12794
Itoi T, 2016, GUT, V65, P193, DOI 10.1136/gutjnl-2015-310348
Itoi T, 2013, DIGEST ENDOSC, V25, P241, DOI 10.1111/den.12048
Itonaga M, 2019, DIGEST ENDOSC, V31, P291, DOI 10.1111/den.13300
Iwashita T, 2012, ENDOSCOPY, V44, P60, DOI 10.1055/s-0030-1256871
Iwashita T, 2016, GASTROINTEST ENDOSC, V83, P394, DOI 10.1016/j.gie.2015.04.043
James TW, 2020, GASTROINTEST ENDOSC, V91, P537, DOI 10.1016/j.gie.2019.11.017
James TW, 2019, ENDOSC INT OPEN, V7, pE1099, DOI 10.1055/a-0965-6662
James TW, 2019, OBES SURG, V29, P451, DOI 10.1007/s11695-018-3531-2
Jang JW, 2012, GASTROENTEROLOGY, V142, P805, DOI 10.1053/j.gastro.2011.12.051
Johnson G, 2021, ENDOSCOPY, V53, P1071, DOI 10.1055/a-1537-8999
Jovani M, 2021, GASTROINTEST ENDOSC, V93, P1088, DOI 10.1016/j.gie.2020.09.041
Kahaleh M, 2007, GASTROINTEST ENDOSC, V65, P224, DOI 10.1016/j.gie.2006.05.008

Kamata K, 2017, ARAB J GASTROENTEROL, V18, P120, DOI 10.1016/j.ajg.2017.05.016
Kamata K, 2017, WORLD J GASTROENTERO, V23, P661, DOI 10.3748/wjg.v23.i4.661
Kastelijn JB, 2020, ENDOSC INT OPEN, V08, pE1194, DOI 10.1055/a-1214-5659
Kawakami H, 2020, J HEPATO-BIL-PAN SCI, V27, pE11, DOI 10.1002/jhbp.676
Keane MG, 2021, GASTROINTEST ENDOSC, V93, pAB208, DOI 10.1016/j.gie.2021.03.465
Kedia P, 2019, J CLIN GASTROENTEROL, V53, P304, DOI 10.1097/MCG.0000000000001037
Kedia P, 2015, GASTROINTEST ENDOSC, V82, P560, DOI 10.1016/j.gie.2015.03.1913
Kedia P, 2015, ENDOSCOPY, V47, P159, DOI 10.1055/s-0034-1390771
Kedia P, 2014, GASTROENTEROLOGY, V147, P566, DOI 10.1053/j.gastro.2014.05.045
Khashab MA, 2019, ENDOSCOPY, V51, P772, DOI 10.1055/a-0921-5579
Khashab MA, 2017, ENDOSC INT OPEN, V5, pE275, DOI 10.1055/s-0043-101695
Khashab MA, 2015, GASTROINTEST ENDOSC, V82, P932, DOI 10.1016/j.gie.2015.06.017
Khashab MA, 2015, GASTROINTEST ENDOSC, V82, P745, DOI 10.1016/j.gie.2015.05.017
Kochhar Gursimran S, 2020, Endosc Int Open, V8, pE877, DOI [10.1055/a-1164-6282, 10.1055/a-1164-6282]
Krafft MR, 2020, GASTROINTEST ENDOSC, V92, P1055, DOI 10.1016/j.gie.2020.04.061
Krafft MR, 2021, DIGEST DIS SCI, V66, P2776, DOI 10.1007/s10620-020-06551-5
Krafft MR, 2019, DIGEST DIS SCI, V64, P1770, DOI 10.1007/s10620-019-05495-9
Krishnamoorthi R, 2020, SURG ENDOSC, V34, P1904, DOI 10.1007/s00464-020-07409-7
Kurihara T, 2013, DIGEST ENDOSC, V25, P109, DOI 10.1111/den.12100
Lakhtakia Sundeep, 2020, VideoGIE, V5, P359, DOI [10.1016/j.vgie.2020.04.023, 10.1016/j.vgie.2020.04.023]
Law R, 2016, CLIN GASTROENTEROL H, V14, P476, DOI 10.1016/j.cgh.2015.10.026
Ma KW, 2020, J GASTROEN HEPATOL, V35, P1753, DOI 10.1111/jgh.15089
Mangas-Sanjuan C, 2020, ENDOSCOPY, V52, pE47, DOI 10.1055/a-0991-7763
Manno M, 2020, ENDOSCOPY, V52, pE144, DOI 10.1055/a-1032-8218
Marrache MK, 2020, ENDOSCOPY, V52, pE355, DOI 10.1055/a-1127-2820
Marrache MK, 2021, GASTROINTEST ENDOSC, V93, P34, DOI 10.1016/j.gie.2020.06.057
Martínez B, 2019, ENDOSC INT OPEN, V7, pE1038, DOI 10.1055/a-0918-5931
Martins FP, 2010, ENDOSCOPY, V42, pE126, DOI 10.1055/s-0029-1243911
Matsumoto K, 2018, BMC GASTROENTEROL, V18, DOI 10.1186/s12876-018-0886-8
Matsunami Y, 2021, ENDOSC ULTRASOUND, V10, P294, DOI 10.4103/EUS-D-20-00232
McCarty TR, 2021, SURG ENDOSC, V35, P4964, DOI 10.1007/s00464-020-07758-3
McCarty Thomas R, 2019, Endosc Int Open, V7, pE1474, DOI [10.1055/a-0996-8178, 10.1055/a-0996-8178]
Minaga K, 2018, ENDOSCOPY, V50, pE153, DOI 10.1055/a-0596-7171
Minaga K, 2017, GASTROINTEST ENDOSC, V85, P263, DOI 10.1016/j.gie.2016.03.016
Miyano A, 2018, J GASTROINTEST SURG, V22, P1312, DOI 10.1007/s11605-018-3758-1
Moon Eunice, 2010, Semin Intervent Radiol, V27, P327, DOI 10.1055/s-0030-1267853
Moryoussef F, 2017, THER ADV GASTROENTER, V10, P537, DOI 10.1177/1756283X17702614
Mosquera-Klinger G, 2019, REV ESP ENFERM DIG, V111, P74, DOI 10.17235/reed.2018.5815/2018
Ogura T, 2020, GASTROINTEST ENDOSC, V92, P659, DOI 10.1016/j.gie.2020.04.038
Ogura T, 2019, ENDOSC ULTRASOUND, V8, P133, DOI 10.4103/eus.eus_68_18
Ogura T, 2016, WORLD J GASTROENTERO, V22, P3945, DOI 10.3748/wjg.v22.i15.3945
Ogura T, 2016, GASTROINTEST ENDOSC, V83, P831, DOI 10.1016/j.gie.2015.10.008

Ogura T, 2015, J GASTROEN HEPATOL, V30, P1748, DOI 10.1111/jgh.13021
Oh D, 2017, THER ADV GASTROENTER, V10, P42, DOI 10.1177/1756283X16671671
Oh D, 2016, GASTROINTEST ENDOSC, V83, P366, DOI 10.1016/j.gie.2015.07.015
Okuno N, 2018, J GASTROEN HEPATOL, V33, P1413, DOI 10.1111/jgh.14112
Okuno N, 2015, ENDOSCOPY, V47, pE311, DOI 10.1055/s-0034-1392314
Paik WH, 2019, ENDOSC ULTRASOUND, V8, pS44, DOI 10.4103/eus.eus_51_19
Park DH, 2011, GASTROINTEST ENDOSC, V74, P1276, DOI 10.1016/j.gie.2011.07.054
Park SJ, 2013, GASTROINTEST ENDOSC, V78, P374, DOI 10.1016/j.gie.2013.04.183
Perez-Miranda M, 2018, ENDOSC ULTRASOUND, V7, P79, DOI 10.4103/eus.eus_5_18
Podboy A, 2021, GASTROINTEST ENDOSC, V93, P797, DOI 10.1016/j.gie.2020.09.040
Poincloux L, 2015, ENDOSCOPY, V47, P794, DOI [10.1055/s-0035-1545399, 10.1055/s-0034-1391988]
Prachayakul V, 2015, WORLD J GASTROENTERO, V21, P2725, DOI 10.3748/wjg.v21.i9.2725
Prakash S, 2022, ENDOSCOPY, V54, P52, DOI 10.1055/a-1376-2394
Runge TM, 2021, ENDOSCOPY, V53, P611, DOI 10.1055/a-1254-3942
Saftoiu A, 2013, BMC GASTROENTEROL, V13, DOI 10.1186/1471-230X-13-93
Sato Tatsuya, 2019, VideoGIE, V4, P420, DOI [10.1016/j.vgie.2019.04.017, 10.1016/j.vgie.2019.04.017]
Saumoy M, 2018, ENDOSC ULTRASOUND, V7, P97, DOI 10.4103/eus.eus_9_18
Shima Y, 2014, ENDOSCOPY, V46, pE563, DOI 10.1055/s-0034-1377945
Shimamura Y, 2017, CLIN ENDOSC, V50, P112, DOI 10.5946/ce.2017.046
Shinn B, 2021, GASTROINTEST ENDOSC, V94, P727, DOI 10.1016/j.gie.2021.04.022
Sodarat P, 2021, ENDOSCOPY, V53, pE350, DOI 10.1055/a-1290-7221
Song TJ, 2010, GASTROINTEST ENDOSC, V71, P634, DOI 10.1016/j.gie.2009.11.024
Sportes A, 2019, ENDOSCOPY, V51, pE135, DOI 10.1055/a-0866-8986
Sundaram S, 2021, ENDOSC ULTRASOUND, V10, P154, DOI 10.4103/EUS-D-21-00004
Teoh AY, 2019, ENDOSC INT OPEN, V7, pE964, DOI 10.1055/a-0915-2098
Teoh AYB, 2020, GUT, V69, P1085, DOI 10.1136/gutjnl-2019-319996
Teoh AYB, 2021, GASTROINTEST ENDOSC, V93, P577, DOI 10.1016/j.gie.2020.06.066
Teoh AYB, 2019, ENDOSC ULTRASOUND, V8, pS40, DOI 10.4103/eus.eus_49_19
Teoh AYB, 2017, ENDOSCOPY, V49, P130, DOI 10.1055/s-0042-119036
Teoh AYB, 2014, GASTROINTEST ENDOSC, V80, P1171, DOI 10.1016/j.gie.2014.03.038
Tessier G, 2007, GASTROINTEST ENDOSC, V65, P233, DOI 10.1016/j.gie.2006.06.029
Tonozuka R, 2020, CLIN ENDOSC, V53, P510, DOI 10.5946/ce.2020.151
Tyberg A, 2020, ENDOSC ULTRASOUND, V9, P392, DOI 10.4103/eus.eus_42_20
Tyberg A, 2020, J CLIN GASTROENTEROL, V54, P569, DOI 10.1097/MCG.0000000000001326
Tyberg A, 2017, GASTROINTEST ENDOSC, V85, P164, DOI 10.1016/j.gie.2016.07.030
Umeda J, 2015, GASTROINTEST ENDOSC, V82, P390, DOI 10.1016/j.gie.2015.02.041
van der Merwe SW, 2022, ENDOSCOPY, V54, P185, DOI 10.1055/a-1717-1391
van Huijgevoort NCM, 2020, ENDOSC INT OPEN, V08, pE1070, DOI 10.1055/a-1171-1322
Vanella G, 2020, ENDOSC INT OPEN, V08, pE1782, DOI 10.1055/a-1264-7511
Wang SP, 2016, ENDOSCOPY, V48, pE28, DOI 10.1055/s-0042-100454
Wang TJ, 2019, SURG ENDOSC, V33, P2024, DOI 10.1007/s00464-019-06715-z
Yamamoto Y, 2020, ENDOSC ULTRASOUND, V9, P110, DOI 10.4103/eus.eus_68_19

Zulli C, 2020, ENDOSCOPY, V52, pE215, DOI 10.1055/a-1073-7317
NR 155
TC 147
Z9 165
U1 2
U2 28
PU GEORG THIEME VERLAG KG
PI STUTTGART
PA Oswald-Hesse-Strasse 50, D-70469 STUTTGART, GERMANY
SN 0013-726X
EI 1438-8812
J9 ENDOSCOPY
JI Endoscopy
PD MAR
PY 2022
VL 54
IS 03
BP 310
EP 332
DI 10.1055/a-1738-6780
EA FEB 2022
PG 23
WC Gastroenterology & Hepatology; Surgery
WE Science Citation Index Expanded (SCI-EXPANDED)
SC Gastroenterology & Hepatology; Surgery
GA ZP4AO
UT WOS:000750544000001
PM 35114696
OA Green Submitted, Green Published, Bronze
DA 2026-05-09
ER

PT J
AU Chan, SM
Teoh, AYB
Chiu, PWY
Ng, EKW
Lau, JYW
AF Chan, Shannon Melissa
Teoh, Anthony Yuen Bun
Chiu, Philip Wai Yan
Ng, Enders Kwok Wai

Lau, James Yun Wong
TI Advanced gallbladder interventions after endoscopic
ultrasonography-guided gallbladder drainage
SO TECHNIQUES AND INNOVATIONS IN GASTROINTESTINAL ENDOSCOPY
LA English
DT Article
DE Cholecystoscopy; Gallbladder interventions; Endoscopic ultrasound guided
gallbladder; drainage; EGBD
AB The development of EUS-guided gallbladder drainage (EGBD) with a lumen-apposing stent has made endoscopic assessment and advanced gallbladder interventions via the stent possible. This is a review article on the different interventions reported in literature. Diagnostic procedures with magnifying endoscopy, endocytoscopy, endoscopic ultrasound, and confocal laser microscopy for polyps and carcinoma of gallbladder have been reported. Therapeutic interventions for stone removal with snare, Roth net, mechanical basket lithotripsy, or laser lithotripsy. Polypectomy can also be performed with snare. With EGBD becoming more popular, more upcoming new interventions may be feasible. (c) 2019 Elsevier Inc. All rights reserved.
C1 [Chan, Shannon Melissa; Teoh, Anthony Yuen Bun; Chiu, Philip Wai Yan; Ng, Enders Kwok Wai; Lau, James Yun Wong] Chinese Univ Hong Kong, Prince Wales Hosp, Dept Surg, Shatin, Hong Kong, Peoples R China.
[Chan, Shannon Melissa; Teoh, Anthony Yuen Bun; Chiu, Philip Wai Yan; Ng, Enders Kwok Wai; Lau, James Yun Wong] Chinese Univ Hong Kong, Prince Wales Hosp, Inst Digest Dis, Hong Kong, Peoples R China.
C3 Prince of Wales Hospital Hong Kong; Chinese University of Hong Kong;
Prince of Wales Hospital Hong Kong; Chinese University of Hong Kong
RP Teoh, AYB (corresponding author), Chinese Univ Hong Kong, Prince Wales Hosp, Dept Surg, Shatin, Hong Kong, Peoples R China.; Teoh, AYB (corresponding author), Chinese Univ Hong Kong, Prince Wales Hosp, Inst Digest Dis, Hong Kong, Peoples R China.
EM anthonyteoh@surgery.cuhk.edu.hk
RI Ng, Enders/E-8276-2016; Teoh, Anthony/L-4796-2016; Lau,
James/O-2612-2016; Chiu, Philip/A-4019-2009
CR Chan SM, 2017, GASTROINTEST ENDOSC, V85, P1225, DOI 10.1016/j.gie.2016.10.014
CHESLYNCURTIS S, 1992, GUT, V33, P1253, DOI 10.1136/gut.33.9.1253
Choi JH, 2015, DIGEST ENDOSC, V27, P1, DOI 10.1111/den.12386
Choi JH, 2014, ENDOSCOPY, V46, P656, DOI 10.1055/s-0034-1365720
DONALD JJ, 1994, GUT, V35, P692, DOI 10.1136/gut.35.5.692
FROMM H, 1986, GASTROENTEROLOGY, V91, P1560, DOI 10.1016/0016-5085(86)90216-7
Ge N, 2016, BMC GASTROENTEROL, V16, DOI 10.1186/s12876-016-0420-9
Jang JW, 2012, GASTROENTEROLOGY, V142, P805, DOI 10.1053/j.gastro.2011.12.051
Jang JW, 2011, GASTROINTEST ENDOSC, V74, P176, DOI 10.1016/j.gie.2011.03.1120
Kamata K, 2017, WORLD J GASTROENTERO, V23, P661, DOI 10.3748/wjg.v23.i4.661
Ogura T, 2019, DIGEST ENDOSC, V31, P55, DOI 10.1111/den.13334
Okamoto K, 2018, J HEPATO-BIL-PAN SCI, V25, P55, DOI 10.1002/jhbp.516
Paumgartner G, 2005, EUR J GASTROEN HEPAT, V17, P525, DOI 10.1097/00042737-200505000-00009
Teoh AY, 2018, GASTROINTEST ENDOSC, V87, pAB62, DOI 10.1016/j.gie.2018.04.045
Teoh AYB, 2017, ENDOSCOPY, V49, P130, DOI 10.1055/s-0042-119036

Walter D, 2016, GUT, V65, P6, DOI 10.1136/gutjnl-2015-309925
Yang MJ, 2016, SCAND J GASTROENTERO, V51, P472, DOI 10.3109/00365521.2015.1115116
NR 17
TC 2
Z9 2
U1 0
U2 0
PU ELSEVIER SCIENCE INC
PI NEW YORK
PA STE 800, 230 PARK AVE, NEW YORK, NY 10169 USA
SN 2666-5107
EI 2590-0307
J9 TECH INNOVAT GASTROI
JI Tech. Innovation Gastrointes. Endoscopy
PD JAN
PY 2020
VL 22
IS 1
SI SI
BP 24
EP 26
DI 10.1016/j.tgie.2019.150629
PG 3
WC Gastroenterology & Hepatology
WE Emerging Sources Citation Index (ESCI)
SC Gastroenterology & Hepatology
GA VK9JD
UT WOS:000768800500007
DA 2026-05-09
ER

PT J
AU Ashat, M
El-Abiad, R
Shrigiriwar, A
Khashab, MA
AF Ashat, Munish
El-Abiad, Rami
Shrigiriwar, Apurva
Khashab, Mouen A.
TI Interventional Endoscopic Ultrasound: Current Status and Future
Frontiers

SO AMERICAN JOURNAL OF GASTROENTEROLOGY
LA English
DT Review
DE EUS-guided; gastroenterostomy; endohepatology; biliary drainage; lumen
apposing metal stent
ID CELIAC PLEXUS NEUROLYSIS; INTRAHEPATIC PORTOSYSTEMIC SHUNT; GUIDED
RADIOFREQUENCY ABLATION; GASTRIC FUNDAL VARICES; INTERNATIONAL
MULTICENTER; CYANOACRYLATE GLUE; LIVER-ABSCESS; EUS; DRAINAGE;
MANAGEMENT
AB Interventional endoscopic ultrasound has fueled remarkable advancements in the field of therapeutic procedures, revolutionizing minimally invasive interventions for a diverse range of conditions. This review highlights the latest breakthroughs and advancements in therapeutic endoscopic ultrasound, showcasing its potential to transform patient care.
C1 [Ashat, Munish; El-Abiad, Rami] Univ Iowa Hosp & Clin, Dept Gastroenterol & Hepatol, Iowa City, IA USA.
[Shrigiriwar, Apurva; Khashab, Mouen A.] Johns Hopkins Univ Hosp, Div Gastroenterol & Hepatol, Baltimore, MD 21287 USA.
C3 University of Iowa; Johns Hopkins University; Johns Hopkins Medicine
RP Khashab, MA (corresponding author), Johns Hopkins Univ Hosp, Div Gastroenterol & Hepatol, Baltimore, MD 21287 USA.
EM ashatmunish@gmail.com; rami-elabiad@uiowa.edu; ashrigi1@jhmi.edu;
mkhasha1@jhmi.edu
RI khashab, mouen/F-6643-2017
CR Abdel-Aal AK, 2014, VASC ENDOVASC SURG, V48, P460, DOI 10.1177/1538574414547087
Aggarwal SN, 2021, GASTROINTEST ENDOSC, V93, P1133, DOI 10.1016/j.gie.2020.09.050
Armellini E, 2023, MEDICINA-LITHUANIA, V59, DOI 10.3390/medicina59020359
Arvanitakis M, 2018, ENDOSCOPY, V50, P524, DOI 10.1055/a-0588-5365
Bang JY, 2019, GUT, V68, P1200, DOI 10.1136/gutjnl-2017-315335
Barthet M, 2021, ENDOSC INT OPEN, V09, pE1178, DOI 10.1055/a-1479-2199
Barthet M, 2019, ENDOSCOPY, V51, P836, DOI 10.1055/a-0824-7067
Bhat YM, 2016, GASTROINTEST ENDOSC, V83, P1164, DOI 10.1016/j.gie.2015.09.040
Binmoeller KF, 2011, GASTROINTEST ENDOSC, V74, P1019, DOI 10.1016/j.gie.2011.06.030
Buscaglia JM, 2009, GASTROINTEST ENDOSC, V69, P941, DOI 10.1016/j.gie.2008.09.051
Campos S, 2019, ENDOSC INT OPEN, V7, pE1262, DOI 10.1055/a-0958-2183
Chen YI, 2017, SURG ENDOSC, V31, P2946, DOI 10.1007/s00464-016-5311-1
Chen YI, 2017, GASTROINTEST ENDOSC, V85, P170, DOI 10.1016/j.gie.2016.07.031
Chin MA, 2018, ENDOSC ULTRASOUND, V7, P223, DOI 10.4103/eus.eus_31_18
Chin YK, 2020, SAGE OPEN MED, V8, DOI 10.1177/2050312120921273
Ching-Companioni RA, 2021, GASTROINTEST ENDOSC, V94, P551, DOI 10.1016/j.gie.2021.03.023
Choi JH, 2018, ENDOSCOPY, V50, P1099, DOI 10.1055/a-0583-8387
Chua Tiffany, 2019, Gastrointest Endosc Clin N Am, V29, P369, DOI [10.1016/j.giec.2018.11.007, 10.1016/j.giec.2018.11.007]
Crino SF., 2023, Clin Gastroenterol Hepatol
Dhindsa BS, 2020, ENDOSC ULTRASOUND, V9, P101, DOI 10.4103/eus.eus_80_19

Doi S, 2013, ENDOSCOPY, V45, P362, DOI 10.1055/s-0032-1326225
Faigel DO, 2016, GASTROINTEST ENDOSC, V83, P444, DOI 10.1016/j.gie.2015.08.064
Fujii-Lau LL, 2015, J HEPATO-BIL-PAN SCI, V22, P51, DOI 10.1002/jhbp.187
Ghandour B, 2023, GASTROINTEST ENDOSC, V97, P260, DOI 10.1016/j.gie.2022.09.028
Ghandour B, 2022, GASTROINTEST ENDOSC, V95, P80, DOI 10.1016/j.gie.2021.07.023
Hashimoto R, 2021, DIGEST DIS SCI, V66, P1700, DOI 10.1007/s10620-020-06391-3
Huang JY, 2017, GASTROINTEST ENDOSC, V85, P996, DOI 10.1016/j.gie.2016.09.026
Irani SS, 2023, CLIN GASTROENTEROL H, V21, P1141, DOI 10.1016/j.cgh.2022.12.039
Issa D, 2021, ENDOSCOPY, V53, P827, DOI 10.1055/a-1259-0349
Itoi T, 2016, GUT, V65, P193, DOI 10.1136/gutjnl-2015-310348
James HJ, 2019, ENDOSCOPY, V51, P1051, DOI 10.1055/a-0938-3918
Jiang TA, 2016, SCI REP-UK, V6, DOI 10.1038/srep36098
Kalva NR, 2018, CAN J GASTROENTEROL, V2018, DOI 10.1155/2018/7070961
Kaufman M, 2010, J CLIN GASTROENTEROL, V44, P127, DOI 10.1097/MCG.0b013e3181bb854d
Kedia P, 2019, J CLIN GASTROENTEROL, V53, P304, DOI 10.1097/MCG.0000000000001037
Khashab MA, 2017, ENDOSC INT OPEN, V5, pE275, DOI 10.1055/s-0043-101695
Khashab MA, 2015, GASTROINTEST ENDOSC, V82, P932, DOI 10.1016/j.gie.2015.06.017
Khurana S, 2019, HEPATOL INT, V13, P323, DOI 10.1007/s12072-019-09945-4
Klair JS, 2023, J CLIN GASTROENTEROL, V57, P211, DOI 10.1097/MCG.0000000000001543
Köker IH, 2017, SCAND J GASTROENTERO, V52, P1158, DOI 10.1080/00365521.2017.1335771
Krafft MR, 2022, DIGEST DIS SCI, V67, P2337, DOI 10.1007/s10620-021-07003-4
Krishnamoorthi R, 2020, SURG ENDOSC, V34, P1904, DOI 10.1007/s00464-020-07409-7
Lee S, 2014, GASTROINTEST ENDOSC, V80, P1014, DOI 10.1016/j.gie.2014.03.037
Lester C, 2022, CLIN GASTROENTEROL H, V20, pE326, DOI 10.1016/j.cgh.2021.03.041
Levy MJ, 2019, CLIN GASTROENTEROL H, V17, P728, DOI 10.1016/j.cgh.2018.08.040
Loeve US, 2013, GASTROINTEST ENDOSC, V77, P151, DOI 10.1016/j.gie.2012.03.005
Matthes K, 2005, ACTA GASTRO-ENT BELG, V68, P412
McCarty TR, 2020, ENDOSC ULTRASOUND, V9, P6, DOI 10.4103/eus.eus_37_19
Minaga K, 2016, GASTROINTEST ENDOSC, V83, P1039, DOI 10.1016/j.gie.2015.10.044
Mohan BP, 2019, Gastrointest Endosc, V89
Mok SRS, 2018, GASTROINTEST ENDOSC, V88, P919, DOI 10.1016/j.gie.2018.07.036
Moyer MT, 2017, GASTROENTEROLOGY, V153, P1295, DOI 10.1053/j.gastro.2017.08.009
Napoléon B, 2023, GASTROINTEST ENDOSC, V98, P392, DOI 10.1016/j.gie.2023.04.003
Nieto Jose, 2020, Endosc Int Open, V8, pE938, DOI [10.1055/a-1165-1767, 10.1055/a-1165-1767]
Nieto J, 2018, GASTROINTEST ENDOSC, V87, P469, DOI 10.1016/j.gie.2017.05.013
Ogura T, 2016, DIGEST DIS SCI, V61, P303, DOI 10.1007/s10620-015-3841-3
Ogura T, 2015, ENDOSCOPY, V47, pE397, DOI 10.1055/s-0034-1392632
Oleinikov K, 2019, J CLIN ENDOCR METAB, V104, P2637, DOI 10.1210/jc.2019-00282
Park TY, 2016, ENDOSC ULTRASOUND, V5, P315, DOI 10.4103/2303-9027.191611
Pineda JJ, 2016, GASTROINTEST ENDOSC, V83, P360, DOI 10.1016/j.gie.2015.08.025
Podboy A, 2021, GASTROINTEST ENDOSC, V93, P797, DOI 10.1016/j.gie.2020.09.040
Poincloux L, 2017, ENDOSC ULTRASOUND, V6, P394, DOI 10.4103/eus.eus_42_17

Prakash S, 2022, ENDOSCOPY, V54, P52, DOI 10.1055/a-1376-2394
Robles-Medranda C, 2019, DIGEST ENDOSC, V31, P283, DOI 10.1111/den.13305
Romero-Castro R, 2013, GASTROINTEST ENDOSC, V78, P711, DOI 10.1016/j.gie.2013.05.009
Samarasena Jason B, 2019, Gastrointest Endosc Clin N Am, V29, P311, DOI [10.1016/j.giec.2018.12.004, 10.1016/j.giec.2018.12.004]
Schulman AR, 2017, GASTROINTEST ENDOSC, V85, P243, DOI 10.1016/j.gie.2016.07.041
Shah Manisha H, 2021, J Natl Compr Canc Netw, V19, P839, DOI [10.6004/jnccn.2021.0032, 10.6004/jnccn.2021.0032]
Sharma M, 2010, GASTROINTEST ENDOSC, V72, P1106, DOI 10.1016/j.gie.2010.02.054
Shi G, 2017, ENDOSC ULTRASOUND, V6, P343, DOI 10.4103/2303-9027.210609
Shinn B, 2021, GASTROINTEST ENDOSC, V94, P727, DOI 10.1016/j.gie.2021.04.022
So H, 2016, AM J GASTROENTEROL, V111, P925, DOI 10.1038/ajg.2016.73
Teoh AYB, 2023, GASTROENTEROLOGY, V165, P473, DOI 10.1053/j.gastro.2023.04.016
Teoh AYB, 2021, GASTROINTEST ENDOSC, V93, P577, DOI 10.1016/j.gie.2020.06.066
Teoh AYB, 2019, ENDOSC ULTRASOUND, V8, pS40, DOI 10.4103/eus.eus_49_19
Tyberg A, 2017, GASTROINTEST ENDOSC, V85, P164, DOI 10.1016/j.gie.2016.07.030
van der Merwe SW, 2022, ENDOSCOPY, V54, P185, DOI 10.1055/a-1717-1391
van Wanrooij RLJ, 2022, ENDOSCOPY, V54, P310, DOI 10.1055/a-1738-6780
Vanek P, 2023, GASTROINTEST ENDOSC, V97, P1070, DOI 10.1016/j.gie.2022.12.026
Varadarajulu S, 2009, GASTROINTEST ENDOSC, V70, P372, DOI 10.1016/j.gie.2009.03.008
Vazquez-Sequeiros E, 2010, WORLD J GASTRO ENDOS, V2, P198, DOI 10.4253/wjge.v2.i6.198
Weilert F, 2012, GASTROINTEST ENDOSC, V76, P915, DOI 10.1016/j.gie.2011.09.027
Yang JL, 2019, ENDOSCOPY, V51, P1035, DOI 10.1055/a-0759-1353
NR 83
TC 4
Z9 5
U1 1
U2 6
PU LIPPINCOTT WILLIAMS & WILKINS
PI PHILADELPHIA
PA TWO COMMERCE SQ, 2001 MARKET ST, PHILADELPHIA, PA 19103 USA
SN 0002-9270
EI 1572-0241
J9 AM J GASTROENTEROL
JI Am. J. Gastroenterol.
PD OCT
PY 2023
VL 118
IS 10
BP 1768
EP 1778
DI 10.14309/ajg.0000000000002487

PG 11
WC Gastroenterology & Hepatology
WE Science Citation Index Expanded (SCI-EXPANDED)
SC Gastroenterology & Hepatology
GA EV9G9
UT WOS:001141819600020
PM 37646335
DA 2026-05-09
ER

PT J
AU Matsumoto, K
Yamao, K
Okubo, K
Hara, K
Sawaki, A
Mizuno, N
Tajika, M
Kawai, H
Ashida, R
AF Matsumoto, Kakuya
Yamao, Kenji
Okubo, Kenji
Hara, Kazuo
Sawaki, Akira
Mizuno, Nobumasa
Tajika, Masahiro
Kawai, Hiroki
Ashida, Reiko
TI Endoscopic ultrasound-guided ethanol injection in the pancreas in a
porcine model: A preliminary study
SO JOURNAL OF GASTROENTEROLOGY AND HEPATOLOGY
LA English
DT Article
DE endoscopic ultrasound; ethanol injection; pancreas; porcine model;
preliminary study
ID RADIOFREQUENCY THERMAL ABLATION; SMALL HEPATOCELLULAR-CARCINOMA;
FINE-NEEDLE ASPIRATION; THERAPY; CANCER; TRIAL; GEMCITABINE;
RADIOTHERAPY; PSEUDOCYSTS; RADIATION
AB Background and Aim: Despite aggressive multimodal treatments, survival rates for patients with pancreatic cancer remain disappointing. Local progression is problematic, and minimally invasive procedures allowing locoregional control are needed. In this study, we attempted endoscopic ultrasound (EUS)-guided injection of ethanol into the pancreas.

Methods: Under EUS guidance, pure ethanol (2 mL) was injected into normal tissue of the pancreatic body in two anesthetized domestic pigs. Serum concentrations of amylase, aspartame aminotransferase, alanine aminotransferase, alkaline phosphatase, and lactate dehydrogenase were measured before treatment and at 2 h, 48 h and 2 weeks after injection. Body weight and clinical signs were also observed. After the animals were euthanized, the pancreases were analyzed histologically.

Results: EUS imaging allows real-time surveillance of the injection procedure. Mild diarrhea was noted in one animal, but no other adverse effects were observed. No marked changes in laboratory tests were noted. Histologically, parenchymal necrosis extending over a wide area was seen without severe inflammation.

Conclusion: EUS-guided ethanol injection in the pancreas seems to be technically simple. More detailed assessments of the safety and dose-effect relationship issues associated with this procedure are required.
C1 [Matsumoto, Kakuya] Asahikawa Med Coll, Dept Med 2, Asahikawa, Hokkaido 0788510, Japan.

[Matsumoto, Kakuya; Yamao, Kenji; Okubo, Kenji; Hara, Kazuo; Sawaki, Akira; Mizuno, Nobumasa; Tajika, Masahiro; Kawai, Hiroki; Ashida, Reiko] Aichi Canc Ctr Hosp, Dept Gastroenterol, Nagoya, Aichi 464, Japan.

C3 Asahikawa Medical University; Aichi Cancer Center

RP Matsumoto, K (corresponding author), Asahikawa Med Coll, Dept Med 2, 2-1-1-1 Midorigaokahigashi, Asahikawa, Hokkaido 0788510, Japan.

EM kmatsumo@asahikawa-med.ac.jp
CR Alfieri S, 2001, ARCH SURG-CHICAGO, V136, P343, DOI 10.1001/archsurg.136.3.343

Aslanian H, 2005, GASTROINTEST ENDOSC, V62, P723, DOI 10.1016/j.gie.2005.06.048

Bassi C, 2001, DIGEST SURG, V18, P453, DOI 10.1159/000050193

Burcharth F, 2003, HEPATO-GASTROENTEROL, V50, P563

Burris HA, 1997, J CLIN ONCOL, V15, P2403, DOI 10.1200/JCO.1997.15.6.2403

Chan HH, 2004, GASTROINTEST ENDOSC, V59, P95, DOI 10.1016/S0016-5107(03)02361-7

Chang KJ, 1997, GASTROINTEST ENDOSC, V45, P387, DOI 10.1016/S0016-5107(97)70149-4

Chang KJ, 2000, CANCER-AM CANCER SOC, V88, P1325, DOI 10.1002/(SICI)1097-0142(20000315)88:6<1325::AID-CNCR8>3.0.CO;2-T

Chiang PH, 2003, UROLOGY, V61, P797, DOI 10.1016/S0090-4295(02)02514-1

DOBELBOWER RR, 1978, CANCER-AM CANCER SOC, V41, P1087, DOI 10.1002/1097-0142(197803)41:3<1087::AID-CNCR2820410342>3.0.CO;2-9

ETZKORN KP, 1995, ENDOSCOPY, V27, P329, DOI 10.1055/s-2007-1005702

Fosh B G, 2001, BMC Gastroenterol, V1, P9, DOI 10.1186/1471-230X-1-9

Gan SI, 2005, GASTROINTEST ENDOSC, V61, P746, DOI 10.1016/S0016-5107(05)00320-2

GIOVANNINI M, 1994, CANCER, V73, P294, DOI 10.1002/1097-0142(19940115)73:2<294::AID-CNCR2820730210>3.0.CO;2-J

Giovannini M, 2001, ENDOSCOPY, V33, P898, DOI 10.1055/s-2001-17324

Giovannini M, 2001, ENDOSCOPY, V33, P473, DOI 10.1055/s-2001-14967

Goldberg SN, 1999, GASTROINTEST ENDOSC, V50, P392, DOI 10.1053/ge.1999.v50.98847

Gress F, 2001, AM J GASTROENTEROL, V96, P409

Gress FG, 1997, GASTROINTEST ENDOSC, V45, P243, DOI 10.1016/S0016-5107(97)70266-9

Günter E, 2003, GASTROINTEST ENDOSC, V57, P113, DOI 10.1067/mge.2003.39

Hecht JR, 2003, CLIN CANCER RES, V9, P555

Homma H, 2000, CANCER, V89, P303, DOI 10.1002/1097-0142(20000715)89:2<303::AID-CNCR15>3.0.CO;2-1

Ikeda M, 2001, JPN J CLIN ONCOL, V31, P322, DOI 10.1093/jjco/hye063
Kim JH, 2003, AM J ROENTGENOL, V180, P1723, DOI 10.2214/ajr.180.6.1801723
Krinsky ML, 2000, GASTROINTEST ENDOSC, V52, pS35, DOI 10.1067/mge.2000.110717
LEE MJ, 1995, AM J ROENTGENOL, V164, P215, DOI 10.2214/ajr.164.1.7998542
Lin SM, 2005, GUT, V54, P1151, DOI 10.1136/gut.2004.045203
Livraghi T, 1999, RADIOLOGY, V210, P655, DOI 10.1148/radiology.210.3.r99fe40655
Livraghi T, 2001, HEPATO-GASTROENTEROL, V48, P20
Merkle EM, 1999, RADIOLOGY, V213, P461, DOI 10.1148/radiology.213.2.r99nv21461
MOERTEL CG, 1981, CANCER-AM CANCER SOC, V48, P1705, DOI 10.1002/1097-0142(19811015)48:8<1705::AID-CNCR2820480803>3.0.CO;2-4
Mortensen M B, 1999, Eur J Ultrasound, V10, P93, DOI 10.1016/S0929-8266(99)00057-9
Paulino AC, 2000, CANCER INVEST, V18, P309, DOI 10.3109/07357900009012173
Rothenberg ML, 1996, ANN ONCOL, V7, P347
Sindelar WF, 1999, ANN ONCOL, V10, P226, DOI 10.1023/A:1008395827074
VEHMAS T, 1993, SCAND J GASTROENTERO, V28, P791, DOI 10.3109/00365529309104011
NR 36
TC 14
Z9 15
U1 0
U2 1
PU WILEY
PI HOBOKEN
PA 111 RIVER ST, HOBOKEN 07030-5774, NJ USA
SN 0815-9319
EI 1440-1746
J9 J GASTROEN HEPATOL
JI J. Gastroenterol. Hepatol.
PD JUL
PY 2008
VL 23
IS 7
BP E1
EP E6
DI 10.1111/j.1440-1746.2007.04918.x
PN 2
PG 6
WC Gastroenterology & Hepatology
WE Science Citation Index Expanded (SCI-EXPANDED)
SC Gastroenterology & Hepatology
GA 327VL
UT WOS:000257756500001
PM 18702683

DA 2026-05-09
ER

PT J
AU Anderloni, A
Di Leo, M
Carrara, S
Fugazza, A
Maselli, R
Buda, A
Amato, A
Auriemma, F
Repici, A
AF Anderloni, Andrea
Di Leo, Milena
Carrara, Silvia
Fugazza, Alessandro
Maselli, Roberta
Buda, Andrea
Amato, Arnaldo
Auriemma, Francesco
Repici, Alessandro
TI Endoscopic ultrasound-guided transmural drainage by cautery-tipped lumen-apposing metal stent: exploring the possible indications
SO ANNALS OF GASTROENTEROLOGY
LA English
DT Article
DE Pancreatic fluid collection drainage; gallbladder drainage; biliary drainage; lumen apposing metal stent; Hot AXIOS stent
ID PANCREATIC FLUID COLLECTIONS; GALLBLADDER DRAINAGE; CONSENSUS GUIDELINES; PLACEMENT; THERAPY; MANAGEMENT; NECROSIS
AB Background The recently introduced Hot AXIOS (TM) system for endoscopic ultrasound (EUS)-guided transenteric drainage has the potential to change interventional endoscopy significantly. The aim of our study was to assess the effectiveness and safety of this new type of lumen-apposing metal stent (LAMS) with cautery system for pancreatic collection, and gallbladder and biliary tree drainage.

Methods We retrospectively reviewed consecutive patients undergoing EUS-guided drainage by LAMS with cautery system in a tertiary-care academic medical center between March 2014 and March 2017. All patients were included in our prospectively maintained institutional EUS database. The main outcome measures were technical success, clinical effectiveness, and adverse events.

Results A total of 45 patients (20 men, mean age 69.6 years) underwent LAMS placement. Indications were pancreatic fluid collections (19 patients, 42.2%), acute cholecystitis (10 patients, 22.2%), and biliary drainage (16 patients, 35.5%). Technical success was achieved in all patients except one (97.7%). Clinical success was achieved in 86.4% (38/44) of cases

and adverse events occurred in 5 (11.4%) of patients.

Conclusions In our experience, EUS-guided LAMS placement performed by expert endoscopists was feasible and effective in the endoscopic management of pancreatic fluid collection, and biliary and gallbladder drainage. Optimization of transmural drainage by new dedicated devices could improve efficacy and safety in appropriately selected patients.

C1 [Anderloni, Andrea; Di Leo, Milena; Carrara, Silvia; Fugazza, Alessandro; Maselli, Roberta; Auriemma, Francesco; Repici, Alessandro] Humanitas Res Hosp, Dept Gastroenterol, Digest Endoscopy Unit, 56 Via Manzoni, I-20089 Milan, Italy.

[Di Leo, Milena; Repici, Alessandro] Humanitas Univ, Milan, Italy.

[Buda, Andrea] Digest Endoscopy Unit, Feltre, Italy.

[Amato, Arnaldo] Valduce, Gastroenterol Div, Como, Italy.

C3 Humanitas University

RP Anderloni, A (corresponding author), Humanitas Res Hosp, Dept Gastroenterol, Digest Endoscopy Unit, 56 Via Manzoni, I-20089 Milan, Italy.

EM andrea.anderloni@humanitas.it

RI Carrara, Silvia/AAC-5298-2022; Maselli, Roberta/LFU-9657-2024; Buda, Anea/O-4341-2019; Anderloni, Andrea/ABE-9139-2020; Amato, Arnaldo/ABC-5688-2020; Repici, Alessandro/HFH-8162-2022; Fugazza, Alessandro/ABG-9381-2021

OI Carrara, Silvia/0000-0003-4206-9463; Maselli, Roberta/0000-0001-7291-9110; Di Leo, Milena/0000-0002-5933-8474; Buda, Anea/0000-0002-3099-8612; Anderloni, Andrea/0000-0002-1021-0031; Amato, Arnaldo/0000-0002-4397-4142; Repici, Alessandro/0000-0002-1621-6450; Fugazza, Alessandro/0000-0003-0485-4903

CR Anderloni A, 2017, ENDOSC INT OPEN, V5, pE1100, DOI 10.1055/s-0043-118828

Anderloni A, 2017, ENDOSC INT OPEN, V5, pE25, DOI 10.1055/s-0042-122009

Anderloni A, 2016, SURG ENDOSC, V30, P5200, DOI 10.1007/s00464-016-4894-x

Anderloni A, 2016, ENDOSCOPY, V48, pE321, DOI 10.1055/s-0042-117425

Bang JY, 2019, GUT, V68, P1200, DOI 10.1136/gutjnl-2017-315335

Chandran S, 2015, GASTROINTEST ENDOSC, V81, P127, DOI 10.1016/j.gie.2014.06.025

Dollhopf M, 2017, GASTROINTEST ENDOSC, V86, P636, DOI 10.1016/j.gie.2017.02.027

Guo JT, 2017, ENDOSC ULTRASOUND, V6, P285, DOI 10.4103/eus.eus_85_17

Irani S, 2017, CLIN GASTROENTEROL H, V15, P738, DOI 10.1016/j.cgh.2016.12.021

Kunda R, 2016, SURG ENDOSC, V30, P5002, DOI 10.1007/s00464-016-4845-6

Muthusamy VR, 2016, GASTROINTEST ENDOSC, V83, P481, DOI 10.1016/j.gie.2015.11.027

Patil R, 2016, ANN GASTROENTEROL, V29, P168, DOI 10.20524/aog.2016.0008

Pezzilli R, 2015, DIGEST LIVER DIS, V47, P532, DOI 10.1016/j.dld.2015.03.022

Rinninella E, 2015, GASTROINTEST ENDOSC, V82, P1039, DOI 10.1016/j.gie.2015.04.006

Sharaiha RZ, 2016, CLIN GASTROENTEROL H, V14, P1797, DOI 10.1016/j.cgh.2016.05.011

Siddiqui AA, 2016, GASTROINTEST ENDOSC, V83, P699, DOI 10.1016/j.gie.2015.10.020

Teoh AYB, 2018, GUT, V67, P1209, DOI 10.1136/gutjnl-2017-314341

Vazquez-Sequeiros E, 2016, GASTROINTEST ENDOSC, V84, P450, DOI 10.1016/j.gie.2016.02.044

Veitch AM, 2016, GUT, V65, P374, DOI 10.1136/gutjnl-2015-311110

Voermans RP, 2016, GASTROINTEST ENDOSC, V83, P708, DOI 10.1016/j.gie.2015.12.032
Walter D, 2016, GUT, V65, P6, DOI 10.1136/gutjnl-2015-309925
Walter D, 2015, ENDOSCOPY, V47, P63, DOI 10.1055/s-0034-1378113
Yokoe M, 2013, J HEPATO-BIL-PAN SCI, V20, P35, DOI 10.1007/s00534-012-0568-9
NR 23
TC 29
Z9 31
U1 0
U2 0
PU HELLENIC SOC GASTROENTEROLOGY
PI ATHENS
PA DEMOKRATIAS AVE 67, ATHENS, 15451, GREECE
SN 1108-7471
EI 1792-7463
J9 ANN GASTROENTEROL
JI Ann. Gastroenterol.
PY 2018
VL 31
IS 6
BP 735
EP 741
DI 10.20524/aog.2018.0299
PG 7
WC Gastroenterology & Hepatology
WE Emerging Sources Citation Index (ESCI)
SC Gastroenterology & Hepatology
GA HF5JT
UT WOS:000454269300013
PM 30386125
OA Green Submitted, gold
DA 2026-05-09
ER

PT J
AU Itoi, T
Sofuni, A
Itokawa, F
Tsuchiya, T
Kurihara, T
Ishii, K
Tsuji, S
Ikeuchi, N

Umeda, J
Moriyasu, F
Tsuchida, A
AF Itoi, Takao
Sofuni, Atsushi
Itokawa, Fumihide
Tsuchiya, Takayoshi
Kurihara, Toshio
Ishii, Kentaro
Tsuji, Shujiro
Ikeuchi, Nobuhito
Umeda, Junko
Moriyasu, Fuminori
Tsuchida, Akihiko
TI Endoscopic ultrasonography-guided biliary drainage
SO JOURNAL OF HEPATO-BILIARY-PANCREATIC SCIENCES
LA English
DT Article
DE Endoscopic ultrasonography-guided biliary drainage; Endoscopic ultrasonography-guided choledochoduodenostomy; Endoscopic ultrasonography-guided hepaticogastrostomy; Endoscopic ultrasonography-guided gallbladder drainage
ID COVERED METAL STENT; ACUTE CHOLECYSTITIS; PROSPECTIVE FEASIBILITY; GALLBLADDER DRAINAGE; ACUTE CHOLANGITIS; CASE SERIES; OBSTRUCTION; CHOLEDOCHODUODENOSTOMY; CHOLECYSTOSTOMY; DEPLOYMENT
AB Background and purpose Endoscopic ultrasonography-guided biliary drainage (EUS-BD) has been developed as an alternative drainage method in patients with obstructive jaundice. EUS-BD is divided into EUS-guided choledo-choduodenostomy (EUS-CDS), EUS-guided hepaticogastrostomy (EUS-HGS) and EUS-guided gallbladder drainage (EUS-GBD). The aim of this review is to focus on the current status and limitations of EUS-BD.

Methods A systematic review was performed to evaluate EUS-BD. MEDLINE, EMBASE and manual searches were performed to identify the pertinent English language full articles.

Results The high success rate without fatal adverse events for EUS-CDS (93%; 28/30) and EUS-HGS (97%; 28/ 29) suggest the feasibility and safety of the procedures in high-volume endoscopic centers adopting various procedural techniques. Although the number of reported cases was very small, the success rate of EUS-GBD was high (100%; 14/14), without serious adverse events.

Conclusions Although all procedures require further assessment in a larger cohort of patients, including comparative studies between EUS-CDS or EUS-HGS versus PTBD, and EUS-GBD versus PTGBD, EUS-BD may be a promising procedure for the treatment of obstructive jaundice. However, dedicated devices for EUS-guided drainage are needed for reliable procedures.
C1 [Itoi, Takao; Sofuni, Atsushi; Itokawa, Fumihide; Tsuchiya, Takayoshi; Kurihara, Toshio; Ishii, Kentaro; Tsuji, Shujiro; Ikeuchi, Nobuhito; Umeda, Junko; Moriyasu, Fuminori] Tokyo Med Univ, Dept Gastroenterol & Hepatol, Shinjuku Ku, Tokyo, Japan.

[Tsuchida, Akihiko] Tokyo Med Univ, Dept Surg 3, Tokyo, Japan.
C3 Tokyo Medical University; Tokyo Medical University
RP Itoi, T (corresponding author), Tokyo Med Univ, Dept Gastroenterol & Hepatol, Shinjuku Ku, Nishishinjuku 6-7-1, Tokyo, Japan.
EM itoi@tokyo-med.ac.jp
RI Ishii, Kentaro/AAW-1590-2020
CR ANG TL, 2007, JOP, V9, P438
Artifon ELA, 2007, CLINICS, V62, P799, DOI 10.1590/S1807-59322007000600023
Baron TH, 2002, GASTROINTEST ENDOSC, V56, P753, DOI 10.1067/mge.2002.129217
Baron TH, 2007, GASTROINTEST ENDOSC, V65, P735, DOI 10.1016/j.gie.2006.07.041
Bories E, 2007, ENDOSCOPY, V39, P287, DOI 10.1055/s-2007-966212
Burmester E, 2003, GASTROINTEST ENDOSC, V57, P246, DOI 10.1067/mge.2003.85
Fogel EL, 2001, ENDOSCOPY, V33, P31, DOI 10.1055/s-2001-11186
Fujita N, 2008, J GASTROENTEROL, V43, P637, DOI 10.1007/s00535-008-2204-7
Fujita N, 2007, WORLD J GASTROENTERO, V13, P5512, DOI 10.3748/wjg.v13.i41.5512
Giovannini M, 2001, ENDOSCOPY, V33, P898, DOI 10.1055/s-2001-17324
Hawes RH, 2009, GASTROINTEST ENDOSC, V69, pS1, DOI 10.1016/j.gie.2008.11.013
Itoi T, 2008, WORLD J GASTROENTERO, V14, P6078, DOI 10.3748/wjg.14.6078
Itoi T, 2008, GASTROINTEST ENDOSC, V68, P84, DOI 10.1016/j.gie.2007.11.050
Itoi T, 2009, GASTROINTEST ENDOSC, V69, pS8, DOI 10.1016/j.gie.2008.11.003
Kahaleh M, 2006, GASTROINTEST ENDOSC, V64, P52, DOI 10.1016/j.gie.2006.01.063
Kwan V, 2007, GASTROINTEST ENDOSC, V66, P582, DOI 10.1016/j.gie.2007.02.065
LAI EC, 1992, NEW ENGL J MED, V24, P1582
Lee SS, 2007, GASTROINTEST ENDOSC, V66, P1008, DOI 10.1016/j.gie.2007.03.1080
Park DH, 2009, AM J GASTROENTEROL, V104, P2168, DOI 10.1038/ajg.2009.254
Patterson EJ, 1996, SURG ENDOSC, V10, P1185, DOI 10.1007/s004649900275
PESSA ME, 1987, ANN SURG, V205, P389, DOI 10.1097/00000658-198704000-00008
Püspök A, 2005, AM J GASTROENTEROL, V100, P1743, DOI 10.1111/j.1572-0241.2005.41806.x
SMITH AC, 1994, LANCET, V344, P1655, DOI 10.1016/S0140-6736(94)90455-3
Takasawa O, 2009, DIGEST ENDOSC, V21, P43, DOI 10.1111/j.1443-1661.2008.00822.x
Tarantino I, 2008, ENDOSCOPY, V40, P336, DOI 10.1055/s-2007-995455
Will U, 2007, ENDOSCOPY, V39, P292, DOI 10.1055/s-2007-966215
Yamao K, 2008, ENDOSCOPY, V40, P340, DOI 10.1055/s-2007-995485
YAMAO K, 2006, SUIZO, V21, P353
Yamao K, 2006, GASTROINTEST ENDOSC, V64, P663, DOI 10.1016/j.gie.2006.07.003
NR 29
TC 43
Z9 55
U1 0
U2 4
PU SPRINGER TOKYO
PI TOKYO

PA 1-11-11 KUDAN-KITA, CHIYODA-KU, TOKYO, 102-0073, JAPAN
SN 1868-6974
J9 J HEPATO-BIL-PAN SCI
JI J. Hepato-Biliary-Pancreat. Sci.
PD SEP
PY 2010
VL 17
IS 5
BP 611
EP 616
DI 10.1007/s00534-009-0196-1
PG 6
WC Gastroenterology & Hepatology; Surgery
WE Science Citation Index Expanded (SCI-EXPANDED)
SC Gastroenterology & Hepatology; Surgery
GA 637UV
UT WOS:000280845100014
PM 19806298
OA Bronze
DA 2026-05-09
ER

PT J
AU Yuan, T
Liu, SX
Zhu, C
Dong, YY
Zhu, HJ
Wu, X
Tang, Y
Zhao, WG
AF Yuan, Tao
Liu, Shixuan
Zhu, Cheng
Dong, Yingyue
Zhu, Huijuan
Wu, Xi
Tang, Yan
Zhao, Weigang
TI Continuous Glucose Monitoring in Patients With Insulinoma Treated by Endoscopic Ultrasound-Guided Ethanol Injection
SO PANCREAS

LA English
DT Article
DE continuous glucose monitoring; endoscopic ultrasound– guided
ethanol injection; hypoglycemia; insulinoma; self-monitoring of blood
glucose; therapeutic evaluation
AB Objectives
The aims of this study were to analyze the continuous glucose monitoring (CGM) profiles of patients with insulinoma before and after treatment with endoscopic ultrasound-guided ethanol injection and assess the value of CGM in curative effect evaluating. Methods
We included 8 patients, and CGM was performed for 3 to 5 days before and after treatment. Results
The proportion of monitoring points at which the glucose level was lower than 3.9 mmol/L after treatment decreased in patient 5 (from 4% to 3%) and patient 8 (from 30% to 12%), whereas the proportion increased in patient 1 (from 1% to 16%), patient 3 (from 5% to 23%), and patient 7 (from 7% to 63%). There was no mean significant difference between CGM values (5.75 [standard deviation, 2.49] mmol/L) and self-monitoring of blood glucose values (5.76 [standard deviation, 2.32] mmol/L) ($P > 0.05$). Pearson correlation analysis showed positive correlation between CGM values and self-monitoring of blood glucose values ($r = 0.88$, $P < 0.05$). Clarke Error Grid Analysis showed that 91.5% of pairs were located in areas A and B. Conclusions
Continuous glucose monitoring is useful for detecting hypoglycemia and evaluating curative effect, but the correction of fingertip blood glucose is necessary when the blood glucose is relatively low.
C1 [Yuan, Tao; Liu, Shixuan; Dong, Yingyue; Zhu, Huijuan; Zhao, Weigang] Peking Union Med Coll & Chinese Acad Med Sci, Dept Endocrinol, Key Lab Endocrinol, Natl Hlth Commiss,Peking Union Med Coll Hosp, Beijing, Peoples R China.
[Zhu, Cheng; Wu, Xi] Peking Union Med Coll & Chinese Acad Med Sci, Dept Gastroenterol, Peking Union Med Coll Hosp, Beijing, Peoples R China.
[Tang, Yan] Peking Union Med Coll & Chinese Acad Med Sci, Dept Pharm, Peking Union Med Coll Hosp, Beijing, Peoples R China.
C3 Chinese Academy of Medical Sciences - Peking Union Medical College;
Peking Union Medical College; Peking Union Medical College Hospital;
Chinese Academy of Medical Sciences - Peking Union Medical College;
Peking Union Medical College; Peking Union Medical College Hospital;
Chinese Academy of Medical Sciences - Peking Union Medical College;
Peking Union Medical College Hospital; Peking Union Medical College
RP Zhao, WG (corresponding author), Peking Union Med Coll Hosp, Shuaifuyuan St 1, Beijing 0086100730, Peoples R China.
EM xiehezhaoweigang@163.com
RI Zhu, Cheng/JXN-6166-2024
FU CAMS Innovation Fund for Medical Science, the Non-profit Central
Research Institute Fund of Chinese Academy of Medical Sciences
[2016-I2M-4-001]; "13th Five-Year" National Science and Technology Major
Project for New Drugs [2019ZX09734001]; Non-Profit Central Research
Institute Fund of Chinese Academy of Medical Sciences [2017PT32020,
2018PT32001]
FX This work was supported by the CAMS Innovation Fund for Medical Science,

the Non-profit Central Research Institute Fund of Chinese Academy of
Medical Sciences (2016-I2M-4-001 to T.Y.); the "13th Five-Year" National
Science and Technology Major Project for New Drugs (2019ZX09734001 to
W.Z.), and the Non-Profit Central Research Institute Fund of Chinese
Academy of Medical Sciences (no. 2017PT32020, No: 2018PT32001 to T.Y.).
CR [Anonymous], 2014, J DIABETES SCI TECHN
CLARKE WL, 1987, DIABETES CARE, V10, P622, DOI 10.2337/diacare.10.5.622
Cryer PE, 1999, ENDOCRIN METAB CLIN, V28, P495, DOI 10.1016/S0889-8529(05)70084-0
Gu WJ, 2017, DIABETOL METAB SYNDR, V9, DOI 10.1186/s13098-017-0215-3
Guerci B, 2003, DIABETES CARE, V26, P582, DOI 10.2337/diacare.26.3.582
Halfdanarson TR, 2008, ENDOCR-RELAT CANCER, V15, P409, DOI 10.1677/ERC-07-0221
Iglesias P, 2015, ENDOCRINOL NUTR, V62, P306, DOI 10.1016/j.endonu.2015.04.004
Kandaswamy L, 2016, ENDOCRINE, V53, P47, DOI 10.1007/s12020-016-0902-0
Kikuchi T, 2017, INTERNAL MED, V56, P3067, DOI 10.2169/internalmedicine.8766-16
Klonoff DC, 2017, DIABETES RES CLIN PR, V133, P178, DOI 10.1016/j.diabres.2017.08.005
Matej A, 2016, HORM-INT J ENDOCRINO, V15, P489, DOI 10.14310/horm.2002.1706
Munir A, 2008, CLIN ENDOCRINOL, V68, P912, DOI 10.1111/j.1365-2265.2007.03161.x
Okabayashi T, 2013, WORLD J GASTROENTERO, V19, P829, DOI 10.3748/wjg.v19.i6.829
Rodbard D, 2017, DIABETES TECHNOL THE, V19, pS25, DOI 10.1089/dia.2017.0035
Sato J, 2012, J DIABETES INVEST, V3, P225, DOI 10.1111/j.2040-1124.2012.00197.x
Wentholt IM, 2005, DIABETES CARE, V28, P2871, DOI 10.2337/diacare.28.12.2871
NR 16
TC 9
Z9 12
U1 0
U2 5
PU LIPPINCOTT WILLIAMS & WILKINS
PI PHILADELPHIA
PA TWO COMMERCE SQ, 2001 MARKET ST, PHILADELPHIA, PA 19103 USA
SN 0885-3177
EI 1536-4828
J9 PANCREAS
JI Pancreas
PD FEB
PY 2021
VL 50
IS 2
BP 183
EP 188
DI 10.1097/MPA.0000000000001735
PG 6
WC Gastroenterology & Hepatology

WE Science Citation Index Expanded (SCI-EXPANDED)
SC Gastroenterology & Hepatology
GA QF0YI
UT WOS:000616626100017
PM 33560091
OA hybrid
DA 2026-05-09
ER

PT J
AU Fabbri, C
Luigiano, C
Lisotti, A
Cennamo, V
Virgilio, C
Caletti, G
Fusaroli, P
AF Fabbri, Carlo
Luigiano, Carmelo
Lisotti, Andrea
Cennamo, Vincenzo
Virgilio, Clara
Caletti, Giancarlo
Fusaroli, Pietro
TI Endoscopic ultrasound-guided treatments: Are we getting evidence based - a systematic review
SO WORLD JOURNAL OF GASTROENTEROLOGY
LA English
DT Article
DE Endoscopic ultrasound; Pseudocyst drainage; Necrosectomy; Celiac plexus neurolysis; Levels of evidence; Fine needle injection
ID PANCREATIC-FLUID COLLECTIONS; CELIAC PLEXUS NEUROLYSIS; GROUP DOCUMENT EVALUATION; SPLENIC ARTERY PSEUDOANEURYSM; MALIGNANT BILIARY OBSTRUCTION; RANDOMIZED CONTROLLED-TRIAL; EXPANDABLE METAL STENT; SPINAL-CORD INFARCTION; FINE-NEEDLE INJECTION; TERM-FOLLOW-UP
AB The continued need to develop less invasive alternatives to surgical and radiologic interventions has driven the development of endoscopic ultrasound (EUS)-guided treatments. These include EUS-guided drainage of pancreatic fluid collections, EUS-guided necrosectomy, EUS-guided cholangiography and biliary drainage, EUS-guided pancreatography and pancreatic duct drainage, EUS-guided gallbladder drainage, EUS-guided drainage of abdominal and pelvic fluid collections, EUS-guided celiac plexus block and celiac plexus neurolysis, EUS-guided pancreatic cyst ablation, EUS-guided vascular interventions, EUS-guided delivery of antitumoral agents and EUS-guided fiducial placement and brachytherapy. However these procedures are technically challenging and require expertise in both EUS and interventional endoscopy, such

as endoscopic retrograde cholangiopancreatography and gastrointestinal stenting. We undertook a systematic review to record the entire body of literature accumulated over the past 2 decades on EUS-guided interventions with the objective of performing a critical appraisal of published articles, based on the classification of studies according to levels of evidence, in order to assess the scientific progress made in this field. (C) 2014 Baishideng Publishing Group Inc. All rights reserved.
C1 [Fabbri, Carlo; Cennamo, Vincenzo] AUSL Bologna Bellaria Maggiore Hosp, Unit Gastroenterol & Digest Endoscopy, I-40139 Bologna, Italy.
[Luigiano, Carmelo; Virgilio, Clara] ARNAS Garibaldi, Unit Gastroenterol & Digest Endoscopy, I-95122 Catania, Italy.
[Lisotti, Andrea; Caletti, Giancarlo; Fusaroli, Pietro] Univ Bologna, Hosp S Maria Scaletta, Dept Med & Surg Sci DIMEC, I-40026 Imola, Italy.
C3 AUSL di Bologna; University of Bologna; Azienda USL di Imola
RP Fusaroli, P (corresponding author), Univ Bologna, Hosp S Maria Scaletta, Dept Med & Surg Sci DIMEC, Via Montericco 4, I-40026 Imola, Italy.
EM pietro.fusaroli@unibo.it
RI Fabbri, Carlo/AAC-4467-2022; Lisotti, Andrea/L-6762-2019; Fusaroli, Pietro/AAC-2182-2020; cennamo, Vincenzo/AAC-7601-2022
OI Fabbri, Carlo/0000-0003-0670-9910; Lisotti, Andrea/0000-0002-7724-7402; Fusaroli, Pietro/0000-0002-4397-9314;
CR Ahlawat SK, 2006, J PANCREAS, V7, P616
Ahmed HM, 2009, ENDOSCOPY, V41, pE218, DOI 10.1055/s-0029-1214941
Ahn JY, 2010, GUT LIVER, V4, P524, DOI 10.5009/gnl.2010.4.4.524
Allum WH, 2011, GUT, V60, P1449, DOI 10.1136/gut.2010.228254
Ammar T, 2010, GASTROINTEST ENDOSC, V71, P630, DOI 10.1016/j.gie.2009.11.023
Ang TL, 2009, ENDOSCOPY, V41, pE21, DOI 10.1055/s-0028-1103468
Ang TL, 2013, SINGAP MED J, V54, P206, DOI 10.11622/smedj.2013074
Ang TL, 2009, J DIGEST DIS, V10, P213, DOI 10.1111/j.1751-2980.2009.00388.x
[Anonymous], J SUPPORT ONCOL
Antillon MR, 2006, GASTROINTEST ENDOSC, V63, P797, DOI 10.1016/j.gie.2005.10.025
Arcidiacono PG, 2011, COCHRANE DB SYST REV, DOI 10.1002/14651858.CD007519.pub2
Arcidiacono PG, 2012, GASTROINTEST ENDOSC, V76, P1142, DOI 10.1016/j.gie.2012.08.006
Ardengh JC, 2008, DIGEST DIS, V26, P370, DOI 10.1159/000177024
Artifon ELA, 2007, GASTROINTEST ENDOSC, V66, P1201, DOI 10.1016/j.gie.2007.05.051
Artifon ELA, 2012, J CLIN GASTROENTEROL, V46, P768, DOI 10.1097/MCG.0b013e31825f264c
Arvanitakis M, 2007, GASTROINTEST ENDOSC, V65, P609, DOI 10.1016/j.gie.2006.06.083
Ascunce G, 2011, GASTROINTEST ENDOSC, V73, P267, DOI 10.1016/j.gie.2010.10.029
Attwell AR, 2003, GASTROINTEST ENDOSC, V58, P612, DOI 10.1067/S0016-5107(03)01966-7
Azar RR, 2006, GASTROINTEST ENDOSC, V63, P688, DOI 10.1016/j.gie.2005.10.032
Bakker OJ, 2012, JAMA-J AM MED ASSOC, V307, P1053, DOI 10.1001/jama.2012.276
Barclay RL, 2002, GASTROINTEST ENDOSC, V55, P266, DOI 10.1067/mge.2002.120784
Barkay O, 2010, GASTROINTEST ENDOSC, V71, P1166, DOI 10.1016/j.gie.2009.10.048
Baron TH, 2007, GASTROINTEST ENDOSC, V65, P735, DOI 10.1016/j.gie.2006.07.041
Barthet M, 2008, GASTROINTEST ENDOSC, V67, P245, DOI 10.1016/j.gie.2007.06.014
Binmoeller K F, 1995, Gastrointest Endosc Clin N Am, V5, P805

Binmoeller KF, 2013, SURG ENDOSC, V27, P1835, DOI 10.1007/s00464-012-2682-9
Binmoeller KF, 2011, GASTROINTEST ENDOSC, V74, P1019, DOI 10.1016/j.gie.2011.06.030
Binmoeller KF, 2011, J HEPATO-BIL-PAN SCI, V18, P319, DOI 10.1007/s00534-010-0358-1
Bories E, 2007, ENDOSCOPY, V39, P287, DOI 10.1055/s-2007-966212
Brauer BC, 2009, GASTROINTEST ENDOSC, V70, P471, DOI 10.1016/j.gie.2008.12.233
Súbtil JC, 2010, WORLD J GASTRO ENDOS, V2, P203, DOI 10.4253/wjge.v2.i6.203
Chang KJ, 2008, NAT CLIN PRACT GASTR, V5, P107, DOI 10.1038/ncpgasthep1033
Chang KJ, 2012, GASTROINTEST ENDOSC, V75, P1139, DOI 10.1016/j.gie.2012.01.042
Chang KJ, 2000, CANCER-AM CANCER SOC, V88, P1325, DOI 10.1002/(SICI)1097-0142(20000315)88:6<1325::AID-CNCR8>3.0.CO;2-T
Charnley RM, 2006, ENDOSCOPY, V38, P925, DOI 10.1055/s-2006-944731
Chaves DM, 2012, ENDOSCOPY, V44, pE99, DOI 10.1055/s-0030-1256740
de la Serna-Higuera C, 2013, GASTROINTEST ENDOSC, V77, P303, DOI 10.1016/j.gie.2012.09.021
de Paulo GA, 2006, GASTROINTEST ENDOSC, V63, P396, DOI 10.1016/j.gie.2005.10.039
Decker C, 2011, GASTROINTEST ENDOSC, V73, P1056, DOI 10.1016/j.gie.2010.09.006
Deprez PH, 2008, ACTA GASTRO-ENT BELG, V71, P333
Desilets DJ, 2013, GASTROINTEST ENDOSC, V77, P835, DOI 10.1016/j.gie.2013.02.017
DeWitt J, 2011, GASTROINTEST ENDOSC, V74, P446, DOI 10.1016/j.gie.2011.01.064
DeWitt J, 2010, GASTROINTEST ENDOSC, V72, P862, DOI 10.1016/j.gie.2010.02.039
DeWitt J, 2009, GASTROINTEST ENDOSC, V70, P710, DOI 10.1016/j.gie.2009.03.1173
Dhir V, 2013, UNITED EUR GASTROENT, V1, P103, DOI 10.1177/2050640613480145
Dhir V, 2014, DIGEST ENDOSC, V26, P430, DOI 10.1111/den.12153
Dhir V, 2012, GASTROINTEST ENDOSC, V75, P354, DOI 10.1016/j.gie.2011.07.075
DiMaio CJ, 2011, PANCREAS, V40, P664, DOI 10.1097/MPA.0b013e3182128d06
DiMaio CJ, 2010, GASTROINTEST ENDOSC, V71, P1204, DOI 10.1016/j.gie.2010.01.003
Doi S, 2013, ENDOSCOPY, V45, P362, DOI 10.1055/s-0032-1326225
Eccles Martin, 2002, J Health Serv Res Policy, V7, P98, DOI 10.1258/1355819021927746
Enya M, 2003, DIGEST ENDOSC, V15, P323, DOI [10.1046/j.1443-1661.2003.t01-2-00264.x, DOI 10.1046/J.1443-1661.2003.T01-2-00264.X]
Ergun M, 2011, ENDOSCOPY, V43, P518, DOI 10.1055/s-0030-1256333
Escourrou J, 2008, ANN SURG, V248, P1074, DOI 10.1097/SLA.0b013e31818b728b
Fabbri C, 2012, ENDOSCOPY, V44, P429, DOI 10.1055/s-0031-1291624
Fabbri C, 2011, ENDOSCOPY, V43, P438, DOI 10.1055/s-0030-1256097
Fabbri C, 2012, WORLD J GASTRO ENDOS, V4, P479, DOI 10.4253/wjge.v4.i11.479
Fockens P, 1996, GASTROINTEST ENDOSC, V44, P58, DOI 10.1016/S0016-5107(96)70230-4
Fujii L, 2012, ENDOSCOPY, V44, pE265, DOI 10.1055/s-0032-1309708
Fujii LL, 2013, GASTROINTEST ENDOSC, V78, P854, DOI 10.1016/j.gie.2013.05.016
Fusaroli P, 2002, ENDOSCOPY, V34, P451, DOI 10.1055/s-2002-32006
Fusaroli P, 2012, WORLD J GASTROENTERO, V18, P4243, DOI 10.3748/wjg.v18.i32.4243
Fusaroli P, 2012, DIGEST DIS SCI, V57, P602, DOI 10.1007/s10620-011-1961-y
Fusaroli P, 2011, J CLIN GASTROENTEROL, V45, P400, DOI 10.1097/MCG.0b013e3181fbde42
Gan SI, 2005, GASTROINTEST ENDOSC, V61, P746, DOI 10.1016/S0016-5107(05)00320-2

Ge N, 2013, GASTROENT RES PRACT, V2013, DOI 10.1155/2013/785483
Gimeno-García AZ, 2012, ENDOSCOPY, V44, pE267, DOI 10.1055/s-0032-1309709
Giovannini M, 2003, ENDOSCOPY, V35, P511
Giovannini M, 2001, ENDOSCOPY, V33, P473, DOI 10.1055/s-2001-14967
Gonzalez JM, 2012, ENDOSCOPY, V44, P539, DOI 10.1055/s-0031-1291609
Gonzalez JM, 2009, ENDOSCOPY, V41, P721, DOI 10.1055/s-0029-1214874
Gornals JB, 2013, DIGEST LIVER DIS, V45, P578, DOI 10.1016/j.dld.2013.01.023
Gornals JB, 2013, SURG ENDOSC, V27, P1428, DOI 10.1007/s00464-012-2591-y
Gress F, 1999, AM J GASTROENTEROL, V94, P900
Gress F, 2001, AM J GASTROENTEROL, V96, P409
GRESS F, 1997, GASTROINTEST ENDOSC, V45, pAB173
Gunaratnam NT, 2001, GASTROINTEST ENDOSC, V54, P316, DOI 10.1067/mge.2001.117515
Günter E, 2003, GASTROINTEST ENDOSC, V57, P113, DOI 10.1067/mge.2003.39
Gupta K, 2014, J CLIN GASTROENTEROL, V48, P80, DOI 10.1097/MCG.0b013e31828c6822
Gupta T, 2012, GASTROINTEST ENDOSC, V76, P1259, DOI 10.1016/j.gie.2012.07.037
Haghshenasskashani A, 2011, SURG ENDOSC, V25, P3724, DOI 10.1007/s00464-011-1795-x
Hanna N, 2012, CANCER GENE THER, V19, P374, DOI 10.1038/cgt.2012.10
Hara K, 2013, ENDOSCOPY, V45, P392, DOI 10.1055/s-0032-1326076
Hara K, 2011, AM J GASTROENTEROL, V106, P1239, DOI 10.1038/ajg.2011.84
Hecht JR, 2012, GASTROINTEST ENDOSC, V75, P332, DOI 10.1016/j.gie.2011.10.007
Hecht JR, 2003, CLIN CANCER RES, V9, P555
Heinzow HS, 2011, SCAND J GASTROENTERO, V46, P1004, DOI 10.3109/00365521.2011.571709
Ho KY, 2009, GASTROINTEST ENDOSC, V69, pS22, DOI 10.1016/j.gie.2008.10.059
Hocke M, 2008, Z GASTROENTEROL, V46, P1363, DOI 10.1055/s-2008-1027616
Hookey LC, 2006, GASTROINTEST ENDOSC, V63, P635, DOI 10.1016/j.gie.2005.06.028
Horaguchi J, 2012, DIGEST ENDOSC, V24, P457, DOI 10.1111/j.1443-1661.2012.01316.x
Horaguchi J, 2009, DIGEST ENDOSC, V21, P239, DOI 10.1111/j.1443-1661.2009.00899.x
Hritz I, 2013, WORLD J GASTROENTERO, V19, P3685, DOI 10.3748/wjg.v19.i23.3685
Hu YH, 2010, ENDOSCOPY, V42, pE256, DOI 10.1055/s-0030-1255653
Irisawa A, 2007, PANCREAS, V35, P189, DOI 10.1097/01.mpa.0000250141.25639.e9
Itoi T, 2013, DIGEST ENDOSC, V25, P137, DOI 10.1111/den.12084
Itoi T, 2012, GASTROINTEST ENDOSC, V75, P870, DOI 10.1016/j.gie.2011.10.020
Itoi T, 2011, DIGEST ENDOSC, V23, P158, DOI 10.1111/j.1443-1661.2011.01115.x
Itoi T, 2011, J HEPATO-BIL-PAN SCI, V18, P282, DOI 10.1007/s00534-010-0310-4
Itoi T, 2009, DIGEST ENDOSC, V21, pS53, DOI 10.1111/j.1443-1661.2009.00851.x
Iwashita T, 2012, ENDOSCOPY, V44, P60, DOI 10.1055/s-0030-1256871
Iwata K, 2011, DIGEST ENDOSC, V23, P140, DOI 10.1111/j.1443-1661.2010.01046.x
Jah A, 2008, WORLD J GASTROENTERO, V14, P6867, DOI 10.3748/wjg.14.6867
Jang HY, 2013, CLIN ENDOSC, V46, P306, DOI 10.5946/ce.2013.46.3.306
Jang JW, 2012, GASTROENTEROLOGY, V142, P805, DOI 10.1053/j.gastro.2011.12.051
Jang JW, 2011, GASTROINTEST ENDOSC, V74, P176, DOI 10.1016/j.gie.2011.03.1120
Jazrawi SF, 2011, DIGEST DIS SCI, V56, P902, DOI 10.1007/s10620-010-1350-y

Jin Z, 2008, ENDOSCOPY, V40, P314, DOI 10.1055/s-2007-995476
Jürgensen C, 2012, SURG ENDOSC, V26, P1359, DOI 10.1007/s00464-011-2039-9
Jürgensen C, 2006, GASTROINTEST ENDOSC, V63, P1059, DOI 10.1016/j.gie.2005.10.034
Kahaleh M, 2006, ENDOSCOPY, V38, P355, DOI 10.1055/s-2006-925249
Kahaleh M, 2005, ENDOSCOPY, V37, P393, DOI 10.1055/s-2005-860998
Kahaleh M, 2007, GASTROINTEST ENDOSC, V65, P224, DOI 10.1016/j.gie.2006.05.008
Kahaleh M, 2013, WORLD J GASTROENTERO, V19, P1372, DOI 10.3748/wjg.v19.i9.1372
Kamata K, 2010, ENDOSCOPY, V42, pE331, DOI 10.1055/s-0030-1255941
Kamata K, 2009, ENDOSCOPY, V41, pE315, DOI 10.1055/s-0029-1215258
Kato Shin, 2013, Diagn Ther Endosc, V2013, P924291, DOI 10.1155/2013/924291
Kaufman M, 2010, J CLIN GASTROENTEROL, V44, P127, DOI 10.1097/MCG.0b013e3181bb854d
Kawakubo K, 2014, J HEPATO-BIL-PAN SCI, V21, P328, DOI 10.1002/jhbp.27
Kawakubo K, 2013, SURG ENDOSC, V27, P3437, DOI 10.1007/s00464-013-2896-5
Khashab MA, 2013, GASTROINTEST ENDOSC, V78, P734, DOI 10.1016/j.gie.2013.05.013
Khashab MA, 2012, GASTROINTEST ENDOSC, V76, P962, DOI 10.1016/j.gie.2012.07.006
Khashab MA, 2012, SURG ENDOSC, V26, P3320, DOI 10.1007/s00464-012-2297-1
Kim TH, 2012, WORLD J GASTROENTERO, V18, P2526, DOI 10.3748/wjg.v18.i20.2526
Kim YS, 2010, ENDOSCOPY, V42, P496, DOI 10.1055/s-0029-1244082
Knuth J, 2013, ENDOSCOPY, V45, pE3, DOI 10.1055/s-0032-1326123
Komaki T, 2011, PANCREATOLOGY, V11, P47, DOI 10.1159/000323508
Krüger M, 2006, GASTROINTEST ENDOSC, V63, P409, DOI 10.1016/j.gie.2005.11.047
Künzli HT, 2013, EUR J GASTROEN HEPAT, V25, P958, DOI 10.1097/MEG.0b013e3283612f03
Kurihara T, 2013, DIGEST ENDOSC, V25, P109, DOI 10.1111/den.12100
Kwan V, 2007, GASTROINTEST ENDOSC, V66, P582, DOI 10.1016/j.gie.2007.02.065
Lah JJ, 2005, GASTROINTEST ENDOSC, V62, P805, DOI 10.1016/j.gie.2005.07.019
Lahoti S, 2000, GASTROINTEST ENDOSC, V51, P331, DOI 10.1016/S0016-5107(00)70363-4
Lalueza A, 2011, ENDOSCOPY, V43, pE9, DOI 10.1055/s-0030-1255804
Lameris R, 2011, GASTROINTEST ENDOSC, V73, P392, DOI 10.1016/j.gie.2010.05.019
Law JK, 2013, SURG ENDOSC, V27, P3921, DOI 10.1007/s00464-013-2975-7
Leblanc Julia K, 2013, Diagn Ther Endosc, V2013, P327036, DOI 10.1155/2013/327036
LeBlanc JK, 2011, GASTROINTEST ENDOSC, V74, P1300, DOI 10.1016/j.gie.2011.07.073
LeBlanc JK, 2009, GASTROINTEST ENDOSC, V69, P835, DOI 10.1016/j.gie.2008.05.071
Lee DH, 2006, GASTROINTEST ENDOSC, V64, P631, DOI 10.1016/j.gie.2006.04.031
Lee SS, 2007, GASTROINTEST ENDOSC, V66, P1008, DOI 10.1016/j.gie.2007.03.1080
Lee YT, 2000, GASTROINTEST ENDOSC, V52, P168, DOI 10.1067/mge.2000.107911
Levy MJ, 2008, GASTROINTEST ENDOSC, V67, P572, DOI 10.1016/j.gie.2007.06.063
Levy MJ, 2008, AM J GASTROENTEROL, V103, P352, DOI 10.1111/j.1572-0241.2007.01616.x
Levy MJ, 2008, AM J GASTROENTEROL, V103, P98, DOI 10.1111/j.1572-0241.2007.01607.x
Levy MJ, 2012, GASTROINTEST ENDOSC, V75, P200, DOI 10.1016/j.gie.2011.09.019
Levy MJ, 2009, GASTROINTEST ENDOSC, V69, pS37, DOI 10.1016/j.gie.2008.11.009
Loeve US, 2013, GASTROINTEST ENDOSC, V77, P151, DOI 10.1016/j.gie.2012.03.005
Lopes CV, 2007, SCAND J GASTROENTERO, V42, P524, DOI 10.1080/00365520601065093

Lopes César Vivian, 2008, Arq. Gastroenterol., V45, P17
Luigiano C, 2013, ENDOSCOPY, V45, pE245, DOI 10.1055/s-0032-1326250
Ulla-Rocha JL, 2012, THER ADV GASTROENTER, V5, P95, DOI 10.1177/1756283X11427420
Mahajan RJ, 2002, GASTROINTEST ENDOSC, V55, pAB101
Maier W, 1999, J LARYNGOL OTOL, V113, P41, DOI 10.1017/S0022215100143117
Majumder S, 2013, PANCREAS, V42, P692, DOI 10.1097/MPA.0b013e3182864559
Mangiavillano B, 2012, J DIGEST DIS, V13, P47, DOI 10.1111/j.1751-2980.2011.00547.x
Maranki J, 2009, ENDOSCOPY, V41, P532, DOI 10.1055/s-0029-1214712
Martínez-Monge R, 2006, LANCET ONCOL, V7, P781, DOI 10.1016/S1470-2045(06)70865-8
Mathew A, 2008, GASTROINTEST ENDOSC, V68, P776, DOI 10.1016/j.gie.2008.05.010
Mittal MK, 2012, NEUROLOGY, V78, pE57, DOI 10.1212/WNL.0b013e318248df51
Muscatiello N, 2006, ENDOSCOPY, V38, P858, DOI 10.1055/s-2006-925382
Muscatiello N, 2008, ENDOSCOPY, V40, pE83, DOI 10.1055/s-2007-995540
Noh SH, 2010, GASTROINTEST ENDOSC, V71, P1314, DOI 10.1016/j.gie.2009.12.045
Norton ID, 2001, MAYO CLIN PROC, V76, P794
O'Toole TM, 2009, ENDOSCOPY, V41, P593, DOI 10.1055/s-0029-1214868
Oh HC, 2008, GASTROINTEST ENDOSC, V67, P636, DOI 10.1016/j.gie.2007.09.038
Oh HC, 2012, DIGEST DIS SCI, V57, P1965, DOI 10.1007/s10620-012-2103-x
Oh HC, 2011, GASTROENTEROLOGY, V140, P172, DOI 10.1053/j.gastro.2010.10.001
Oh HC, 2009, SCAND J GASTROENTERO, V44, P242, DOI 10.1080/00365520802495537
Panamonta N, 2012, EUR J GASTROEN HEPAT, V24, P1355, DOI 10.1097/MEG.0b013e32835871eb
Park DH, 2009, ENDOSCOPY, V41, P842, DOI 10.1055/s-0029-1215133
Park DH, 2013, GASTROINTEST ENDOSC, V78, P91, DOI 10.1016/j.gie.2013.01.042
Park DH, 2011, GASTROINTEST ENDOSC, V74, P1276, DOI 10.1016/j.gie.2011.07.054
Park WG, 2010, GASTROINTEST ENDOSC, V71, P513, DOI 10.1016/j.gie.2009.10.030
Penman ID, 2009, GASTROINTEST ENDOSC, V69, pS28, DOI 10.1016/j.gie.2008.11.004
Penn DE, 2012, GASTROINTEST ENDOSC, V76, P679, DOI 10.1016/j.gie.2012.04.457
Perez-Miranda M, 2013, ENDOSCOPY, V45, P300, DOI 10.1055/s-0032-1326272
Pfaffenbach B, 1998, DEUT MED WOCHENSCHR, V123, P1439, DOI 10.1055/s-2007-1024199
Piraka C, 2009, GASTROINTEST ENDOSC, V70, P786, DOI 10.1016/j.gie.2009.04.049
Pishvaian AC, 2006, GASTROINTEST ENDOSC, V64, P412, DOI 10.1016/j.gie.2006.01.048
Puli SR, 2009, DIGEST DIS SCI, V54, P2330, DOI 10.1007/s10620-008-0651-x
Puri R, 2012, J GASTROEN HEPATOL, V27, P722, DOI 10.1111/j.1440-1746.2012.07089.x
Puri R, 2010, J GASTROEN HEPATOL, V25, P1416, DOI 10.1111/j.1440-1746.2010.06328.x
Ramesh J, 2013, J GASTROEN HEPATOL, V28, P620, DOI 10.1111/jgh.12081
Ramírez-Luna MA, 2011, ENDOSCOPY, V43, P826, DOI 10.1055/s-0030-1256406
Ramirez-Luna M A, 2008, Rev Gastroenterol Mex, V73, P63
Rana SS, 2013, DIGEST ENDOSC, V25, P47, DOI 10.1111/j.1443-1661.2012.01318.x
Rasmussen DN, 2012, DAN MED J, V59
Rische S, 2013, SCAND J GASTROENTERO, V48, P231, DOI 10.3109/00365521.2012.752029
Roach H, 2005, ENDOSCOPY, V37, P876, DOI 10.1055/s-2005-870201
Roberts KJ, 2012, HPB, V14, P489, DOI 10.1111/j.1477-2574.2012.00470.x

Robinson M, 2007, CARDIOVASC INTER RAD, V30, P515, DOI 10.1007/s00270-006-0081-0
Romero-Castro R, 2010, ENDOSCOPY, V42, pE35, DOI 10.1055/s-0029-1215261
Romero-Castro R, 2007, GASTROINTEST ENDOSC, V66, P402, DOI 10.1016/j.gie.2007.03.008
Romero-Castro R, 2013, GASTROINTEST ENDOSC, V78, P711, DOI 10.1016/j.gie.2013.05.009
Sadik R, 2011, WORLD J GASTROENTERO, V17, P499, DOI 10.3748/wjg.v17.i4.499
Sahai AV, 2009, AM J GASTROENTEROL, V104, P326, DOI 10.1038/ajg.2008.64
Sakamoto H, 2010, AM J GASTROENTEROL, V105, P2599, DOI 10.1038/ajg.2010.339
Sanders MK, 2010, GASTROINTEST ENDOSC, V71, P1178, DOI 10.1016/j.gie.2009.12.020
Santosh D, 2009, ALIMENT PHARM THER, V29, P979, DOI 10.1111/j.1365-2036.2009.03963.x
Sarkaria S, 2014, J CLIN GASTROENTEROL, V48, P145, DOI 10.1097/MCG.0b013e3182972219
Schrover IM, 2008, PANCREATOLOGY, V8, P271, DOI 10.1159/000134275
Seewald S, 2005, GASTROINTEST ENDOSC, V62, P92, DOI 10.1016/S0016-5107(05)00541-9
Seewald S, 2005, GASTROINTEST ENDOSC, V61, P495, DOI 10.1016/S0016-5107(04)02848-2
Seewald S, 2004, GASTROINTEST ENDOSC, V59, P578, DOI 10.1016/S0016-5107(03)02878-5
Seewald S, 2012, DIGEST ENDOSC, V24, P36, DOI 10.1111/j.1443-1661.2011.01162.x
Seicean A, 2013, J GASTROINTEST LIVER, V22, P59
Seicean A, 2011, J GASTROINTEST LIVER, V20, P39
Shah JN, 2012, GASTROINTEST ENDOSC, V75, P56, DOI 10.1016/j.gie.2011.08.032
Shami VM, 2008, GASTROINTEST ENDOSC, V67, P136, DOI 10.1016/j.gie.2007.07.040
Shin SK, 2010, KOREAN J PAIN, V23, P274, DOI 10.3344/kjp.2010.23.4.274
Siddiqui AA, 2013, GASTROINTEST ENDOSC, V78, P589, DOI 10.1016/j.gie.2013.03.1337
Singhal S, 2013, CLIN ENDOSC, V46, P506, DOI 10.5946/ce.2013.46.5.506
Song TJ, 2012, WORLD J GASTROENTERO, V18, P4435, DOI 10.3748/wjg.v18.i32.4435
Song TJ, 2010, GASTROINTEST ENDOSC, V71, P634, DOI 10.1016/j.gie.2009.11.024
Stevens T, 2012, CLIN GASTROENTEROL H, V10, P186, DOI 10.1016/j.cgh.2011.09.006
Sun S, 2006, ENDOSCOPY, V38, P399, DOI 10.1055/s-2006-925253
Takasawa O, 2009, DIGEST ENDOSC, V21, P43, DOI 10.1111/j.1443-1661.2008.00822.x
Talreja JP, 2008, GASTROINTEST ENDOSC, V68, P1199, DOI 10.1016/j.gie.2008.06.015
Téllez-Avila FI, 2013, ENDOSC ULTRASOUND, V2, P153, DOI [10.4103/2303-9027.117664, 10.7178/eus.06.007]
Téllez-Avila FI, 2013, WORLD J GASTRO ENDOS, V5, P297, DOI 10.4253/wjge.v5.i6.297
Tessier G, 2007, GASTROINTEST ENDOSC, V65, P233, DOI 10.1016/j.gie.2006.06.029
Tran Quy N H, 2006, J Support Oncol, V4, P460
Trevino JM, 2009, GASTROINTEST ENDOSC, V69, P361, DOI 10.1016/j.gie.2008.09.021
Varadarajulu S, 2012, ENDOSCOPY, V44, pE92, DOI 10.1055/s-0030-1256946
Varadarajulu S, 2010, ENDOSCOPY, V42, P423, DOI 10.1055/s-0029-1243989
Varadarajulu S, 2008, GASTROINTEST ENDOSC, V68, P1102, DOI 10.1016/j.gie.2008.04.028
Varadarajulu S, 2008, GASTROINTEST ENDOSC, V68, P656, DOI 10.1016/j.gie.2008.03.1091
Varadarajulu S, 2008, GASTROINTEST ENDOSC, V68, P649, DOI 10.1016/j.gie.2008.02.057
Varadarajulu S, 2007, GASTROINTEST ENDOSC, V66, P1107, DOI 10.1016/j.gie.2007.03.1027
Varadarajulu S, 2013, GASTROENTEROLOGY, V145, P583, DOI 10.1053/j.gastro.2013.05.046
Varadarajulu S, 2012, GASTROINTEST ENDOSC, V76, P336, DOI 10.1016/j.gie.2012.04.455
Varadarajulu S, 2011, J GASTROEN HEPATOL, V26, P1504, DOI 10.1111/j.1440-1746.2011.06771.x

Varadarajulu S, 2011, GASTROINTEST ENDOSC, V74, P418, DOI 10.1016/j.gie.2011.03.1242
Varadarajulu S, 2011, GASTROINTEST ENDOSC, V74, P74, DOI 10.1016/j.gie.2011.03.1122
Varadarajulu S, 2009, GASTROINTEST ENDOSC, V70, P1260, DOI 10.1016/j.gie.2009.06.007
Vila JJ, 2012, GASTROINTEST ENDOSC, V76, P1133, DOI 10.1016/j.gie.2012.08.001
Vleggaar FP, 2011, ENDOSCOPY, V43, pE328, DOI 10.1055/s-0030-1256775
Voermans RP, 2007, GASTROINTEST ENDOSC, V66, P909, DOI 10.1016/j.gie.2007.05.043
Voermans RP, 2011, GASTROINTEST ENDOSC, V74, P1285, DOI 10.1016/j.gie.2011.07.059
Vosoghi Mehrdad, 2002, MedGenMed, V4, P2
Wallace MB, 2009, GASTROINTEST ENDOSC, V69, pS59, DOI 10.1016/j.gie.2008.11.010
Wang KX, 2012, GASTROINTEST ENDOSC, V76, P945, DOI 10.1016/j.gie.2012.05.032
Weilert F, 2012, ENDOSCOPY, V44, P780, DOI 10.1055/s-0032-1309839
Weilert F, 2012, GASTROINTEST ENDOSC, V76, P915, DOI 10.1016/j.gie.2011.09.027
Widmer J, 2014, DIGEST ENDOSC, V26, P599, DOI 10.1111/den.12163
Wiechowska-Kozlowska A, 2012, GASTROENT RES PRACT, V2012, DOI 10.1155/2012/503098
Wiersema MJ, 1996, GASTROINTEST ENDOSC, V44, P656, DOI 10.1016/S0016-5107(96)70047-0
Will U, 2011, ULTRASCHALL MED, V32, P176, DOI 10.1055/s-0031-1273347
Will U, 2007, J HEPATO-BILIARY-PAN, V14, P377, DOI 10.1007/s00534-006-1139-8
Will U, 2012, PANCREAS, V41, P652, DOI 10.1097/MPA.0b013e31823d7b1e
Wyse JM, 2011, J CLIN ONCOL, V29, P3541, DOI 10.1200/JCO.2010.32.2750
Yamamoto N, 2013, GASTROINTEST ENDOSC, V77, P809, DOI 10.1016/j.gie.2013.01.009
Yang JL, 2011, GASTROINTEST ENDOSC, V73, P1302, DOI 10.1016/j.gie.2011.01.045
Yang J, 2009, GASTROINTEST ENDOSC, V70, P579, DOI 10.1016/j.gie.2009.03.001
Yasuda I, 2013, ENDOSCOPY, V45, P627, DOI 10.1055/s-0033-1344027
Yasuda I, 2009, DIGEST ENDOSC, V21, pS82, DOI 10.1111/j.1443-1661.2009.00875.x
Zheng MW, 2011, HEPATO-GASTROENTEROL, V58, P1106, DOI 10.5754/hge11059
NR 258
TC 99
Z9 123
U1 0
U2 7
PU BAISHIDENG PUBLISHING GROUP INC
PI PLEASANTON
PA 8226 REGENCY DR, PLEASANTON, CA 94588 USA
SN 1007-9327
EI 2219-2840
J9 WORLD J GASTROENTERO
JI World J. Gastroenterol.
PD JUL 14
PY 2014
VL 20
IS 26
BP 8424

EP 8448
DI 10.3748/wjg.v20.i26.8424
PG 25
WC Gastroenterology & Hepatology
WE Science Citation Index Expanded (SCI-EXPANDED)
SC Gastroenterology & Hepatology
GA AL8LF
UT WOS:000339389500009
PM 25024600
OA Green Submitted
DA 2026-05-09
ER

PT J
AU Kunda, R
AF Kunda, Rastislav
TI Recent development of endoscopic ultrasound-guided biliary drainage
SO INTERNATIONAL JOURNAL OF GASTROINTESTINAL INTERVENTION
LA English
DT Review
DE Endoscopic ultrasound; Endoscopic ultrasound-guided antegrade drainage;
Endoscopic ultrasound-guided biliary drainage; Endoscopic
ultrasound-guided choledochoduodenostomy; Endoscopic ultrasound-guided
hepaticogastrostomy
ID METAL STENT; BILE-DUCT; MULTICENTER; OBSTRUCTION; ACCESS
AB Endoscopic ultrasound (EUS)-guided drainage of bile ducts is experiencing widespread introduction into clinical algorithms and utilization of its benefits on regular basis. Various types of procedures, including EUS-guided choledochoduodenostomy, EUS-guided hepaticogastrostomy, EUS-guided antegrade drainage facilitate biliary interventions particularly in those cases when endoscopic retrograde cholangiopancreatography fails or is not possible at all, for example in cases with surgically altered anatomy. Available clinical data are encouraging, but prospective and randomized controlled trials are warranted. The need for development of special tools and accessories seems to be essential for safer spreading of these procedures into algorithms of clinical care. Copyright (C) 2020, Society of Gastrointestinal Intervention.
C1 [Kunda, Rastislav] Vrije Univ Brussel, Univ Ziekenhuis Brussel, Dept Surg, Brussels, Belgium.
[Kunda, Rastislav] Vrije Univ Brussel, Univ Ziekenhuis Brussel, Dept Gastroenterol Hepatol, Brussels, Belgium.
[Kunda, Rastislav] Vrije Univ Brussel, Univ Ziekenhuis Brussel, Dept Adv Intervent Endoscopy, Brussels, Belgium.
C3 University Hospital Brussels; Vrije Universiteit Brussel; Vrije
Universiteit Brussel; University Hospital Brussels; Vrije Universiteit
Brussel; University Hospital Brussels
RP Kunda, R (corresponding author), Univ Ziekenhuis Brussel, Dept Adv Intervent Endoscopy, Laarbeeklaan 101, B-1090 Brussels, Belgium.
EM rkunda@uzbrussel.be
RI Kunda, Rastislav/N-1775-2019

CR Anderloni A, 2019, GASTROINTEST ENDOSC, V89, P69, DOI [10.1016/j.gie.2018.04.1746, 10.1016/j.gie.2018.08.047]
Bang JY, 2018, GASTROINTEST ENDOSC, V88, P9, DOI 10.1016/j.gie.2018.03.012
Burmester E, 2003, GASTROINTEST ENDOSC, V57, P246, DOI 10.1067/mge.2003.85
Chan Shannon Melissa, 2015, Curr Treat Options Gastroenterol, V13, P171, DOI [10.1007/s11938-015-0047-x, 10.1007/s11938-015-0047-x]
Dhir V, 2014, DIGEST ENDOSC, V26, P430, DOI 10.1111/den.12153
DIMAGNO EP, 1980, LANCET, V1, P629
Giovannini M, 2001, ENDOSCOPY, V33, P898, DOI 10.1055/s-2001-17324
Gupta K, 2014, J CLIN GASTROENTEROL, V48, P80, DOI 10.1097/MCG.0b013e31828c6822
HISANAGA K, 1980, AM J ROENTGENOL, V135, P627, DOI 10.2214/ajr.135.3.627
HISANAGA K, 1979, LANCET, V1, P53
Ikeuchi N, 2015, GASTROINTEST ENDOSC, V82, P166, DOI 10.1016/j.gie.2015.01.010
Iwashita T, 2017, DIGEST ENDOSC, V29, P362, DOI 10.1111/den.12800
Khashab MA, 2015, DIGEST DIS SCI, V60, P557, DOI 10.1007/s10620-014-3300-6
Krishnamoorthi R, 2020, SURG ENDOSC, V34, P2866, DOI 10.1007/s00464-020-07484-w
Kunda R, 2019, P 20 BELG SURG WEEK
Kunda R, 2014, P 87 C JAP GASTR END
Kunda R, 2016, SURG ENDOSC, V30, P5002, DOI 10.1007/s00464-016-4845-6
Logiudice FP, 2019, WORLD J GASTRO ENDOS, V11, P281, DOI 10.4253/wjge.v11.i4.281
Ogura T, 2020, ENDOSC ULTRASOUND, V9, P267, DOI 10.4103/eus.eus_38_20
Ogura T, 2019, DIGEST LIVER DIS, V51, P247, DOI 10.1016/j.dld.2018.09.015
Ogura T, 2014, ENDOSCOPY, V46, P416, DOI 10.1055/s-0034-1365020
Paik WH, 2019, ENDOSC ULTRASOUND, V8, pS44, DOI 10.4103/eus.eus_51_19
Paik WH, 2018, AM J GASTROENTEROL, V113, P987, DOI 10.1038/s41395-018-0122-8
Park DH, 2013, GASTROINTEST ENDOSC, V78, P91, DOI 10.1016/j.gie.2013.01.042
Park JK, 2018, GASTROINTEST ENDOSC, V88, P277, DOI 10.1016/j.gie.2018.03.015
STROHM WD, 1980, ENDOSCOPY, V12, P241, DOI 10.1055/s-2007-1021752
Uemura RS, 2018, J CLIN GASTROENTEROL, V52, P123, DOI 10.1097/MCG.0000000000000948
NR 27
TC 0
Z9 0
U1 1
U2 6
PU SOC GASTROINTESTINAL INTERVENTION
PI SEOUL
PA 12, HAKDONG-RO 31, GANGNAM-GU, SEOUL, 00000, SOUTH KOREA
SN 2636-0004
EI 2636-0012
J9 INT J GASTROINT INT
JI Int. J. Gastrointest. Interv.
PD OCT

PY 2020
VL 9
IS 4
BP 142
EP 147
DI 10.18528/ijgii200047
PG 6
WC Gastroenterology & Hepatology
WE Emerging Sources Citation Index (ESCI)
SC Gastroenterology & Hepatology
GA OJ3OQ
UT WOS:000583875100004
OA Green Submitted, gold
DA 2026-05-09
ER

PT J
AU de Nucci, G
Imperatore, N
Picascia, D
Mandelli, ED
Bezzio, C
Arena, I
Omazzi, B
Larghi, A
Manes, G
AF de Nucci, Germana
Imperatore, Nicola
Picascia, Desiree
Mandelli, Enzo Domenico
Bezzio, Cristina
Arena, Ilaria
Omazzi, Barbara
Larghi, Alberto
Manes, Gianpiero
TI Endoscopic ultrasound-guided gallbladder drainage in pancreatic cancer
and cholangitis: A case report
SO WORLD JOURNAL OF GASTROINTESTINAL ENDOSCOPY
LA English
DT Article
DE Gallbladder drainage; Endoscopy ultrasound; Pancreatic cancer;
Cholangitis; Case report; Axios stent

ID BILIARY OBSTRUCTION; METAL STENT; BILE-DUCT

AB BACKGROUND

Head pancreatic cancers often present with clinical challenges requiring biliary drainage for chemotherapy or palliative scope. If usual endoscopic modalities fail or if percutaneous approach is not feasible, endoscopic ultrasound (EUS) guided biliary drainage can be considered. Here we describe and discuss an interesting clinical case in which EUS-guided gallbladder drainage (EUS-GBD) was chosen to treat acute severe cholangitis in a patient with advanced pancreatic cancer.

CASE SUMMARY

An 84-year-old female with a previous EUS-biopsy proven diagnosis of head pancreatic cancer presented with clinical signs of acute cholangitis. In September 2018 she had positioned a biliary and duodenal stent to relieve jaundice and an initial duodenal substenosis. In the emergency ward, an abdominal computed tomography scan showed proximal biliary stent occlusion due to neoplastic progression, but endoscopic retrograde cholangiopancreatography was impossible because of worsening duodenal stenosis and the absence of a chance to reach the Vater's papilla area. EUS-guided choledocoduodenostomy was not technically feasible but because the cystic duct was free of neoplastic infiltration, an EUS-GBD using an Axios (TM) stent was successfully performed. The patient started to feed after 48 h and was discharged 1 wk later. No other hospitalizations due to cholangitis or symptoms of Axios (TM) stent occlusion/dysfunction were observed up until her death 6 mo later due to underlying disease.

CONCLUSION

This case demonstrated how different EUS therapeutic approaches could have a key role to treat critical and seemingly unsolvable situations and that they could play a more fundamental role in the next future.

C1 [de Nucci, Germana; Mandelli, Enzo Domenico; Bezzio, Cristina; Arena, Ilaria; Omazzi, Barbara; Manes, Gianpiero] Garbagnate Milanese Hosp, ASST Rhodense, Dept Gastroenterol, 95 Garbagnate Milanese, I-20024 Milan, Italy.

[Imperatore, Nicola] Cardarelli Hosp, Dept Gastroenterol, I-80131 Naples, Italy.

[Picascia, Desiree] Univ Naples Federico II, Dept Gastroenterol, I-80100 Naples, Italy.

[Larghi, Alberto] Fdn Policlin Univ A Gemelli IRCCS, Digest Endoscopy Unit, I-00168 Rome, Italy.

C3 Antonio Cardarelli Hospital; University of Naples Federico II; Catholic

University of the Sacred Heart; IRCCS Policlinico Gemelli

RP de Nucci, G (corresponding author), Garbagnate Milanese Hosp, ASST Rhodense, Dept Gastroenterol, 95 Garbagnate Milanese, I-20024 Milan, Italy.

EM germanadenucci1@gmail.com

RI Bezzio, Cristina/K-4936-2018; Imperatore, Nicola/ABH-5881-2020; de

Nucci, Germana/ABE-2040-2020; Larghi, Alberto/AAC-7448-2022

OI Larghi, Alberto/0000-0001-7131-3686

CR Anderloni A, 2017, ENDOSC INT OPEN, V5, pE1100, DOI 10.1055/s-0043-118828

Burmester E, 2003, GASTROINTEST ENDOSC, V57, P246, DOI 10.1067/mge.2003.85

Chang JI, 2019, ENDOSC INT OPEN, V7, pE655, DOI 10.1055/a-0826-4309

Imai H, 2016, GASTROINTEST ENDOSC, V84, P147, DOI 10.1016/j.gie.2015.12.024

Itoi T, 2013, DIGEST ENDOSC, V25, P137, DOI 10.1111/den.12084

Iwashita T, 2014, CLIN J GASTROENTEROL, V7, P94, DOI 10.1007/s12328-014-0467-5

Maekawa S, 2013, BMC GASTROENTEROL, V13, DOI 10.1186/1471-230X-13-65

Sharaiha RZ, 2017, GASTROINTEST ENDOSC, V85, P904, DOI 10.1016/j.gie.2016.12.023

Teoh AYB, 2020, GUT, V69, P1085, DOI 10.1136/gutjnl-2019-319996

Will U, 2007, ENDOSCOPY, V39, P292, DOI 10.1055/s-2007-966215

NR 10
TC 0
Z9 0
U1 0
U2 0
PU BAISHIDENG PUBLISHING GROUP INC
PI PLEASANTON
PA 7041 Koll Center Parkway, Suite 160, PLEASANTON, CA, UNITED STATES
SN 1948-5190
J9 WORLD J GASTRO ENDOS
JI World J. Gastrointest. Endosc.
PD NOV 16
PY 2020
VL 12
IS 11
DI 10.4253/wjge.v12.i11.488
PG 5
WC Gastroenterology & Hepatology
WE Emerging Sources Citation Index (ESCI)
SC Gastroenterology & Hepatology
GA PA1SU
UT WOS:000595408900005
PM 33269057
OA Green Accepted, gold
DA 2026-05-09
ER

PT J
AU Wang, GX
Zhang, K
Sun, SY
AF Wang, Guo-Xin
Zhang, Kai
Sun, Si-Yu
TI Retrievable puncture anchor traction method for endoscopic
ultrasound-guided gastroenterostomy: A porcine study
SO WORLD JOURNAL OF GASTROENTEROLOGY
LA English
DT Article
DE Retrievable puncture anchor; Endoscopic ultrasound; Endoscopic
ultrasound-guided gastroenterostomy; Gastric outlet obstruction;
Gastroenterostomy; Electrocautery-enhanced delivery of lumen-apposing

metal stents
ID APPOSING METAL STENT; PANCREATIC FLUID COLLECTIONS; GALLBLADDER DRAINAGE; GASTROJEJUNOSTOMY; DELIVERY; READY
AB BACKGROUND Endoscopic ultrasound-guided gastroenterostomy (EUS-GE) is an alternative method for the surgical treatment of gastric outlet obstruction, but it is regarded as a challenging technique for endoscopists as the bowel is highly mobile and can tent away. Thus, the technique requires superb skill. In order to improve EUS-GE, we have developed a retrievable puncture anchor traction (RPAT) device for EUS-GE to address the issue of bowel tenting. AIM To evaluate the feasibility of RPAT-assisted EUS-GE using an animal model. METHODS Six Bama mini pigs each weighing between 15 and 20 kg underwent the RPAT-assisted EUS-GE procedure. Care was taken to ensure that the animals experienced minimal pain and discomfort. Two days prior to the procedure the animals were limited to a liquid diet. No oral intake was allowed on the day before the procedure. A fully covered metal stent was placed between the stomach and the intestine using the RPAT-assisted EUS-GE method. Infection in the animals was determined. Four weeks after the procedure, a standard gastroscope was inserted into the pig's intestine through a previously created fistula in order to check the status of the stents under anesthesia. The pig was euthanized after examination. RESULTS The RPAT-assisted EUS-GE method allowed placement of the stents with no complications in all six animals. All the pigs tolerated a regular diet within hours of the procedure. The animals were monitored for four weeks after the RPAT-assisted EUS-GE, during which time all of the animals exhibited normal eating behavior and no signs of infection were observed. Endoscopic imaging performed four weeks after the RPAT-assisted EUS-GE showed that the stents remained patent and stable in all the animals. No tissue overgrowth or ingrowth was observed in any case. Each animal had a mature fistula, and the stents were removed without significant bleeding. Autopsies of all six pigs revealed complete adhesion between the intestine and the stomach wall. CONCLUSION The RPAT method helps reduce mobility of the bowel. Therefore, the RPAT-assisted EUS-GE method is a minimally invasive treatment modality.
C1 [Wang, Guo-Xin; Zhang, Kai; Sun, Si-Yu] China Med Univ, Shengjing Hosp, Dept Gastroenterol, Endoscop Ctr, Shenyang 110001, Liaoning, Peoples R China.
C3 China Medical University
RP Sun, SY (corresponding author), China Med Univ, Shengjing Hosp, Dept Gastroenterol, Endoscop Ctr, 36 Sanhao St, Shenyang 110004, Liaoning, Peoples R China.
EM sun-siyu@163.com
RI sun, siyu/AAD-4972-2019
FU China Postdoctoral Science Foundation [2019M661174]; National Natural Science Foundation of China [81770655]; Natural Science Foundation of Liaoning Province [2019-MS359]
FX Supported by the China Postdoctoral Science Foundation, No. 2019M661174; National Natural Science Foundation of China, No. 81770655; and the Natural Science Foundation of Liaoning Province, No. 2019-MS359.
CR Adler DG, 2017, ENDOSC ULTRASOUND, V6, P389, DOI 10.4103/eus.eus_4_17
Binmoeller KF, 2012, ENDOSCOPY, V44, P499, DOI 10.1055/s-0032-1309382
Binmoeller KF, 2011, ENDOSCOPY, V43, P337, DOI 10.1055/s-0030-1256127
Chen YI, 2018, ENDOSC INT OPEN, V6, pE363, DOI 10.1055/s-0043-123468
De Cobelli F, 2017, ENDOSC ULTRASOUND, V6, pS127, DOI 10.4103/eus.eus_77_17
DOBERNECK RC, 1987, ARCH SURG-CHICAGO, V122, P827
Fritscher-Ravens A, 2002, GASTROINTEST ENDOSC, V56, P737, DOI 10.1067/mge.2002.129084

Giovannini M, 2017, ENDOSC ULTRASOUND, V6, P281, DOI 10.4103/eus.eus_86_17
Irani S, 2017, CURR OPIN GASTROEN, V33, P320, DOI 10.1097/MOG.0000000000000389
Itoi T, 2016, GUT, V65, P193, DOI 10.1136/gutjnl-2015-310348
Itoi T, 2015, J HEPATO-BIL-PAN SCI, V22, P3, DOI 10.1002/jhbp.148
Itoi T, 2013, GASTROINTEST ENDOSC, V78, P934, DOI 10.1016/j.gie.2013.09.025
Itoi T, 2012, GASTROINTEST ENDOSC, V75, P870, DOI 10.1016/j.gie.2011.10.020
Khashab M, 2013, SURG ENDOSC, V27, P2068, DOI 10.1007/s00464-012-2712-7
Manvar A, 2017, ENDOSC ULTRASOUND, V6, P217, DOI 10.4103/eus.eus_46_17
Rimbas Mihai, 2017, Gastrointest Endosc Clin N Am, V27, P681, DOI [10.1016/j.giec.2017.06.006, 10.1016/j.giec.2017.06.006]
Rimbas M, 2017, ENDOSC ULTRASOUND, V6, P235, DOI 10.4103/eus.eus_47_17
Saumoy M, 2017, ENDOSC ULTRASOUND, V6, pS132, DOI 10.4103/eus.eus_80_17
Telford JJ, 2004, GASTROINTEST ENDOSC, V60, P916, DOI 10.1016/S0016-5107(04)02228-X
Teoh AYB, 2014, GASTROINTEST ENDOSC, V80, P1171, DOI 10.1016/j.gie.2014.03.038
van Hooft JE, 2009, GASTROINTEST ENDOSC, V69, P1059, DOI 10.1016/j.gie.2008.07.026
Zhang K, 2018, GASTROINTEST ENDOSC, V88, P957, DOI 10.1016/j.gie.2018.07.019
NR 22
TC 5
Z9 9
U1 2
U2 13
PU BAISHIDENG PUBLISHING GROUP INC
PI PLEASANTON
PA 7041 Koll Center Parkway, Suite 160, PLEASANTON, CA, UNITED STATES
SN 1007-9327
EI 2219-2840
J9 WORLD J GASTROENTERO
JI World J. Gastroenterol.
PD JUL 7
PY 2020
VL 26
IS 25
BP 3603
EP 3610
DI 10.3748/wjg.v26.i25.3603
PG 8
WC Gastroenterology & Hepatology
WE Science Citation Index Expanded (SCI-EXPANDED)
SC Gastroenterology & Hepatology
GA MS7KD
UT WOS:000554453400007
PM 32742129

OA Green Accepted, hybrid
DA 2026-05-09
ER

PT J
AU Itoi, T
Binmoeller, K
Itokawa, F
Umeda, J
Tanaka, R
AF Itoi, Takao
Binmoeller, Kenneth
Itokawa, Fumihide
Umeda, Junko
Tanaka, Reina
TI Endoscopic ultrasonography-guided cholecystogastrostomy using a
lumen-apposing metal stent as an alternative to extrahepatic bile duct
drainage in pancreatic cancer with duodenal invasion
SO DIGESTIVE ENDOSCOPY
LA English
DT Article
DE endoscopic ultrasonography (EUS); gallbladder drainage; lumen-apposing
self-expandable metallic stent (SEMS)
ID GALLBLADDER DRAINAGE
AB Various approaches to biliary drainage, especially endoscopic ultrasonography (EUS)-guided drainage, have become commonly used as an alternative method for biliary decompression for malignant biliary obstruction. Occasionally, however, duodenal obstruction and non-dilated intrahepatic bile duct impede conventional EUS-guided biliary drainage. Herein, we describe a case of cholecystogastrostomy successfully carried out using a newly developed fully covered lumen-apposing self-expandable metallic stent (SEMS). EUS-guided cholecystogastrostomy should be considered an option for biliary decompression. This is aparticularly ideal alternative if the patient has duodenal strictures with or without a duodenal metal stent and a non-dilated intrahepatic bile duct, which suggests the impossibility of choledochoduodenostomy and hepaticogastrostomy. Furthermore, the newly developed fully covered lumen-apposing SEMS seems ideal for EUS-guided cholecystoenterostomy.
C1 [Itoi, Takao; Itokawa, Fumihide; Umeda, Junko; Tanaka, Reina] Tokyo Med Univ, Dept Gastroenterol & Hepatol, Tokyo 1600023, Japan.
[Binmoeller, Kenneth] Calif Pacific Med Ctr, Intervent Endoscopy Serv, San Francisco, CA USA.
C3 Tokyo Medical University; California Pacific Medical Center
RP Itoi, T (corresponding author), Tokyo Med Univ, Dept Gastroenterol & Hepatol, Shinjuku Ku, 6-7-1 Nishishinjuku, Tokyo 1600023, Japan.
EM itoi@tokyo-med.ac.jp
CR Itoi T, 2012, GASTROINTEST ENDOSC, V75, P870, DOI 10.1016/j.gie.2011.10.020
Itoi T, 2011, J HEPATO-BIL-PAN SCI, V18, P664, DOI 10.1007/s00534-011-0410-9

Itoi T, 2011, J HEPATO-BIL-PAN SCI, V18, P282, DOI 10.1007/s00534-010-0310-4
Itoi T, 2010, J HEPATO-BIL-PAN SCI, V17, P611, DOI 10.1007/s00534-009-0196-1
Itoi T, 2010, GASTROINTEST ENDOSC, V71, P1038, DOI 10.1016/j.gie.2010.01.026
Jang JW, 2011, GASTROINTEST ENDOSC, V74, P176, DOI 10.1016/j.gie.2011.03.1120
Seewald S, 2012, DIGEST ENDOSC, V24, P36, DOI 10.1111/j.1443-1661.2011.01162.x
NR 7
TC 32
Z9 41
U1 1
U2 2
PU WILEY-BLACKWELL
PI HOBOKEN
PA 111 RIVER ST, HOBOKEN 07030-5774, NJ USA
SN 0915-5635
J9 DIGEST ENDOSC
JI Dig. Endosc.
PD MAY
PY 2013
VL 25
SU 2
SI SI
BP 137
EP 141
DI 10.1111/den.12084
PG 5
WC Gastroenterology & Hepatology; Surgery
WE Science Citation Index Expanded (SCI-EXPANDED)
SC Gastroenterology & Hepatology; Surgery
GA 137KK
UT WOS:000318434500027
PM 23617665
OA Bronze
DA 2026-05-09
ER

PT J
AU Almadi, MA
Eltayeb, M
Thaniah, S
AF Almadi, Majid A.
Eltayeb, Mohanned
Thaniah, Salem

TI Endoscopic Ultrasound-guided Antegrade Stenting in an Occluded Biliary
Self-expandable Metal Stent
SO SAUDI JOURNAL OF GASTROENTEROLOGY
LA English
DT Article
DE Biliary drainage; endoscopic ultrasonography; endoscopic
ultrasound-guided biliary drainage; self-expandable metal stents; stents
ID PLASTIC STENTS; OBSTRUCTION; METAANALYSIS; MULTICENTER; PLACEMENT
AB Endoscopic ultrasound-guided biliary drainage (EUS-BD) is an attractive option for patients who cannot undergo conventional endoscopic retrograde cholangiopancreatography (ERCP) and do not want surgery or percutaneous drainage procedures. We present the use of EUS-antegrade (EUS-AG) insertion of a self-expandable metal stent (SEMS) in a patient with a common hepatic duct cholangiocarcinoma, as well as a huge gastric lipoma, after recurrent biliary obstruction of a prior SEMS inserted via ERCP in the same session as a duodenal stent insertion for gastric outlet obstruction.
C1 [Almadi, Majid A.; Eltayeb, Mohanned; Thaniah, Salem] King Saud Univ, King Khalid Univ Hosp, Div Gastroenterol, Riyadh, Saudi Arabia.
[Almadi, Majid A.] McGill Univ, Montreal Gen Hosp, Ctr Hlth, Div Gastroenterol, Montreal, PQ, Canada.
C3 King Saud University; King Khalid University Hospital; McGill University
RP Almadi, MA (corresponding author), King Saud Univ, King Saud Univ Med City, Div Gastroenterol, Dept Med, POB 2925 59, Riyadh 11461, Saudi Arabia.
EM maalmadi@ksu.edu.sa
RI Almadi, Majid/KIA-2074-2024
OI Almadi, Majid/0000-0001-9917-2758
FU College of Medicine Research Center, Deanship of Scientific Research,
King Saud University [RGP-279]
FX College of Medicine Research Center, Deanship of Scientific Research,
King Saud University through the Research Group Project number RGP-279.
CR Almadi Majid A, 2015, Gastrointest Endosc Clin N Am, V25, P691, DOI [10.1016/j.giec.2015.06.002, 10.1016/j.giec.2015.06.002]
Almadi MA, 2013, CLIN GASTROENTEROL H, V11, P27, DOI 10.1016/j.cgh.2012.10.019
Barkun AN, 2015, CAN J GASTROENTEROL, V29, P377, DOI 10.1155/2015/743417
Dhir V, 2015, GASTROINTEST ENDOSC, V81, P913, DOI 10.1016/j.gie.2014.09.054
Dhir V, 2014, DIGEST ENDOSC, V26, P430, DOI 10.1111/den.12153
Sawas T, 2015, GASTROINTEST ENDOSC, V82, P256, DOI 10.1016/j.gie.2015.03.1980
Shah T, 2012, DIGEST DIS SCI, V57, P2765, DOI 10.1007/s10620-012-2272-7
NR 7
TC 2
Z9 2
U1 0
U2 2
PU MEDKNOW PUBLICATIONS & MEDIA PVT LTD
PI MUMBAI
PA B-9, KANARA BUSINESS CENTRE, OFF LINK RD, GHAKTOPAR-E, MUMBAI, 400075,

INDIA
SN 1319-3767
EI 1998-4049
J9 SAUDI J GASTROENTERO
JI Saudi J. Gastroenterol.
PD JUL-AUG
PY 2016
VL 22
IS 4
BP 337
EP 340
DI 10.4103/1319-3767.187597
PG 4
WC Gastroenterology & Hepatology
WE Science Citation Index Expanded (SCI-EXPANDED)
SC Gastroenterology & Hepatology
GA DW6VZ
UT WOS:000383790800013
PM 27488330
OA Green Submitted, gold
DA 2026-05-09
ER

PT J
AU Venkatachalapathy, SV
James, MW
Huggett, MT
Paranandi, B
Pereira, SP
Johnson, G
Aravinthan, AD
Aithal, GP
AF Venkatachalapathy, Suresh Vasan
James, Martin W.
Huggett, Matthew T.
Paranandi, Bharat
Pereira, Stephen P.
Johnson, Gavin
Aravinthan, Aloysious D.
Aithal, Guruprasad P.
TI Utility of palliative EUS-guided biliary drainage using lumen-apposing
metal stents: a prospective multicenter feasibility study (with video)

SO GASTROINTESTINAL ENDOSCOPY

LA English

DT Article

ID ENDOSCOPIC ULTRASOUND; ADVERSE EVENTS; CANCER; ERCP; OBSTRUCTION; CHOLANGIOCARCINOMA; CHEMOTHERAPY; PREDICTORS; JAUNDICE

AB Background and Aims: Biliary drainage with ERCP is successful in only 80% to 90% of cases of extrahepatic cholangiocarcinoma and pancreatic cancer. We present the results of a multicenter prospective study assessing the safety, feasibility, and quality of life of patients after EUS-guided biliary drainage (EUS-BD) with lumenapposing metal stents after failed ERCP.

Methods: All consecutive adults with a dilated common bile duct (CBD) >= 14 mm secondary to inoperable malignant distal CBD stricture and failed ERCP biliary drainage were screened and recruited from 3 tertiary UK centers. Technical success of EUS-BD using lumen-apposing metal stents was the primary endpoint. Improvement in serum bilirubin level, 30-day mortality, procedure-related adverse events, and quality of life were secondary endpoints. Improvement in quality of life was measured using a validated questionnaire (EORTC QLQ-BIL21).

Results: Twenty patients were included in the analysis. EUS-BD was technically successful in all patients and the clinical success rate was 95% (19 of 20) at day 7 (>50% reduction in bilirubin level) and 92.3% (12 of 13) at day 30 (bilirubin <50 mmol/L). There were significant improvements in overall quality of life score (49 vs 42, P Z.03) at day 30. All-cause 30-day mortality was 20% and the moderate adverse event rate was 10% (1 cholangitis and 1 stent migration).

Conclusion: EUS-BD has acceptable technical success and safety as a second-line palliative treatment for inoperable malignant distal CBD strictures. Randomized controlled studies comparing EUS-BD with percutaneous transhepatic biliary drainage are needed to determine their effectiveness in clinical practice.

C1 [Venkatachalapathy, Suresh Vasan; James, Martin W.; Aravinthan, Aloysious D.; Aithal, Guruprasad P.] Nottingham Univ Hosp NHS Trust, NIHR Nottingham Biomed Res Ctr, Derby Rd, Nottingham NG7 2UH, England.

[Venkatachalapathy, Suresh Vasan; James, Martin W.; Aravinthan, Aloysious D.; Aithal, Guruprasad P.] Univ Nottingham, Derby Rd, Nottingham NG7 2UH, England.

[Huggett, Matthew T.; Paranandi, Bharat] Leeds Teaching Hosp NHS Trust, Gastroenterol, Leeds, W Yorkshire, England.

[Pereira, Stephen P.] UCL, UCL Inst Liver & Digest Hlth, London, England.

[Pereira, Stephen P.; Johnson, Gavin] Univ Coll Hosp NHS Fdn Trust, Dept Gastroenterol, London, England.

[Aravinthan, Aloysious D.; Aithal, Guruprasad P.] Univ Nottingham, Sch Med, Nottingham Digest Dis Ctr, Nottingham, England.

C3 Nottingham University Hospital NHS Trust; University of Nottingham; University of Leeds; University of London; University College London; University of London; University College London; University of Nottingham

RP Venkatachalapathy, SV (corresponding author), Nottingham Univ Hosp NHS Trust, NIHR Nottingham Biomed Res Ctr, Derby Rd, Nottingham NG7 2UH, England.; Venkatachalapathy, SV (corresponding author), Univ Nottingham, Derby Rd, Nottingham NG7 2UH, England.

RI ; Aravinthan, Aloysious/I-2557-2019; Pereira, Stephen/C-5639-2009

OI James, Martin/0000-0002-6438-8484; Aithal, Guruprasad/0000-0003-3924-4830; Aravinthan, Aloysious/0000-0003-0527-5137; Pereira, Stephen/0000-0003-0821-1809

FU National Institute for Health Research Clinical Research Network (NIHR
CRN); NIHR Nottingham Biomedical Research Centre (CPMS) [38822];
Nottingham University Hospitals NHS Trust; University of Nottingham,
Nottingham, United Kingdom; UCLH/UCL Comprehensive Biomedical Centre
from Department of Health's NIHR Biomedical Research Centres funding
scheme; Boston Scientific; Pancreatic Cancer UK [PCUK-2018-EDA Pereira]
Funding Source: researchfish
FX The research team acknowledges the support of the National Institute for
Health Research Clinical Research Network (NIHR CRN), NIHR Nottingham
Biomedical Research Centre (CPMS ID 38822), Nottingham University
Hospitals NHS Trust and the University of Nottingham, Nottingham, United
Kingdom, for the study. S.P.P. was partly supported by the UCLH/UCL
Comprehensive Biomedical Centre which receives a proportion of funding
from the Department of Health's NIHR Biomedical Research Centres funding
scheme. Boston Scientific funded in kind by reducing the price of the
stents. This was an investigator led study and neither NIHR Nottingham
Biomedical Research Centre nor Boston Scientific had a role in the
design, recruitment, analysis, and delivery of the study.
CR Abraham NS, 2002, GASTROINTEST ENDOSC, V56, P835, DOI 10.1067/mge.2002.129868
Anderloni A, 2019, GASTROINTEST ENDOSC, V89, P69, DOI [10.1016/j.gie.2018.04.1746, 10.1016/j.gie.2018.08.047]
Cotton PB, 2010, GASTROINTEST ENDOSC, V71, P446, DOI 10.1016/j.gie.2009.10.027
Dumonceau JM, 2012, ENDOSCOPY, V44, P277, DOI 10.1055/s-0031-1291633
Ekkelenkamp VE, 2015, ENDOSCOPY, V47, P504, DOI 10.1055/s-0034-1391231
Eklund JW, 2005, ONCOLOGY-NY, V19, P1057
Gatto M, 2010, EUR REV MED PHARMACO, V14, P363
Gillen S, 2010, PLOS MED, V7, DOI 10.1371/journal.pmed.1000267
Giovannini M, 2001, ENDOSCOPY, V33, P898, DOI 10.1055/s-2001-17324
Hedjoudje A, 2019, UNITED EUR GASTROENT, V7, P60, DOI 10.1177/2050640618808147
Hidalgo M, 2010, NEW ENGL J MED, V362, P1605, DOI 10.1056/NEJMra0901557
HOLLAND JC, 1986, AM J PSYCHIAT, V143, P982
Jacques J, 2019, ENDOSCOPY, V51, P540, DOI 10.1055/a-0735-9137
Kaupp-Roberts SD, 2016, BRIT J CANCER, V115, P1032, DOI 10.1038/bjc.2016.284
Kawarabayashi N, 2010, SHOCK, V33, P500, DOI 10.1097/SHK.0b013e3181c4e44a
Khashab MA, 2016, ENDOSC INT OPEN, V4, pE1322, DOI 10.1055/s-0042-110790
Khorana AA, 2013, CANCER-AM CANCER SOC, V119, P648, DOI 10.1002/cncr.27772
Kunda R, 2016, SURG ENDOSC, V30, P5002, DOI 10.1007/s00464-016-4845-6
Larsen AC, 2015, J THROMB HAEMOST, V13, P555, DOI 10.1111/jth.12843
Mayr M, 2010, DIGESTION, V82, P1, DOI 10.1159/000272422
Nehéz S, 2002, EUR J SURG, V168, P315, DOI 10.1080/11024150260284815
Park DH, 2011, GASTROINTEST ENDOSC, V74, P1276, DOI 10.1016/j.gie.2011.07.054
Roy M, 2019, BRIT J RADIOL, V92, DOI 10.1259/bjr.20180814

Sharaiha RZ, 2017, GASTROINTEST ENDOSC, V85, P904, DOI 10.1016/j.gie.2016.12.023
Strasberg SM, 2014, HPB, V16, P150, DOI 10.1111/hpb.12094
Tsuchiya T, 2018, GASTROINTEST ENDOSC, V87, P1138, DOI 10.1016/j.gie.2017.08.017
Venkatachalapathy SV, 2018, ENDOSC INT OPEN, V6, pE259, DOI 10.1055/s-0043-125362
Wani S, 2017, CLIN GASTROENTEROL H, V15, P1758, DOI 10.1016/j.cgh.2017.06.012
NR 28
TC 15
Z9 15
U1 0
U2 2
PU MOSBY-ELSEVIER
PI NEW YORK
PA 360 PARK AVENUE SOUTH, NEW YORK, NY 10010-1710 USA
SN 0016-5107
EI 1097-6779
J9 GASTROINTEST ENDOSC
JI Gastrointest. Endosc.
PD AUG
PY 2021
VL 94
IS 2
BP 321
EP 328
DI 10.1016/j.gie.2021.01.029
EA JUL 2021
PG 8
WC Gastroenterology & Hepatology
WE Science Citation Index Expanded (SCI-EXPANDED)
SC Gastroenterology & Hepatology
GA TI4UH
UT WOS:000672795700015
PM 33545136
DA 2026-05-09
ER

PT J
AU Dietrich, CF
Braden, B
Burmeister, S
Aabakken, L
Arciadacono, PG
Bhutani, MS

Götzberger, M
Healey, AJ
Hocke, M
Hollerbach, S
Ignee, A
Jenssen, C
Jürgensen, C
Larghi, A
Moeller, K
Napoléon, B
Rimbas, M
Saftoiu, A
Sun, SY
Teoh, AYB
Vanella, G
Fusaroli, P
Carrara, S
Will, U
Dong, Y
Burmester, E
AF Dietrich, Christoph F.
Braden, Barbara
Burmeister, Sean
Aabakken, Lars
Arciadacono, Paolo Giorgio
Bhutani, Manoop S.
Gotzberger, Manuela
Healey, Andrew J.
Hocke, Michael
Hollerbach, Stephan
Ignee, Andre
Jenssen, Christian
Jurgensen, Christian
Larghi, Alberto
Moeller, Kathleen
Napoleon, Bertrand
Rimbas, Mihai
Saftoiu, Adrian
Sun, Siyu
Teoh, Anthony Yuen Bun
Vanella, Giuseppe
Fusaroli, Pietro

Carrara, Silvia
Will, Uwe
Dong, Yi
Burmester, Eike
TI How to perform EUS-guided biliary drainage
SO ENDOSCOPIC ULTRASOUND
LA English
DT Review
DE acute cholecystitis; ERCP; EUS; EUS-guided biliary drainage; EUS-guided gallbladder drainage; obstructive jaundice
ID METAL STENT; ENDOSCOPIC ULTRASONOGRAPHY; PERCUTANEOUS DRAINAGE; GALLBLADDER DRAINAGE; DELIVERY-SYSTEM; ADVERSE EVENTS; FAILED ERCP; BILE-DUCT; HEPATICOGASTROSTOMY; MULTICENTER
AB EUS-guided biliary drainage (EUS-BD) has recently gained widespread acceptance as a minimally invasive alternative method for biliary drainage. Even in experienced endoscopy centers, ERCP may fail due to inaccessibility of the papillary region, altered anatomy (particularly postsurgical alterations), papillary obstruction, or neoplastic gastric outlet obstruction. Biliary cannulation fails at first attempt in 5%-10% of cases even in the absence of these factors. In such cases, alternative options for biliary drainage must be provided since biliary obstruction is responsible for poor quality of life and even reduced survival, particularly due to septic cholangitis. The standard of care in many centers remains percutaneous transhepatic biliary drainage (PTBD). However, despite the high technical success rate with experienced operators, the percutaneous approach is more invasive and associated with poor quality of life. PTBD may result in long-term external catheters for biliary drainage and carry the risk of serious adverse events (SAEs) in up to 10% of patients, including bile leaks, hemorrhage, and sepsis. PTBD following a failed ERCP also requires scheduling a second procedure, resulting in prolonged hospital stay and additional costs. EUS-BD may overcome many of these limitations and offer some distinct advantages in accessing the biliary tree. Current data suggest that EUS-BD is safe and effective when performed by experts, although SAEs have been also reported. Despite the high number of clinical reports and case series, high-quality comparative studies are still lacking. The purpose of this article is to report on the current status of this procedure and to discuss the tools and techniques for EUS-BD in different clinical scenarios.
C1 [Dietrich, Christoph F.] Zhengzhou Univ, Dept Ultrasound, Affiliated Hosp 1, Zhengzhou, Peoples R China.
[Dietrich, Christoph F.] Hirslanden Beau Site, Dept Allgemeine Innere Med Kliniken DAIM, Bern, Switzerland.
[Braden, Barbara] Oxford Univ Hosp, Translat Gastroenterol Unit, Oxford, England.
[Burmeister, Sean] Groote Schuur Hosp, Hepatopancreat Biliary Unit, Cape Town, South Africa.
[Aabakken, Lars] Oslo Univ Hosp, Dept Transplantat Med, Oslo, Norway.
[Arciadacono, Paolo Giorgio; Vanella, Giuseppe] Univ Vita Salute San Raffaele, IRCCS San Raffaele Sci Inst, Div Pancreatobiliary Endoscopy & Endosonog, Milan, Italy.
[Bhutani, Manoop S.] UTMD Anderson Canc Ctr, Dept Gastroenterol Hepatol & Nutr, Houston, TX USA.
[Gotzberger, Manuela] Munchen Klin Neuperlach & Harlaching, Dept Gastroenterol & Hepatol, Munich, Germany.
[Healey, Andrew J.] Royal Infirm Edinburgh NHS Trust, Edinburgh, Midlothian, Scotland.
[Hocke, Michael] Helios Klinikum Meiningen, Med Dept 2, Meiningen, Germany.
[Hollerbach, Stephan] Allgemeines Krankenhaus Celle, Dept Gastroenterol, Celle, Germany.
[Ignee, Andre] Caritas Krankenhaus, Bad Mergentheim, Germany.
[Jenssen, Christian] Med Univ Brandenburg, Strausberg & Brandenburg Inst Clin Ultrasound, Krankenhaus Maerk

Oderland, Med Dept, Neuruppin, Germany.
[Jurgensen, Christian] Charite, Dept Gastroenterol, Berlin, Germany.
[Larghi, Alberto] Fdn Policlin Univ A Gemelli IRCCS, Digest Endoscopy Unit, Rome, Italy.
[Moeller, Kathleen] SANA Hosp Lichtenberg, Dept Med Gastroenterol 1, Berlin, Germany.
[Napoleon, Bertrand] Hop Prive J Mermoz Ramsay Gen Sante, Lyon, France.
[Rimbas, Mihai] Carol Davila Univ Med, Colentina Clin Hosp, Clin Internal Med, Dept Gastroenterol, Bucharest, Romania.
[Saftoiu, Adrian] Univ Med & Pharm Craiova, Res Ctr Gastroenterol & Hepatol, Dept Gastroenterol, Craiova, Romania.
[Sun, Siyu] Shengjing Hosp China Med Univ, Dept Endoscopy Ctr, Shenyang, Liaoning, Peoples R China.
[Teoh, Anthony Yuen Bun] Chinese Univ Hong Kong, Prince Wales Hosp, Dept Surg, Hong Kong, Peoples R China.
[Fusaroli, Pietro] Univ Bologna, Hosp Imola, Gastrointestinal Unit, Dept Med & Surg Sci, Imola, Italy.
[Carrara, Silvia] Humanitas Clin & Res Ctr, Digest Endoscopy Unit, Div Gastroenterol, Milan, Italy.
[Will, Uwe] SRH Klinikum Gera, Dept Gastroenterol, Gera, Germany.
[Dong, Yi] Fudan Univ, Zhongshan Hosp, Dept Ultrasound, Shanghai, Peoples R China.
[Burmester, Eike] Sana Kliniken Luebeck, Med Klin 1, Lubeck, Germany.
C3 Zhengzhou University; University of Cape Town; University of Oslo;
Vita-Salute San Raffaele University; IRCCS Ospedale San Raffaele;
University of Texas System; UTMD Anderson Cancer Center; University of
Edinburgh; Royal Infirmary of Edinburgh; Helios Kliniken; Caritas
Hospital Bad Mergentheim; Free University of Berlin; Humboldt University
of Berlin; Charite Universitatsmedizin Berlin; Catholic University of
the Sacred Heart; IRCCS Policlinico Gemelli; Carol Davila University of
Medicine & Pharmacy; University of Medicine & Pharmacy of Craiova;
Chinese University of Hong Kong; Prince of Wales Hospital Hong Kong;
University of Bologna; Azienda USL di Imola; Fudan University
RP Dietrich, CF (corresponding author), Kliniken Hirslanden Beau Site, Dept Allgemeine Innere Med, Bern, Switzerland.
EM c.f.dietrich@googlemail.com
RI 孙, 思宇/GQY-4400-2022; Braden, Barbara/AAH-8693-2021; Larghi,
Alberto/AAC-7448-2022; Fusaroli, Pietro/AAC-2182-2020; Rimbas,
Mihai/AAF-5521-2020; Saftoiu, Adrian/C-2792-2011; Burmeister,
Sean/IQW-5871-2023; Vanella, Giuseppe/LQI-8930-2024; Dietrich, Christoph
Frank/AFQ-4722-2022; Teoh, Anthony/L-4796-2016; Carrara,
Silvia/AAC-5298-2022; Juergensen, Christian/HSB-2094-2023
OI Burmeister, Sean/0000-0003-0888-7606; Healey, Anew
James/0000-0001-9297-9401; Dietrich, Christoph
Frank/0000-0001-6015-6347; Möller, Kathleen/0009-0002-9492-0141;
Carrara, Silvia/0000-0003-4206-9463; Juergensen,
Christian/0000-0002-0540-822X
CR Alvarez-Sanchez MV, 2014, SURG ENDOSC, V28, P712, DOI 10.1007/s00464-013-3260-5
Artifon ELA, 2015, GASTROINTEST ENDOSC, V81, P950, DOI 10.1016/j.gie.2014.09.047
Attili F, 2017, ENDOSC ULTRASOUND, V6, P402, DOI 10.4103/eus.eus_54_17
Attili F, 2015, ENDOSCOPY, V47, pE619, DOI 10.1055/s-0034-1393669

Bang JY, 2019, GASTROENTEROLOGY, V156, P1027, DOI 10.1053/j.gastro.2018.11.031

Binmoeller KF, 2011, ENDOSCOPY, V43, P337, DOI 10.1055/s-0030-1256127

Bories E, 2007, ENDOSCOPY, V39, P287, DOI 10.1055/s-2007-966212

Burmester E, 2003, GASTROINTEST ENDOSC, V57, P246, DOI 10.1067/mge.2003.85

Chan SM, 2017, GASTROINTEST ENDOSC, V85, P1225, DOI 10.1016/j.gie.2016.10.014

Cho DH, 2017, GASTROINTEST ENDOSC, V85, P1067, DOI 10.1016/j.gie.2016.09.010

Artifon ELD, 2019, ENDOSC ULTRASOUND, V8, pS72, DOI 10.4103/eus.eus_62_19

De Cassan C, 2017, ENDOSC ULTRASOUND, V6, P329, DOI 10.4103/2303-9027.209869

Dhir V, 2014, DIGEST ENDOSC, V26, P430, DOI 10.1111/den.12153

El Chafic AH, 2019, DIGEST DIS SCI, V64, P3321, DOI 10.1007/s10620-019-05688-2

Fueldner F, 2020, Z GASTROENTEROL, V58, P1081, DOI 10.1055/a-1250-8834

Fusaroli P, 2016, ULTRASCHALL MED, V37, P408, DOI 10.1055/s-0035-1553742

Fusaroli P, 2016, ULTRASCHALL MED, V37, pE77, DOI 10.1055/s-0035-1553738

Giovannini M, 2001, ENDOSCOPY, V33, P898, DOI 10.1055/s-2001-17324

Guo JT, 2018, ENDOSC ULTRASOUND, V7, P356, DOI 10.4103/eus.eus_53_18

Jang JW, 2012, GASTROENTEROLOGY, V142, P805, DOI 10.1053/j.gastro.2011.12.051

Khan MA, 2016, DIGEST DIS SCI, V61, P684, DOI 10.1007/s10620-015-3933-0

Khashab MA, 2016, ENDOSC INT OPEN, V4, pE175, DOI 10.1055/s-0041-109083

Kim YS, 2010, ENDOSCOPY, V42, P496, DOI 10.1055/s-0029-1244082

Krishnamoorthi R, 2020, SURG ENDOSC, V34, P2866, DOI 10.1007/s00464-020-07484-w

Kunda R, 2016, SURG ENDOSC, V30, P5002, DOI 10.1007/s00464-016-4845-6

Lakhtakia Sundeep, 2020, VideoGIE, V5, P359, DOI [10.1016/j.vgie.2020.04.023, 10.1016/j.vgie.2020.04.023]

Laquière A, 2019, ENDOSCOPY, V51, P436, DOI 10.1055/a-0757-7714

Law R, 2016, CLIN GASTROENTEROL H, V14, P476, DOI 10.1016/j.cgh.2015.10.026

Lee TH, 2016, CLIN GASTROENTEROL H, V14, P1011, DOI 10.1016/j.cgh.2015.12.032

Luk SWY, 2019, ENDOSCOPY, V51, P722, DOI 10.1055/a-0929-6603

Maehara K, 2020, ENDOSC INT OPEN, V08, pE1034, DOI 10.1055/a-1169-3749

Martínez B, 2019, ENDOSC INT OPEN, V7, pE1038, DOI 10.1055/a-0918-5931

Minaga K, 2019, DIGEST ENDOSC, V31, P575, DOI 10.1111/den.13406

Mohan BP, 2019, J CLIN GASTROENTEROL, V53, P243, DOI 10.1097/MCG.0000000000001167

Mukai S, 2019, GASTROINTEST ENDOSC, V89, P399, DOI 10.1016/j.gie.2018.07.030

Ogura T, 2016, ENDOSCOPY, V48, P156, DOI 10.1055/s-0034-1392859

Oh D, 2017, THER ADV GASTROENTER, V10, P42, DOI 10.1177/1756283X16671671

Okuno N, 2018, J GASTROEN HEPATOL, V33, P1413, DOI 10.1111/jgh.14112

Paik WH, 2019, ENDOSC ULTRASOUND, V8, pS44, DOI 10.4103/eus.eus_51_19

Paik WH, 2018, AM J GASTROENTEROL, V113, P987, DOI 10.1038/s41395-018-0122-8

Park DH, 2011, GASTROINTEST ENDOSC, V74, P1276, DOI 10.1016/j.gie.2011.07.054

Pizzicannella M, 2020, ENDOSC ULTRASOUND, V9, P45, DOI 10.4103/eus.eus_55_19

Podboy A, 2021, GASTROINTEST ENDOSC, V93, P797, DOI 10.1016/j.gie.2020.09.040

Rimbas M, 2015, GASTROINTEST ENDOSC, V82, P405, DOI 10.1016/j.gie.2015.03.1907

Ryou M, 2020, GASTROINTEST ENDOSC, V91, P178, DOI 10.1016/j.gie.2019.07.035

Saftoiu A, 2013, CHIRURGIA-BUCHAREST, V108, P62

Sakai Y, 2017, WORLD J GASTROINTEST, V9, P77, DOI 10.4253/wjge.v9.i2.77
Saumoy M, 2019, J CLIN GASTROENTEROL, V53, P231, DOI 10.1097/MCG.0000000000001036
Sharma M, 2013, ENDOSC ULTRASOUND, V2, P171, DOI [10.4103/2303-9027.117679, 10.7178/eus.06.0013]
Suenaga S, 2021, ENDOSC ULTRASOUND, V10, P387, DOI 10.4103/EUS-D-20-00225
Tang Zhouwen, 2016, Diagn Ther Endosc, V2016, P3125962, DOI 10.1155/2016/3125962
Teoh AYB, 2020, GUT, V69, P1085, DOI 10.1136/gutjnl-2019-319996
Teoh AYB, 2021, GASTROINTEST ENDOSC, V93, P577, DOI 10.1016/j.gie.2020.06.066
Yuste RT, 2019, ANN GASTROENTEROL, V32, P514, DOI 10.20524/aog.2019.0395
Uemura RS, 2018, J CLIN GASTROENTEROL, V52, P123, DOI 10.1097/MCG.0000000000000948
van Brunschot S, 2018, LANCET, V391, P51, DOI 10.1016/S0140-6736(17)32404-2
Vanella G, 2020, ENDOSC INT OPEN, V08, pE1782, DOI 10.1055/a-1264-7511
Varadarajulu S, 2012, GASTROINTEST ENDOSC, V76, P336, DOI 10.1016/j.gie.2012.04.455
Vila JJ, 2012, GASTROINTEST ENDOSC, V76, P1133, DOI 10.1016/j.gie.2012.08.001
Wang KX, 2016, GASTROINTEST ENDOSC, V83, P1218, DOI 10.1016/j.gie.2015.10.033
Yamamoto Y, 2020, ENDOSC ULTRASOUND, V9, P110, DOI 10.4103/eus.eus_68_19
NR 61
TC 33
Z9 38
U1 2
U2 11
PU WOLTERS KLUWER MEDKNOW PUBLICATIONS
PI MUMBAI
PA WOLTERS KLUWER INDIA PVT LTD , A-202, 2ND FLR, QUBE, C T S NO 1498A-2
VILLAGE MAROL, ANDHERI EAST, MUMBAI, Maharashtra, INDIA
SN 2303-9027
EI 2226-7190
J9 ENDOSC ULTRASOUND
JI Endosc. Ultrasound
PD SEP-OCT
PY 2022
VL 11
IS 5
BP 342
EP 354
DI 10.4103/EUS-D-21-00188
PG 13
WC Gastroenterology & Hepatology
WE Science Citation Index Expanded (SCI-EXPANDED)
SC Gastroenterology & Hepatology
GA 5U8KV
UT WOS:000876791100002
PM 36255022

OA Green Submitted
DA 2026-05-09
ER

PT J
AU Adler, DG
Taylor, LJ
Hasan, R
Siddiqui, AA
AF Adler, Douglas G.
Taylor, Linda Jo
Hasan, Raza
Siddiqui, Ali A.
TI A retrospective study evaluating endoscopic ultrasound-guided drainage
of pancreatic fluid collections using a novel lumen-apposing metal stent
on an electrocautery enhanced delivery system
SO ENDOSCOPIC ULTRASOUND
LA English
DT Article
DE Endosonography; metal stents; pancreatic fluid collection
ID PSEUDOCYSTS; MANAGEMENT; NECROSIS
AB Objectives: To report the safety and clinical efficacy with the novel lumen-apposing metal stent (LAMS) with an electrocautery enhanced delivery system for the drainage of pancreatic fluid collections (PFCs). Methods: This was a retrospective analysis of all consecutive patients with PFCs who underwent endoscopic ultrasound (EUS)-guided drainage using the LAMS with an electrocautery enhanced delivery system in 2 US centers. Results: Thirteen patients with PFCs (69% with walled-off necrosis [WON]) underwent drainage using the study device. Successful stent placement was accomplished in all patients. Direct endoscopic necrosectomy was carried out in all nine patients with WON complete resolution of the PFC was obtained in all 13 cases, with no recurrence during follow-up. There was one procedure-related adverse event. In one patient, the LAMS was dislodged immediately after deployment, falling into the stomach where it was removed. A second electrocautery enhanced LAMS was placed in this patient immediately afterward. Conclusions: EUS-guided drainage using the LAMS with the electrocautery-enhanced delivery system is a safe, easily performed, and a highly effective for the drainage of PFCs.
C1 [Adler, Douglas G.; Taylor, Linda Jo] Univ Utah Hosp, Div Gastroenterol, Dept Internal Med, Salt Lake City, UT USA.
[Hasan, Raza; Siddiqui, Ali A.] Thomas Jefferson Univ Hosp, Div Gastroenterol & Hepatol, Philadelphia, PA 19107 USA.
C3 Utah System of Higher Education; University of Utah; University of Utah
Hospital; Thomas Jefferson University
RP Siddiqui, AA (corresponding author), Thomas Jefferson Univ Hosp, Dept Med, Philadelphia, PA 19107 USA.
EM Ali.Siddiqui@Jefferson.edu
CR Baillie J, 2004, GASTROINTEST ENDOSC, V59, P873, DOI 10.1016/S0016-5107(04)00354-2
Banks PA, 2013, GUT, V62, P102, DOI 10.1136/gutjnl-2012-302779
Baron TH, 2002, GASTROINTEST ENDOSC, V56, P7, DOI 10.1067/mge.2002.125106

Binmoeller KF, 2011, ENDOSCOPY, V43, P337, DOI 10.1055/s-0030-1256127
BINMOELLER KF, 1995, GASTROINTEST ENDOSC, V42, P219, DOI 10.1016/S0016-5107(95)70095-1
Brun A, 2011, J CLIN GASTROENTEROL, V45, P614, DOI 10.1097/MCG.0b013e318213ef3e
Itoi T, 2012, GASTROINTEST ENDOSC, V75, P870, DOI 10.1016/j.gie.2011.10.020
Jacobson BC, 2005, GASTROINTEST ENDOSC, V61, P363, DOI 10.1016/S0016-5107(04)02779-8
Nealon WH, 2002, ANN SURG, V235, P751, DOI 10.1097/00000658-200206000-00001
Shah RJ, 2015, CLIN GASTROENTEROL H, V13, P747, DOI 10.1016/j.cgh.2014.09.047
Siddiqui AA, 2016, GASTROINTEST ENDOSC, V83, P699, DOI 10.1016/j.gie.2015.10.020
Tsiotos G G, 1999, Curr Gastroenterol Rep, V1, P139
Varadarajulu Shyam, 2013, Gastrointest Endosc Clin N Am, V23, P863, DOI 10.1016/j.giec.2013.06.008
Varadarajulu S, 2011, J GASTROINTEST SURG, V15, P2080, DOI 10.1007/s11605-011-1621-8
VITAS GJ, 1992, SURGERY, V111, P123
YEO CJ, 1990, SURG GYNECOL OBSTET, V170, P411
NR 16
TC 17
Z9 20
U1 0
U2 0
PU MEDKNOW PUBLICATIONS & MEDIA PVT LTD
PI MUMBAI
PA B-9, KANARA BUSINESS CENTRE, OFF LINK RD, GHAKTOPAR-E, MUMBAI, 400075,
INDIA
SN 2303-9027
EI 2226-7190
J9 ENDOSC ULTRASOUND
JI Endosc. Ultrasound
PD NOV-DEC
PY 2017
VL 6
IS 6
BP 389
EP 393
DI 10.4103/eus.eus_4_17
PG 5
WC Gastroenterology & Hepatology
WE Science Citation Index Expanded (SCI-EXPANDED)
SC Gastroenterology & Hepatology
GA FQ0NV
UT WOS:000418054900006
PM 29251273
OA Green Submitted
DA 2026-05-09

ER

PT J

AU Prachayakul, V

Aswakul, P

AF Prachayakul, Varayu

Aswakul, Pitulak

TI Endoscopic ultrasound-guided biliary drainage: Bilateral systems

drainage via left duct approach

SO WORLD JOURNAL OF GASTROENTEROLOGY

LA English

DT Article

DE Biliary drainage; Endoscopic ultrasound-guided; Bilateral systems;

Transmural drainage

ID BILE-DUCT; PERCUTANEOUS DRAINAGE; METAL STENT; OBSTRUCTION; MULTICENTER;

ACCESS; ERCP

AB Endoscopic ultrasound (EUS)-guided biliary drainage is accepted as a less invasive, alternative treatment for patients in whom endoscopic retrograde cholangiopancreatography has failed. Most patients with malignant hilar obstruction undergo EUS-guided hepaticogastrostomy. The authors present the case of a 77-year-old man with advanced hilar cholangiocarcinoma who had undergone a rouxen- Y hepaticojejunostomy several months prior. He developed progressive jaundice and a low-grade fever that persisted for one week. The enteroscopic-assisted endoscopic retrograde cholangiopancreatography failed, thus the patient was scheduled for EUS-guided biliary drainage. In order to obtain adequate drainage, both intrahepatic systems were drained. This report describes the technique used for bilateral drainage via a transgastric approach. Currently, only a few different techniques for EUS-guided right system drainage have been reported in the literature. This case demonstrates that bilateral EUS-guided biliary drainage is feasible and effective in patients with hilar cholangiocarcinoma, and thus can be used as an alternative to percutaneous biliary drainage.

C1 [Prachayakul, Varayu] Mahidol Univ, Siriraj Hosp, Div Gastroenterol, Siriraj GI Endoscopy Ctr,Dept Internal Med, Bangkok 10700, Thailand.

[Aswakul, Pitulak] Samitivej Sukhumvit Hosp, Liver & Digest Inst, Bangkok 10120, Thailand.

C3 Mahidol University

RP Prachayakul, V (corresponding author), Mahidol Univ, Siriraj Hosp, Div Gastroenterol, Siriraj GI Endoscopy Ctr,Dept Internal Med, Bangkok 10700, Thailand.

EM kaiyjr@gmail.com

CR Artifon ELA, 2012, J CLIN GASTROENTEROL, V46, P768, DOI 10.1097/MCG.0b013e31825f264c

Artifon ELA, 2012, KOREAN J RADIOL, V13, pS74, DOI 10.3348/kjr.2012.13.S1.S74

Burmester E, 2003, GASTROINTEST ENDOSC, V57, P246, DOI 10.1067/mge.2003.85

Dhir V, 2014, DIGEST ENDOSC, V26, P430, DOI 10.1111/den.12153

Fabbri C, 2014, WORLD J GASTROENTERO, V20, P8424, DOI 10.3748/wjg.v20.i26.8424

Giovannini M, 2001, ENDOSCOPY, V33, P898, DOI 10.1055/s-2001-17324

Gupta K, 2014, J CLIN GASTROENTEROL, V48, P80, DOI 10.1097/MCG.0b013e31828c6822

Iqbal S, 2013, GASTROENT RES PRACT, V2013, DOI 10.1155/2013/869214

Khashab MA, 2015, DIGEST DIS SCI, V60, P557, DOI 10.1007/s10620-014-3300-6

Ogura T, 2015, ENDOSCOPY, V47, P72, DOI 10.1055/s-0034-1378111
Park SJ, 2013, GASTROINTEST ENDOSC, V78, P374, DOI 10.1016/j.gie.2013.04.183
Prachayakul V, 2015, WORLD J GASTRO ENDOS, V7, P37, DOI 10.4253/wjge.v7.i1.37
Song TJ, 2014, GASTROINTEST ENDOSC, V80, P707, DOI 10.1016/j.gie.2014.05.327
NR 13
TC 9
Z9 9
U1 0
U2 8
PU BAISHIDENG PUBLISHING GROUP INC
PI PLEASANTON
PA 8226 REGENCY DR, PLEASANTON, CA 94588 USA
SN 1007-9327
EI 2219-2840
J9 WORLD J GASTROENTERO
JI World J. Gastroenterol.
PD SEP 14
PY 2015
VL 21
IS 34
BP 10045
EP 10048
DI 10.3748/wjg.v21.i34.10045
PG 4
WC Gastroenterology & Hepatology
WE Science Citation Index Expanded (SCI-EXPANDED)
SC Gastroenterology & Hepatology
GA CS0QI
UT WOS:000361766800021
PM 26379410
OA hybrid
DA 2026-05-09
ER

PT J
AU Nakai, Y
Isayama, H
Matsubara, S
Koike, K
AF Nakai, Yousuke
Isayama, Hiroyuki
Matsubara, Saburo

Koike, Kazuhiko
TI Conversion of transpapillary drainage to endoscopic ultrasound-guided hepaticogastrostomy and gallbladder drainage in a case of malignant biliary obstruction with recurrent cholangitis and cholecystitis (with videos)
SO ENDOSCOPIC ULTRASOUND
LA English
DT Article
DE Biliary drainage; endoscopic ultrasound; malignant biliary obstruction
ID STENT
AB Endoscopic ultrasound-guided biliary drainage (EUS-BD) has been increasingly reported as an alternative to percutaneous transhepatic biliary drainage in failed endoscopic retrograde cholangiopancreatography. Moreover, conversion to EUS-BD can be a good alternative when transpapillary biliary drainage is technically possible but complicated by cholangitis because EUS-BD enables one-step internal drainage not traversing the tumor. Herein, we report a case of recurrent cholangitis due to hemobilia and cholecystitis due to tumor involvement to the cystic duct after transpapillary stent placement, which was successfully managed by conversion to EUS-BD and EUS-guided gallbladder drainage in one session.
C1 [Nakai, Yousuke; Isayama, Hiroyuki; Matsubara, Saburo; Koike, Kazuhiko] Univ Tokyo, Grad Sch Med, Dept Gastroenterol, Tokyo, Japan.
C3 University of Tokyo
RP Isayama, H (corresponding author), Univ Tokyo, Grad Sch Med, Dept Gastroenterol, Bunkyo Ku, 7-3-1 Hongo, Tokyo 1138655, Japan.
EM isayama-tky@umin.ac.jp
RI Isayama, Hiroyuki/AER-9916-2022; Nakai, Yousuke/H-2964-2019
CR Isayama H, 2006, CLIN GASTROENTEROL H, V4, P1148, DOI 10.1016/j.cgh.2006.06.004
Khashab MA, 2015, GASTROINTEST ENDOSC, V82, P993, DOI 10.1016/j.gie.2015.06.043
Nakai Y, ENDOSC ULTRASOUND, DOI [10.4103/2303-9027.199763, DOI 10.4103/2303-9027.199763]
Nakai Y, 2016, ENDOSCOPY, V48, P1125, DOI 10.1055/s-0042-116595
NR 4
TC 8
Z9 9
U1 0
U2 1
PU MEDKNOW PUBLICATIONS & MEDIA PVT LTD
PI MUMBAI
PA B-9, KANARA BUSINESS CENTRE, OFF LINK RD, GHAKTOPAR-E, MUMBAI, 400075, INDIA
SN 2303-9027
EI 2226-7190
J9 ENDOSC ULTRASOUND
JI Endosc. Ultrasound
PD MAY-JUN

PY 2017
VL 6
IS 3
BP 205
EP 207
DI 10.4103/2303-9027.208172
PG 3
WC Gastroenterology & Hepatology
WE Science Citation Index Expanded (SCI-EXPANDED)
SC Gastroenterology & Hepatology
GA EY9EK
UT WOS:000404301700011
PM 28621299
DA 2026-05-09
ER

PT J
AU Ueki, T
Maruo, T
Igarashi, Y
Yamamiya, A
Tominaga, K
Irisawa, A
Yoshida, H
Kamisawa, T
Takenaka, M
Isayama, H
AF Ueki, Toshiharu
Maruo, Toru
Igarashi, Yoshinori
Yamamiya, Akira
Tominaga, Keiichi
Irisawa, Atsushi
Yoshida, Hitoshi
Kamisawa, Terumi
Takenaka, Mamoru
Isayama, Hiroyuki
TI Role of advanced endoscopy in the management of inflammatory digestive
diseases (pancreas and biliary tract)
SO DIGESTIVE ENDOSCOPY
LA English
DT Review

DE biliary tract; endoscopy; inflammation; pancreas
ID WALLED-OFF NECROSIS; TERM-FOLLOW-UP; TRANSMURAL DRAINAGE; GALLBLADDER
DRAINAGE; ACUTE CHOLANGITIS; PERCUTANEOUS DRAINAGE; CLINICAL-OUTCOMES;
DUCT DRAINAGE; METAL STENTS; ERCP FAILS
AB The progress of endoscopic diagnosis and treatment for inflammatory diseases of the biliary tract and pancreas have been remarkable. Endoscopic ultrasonography (EUS) and EUS-elastography are used for the diagnosis of early chronic pancreatitis and evaluation of endocrine and exocrine function in chronic pancreatitis. Notably, extracorporeal shock wave lithotripsy and electrohydraulic shock wave lithotripsy have improved the endoscopic stone removal rate in patients for whom pancreatic stone removal is difficult. Studies have reported the use of self-expanding metal stents for stent placement for pancreatic duct stenosis and EUS-guided pancreatic drainage for refractory pancreatic duct strictures. Furthermore, EUS-guided drainage using a double-pigtailed plastic stent has been performed for the management of symptomatic pancreatic fluid collection after acute pancreatitis. Recently, lumen-apposing metal stents have led to advances in the treatment of walled-off necrosis after acute pancreatitis. EUS-guided biliary drainage is an alternative to refractory endoscopic biliary drainage and percutaneous transhepatic biliary drainage for the treatment of acute cholangitis. The placement of an inside stent followed by switching to uncovered self-expanding metal stents in difficult-to-treat cases has been proposed for acute cholangitis by malignant biliary obstruction. Endoscopic transpapillary gallbladder drainage is an alternative to percutaneous transhepatic gallbladder drainage for severe and some cases of moderate acute cholecystitis. EUS-guided gallbladder drainage has been reported as an alternative to percutaneous transhepatic gallbladder drainage and endoscopic transpapillary gallbladder drainage. However, it is important to understand the advantages and disadvantages of each drainage method and select the optimal drainage method for each case.
C1 [Ueki, Toshiharu; Maruo, Toru] Fukuoka Univ, Chikushi Hosp, Dept Gastroenterol, 1-1-1 Zokumyouin, Chikushino, Fukuoka 8188502, Japan.
[Igarashi, Yoshinori] Toho Univ, Omori Med Ctr, Div Gastroenterol & Hepatol, Tokyo, Japan.
[Yoshida, Hitoshi] Showa Univ, Sch Med, Dept Gastroenterol, Tokyo, Japan.
[Kamisawa, Terumi] Komagome Hosp, Tokyo Metropolitan Canc & Infect Dis Ctr, Dept Gastroenterol, Tokyo, Japan.
[Isayama, Hiroyuki] Juntendo Univ, Grad Sch Med, Dept Gastroenterol, Tokyo, Japan.
[Yamamiya, Akira; Tominaga, Keiichi; Irisawa, Atsushi] Dokkyo Med Univ, Dept Gastroenterol, Sch Med, Tochigi, Japan.
[Takenaka, Mamoru] Kindai Univ Hosp, Fac Med, Dept Gastroenterol & Hepatol, Osaka, Japan.
C3 Fukuoka University; Toho University; Showa Medical University; Tokyo
Metropolitan Cancer & Infectious Diseases Center Komagome Hospital;
Juntendo University; Dokkyo Medical University
RP Ueki, T (corresponding author), Fukuoka Univ, Chikushi Hosp, Dept Gastroenterol, 1-1-1 Zokumyouin, Chikushino, Fukuoka 8188502, Japan.
EM tosiueki@fukuoka-u.ac.jp
RI ; Tominaga, Keiichi/AAN-2986-2020; Isayama, Hiroyuki/AER-9916-2022
OI Irisawa, Atsushi/0000-0002-2271-2717; Tominaga,
Keiichi/0000-0002-3716-3386; Takenaka, Mamoru/0000-0001-7308-4311; Ueki,
Toshiharu/0000-0002-4142-5895;
FU Grants-in-Aid for Scientific Research [20K11609] Funding Source: KAKEN
CR Abu Dayyeh BK, 2023, ANN SURG, V277, pE1072, DOI 10.1097/SLA.0000000000005274
Angadi S, 2021, PANCREATOLOGY, V21, P1291, DOI 10.1016/j.pan.2021.06.006

Artifon ELA, 2012, J CLIN GASTROENTEROL, V46, P768, DOI 10.1097/MCG.0b013e31825f264c
Bang JY, 2020, DIGEST ENDOSC, V32, P298, DOI 10.1111/den.13470
Bang JY, 2019, GUT, V68, P1200, DOI 10.1136/gutjnl-2017-315335
Banks PA, 2013, GUT, V62, P102, DOI 10.1136/gutjnl-2012-302779
Bapaye A, 2017, DIGEST ENDOSC, V29, P104, DOI 10.1111/den.12704
Bapaye A, 2013, UNITED EUR GASTROENT, V1, P285, DOI 10.1177/2050640613490928
Baron TH, 2015, NEW ENGL J MED, V373, P357, DOI 10.1056/NEJMra1411372
Boxhoorn L, 2021, NEW ENGL J MED, V385, P1372, DOI 10.1056/NEJMoa2100826
Chantarojanasiri Tanyaporn, 2017, Saudi J Gastroenterol, V23, P318, DOI 10.4103/sjg.SJG_115_17
Dumonceau JM, 2019, ENDOSCOPY, V51, P179, DOI 10.1055/a-0822-0832
Dumonceau JM, 2018, ENDOSCOPY, V50, P910, DOI 10.1055/a-0659-9864
Eto K, 2015, J HEPATO-BIL-PAN SCI, V22, P825, DOI 10.1002/jhbp.296
Gardner TB, 2011, GASTROINTEST ENDOSC, V73, P718, DOI 10.1016/j.gie.2010.10.053
Garg PK, 2019, GASTROENTEROLOGY, V156, P2008, DOI 10.1053/j.gastro.2018.12.041
Gravito-Soares E, 2018, SCAND J GASTROENTERO, V53, P329, DOI 10.1080/00365521.2018.1430255
Iino C, 2018, ENDOSC INT OPEN, V6, pE594, DOI 10.1055/s-0044-102091
Irisawa A, 2022, DIGEST ENDOSC, V34, P124, DOI 10.1111/den.14173
Irisawa A, 2009, J HEPATO-BILIARY-PAN, V16, P598, DOI 10.1007/s00534-009-0131-5
Isayama H, 2019, J HEPATO-BIL-PAN SCI, V26, P249, DOI 10.1002/jhbp.631
Isayama H, 2015, DIGEST ENDOSC, V27, P259, DOI 10.1111/den.12379
Itoi T, 2018, ENDOSC ULTRASOUND, V7, P76, DOI 10.4103/eus.eus_4_18
Jain D, 2018, CLIN ENDOSC, V51, P450, DOI 10.5946/ce.2018.024
Karstensen JG, 2023, GUT, V72, P1167, DOI 10.1136/gutjnl-2022-328225
Khan MA, 2019, J CLIN GASTROENTEROL, V53, P81, DOI 10.1097/MCG.0000000000001141
Khashab MA, 2015, DIGEST DIS SCI, V60, P557, DOI 10.1007/s10620-014-3300-6
Kogure H, 2021, J CLIN MED, V10, DOI 10.3390/jcm10132936
Lee TH, 2016, CLIN GASTROENTEROL H, V14, P1011, DOI 10.1016/j.cgh.2015.12.032
Li TT, 2020, J GASTROEN HEPATOL, V35, P1099, DOI 10.1111/jgh.14972
Manta R, 2018, EUR J GASTROEN HEPAT, V30, P695, DOI 10.1097/MEG.0000000000001112
Maruta A, 2020, J HEPATO-BIL-PAN SCI, V27, P429, DOI 10.1002/jhbp.744
Masamune A, 2020, J GASTROENTEROL, V55, P1062, DOI 10.1007/s00535-020-01704-9
Matsunami Y, 2018, ENDOSC INT OPEN, V6, pE505, DOI 10.1055/s-0044-101753
Mayumi T, 2018, J HEPATO-BIL-PAN SCI, V25, P96, DOI 10.1002/jhbp.519
McKay A, 2012, SURG ENDOSC, V26, P1343, DOI 10.1007/s00464-011-2035-0
Minaguchi T, 2022, DIGEST ENDOSC, V34, P1214, DOI 10.1111/den.14261
Miura F, 2018, J HEPATO-BIL-PAN SCI, V25, P31, DOI 10.1002/jhbp.509
Mori Y, 2018, J HEPATO-BIL-PAN SCI, V25, P87, DOI 10.1002/jhbp.504
Mukai T, 2013, J HEPATO-BIL-PAN SCI, V20, P214, DOI 10.1007/s00534-012-0508-8
Nagino M, 2021, J HEPATO-BIL-PAN SCI, V28, P26, DOI 10.1002/jhbp.870
Nakai Y, 2019, SAUDI J GASTROENTERO, V25, P210, DOI 10.4103/sjg.SJG_474_18
Park DH, 2009, ENDOSCOPY, V41, P842, DOI 10.1055/s-0029-1215133
Podboy A, 2021, GASTROINTEST ENDOSC, V93, P797, DOI 10.1016/j.gie.2020.09.040

Sagami R, 2020, DIGEST ENDOSC, V32, P1092, DOI 10.1111/den.13650
Saghir SM, 2020, WORLD J GASTROENTERO, V26, P5207, DOI 10.3748/wjg.v26.i34.5207
Sakai T, 2022, PANCREATOLOGY, V22, P58, DOI 10.1016/j.pan.2021.10.006
Sato T, 2020, ENDOSCOPY, V52, P368, DOI 10.1055/a-1111-8666
Saumoy M, 2021, GASTROINTEST ENDOSC, V94, P671, DOI 10.1016/j.gie.2021.05.031
Seifert H, 2009, GUT, V58, P1260, DOI 10.1136/gut.2008.163733
Sekine M, 2021, PANCREAS, V50, P1173, DOI 10.1097/MPA.0000000000001893
Sharaiha RZ, 2017, GASTROINTEST ENDOSC, V85, P904, DOI 10.1016/j.gie.2016.12.023
Sharaiha RZ, 2016, SURG ENDOSC, V30, P5500, DOI 10.1007/s00464-016-4913-y
Sharma BC, 2005, ENDOSCOPY, V37, P439, DOI 10.1055/s-2005-861054
Sherman S, 2023, GASTROINTEST ENDOSC, V97, DOI 10.1016/j.gie.2022.09.021
Siddiqui AA, 2017, GASTROINTEST ENDOSC, V85, P758, DOI 10.1016/j.gie.2016.08.014
Takahashi S, 2023, J HEPATO-BIL-PAN SCI, V30, P408, DOI 10.1002/jhbp.1221
Takenaka M, 2022, ENDOSCOPY, V54, pE102, DOI 10.1055/a-1396-3606
Yuste RT, 2020, DIGEST ENDOSC, V32, P608, DOI 10.1111/den.13562
Tsujino T, 2007, EUR J GASTROEN HEPAT, V19, P585, DOI 10.1097/MEG.0b013e3281532b78
van Brunschot S, 2018, LANCET, V391, P51, DOI 10.1016/S0140-6736(17)32404-2
van der Wiel SE, 2022, GASTROINTEST ENDOSC, V95, P905, DOI 10.1016/j.gie.2021.11.047
van Huijgevoort NCM, 2020, ENDOSC INT OPEN, V08, pE1070, DOI 10.1055/a-1171-1322
Varadarajulu S, 2008, GASTROINTEST ENDOSC, V68, P1102, DOI 10.1016/j.gie.2008.04.028
Vila JJ, 2012, GASTROINTEST ENDOSC, V76, P1133, DOI 10.1016/j.gie.2012.08.001
Whitcomb DC, 2016, PANCREATOLOGY, V16, P218, DOI 10.1016/j.pan.2016.02.001
Yamabe A, 2021, DIGESTION, V102, P289, DOI 10.1159/000504780
Yamamiya A, 2021, J CLIN MED, V10, DOI 10.3390/jcm10163739
Yamamiya A, 2021, DIAGNOSTICS, V11, DOI 10.3390/diagnostics11030431
Yamamiya A, 2020, DIAGNOSTICS, V10, DOI 10.3390/diagnostics10110953
Yamashita Y, 2021, J GASTROEN HEPATOL, V36, P391, DOI 10.1111/jgh.15137
Yasuda I, 2013, ENDOSCOPY, V45, P627, DOI 10.1055/s-0033-1344027
NR 72
TC 4
Z9 4
U1 0
U2 6
PU WILEY
PI HOBOKEN
PA 111 RIVER ST, HOBOKEN 07030-5774, NJ USA
SN 0915-5635
EI 1443-1661
J9 DIGEST ENDOSC
JI Dig. Endosc.
PD MAY
PY 2024

VL 36
IS 5
BP 546
EP 553
DI 10.1111/den.14756
EA MAR 2024
PG 8
WC Gastroenterology & Hepatology; Surgery
WE Science Citation Index Expanded (SCI-EXPANDED)
SC Gastroenterology & Hepatology; Surgery
GA PT4F3
UT WOS:001183514700001
PM 38475671
OA Bronze
DA 2026-05-09
ER

PT J
AU Ogura, T
Itoi, T
AF Ogura, Takeshi
Itoi, Takao
TI Technical tips and recent development of endoscopic ultrasound-guided choledochoduodenostomy
SO DEN OPEN
LA English
DT Review
DE adverse events; EUS; EUS-CDS; EUS-guided biliary drainage; lumen-apposing metal stent
AB Various efforts to improve technical success rates and decrease adverse event rates have also been described in endoscopic ultrasound (EUS)-guided choledochoduodenostomy (CDS). In particular, lumen-apposing metal stents (LAMS) may open novel opportunities in EUS-biliary drainage (BD). To date, various studies have been reported with EUS-CDS using LAMS, so we should clarify the benefits and limitations of recent EUS-CDS based on developments in both techniques and devices. In this review, we provide technical tips and describe recent developments in EUS-CDS, along with a review of the recent literature (between 2015 and 2020). The overall technical success rate is 95.0% (939/988), and the overall clinical success rate is 97.0% (820/845). The most frequent adverse event is cholangitis or cholecystitis (24.5%, 27/110). According to previous review, pneumoperitoneum (28%, 9/34) or peritonitis associated with bile leak (23.5%, 8/34) was most commonly observed. This difference might be based on improvements in dilation devices or the use of covered metal stents. Several randomized controlled trials comparing EUS-CDS and endoscopic retrograde cholangiopancreatography (ERCP) for malignant biliary obstruction have recently been reported. To summarize, overall technical success rates for ERCP and EUS-CDS were 92.7% (101/109) and 91.1% (72/79), respectively ($p = 0.788$). Overall clinical success rates for ERCP and EUS-CDS were 94.1% (96/102) and 93.6% (72/78), respectively ($p = 0.765$). Further

high-quality evidence is needed to establish EUS-CDS as a primary drainage technique.

C1 [Ogura, Takeshi] Osaka Med Coll, Dept Internal Med 2, Osaka, Japan.
[Itoi, Takao] Tokyo Med Univ, Dept Gastroenterol & Hepatol, Tokyo, Japan.
C3 Osaka Medical & Pharmaceutical University; Tokyo Medical University
RP Ogura, T (corresponding author), Osaka Med Coll, Dept Internal Med, 2-7 Daigakuchou, Takatsuki, Osaka 5698686, Japan.
EM oguratakeshi0411@yahoo.co.jp
RI Ogura, Takeshi/AAU-5643-2021

CR Amano M, 2017, J GASTROEN HEPATOL, V32, P716, DOI 10.1111/jgh.13489
Anderloni A, 2019, GASTROINTEST ENDOSC, V89, P69, DOI [10.1016/j.gie.2018.04.1746, 10.1016/j.gie.2018.08.047]
Anderloni A, 2018, AM J GASTROENTEROL, V113, P644, DOI 10.1038/s41395-018-0040-9
Anderloni A, 2017, ENDOSC INT OPEN, V5, pE25, DOI 10.1055/s-0042-122009
Bang JY, 2018, GASTROINTEST ENDOSC, V88, P9, DOI 10.1016/j.gie.2018.03.012
Binmoeller KF, 2011, ENDOSCOPY, V43, P337, DOI 10.1055/s-0030-1256127
Chin JYL, 2020, ENDOSC INT OPEN, V08, pE1633, DOI 10.1055/a-1236-3217
Cho DH, 2017, GASTROINTEST ENDOSC, V85, P1067, DOI 10.1016/j.gie.2016.09.010
Sanz MD, 2021, SURG ENDOSC, V35, P6754, DOI 10.1007/s00464-020-08179-y
El Chafic AH, 2019, DIGEST DIS SCI, V64, P3321, DOI 10.1007/s10620-019-05688-2
Hara K, 2011, AM J GASTROENTEROL, V106, P1239, DOI 10.1038/ajg.2011.84
Honjo M, 2018, ENDOSC ULTRASOUND, V7, P376, DOI 10.4103/eus.eus_2_18
Itonaga M, 2019, DIGEST ENDOSC, V31, P291, DOI 10.1111/den.13300
Jacques J, 2020, GASTROINTEST ENDOSC, V92, P134, DOI 10.1016/j.gie.2020.01.055
Jacques J, 2019, ENDOSCOPY, V51, P540, DOI 10.1055/a-0735-9137
Kakked G, 2020, ENDOSC ULTRASOUND, V9, P298, DOI 10.4103/eus.eus_10_20
Kawakubo K, 2016, ENDOSCOPY, V48, P164, DOI 10.1055/s-0034-1393179
Khashab MA, 2016, ENDOSC INT OPEN, V4, pE175, DOI 10.1055/s-0041-109083
Kunda R, 2016, SURG ENDOSC, V30, P5002, DOI 10.1007/s00464-016-4845-6
Kuraoka Naosuke, 2020, Endosc Int Open, V8, pE861, DOI [10.1055/a-1161-8488, 10.1055/a-1161-8488]
Lu L, 2017, GASTROENT RES PRACT, V2017, DOI 10.1155/2017/6284094
Matsumoto S, 2020, DIGEST ENDOSC, V32, P957, DOI 10.1111/den.13620
Minaga K, 2019, DIGEST ENDOSC, V31, P575, DOI 10.1111/den.13406
Miyano A, 2018, J GASTROINTEST SURG, V22, P1312, DOI 10.1007/s11605-018-3758-1
Nakai Y, 2019, ENDOSC ULTRASOUND, V8, P111, DOI 10.4103/eus.eus_17_18
Nennstiel S, 2015, J CLIN GASTROENTEROL, V49, P764, DOI 10.1097/MCG.0000000000000275
Ogura T, 2021, DIGEST DIS, V39, P165, DOI 10.1159/000510560
Ogura T, 2019, ENDOSC INT OPEN, V7, pE1652, DOI 10.1055/a-0961-7890
Ogura T, 2016, GASTROINTEST ENDOSC, V83, P834, DOI 10.1016/j.gie.2015.10.009
Ogura T, 2015, WORLD J GASTROENTERO, V21, P820, DOI 10.3748/wjg.v21.i3.820
Paik WH, 2018, AM J GASTROENTEROL, V113, P987, DOI 10.1038/s41395-018-0122-8
Park JK, 2018, GASTROINTEST ENDOSC, V88, P277, DOI 10.1016/j.gie.2018.03.015
Rai P, 2018, ENDOSC INT OPEN, V6, pE67, DOI 10.1055/s-0043-120664

Rodrigues-Pinto E, 2016, EXPERT REV MED DEVIC, V13, P793, DOI 10.1080/17434440.2016.1222898
Ryou M, 2020, GASTROINTEST ENDOSC, V91, P178, DOI 10.1016/j.gie.2019.07.035
Sassatelli R, 2020, DIGEST LIVER DIS, V52, P57, DOI 10.1016/j.dld.2019.07.009
Shiomi H, 2019, DIGEST ENDOSC, V31, pe50, DOI 10.1111/den.13328
Song TJ, 2019, GASTROINTEST ENDOSC, V90, P507, DOI 10.1016/j.gie.2019.05.033
Teoh AYB, 2021, DIGEST ENDOSC, V33, P1139, DOI 10.1111/den.13911
Tsuchiya T, 2018, GASTROINTEST ENDOSC, V87, P1138, DOI 10.1016/j.gie.2017.08.017
Yamamoto Y, 2020, ENDOSC ULTRASOUND, V9, P110, DOI 10.4103/eus.eus_68_19
Zhao XQ, 2015, DIGEST ENDOSC, V27, P137, DOI 10.1111/den.12320
NR 42
TC 41
Z9 43
U1 0
U2 1
PU WILEY
PI HOBOKEN
PA 111 RIVER ST, HOBOKEN 07030-5774, NJ USA
SN 2692-4609
J9 DEN OPEN
JI DEN Open
PD APR
PY 2021
VL 1
IS 1
AR e8
DI 10.1002/deo2.8
PG 10
WC Gastroenterology & Hepatology
WE Emerging Sources Citation Index (ESCI)
SC Gastroenterology & Hepatology
GA VM8AO
UT WOS:001047018700007
PM 35310149
OA Green Submitted, gold
DA 2026-05-09
ER

PT J
AU Hemerly, MC
de Moura, DTH
do Monte, ES Jr
Proença, IM

Ribeiro, IB
Yvamoto, EY
Ribas, PHBV
Sánchez-Luna, SA
Bernardo, WM
de Moura, EGH
AF Hemerly, Matheus Candido
Hourneaux de Moura, Diogo Turiani
do Monte Junior, Epifanio Silvino
Proenca, Igor Mendonca
Ribeiro, Igor Braga
Yvamoto, Erika Yuki
Boraschi Vieira Ribas, Pedro Henrique
Sanchez-Luna, Sergio A.
Bernardo, Wanderley Marques
Hourneaux de Moura, Eduardo Guimaraes
TI Endoscopic ultrasound (EUS)-guided cholecystostomy versus percutaneous
cholecystostomy (PTC) in the management of acute cholecystitis in
patients unfit for surgery: a systematic review and meta-analysis
SO SURGICAL ENDOSCOPY AND OTHER INTERVENTIONAL TECHNIQUES
LA English
DT Review
DE Endoscopic ultrasound; Cholecystitis; Cholecystectomy; Endoscopy;
Interventional radiology; Surgery; Biliary; Gallbladder; Lumen-apposing
metal stents (LAMS); Fully covered self-expanding metal stents (FCSEMS)
ID GUIDED GALLBLADDER DRAINAGE; LONG-TERM OUTCOMES
AB Background and aim Surgical cholecystectomy is the gold standard strategy for the management of acute cholecystitis (AC). However, some patients are considered unfit for surgery due to certain comorbid conditions. As such, we aimed to compare less invasive treatment strategies such as endoscopic ultrasound-guided gallbladder drainage (EUS-GBD) and percutaneous gallbladder drainage (PT-GBD) for the management of patients with AC who are suboptimal candidates for surgical cholecystectomy. Methods A comprehensive search of multiple electronic databases was performed to identify all the studies comparing EUS-GBD versus PT-GBD for patients with AC who were unfit for surgery. A subgroup analysis was also performed for comparison of the group undergoing drainage via cautery-enhanced lumen-apposing metal stents (LAMS) versus PT-GBD. The outcomes included technical and clinical success, adverse events (AEs), recurrent cholecystitis, reintervention, and hospital readmission. Results Eleven studies including 1155 patients were included in the statistical analysis. There was no difference between PT-GBD and EUS-GBD in all the evaluated outcomes. On the subgroup analysis, the endoscopic approach with cautery-enhanced LAMS was associated with lower rates of adverse events (RD = - 0.33 (95% CI - 0.52 to - 0.14; p = 0.0006), recurrent cholecystitis (- 0.05 RD (95% CI - 0.09 to - 0.02; p = 0.02), and hospital readmission (- 0.36 RD (95% CI-0.70 to - 0.03; p = 0.03) when compared to PT-GBD. All other outcomes were similar in the subgroup analyses. Conclusions EUS-GBD using cautery-enhanced LAMS is superior to PT-GBD in terms of safety profile, recurrent cholecystitis, and hospital readmission rates in the management of patients with acute cholecystitis who are suboptimal candidates for cholecystectomy. However, when cautery-enhanced LAMS are not

used, the outcomes of EUS-GBD and PT-GBD are similar. Thus, EUS-GBD with cautery-enhanced LAMS should be considered the preferable approach for gallbladder drainage for this challenging population.

C1 [Hemerly, Matheus Candido; Hourneaux de Moura, Diogo Turiani; do Monte Junior, Epifanio Silvino; Proenca, Igor Mendonca; Ribeiro, Igor Braga; Yvamoto, Erika Yuki; Boraschi Vieira Ribas, Pedro Henrique; Bernardo, Wanderley Marques; Hourneaux de Moura, Eduardo Guimaraes] Univ Sao Paulo, Serv Endoscopia Gastrointestinal, Fac Med, Hosp Clin HCFMUSP,Dept Gastroenterol, Av Dr Eneas de Carvalho Aguiar 255, BR-05403000 Sao Paulo, SP, Brazil.

[Sanchez-Luna, Sergio A.] Univ Alabama Birmingham, Heersink Sch Med, Basil I Hirschowitz Endoscop Ctr Excellence, Div Gastroenterol & Hepatol,Dept Internal Med, Birmingham, AL USA.

C3 Universidade de Sao Paulo; University of Alabama System; University of Alabama Birmingham

RP Ribeiro, IB (corresponding author), Univ Sao Paulo, Serv Endoscopia Gastrointestinal, Fac Med, Hosp Clin HCFMUSP,Dept Gastroenterol, Av Dr Eneas de Carvalho Aguiar 255, BR-05403000 Sao Paulo, SP, Brazil.

EM hemerly1@hotmail.com; dthmoura@hotmail.com; epifaniosmjr@gmail.com; igor.epm76@gmail.com; igorbraga1@gmail.com; erika.yvamoto@gmail.com; phbvr93@hotmail.com; ssanchezluna@gmail.com; wmbernardo@usp.br; eduardoghdemoura@gmail.com

RI Junior, Epifanio/AAX-1834-2021; de Moura, Eduardo/D-3885-2018; Sánchez-Luna, Sergio/HKO-9537-2023; Moura, Diogo/J-6190-2019; de Moura, Eduardo/J-6190-2019; Ribeiro, Igor/U-2003-2019

OI de Moura, Eduardo/0000-0002-8023-3722; Yvamoto, Erika Yuki/0000-0002-7483-8047; Moura, Diogo/0000-0002-7446-0355; de Moura, Eduardo/0000-0002-8023-3722;

CR Baron TH, 2007, GASTROINTEST ENDOSC, V65, P735, DOI 10.1016/j.gie.2006.07.041

Boregowda Umesha, 2018, World J Gastrointest Pharmacol Ther, V9, P47, DOI [10.4292/wjgpt.v9.i6.47, 10.4292/wjgpt.v9.i6.47]

Choi JH, 2014, ENDOSCOPY, V46, P656, DOI 10.1055/s-0034-1365720

DERSIMONIAN R, 1986, CONTROL CLIN TRIALS, V7, P177, DOI 10.1016/0197-2456(86)90046-2

Fugazza A, 2020, CLIN EXP GASTROENTER, V13, P193, DOI 10.2147/CEG.S203626

Gomes CA, 2017, WORLD J GASTRO SURG, V9, P118, DOI 10.4240/wjgs.v9.i5.118

Guyatt GH, 2008, BRIT MED J, V336, P924, DOI 10.1136/bmj.39489.470347.AD

Irani S, 2017, CLIN GASTROENTEROL H, V15, P738, DOI 10.1016/j.cgh.2016.12.021

James TW, 2019, ENDOSC ULTRASOUND, V8, pS28, DOI 10.4103/eus.eus_41_19

Jang JW, 2012, GASTROENTEROLOGY, V142, P805, DOI 10.1053/j.gastro.2011.12.051

Jo S, 2018, GASTROINTEST ENDOSC, V87, pAB319, DOI [10.1016/j.gie.2018.04.1690, 10.1016/j.gie.2018.04.1690, DOI 10.1016/J.GIE.2018.04.1690]

Kamata K, 2017, WORLD J GASTROENTERO, V23, P661, DOI 10.3748/wjg.v23.i4.661

Kozakai F, 2019, CLIN ENDOSC, V52, P262, DOI 10.5946/ce.2018.183

Law Ryan, 2018, Gastrointest Endosc Clin N Am, V28, P187, DOI [10.1016/j.giec.2017.11.006, 10.1016/j.giec.2017.11.006]

dos Santos MEL, 2022, ENDOSCOPY, V54, pE79, DOI 10.1055/a-1388-6254

Lyu YX, 2021, J LAPAROENDOSC ADV S, V31, P1232, DOI 10.1089/lap.2020.0786

McCarty TR, 2021, SURG ENDOSC, V35, P4964, DOI 10.1007/s00464-020-07758-3

Moher D, 2015, SYST REV-LONDON, V4, DOI 10.1186/2046-4053-4-1
Mori Y, 2018, J HEPATO-BIL-PAN SCI, V25, P87, DOI 10.1002/jhbp.504
Nam K, 2018, ENDOSC ULTRASOUND, V7, P48, DOI 10.4103/eus.eus_100_17
Park SW, 2022, DIGEST ENDOSC, V34, P439, DOI 10.1111/den.14083
Piyachaturawat P., 2020, GASTROINTEST ENDOSC, DOI [10.1016/j.gie.2020.03.2102, DOI 10.1016/J.GIE.2020.03.2102]
Podboy A, 2021, GASTROINTEST ENDOSC, V93, P797, DOI 10.1016/j.gie.2020.09.040
Rana SS, 2021, ANN GASTROENTEROL, V34, P142, DOI 10.20524/aog.2020.0568
Saumoy M, 2019, J CLIN GASTROENTEROL, V53, P231, DOI 10.1097/MCG.0000000000001036
Shah RN, 2020, GASTROINTEST ENDOSC, V91, pAB170, DOI [10.1016/j.gie.2020.03.1128, 10.1016/j.gie.2020.03.1128, DOI 10.1016/J.GIE.2020.03.1128]
Siddiqui A, 2019, SURG ENDOSC, V33, P1260, DOI 10.1007/s00464-018-6406-7
Teoh AYB, 2020, GUT, V69, P1085, DOI 10.1136/gutjnl-2019-319996
Teoh AYB, 2019, ENDOSC ULTRASOUND, V8, pS40, DOI 10.4103/eus.eus_49_19
Teoh AYB, 2017, ENDOSCOPY, V49, P130, DOI 10.1055/s-0042-119036
Tyberg A, 2018, J CLIN GASTROENTEROL, V52, P79, DOI 10.1097/MCG.0000000000000786
NR 31
TC 39
Z9 42
U1 0
U2 2
PU SPRINGER
PI NEW YORK
PA ONE NEW YORK PLAZA, SUITE 4600, NEW YORK, NY, UNITED STATES
SN 0930-2794
EI 1432-2218
J9 SURG ENDOSC
JI Surg. Endosc.
PD APR
PY 2023
VL 37
IS 4
BP 2421
EP 2438
DI 10.1007/s00464-022-09712-x
EA OCT 2022
PG 18
WC Surgery
WE Science Citation Index Expanded (SCI-EXPANDED)
SC Surgery
GA D2UK8
UT WOS:000875263700001

PM 36289089
DA 2026-05-09
ER

PT J
AU Siddiqui, UD
Levy, MJ
AF Siddiqui, Uzma D.
Levy, Michael J.
TI EUS-Guided Transluminal Interventions
SO GASTROENTEROLOGY
LA English
DT Article
DE EUS-Guided Gallbladder Drainage (EUS-GBD); EUS- Guided Gastroenterostomy (EUS-GE); EUS-Guided Pancreatic Fluid Collection Drainage (EUS-PFC Drainage)
ID APPOSING METAL STENT; MALIGNANT BILIARY OBSTRUCTION; PANCREATIC FLUID COLLECTIONS; OCCLUDED GASTROJEJUNOSTOMY BYPASS; ESOPHAGEAL COLLATERAL VEINS; GASTRIC OUTLET OBSTRUCTION; WALLED-OFF NECROSIS; TERM-FOLLOW-UP; ENDOSCOPIC ULTRASOUND; FAILED ERCP
AB The role of endoscopic ultrasound (EUS) has transitioned from a diagnostic to a therapeutic one over the past 40 years. With the advent of curvilinear array echoendo-scopes in the 1990s with an accessory channel, multiple tools and devices have been developed and used for a variety of transluminal interventions. EUS provides a viable option and is becoming the procedure of choice for many interventions, including bile and pancreatic duct drainage, guiding angiotherapy, pancreatic fluid collection management, gallbladder drainage, and creating a gastro-jejunostomy. Although reports demonstrate the technical success of these interventions, there is tremendous study heterogeneity and a relative lack of controlled randomized trials, which may limit our understanding of their role and utility. Furthermore, adverse events are relatively common and occasionally severe. Despite the limitations, available data strongly indicate the efficacy of EUS interventions when performed by well-trained endosonographers in carefully selected patients and managed in a multidisciplinary setting.
C1 [Siddiqui, Uzma D.] Univ Chicago Med, CERT, Chicago, IL USA.
[Levy, Michael J.] Mayo Clin, Div Gastroenterol & Hepatol, Rochester, MN USA.
C3 University of Chicago; Mayo Clinic
RP Siddiqui, UD (corresponding author), Univ Chicago Med, CERT, Med, 5700 S Maryland Ave, Chicago, IL 60637 USA.
EM usiddiqui@bsd.uchicago.edu
CR Anderloni A, 2016, SURG ENDOSC, V30, P5200, DOI 10.1007/s00464-016-4894-x
Artifon ELA, 2015, GASTROINTEST ENDOSC, V81, P950, DOI 10.1016/j.gie.2014.09.047
Artifon ELA, 2012, J CLIN GASTROENTEROL, V46, P768, DOI 10.1097/MCG.0b013e31825f264c
Bakker OJ, 2012, JAMA-J AM MED ASSOC, V307, P1053, DOI 10.1001/jama.2012.276
Banks PA, 2013, GUT, V62, P102, DOI 10.1136/gutjnl-2012-302779
Bapaye A, 2017, DIGEST ENDOSC, V29, P104, DOI 10.1111/den.12704
Barkay O, 2010, GASTROINTEST ENDOSC, V71, P1166, DOI 10.1016/j.gie.2009.10.048
Bazerbachi F, 2018, GASTROINTEST ENDOSC, V87, P30, DOI 10.1016/j.gie.2017.08.025

Binmoeller KF, 2012, ENDOSCOPY, V44, P499, DOI 10.1055/s-0032-1309382
Binmoeller KF, 2011, GASTROINTEST ENDOSC, V74, P1019, DOI 10.1016/j.gie.2011.06.030
Bollen TL, 2012, AM J GASTROENTEROL, V107, P612, DOI 10.1038/ajg.2011.438
Brauer BC, 2009, GASTROINTEST ENDOSC, V70, P471, DOI 10.1016/j.gie.2008.12.233
Brückner S, 2015, ENDOSCOPY, V47, P858, DOI 10.1055/s-0034-1392104
Chang KJ, 1996, GASTROINTEST ENDOSC, V43, pA417
Chaves DM, 2012, ENDOSCOPY, V44, pE99, DOI 10.1055/s-0030-1256740
Chen YI, 2017, GASTROINTEST ENDOSC, V85, P170, DOI 10.1016/j.gie.2016.07.031
Conrad C, 2012, CANCER J, V18, P577, DOI 10.1097/PPO.0b013e3182797dfe
de Paulo GA, 2006, GASTROINTEST ENDOSC, V63, P396, DOI 10.1016/j.gie.2005.10.039
Dhir V, 2015, GASTROINTEST ENDOSC, V81, P913, DOI 10.1016/j.gie.2014.09.054
Dhir V, 2014, DIGEST ENDOSC, V26, P430, DOI 10.1111/den.12153
Dhir V, 2012, GASTROINTEST ENDOSC, V75, P354, DOI 10.1016/j.gie.2011.07.075
DIMAGNO EP, 1980, LANCET, V1, P629
Dollhopf M, 2017, GASTROINTEST ENDOSC, V86, P636, DOI 10.1016/j.gie.2017.02.027
Folvik G, 2001, ENDOSCOPY, V33, P636, DOI 10.1055/s-2001-15322
Fujii LL, 2013, GASTROINTEST ENDOSC, V78, P854, DOI 10.1016/j.gie.2013.05.016
Fujii-Lau LL, 2016, SURG ENDOSC, V30, P1396, DOI 10.1007/s00464-015-4342-3
Fujii-Lau LL, 2015, J HEPATO-BIL-PAN SCI, V22, P51, DOI 10.1002/jhbp.187
Garcia-Tsao G, 2007, HEPATOLOGY, V46, P922, DOI 10.1002/hep.21907
Gardner TB, 2011, GASTROINTEST ENDOSC, V73, P718, DOI 10.1016/j.gie.2010.10.053
Ge PS, 2016, GASTROENTEROL CLIN N, V45, P9, DOI 10.1016/j.gtc.2015.10.003
Giovannini M, 2001, ENDOSCOPY, V33, P898, DOI 10.1055/s-2001-17324
Gonzalez JM, 2009, ENDOSCOPY, V41, P721, DOI 10.1055/s-0029-1214874
Gupta K, 2014, J CLIN GASTROENTEROL, V48, P80, DOI 10.1097/MCG.0b013e31828c6822
Hara K, 2011, AM J GASTROENTEROL, V106, P1239, DOI 10.1038/ajg.2011.84
HARADA N, 1995, ENDOSCOPY, V27, P612, DOI 10.1055/s-2007-1005769
Holt BA, 2015, GASTROINTEST ENDOSC, V81, P804, DOI 10.1016/j.gie.2014.12.026
Irani S, 2017, CLIN GASTROENTEROL H, V15, P738, DOI 10.1016/j.cgh.2016.12.021
Irani S, 2015, GASTROINTEST ENDOSC, V82, P1110, DOI 10.1016/j.gie.2015.05.045
Irisawa A, 2001, GASTROINTEST ENDOSC, V53, P77, DOI 10.1067/mge.2001.108479
Itoi T, 2016, GASTROINTEST ENDOSC, V83, P461, DOI 10.1016/j.gie.2015.08.030
Itoi T, 2016, GUT, V65, P193, DOI 10.1136/gutjnl-2015-310348
Itoi T, 2013, DIGEST ENDOSC, V25, P137, DOI 10.1111/den.12084
Itoi T, 2012, GASTROINTEST ENDOSC, V75, P870, DOI 10.1016/j.gie.2011.10.020
Iwashita T, 2012, ENDOSCOPY, V44, P60, DOI 10.1055/s-0030-1256871
Iwashita T, 2013, DIGEST DIS SCI, V58, P2417, DOI 10.1007/s10620-013-2645-6
Kahaleh M, 2004, GASTROINTEST ENDOSC, V60, P138, DOI 10.1016/S0016-5107(04)01528-7
Kahaleh M, 2007, GASTROINTEST ENDOSC, V65, P224, DOI 10.1016/j.gie.2006.05.008
Kawakubo K, 2014, J HEPATO-BIL-PAN SCI, V21, P328, DOI 10.1002/jhbp.27
Khashab M, 2013, SURG ENDOSC, V27, P2068, DOI 10.1007/s00464-012-2712-7
Khashab MA, 2017, ENDOSC INT OPEN, V5, pE275, DOI 10.1055/s-0043-101695

Khashab MA, 2015, GASTROINTEST ENDOSC, V82, P932, DOI 10.1016/j.gie.2015.06.017
Khashab MA, 2015, DIGEST DIS SCI, V60, P557, DOI 10.1007/s10620-014-3300-6
Komaki T, 2011, PANCREATOLOGY, V11, P47, DOI 10.1159/000323508
Kumbhari V, 2015, GASTROINTEST ENDOSC, V81, P1015, DOI 10.1016/j.gie.2014.10.019
Kumbhari V, 2013, ENDOSCOPY, V45, pE326, DOI 10.1055/s-0033-1344875
Kurihara T, 2013, DIGEST ENDOSC, V25, P109, DOI 10.1111/den.12100
Kwan V, 2007, GASTROINTEST ENDOSC, V66, P582, DOI 10.1016/j.gie.2007.02.065
Lahoti S, 2000, GASTROINTEST ENDOSC, V51, P331, DOI 10.1016/S0016-5107(00)70363-4
Lameris R, 2011, GASTROINTEST ENDOSC, V73, P392, DOI 10.1016/j.gie.2010.05.019
Law R, 2015, CLIN GASTROENTEROL H, V13, P808, DOI 10.1016/j.cgh.2014.09.030
Lee YT, 2000, GASTROINTEST ENDOSC, V52, P168, DOI 10.1067/mge.2000.107911
Levy MJ, 2008, GASTROINTEST ENDOSC, V67, P572, DOI 10.1016/j.gie.2007.06.063
Levy MJ, 2008, AM J GASTROENTEROL, V103, P352, DOI 10.1111/j.1572-0241.2007.01616.x
Mallery S, 2004, GASTROINTEST ENDOSC, V59, P100, DOI 10.1016/S0016-5107(03)02300-9
Maranki J, 2009, ENDOSCOPY, V41, P532, DOI 10.1055/s-0029-1214712
MCGAHAN JP, 1989, RADIOLOGY, V173, P481, DOI 10.1148/radiology.173.2.2678261
Nguyen AJ, 2002, GASTROINTEST ENDOSC, V55, P572, DOI 10.1067/mge.2002.122032
Ogura T, 2014, J GASTROEN HEPATOL, V29, P1815, DOI 10.1111/jgh.12598
Oh HC, 2007, ENDOSCOPY, V39, P731, DOI 10.1055/s-2007-966577
Paik WH, 2014, WORLD J GASTROENTERO, V20, P5051, DOI 10.3748/wjg.v20.i17.5051
Park DH, 2013, GASTROINTEST ENDOSC, V78, P91, DOI 10.1016/j.gie.2013.01.042
Park DH, 2011, GASTROINTEST ENDOSC, V74, P1276, DOI 10.1016/j.gie.2011.07.054
Park DH, 2009, AM J GASTROENTEROL, V104, P2168, DOI 10.1038/ajg.2009.254
Park SJ, 2013, GASTROINTEST ENDOSC, V78, P374, DOI 10.1016/j.gie.2013.04.183
Peñas-Herrero I, 2015, J HEPATO-BIL-PAN SCI, V22, P35, DOI 10.1002/jhbp.182
Penn DE, 2012, GASTROINTEST ENDOSC, V76, P679, DOI 10.1016/j.gie.2012.04.457
Perez-Miranda M, 2017, J CLIN GASTROENTEROL, V51, P896, DOI 10.1097/MCG.0000000000000887
Poincloux L, 2015, ENDOSCOPY, V47, P794, DOI [10.1055/s-0035-1545399, 10.1055/s-0034-1391988]
Rinninella E, 2015, GASTROINTEST ENDOSC, V82, P1039, DOI 10.1016/j.gie.2015.04.006
Roach H, 2005, ENDOSCOPY, V37, P876, DOI 10.1055/s-2005-870201
Romero-Castro R, 2010, ENDOSCOPY, V42, pE35, DOI 10.1055/s-0029-1215261
Romero-Castro R, 2007, GASTROINTEST ENDOSC, V66, P402, DOI 10.1016/j.gie.2007.03.008
Romero-Castro R, 2013, GASTROINTEST ENDOSC, V78, P711, DOI 10.1016/j.gie.2013.05.009
SARIN SK, 1992, HEPATOLOGY, V16, P1343, DOI 10.1002/hep.1840160607
Saxena P, 2015, GASTROINTEST ENDOSC, V81, P1010, DOI 10.1016/j.gie.2014.06.038
Seewald S, 2008, GASTROINTEST ENDOSC, V68, P447, DOI 10.1016/j.gie.2008.02.050
Seewald S, 2009, DIGEST ENDOSC, V21, pS61, DOI 10.1111/j.1443-1661.2009.00860.x
Seifert H, 2009, GUT, V58, P1260, DOI 10.1136/gut.2008.163733
Shah JN, 2012, GASTROINTEST ENDOSC, V75, P56, DOI 10.1016/j.gie.2011.08.032
Shah RJ, 2015, CLIN GASTROENTEROL H, V13, P747, DOI 10.1016/j.cgh.2014.09.047
Sharaiha RZ, 2016, CLIN GASTROENTEROL H, V14, P1797, DOI 10.1016/j.cgh.2016.05.011
Sharaiha RZ, 2015, GASTROINTEST ENDOSC, V82, P822, DOI 10.1016/j.gie.2015.02.035

Sharma M, 2010, GASTROINTEST ENDOSC, V72, P1106, DOI 10.1016/j.gie.2010.02.054
Singhal S, 2013, CLIN ENDOSC, V46, P506, DOI 10.5946/ce.2013.46.5.506
Storm AC, 2014, GASTROINTEST ENDOSC, V80, P164, DOI 10.1016/j.gie.2014.04.005
Tabibian JH, 2016, HEPATOLOGY, V63, P1398, DOI 10.1002/hep.27925
Tan YM, 2002, GASTROINTEST ENDOSC, V55, P276, DOI 10.1067/mge.2001.118651
Teoh AYB, 2017, ENDOSCOPY, V49, P130, DOI 10.1055/s-0042-119036
Teoh AYB, 2014, GASTROINTEST ENDOSC, V80, P1171, DOI 10.1016/j.gie.2014.03.038
Tessier G, 2007, GASTROINTEST ENDOSC, V65, P233, DOI 10.1016/j.gie.2006.06.029
Trikudanathan G, 2014, AM J GASTROENTEROL, V109, P969, DOI 10.1038/ajg.2014.130
Tringali A, 2014, GASTROINTEST ENDOSC, V79, P66, DOI 10.1016/j.gie.2013.06.032
Tsuyuguchi T, 2013, J HEPATO-BIL-PAN SCI, V20, P81, DOI 10.1007/s00534-012-0570-2
Tyberg A, 2017, GASTROINTEST ENDOSC, V85, P164, DOI 10.1016/j.gie.2016.07.030
Tyberg A, 2016, ENDOSC INT OPEN, V4, pE276, DOI 10.1055/s-0042-101789
Tyberg A, 2016, GASTROINTEST ENDOSC, V83, P254, DOI 10.1016/j.gie.2015.08.003
Varadarajulu S, 2008, GASTROINTEST ENDOSC, V68, P649, DOI 10.1016/j.gie.2008.02.057
Varadarajulu S, 2013, GASTROENTEROLOGY, V145, P583, DOI 10.1053/j.gastro.2013.05.046
Varadarajulu S, 2011, J GASTROINTEST SURG, V15, P2080, DOI 10.1007/s11605-011-1621-8
Vila JJ, 2012, GASTROINTEST ENDOSC, V76, P1133, DOI 10.1016/j.gie.2012.08.001
VOEGELI DR, 1985, AM J SURG, V150, P243, DOI 10.1016/0002-9610(85)90129-1
Walter D, 2016, GUT, V65, P6, DOI 10.1136/gutjnl-2015-309925
Walter D, 2015, ENDOSCOPY, V47, P63, DOI 10.1055/s-0034-1378113
Wang KX, 2016, GASTROINTEST ENDOSC, V83, P1218, DOI 10.1016/j.gie.2015.10.033
Weilert F, 2014, SURG ENDOSC, V28, P3193, DOI 10.1007/s00464-014-3588-5
Weilert F, 2012, GASTROINTEST ENDOSC, V76, P915, DOI 10.1016/j.gie.2011.09.027
Weilert Frank, 2012, Gastrointest Endosc Clin N Am, V22, P303, DOI 10.1016/j.giec.2012.04.019
WIERSEMA MJ, 1994, GASTROINTEST ENDOSC, V40, P700, DOI 10.1016/S0016-5107(94)70113-X
Wiersema MJ, 1996, GASTROINTEST ENDOSC, V43, P102, DOI 10.1016/S0016-5107(06)80108-2
Will U, 2007, ENDOSCOPY, V39, P292, DOI 10.1055/s-2007-966215
Will U, 2015, WORLD J GASTROENTERO, V21, P13140, DOI 10.3748/wjg.v21.i46.13140
Wright G, 2009, ENDOSCOPY, V41, pE56, DOI 10.1055/s-2008-1077564
Yu C. F., 2007, AM J EMERG MED, V25, P860
Zerem E, 2014, WORLD J GASTROENTERO, V20, P13879, DOI 10.3748/wjg.v20.i38.13879
NR 124
TC 36
Z9 44
U1 0
U2 4
PU W B SAUNDERS CO-ELSEVIER INC
PI PHILADELPHIA
PA 1600 JOHN F KENNEDY BOULEVARD, STE 1800, PHILADELPHIA, PA 19103-2899 USA
SN 0016-5085
EI 1528-0012

J9 GASTROENTEROLOGY
JI Gastroenterology
PD MAY
PY 2018
VL 154
IS 7
SI SI
BP 1911
EP 1924
DI 10.1053/j.gastro.2017.12.046
PG 14
WC Gastroenterology & Hepatology
WE Science Citation Index Expanded (SCI-EXPANDED)
SC Gastroenterology & Hepatology
GA GG1RW
UT WOS:000432465500006
PM 29458153
DA 2026-05-09
ER

PT J
AU Song, TJ
Moon, JH
Lee, YN
Lee, SS
AF Song, Tae Jun
Moon, Jong Ho
Lee, Yun Nah
Lee, Sang Soo
TI Design considerations of the novel lumen-apposing metal stents (Niti-S
SPAXUS)
SO TECHNIQUES AND INNOVATIONS IN GASTROINTESTINAL ENDOSCOPY
LA English
DT Article
DE Lumen-apposing metal stents; Pseudocyst; Gallbladder; Drainage
AB Endoscopic ultrasound (EUS)-guided drainage, a minimally invasive procedure, is being used more frequently for various pancreatobiliary diseases. However, one of drawbacks of various EUS-guided procedures is that they need advanced endoscopic techniques. Therefore, dedicated devices for EUS-guided interventions are required to ensure safe and effective procedures. The SPAXUS stent (Taewoong Medical, Gyeonggi-do, South Korea), a novel lumen-apposing metal stent, was developed for EUS-guided drainage procedures and has shown promising technical and clinical success rates for pancreatic fluid collections, bile duct, or gallbladder drainage. Its distinctive design improves on previous lumen-apposing metal stent designs and decreases stent-related adverse events. EUS-guided interventions with a dedicated stent are promising. This

manuscript will review the characteristics of the SPAXUS stent, its usage and clinical outcomes. (c) 2019 Elsevier Inc. All rights reserved.

C1 [Song, Tae Jun; Lee, Sang Soo] Univ Ulsan, Coll Med, Asan Med Ctr, Dept Gastroenterol, Seoul, South Korea.

[Moon, Jong Ho; Lee, Yun Nah] SoonChunHyang Univ, Bucheon Hosp, Sch Med, Digest Dis Ctr,Dept Internal Med, 170 Jomaru Ro, Bucheon 14584, South Korea.

C3 University of Ulsan; Asan Medical Center; Soonchunhyang University

RP Moon, JH (corresponding author), SoonChunHyang Univ, Bucheon Hosp, Sch Med, Digest Dis Ctr,Dept Internal Med, 170 Jomaru Ro, Bucheon 14584, South Korea.

EM jhmoon@schmc.ac.kr

RI Lee, Yun/J-1260-2014

CR Adler DG, 2017, ENDOSC ULTRASOUND, V6, P389, DOI 10.4103/eus.eus_4_17

Anthony Y, 2019, GASTROINTEST ENDOSC, V89

Bang JY, 2017, GUT, V66, P2054, DOI 10.1136/gutjnl-2016-312812

Binmoeller KF, 2011, ENDOSCOPY, V43, P337, DOI 10.1055/s-0030-1256127

Bukhari M, 2018, GASTROINTEST ENDOSC, V88, P486, DOI 10.1016/j.gie.2018.04.2356

Cho DH, 2019, SURG ENDOSC, V33, P2135, DOI 10.1007/s00464-018-6485-5

DeSimone ML, 2017, WORLD J GASTRO ENDOS, V9, P456, DOI 10.4253/wjge.v9.i9.456

Dollhopf M, 2017, GASTROINTEST ENDOSC, V86, P636, DOI 10.1016/j.gie.2017.02.027

Fabbri C, 2015, GASTROINTEST ENDOSC, V82, P585, DOI 10.1016/j.gie.2015.04.035

Itoi T, 2016, GUT, V65, P193, DOI 10.1136/gutjnl-2015-310348

Itoi T, 2013, GASTROINTEST ENDOSC, V78, P934, DOI 10.1016/j.gie.2013.09.025

Itoi T, 2012, GASTROINTEST ENDOSC, V75, P870, DOI 10.1016/j.gie.2011.10.020

Itoi T, 2010, GASTROINTEST ENDOSC, V71, P1038, DOI 10.1016/j.gie.2010.01.026

Jonnalagadda SS., 2015, GASRTROINTESTINAL EN

Khashab MA, 2015, GASTROINTEST ENDOSC, V81, P1234, DOI 10.1016/j.gie.2014.12.053

Lee JY, 2019, GASTROINTEST ENDOSC, V89, pAB321, DOI 10.1016/j.gie.2019.03.420

Moon JH, 2018, GASTROINTEST ENDOSC, V87, pAB465, DOI 10.1016/j.gie.2018.04.2016

Moon JH, 2014, GASTROINTEST ENDOSC, V79, P990, DOI 10.1016/j.gie.2014.02.015

Rinninella E, 2015, GASTROINTEST ENDOSC, V82, P1039, DOI 10.1016/j.gie.2015.04.006

Seewald S, 2009, GASTROINTEST ENDOSC, V69, pS13, DOI 10.1016/j.gie.2008.10.061

Song TJ, 2019, GASTROINTEST ENDOSC, V90, P507, DOI 10.1016/j.gie.2019.05.033

Stier Matthew W, 2018, Gastrointest Endosc Clin N Am, V28, P207, DOI [10.1016/j.giec.2017.11.008, 10.1016/j.giec.2017.11.008]

Teoh AYB, 2020, DIGEST ENDOSC, V32, P391, DOI 10.1111/den.13494

NR 23

TC 6

Z9 8

U1 0

U2 0

PU ELSEVIER SCIENCE INC

PI NEW YORK

PA STE 800, 230 PARK AVE, NEW YORK, NY 10169 USA

SN 2666-5107
EI 2590-0307
J9 TECH INNOVAT GASTROI
JI Tech. Innovation Gastrointes. Endoscopy
PD JAN
PY 2020
VL 22
IS 1
SI SI
BP 9
EP 13
DI 10.1016/j.tgie.2019.150637
PG 5
WC Gastroenterology & Hepatology
WE Emerging Sources Citation Index (ESCI)
SC Gastroenterology & Hepatology
GA VK9JD
UT WOS:000768800500004
OA Bronze
DA 2026-05-09
ER

PT J
AU Lam, BKH
Teoh, AYB
AF Lam, Brian Kin Hang
Teoh, Anthony Yuen Bun
TI Recent development of endoscopic ultrasound-guided gallbladder drainage
SO INTERNATIONAL JOURNAL OF GASTROINTESTINAL INTERVENTION
LA English
DT Review
DE Acute cholecystitis; Endoscopic ultrasonography-guided gallbladder drainage; Malignant biliary obstruction; Percutaneous cholecystostomy
ID LONG-TERM OUTCOMES; LUMEN-APPOSING STENT; PERCUTANEOUS CHOLECYSTOSTOMY; ACUTE CHOLECYSTITIS; METAL STENT; OBSTRUCTION; MANAGEMENT; PLACEMENT
AB Endoscopic ultrasonography-guided gallbladder drainage (EUS-GBD) has emerged as a promising alternative treatment to high-risk patients suffering from acute cholecystitis and are unfit for surgery. EUS-GBD has similar rates of technical and clinical success compared to other alternative treatments, but the procedure has been shown to reduce adverse event rates and the need for repeated interventions. This review aims to provide a summary of the recent development in the EUS-GBD technique with various devices employed in the procedure. Copyright (C) 2020, Society of Gastrointestinal Intervention.
C1 [Lam, Brian Kin Hang; Teoh, Anthony Yuen Bun] Chinese Univ Hong Kong, Prince Wales Hosp, Dept Surg, Shatin, 4-F Li Ka Shing Med Sci Bldg,Ngan Shing St, Hong Kong, Peoples R China.

C3 Prince of Wales Hospital Hong Kong; Chinese University of Hong Kong
RP Teoh, AYB (corresponding author), Chinese Univ Hong Kong, Prince Wales Hosp, Dept Surg, Shatin, 4-F Li Ka Shing Med Sci Bldg,Ngan Shing St, Hong Kong, Peoples R China.
EM anthonyteoh@surgery.cuhk.edu.hk
RI Teoh, Anthony/L-4796-2016
OI Teoh, Anthony/0000-0002-1885-8035
CR Alvino DML, 2017, J GASTROINTEST SURG, V21, P761, DOI 10.1007/s11605-017-3375-4
Chan JHY, 2018, CLIN ENDOSC, V51, P150, DOI 10.5946/ce.2017.125
Chang JI, 2019, ENDOSC INT OPEN, V7, pE655, DOI 10.1055/a-0826-4309
Chantarojanasiri T, 2017, SAUDI J GASTROENTERO, V23, P218, DOI 10.4103/sjg.SJG_115_17
Cho SH, 2020, GASTROINTEST ENDOSC, V91, P543, DOI 10.1016/j.gie.2019.09.042
Choi JH, 2014, ENDOSCOPY, V46, P656, DOI 10.1055/s-0034-1365720
Dollhopf M, 2017, GASTROINTEST ENDOSC, V86, P636, DOI 10.1016/j.gie.2017.02.027
Higa JT, 2019, GASTROINTEST ENDOSC, V90, P483, DOI 10.1016/j.gie.2019.04.238
Imai H, 2016, GASTROINTEST ENDOSC, V84, P147, DOI 10.1016/j.gie.2015.12.024
Irani S, 2017, CLIN GASTROENTEROL H, V15, P738, DOI 10.1016/j.cgh.2016.12.021
James TW, 2019, ENDOSC ULTRASOUND, V8, pS28, DOI 10.4103/eus.eus_41_19
Jang JW, 2012, GASTROENTEROLOGY, V142, P805, DOI 10.1053/j.gastro.2011.12.051
Kahaleh M, 2016, DIGEST LIVER DIS, V48, P1054, DOI 10.1016/j.dld.2016.05.021
Kamata K, 2017, WORLD J GASTROENTERO, V23, P661, DOI 10.3748/wjg.v23.i4.661
Kedia P, 2015, GASTROINTEST ENDOSC, V82, P1031, DOI 10.1016/j.gie.2015.03.1912
Krishnamoorthi R, 2020, SURG ENDOSC, V34, P1904, DOI 10.1007/s00464-020-07409-7
Law R, 2016, CLIN GASTROENTEROL H, V14, P476, DOI 10.1016/j.cgh.2015.10.026
Lee SS, 2007, GASTROINTEST ENDOSC, V66, P1008, DOI 10.1016/j.gie.2007.03.1080
McKay A, 2012, SURG ENDOSC, V26, P1343, DOI 10.1007/s00464-011-2035-0
Minaga K, 2019, DIGEST ENDOSC, V31, P180, DOI 10.1111/den.13242
Mohan BP, 2020, ENDOSCOPY, V52, P96, DOI 10.1055/a-1020-3932
Mori Y, 2018, J HEPATO-BIL-PAN SCI, V25, P87, DOI 10.1002/jhbp.504
Ogura T, 2019, DIGEST ENDOSC, V31, P55, DOI 10.1111/den.13334
Oh D, 2019, GASTROINTEST ENDOSC, V89, P289, DOI 10.1016/j.gie.2018.08.052
Peñas-Herrero I, 2015, J HEPATO-BIL-PAN SCI, V22, P35, DOI 10.1002/jhbp.182
Podboy A, 2021, GASTROINTEST ENDOSC, V93, P797, DOI 10.1016/j.gie.2020.09.040
Siddiqui A, 2019, SURG ENDOSC, V33, P1260, DOI 10.1007/s00464-018-6406-7
Song TJ, 2010, GASTROINTEST ENDOSC, V71, P634, DOI 10.1016/j.gie.2009.11.024
Teoh AYB, 2020, GUT, V69, P1085, DOI 10.1136/gutjnl-2019-319996
Teoh AY, 2016, GASTROINTEST ENDOSC, V84, P62, DOI 10.1016/j.gie.2015.11.041
Teoh AYB, 2021, GASTROINTEST ENDOSC, V93, P577, DOI 10.1016/j.gie.2020.06.066
Teoh AYB, 2017, ENDOSCOPY, V49, P130, DOI 10.1055/s-0042-119036
Teoh AYB, 2014, GASTROINTEST ENDOSC, V80, P1171, DOI 10.1016/j.gie.2014.03.038
TEOH AY, 2018, GASTROINTEST ENDOS S, V87, pAB33
Tsuyuguchi T, 2013, J HEPATO-BIL-PAN SCI, V20, P81, DOI 10.1007/s00534-012-0570-2
Tyberg A, 2018, J CLIN GASTROENTEROL, V52, P79, DOI 10.1097/MCG.0000000000000786

Walter D, 2016, GUT, V65, P6, DOI 10.1136/gutjnl-2015-309925
Widmer J, 2014, DIGEST ENDOSC, V26, P525, DOI 10.1111/den.12221
NR 38
TC 1
Z9 1
U1 0
U2 1
PU SOC GASTROINTESTINAL INTERVENTION
PI SEOUL
PA 12, HAKDONG-RO 31, GANGNAM-GU, SEOUL, 00000, SOUTH KOREA
SN 2636-0004
EI 2636-0012
J9 INT J GASTROINT INT
JI Int. J. Gastrointest. Interv.
PD OCT
PY 2020
VL 9
IS 4
BP 148
EP 153
DI 10.18528/ijgii200046
PG 6
WC Gastroenterology & Hepatology
WE Emerging Sources Citation Index (ESCI)
SC Gastroenterology & Hepatology
GA OJ3OQ
UT WOS:000583875100005
OA Green Submitted, gold
DA 2026-05-09
ER

PT J
AU Parihar, V
Basir, Y
Nally, D
Mellotte, G
Manoharan, T
Walker, C
Ridgway, PF
Conlon, KC
Breslin, N
Harewood, GC

Ryan, BM

AF Parihar, Vikrant

Basir, Yasir

Nally, Deirdre

Mellotte, Gregory

Manoharan, Thilagraj

Walker, Caroline

Ridgway, P. F.

Conlon, K. C.

Breslin, Niall

Harewood, Gavin C.

Ryan, Barbara M.

TI A novel value-based scoring system for endoscopic ultrasound-guided drainage of pancreatic fluid collections: a single-centre comparative study of plastic and lumen-apposing metal stents (NOVA study)

SO EUROPEAN JOURNAL OF GASTROENTEROLOGY & HEPATOLOGY

LA English

DT Article

DE complexity; double pigtail plastic stent; endoscopic ultrasound; lumen-apposing metal stent; pancreatic fluid collection; value

ID WALLED-OFF NECROSIS; STEP-UP APPROACH; MANAGEMENT; NECROSECTOMY; SEDATION; QUALITY

AB Objective Healthcare resources are finite. Value in healthcare can be defined as patient health outcomes achieved per monetary unit spent. Attempts have been made to quantify the value of luminal endoscopy, but there is little in the medical literature describing the value of the complex therapeutic endoscopic activity. This study aimed to characterise the value of endoscopic ultrasound (EUS)-guided drainage of pancreatic fluid collections (PFCs) with either plastic or lumen-apposing metal stents (LAMSs).

Methods This is a single-centre, retrospective-prospective comparative study of 39 patients, who underwent EUS-guided PFC drainage between 2009 and 2018. Procedure value was calculated using the formula Q/(T/C), where Q is the quality of procedure adjusted for complications, T procedure duration and C is the complexity adjustment. Quality and complexity were estimated on a 1-4 Likert scale based on the American Society for Gastrointestinal Endoscopy criteria. Time (in minutes) was recorded from the patient entering and leaving the procedure room. Endoscopy time calculated from procedure time was considered a surrogate marker of cost as individual components of procedure cost were not itemized.

Results Of 39 identified patients who underwent EUS-guided PFC drainage, 11 received double pigtail plastic stents (DPPSs) and 28 received LAMSs. The two groups were comparable in age, gender and aetiology. Nearly 40% of the LAMS interventions were considered high value but only 11% of the plastic stent interventions achieved the same. The difference predominantly was due to a higher rate of complications and longer procedure time.

Conclusion In this single-centre study, EUS-guided PFC drainage using LAMS was found to be a higher value procedure compared to the use of DPPS. Copyright (C) 2020 Wolters Kluwer Health, Inc. All rights reserved.

C1 [Parihar, Vikrant; Mellotte, Gregory; Manoharan, Thilagraj; Walker, Caroline; Breslin, Niall; Ryan, Barbara M.] Tallaght Univ Hosp, Dept Gastroenterol, Dublin 24, Ireland.

[Basir, Yasir; Nally, Deirdre; Ridgway, P. F.; Conlon, K. C.] Tallaght Univ Hosp, Dept Surg, Dublin, Ireland.

[Basir, Yasir; Nally, Deirdre; Ridgway, P. F.; Conlon, K. C.] Trinity Coll Dublin, Dublin, Ireland.
[Ryan, Barbara M.] Trinity Coll Dublin, Dept Clin Med, Dublin, Ireland.
[Harewood, Gavin C.] Beaumont Univ Hosp, Dept Gastroenterol, Dublin, Ireland.
C3 Trinity College Dublin; Trinity College Dublin
RP Parihar, V (corresponding author), Tallaght Univ Hosp, Dept Gastroenterol, Dublin 24, Ireland.
EM Vikpar37@yahoo.com
RI ; Harewood, Gavin/GQB-0141-2022
OI Ridgway, Paul/0000-0002-8500-8532; Nally, Deire/0000-0001-8590-0021;
Bashir, Yasir/0000-0001-8902-8883;
CR Bang JY, 2019, GASTROENTEROLOGY, V156, P1027, DOI 10.1053/j.gastro.2018.11.031
Bang JY, 2019, GUT, V68, P1200, DOI 10.1136/gutjnl-2017-315335
Bang JY, 2018, ANN SURG, V267, P561, DOI 10.1097/SLA.0000000000002082
Banks PA, 2013, GUT, V62, P102, DOI 10.1136/gutjnl-2012-302779
Campbell JA, 2017, WORLD J GASTROENTERO, V23, P560, DOI 10.3748/wjg.v23.i3.560
Cotton PB, 2011, GASTROINTEST ENDOSC, V73, P868, DOI 10.1016/j.gie.2010.12.036
De Angelis CG, 2019, SAUDI J GASTROENTERO, V25, P335, DOI 10.4103/sjg.SJG_166_19
Habashi S, 2009, WORLD J GASTROENTERO, V15, P38, DOI 10.3748/wjg.15.38
Harewood GC, 2015, CLIN GASTROENTEROL H, V13, P1377, DOI 10.1016/j.cgh.2014.07.048
Jacobson BC, 2005, GASTROINTEST ENDOSC, V61, P363, DOI 10.1016/S0016-5107(04)02779-8
Lakhtakia S, 2017, GASTROINTEST ENDOSC, V85, P1243, DOI 10.1016/j.gie.2016.10.037
Lang GD, 2018, GASTROINTEST ENDOSC, V87, P150, DOI 10.1016/j.gie.2017.06.029
Moran CP, 2017, GASTROINTEST ENDOSC, V85, pAB92, DOI 10.1016/j.gie.2017.03.134
Nayar DS, 2010, DIGEST DIS SCI, V55, P2537, DOI 10.1007/s10620-010-1308-0
Parihar V, 2018, EUR J GASTROEN HEPAT, V30, P718, DOI 10.1097/MEG.0000000000001129
Parihar V, 2017, EUR J GASTROEN HEPAT, V29, P371, DOI 10.1097/MEG.0000000000000818
Porter ME, 2010, NEW ENGL J MED, V363, P2477, DOI 10.1056/NEJMp1011024
Rosenthal MB, 2006, NEW ENGL J MED, V355, P1895, DOI 10.1056/NEJMsa063682
Seewald S, 2009, DIGEST ENDOSC, V21, pS61, DOI 10.1111/j.1443-1661.2009.00860.x
Siddiqui AA, 2017, GASTROINTEST ENDOSC, V85, P758, DOI 10.1016/j.gie.2016.08.014
Singhal S, 2013, CLIN ENDOSC, V46, P506, DOI 10.5946/ce.2013.46.5.506
Song ZR, 2012, HEALTH AFFAIR, V31, P1885, DOI 10.1377/hlthaff.2012.0327
Tyberg A, 2016, WORLD J GASTROENTERO, V22, P2256, DOI 10.3748/wjg.v22.i7.2256
van Santvoort HC, 2010, NEW ENGL J MED, V362, P1491, DOI 10.1056/NEJMoa0908821
Varadarajulu S, 2011, J GASTROINTEST SURG, V15, P2080, DOI 10.1007/s11605-011-1621-8
Venkatachalapathy SV, 2018, ENDOSC INT OPEN, V6, pE259, DOI 10.1055/s-0043-125362
Wani S, 2015, AM J GASTROENTEROL, V110, P102, DOI 10.1038/ajg.2014.387
Yang JL, 2019, ENDOSCOPY, V51, P1035, DOI 10.1055/a-0759-1353
Zauber Ann G, 2010, Gastrointest Endosc Clin N Am, V20, P751, DOI 10.1016/j.giec.2010.07.008
NR 29
TC 2
Z9 3
U1 0

U2 0
PU LIPPINCOTT WILLIAMS & WILKINS
PI PHILADELPHIA
PA TWO COMMERCE SQ, 2001 MARKET ST, PHILADELPHIA, PA 19103 USA
SN 0954-691X
EI 1473-5687
J9 EUR J GASTROEN HEPAT
JI Eur. J. Gastroenterol. Hepatol.
PD FEB
PY 2021
VL 33
IS 2
BP 157
EP 162
DI 10.1097/MEG.0000000000001891
PG 6
WC Gastroenterology & Hepatology
WE Science Citation Index Expanded (SCI-EXPANDED)
SC Gastroenterology & Hepatology
GA ZB6TD
UT WOS:000756971300005
PM 32804857
DA 2026-05-09
ER

PT J
AU Okuzono, T
Miyamoto, K
AF Okuzono, Toru
Miyamoto, Ko-ichiro
TI Novel anchoring device for endoscopic ultrasound-guided gallbladder
drainage: Secondary publication
SO JOURNAL OF HEPATO-BILIARY-PANCREATIC SCIENCES
LA English
DT Article
DE acute cholecystitis; drainage; EUS; gallbladder; PTGBD
ID APPOSING METAL STENT; HIGH-RISK PATIENTS; PERCUTANEOUS CHOLECYSTOSTOMY;
ACUTE CHOLECYSTITIS; BILIARY OBSTRUCTION; MANAGEMENT
AB Background Recently, endoscopic ultrasound-guided gallbladder drainage has attracted much attention. However, the risk management of adverse events and techniques to avoid them are not yet mature. Difficulty dilating the fistula with a dilator or placing a stent for drainage often prolongs the procedure time, which increases the risk of peritonitis or the procedure failure rate. Therefore, the result of the procedure will be unstable, and one cause is the lack of adhesion between

the gallbladder and the digestive tract walls. Methods We developed an anchor to fix the stomach and gallbladder walls prior to endoscopic ultrasound-guided gallbladder drainage in four live pigs using the anchor. Results The stomach and gallbladder walls were fixed in three pigs, and technical success was achieved in all three pigs. In two pigs that were dissected 17 and 34 days post-procedure, respectively, fixation occurred in one pig. In the other pig, anchoring of the gallbladder and stomach walls did not occur because the wings of the anchor in the gallbladder were damaged. Conclusions Although issues remain regarding efficacy and safety, we plan to make improvements in this novel device and aim for clinical application.
C1 [Okuzono, Toru] Sendai Kousei Hosp, Dept Gastroenterol, Sendai, Miyagi, Japan.
[Miyamoto, Ko-ichiro] Tohoku Univ, Dept Elect Engn, Sendai, Miyagi, Japan.
C3 Sendai Kousei Hospital; Tohoku University
RP Okuzono, T (corresponding author), Sendai Kousei Hosp, Dept Gastroenterol, Aoba Ku, Hirosecho 4-15, Sendai, Miyagi, Japan.
EM okuzonotoru@gmail.com
RI MIYAMOTO, Ko-ichiro/E-1673-2013
OI MIYAMOTO, Ko-ichiro/0000-0001-7889-6714; Okuzono, Toru/0000-0001-6999-6254
FU Japan Science and Technology Agency [JPMJST2018, JPMJST1940]; Japan Agency for Medical Research and Development [JPA-153]
FX Japan Science and Technology Agency, Grant/Award Number: JPMJST2018 and JPMJST1940; Japan Agency for Medical Research and Development, Grant/Award Number: JPA-153
CR Baron TH, 2007, GASTROINTEST ENDOSC, V65, P735, DOI 10.1016/j.gie.2006.07.041
Choi JH, 2014, ENDOSCOPY, V46, P656, DOI 10.1055/s-0034-1365720
Davis CA, 1999, ARCH SURG-CHICAGO, V134, P727, DOI 10.1001/archsurg.134.7.727
de la Serna-Higuera C, 2013, GASTROINTEST ENDOSC, V77, P303, DOI 10.1016/j.gie.2012.09.021
Granlund N, 2001, LANGENBECK ARCH SURG, V386, P212, DOI 10.1007/s004230100211
Imai H, 2016, GASTROINTEST ENDOSC, V84, P147, DOI 10.1016/j.gie.2015.12.024
Irani S, 2017, CLIN GASTROENTEROL H, V15, P738, DOI 10.1016/j.cgh.2016.12.021
Itoi T, 2012, GASTROINTEST ENDOSC, V75, P870, DOI 10.1016/j.gie.2011.10.020
Jang JW, 2012, GASTROENTEROLOGY, V142, P805, DOI 10.1053/j.gastro.2011.12.051
Kiviniemi H, 1998, INT SURG, V83, P299
Kwan V, 2007, GASTROINTEST ENDOSC, V66, P582, DOI 10.1016/j.gie.2007.02.065
LEE MJ, 1991, AM J ROENTGENOL, V156, P1163, DOI 10.2214/ajr.156.6.2028859
Lee SS, 2007, GASTROINTEST ENDOSC, V66, P1008, DOI 10.1016/j.gie.2007.03.1080
LO LD, 1995, J VASC INTERV RADIOL, V6, P629, DOI 10.1016/S1051-0443(95)71150-2
Luigiano C, 2012, DIGEST LIVER DIS, V44, P128, DOI 10.1016/j.dld.2011.08.015
Maruta A, 2021, J HEPATO-BIL-PAN SCI, V28, P1138, DOI 10.1002/jhbp.967
Maruta A, 2020, J HEPATO-BIL-PAN SCI, V27, P429, DOI 10.1002/jhbp.744
Matsubara S., 2015, J BILIARY TRACT PANC, V36, P785
Miura F, 2018, J HEPATO-BIL-PAN SCI, V25, P31, DOI 10.1002/jhbp.509
Mori Y, 2018, J HEPATO-BIL-PAN SCI, V25, P87, DOI 10.1002/jhbp.504
Murabayashi T, 2018, ENDOSCOPY, V50, pE642, DOI 10.1055/a-0591-2630

Takagi W, 2016, THER ADV GASTROENTER, V9, P19, DOI 10.1177/1756283X15609285
Walter D, 2016, GUT, V65, P6, DOI 10.1136/gutjnl-2015-309925
Zhang K, 2018, GASTROINTEST ENDOSC, V88, P957, DOI 10.1016/j.gie.2018.07.019
NR 24
TC 1
Z9 2
U1 0
U2 2
PU WILEY
PI HOBOKEN
PA 111 RIVER ST, HOBOKEN 07030-5774, NJ USA
SN 1868-6974
EI 1868-6982
J9 J HEPATO-BIL-PAN SCI
JI J. Hepato-Biliary-Pancreat. Sci.
PD JUL
PY 2022
VL 29
IS 7
BP 825
EP 831
DI 10.1002/jhbp.1139
EA APR 2022
PG 7
WC Gastroenterology & Hepatology; Surgery
WE Science Citation Index Expanded (SCI-EXPANDED)
SC Gastroenterology & Hepatology; Surgery
GA 3C2FN
UT WOS:000776552800001
PM 35315973
DA 2026-05-09
ER

PT J
AU Pinto-Carta, R
Solano, J
Cabrera, LF
Sanchez, A
Moreno, L
Pedraza, M
AF Pinto-Carta, Renzo
Solano, Jaime

Cabrera, Luis Felipe
Sanchez, Alvaro
Moreno, Luisa
Pedraza, Mauricio
TI Endoscopic ultrasound (EUS) guided choledocoduodenostomy in a patient with irresecable pancreas cancer and biliar obstruction using a luminal apposing metal stent (LAMS)
SO INTERNATIONAL JOURNAL OF SURGERY CASE REPORTS
LA English
DT Article
DE Endoscopic ultrasound; Endoscopic ultrasound-guided biliary drainage; Choledocoduodenostomy; Luminal apposing stent (LAMS); Biliary obstruction; Pancreatic cancer
AB The endoscopic retrograde cholangiopancreatography (ERCP) is the election treatment of biliary obstruction. EUS-guided biliary drainage (EUS-BD) has emerged as an alternative to PTBD with a high technical and clinical success rate, low risk of complications and a better quality of life for the patient. Case presentation we present the first case in Colombia of EUS-guided biliary drainage using luminal apposing metal stent (LAMS) in a patient with unresectable pancreatic adenocarcinoma with biliary obstruction in who ERCP was failed due to neoplastic invasion of the Vater papilla. Discussion Transhepatic biliary drainage has been the most common procedure for treatment of malignant biliary obstruction in cases which ERCP fails due to tumor infiltration of the duodenum or the Vater papilla. During the last decade the development of endoscopic ultrasound (EUS) has implied an alternative for biliary drainage in cases of failed ERCP, demonstrating advantages over Trans hepatic biliary drainage Conclusion Considering that EUS-CD is a safe procedure, with a high rate of technical and clinical success, low risk of complications. (C) 2020 The Author(s). Published by Elsevier Ltd on behalf of IJS Publishing Group Ltd.
C1 [Pinto-Carta, Renzo; Solano, Jaime] Santa Fe Fdn Univ Hosp Bogota, Dept Gastroenterol & Digest Endoscopy, Bogota, Colombia.
[Solano, Jaime; Cabrera, Luis Felipe] Fdn Santa Fe Bogota, Dept Surg, Bogota, Colombia.
[Cabrera, Luis Felipe; Sanchez, Alvaro; Pedraza, Mauricio] Univ El Bosque, Dept Gen Surg, Bogota, Colombia.
[Moreno, Luisa] Univ El Bosque, Dept Internal Med, Bogota, Colombia.
[Cabrera, Luis Felipe] Univ Los Andes, Dept Med, Bogota, Colombia.
C3 Universidad El Bosque; Universidad El Bosque; Universidad de los Andes (Colombia)
RP Pinto-Carta, R (corresponding author), Fdn Santa Fe Bogota, Gastroenterol Intervencionista Endosonografista, Bogota, Colombia.
EM renso.pinto@fsfb.org.co; luis.felipe.cabrera@hotmail.com; mpedraza93@gmail.com
OI Sánchez Hernández, Alvaro Eduardo/0000-0002-5899-1981; Peaza Ciro, Mauricio/0000-0001-8726-5578
CR Agha RA, 2018, INT J SURG, V60, P279, DOI 10.1016/j.ijsu.2018.10.031
El Chafic AH, 2019, DIGEST DIS SCI, V64, P3321, DOI 10.1007/s10620-019-05688-2
Giovannini M, 2001, ENDOSCOPY, V33, P898, DOI 10.1055/s-2001-17324
Han SY, 2019, SCI REP-UK, V9, DOI 10.1038/s41598-019-52993-x

Ki ELL, 2019, WORLD J GASTRO ENDOS, V11, P345, DOI 10.4253/wjge.v11.i5.345
Lesmana C, 2019, CASE REP GASTROENTER, V13, P385, DOI 10.1159/000502835
Mangas-Sanjuan C., 2019, ENDOSCOPY
Mora-Soler AM, 2018, REV ESP ENFERM DIG, V110, P299, DOI 10.17235/reed.2018.5040/2017
Ogura T, 2015, WORLD J GASTROENTERO, V21, P820, DOI 10.3748/wjg.v21.i3.820
Sharaiha RZ, 2017, GASTROINTEST ENDOSC, V85, P904, DOI 10.1016/j.gie.2016.12.023
Teoh AYB, 2018, GUT, V67, P1209, DOI 10.1136/gutjnl-2017-314341
Wong YJ, 2019, ENDOSCOPY, V51, pE394, DOI 10.1055/a-0929-4927
NR 12
TC 3
Z9 3
U1 0
U2 0
PU ELSEVIER SCI LTD
PI OXFORD
PA THE BOULEVARD, LANGFORD LANE, KIDLINGTON, OXFORD OX5 1GB, OXON, ENGLAND
SN 2210-2612
J9 INT J SURG CASE REP
JI Int. J. Surg. Case Rep.
PY 2020
VL 74
BP 23
EP 26
DI 10.1016/j.ijscr.2020.07.041
PG 4
WC Surgery
WE Emerging Sources Citation Index (ESCI)
SC Surgery
GA NU8ZI
UT WOS:000573925500005
PM 32771936
OA Green Published, Green Submitted, gold
DA 2026-05-09
ER

PT J
AU Yuste, RT
Garcia-Alonso, FJ
Sánchez-Ocana, R
Cimavilla, M
Herrero, IP
Carbajo, AY

Sanz, MD
Higuera, CD
Pérez-Miranda, M
AF Torres Yuste, Raul
Javier Garcia-Alonso, Francisco
Sanchez-Ocana, Ramon
Cimavilla, Marta
Penas Herrero, Irene
Yaiza Carbajo, Ana
De Benito Sanz, Marina
De la Serna Higuera, Carlos
Perez-Miranda, Manuel
TI Safety and clinical outcomes of endoscopic ultrasound-guided gallbladder drainage with lumen-apposing metal stents in patients with dwell time over one year
SO ANNALS OF GASTROENTEROLOGY
LA English
DT Article
DE Cholecystitis; drainage; endoscopy; digestive system; stents
ID ACUTE CHOLECYSTITIS
AB Background Endoscopic ultrasound-guided gallbladder drainage (EUS-GBD) has proved effective in patients with cholecystitis at high surgical risk. The long-term risks of gallstone-related disease and stent-related adverse events are unknown.

Methods We performed a retrospective evaluation of a case series including subjects who underwent EUS-GBD using lumen-apposing metal stents (LAMS). Patients were identified from a prospective LAMS registry at a single tertiary center. Patients with a stent indwell time <1 year were excluded. Data regarding stent deployment and adverse events were retrieved from the prospective LAMS registry, while emergency room visits, admissions and causes of death were retrieved from electronic medical records.

Results We included 22 patients with a median age of 88.3 years (interquartile range [IQR]: 82.6-92.7), 14 (63.6%) were male. Median follow up was 24.4 months (IQR: 18.2-42.4) and median time to the last available imaging procedure was 607 days (IQR: 463-938). No LAMS-related adverse events were identified beyond the first year of follow up. During follow up, 12 patients (54.5%) visited the emergency room 34 times (1 visit/patient, IQR: 0-3) and a total of 36 hospital admissions were required, with a median of 1 admission/patient (IQR: 0-3). Fourteen (63.6%) patients died during follow up. Only 1 patient (4.5%) required new hospital admissions for gallstone-related disease.

Conclusions There were no adverse events beyond the first year after stent deployment, with only 4.5% of subjects requiring gallstone-related admissions. Permanent EUS-GBD with LAMS may be a definitive treatment for acute cholecystitis in patients ineligible for cholecystectomy.
C1 [Torres Yuste, Raul; Javier Garcia-Alonso, Francisco; Sanchez-Ocana, Ramon; Yaiza Carbajo, Ana; De Benito Sanz, Marina; De la Serna Higuera, Carlos; Perez-Miranda, Manuel] Hosp Univ Rio Hortega, Gastroenterol Dept, Valladolid, Spain.
[Torres Yuste, Raul] Hosp Gen Segovia, Segovia, Spain.
[Cimavilla, Marta; Penas Herrero, Irene] Hosp Rio Carrion, Palencia, Spain.

C3 Hospital del Rio Hortega
RP Yuste, RT (corresponding author), Calle Dulzaina 2, Valladolid 47012, Spain.
EM tyraul89@gmail.com
RI ; García-Alonso, Francisco Javier/GOK-0879-2022
OI Peñas Herrero, Irene/0009-0007-9986-8107; García-Alonso, Francisco
Javier/0000-0002-1425-8534
CR Bang JY, 2017, GUT, V66, P2054, DOI 10.1136/gutjnl-2016-312812
Baron TH, 2015, NEW ENGL J MED, V373, P357, DOI 10.1056/NEJMra1411372
Binmoeller KF, 2011, ENDOSCOPY, V43, P337, DOI 10.1055/s-0030-1256127
Choi JH, 2014, ENDOSCOPY, V46, P656, DOI 10.1055/s-0034-1365720
Cotton PB, 2010, GASTROINTEST ENDOSC, V71, P446, DOI 10.1016/j.gie.2009.10.027
de la Serna-Higuera C, 2013, GASTROINTEST ENDOSC, V77, P303, DOI 10.1016/j.gie.2012.09.021
Itoi T, 2008, GASTROINTEST ENDOSC, V68, P455, DOI 10.1016/j.gie.2008.02.052
Jang JW, 2012, GASTROENTEROLOGY, V142, P805, DOI 10.1053/j.gastro.2011.12.051
García-Alonso FJ, 2015, DIGEST DIS SCI, V60, P1770, DOI 10.1007/s10620-014-3497-4
Kahaleh M, 2016, DIGEST LIVER DIS, V48, P1054, DOI 10.1016/j.dld.2016.05.021
Lakhtakia S, 2017, GASTROINTEST ENDOSC, V85, P1243, DOI 10.1016/j.gie.2016.10.037
Peñas-Herrero I, 2015, J HEPATO-BIL-PAN SCI, V22, P35, DOI 10.1002/jhbp.182
Riall TS, 2010, J AM COLL SURGEONS, V210, P668, DOI 10.1016/j.jamcollsurg.2009.12.031
Rinninella E, 2015, GASTROINTEST ENDOSC, V82, P1039, DOI 10.1016/j.gie.2015.04.006
Ryan BM, 2017, GUT, V66, DOI 10.1136/gutjnl-2016-313388
Sharaiha RZ, 2016, CLIN GASTROENTEROL H, V14, P1797, DOI 10.1016/j.cgh.2016.05.011
Teoh AYB, 2017, ENDOSCOPY, V49, P130, DOI 10.1055/s-0042-119036
Tsuyuguchi T, 2013, J HEPATO-BIL-PAN SCI, V20, P81, DOI 10.1007/s00534-012-0570-2
Walter D, 2016, GUT, V65, P6, DOI 10.1136/gutjnl-2015-309925
Yamashita Y, 2013, J HEPATO-BIL-PAN SCI, V20, P89, DOI 10.1007/s00534-012-0567-x
Yokoe M, 2013, J HEPATO-BIL-PAN SCI, V20, P35, DOI 10.1007/s00534-012-0568-9
NR 21
TC 24
Z9 26
U1 0
U2 0
PU HELLENIC SOC GASTROENTEROLOGY
PI ATHENS
PA DEMOKRATIAS AVE 67, ATHENS, 15451, GREECE
SN 1108-7471
EI 1792-7463
J9 ANN GASTROENTEROL
JI Ann. Gastroenterol.
PY 2019
VL 32
IS 5

BP 514
EP 521
DI 10.20524/aog.2019.0395
PG 8
WC Gastroenterology & Hepatology
WE Emerging Sources Citation Index (ESCI)
SC Gastroenterology & Hepatology
GA IU1BT
UT WOS:000483312500013
PM 31474799
OA Green Published, gold
DA 2026-05-09
ER

PT J
AU De Angelis, CG
Venezia, L
Valdivia, PC
Rizza, S
Bruno, M
Pellicano, R
AF De Angelis, Claudio Giovanni
Venezia, Ludovica
Valdivia, Pablo Cortegoso
Rizza, Stefano
Bruno, Mauro
Pellicano, Rinaldo
TI Lumen-apposing metal stents in management of pancreatic fluid
collections: The nobody's land of removal timing
SO SAUDI JOURNAL OF GASTROENTEROLOGY
LA English
DT Review
DE EUS; EUS-guided drainage; lumen apposing metal stent; pancreatic fluid
collection; pancreatic pseudocyst; removal timing; walled-off necrosis
ID ULTRASOUND-GUIDED DRAINAGE; ENHANCED DELIVERY-SYSTEM; OUTCOMES;
EFFICACY; NECROSIS; SAFETY; LAMS
AB Pancreatic fluid collections (PFCs) develop as a result of damage to the major or peripheral pancreatic ducts, complication due to acute or chronic pancreatitis, trauma or iatrogenic causes. PFCs include pancreatic pseudocysts (PPs) and walled-off necrosis (WON). PFCs usually resolve spontaneously and are asymptomatic, but if they persist, increase in dimension or became symptomatics, therapeutic intervention is required. Available therapeutic interventions include surgical, percutaneous, and endoscopic drainage. The endoscopic approach is nowadays considered the first line-treatment of PFCs due to various advantages when compared with surgical or percutaneous drainage: decreased morbidity, length of

hospital stay, and reduced costs. In the last few years, the endoscopic ultrasound (EUS)-guided transmural drainage, initially with plastic stents, gained popularity. More recently, fully covered self-expanding lumen-apposing metal stents (LAMS) have been demonstrated to be both, safe and effective with high clinical and technical success, reducing the risk of perforation, peritoneal leakage, migration and facilitating the drainage of necrotic contents. In the last few years, several studies evaluating the safety and efficacy of LAMS and their differences with plastic stents have been performed, but literature on the removal timing of this device and associated complications is still limited. The aim of this review is to analyze studies reporting information about the retrieval timing of LAMS and the related adverse events.
C1 [De Angelis, Claudio Giovanni; Bruno, Mauro; Pellicano, Rinaldo] City Hlth & Sci, Gastroenterol Unit, Dept Med Sci, Turin, Italy.

[Venezia, Ludovica; Valdivia, Pablo Cortegoso; Rizza, Stefano] Univ Turin, Dept Med Sci, Gastroenterol Unit, City Hlth & Sci, Turin, Italy.

C3 A.O.U. Citta della Salute e della Scienza di Torino; A.O.U. Citta della
Salute e della Scienza di Torino; University of Turin

RP Venezia, L (corresponding author), AOU Citta Salute & Sci, Gastroenterol Unit, Corso Bramante 89, I-10100 Turin, Italy.

EM ludovica.venezia@gmail.com

RI Cortegoso Valdivia, Pablo/JPW-9838-2023

OI Cortegoso Valdivia, Pablo/0000-0002-8793-5890; Rizza,
Stefano/0000-0002-4019-6162; PELLICANO, RINALDO/0000-0003-3438-0649
CR Adler DG, 2017, ENDOSC ULTRASOUND, V6, P389, DOI 10.4103/eus.eus_4_17
Arvanitakis M, 2018, ENDOSCOPY, V50, P524, DOI 10.1055/a-0588-5365
Bang JY, 2017, GUT, V66, P2054, DOI 10.1136/gutjnl-2016-312812
Bekkali NLH, 2017, ENDOSC INT OPEN, V5, pE1189, DOI 10.1055/s-0043-120831
Fabbri C, 2015, GASTROINTEST ENDOSC, V82, P585, DOI 10.1016/j.gie.2015.04.035
Gornals JB, 2013, SURG ENDOSC, V27, P1428, DOI 10.1007/s00464-012-2591-y
Guo JT, 2017, ENDOSC ULTRASOUND, V6, P285, DOI 10.4103/eus.eus_85_17
Itoi T, 2012, GASTROINTEST ENDOSC, V75, P870, DOI 10.1016/j.gie.2011.10.020
KOZAREK RA, 1989, ANN SURG, V209, P261, DOI 10.1097/00000658-198903000-00002
Lang GD, 2018, GASTROINTEST ENDOSC, V87, P150, DOI 10.1016/j.gie.2017.06.029
Law ST, 2018, SURG ENDOSC, V32, P2448, DOI 10.1007/s00464-017-5946-6
Rinninella E, 2015, GASTROINTEST ENDOSC, V82, P1039, DOI 10.1016/j.gie.2015.04.006
Sahar N, 2017, ENDOSC INT OPEN, V5, pE1052, DOI 10.1055/s-0043-111794
Seerden TC, 2016, ENDOSCOPY, V48, pE179, DOI 10.1055/s-0042-107073
Shah RJ, 2015, CLIN GASTROENTEROL H, V13, P747, DOI 10.1016/j.cgh.2014.09.047
Siddiqui AA, 2016, GASTROINTEST ENDOSC, V83, P699, DOI 10.1016/j.gie.2015.10.020
Walter D, 2015, ENDOSCOPY, V47, P63, DOI 10.1055/s-0034-1378113
Yoo J, 2017, ENDOSC ULTRASOUND, V6, P131, DOI 10.4103/2303-9027.204814
Zhu HY, 2017, GASTROINTEST ENDOSC, V86, P235, DOI 10.1016/j.gie.2016.11.009
NR 19

TC 10

Z9 12

U1 0

U2 4
PU WOLTERS KLUWER MEDKNOW PUBLICATIONS
PI MUMBAI
PA WOLTERS KLUWER INDIA PVT LTD , A-202, 2ND FLR, QUBE, C T S NO 1498A-2
VILLAGE MAROL, ANDHERI EAST, MUMBAI, 400059, INDIA
SN 1319-3767
EI 1998-4049
J9 SAUDI J GASTROENTERO
JI Saudi J. Gastroenterol.
PD NOV-DEC
PY 2019
VL 25
IS 6
BP 335
EP 340
DI 10.4103/sjg.SJG_166_19
PG 6
WC Gastroenterology & Hepatology
WE Science Citation Index Expanded (SCI-EXPANDED)
SC Gastroenterology & Hepatology
GA KJ4YI
UT WOS:000512065400002
PM 31823862
OA Green Submitted, gold
DA 2026-05-09
ER

PT J
AU Bhakta, D
de Latour, R
Khanna, L
AF Bhakta, Dimpal
de Latour, Rabia
Khanna, Lauren
TI Management of pancreatic fluid collections
SO TRANSLATIONAL GASTROENTEROLOGY AND HEPATOLOGY
LA English
DT Review
DE Drainage; pancreatitis; pancreatic fluid collection; stents; walled-off
necrosis (WON)
ID APPOSING METAL STENTS; EUS-GUIDED DRAINAGE; WALLED-OFF NECROSIS; STEP-UP
APPROACH; TRANSMURAL DRAINAGE; PLASTIC STENTS; PERCUTANEOUS DRAINAGE;

PSEUDOCYST DRAINAGE; ENDOSCOPIC DRAINAGE; CLINICAL-OUTCOMES

AB Pancreatic fluid collections often develop as a complication of acute pancreatitis but can be seen in a variety of conditions including chronic pancreatitis, trauma, malignancy or post-operatively. It is important to classify a pancreatic fluid collection in order to optimize treatment strategies and management. Most interventions are targeted towards the management of delayed complications of pancreatitis, including pancreatic pseudocysts and walled-off necrosis (WON), which often develop days to weeks after the initial episode of pancreatitis. Surgical, percutaneous, and endoscopic interventions are all possible methods for treatment of pancreatic fluid collections, however endoscopic drainage with endoscopic ultrasound has become first-line. Advances within endoscopic drainage strategies have also led to innovative changes in the specific stents used for treatment, with possible options including double pigtail plastic stents, fully covered self-expanding metal stents and lumen-apposing metal stents (LAMS).

C1 [Bhakta, Dimpal; de Latour, Rabia; Khanna, Lauren] New York Univ Sch Med, New York, NY USA.

C3 New York University

RP Khanna, L (corresponding author), 240 East 38th St,Floor 23, New York, NY 10016 USA.

EM lauren.khanna@nyulangone.org

CR Aburajab M, 2018, GASTROINTEST ENDOSC, V87, P1248, DOI 10.1016/j.gie.2017.11.033

ADAMS DB, 1992, ANN SURG, V215, P571, DOI 10.1097/00000658-199206000-00003

Aghdassi Alexander A, 2006, HPB (Oxford), V8, P432, DOI 10.1080/13651820600748012

Akshintala VS, 2014, GASTROINTEST ENDOSC, V79, P921, DOI 10.1016/j.gie.2013.10.032

Arvanitakis M, 2007, GASTROINTEST ENDOSC, V65, P609, DOI 10.1016/j.gie.2006.06.083

BALTHAZAR EJ, 1990, RADIOLOGY, V174, P331, DOI 10.1148/radiology.174.2.2296641

Banks PA, 2013, GUT, V62, P102, DOI 10.1136/gutjnl-2012-302779

Baron TH, 2020, GASTROENTEROLOGY, V158, P67, DOI 10.1053/j.gastro.2019.07.064

Baron Todd H, 2003, Gastrointest Endosc Clin N Am, V13, P743, DOI 10.1016/S1052-5157(03)00100-4

Benali ZEA, 2013, PAN AFR MED J, V16, DOI 10.11604/pamj.2013.16.124.3499

Binmoeller KF, 2011, ENDOSCOPY, V43, P337, DOI 10.1055/s-0030-1256127

Boxhoorn Lotte, 2018, Curr Treat Options Gastroenterol, V16, P333, DOI [10.1007/s11938-018-0189-8, 10.1007/s11938-018-0189-8]

Brimhall B, 2018, CLIN GASTROENTEROL H, V16, P1521, DOI 10.1016/j.cgh.2018.02.021

Chen YI, 2018, ENDOSC INT OPEN, V6, pE780, DOI 10.1055/a-0611-5082

Chen YI, 2018, GASTROINTEST ENDOSC, V88, P267, DOI 10.1016/j.gie.2018.03.021

Cui ML, 2014, DIGEST DIS SCI, V59, P1055, DOI 10.1007/s10620-013-2967-4

Dawod E, 2017, AM J GASTROENTEROL, V112, pS29, DOI 10.14309/00000434-201710001-00064

DEVIERE J, 1995, GASTROINTEST ENDOSC, V42, P445, DOI 10.1016/S0016-5107(95)70048-X

Elmunzer BJ, 2018, CLIN GASTROENTEROL H, V16, P1851, DOI 10.1016/j.cgh.2018.03.021

Fabbri C, 2012, WORLD J GASTRO ENDOS, V4, P479, DOI 10.4253/wjge.v4.i11.479

Fischer TD, 2014, J AM COLL SURGEONS, V219, P704, DOI 10.1016/j.jamcollsurg.2014.03.055

Forsmark CE, 2016, NEW ENGL J MED, V375, P1972, DOI 10.1056/NEJMra1505202

Gambitta P, 2020, HEPATOB PANCREAT DIS, V19, P68, DOI 10.1016/j.hbpd.2019.09.008

GRIMM H, 1992, GASTROINTEST ENDOSC, V38, P170, DOI 10.1016/S0016-5107(92)70384-8

Habashi S, 2009, WORLD J GASTROENTERO, V15, P38, DOI 10.3748/wjg.15.38

Haller G, 2002, BRIT J ANAESTH, V88, P597, DOI 10.1093/bja/88.4.597

Heider R, 1999, ANN SURG, V229, P781, DOI 10.1097/00000658-199906000-00004

Holt BA, 2015, GASTROINTEST ENDOSC, V81, P804, DOI 10.1016/j.gie.2014.12.026
Isenmann R, 2004, GASTROENTEROLOGY, V126, P997, DOI 10.1053/j.gastro.2003.12.050
KOZAREK RA, 1985, GASTROINTEST ENDOSC, V31, P322, DOI 10.1016/S0016-5107(85)72215-8
Kumta NA, 2019, DIGEST LIVER DIS, V51, P1557, DOI 10.1016/j.dld.2019.05.033
Lakhtakia S, 2017, GASTROINTEST ENDOSC, V85, P1243, DOI 10.1016/j.gie.2016.10.037
Lakhtakia S, 2016, BEST PRACT RES CL GA, V30, P807, DOI 10.1016/j.bpg.2016.10.008
Lang GD, 2018, GASTROINTEST ENDOSC, V87, P150, DOI 10.1016/j.gie.2017.06.029
Larsen Michael, 2016, Curr Treat Options Gastroenterol, V14, P348, DOI [10.1007/s11938-016-0098-7, 10.1007/s11938-016-0098-7]
Lehman GA, 1999, GASTROINTEST ENDOSC, V49, pS81, DOI 10.1016/S0016-5107(99)70533-X
Lenhart DK, 2008, AM J ROENTGENOL, V190, P643, DOI 10.2214/AJR.07.2761
Melman L, 2009, SURG ENDOSC, V23, P267, DOI 10.1007/s00464-008-0196-2
Mussetto A, 2018, ANN GASTROENTEROL, V31, P535, DOI 10.20524/aog.2018.0287
Muthusamy VR, 2016, GASTROINTEST ENDOSC, V83, P481, DOI 10.1016/j.gie.2015.11.027
Othman MO, 2017, DIAGN THER ENDOSC, V2017
Panamonta N, 2012, EUR J GASTROEN HEPAT, V24, P1355, DOI 10.1097/MEG.0b013e32835871eb
Papachristou GI, 2007, ANN SURG, V245, P943, DOI 10.1097/01.sla.0000254366.19366.69
Park DH, 2009, ENDOSCOPY, V41, P842, DOI 10.1055/s-0029-1215133
Penn DE, 2012, GASTROINTEST ENDOSC, V76, P679, DOI 10.1016/j.gie.2012.04.457
Pitchumoni CS, 1999, GASTROENTEROL CLIN N, V28, P615, DOI 10.1016/S0889-8553(05)70077-7
Powers PC, 2019, ENDOSC ULTRASOUND, V8, P194, DOI 10.4103/eus.eus_59_18
Rana SS, 2019, ANN GASTROENTEROL, V32, P441, DOI 10.20524/aog.2019.0404
Renelus BD, 2019, THER ADV GASTROINTES, V12, DOI 10.1177/2631774519843400
Rerknimitr R, 2020, CLIN ENDOSC, V53, P49
ROGERS BHG, 1975, GASTROINTEST ENDOSC, V21, P133
Ross A, 2010, GASTROINTEST ENDOSC, V71, P79, DOI 10.1016/j.gie.2009.06.037
Ross AS, 2014, GASTROINTEST ENDOSC, V79, P929, DOI 10.1016/j.gie.2013.10.014
Sahar N, 2018, J GASTROEN HEPATOL, V33, P1548, DOI 10.1111/jgh.14111
Sandrasegaran K, 2007, RADIOGRAPHICS, V27, DOI 10.1148/rg.275065163
Shah RJ, 2015, CLIN GASTROENTEROL H, V13, P747, DOI 10.1016/j.cgh.2014.09.047
Sharaiha RZ, 2016, CLIN GASTROENTEROL H, V14, P1797, DOI 10.1016/j.cgh.2016.05.011
Sharaiha RZ, 2015, GASTROINTEST ENDOSC, V82, P822, DOI 10.1016/j.gie.2015.02.035
Siddiqui AA, 2017, GASTROINTEST ENDOSC, V85, P758, DOI 10.1016/j.gie.2016.08.014
Siddiqui AA, 2016, GASTROINTEST ENDOSC, V83, P699, DOI 10.1016/j.gie.2015.10.020
Siddiqui AA, 2014, DIGEST DIS SCI, V59, P687, DOI 10.1007/s10620-013-2945-x
Siddiqui AA, 2013, GASTROINTEST ENDOSC, V78, P589, DOI 10.1016/j.gie.2013.03.1337
Tenner S, 2013, AM J GASTROENTEROL, V108, P1400, DOI 10.1038/ajg.2013.218
Tyberg A, 2016, WORLD J GASTROENTERO, V22, P2256, DOI 10.3748/wjg.v22.i7.2256
van Santvoort HC, 2011, GASTROENTEROLOGY, V141, P1254, DOI 10.1053/j.gastro.2011.06.073
van Santvoort HC, 2010, NEW ENGL J MED, V362, P1491, DOI 10.1056/NEJMoa0908821
Varadarajulu S, 2008, GASTROINTEST ENDOSC, V68, P1102, DOI 10.1016/j.gie.2008.04.028
Varadarajulu S, 2013, GASTROENTEROLOGY, V145, P583, DOI 10.1053/j.gastro.2013.05.046

Varadarajulu S, 2011, J GASTROINTEST SURG, V15, P2080, DOI 10.1007/s11605-011-1621-8
Varadarajulu S, 2011, J GASTROEN HEPATOL, V26, P1504, DOI 10.1111/j.1440-1746.2011.06771.x
Varadarajulu S, 2011, GASTROINTEST ENDOSC, V74, P74, DOI 10.1016/j.gie.2011.03.1122
Vilmann P, 1998, ENDOSCOPY, V30, P730, DOI 10.1055/s-2007-1001399
Wiersema MJ, 1996, GASTROINTEST ENDOSC, V44, P614, DOI 10.1016/S0016-5107(96)70022-6
Yan L, 2019, ENDOSC ULTRASOUND, V8, P172, DOI 10.4103/eus.eus_108_17
YEO CJ, 1990, SURG GYNECOL OBSTET, V170, P411
Yoon SB, 2018, UNITED EUR GASTROENT, V6, P729, DOI 10.1177/2050640618761702
NR 76
TC 14
Z9 24
U1 0
U2 11
PU AME PUBL CO
PI SHATIN
PA FLAT-RM C 16F, KINGS WING PLAZA 1, NO 3 KWAN ST, SHATIN, HONG KONG
00000, PEOPLES R CHINA
EI 2415-1289
J9 TRANSL GASTROENT HEP
JI Transl. Gastroenterol. Hepatol.
PD APR
PY 2022
VL 7
DI 10.21037/tgh-2020-06
EA FEB 2021
PG 15
WC Gastroenterology & Hepatology
WE Emerging Sources Citation Index (ESCI)
SC Gastroenterology & Hepatology
GA 0Z5NX
UT WOS:000675469700001
PM 35548474
OA Green Submitted, gold
DA 2026-05-09
ER

PT J
AU Minaga, K
Takenaka, M
Kamata, K
Yoshikawa, T
Nakai, A

Omoto, S
Miyata, T
Yamao, K
Imai, H
Sakamoto, H
Kitano, M
Kudo, M
AF Minaga, Kosuke
Takenaka, Mamoru
Kamata, Ken
Yoshikawa, Tomoe
Nakai, Atsushi
Omoto, Shunsuke
Miyata, Takeshi
Yamao, Kentaro
Imai, Hajime
Sakamoto, Hiroki
Kitano, Masayuki
Kudo, Masatoshi
TI Alleviating Pancreatic Cancer-Associated Pain Using Endoscopic
Ultrasound-Guided Neurolysis
SO CANCERS
LA English
DT Review
DE endoscopic ultrasound; EUS; EUS-guided neurolysis; neurolysis;
interventional EUS; pancreatic cancer; pain
ID CELIAC PLEXUS NEUROLYSIS; SPINAL-CORD INFARCTION; GANGLIA NEUROLYSIS;
INITIAL EVALUATION; EUS; BLOCK; COMPLICATION; MANAGEMENT; NEEDLE;
ULTRASONOGRAPHY
AB The most common symptom in patients with advanced pancreatic cancer is abdominal pain. This has traditionally been treated with nonsteroidal anti-inflammatory drugs and opioid analgesics. However, these treatments result in inadequate pain control or drug-related adverse effects in some patients. An alternative pain-relief modality is celiac plexus neurolysis, in which the celiac plexus is chemically ablated. This procedure was performed percutaneously or intraoperatively until 1996, when endoscopic ultrasound (EUS)-guided celiac plexus neurolysis was first described. In this transgastric anterior approach, a neurolytic agent is injected around the celiac trunk under EUS guidance. The procedure gained popularity as a minimally invasive approach and is currently widely used to treat pancreatic cancer-associated pain. We focus on two relatively new techniques of EUS-guided neurolysis: EUS-guided celiac ganglia neurolysis and EUS-guided broad plexus neurolysis, which have been developed to improve efficacy. Although the techniques are safe and effective in general, some serious adverse events including ischemic and infectious complications have been reported as the procedure has gained widespread popularity. We summarize reported clinical outcomes of EUS-guided neurolysis in pancreatic cancer (from the PubMed and Embase databases) with a goal of providing information useful in developing strategies for pancreatic cancer-associated pain alleviation.

C1 [Minaga, Kosuke; Takenaka, Mamoru; Kamata, Ken; Yoshikawa, Tomoe; Nakai, Atsushi; Omoto, Shunsuke; Miyata, Takeshi; Yamao, Kentaro; Imai, Hajime; Kudo, Masatoshi] Kindai Univ, Fac Med, Dept Gastroenterol & Hepatol, Osaka 5898511, Japan.
[Sakamoto, Hiroki] Katsuragi Hosp, Dept Gastroenterol, Kishiwada 5960825, Japan.
[Kitano, Masayuki] Wakayama Med Univ, Dept Internal Med 2, Wakayama 6418509, Japan.
C3 Kindai University (Kinki University); Wakayama Medical University
RP Minaga, K (corresponding author), Kindai Univ, Fac Med, Dept Gastroenterol & Hepatol, Osaka 5898511, Japan.
EM kousukeminaga@yahoo.co.jp; mamoxyo45@gmail.com; ky11@leto.eonet.ne.jp;
t.yoshikawa113@gmail.com; nakai_agmc@yahoo.co.jp;
shunsuke.oomoto@gmail.com; miyatchi77@yahoo.co.jp;
yamaken_volvo@yahoo.co.jp; codenamegenchan1023@gmail.com;
hiroki.sakamoto@nifty.com; kitano@wakayama-med.ac.jp;
m-kudo@med.kindai.ac.jp
RI ; Minaga, kosuke/AAD-2107-2020; Kudo, Masatoshi/AAA-9744-2019
OI Yamao, Kentaro/0000-0003-2497-7740; Minaga, kosuke/0000-0001-5407-7925;
FU Japan Society for the Promotion of Science
FX This study was supported by grants from the Japan Society for the
Promotion of Science.
CR Adler DG, 2007, GASTROINTEST ENDOSC, V66, P1076, DOI 10.1016/j.gie.2007.07.035
Ahmed HM, 2009, ENDOSCOPY, V41, pE218, DOI 10.1055/s-0029-1214941
Alvarez-Sanchez MV, 2014, SURG ENDOSC, V28, P712, DOI 10.1007/s00464-013-3260-5
[Anonymous], 2017, GUID PANCR AD VERS 3
Arcidiacono PG, 2011, COCHRANE DB SYST REV, DOI 10.1002/14651858.CD007519.pub2
Ascunce G, 2011, GASTROINTEST ENDOSC, V73, P267, DOI 10.1016/j.gie.2010.10.029
Bang JY, 2016, GASTROINTEST ENDOSC, V84, P773, DOI 10.1016/j.gie.2016.03.1496
Barreto SG, 2012, PANCREATOLOGY, V12, P104, DOI 10.1016/j.pan.2012.02.010
Bhutani MS, 1998, ENDOSCOPY, V30, P605, DOI 10.1055/s-2007-1001364
Bilimoria KY, 2007, CANCER-AM CANCER SOC, V110, P738, DOI 10.1002/cncr.22852
Caraceni A, 1996, CANCER-AM CANCER SOC, V78, P639
Doi S, 2013, ENDOSCOPY, V45, P362, DOI 10.1055/s-0032-1326225
Eloubeidi MA, 2011, J GASTROEN HEPATOL, V26, P63, DOI 10.1111/j.1440-1746.2010.06409.x
Facciorusso A, 2017, J GASTROEN HEPATOL, V32, P439, DOI 10.1111/jgh.13478
Facciorusso A, 2015, ENDOSC ULTRASOUND, V4, P342, DOI 10.4103/2303-9027.170428
Fujii L, 2012, ENDOSCOPY, V44, pE265, DOI 10.1055/s-0032-1309708
Fujii-Lau LL, 2015, GASTROINTEST ENDOSC, V82, P46, DOI 10.1016/j.gie.2014.12.036
Gimeno-García AZ, 2012, ENDOSCOPY, V44, pE267, DOI 10.1055/s-0032-1309709
Gleeson FC, 2007, ENDOSCOPY, V39, P620, DOI 10.1055/s-2007-966337
Gohil Vishal B, 2017, Curr Treat Options Gastroenterol, V15, P333, DOI [10.1007/s11938-017-0145-z, 10.1007/s11938-017-0145-z]
Gunaratnam NT, 2001, GASTROINTEST ENDOSC, V54, P316, DOI 10.1067/mge.2001.117515
Ha Tae-In, 2008, Korean Journal of Internal Medicine, V23, P5, DOI 10.3904/kjim.2008.23.1.5
Hao SJ, 2014, SURG LAPARO ENDO PER, V24, P31, DOI 10.1097/SLE.0000000000000032

Ishiwatari H, 2015, GASTROINTEST ENDOSC, V81, P479, DOI 10.1016/j.gie.2014.10.031
Ishiwatari H, 2014, WORLD J GASTROENTERO, V20, P10512, DOI 10.3748/wjg.v20.i30.10512
Iwata K, 2011, DIGEST ENDOSC, V23, P140, DOI 10.1111/j.1443-1661.2010.01046.x
Jang HY, 2013, CLIN ENDOSC, V46, P306, DOI 10.5946/ce.2013.46.3.306
Kappelle WFW, 2017, GASTROINTEST ENDOSC, V86, P655, DOI 10.1016/j.gie.2017.01.041
Kaufman M, 2010, J CLIN GASTROENTEROL, V44, P127, DOI 10.1097/MCG.0b013e3181bb854d
Kida M, 2011, ENDOSCOPY, V43, P796, DOI 10.1055/s-0030-1256508
Kitoh Takeshi, 2005, J Anesth, V19, P328, DOI 10.1007/s00540-005-0342-0
Köker IH, 2017, SCAND J GASTROENTERO, V52, P1158, DOI 10.1080/00365521.2017.1335771
Leblanc Julia K, 2013, Diagn Ther Endosc, V2013, P327036, DOI 10.1155/2013/327036
LeBlanc JK, 2011, GASTROINTEST ENDOSC, V74, P1300, DOI 10.1016/j.gie.2011.07.073
Levy MJ, 2008, AM J GASTROENTEROL, V103, P98, DOI 10.1111/j.1572-0241.2007.01607.x
Levy MJ, 2003, GASTROINTEST ENDOSC, V57, P923, DOI 10.1016/S0016-5107(03)70036-4
Lu F, 2018, SUPPORT CARE CANCER, V26, P353, DOI 10.1007/s00520-017-3888-0
Mekaroonkamol P, 2015, J PANCREAS, V16, P33, DOI 10.6092/1590-8577/2890
Michaels AJ, 2007, WORLD J GASTROENTERO, V13, P3575, DOI 10.3748/wjg.v13.i26.3575
Minaga K, 2016, THER ADV GASTROENTER, V9, P483, DOI 10.1177/1756283X16644248
Minaga K, 2016, GASTROINTEST ENDOSC, V83, P1039, DOI 10.1016/j.gie.2015.10.044
Mittal MK, 2012, NEUROLOGY, V78, pE57, DOI 10.1212/WNL.0b013e318248df51
Mulhall AM, 2016, ANN AM THORAC SOC, V13, P1660, DOI 10.1513/AnnalsATS.201603-190LE
Muscatiello N, 2006, ENDOSCOPY, V38, P858, DOI 10.1055/s-2006-925382
Nagels W, 2013, PAIN MED, V14, P1140, DOI 10.1111/pme.12176
O'Toole TM, 2009, ENDOSCOPY, V41, P593, DOI 10.1055/s-0029-1214868
Obstein KL, 2010, WORLD J GASTROENTERO, V16, P728, DOI 10.3748/wjg.v16.i6.728
Penman ID, 2009, GASTROINTEST ENDOSC, V69, pS174, DOI 10.1016/j.gie.2008.12.023
Puli SR, 2009, DIGEST DIS SCI, V54, P2330, DOI 10.1007/s10620-008-0651-x
Ramirez-Luna M A, 2008, Rev Gastroenterol Mex, V73, P63
Sahai AV, 2009, AM J GASTROENTEROL, V104, P326, DOI 10.1038/ajg.2008.64
Sakamoto H., 2006, DIGEST ENDOSC, V18, P206
Sakamoto H, 2010, AM J GASTROENTEROL, V105, P2599, DOI 10.1038/ajg.2010.339
Seicean A, 2013, J GASTROINTEST LIVER, V22, P59
Sirri E, 2016, PANCREAS, V45, P908, DOI 10.1097/MPA.0000000000000588
Soetikno RM, 2002, GASTROINTEST ENDOSC, V56, P136, DOI 10.1067/mge.2002.125109
Soweid AM, 2010, WORLD J GASTRO ENDOS, V2, P228, DOI 10.4253/wjge.v2.i6.228
Téllez-Avila FI, 2013, ENDOSC ULTRASOUND, V2, P153, DOI [10.4103/2303-9027.117664, 10.7178/eus.06.007]
Tran Quy N H, 2006, J Support Oncol, V4, P460
Varadarajulu S, 2012, GASTROINTEST ENDOSC, V76, P336, DOI 10.1016/j.gie.2012.04.455
Wang KX, 2012, GASTROINTEST ENDOSC, V76, P945, DOI 10.1016/j.gie.2012.05.032
Wiechowska-Kozlowska A, 2012, GASTROENT RES PRACT, V2012, DOI 10.1155/2012/503098
Wiersema MJ, 1996, GASTROINTEST ENDOSC, V44, P656, DOI 10.1016/S0016-5107(96)70047-0
World Health Organization, 2006, CANC PAIN REL
Wyse JM, 2011, J CLIN ONCOL, V29, P3541, DOI 10.1200/JCO.2010.32.2750

Yan BM, 2007, AM J GASTROENTEROL, V102, P430, DOI 10.1111/j.1572-0241.2006.00967.x
Yasuda I, 2017, DIGEST ENDOSC, V29, P455, DOI 10.1111/den.12824
Zou XP, 2012, PANCREAS, V41, P655, DOI 10.1097/MPA.0b013e31823d06a9
NR 68
TC 17
Z9 24
U1 1
U2 11
PU MDPI
PI BASEL
PA ST ALBAN-ANLAGE 66, CH-4052 BASEL, SWITZERLAND
SN 2072-6694
J9 CANCERS
JI Cancers
PD FEB
PY 2018
VL 10
IS 2
AR 50
DI 10.3390/cancers10020050
PG 17
WC Oncology
WE Science Citation Index Expanded (SCI-EXPANDED)
SC Oncology
GA GA1OG
UT WOS:000428085900022
PM 29462851
OA Green Submitted, gold
DA 2026-05-09
ER

PT J
AU Libânio, D
Giestas, S
Martinez-Ares, D
Ferreira, F
Canena, J
Certo, M
Lopes, L
AF Libanio, Diogo
Giestas, Silvia
Martinez-Ares, David

Ferreira, Frederico
Canena, Jorge
Certo, Manuela
Lopes, Luis
TI Endoscopic Ultrasound-Guided Biliary Drainage in Two Patients with Difficult Biliary Access
SO GE PORTUGUESE JOURNAL OF GASTROENTEROLOGY
LA English
DT Article
DE Endoscopic retrograde cholangiopancreatography; Biliary drainage; Endoscopic ultrasonography; Palliative therapy; Gastrointestinal cancer
AB Introduction: Endoscopic retrograde cholangiopancreatography is the method of choice for biliary drainage, although in some cases standard biliary access is difficult or even impossible. Endoscopic ultrasound (EUS)-guided endoluminal procedures are an alternative in these cases, although experience with these techniques is still limited. Clinical Case: We present two cases of successful EUS-guided biliary drainage. In the first case, a hepaticogastrostomy was performed in a patient with stage IV gastric adenocarcinoma with obstructive jaundice due to compression of the hilum, where malignant gastric stenosis and previous palliative gastrojejunostomy precluded access to the second part of the duodenum. In the second case, a patient with a pancreatic head adenocarcinoma with duodenal invasion that precluded major papillae identification was submitted to a choledochoduodenostomy. Both procedures occurred without immediate or delayed adverse events, with technical and clinical success. Discussion: Although experience with EUS-guided biliary drainage is still limited, its efficacy and safety is favorable when compared with percutaneous and surgical drainage, and should be considered an alternative to these techniques where sufficient expertise exists. (c) 2017 Sociedade Portuguesa de Gastrenterologia Published by S. Karger AG, Basel
C1 [Libanio, Diogo] Portuguese Oncol Inst Porto, Gastroenterol Dept, Rua Dr Antonio Bernardino Almeida, PT-4200072 Porto, Portugal.
[Libanio, Diogo; Giestas, Silvia; Martinez-Ares, David; Ferreira, Frederico; Lopes, Luis] Unidade Local Satide Alto Minho, Gastroenterol Dept, Viana Do Castelo, Portugal.
[Canena, Jorge] Univ Ctr Gastroenterol, CUF Infante Santo Hosp, Fac Ciencias Med, Nova Med Sch, Lisbon, Portugal.
[Certo, Manuela] Ctr Hosp Porto, Radiol Dept, Porto, Portugal.
[Lopes, Luis] Univ Minho, Sch Med, Braga, Portugal.
C3 Portuguese Institute of Oncology; Universidade Nova de Lisboa; Universidade do Minho
RP Libânio, D (corresponding author), Portuguese Oncol Inst Porto, Gastroenterol Dept, Rua Dr Antonio Bernardino Almeida, PT-4200072 Porto, Portugal.
EM diogolibaniomonteiro@gmail.com
RI Ferreira, Frederico/KIC-9514-2024; Libânio, Diogo/AAN-7077-2020; canena, Jorge/I-1379-2015; Lopes, Luís/D-6238-2016
OI Libânio, Diogo/0000-0003-2691-7522; Certo, Manuela/0000-0002-8447-5024;
CR De Cassan C, 2017, ENDOSC ULTRASOUND, V6, P329, DOI 10.4103/2303-9027.209869
Dhir V, 2017, DIGEST ENDOSC, V29, P472, DOI 10.1111/den.12818
Kawakubo K, 2016, ENDOSCOPY, V48, P164, DOI 10.1055/s-0034-1393179
Kawakubo K, 2014, J HEPATO-BIL-PAN SCI, V21, P328, DOI 10.1002/jhbp.27

Khashab MA, 2016, ENDOSC INT OPEN, V4, pE1322, DOI 10.1055/s-0042-110790
Khashab MA, 2016, ENDOSC INT OPEN, V4, pE175, DOI 10.1055/s-0041-109083
Liao WC, 2017, GASTROINTEST ENDOSC, V85, P295, DOI 10.1016/j.gie.2016.09.037
Mandai K, 2016, ENDOSC INT OPEN, V4, pE287, DOI 10.1055/s-0041-111502
Ogura T, 2016, ENDOSCOPY, V48, P156, DOI 10.1055/s-0034-1392859
Ogura T, 2015, J GASTROEN HEPATOL, V30, P1748, DOI 10.1111/jgh.13021
Sharaiha RZ, 2017, GASTROINTEST ENDOSC, V85, P904, DOI 10.1016/j.gie.2016.12.023
NR 11
TC 0
Z9 0
U1 0
U2 0
PU KARGER
PI BASEL
PA ALLSCHWILERSTRASSE 10, CH-4009 BASEL, SWITZERLAND
SN 2341-4545
EI 2387-1954
J9 GE PORT J GASTROENT
JI GE Port. J. Gastroenterol.
PY 2018
VL 25
IS 5
BP 258
EP 263
DI 10.1159/000485429
PG 6
WC Gastroenterology & Hepatology
WE Emerging Sources Citation Index (ESCI)
SC Gastroenterology & Hepatology
GA VJ2ZL
UT WOS:000573269200008
PM 30320165
OA Green Submitted, gold
DA 2026-05-09
ER

PT J
AU Prachayakul, V
Aswakul, P
AF Prachayakul, Varayu
Aswakul, Pitulak
TI A novel technique for endoscopic ultrasound-guided biliary drainage

SO WORLD JOURNAL OF GASTROENTEROLOGY
LA English
DT Article
DE Endoscopic ultrasound; Biliary drainage; Hepaticogastrostomy;
Choledochoduodenostomy; Endoscopic ultrasound-guided
ID METAL STENT; FAILED ERCP; CHOLEDOCHODUODENOSTOMY; PLACEMENT
AB AIM: To describe a successful endoscopic ultrasound (EUS)-guided biliary drainage technique with high success and low complication rates.

METHODS: The recorded data of consecutive patients who presented to Siriraj Gastrointestinal Endoscopy Center, Siriraj Hospital in Bangkok, Thailand for treatment of malignant obstructive jaundice but failed endoscopic retrograde cholangiopancreatography and underwent subsequent EUS-guided biliary drainage were retrospectively reviewed. The patients' baseline characteristics, clinical manifestations, procedure details, and post-procedure follow-up data were recorded and analyzed. Clinical outcomes were assessed by physical exam and standard laboratory tests. Technical success of the procedure was defined as completion of the stent insertion. Clinical success was defined as improvement of the patient's overall clinical manifestations, in terms of general well-being evidenced by physical examination, restoration of normal appetite, and adequate biliary drainage. Overall median survival time was calculated as the time from the procedure until the time of death, and survival analysis was performed by the Kaplan-Meier method. The Student's t-test and the chi(2) test were used to assess the significance of inter-group differences.

RESULTS: A total of 21 cases were enrolled, a single endoscopist performed all the procedures. The mean age was 62.8 years (range: 46-84 years). The sex distribution was almost equal, including 11 women and 10 men. Patients with failed papillary cannulation (33.3%), duodenal obstruction (42.9%), failed selective cannulation (19.0%), and surgical altered anatomy (4.8%) were considered candidates for EUS-guided biliary drainage. Six patients underwent EUS-guided choledochoduodenostomy and 15 underwent EUS-guided hepaticogastrostomy. The technique using non-cauterization and no balloon dilation was performed for all cases, employing the in-house manufactured tapered tip Teflon catheter to achieve the dilation. The technical success and clinical success rates of this technique were 95.2% and 90.5%, respectively. Complications included bile leakage and pneumoperitoneum, occurred at a rate of 9.5%. None of the patients died from the procedure. One patient presented with a biloma, a major complication that was successfully treated by another endoscopic procedure.

CONCLUSION: We present a highly effective EUS-guided biliary drainage technique that does not require cauterization or balloon dilation. (C) 2013 Baishideng. All rights reserved.
C1 [Prachayakul, Varayu] Mahidol Univ, Siriraj Hosp, Fac Med, Dept Internal Med,Div Gastroenterol, Bangkok 10700, Thailand.

[Aswakul, Pitulak] Samitivej Sukhumvit Hosp, Liver & Digest Inst, Bangkok 10110, Thailand.
C3 Mahidol University
RP Prachayakul, V (corresponding author), Mahidol Univ, Siriraj Hosp, Fac Med, Dept Internal Med,Div Gastroenterol, 2 Prannok Rd, Bangkok 10700, Thailand.
EM kaiyjr@gmail.com
CR Ahn JY, 2010, GUT LIVER, V4, P524, DOI 10.5009/gnl.2010.4.4.524
Artifon E L A, 2011, Rev Gastroenterol Mex, V76, P270
Attasaranya S, 2012, GASTROENT RES PRACT, V2012, DOI 10.1155/2012/680753
Belletrutti Paul J, 2011, J Gastrointest Cancer, V42, P137, DOI 10.1007/s12029-010-9175-7
Burmester E, 2003, GASTROINTEST ENDOSC, V57, P246, DOI 10.1067/mge.2003.85

Súbtil JC, 2010, WORLD J GASTRO ENDOS, V2, P203, DOI 10.4253/wjge.v2.i6.203
Chavalitdhamrong D, 2012, WORLD J GASTROENTERO, V18, P491, DOI 10.3748/wjg.v18.i6.491
Fabbri C, 2011, ENDOSCOPY, V43, P438, DOI 10.1055/s-0030-1256097
Giovannini M, 2012, GASTROENT RES PRACT, V2012, DOI 10.1155/2012/348719
Hara K, 2011, AM J GASTROENTEROL, V106, P1239, DOI 10.1038/ajg.2011.84
Irisawa A, 2009, J HEPATO-BILIARY-PAN, V16, P598, DOI 10.1007/s00534-009-0131-5
Ito K, 2010, DIAGN THER ENDOSC, V2010, DOI 1.1155/01/426534
Itoi T, 2010, J HEPATO-BIL-PAN SCI, V17, P611, DOI 10.1007/s00534-009-0196-1
Iwashita T, 2012, ENDOSCOPY, V44, P60, DOI 10.1055/s-0030-1256871
Kim TH, 2012, WORLD J GASTROENTERO, V18, P2526, DOI 10.3748/wjg.v18.i20.2526
Lee KH, 2011, CLIN ENDOSC, V44, P6, DOI 10.5946/ce.2011.44.1.6
Levy Michael J, 2010, Curr Gastroenterol Rep, V12, P141, DOI 10.1007/s11894-010-0090-7
Nguyen-Tang T, 2010, ENDOSCOPY, V42, P232, DOI 10.1055/s-0029-1243858
Park DH, 2011, GASTROINTEST ENDOSC, V74, P1276, DOI 10.1016/j.gie.2011.07.054
Ramírez-Luna MA, 2011, ENDOSCOPY, V43, P826, DOI 10.1055/s-0030-1256406
Shah JN, 2012, GASTROINTEST ENDOSC, V75, P56, DOI 10.1016/j.gie.2011.08.032
Shami VM, 2008, GASTROINTEST ENDOSC, V67, P136, DOI 10.1016/j.gie.2007.07.040
Shami VM, 2010, DIGEST LIVER DIS, V42, P419, DOI 10.1016/j.dld.2009.09.009
Siddiqui AA, 2011, SURG ENDOSC, V25, P549, DOI 10.1007/s00464-010-1216-6
Sreenarasimhaiah J, 2009, AM J MED SCI, V338, P319, DOI 10.1097/MAJ.0b013e3181a44405
Tarantino I, 2012, WORLD J GASTRO ENDOS, V4, P306, DOI 10.4253/wjge.v4.i7.306
Tarantino I, 2009, WORLD J GASTRO ENDOS, V1, P39, DOI 10.4253/wjge.v1.i1.39
Wiersema MJ, 1996, GASTROINTEST ENDOSC, V43, P102, DOI 10.1016/S0016-5107(06)80108-2
Yamao K, 2008, ENDOSCOPY, V40, P340, DOI 10.1055/s-2007-995485
Yamao K, 2010, GUT LIVER, V4, pS67, DOI 10.5009/gnl.2010.4.S1.S67
NR 30
TC 30
Z9 31
U1 0
U2 0
PU BAISHIDENG PUBLISHING GROUP INC
PI PLEASANTON
PA 8226 REGENCY DR, PLEASANTON, CA 94588 USA
SN 1007-9327
EI 2219-2840
J9 WORLD J GASTROENTERO
JI World J. Gastroenterol.
PD AUG 7
PY 2013
VL 19
IS 29
BP 4758

EP 4763
DI 10.3748/wjg.v19.i29.4758
PG 6
WC Gastroenterology & Hepatology
WE Science Citation Index Expanded (SCI-EXPANDED)
SC Gastroenterology & Hepatology
GA 194OE
UT WOS:000322641800014
PM 23922474
OA hybrid
DA 2026-05-09
ER

PT J
AU Kastelijn, JB
Van der Voort, V
Bijlsma, A
Moons, LMG
Schwartz, MP
Vleggaar, FP
AF Kastelijn, Janine B.
Van der Voort, Veronique
Bijlsma, Alderina
Moons, Leon M. G.
Schwartz, Matthijs P.
Vleggaar, Frank P.
TI Early Lumen-Apposing Metal Stent Dysfunction Complicating Endoscopic
Ultrasound-Guided Gastroenterostomy: A Report of Two Cases
SO CLINICAL ENDOSCOPY
LA English
DT Article
DE Endoscopy; Gastric outlet obstruction; Gastroenterostomy; Palliative
care; Stents
ID DRAINAGE
AB Endoscopic ultrusonography-guided gastroenterostomy using a lumen-apposing metal steal has emerged as a novel technique in the palliative treatment of malignant gastric outlet obstruction. Endoscopic ultrasonography-guided gastroenterostomy seems to have the potential to provide long-lasting patency in a minimally invasive manner. Low reintervention rates have been described. We report two cases with early lumen-apposing metal stent dysfunction, compromising patency. One case showed food impaction after three weeks, and hyperplastic tissue overgrowth with a buried distal flange six weeks after stent placement. The latter was successfully treated by argon plasma coagulation, stent removal, and deployment of a larger-diameter lumen-apposing metal stent. The second case showed a narrowed luminal diameter of the stent and jejunal pressure ulcerations after three weeks. The narrowing was successfully treated by balloon

dilation. Eight weeks later, hyperplastic tissue overgrowth at the distal flange of the stem and a gastro-colonic fistula were diagnosed, followed by extensive reconstructive surgery.

C1 [Kastelijn, Janine B.; Moons, Leon M. G.; Vleggaar, Frank P.] Univ Med Ctr Utrecht, Dept Gastroenterol & Hepatol, NL-3508 GA Utrecht, Netherlands.
[Van der Voort, Veronique; Schwartz, Matthijs P.] Meander Med Ctr, Amersfoort, Netherlands.
[Bijlsma, Alderina] Martini Hosp, Groningen, Netherlands.
C3 Utrecht University; Utrecht University Medical Center; Meander Medisch
Centrum; Martini Hospital
RP Vleggaar, FP (corresponding author), Univ Med Ctr Utrecht, Dept Gastroenterol & Hepatol, NL-3508 GA Utrecht, Netherlands.
EM f.vleggaar@umcutrecht.nl
OI Schwartz, Matthijs/0000-0003-3106-3373; Van der Voort,
Veronique/0000-0003-3163-5941; Bijlsma, Rina/0000-0002-8512-9174
CR Bang JY, 2020, GUT, V69, P1379, DOI 10.1136/gutjnl-2019-320539
Bang JY, 2017, GUT, V66, P2054, DOI 10.1136/gutjnl-2016-312812
Chen YI, 2018, GASTROINTEST ENDOSC, V87, P1215, DOI 10.1016/j.gie.2017.07.030
Cyrany J, 2016, WORLD J GASTROENTERO, V22, P618, DOI 10.3748/wjg.v22.i2.618
Dioscoridi L, 2020, ENDOSCOPY, V52, pE134, DOI 10.1055/a-1025-1919
Ge PS, 2019, SURG ENDOSC, V33, P3404, DOI 10.1007/s00464-018-06636-3
Iqbal U, 2020, ENDOSC ULTRASOUND, V9, P16, DOI 10.4103/eus.eus_70_19
Irani S, 2017, GASTROINTEST ENDOSC, V85, P1285, DOI 10.1016/j.gie.2016.08.028
Kerdsirichairat T, 2019, ENDOSC INT OPEN, V7, pE144, DOI 10.1055/a-0799-9939
Madanat L, 2018, ENDOSCOPY, V50, pE331, DOI 10.1055/a-0640-2630
Parekh PJ, 2018, ENDOSC INT OPEN, V6, pE1390, DOI 10.1055/a-0658-1211
Taibi A, 2020, DIGEST ENDOSC, V32, P816, DOI 10.1111/den.13645
Tyberg A, 2016, ENDOSC INT OPEN, V4, pE276, DOI 10.1055/s-0042-101789
Upchurch E, 2018, COCHRANE DB SYST REV, DOI 10.1002/14651858.CD012506.pub2
NR 14
TC 2
Z9 2
U1 0
U2 0
PU KOREAN SOC GASTROINTESTINAL ENDOSCOPY
PI SEOUL
PA 2003 LG PALACE, 165-8 DONGGYO-DONG, MAPO-GU, SEOUL, 121-754, SOUTH KOREA
SN 2234-2400
EI 2234-2443
J9 CLIN ENDOSC
JI Clin. Endosc.
PD JUL
PY 2021
VL 54

IS 4
BP 603
EP 607
DI 10.5946/ce.2020.201
PG 5
WC Gastroenterology & Hepatology
WE Emerging Sources Citation Index (ESCI)
SC Gastroenterology & Hepatology
GA TU2CY
UT WOS:000680848600022
PM 33434963
OA Green Submitted, gold
DA 2026-05-09
ER

PT J
AU Giovannini, M
Binmoeller, K
Seifert, H
AF Giovannini, M
Binmoeller, K
Seifert, H
TI Endoscopic ultrasound-guided cystogastrostomy
SO ENDOSCOPY
LA English
DT Article
ID CHANNEL ECHO ENDOSCOPE; PANCREATIC PSEUDOCYSTS; TRANSMURAL DRAINAGE; ENDOSONOGRAPHY; CYSTODUODENOSTOMY; LESIONS; CYSTS
C1 Inst J Paoli I Calmettes, Oncol Unit 1, F-13273 Marseille 9, France.
Inst J Paoli I Calmettes, Endoscop Unit, F-13273 Marseille, France.
Calif Pacific Med Ctr, Intervent Endoscopy Serv, San Francisco, CA USA.
Univ Frankfurt, Zentrum Inneren Med, Med Klin 2, D-6000 Frankfurt, Germany.
C3 UNICANCER; Institut Paoli-Calmette (IPC); UNICANCER; Institut Paoli-Calmette (IPC); California Pacific Medical Center; Goethe University Frankfurt
RP Giovannini, M (corresponding author), Inst J Paoli I Calmettes, Oncol Unit 1, 232 Bd St Marguerite, F-13273 Marseille 9, France.
CR Binmoeller K F, 1995, Gastrointest Endosc Clin N Am, V5, P805
BINMOELLER KF, 1994, GASTROINTEST ENDOSC, V40, P112, DOI 10.1016/S0016-5107(94)70031-1
CREMER M, 1989, GASTROINTEST ENDOSC, V35, P1, DOI 10.1016/S0016-5107(89)72677-8
Gerolami R, 1997, ENDOSCOPY, V29, P106, DOI 10.1055/s-2007-1004083
Giovannini M, 1998, GASTROINTEST ENDOSC, V48, P200, DOI 10.1016/S0016-5107(98)70165-8

Giovannini M, 2001, ENDOSCOPY, V33, P473, DOI 10.1055/s-2001-14967
GRIMM H, 1992, GASTROINTEST ENDOSC, V38, P170, DOI 10.1016/S0016-5107(92)70384-8
SAHEL J, 1987, PANCREAS, V2, P447, DOI 10.1097/00006676-198707000-00012
Seifert H, 2000, ENDOSCOPY, V32, P255, DOI 10.1055/s-2000-93
Seifert H, 2001, ENDOSCOPY, V33, P1022, DOI 10.1055/s-2001-18927
Wiersema MJ, 1996, GASTROINTEST ENDOSC, V44, P614, DOI 10.1016/S0016-5107(96)70022-6
NR 11
TC 40
Z9 41
U1 0
U2 0
PU GEORG THIEME VERLAG KG
PI STUTTGART
PA RUDIGERSTR 14, D-70469 STUTTGART, GERMANY
SN 0013-726X
J9 ENDOSCOPY
JI Endoscopy
PD MAR
PY 2003
VL 35
IS 3
BP 239
EP 245
DI 10.1055/s-2003-37268
PG 7
WC Gastroenterology & Hepatology; Surgery
WE Science Citation Index Expanded (SCI-EXPANDED)
SC Gastroenterology & Hepatology; Surgery
GA 651BV
UT WOS:000181301100010
PM 12584645
DA 2026-05-09
ER

PT J
AU Mishra, A
Tyberg, A
AF Mishra, Avantika
Tyberg, Amy
TI Endoscopic ultrasound guided biliary drainage: a comprehensive review
SO TRANSLATIONAL GASTROENTEROLOGY AND HEPATOLOGY
LA English

DT Review
DE Endoscopic ultrasound-guided biliary drainage (EUS-BD);
hepaticogastrostomy (HG); choledochoduodenostomy (CD)
ID BILE-DUCT OBSTRUCTION; RETROGRADE CHOLANGIOPANCREATOGRAPHY; PERCUTANEOUS DRAINAGE; METAL STENT; FAILED ERCP; CHOLEDOCHODUODENOSTOMY; HEPATICOGASTROSTOMY; ACCESS; CHOLANGIOGRAPHY; COMPLICATIONS
AB Endoscopic ultrasound-guided biliary drainage (EUS-BD) has become widely accepted over the past several years for complicated biliary drainage. When conventional endoscopic retrograde cholangiopancreatography (ERCP) is not successful or feasible due to a variety of constraints, an ultrasound-guided technique through intrahepatic and extrahepatic approaches allows adequate management of hepatobiliary obstruction. Other management alternatives include percutaneous transhepatic biliary drainages (PTBD) and complicated surgical approaches, both of which can be associated with increased morbidity and mortality. There are various technical approaches to allow successful biliary drainage which will be reviewed in this article.
C1 [Mishra, Avantika; Tyberg, Amy] Rutgers Robert Wood Johnson Univ Hosp, Dept Gastroenterol, New Brunswick, NJ 08901 USA.
C3 Rutgers University System; Rutgers University New Brunswick; Rutgers
University Biomedical & Health Sciences
RP Tyberg, A (corresponding author), Rutgers Robert Wood Johnson Univ Hosp, Dept Gastroenterol, New Brunswick, NJ 08901 USA.
EM amy.tyberg@gmail.com
CR Artifon ELA, 2015, ENDOSC ULTRASOUND, V4, P235, DOI 10.4103/2303-9027.163010
Artifon ELA, 2015, GASTROINTEST ENDOSC, V81, P950, DOI 10.1016/j.gie.2014.09.047
Bang JY, 2018, GASTROINTEST ENDOSC, V88, P9, DOI 10.1016/j.gie.2018.03.012
Baron TH, 2006, AM J GASTROENTEROL, V101, P892, DOI 10.1111/j.1572-0241.2006.00675.x
Boulay Brian R, 2018, Gastrointest Endosc Clin N Am, V28, P171, DOI [10.1016/j.giec.2017.11.005, 10.1016/j.giec.2017.11.005]
Carr-Locke DL, 2002, GASTROINTEST ENDOSC, V56, pS157, DOI 10.1067/mge.2002.129023
Coté GA, 2012, CLIN GASTROENTEROL H, V10, P920, DOI 10.1016/j.cgh.2012.02.019
Dhir V, 2013, UNITED EUR GASTROENT, V1, P103, DOI 10.1177/2050640613480145
Dhir V, 2014, DIGEST ENDOSC, V26, P430, DOI 10.1111/den.12153
Dhir V, 2012, GASTROINTEST ENDOSC, V75, P354, DOI 10.1016/j.gie.2011.07.075
Enochsson L, 2010, GASTROINTEST ENDOSC, V72, P1175, DOI 10.1016/j.gie.2010.07.047
Dominguez-Muñoz JE, 2017, ENDOSC ULTRASOUND, V6, pS119, DOI 10.4103/eus.eus_79_17
FERRUCCI JT, 1980, RADIOLOGY, V135, P1
Fogel EL, 2003, ENDOSCOPY, V35, P156, DOI 10.1055/s-2003-37011
Giovannini M, 2001, ENDOSCOPY, V33, P898, DOI 10.1055/s-2001-17324
Godat S, 2017, ENDOSC ULTRASOUND, V6, P181, DOI 10.4103/2303-9027.208174
Gupta K, 2014, J CLIN GASTROENTEROL, V48, P80, DOI 10.1097/MCG.0b013e31828c6822
Holt BA, 2016, GASTROINTEST ENDOSC, V83, P160, DOI 10.1016/j.gie.2015.06.019
Imai H, 2017, ONCOLOGY-BASEL, V93, P69, DOI 10.1159/000481233
Itoi T, 2016, GASTROINTEST ENDOSC, V83, P401, DOI 10.1016/j.gie.2015.05.049
Kahaleh M, 2006, GASTROINTEST ENDOSC, V64, P52, DOI 10.1016/j.gie.2006.01.063

Kahaleh M, 2005, GASTROINTEST ENDOSC, V61, P307, DOI 10.1016/S0016-5107(04)02585-4
Kawakubo K, 2016, ENDOSCOPY, V48, P164, DOI 10.1055/s-0034-1393179
Khashab MA, 2016, ENDOSC INT OPEN, V4, pE487, DOI 10.1055/s-0042-102648
Khashab MA, 2015, DIGEST DIS SCI, V60, P557, DOI 10.1007/s10620-014-3300-6
KUMAR S, 1995, GASTROINTEST ENDOSC, V41, P445, DOI 10.1016/S0016-5107(05)80001-X
Kumbhari V, 2015, GASTROINTEST ENDOSC, V81, P1015, DOI 10.1016/j.gie.2014.10.019
Lee TH, 2016, CLIN GASTROENTEROL H, V14, P1011, DOI 10.1016/j.cgh.2015.12.032
Mallery S, 2004, GASTROINTEST ENDOSC, V59, P100, DOI 10.1016/S0016-5107(03)02300-9
Mangiavillano B, 2008, ENDOSCOPY, V40, pE87, DOI 10.1055/s-2007-995548
Minaga K, 2018, DIGEST ENDOSC, V30, P38, DOI 10.1111/den.12910
Nennstiel S, 2015, J CLIN GASTROENTEROL, V49, P764, DOI 10.1097/MCG.0000000000000275
Ogura T, 2016, WORLD J GASTROENTERO, V22, P3945, DOI 10.3748/wjg.v22.i15.3945
Ogura T, 2016, ENDOSCOPY, V48, P156, DOI 10.1055/s-0034-1392859
Ogura T, 2015, ENDOSCOPY, V47, P72, DOI 10.1055/s-0034-1378111
Oh D, 2017, THER ADV GASTROENTER, V10, P42, DOI 10.1177/1756283X16671671
Oh HC, 2007, ENDOSCOPY, V39, P731, DOI 10.1055/s-2007-966577
Paik WH, 2018, AM J GASTROENTEROL, V113, P987, DOI 10.1038/s41395-018-0122-8
Park SJ, 2013, GASTROINTEST ENDOSC, V78, P374, DOI 10.1016/j.gie.2013.04.183
POLLOCK TW, 1979, ARCH SURG-CHICAGO, V114, P148
Sarkaria S, 2013, GUT LIVER, V7, P129, DOI 10.5009/gnl.2013.7.2.129
Schöfl R, 2001, ENDOSCOPY, V33, P147
Shami VM, 2010, DIGEST LIVER DIS, V42, P419, DOI 10.1016/j.dld.2009.09.009
Sharaiha RZ, 2017, GASTROINTEST ENDOSC, V85, P904, DOI 10.1016/j.gie.2016.12.023
Sharaiha RZ, 2016, SURG ENDOSC, V30, P5500, DOI 10.1007/s00464-016-4913-y
Teoh AYB, 2018, GUT, V67, P1209, DOI 10.1136/gutjnl-2017-314341
Tsuchiya T, 2018, GASTROINTEST ENDOSC, V87, P1138, DOI 10.1016/j.gie.2017.08.017
Tyberg A, 2016, GASTROINTEST ENDOSC, V84, P941, DOI 10.1016/j.gie.2016.05.035
Uemura RS, 2018, J CLIN GASTROENTEROL, V52, P123, DOI 10.1097/MCG.0000000000000948
van der Gaag NA, 2010, NEW ENGL J MED, V362, P129, DOI 10.1056/NEJMoa0903230
VOEGELI DR, 1985, AM J SURG, V150, P243, DOI 10.1016/0002-9610(85)90129-1
Wang KX, 2016, GASTROINTEST ENDOSC, V83, P1218, DOI 10.1016/j.gie.2015.10.033
Yamao K, 2010, GUT LIVER, V4, pS67, DOI 10.5009/gnl.2010.4.S1.S67
NR 53
TC 22
Z9 25
U1 0
U2 4
PU AME PUBLISHING COMPANY
PI SHATIN
PA FLAT-RM C 16F, KINGS WING PLAZA 1, NO 3 KWAN ST, SHATIN, HONG KONG
00000, PEOPLES R CHINA
EI 2415-1289

J9 TRANSL GASTROENT HEP
JI Transl. Gastroenterol. Hepatol.
PD FEB
PY 2019
VL 4
AR 10
DI 10.21037/tgh.2019.01.03
PG 8
WC Gastroenterology & Hepatology
WE Emerging Sources Citation Index (ESCI)
SC Gastroenterology & Hepatology
GA HM8YV
UT WOS:000459770300003
PM 30976713
OA Green Published, gold
DA 2026-05-09
ER

PT J
AU Cho, SH
Oh, D
Song, TJ
Park, DH
Seo, DW
Lee, SK
Kim, MH
Lee, YN
Moon, JH
Lee, SS
AF Cho, Sung Hyun
Oh, Dongwook
Song, Tae Jun
Park, Do Hyun
Seo, Dong-Wan
Lee, Sung Koo
Kim, Myung-Hwan
Lee, Yun Nah
Moon, Jong Ho
Lee, Sang Soo
TI Comparison of the effectiveness and safety of lumen-apposing metal
stents and anti-migrating tubular self-expandable metal stents for
EUS-guided gallbladder drainage in high surgical risk patients with

acute cholecystitis
SO GASTROINTESTINAL ENDOSCOPY
LA English
DT Article
ID LONG-TERM OUTCOMES; PERCUTANEOUS CHOLECYSTOSTOMY; FEASIBILITY
AB Background and Aims: Endoscopic ultrasound-guided gallbladder drainage (EUS-GBD) using an anti-migrating tubular self-expandable metal stent (ATSEMS) is performed in high surgical risk patients with acute cholecystitis. The newly introduced lumen-apposing metal stent (LAMS) is expected to reduce the risk of tubular self-expandable metal stent-related adverse events such as stent migration, but no comparative studies have been carried out between LAMSs and ATSEMSs for EUS-GBD.

Methods: We reviewed the prospectively collected EUS-GBD database at Asan Medical Center and Bucheon Soonchunhyang hospital to analyze consecutive patients with acute cholecystitis who underwent EUS-GBD with LAMSs or ATSEMSs between January 2015 and December 2017. Technical success, clinical success, adverse events, and recurrence of cholecystitis were evaluated.

Results: A total of 71 patients (36 with LAMSs, 35 with ATSEMSs) were analyzed. The LAMS group had longer median procedure time (15.5 minutes) than the ATSEMS group (11 minutes; P = .017). The 2 groups did not show significant differences in terms of technical success (LAMS, 94% vs ATSEMS, 100%; P = .49), clinical success (94% vs 100%; P = .49), procedure-related adverse events (0% vs 2.9%; P = .99), and stent-related late adverse events (11.8% vs 5.8%; P = .43). During follow-up, the 2 groups had similar rates of cholecystitis recurrence at 6 months (LAMS, 3.4% vs ATSEMS, 3.1%, P = .99) and 12 months (8.3% vs 3.1%, P = .56).

Conclusions: In high surgical risk patients with acute cholecystitis, LAMSs and ATSEMSs for EUS-GBD showed similar rates of technical success, clinical success, procedure-related adverse events, stent-related late adverse events, and recurrence of cholecystitis.
C1 [Cho, Sung Hyun] Univ Ulsan, Asan Med Ctr, Coll Med, Dept Internal Med, Seoul, South Korea.

[Oh, Dongwook; Song, Tae Jun; Park, Do Hyun; Seo, Dong-Wan; Lee, Sung Koo; Kim, Myung-Hwan; Lee, Sang Soo] Univ Ulsan, Asan Med Ctr, Coll Med, Dept Gastroenterol, 88 Olymp Ro,43 Gil, Seoul 05505, South Korea.

[Lee, Yun Nah; Moon, Jong Ho] Soonchunhyang Univ, Sch Med, Dept Gastroenterol, Bucheon, South Korea.
C3 University of Ulsan; Asan Medical Center; University of Ulsan; Asan
Medical Center; Soonchunhyang University
RP Lee, SS (corresponding author), Univ Ulsan, Asan Med Ctr, Coll Med, Dept Gastroenterol, 88 Olymp Ro,43 Gil, Seoul 05505, South Korea.
RI MOON, JONG/AAJ-1316-2020; Lee, Yun/J-1260-2014
OI Cho, Sung Hyun/0000-0001-8664-4090
CR Anderloni A, 2016, SURG ENDOSC, V30, P5200, DOI 10.1007/s00464-016-4894-x
Berman M, 2002, ISRAEL MED ASSOC J, V4, P331
Chan SM, 2017, GASTROINTEST ENDOSC, V85, P1225, DOI 10.1016/j.gie.2016.10.014
Cho DH, 2019, SURG ENDOSC, V33, P2135, DOI 10.1007/s00464-018-6485-5
Choi JH, 2014, ENDOSCOPY, V46, P656, DOI 10.1055/s-0034-1365720
Irani S, 2015, GASTROINTEST ENDOSC, V82, P1110, DOI 10.1016/j.gie.2015.05.045
Jain D, 2018, DIGESTION, V98, P1, DOI 10.1159/000487185
Jang JW, 2012, GASTROENTEROLOGY, V142, P805, DOI 10.1053/j.gastro.2011.12.051
Jang JW, 2011, GASTROINTEST ENDOSC, V74, P176, DOI 10.1016/j.gie.2011.03.1120

Kamata K, 2017, WORLD J GASTROENTERO, V23, P661, DOI 10.3748/wjg.v23.i4.661
Khan MA, 2017, GASTROINTEST ENDOSC, V85, P76, DOI 10.1016/j.gie.2016.06.032
Lo CM, 1998, ANN SURG, V227, P461, DOI 10.1097/00000658-199804000-00001
Loozen CS, 2017, DIGEST SURG, V34, P371, DOI 10.1159/000455241
Mayumi T, 2018, J HEPATO-BIL-PAN SCI, V25, P96, DOI 10.1002/jhbp.519
Oh D, 2019, GASTROINTEST ENDOSC, V89, P289, DOI 10.1016/j.gie.2018.08.052
Sanjay P, 2013, HPB, V15, P511, DOI 10.1111/j.1477-2574.2012.00610.x
Teoh AYB, 2017, ENDOSCOPY, V49, P130, DOI 10.1055/s-0042-119036
Tyberg A, 2018, J CLIN GASTROENTEROL, V52, P79, DOI 10.1097/MCG.0000000000000786
Walter D, 2016, GUT, V65, P6, DOI 10.1136/gutjnl-2015-309925
Yokoe M, 2018, J HEPATO-BIL-PAN SCI, V25, P41, DOI 10.1002/jhbp.515
NR 20
TC 37
Z9 44
U1 0
U2 0
PU MOSBY-ELSEVIER
PI NEW YORK
PA 360 PARK AVENUE SOUTH, NEW YORK, NY 10010-1710 USA
SN 0016-5107
EI 1097-6779
J9 GASTROINTEST ENDOSC
JI Gastrointest. Endosc.
PD MAR
PY 2020
VL 91
IS 3
BP 543
EP 550
DI 10.1016/j.gie.2019.09.042
PG 8
WC Gastroenterology & Hepatology
WE Science Citation Index Expanded (SCI-EXPANDED)
SC Gastroenterology & Hepatology
GA KN4ZL
UT WOS:000514847000006
PM 31629721
DA 2026-05-09
ER

PT J
AU Lee, HS

Chung, MJ
AF Lee, Hee Seung
Chung, Moon Jae
TI Past, Present, and Future of Gastrointestinal Stents: New Endoscopic Ultrasonography-Guided Metal Stents and Future Developments
SO CLINICAL ENDOSCOPY
LA English
DT Review
DE Endosonography; Metal stent
ID PANCREATIC FLUID COLLECTIONS; MALIGNANT BILIARY OBSTRUCTION; GALLBLADDER DRAINAGE; PSEUDOCYST DRAINAGE; PERCUTANEOUS CHOLECYSTOSTOMY; TRANSMURAL DRAINAGE; ACUTE CHOLECYSTITIS; RANDOMIZED-TRIAL; STEP PLACEMENT; BILE-DUCT
AB Innovations in stent technology and technological advances in endoscopic ultrasonography have led to rapid expansion of their use in the field of gastrointestinal diseases. In particular, endoscopic ultrasonography-guided metal stent insertion has been used for the management of pancreatic fluid collection, bile duct drainage, gallbladder decompression, and gastric bypass. Endoscopic ultrasonography-guided drainage of intra-abdominal fluid collections using a plastic or metal stent is well established. Because of the various limitations-such as stent migration, injury and bleeding in the lumen-recently developed, fully covered self-expanding metal stents or lumen-apposing metal stents have been introduced for those fluids management. This article reviews the recent literature on newly developed endoscopic ultrasonography-guided metal stents and the efficacy thereof.
C1 [Lee, Hee Seung; Chung, Moon Jae] Yonsei Univ, Coll Med, Inst Gastroenterol, Dept Internal Med, Seoul, South Korea.
C3 Yonsei University; Yonsei University Health System
RP Chung, MJ (corresponding author), Yonsei Univ, Coll Med, Dept Internal Med, Div Gastroenterol, 50-1 Yonsei Ro,Seodaemun Gu, Seoul 03722, South Korea.
EM MJCHUNG@yuhs.ac
OI Lee, HEE SEUNG/0000-0002-2825-3160; Chung, Moon Jae/0000-0002-5920-8549
CR Bang JY, 2015, DIGEST ENDOSC, V27, P486, DOI 10.1111/den.12418
Bapaye A, 2015, DIGEST ENDOSC, V27, P499, DOI 10.1111/den.12421
Binmoeller KF, 2012, ENDOSCOPY, V44, P499, DOI 10.1055/s-0032-1309382
Bories E, 2007, ENDOSCOPY, V39, P287, DOI 10.1055/s-2007-966212
Burmester E, 2003, GASTROINTEST ENDOSC, V57, P246, DOI 10.1067/mge.2003.85
Chandran S, 2015, GASTROINTEST ENDOSC, V81, P127, DOI 10.1016/j.gie.2014.06.025
Chang L, 2000, AM J SURG, V180, P198, DOI 10.1016/S0002-9610(00)00476-1
Dhir V, 2015, GASTROINTEST ENDOSC, V82, P650, DOI 10.1016/j.gie.2015.01.061
Dhir V, 2015, GASTROINTEST ENDOSC, V81, P913, DOI 10.1016/j.gie.2014.09.054
Dhir V, 2014, DIGEST ENDOSC, V26, P430, DOI 10.1111/den.12153
Giovannini M, 2003, ENDOSCOPY, V35, P1076, DOI 10.1055/s-2003-44596
Giovannini M, 2001, ENDOSCOPY, V33, P898, DOI 10.1055/s-2001-17324
Giovannini M, 2012, ENDOSC ULTRASOUND, V1, P119, DOI 10.7178/eus.03.002
GLENN F, 1977, ANN SURG, V185, P185, DOI 10.1097/00000658-197702000-00009
Hamada T, 2014, DIGEST DIS SCI, V59, P1931, DOI 10.1007/s10620-014-3062-1

Irani S, 2015, GASTROINTEST ENDOSC, V82, P1110, DOI 10.1016/j.gie.2015.05.045
Itoi T, 2016, GUT, V65, P193, DOI 10.1136/gutjnl-2015-310348
Itoi T, 2015, J HEPATO-BIL-PAN SCI, V22, P3, DOI 10.1002/jhbp.148
Itoi T, 2013, GASTROINTEST ENDOSC, V78, P934, DOI 10.1016/j.gie.2013.09.025
Itoi T, 2012, GASTROINTEST ENDOSC, V75, P870, DOI 10.1016/j.gie.2011.10.020
Jang JW, 2012, GASTROENTEROLOGY, V142, P805, DOI 10.1053/j.gastro.2011.12.051
Jang JW, 2011, GASTROINTEST ENDOSC, V74, P176, DOI 10.1016/j.gie.2011.03.1120
Kawakubo K, 2016, ENDOSCOPY, V48, P164, DOI 10.1055/s-0034-1393179
Kawakubo K, 2014, J HEPATO-BIL-PAN SCI, V21, P328, DOI 10.1002/jhbp.27
Khashab MA, 2015, GASTROINTEST ENDOSC, V82, P932, DOI 10.1016/j.gie.2015.06.017
Khashab MA, 2013, GASTROINTEST ENDOSC, V78, P734, DOI 10.1016/j.gie.2013.05.013
Kiviluoto T, 1998, LANCET, V351, P321, DOI 10.1016/S0140-6736(97)08447-X
Law R, 2016, CLIN GASTROENTEROL H, V14, P476, DOI 10.1016/j.cgh.2015.10.026
Lee BU, 2014, ENDOSCOPY, V46, P1078, DOI 10.1055/s-0034-1390871
McKay A, 2012, SURG ENDOSC, V26, P1343, DOI 10.1007/s00464-011-2035-0
Mcvay T, 2015, ENDOSC ULTRASOUND, V4, P1, DOI 10.4103/2303-9027.151295
Moon JH, 2014, GASTROINTEST ENDOSC, V79, P990, DOI 10.1016/j.gie.2014.02.015
Oh D, 2016, GASTROINTEST ENDOSC, V83, P366, DOI 10.1016/j.gie.2015.07.015
Park DH, 2015, J GASTROEN HEPATOL, V30, P1461, DOI 10.1111/jgh.13027
Park DH, 2009, AM J GASTROENTEROL, V104, P2168, DOI 10.1038/ajg.2009.254
Perez-Miranda M, 2010, WORLD J GASTRO ENDOS, V2, P212, DOI 10.4253/wjge.v2.i6.212
Poincloux L, 2015, ENDOSCOPY, V47, P794, DOI [10.1055/s-0035-1545399, 10.1055/s-0034-1391988]
Püspök A, 2005, AM J GASTROENTEROL, V100, P1743, DOI 10.1111/j.1572-0241.2005.41806.x
Raijman I, 2015, ENDOSC ULTRASOUND, V4, P213, DOI 10.4103/2303-9027.163000
Seewald S, 2009, DIGEST ENDOSC, V21, pS61, DOI 10.1111/j.1443-1661.2009.00860.x
Shah RJ, 2015, CLIN GASTROENTEROL H, V13, P747, DOI 10.1016/j.cgh.2014.09.047
Sharaiha RZ, 2015, GASTROINTEST ENDOSC, V82, P822, DOI 10.1016/j.gie.2015.02.035
Siddiqui AA, 2015, GASTROINTEST ENDOSC
Smith IB, 2015, ENDOSC INT OPEN, V3, pE154, DOI 10.1055/s-0034-1391481
Song TJ, 2014, GASTROINTEST ENDOSC, V80, P707, DOI 10.1016/j.gie.2014.05.327
Varadarajulu S, 2013, GASTROENTEROLOGY, V145, P583, DOI 10.1053/j.gastro.2013.05.046
WALLACE MJ, 1986, RADIOLOGY, V158, P309, DOI 10.1148/radiology.158.2.3941857
Walter D, 2016, GUT, V65, P6, DOI 10.1136/gutjnl-2015-309925
Walter D, 2015, ENDOSCOPY, V47, P63, DOI 10.1055/s-0034-1378113
Wiersema MJ, 1996, GASTROINTEST ENDOSC, V43, P102, DOI 10.1016/S0016-5107(06)80108-2
Will U, 2007, ENDOSCOPY, V39, P292, DOI 10.1055/s-2007-966215
Yamamoto N, 2013, GASTROINTEST ENDOSC, V77, P809, DOI 10.1016/j.gie.2013.01.009
Yi SW, 2014, SURG ENDOSC, V28, P1439, DOI 10.1007/s00464-013-3371-z
NR 53
TC 7
Z9 11
U1 0

U2 8
PU KOREAN SOC GASTROINTESTINAL ENDOSCOPY
PI SEOUL
PA 2003 LG PALACE, 165-8 DONGGYO-DONG, MAPO-GU, SEOUL, 121-754, SOUTH KOREA
SN 2234-2400
EI 2234-2443
J9 CLIN ENDOSC
JI Clin. Endosc.
PD MAR
PY 2016
VL 49
IS 2
BP 131
EP 138
DI 10.5946/ce.2016.044
PG 8
WC Gastroenterology & Hepatology
WE Emerging Sources Citation Index (ESCI)
SC Gastroenterology & Hepatology
GA FD1EN
UT WOS:000407280300007
PM 27000424
OA Green Submitted, gold
DA 2026-05-09
ER

PT J
AU Iwashita, T
Doi, S
Yasuda, I
AF Iwashita, Takuji
Doi, Shinpei
Yasuda, Ichiro
TI Endoscopic ultrasound-guided biliary drainage: a review
SO CLINICAL JOURNAL OF GASTROENTEROLOGY
LA English
DT Review
DE Endoscopic ultrasound; Endoscopic retrograde cholangiopancreatography;
Biliary drainage; Rendezvous; Antegrade
AB Endoscopic retrograde cholangiography (ERCP) is widely used as a first-line therapy for biliary drainage. ERCP occasionally fails owing to anatomical or technical problems, despite high reported success rates. Endoscopic ultrasound-guided biliary drainage (EUS-BD) has recently emerged as an effective alternative biliary drainage method after

unsuccessful ERCP. EUS-BD can be essentially divided into 3 different techniques-(1) EUS-guided transluminal biliary drainage including choledoco-duodenostomy and hepaticogastrostomy, (2) EUS-rendezvous technique, and (3) EUS-antegrade approach. Here, we focus on the current status of EUS-BD in light of these 3 different techniques.

C1 [Iwashita, Takuji; Doi, Shinpei; Yasuda, Ichiro] Gifu Univ Hosp, Dept Internal Med 1, 1-1 Yanagido, Gifu 5011194, Japan.
C3 Gifu University
RP Iwashita, T (corresponding author), Gifu Univ Hosp, Dept Internal Med 1, 1-1 Yanagido, Gifu 5011194, Japan.
EM takuji@w7.dion.ne.jp
RI Yasuda, Ichiro/IUP-0131-2023
OI Yasuda, Ichiro/0000-0002-6888-0310

CR Ang TL, 2007, J PANCREAS, V8, P438
Artifon ELA, 2007, CLINICS, V62, P799, DOI 10.1590/S1807-59322007000600023
Artifon ELA, 2012, J CLIN GASTROENTEROL, V46, P768, DOI 10.1097/MCG.0b013e31825f264c
Artifon ELA, 2011, J PANCREAS, V12, P610
Attasaranya S, 2012, GASTROENT RES PRACT, V2012, DOI 10.1155/2012/680753
Beissert M, 2002, Z GASTROENTEROL, V40, P503, DOI 10.1055/s-2002-32806
Belletrutti Paul J, 2011, J Gastrointest Cancer, V42, P137, DOI 10.1007/s12029-010-9175-7
Bories E, 2007, ENDOSCOPY, V39, P287, DOI 10.1055/s-2007-966212
Brauer BC, 2009, GASTROINTEST ENDOSC, V70, P471, DOI 10.1016/j.gie.2008.12.233
Burmester E, 2003, GASTROINTEST ENDOSC, V57, P246, DOI 10.1067/mge.2003.85
Dhir V, 2012, GASTROINTEST ENDOSC, V75, P354, DOI 10.1016/j.gie.2011.07.075
Fabbri C, 2011, ENDOSCOPY, V43, P438, DOI 10.1055/s-0030-1256097
Fujita N, 2007, WORLD J GASTROENTERO, V13, P5512, DOI 10.3748/wjg.v13.i41.5512
Giovannini M, 2003, ENDOSCOPY, V35, P1076, DOI 10.1055/s-2003-44596
Giovannini M, 2001, ENDOSCOPY, V33, P898, DOI 10.1055/s-2001-17324
Hanada K, 2009, DIGEST ENDOSC, V21, pS75, DOI 10.1111/j.1443-1661.2009.00855.x
Hara K, 2013, ENDOSCOPY, V45, P392, DOI 10.1055/s-0032-1326076
Hara K, 2011, AM J GASTROENTEROL, V106, P1239, DOI 10.1038/ajg.2011.84
Horaguchi J, 2009, DIGEST ENDOSC, V21, P239, DOI 10.1111/j.1443-1661.2009.00899.x
Itoi T, 2008, WORLD J GASTROENTERO, V14, P6078, DOI 10.3748/wjg.14.6078
Iwamuro M, 2010, DIGEST ENDOSC, V22, P236, DOI 10.1111/j.1443-1661.2010.00997.x
Iwashita T, 2012, ENDOSCOPY, V44, P60, DOI 10.1055/s-0030-1256871
Iwashita T, 2013, DIGEST DIS SCI, V58, P2417, DOI 10.1007/s10620-013-2645-6
Iwashita Takuji, 2012, Gastrointest Endosc Clin N Am, V22, P249, DOI 10.1016/j.giec.2012.04.018
Kahaleh M, 2006, GASTROINTEST ENDOSC, V64, P52, DOI 10.1016/j.gie.2006.01.063
Kahaleh M, 2005, GASTROINTEST ENDOSC, V61, P307, DOI 10.1016/S0016-5107(04)02585-4
Kama NA, 1999, HEPATO-GASTROENTEROL, V46, P103
Katanuma A, 2012, DIGEST ENDOSC, V24, P34, DOI 10.1111/j.1443-1661.2012.01256.x
Kawakubo K, 2013, J HEPATOBILIARY PANC
Kawakubo K, 2013, SURG ENDOSC, V27, P3437, DOI 10.1007/s00464-013-2896-5
Kawakubo K, 2012, GUT LIVER, V6, P399, DOI 10.5009/gnl.2012.6.3.399
Khashab MA, 2013, GASTROINTEST ENDOSC, V78, P734, DOI 10.1016/j.gie.2013.05.013

Kim TH, 2012, WORLD J GASTROENTERO, V18, P2526, DOI 10.3748/wjg.v18.i20.2526
Kim YS, 2010, ENDOSCOPY, V42, P496, DOI 10.1055/s-0029-1244082
Komaki T, 2011, PANCREATOLOGY, V11, P47, DOI 10.1159/000323508
Lai R, 2005, ENDOSCOPY, V37, P487, DOI 10.1055/s-2005-861250
Mallery S, 2004, GASTROINTEST ENDOSC, V59, P100, DOI 10.1016/S0016-5107(03)02300-9
Maranki J, 2009, ENDOSCOPY, V41, P532, DOI 10.1055/s-0029-1214712
Nguyen-Tang T, 2010, ENDOSCOPY, V42, P232, DOI 10.1055/s-0029-1243858
Park DH, 2013, GASTROINTEST ENDOSC, V78, P91, DOI 10.1016/j.gie.2013.01.042
Park DH, 2012, GASTROINTEST ENDOSC, V75, P692, DOI 10.1016/j.gie.2011.04.013
Park DH, 2011, GASTROINTEST ENDOSC, V74, P1276, DOI 10.1016/j.gie.2011.07.054
Park DH, 2010, GASTROINTEST ENDOSC, V71, P413, DOI 10.1016/j.gie.2009.10.015
Park DH, 2009, AM J GASTROENTEROL, V104, P2168, DOI 10.1038/ajg.2009.254
Püspök A, 2005, AM J GASTROENTEROL, V100, P1743, DOI 10.1111/j.1572-0241.2005.41806.x
Ramírez-Luna MA, 2011, ENDOSCOPY, V43, P826, DOI 10.1055/s-0030-1256406
Shah JN, 2012, GASTROINTEST ENDOSC, V75, P56, DOI 10.1016/j.gie.2011.08.032
Siddiqui AA, 2011, SURG ENDOSC, V25, P549, DOI 10.1007/s00464-010-1216-6
SMITH AC, 1994, LANCET, V344, P1655, DOI 10.1016/S0140-6736(94)90455-3
Song TJ, 2012, WORLD J GASTROENTERO, V18, P4435, DOI 10.3748/wjg.v18.i32.4435
Tarantino I, 2008, ENDOSCOPY, V40, P336, DOI 10.1055/s-2007-995455
Tonozuka R, 2013, DIGEST ENDOSC, V25, P100, DOI 10.1111/den.12063
Vila JJ, 2012, GASTROINTEST ENDOSC, V76, P1133, DOI 10.1016/j.gie.2012.08.001
Will U, 2007, ENDOSCOPY, V39, P292, DOI 10.1055/s-2007-966215
Yamao K, 2008, ENDOSCOPY, V40, P340, DOI 10.1055/s-2007-995485
Yamao K, 2006, GASTROINTEST ENDOSC, V64, P663, DOI 10.1016/j.gie.2006.07.003
NR 56
TC 111
Z9 134
U1 0
U2 3
PU SPRINGER JAPAN KK
PI TOKYO
PA SHIROYAMA TRUST TOWER 5F, 4-3-1 TORANOMON, MINATO-KU, TOKYO, 105-6005, JAPAN
SN 1865-7257
EI 1865-7265
J9 CLIN J GASTROENTEROL
JI Clin. J. Gastroenterol.
PD APR
PY 2014
VL 7
IS 2
BP 94

EP 102
DI 10.1007/s12328-014-0467-5
PG 9
WC Gastroenterology & Hepatology
WE Emerging Sources Citation Index (ESCI)
SC Gastroenterology & Hepatology
GA VC3JS
UT WOS:000433682300002
PM 24765215
OA Green Submitted, hybrid
DA 2026-05-09
ER

PT J
AU Ogura, T
Nakai, Y
Iwashita, T
Higuchi, K
Itoi, T
AF Ogura, Takeshi
Nakai, Yosuke
Iwashita, Takuji
Higuchi, Kazuhide
Itoi, Takao
TI Novel fine gauge electrocautery dilator for endoscopic ultrasound-guided
biliary drainage: experimental and clinical evaluation study (with
video)
SO ENDOSCOPY INTERNATIONAL OPEN
LA English
DT Article
ID ADVERSE EVENTS; HEPATICOGASTROSTOMY; STENT
C1 [Ogura, Takeshi; Higuchi, Kazuhide] Osaka Med Coll, Dept Internal Med 2, Osaka, Japan.
[Nakai, Yosuke] Univ Tokyo, Grad Sch Med, Dept Gastroenterol, Tokyo, Japan.
[Iwashita, Takuji] Gifu Univ Hosp, Dept Internal Med 1, Gifu, Japan.
[Itoi, Takao] Tokyo Med Univ, Dept Gastroenterol & Hepatol, Tokyo, Japan.
C3 Osaka Medical & Pharmaceutical University; University of Tokyo; Gifu
University; Tokyo Medical University
RP Itoi, T (corresponding author), Tokyo Med Univ, Dept Gastroenterol & Hepatol, Shinjuku Ku, 6-7-1 Nishishinjuku, Tokyo 1600023, Japan.
EM itoitakao@gmail.com
RI Ogura, Takeshi/AAU-5643-2021
CR Amano M, 2017, J GASTROEN HEPATOL, V32, P716, DOI 10.1111/jgh.13489

Cotton PB, 2010, GASTROINTEST ENDOSC, V71, P446, DOI 10.1016/j.gie.2009.10.027
Giovannini M, 2003, ENDOSCOPY, V35, P1076, DOI 10.1055/s-2003-44596
Hedjoudje A, 2019, UNITED EUR GASTROENT, V7, P60, DOI 10.1177/2050640618808147
Honjo M, 2018, ENDOSC ULTRASOUND, V7, P376, DOI 10.4103/eus.eus_2_18
Ogura T, 2019, DIGEST ENDOSC, V31, P55, DOI 10.1111/den.13334
Ogura T, 2019, J HEPATO-BIL-PAN SCI, V26, pE3, DOI 10.1002/jhbp.609
Paik WH, 2014, WORLD J GASTROENTERO, V20, P5051, DOI 10.3748/wjg.v20.i17.5051
Park DH, 2011, GASTROINTEST ENDOSC, V74, P1276, DOI 10.1016/j.gie.2011.07.054
Walter D, 2016, GUT, V65, P6, DOI 10.1136/gutjnl-2015-309925
NR 10
TC 17
Z9 19
U1 0
U2 0
PU GEORG THIEME VERLAG KG
PI STUTTGART
PA RUDIGERSTR 14, D-70469 STUTTGART, GERMANY
SN 2364-3722
EI 2196-9736
J9 ENDOSC INT OPEN
JI Endosc. Int. Open
PD DEC
PY 2019
VL 7
IS 12
BP E1652
EP E1657
DI 10.1055/a-0961-7890
PG 6
WC Gastroenterology & Hepatology; Surgery
WE Emerging Sources Citation Index (ESCI)
SC Gastroenterology & Hepatology; Surgery
GA JU6TW
UT WOS:000501807800013
PM 31788548
OA Green Submitted, gold
DA 2026-05-09
ER

PT J
AU Shah, RH
Udawat, P

Dhir, V
AF Shah, Rahul Haresh
Udawat, Priyanka
Dhir, Vinay
TI Endoscopic Ultrasonography-Specific Stents
SO JOURNAL OF DIGESTIVE ENDOSCOPY
LA English
DT Review
DE BFMS; endoscopic ultrasound; LAMS
ID GUIDED BILIARY DRAINAGE; APPOSING METAL STENT; LONG-TERM OUTCOMES; MANAGEMENT; EFFICACY; SAFETY; ERCP
AB The evolution of the endoscopic ultrasonography (EUS) technique has necessitated the development of EUS-specific accessories and stents. In the earlier period, EUS-guided interventions mostly relied on borrowed accessories and stents from therapeutic endoscopy in particular ERCP. Traditional stents do not serve the purpose of EUS-guided transluminal stenting in all cases due to the length of the stent, no anchoring mechanism, and chance of migration. From 2011 onward, several EUS-specific stents became available, bringing a paradigm shift in interventional EUS. This article reviews current EUS-specific stents, indications, strengths, and weaknesses.
C1 [Shah, Rahul Haresh; Udawat, Priyanka; Dhir, Vinay] SL Raheja Hosp, Inst Digest & Liver Care, Dept Gastroenterol, Mumbai, Maharashtra, India.
RP Shah, RH (corresponding author), SL Raheja Hosp, Inst Digest & Liver Care, Dept Gastroenterol, Med Gastroenterol, Mumbai 400016, Maharashtra, India.
EM rahulhshah@hotmail.com
CR Bang JY, 2018, GASTROINTEST ENDOSC, V88, P9, DOI 10.1016/j.gie.2018.03.012
Binmoeller KF, 2011, ENDOSCOPY, V43, P337, DOI 10.1055/s-0030-1256127
Bukhari M, 2018, GASTROINTEST ENDOSC, V88, P486, DOI 10.1016/j.gie.2018.04.2356
Cho DH, 2017, GASTROINTEST ENDOSC, V85, P1067, DOI 10.1016/j.gie.2016.09.010
Dhindsa BS, 2020, ENDOSC ULTRASOUND, V9, P101, DOI 10.4103/eus.eus_80_19
Dhir V, 2017, DIGEST ENDOSC, V29, P472, DOI 10.1111/den.12818
Giovannini M, 2001, ENDOSCOPY, V33, P898, DOI 10.1055/s-2001-17324
Giovannini M, 2017, ENDOSC ULTRASOUND, V6, P281, DOI 10.4103/eus.eus_86_17
Hammad T, 2018, DIGEST DIS SCI, V63, P289, DOI 10.1007/s10620-017-4851-0
Itoi T, 2016, GUT, V65, P193, DOI 10.1136/gutjnl-2015-310348
Kedia P, 2015, GASTROINTEST ENDOSC, V82, P560, DOI 10.1016/j.gie.2015.03.1913
Lee TH, 2016, CLIN GASTROENTEROL H, V14, P1011, DOI 10.1016/j.cgh.2015.12.032
Mangiavillano B, 2016, WORLD J GASTROINTEST, V8, P143, DOI 10.4253/wjge.v8.i3.143
Paik WH, 2018, AM J GASTROENTEROL, V113, P987, DOI 10.1038/s41395-018-0122-8
Shah R, 2021, J DIG ENDOSC, V12, P3, DOI 10.1055/s-0041-1728956
Sharaiha RZ, 2017, GASTROINTEST ENDOSC, V85, P904, DOI 10.1016/j.gie.2016.12.023
Teoh AYB, 2018, GUT, V67, P1209, DOI 10.1136/gutjnl-2017-314341
Teoh AY, 2016, GASTROINTEST ENDOSC, V84, P62, DOI 10.1016/j.gie.2015.11.041
Teoh AYB, 2017, ENDOSCOPY, V49, P130, DOI 10.1055/s-0042-119036
Tsuchiya T, 2018, GASTROINTEST ENDOSC, V87, P1138, DOI 10.1016/j.gie.2017.08.017

Umeda J, 2015, GASTROINTEST ENDOSC, V82, P390, DOI 10.1016/j.gie.2015.02.041
Walter D, 2016, GUT, V65, P6, DOI 10.1136/gutjnl-2015-309925
Weilert Frank., 2015, GASTROINTESTINAL INT, V4, P40, DOI [10.1016/j.gii.2015.03.003, DOI 10.1016/j.gii.2015.03.003, 10.1016/j.gii.2015.03.003, DOI 10.1016/J.GII.2015.03.003]
Yang JL, 2019, ENDOSCOPY, V51, P1035, DOI 10.1055/a-0759-1353
NR 24
TC 1
Z9 1
U1 0
U2 1
PU THIEME MEDICAL PUBL INC
PI NEW YORK
PA 333 SEVENTH AVE, NEW YORK, NY 10001 USA
SN 0976-5042
EI 0976-5050
J9 J DIG ENDOSC
JI J. Dig. Endosc.
PD DEC
PY 2021
VL 12
IS 04
BP 229
EP 234
DI 10.1055/s-0041-1741514
PG 6
WC Gastroenterology & Hepatology
WE Emerging Sources Citation Index (ESCI)
SC Gastroenterology & Hepatology
GA YG9AA
UT WOS:000742770600006
OA Green Submitted, gold
DA 2026-05-09
ER

PT J
AU Hedjoudje, A
Sportes, A
Grabar, S
Zhang, A
Koch, S
Vuitton, L
Prat, F

AF Hedjoudje, A.
Sportes, A.
Grabar, S.
Zhang, A.
Koch, S.
Vuitton, L.
Prat, F.
TI Outcomes of endoscopic ultrasound-guided biliary drainage: A systematic
review and meta-analysis
SO UNITED EUROPEAN GASTROENTEROLOGY JOURNAL
LA English
DT Review
DE Biliary drainage; biliary obstruction; choledochoduodenostomy;
endoscopy-guided; hepaticogastrostomy
ID HYBRID METAL STENT; FAILED ERCP; CHOLEDOCHODUODENOSTOMY;
HEPATICOGASTROSTOMY; LONG; OBSTRUCTION; BENIGN;
CHOLANGIOPANCREATOGRAPHY; EXPERIENCE; SAFETY
AB Background Success and event rates of endoscopic ultrasound (EUS)-guided biliary drainage vary with techniques, and results from different studies remain inconsistent. Objective We conducted a proportion meta-analysis to evaluate the efficacy and safety of EUS-guided biliary drainage and compare the outcomes of current procedures. Methods We searched MEDLINE, Embase, Cochrane and Web of knowledge to identify studies reporting technical success, clinical success and complication rates of EUS-guided biliary drainage techniques to estimate their clinical and technical efficacy and safety. Results We identified 17 studies including a total of 686 patients. The overall clinical success and technical success rates were respectively 84% confidence interval (CI) 95% (80-88) and 96% CI 95% (93-98) for hepaticogastrostomy, and respectively 87% CI 95% (82-91) and 95% CI 95 (91-97) for choledochoduodenostomy. Reported adverse event rates were significantly higher ($p = 0.01$) for hepaticogastrostomy (29% CI 95% (24-34)) compared to choledochoduodenostomy (20% CI 95% (16-25)). Compared with hepaticogastrostomy, the pooled odds ratio for the complication rate of choledochoduodenostomy was 2.01 (1.25; 3.24) ($p = 0.0042$), suggesting that choledochoduodenostomy might be safer than hepaticogastrostomy. Conclusion The available literature suggests choledochoduodenostomy may be a safer approach compared to hepaticogastrostomy. Randomized controlled trials with sufficiently large cohorts are needed to compare techniques and confirm these findings.
C1 [Hedjoudje, A.; Koch, S.; Vuitton, L.] Univ Franche Comte, Gastroenterol & Endoscopy Unit, Hop Jean Minjoz, Besancon, France.
[Sportes, A.] Univ Paris 13, Dept Gastroenterol, Hop Avicenne, Bobigny, France.
[Grabar, S.] Univ Paris 05, Hop Hotel Dieu, AP HP, PRES Sorbonne Paris,Biostat & Epidemiol Unit, Paris, France.
[Grabar, S.] Inst Pierre Louis Epidemiol & Sante Publ, INSERM, UMR S 1136, Paris, France.
[Zhang, A.] Johns Hopkins Univ, Evidence Based Practice Ctr, Baltimore, MD USA.
[Prat, F.] Univ Paris 05, Gastroenterol & Endoscopy Unit, Hop Cochin, AP HP,Sorbonne Paris Cite,Fac Med, Paris, France.
C3 Universite Marie et Louis Pasteur; CHU Besancon; Universite Paris 13;
Assistance Publique Hopitaux Paris (APHP); Hopital Universitaire
Avicenne - APHP; Assistance Publique Hopitaux Paris (APHP); Universite

Paris Cite; Hopital Universitaire Hotel-Dieu - APHP; Sorbonne
Universite; Institut National de la Sante et de la Recherche Medicale
(Inserm); Johns Hopkins University; Universite Paris Cite; Assistance
Publique Hopitaux Paris (APHP); Hopital Universitaire Cochin - APHP
RP Hedjoudje, A (corresponding author), CHRU Jean Minjoz, 1 Rue Alexandre Fleming, F-25000 Besancon, France.
EM abdellah.hedjoudje@gmail.com
RI Abdellah, Hedjoudje/GZL-5605-2022; Grabar, Sophie/AAM-9857-2020
OI Grabar, Sophie/0000-0002-4816-4261
CR [Anonymous], 2009, BMJ-BRIT MED J, V21, P7, DOI [DOI 10.1136/bmj.b2535, DOI 10.1136/BMJ.B2700, 10.7326/0003-4819-151-4-200908180-00135, 10.1371/journal.pmed.1000097, DOI 10.1371/JOURNAL.PMED.1000097]
Artifon ELA, 2015, GASTROINTEST ENDOSC, V81, P950, DOI 10.1016/j.gie.2014.09.047
Artifon ELA, 2012, J PANCREAS, V13, P7
Born P, 1998, SCAND J GASTROENTERO, V33, P544
Cho DH, 2017, GASTROINTEST ENDOSC, V85, P1067, DOI 10.1016/j.gie.2016.09.010
Deeks JJ, 1998, LANCET, V352, P1908, DOI 10.1016/S0140-6736(05)60399-6
DERSIMONIAN R, 1986, CONTROL CLIN TRIALS, V7, P177, DOI 10.1016/0197-2456(86)90046-2
Egger M, 1997, BMJ-BRIT MED J, V315, P629, DOI 10.1136/bmj.315.7109.629
Giovannini M, 2001, ENDOSCOPY, V33, P898, DOI 10.1055/s-2001-17324
Hara K, 2013, ENDOSCOPY, V45, P392, DOI 10.1055/s-0032-1326076
Hara K, 2011, AM J GASTROENTEROL, V106, P1239, DOI 10.1038/ajg.2011.84
Kedia P, 2013, CLIN ENDOSC, V46, P552, DOI 10.5946/ce.2013.46.5.552
Khajanchee YS, 2012, J GASTROINTEST SURG, V16, P801, DOI 10.1007/s11605-011-1768-3
Khashab MA, 2016, ENDOSC INT OPEN, V4, pE175, DOI 10.1055/s-0041-109083
Kunda R, 2016, SURG ENDOSC, V30, P5002, DOI 10.1007/s00464-016-4845-6
Luu C, 2013, AM SURGEON, V79, P1054
Mallery S, 2004, GASTROINTEST ENDOSC, V59, P100, DOI 10.1016/S0016-5107(03)02300-9
Minaga K, 2017, SURG ENDOSC, V31, P4764, DOI 10.1007/s00464-017-5553-6
Moryoussef F, 2017, THER ADV GASTROENTER, V10, P537, DOI 10.1177/1756283X17702614
Nakai Y, 2016, ENDOSCOPY, V48, P1125, DOI 10.1055/s-0042-116595
Paik WH, 2017, ENDOSCOPY, V49, P544, DOI 10.1055/s-0043-102388
Paik WH, 2014, WORLD J GASTROENTERO, V20, P5051, DOI 10.3748/wjg.v20.i17.5051
Park DH, 2015, J GASTROEN HEPATOL, V30, P1461, DOI 10.1111/jgh.13027
Park DH, 2011, GASTROINTEST ENDOSC, V74, P1276, DOI 10.1016/j.gie.2011.07.054
Poincloux L, 2015, ENDOSCOPY, V47, P794, DOI [10.1055/s-0035-1545399, 10.1055/s-0034-1391988]
Sharaiha RZ, 2017, GASTROINTEST ENDOSC, V85, P904, DOI 10.1016/j.gie.2016.12.023
Siddiqui AA, 2011, SURG ENDOSC, V25, P549, DOI 10.1007/s00464-010-1216-6
Sohn TA, 1999, J AM COLL SURGEONS, V188, P658, DOI 10.1016/S1072-7515(99)00049-6
Song TJ, 2014, GASTROINTEST ENDOSC, V80, P707, DOI 10.1016/j.gie.2014.05.327
Spanheimer PM, 2014, J SURG ONCOL, V109, P697, DOI 10.1002/jso.23560
Sportes A, 2017, THER ADV GASTROENTER, V10, P483, DOI 10.1177/1756283X17702096
Sterne JAC, 2000, J CLIN EPIDEMIOL, V53, P1119, DOI 10.1016/S0895-4356(00)00242-0
Stroup DF, 2000, JAMA-J AM MED ASSOC, V283, P2008, DOI 10.1001/jama.283.15.2008

Uemura RS, 2018, J CLIN GASTROENTEROL, V52, P123, DOI 10.1097/MCG.0000000000000948
Umeda J, 2015, GASTROINTEST ENDOSC, V82, P390, DOI 10.1016/j.gie.2015.02.041
Vila JJ, 2012, GASTROINTEST ENDOSC, V76, P1133, DOI 10.1016/j.gie.2012.08.001
Wiersema MJ, 1996, GASTROINTEST ENDOSC, V44, P614, DOI 10.1016/S0016-5107(96)70022-6
Winick A B, 2001, Tech Vasc Interv Radiol, V4, P200, DOI 10.1016/S1089-2516(01)90026-5
Yamao K, 2008, ENDOSCOPY, V40, P340, DOI 10.1055/s-2007-995485
NR 39
TC 54
Z9 69
U1 0
U2 2
PU WILEY
PI HOBOKEN
PA 111 RIVER ST, HOBOKEN 07030-5774, NJ USA
SN 2050-6406
EI 2050-6414
J9 UNITED EUR GASTROENT
JI United European Gastroenterol. J.
PD FEB
PY 2019
VL 7
IS 1
BP 60
EP 68
DI 10.1177/2050640618808147
PG 9
WC Gastroenterology & Hepatology
WE Science Citation Index Expanded (SCI-EXPANDED)
SC Gastroenterology & Hepatology
GA HJ9CU
UT WOS:000457497200006
PM 30788117
OA Green Submitted, Bronze
DA 2026-05-09
ER

PT J
AU Alvarez-Sanchez, MV
Jenssen, C
Faiss, S
Napoléon, B
AF Alvarez-Sanchez, Maria Victoria

Jenssen, Christian
Faiss, Siegbert
Napoleon, Bertrand
TI Interventional endoscopic ultrasonography: an overview of safety and complications
SO SURGICAL ENDOSCOPY AND OTHER INTERVENTIONAL TECHNIQUES
LA English
DT Review
DE Endoscopic ultrasound; Celiac plexus; Pancreatic collections; Pelvic collections; Biliary drainage; Pancreatic drainage
ID PANCREATIC-FLUID COLLECTIONS; CELIAC PLEXUS NEUROLYSIS; GUIDED BILIARY DRAINAGE; PROSPECTIVE RANDOMIZED-TRIAL; TRANSMURAL DRAINAGE; SINGLE-STEP; PSEUDOCYST DRAINAGE; GANGLIA NEUROLYSIS; TRANSLUMINAL DRAINAGE; RENDEZVOUS TECHNIQUE
AB In recent years, endoscopic ultrasonography (EUS)-guided techniques have been developed as alternatives to surgical, radiologic, or conventional endoscopic approaches for the treatment or palliation of several digestive diseases. The use of EUS guidance allows the therapeutic area to be targeting more precisely, with a possible clinical benefit and less morbidity. Nevertheless, the risks persist and must be taken into consideration. This review gives an overview of the complications observed with the most established procedures of therapeutic EUS.

The PubMed and Embase databases were used to search English language articles on interventional EUS. The studies considered for inclusion were those reporting on complications of EUS-guided celiac plexus block (EUS-CPB), EUS-guided celiac plexus neurolysis (EUS-CPN), drainage of fluid pancreatic and pelvic collections, and EUS-guided biliary and pancreatic drainage (EUS-BD and EUS-PD). Variations in methodology and design in most studies made a thorough statistical analysis difficult. Instead, a frequency analysis of complications and a critical discussion were performed.

Although EUS-guided celiac plexus injection causes mainly mild and transient complications, growing experience shows that EUS-CPN is not as benign a procedure as previously thought. Most of the major complications have been observed in patients with chronic pancreatitis. The findings show that EUS-guided drainage of fluid collections is a safe procedure. Complications occur more often after the drainage of pancreatic abscesses and necrosis. Although the heterogeneity of studies dealing with pancreatobiliary drainage makes the evaluation of risks after these procedures difficult, complications after EUS-BD and EUS-PD are relatively frequent and can be severe. The technical complexity and the lack of specifically designed devices may account for their complication rates.

Clinicians can consider EUS-guided celiac injection and EUS-guided drainage of fluid collections to be safe alternatives to surgical and radiologic interventions. Well-designed prospective trials are needed to assess the risks of EUS-BD and EUS-PD accurately before they are broadly advocated after a failed endoscopic retrograde cholangiopancreatography (ERCP).
C1 [Alvarez-Sanchez, Maria Victoria; Napoleon, Bertrand] Hop Prive Jean Mermoz, Dept Gastroenterol & Hepatol, F-69008 Lyon, France.
[Jenssen, Christian] Krankenhaus Markisch Oderland, Dept Internal Med, Wriezen, Strausberg, Germany.
[Faiss, Siegbert] Asklepios Klin Barmbek, Dept Gastroenterol & Hepatol, Hamburg, Germany.
RP Alvarez-Sanchez, MV (corresponding author), Hop Prive Jean Mermoz, Dept Gastroenterol & Hepatol, 55 Ave Jean Mermoz, F-69008 Lyon, France.
EM victoria.alvarez.sanchez@hotmail.com

OI Napoleon, Bertrand/0000-0001-5866-8132

CR ADAMS DB, 1994, ANN SURG, V219, P481, DOI 10.1097/00000658-199405000-00006
Ahlawat SK, 2006, J PANCREAS, V7, P616
Ahmed HM, 2009, ENDOSCOPY, V41, pE218, DOI 10.1055/s-0029-1214941
Ahn JY, 2010, GUT LIVER, V4, P524, DOI 10.5009/gnl.2010.4.4.524
[Anonymous], GASTROENTEROL RES PR, DOI DOI 10.1155/2012/503098
Antillon MR, 2006, GASTROINTEST ENDOSC, V63, P797, DOI 10.1016/j.gie.2005.10.025
Artifon ELA, 2012, J CLIN GASTROENTEROL, V46, P768, DOI 10.1097/MCG.0b013e31825f264c
Ascunce G, 2011, GASTROINTEST ENDOSC, V73, P267, DOI 10.1016/j.gie.2010.10.029
Azar RR, 2006, GASTROINTEST ENDOSC, V63, P688, DOI 10.1016/j.gie.2005.10.032
Barkay O, 2010, GASTROINTEST ENDOSC, V71, P1166, DOI 10.1016/j.gie.2009.10.048
Baron TH, 2000, GASTROINTEST ENDOSC, V52, P545, DOI 10.1067/mge.2000.108928
Baron TH, 2002, GASTROINTEST ENDOSC, V56, P7, DOI 10.1067/mge.2002.125106
Barthet M, 2008, GASTROINTEST ENDOSC, V67, P245, DOI 10.1016/j.gie.2007.06.014
Binmoeller KF, 2013, SURG ENDOSC, V27, P1835, DOI 10.1007/s00464-012-2682-9
Bories E, 2007, ENDOSCOPY, V39, P287, DOI 10.1055/s-2007-966212
Brauer BC, 2009, GASTROINTEST ENDOSC, V70, P471, DOI 10.1016/j.gie.2008.12.233
Calvo MM, 2001, GASTROINTEST ENDOSC, V54, P511, DOI 10.1067/mge.2001.118441
Dhir V, 2012, GASTROINTEST ENDOSC, V75, P354, DOI 10.1016/j.gie.2011.07.075
Dietrich CF, 2011, ULTRASCHALL MED, V32, P8, DOI 10.1055/s-0029-1246017
Doi S, 2013, ENDOSCOPY, V45, P362, DOI 10.1055/s-0032-1326225
Ergun M, 2011, ENDOSCOPY, V43, P518, DOI 10.1055/s-0030-1256333
Fabbri C, 2012, ENDOSCOPY, V44, P429, DOI 10.1055/s-0031-1291624
Fabbri C, 2011, ENDOSCOPY, V43, P438, DOI 10.1055/s-0030-1256097
Fockens P, 1997, GASTROINTEST ENDOSC, V46, P412, DOI 10.1016/S0016-5107(97)70033-6
Frey CF, 1990, CHRONIC PANCREATITIS, P415
Fujii L, 2012, ENDOSCOPY, V44, pE265, DOI 10.1055/s-0032-1309708
Gimeno-García AZ, 2012, ENDOSCOPY, V44, pE267, DOI 10.1055/s-0032-1309709
Giovannini M, 2003, ENDOSCOPY, V35, P511
Giovannini M, 2001, ENDOSCOPY, V33, P898, DOI 10.1055/s-2001-17324
Gress F, 1999, AM J GASTROENTEROL, V94, P900
Gress F, 2001, AM J GASTROENTEROL, V96, P409
GRIMM H, 1992, GASTROINTEST ENDOSC, V38, P170, DOI 10.1016/S0016-5107(92)70384-8
Gunaratnam NT, 2001, GASTROINTEST ENDOSC, V54, P316, DOI 10.1067/mge.2001.117515
Gupta Ruchi, 2007, MedGenMed, V9, P8
Hara K, 2013, ENDOSCOPY, V45, P392, DOI 10.1055/s-0032-1326076
Hara K, 2011, AM J GASTROENTEROL, V106, P1239, DOI 10.1038/ajg.2011.84
Heinzow HS, 2011, SCAND J GASTROENTERO, V46, P1004, DOI 10.3109/00365521.2011.571709
Hookey LC, 2006, GASTROINTEST ENDOSC, V63, P635, DOI 10.1016/j.gie.2005.06.028
Horaguchi J, 2009, DIGEST ENDOSC, V21, P239, DOI 10.1111/j.1443-1661.2009.00899.x
Itoi T, 2008, WORLD J GASTROENTERO, V14, P6078, DOI 10.3748/wjg.14.6078
Itoi T, 2012, GASTROINTEST ENDOSC, V75, P870, DOI 10.1016/j.gie.2011.10.020

Iwashita T, 2012, ENDOSCOPY, V44, P60, DOI 10.1055/s-0030-1256871
Iwata K, 2011, DIGEST ENDOSC, V23, P140, DOI 10.1111/j.1443-1661.2010.01046.x
Jenssen C, 2008, Z GASTROENTEROL, V46, P1177, DOI 10.1055/s-2008-1027334
Jenssen C, 2012, WORLD J GASTROENTERO, V18, P4659, DOI 10.3748/wjg.v18.i34.4659
Kahaleh M, 2006, ENDOSCOPY, V38, P355, DOI 10.1055/s-2006-925249
Kahaleh M, 2007, GASTROINTEST ENDOSC, V64, P52
Kahaleh M, 2007, GASTROINTEST ENDOSC, V65, P224, DOI 10.1016/j.gie.2006.05.008
Kato S, 2013, DIAGN THER ENDOSC
Kawakubo K, 2013, SURG ENDOSC, V27, P3437, DOI 10.1007/s00464-013-2896-5
Khashab MA, 2012, SURG ENDOSC, V26, P3320, DOI 10.1007/s00464-012-2297-1
Kim TH, 2012, WORLD J GASTROENTERO, V18, P2526, DOI 10.3748/wjg.v18.i20.2526
Kim YS, 2010, ENDOSCOPY, V42, P496, DOI 10.1055/s-0029-1244082
Kiriluk S, 2012, AM J GASTROENTEROL, V107, pS48, DOI 10.14309/00000434-201210001-00113
Komaki T, 2011, PANCREATOLOGY, V11, P47, DOI 10.1159/000323508
Krüger M, 2006, GASTROINTEST ENDOSC, V63, P409, DOI 10.1016/j.gie.2005.11.047
Künzli HT, 2013, EUR J GASTROEN HEPAT, V25, P958, DOI 10.1097/MEG.0b013e3283612f03
Kurihara T, 2013, DIGEST ENDOSC, V25, P109, DOI 10.1111/den.12100
Kwon YM, 2013, SURG ENDOSC, V27, P2422, DOI 10.1007/s00464-012-2752-z
LAI ECS, 1992, NEW ENGL J MED, V326, P1582, DOI 10.1056/NEJM199206113262401
Lalueza A, 2011, ENDOSCOPY, V43, pE9, DOI 10.1055/s-0030-1255804
Leblanc J, 2008, GASTROINTEST ENDOSC, V67, pAB224, DOI 10.1016/j.gie.2008.03.560
LeBlanc JK, 2011, GASTROINTEST ENDOSC, V74, P1300, DOI 10.1016/j.gie.2011.07.073
Levy MJ, 2008, AM J GASTROENTEROL, V103, P98, DOI 10.1111/j.1572-0241.2007.01607.x
Loeve US, 2013, GASTROINTEST ENDOSC, V77, P151, DOI 10.1016/j.gie.2012.03.005
Lopes CV, 2007, SCAND J GASTROENTERO, V42, P524, DOI 10.1080/00365520601065093
Mahajan RJ, 2002, GASTROINTEST ENDOSC, V55, pAB101
Maluf F, 2012, J PANCREAS, V13, P210
Mangiavillano B, 2012, J DIGEST DIS, V13, P47, DOI 10.1111/j.1751-2980.2011.00547.x
Maranki J, 2009, ENDOSCOPY, V41, P532, DOI 10.1055/s-0029-1214712
MARKOWITZ JS, 1994, ARCH SURG-CHICAGO, V129, P374
Martins FP, 2010, ENDOSCOPY, V42, pE126, DOI 10.1055/s-0029-1243911
Mittal MK, 2012, NEUROLOGY, V78, pE57, DOI 10.1212/WNL.0b013e318248df51
Mönkemüller KE, 1998, GASTROINTEST ENDOSC, V48, P195, DOI 10.1016/S0016-5107(98)70164-6
Muscatiello N, 2006, ENDOSCOPY, V38, P858, DOI 10.1055/s-2006-925382
O'Toole TM, 2009, ENDOSCOPY, V41, P593, DOI 10.1055/s-0029-1214868
Panamonta N, 2012, EUR J GASTROEN HEPAT, V24, P1355, DOI 10.1097/MEG.0b013e32835871eb
Park DH, 2009, ENDOSCOPY, V41, P842, DOI 10.1055/s-0029-1215133
Park DH, 2013, GASTROINTEST ENDOSC, V78, P91, DOI 10.1016/j.gie.2013.01.042
Park DH, 2011, GASTROINTEST ENDOSC, V74, P1276, DOI 10.1016/j.gie.2011.07.054
Penn DE, 2012, GASTROINTEST ENDOSC, V76, P679, DOI 10.1016/j.gie.2012.04.457
Pfaffenbach B, 1998, DEUT MED WOCHENSCHR, V123, P1439, DOI 10.1055/s-2007-1024199
Puri R, 2012, J GASTROEN HEPATOL, V27, P722, DOI 10.1111/j.1440-1746.2012.07089.x

Puri R, 2010, J GASTROEN HEPATOL, V25, P1416, DOI 10.1111/j.1440-1746.2010.06328.x
Ramírez-Luna MA, 2011, ENDOSCOPY, V43, P826, DOI 10.1055/s-0030-1256406
Rasmussen DN, 2012, DAN MED J, V59
Rawl S, 2013, DIAGN THER ENDOSC
Sadik R, 2011, WORLD J GASTROENTERO, V17, P499, DOI 10.3748/wjg.v17.i4.499
Saftoiu A, 2006, ENDOSCOPY, V38, P538, DOI 10.1055/s-2006-925229
Sahai AV, 2009, AM J GASTROENTEROL, V104, P326, DOI 10.1038/ajg.2008.64
Sakamoto H, 2010, AM J GASTROENTEROL, V105, P2599, DOI 10.1038/ajg.2010.339
SALTZSTEIN EC, 1983, SURGERY, V94, P704
Santosh D, 2009, ALIMENT PHARM THER, V29, P979, DOI 10.1111/j.1365-2036.2009.03963.x
Seewald S, 2012, DIGEST ENDOSC, V24, P36, DOI 10.1111/j.1443-1661.2011.01162.x
Seicean A, 2013, J GASTROINTEST LIVER, V22, P59
Seicean A, 2011, J GASTROINTEST LIVER, V20, P39
Shah JN, 2012, GASTROINTEST ENDOSC, V75, P56, DOI 10.1016/j.gie.2011.08.032
Song TJ, 2012, WORLD J GASTROENTERO, V18, P4435, DOI 10.3748/wjg.v18.i32.4435
Stevens T, 2012, CLIN GASTROENTEROL H, V10, P186, DOI 10.1016/j.cgh.2011.09.006
Tessier G, 2007, GASTROINTEST ENDOSC, V65, P233, DOI 10.1016/j.gie.2006.06.029
van Delden OM, 2008, EUR RADIOL, V18, P448, DOI 10.1007/s00330-007-0796-6
Varadarajulu S, 2012, ENDOSCOPY, V44, pE92, DOI 10.1055/s-0030-1256946
Varadarajulu S, 2008, GASTROINTEST ENDOSC, V68, P1102, DOI 10.1016/j.gie.2008.04.028
Varadarajulu S, 2011, J GASTROEN HEPATOL, V26, P1504, DOI 10.1111/j.1440-1746.2011.06771.x
Varadarajulu S, 2009, GASTROINTEST ENDOSC, V70, P1260, DOI 10.1016/j.gie.2009.06.007
Varadarajulu S, 2009, GASTROINTEST ENDOSC, V70, P1121, DOI 10.1016/j.gie.2009.08.034
Vila JJ, 2012, GASTROINTEST ENDOSC, V76, P1133, DOI 10.1016/j.gie.2012.08.001
Voermans RP, 2011, GASTROINTEST ENDOSC, V74, P1285, DOI 10.1016/j.gie.2011.07.059
Vosoghi Mehrdad, 2002, MedGenMed, V4, P2
Weilert F, 2012, ENDOSCOPY, V44, P780, DOI 10.1055/s-0032-1309839
Wiersema MJ, 1996, GASTROINTEST ENDOSC, V44, P656, DOI 10.1016/S0016-5107(96)70047-0
Wiersema MJ, 1996, GASTROINTEST ENDOSC, V43, P102, DOI 10.1016/S0016-5107(06)80108-2
Will U, 2007, ENDOSCOPY, V39, P292, DOI 10.1055/s-2007-966215
Will U, 2011, ULTRASCHALL MED, V32, P176, DOI 10.1055/s-0031-1273347
Will U, 2007, J HEPATO-BILIARY-PAN, V14, P377, DOI 10.1007/s00534-006-1139-8
Wyse JM, 2011, J CLIN ONCOL, V29, P3541, DOI 10.1200/JCO.2010.32.2750
Yamao Kenji, 2012, Gastrointest Endosc Clin N Am, V22, P259, DOI 10.1016/j.giec.2012.04.008
Zou XP, 2012, PANCREAS, V41, P655, DOI 10.1097/MPA.0b013e31823d06a9
NR 118
TC 77
Z9 89
U1 0
U2 18
PU SPRINGER
PI NEW YORK

PA 233 SPRING ST, NEW YORK, NY 10013 USA
SN 0930-2794
EI 1432-2218
J9 SURG ENDOSC
JI Surg. Endosc.
PD MAR
PY 2014
VL 28
IS 3
BP 712
EP 734
DI 10.1007/s00464-013-3260-5
PG 23
WC Surgery
WE Science Citation Index Expanded (SCI-EXPANDED)
SC Surgery
GA AB7HX
UT WOS:000331961500003
PM 24196551
DA 2026-05-09
ER

PT J
AU Posner, H
Widmer, J
AF Posner, Hannah
Widmer, Jessica
TI EUS guided gallbladder drainage
SO TRANSLATIONAL GASTROENTEROLOGY AND HEPATOLOGY
LA English
DT Review
DE Acute cholecystitis; endoscopic gallbladder drainage; endoscopic
ultrasound guided gallbladder drainage
ID APPOSING METAL STENT; LONG-TERM OUTCOMES; HIGH-RISK PATIENTS; ACUTE
CHOLECYSTITIS; PERCUTANEOUS CHOLECYSTOSTOMY; PANCREATIC-CANCER;
EFFICACY; BILIARY; FEASIBILITY; THERAPY
AB Cholecystectomy is the gold standard treatment for acute cholecystitis, but it may not be appropriate for patients with significant comorbidities. Percutaneous gallbladder drainage (PT-GBD) and endoscopic transpapillary gallbladder drainage (ET-GBD) are alternatives with good technical and clinical success rates, but are limited by technical challenges and the need for definitive therapy. EUS-guided gallbladder drainage (EUS-GBD) is quickly becoming the preferred modality of treatment at expert centers in this cohort of patients due to increased efficacy and minimal adverse events. Technicalities of the procedure, including selection of access site, should be informed by the ultimate needs and anatomy of each patient.

With the evolution of new stents and accessories, including a cautery-enhanced lumen apposing metal stent deployment system, success rates and adverse events are favorable. A review of published case series demonstrates an overall clinical success rate of approximately 97% for EUS-GBD. The most common complication is pneumoperitoneum, so the evolution of self-expanding LAMS is promising. EUS-GBD has been successfully described in cases where definitive therapy or a bridge to cholecystectomy is needed. As the procedure's applications continue to evolve, there should be greater discussion about specific details including access site and stent selection.
C1 [Posner, Hannah; Widmer, Jessica] NYU, Div Gastroenterol Hepatol & Nutr, Winthrop Hosp, Mineola, NY USA.
C3 New York University
RP Widmer, J (corresponding author), NYU Winthrop Hosp, 259 First St, Mineola, NY 11501 USA.
EM jessica.widmer@nyulangone.org
OI Posner, Hannah/0000-0003-1323-1724
CR Ahmed O, 2018, SAUDI J GASTROENTERO, V24, P183, DOI 10.4103/sjg.SJG_506_17
Akhan O, 2002, EUR J RADIOL, V43, P229, DOI 10.1016/S0720-048X(02)00158-4
Al-Jundi W, 2012, ANN ROY COLL SURG, V94, P99, DOI 10.1308/003588412X13171221501302
Anderloni A, 2017, ENDOSC INT OPEN, V5, pE1100, DOI 10.1055/s-0043-118828
Ballard DD, 2018, ENDOSC INT OPEN, V6, pE714, DOI 10.1055/a-0599-6190
Baron TH, 2007, GASTROINTEST ENDOSC, V65, P735, DOI 10.1016/j.gie.2006.07.041
Súbtil JC, 2010, WORLD J GASTRO ENDOS, V2, P203, DOI 10.4253/wjge.v2.i6.203
Chantarojanasiri T, 2017, SAUDI J GASTROENTERO, V23, P218, DOI 10.4103/sjg.SJG_115_17
Choi JH, 2017, GASTROINTEST ENDOSC, V85, P357, DOI 10.1016/j.gie.2016.07.067
Choi JH, 2014, ENDOSCOPY, V46, P656, DOI 10.1055/s-0034-1365720
de la Serna-Higuera C, 2013, GASTROINTEST ENDOSC, V77, P303, DOI 10.1016/j.gie.2012.09.021
Dollhopf M, 2017, GASTROINTEST ENDOSC, V86, P636, DOI 10.1016/j.gie.2017.02.027
Gan SI, 2007, GASTROINTEST ENDOSC, V66, P425, DOI 10.1016/j.gie.2007.05.026
Ge N, 2016, BMC GASTROENTEROL, V16, DOI 10.1186/s12876-016-0420-9
Hasan Muhammad K, 2013, Gastrointest Endosc Clin N Am, V23, P453, DOI 10.1016/j.giec.2012.12.010
Iino C, 2018, ENDOSC INT OPEN, V6, pE594, DOI 10.1055/s-0044-102091
Imai H, 2016, GASTROINTEST ENDOSC, V84, P147, DOI 10.1016/j.gie.2015.12.024
Irani S, 2017, CLIN GASTROENTEROL H, V15, P738, DOI 10.1016/j.cgh.2016.12.021
Irani S, 2015, GASTROINTEST ENDOSC, V82, P1110, DOI 10.1016/j.gie.2015.05.045
Itoi T, 2008, GASTROINTEST ENDOSC, V68, P455, DOI 10.1016/j.gie.2008.02.052
Itoi T, 2018, ENDOSC ULTRASOUND, V7, P76, DOI 10.4103/eus.eus_4_18
Itoi T, 2014, GASTROINTEST ENDOSC, V79, P547, DOI 10.1016/j.gie.2013.12.010
Itoi T, 2013, DIGEST ENDOSC, V25, P137, DOI 10.1111/den.12084
Itoi T, 2012, GASTROINTEST ENDOSC, V75, P870, DOI 10.1016/j.gie.2011.10.020
Itoi T, 2011, J HEPATO-BIL-PAN SCI, V18, P282, DOI 10.1007/s00534-010-0310-4
Itoi T, 2010, GASTROINTEST ENDOSC, V71, P1038, DOI 10.1016/j.gie.2010.01.026
Jain D, 2018, CLIN ENDOSC, V51, P450, DOI 10.5946/ce.2018.024
James TW, 2018, ENDOSC ULTRASOUND, V7, P93, DOI 10.4103/eus.eus_110_17
Jamwal KD, 2018, J CLIN TRANSL HEPATO, V6, P35, DOI 10.14218/JCTH.2017.00018
Jang JW, 2012, GASTROENTEROLOGY, V142, P805, DOI 10.1053/j.gastro.2011.12.051
Jang JW, 2011, GASTROINTEST ENDOSC, V74, P176, DOI 10.1016/j.gie.2011.03.1120

Kalva NR, 2018, CAN J GASTROENTEROL, V2018, DOI 10.1155/2018/7070961
Kamata K, 2009, ENDOSCOPY, V41, pE315, DOI 10.1055/s-0029-1215258
Kamata K, 2017, WORLD J GASTROENTERO, V23, P661, DOI 10.3748/wjg.v23.i4.661
Khan MA, 2017, GASTROINTEST ENDOSC, V85, P76, DOI 10.1016/j.gie.2016.06.032
Kumta NA, 2016, ENDOSCOPY, V48, pE327, DOI 10.1055/s-0042-117634
Kwan V, 2007, GASTROINTEST ENDOSC, V66, P582, DOI 10.1016/j.gie.2007.02.065
Lang GD, 2018, GASTROINTEST ENDOSC, V87, P150, DOI 10.1016/j.gie.2017.06.029
Law R, 2016, CLIN GASTROENTEROL H, V14, P476, DOI 10.1016/j.cgh.2015.10.026
Lee SS, 2007, GASTROINTEST ENDOSC, V66, P1008, DOI 10.1016/j.gie.2007.03.1080
Manta R, 2018, EUR J GASTROEN HEPAT, V30, P695, DOI 10.1097/MEG.0000000000001112
Manta R, 2017, ENDOSC INT OPEN, V5, pE1111, DOI 10.1055/s-0043-118659
MCGAHAN JP, 1989, RADIOLOGY, V173, P481, DOI 10.1148/radiology.173.2.2678261
Minaga K, 2019, DIGEST ENDOSC, V31, P180, DOI 10.1111/den.13242
Mönkemüller K, 2013, ENDOSCOPY, V45, pE164, DOI 10.1055/s-0032-1325776
Moon JH, 2014, GASTROINTEST ENDOSC, V79, P990, DOI 10.1016/j.gie.2014.02.015
Ogura T, 2014, ENDOSCOPY, V46, pE75, DOI 10.1055/s-0033-1359135
Peñas-Herrero I, 2015, J HEPATO-BIL-PAN SCI, V22, P35, DOI 10.1002/jhbp.182
Perez-Miranda M, 2018, ENDOSC ULTRASOUND, V7, P79, DOI 10.4103/eus.eus_5_18
Saumoy M, 2019, J CLIN GASTROENTEROL, V53, P231, DOI 10.1097/MCG.0000000000001036
Saumoy M, 2018, ENDOSC ULTRASOUND, V7, P97, DOI 10.4103/eus.eus_9_18
Saumoy M, 2017, GASTROINTEST ENDOSC, V85, P1253, DOI 10.1016/j.gie.2017.03.008
Sharaiha R, 2013, GASTROINTEST ENDOSC, V78, P638, DOI 10.1016/j.gie.2013.05.034
Small AJ, 2018, ENDOSC ULTRASOUND, V7, P89, DOI 10.4103/eus.eus_8_18
Song TJ, 2010, GASTROINTEST ENDOSC, V71, P634, DOI 10.1016/j.gie.2009.11.024
Takagi W, 2016, THER ADV GASTROENTER, V9, P19, DOI 10.1177/1756283X15609285
Takasawa O, 2009, DIGEST ENDOSC, V21, P43, DOI 10.1111/j.1443-1661.2008.00822.x
Teoh AYB, 2017, SAUDI J GASTROENTERO, V23, P209, DOI 10.4103/sjg.SJG_436_17
Teoh AYB, 2017, ENDOSCOPY, V49, P130, DOI 10.1055/s-0042-119036
Teoh AYB, 2014, GASTROINTEST ENDOSC, V80, P1171, DOI 10.1016/j.gie.2014.03.038
Tharian B, 2016, GASTROINTEST ENDOSC, V83, P460, DOI 10.1016/j.gie.2015.08.016
Tyberg A, 2018, J CLIN GASTROENTEROL, V52, P79, DOI 10.1097/MCG.0000000000000786
Walter D, 2016, GUT, V65, P6, DOI 10.1136/gutjnl-2015-309925
Widmer J, 2014, DIGEST ENDOSC, V26, P525, DOI 10.1111/den.12221
Widmer J, 2014, DIGEST ENDOSC, V26, P599, DOI 10.1111/den.12163
Winbladh A, 2009, HPB, V11, P183, DOI 10.1111/j.1477-2574.2009.00052.x
NR 66
TC 19
Z9 19
U1 0
U2 9
PU AME PUBLISHING COMPANY
PI SHATIN

PA FLAT-RM C 16F, KINGS WING PLAZA 1, NO 3 KWAN ST, SHATIN, HONG KONG
00000, PEOPLES R CHINA
EI 2415-1289
J9 TRANSL GASTROENT HEP
JI Transl. Gastroenterol. Hepatol.
PD JUL
PY 2020
VL 5
AR 41
DI 10.21037/tgh.2019.12.20
PG 11
WC Gastroenterology & Hepatology
WE Emerging Sources Citation Index (ESCI)
SC Gastroenterology & Hepatology
GA KN5CJ
UT WOS:000514854600010
PM 32632392
OA Green Submitted, gold
DA 2026-05-09
ER

PT J
AU Han, SM
Papachristou, GI
AF Han, Samuel
Papachristou, Georgios I.
TI Therapeutic Endoscopic Ultrasound for Complications of Pancreatic Cancer
SO CANCERS
LA English
DT Review
DE pancreatic cancer; choledochoduodenostomy; endoscopic ultrasound;
hepaticogastrostomy; biliary obstruction; gastric outlet obstruction
ID CELIAC PLEXUS NEUROLYSIS; EUS-GUIDED GASTROENTEROSTOMY; GASTRIC OUTLET
OBSTRUCTION; DISTAL BILIARY OBSTRUCTION; METAL STENT; INTERNATIONAL
MULTICENTER; PERCUTANEOUS DRAINAGE; GALLBLADDER DRAINAGE; FAILED ERCP;
CHOLEDOCHODUODENOSTOMY
AB Simple Summary Complications of pancreatic cancer include the development of blockage of the bile duct preventing drainage of the liver, blockage of the stomach and duodenum preventing the passage of food and increase in pain levels. Endoscopic ultrasound is a procedure performed through the mouth where a flexible tube with a camera and an ultrasound probe attached allow for visualization of organs around the stomach and small intestine, such as the pancreas, the bile duct, and the liver. It was originally designed to help diagnose pancreatic cancer, but with advances in technology, it can now be used to treat the aforementioned complications without performing surgery. This paper reviews the new advances in

technologies and techniques performed through endoscopic ultrasound with an emphasis on clinical trials that have been done to provide an overview of the safety and effectiveness of these procedures.Abstract Progression of pancreatic adenocarcinoma can result in disease complications such as biliary obstruction and gastric outlet obstruction. The recent advances in endoscopic ultrasound (EUS) have transformed EUS from a purely diagnostic technology to a therapeutic modality, particularly with the development of lumen-apposing metal stents. In terms of biliary drainage, EUS-guided choledochoduodenostomy and EUS-guided hepaticogastrostomy offer safe and effective techniques when conventional transpapillary stent placement via ERCP fails or is not possible. If these modalities are not feasible, EUS-guided gallbladder drainage offers yet another salvage technique when the cystic duct is non-involved by the cancer. Lastly, EUS-guided gastroenterostomy allows for an effective bypass treatment for cases of gastric outlet obstruction that enables patients to resume eating within several days. Future randomized studies comparing these techniques to current standard-of-care options are warranted to firmly establish therapeutic EUS procedures within the treatment algorithm for this challenging disease.
C1 [Han, Samuel; Papachristou, Georgios I.] Ohio State Univ, Div Gastroenterol Hepatol & Nutr, Wexner Med Ctr, Columbus, OH 43210 USA.
C3 University System of Ohio; Ohio State University
RP Papachristou, GI (corresponding author), Ohio State Univ, Div Gastroenterol Hepatol & Nutr, Wexner Med Ctr, Columbus, OH 43210 USA.
EM samuel.han@osumc.edu; georgios.papachristou@osumc.edu
RI Papachristou, Georgios/AAN-7619-2021
CR Bang JY, 2019, GASTROINTEST ENDOSC, V89, P58, DOI 10.1016/j.gie.2018.08.005
Bang JY, 2018, GASTROINTEST ENDOSC, V88, P9, DOI 10.1016/j.gie.2018.03.012
Binda C, 2023, GASTROINTEST ENDOSC, V98, P765, DOI 10.1016/j.gie.2023.06.054
Bray F, 2018, CA-CANCER J CLIN, V68, P394, DOI [10.3322/caac.21609, 10.3322/caac.21492]
Bronswijk M, 2023, GASTROINTEST ENDOSC, V98, DOI 10.1016/j.gie.2023.03.019
Bronswijk M, 2021, GASTROINTEST ENDOSC, V94, P526, DOI 10.1016/j.gie.2021.04.006
Canakis A, 2022, J HEPATO-BIL-PAN SCI, V29, P941, DOI 10.1002/jhbp.1055
Chandan S, 2021, ENDOSC INT OPEN, V09, pE496, DOI 10.1055/a-1341-0788
Chen YI, 2023, GASTROENTEROLOGY, V165, P1249, DOI 10.1053/j.gastro.2023.07.024
Conrad C, 2012, CANCER J, V18, P577, DOI 10.1097/PPO.0b013e3182797dfe
Crinò SF, 2023, CLIN GASTROENTEROL H, V21, P2834, DOI 10.1016/j.cgh.2023.02.022
Crinò SF, 2018, J GASTROINTEST LIVER, V27, P67, DOI 10.15403/jgld.2014.1121.271.eus
De Bie C, 2022, SURG ENDOSC, V36, P2393, DOI 10.1007/s00464-021-08520-z
Doi S, 2013, ENDOSCOPY, V45, P362, DOI 10.1055/s-0032-1326225
Facciorusso A, 2022, CANCERS, V14, DOI 10.3390/cancers14133291
Faraoni EY, 2023, CANCER IMMUNOL RES, V11, P4, DOI 10.1158/2326-6066.CIR-22-0379
Fegrachi S, 2019, EJSO-EUR J SURG ONC, V45, P2166, DOI 10.1016/j.ejso.2019.06.008
Ferreira MF, 2022, ENDOSC INT OPEN, V10, pE1380, DOI 10.1055/a-1922-4536
Fritzsche J, 2023, GASTROINTEST ENDOSC, V97, pAB864
Fugazza A, 2022, GASTROINTEST ENDOSC, V95, P896, DOI 10.1016/j.gie.2021.12.032
Garcia-Alonso FJ, 2023, GASTROINTEST ENDOSC, V98, P28, DOI 10.1016/j.gie.2023.02.015
Ginestet C, 2022, SURG ENDOSC, V36, P3365, DOI 10.1007/s00464-021-08653-1
Gutierrez OIB, 2018, ENDOSCOPY, V50, P891, DOI 10.1055/s-0044-102254

Han S, 2021, ENDOSC INT OPEN, V09, pE853, DOI 10.1055/a-1381-7301
Honjo M, 2018, ENDOSC ULTRASOUND, V7, P376, DOI 10.4103/eus.eus_2_18
Imai H, 2016, GASTROINTEST ENDOSC, V84, P147, DOI 10.1016/j.gie.2015.12.024
Inoue T, 2023, DIGEST DIS SCI, V68, P2090, DOI 10.1007/s10620-022-07749-5
Issa D, 2021, ENDOSCOPY, V53, P827, DOI 10.1055/a-1259-0349
Itonaga M, 2022, GASTROINTEST ENDOSC, V96, P57, DOI 10.1016/j.gie.2022.02.005
Iwashita T, 2022, J HEPATO-BIL-PAN SCI, V29, P810, DOI 10.1002/jhbp.1021
Jacques J, 2019, ENDOSCOPY, V51, P540, DOI 10.1055/a-0735-9137
Janet J, 2023, ANN SURG ONCOL, V30, P5036, DOI 10.1245/s10434-023-13466-8
Jung JH, 2021, SURG ENDOSC, V35, P2679, DOI 10.1007/s00464-020-07694-2
Kanno Y, 2020, GASTROINTEST ENDOSC, V92, P120, DOI 10.1016/j.gie.2020.01.011
Khashab MA, 2016, ENDOSC INT OPEN, V4, pE487, DOI 10.1055/s-0042-102648
Khashab MA, 2016, ENDOSC INT OPEN, V4, pE175, DOI 10.1055/s-0041-109083
Kloek JJ, 2010, J CLIN GASTROENTEROL, V44, P646, DOI 10.1097/MCG.0b013e3181ce5b36
Koulouris AI, 2021, PANCREATOLOGY, V21, P434, DOI 10.1016/j.pan.2020.12.016
Kumar A, 2022, ENDOSC INT OPEN, V10, pE448, DOI 10.1055/a-1765-4035
Law RJ, 2021, GASTROINTEST ENDOSC, V94, P457, DOI 10.1016/j.gie.2021.05.020
LeBlanc JK, 2011, GASTROINTEST ENDOSC, V74, P1300, DOI 10.1016/j.gie.2011.07.073
Lee TH, 2016, CLIN GASTROENTEROL H, V14, P1011, DOI 10.1016/j.cgh.2015.12.032
Levy MJ, 2019, CLIN GASTROENTEROL H, V17, P728, DOI 10.1016/j.cgh.2018.08.040
Mangiavillano B, 2024, DIGEST ENDOSC, V36, P351, DOI 10.1111/den.14606
Mangiavillano B, 2022, GASTROINTEST ENDOSC, V95, P115, DOI 10.1016/j.gie.2021.07.021
Nakai Y, 2020, GASTROINTEST ENDOSC, V92, P623, DOI 10.1016/j.gie.2020.03.3856
Ogura T, 2021, DEN OPEN, V1, DOI 10.1002/deo2.8
Ogura T, 2018, DIGEST ENDOSC, V30, P252, DOI 10.1111/den.12976
Ogura T, 2016, ENDOSCOPY, V48, P156, DOI 10.1055/s-0034-1392859
Paik WH, 2018, AM J GASTROENTEROL, V113, P987, DOI 10.1038/s41395-018-0122-8
Park DH, 2011, GASTROINTEST ENDOSC, V74, P1276, DOI 10.1016/j.gie.2011.07.054
Park JK, 2018, GASTROINTEST ENDOSC, V88, P277, DOI 10.1016/j.gie.2018.03.015
Pérez-Cuadrado-Robles E, 2022, DIGEST ENDOSC, V34, P1433, DOI 10.1111/den.14330
Robles-Medranda C, 2023, GASTROINTEST ENDOSC, V97, P445, DOI 10.1016/j.gie.2022.10.037
Sánchez-Aldehuelo R, 2022, GASTROINTEST ENDOSC, V96, P1012, DOI 10.1016/j.gie.2022.07.018
Sawas T, 2022, ENDOSC ULTRASOUND, V11, P223, DOI 10.4103/EUS-D-21-00031
Scopelliti F, 2018, SURG ENDOSC, V32, P4022, DOI 10.1007/s00464-018-6217-x
Seo DW, 2019, GASTROINTEST ENDOSC, V90, P602, DOI 10.1016/j.gie.2019.06.032
Shibuki T, 2023, CLIN ENDOSC, V56, P802, DOI 10.5946/ce.2022.211
Suk KT, 2006, GASTROINTEST ENDOSC, V64, P522, DOI 10.1016/j.gie.2006.06.022
Teoh AYB, 2020, GUT, V69, P1085, DOI 10.1136/gutjnl-2019-319996
Teoh AYB, 2024, LANCET GASTROENTEROL, V9, P124, DOI 10.1016/S2468-1253(23)00242-X
Teoh AYB, 2023, GASTROENTEROLOGY, V165, P473, DOI 10.1053/j.gastro.2023.04.016
Thosani N, 2022, SCI REP-UK, V12, DOI 10.1038/s41598-022-20316-2
Tyberg Amy, 2023, J Clin Gastroenterol, V57, P962, DOI 10.1097/MCG.0000000000001795

Vanella G, 2023, GASTROINTEST ENDOSC, V98, DOI 10.1016/j.gie.2023.04.2072
Vanella G, 2023, DEN OPEN, V3, DOI 10.1002/deo2.132
Vanella G, 2022, ENDOSCOPY, V54, pE143, DOI 10.1055/a-1408-1180
Wyse JM, 2011, J CLIN ONCOL, V29, P3541, DOI 10.1200/JCO.2010.32.2750
Zakaria A, 2022, CANCERS, V14, DOI 10.3390/cancers14061373
NR 70
TC 4
Z9 5
U1 0
U2 6
PU MDPI
PI BASEL
PA MDPI AG, Grosspeteranlage 5, CH-4052 BASEL, SWITZERLAND
EI 2072-6694
J9 CANCERS
JI Cancers
PD JAN
PY 2024
VL 16
IS 1
AR 29
DI 10.3390/cancers16010029
PG 14
WC Oncology
WE Science Citation Index Expanded (SCI-EXPANDED)
SC Oncology
GA EL8P7
UT WOS:001139177100001
PM 38201458
OA Green Submitted, gold
DA 2026-05-09
ER

PT J
AU Altonbary, AY
Galal, A
El-Nady, M
Hakim, H
AF Altonbary, Ahmed Youssef
Galal, Ahmed
El-Nady, Mohamed
Hakim, Hazem

TI Endoscopic ultrasound guided biliary drainage: a multicenter
retrospective experience of a technique slowly gaining acceptance in
Egypt

SO THERAPEUTIC ADVANCES IN GASTROINTESTINAL ENDOSCOPY

LA English

DT Article

DE biliary drainage; Egyptian experience; endoscopic ultrasound

ID OBSTRUCTION; CHOLEDOCHODUODENOSTOMY; ERCP; HEPATICOGASTROSTOMY;
PLACEMENT

AB Background and aim: Endoscopic ultrasound-guided biliary drainage is an alternative to failed endoscopic retrograde cholangiopancreatography. Unfortunately, this procedure remains relatively less explored in Egypt due to its high cost, lack of adequate training, and the perception of increased risk. This study is the first multicenter Egyptian experience of an endoscopic ultrasound-guided biliary drainage in patients with malignant biliary obstruction. Patients and methods: We retrospectively reviewed 15 patients (10 men and five women) with malignant biliary obstruction who from October 2013 to May 2019, following a failed or inaccessible endoscopic retrograde cholangiopancreatography, underwent an endoscopic ultrasound-guided choledochoduodenostomy, endoscopic ultrasound-guided hepaticogastrostomy, or endoscopic ultrasound-guided rendezvous. Their mean age was 57.4 years and mean bilirubin was 18.2 mg/dL. The outcome parameters included technical and clinical success. Technical success was defined as the successful placement of a stent in the biliary system, while clinical success was defined as a greater than 50% decrease in the bilirubin levels 2 weeks after the procedure. Patients were monitored for complications during and after the procedure. Results: In total, 15 patients underwent endoscopic ultrasound-guided biliary drainage (eight underwent endoscopic ultrasound-guided choledochoduodenostomy, five underwent endoscopic ultrasound-guided hepaticogastrostomy, and two underwent endoscopic ultrasound-guided rendezvous). The technical and clinical success rates were 100% (15/15 patients) and 93.3% (14/15 patients), respectively. The complication rate was 26.6% (4/15 patients). All complications were mild and self-limited, and included fever, mild biliary peritonitis, pneumoperitoneum, and a slight migration of one plastic stent during insertion. Conclusion: Although slowly gaining acceptance in Egypt, endoscopic ultrasound-guided biliary drainage is an effective and safe procedure in patients with a malignant biliary obstruction after a failed or inaccessible endoscopic retrograde cholangiopancreatography.

C1 [Altonbary, Ahmed Youssef; Hakim, Hazem] Mansoura Univ, Mansoura Specialized Med Hosp, Dept Gastroenterol & Hepatol, Mansoura 35516, Egypt.
[Galal, Ahmed] Alexandria Gastroenterol Hepatol & Fever Hosp, Dept Gastroenterol & Hepatol, Alexandria, Egypt.
[El-Nady, Mohamed] Cairo Univ, Dept Gastroenterol & Hepatol, Kasr Al Aini Hosp, Cairo, Egypt.

C3 Egyptian Knowledge Bank (EKB); Mansoura University; Egyptian Knowledge
Bank (EKB); Cairo University

RP Altonbary, AY (corresponding author), Mansoura Univ, Mansoura Specialized Med Hosp, Dept Gastroenterol & Hepatol, Mansoura 35516, Egypt.

EM a.tonbary@gmail.com

RI Hakim, Hazem/AAJ-4849-2021; Altonbary, Ahmed/ABG-3828-2020; Galal,
Ahmed/LHA-3466-2024

OI Hakim, Hazem/0000-0001-5324-7670; Altonbary, Ahmed/0000-0001-8850-9829;

CR Altonbary AY, 2014, ENDOSC ULTRASOUND, V3, P137, DOI 10.4103/2303-9027.131043
Artifon ELA, 2015, GASTROINTEST ENDOSC, V81, P950, DOI 10.1016/j.gie.2014.09.047
Bapaye A, 2013, UNITED EUR GASTROENT, V1, P285, DOI 10.1177/2050640613490928

Cotton PB, 2010, GASTROINTEST ENDOSC, V71, P446, DOI 10.1016/j.gie.2009.10.027
Dhir V, 2015, GASTROINTEST ENDOSC, V81, P913, DOI 10.1016/j.gie.2014.09.054
Dhir V, 2014, DIGEST ENDOSC, V26, P430, DOI 10.1111/den.12153
Enochsson L, 2010, GASTROINTEST ENDOSC, V72, P1175, DOI 10.1016/j.gie.2010.07.047
FERRUCCI JT, 1980, RADIOLOGY, V135, P1
Giovannini M, 2001, ENDOSCOPY, V33, P898, DOI 10.1055/s-2001-17324
Ikeuchi N., 2015, GASTROINTEST INTERV, V4, P31, DOI [10.1016/j.gii.2015.04.002, 10.1016/j.gii.2015.04.002, DOI 10.1016/J.GII.2015.04.002]
Khashab MA, 2016, ENDOSC INT OPEN, V4, pE487, DOI 10.1055/s-0042-102648
Khashab MA, 2015, DIGEST DIS SCI, V60, P557, DOI 10.1007/s10620-014-3300-6
Minaga K, 2018, DIGEST ENDOSC, V30, P38, DOI 10.1111/den.12910
Nennstiel S, 2015, J CLIN GASTROENTEROL, V49, P764, DOI 10.1097/MCG.0000000000000275
Sharaiha RZ, 2017, GASTROINTEST ENDOSC, V85, P904, DOI 10.1016/j.gie.2016.12.023
Sharaiha RZ, 2016, SURG ENDOSC, V30, P5500, DOI 10.1007/s00464-016-4913-y
Singla V, 2018, J DIG ENDOSC, V9, P6, DOI 10.4103/jde.JDE_49_17
Uemura RS, 2018, J CLIN GASTROENTEROL, V52, P123, DOI 10.1097/MCG.0000000000000948
Wang KX, 2016, GASTROINTEST ENDOSC, V83, P1218, DOI 10.1016/j.gie.2015.10.033
Wiersema MJ, 1996, GASTROINTEST ENDOSC, V43, P102, DOI 10.1016/S0016-5107(06)80108-2
NR 20
TC 5
Z9 5
U1 0
U2 0
PU SAGE PUBLICATIONS LTD
PI LONDON
PA 1 OLIVERS YARD, 55 CITY ROAD, LONDON EC1Y 1SP, ENGLAND
SN 2631-7745
J9 THER ADV GASTROINTES
JI Ther. Adv. Gastrointest. Endosc.
PD DEC
PY 2019
VL 12
AR 2631774519889456
DI 10.1177/2631774519889456
PG 9
WC Gastroenterology & Hepatology
WE Emerging Sources Citation Index (ESCI)
SC Gastroenterology & Hepatology
GA JV4VM
UT WOS:000502363300001
PM 31853522
OA Green Submitted, gold

DA 2026-05-09
ER

PT J
AU Rana, SS
Shah, J
Bhujade, H
Gorsi, U
Kang, M
Gupta, R
AF Rana, Surinder Singh
Shah, Jimil
Bhujade, Harish
Gorsi, Ujjwal
Kang, Mandeep
Gupta, Rajesh
TI Prevention of Serious Complications during Endoscopic Ultrasound-Guided
Biliary Drainage: A Case-Based Technical Review
SO JOURNAL OF DIGESTIVE ENDOSCOPY
LA English
DT Review
DE endoscopic ultrasound; stent; biliary drainage; endoscopic retrograde
cholangio-pancreatography
ID METAL STENT PLACEMENT; RISK-FACTORS; CHOLECYSTITIS; OBSTRUCTION;
MANAGEMENT; HEPATICOGASTROSTOMY; EXPERIENCE; EFFICACY; SAFETY
AB Endoscopic ultrasound-guided biliary drainage (EUS-BD) has a potential risk of clinically significant adverse events including fatal complications. Learning from complications improves the results from interventional procedures especially the high-risk procedure like EUS-BD. The various complications that have been reported following EUS-BD include bile leak, bleeding, cholangitis, peritonitis, stent migration both internal and external as well as in the peritoneal cavity and fatal perforations. In this technical review, we discuss technical strategies to prevent serious adverse events during EUS-BD using a case based approach.
C1 [Rana, Surinder Singh; Shah, Jimil] Postgrad Inst Med Educ & Res PGIMER, Dept Gastroenterol, Chandigarh 160012, India.
[Bhujade, Harish; Gorsi, Ujjwal; Kang, Mandeep] Postgrad Inst Med Educ & Res PGIMER, Dept Radiodiag, Chandigarh, India.
[Gupta, Rajesh] Postgrad Inst Med Educ & Res PGIMER, Dept Surg Gastroenterol, Chandigarh, India.
C3 Post Graduate Institute of Medical Education & Research (PGIMER),
Chandigarh; Post Graduate Institute of Medical Education & Research
(PGIMER), Chandigarh; Post Graduate Institute of Medical Education &
Research (PGIMER), Chandigarh
RP Rana, SS (corresponding author), Postgrad Inst Med Educ & Res PGIMER, Dept Gastroenterol, Chandigarh 160012, India.

EM drsurinderrana@gmail.com

RI Bhujade, Harish/ABC-6422-2021

CR Amato A, 2021, ENDOSCOPY, V53, P1037, DOI 10.1055/a-1324-7919

Baars JE, 2018, ENDOSC ULTRASOUND, V7, P4, DOI 10.4103/eus.eus_105_17

Chantarojanasiri T, 2021, CLIN ENDOSC, V54, P301

Fidelman N, 2008, RADIOLOGY, V247, P880, DOI 10.1148/radiol.2473070529

Gosain S, 2010, DIGEST DIS SCI, V55, P2406, DOI 10.1007/s10620-009-1024-9

Hatamaru K, 2019, ENDOSC ULTRASOUND, V8, pS67, DOI 10.4103/eus.eus_60_19

Honjo M, 2018, ENDOSC ULTRASOUND, V7, P376, DOI 10.4103/eus.eus_2_18

Isayama H, 2006, CLIN GASTROENTEROL H, V4, P1148, DOI 10.1016/j.cgh.2006.06.004

Isayama Hiroyuki, 2017, International Journal of Gastrointestinal Intervention, V6, P114, DOI 10.18528/gii160012

Itoi T, 2011, J HEPATO-BIL-PAN SCI, V18, P664, DOI 10.1007/s00534-011-0410-9

Kahaleh M, 2005, GASTROINTEST ENDOSC, V61, P528, DOI 10.1016/S0016-5107(04)02593-3

Kakked G, 2020, ENDOSC ULTRASOUND, V9, P298, DOI 10.4103/eus.eus_10_20

Kanno Y, 2019, J GASTROINTEST CANC, V50, P469, DOI 10.1007/s12029-018-0096-1

Khan MA, 2016, DIGEST DIS SCI, V61, P684, DOI 10.1007/s10620-015-3933-0

Kim GH, 2019, CLIN ENDOSC, V52, P598, DOI 10.5946/ce.2018.177

Liu YS, 2018, ABDOM RADIOL, V43, P713, DOI 10.1007/s00261-017-1258-5

Lyu YX, 2021, DIGEST LIVER DIS, V53, P1247, DOI 10.1016/j.dld.2021.03.029

Nakai Y, 2019, ENDOSC ULTRASOUND, V8, P111, DOI 10.4103/eus.eus_17_18

Nakai Y, 2014, J GASTROEN HEPATOL, V29, P1557, DOI 10.1111/jgh.12582

Ogura T, 2021, ENDOSC ULTRASOUND, V10, P355, DOI 10.4103/EUS-D-20-00206

Ogura T, 2021, CLIN ENDOSC, V54, P651, DOI 10.5946/ce.2021.020-KDDW

Ogura T, 2021, DIGEST DIS, V39, P165, DOI 10.1159/000510560

Ogura T, 2021, GUT LIVER, V15, P196

Patel IJ, 2012, J VASC INTERV RADIOL, V23, P727, DOI 10.1016/j.jvir.2012.02.012

Poincloux L, 2015, ENDOSCOPY, V47, P794, DOI [10.1055/s-0035-1545399, 10.1055/s-0034-1391988]

Quencer KB, 2018, J CLIN MED, V7, DOI 10.3390/jcm7050094

Rai P, 2018, ENDOSC INT OPEN, V6, pE67, DOI 10.1055/s-0043-120664

Saad WEA, 2008, TECH VASC INTERV RAD, V11, P60, DOI 10.1053/j.tvir.2008.05.007

Saxena P, 2013, GASTROINTEST ENDOSC, V78, P175, DOI 10.1016/j.gie.2013.02.038

Suk KT, 2006, GASTROINTEST ENDOSC, V64, P522, DOI 10.1016/j.gie.2006.06.022

Takaoka M, 2020, JGH OPEN, V4, P1140, DOI 10.1002/jgh3.12409

Ting PH, 2020, J CHIN MED ASSOC, V83, P733, DOI 10.1097/JCMA.0000000000000383

Weber A, 2009, EUR J RADIOL, V72, P412, DOI 10.1016/j.ejrad.2008.08.012

Yokota Y, 2017, BMC GASTROENTEROL, V17, DOI 10.1186/s12876-017-0662-1

NR 34

TC 4

Z9 5

U1 0

U2 8

PU THIEME MEDICAL PUBL INC

PI NEW YORK
PA 333 SEVENTH AVE, NEW YORK, NY 10001 USA
SN 0976-5042
EI 0976-5050
J9 J DIG ENDOSC
JI J. Dig. Endosc.
PD JUN
PY 2022
VL 13
IS 02
BP 82
EP 88
DI 10.1055/s-0042-1743519
PG 7
WC Gastroenterology & Hepatology
WE Emerging Sources Citation Index (ESCI)
SC Gastroenterology & Hepatology
GA 2T6YK
UT WOS:000822617400004
OA Green Submitted, gold
DA 2026-05-09
ER

PT J
AU Parihar, V
Ridgway, PF
Conlon, KC
Huggett, M
Ryan, BM
AF Parihar, Vikrant
Ridgway, Paul F.
Conlon, Kevin C.
Huggett, Matthew
Ryan, Barbara M.
TI The role of endoscopic intervention in the management of inflammatory pancreatic fluid collections
SO EUROPEAN JOURNAL OF GASTROENTEROLOGY & HEPATOLOGY
LA English
DT Review
DE double-pigtail stents; endoscopic necrosectomy; endoscopic ultrasound-guided drainage; endoscopic ultrasound; lumen apposing metal stents; pancreatic fluid collections

ID EUS-GUIDED DRAINAGE; EXPANDING METAL STENTS; TRANSMURAL DRAINAGE; PSEUDOCYST DRAINAGE; CLINICAL-EVALUATION; TREATMENT OUTCOMES; NATURAL-HISTORY; PLASTIC STENTS; EFFICACY; NECROSIS

AB Pancreatic fluid collections (PFCs) are a frequent complication of pancreatitis, or less commonly, pancreatic trauma or surgery. The revised Atlanta Classification categorizes PFCs as acute or chronic, with further subclassification of acute collections into acute peripancreatic collections and acute necrotic collections and of chronic fluid collections into pseudocysts and walled-off pancreatic necrosis. Acute PFCs are generally only subjected to an intervention when they are infected and not responding to antibiotics and are not managed endoscopically. Chronic PFCs, both pseudocysts and walled-off pancreatic necrosis, require intervention only when symptomatic or enlarging over time. Endoscopic ultrasound-guided drainage has become the mainstay of management for chronic PFCs that require intervention. Developments in medical devices over the past few years have significantly simplified and shortened the duration of the procedure itself, but the optimum choice of stent in different clinical scenarios remains to be defined, as does the place of endoscopic necrosectomy. To optimize outcomes, these patients should undergo a careful preprocedure workup and discussion in a multidisciplinary environment and procedures should be carried out in high-volume pancreatic units. Copyright (C) 2017 Wolters Kluwer Health, Inc. All rights reserved.

C1 [Parihar, Vikrant; Ryan, Barbara M.] Trinity Coll Dublin, Tallaght Hosp, Dept Gastroenterol, Dublin 24, Ireland.
[Ridgway, Paul F.; Conlon, Kevin C.] Trinity Coll Dublin, Dept Surg, Tallaght Hosp, Dublin, Ireland.
[Ridgway, Paul F.; Conlon, Kevin C.] Trinity Coll Dublin, Professorial Surg Unit, Dublin, Ireland.
[Huggett, Matthew] Leeds Teaching Hosp NHS Trust, Dept Gastroenterol, Leeds, W Yorkshire, England.
C3 Trinity College Dublin; Trinity College Dublin; Trinity College Dublin;
University of Leeds
RP Parihar, V (corresponding author), Trinity Coll Dublin, Tallaght Hosp, Dept Gastroenterol, Dublin 24, Ireland.
EM vikpar37@yahoo.com
RI ; Ryan, Barbara/AAS-4854-2020
OI Ridgway, Paul/0000-0002-8500-8532; Ryan, Barbara/0000-0001-8400-9202

CR Al-Omran M, 2010, COCHRANE DB SYST REV, V1
[Anonymous], GUT
[Anonymous], DIG ENDOSC
Arvanitakis M, 2004, GASTROENTEROLOGY, V126, P715, DOI 10.1053/j.gastro.2003.12.006
Arvanitakis M, 2007, GASTROINTEST ENDOSC, V65, P609, DOI 10.1016/j.gie.2006.06.083
Bang JY, 2015, DIGEST ENDOSC, V27, P486, DOI 10.1111/den.12418
Bang JY, 2014, CLIN ENDOSC, V47, P429, DOI 10.5946/ce.2014.47.5.429
Bang JY, 2013, CLIN ENDOSC, V46, P500, DOI 10.5946/ce.2013.46.5.500
Bang JY, 2013, J GASTROEN HEPATOL, V28, P1725, DOI 10.1111/jgh.12328
Banks PA, 2013, GUT, V62, P102, DOI 10.1136/gutjnl-2012-302779
Baron TH, 2002, GASTROINTEST ENDOSC, V56, P7, DOI 10.1067/mge.2002.125106
Besselink M, 2013, PANCREATOLOGY, V13, pE1, DOI 10.1016/j.pan.2013.07.063
Cavallini A, 2011, SURG ENDOSC, V25, P1518, DOI 10.1007/s00464-010-1428-9
Chak A, 2000, GASTROINTEST ENDOSC, V52, pS23, DOI 10.1067/mge.2000.110719
Elkhatib I, 2012, TECH GASTROINTEST EN, V14, P186, DOI 10.1016/j.tgie.2012.06.003
Fabbri C, 2012, WORLD J GASTRO ENDOS, V4, P479, DOI 10.4253/wjge.v4.i11.479
FATAAR S, 1990, Australasian Radiology, V34, P334, DOI 10.1111/j.1440-1673.1990.tb02668.x

Gornals JB, 2013, SURG ENDOSC, V27, P1428, DOI 10.1007/s00464-012-2591-y
Habashi S, 2009, WORLD J GASTROENTERO, V15, P38, DOI 10.3748/wjg.15.38
Hirota M, 2002, PANCREAS, V25, P63, DOI 10.1097/00006676-200207000-00015
Hookey LC, 2006, GASTROINTEST ENDOSC, V63, P635, DOI 10.1016/j.gie.2005.06.028
Itoi T, 2012, GASTROINTEST ENDOSC, V75, P870, DOI 10.1016/j.gie.2011.10.020
Jacobson BC, 2005, GASTROINTEST ENDOSC, V61, P363, DOI 10.1016/S0016-5107(04)02779-8
Jedlicka R, 1923, ZENTRALBL CHIR, V50
Kahaleh M, 2006, ENDOSCOPY, V38, P355, DOI 10.1055/s-2006-925249
Khashab MA, 2015, GASTROINTEST ENDOSC, V81, P81, DOI 10.1016/j.gie.2014.08.008
Kim YK, 2009, J COMPUT ASSIST TOMO, V33, P651, DOI 10.1097/RCT.0b013e3181979282
Lang GD, 2016, GASTROINTEST ENDOSC, V83, pAB171, DOI 10.1016/j.gie.2016.03.146
Libera E D, 2000, HPB Surg, V11, P333
Melman L, 2009, SURG ENDOSC, V23, P267, DOI 10.1007/s00464-008-0196-2
Morgan DE, 1997, RADIOLOGY, V203, P773, DOI 10.1148/radiology.203.3.9169703
Mukai S, 2015, ENDOSC ULTRASOUND, V4, P120, DOI 10.4103/2303-9027.156738
Muthusamy VR, 2016, GASTROINTEST ENDOSC, V83, P481, DOI 10.1016/j.gie.2015.11.027
Navaneethan U, 2014, GASTROINTEST ENDOSC, V79, pAB167
Nealon WH, 2009, J AM COLL SURGEONS, V208, P790, DOI 10.1016/j.jamcollsurg.2008.12.027
NEOPTOLEMOS JP, 1993, BRIT J SURG, V80, P94, DOI 10.1002/bjs.1800800131
Ng PY, 2013, ENDOSC ULTRASOUND, V2, P199, DOI 10.4103/2303-9027.121245
Parihar V, 2015, IRISH J MED SCI, V184, pS47
Penn DE, 2012, GASTROINTEST ENDOSC, V76, P679, DOI 10.1016/j.gie.2012.04.457
Rinninella E, 2015, GASTROINTEST ENDOSC, V82, P1039, DOI 10.1016/j.gie.2015.04.006
Rosso E, 2003, DIGEST SURG, V20, P397, DOI 10.1159/000072706
Seewald S, 2009, GASTROINTEST ENDOSC, V69, pS13, DOI 10.1016/j.gie.2008.10.061
Shah RJ, 2015, CLIN GASTROENTEROL H, V13, P747, DOI 10.1016/j.cgh.2014.09.047
Sharaiha RZ, 2015, GASTROINTEST ENDOSC, V82, P822, DOI 10.1016/j.gie.2015.02.035
Shrode CW, 2013, DIGEST LIVER DIS, V45, P129, DOI 10.1016/j.dld.2012.08.026
Siddiqui A, 2015, GASTROINTEST ENDOSC, V81, pAB131, DOI 10.1016/j.gie.2015.03.044
Siddiqui AA, 2016, GASTROINTEST ENDOSC, V83, pAB136, DOI 10.1016/j.gie.2016.03.069
Smoczynski M, 2014, PANCREATOLOGY, V14, P137, DOI 10.1016/j.pan.2013.11.005
Talreja JP, 2008, GASTROINTEST ENDOSC, V68, P1199, DOI 10.1016/j.gie.2008.06.015
Teoh AYB, 2016, WORLD J GASTRO ENDOS, V8, P310, DOI 10.4253/wjge.v8.i6.310
Trevino JM, 2010, J GASTROEN HEPATOL, V25, P526, DOI 10.1111/j.1440-1746.2009.06109.x
Tyberg A, 2016, WORLD J GASTROENTERO, V22, P2256, DOI 10.3748/wjg.v22.i7.2256
Uhl W, 2003, PANCREATOLOGY, V3, DOI 10.1159/000067684
van Brunschot S, 2012, CLIN GASTROENTEROL H, V10, P1190, DOI 10.1016/j.cgh.2012.05.005
Van Os EC, 1999, GASTROINTEST ENDOSC, V50, P536, DOI 10.1016/S0016-5107(99)70079-9
van Santvoort HC, 2011, GASTROENTEROLOGY, V141, P1254, DOI 10.1053/j.gastro.2011.06.073
Varadarajulu S, 2008, GASTROINTEST ENDOSC, V68, P649, DOI 10.1016/j.gie.2008.02.057
Varadarajulu S, 2007, GASTROINTEST ENDOSC, V66, P1107, DOI 10.1016/j.gie.2007.03.1027
Varadarajulu S, 2013, GASTROENTEROLOGY, V145, P583, DOI 10.1053/j.gastro.2013.05.046

Varadarajulu S, 2011, J GASTROINTEST SURG, V15, P2080, DOI 10.1007/s11605-011-1621-8
Varadarajulu S, 2011, J GASTROEN HEPATOL, V26, P1504, DOI 10.1111/j.1440-1746.2011.06771.x
Varadarajulu S, 2011, GASTROINTEST ENDOSC, V74, P74, DOI 10.1016/j.gie.2011.03.1122
Veitch AM, 2016, GUT, V65, P374, DOI 10.1136/gutjnl-2015-311110
Venkatachalapathy SV, 2016, PANCREATOLOGY, V16, pS105
Walter D, 2015, ENDOSCOPY, V47, P63, DOI 10.1055/s-0034-1378113
YEO CJ, 1990, SURG GYNECOL OBSTET, V170, P411
NR 66
TC 6
Z9 7
U1 0
U2 5
PU LIPPINCOTT WILLIAMS & WILKINS
PI PHILADELPHIA
PA TWO COMMERCE SQ, 2001 MARKET ST, PHILADELPHIA, PA 19103 USA
SN 0954-691X
EI 1473-5687
J9 EUR J GASTROEN HEPAT
JI Eur. J. Gastroenterol. Hepatol.
PD APR
PY 2017
VL 29
IS 4
BP 371
EP 379
DI 10.1097/MEG.0000000000000818
PG 9
WC Gastroenterology & Hepatology
WE Science Citation Index Expanded (SCI-EXPANDED)
SC Gastroenterology & Hepatology
GA EM8JQ
UT WOS:000395557800002
PM 28009718
DA 2026-05-09
ER

PT J
AU Tanikawa, T
Ishii, K
Katsumata, R
Urata, N
Nishino, K

Suehiro, M
Kawanaka, M
Haruma, K
Kawamoto, H
AF Tanikawa, Tomohiro
Ishii, Katsunori
Katsumata, Ryo
Urata, Noriyo
Nishino, Ken
Suehiro, Mitsuhiko
Kawanaka, Miwa
Haruma, Ken
Kawamoto, Hirofumi
TI Efficacy of primary drainage by endoscopic ultrasound-guided biliary drainage for unresectable pancreatic adenocarcinoma
SO JGH OPEN
LA English
DT Article
DE biliary drainage; endoscopic retrograde cholangiopancreatography; endoscopic ultrasonography-guided biliary drainage; metal stent; obstructive jaundice
ID MALIGNANT BILIARY; OBSTRUCTION; MULTICENTER; STENT
AB Background and Aim: Obstructive jaundice induced by pancreatic adenocarcinoma is typically treated with biliary drainage with endoscopic retrograde cholangiopancreatography (ERCP)-guided biliary drainage (ERCP-BD). Recently, endoscopic ultrasonography-guided biliary drainage (EUS-BD) was employed as an alternative method after ERCP-BD failed. We aimed to determine the efficacy and safety of EUS-BD for primary biliary drainage.

Methods: Between December 2011 and February 2019, at Kawasaki General Medical Center, we retrospectively enrolled 33 patients who had undergone endoscopic biliary drainage with a metal stent, in a first attempt to relieve obstructive jaundice caused by unresectable pancreatic adenocarcinoma. We compared the technical and clinical outcomes between ERCP-BD and EUS-BD.

Results: Twenty-three patients underwent ERCP-BD and 10 underwent EUS-BD. Both groups achieved 100% technical success. The clinical success rates were similar between the groups: 91% (21/23 patients) for ERCP-BD and 100% (10/10 patients) for EUS-BD ($P = 0.48$). Biliary obstruction recurred in 6/23 patients (26%) treated with ERCP-BD and 1/10 patients (10%) treated with EUS-BD ($P = 0.40$). Other adverse events occurred in 4/23 patients (17%) in the ERCP-BD group and 1/10 patients (10%) in the EUS-BD group ($P = 0.99$).

Conclusion: We suggest that EUS-BD could be employed for primary biliary drainage in patients with obstructive jaundice caused by unresectable pancreatic adenocarcinoma.
C1 [Tanikawa, Tomohiro; Ishii, Katsunori; Katsumata, Ryo; Urata, Noriyo; Nishino, Ken; Suehiro, Mitsuhiko; Kawanaka, Miwa; Haruma, Ken; Kawamoto, Hirofumi] Kawasaki Med Sch, Dept Gen Internal Med 2, Okayama, Japan.
C3 Kawasaki Medical School
RP Tanikawa, T (corresponding author), Kawasaki Med Sch, Dept Gen Internal Med 2, Kita Ku, 2-6-1 Nakasange, Okayama 7008505, Japan.

EM t-tanikawa@med.kawasaki-m.ac.jp
RI ; Katsumata, Ryo/E-3893-2016
OI Tanikawa, Tomohiro/0000-0001-9766-9007; Katsumata,
Ryo/0000-0002-9602-3391
CR Bang JY, 2018, GASTROINTEST ENDOSC, V88, P9, DOI 10.1016/j.gie.2018.03.012
Dhindsa BS, 2020, ENDOSC ULTRASOUND, V9, P101, DOI 10.4103/eus.eus_80_19
Giovannini M, 2001, ENDOSCOPY, V33, P898, DOI 10.1055/s-2001-17324
Han SY, 2019, SCI REP-UK, V9, DOI 10.1038/s41598-019-52993-x
Hathorn KE, 2019, ENDOSC INT OPEN, V7, pE1432, DOI 10.1055/a-0990-9488
Iwashita T, 2020, J HEPATO-BIL-PAN SCI, V27, P968, DOI 10.1002/jhbp.823
Jin Z, 2020, DIGEST ENDOSC, V32, P16, DOI 10.1111/den.13456
Kakked G, 2020, ENDOSC ULTRASOUND, V9, P298, DOI 10.4103/eus.eus_10_20
Kawakubo K, 2014, J HEPATO-BIL-PAN SCI, V21, P328, DOI 10.1002/jhbp.27
Khashab MA, 2016, ENDOSC INT OPEN, V4, pE487, DOI 10.1055/s-0042-102648
Li DF, 2019, REV ESP ENFERM DIG, V111, P953, DOI 10.17235/reed.2019.6125/2018
Matsumoto K, 2017, DIGEST ENDOSC, V29, P617, DOI 10.1111/den.12830
Miller CS, 2019, ENDOSC INT OPEN, V7, pE1563, DOI 10.1055/a-0998-8129
Nakai Y, 2014, J GASTROEN HEPATOL, V29, P1557, DOI 10.1111/jgh.12582
Paik WH, 2018, AM J GASTROENTEROL, V113, P987, DOI 10.1038/s41395-018-0122-8
Park JK, 2018, GASTROINTEST ENDOSC, V88, P277, DOI 10.1016/j.gie.2018.03.015
Prachayakul V, 2013, ENDOSCOPY, V45, pE223, DOI 10.1055/s-0033-1344064
Sassatelli R, 2020, DIGEST LIVER DIS, V52, P57, DOI 10.1016/j.dld.2019.07.009
Satoh T, 2019, INTERNAL MED, V58, P1453, DOI 10.2169/internalmedicine.1862-18
Téllez-Avila FI, 2021, SURG ENDOSC, V35, P2531, DOI 10.1007/s00464-020-07667-5
Watanabe M, 2012, WORLD J RADIOL, V4, P115, DOI 10.4329/wjr.v4.i3.115
Yamao K, 2018, GASTROINTEST ENDOSC, V88, P66, DOI 10.1016/j.gie.2018.01.021
NR 22
TC 2
Z9 2
U1 0
U2 1
PU WILEY
PI HOBOKEN
PA 111 RIVER ST, HOBOKEN 07030-5774, NJ USA
SN 2397-9070
J9 JGH OPEN
JI JGH Open
PD APR
PY 2022
VL 6
IS 4
BP 251

EP 256
DI 10.1002/jgh3.12732
EA APR 2022
PG 6
WC Gastroenterology & Hepatology
WE Emerging Sources Citation Index (ESCI)
SC Gastroenterology & Hepatology
GA 0P2XD
UT WOS:000782197200001
PM 35475205
OA Green Submitted, gold
DA 2026-05-09
ER

PT J
AU Brückner, S
Arlt, A
Hampe, J
AF Brueckner, Stefan
Arlt, Alexander
Hampe, Jochen
TI Endoscopic ultrasound-guided biliary drainage using a lumen-apposing
self-expanding metal stent: a case series
SO ENDOSCOPY
LA English
DT Article
ID MALIGNANT BILIARY; CHOLEDOCHODUODENOSTOMY; OBSTRUCTION;
CHOLANGIOPANCREATOGRAPHY; PLACEMENT
AB Endoscopic ultrasound-guided biliary drainage (EUS-BD) might be an alternative to percutaneous or transpapillary biliary drainage in unresectable pancreatic or biliary cancer. A lumen-apposing, fully covered, self-expanding metal stent, which creates a sealed transluminal conduit between the biliary and gastrointestinal tract may offer advantages over conventional plastic and metal stents. In this retrospective, observational, open-label case study, five patients underwent EUS-BD for obstructive jaundice in pancreatic cancer (n = 4) or distal cholangiocarcinoma (n = 1). Technical and functional success was achieved in all patients without complications. The development of specialized stent and delivery systems may render EUS-BD an effective and safe alternative to percutaneous or transpapillary approaches.
C1 [Brueckner, Stefan; Hampe, Jochen] Tech Univ Dresden, Univ Hosp Dresden, Dept Med 1, D-01062 Dresden, Germany.
[Arlt, Alexander] Univ Kiel, Dept Med 1, Univ Hosp Schleswig Holstein, Kiel, Germany.
C3 Technische Universitat Dresden; Carl Gustav Carus University Hospital;
University of Kiel; Schleswig Holstein University Hospital
RP Hampe, J (corresponding author), Univ Hosp Dresden, Dept Med 1, Fetscherstr 74, D-01307 Dresden, Germany.
EM Jochen.Hampe@uniklinikum-dresden.de
RI Arlt, Alexander/G-7308-2019; Hampe, Jochen/A-2555-2010

OI Arlt, Alexander/0000-0002-6160-1059; Hampe, Jochen/0000-0002-2421-6127
CR Binmoeller KF, 2011, ENDOSCOPY, V43, P337, DOI 10.1055/s-0030-1256127
Burmester E, 2003, GASTROINTEST ENDOSC, V57, P246, DOI 10.1067/mge.2003.85
Glessing BR, 2015, GASTROINTEST ENDOSC, V81, P1019, DOI 10.1016/j.gie.2014.09.061
Hara K, 2011, AM J GASTROENTEROL, V106, P1239, DOI 10.1038/ajg.2011.84
Itoi T, 2008, WORLD J GASTROENTERO, V14, P6078, DOI 10.3748/wjg.14.6078
Itoi T, 2014, GASTROINTEST ENDOSC, V79, P715, DOI 10.1016/j.gie.2013.11.021
Park DH, 2009, AM J GASTROENTEROL, V104, P2168, DOI 10.1038/ajg.2009.254
Varadarajulu S, 2013, GASTROINTEST ENDOSC, V78, P742, DOI 10.1016/j.gie.2013.06.009
Wiersema MJ, 1996, GASTROINTEST ENDOSC, V43, P102, DOI 10.1016/S0016-5107(06)80108-2
Will U, 2007, ENDOSCOPY, V39, P292, DOI 10.1055/s-2007-966215
NR 10
TC 25
Z9 30
U1 0
U2 2
PU GEORG THIEME VERLAG KG
PI STUTTGART
PA RUDIGERSTR 14, D-70469 STUTTGART, GERMANY
SN 0013-726X
EI 1438-8812
J9 ENDOSCOPY
JI Endoscopy
PD SEP
PY 2015
VL 47
IS 9
BP 858
EP 861
DI 10.1055/s-0034-1392104
PG 4
WC Gastroenterology & Hepatology; Surgery
WE Science Citation Index Expanded (SCI-EXPANDED)
SC Gastroenterology & Hepatology; Surgery
GA CQ0GM
UT WOS:000360273400013
PM 26021309
DA 2026-05-09
ER

PT J
AU Mukai, S

Sofuni, A
Tsuchiya, T
Tanaka, R
Tonozuka, R
Yamamoto, K
Matsunami, Y
Nagai, K
Kojima, H
Minami, H
Asano, K
Itoi, T
AF Mukai, Shuntaro
Sofuni, Atsushi
Tsuchiya, Takayoshi
Tanaka, Reina
Tonozuka, Ryosuke
Yamamoto, Kenjiro
Matsunami, Yukitoshi
Nagai, Kazumasa
Kojima, Hiroyuki
Minami, Hirohito
Asano, Kyoko
Itoi, Takao
TI A novel puzzle ring replacement technique of a lumen-apposing metal stent with a double-pigtail plastic stent to prevent walled-off necrosis recurrence (with video)
SO DEN OPEN
LA English
DT Article
DE double-pigtail plastic stent; endoscopic ultrasound-guided transluminal pancreatic fluid collection drainage; lumen-apposing metal stent; necrotizing pancreatitis; walled-off necrosis
ID PANCREATIC-DUCT SYNDROME; DRAINAGE
AB Replacing a lumen-apposing metal stent (LAMS) with a double-pigtail plastic stent (DPS) after treatment for walled-off necrosis contributes to the prevention of recurrence. However, the success rate is not very high. To overcome this issue, we devised a novel stent-replacement technique. In the final treatment procedure, a 7-F DPS was placed in the lumen of the LAMS. Subsequently, the walled-off necrosis shrank, and granulation formed over the pigtail portion, which fixed the DPS. The LAMS alone was removed with grasping forceps, leaving the DPS in the lumen of the LAMS (i.e., a puzzle-ring technique; direct or rotary removal technique). Between August 2021 and August 2023, 18 patients were evaluated for recurrence prevention using this novel technique (median duration of LAMS placement, 37 days). In 17 patients (94.4%), the LAMS was successfully replaced with a 7-F DPS (direct technique 14, rotary technique 3; median removal procedure time, 3 min). No recurrence was observed during the median observation period of 385 days. Before using this technique

(April 2012 to August 2022), the technical success rate of replacement of LAMS with 7-F DPS was significantly lower (61.8% [42/68, p = 0.02]). Recurrence of pancreatic fluid collection occurred in 15.3% (4/26) of the patients who could not undergo replacement with a 7-F DPS. The novel puzzle ring technique, which improves the success rate of LAMS for DPS replacement, may be useful in reducing recurrence after walled-off necrosis treatment.
C1 [Mukai, Shuntaro; Sofuni, Atsushi; Tsuchiya, Takayoshi; Tanaka, Reina; Tonozuka, Ryosuke; Yamamoto, Kenjiro; Matsunami, Yukitoshi; Nagai, Kazumasa; Kojima, Hiroyuki; Minami, Hirohito; Asano, Kyoko; Itoi, Takao] Tokyo Med Univ, Dept Gastroenterol & Hepatol, 6-7-1 Nishishinjuku,Shinjuku Ku, Tokyo 1600023, Japan.
C3 Tokyo Medical University
RP Itoi, T (corresponding author), Tokyo Med Univ, Dept Gastroenterol & Hepatol, 6-7-1 Nishishinjuku,Shinjuku Ku, Tokyo 1600023, Japan.
EM itoi@tokyo-med.ac.jp
RI Mukai, Shuntaro/AAD-4278-2020
OI Mukai, Shuntaro/0000-0001-5737-620X
FX The authors are grateful to Brian Quinn, managing editor of Japan
Medical Communication, for editing the English in our manuscript.
CR Bang JY, 2022, DIGEST ENDOSC, V34, P612, DOI 10.1111/den.14099
Bang JY, 2021, CLIN GASTROENTEROL H, V19, P1275, DOI 10.1016/j.cgh.2020.07.022
Bang JY, 2020, GUT, V69, P1379, DOI 10.1136/gutjnl-2019-320539
Bang JY, 2016, ENDOSC INT OPEN, V4, pE883, DOI 10.1055/s-0042-112586
Chen Y, 2019, BMC GASTROENTEROL, V19, DOI 10.1186/s12876-019-0977-1
Itoi T, 2012, GASTROINTEST ENDOSC, V75, P870, DOI 10.1016/j.gie.2011.10.020
Maatman TK, 2020, PANCREATOLOGY, V20, P362, DOI 10.1016/j.pan.2020.01.014
Pawa R, 2022, DIGEST ENDOSC, V34, P1234, DOI 10.1111/den.14266
Tonozuka R, 2022, J HEPATO-BIL-PAN SCI, V29, pE39, DOI 10.1002/jhbp.1115
van Brunschot S, 2018, LANCET, V391, P51, DOI 10.1016/S0140-6736(17)32404-2
Vanek P, 2023, GASTROINTEST ENDOSC, V97, P1070, DOI 10.1016/j.gie.2022.12.026
Yamauchi H, 2019, DIGEST DIS SCI, V64, P1976, DOI 10.1007/s10620-019-05508-7
NR 12
TC 3
Z9 3
U1 0
U2 1
PU WILEY
PI HOBOKEN
PA 111 RIVER ST, HOBOKEN 07030-5774, NJ USA
SN 2692-4609
J9 DEN OPEN
JI DEN Open
PD OCT 14
PY 2024
VL 5
IS 1

AR e70020
DI 10.1002/deo2.70020
PG 7
WC Gastroenterology & Hepatology
WE Emerging Sources Citation Index (ESCI)
SC Gastroenterology & Hepatology
GA J1E8Y
UT WOS:001334581700001
PM 39403557
OA Green Submitted, gold
DA 2026-05-09
ER

PT J
AU Chapman, CG
Waxman, I
Siddiqui, UD
AF Chapman, Christopher G.
Waxman, Irving
Siddiqui, Uzma D.
TI Endoscopic Ultrasound (EUS)-Guided Pancreatic Duct Drainage: The Basics
of When and How to Perform EUS-Guided Pancreatic Duct Interventions
SO CLINICAL ENDOSCOPY
LA English
DT Review
DE Endosonography; Stents; Pancreatic ducts; Drainage; Pancreatic duct
intervention
ID LONG-TERM; RENDEZVOUS TECHNIQUES; PANCREATICODUODENECTOMY; STENT;
CHOLANGIOPANCREATOGRAPHY; PANCREATICOGASTROSTOMY; PANCREATOGRAPHY;
DISRUPTION; EXPERIENCE; PLACEMENT
AB Despite the advances in endoscopy, endoscopic ultrasound-guided pancreatic duct drainage (EUS-PDD) remains a technically challenging procedure. Technical success rates are greater than 70%; however, the average rate of adverse events is nearly 20%, which increases to 55% when stent migration is included. Until recently, a significant difficulty with this technique was the absence of dedicated devices. Proper patient selection is of utmost importance, and EUS-PDD should be reserved for patients who have failed endoscopic retrograde pancreatography. Furthermore, EUS-PDD must be performed by experienced endoscopists who are familiar with the technique. The most common indications include chronic pancreatitis induced strictures and stones, disconnected pancreatic ducts, inaccessible ampulla, and post-surgical altered anatomy. This manuscript will review the accessories used, techniques employed, and published literature reporting outcomes as well as adverse events regarding EUS-PDD.
C1 [Chapman, Christopher G.; Waxman, Irving; Siddiqui, Uzma D.] Univ Chicago Med, Ctr Endoscop Res & Therapeut, Chicago, IL USA.
C3 University of Chicago

RP Siddiqui, UD (corresponding author), Univ Chicago Med & Biol Sci, Ctr Endoscop Res & Therapeut, 5700 S Maryland Ave MC 8043, Chicago, IL 60637 USA.
EM usiddiqui@bsd.uchicago.edu
CR Amano H, 1998, HEPATO-GASTROENTEROL, V45, P2382
AMMANN RW, 1988, GASTROENTEROLOGY, V95, P1018, DOI 10.1016/0016-5085(88)90178-3
Bakker OJ, 2011, ANN SURG, V253, P961, DOI 10.1097/SLA.0b013e318212e901
Bataille L, 2002, GASTROINTEST ENDOSC, V55, P740, DOI 10.1067/mge.2002.123621
BENAGE D, 1990, GASTROINTEST ENDOSC, V36, P553, DOI 10.1016/S0016-5107(90)71162-5
Brennan PM, 2006, DIGEST SURG, V23, P250, DOI 10.1159/000095398
Dumonceau JM, 2012, ENDOSCOPY, V44, P784, DOI 10.1055/s-0032-1309840
Ergun M, 2011, ENDOSCOPY, V43, P518, DOI 10.1055/s-0030-1256333
Fujii LL, 2013, GASTROINTEST ENDOSC, V78, P854, DOI 10.1016/j.gie.2013.05.016
Fujii-Lau LL, 2015, J HEPATO-BIL-PAN SCI, V22, P51, DOI 10.1002/jhbp.187
Giovannini M, 2013, GASTROINTEST ENDOSC, V78, P865, DOI 10.1016/j.gie.2013.10.019
Irani S, 2012, GASTROINTEST ENDOSC, V76, P586, DOI 10.1016/j.gie.2012.05.006
Itoi T, 2015, ENDOSCOPY, V47, P462, DOI 10.1055/s-0034-1391083
Itoi T, 2013, DIGEST ENDOSC, V25, P241, DOI 10.1111/den.12048
Kahaleh M, 2006, GASTROINTEST ENDOSC, V64, P52, DOI 10.1016/j.gie.2006.01.063
Kikuyama M, 2011, GASTROINTEST ENDOSC, V73, P376, DOI 10.1016/j.gie.2010.10.015
Kurihara T, 2013, DIGEST ENDOSC, V25, P109, DOI 10.1111/den.12100
Mallery S, 2004, GASTROINTEST ENDOSC, V59, P100, DOI 10.1016/S0016-5107(03)02300-9
Morgan KA, 2010, HPB, V12, P211, DOI 10.1111/j.1477-2574.2009.00154.x
Oh D, 2016, GASTROINTEST ENDOSC, V83, P366, DOI 10.1016/j.gie.2015.07.015
Reid-Lombardo KM, 2007, J GASTROINTEST SURG, V11, P1704, DOI 10.1007/s11605-007-0369-7
Takano S, 2000, HEPATO-GASTROENTEROL, V47, P1454
Tessier G, 2007, GASTROINTEST ENDOSC, V65, P233, DOI 10.1016/j.gie.2006.06.029
Varadarajulu S, 2005, GASTROINTEST ENDOSC, V61, P568, DOI 10.1016/S0016-5107(04)02832-9
Vila JJ, 2012, GASTROINTEST ENDOSC, V76, P1133, DOI 10.1016/j.gie.2012.08.001
Will U, 2007, J HEPATO-BILIARY-PAN, V14, P377, DOI 10.1007/s00534-006-1139-8
NR 26
TC 29
Z9 36
U1 0
U2 2
PU KOREAN SOC GASTROINTESTINAL ENDOSCOPY
PI SEOUL
PA 2003 LG PALACE, 165-8 DONGGYO-DONG, MAPO-GU, SEOUL, 121-754, SOUTH KOREA
SN 2234-2400
EI 2234-2443
J9 CLIN ENDOSC
JI Clin. Endosc.
PD MAR

PY 2016
VL 49
IS 2
BP 161
EP 167
DI 10.5946/ce.2016.011
PG 7
WC Gastroenterology & Hepatology
WE Emerging Sources Citation Index (ESCI)
SC Gastroenterology & Hepatology
GA FD1EN
UT WOS:000407280300011
PM 27012290
OA Green Submitted, gold
DA 2026-05-09
ER

PT J
AU Chantarojanasiri, T
Aswakul, P
Prachayakul, V
AF Chantarojanasiri, Tanyaporn
Aswakul, Pitulak
Prachayakul, Varayu
TI Uncommon complications of therapeutic endoscopic ultrasonography: What,
why, and how to prevent
SO WORLD JOURNAL OF GASTROINTESTINAL ENDOSCOPY
LA English
DT Review
DE Complications; Endoscopic ultrasonography; Rare; Therapeutic; Uncommon
ID GUIDED BILIARY DRAINAGE; CELIAC PLEXUS BLOCK; PANCREATIC CYSTIC LESIONS;
SPINAL-CORD INFARCTION; FIDUCIAL PLACEMENT; RADIATION-THERAPY; ALCOHOL
ABLATION; GASTRIC VARICES; ETHANOL-LAVAGE; ABSCESS DRAINAGE
AB There is an increasing role for endoscopic ultrasound (EUS)-guided interventions in the treatment of many conditions. Although it has been shown that these types of interventions are effective and safe, they continue to be considered only as alternative treatments in some situations. This is in part due to the occurrence of complications with these techniques, which can occur even when performed by experienced endosonographers. Although common complications have been described for many procedures, it is also crucial to be aware of uncommon complications. This review describes rare complications that have been reported with several EUS-guided interventions. EUS-guided biliary drainage is accepted as an alternative treatment for malignant biliary obstruction. Most of the uncommon complications related to this procedure involve stent malfunction, such as the migration or malposition of stents. Rare complications of EUS-guided pancreatic pseudocyst drainage can result from air embolism and infection. Finally, a range of uncommon complications has been reported for

EUS-guided celiac plexus neurolysis, involving neural and vascular injuries that can be fatal. The goal of this review is to identify possible complications and promote an understanding of how they occur in order to increase general awareness of these adverse events with the hope that they can be avoided in the future.
C1 [Chantarojanasiri, Tanyaporn] Police Gen Hosp, Dept Internal Med, Div Gastroenterol, Bangkok 10330, Thailand.
[Aswakul, Pitulak] Samitivej Sukhumvit Hosp, Liver & Digest Inst, Bangkok 10120, Thailand.
[Prachayakul, Varayu] Mahidol Univ, Fac Med, Siriraj Hosp, Div Gastroenterol,Dept Internal Med,Siriraj GI En, Bangkok 10700, Thailand.
C3 Mahidol University
RP Prachayakul, V (corresponding author), Mahidol Univ, Fac Med, Siriraj Hosp, Div Gastroenterol,Dept Internal Med,Siriraj GI En, Bangkok 10700, Thailand.
EM kaiyjr@gmail.com
RI Chantarojanasiri, Tanyaporn/AAM-8488-2020
CR Abdalla EK, 1999, J GASTROINTEST SURG, V3, P668, DOI 10.1016/S1091-255X(99)80091-2
Ahmed HM, 2009, ENDOSCOPY, V41, pE218, DOI 10.1055/s-0029-1214941
Ang TL, 2009, ENDOSCOPY, V41, pE21, DOI 10.1055/s-0028-1103468
Ang TL, 2007, J PANCREAS, V8, P438
[Anonymous], 2013, J INTERV GASTROENTER, DOI DOI 10.7178/JIG.128
Artifon ELA, 2007, GASTROINTEST ENDOSC, V66, P1201, DOI 10.1016/j.gie.2007.05.051
Artifon ELA, 2010, J PANCREAS, V11, P597
Attasaranya S, 2012, GASTROENT RES PRACT, V2012, DOI 10.1155/2012/680753
Barclay RL, 2002, GASTROINTEST ENDOSC, V55, P266, DOI 10.1067/mge.2002.120784
Barkay O, 2010, GASTROINTEST ENDOSC, V71, P1166, DOI 10.1016/j.gie.2009.10.048
Binmoeller KF, 2011, GASTROINTEST ENDOSC, V74, P1019, DOI 10.1016/j.gie.2011.06.030
Bokun T, 2013, GASTROENT RES PRACT, V2013, DOI 10.1155/2013/865945
Bories E, 2007, ENDOSCOPY, V39, P287, DOI 10.1055/s-2007-966212
Choi JH, 2014, GUT LIVER, V8, P88, DOI 10.5009/gnl.2014.8.1.88
DAVIES DD, 1993, J ROY SOC MED, V86, P264
Deprez PH, 2008, ACTA GASTRO-ENT BELG, V71, P333
DeWitt J, 2011, GASTROINTEST ENDOSC, V74, P446, DOI 10.1016/j.gie.2011.01.064
DeWitt J, 2009, GASTROINTEST ENDOSC, V70, P710, DOI 10.1016/j.gie.2009.03.1173
DiMaio CJ, 2011, PANCREAS, V40, P664, DOI 10.1097/MPA.0b013e3182128d06
DiMaio CJ, 2010, GASTROINTEST ENDOSC, V71, P1204, DOI 10.1016/j.gie.2010.01.003
Donepudi S, 2013, WORLD J GASTRO ENDOS, V5, P359, DOI 10.4253/wjge.v5.i8.359
EISENBERG E, 1995, ANESTH ANALG, V80, P290, DOI 10.1097/00000539-199502000-00015
Ergun M, 2011, ENDOSCOPY, V43, P518, DOI 10.1055/s-0030-1256333
Fabbri C, 2014, WORLD J GASTROENTERO, V20, P8424, DOI 10.3748/wjg.v20.i26.8424
Fabbri C, 2012, WORLD J GASTRO ENDOS, V4, P479, DOI 10.4253/wjge.v4.i11.479
Fujii L, 2012, ENDOSCOPY, V44, pE265, DOI 10.1055/s-0032-1309708
Fujii LL, 2013, GASTROINTEST ENDOSC, V78, P854, DOI 10.1016/j.gie.2013.05.016
Fujii-Lau LL, 2015, J HEPATO-BIL-PAN SCI, V22, P51, DOI 10.1002/jhbp.187
GALIZIA EJ, 1974, BRIT J ANAESTH, V46, P539, DOI 10.1093/bja/46.7.539
Gan SI, 2005, GASTROINTEST ENDOSC, V61, P746, DOI 10.1016/S0016-5107(05)00320-2

Gimeno-García AZ, 2012, ENDOSCOPY, V44, pE267, DOI 10.1055/s-0032-1309709
Giovannini M, 2001, ENDOSCOPY, V33, P898, DOI 10.1055/s-2001-17324
Gonzalez JM, 2012, ENDOSCOPY, V44, P539, DOI 10.1055/s-0031-1291609
Günter E, 2003, GASTROINTEST ENDOSC, V57, P113, DOI 10.1067/mge.2003.39
Hadithi M, 2014, WORLD J GASTRO ENDOS, V6, P373, DOI 10.4253/wjge.v6.i8.373
Hanada K, 2009, DIGEST ENDOSC, V21, pS75, DOI 10.1111/j.1443-1661.2009.00855.x
Hara K, 2011, AM J GASTROENTEROL, V106, P1239, DOI 10.1038/ajg.2011.84
Hecht JR, 2012, GASTROINTEST ENDOSC, V75, P332, DOI 10.1016/j.gie.2011.10.007
Hecht JR, 2003, CLIN CANCER RES, V9, P555
Hikichi Takuto, 2007, Fukushima J Med Sci, V53, P11
Holt B, 2015, J HEPATO-BIL-PAN SCI, V22, P12, DOI 10.1002/jhbp.150
Horaguchi J, 2012, DIGEST ENDOSC, V24, P457, DOI 10.1111/j.1443-1661.2012.01316.x
Hu YH, 2010, ENDOSCOPY, V42, pE256, DOI 10.1055/s-0030-1255653
Itoi T, 2013, DIGEST ENDOSC, V25, P241, DOI 10.1111/den.12048
Itoi T, 2011, DIGEST ENDOSC, V23, P158, DOI 10.1111/j.1443-1661.2011.01115.x
Iwashita T, 2014, CLIN J GASTROENTEROL, V7, P94, DOI 10.1007/s12328-014-0467-5
Jang HY, 2013, CLIN ENDOSC, V46, P306, DOI 10.5946/ce.2013.46.3.306
Jow AZ, 2012, GASTROINTEST ENDOSC, V75, P220, DOI 10.1016/j.gie.2011.01.047
Jürgensen C, 2006, GASTROINTEST ENDOSC, V63, P1059, DOI 10.1016/j.gie.2005.10.034
Kawakubo K, 2014, J HEPATO-BIL-PAN SCI, V21, P328, DOI 10.1002/jhbp.27
Khashab MA, 2015, GASTROINTEST ENDOSC, V81, P81, DOI 10.1016/j.gie.2014.08.008
Khashab MA, 2012, GASTROINTEST ENDOSC, V76, P921, DOI 10.1016/j.gie.2012.05.018
Kim TH, 2012, WORLD J GASTROENTERO, V18, P2526, DOI 10.3748/wjg.v18.i20.2526
Kinoshita H, 1996, Masui, V45, P1244
Kinzel J, 2014, J CLIN GASTROENTEROL, V48, P362, DOI 10.1097/MCG.0000000000000004
Komaki T, 2011, PANCREATOLOGY, V11, P47, DOI 10.1159/000323508
Kumar A, 2001, REGION ANESTH PAIN M, V26, P75, DOI 10.1053/rapm.2001.20090
Kurihara T, 2013, DIGEST ENDOSC, V25, P109, DOI 10.1111/den.12100
Lalueza A, 2011, ENDOSCOPY, V43, pE9, DOI 10.1055/s-0030-1255804
Lee YT, 2000, GASTROINTEST ENDOSC, V52, P168, DOI 10.1067/mge.2000.107911
Levy MJ, 2008, GASTROINTEST ENDOSC, V67, P572, DOI 10.1016/j.gie.2007.06.063
Levy MJ, 2008, AM J GASTROENTEROL, V103, P352, DOI 10.1111/j.1572-0241.2007.01616.x
Levy MJ, 2008, AM J GASTROENTEROL, V103, P98, DOI 10.1111/j.1572-0241.2007.01607.x
Levy MJ, 2012, GASTROINTEST ENDOSC, V75, P200, DOI 10.1016/j.gie.2011.09.019
Mallery S, 2004, GASTROINTEST ENDOSC, V59, P100, DOI 10.1016/S0016-5107(03)02300-9
Martins FP, 2010, ENDOSCOPY, V42, pE126, DOI 10.1055/s-0029-1243911
Mittal MK, 2012, NEUROLOGY, V78, pE57, DOI 10.1212/WNL.0b013e318248df51
Muscatiello N, 2006, ENDOSCOPY, V38, P858, DOI 10.1055/s-2006-925382
Muscatiello N, 2008, ENDOSCOPY, V40, pE83, DOI 10.1055/s-2007-995540
Noh SH, 2010, GASTROINTEST ENDOSC, V71, P1314, DOI 10.1016/j.gie.2009.12.045
O'Toole TM, 2009, ENDOSCOPY, V41, P593, DOI 10.1055/s-0029-1214868
Oh HC, 2008, GASTROINTEST ENDOSC, V67, P636, DOI 10.1016/j.gie.2007.09.038

Oh HC, 2011, GASTROENTEROLOGY, V140, P172, DOI 10.1053/j.gastro.2010.10.001
Oh HC, 2009, SCAND J GASTROENTERO, V44, P242, DOI 10.1080/00365520802495537
Park DH, 2013, GASTROINTEST ENDOSC, V78, P91, DOI 10.1016/j.gie.2013.01.042
Park DH, 2011, GASTROINTEST ENDOSC, V74, P1276, DOI 10.1016/j.gie.2011.07.054
Park WG, 2010, GASTROINTEST ENDOSC, V71, P513, DOI 10.1016/j.gie.2009.10.030
Pishvaian AC, 2006, GASTROINTEST ENDOSC, V64, P412, DOI 10.1016/j.gie.2006.01.048
Prachayakul V, 2013, ENDOSCOPY, V45, pE223, DOI 10.1055/s-0033-1344064
Prachayakul Varayu, 2012, J Interv Gastroenterol, V2, P202
Prachayakul V, 2013, WORLD J GASTROENTERO, V19, P4758, DOI 10.3748/wjg.v19.i29.4758
Püspök A, 2005, AM J GASTROENTEROL, V100, P1743, DOI 10.1111/j.1572-0241.2005.41806.x
Romagnuolo J, 2011, ENDOSCOPY, V43, pE356, DOI 10.1055/s-0030-1256859
Romero-Castro R, 2007, GASTROINTEST ENDOSC, V66, P402, DOI 10.1016/j.gie.2007.03.008
Romero-Castro R, 2013, GASTROINTEST ENDOSC, V78, P711, DOI 10.1016/j.gie.2013.05.009
Ryan DP, 2014, NEW ENGL J MED, V371, P1039, DOI 10.1056/NEJMra1404198
Sanders MK, 2010, GASTROINTEST ENDOSC, V71, P1178, DOI 10.1016/j.gie.2009.12.020
Saxena P, 2014, ENDOSCOPY, V46, pE140, DOI 10.1055/s-0033-1359240
Seewald S, 2005, GASTROINTEST ENDOSC, V61, P495, DOI 10.1016/S0016-5107(04)02848-2
Seewald S, 2009, GASTROINTEST ENDOSC, V69, pS13, DOI 10.1016/j.gie.2008.10.061
Siddiqui AA, 2011, SURG ENDOSC, V25, P549, DOI 10.1007/s00464-010-1216-6
Singhal S, 2014, THER ADV GASTROENTER, V7, P93, DOI 10.1177/1756283X13506178
Tessier G, 2007, GASTROINTEST ENDOSC, V65, P233, DOI 10.1016/j.gie.2006.06.029
Trevino JM, 2008, GASTROINTEST ENDOSC, V68, P1215, DOI 10.1016/j.gie.2008.07.016
VANDONGEN RTM, 1991, ANAESTHESIA, V46, P862
Varadarajulu S, 2007, GASTROINTEST ENDOSC, V66, P372, DOI 10.1016/j.gie.2007.02.054
Varadarajulu S, 2011, J GASTROINTEST SURG, V15, P2080, DOI 10.1007/s11605-011-1621-8
Varadarajulu S, 2009, GASTROINTEST ENDOSC, V70, P1121, DOI 10.1016/j.gie.2009.08.034
Weilert F, 2012, GASTROINTEST ENDOSC, V76, P915, DOI 10.1016/j.gie.2011.09.027
Wiechowska-Kozlowska A, 2012, GASTROENT RES PRACT, V2012, DOI 10.1155/2012/503098
Wiersema MJ, 1996, GASTROINTEST ENDOSC, V44, P656, DOI 10.1016/S0016-5107(96)70047-0
Will U, 2015, ULTRASCHALL MED, V36, P276, DOI 10.1055/s-0034-1366557
WONG GY, 1995, REGION ANESTH, V20, P352, DOI 10.1136/rapm-00115550-199520040-00013
NR 103
TC 9
Z9 11
U1 0
U2 2
PU BAISHIDENG PUBLISHING GROUP INC
PI PLEASANTON
PA 8226 REGENCY DR, PLEASANTON, CA 94588 USA
SN 1948-5190
J9 WORLD J GASTRO ENDOS
JI World J. Gastrointest. Endosc.

PD AUG 10
PY 2015
VL 7
IS 10
BP 960
EP 968
DI 10.4253/wjge.v7.i10.960
PG 9
WC Gastroenterology & Hepatology
WE Emerging Sources Citation Index (ESCI)
SC Gastroenterology & Hepatology
GA CR0HO
UT WOS:000360999500004
PM 26265989
OA Green Submitted, hybrid
DA 2026-05-09
ER

PT J
AU James, TW
Krafft, M
Croglio, M
Nasr, J
Baron, T
AF James, Theodore W.
Krafft, Matthew
Croglio, Michael
Nasr, John
Baron, Todd
TI EUS-guided gallbladder drainage in patients with cirrhosis: results of a
multicenter retrospective study
SO ENDOSCOPY INTERNATIONAL OPEN
LA English
DT Article
ID ACUTE CHOLECYSTITIS; PERCUTANEOUS CHOLECYSTOSTOMY; RISK;
CHOLECYSTODUODENOSTOMY; MANAGEMENT; ASPIRATION
AB Background and study aims Cirrhosis has historically been considered a relative, if not absolute, contraindication to cholecystectomy. Endoscopic ultrasound-guided gallbladder drainage (EUS-GBD) has been developed for use in non-operative candidates with cholecystitis; however, little data exist for use of the procedure in patients with cirrhosis. Patients and methods This was a retrospective series involving two large tertiary referral centers performing EUS-GBD. Patients with cirrhosis who underwent EUS-GBD for cholecystitis between August 2014 and December 2018 were identified. The primary endpoint was the rate of technical success, defined as EUS-guided placement of a lumen-apposing metal stent

(LAMS) from duodenum to gallbladder. Patient demographics, procedural details, adverse events (AEs), post-procedural symptoms, and clinical success were recorded. Results Fifteen patients (9 females, 6 males) with cirrhosis underwent EUS-GBD during the study period. Mean patient age was 61 +/- 17.1yrs, mean MELD-Na 15 +/- 7. Etiology of cirrhosis was HCV (n = 2), alcohol (n = 4), non-alcoholic fatty liver disease (n = 8), and autoimmune hepatitis (n = 1). The technical success rate was 93.3 % and mean procedure time was 64 +/- 59 minutes. Initial puncture site was duodenum (n = 11), stomach (n = 3) and jejunum (n = 1) and portion of gallbladder used for drainage was neck (n = 4) and body (n = 11). Fourteen patients went on to clinical success and two AEs occurred in this cohort. One decompensation event occurred in a patient with Child-Pugh class C disease 3 weeks post-procedure. Mean length of follow-up was 373 +/- 367.3 days; one death occurred due to underlying malignancy. Conclusion EUS-GBD is safe and efficacious in managing cholecystitis in patients with Child-Pugh A and B cirrhosis who are non-operative candidates. Further studies are needed to determine optimal patient selection and procedural technique.
C1 [James, Theodore W.; Baron, Todd] Univ N Carolina, Div Gastroenterol & Hepatol, 101 Manning Dr, Chapel Hill, NC 27514 USA.

[Krafft, Matthew; Nasr, John] West Virginia Ruby Mem Hosp Digest Dis, Morgantown, WV USA.

[Croglio, Michael] Univ N Carolina, Dept Med, Chapel Hill, NC 27514 USA.

C3 University of North Carolina; University of North Carolina Chapel Hill;

University of North Carolina; University of North Carolina Chapel Hill

RP James, TW (corresponding author), Univ N Carolina, Div Gastroenterol & Hepatol, 101 Manning Dr, Chapel Hill, NC 27514 USA.

EM Ted@unc.edu
CR Agresta F, 2015, LANGENBECK ARCH SURG, V400, P429, DOI 10.1007/s00423-015-1300-4

Baron TH, 2007, GASTROINTEST ENDOSC, V65, P735, DOI 10.1016/j.gie.2006.07.041

Baron TH, 2015, LIVER TRANSPLANT, V21, P1322, DOI 10.1002/lt.24168

Baron TH, 2015, CLIN GASTROENTEROL H, V13, pE13, DOI 10.1016/j.cgh.2014.09.001

Chopra S, 2001, AM J ROENTGENOL, V176, P1025, DOI 10.2214/ajr.176.4.1761025

Cotton PB, 2010, GASTROINTEST ENDOSC, V71, P446, DOI 10.1016/j.gie.2009.10.027

Csikesz NG, 2008, SURGERY, V144, P283, DOI 10.1016/j.surg.2008.03.033

Curro Giuseppe, 2005, JSLS, V9, P311

Duncan CB, 2012, J GASTROINTEST SURG, V16, P2011, DOI 10.1007/s11605-012-2024-1

EASL Clinical Practice, 2018, J HEPATOL, V69, P406, DOI DOI 10.1016/j.jhep.2018.03.024

Friedman LS, 1999, HEPATOLOGY, V29, P1617, DOI 10.1002/hep.510290639

Granlund N, 2001, LANGENBECK ARCH SURG, V386, P212, DOI 10.1007/s004230100211

Ito K, 2004, AM J ROENTGENOL, V183, P193, DOI 10.2214/ajr.183.1.1830193

Itoi T, 2008, GASTROINTEST ENDOSC, V68, P455, DOI 10.1016/j.gie.2008.02.052

Itoi T, 2010, GASTROINTEST ENDOSC, V71, P1038, DOI 10.1016/j.gie.2010.01.026

Oku Takatomi, 2013, Nihon Shokakibyo Gakkai Zasshi, V110, P989

Park JK, 2018, GASTROINTEST ENDOSC, V88, P277, DOI 10.1016/j.gie.2018.03.015

Patel M, 2000, AM SURGEON, V66, P33

Shi XJ, 2018, MEDICINE, V97, DOI 10.1097/MD.0000000000010414

Singer M, 2016, JAMA-J AM MED ASSOC, V315, P801, DOI 10.1001/jama.2016.0287

STEINBERG HV, 1988, GASTROINTEST RADIOL, V13, P347, DOI 10.1007/BF01889096

Tujios SR, 2015, J CLIN GASTROENTEROL, V49, P771, DOI 10.1097/MCG.0000000000000269

Wiesner R, 2003, GASTROENTEROLOGY, V124, P91, DOI 10.1053/gast.2003.50016
NR 23
TC 32
Z9 33
U1 0
U2 3
PU GEORG THIEME VERLAG KG
PI STUTTGART
PA RUDIGERSTR 14, D-70469 STUTTGART, GERMANY
SN 2364-3722
EI 2196-9736
J9 ENDOSC INT OPEN
JI Endosc. Int. Open
PD SEP
PY 2019
VL 7
IS 9
BP E1099
EP E1104
DI 10.1055/a-0965-6662
PG 6
WC Gastroenterology & Hepatology; Surgery
WE Emerging Sources Citation Index (ESCI)
SC Gastroenterology & Hepatology; Surgery
GA IU5QY
UT WOS:000483643600007
PM 31475226
OA Green Submitted, gold
DA 2026-05-09
ER

PT J
AU Grover, D
Fatima, I
Dharan, M
AF Grover, Dheera
Fatima, Ifrah
Dharan, Murali
TI Comparison of trans-gastric vs trans-enteric (trans-duodenal or
trans-jejunal) endoscopic ultrasound guided gallbladder drainage using
lumen apposing metal stents
SO WORLD JOURNAL OF GASTROINTESTINAL ENDOSCOPY

LA English
DT Article
DE Transduodenal; Transgastric; Cautery; Endoscopic ultrasound guided
gallbladder drainage; AXIOS
ID ACUTE CHOLECYSTITIS; OUTCOMES; UNFIT
AB BACKGROUNDEndoscopic ultrasound guided gallbladder drainage (EUS-GBD) is being increasingly used in practice (either as a bridge to cholecystectomy in high-risk patients or as destination therapy in non-surgical patients). Stents are used to create a conduit between the lumen of the gallbladder (GB) and the intestinal lumen through the gastric or enteric routes. Among the various types of stents used, cautery-enhanced lumen apposing metallic stents (LAMS) may be associated with fewer adverse events (AEs).AIMTo compare the clinical success, technical success, and rate of AEs between transgastric (TG) and trans-enteric [transduodenal (TD)/transjejunal (TJ)] approach to GB drainage. Further, we analyzed whether using cautery enhanced stents during EUS-GBD impacts the above parameters.METHODSStudy was registered in PROSPERO (CRD42022319019) and comprehensive literature review was conducted. Manuscripts were reviewed for the data collection: Rate of AEs, clinical success, and technical success. Random effects model was utilized for the analysis.RESULTSNo statistically significant difference in clinical and technical success between the TD/TJ and TG approaches ($P > 0.05$) were noted. There was no statistically significant difference in the rate of AEs when comparing two-arm studies only. However, when all studies were included in the analysis difference was almost significant favoring the TD/TJ approach. When comparing cautery-enhanced LAMS with non-cautery enhanced LAMS, a statistically significant difference in the rate of AEs was observed when all the studies were included, with the rate being higher in non-cautery enhanced stents (14.0% vs 37.8%; $P < 0.01$).CONCLUSIONAs per our study results, TD/TJ approach appears to be associated with lower rate of adverse events and comparable efficacy when compared to the TG approach for the EUS-GBD. Additionally, use of cautery-enhanced LAMS for EUS-GBD is associated with a more favorable adverse event profile compared to cold LAMS. Though the approach chosen depends on several patient and physician factors, the above findings could help in deciding the ideal drainage route when both TG and TD/TJ approaches are feasible.
C1 [Grover, Dheera] Univ Connecticut, Dept Internal Med, Farmington, CT 06030 USA.
[Fatima, Ifrah] Univ Missouri, Dept Internal Med, Kansas City, MO 64110 USA.
[Dharan, Murali] Univ Connecticut Hlth Ctr, Dept Gastroenterol & Hepatol, 263 Farmington Ave, Farmington, CT 06030 USA.
C3 University of Connecticut; University of Missouri System; University of
Missouri Kansas City; University of Connecticut
RP Dharan, M (corresponding author), Univ Connecticut Hlth Ctr, Dept Gastroenterol & Hepatol, 263 Farmington Ave, Farmington, CT 06030 USA.
EM dharan@uchc.edu
CR Anderloni A, 2016, SURG ENDOSC, V30, P5200, DOI 10.1007/s00464-016-4894-x
Binmoeller KF, 2011, ENDOSCOPY, V43, P337, DOI 10.1055/s-0030-1256127
Choi JH, 2014, ENDOSCOPY, V46, P656, DOI 10.1055/s-0034-1365720
Gallaher JR, 2022, JAMA-J AM MED ASSOC, V327, P965, DOI 10.1001/jama.2022.2350
James TW, 2019, ENDOSC ULTRASOUND, V8, pS28, DOI 10.4103/eus.eus_41_19
Jang JW, 2012, GASTROENTEROLOGY, V142, P805, DOI 10.1053/j.gastro.2011.12.051
Jang JW, 2011, GASTROINTEST ENDOSC, V74, P176, DOI 10.1016/j.gie.2011.03.1120
Kalva NR, 2018, CAN J GASTROENTEROL, V2018, DOI 10.1155/2018/7070961
Loozen CS, 2018, BMJ-BRIT MED J, V363, DOI 10.1136/bmj.k3965

Luk SWY, 2019, ENDOSCOPY, V51, P722, DOI 10.1055/a-0929-6603
Manta R, 2018, EUR J GASTROEN HEPAT, V30, P695, DOI 10.1097/MEG.0000000000001112
Molina H, 2021, SEMIN INTERVENT RAD, V38, P364, DOI 10.1055/s-0041-1731375
Mori Y, 2018, J HEPATO-BIL-PAN SCI, V25, P87, DOI 10.1002/jhbp.504
Perez-Miranda M, 2018, ENDOSC ULTRASOUND, V7, P79, DOI 10.4103/eus.eus_5_18
Podboy A, 2021, GASTROINTEST ENDOSC, V93, P797, DOI 10.1016/j.gie.2020.09.040
Posner H, 2020, TRANSL GASTROENT HEP, V5, DOI 10.21037/tgh.2019.12.20
Saumoy M, 2017, GASTROINTEST ENDOSC, V85, pAB481, DOI 10.1016/j.gie.2017.03.1111
Teoh AY, 2018, GASTROINTEST ENDOSC, V87, pAB62, DOI 10.1016/j.gie.2018.04.045
Teoh AYB, 2017, ENDOSCOPY, V49, P130, DOI 10.1055/s-0042-119036
Tyberg A, 2018, J CLIN GASTROENTEROL, V52, P79, DOI 10.1097/MCG.0000000000000786
van der Merwe SW, 2022, ENDOSCOPY, V54, P185, DOI 10.1055/a-1717-1391
Walter D, 2016, GUT, V65, P6, DOI 10.1136/gutjnl-2015-309925
NR 22
TC 4
Z9 5
U1 0
U2 0
PU BAISHIDENG PUBLISHING GROUP INC
PI PLEASANTON
PA 7041 Koll Center Parkway, Suite 160, PLEASANTON, CA, UNITED STATES
SN 1948-5190
J9 WORLD J GASTRO ENDOS
JI World J. Gastrointest. Endosc.
PD SEP 16
PY 2023
VL 15
IS 9
DI 10.4253/wjge.v15.i9.574
PG 11
WC Gastroenterology & Hepatology
WE Emerging Sources Citation Index (ESCI)
SC Gastroenterology & Hepatology
GA S8IW5
UT WOS:001073559400004
PM 37744320
OA Green Submitted, gold
DA 2026-05-09
ER

PT J
AU Mora-Soler, AM

Alvarez-Delgado, A
Piñero-Pérez, MC
Velasco-Guardado, A
Marcos-Prieto, H
Rodríguez-Pérez, A
AF Maria Mora-Soler, Ana
Alvarez-Delgado, Alberto
Concepcion Pinero-Perez, Maria
Velasco-Guardado, Antonio
Marcos-Prieto, Hector
Rodriguez-Perez, Antonio
TI Endoscopic ultrasound-guided choledochoduodenostomy after a failed or impossible ERCP
SO REVISTA ESPANOLA DE ENFERMEDADES DIGESTIVAS
LA English
DT Article
DE Biliary drainage; EUS-BD; Choledochoduodenostomy; EUCD; Endoscopic ultrasound; EUS; Failed ERCP
ID MALIGNANT BILIARY OBSTRUCTION; DRAINAGE; MULTICENTER; EXPERIENCE
AB Introduction: endoscopic ultrasound-guided biliary drainage (EUS-BD) is an alternative to percutaneous trans-hepatic biliary drainage (PTBD) in cases of failed endoscopic retrograde cholangiopancreatography (ERCP).

Methods: this is a retrospective description of six cases of endoscopic ultrasound-guided biliary drainage via choledochoduodenostomy (EUCD), as well as the clinical characteristics, endoscopic procedure, complications and monitoring.

Results: all cases had malignant distal biliary obstruction. The procedure was concluded with good drainage in four out of six patients. Two late complications were recorded that were caused by stent migration and there were no deaths related with the procedure. The average monitoring period was six months.

Conclusions: EUCD can be considered as a valid therapeutic choice in some selected cases and when performed by a team of expert endoscopists in cases of failed ERCP drainage or as an alternative to PTBD. However, the procedure has some associated complications.
C1 [Maria Mora-Soler, Ana; Alvarez-Delgado, Alberto; Concepcion Pinero-Perez, Maria; Velasco-Guardado, Antonio; Marcos-Prieto, Hector; Rodriguez-Perez, Antonio] Hosp Univ Salamanca, Inst Biomed Res Salamanca IBSAL, Serv Digest Dis, Salamanca, Spain.
C3 University of Salamanca
RP Mora-Soler, AM (corresponding author), Hosp Univ Salamanca, Inst Biomed Res Salamanca IBSAL, Dept Digest Dis, Pso San Vicente 55-182, Salamanca 37007, Spain.
EM ana.mora.soler@gmail.com
RI Velasco Guardado, Antonio/H-7237-2019
OI Velasco Guardado, Antonio/0000-0002-9458-0602
CR [Anonymous], 2013, J INTERV GASTROENTER, DOI DOI 10.7178/JIG.128
Artifon ELA, 2015, GASTROINTEST ENDOSC, V81, P950, DOI 10.1016/j.gie.2014.09.047
Giovannini M, 2001, ENDOSCOPY, V33, P898, DOI 10.1055/s-2001-17324

Hara K, 2016, WORLD J GASTROENTERO, V22, P1297, DOI 10.3748/wjg.v22.i3.1297
Kawakubo K, 2014, J HEPATO-BIL-PAN SCI, V21, P328, DOI 10.1002/jhbp.27
Khashab MA, 2015, DIGEST DIS SCI, V60, P557, DOI 10.1007/s10620-014-3300-6
Komaki T, 2011, PANCREATOLOGY, V11, P47, DOI 10.1159/000323508
Kunda R, 2016, SURG ENDOSC, V30, P5002, DOI 10.1007/s00464-016-4845-6
Mallery S, 2004, GASTROINTEST ENDOSC, V59, P100, DOI 10.1016/S0016-5107(03)02300-9
Maluf F, 2012, J PANCREAS, V13, P210
Maranki J, 2009, ENDOSCOPY, V41, P532, DOI 10.1055/s-0029-1214712
Moon JH, 2014, GASTROINTEST ENDOSC, V79, P990, DOI 10.1016/j.gie.2014.02.015
Ogura T, 2015, WORLD J GASTROENTERO, V21, P820, DOI 10.3748/wjg.v21.i3.820
Park DH, 2011, GASTROINTEST ENDOSC, V73, P64, DOI 10.1016/j.gie.2010.09.039
Rai P, 2014, ENDOSCOPY, V46, DOI 10.1055/s-0034-1377286
Vila JJ, 2012, GASTROINTEST ENDOSC, V76, P1133, DOI 10.1016/j.gie.2012.08.001
Wang KX, 2016, GASTROINTEST ENDOSC, V83, P1218, DOI 10.1016/j.gie.2015.10.033
Wiersema MJ, 1996, GASTROINTEST ENDOSC, V43, P102, DOI 10.1016/S0016-5107(06)80108-2
NR 18
TC 6
Z9 9
U1 0
U2 0
PU ARAN EDICIONES, S A
PI MADRID
PA CASTELLO, 128, 28006 MADRID, SPAIN
SN 1130-0108
EI 2340-4167
J9 REV ESP ENFERM DIG
JI Rev. Esp. Enferm. Dig.
PY 2018
VL 110
IS 5
BP 299
EP 305
DI 10.17235/reed.2018.5040/2017
PG 7
WC Gastroenterology & Hepatology
WE Science Citation Index Expanded (SCI-EXPANDED)
SC Gastroenterology & Hepatology
GA GH5CS
UT WOS:000433434200005
PM 29332405
OA Green Submitted
DA 2026-05-09

ER

PT J
AU Gopakumar, H
Revanur, V
Kandula, R
Puli, SR
AF Gopakumar, Harishankar
Revanur, Vakya
Kandula, Rajanikanth
Puli, Srinivas R.
TI Endoscopic ultrasound-guided lumen-apposing metal stent with or without
coaxial plastic stent for pancreatic fluid collections: a systematic
review and meta-analysis comparing safety and efficacy
SO ANNALS OF GASTROENTEROLOGY
LA English
DT Review
DE Pancreatic fluid collections; walled-off pancreatic necrosis;
lumen-apposing metal stent; double pigtail plastic stents; endoscopic
ultrasound
ID DRAINAGE; PSEUDOCYST; OUTCOMES
AB Background Endoscopic ultrasound (EUS)-guided lumen-apposing metal stents (LAMS) are preferred for draining symptomatic large pancreatic fluid collections (PFCs). A concurrent coaxial double-pigtail plastic stent (DPPS) is proposed to reduce adverse events associated with LAMS. We aimed to perform a comparative outcome analysis of LAMS with or without DPPS for PFCs. Methods Electronic databases from January 2005 through July 2023 were searched for studies comparing the use of LAMS with or without DPPS for PFCs. Pooled proportions were calculated using fixed (inverse variance) and random-effects (DerSimonian-Laird) models. Results After reviewing 1780 studies, we extracted data from 6 studies comprising 348 patients. The weighted odds of overall technical success, using LAMS plus DPPS compared to LAMS alone, were 0.53 (95% confidence interval [CI] 0.15-1.83), and the odds of clinical success were 1.10 (95%CI 0.59-2.05). The weighted odds of total adverse events with LAMS compared to LAMS plus DPPS were 2.21 (95%CI 1.37-3.59). Analysis of individual adverse events showed that the odds of stent occlusion when LAMS alone was used compared to LAMS plus DPPS was 2.36 (95%CI 1.12-4.98). The odds of bleeding were 1.84 (95%CI 0.77-4.38), and the odds of stent migration 0.95 (95%CI 0.40-2.23). Conclusions EUS-guided LAMS placement is the current standard of care for managing symptomatic large PFCs. Concurrent use of coaxial DPPS can mitigate the overall adverse events observed with LAMS, while maintaining similar technical and clinical success.
C1 [Gopakumar, Harishankar; Revanur, Vakya; Kandula, Rajanikanth; Puli, Srinivas R.] Univ Illinois, Coll Med Peoria, Dept Gastroenterol & Hepatol, Peoria, IL USA.
[Gopakumar, Harishankar] Dept Gastroenterol & Hepatol, 530 NE Glen Oak Ave, Peoria, IL 61637 USA.
C3 University of Illinois System; University of Illinois Peoria
RP Gopakumar, H (corresponding author), Dept Gastroenterol & Hepatol, 530 NE Glen Oak Ave, Peoria, IL 61637 USA.
EM hgopakumarmd@gmail.com
RI Gopakumar, Harishankar/GVU-4901-2022

OI Gopakumar, Harishankar/0000-0002-4921-8794
CR Aburajab M, 2018, GASTROINTEST ENDOSC, V87, P1248, DOI 10.1016/j.gie.2017.11.033
Ali SE, 2019, ANN GASTROENTEROL, V32, P620, DOI 10.20524/aog.2019.0414
Bang JY, 2022, DIGEST ENDOSC, V34, P612, DOI 10.1111/den.14099
Bang JY, 2019, GUT, V68, P1200, DOI 10.1136/gutjnl-2017-315335
Banks PA, 2013, GUT, V62, P102, DOI 10.1136/gutjnl-2012-302779
Beran A, 2023, GASTROENTEROL RES, V16, P59, DOI 10.14740/gr1601
Brimhall B, 2018, CLIN GASTROENTEROL H, V16, P1521, DOI 10.1016/j.cgh.2018.02.021
Elkhatib I, 2012, TECH GASTROINTEST EN, V14, P186, DOI 10.1016/j.tgie.2012.06.003
Giri S, 2023, THER ADV GASTROINTES, V16, DOI 10.1177/26317745231199364
Gornals JB, 2016, SURG ENDOSC, V30, P2592, DOI 10.1007/s00464-015-4505-2
Gornals JB, 2013, SURG ENDOSC, V27, P1428, DOI 10.1007/s00464-012-2591-y
Haddad JD, 2023, TECH INNOVAT GASTROI, V25, P113, DOI 10.1016/j.tige.2022.12.001
Hammad T, 2018, DIGEST DIS SCI, V63, P289, DOI 10.1007/s10620-017-4851-0
Itoi T, 2012, GASTROINTEST ENDOSC, V75, P870, DOI 10.1016/j.gie.2011.10.020
Jo SJ, 2023, J HEPATO-BIL-PAN SCI, V30, P263, DOI 10.1002/jhbp.1180
Kahaleh M, 2006, ENDOSCOPY, V38, P355, DOI 10.1055/s-2006-925249
Khan S, 2021, J GASTROEN HEPATOL, V36, P3395, DOI 10.1111/jgh.15658
Lang GD, 2018, GASTROINTEST ENDOSC, V87, P150, DOI 10.1016/j.gie.2017.06.029
Li P, 2023, ENDOSC ULTRASOUND, V12, P259, DOI 10.4103/EUS-D-22-00058
Manrai M, 2018, ANN SURG, V267, P357, DOI 10.1097/SLA.0000000000002065
Mukai S, 2022, DIGEST ENDOSC, V34, P459, DOI 10.1111/den.14125
Muthusamy VR, 2016, GASTROINTEST ENDOSC, V83, P481, DOI 10.1016/j.gie.2015.11.027
Neyeloff Jeruza L, 2012, BMC Res Notes, V5, P52, DOI 10.1186/1756-0500-5-52
Nieto J, 2023, J CLIN GASTROENTEROL, V57, P218, DOI 10.1097/MCG.0000000000001545
Puga M, 2018, ENDOSCOPY, V50, P1022, DOI 10.1055/a-0582-9127
Shamah SP, 2022, ENDOSC ULTRASOUND, V11, P53, DOI 10.4103/EUS-D-21-00030
Siddiqui AA, 2017, GASTROINTEST ENDOSC, V85, P758, DOI 10.1016/j.gie.2016.08.014
Vanek P, 2023, GASTROINTEST ENDOSC, V97, P1070, DOI 10.1016/j.gie.2022.12.026
Varadarajulu S, 2011, J GASTROINTEST SURG, V15, P2080, DOI 10.1007/s11605-011-1621-8
Wang ZJ, 2019, J GASTROEN HEPATOL, V34, P791, DOI 10.1111/jgh.14557
NR 30
TC 6
Z9 7
U1 0
U2 0
PU HELLENIC SOC GASTROENTEROLOGY
PI ATHENS
PA DEMOKRATIAS AVE 67, ATHENS, 15451, GREECE
SN 1108-7471
EI 1792-7463
J9 ANN GASTROENTEROL

JI Ann. Gastroenterol.
PY 2024
VL 37
IS 2
BP 242
EP 250
DI 10.20524/aog.2024.0858
EA FEB 2024
PG 9
WC Gastroenterology & Hepatology
WE Emerging Sources Citation Index (ESCI)
SC Gastroenterology & Hepatology
GA LR5P2
UT WOS:001168078500001
PM 38481778
OA Green Submitted, gold
DA 2026-05-09
ER

PT J
AU Carbajo, AY
Kahaleh, M
Tyberg, A
AF Carbajo, Ana Y.
Kahaleh, Michel
Tyberg, Amy
TI Clinical Review of EUS-guided Gastroenterostomy (EUS-GE)
SO JOURNAL OF CLINICAL GASTROENTEROLOGY
LA English
DT Review
DE therapeutic endoscopic ultrasound; EUS-guided gastrojejunostomy; gastric
outlet obstruction; lumen-apposing metal stent
ID GASTRIC OUTLET OBSTRUCTION; APPOSING METAL STENT; SURGICAL
GASTROJEJUNOSTOMY; FLEXIBLE ENDOSCOPY; ANASTOMOSIS; PALLIATION;
EXPERIENCE; PLACEMENT; CREATION; MULTICENTER
AB Gastric outlet obstruction (GOO) refers to mechanical obstruction of the distal stomach or proximal duodenum and it is associated with a significant decrease in quality of life. Surgical gastrojejunostomy and self-expandable metal stents were the traditional treatment for GOO. Recently, endoscopic ultrasound guided gastroenterostomy (EUS-GE) has emerged as a third therapeutic option for patients with GOO. Most EUS-GE techniques utilize the placement of a lumen-apposing metal stent under echoendoscopy but differ in the method of localizing the jejunal loop prior to EUS puncture. Data supporting EUS-GE have been promising. Case series including 10 or more cases showed the technical success rate to be approximately 90%. Clinical success is achieved in approximately 85-90% and a less than 18% risk of adverse events is

reported. EUS-GE was associated with a lower recurrence of GOO and need for re-intervention when compared to enteral stenting. In addition, EUS-GE shows significantly fewer adverse events compared with surgical gastrojejunostomy. In conclusion, EUS-GE provides symptom relief without the risks of surgical intervention and the limited patency of enteral SEMS placement. EUS-GE is an exciting new option in the management of GOO. Despite the excellent results, randomized studies comparing these different modalities of treatment for GOO are needed before EUS-GE can be accepted as standard of care.
C1 [Carbajo, Ana Y.] Univ Hosp Rio Hortega, Valladolid, Spain.
[Kahaleh, Michel; Tyberg, Amy] Rutgers Robert Wood Johnson Univ Hosp, New Brunswick, NJ USA.
C3 Hospital del Rio Hortega; Rutgers University System; Rutgers University New Brunswick; Rutgers University Biomedical & Health Sciences
RP Kahaleh, M (corresponding author), Rutgers State Univ, Robert Wood Johnson Univ Hosp, 1 RWJ Pl,MEB 491B, New Brunswick, NJ 08901 USA.
EM mkahaleh@gmail.com
RI Kahaleh, Michel/A-9408-2011
FU Boston Scientific; MaunaKea; Apollo Endosurgery; Cook Endoscopy; ASPIRE Bariatrics; NinePoint Medical; Merit Medical; Olympus; Interscope Med
FX A.T. has done consulting work for EndoGastric Solutions, NinePoint Medical, and Obalon Therapeutics. M.K. has Grant support from Boston Scientific, MaunaKea, Apollo Endosurgery, Cook Endoscopy, ASPIRE Bariatrics, NinePoint Medical, Merit Medical, Olympus, and Interscope Med. Consultant for Boston Scientific, Concordia Laboratories Inc., Interscope Med. and ABBvie. A.Y.C. declares that there is nothing to disclose.
CR Adler DG, 2002, AM J GASTROENTEROL, V97, P72
Mahler MA, 2018, ENDOSCOPY, V50, P78, DOI 10.1055/s-0043-119972
Barthet M, 2015, GASTROINTEST ENDOSC, V81, P215, DOI 10.1016/j.gie.2014.09.039
Bartlett EK, 2014, J GASTROINTEST SURG, V18, P1292, DOI 10.1007/s11605-014-2502-8
Benallal DC, 2018, ENDOSC ULTRASOUND, V7, P418, DOI 10.4103/eus.eus_41_17
Bergström M, 2006, GASTROINTEST ENDOSC, V63, P307, DOI 10.1016/j.gie.2005.09.035
Binmoeller KF, 2012, ENDOSCOPY, V44, P499, DOI 10.1055/s-0032-1309382
Chen YI, 2018, GASTROINTEST ENDOSC, V87, P1215, DOI 10.1016/j.gie.2017.07.030
Chen YI, 2018, ENDOSC INT OPEN, V6, pE363, DOI 10.1055/s-0043-123468
Chen YI, 2017, SURG ENDOSC, V31, P2946, DOI 10.1007/s00464-016-5311-1
Chopita N, 2005, ENDOSCOPY, V37, P313, DOI 10.1055/s-2005-861358
Chowdhury A, 1996, AM J GASTROENTEROL, V91, P1679
Chowdhury SD, 2016, ENDOSCOPY, V48, pE263, DOI 10.1055/s-0042-112971
Fritscher-Ravens A, 2003, GASTROINTEST ENDOSC, V58, P585
Fritscher-Ravens A, 2002, GASTROINTEST ENDOSC, V56, P737, DOI 10.1067/mge.2002.129084
Gutierrez OIB, 2017, ENDOSC INT OPEN, V5, pE893, DOI 10.1055/s-0043-115386
Gutierrez OIB, 2017, ENDOSCOPY, V49, P921, DOI 10.1055/s-0043-111711
Haito-Chavez Yamile, 2016, VideoGIE, V1, P47, DOI [10.1016/j.vgie.2016.08.009, 10.1016/j.vgie.2016.08.009]
Ikeuchi N, 2015, GASTROINTEST ENDOSC, V82, P166, DOI 10.1016/j.gie.2015.01.010

Itoi T, 2016, GUT, V65, P193, DOI 10.1136/gutjnl-2015-310348
Itoi T, 2015, J HEPATO-BIL-PAN SCI, V22, P3, DOI 10.1002/jhbp.148
Itoi T, 2013, GASTROINTEST ENDOSC, V78, P934, DOI 10.1016/j.gie.2013.09.025
Jeurnink SM, 2007, J SURG ONCOL, V96, P389, DOI 10.1002/jso.20828
Jeurnink SM, 2010, GASTROINTEST ENDOSC, V71, P490, DOI 10.1016/j.gie.2009.09.042
JOHNSON CD, 1995, AM J GASTROENTEROL, V90, P1740
Kantsevoy SV, 2005, GASTROINTEST ENDOSC, V62, P287, DOI 10.1016/S0016-5107(05)01565-8
Khashab M, 2013, SURG ENDOSC, V27, P2068, DOI 10.1007/s00464-012-2712-7
Khashab Mouen A, 2017, Gastroenterol Hepatol (N Y), V13, P311
Khashab MA, 2017, ENDOSC INT OPEN, V5, pE275, DOI 10.1055/s-0043-101695
Khashab MA, 2015, GASTROINTEST ENDOSC, V82, P932, DOI 10.1016/j.gie.2015.06.017
Khashab MA, 2015, GASTROINTEST ENDOSC, V81, P1234, DOI 10.1016/j.gie.2014.12.053
Krafft Matthew R, 2018, VideoGIE, V3, P213, DOI [10.1016/j.vgie.2018.04.007, 10.1016/j.vgie.2018.04.007]
Law R, 2015, GASTROINTEST ENDOSC, V82, P567, DOI 10.1016/j.gie.2015.04.010
Maetani I, 2004, ENDOSCOPY, V36, P73
Ngamruengphong S, 2016, GASTROINTEST ENDOSC, V84, P527, DOI 10.1016/j.gie.2016.03.1493
Perez-Miranda M, 2017, J CLIN GASTROENTEROL, V51, P896, DOI 10.1097/MCG.0000000000000887
Sanchez-Ocana Ramon, 2017, VideoGIE, V2, P346, DOI [10.1016/j.vgie.2017.08.004, 10.1016/j.vgie.2017.08.004]
Shah A, 2015, ENDOSCOPY, V47, pE309, DOI 10.1055/s-0034-1392210
SHONE DN, 1995, AM J GASTROENTEROL, V90, P1769
SWAIN CP, 1991, GASTROINTEST ENDOSC, V37, P628, DOI 10.1016/S0016-5107(91)70870-5
Takeno A, 2013, ANN SURG ONCOL, V20, P3527, DOI 10.1245/s10434-013-3033-3
Tyberg A, 2017, ENDOSCOPY, V49, pE252, DOI 10.1055/s-0043-115886
Tyberg A, 2017, ENDOSCOPY, V49, P1007, DOI 10.1055/s-0043-113551
Tyberg A, 2016, ENDOSC INT OPEN, V4, pE276, DOI 10.1055/s-0042-101789
Tyberg A, 2016, GASTROINTEST ENDOSC, V83, P254, DOI 10.1016/j.gie.2015.08.003
Tyberg A, 2015, GASTROINTEST ENDOSC, V81, P1011, DOI 10.1016/j.gie.2014.10.018
Tyberg A, 2015, GASTROENTEROLOGY, V148, P506, DOI 10.1053/j.gastro.2014.12.029
Ungureanu BS, 2018, SURG INNOV, V25, P16, DOI 10.1177/1553350617748278
van Halsema EE, 2015, WORLD J GASTROENTERO, V21, P12468, DOI 10.3748/wjg.v21.i43.12468
Yi SW, 2014, SURG ENDOSC, V28, P1439, DOI 10.1007/s00464-013-3371-z
NR 50
TC 41
Z9 49
U1 2
U2 7
PU LIPPINCOTT WILLIAMS & WILKINS
PI PHILADELPHIA
PA TWO COMMERCE SQ, 2001 MARKET ST, PHILADELPHIA, PA 19103 USA
SN 0192-0790
EI 1539-2031
J9 J CLIN GASTROENTEROL

JI J. Clin. Gastroenterol.
PD JAN
PY 2020
VL 54
IS 1
BP 1
EP 7
DI 10.1097/MCG.0000000000001262
PG 7
WC Gastroenterology & Hepatology
WE Science Citation Index Expanded (SCI-EXPANDED)
SC Gastroenterology & Hepatology
GA KA2BR
UT WOS:000505603300002
PM 31567785
DA 2026-05-09
ER

PT J
AU Chavan, R
Singla, V
Sundaram, S
Zanwar, S
Shah, CR
Sud, S
Singh, P
Gandhi, C
Bhatt, P
Goel, A
Rajput, S
AF Chavan, Radhika
Singla, Vikas
Sundaram, Sridhar
Zanwar, Shankar
Shah, Chirag
Sud, Sukrit
Singh, Pankaj
Gandhi, Chaiti
Bhatt, Pratin
Goel, Akash
Rajput, Sanjay
TI Outcomes of endoscopic ultrasound-guided gallbladder drainage: A

multicenter study from India (with video)
SO INDIAN JOURNAL OF GASTROENTEROLOGY
LA English
DT Article
DE Acute cholecystitis; Adverse events; Endoscopic gallbladder drainage;
Endoscopic ultrasound; Gallbladder drainage
ID ACUTE CHOLECYSTITIS
AB BackgroundEndoscopic ultrasound-guided gallbladder drainage (EUS-GBD) offers a safe and minimally invasive alternative for percutaneous cholecystostomy (PCC) in acute cholecystitis patients with high-surgical risk. Additionally, EUS-GBD serves as a rescue biliary drainage in malignant distal biliary obstruction. Despite its widespread application, data within the Indian context remains sparse. This study aims to report the outcomes of EUS-GBD through the first multi-center study from India. MethodsWe retrospectively analyzed patients undergoing EUS-GBD at six tertiary care centers of India from March 2022 to November 2023. EUS-GBD was performed by free hand or over-the-guidewire technique with lumen-apposing metal stent (LAMS) or large caliber metal stent (LCMS). The primary outcome was technical success (defined as successful deployment of stent between gallbladder and stomach/duodenal lumen). The secondary outcomes were clinical success (defined as resolution of symptoms of acute cholecystitis and more than > 50% reduction in bilirubin level within two weeks in distal biliary obstruction), adverse event rate, 30-day mortality rate and 90-day reintervention rate. ResultsTotal 29 patients (mean age 65.86 +/- 12.91, 11 female) underwent EUS-GBD. The indication for EUS-GBD were acute cholecystitis (79.31%) and rescue biliary drainage for malignant distal biliary obstruction (20.69%). LAMS was deployed in 92.86%, predominantly by free-hand technique (78.57%). Technical and clinical success rates were 96.55% and 82.75%, respectively. Adverse events occurred in 27.59% patients, with severe adverse events (bile leak and bleeding) being uncommon (10%). Both 30-day mortality rate and 90-day reintervention rate were 13.79% in patients. Cholecysto-duodenal fistula facilitated cholecystoscopic intervention and stone removal in one patient and transgastric EUS-GBD did not hamper bilio-enteric anastomosis during Whipple surgery in two patients. ConclusionEUS-GBD is a safe and effective technique for managing acute cholecystitis in high-risk patients and for biliary drainage in cases with malignant distal biliary obstruction.
C1 [Chavan, Radhika; Gandhi, Chaiti; Rajput, Sanjay] Ansh Clin, Dept Gastroenterol & Endoscopy, Ahmadabad 380008, India.
[Singla, Vikas; Singh, Pankaj; Goel, Akash] Max Super Special Hosp, Ctr Gastroenterol Hepatol & Endoscopy, New Delhi, India.
[Sundaram, Sridhar] Tata Mem Hosp, Dept Digest Dis & Clin Nutr, Mumbai, India.
[Zanwar, Shankar] Max Nanavati Hosp, Mumbai, Maharashtra, India.
[Shah, Chirag; Bhatt, Pratin] Mission Gastro Hosp, Ahmadabad, Gujarat, India.
[Sud, Sukrit] Inst Digest & Hepatobiliary Sci, Medanta Med, Gurugram, India.
C3 Tata Memorial Centre (TMC); Tata Memorial Hospital
RP Chavan, R (corresponding author), Ansh Clin, Dept Gastroenterol & Endoscopy, Ahmadabad 380008, India.
EM drradhikachavan@gmail.com
RI S, Sridhar/R-6416-2017
CR Anderloni A, 2016, SURG ENDOSC, V30, P5200, DOI 10.1007/s00464-016-4894-x
Armellini E, 2023, WORLD J GASTROENTERO, V29, P3341, DOI 10.3748/wjg.v29.i21.3341
Bang JY, 2024, GUT, V73, P395, DOI 10.1136/gutjnl-2023-331245
Binda C, 2023, GASTROINTEST ENDOSC, V98, P765, DOI 10.1016/j.gie.2023.06.054
Boregowda U, 2023, DIAGNOSTICS, V13, DOI 10.3390/diagnostics13040657

Cotton PB, 2010, GASTROINTEST ENDOSC, V71, P446, DOI 10.1016/j.gie.2009.10.027
Daabiss M, 2011, INDIAN J ANAESTH, V55, P111, DOI 10.4103/0019-5049.79879
Dollhopf M, 2017, GASTROINTEST ENDOSC, V86, P636, DOI 10.1016/j.gie.2017.02.027
Irani SS, 2023, CLIN GASTROENTEROL H, V21, P1141, DOI 10.1016/j.cgh.2022.12.039
Issa D, 2021, ENDOSCOPY, V53, P827, DOI 10.1055/a-1259-0349
Jamwal KD, 2018, J CLIN TRANSL HEPATO, V6, P35, DOI 10.14218/JCTH.2017.00018
Kalva NR, 2018, CAN J GASTROENTEROL, V2018, DOI 10.1155/2018/7070961
Ligresti D, 2019, ENDOSCOPY, V51, P94, DOI 10.1055/a-0754-1847
Lisotti A, 2022, SURG ENDOSC, V36, P569, DOI 10.1007/s00464-021-08318-z
Maruta A, 2020, J HEPATO-BIL-PAN SCI, V27, P429, DOI 10.1002/jhbp.744
Mohan BP, 2020, ENDOSCOPY, V52, P96, DOI 10.1055/a-1020-3932
Ogura T, 2019, DIGEST ENDOSC, V31, P55, DOI 10.1111/den.13334
Osman Karim T, 2023, IGIE, V2, P324, DOI [10.1016/j.igie.2023.07.003, 10.1016/j.igie.2023.07.003]
Perez-Miranda M, 2018, ENDOSC ULTRASOUND, V7, P79, DOI 10.4103/eus.eus_5_18
Podboy A, 2021, GASTROINTEST ENDOSC, V93, P797, DOI 10.1016/j.gie.2020.09.040
Rana SS, 2021, ANN GASTROENTEROL, V34, P142, DOI 10.20524/aog.2020.0568
Saumoy M, 2019, J CLIN GASTROENTEROL, V53, P231, DOI 10.1097/MCG.0000000000001036
Tandon RK., 2000, WORLD J GASTROENTERO, V6, P4, DOI [DOI 10.3748/WJG.V6.ISUPPL3.4, 10.3748/wjg.v6.iSuppl3.4]
Teoh AYB, 2020, GUT, V69, P1085, DOI 10.1136/gutjnl-2019-319996
Teoh AYB, 2021, GASTROINTEST ENDOSC, V93, P577, DOI 10.1016/j.gie.2020.06.066
Teoh AYB, 2017, ENDOSCOPY, V49, P130, DOI 10.1055/s-0042-119036
Tyberg A, 2023, CLIN TRANSL GASTROEN, V14, DOI 10.14309/ctg.0000000000000593
Unisa S, 2011, HPB, V13, P117, DOI 10.1111/j.1477-2574.2010.00255.x
Yokoe M, 2018, J HEPATO-BIL-PAN SCI, V25, P41, DOI 10.1002/jhbp.515
Yue Q, 2023, GASTROENTEROL ENDOSC, V1, P125, DOI [10.1016/j.gande.2023.05.003, 10.1016/j.gande.2023.05.003]
NR 30
TC 2
Z9 3
U1 0
U2 0
PU SPRINGER INDIA
PI NEW DELHI
PA 7TH FLOOR, VIJAYA BUILDING, 17, BARAKHAMBA ROAD, NEW DELHI, 110 001, INDIA
SN 0254-8860
EI 0975-0711
J9 INDIAN J GASTROENTER
JI Indian J. Gastroenterol.
PD DEC
PY 2024

VL 43
IS 6
BP 1184
EP 1193
DI 10.1007/s12664-024-01614-1
EA JUN 2024
PG 10
WC Gastroenterology & Hepatology
WE Emerging Sources Citation Index (ESCI)
SC Gastroenterology & Hepatology
GA K9B4U
UT WOS:001251852400001
PM 38902473
DA 2026-05-09
ER

PT J
AU Yoo, HW
Moon, JH
Lee, YN
Shin, IS
Yang, JK
Lee, TH
Cha, SW
Cho, YD
Park, SH
AF Yoo, Hae Won
Moon, Jong Ho
Lee, Yun Nah
Shin, Il Sang
Yang, Jae Kook
Lee, Tae Hoon
Cha, Sang-Woo
Cho, Young Deok
Park, Sang-Heum
TI Feasibility of Cholangioscopy Using Multibending Ultra-Slim Endoscope
Through a Lumen-Apposing Metal Stent for Choledochoduodenostomy
SO DIGESTIVE DISEASES AND SCIENCES
LA English
DT Article
DE Choledochostomy; Pancreatic neoplasms; Cholangiocarcinoma; Stents;
Endoscopes

ID GUIDED BILIARY DRAINAGE; GUIDELINES

AB Background and Aims Endoscopic ultrasound (EUS) guided choledochoduodenostomy using a lumen-apposing metal stent (LAMS) allows access to the biliary system with a sufficiently large diameter stent. However, an appropriate endoscope for this purpose is required. We evaluated the feasibility of per-oral cholangioscopy (POC) using a multibending ultra-slim endoscope through a LAMS for EUS-guided choledochoduodenostomy. Methods Twelve patients who underwent EUS-guided choledochoduodenostomy, followed by POC via a LAMS, were enrolled. POC was performed with an multibending ultra-slim endoscope. The primary outcome was technical success, defined as examination of both hepatic ducts and either the distal common bile duct or stricture site within 10 min. The types of intervention and adverse events were also assessed. Results Technical success was achieved in all cases with the multibending ultra-slim endoscope. Narrow-band imaging endoscopy was performed in five cases (41.7%) and POC-guided targeted biopsy was performed in three cases (25.0%). Stone extraction during POC was performed in two patients (16.7%) and foreign body removal from the intrahepatic duct was performed in one patient. One patient underwent additional metal stent insertion under direct visualization. After POC, no stent migration or severe adverse events were seen in any case. Conclusions POC using a multibending ultra-slim endoscope for diagnostic or therapeutic purposes can be performed effectively and safely through a LAMS after EUS-guided choledochoduodenostomy.

C1 [Yoo, Hae Won; Moon, Jong Ho; Lee, Yun Nah; Shin, Il Sang] SoonChunHyang Univ, Sch Med, Digest Dis Ctr & Res Inst, Dept Internal Med, 170 Jomaru Ro, Bucheon 14584, South Korea.

[Yang, Jae Kook; Lee, Tae Hoon; Park, Sang-Heum] SoonChunHyang Univ, Sch Med, Digest Dis Ctr & Res Inst, Dept Internal Med, Cheonan, South Korea.

[Cha, Sang-Woo; Cho, Young Deok] SoonChunHyang Univ, Sch Med, Digest Dis Ctr & Res Inst, Dept Internal Med, Seoul, South Korea.

C3 Soonchunhyang University; Soonchunhyang University; Soonchunhyang
University

RP Moon, JH (corresponding author), SoonChunHyang Univ, Sch Med, Digest Dis Ctr & Res Inst, Dept Internal Med, 170 Jomaru Ro, Bucheon 14584, South Korea.

EM jhmoon@schmc.ac.kr

RI ; Shin, Il Sang/HTL-6962-2023; Lee, Tae-Hoon/AAR-3708-2021; Lee,
Yun/J-1260-2014

OI Moon, Jong Ho/0000-0002-3946-9944; Shin, Il Sang/0000-0003-2199-1942;

FU SoonChunHyang University Research Fund

FX None of the other authors have any financial relationships relevant to
this publication. This work was supported, in part, by the SoonChunHyang
University Research Fund.

CR Baars JE, 2018, ENDOSC ULTRASOUND, V7, P4, DOI 10.4103/eus.eus_105_17
Chan SM, 2017, GASTROINTEST ENDOSC, V85, P1225, DOI 10.1016/j.gie.2016.10.014
Chen YI, 2018, GASTROINTEST ENDOSC, V88, P267, DOI 10.1016/j.gie.2018.03.021
Cotton PB, 2010, GASTROINTEST ENDOSC, V71, P446, DOI 10.1016/j.gie.2009.10.027
Hatamaru K, 2019, ENDOSC ULTRASOUND, V8, pS67, DOI 10.4103/eus.eus_60_19
Isayama H, 2019, J HEPATO-BIL-PAN SCI, V26, P249, DOI 10.1002/jhbp.631
Lee YN, 2020, GASTROINTEST ENDOSC, V91, P92, DOI 10.1016/j.gie.2019.08.007
Mandai K, 2020, J HEPATO-BIL-PAN SCI, V27, P437, DOI 10.1002/jhbp.766
Moon JH, 2013, GASTROENTEROLOGY, V144, P276, DOI 10.1053/j.gastro.2012.10.045

Mounzer R, 2017, GASTROINTEST ENDOSC, V85, P509, DOI 10.1016/j.gie.2016.11.020
Mukai S, 2020, DIGEST ENDOSC, V32, pE32, DOI 10.1111/den.13556
Mukai S, 2019, CURR OPIN GASTROEN, V35, P408, DOI 10.1097/MOG.0000000000000565
Mukai S, 2016, GASTROINTEST ENDOSC, V83, P832, DOI 10.1016/j.gie.2015.09.047
Robles-Medranda C, 2018, ENDOSCOPY, V50, P1059, DOI 10.1055/a-0607-2534
Sanchez-Ocaña R, 2016, GASTROINTEST ENDOSC, V83, P466, DOI 10.1016/j.gie.2015.08.052
Teoh AYB, 2018, GUT, V67, P1209, DOI 10.1136/gutjnl-2017-314341
Tsuchiya T, 2018, GASTROINTEST ENDOSC, V87, P1138, DOI 10.1016/j.gie.2017.08.017
Yan L, 2019, ENDOSC ULTRASOUND, V8, P172, DOI 10.4103/eus.eus_108_17
NR 18
TC 3
Z9 3
U1 0
U2 5
PU SPRINGER
PI DORDRECHT
PA VAN GODEWIJCKSTRAAT 30, 3311 GZ DORDRECHT, NETHERLANDS
SN 0163-2116
EI 1573-2568
J9 DIGEST DIS SCI
JI Dig. Dis. Sci.
PD JAN
PY 2023
VL 68
IS 1
BP 268
EP 273
DI 10.1007/s10620-022-07531-7
EA MAY 2022
PG 6
WC Gastroenterology & Hepatology
WE Science Citation Index Expanded (SCI-EXPANDED)
SC Gastroenterology & Hepatology
GA 8K7DP
UT WOS:000805472000002
PM 35616805
DA 2026-05-09
ER

PT J
AU Morita, M
Yokota, T

Yano, R
Amano, M
Ochi, H
Azemoto, N
Mashiba, T
Joko, K
AF Morita, Makoto
Yokota, Tomoyuki
Yano, Ryo
Amano, Michiko
Ochi, Hironori
Azemoto, Nobuaki
Mashiba, Toshie
Joko, Kouji
TI A case of walled-off necrosis extending into the pelvic cavity
successfully treated by endoscopic necrosectomy using a lumen-apposing
metal stent
SO DEN OPEN
LA English
DT Article
DE acute pancreatitis; endoscopic necrosectomy; endoscopic
ultrasound-guided pancreatic cyst drainage; lumen-apposing metal stent;
walled-off necrosis
ID EUS-GUIDED DRAINAGE; INTERVENTIONS; MIGRATION
AB A 31-year-old man developed massive walled-off necrosis extending into the pelvic cavity following severe acute alcoholic pancreatitis. Endoscopic ultrasound-guided fistula drainage was performed using a lumen-apposing metal stent, but this was insufficiently effective, and endoscopic necrosectomy was also performed, after which the patient improved. Percutaneous drainage and surgery are other options for the treatment of walled-off necrosis extending into the pelvic cavity, but a valuable case in which the patient improved with endoscopic treatment alone is presented.
C1 [Morita, Makoto; Yokota, Tomoyuki; Yano, Ryo; Amano, Michiko; Ochi, Hironori; Azemoto, Nobuaki; Mashiba, Toshie; Joko, Kouji] Matsuyama Red Cross Hosp, Ctr Liver Biliary Pancreat Dis, Matsuyama, Ehime, Japan.
C3 Matsuyama Red Cross Hospital
RP Morita, M (corresponding author), Matsuyama Red Cross Hosp, Ctr Liver Biliary Pancreat Dis, 1 Bunkyocho, Matsuyama, Ehime 7908524, Japan.
EM project.of.life20080308@gmail.com
CR Itoi Takao, 2018, Nihon Shokakibyo Gakkai Zasshi, V115, P1046, DOI 10.11405/nisshoshi.115.1046
Garcia-Alonso FJ, 2018, ENDOSCOPY, V50, P386, DOI 10.1055/a-0581-9040
Mukai S, 2015, J GASTROENTEROL, V50, P211, DOI 10.1007/s00535-014-0957-8
Mukai S, 2015, ENDOSCOPY, V47, P47, DOI 10.1055/s-0034-1377966
Mukai S, 2014, GASTROINTEST ENDOSC, V79, P531, DOI 10.1016/j.gie.2013.10.004
Navarrete C, 2011, GASTROINTEST ENDOSC, V73, P609, DOI 10.1016/j.gie.2010.08.015
Nemoto Y, 2017, PANCREATOLOGY, V17, P663, DOI 10.1016/j.pan.2017.07.195

Rana SS, 2015, ENDOSC ULTRASOUND, V4, P208, DOI 10.4103/2303-9027.162999
Varadarajulu S, 2011, GASTROINTEST ENDOSC, V74, P74, DOI 10.1016/j.gie.2011.03.1122
NR 9
TC 2
Z9 3
U1 0
U2 0
PU WILEY
PI HOBOKEN
PA 111 RIVER ST, HOBOKEN 07030-5774, NJ USA
SN 2692-4609
J9 DEN OPEN
JI DEN Open
PD APR
PY 2022
VL 2
IS 1
AR e120
DI 10.1002/deo2.120
PG 4
WC Gastroenterology & Hepatology
WE Emerging Sources Citation Index (ESCI)
SC Gastroenterology & Hepatology
GA O9NX7
UT WOS:001047024900021
PM 35873503
OA Green Submitted, gold
DA 2026-05-09
ER

PT J
AU Artifon, ELA
Safatle-Ribeiro, AV
Ferreira, FC
Poli-de-Figueiredo, L
Rasslan, S
Carnevale, F
Otoch, JP
Sakai, P
Kahaleh, M
AF Artifon, Everson L. A.
Safatle-Ribeiro, Adriana Vaz

Ferreira, Flavio Coelho
Poli-de-Figueiredo, Luiz
Rasslan, Samir
Carnevale, Francisco
Otoch, Jose Pinhata
Sakai, Paulo
Kahaleh, Michel
TI EUS-Guided Antegrade Transhepatic Placement of a Self-Expandable Metal
Stent in Hepatico-Jejunal Anastomosis
SO JOURNAL OF THE PANCREAS
LA English
DT Article
DE Biliary Tract; Drainage; Endoscopy; Gastrointestinal; Endosonography;
Jaundice; Obstructive
AB Context To demonstrate an EUS-guided biliary drainage in patient with gastrointestinal tract modified surgically. Case report An EUS guided access to the left intra hepatic duct, followed by an antegrade passage of a partially self-expandable metal stent that was removed by using an enteroscope, in one patient with hepatico-jejunal anastomosis. There were no early or delayed complications and the procedure was effective in relieving jaundice until the self-expandable metal stent was removed, 3 months later. A cholangiogram was obtained via enteroscopy, after removal of self-expandable metal stent, and found to be normal. The patient had an uneventful evaluation afterwards. Conclusion The indication of these procedures must be made under a multidisciplinary view while sharing information with the patient or legal guardian. EUS-guided biliary drainage is feasible when performed by professionals with expertise in biliopancreatic endoscopy and advanced echo-endoscopy and should be performed currently under rigorous protocol in educational institutions.
C1 [Artifon, Everson L. A.; Safatle-Ribeiro, Adriana Vaz; Ferreira, Flavio Coelho; Poli-de-Figueiredo, Luiz; Rasslan, Samir; Carnevale, Francisco; Otoch, Jose Pinhata; Sakai, Paulo] Univ Sao Paulo, Sao Paulo, SP, Brazil.
[Kahaleh, Michel] Univ Virginia Hlth System, Charlottesville, VA USA.
C3 Universidade de Sao Paulo; University of Virginia; University of
Virginia (UVA) Health System
RP Artifon, ELA (corresponding author), Univ Sao Paulo, Med Sch, Av Dr Eneas Carvalho Aguiar 255,Cerqueira Cesar, BR-05403000 Sao Paulo, SP, Brazil.
EM eartifon@hotmail.com
RI Carnevale, Francisco Cesar/I-6135-2015; Kahaleh, Michel/A-9408-2011;
Ferreira, Flavio Coelho/K-7958-2017; Safatle-Ribeiro, Adriana
Vaz/H-7378-2012
OI Carnevale, Francisco Cesar/0000-0003-2659-9624; Kahaleh,
Michel/0000-0003-0836-6114; Ferreira, Flavio Coelho/0000-0002-8857-2438;
Safatle-Ribeiro, Adriana Vaz/0000-0001-7686-8859
CR Artifon ELA, 2010, J PANCREAS, V11, P597
Becker C D, 1993, J Vasc Interv Radiol, V4, P597, DOI 10.1016/S1051-0443(93)71930-2
Itoi T, 2009, GASTROINTEST ENDOSC, V69, pS8, DOI 10.1016/j.gie.2008.11.003
Kahaleh M, 2006, GASTROINTEST ENDOSC, V64, P52, DOI 10.1016/j.gie.2006.01.063
Kahaleh M, 2008, GASTROINTEST ENDOSC, V67, P446, DOI 10.1016/j.gie.2007.06.057

Maranki J, 2009, ENDOSCOPY, V41, P532, DOI 10.1055/s-0029-1214712
Nguyen-Tang T, 2010, ENDOSCOPY, V42, P232, DOI 10.1055/s-0029-1243858
Savides TJ, 2009, GASTROINTEST ENDOSC, V69, pS3, DOI 10.1016/j.gie.2008.10.060
NR 8
TC 39
Z9 45
U1 0
U2 0
PU E S BURIONI RICERCHE BIBLIOGRAFICHE
PI GENOVA
PA CORSO FIRENZE 41-2, GENOVA, 16136, ITALY
SN 1590-8577
J9 J PANCREAS
JI J. Pancreas
PD NOV
PY 2011
VL 12
IS 6
BP 610
EP 613
PG 4
WC Gastroenterology & Hepatology
WE Emerging Sources Citation Index (ESCI)
SC Gastroenterology & Hepatology
GA V04ZN
UT WOS:000213843000010
PM 22072253
DA 2026-05-09
ER

PT J
AU Pavic, T
Hrabar, D
Kralj, D
Lerotic, I
Ogresta, D
AF Pavic, Tajana
Hrabar, Davor
Kralj, Dominik
Lerotic, Ivan
Ogresta, Doris
TI Candidemia after endoscopic therapy with lumen-apposing metal stent for

pancreatic walled-off necrosis
SO CLINICAL JOURNAL OF GASTROENTEROLOGY
LA English
DT Article
DE Necrotizing pancreatitis; Walled-off necrosis; Endoscopic necrosectomy;
Candida
ID INFECTED NECROTIZING PANCREATITIS; INTRAABDOMINAL FUNGAL-INFECTIONS;
ULTRASOUND-GUIDED DRAINAGE; FLUID COLLECTIONS; ORGAN FAILURE; UP
APPROACH; NECROSECTOMY; MORTALITY; MULTICENTER; IMPACT
AB Necrotizing pancreatitis remains a challenging and unpredictable condition accompanied by various complications. Endoscopic ultrasound-guided transmural drainage and necrosectomy have become the standard treatment for patients with walled-off necrosis (WON). Endoscopic therapy via lumen-apposing metal stents (LAMS) with large diameters has shown success in the management of pancreatic fluid collections, but there are few data on specific complications of that therapy. We report a case of infected WON and concomitant fungemia following LAMS placement and necrosectomy. In addition, a systematic literature review of current related studies has been provided.
C1 [Pavic, Tajana; Hrabar, Davor; Kralj, Dominik; Lerotic, Ivan; Ogresta, Doris] Univ Zagreb, Sch Med, Clin Hosp Ctr Sestre Milosrdnice, Dept Gastroenterol & Hepatol, Zagreb 10000, Croatia.
C3 University of Zagreb
RP Pavic, T (corresponding author), Univ Zagreb, Sch Med, Clin Hosp Ctr Sestre Milosrdnice, Dept Gastroenterol & Hepatol, Zagreb 10000, Croatia.
EM tajana.pavic@gmail.com
RI Kralj, Dominik/AAL-3160-2020; Pavic, Tajana/B-2705-2015
OI Pavic, Tajana/0000-0002-0370-5001
CR Bakker OJ, 2012, JAMA-J AM MED ASSOC, V307, P1053, DOI 10.1001/jama.2012.276
Bang JY, 2017, GUT, V66, P2054, DOI 10.1136/gutjnl-2016-312812
Bang JY, 2017, DIGEST ENDOSC, V29, P83, DOI 10.1111/den.12681
Banks PA, 2013, GUT, V62, P102, DOI 10.1136/gutjnl-2012-302779
Besselink MG, 2009, BRIT J SURG, V96, P267, DOI 10.1002/bjs.6447
Gardner TB, 2009, GASTROINTEST ENDOSC, V69, P1085, DOI 10.1016/j.gie.2008.06.061
Han D, 2018, J CLIN GASTROENTEROL, V52, P835, DOI 10.1097/MCG.0000000000000934
He YM, 2003, WORLD J GASTROENTERO, V9, P2619
Hoerauf A, 1998, CRIT CARE MED, V26, P2010, DOI 10.1097/00003246-199812000-00031
Inoue T, 2015, MEDICINE, V94, DOI 10.1097/MD.0000000000000558
Kochhar R, 2011, J GASTROEN HEPATOL, V26, P952, DOI 10.1111/j.1440-1746.2011.06685.x
Lakhtakia S, 2017, GASTROINTEST ENDOSC, V85, P1243, DOI 10.1016/j.gie.2016.10.037
Leeds JS, 2017, GUT, V66, P1530, DOI 10.1136/gutjnl-2016-313187
Patil R, 2016, ANN GASTROENTEROL, V29, P168, DOI 10.20524/aog.2016.0008
Petrov MS, 2010, GASTROENTEROLOGY, V139, P813, DOI 10.1053/j.gastro.2010.06.010
Rinninella E, 2015, GASTROINTEST ENDOSC, V82, P1039, DOI 10.1016/j.gie.2015.04.006
Schmidt PN, 2014, PANCREATOLOGY, V14, P444, DOI 10.1016/j.pan.2014.09.001
Schwender BJ, 2015, PANCREAS, V44, P805, DOI 10.1097/MPA.0000000000000334
Seifert H, 2009, GUT, V58, P1260, DOI 10.1136/gut.2008.163733

Shah RJ, 2015, CLIN GASTROENTEROL H, V13, P747, DOI 10.1016/j.cgh.2014.09.047
Sharaiha RZ, 2016, CLIN GASTROENTEROL H, V14, P1797, DOI 10.1016/j.cgh.2016.05.011
Siddiqui AA, 2017, GASTROINTEST ENDOSC, V85, P758, DOI 10.1016/j.gie.2016.08.014
Thompson CC, 2016, PANCREATOLOGY, V16, P66, DOI 10.1016/j.pan.2015.12.001
Trikudanathan G, 2011, AM J GASTROENTEROL, V106, P1188, DOI 10.1038/ajg.2010.497
Umapathy C, 2016, J GASTROINTEST SURG, V20, P1844, DOI 10.1007/s11605-016-3264-2
van Brunschot S, 2013, BMC GASTROENTEROL, V13, DOI 10.1186/1471-230X-13-161
Vazquez-Sequeiros E, 2016, GASTROINTEST ENDOSC, V84, P450, DOI 10.1016/j.gie.2016.02.044
Walter D, 2015, ENDOSCOPY, V47, P63, DOI 10.1055/s-0034-1378113
Werge M, 2016, PANCREAS, V45, P1447, DOI 10.1097/MPA.0000000000000675
NR 29
TC 1
Z9 3
U1 0
U2 4
PU SPRINGER JAPAN KK
PI TOKYO
PA CHIYODA FIRST BLDG EAST, 3-8-1 NISHI-KANDA, CHIYODA-KU, TOKYO, 101-0065,
JAPAN
SN 1865-7257
EI 1865-7265
J9 CLIN J GASTROENTEROL
JI Clin. J. Gastroenterol.
PD JUN
PY 2018
VL 11
IS 3
BP 206
EP 211
DI 10.1007/s12328-018-0823-y
PG 6
WC Gastroenterology & Hepatology
WE Emerging Sources Citation Index (ESCI)
SC Gastroenterology & Hepatology
GA GG7XA
UT WOS:000432910500006
PM 29383496
DA 2026-05-09
ER

PT J
AU Jang, DK

AF Jang, Dong Kee
TI Endoscopic ultrasound-guided biliary drainage in malignant distal
biliary obstruction
SO INTERNATIONAL JOURNAL OF GASTROINTESTINAL INTERVENTION
LA English
DT Review
DE Cholestasis; extrahepatic; Drainage; Endoscopic retrograde
cholangiopancreatography; Endosonography
ID DIFFICULT BILIARY; METAL STENT; HEPATICOGASTROSTOMY
AB Endoscopic retrograde cholangiopancreatography is the standard therapeutic approach for malignant distal biliary obstruction (MDBO). However, it can be challenging in patients with surgically altered anatomy, pyloric/duodenal obstruction, or failed biliary cannulation. Since the development of endoscopic ultrasound (EUS), EUS-guided biliary drainage (EUS-BD) has been increasingly performed. The current article provides a concise review of the latest practices and techniques of EUS-BD procedures for MDBO including EUS-assisted rendezvous, EUS-guided antegrade stenting, EUSguided choledochoduodenostomy, and EUS-guided hepaticogastrostomy. Copyright (C) 2022, Society of Gastrointestinal Intervention.
C1 [Jang, Dong Kee] Seoul Natl Univ, Seoul Metropolitan Govt, Boramae MedCtr, Dept Internal Med,Coll Med, Seoul, South Korea.
C3 Seoul National University (SNU)
RP Jang, DK (corresponding author), Seoul Natl Univ, Seoul Metropolitan Govt, Boramae Med Ctr, Dept Internal Med, 20 Boramae Ro 5 Gil, Seoul 07061, South Korea.
EM mapmap05@snu.ac.kr
OI Jang, Dong Kee/0000-0001-6642-6635
CR Amato A, 2021, ENDOSCOPY, V53, P1037, DOI 10.1055/a-1324-7919
Bang JY, 2018, GASTROINTEST ENDOSC, V88, P9, DOI 10.1016/j.gie.2018.03.012
Cho DH, 2017, GASTROINTEST ENDOSC, V85, P1067, DOI 10.1016/j.gie.2016.09.010
De Cassan C, 2017, ENDOSC ULTRASOUND, V6, P329, DOI 10.4103/2303-9027.209869
Garcia-Sumalla Albert, 2021, VideoGIE, V6, P263, DOI [10.1016/j.vgie.2021.03.002, 10.1016/j.vgie.2021.03.002]
Iwashita T, 2012, ENDOSCOPY, V44, P60, DOI 10.1055/s-0030-1256871
Iwashita T, 2018, PLOS ONE, V13, DOI 10.1371/journal.pone.0202445
Iwashita T, 2016, GASTROINTEST ENDOSC, V83, P394, DOI 10.1016/j.gie.2015.04.043
Kunda R, 2016, SURG ENDOSC, V30, P5002, DOI 10.1007/s00464-016-4845-6
Li JS, 2022, FRONT SURG, V9, DOI 10.3389/fsurg.2022.811005
Lyu YX, 2021, DIGEST LIVER DIS, V53, P1247, DOI 10.1016/j.dld.2021.03.029
Mangas-Sanjuan C, 2020, ENDOSCOPY, V52, pE47, DOI 10.1055/a-0991-7763
Martínez B, 2019, ENDOSC INT OPEN, V7, pE1038, DOI 10.1055/a-0918-5931
Martins FP, 2010, ENDOSCOPY, V42, pE126, DOI 10.1055/s-0029-1243911
Maydeo A, 2011, GASTROINTEST ENDOSC, V74, P1308, DOI 10.1016/j.gie.2011.08.047
Misra SP, 2009, GASTROINTEST ENDOSC, V70, P317, DOI 10.1016/j.gie.2008.12.054
Ogura T, 2021, DEN OPEN, V1, DOI 10.1002/deo2.8
Ogura T, 2022, GASTROINTEST ENDOSC, V96, P262, DOI 10.1016/j.gie.2022.03.013
Ogura T, 2022, ENDOSC ULTRASOUND, V11, P75, DOI 10.4103/EUS-D-20-00257

Paik WH, 2018, AM J GASTROENTEROL, V113, P987, DOI 10.1038/s41395-018-0122-8
Park JK, 2018, GASTROINTEST ENDOSC, V88, P277, DOI 10.1016/j.gie.2018.03.015
Sawas T, 2022, ENDOSC ULTRASOUND, V11, P223, DOI 10.4103/EUS-D-21-00031
So H, 2021, J HEPATO-BIL-PAN SCI, V28, P1130, DOI 10.1002/jhbp.1011
Sodarat P, 2021, ENDOSCOPY, V53, pE350, DOI 10.1055/a-1290-7221
Umeda J, 2015, GASTROINTEST ENDOSC, V82, P390, DOI 10.1016/j.gie.2015.02.041
van der Merwe SW, 2022, ENDOSCOPY, V54, P185, DOI 10.1055/a-1717-1391
van Wanrooij RLJ, 2022, ENDOSCOPY, V54, P310, DOI 10.1055/a-1738-6780
NR 27
TC 0
Z9 1
U1 2
U2 8
PU SOC GASTROINTESTINAL INTERVENTION
PI SEOUL
PA 12, HAKDONG-RO 31, GANGNAM-GU, SEOUL, 00000, SOUTH KOREA
SN 2636-0004
EI 2636-0012
J9 INT J GASTROINT INT
JI Int. J. Gastrointest. Interv.
PD JUL
PY 2022
VL 11
IS 3
BP 102
EP 104
DI 10.18528/ijgii220022
PG 3
WC Gastroenterology & Hepatology
WE Emerging Sources Citation Index (ESCI)
SC Gastroenterology & Hepatology
GA 3L8CE
UT WOS:000834989400003
OA Green Submitted, gold
DA 2026-05-09
ER

PT J
AU Coluccio, C
Facciorusso, A
Forti, E
De Nucci, G

Di Mitri, R
Togliani, T
Cugia, L
Ofosu, A
Anderloni, A
Tarantino, I
Fabbri, C
Crinò, SF
AF Coluccio, Chiara
Facciorusso, Antonio
Forti, Edoardo
De Nucci, Germana
Di Mitri, Roberto
Togliani, Thomas
Cugia, Luigi
Ofosu, Andrew
Anderloni, Andrea
Tarantino, Ilaria
Fabbri, Carlo
Crino, Stefano Francesco
CA I Eus Grp
TI Endoscopic ultrasound-guided drainage of pancreatic collections with de
dicate d metal stents: A nationwide, multicenter, propensity
score-matched comparison
SO DIGESTIVE AND LIVER DISEASE
LA English
DT Article
DE Lumen-apposing metal stents; Bi-flanged metal stents; Pancreatic fluid
collection
ID WALLED-OFF NECROSIS; ENHANCED DELIVERY-SYSTEM; FLUID COLLECTIONS;
GALLBLADDER DRAINAGE; CLINICAL-EVALUATION; PLASTIC STENTS; MANAGEMENT;
SAFETY; PSEUDOCYST; EXPERIENCE
AB Background: The new dedicated stents for endoscopic ultrasound (EUS)-guided transluminal drainage of peri-pancreatic fluid collections (PFCs) demonstrated optimal efficacy and safety profiles.Aims: This study aimed to evaluate the safety, technical and clinical success, and recurrence rate of PFCs drained with Lumen Apposing Metal Stent (LAMS) or Bi-Flanged Metal Stent (BFMS).Methods: Data from a multicenter series of PFCs treated with LAMS or BFMS at 30 Italian centers during a 5-year period were retrieved. The rate of adverse events (AEs), technical success, clinical success, PFC recurrence were evaluated. To overcome biases, a 1-to-1 match was created using propensity score analysis.Results: Out of 476 patients, 386 were treated with LAMS and 90 with BFMS, with a median follow-up of 290 days (95% CI 244 to 361). Using propensity score matching, 84 patients were assigned to each group. The incidence of AEs did not differ between the two stents (13.1% versus 15.5%, p = 0.29), mainly bleeding or recurrence rate (4.7% versus 3.5%, p = 1). Technical and clinical success in the BFMS and LAMS groups were 92% versus 95% (p = 0.36) and 91% versus 94% (p =

0.64), respectively.Conclusion: Our study demonstrates that LAMS and BFMS have comparable safety profiles with similar technical and clinical success rates for EUS-guided PFC drainage.(c) 2023 Editrice Gastroenterologica Italiana S.r.l. Published by Elsevier Ltd. All rights reserved.
C1 [Coluccio, Chiara; Fabbri, Carlo] AUSL Romagna, Forli Cesena Hosp, Gastroenterol & Endoscopy Unit, I-47121 Forli, Italy.

[Facciorusso, Antonio] Univ Foggia, Dept Med Sci, Sect Gastroenterol, I-71100 Foggia, Italy.

[Forti, Edoardo] Osped Niguarda Ca Granda, Operat Endoscopy Unit, I-20100 Milan, Italy.

[De Nucci, Germana] Salvini Hosp, Dept Gastroenterol, I-20024 Garbagnate, Italy.

[Di Mitri, Roberto] Arnas Civ Hosp, Gastroenterol & Endoscopy Unit, I-90100 Palermo, Italy.

[Togliani, Thomas] Gastroenterol & Endoscopy Unit, ASST Carlo Poma, I-46100 Mantua, Italy.

[Cugia, Luigi] Gastroenterol & Digest Endoscopy Dept, Azienda Osped Univ Sassari, I-07100 Sassari, Italy.

[Ofosu, Andrew] Univ Cincinnati, Div Digest Dis, Cincinnati, OH 45219 USA.

[Anderloni, Andrea] Univ Pavia, Fdn IRCCS Policlin San Matteo, Dept Internal Med 1, Endoscopy Unit, I-27100 Pavia, Italy.

[Tarantino, Ilaria] Dept Diagnost, Dept Diagnost & Therapeut Serv, Therapeut Serv, I-90100 Palermo, Italy.

[Crino, Stefano Francesco] Univ Hosp Verona, Pancreas Inst, Gastroenterol & Digest Endoscopy Unit, I-37134 Verona, Italy.

C3 AUSL della Romagna; University of Foggia; IRCCS Ca Granda Ospedale Maggiore Policlinico; Ospedale Niguarda Ca' Granda; A.R.N.A.S. Ospedali Civico Di Cristina Benfratelli; Hospital Carlo Poma; University of Sassari; University System of Ohio; University of Cincinnati; University of Pavia; IRCCS Fondazione San Matteo; University of Verona; Azienda Ospedaliera Universitaria Integrata Verona

RP Coluccio, C (corresponding author), AUSL Romagna, Forli Cesena Hosp, Gastroenterol & Endoscopy Unit, I-47121 Forli, Italy.

EM chiara.coluccio@auslromagna.it

RI Di Mitri, Roberto/AAC-7452-2022; TARANTINO, ILARIA/AFS-0726-2022; Facciorusso, Antonio/AAA-5966-2019; Anderloni, Andrea/ABE-9139-2020; de Nucci, Germana/ABE-2040-2020

OI Coluccio, Chiara/0000-0002-8791-7764; TARANTINO, ILARIA/0000-0001-8791-3395; Anderloni, Andrea/0000-0002-1021-0031;
CR Anderloni A, 2021, SURG ENDOSC, V35, P1741, DOI 10.1007/s00464-020-07567-8

Arvanitakis M, 2018, ENDOSCOPY, V50, P524, DOI 10.1055/a-0588-5365

Austin PC, 2008, STAT MED, V27, P2037, DOI 10.1002/sim.3150

Austin PC, 2011, MULTIVAR BEHAV RES, V46, P119, DOI 10.1080/00273171.2011.540480

Banks PA, 2013, GUT, V62, P102, DOI 10.1136/gutjnl-2012-302779

Bapaye A, 2017, DIGEST ENDOSC, V29, P104, DOI 10.1111/den.12704

Bapaye A, 2015, DIGEST ENDOSC, V27, P499, DOI 10.1111/den.12421

Baron TH, 2020, GASTROENTEROLOGY, V158, P67, DOI 10.1053/j.gastro.2019.07.064

Bekkali NLH, 2017, ENDOSC INT OPEN, V5, pE1189, DOI 10.1055/s-0043-120831

Binda C, 2020, EUR J GASTROEN HEPAT, V32, P1401, DOI 10.1097/MEG.0000000000001793

Binmoeller KF, 2011, ENDOSCOPY, V43, P337, DOI 10.1055/s-0030-1256127

Calo NC, 2022, J CAN ASSOC GASTROEN, V5, P68, DOI [10.1093/jcag/gwab024, 10.1093/jcag/gwab024]
Chandran S, 2015, GASTROINTEST ENDOSC, V81, P127, DOI 10.1016/j.gie.2014.06.025
Chandrasekhara V, 2020, ENDOSC INT OPEN, V08, pE1639, DOI 10.1055/a-1243-0092
Cotton PB, 2010, GASTROINTEST ENDOSC, V71, P446, DOI 10.1016/j.gie.2009.10.027
Facciorusso A, 2015, GASTROINTEST ENDOSC, V82, P350, DOI 10.1016/j.gie.2015.01.003
Fugazza A, 2020, GASTROINTEST ENDOSC, V91, P574, DOI 10.1016/j.gie.2019.11.021
Gornals JB, 2013, SURG ENDOSC, V27, P1428, DOI 10.1007/s00464-012-2591-y
Irani S, 2015, GASTROINTEST ENDOSC, V82, P1110, DOI 10.1016/j.gie.2015.05.045
Itoi T, 2013, J HEPATO-BIL-PAN SCI, V20, P403, DOI 10.1007/s00534-012-0551-5
Itoi T, 2012, GASTROINTEST ENDOSC, V75, P870, DOI 10.1016/j.gie.2011.10.020
Karstensen JG, 2023, GUT, V72, P1167, DOI 10.1136/gutjnl-2022-328225
Khan S, 2021, J GASTROEN HEPATOL, V36, P3395, DOI 10.1111/jgh.15658
Lakhtakia S, 2017, GASTROINTEST ENDOSC, V85, P1243, DOI 10.1016/j.gie.2016.10.037
Mukai S, 2015, ENDOSC ULTRASOUND, V4, P120, DOI 10.4103/2303-9027.156738
Nabi Z, 2017, WORLD J GASTROENTERO, V23, P2660, DOI 10.3748/wjg.v23.i15.2660
Parsa N, 2020, ENDOSCOPY, V52, P211, DOI 10.1055/a-1096-3299
Petrone MC, 2018, UNITED EUR GASTROENT, V6, P1363, DOI 10.1177/2050640618785078
Pinto S, 2021, MEDICINA-LITHUANIA, V57, DOI 10.3390/medicina57121305
Rinninella E, 2015, GASTROINTEST ENDOSC, V82, P1039, DOI 10.1016/j.gie.2015.04.006
Sharaiha RZ, 2016, CLIN GASTROENTEROL H, V14, P1797, DOI 10.1016/j.cgh.2016.05.011
Siddiqui A, 2021, ANN GASTROENTEROL, V34, P273, DOI 10.20524/aog.2021.0570
Siddiqui AA, 2016, GASTROINTEST ENDOSC, V83, P699, DOI 10.1016/j.gie.2015.10.020
Takasaki Y, 2023, DIGEST ENDOSC, V35, P96, DOI 10.1111/den.14406
Teoh AYB, 2016, WORLD J GASTRO ENDOS, V8, P310, DOI 10.4253/wjge.v8.i6.310
Tyberg A, 2016, WORLD J GASTROENTERO, V22, P2256, DOI 10.3748/wjg.v22.i7.2256
Venkatachalapathy SV, 2018, ENDOSC INT OPEN, V6, pE259, DOI 10.1055/s-0043-125362
Yamamoto N, 2013, GASTROINTEST ENDOSC, V77, P809, DOI 10.1016/j.gie.2013.01.009
Yao Y, 2019, MEDICINE, V98, DOI 10.1097/MD.0000000000014179
Yoo J, 2017, ENDOSC ULTRASOUND, V6, P131, DOI 10.4103/2303-9027.204814
NR 40
TC 3
Z9 3
U1 0
U2 0
PU ELSEVIER SCIENCE INC
PI NEW YORK
PA STE 800, 230 PARK AVE, NEW YORK, NY 10169 USA
SN 1590-8658
EI 1878-3562
J9 DIGEST LIVER DIS
JI Dig. Liver Dis.
PD JAN

PY 2024
VL 56
IS 1
BP 159
EP 169
DI 10.1016/j.dld.2023.07.012
EA DEC 2023
PG 11
WC Gastroenterology & Hepatology
WE Science Citation Index Expanded (SCI-EXPANDED)
SC Gastroenterology & Hepatology
GA FR9X1
UT WOS:001147710800001
PM 37517962
OA Green Submitted, Bronze
DA 2026-05-09
ER

PT J
AU Vedantam, S
Amin, S
AF Vedantam, Shyam
Amin, Sunil
TI Endoscopic Ultrasound-Guided Biliary Interventions in Liver Disease
SO CLINICS IN LIVER DISEASE
LA English
DT Article
DE Endoscopic ultrasound; EUS; ERCP; Endoscopic retrograde
cholangiopancreatography; Choledocoduodenostomy; Hepaticogastrostomy;
EUS-guided gallbladder drainage; Therapeutic endoscopy
ID HIGH-RISK PATIENTS; ACUTE CHOLECYSTITIS; MALIGNANT DISTAL; DRAINAGE;
ERCP; OBSTRUCTION; MANAGEMENT; THERAPY; CHOLECYSTOSTOMY; CANNULATION
C1 [Vedantam, Shyam] Univ Miami, Dept Med, Miami, FL USA.
[Amin, Sunil] Univ Miami, Dept Med, Div Digest Hlth & Liver Dis, 1120 NW 14th St,Clin Res Bldg,Suite 11145 D-49, Miami, FL 33136 USA.
C3 University of Miami; University of Miami
RP Amin, S (corresponding author), Univ Miami, Dept Med, Div Digest Hlth & Liver Dis, 1120 NW 14th St,Clin Res Bldg,Suite 11145 D-49, Miami, FL 33136 USA.
EM sunil.amin@med.miami.edu
OI Amin, Sunil/0000-0002-3067-4730
CR Anderloni A, 2019, GASTROINTEST ENDOSC, V89, P69, DOI [10.1016/j.gie.2018.04.1746, 10.1016/j.gie.2018.08.047]

Anderloni A, 2017, ENDOSC INT OPEN, V5, pE1100, DOI 10.1055/s-0043-118828
Attasaranya S, 2012, GASTROENT RES PRACT, V2012, DOI 10.1155/2012/680753
Bailey AA, 2008, ENDOSCOPY, V40, P296, DOI 10.1055/s-2007-995566
Bang JY, 2018, GASTROINTEST ENDOSC, V88, P9, DOI 10.1016/j.gie.2018.03.012
Born P, 1998, SCAND J GASTROENTERO, V33, P544
Brimhall B, 2018, CLIN GASTROENTEROL H, V16, P1521, DOI 10.1016/j.cgh.2018.02.021
Chan SM, 2017, GASTROINTEST ENDOSC, V85, P1225, DOI 10.1016/j.gie.2016.10.014
Conio M, 2001, CRIT REV ONCOL HEMAT, V37, P127, DOI 10.1016/S1040-8428(00)00108-6
Dhindsa BS, 2020, ENDOSC ULTRASOUND, V9, P101, DOI 10.4103/eus.eus_80_19
EDLUND G, 1990, AM J SURG, V159, P414, DOI 10.1016/S0002-9610(05)81285-1
El Chafic Abdul H, 2020, Curr Gastroenterol Rep, V22, P62, DOI [10.1007/s11894-020-00800-3, 10.1007/s11894-020-00800-3]
Enochsson L, 2010, GASTROINTEST ENDOSC, V72, P1175, DOI 10.1016/j.gie.2010.07.047
Fujimoto K, 2014, DIGEST ENDOSC, V26, P1, DOI 10.1111/den.12183
Garcia-Sumalla A, 2021, SURG ENDOSC, V35, P4873, DOI 10.1007/s00464-021-08435-9
Giovannini M, 2001, ENDOSCOPY, V33, P898, DOI 10.1055/s-2001-17324
Gomi H, 2018, J HEPATO-BIL-PAN SCI, V25, P3, DOI 10.1002/jhbp.518
Halttunen J, 2014, SCAND J GASTROENTERO, V49, P752, DOI 10.3109/00365521.2014.894120
Han SY, 2019, SCI REP-UK, V9, DOI 10.1038/s41598-019-52993-x
Hatzidakis AA, 2002, EUR RADIOL, V12, P1778, DOI 10.1007/s00330-001-1247-4
Hedjoudje A, 2019, UNITED EUR GASTROENT, V7, P60, DOI 10.1177/2050640618808147
Iwashita T, 2014, CLIN J GASTROENTEROL, V7, P94, DOI 10.1007/s12328-014-0467-5
Kakked G, 2020, ENDOSC ULTRASOUND, V9, P298, DOI 10.4103/eus.eus_10_20
Kalva NR, 2018, CAN J GASTROENTEROL, V2018, DOI 10.1155/2018/7070961
Khan MA, 2016, DIGEST DIS SCI, V61, P684, DOI 10.1007/s10620-015-3933-0
Khoo S, 2020, ENDOSC ULTRASOUND, V9, P369, DOI 10.4103/eus.eus_59_20
Krishnamoorthi R, 2020, SURG ENDOSC, V34, P2866, DOI 10.1007/s00464-020-07484-w
Krishnamoorthi Rajesh, 2017, Curr Treat Options Gastroenterol, V15, P397, DOI [10.1007/s11938-017-0139-x, 10.1007/s11938-017-0139-x]
Lee SS, 2007, GASTROINTEST ENDOSC, V66, P1008, DOI 10.1016/j.gie.2007.03.1080
Luk SWY, 2019, ENDOSCOPY, V51, P722, DOI 10.1055/a-0929-6603
Miller CS, 2019, ENDOSC INT OPEN, V7, pE1563, DOI 10.1055/a-0998-8129
Mohan BP, 2020, ENDOSCOPY, V52, P96, DOI 10.1055/a-1020-3932
Moss AC, 2006, COCHRANE DB SYST REV, DOI 10.1002/14651858.CD004200.pub2
Mutneja HR, 2021, ENDOSCOPY, V53, P357, DOI 10.1055/a-1220-2242
Ogura T, 2021, DIGEST DIS, V39, P165, DOI 10.1159/000510560
Okuno N, 2021, CLIN ENDOSC, V54, P596, DOI 10.5946/ce.2020.194
Paik WH, 2018, AM J GASTROENTEROL, V113, P987, DOI 10.1038/s41395-018-0122-8
Park DH, 2015, J GASTROEN HEPATOL, V30, P1461, DOI 10.1111/jgh.13027
Pécsi D, 2019, DIGEST DIS SCI, V64, P2429, DOI 10.1007/s10620-019-05640-4
Podboy A, 2021, GASTROINTEST ENDOSC, V93, P797, DOI 10.1016/j.gie.2020.09.040
Saad WEA, 2010, J VASC INTERV RADIOL, V21, P789, DOI 10.1016/j.jvir.2010.01.012

Sharaiha RZ, 2017, GASTROINTEST ENDOSC, V85, P904, DOI 10.1016/j.gie.2016.12.023
Teoh AYB, 2020, GUT, V69, P1085, DOI 10.1136/gutjnl-2019-319996
Teoh AYB, 2021, GASTROINTEST ENDOSC, V93, P577, DOI 10.1016/j.gie.2020.06.066
Teoh AYB, 2019, ENDOSC ULTRASOUND, V8, pS40, DOI 10.4103/eus.eus_49_19
Tringali A, 2018, ENDOSCOPY, V50, P631, DOI 10.1055/s-0043-125062
Tyberg A, 2016, GASTROINTEST ENDOSC, V84, P941, DOI 10.1016/j.gie.2016.05.035
Ulusakarya A, 2019, MEDICINE, V98, DOI 10.1097/MD.0000000000015341
Varadarajulu S, 2013, GASTROINTEST ENDOSC, V78, P742, DOI 10.1016/j.gie.2013.06.009
Veitch AM, 2008, GUT, V57, P1322, DOI 10.1136/gut.2007.142497
Vila JJ, 2012, GASTROINTEST ENDOSC, V76, P1133, DOI 10.1016/j.gie.2012.08.001
Vozzo Catherine Frakes, 2020, VideoGIE, V5, P500, DOI [10.1016/j.vgie.2020.07.009, 10.1016/j.vgie.2020.07.009]
Wang KX, 2016, GASTROINTEST ENDOSC, V83, P1218, DOI 10.1016/j.gie.2015.10.033
NR 53
TC 3
Z9 3
U1 0
U2 3
PU W B SAUNDERS CO-ELSEVIER INC
PI PHILADELPHIA
PA 1600 JOHN F KENNEDY BOULEVARD, STE 1800, PHILADELPHIA, PA 19103-2899 USA
SN 1089-3261
EI 1557-8224
J9 CLIN LIVER DIS
JI Clin. Liver Dis.
PD FEB
PY 2022
VL 26
IS 1
BP 101
EP 114
DI 10.1016/j.cld.2021.08.009
EA NOV 2021
PG 14
WC Gastroenterology & Hepatology
WE Science Citation Index Expanded (SCI-EXPANDED)
SC Gastroenterology & Hepatology
GA XO3RP
UT WOS:000730105900010
PM 34802656
DA 2026-05-09
ER

PT J
AU Nunes, N
de Lima, MF
Caldeira, A
Leite, S
Marques, S
Moreira, T
Moutinho-Ribeiro, P
Bispo, M
AF Nunes, Nuno
de Lima, Margarida Flor
Caldeira, Ana
Leite, Silvia
Marques, Susana
Moreira, Teresa
Moutinho-Ribeiro, Pedro
Bispo, Miguel
CA Portuguese Grp Ultrasound Gastroen
TI GRUPUGE PERSPECTIVE: Endoscopic Ultrasound-Guided Biliary Drainage
SO GE PORTUGUESE JOURNAL OF GASTROENTEROLOGY
LA English
DT Article
DE Endoscopic ultrasound; Biliary obstruction; Biliary drainage;
Hepaticogastrostomy; Choledochoduodenostomy; Rendez-vous technique;
Gallbladder drainage
ID INTERNATIONAL MULTICENTER; METAL STENT; CHOLEDOCHODUODENOSTOMY; ACCESS;
OBSTRUCTION
AB Endoscopic ultrasound-guided biliary drainage (EUS-BD) is an alternative to percutaneous and surgical drainage in bile duct obstruction when endoscopic retrograde cholangiopancreatography fails. EUS-BD can be achieved with several techniques, including EUS-guided hepaticogastrostomy (HGS), anterograde transpapillary stent placement, choledochoduodenostomy (CDS), and rendez-vous technique. Lately, with increased experience and development of directed equipment, elevated technical and clinical success as well of lower adverse event rates have been reported. In this article, GRUPUGE presents an updated perspective of the potential role of EUS-guided biliary drainage, addressing the selection criteria and technical issues of different techniques and analyzing recent data on their safety and efficacy.
C1 [Nunes, Nuno; de Lima, Margarida Flor] Hosp Divino Espirito Santo Ponta Delgada, Dept Gastroenterol, Ave D Manuel I, P-9500370 Ponta Delgada, Portugal.
[Caldeira, Ana] Hosp Amato Lusitano, Dept Gastroenterol, Castelo Branco, Portugal.
[Leite, Silvia] Hosp Senhora da Oliveira, Dept Gastroenterol, Guimaraes, Portugal.
[Marques, Susana; Bispo, Miguel] Champalimaud Fdn, Dept Gastroenterol & Digest Endoscopy, Lisbon, Portugal.
[Moreira, Teresa] Ctr Hosp Univ Porto, Dept Gastroenterol, Hosp Santo Antonio, Porto, Portugal.
[Moutinho-Ribeiro, Pedro] Ctr Hosp & Univ Sao Joao, Dept Gastroenterol, Porto, Portugal.
C3 Fundacao Champalimaud; Universidade do Porto

RP Nunes, N (corresponding author), Hosp Divino Espirito Santo Ponta Delgada, Dept Gastroenterol, Ave D Manuel I, P-9500370 Ponta Delgada, Portugal.
EM golfando@gmail.com
RI ; Marques, Susana/E-9291-2010; Nunes, Nuno/GXH-4259-2022
OI Flor de Lima, Margarida/0000-0003-0404-3895; Bispo,
Miguel/0000-0003-0488-3253;
CR Anderloni A, 2019, GASTROINTEST ENDOSC, V89, P69, DOI [10.1016/j.gie.2018.04.1746, 10.1016/j.gie.2018.08.047]
Andriulli A, 2007, AM J GASTROENTEROL, V102, P1781, DOI 10.1111/j.1572-0241.2007.01279.x
Bang JY, 2018, GASTROINTEST ENDOSC, V88, P9, DOI 10.1016/j.gie.2018.03.012
Boulay Brian R, 2018, Gastrointest Endosc Clin N Am, V28, P171, DOI [10.1016/j.giec.2017.11.005, 10.1016/j.giec.2017.11.005]
Brückner S, 2015, ENDOSCOPY, V47, P858, DOI 10.1055/s-0034-1392104
Dhir V, 2014, DIGEST ENDOSC, V26, P430, DOI 10.1111/den.12153
Dhir V, 2012, GASTROINTEST ENDOSC, V75, P354, DOI 10.1016/j.gie.2011.07.075
Dumonceau JM, 2018, ENDOSCOPY, V50, P910, DOI 10.1055/a-0659-9864
Ekkelenkamp VE, 2015, ENDOSCOPY, V47, P504, DOI 10.1055/s-0034-1391231
Hara K, 2013, ENDOSCOPY, V45, P392, DOI 10.1055/s-0032-1326076
Hathorn KE, 2019, ENDOSC INT OPEN, V7, pE1432, DOI 10.1055/a-0990-9488
Hindryckx P, 2019, WORLD J GASTRO ENDOS, V11, P103, DOI 10.4253/wjge.v11.i2.103
Jacques J, 2019, ENDOSCOPY, V51, P540, DOI 10.1055/a-0735-9137
Kawakubo K, 2016, ENDOSCOPY, V48, P164, DOI 10.1055/s-0034-1393179
Khashab MA, 2016, ENDOSC INT OPEN, V4, pE487, DOI 10.1055/s-0042-102648
Khashab MA, 2016, ENDOSC INT OPEN, V4, pE175, DOI 10.1055/s-0041-109083
Kunda R, 2016, SURG ENDOSC, V30, P5002, DOI 10.1007/s00464-016-4845-6
Law Ryan, 2018, Gastrointest Endosc Clin N Am, V28, P187, DOI [10.1016/j.giec.2017.11.006, 10.1016/j.giec.2017.11.006]
Mishra A, 2019, TRANSL GASTROENT HEP, V4, DOI 10.21037/tgh.2019.01.03
Mohan BP, 2020, ENDOSCOPY, V52, P96, DOI 10.1055/a-1020-3932
Mori Y, 2018, J HEPATO-BIL-PAN SCI, V25, P87, DOI 10.1002/jhbp.504
Nakai Y, 2019, CLIN ENDOSC, V52, P212, DOI 10.5946/ce.2018.188
Park DH, 2013, GASTROINTEST ENDOSC, V78, P91, DOI 10.1016/j.gie.2013.01.042
Park DH, 2011, GASTROINTEST ENDOSC, V74, P1276, DOI 10.1016/j.gie.2011.07.054
Park DH, 2010, GASTROINTEST ENDOSC, V71, pAB288, DOI 10.1016/j.gie.2010.03.695
Park JK, 2018, GASTROINTEST ENDOSC, V88, P277, DOI 10.1016/j.gie.2018.03.015
Poincloux L, 2015, ENDOSCOPY, V47, P794, DOI [10.1055/s-0035-1545399, 10.1055/s-0034-1391988]
Teoh AYB, 2020, GUT, V69, P1085, DOI 10.1136/gutjnl-2019-319996
Uemura RS, 2018, J CLIN GASTROENTEROL, V52, P123, DOI 10.1097/MCG.0000000000000948
Weilert F, 2014, SURG ENDOSC, V28, P3193, DOI 10.1007/s00464-014-3588-5
NR 30
TC 8
Z9 8

U1 0
U2 3
PU KARGER
PI BASEL
PA ALLSCHWILERSTRASSE 10, CH-4009 BASEL, SWITZERLAND
SN 2341-4545
EI 2387-1954
J9 GE PORT J GASTROENT
JI GE Port. J. Gastroenterol.
PD APR
PY 2021
VL 28
IS 3
BP 179
EP 184
DI 10.1159/000510026
PG 6
WC Gastroenterology & Hepatology
WE Emerging Sources Citation Index (ESCI)
SC Gastroenterology & Hepatology
GA RY0VK
UT WOS:000647633700004
PM 34056040
OA Green Submitted, gold
DA 2026-05-09
ER

PT J
AU Minaga, K
Kitano, M
AF Minaga, Kosuke
Kitano, Masayuki
TI Recent advances in endoscopic ultrasound-guided biliary drainage
SO DIGESTIVE ENDOSCOPY
LA English
DT Review
DE biliary drainage; endoscopic ultrasound; endoscopic ultrasound-guided
biliary drainage (EUS-BD); interventional EUS; malignant biliary
obstruction
AB Endoscopic ultrasound-guided biliary drainage (EUS-BD) is increasingly used as an alternative in patients with biliary obstruction who fail standard endoscopic retrograde cholangiopancreatography (ERCP). The two major endoscopic approach routes for EUS-BD are the transgastric intrahepatic and the transduodenal extrahepatic approaches. Biliary

drainage can be achieved by three different methods, transluminal biliary stenting, transpapillary rendezvous technique, and antegrade biliary stenting. Choice of approach route and drainage method depends on individual anatomy, underlying disease, and location of the biliary stricture. Recent meta-analyses have revealed that cumulative technical success and adverse event rates were 90-94% and 16-23%, respectively. Development of new dedicated devices for EUS-BD would help refine the technical aspects and minimize the possibility of complications, making it a more promising procedure.
C1 [Minaga, Kosuke] Kindai Univ, Fac Med, Dept Gastroenterol & Hepatol, Osaka, Japan.
[Kitano, Masayuki] Wakayama Med Univ, Sch Med, Dept Internal Med 2, 811-1 Kimiidera, Wakayama, Wakayama 6418509, Japan.
C3 Kindai University (Kinki University); Wakayama Medical University
RP Kitano, M (corresponding author), Wakayama Med Univ, Sch Med, Dept Internal Med 2, 811-1 Kimiidera, Wakayama, Wakayama 6418509, Japan.
EM kitano@wakayama-med.ac.jp
RI Minaga, kosuke/AAD-2107-2020
OI Minaga, kosuke/0000-0001-5407-7925
FU Japan Society for the Promotion of Science
FX THIS STUDY WAS supported by grants from the Japan Society for the
Promotion of Science.
CR Cotton PB, 2011, GASTROINTEST ENDOSC, V73, P868, DOI 10.1016/j.gie.2010.12.036
Cotton PB, 2010, GASTROINTEST ENDOSC, V71, P446, DOI 10.1016/j.gie.2009.10.027
Jorgensen J, 2016, GASTROINTEST ENDOSC, V83, P279, DOI 10.1016/j.gie.2015.11.006
Kimura K, 1969, ENDOSCOPY, V3, P87, DOI [10.1055/s-0028-1098086, DOI 10.1055/S-0028-1098086]
Matsuda K, 2018, DIGEST ENDOSC, V30, P5, DOI 10.1111/den.12964
Schutz SM, 2000, GASTROINTEST ENDOSC, V51, P535, DOI 10.1016/S0016-5107(00)70285-9
NR 6
TC 67
Z9 79
U1 0
U2 6
PU WILEY
PI HOBOKEN
PA 111 RIVER ST, HOBOKEN 07030-5774, NJ USA
SN 0915-5635
EI 1443-1661
J9 DIGEST ENDOSC
JI Dig. Endosc.
PD JAN
PY 2018
VL 30
IS 1
BP 38
EP 47
DI 10.1111/den.12910

PG 10
WC Gastroenterology & Hepatology; Surgery
WE Science Citation Index Expanded (SCI-EXPANDED)
SC Gastroenterology & Hepatology; Surgery
GA FS0RQ
UT WOS:000419481800004
PM 28656640
OA Bronze
DA 2026-05-09
ER

PT J
AU Binda, C
Coluccio, C
Sbrancia, M
Fabbri, C
AF Binda, Cecilia
Coluccio, Chiara
Sbrancia, Monica
Fabbri, Carlo
TI Role of endoscopic ultrasonography in the management of peripancreatic collections
SO MINERVA GASTROENTEROLOGY
LA English
DT Review
DE Endoscopy; Ultrasonography; Pancreatic juice; Pancreatic pseudocyst; Necrosis
ID PANCREATIC-FLUID COLLECTIONS; EUS-GUIDED DRAINAGE; APPOSING METAL STENTS; WALLED-OFF NECROSIS; INFECTED NECROTIZING PANCREATITIS; REVISED ATLANTA CLASSIFICATION; STEP-UP APPROACH; PERCUTANEOUS DRAINAGE; PLASTIC STENTS; TRANSMURAL DRAINAGE
AB Pancreatic fluid collections are surrounded by a wall of granulation tissue and may contain necrotic debris. They occur following a severe acute pancreatitis and most of the cases resolve spontaneously after several weeks. However, their management may lead to a very hard-to-treat condition, requiring a multidisciplinary approach. During the last decades we assisted to a change of paradigm involving Endoscopic Ultrasonography, from a pure diagnostic technique to an interventional-therapeutic one, allowing an effective, safe and less invasive approach than other existing treatment standards, historically consisting of surgical and percutaneous drainage. Treatment of pancreatic fluid collections is indicated if they become infected or symptomatic. Over the past years, exponential developments were done in interventional endoscopic approach, making it the first line suggested modality. The use of endoscopic ultrasound allows assessment of the collection, even when it is not directly bulging on gastrointestinal wall, creation of an internal fistulous tract, checking for surrounding vessels with the use of Doppler, and deployment of a stent avoiding the discomfort of external tubes. Several types of stent have been used for endoscopic drainage: plastic double pigtail stents, fully covered

self-expanding metal stents and, more recently, lumen apposing metal stents, which are considered revolutionary because of their two-side flanges and wide and short internal channel, a new design that made easier direct endoscopic necrosectomy. This review aims to go through currently available literature on the diagnostic and therapeutic role of endoscopic ultrasonography to handle pancreatic fluid collections. (Cite this article as: Binda C, Coluccio C, Sbrancia M, Fabbri C. Role of endoscopic ultrasonography in the management of peripancreatic collections. Minerva Gastroenterol 2022;68:162-76. DOI: 10.23736/S2724-5985.21.02874-6)
C1 [Binda, Cecilia; Coluccio, Chiara; Sbrancia, Monica; Fabbri, Carlo] AUSL Romagna, Forli Cesena Hosp, Gastroenterol & Digest Endoscopy Unit, Forli Cesena, Italy.
C3 AUSL della Romagna
RP Coluccio, C (corresponding author), AUSL Romagna, Forli Cesena Hosp, Gastroenterol & Digest Endoscopy Unit, Forli Cesena, Italy.
EM chiara.coluccio@auslromagna.it
RI Binda, Cecilia/ABH-1588-2020
CR Aburajab M, 2018, GASTROINTEST ENDOSC, V87, P1248, DOI 10.1016/j.gie.2017.11.033
Anderloni A, 2021, SURG ENDOSC, V35, P1741, DOI 10.1007/s00464-020-07567-8
Anderloni A, 2017, ENDOSC INT OPEN, V5, pE25, DOI 10.1055/s-0042-122009
Arvanitakis M, 2004, GASTROENTEROLOGY, V126, P715, DOI 10.1053/j.gastro.2003.12.006
Arvanitakis M, 2018, ENDOSCOPY, V50, P524, DOI 10.1055/a-0588-5365
Azeem N, 2012, J AM COLL SURGEONS, V215, P177, DOI 10.1016/j.jamcollsurg.2012.03.015
Badat N, 2019, EUR RADIOL, V29, P2302, DOI 10.1007/s00330-018-5906-0
Bang JY, 2019, DIGEST ENDOSC, V31, P619, DOI 10.1111/den.13428
Bang JY, 2019, GUT, V68, P1200, DOI 10.1136/gutjnl-2017-315335
Bang JY, 2013, J GASTROEN HEPATOL, V28, P1725, DOI 10.1111/jgh.12328
Banks PA, 2013, GUT, V62, P102, DOI 10.1136/gutjnl-2012-302779
Baron TH, 1996, GASTROENTEROLOGY, V111, P755, DOI 10.1053/gast.1996.v111.pm8780582
Baron TH, 2020, GASTROENTEROLOGY, V158, P67, DOI 10.1053/j.gastro.2019.07.064
Bazerbachi F, 2018, GASTROINTEST ENDOSC, V87, P30, DOI 10.1016/j.gie.2017.08.025
Binda C, 2020, EUR J GASTROEN HEPAT, V32, P1401, DOI 10.1097/MEG.0000000000001793
Binmoeller KF, 2011, ENDOSCOPY, V43, P337, DOI 10.1055/s-0030-1256127
Bollen FL, 2008, BRIT J SURG, V95, P6, DOI 10.1002/bjs.6010
Bouwense SA, 2017, PANCREAS, V46, P850, DOI 10.1097/MPA.0000000000000863
Bradley EL, 2006, PANCREAS, V33, P321
BRADLEY EL, 1993, ARCH SURG-CHICAGO, V128, P586
Chantarojanasiri T, 2018, ENDOSC INT OPEN, V6, pE1398, DOI 10.1055/a-0751-2698
Chen YI, 2019, ENDOSC INT OPEN, V7, pE347, DOI 10.1055/a-0828-7630
Fabbri C, 2020, ENDOSCOPY, V52, pS57
Fabbri C, 2014, WORLD J GASTROENTERO, V20, P8424, DOI 10.3748/wjg.v20.i26.8424
Fugazza A, 2020, GASTROINTEST ENDOSC, V91, P574, DOI 10.1016/j.gie.2019.11.021
Fusaroli P, 2015, ENDOSC ULTRASOUND, V4, P336, DOI 10.4103/2303-9027.170427
Fusaroli P, 2012, DIGEST DIS SCI, V57, P602, DOI 10.1007/s10620-011-1961-y
Gambitta P, 2020, HEPATOB PANCREAT DIS, V19, P68, DOI 10.1016/j.hbpd.2019.09.008
Gardner TB, 2011, GASTROINTEST ENDOSC, V73, P718, DOI 10.1016/j.gie.2010.10.053

Gardner TB, 2009, GASTROINTEST ENDOSC, V69, P1085, DOI 10.1016/j.gie.2008.06.061
Giovannini Marc, 2018, Gastrointest Endosc Clin N Am, V28, P157, DOI 10.1016/j.giec.2017.11.004
Guo JT, 2020, SURG ENDOSC, V34, P1177, DOI 10.1007/s00464-019-06870-3
Guo JT, 2017, ENDOSC ULTRASOUND, V6, P285, DOI 10.4103/eus.eus_85_17
Hao SJ, 2017, WORLD J GASTRO ENDOS, V9, P486, DOI 10.4253/wjge.v9.i9.486
Itoi T, 2012, GASTROINTEST ENDOSC, V75, P870, DOI 10.1016/j.gie.2011.10.020
Jenssen C, 2016, ULTRASCHALL MED, V37, P157, DOI 10.1055/s-0035-1553788
Kadah A, 2020, EUR J GASTROEN HEPAT, V32, P1, DOI 10.1097/MEG.0000000000001571
Kamal A, 2015, ABDOM IMAGING, V40, P1608, DOI 10.1007/s00261-014-0303-x
Kamata K, 2018, DIGEST ENDOSC, V30, P98, DOI 10.1111/den.12900
Keane MG, 2016, SURG ENDOSC, V30, P3730, DOI 10.1007/s00464-015-4668-x
Kedia P, 2015, GASTROINTEST ENDOSC, V81, P1252, DOI 10.1016/j.gie.2014.07.043
Khan MA, 2018, ENDOSC INT OPEN, V6, pE474, DOI 10.1055/s-0044-102299
Kumar N, 2014, PANCREAS, V43, P1334, DOI 10.1097/MPA.0000000000000213
Lang GD, 2018, GASTROINTEST ENDOSC, V87, P150, DOI 10.1016/j.gie.2017.06.029
Lankisch PG, 2015, LANCET, V386, P85, DOI 10.1016/S0140-6736(14)60649-8
Messallam AA, 2021, AM J GASTROENTEROL, V116, P700, DOI 10.14309/ajg.0000000000000987
Morgan DE, 1997, RADIOLOGY, V203, P773, DOI 10.1148/radiology.203.3.9169703
Mukai S, 2015, J GASTROENTEROL, V50, P211, DOI 10.1007/s00535-014-0957-8
Muthusamy VR, 2016, GASTROINTEST ENDOSC, V83, P481, DOI 10.1016/j.gie.2015.11.027
Navarrete C, 2011, GASTROINTEST ENDOSC, V73, P609, DOI 10.1016/j.gie.2010.08.015
Oblizajek N, 2020, GASTROINTEST ENDOSC, V91, P1303, DOI 10.1016/j.gie.2020.01.017
Papachristou GI, 2007, ANN SURG, V245, P943, DOI 10.1097/01.sla.0000254366.19366.69
Parihar V, 2017, EUR J GASTROEN HEPAT, V29, P371, DOI 10.1097/MEG.0000000000000818
Parsa N, 2020, ENDOSCOPY, V52, P211, DOI 10.1055/a-1096-3299
Powers PC, 2019, ENDOSC ULTRASOUND, V8, P194, DOI 10.4103/eus.eus_59_18
Puga M, 2018, ENDOSCOPY, V50, P1022, DOI 10.1055/a-0582-9127
Puli SR, 2014, CAN J GASTROENTEROL, V28, P50, DOI 10.1155/2014/539783
Rana SS, 2019, ANN GASTROENTEROL, V32, P441, DOI 10.20524/aog.2019.0404
Rana SS, 2017, PANCREATOLOGY, V17, P203, DOI 10.1016/j.pan.2017.02.007
Rana SS, 2016, GASTROENTEROL REP, V4, P50, DOI 10.1093/gastro/gou088
Rimbas M, 2019, GUT, V68, P1334, DOI 10.1136/gutjnl-2018-316994
Rinninella E, 2015, GASTROINTEST ENDOSC, V82, P1039, DOI 10.1016/j.gie.2015.04.006
Rizzatti G, 2020, ENDOSCOPY, V52, pE420, DOI 10.1055/a-1151-4694
Rodriguez JR, 2008, ANN SURG, V247, P294, DOI 10.1097/SLA.0b013e31815b6976
Saumoy M, 2018, J CLIN GASTROENTEROL, V52, P458, DOI 10.1097/MCG.0000000000000895
Seewald S, 2009, GASTROINTEST ENDOSC, V69, pS13, DOI 10.1016/j.gie.2008.10.061
Sharaiha RZ, 2016, CLIN GASTROENTEROL H, V14, P1797, DOI 10.1016/j.cgh.2016.05.011
Siddiqui AA, 2017, GASTROINTEST ENDOSC, V85, P758, DOI 10.1016/j.gie.2016.08.014
Siddiqui AA, 2016, GASTROINTEST ENDOSC, V83, P699, DOI 10.1016/j.gie.2015.10.020
Sternby H, 2016, PANCREATOLOGY, V16, P791, DOI 10.1016/j.pan.2016.08.007
Szakó L, 2020, PANCREATOLOGY, V20, P132, DOI 10.1016/j.pan.2019.10.006

Téllez-Avila F, 2015, DIGEST ENDOSC, V27, P762, DOI 10.1111/den.12475
Teoh AYB, 2018, GUT, V67, P1209, DOI 10.1136/gutjnl-2017-314341
Teoh AYB, 2015, ENDOSC INT OPEN, V3, pE130, DOI 10.1055/s-0034-1390890
Thompson CC, 2016, PANCREATOLOGY, V16, P66, DOI 10.1016/j.pan.2015.12.001
Trikudanathan G, 2019, GASTROENTEROLOGY, V156, P1994, DOI 10.1053/j.gastro.2019.01.269
Trikudanathan G, 2018, AM J GASTROENTEROL, V113, P1550, DOI 10.1038/s41395-018-0232-3
Tringali A, 2018, ENDOSC INT OPEN, V6, pE274, DOI 10.1055/s-0043-125313
van Baal MC, 2014, SURGERY, V155, P442, DOI 10.1016/j.surg.2013.10.001
van Brunschot S, 2018, GUT, V67, P697, DOI 10.1136/gutjnl-2016-313341
van Brunschot S, 2018, LANCET, V391, P51, DOI 10.1016/S0140-6736(17)32404-2
van Brunschot S, 2014, SURG ENDOSC, V28, P1425, DOI 10.1007/s00464-013-3382-9
van Brunschot S, 2012, CLIN GASTROENTEROL H, V10, P1190, DOI 10.1016/j.cgh.2012.05.005
van der Wiel SE, 2020, ENDOSC INT OPEN, V8, pE274, DOI 10.1055/a-1079-5015
van Santvoort HC, 2008, PANCREATOLOGY, V8, P593, DOI 10.1159/000161010
Varadarajulu S, 2011, GASTROINTEST ENDOSC, V74, P74, DOI 10.1016/j.gie.2011.03.1122
Wang L, 2021, ENDOSCOPY, V53, P603, DOI 10.1055/a-1213-1489
Wang ZJ, 2019, J GASTROEN HEPATOL, V34, P791, DOI 10.1111/jgh.14557
Watanabe Y, 2017, WORLD J GASTROENTERO, V23, P7110, DOI 10.3748/wjg.v23.i39.7110
Xie LT, 2019, MED SCI MONITOR, V25, P5785, DOI 10.12659/MSM.915193
Yamashita Y, 2019, DIGEST ENDOSC, V31, P125, DOI 10.1111/den.13290
Yan L, 2019, ENDOSC ULTRASOUND, V8, P172, DOI 10.4103/eus.eus_108_17
Yang CJ, 2014, DIGEST LIVER DIS, V46, P446, DOI 10.1016/j.dld.2014.01.158
NR 93
TC 1
Z9 2
U1 0
U2 11
PU EDIZIONI MINERVA MEDICA
PI TURIN
PA CORSO BRAMANTE 83-85 INT JOURNALS DEPT., 10126 TURIN, ITALY
SN 2724-5985
EI 2724-5365
J9 MINERVA GASTROENTERO
JI Minerva Gastroenterol.
PD JUN
PY 2022
VL 68
IS 2
BP 162
EP 176
DI 10.23736/S2724-5985.21.02874-6
PG 15

WC Gastroenterology & Hepatology
WE Science Citation Index Expanded (SCI-EXPANDED)
SC Gastroenterology & Hepatology
GA 2P1CA
UT WOS:000819487200005
PM 33988009
DA 2026-05-09
ER

PT J
AU Sugimoto, M
Takagi, T
Suzuki, R
Waragai, Y
Konno, N
Asama, H
Sato, Y
Irie, H
Nakamura, J
Takasumi, M
Hashimoto, M
Kato, T
Hikichi, T
Ohira, H
AF Sugimoto, Mitsuru
Takagi, Tadayuki
Suzuki, Rei
Waragai, Yuichi
Konno, Naoki
Asama, Hiroyuki
Sato, Yuki
Irie, Hiroki
Nakamura, Jun
Takasumi, Mika
Hashimoto, Minami
Kato, Tsunetaka
Hikichi, Takuto
Ohira, Hiromasa
TI Factors Associated With Successful Fistula Dilation in Endoscopic
Ultrasound-guided Biliary Drainage
SO TECHNIQUES AND INNOVATIONS IN GASTROINTESTINAL ENDOSCOPY
LA English

DT Article
DE Endoscopic ultrasound-guided biliary drainage; EUS-HES; EUS-CDS;
Successful fistula dilation; Guidewire position
ID HEPATICOGASTROSTOMY; EFFICACY; STENT; OBSTRUCTION; MIGRATION; SAFETY
AB BACKGROUND AND AIMS: Endoscopic ultrasound-guided biliary drainage (EUS-BD) is an effective treatment for biliary obstruction comparable to endoscopic transpapillary drainage. However, fistula dilation is a critical step of EUS-BD. In this study, we aimed to identify factors associated with successful fistula dilation. METHODS: This observational study enrolled malignant biliary obstruction patients who underwent EUS-BD between May 2005 and April 2022. The factors (period, past history of gastrectomy, stricture part of biliary duct, method of EUS-BD, diameter of the punctured bile duct, distance of puncture route, needle diameter, guidewire diameter, guidewire position, dilator device, state of the scope and devices) associated with successful fistula dilation were investigated.RESULTS: Deep placement of the guidewire into another segment of the bile duct (from the common bile duct [CBD] to the right or left hepatic duct, from B3 or B2 to the right hepatic duct or CBD) was the only factor significantly associated with successful fistula dilation (odds ratio 33.5; 95% CI 2.2-510, P = 0.01) in multivariate analysis. The other factors were not statistically significant. CONCLUSION: For successful fistula dilation, guidewires should be deeply advanced to another area. In EUS-hepaticoenterostomy, the guidewire should be advanced to the right hepatic duct or CBD. In EUS-choledochoduodenostomy, the guidewire should be advanced to the right or left hepatic duct.
C1 [Sugimoto, Mitsuru; Takagi, Tadayuki; Suzuki, Rei; Konno, Naoki; Asama, Hiroyuki; Sato, Yuki; Irie, Hiroki; Nakamura, Jun; Takasumi, Mika; Hashimoto, Minami; Kato, Tsunetaka; Ohira, Hiromasa] Fukushima Med Univ, Sch Med, Dept Gastroenterol, Okuma, Fukushima, Japan.
[Waragai, Yuichi] Soma Gen Hosp, Dept Gastroenterol, Soma, Japan.
[Nakamura, Jun; Hashimoto, Minami; Kato, Tsunetaka; Hikichi, Takuto] Fukushima Med Univ Hosp, Dept Endoscopy, Okuma, Fukushima, Japan.
[Sugimoto, Mitsuru] Fukushima Med Univ, Sch Med, Dept Gastroenterol, Okuma, Fukushima 9601295, Japan.
RP Sugimoto, M (corresponding author), Fukushima Med Univ, Sch Med, Dept Gastroenterol, Okuma, Fukushima 9601295, Japan.
EM kita335@fmu.ac.jp
RI Nakamura, Jun/ABO-6548-2022; Hikichi, Takuto/AAA-6474-2022; Sugimoto, Mitsuru/HSF-9732-2023; Kato, Tsunetaka/LVA-0860-2024
CR Brückner S, 2015, ENDOSCOPY, V47, P858, DOI 10.1055/s-0034-1392104
Chin JYL, 2020, ENDOSC INT OPEN, V08, pE1633, DOI 10.1055/a-1236-3217
Sanz MD, 2021, SURG ENDOSC, V35, P6754, DOI 10.1007/s00464-020-08179-y
De Davide Laurence, 2019, VideoGIE, V4, P131, DOI [10.1016/j.vgie.2018.11.004, 10.1016/j.vgie.2018.11.004]
Di Mitri R, 2022, SURG ENDOSC, V36, P4553, DOI 10.1007/s00464-021-08808-0
Ginestet C, 2022, SURG ENDOSC, V36, P3365, DOI 10.1007/s00464-021-08653-1
Giovannini M, 2001, ENDOSCOPY, V33, P898, DOI 10.1055/s-2001-17324
Honjo M, 2018, ENDOSC ULTRASOUND, V7, P376, DOI 10.4103/eus.eus_2_18
Iwashita T, 2021, J HEPATOBILIARY PANC
Garcia-Alonso FJ, 2018, ENDOSCOPY, V50, P386, DOI 10.1055/a-0581-9040
Jin Z, 2020, DIGEST ENDOSC, V32, P16, DOI 10.1111/den.13456
Kakked G, 2020, ENDOSC ULTRASOUND, V9, P298, DOI 10.4103/eus.eus_10_20
Kanno Y, 2017, WORLD J GASTRO ENDOS, V9, P304, DOI 10.4253/wjge.v9.i7.304

Martins FP, 2010, ENDOSCOPY, V42, pE126, DOI 10.1055/s-0029-1243911
Miller CS, 2019, ENDOSC INT OPEN, V7, pE1563, DOI 10.1055/a-0998-8129
Moole H, 2017, MEDICINE, V96, DOI 10.1097/MD.0000000000005154
Mukai S, 2017, J HEPATO-BIL-PAN SCI, V24, P537, DOI 10.1002/jhbp.496
Ogura T, 2021, ENDOSCOPY, V53, P369, DOI 10.1055/a-1199-5418
Ogura T, 2022, J HEPATO-BIL-PAN SCI, V29, pE88, DOI 10.1002/jhbp.904
Ogura T, 2019, DIGEST LIVER DIS, V51, P247, DOI 10.1016/j.dld.2018.09.015
Oh D, 2017, THER ADV GASTROENTER, V10, P42, DOI 10.1177/1756283X16671671
Paccard JR, 2022, ENDOSCOPY, V54, pE322, DOI 10.1055/a-1529-5164
Paik WH, 2019, ENDOSC ULTRASOUND, V8, pS44, DOI 10.4103/eus.eus_51_19
Rajadurai A, 2022, J GASTROEN HEPATOL, V37, P179, DOI 10.1111/jgh.15688
Ramírez-Luna MA, 2011, ENDOSCOPY, V43, P826, DOI 10.1055/s-0030-1256406
Sharaiha RZ, 2017, GASTROINTEST ENDOSC, V85, P904, DOI 10.1016/j.gie.2016.12.023
Sportes A, 2018, ENDOSC INT OPEN, V6, pE1395, DOI 10.1055/a-0735-9254
Tarantino I, 2021, ENDOSC INT OPEN, V09, pE110, DOI 10.1055/a-1313-6850
Venkatachalapathy SV, 2021, GASTROINTEST ENDOSC, V94, P321, DOI 10.1016/j.gie.2021.01.029
NR 29
TC 1
Z9 1
U1 0
U2 1
PU ELSEVIER SCIENCE INC
PI NEW YORK
PA STE 800, 230 PARK AVE, NEW YORK, NY 10169 USA
SN 2666-5107
EI 2590-0307
J9 TECH INNOVAT GASTROI
JI Tech. Innovation Gastrointes. Endoscopy
PY 2022
VL 24
IS 4
BP 316
EP 323
DI 10.1016/j.tige.2022.05.003
PG 8
WC Gastroenterology & Hepatology
WE Emerging Sources Citation Index (ESCI)
SC Gastroenterology & Hepatology
GA 5W2ZB
UT WOS:000877787700002
DA 2026-05-09
ER

PT J
AU Alali, A
Mosko, J
May, G
Teshima, C
AF Alali, Ali
Mosko, Jeffrey
May, Gary
Teshima, Christopher
TI Endoscopic Ultrasound-Guided Management of Pancreatic Fluid Collections:
Update and Review of the Literature
SO CLINICAL ENDOSCOPY
LA English
DT Review
DE Endosonography; Pancreatic fluid collection; Pseudocyst; Walled-off
necrosis
ID EXPANDING METAL STENTS; PROSPECTIVE RANDOMIZED-TRIAL; TRANSMURAL
DRAINAGE; NECROTIZING PANCREATITIS; PSEUDOCYST DRAINAGE; TREATMENT
OUTCOMES; PLASTIC STENTS; CLINICAL-OUTCOMES; NATURAL-HISTORY; DUCT
SYNDROME
AB Severe acute pancreatitis is often complicated by the development of pancreatic fluid collections (PFCs), which may be associated with significant morbidity and mortality. It is crucial to accurately classify these collections as a pseudocyst or walled-off necrosis (WON) given significant differences in outcomes and management. Interventions for PFCs have increasingly shifted to less invasive strategies, with endoscopic ultrasound (EUS)-guided methods being shown to be safer and equally effective as more invasive surgical techniques. In recent years, many new developments have improved the safety and efficacy of EUS-guided interventions, such as the introduction of lumen-apposing metal stents (LAMS), direct endoscopic necrosectomy (DEN) and multiple other adjunctive techniques. Despite these developments, treatment of PFCs, and infected WON in particular, continues to be associated with significant morbidity and mortality. In this article, we discuss the EUS-guided management of PFCs while reviewing the latest developments and controversies in the field. We end by summarizing our own approach to managing PFCs.
C1 [Alali, Ali; Mosko, Jeffrey; May, Gary; Teshima, Christopher] Univ Toronto, St Michaels Hosp, Ctr Therapeut Endoscopy & Endoscop Oncol, 16-054 Cardinal Carter Wing,30 Bond St, Toronto, ON M5B 1W8, Canada.
C3 University of Toronto; Saint Michaels Hospital Toronto
RP Teshima, C (corresponding author), Univ Toronto, St Michaels Hosp, Ctr Therapeut Endoscopy & Endoscop Oncol, 16-054 Cardinal Carter Wing,30 Bond St, Toronto, ON M5B 1W8, Canada.
EM teshimac@smh.ca
RI Alali, Ali/JMB-5662-2023
OI Alali, Ali/0000-0003-4174-9977; Teshima,
Christopher/0000-0002-5525-8565; Mosko, Jeffrey/0000-0003-2983-524X
CR [Anonymous], GUT
Arvanitakis M, 2007, GASTROINTEST ENDOSC, V65, P609, DOI 10.1016/j.gie.2006.06.083

Bang JY, 2015, DIGEST ENDOSC, V27, P486, DOI 10.1111/den.12418
Bang JY, 2014, SURG ENDOSC, V28, P2877, DOI 10.1007/s00464-014-3541-7
Banks PA, 2013, GUT, V62, P102, DOI 10.1136/gutjnl-2012-302779
Bapaye A, 2017, DIGEST ENDOSC, V29, P104, DOI 10.1111/den.12704
Baron TH, 2002, GASTROINTEST ENDOSC, V56, P7, DOI 10.1067/mge.2002.125106
Baron TH, 1997, AM J MED, V102, P555
BRADLEY EL, 1979, AM J SURG, V137, P135, DOI 10.1016/0002-9610(79)90024-2
Cui ML, 2014, DIGEST DIS SCI, V59, P1055, DOI 10.1007/s10620-013-2967-4
DEVIERE J, 1995, GASTROINTEST ENDOSC, V42, P445, DOI 10.1016/S0016-5107(95)70048-X
Fabbri C, 2012, ENDOSCOPY, V44, P429, DOI 10.1055/s-0031-1291624
Gardner TB, 2011, GASTROINTEST ENDOSC, V73, P718, DOI 10.1016/j.gie.2010.10.053
Gardner TB, 2009, GASTROINTEST ENDOSC, V69, P1085, DOI 10.1016/j.gie.2008.06.061
Gluck M, 2012, J GASTROINTEST SURG, V16, P248, DOI 10.1007/s11605-011-1759-4
Gomatos IP, 2016, ANN SURG, V263, P992, DOI 10.1097/SLA.0000000000001407
Gornals JB, 2013, SURG ENDOSC, V27, P1428, DOI 10.1007/s00464-012-2591-y
Gouyon B, 1997, GUT, V41, P821, DOI 10.1136/gut.41.6.821
Gurusamy KS, 2016, COCHRANE DB SYST REV, V4, DOI DOI 10.1002/14651858.CD011392.PUB2
Hookey LC, 2006, GASTROINTEST ENDOSC, V63, P635, DOI 10.1016/j.gie.2005.06.028
Itoi T, 2012, GASTROINTEST ENDOSC, V75, P870, DOI 10.1016/j.gie.2011.10.020
Lee BU, 2014, ENDOSCOPY, V46, P1078, DOI 10.1055/s-0034-1390871
Medarapalem JB, 2014, Gastrointest. Endosc, V79, pAB445, DOI [10.1016/j.gie.2014, DOI 10.1016/J.GIE.2014.02.637]]
Mier J, 1997, AM J SURG, V173, P71, DOI 10.1016/S0002-9610(96)00425-4
Mouli VP, 2013, GASTROENTEROLOGY, V144, P333, DOI 10.1053/j.gastro.2012.10.004
Park DH, 2009, ENDOSCOPY, V41, P842, DOI 10.1055/s-0029-1215133
Patra PS, 2014, BRIT J SURG, V101, P1721, DOI 10.1002/bjs.9666
Pelaez-Luna M, 2008, GASTROINTEST ENDOSC, V68, P91, DOI 10.1016/j.gie.2007.11.041
Penn DE, 2012, GASTROINTEST ENDOSC, V76, P679, DOI 10.1016/j.gie.2012.04.457
Rinninella E, 2015, GASTROINTEST ENDOSC, V82, P1039, DOI 10.1016/j.gie.2015.04.006
Ross A, 2010, GASTROINTEST ENDOSC, V71, P79, DOI 10.1016/j.gie.2009.06.037
Seifert H, 2009, GUT, V58, P1260, DOI 10.1136/gut.2008.163733
Shah RJ, 2015, CLIN GASTROENTEROL H, V13, P747, DOI 10.1016/j.cgh.2014.09.047
Sharaiha RZ, 2016, CLIN GASTROENTEROL H, V14, P1797, DOI 10.1016/j.cgh.2016.05.011
Sharaiha RZ, 2015, GASTROINTEST ENDOSC, V82, P822, DOI 10.1016/j.gie.2015.02.035
Shrode CW, 2013, DIGEST LIVER DIS, V45, P129, DOI 10.1016/j.dld.2012.08.026
Siddiqui AA, 2017, GASTROINTEST ENDOSC, V85, P758, DOI 10.1016/j.gie.2016.08.014
Siddiqui AA, 2013, GASTROINTEST ENDOSC, V78, P589, DOI 10.1016/j.gie.2013.03.1337
Smith IB, 2015, ENDOSC INT OPEN, V3, pE154, DOI 10.1055/s-0034-1391481
Talreja JP, 2008, GASTROINTEST ENDOSC, V68, P1199, DOI 10.1016/j.gie.2008.06.015
Tarantino I, 2009, GASTROINTEST ENDOSC, V69, P1401, DOI 10.1016/j.gie.2008.08.018
Trevino JM, 2010, J GASTROEN HEPATOL, V25, P526, DOI 10.1111/j.1440-1746.2009.06109.x
van Brunschot S, 2014, SURG ENDOSC, V28, P1425, DOI 10.1007/s00464-013-3382-9

van Brunschot S, 2012, CLIN GASTROENTEROL H, V10, P1190, DOI 10.1016/j.cgh.2012.05.005
Varadarajulu S, 2008, GASTROINTEST ENDOSC, V68, P1102, DOI 10.1016/j.gie.2008.04.028
Varadarajulu S, 2013, GASTROENTEROLOGY, V145, P583, DOI 10.1053/j.gastro.2013.05.046
Varadarajulu S, 2011, GASTROINTEST ENDOSC, V74, P1408, DOI 10.1016/j.gie.2011.07.049
Varadarajulu S, 2011, J GASTROINTEST SURG, V15, P2080, DOI 10.1007/s11605-011-1621-8
Varadarajulu S, 2011, J GASTROEN HEPATOL, V26, P1504, DOI 10.1111/j.1440-1746.2011.06771.x
Varadarajulu S, 2011, GASTROINTEST ENDOSC, V74, P74, DOI 10.1016/j.gie.2011.03.1122
Vazquez-Sequeiros E, 2016, GASTROINTEST ENDOSC, V84, P450, DOI 10.1016/j.gie.2016.02.044
Yang D, 2016, GASTROINTEST ENDOSC, V83, P720, DOI 10.1016/j.gie.2015.10.040
Yasuda I, 2013, ENDOSCOPY, V45, P627, DOI 10.1055/s-0033-1344027
YEO CJ, 1990, SURG GYNECOL OBSTET, V170, P411
Zheng MW, 2011, HEPATO-GASTROENTEROL, V58, P1106, DOI 10.5754/hge11059
NR 55
TC 37
Z9 47
U1 0
U2 1
PU KOREAN SOC GASTROINTESTINAL ENDOSCOPY
PI SEOUL
PA 2003 LG PALACE, 165-8 DONGGYO-DONG, MAPO-GU, SEOUL, 121-754, SOUTH KOREA
SN 2234-2400
EI 2234-2443
J9 CLIN ENDOSC
JI Clin. Endosc.
PD MAR
PY 2017
VL 50
IS 2
BP 117
EP 125
DI 10.5946/ce.2017.045
PG 9
WC Gastroenterology & Hepatology
WE Emerging Sources Citation Index (ESCI)
SC Gastroenterology & Hepatology
GA FD1IS
UT WOS:000407291600006
PM 28391671
OA Green Submitted, gold
DA 2026-05-09
ER

PT J
AU Park, DH
Choi, JH
Oh, D
Lee, SS
Seo, DW
Lee, SK
Kim, MH
AF Park, Do Hyun
Choi, Jun-Ho
Oh, Dongwook
Lee, Sang Soo
Seo, Dong-Wan
Lee, Sung Koo
Kim, Myung-Hwan
TI Endoscopic Ultrasonography-Guided Ethanol Ablation for Small Pancreatic Neuroendocrine Tumors: Results of a Pilot Study
SO CLINICAL ENDOSCOPY
LA English
DT Article
DE Endosonography; Ethanol; Ablation; Neuroendocrine tumors
ID ULTRASOUND; ALCOHOL; TISSUE
AB Background/Aims: Endoscopic ultrasonography (EUS)-guided ethanol ablation is gaining popularity for the treatment of focal pancreatic lesions. The aim of this study was to evaluate the safety, feasibility, and treatment response after EUS-guided ethanol injection for small pancreatic neuroendocrine tumors (p-NETs).

Methods: This was a retrospective analysis of a prospectively collected database including 11 consecutive patients with p-NETs who underwent EUS-guided ethanol injection.

Results: EUS-guided ethanol injection was successfully performed in 11 patients with 14 tumors. The final diagnosis was based on histology and clinical signs as follows: 10 non-functioning neuroendocrine tumors and four insulinomas. During follow-up (median, 370 days; range, 152 to 730 days), 10 patients underwent clinical follow-up after treatment, and one patient was excluded because of loss to follow-up. A single treatment session with an injection of 0.5 to 3.8 mL of ethanol resulted in complete responses (CRs) at the 3-month radiologic imaging for seven of 13 tumors (response rate, 53.8%). Multiple treatment sessions performed in three tumors with residual viable enhancing tissue increased the number of tumors with CRs to eight of 13 (response rate, 61.5%). Mild pancreatitis occurred in three of 11 patients.

Conclusions: EUS-guided ethanol injection appears to be a safe, feasible, and potentially effective method for treating small p-NETs in patients who are poor surgical candidates.
C1 [Park, Do Hyun; Oh, Dongwook; Lee, Sang Soo; Seo, Dong-Wan; Lee, Sung Koo; Kim, Myung-Hwan] Univ Ulsan, Coll Med, Asan Med Ctr, Dept Internal Med,Div Gastroenterol, 88 Olympic Ro, Seoul 138736, South Korea.
[Choi, Jun-Ho] Dankook Univ, Coll Med, Dept Internal Med, Cheonan, South Korea.
C3 University of Ulsan; Asan Medical Center; Dankook University
RP Park, DH (corresponding author), Univ Ulsan, Coll Med, Asan Med Ctr, Dept Internal Med,Div Gastroenterol, 88 Olympic Ro, Seoul 138736, South Korea.

EM dhpark@amc.seoul.kr

RI Choi, Jah Yeon/AGN-2637-2022

FU Korean Gastrointestinal Endoscopy Foundation

FX This study was supported by a 2013 Weolbong grant from the Korean
Gastrointestinal Endoscopy Foundation (2013 Investigation Grant).

CR Aslanian H, 2005, GASTROINTEST ENDOSC, V62, P723, DOI 10.1016/j.gie.2005.06.048
Bettini R, 2011, SURGERY, V150, P75, DOI 10.1016/j.surg.2011.02.022
Cherenfant J, 2013, SURGERY, V154, P785, DOI 10.1016/j.surg.2013.07.004
Deprez PH, 2008, ACTA GASTRO-ENT BELG, V71, P333
Falconi M, 2006, NEUROENDOCRINOLOGY, V84, P196, DOI 10.1159/000098012
Gelczer RK, 1998, J ULTRAS MED, V17, P531
Giday SA, 2007, ENDOSCOPY, V39, P525, DOI 10.1055/s-2007-966391
Jürgensen C, 2006, GASTROINTEST ENDOSC, V63, P1059, DOI 10.1016/j.gie.2005.10.034
Kitano Masayuki, 2012, Gastrointest Endosc Clin N Am, V22, P349, DOI 10.1016/j.giec.2012.04.013
Larssen TB, 2003, EUR RADIOL, V13, P2627, DOI 10.1007/s00330-003-1923-7
Lencioni R, 2010, J VASC INTERV RADIOL, V21, P1533, DOI 10.1016/j.jvir.2010.06.019
Levy MJ, 2012, GASTROINTEST ENDOSC, V75, P200, DOI 10.1016/j.gie.2011.09.019
Matthes K, 2007, GASTROINTEST ENDOSC, V65, P272, DOI 10.1016/j.gie.2006.04.043
Modlin IM, 2008, LANCET ONCOL, V9, P61, DOI 10.1016/S1470-2045(07)70410-2
O'Grady HL, 2008, EJSO-EUR J SURG ONC, V34, P324, DOI 10.1016/j.ejso.2007.07.209
Öberg K, 2005, BEST PRACT RES CL GA, V19, P753, DOI 10.1016/j.bpg.2005.06.002
Oh HC, 2011, GASTROENTEROLOGY, V140, P172, DOI 10.1053/j.gastro.2010.10.001
Vleggaar FP, 2011, ENDOSCOPY, V43, pE328, DOI 10.1055/s-0030-1256775
Zhang WY, 2013, WORLD J GASTROENTERO, V19, P3397, DOI 10.3748/wjg.v19.i22.3397

NR 19

TC 74

Z9 87

U1 0

U2 3

PU KOREAN SOC GASTROINTESTINAL ENDOSCOPY

PI SEOUL

PA 2003 LG PALACE, 165-8 DONGGYO-DONG, MAPO-GU, SEOUL, 121-754, SOUTH KOREA

SN 2234-2400

EI 2234-2443

J9 CLIN ENDOSC

JI Clin. Endosc.

PD MAR

PY 2015

VL 48

IS 2

BP 158

EP 164

DI 10.5946/ce.2015.48.2.158
PG 7
WC Gastroenterology & Hepatology
WE Emerging Sources Citation Index (ESCI)
SC Gastroenterology & Hepatology
GA VA5KK
UT WOS:000410066200014
PM 25844345
OA gold
DA 2026-05-09
ER

PT J
AU Ban, T
Kubota, Y
Joh, T
AF Ban, Tesshin
Kubota, Yoshimasa
Joh, Takashi
TI Failed endoscopic ultrasound-guided gallbladder drainage across the
duodenal covered metallic stent salvaged by using a forward-viewing
linear echoendoscope
SO DIGESTIVE ENDOSCOPY
LA English
DT Article
AB a video of this article.
C1 [Ban, Tesshin; Kubota, Yoshimasa; Joh, Takashi] Gamagori City Hosp, Dept Gastroenterol & Hepatol, 1-1 Mukaida, Gamagori, Aichi 4438501, Japan.
RP Ban, T (corresponding author), Gamagori City Hosp, Dept Gastroenterol & Hepatol, 1-1 Mukaida, Gamagori, Aichi 4438501, Japan.
EM venzosan0204@gmail.com
CR Hara K, 2024, CLIN ENDOSC, V57, P175, DOI 10.5946/ce.2023.271
Mori Y, 2018, J HEPATO-BIL-PAN SCI, V25, P87, DOI 10.1002/jhbp.504
Ogura T, 2019, DIGEST ENDOSC, V31, P55, DOI 10.1111/den.13334
Tozuka Y, 2024, ENDOSCOPY, V56, pE238, DOI 10.1055/a-2271-4124
van der Merwe SW, 2022, ENDOSCOPY, V54, P185, DOI 10.1055/a-1717-1391
NR 5
TC 0
Z9 0
U1 0
U2 1
PU WILEY

PI HOBOKEN
PA 111 RIVER ST, HOBOKEN 07030-5774, NJ USA
SN 0915-5635
EI 1443-1661
J9 DIGEST ENDOSC
JI Dig. Endosc.
PD DEC
PY 2024
VL 36
IS 12
BP 1389
EP 1390
DI 10.1111/den.14931
EA SEP 2024
PG 2
WC Gastroenterology & Hepatology; Surgery
WE Science Citation Index Expanded (SCI-EXPANDED)
SC Gastroenterology & Hepatology; Surgery
GA P3M1G
UT WOS:001314915200001
PM 39295276
OA Bronze
DA 2026-05-09
ER

PT J
AU Hara, K
Okuno, N
Haba, S
Kuwahara, T
AF Hara, Kazuo
Okuno, Nozomi
Haba, Shin
Kuwahara, Takamichi
TI Forward viewing liner echoendoscopy for therapeutic interventions
SO CLINICAL ENDOSCOPY
LA English
DT Article
DE Endoscopic ultrasonography-guided biliary drainage; Endosonography;
Forward-viewing endoscopic ultrasonography; In- terventional endoscopic
ultrasonography; Therapeutic endoscopic ultrasonography
ID ULTRASOUND-GUIDED CHOLEDOCHODUODENOSTOMY; TRANSMURAL DRAINAGE; STRICTURE

AB Therapeutic endoscopic ultrasonography (EUS) procedures using the forward-viewing convex EUS (FV-EUS) have been reviewed based on the articles reported to date. The earliest reported procedure is the drainage of pancreatic pseudocysts using FV-EUS. However, the study on drainage of pancreatic pseudocysts focused on showing that drainage is possible with FV-EUS rather than leveraging its features. Subsequently, studies describing the characteristics of FV-EUS have been reported. By using FV-EUS in EUS-guided choledochoduodenostomy, double punctures in the gastrointestinal tract can be avoided. In postoperative modified anatomical cases, using the endoscopic function of FV-EUS, procedures such as bile duct drainage from anastomosis, pancreatic duct drainage from the afferent limb, and abscess drainage from the digestive tract have been reported. When a perpendicular puncture to the gastrointestinal tract is required or when there is a need to insert the endoscope deep into the gastrointestinal tract, FV-EUS is considered among the options.
C1 [Hara, Kazuo; Okuno, Nozomi; Haba, Shin; Kuwahara, Takamichi] Aichi Canc Ctr Hosp, Dept Gastroenterol, 1-1 Kanokoden,Chikusa Ku, Nagoya, Aichi 4658681, Japan.
C3 Aichi Cancer Center
RP Hara, K (corresponding author), Aichi Canc Ctr Hosp, Dept Gastroenterol, 1-1 Kanokoden,Chikusa Ku, Nagoya, Aichi 4658681, Japan.
EM khara@aichi-cc.jp
RI ; Okuno, Nozomi/AHE-2009-2022
OI Kuwahara, Takamichi/0000-0001-8348-8926; Okuno, Nozomi/0000-0001-6376-687X; Haba, Shin/0000-0002-7578-5610; HARA, KAZUO/0000-0002-4699-6136
CR Elmunzer BJ, 2010, GASTROINTEST ENDOSC, V72, P611, DOI 10.1016/j.gie.2010.04.053
Elshair M, 2022, ENDOSCOPY, V54, pE516, DOI 10.1055/a-1640-8177
Elshair M, 2021, J HEPATO-BIL-PAN SCI, V28, pE40, DOI 10.1002/jhbp.1002
Fernández-de-Castro C, 2016, REV ESP ENFERM DIG, V108, P680, DOI 10.17235/reed.2016.4395/2016
Fuccio L, 2015, J HEPATO-BIL-PAN SCI, V22, P27, DOI 10.1002/jhbp.181
Hara K, 2013, ENDOSCOPY, V45, P392, DOI 10.1055/s-0032-1326076
Hara K, 2020, J HEPATO-BIL-PAN SCI, V27, P563, DOI 10.1002/jhbp.774
Hara K, 2016, WORLD J GASTROENTERO, V22, P1297, DOI 10.3748/wjg.v22.i3.1297
Hisada Y, 2021, ENDOSCOPY, V53, pE340, DOI 10.1055/a-1290-6561
Hodo Yuji, 2018, VideoGIE, V3, P196, DOI [10.1016/j.vgie.2018.03.009, 10.1016/j.vgie.2018.03.009]
Ishikawa S, 2024, CLIN ENDOSC, V57, P122, DOI 10.5946/ce.2022.149
Ishiwatari H, 2019, DIGEST ENDOSC, V31, pE82, DOI 10.1111/den.13390
Iwai T, 2021, ENDOSC ULTRASOUND, V10, P33, DOI 10.4103/eus.eus_72_20
Kawasaki Y, 2023, ENDOSCOPY, V55, pE233, DOI 10.1055/a-1966-0478
Kawasaki Y, 2022, ENDOSCOPY, V54, pE815, DOI 10.1055/a-1816-7943
Kida M, 2011, ENDOSCOPY, V43, P796, DOI 10.1055/s-0030-1256508
Kida M, 2015, ENDOSCOPY, V47, pE303, DOI 10.1055/s-0034-1392208
Kin T, 2019, DIGEST ENDOSC, V31, pE97, DOI 10.1111/den.13460
Koizumi K, 2023, ENDOSCOPY, V55, pE384, DOI 10.1055/a-1996-0657
Koizumi K, 2022, ENDOSCOPY, V54, pE933, DOI 10.1055/a-1881-4068
Kuraoka Naosuke, 2020, Endosc Int Open, V8, pE861, DOI [10.1055/a-1161-8488, 10.1055/a-1161-8488]
Kuwatani M, 2019, DIGEST ENDOSC, V31, pE18, DOI 10.1111/den.13284
Matsumoto S, 2020, DIGEST ENDOSC, V32, P957, DOI 10.1111/den.13620

Nakaji S, 2015, ENDOSCOPY, V47, pE41, DOI 10.1055/s-0034-1391245
Nakamura K, 2022, ENDOSCOPY, V54, pE75, DOI 10.1055/a-1388-5913
Nakatsubo R, 2022, DIGEST ENDOSC, V34, pE162, DOI 10.1111/den.14421
Ogura T, 2015, GASTROENTEROLOGY, V149, P541, DOI 10.1053/j.gastro.2015.03.055
Okuno N, 2022, ENDOSC ULTRASOUND, V11, P319, DOI 10.4103/EUS-D-21-00154
Rana SS, 2021, ANN GASTROENTEROL, V34, P123, DOI 10.20524/aog.2020.0555
Sadek A, 2023, DIGEST ENDOSC, V35, pe136, DOI 10.1111/den.14655
Shimizu H, 2022, DEN OPEN, V2, DOI 10.1002/deo2.114
Shimizu T, 2020, ENDOSCOPY, V52, pE437, DOI 10.1055/a-1158-8408
Teh JL, 2023, J CLIN MED, V12, DOI 10.3390/jcm12041626
Tomoda T, 2015, CLIN J GASTROENTEROL, V8, P228, DOI 10.1007/s12328-015-0580-0
Trevino JM, 2009, GASTROINTEST ENDOSC, V69, P361, DOI 10.1016/j.gie.2008.09.021
Voermans RP, 2007, GASTROINTEST ENDOSC, V66, P1013, DOI 10.1016/j.gie.2007.02.057
Yokoyama K, 2021, CASE REP GASTROENTER, V15, P28, DOI 10.1159/000510132
Yoshimura Shigenobu, 2021, VideoGIE, V6, P549, DOI [10.1016/j.vgie.2021.09.004, 10.1016/j.vgie.2021.09.004]
NR 38
TC 8
Z9 8
U1 0
U2 3
PU KOREAN SOC GASTROINTESTINAL ENDOSCOPY
PI SEOUL
PA 2003 LG PALACE, 165-8 DONGGYO-DONG, MAPO-GU, SEOUL, 121-754, SOUTH KOREA
SN 2234-2400
EI 2234-2443
J9 CLIN ENDOSC
JI Clin. Endosc.
PD MAR
PY 2024
VL 57
IS 2
BP 175
EP 180
DI 10.5946/ce.2023.271
PG 6
WC Gastroenterology & Hepatology
WE Emerging Sources Citation Index (ESCI)
SC Gastroenterology & Hepatology
GA OK9P9
UT WOS:001207287000003
PM 38419166
OA Green Submitted, gold

DA 2026-05-09
ER

PT J
AU Binda, C
Dabizzi, E
Anderloni, A
Cennamo, V
Fiscaletti, M
Fugazza, A
Jovine, E
Ercolani, G
Gasbarrini, A
Fabbri, C
AF Binda, Cecilia
Dabizzi, Emanuele
Anderloni, Andrea
Cennamo, Vincenzo
Fiscaletti, Marta
Fugazza, Alessandro
Jovine, Elio
Ercolani, Giorgio
Gasbarrini, Antonio
Fabbri, Carlo
TI Single-step endoscopic ultrasound-guided multiple gateway drainage of complex walled-off necrosis with lumen apposing metal stents
SO EUROPEAN JOURNAL OF GASTROENTEROLOGY & HEPATOLOGY
LA English
DT Article
DE endoscopic ultrasound; endoscopic ultrasound-guided drainage; lumen-apposing metal stent; multiple transluminal gateway; walled-off necrosis
ID PERIPANCREATIC FLUID COLLECTIONS; NECROTIZING PANCREATITIS; MANAGEMENT; THERAPY
AB In this study we analyzed the feasibility and safety of single-step multiple transluminal gateway drainage (MTGD) for complex walled-off necrosis (WON) using lumen-apposing metal stents (LAMSs). Six patients underwent endoscopic ultrasound (EUS)-guided MTGD using two LAMSs. Technical success was 100%. The mean procedure time was 29 min. The mean number of direct endoscopic necrosectomy sessions per patient was 2. Two of six patients developed adverse events, which was bleeding in both cases and treated endoscopically and surgically, respectively. The mean hospital stay was 52.5 days. No patients had residual necrosis or WON recurrence. Although the limited number of patients, the single-step MTGD using electrocautery-LAMSs can be considered a feasible and well-tolerated treatment option for patients with complex WON. Nevertheless, larger randomized controlled studies are needed in order to confirm our data and better define

the advantages of this technique.

C1 [Binda, Cecilia; Fabbri, Carlo] AUSL Romagna, Gastroenterol & Digest Endoscopy Unit, Med Dept, Forli Cesena Hosp, Forli, Italy.

[Dabizzi, Emanuele; Cennamo, Vincenzo] AUSL Bologna, Dept Surg, Maggiore Bellaria Hosp, Unit Gastroenterol & Digest Endoscopy, Bologna, Italy.

[Anderloni, Andrea; Fugazza, Alessandro] IRCCS, Humanitas Clin & Res Ctr, Dept Gastroenterol, Digest Endoscopy Unit, Via Manzoni 56, I-20089 Rozzano, MI, Italy.

[Fiscaletti, Marta] AUSL Bologna, Maggiore Bellaria Hosp, Dept Radiol, Bologna, Italy.

[Jovine, Elio] AUSL Bologna, Maggiore Bellaria Hosp, Dept Surg, Bologna, Italy.

[Ercolani, Giorgio] Univ Bologna, Alma Mater Studiorum, S Orsola Malpighi Hosp, Dept Med & Surg Sci,DIMEC, Bologna, Italy.

[Ercolani, Giorgio] AUSL Romagna, Gen & Oncol Surg, Morgagni Pierantoni Hosp, Forli, Italy.

[Gasbarrini, Antonio] Fdn Policlin Univ A Gemelli IRCCS, Unita Operat Complessa Med Interne & Gastroentero, Dipartimento Sci Med & Chirurg, Rome, Italy.

C3 AUSL della Romagna; AUSL di Bologna; IRCCS Humanitas Research Hospital;
AUSL di Bologna; AUSL di Bologna; IRCCS Azienda
Ospedaliero-Universitaria di Bologna; University of Bologna; AUSL della
Romagna; Catholic University of the Sacred Heart; IRCCS Policlinico
Gemelli

RP Binda, C (corresponding author), AUSL Romagna, Gastroenterol & Digest Endoscopy Unit, Med Dept, Forli Cesena Hosp, Forli, Italy.

EM cecilia.binda@gmail.com

RI Gasbarrini, Antonio/AAB-8487-2019; cennamo, Vincenzo/AAC-7601-2022;
Gasbarrini, Antonio/AAB-8487-2019; Ercolani, Giorgio/AAD-1462-2022;
Binda, Cecilia/ABH-1588-2020; Fugazza, Alessandro/ABG-9381-2021;
Anderloni, Andrea/ABE-9139-2020; Fabbri, Carlo/AAC-4467-2022

OI Gasbarrini, Antonio/0000-0002-6230-1779; Gasbarrini,
Antonio/0000-0003-4863-6924; Ercolani, Giorgio/0000-0003-4334-5167;
Fugazza, Alessandro/0000-0003-0485-4903; Anderloni,
Andrea/0000-0002-1021-0031; Fabbri, Carlo/0000-0003-0670-9910

CR Abu Dayyeh BK, 2018, GASTROINTEST ENDOSC, V87, P141, DOI 10.1016/j.gie.2017.04.032
Anderloni A, 2017, ENDOSC INT OPEN, V5, pE1100, DOI 10.1055/s-0043-118828
Arvanitakis M, 2018, ENDOSCOPY, V50, P524, DOI 10.1055/a-0588-5365
Bang JY, 2019, GUT, V68, P1200, DOI 10.1136/gutjnl-2017-315335
Bang JY, 2013, J GASTROEN HEPATOL, V28, P1725, DOI 10.1111/jgh.12328
Banks PA, 2013, GUT, V62, P102, DOI 10.1136/gutjnl-2012-302779
Chen YI, 2018, GASTROINTEST ENDOSC, V88, P267, DOI 10.1016/j.gie.2018.03.021
Fugazza A, 2020, GASTROINTEST ENDOSC, V91, P574, DOI 10.1016/j.gie.2019.11.021
Giovannini Marc, 2018, Gastrointest Endosc Clin N Am, V28, P157, DOI 10.1016/j.giec.2017.11.004
Guo JT, 2017, ENDOSC ULTRASOUND, V6, P376, DOI 10.4103/eus.eus_87_17
Han D, 2018, J CLIN GASTROENTEROL, V52, P835, DOI 10.1097/MCG.0000000000000934
Khalid S, 2018, CUREUS J MED SCIENCE, V10, DOI 10.7759/cureus.2047

Lang GD, 2018, GASTROINTEST ENDOSC, V87, P150, DOI 10.1016/j.gie.2017.06.029
Mukai S, 2015, J GASTROENTEROL, V50, P211, DOI 10.1007/s00535-014-0957-8
Rinninella E, 2015, GASTROINTEST ENDOSC, V82, P1039, DOI 10.1016/j.gie.2015.04.006
Sharaiha RZ, 2016, CLIN GASTROENTEROL H, V14, P1797, DOI 10.1016/j.cgh.2016.05.011
Siddiqui AA, 2016, GASTROINTEST ENDOSC, V83, P699, DOI 10.1016/j.gie.2015.10.020
van Brunschot S, 2018, LANCET, V391, P51, DOI 10.1016/S0140-6736(17)32404-2
van Brunschot S, 2014, SURG ENDOSC, V28, P1425, DOI 10.1007/s00464-013-3382-9
Varadarajulu S, 2011, GASTROINTEST ENDOSC, V74, P74, DOI 10.1016/j.gie.2011.03.1122
NR 20
TC 18
Z9 22
U1 0
U2 0
PU LIPPINCOTT WILLIAMS & WILKINS
PI PHILADELPHIA
PA TWO COMMERCE SQ, 2001 MARKET ST, PHILADELPHIA, PA 19103 USA
SN 0954-691X
EI 1473-5687
J9 EUR J GASTROEN HEPAT
JI Eur. J. Gastroenterol. Hepatol.
PD OCT
PY 2020
VL 32
IS 10
BP 1401
EP 1404
DI 10.1097/MEG.0000000000001793
PG 4
WC Gastroenterology & Hepatology
WE Science Citation Index Expanded (SCI-EXPANDED)
SC Gastroenterology & Hepatology
GA NK8UZ
UT WOS:000567007100020
PM 32804839
DA 2026-05-09
ER

PT J
AU Ali, SE
Benrajab, K
Mardini, H
Su, L

Gabr, M
Frandah, WM
AF Ali, Saad Emhmed
Benrajab, Karim
Mardini, Houssam
Su, Leon
Gabr, Moamen
Frandah, Wesam M.
TI Anchoring lumen-apposing metal stent with coaxial plastic stent for endoscopic ultrasound-guided drainage of pancreatic fluid collections: any benefit?
SO ANNALS OF GASTROENTEROLOGY
LA English
DT Article
DE Endosonography; pancreatic diseases; stents; metals
ID TRANSMURAL DRAINAGE; CLINICAL-OUTCOMES; NECROSIS; EUS; EFFICACY; PSEUDOCYSTS; SAFETY; MANAGEMENT
AB Background Anchoring double-pigtail plastic stents (DPSs) within lumen-apposing metal stents (LAMSs) has been proposed to prevent adverse events during endoscopic drainage of pancreatic fluid collections (PFCs). We sought to compare the outcomes of patients who received LAMSs alone and those who received both LAMSs and anchoring DPSs for drainage of PFCs.

Methods A retrospective study was conducted at the University of Kentucky. Patients with PFCs who underwent endoscopic ultrasound-guided drainage using LAMSs, with or without DPSs, between January 2016 and March 2018 were included. Categorical data were analyzed using chi-square tests, and continuous variables using 2-sample t-tests. Adverse events were defined according to the American Society for Gastrointestinal Endoscopy's Lexicon. The primary outcome was to evaluate the efficacy (PFC resolution), and safety (adverse events) of LAMSs with or without DPSs used to drain PFCs.

Results Fifty-seven patients with PFCs were treated by 2 experienced endoscopists over 26 months. Twenty-one (37%) patients received LAMSs alone, and 36 (63%) received LAMSs plus DPSs. Forty-three patients had walled-off pancreatic necrosis, and 14 patients had pancreatic pseudocyst. Clinical success (resolution of PFCs) was achieved in 15 patients (71.4%) in the LAMSs alone group, and 21 patients (58.3%) with LAMSs plus DPSs (P=0.32). In patients with LAMSs alone, 6 patients (28.6%) had adverse events, while in those with LAMSs plus DPSs, 14 (38.9%) patients had adverse events (P=0.43).

Conclusion No significant difference was identified in fluid resolution or adverse events between patients with LAMSs alone and those with LAMSs plus DPSs.
C1 [Ali, Saad Emhmed] Univ Kentucky, Dept Internal Med, Div Hosp Med, Lexington, KY USA.
[Benrajab, Karim; Mardini, Houssam; Gabr, Moamen; Frandah, Wesam M.] Univ Kentucky, Dept Gastroenterol & Hepatol, Lexington, KY USA.
[Su, Leon] Univ Kentucky, Dept Stat, Coll Arts & Sci, Coll Publ Hlth, Lexington, KY USA.
C3 University of Kentucky; University of Kentucky; University of Kentucky
RP Ali, SE (corresponding author), 800 Rose St, Lexington, KY 40536 USA.
EM saad.ali@uky.edu
RI Gabr, Moamen/C-5545-2018

CR Aburajab M, 2018, GASTROINTEST ENDOSC, V87, P1248, DOI 10.1016/j.gie.2017.11.033
Bang JY, 2017, GUT, V66, P2054, DOI 10.1136/gutjnl-2016-312812
Bang JY, 2015, DIGEST ENDOSC, V27, P486, DOI 10.1111/den.12418
Bang JY, 2013, CLIN ENDOSC, V46, P500, DOI 10.5946/ce.2013.46.5.500
Banks PA, 2013, GUT, V62, P102, DOI 10.1136/gutjnl-2012-302779
Baron TH, 2002, GASTROINTEST ENDOSC, V56, P7, DOI 10.1067/mge.2002.125106
Besselink M, 2013, PANCREATOLOGY, V13, pE1, DOI 10.1016/j.pan.2013.07.063
Chen YI, 2018, GASTROINTEST ENDOSC, V88, P267, DOI 10.1016/j.gie.2018.03.021
Cotton PB, 2010, GASTROINTEST ENDOSC, V71, P446, DOI 10.1016/j.gie.2009.10.027
Gluck M, 2010, CLIN GASTROENTEROL H, V8, P1083, DOI 10.1016/j.cgh.2010.09.010
Gornals JB, 2016, SURG ENDOSC, V30, P2592, DOI 10.1007/s00464-015-4505-2
Gornals JB, 2013, SURG ENDOSC, V27, P1428, DOI 10.1007/s00464-012-2591-y
Itoi T, 2012, GASTROINTEST ENDOSC, V75, P870, DOI 10.1016/j.gie.2011.10.020
Johnson CD, 2004, GUT, V53, P1340, DOI 10.1136/gut.2004.039883
Lang GD, 2018, GASTROINTEST ENDOSC, V87, P150, DOI 10.1016/j.gie.2017.06.029
Peery AF, 2015, GASTROENTEROLOGY, V149, P1731, DOI [10.1053/j.gastro.2015.08.045, 10.1053/j.gastro.2018.08.063]
Puga M, 2018, ENDOSCOPY, V50, P1022, DOI 10.1055/a-0582-9127
Rinninella E, 2015, GASTROINTEST ENDOSC, V82, P1039, DOI 10.1016/j.gie.2015.04.006
Sadik R, 2011, WORLD J GASTROENTERO, V17, P499, DOI 10.3748/wjg.v17.i4.499
Shah RJ, 2015, CLIN GASTROENTEROL H, V13, P747, DOI 10.1016/j.cgh.2014.09.047
Sharaiha RZ, 2016, CLIN GASTROENTEROL H, V14, P1797, DOI 10.1016/j.cgh.2016.05.011
Sharaiha RZ, 2015, GASTROINTEST ENDOSC, V82, P822, DOI 10.1016/j.gie.2015.02.035
Siddiqui AA, 2017, GASTROINTEST ENDOSC, V85, P758, DOI 10.1016/j.gie.2016.08.014
Siddiqui AA, 2016, GASTROINTEST ENDOSC, V83, P699, DOI 10.1016/j.gie.2015.10.020
Singhal S, 2013, CLIN ENDOSC, V46, P506, DOI 10.5946/ce.2013.46.5.506
Talreja JP, 2008, GASTROINTEST ENDOSC, V68, P1199, DOI 10.1016/j.gie.2008.06.015
Varadarajulu S, 2008, GASTROINTEST ENDOSC, V68, P1102, DOI 10.1016/j.gie.2008.04.028
Varadarajulu S, 2011, AM SURGEON, V77, P1650
Vazquez-Sequeiros E, 2016, GASTROINTEST ENDOSC, V84, P450, DOI 10.1016/j.gie.2016.02.044
Yadav D, 2010, NAT REV GASTRO HEPAT, V7, P131, DOI 10.1038/nrgastro.2010.6
NR 30
TC 23
Z9 26
U1 0
U2 1
PU HELLENIC SOC GASTROENTEROLOGY
PI ATHENS
PA DEMOKRATIAS AVE 67, ATHENS, 15451, GREECE
SN 1108-7471
EI 1792-7463
J9 ANN GASTROENTEROL

JI Ann. Gastroenterol.
PY 2019
VL 32
IS 6
BP 620
EP 625
DI 10.20524/aog.2019.0414
PG 6
WC Gastroenterology & Hepatology
WE Emerging Sources Citation Index (ESCI)
SC Gastroenterology & Hepatology
GA JO1BD
UT WOS:000497319500012
PM 31700240
OA Green Submitted, gold
DA 2026-05-09
ER

PT J
AU Hara, K
Yamao, K
Mizuno, N
Hijioka, S
Imaoka, H
Tajika, M
Tanaka, T
Ishihara, M
Okuno, N
Hieda, N
Yoshida, T
Niwa, Y
AF Hara, Kazuo
Yamao, Kenji
Mizuno, Nobumasa
Hijioka, Susumu
Imaoka, Hiroshi
Tajika, Masahiro
Tanaka, Tutomu
Ishihara, Makoto
Okuno, Nozomi
Hieda, Nobuhiro
Yoshida, Tukasa

Niwa, Yasumasa

TI Endoscopic ultrasonography-guided biliary drainage: Who, when, which, and how?

SO WORLD JOURNAL OF GASTROENTEROLOGY

LA English

DT Review

DE Endoscopic ultrasonography; Endoscopic ultrasonography-guided biliary drainage; Interventional endoscopic ultrasonography; Endoscopic ultrasono-graphy-guided choledochoduodenostomy; Endo-scopic ultrasonography-guided rendezvous technique

ID METAL STENT; BALLOON-ENTEROSCOPE; ASSISTED ERCP; OBSTRUCTION; MULTICENTER; PLACEMENT; CHOLEDOCHODUODENOSTOMY; HEPATICOGASTROSTOMY; ACCESS; CHOLANGIOPANCREATOGRAPHY

AB Both endoscopic ultrasonography (EUS)-guided choledochoduodenostomy (EUS-CDS) and EUS-guided hepaticogastrostomy (EUS-HGS) are relatively well established as alternatives to percutaneous transhepatic biliary drainage (PTBD). Both EUS-CDS and EUS-HGS have high technical and clinical success rates (more than 90%) in high-volume centers. Complications for both procedures remain high at 10%-30%. Procedures performed by endoscopists who have done fewer than 20 cases sometimes result in severe or fatal complications. When learning EUS-guided biliary drainage (EUS-BD), we recommend a mentor's supervision during at least the first 20 cases. For inoperable malignant lower biliary obstruction, a skillful endoscopist should perform EUS-BD before EUS-guided rendezvous technique (EUS-RV) and PTBD. We should be select EUS-BD for patients having altered anatomy from malignant tumors before balloon-enteroscope-assisted endoscopic retrograde cholangiopancreatography, EUS-RV, and PTBD. If both EUS-CDS and EUS-HGS are available, we should select EUS-CDS, according to published data. EUS-BD will potentially become a first-line biliary drainage procedure in the near future.

C1 [Hara, Kazuo; Yamao, Kenji; Mizuno, Nobumasa; Hijioka, Susumu; Imaoka, Hiroshi; Okuno, Nozomi; Hieda, Nobuhiro; Yoshida, Tukasa] Aichi Canc Ctr Hosp, Dept Gastroenterol, Nagoya, Aichi 4648681, Japan.

[Tajika, Masahiro; Tanaka, Tutomu; Ishihara, Makoto; Niwa, Yasumasa] Aichi Canc Ctr Hosp, Dept Endoscopy, Nagoya, Aichi 4648681, Japan.

C3 Aichi Cancer Center; Aichi Cancer Center

RP Hara, K (corresponding author), Aichi Canc Ctr Hosp, Dept Gastroenterol, Tikusa Ku, 1-1 Kanokoden, Nagoya, Aichi 4648681, Japan.

EM khara@aichi-cc.jp

RI Okuno, Nozomi/AHE-2009-2022; Hijioka, Susumu/I-1039-2019; Imaoka, Hiroshi/JXN-9765-2024

OI Okuno, Nozomi/0000-0001-6376-687X; Hijioka, Susumu/0000-0002-0394-6876; Imaoka, Hiroshi/0000-0003-0584-0095

CR Artifon ELA, 2015, GASTROINTEST ENDOSC, V81, P950, DOI 10.1016/j.gie.2014.09.047

Artifon ELA, 2012, J CLIN GASTROENTEROL, V46, P768, DOI 10.1097/MCG.0b013e31825f264c

Bapaye A, 2013, UNITED EUR GASTROENT, V1, P285, DOI 10.1177/2050640613490928

Brückner S, 2015, ENDOSCOPY, V47, P858, DOI 10.1055/s-0034-1392104

Dhir V, 2015, GASTROINTEST ENDOSC, V81, P913, DOI 10.1016/j.gie.2014.09.054

Dhir V, 2015, GASTROINTEST ENDOSC, V81, P440, DOI 10.1016/j.gie.2014.09.011

Dhir V, 2013, UNITED EUR GASTROENT, V1, P103, DOI 10.1177/2050640613480145
Dhir V, 2014, DIGEST ENDOSC, V26, P430, DOI 10.1111/den.12153
Fabbri C, 2011, ENDOSCOPY, V43, P438, DOI 10.1055/s-0030-1256097
Gupta K, 2014, J CLIN GASTROENTEROL, V48, P80, DOI 10.1097/MCG.0b013e31828c6822
Hara K, 2013, ENDOSCOPY, V45, P392, DOI 10.1055/s-0032-1326076
Hara K, 2014, DIGEST ENDOSC, V26, P333, DOI 10.1111/den.12201
Inamdar S, 2015, GASTROINTEST ENDOSC, V82, P9, DOI 10.1016/j.gie.2015.02.013
Isayama H, 2013, J HEPATO-BIL-PAN SCI, V20, P413, DOI 10.1007/s00534-012-0577-8
Iwashita T, 2012, ENDOSCOPY, V44, P60, DOI 10.1055/s-0030-1256871
Iwashita T, 2016, GASTROINTEST ENDOSC, V83, P394, DOI 10.1016/j.gie.2015.04.043
Kawakubo K, 2014, J HEPATO-BIL-PAN SCI, V21, P328, DOI 10.1002/jhbp.27
Kawakubo K, 2013, SURG ENDOSC, V27, P3437, DOI 10.1007/s00464-013-2896-5
Khashab MA, 2015, DIGEST DIS SCI, V60, P557, DOI 10.1007/s10620-014-3300-6
Kröner PT, 2015, ENDOSCOPY, V47, pE208, DOI 10.1055/s-0034-1391653
Kumbhari V, 2015, GASTROINTEST ENDOSC, V81, P1015, DOI 10.1016/j.gie.2014.10.019
Lee TH, 2014, WORLD J GASTROENTERO, V20, P5859, DOI 10.3748/wjg.v20.i19.5859
Martins FP, 2010, ENDOSCOPY, V42, pE126, DOI 10.1055/s-0029-1243911
Matsushita M, 2010, AM J GASTROENTEROL, V105, P2294, DOI 10.1038/ajg.2010.255
Ogura T, 2015, WORLD J GASTROENTERO, V21, P820, DOI 10.3748/wjg.v21.i3.820
Okuno N, 2015, ENDOSCOPY, V47, pE311, DOI 10.1055/s-0034-1392314
Paik WH, 2014, WORLD J GASTROENTERO, V20, P5051, DOI 10.3748/wjg.v20.i17.5051
Prachayakul V, 2011, ENDOSCOPY, V43, pE213, DOI 10.1055/s-0030-1256395
Prachayakul Varayu, 2012, J Interv Gastroenterol, V2, P202
Rimbas M, 2015, GASTROINTEST ENDOSC, V82, P405, DOI 10.1016/j.gie.2015.03.1907
Robbins D, 2014, GASTROINTEST ENDOSC, V80, P342, DOI 10.1016/j.gie.2014.03.010
Saxena P, 2015, GASTROINTEST ENDOSC, V81, P1010, DOI 10.1016/j.gie.2014.06.038
Sharaiha R, 2013, GASTROINTEST ENDOSC, V78, P638, DOI 10.1016/j.gie.2013.05.034
Shimatani M, 2009, ENDOSCOPY, V41, P849, DOI 10.1055/s-0029-1215108
Siripun A, 2015, WORLD J GASTRO ENDOS, V7, P283, DOI 10.4253/wjge.v7.i3.283
Song TJ, 2014, GASTROINTEST ENDOSC, V80, P707, DOI 10.1016/j.gie.2014.05.327
Vila JJ, 2012, GASTROINTEST ENDOSC, V76, P1133, DOI 10.1016/j.gie.2012.08.001
Yamauchi H, 2015, WORLD J GASTROENTERO, V21, P6460, DOI 10.3748/wjg.v21.i21.6460
NR 38

TC 85

Z9 97

U1 0

U2 6

PU BAISHIDENG PUBLISHING GROUP INC

PI PLEASANTON

PA 8226 REGENCY DR, PLEASANTON, CA 94588 USA

SN 1007-9327

EI 2219-2840

J9 WORLD J GASTROENTERO
JI World J. Gastroenterol.
PD JAN 21
PY 2016
VL 22
IS 3
BP 1297
EP 1303
DI 10.3748/wjg.v22.i3.1297
PG 7
WC Gastroenterology & Hepatology
WE Science Citation Index Expanded (SCI-EXPANDED)
SC Gastroenterology & Hepatology
GA DB5KD
UT WOS:000368552100032
PM 26811666
OA Green Submitted, Bronze
DA 2026-05-09
ER

PT J
AU Huang, PX
Song, QL
Di, SJ
Fan, Y
Zhang, H
AF Huang, Ping-Xiao
Song, Qi-Long
Di, Shu-Jie
Fan, Yan
Zhang, Heng
TI The Use of Oblique-viewing Endoscopic Ultrasound for Accessing the
Afferent Limb for Endoscopic Ultrasound-guided Biliary Drainage in
Patients with Severe Stenotic Hepaticojejunal Anastomosis: One Case and
Literature Review
SO SURGICAL LAPAROSCOPY ENDOSCOPY & PERCUTANEOUS TECHNIQUES
LA English
DT Review
DE oblique-viewing endoscopic ultrasound; endoscopic ultrasound-guided
biliary drainage; hepaticojejunal anastomosis; surgically altered
anastomosis; anastomotic stricture
ID CHOLEDOCHOJEJUNOSTOMY; STRICTURE; ECHOENDOSCOPE

AB Background:The treatment of hepaticojejunal anastomotic strictures in patients with surgically altered anastomosis is challenging. Endoscopic ultrasound (EUS)-guided biliary drainage is being established as a feasible biliary drainage procedure. How can oblique-viewing endoscopic ultrasound (OV-EUS) safely reach the treatment area in the afferent limb for EUS-guided hepaticojejunostomy? This is a key, meaningful, and challenging question.Methods:A unique case of an OV-EUS-guided hepaticojejunostomy performed in a patient with severe stenotic hepaticojejunal anastomosis was reported, and the relevant literatures were reviewed.Results:There are only 3 previous case reports of EUS-guided transanastomotic drainage using OV-EUS. The above 3 cases reported did not elaborate on the key treatment details of the procedure. Especially how can the OV-EUS safely reach the treatment area in the afferent limb?Conclusions:For patients with severe anastomotic stricture, when the retrograde or antegrade guide wire cannot pass through the stenosis to establish biliary drainage, OV-EUS can safely reach the treatment area in the afferent limb under the guidance of a fluoroscopic view and a guide wire. Thus, an OV-EUS-guided hepaticojejunostomy can be achieved.
C1 [Huang, Ping-Xiao; Song, Qi-Long; Di, Shu-Jie; Fan, Yan; Zhang, Heng] Huazhong Univ Sci & Technol, Cent Hosp Wuhan, Tongji Med Coll, Dept Gastroenterol, Wuhan, Hubei, Peoples R China.

[Fan, Yan] Huazhong Univ Sci & Technol, Cent Hosp Wuhan, Tongji Med Coll, Dept Gastroenterol, 26,Jiangan Dist, Wuhan 430014, Peoples R China.

C3 Huazhong University of Science & Technology; Huazhong University of Science & Technology

RP Fan, Y (corresponding author), Huazhong Univ Sci & Technol, Cent Hosp Wuhan, Tongji Med Coll, Dept Gastroenterol, 26,Jiangan Dist, Wuhan 430014, Peoples R China.

EM wisabilitydom@163.com; wisabilitydom1@163.com; 2575702751@qq.com; 280541077@qq.com; 75123590@qq.com

RI Song, Qilong/IYS-5571-2023; Zhang, Heng/AAD-4450-2020; yanchen, fan/AAF-8658-2019
FU The authors thank the editors and all the anonymous reviewers for their insightful suggestions on this article.
FX The authors thank the editors and all the anonymous reviewers for their insightful suggestions on this article.
CR Arfeen M, 2022, J MED CASES, V13, P183, DOI 10.14740/jmc3842

Boulay Brian R, 2018, Gastrointest Endosc Clin N Am, V28, P171, DOI [10.1016/j.giec.2017.11.005, 10.1016/j.giec.2017.11.005]

Dimou FM, 2016, SURGERY, V160, P691, DOI 10.1016/j.surg.2016.05.021

Gwon DI, 2013, DIGEST DIS SCI, V58, P3270, DOI 10.1007/s10620-013-2784-9

Itoi T, 2015, GASTROINTEST ENDOSC, V81, P1259, DOI 10.1016/j.gie.2014.08.033

Iwai T, 2021, ENDOSC ULTRASOUND, V10, P33, DOI 10.4103/eus.eus_72_20

Kida M, 2015, ENDOSCOPY, V47, pE303, DOI 10.1055/s-0034-1392208

Kikuyama M, 2016, DIGEST ENDOSC, V28, P221, DOI 10.1111/den.12572

Kin T, 2019, DIGEST ENDOSC, V31, pE97, DOI 10.1111/den.13460

Kucukay F, 2012, J VASC INTERV RADIOL, V23, P1347, DOI 10.1016/j.jvir.2012.07.004

Kühn JP, 2010, AM J ROENTGENOL, V195, P851, DOI 10.2214/AJR.09.3461

Nennstiel S, 2015, J CLIN GASTROENTEROL, V49, P764, DOI 10.1097/MCG.0000000000000275

Pizzicannella M, 2020, ENDOSC ULTRASOUND, V9, P45, DOI 10.4103/eus.eus_55_19

Rimbas M, 2017, ENDOSC ULTRASOUND, V6, pS122, DOI 10.4103/eus.eus_78_17

Sharaiha RZ, 2016, SURG ENDOSC, V30, P5500, DOI 10.1007/s00464-016-4913-y
Uemura RS, 2018, J CLIN GASTROENTEROL, V52, P123, DOI 10.1097/MCG.0000000000000948
Yamauchi H, 2019, DIGEST DIS SCI, V64, P3557, DOI 10.1007/s10620-019-05811-3
NR 17
TC 0
Z9 0
U1 0
U2 3
PU LIPPINCOTT WILLIAMS & WILKINS
PI PHILADELPHIA
PA TWO COMMERCE SQ, 2001 MARKET ST, PHILADELPHIA, PA 19103 USA
SN 1530-4515
EI 1534-4908
J9 SURG LAPARO ENDO PER
JI Surg. Laparosc. Endosc. Pct. Tech.
PD OCT
PY 2023
VL 33
IS 5
BP 565
EP 570
DI 10.1097/SLE.0000000000001199
PG 6
WC Surgery
WE Science Citation Index Expanded (SCI-EXPANDED)
SC Surgery
GA T9CN3
UT WOS:001080891500022
PM 37523516
OA Green Submitted, hybrid
DA 2026-05-09
ER

PT J
AU Kida, M
Itoi, T
AF Kida, Mitsuhiro
Itoi, Takao
TI CURRENT STATUS AND FUTURE PERSPECTIVE OF INTERVENTIONAL ENDOSCOPIC
ULTRASOUND IN JAPAN
SO DIGESTIVE ENDOSCOPY
LA English

DT Article
CT 10th Endoscopy Forum Japan
CY AUG 02-03, 2008
CL Otaru, JAPAN
DE biliary drainage; endoscopic ultrasonography; fine needle aspiration;
fine needle injection; pseudocyst drainage
ID ULTRASONOGRAPHY
AB Vilmann and Grimm made the first reports of endoscopic ultrasound (EUS)-fine needle aspiration (FNA), and since then EUS-FNA has become popular in the clinical fields, especially in Western countries. Furthermore interventional EUS such as pseudocyst drainage, EUS-guided biliary drainage, celiac plexus neurolysis, and dendritic cell injection, etc. have been introduced.

We have investigated the current status and future perspectives of interventional EUS in Japan. Standardization of EUS-guided pseudocyst drainage has been achieved, but EUS-guided biliary dranage is still controversial, and EUS-fine needle injection (FNI) including EUS-celiac plexus neurolysis (CPN) and dendritic cell injection have been under investigation. In any case, EUS-FNA seems to be a promising future technique and new applications have to be invented.
C1 [Kida, Mitsuhiro] Kitasato Univ, East Hosp, Dept Gastroenterol, Kanagawa 2288520, Japan.
[Itoi, Takao] Tokyo Med Coll, Dept Gastroenterol, Tokyo 160, Japan.
C3 Kitasato University; Tokyo Medical University
RP Kida, M (corresponding author), Kitasato Univ, East Hosp, Dept Gastroenterol, 2-1-1 Asamizodai, Kanagawa 2288520, Japan.
EM m-kida@kitasato-u.ac.jp
CR GIOVANNINI M, 1995, ENDOSCOPY, V27, P171, DOI 10.1055/s-2007-1005657
GRIMM H, 1992, GASTROINTEST ENDOSC, V38, P170, DOI 10.1016/S0016-5107(92)70384-8
Irisawa A, 2007, PANCREAS, V35, P189, DOI 10.1097/01.mpa.0000250141.25639.e9
Kida M, 2008, NEW CHALLENGES IN GASTROINTESTINAL ENDOSCOPY, P506, DOI 10.1007/978-4-431-78889-8_53
VILMANN P, 1992, GASTROINTEST ENDOSC, V38, P172, DOI 10.1016/S0016-5107(92)70385-X
Vosoghi Mehrdad, 2002, MedGenMed, V4, P2
Wiersema MJ, 1996, GASTROINTEST ENDOSC, V44, P656, DOI 10.1016/S0016-5107(96)70047-0
NR 7
TC 3
Z9 4
U1 0
U2 0
PU WILEY-BLACKWELL PUBLISHING, INC
PI MALDEN
PA COMMERCE PLACE, 350 MAIN ST, MALDEN 02148, MA USA
SN 0915-5635
J9 DIGEST ENDOSC
JI Dig. Endosc.
PD JUL
PY 2009

VL 21
BP S50
EP S52
DI 10.1111/j.1443-1661.2009.00862.x
PG 3
WC Gastroenterology & Hepatology; Surgery
WE Science Citation Index Expanded (SCI-EXPANDED)
SC Gastroenterology & Hepatology; Surgery
GA 460RR
UT WOS:000267205300013
PM 19691735
DA 2026-05-09
ER

PT J
AU Zhang, LY
Kunda, R
Aerts, M
Messaoudi, N
Pawa, R
Pawa, S
Robles-Medranda, C
Oleas, R
Al-Haddad, MA
Obaitan, I
Muniraj, T
Fabbri, C
Binda, C
Anderloni, A
Tarantino, I
Bejjani, M
Ghandour, B
Singh, V
Khashab, MA
AF Zhang, Linda Y.
Kunda, Rastislav
Aerts, Maridi
Messaoudi, Nouredin
Pawa, Rishi
Pawa, Swati
Robles-Medranda, Carlos
Oleas, Roberto

Al-Haddad, Mohammad A.
Obaitan, Itegbemie
Muniraj, Thiruvengadam
Fabbri, Carlo
Binda, Cecilia
Anderloni, Andrea
Tarantino, Ilaria
Bejjani, Michael
Ghandour, Bachir
Singh, Vikesh
Khashab, Mouen A.
TI Novel 15-mm-long lumen-apposing metal stent for endoscopic ultrasound-guided drainage of pancreatic fluid collections located ≥10 mm from the luminal wall
SO ENDOSCOPY
LA English
DT Article
ID MANAGEMENT
AB Background Endoscopic ultrasound (EUS)-guided drainage of pancreatic fluid collections (PFCs) by cautery-enhanced lumen-apposing metal stents (LAMS) has largely been limited to collections located Methods This international, multicenter study analyzed all adults with PFCs located >= 10 mm from the luminal wall who were treated by EUS-guided drainage using the 15-mm-long cautery-enhanced LAMS. The primary outcome was technical success. Secondary outcomes included clinical success (decrease in PFC size by >= 50 % at 30 days and resolution of clinical symptoms without surgical intervention), complications, and recurrence. Results 35 patients (median age 57 years; interquartile range [IQR] 47-64 years; 49 % male) underwent novel LAMS placement for drainage of PFCs (26 walled-off necrosis, 9 pseudocysts), measuring 85 mm (IQR 64-117) maximal diameter and located 11.8 mm (IQR 10-12.3; range 10-14) from the gastric/duodenal wall. Technical and clinical success were high (both 97 %), with recurrence in one patient (3 %) at a median follow-up of 123 days (58-236). Three complications occurred (9 %; one mild, two moderate). Conclusions The 15-mm-long cautery-enhanced LAMS was feasible and safe for drainage of PFCs located 10-14 mm from the luminal wall.
C1 [Zhang, Linda Y.; Bejjani, Michael; Ghandour, Bachir; Singh, Vikesh; Khashab, Mouen A.] Johns Hopkins Med, Dept Gastroenterol & Hepatol, Baltimore, MD USA.
[Kunda, Rastislav] Vrije Univ Brussel, Univ Ziekenhuis Brussel, Dept Surg, Dept Gastroenterol Hepatol,Dept Adv Intervent End, Brussels, Belgium.
[Aerts, Maridi] Vrije Univ Brussel VUB, Dept Gastroenterol & Hepatol, Univ Ziekenhuis Brussel UZB, Brussels, Belgium.
[Messaoudi, Nouredin] Vrije Univ Brussel VUB, Dept Surg, Univ Ziekenhuis Brussel UZB, Brussels, Belgium.
[Pawa, Rishi; Pawa, Swati] Wake Forest Baptist Med Ctr, Dept Gastroenterol, Winston Salem, NC USA.
[Robles-Medranda, Carlos; Oleas, Roberto] Inst Ecuatoriano Enfermedades Digest, Endoscopy Div, Guayaquil, Ecuador.
[Al-Haddad, Mohammad A.; Obaitan, Itegbemie] Indiana Univ Sch Med, Dept Gastroenterol & Hepatol, Indianapolis, IN 46202 USA.
[Muniraj, Thiruvengadam] Yale Univ, Sch Med, Dept Gastroenterol & Hepatol, New Haven, CT USA.

[Fabbri, Carlo; Binda, Cecilia] AUSL Romagna, Gastroenterol & Digest Endoscopy Unit, Forli Cesena Hosp, Forli Cesena, Italy.

[Anderloni, Andrea] Humanitas Clin & Res Ctr IRCCS, Div Gastroenterol, Digest Endoscopy Unit, Milan, Italy.

[Tarantino, Ilaria] IRCCS ISMETT, Endoscopy Serv, Dept Diagnost & Therapeut Serv, Palermo, Italy.

C3 Johns Hopkins University; Johns Hopkins Medicine; Vrije Universiteit Brussel; University Hospital Brussels; Vrije Universiteit Brussel; University Hospital Brussels; Vrije Universiteit Brussel; University Hospital Brussels; Wake Forest University; Wake Forest Baptist Medical Center; Indiana University System; Indiana University Bloomington; Yale University; AUSL della Romagna; IRCCS Istituto Mediterraneo per i Trapianti e Terapie ad Alta Specializzazione (ISMETT)

RP Khashab, MA (corresponding author), Johns Hopkins Univ, Dept Gastroenterol & Hepatol, 1800 Orleans St, Baltimore, MD 21287 USA.

EM mkhasha1@jhmi.edu

RI Binda, Cecilia/ABH-1588-2020; Tarantino, Ilaria/AFS-0726-2022; Anderloni, Andrea/ABE-9139-2020; Kunda, Rastislav/N-1775-2019; Messaoudi, Nouredin/KHW-7611-2024; Obaitan, Itegbemie/HTQ-5001-2023; Fabbri, Carlo/AAC-4467-2022; khashab, mouen/F-6643-2017; Robles-Medranda, Carlos/AFY-9788-2022

OI Messaoudi, Nouredin/0000-0003-2799-2604; Alhaddad, Mohammad/0000-0003-1641-9976; Obaitan, Itegbemie/0000-0001-5002-6821; Fabbri, Carlo/0000-0003-0670-9910; Robles-Medranda, Carlos/0000-0003-2434-3369

CR Arvanitakis M, 2018, ENDOSCOPY, V50, P524, DOI 10.1055/a-0588-5365

Bang JY, 2019, GUT, V68, P1200, DOI 10.1136/gutjnl-2017-315335

Bang JY, 2017, GUT, V66, P2054, DOI 10.1136/gutjnl-2016-312812

Banks PA, 2013, GUT, V62, P102, DOI 10.1136/gutjnl-2012-302779

Baron TH, 2020, GASTROENTEROLOGY, V158, P67, DOI 10.1053/j.gastro.2019.07.064

Bekkali NLH, 2017, ENDOSC INT OPEN, V5, pE1189, DOI 10.1055/s-0043-120831

Chandran S, 2015, GASTROINTEST ENDOSC, V81, P127, DOI 10.1016/j.gie.2014.06.025

Chen YI, 2018, ENDOSC INT OPEN, V6, pE780, DOI 10.1055/a-0611-5082

Cotton PB, 2010, GASTROINTEST ENDOSC, V71, P446, DOI 10.1016/j.gie.2009.10.027

Garcia-Alonso FJ, 2018, ENDOSCOPY, V50, P386, DOI 10.1055/a-0581-9040

Lang GD, 2018, GASTROINTEST ENDOSC, V87, P150, DOI 10.1016/j.gie.2017.06.029

Muthusamy VR, 2016, GASTROINTEST ENDOSC, V83, P481, DOI 10.1016/j.gie.2015.11.027

Puga M, 2018, ENDOSCOPY, V50, P1022, DOI 10.1055/a-0582-9127

Ross AS, 2014, GASTROINTEST ENDOSC, V79, P929, DOI 10.1016/j.gie.2013.10.014

Teoh AYB, 2020, DIGEST ENDOSC, V32, P391, DOI 10.1111/den.13494

Varadarajulu S, 2011, GASTROINTEST ENDOSC, V74, P74, DOI 10.1016/j.gie.2011.03.1122

Yang JL, 2019, ENDOSCOPY, V51, P1035, DOI 10.1055/a-0759-1353

NR 17

TC 8

Z9 9
U1 0
U2 2
PU GEORG THIEME VERLAG KG
PI STUTTGART
PA Oswald-Hesse-Strasse 50, D-70469 STUTTGART, GERMANY
SN 0013-726X
EI 1438-8812
J9 ENDOSCOPY
JI Endoscopy
PD JUL
PY 2022
VL 54
IS 07
BP 706
EP 711
DI 10.1055/a-1682-7095
EA DEC 2021
PG 6
WC Gastroenterology & Hepatology; Surgery
WE Science Citation Index Expanded (SCI-EXPANDED)
SC Gastroenterology & Hepatology; Surgery
GA 2Z1AL
UT WOS:000729957900001
PM 34905796
DA 2026-05-09
ER

PT J
AU Chandan, S
Mohan, BP
Khan, SR
Kassab, LL
Ponnada, S
Ofosu, A
Bhat, I
Singh, S
Adler, DG
AF Chandan, Saurabh
Mohan, Babu P.
Khan, Shahab R.
Kassab, Lena L.

Ponnada, Suresh
Ofosu, Andrew
Bhat, Ishfaq
Singh, Shailender
Adler, Douglas G.
TI Efficacy and safety of endoscopic ultrasound-guided pancreatic duct drainage (EUS-PDD): A systematic review and meta-analysis of 714 patients
SO ENDOSCOPY INTERNATIONAL OPEN
LA English
DT Review
ID FAILED ERCP; CHOLANGIOPANCREATOGRAPHY; MULTICENTER; OBSTRUCTION; PANCREATOGRAPHY; OUTCOMES; STENT
AB Background and study aims Endoscopic ultrasound guided pancreatic duct drainage (EUS-PDD) is a minimal-invasive therapeutic option to surgery and in patients with failed endoscopic retrograde pancreatography (ERP). The aim of this review was to quantitatively appraise the clinical outcomes of EUS-PDD by meta-analysis methods. Methods We searched multiple databases from inception through March 2020 to identify studies that reported on EUS-PDD. Pooled rates of technical success, successful drainage of pancreatic duct, clinical success, and adverse events were calculated. Study heterogeneity was assessed using I-2% and 95% prediction interval. Results A total of 22 studies (714 patients) were included. The pooled rate of technical success in EUS-PDD was 84.8% (95% CI 79.1-89.2). The pooled rate of successful PD drained by EUS-PDD was 77.5% (95% CI 63.1-87.4). The pooled rate of clinical success of EUS-PDD was 89.2% (95% CI 82.1-93.7). The pooled rate of all adverse events was 18.1% (95% CI 14.2-22.9). On sub-group analysis, the pooled technical success and clinical success of EUS-PDD from Japanese data were considerably superior (91.2%, 83-95.6 & 92.5%, 83.9-96.7, respectively). The pooled rate of post EUS-PDD acute pancreatitis was 6.6% (95% CI 4.5-9.4), bleeding was 4.1% (95% CI 2.7-6.2), perforation and/or pneumoperitoneum was 3.1% (95% CI 1.9-5), pancreatic leak and/or pancreatic fluid collection was 2.3% (95% CI 1.4-4), and infection was 2.8% (95% CI 1.7-4.6).

Conclusion EUS-PDD demonstrates high technical success and clinical success rates with acceptable adverse events. Technical success was especially high for anastomotic strictures.
C1 [Chandan, Saurabh; Bhat, Ishfaq; Singh, Shailender] CHI Creighton Univ, Div Gastroenterol & Hepatol, Med Ctr, Omaha, NE USA.

[Mohan, Babu P.; Adler, Douglas G.] Univ Utah, Div Gastroenterol & Hepatol, Sch Med, Salt Lake City, UT 84132 USA.

[Khan, Shahab R.] Rush Univ, Med Ctr, Sect Gastroenterol, Chicago, IL 60612 USA.

[Kassab, Lena L.] Mayo Clin, Internal Med, Rochester, MN USA.

[Ponnada, Suresh] Carilion Roanoke Mem Hosp, Internal Med, Roanoke, VA USA.

[Ofosu, Andrew] Brooklyn Hosp, Gastroenterol, New York, NY USA.

C3 Creighton University; Utah System of Higher Education; University of Utah; Rush University; Mayo Clinic; Brooklyn Hospital Center

RP Adler, DG (corresponding author), Univ Utah, Sch Med, Huntsman Canc Ctr, Med, 30N 1900E 4R1 18, Salt Lake City, UT 84132 USA.; Adler, DG (corresponding author), Univ Utah, Sch Med, Huntsman Canc Ctr, Therapeut Endoscopy, 30N 1900E 4R1 18, Salt Lake City, UT 84132 USA.; Adler, DG (corresponding author), Univ Utah, Sch Med, Huntsman Canc Ctr, GI Fellowship Program,Gastroenterol & Hepatol, 30N 1900E 4R1 18, Salt Lake City, UT 84132 USA.

EM Douglas.adler@hsc.utah.edu
RI Chandan, Saurabh/M-9244-2019; Khan, Shahab/GSO-1694-2022
OI Ofosu, Anew/0000-0001-9784-7264;
CR ADAMS DB, 1994, ANN SURG, V219, P481, DOI 10.1097/00000658-199405000-00006
[Anonymous], 2019, PANCREAS
Baars JE, 2018, ENDOSC ULTRASOUND, V7, P284, DOI 10.4103/eus.eus_104_17
Barkay O, 2010, GASTROINTEST ENDOSC, V71, P1166, DOI 10.1016/j.gie.2009.10.048
Chen YI, 2017, GASTROINTEST ENDOSC, V85, P170, DOI 10.1016/j.gie.2016.07.031
Cotton PB, 2010, GASTROINTEST ENDOSC, V71, P446, DOI 10.1016/j.gie.2009.10.027
Dalal A, 2020, DIGEST ENDOSC, V32, P409, DOI 10.1111/den.13504
DERSIMONIAN R, 1986, CONTROL CLIN TRIALS, V7, P177, DOI 10.1016/0197-2456(86)90046-2
Dumonceau JM, 1996, GASTROINTEST ENDOSC, V43, P547, DOI 10.1016/S0016-5107(96)70189-X
Duval S, 2000, BIOMETRICS, V56, P455, DOI 10.1111/j.0006-341X.2000.00455.x
Ergun M, 2011, ENDOSCOPY, V43, P518, DOI 10.1055/s-0030-1256333
François E, 2002, GASTROINTEST ENDOSC, V56, P128, DOI 10.1067/mge.2002.125547
Fujii LL, 2013, GASTROINTEST ENDOSC, V78, P854, DOI 10.1016/j.gie.2013.05.016
Giovannini M, 2013, GASTROINTEST ENDOSC, V78, P865, DOI 10.1016/j.gie.2013.10.019
Godat S, 2019, SWISS MED WKLY, p11S
Hasegawa S, 2019, PANCREAS, V48, P1439
Higgins JPT, 2003, BMJ-BRIT MED J, V327, P557, DOI 10.1136/bmj.327.7414.557
Higgins JPT, 2009, J R STAT SOC A STAT, V172, P137, DOI 10.1111/j.1467-985X.2008.00552.x
Honjo M, 2018, ENDOSC ULTRASOUND, V7, P376, DOI 10.4103/eus.eus_2_18
Itoi T, 2013, DIGEST ENDOSC, V25, P241, DOI 10.1111/den.12048
Kahaleh M, 2007, GASTROINTEST ENDOSC, V65, P224, DOI 10.1016/j.gie.2006.05.008
Kurihara T, 2013, DIGEST ENDOSC, V25, P109, DOI 10.1111/den.12100
Lee SS., 2012, GASTROINTEST ENDOSC, V1, P845
Matsunami Y, 2018, ENDOSC INT OPEN, V6, pE505, DOI 10.1055/s-0044-101753
Mohan BP, 2019, GASTROINTEST ENDOSC, V89, P238, DOI [10.1016/j.gie.2018.10.018, 10.1016/j.gie.2018.10.036]
Moher D, 2009, BMJ-BRIT MED J, V339, DOI 10.1136/bmj.b2535
Oh D, 2020, J GASTROEN HEPATOL, V35, P994, DOI 10.1111/jgh.14897
Oh D, 2016, GASTROINTEST ENDOSC, V83, P366, DOI 10.1016/j.gie.2015.07.015
Rothstein HR, 2005, PUBLICATION BIAS IN META-ANALYSIS: PREVENTION, ASSESSMENT AND ADJUSTMENTS, P1, DOI 10.1002/0470870168
Shah JN, 2012, GASTROINTEST ENDOSC, V75, P56, DOI 10.1016/j.gie.2011.08.032
Stang A, 2010, EUR J EPIDEMIOL, V25, P603, DOI 10.1007/s10654-010-9491-z
Stroup DF, 2000, JAMA-J AM MED ASSOC, V283, P2008, DOI 10.1001/jama.283.15.2008
Sutton AJ., 2000, METHODS METAANALYSIS
Tessier G, 2007, GASTROINTEST ENDOSC, V65, P233, DOI 10.1016/j.gie.2006.06.029
Tomoda T, 2016, PANCREATOLOGY, V16, P44, DOI 10.1016/j.pan.2015.11.002
Tyberg A, 2017, GASTROINTEST ENDOSC, V85, P164, DOI 10.1016/j.gie.2016.07.030
Uchida D, 2018, CAN J GASTROENTEROL, V2018, DOI 10.1155/2018/8216109

Vila JJ, 2012, GASTROINTEST ENDOSC, V76, P1133, DOI 10.1016/j.gie.2012.08.001
Will U, 2015, WORLD J GASTROENTERO, V21, P13140, DOI 10.3748/wjg.v21.i46.13140
NR 39
TC 27
Z9 33
U1 0
U2 4
PU GEORG THIEME VERLAG KG
PI STUTTGART
PA Oswald-Hesse-Strasse 50, D-70469 STUTTGART, GERMANY
SN 2364-3722
EI 2196-9736
J9 ENDOSC INT OPEN
JI Endosc. Int. Open
PD NOV
PY 2020
VL 08
IS 11
BP E1664
EP E1672
DI 10.1055/a-1236-3350
PG 9
WC Gastroenterology & Hepatology; Surgery
WE Emerging Sources Citation Index (ESCI)
SC Gastroenterology & Hepatology; Surgery
GA OF2FV
UT WOS:000581031400012
PM 33140022
OA Green Submitted, gold
DA 2026-05-09
ER

PT J
AU Ogura, T
Okuda, A
Higuchi, K
AF Ogura, Takeshi
Okuda, Atsushi
Higuchi, Kazuhide
TI Endoscopic ultrasound-guided hepaticogastrostomy for hepaticojejunostomy
stricture using a one-step stent deployment technique (with video)
SO JOURNAL OF HEPATO-BILIARY-PANCREATIC SCIENCES

LA English
DT Article
DE EUS; EUS‐ guided biliary drainage; EUS‐ guided
hepaticogastrostomy; ERCP; hepaticojejunostomy stricture
C1 [Ogura, Takeshi; Okuda, Atsushi; Higuchi, Kazuhide] Osaka Med Coll, Dept Internal Med 2, 2-7 Daigakuchou, Takatsuki, Osaka 5698686, Japan.
C3 Osaka Medical & Pharmaceutical University
RP Ogura, T (corresponding author), Osaka Med Coll, Dept Internal Med 2, 2-7 Daigakuchou, Takatsuki, Osaka 5698686, Japan.
EM oguratakeshi0411@yahoo.co.jp
RI Ogura, Takeshi/AAU-5643-2021
CR Isayama H, 2019, J HEPATO-BIL-PAN SCI, V26, P249, DOI 10.1002/jhbp.631
Mukai S, 2019, GASTROINTEST ENDOSC, V89, P399, DOI 10.1016/j.gie.2018.07.030
Ogura T, 2021, SURG ENDOSC, V35, P209, DOI 10.1007/s00464-020-07381-2
Yamada A, 2019, DIGEST ENDOSC, V31, P86, DOI 10.1111/den.13261
Yamamoto Y, 2020, ENDOSC ULTRASOUND, V9, P110, DOI 10.4103/eus.eus_68_19
NR 5
TC 5
Z9 6
U1 0
U2 2
PU WILEY
PI HOBOKEN
PA 111 RIVER ST, HOBOKEN 07030-5774, NJ USA
SN 1868-6974
EI 1868-6982
J9 J HEPATO-BIL-PAN SCI
JI J. Hepato-Biliary-Pancreat. Sci.
PD AUG
PY 2021
VL 28
IS 8
BP E34
EP E35
DI 10.1002/jhbp.842
EA NOV 2020
PG 2
WC Gastroenterology & Hepatology; Surgery
WE Science Citation Index Expanded (SCI-EXPANDED)
SC Gastroenterology & Hepatology; Surgery
GA UE9GF
UT WOS:000586213700001

PM 33064931
DA 2026-05-09
ER

PT J
AU Han, D
Inamdar, S
Lee, CW
Miller, LS
Trindade, AJ
Sejpal, DV
AF Han, Dennis
Inamdar, Sumant
Lee, Calvin W.
Miller, Larry S.
Trindade, Arvind J.
Sejpal, Divyesh V.
TI Lumen Apposing Metal Stents (LAMSs) for Drainage of Pancreatic and
Gallbladder Collections: A Meta-analysis
SO JOURNAL OF CLINICAL GASTROENTEROLOGY
LA English
DT Article
DE lumen apposing metal stents; pancreatic fluid collections; pancreatic
pseudocyst; walled-off pancreatic necrosis; gallbladder drainage; acute
cholecystitis
ID ULTRASOUND-GUIDED PLACEMENT; WALLED-OFF NECROSIS; FLUID COLLECTIONS;
PERCUTANEOUS CHOLECYSTOSTOMY; ACUTE CHOLECYSTITIS; ENDOSCOPIC
NECROSECTOMY; PSEUDOCYST; MANAGEMENT; EFFICACY; THERAPY
AB Background and Aims:Endoscopic ultrasound-guided transmural drainage using lumen apposing metal stents (LAMSs) is becoming a popular and promising therapeutic approach for drainage of intra-abdominal fluid collections. There has been an increasing number of studies evaluating LAMS for drainage of pancreatic pseudocysts (PP), walled-off pancreatic necrosis (WOPN), and gallbladder (GB) drainage. The aim of this meta-analysis is to analyze the literature to date regarding the clinical success, technical success, and adverse events of LAMS in treatment of pancreatic fluid collections and GB drainage.Methods:A comprehensive search of multiple literature databases through November 2016 was performed. Human studies with at least 10 subjects that examined the clinical success, technical success, and adverse events of LAMS in treating PP, WOPN, and GB drainage were included.Results:A total of 993 patients (608WOPN; 204PP; 181GB drainage) underwent drainage from 20 trials. For drainage of WOPN, the pooled technical success was 98.9% [95% confidence interval (CI): 98.2% to 99.7%] and clinical success was 90% (95% CI: 87% to 93%) ((2)=0.001). For drainage of PP, the pooled technical success was 97% (95% CI: 95% to 99%) and clinical success was 98% (95% CI: 96% to 100%), ((2)=0.001). For GB drainage, the pooled technical success was 95% (95% CI: 91% to 99%) and clinical success was 93% (95% CI: 90% to 97%), ((2)=0.001). Total adverse events occurred in 11% of patients with higher complication rates observed in GB drainage. There was no evidence of publication bias in this meta-analysis.Conclusions:Endoscopic

ultrasound-guided transmural drainage using LAMS is becoming a widely accepted therapeutic approach for the treatment of PP, WOPN, and GB drainage with high clinical and technical success rates and acceptable adverse events. Further prospective randomized trials reporting long-term clinical efficacy and cost-effectiveness are needed to validate LAMS as a therapeutic modality for pancreatic and GB collections.

C1 [Han, Dennis; Inamdar, Sumant; Lee, Calvin W.; Miller, Larry S.; Trindade, Arvind J.; Sejpal, Divyesh V.] North Shore Univ Hosp, Hofstra Northwell Sch Med, Northwell Hlth Syst, Dept Med,Div Gastroenterol, Manhasset, NY USA.
C3 Northwell Health; North Shore University Hospital
RP Sejpal, DV (corresponding author), North Shore Univ Hosp, Hofstra Northwell Sch Med, Northwell Hlth Syst, Div Gastroenterol, 300 Community Dr,4 Levitt, Manhasset, NY 11030 USA.
EM divsejpal@hotmail.com
OI Inaar, Sumant/0000-0002-1002-2823

CR Akhan O, 2002, EUR J RADIOL, V43, P229, DOI 10.1016/S0720-048X(02)00158-4
[Anonymous], GASTROINTEST ENDOSC
Bang JY, 2017, DIGEST ENDOSC, V29, P83, DOI 10.1111/den.12681
Chang L, 2000, AM J SURG, V180, P198, DOI 10.1016/S0002-9610(00)00476-1
De La Serna C, 2015, GASTROINTEST ENDOSC, V81, pAB130, DOI 10.1016/j.gie.2015.03.042
de la Serna-Higuera C, 2013, GASTROINTEST ENDOSC, V77, P303, DOI 10.1016/j.gie.2012.09.021
Gornals JB, 2016, SURG ENDOSC, V30, P2592, DOI 10.1007/s00464-015-4505-2
Irani S, 2015, GASTROINTEST ENDOSC, V82, P1110, DOI 10.1016/j.gie.2015.05.045
Ito K, 2004, AM J ROENTGENOL, V183, P193, DOI 10.2214/ajr.183.1.1830193
Itoi T, 2012, GASTROINTEST ENDOSC, V75, P870, DOI 10.1016/j.gie.2011.10.020
Itoi T, 2010, GASTROINTEST ENDOSC, V71, P1038, DOI 10.1016/j.gie.2010.01.026
Jacobson BC, 2005, GASTROINTEST ENDOSC, V61, P363, DOI 10.1016/S0016-5107(04)02779-8
Kahaleh M, 2006, ENDOSCOPY, V38, P355, DOI 10.1055/s-2006-925249
Kahaleh M, 2016, DIGEST LIVER DIS, V48, P1054, DOI 10.1016/j.dld.2016.05.021
Maple JT, 2017, GASTROINTEST ENDOSC, V85, P465, DOI 10.1016/j.gie.2016.11.021
Moher D, 2009, PLOS MED, V6, DOI 10.1371/journal.pmed.1000097
Mukai S, 2015, ENDOSCOPY, V47, P47, DOI 10.1055/s-0034-1377966
Mukewar SS, 2015, GASTROINTEST ENDOSC, V81, pAB161, DOI 10.1016/j.gie.2015.03.108
Patil R, 2016, ANN GASTROENTEROL, V29, P168, DOI 10.20524/aog.2016.0008
Patil R, 2016, ANN GASTROENTEROL, V29, P162, DOI 10.20524/aog.2016.0001
Peñas I, 2015, GASTROINTEST ENDOSC, V81, pAB426, DOI 10.1016/j.gie.2015.03.1620
Raijman Isaac, 2003, Gastrointest Endosc Clin N Am, V13, P561
Rinninella E, 2015, GASTROINTEST ENDOSC, V82, P1039, DOI 10.1016/j.gie.2015.04.006
Shah RJ, 2015, CLIN GASTROENTEROL H, V13, P747, DOI 10.1016/j.cgh.2014.09.047
Sharaiha RZ, 2017, GASTROINTEST ENDOSC, V85, P904, DOI 10.1016/j.gie.2016.12.023
Sharaiha RZ, 2015, GASTROINTEST ENDOSC, V81, pAB425, DOI 10.1016/j.gie.2015.03.1618
Sharaiha RZ, 2016, CLIN GASTROENTEROL H, V14, P1797, DOI 10.1016/j.cgh.2016.05.011
Siddiqui AA, 2017, GASTROINTEST ENDOSC, V85, P758, DOI 10.1016/j.gie.2016.08.014
Siddiqui AA, 2016, GASTROINTEST ENDOSC, V83, P699, DOI 10.1016/j.gie.2015.10.020
Stang A, 2010, EUR J EPIDEMIOL, V25, P603, DOI 10.1007/s10654-010-9491-z
Teoh AY, 2015, GASTROINTEST ENDOSC, V81, pAB130, DOI 10.1016/j.gie.2015.03.043

Teoh AYB, 2016, WORLD J GASTRO ENDOS, V8, P310, DOI 10.4253/wjge.v8.i6.310
Varadarajulu S, 2013, GASTROENTEROLOGY, V145, P583, DOI 10.1053/j.gastro.2013.05.046
Varadarajulu S, 2011, J GASTROINTEST SURG, V15, P2080, DOI 10.1007/s11605-011-1621-8
Varadarajulu S, 2011, J GASTROEN HEPATOL, V26, P1504, DOI 10.1111/j.1440-1746.2011.06771.x
Walter D, 2016, GUT, V65, P6, DOI 10.1136/gutjnl-2015-309925
Walter D, 2015, ENDOSCOPY, V47, P63, DOI 10.1055/s-0034-1378113
Wells GA., 2020, NEWCASTLE OTTAWA SCA
NR 38
TC 27
Z9 32
U1 0
U2 5
PU LIPPINCOTT WILLIAMS & WILKINS
PI PHILADELPHIA
PA TWO COMMERCE SQ, 2001 MARKET ST, PHILADELPHIA, PA 19103 USA
SN 0192-0790
EI 1539-2031
J9 J CLIN GASTROENTEROL
JI J. Clin. Gastroenterol.
PD OCT
PY 2018
VL 52
IS 9
BP 835
EP 844
DI 10.1097/MCG.0000000000000934
PG 10
WC Gastroenterology & Hepatology
WE Science Citation Index Expanded (SCI-EXPANDED)
SC Gastroenterology & Hepatology
GA GV0LW
UT WOS:000445753800014
PM 29016384
DA 2026-05-09
ER

PT J
AU Yoo, J
Yan, LD
Hasan, R
Somalya, S
Nieto, J

Siddiqui, AA
AF Yoo, Joseph
Yan, Linda
Hasan, Raza
Somalya, Saana
Nieto, Jose
Siddiqui, Ali A.
TI Feasibility, safety, and outcomes of a single-step endoscopic ultrasonography-guided drainage of pancreatic fluid collections without fluoroscopy using a novel electrocautery-enhanced lumen-apposing, self-expanding metal stent
SO ENDOSCOPIC ULTRASOUND
LA English
DT Article
DE Endosonography; pancreatic fluid collections; stents
ID PERCUTANEOUS DRAINAGE; PSEUDOCYSTS; NECROSIS; EXPERIENCE; MANAGEMENT; DIAGNOSIS; EFFICACY
AB Background and Objectives: There are currently limited data available regarding the safety of endoscopic ultrasound (EUS)-guided drainage of pancreatic fluid collections (PFCs) using the lumen-apposing metal stent without fluoroscopic guidance. This study aims to evaluate clinical outcomes and safety of EUS-guided drainage of PFC using the electrocautery-enhanced lumen-apposing metal stents (EC-LAMSs) without fluoroscopic guidance. Methods: We conducted a retrospective study on patients with symptomatic PFC who underwent EUS-guided drainage using EC-LAMS without fluoroscopy. All patients were followed clinically until resolution of their PFC. Technical success (successful placement of EC-LAMS), number of patients who achieved complete resolution of PFC without additional intervention and adverse events were noted. Results: We evaluated 25 patients, including three with pancreatic pseudocysts and 22 with walled-off necrosis (WON). The etiology of the patient's pancreatitis was gallstones (42%), alcohol (27%), and other causes (31%). The mean cyst size was 82 mm (range, 60-170 mm). The indications for endoscopic drainage were abdominal pain, infected WON, or gastric outlet obstruction. Technical success with placement of the EC-LAMS was achieved in all 25 patients. There were no procedure-related complications. The mean patient follow-up was 7.8 months. PFCs resolved in 24 (96%) patients; the one failure was in a patient with WON. Stent occlusion was seen in one patient. There was a spontaneous migration of one stent into the enteral lumen after resolution of WONs. The EC-LAMS were successfully removed using a snare in all the remaining patients. The median number of endoscopy sessions to achieve PFCs resolution was 2 (range, 2-6). Conclusions: Single-step EUS-guided drainage of PFCs without fluoroscopic guidance using the novel EC-LAMS is a safe and effective endoscopic technique for drainage of PFCs with excellent technical and clinical success rates and no complications. Due to its ease of use, EC-LAMS may simplify and streamline EUS-guided management of PFC and help in its widespread adoption as an alternative to surgery.
C1 [Yoo, Joseph; Yan, Linda; Hasan, Raza; Somalya, Saana; Siddiqui, Ali A.] Thomas Jefferson Univ, Div Gastroenterol & Hepatol, Philadelphia, PA 19107 USA.
[Nieto, Jose] Borland Groover Clin, Div Gastroenterol, Jacksonville, FL USA.
C3 Thomas Jefferson University
RP Siddiqui, AA (corresponding author), Thomas Jefferson Univ, Div Gastroenterol, Philadelphia, PA 19107 USA.
EM ali.siddiqui@jefferson.edu

CR Aghdassi A, 2008, PANCREAS, V36, P105, DOI 10.1097/MPA.0b013e31815a8887
Akshintala VS, 2014, GASTROINTEST ENDOSC, V79, P921, DOI 10.1016/j.gie.2013.10.032
Baillie J, 2004, GASTROINTEST ENDOSC, V59, P873, DOI 10.1016/S0016-5107(04)00354-2
Banks PA, 2013, GUT, V62, P102, DOI 10.1136/gutjnl-2012-302779
Baron TH, 2002, GASTROINTEST ENDOSC, V56, P7, DOI 10.1067/mge.2002.125106
BINMOELLER KF, 1995, GASTROINTEST ENDOSC, V42, P219, DOI 10.1016/S0016-5107(95)70095-1
Brun A, 2011, J CLIN GASTROENTEROL, V45, P614, DOI 10.1097/MCG.0b013e318213ef3e
Cahen D, 2005, ENDOSCOPY, V37, P977, DOI 10.1055/s-2005-870336
Itoi T, 2012, GASTROINTEST ENDOSC, V75, P870, DOI 10.1016/j.gie.2011.10.020
Jacobson BC, 2005, GASTROINTEST ENDOSC, V61, P363, DOI 10.1016/S0016-5107(04)02779-8
Law R, 2015, EXPERT REV GASTROENT, V9, P167, DOI 10.1586/17474124.2014.943186
Nealon WH, 2002, ANN SURG, V235, P751, DOI 10.1097/00000658-200206000-00001
Rinninella E, 2015, GASTROINTEST ENDOSC, V82, P1039, DOI 10.1016/j.gie.2015.04.006
Seicean A, 2011, J GASTROINTEST LIVER, V20, P39
Shah RJ, 2015, CLIN GASTROENTEROL H, V13, P747, DOI 10.1016/j.cgh.2014.09.047
Siddiqui AA, 2016, GASTROINTEST ENDOSC, V83, P699, DOI 10.1016/j.gie.2015.10.020
Singhal S, 2013, CLIN ENDOSC, V46, P506, DOI 10.5946/ce.2013.46.5.506
Varadarajulu S, 2013, GASTROENTEROLOGY, V145, P583, DOI 10.1053/j.gastro.2013.05.046
NR 18
TC 30
Z9 41
U1 0
U2 0
PU MEDKNOW PUBLICATIONS & MEDIA PVT LTD
PI MUMBAI
PA B-9, KANARA BUSINESS CENTRE, OFF LINK RD, GHAKTOPAR-E, MUMBAI, 400075,
INDIA
SN 2303-9027
EI 2226-7190
J9 ENDOSC ULTRASOUND
JI Endosc. Ultrasound
PD MAR-APR
PY 2017
VL 6
IS 2
BP 131
EP 135
DI 10.4103/2303-9027.204814
PG 5
WC Gastroenterology & Hepatology
WE Science Citation Index Expanded (SCI-EXPANDED)
SC Gastroenterology & Hepatology

GA ET8DK
UT WOS:000400528400005
PM 28440239
OA Green Submitted
DA 2026-05-09
ER

PT J
AU Puri, R
Thandassery, RB
Alfadda, AA
Al Kaabi, S
AF Puri, Rajesh
Thandassery, Ragesh Babu
Alfadda, Abdulrahman A.
Al Kaabi, Saad
TI Endoscopic ultrasound guided drainage of pancreatic fluid collections:
Assessment of the procedure, technical details and review of the
literature
SO WORLD JOURNAL OF GASTROINTESTINAL ENDOSCOPY
LA English
DT Review
DE Acute pancreatitis; Pancreatic fluid collections; Endoscopic
ultrasound-guided drainage
ID PROSPECTIVE RANDOMIZED-TRIAL; EXPANDING METAL STENTS; TRANSMURAL
DRAINAGE; PSEUDOCYST DRAINAGE; TREATMENT OUTCOMES; SINGLE-STEP; COMBINED
ENDOPROSTHESIS; PERCUTANEOUS DRAINAGE; ECHO ENDOSCOPE; EUS
AB Endoscopic ultrasound (EUS) guided drainage of pancreatic fluid collections (PFC) has become increasingly popular and become first line management option in many centers. Use of therapeutic echoendoscopes has greatly increased the applicability of EUS guided transmural drainage. Drainage is indicated in symptomatic PFCs, PFC related infection, bleed, luminal obstruction, fistulization and biliary obstruction. EUS guided transmural drainage of PFCs is preferred in patients with non bulging lesions, portal hypertension, bleeding tendency and in those whom conventional drainage has failed. In the present decade significant progress has been made in minimally invasive endoscopic techniques. There are newer stent designs, access devices and techniques for more efficient drainage of PFCs. In this review, we discuss the EUS guided drainage of PFCs in acute pancreatitis.
C1 [Puri, Rajesh] Medanta, Inst Digest Hepatobiliary Sci, Gurgaon 110092, Ncr Delhi, India.
[Thandassery, Ragesh Babu; Al Kaabi, Saad] Hamad Med Corp, Dept Med, Div Gastroenterol & Hepatol, Doha, Qatar.
[Alfadda, Abdulrahman A.] King Faisal Specialist Hosp & Res Ctr, Dept Med, Riyadh 12713, Saudi Arabia.
C3 Hamad Medical Corporation; King Faisal Specialist Hospital & Research
Center
RP Thandassery, RB (corresponding author), Hamad Med Corp, Dept Med, Div Gastroenterol & Hepatol, Doha PO 3050, Doha, Qatar.

EM doc.ragesh@gmail.com
RI Thandassery, Ragesh/GPX-8591-2022
CR Akshintala VS, 2014, GASTROINTEST ENDOSC, V79, P921, DOI 10.1016/j.gie.2013.10.032
Antillon MR, 2006, GASTROINTEST ENDOSC, V63, P797, DOI 10.1016/j.gie.2005.10.025
Arvanitakis M, 2005, GASTROINTEST ENDOSC, V62, P143, DOI 10.1016/S0016-5107(05)01627-5
Arvanitakis M, 2007, GASTROINTEST ENDOSC, V65, P609, DOI 10.1016/j.gie.2006.06.083
Azar RR, 2006, GASTROINTEST ENDOSC, V63, P688, DOI 10.1016/j.gie.2005.10.032
Baillie J, 2004, GASTROINTEST ENDOSC, V59, P873, DOI 10.1016/S0016-5107(04)00354-2
Bakker OJ, 2012, JAMA-J AM MED ASSOC, V307, P1053, DOI 10.1001/jama.2012.276
Bang JY, 2014, SURG ENDOSC, V28, P2877, DOI 10.1007/s00464-014-3541-7
Bang JY, 2013, J GASTROEN HEPATOL, V28, P1725, DOI 10.1111/jgh.12328
Baron TH, 2002, GASTROINTEST ENDOSC, V56, P7, DOI 10.1067/mge.2002.125106
BARON TH, 2008, ERCP, P475
Baron TH, 2008, J GASTROINTEST SURG, V12, P369, DOI 10.1007/s11605-007-0334-5
Barthet M, 2008, GASTROINTEST ENDOSC, V67, P245, DOI 10.1016/j.gie.2007.06.014
Berzosa M, 2012, ENDOSCOPY, V44, P543, DOI 10.1055/s-0031-1291710
BINMOELLER KF, 1995, GASTROINTEST ENDOSC, V42, P219, DOI 10.1016/S0016-5107(95)70095-1
Boerma D, 1999, DIGEST SURG, V16, P501, DOI 10.1159/000018776
Cahen D, 2005, ENDOSCOPY, V37, P977, DOI 10.1055/s-2005-870336
Cortes ES, 2002, GASTROINTEST ENDOSC, V56, P380, DOI 10.1067/mge.2002.126883
Dhingra R, 2015, GASTROINTEST ENDOSC, V81, P351, DOI 10.1016/j.gie.2014.07.060
Fabbri C, 2012, ENDOSCOPY, V44, P429, DOI 10.1055/s-0031-1291624
Fabbri C, 2012, WORLD J GASTRO ENDOS, V4, P479, DOI 10.4253/wjge.v4.i11.479
Giovannini M, 1998, GASTROINTEST ENDOSC, V48, P200, DOI 10.1016/S0016-5107(98)70165-8
Giovannini M, 2001, ENDOSCOPY, V33, P473, DOI 10.1055/s-2001-14967
Giovannini M, 2007, TECH GASTROINTEST EN, V9, P32, DOI 10.1016/j.tgie.2006.11.013
Gornals JB, 2013, SURG ENDOSC, V27, P1428, DOI 10.1007/s00464-012-2591-y
GRIMM H, 1992, GASTROINTEST ENDOSC, V38, P170, DOI 10.1016/S0016-5107(92)70384-8
Hirota WK, 2003, GASTROINTEST ENDOSC, V58, P475, DOI 10.1067/S0016-5107(03)01883-2
Hookey LC, 2006, GASTROINTEST ENDOSC, V63, P635, DOI 10.1016/j.gie.2005.06.028
HOWELL DA, 1993, GASTROINTEST ENDOSC, V39, P693, DOI 10.1016/S0016-5107(93)70225-4
Itoi T, 2012, GASTROINTEST ENDOSC, V75, P870, DOI 10.1016/j.gie.2011.10.020
Jacobson BC, 2005, GASTROINTEST ENDOSC, V61, P363, DOI 10.1016/S0016-5107(04)02779-8
Kahaleh M, 2006, ENDOSCOPY, V38, P355, DOI 10.1055/s-2006-925249
Kawakami H, 2014, GUT LIVER, V8, P341, DOI 10.5009/gnl.2014.8.4.341
Klöppel G, 2000, SEMIN DIAGN PATHOL, V17, P7
Krüger M, 2006, GASTROINTEST ENDOSC, V63, P409, DOI 10.1016/j.gie.2005.11.047
Lin H, 2014, GASTROENT RES PRACT, V2014, DOI 10.1155/2014/193562
Lopes CV, 2007, SCAND J GASTROENTERO, V42, P524, DOI 10.1080/00365520601065093
Mangiavillano B, 2012, J DIGEST DIS, V13, P47, DOI 10.1111/j.1751-2980.2011.00547.x
Mukai S, 2014, GASTROINTEST ENDOSC, V79, P531, DOI 10.1016/j.gie.2013.10.004
Panamonta N, 2012, EUR J GASTROEN HEPAT, V24, P1355, DOI 10.1097/MEG.0b013e32835871eb

Park DH, 2009, ENDOSCOPY, V41, P842, DOI 10.1055/s-0029-1215133
Penn DE, 2012, GASTROINTEST ENDOSC, V76, P679, DOI 10.1016/j.gie.2012.04.457
Puri R, 2012, J GASTROEN HEPATOL, V27, P722, DOI 10.1111/j.1440-1746.2012.07089.x
Sarr MG, 2013, POL ARCH MED WEWN, V123, P118, DOI 10.20452/pamw.1627
Schneider CP, 2014, GASTROINTEST ENDOS S, V79, pAB414, DOI DOI 10.1016/J.GIE.2014.02.553]
Seewald S, 2012, DIGEST ENDOSC, V24, P36, DOI 10.1111/j.1443-1661.2011.01162.x
Seifert H, 2001, ENDOSCOPY, V33, P1022, DOI 10.1055/s-2001-18927
Siddiqui AA, 2013, GASTROINTEST ENDOSC, V78, P589, DOI 10.1016/j.gie.2013.03.1337
Singhal S, 2013, CLIN ENDOSC, V46, P506, DOI 10.5946/ce.2013.46.5.506
Talreja JP, 2008, GASTROINTEST ENDOSC, V68, P1199, DOI 10.1016/j.gie.2008.06.015
Téllez-Avila FI, 2013, WORLD J GASTRO ENDOS, V5, P297, DOI 10.4253/wjge.v5.i6.297
Tenner S, 2013, AM J GASTROENTEROL, V108, P1400, DOI 10.1038/ajg.2013.218
VANSONNENBERG E, 1989, RADIOLOGY, V170, P757, DOI 10.1148/radiology.170.3.2644662
Varadarajulu S, 2008, GASTROINTEST ENDOSC, V68, P1102, DOI 10.1016/j.gie.2008.04.028
Varadarajulu S, 2007, GASTROINTEST ENDOSC, V66, P1107, DOI 10.1016/j.gie.2007.03.1027
Varadarajulu S, 2013, GASTROENTEROLOGY, V145, P583, DOI 10.1053/j.gastro.2013.05.046
Varadarajulu S, 2011, J GASTROINTEST SURG, V15, P2080, DOI 10.1007/s11605-011-1621-8
Varadarajulu S, 2011, J GASTROEN HEPATOL, V26, P1504, DOI 10.1111/j.1440-1746.2011.06771.x
Varadarajulu S, 2011, GASTROINTEST ENDOSC, V74, P74, DOI 10.1016/j.gie.2011.03.1122
Weilert F, 2012, ENDOSCOPY, V44, P780, DOI 10.1055/s-0032-1309839
Yeo CJ, 1997, ANN SURG, V226, P248, DOI 10.1097/00000658-199709000-00004
Yusuf TE, 2006, GASTROINTEST ENDOSC, V63, P223, DOI 10.1016/j.gie.2005.09.034
Zheng MW, 2011, HEPATO-GASTROENTEROL, V58, P1106, DOI 10.5754/hge11059
NR 63
TC 8
Z9 14
U1 0
U2 1
PU BAISHIDENG PUBLISHING GROUP INC
PI PLEASANTON
PA 8226 REGENCY DR, PLEASANTON, CA 94588 USA
SN 1948-5190
J9 WORLD J GASTROINTEST
JI World J. Gastrointest. Endosc.
PD APR 16
PY 2015
VL 7
IS 4
BP 354
EP 363
DI 10.4253/wjge.v7.i4.354
PG 10

WC Gastroenterology & Hepatology
WE Emerging Sources Citation Index (ESCI)
SC Gastroenterology & Hepatology
GA CR0GS
UT WOS:000360997100007
PM 25901214
OA Green Submitted, hybrid
DA 2026-05-09
ER

PT J
AU Hara, K
Yamao, K
Mizuno, N
Sawaki, A
Takagi, T
Bhatia, V
AF Hara, Kazuo
Yamao, Kenji
Mizuno, Nobumasa
Sawaki, Akira
Takagi, Tadayuki
Bhatia, Vikram
TI ENDOSCOPIC ULTRASOUND-GUIDED CHOLEDOCHODUODENOSTOMY
SO DIGESTIVE ENDOSCOPY
LA English
DT Article
DE endoscopic biliary drainage; endoscopic ultrasonography (EUS)-guided biliary drainage; EUS-guided choledochoduodenostomy (EUS-CDS); EUS-guided fine-needle aspiration (EUS-FNA); interventional EUS
ID MALIGNANT BILIARY OBSTRUCTION; BILE-DUCT; DRAINAGE; CHOLANGIOGRAPHY
AB Endoscopic biliary drainage (EBD) may be unsuccessful in some patients, because of failed biliary cannulation or tumor infiltration, limiting endoscopic access to major papilla. The alternative method of percutaneous transhepatic biliary drainage carries a risk of complications, such as bleeding, portal vein thrombus, portal vein occlusion and intra- or extra-abdominal bile leakage. Recently, endoscopic ultrasonography (EUS)-guided biliary stent placement has been described in patients with malignant biliary obstruction. Technically, EUS-guided biliary drainage is possible via transgastric or transduodenal routes or through the small intestine using a direct access or rendezvous technique. We describe herein a technique for direct stent insertion from the duodenal bulb for the management of patients with jaundice caused by malignant obstruction of the lower extrahepatic bile duct. We think transduodenal direct access is the best treatment in patients with jaundice caused by inoperable malignant obstruction of the lower extrahepatic bile duct when EBD fails.
C1 [Hara, Kazuo] Aichi Canc Ctr Hosp, Dept Gastroenterol, Chikusa Ku, Nagoya, Aichi 4648681, Japan.
[Bhatia, Vikram] Inst Liver & Biliary Sci, Dept Med Hepatol, Delhi, India.

C3 Aichi Cancer Center; Institute of Liver & Biliary Sciences (ILBS)
RP Hara, K (corresponding author), Aichi Canc Ctr Hosp, Dept Gastroenterol, Chikusa Ku, 1-1 Kanokoden, Nagoya, Aichi 4648681, Japan.
EM khara@aichi-cc.jp
RI Bhatia, Vikram/JUU-4166-2023
CR ANG TL, 2007, JOP, V9, P438
Bories E, 2007, ENDOSCOPY, V39, P287, DOI 10.1055/s-2007-966212
Burmester E, 2003, GASTROINTEST ENDOSC, V57, P246, DOI 10.1067/mge.2003.85
Giovannini M, 2003, ENDOSCOPY, V35, P1076, DOI 10.1055/s-2003-44596
Giovannini M, 2001, ENDOSCOPY, V33, P898, DOI 10.1055/s-2001-17324
Itoi T, 2009, GASTROINTEST ENDOSC, V69, pS8, DOI 10.1016/j.gie.2008.11.003
Kahaleh M, 2006, GASTROINTEST ENDOSC, V64, P52, DOI 10.1016/j.gie.2006.01.063
Kahaleh M, 2004, GASTROINTEST ENDOSC, V60, P138, DOI 10.1016/S0016-5107(04)01528-7
Kahaleh M, 2005, GASTROINTEST ENDOSC, V61, P307, DOI 10.1016/S0016-5107(04)02585-4
Kitano M, 2009, DIGEST ENDOSC, V21, pS66, DOI 10.1111/j.1443-1661.2009.00868.x
Lai R, 2005, ENDOSCOPY, V37, P487, DOI 10.1055/s-2005-861250
Mallery S, 2004, GASTROINTEST ENDOSC, V59, P100, DOI 10.1016/S0016-5107(03)02300-9
Püspök A, 2005, AM J GASTROENTEROL, V100, P1743, DOI 10.1111/j.1572-0241.2005.41806.x
Tarantino I, 2008, ENDOSCOPY, V40, P336, DOI 10.1055/s-2007-995455
Will U, 2007, ENDOSCOPY, V39, P292, DOI 10.1055/s-2007-966215
Winick A B, 2001, Tech Vasc Interv Radiol, V4, P200, DOI 10.1016/S1089-2516(01)90026-5
Yamao K, 2008, ENDOSCOPY, V40, P340, DOI 10.1055/s-2007-995485
Yamao K, 2006, GASTROINTEST ENDOSC, V64, P663, DOI 10.1016/j.gie.2006.07.003
Yamao K, 2009, GASTROINTEST ENDOSC, V69, pS194, DOI 10.1016/j.gie.2008.12.028
NR 19
TC 18
Z9 18
U1 0
U2 0
PU WILEY
PI HOBOKEN
PA 111 RIVER ST, HOBOKEN 07030-5774, NJ USA
SN 0915-5635
EI 1443-1661
J9 DIGEST ENDOSC
JI Dig. Endosc.
PD APR
PY 2010
VL 22
IS 2
BP 147
EP 150

DI 10.1111/j.1443-1661.2010.00944.x
PG 4
WC Gastroenterology & Hepatology; Surgery
WE Science Citation Index Expanded (SCI-EXPANDED)
SC Gastroenterology & Hepatology; Surgery
GA 572YM
UT WOS:000275874200014
PM 20447212
DA 2026-05-09
ER

PT J
AU Kanno, Y
Kozakai, F
Koshita, S
Ogawa, T
Kusunose, H
Masu, K
Sakai, T
Murabayashi, T
Ito, K
AF Kanno, Yoshihide
Kozakai, Fumisato
Koshita, Shinsuke
Ogawa, Takahisa
Kusunose, Hiroaki
Masu, Kaori
Sakai, Toshitaka
Murabayashi, Toji
Ito, Kei
TI Technical issues stemming from endoscopic-ultrasound-guided gallbladder
drainage: A single center experience
SO TURKISH JOURNAL OF GASTROENTEROLOGY
LA English
DT Article
DE EUS-guided biliary drainage; endosonography; lumen-apposing stent;
cholecystoduodenostomy; endoscopic retrograde cholangiopancreatography
ID APPOSING METAL STENT; ACUTE CHOLECYSTITIS; PERCUTANEOUS CHOLECYSTOSTOMY;
ASPIRATION; CONVERSION; OUTCOMES
AB Background/Aims: This study was conducted to evaluate the outcomes of endoscopic-ultrasound-guided gallbladder drainage (EUSGBD) using traditional biliary stents without lumen-apposing stents and to determine technical issues.
Materials and Methods: All 18 patients who underwent EUS-GBD at our center between 2012 and 2018 were evaluated.

After the clinical outcomes (including technical success, clinical effectiveness, adverse events, and recurrence) were analyzed, technical countermeasures for problems extracted from the analysis were developed.

Results: The rates of technical success, clinical effectiveness, severe adverse event occurrence, and recurrence of acute cholecystitis were 94% (17/18), 88% (15/17), 6% (1/18, massive bile leakage), and 27% (4/15), respectively. Distal gastrectomy causing scope instability, the non-swollen gallbladder, and double pigtail stent use caused technical difficulties. A fully covered metal stent (fcMS) should be placed in a shallow position so that it does not wedge into the opposite site. When the puncture route involves the gastric wall, the proximal portion of an fcMS located in the stomach can migrate toward the abdominal cavity.

Conclusion: Although the clinical outcomes of EUS-GBD were relatively favorable, several technical issues related specifically to EUSGBD were observed. Technical countermeasures would improve the outcomes.
C1 [Kanno, Yoshihide; Kozakai, Fumisato; Koshita, Shinsuke; Ogawa, Takahisa; Kusunose, Hiroaki; Masu, Kaori; Sakai, Toshitaka; Murabayashi, Toji; Ito, Kei] Sendai City Med Ctr, Sendai, Miyagi, Japan.
RP Kanno, Y (corresponding author), Sendai City Med Ctr, Sendai, Miyagi, Japan.
EM yoshi-hk@openhp.or.jp
CR Baron TH, 2007, GASTROINTEST ENDOSC, V65, P735, DOI 10.1016/j.gie.2006.07.041
CHARLSON ME, 1987, J CHRON DIS, V40, P373, DOI 10.1016/0021-9681(87)90171-8
Chopra S, 2001, AM J ROENTGENOL, V176, P1025, DOI 10.2214/ajr.176.4.1761025
Dollhopf M, 2017, GASTROINTEST ENDOSC, V86, P636, DOI 10.1016/j.gie.2017.02.027
Hasan Muhammad K, 2013, Gastrointest Endosc Clin N Am, V23, P453, DOI 10.1016/j.giec.2012.12.010
Irani S, 2015, GASTROINTEST ENDOSC, V82, P1110, DOI 10.1016/j.gie.2015.05.045
Ito K, 2004, AM J ROENTGENOL, V183, P193, DOI 10.2214/ajr.183.1.1830193
Itoi T, 2017, J HEPATO-BIL-PAN SCI, V24, P362, DOI 10.1002/jhbp.454
Itoi T, 2012, GASTROINTEST ENDOSC, V75, P870, DOI 10.1016/j.gie.2011.10.020
Itoi T, 2011, J HEPATO-BIL-PAN SCI, V18, P282, DOI 10.1007/s00534-010-0310-4
Kahaleh M, 2016, DIGEST LIVER DIS, V48, P1054, DOI 10.1016/j.dld.2016.05.021
Kamata K, 2017, WORLD J GASTROENTERO, V23, P661, DOI 10.3748/wjg.v23.i4.661
Kozakai F, 2019, CLIN ENDOSC, V52, P262, DOI 10.5946/ce.2018.183
Law R, 2016, CLIN GASTROENTEROL H, V14, P476, DOI 10.1016/j.cgh.2015.10.026
Manta R, 2018, EUR J GASTROEN HEPAT, V30, P695, DOI 10.1097/MEG.0000000000001112
Minaga K, 2019, DIGEST ENDOSC, V31, P180, DOI 10.1111/den.13242
Mori Y, 2018, J HEPATO-BIL-PAN SCI, V25, P87, DOI 10.1002/jhbp.504
Murabayashi T, 2018, ENDOSCOPY, V50, pE642, DOI 10.1055/a-0591-2630
Nakai Y, 2017, ENDOSC ULTRASOUND, V6, P205, DOI 10.4103/2303-9027.208172
OKEN MM, 1982, AM J CLIN ONCOL-CANC, V5, P649, DOI 10.1097/00000421-198212000-00014
Takagi W, 2016, THER ADV GASTROENTER, V9, P19, DOI 10.1177/1756283X15609285
Walter D, 2016, GUT, V65, P6, DOI 10.1136/gutjnl-2015-309925
NR 22
TC 10
Z9 11
U1 0
U2 0
PU AVES

PI SISLI
PA BUYUKDERE CAD 105-9, MECIDIYEKOY, SISLI, ISTANBUL 34394, TURKEY
EI 2148-5607
J9 TURK J GASTROENTEROL
JI Turk. J. Gastroenterol.
PD DEC
PY 2019
VL 30
IS 12
BP 1055
EP 1061
DI 10.5152/tjg.2019.181032
PG 7
WC Gastroenterology & Hepatology
WE Science Citation Index Expanded (SCI-EXPANDED)
SC Gastroenterology & Hepatology
GA JX0QP
UT WOS:000503449100008
PM 31854311
OA Green Submitted
DA 2026-05-09
ER

PT J
AU Chandrasekhara, V
Elhanafi, S
Storm, AC
Takahashi, N
Lee, NJ
Levy, MJ
Kaura, K
Wang, LL
Majumder, S
Vege, SS
Law, RJ
Abu Dayyeh, BK
AF Chandrasekhara, Vinay
Elhanafi, Sherif
Storm, Andrew C.
Takahashi, Naoki
Lee, Nam Ju
Levy, Michael J.

Kaura, Karan
Wang, Lillian
Majumder, Shounak
Vege, Santhi S.
Law, Ryan J.
Abu Dayyeh, Barham K.
TI Predicting the Need for Step-Up Therapy After EUS-Guided Drainage of Pancreatic Fluid Collections With Lumen-Apposing Metal Stents
SO CLINICAL GASTROENTEROLOGY AND HEPATOLOGY
LA English
DT Article
DE Acute Pancreatitis; Pseudocyst; Walled-Off Necrosis; EUS; Lumen-Apposing Metal Stents
ID NECROTIZING PANCREATITIS; PLASTIC STENTS; NECROSIS; NECROSECTOMY; EFFICACY
AB BACKGROUND & AIMS: A significant proportion of individuals with pancreatic fluid collections (PFCs) require step-up therapy after endoscopic drainage with lumen-apposing metal stents. The aim of this study is to identify factors associated with PFCs that require step-up therapy.

METHODS: A retrospective cohort study of patients undergoing endoscopic ultrasound-guided drainage of PFCs with lumen-apposing metal stents from April 2014 to October 2019 at a single center was performed. Step-up therapy included direct endoscopic necrosectomy, additional drainage site (endoscopic or percutaneous), or surgical intervention after the initial drainage procedure. Multivariable logistic regression was performed using a backward stepwise approach with a p <= .2 threshold for variable retention to identify factors predictive for the need for step-up therapy.

RESULTS: One hundred thirty-six patients were included in the final study cohort, of whom 69 (50.7%) required step-up therapy. Independent predictors of step-up therapy included: collection size measuring >= 10 cm (odds ratio [OR], 8.91; 95% confidence interval [CI], 3.36-23.61), paracolic extension of the PFC (OR, 4.04; 95% CI, 1.60-10.23), and >= 30 % solid necrosis (OR, 4.24; 95% CI, 1.48-12.16). In a sensitivity analysis of 81 patients with walled-off necrosis, 51 (63.0%) required step-up therapy. Similarly, factors predictive of the need for step-up therapy for walled-off necrosis included: collection size measuring >= 10 cm (OR, 6.94; 95% CI, 1.76-27.45), paracolic extension of the PFC (OR, 3.79; 95% CI, 1.18-12.14), and >= 30 % solid necrosis (OR, 7.10; 95% CI, 1.16-43.48).

CONCLUSIONS: Half of all patients with PFCs drained with lumen-apposing metal stents required step-up therapy, most commonly direct endoscopic necrosectomy. Individuals with PFCs >= 10 cm in size, paracolic extension, or >= 30 % solid necrosis are more likely to require step-up therapy and should be considered for early endoscopic reintervention.
C1 [Chandrasekhara, Vinay; Storm, Andrew C.; Levy, Michael J.; Wang, Lillian; Majumder, Shounak; Vege, Santhi S.; Law, Ryan J.; Abu Dayyeh, Barham K.] Mayo Clin, Div Gastroenterol & Hepatol, 200 First St SW, Rochester, MN 55905 USA.
[Elhanafi, Sherif] Texas Tech Univ, Hlth Sci Ctr, Div Gastroenterol, El Paso, TX USA.
[Takahashi, Naoki; Lee, Nam Ju] Mayo Clin, Dept Radiol, Rochester, MN USA.
[Kaura, Karan] East Carolina Univ, Dept Internal Med, Greenville, NC USA.
C3 Mayo Clinic; Texas Tech University System; Texas Tech University Health Sciences Center El Paso; Texas Tech University; Mayo Clinic; University of North Carolina; East Carolina University
RP Chandrasekhara, V (corresponding author), Mayo Clin, Div Gastroenterol & Hepatol, 200 First St SW, Rochester, MN

55905 USA.
EM Chandrasekhara.vinay@mayo.edu
RI ; Elhanafi, Sherif/AAK-7871-2021; Wang, Lillian/LBH-7963-2024
OI Kaura, Karan/0009-0007-0308-2290; Chandrasekhara,
Vinay/0000-0001-6209-9905; Takahashi, Naoki/0000-0002-7946-6078;
Majumder, Shounak/0000-0001-7687-049X;
CR Abu Dayyeh BK, 2018, GASTROINTEST ENDOSC, V87, P141, DOI 10.1016/j.gie.2017.04.032
Abu Dayyeh BK, 2020, UNITED EUROPEAN GAST, V8, P13
Babu RY, 2013, ANN SURG, V257, P737, DOI 10.1097/SLA.0b013e318269d25d
Bang JY, 2019, GASTROENTEROLOGY, V156, P1027, DOI 10.1053/j.gastro.2018.11.031
Bang JY, 2020, TECH INNOV GASTROINT, V22, P14
Baron TH, 2020, GASTROENTEROLOGY, V158, P67, DOI 10.1053/j.gastro.2019.07.064
Binmoeller KF, 2020, TECH INNOVAT GASTROI, V22, P3, DOI 10.1016/j.tgie.2019.150653
Chandrasekhara V, 2020, ENDOSC INT OPEN, V08, pE1639, DOI 10.1055/a-1243-0092
Cotton PB, 2010, GASTROINTEST ENDOSC, V71, P446, DOI 10.1016/j.gie.2009.10.027
Fugazza A, 2020, GASTROINTEST ENDOSC, V91, P574, DOI 10.1016/j.gie.2019.11.021
He WH, 2017, SURG ENDOSC, V31, P3004, DOI 10.1007/s00464-016-5324-9
Hollemans RA, 2019, GASTROENTEROLOGY, V156, P1016, DOI 10.1053/j.gastro.2018.10.045
Morgan DE, 2010, CLIN GASTROENTEROL H, V8, P303, DOI 10.1016/j.cgh.2009.10.021
Mouli VP, 2013, GASTROENTEROLOGY, V144, P333, DOI 10.1053/j.gastro.2012.10.004
Ross AS, 2014, GASTROINTEST ENDOSC, V79, P929, DOI 10.1016/j.gie.2013.10.014
van Brunschot S, 2018, LANCET, V391, P51, DOI 10.1016/S0140-6736(17)32404-2
van Santvoort HC, 2011, GASTROENTEROLOGY, V141, P1254, DOI 10.1053/j.gastro.2011.06.073
van Santvoort HC, 2010, NEW ENGL J MED, V362, P1491, DOI 10.1056/NEJMoa0908821
Wang L, 2021, ENDOSCOPY, V53, P603, DOI 10.1055/a-1213-1489
NR 19
TC 60
Z9 70
U1 1
U2 4
PU ELSEVIER SCIENCE INC
PI NEW YORK
PA STE 800, 230 PARK AVE, NEW YORK, NY 10169 USA
SN 1542-3565
EI 1542-7714
J9 CLIN GASTROENTEROL H
JI Clin. Gastroenterol. Hepatol.
PD OCT
PY 2021
VL 19
IS 10
BP 2192

EP 2198
DI 10.1016/j.cgh.2021.05.005
EA SEP 2021
PG 7
WC Gastroenterology & Hepatology
WE Science Citation Index Expanded (SCI-EXPANDED)
SC Gastroenterology & Hepatology
GA YI9OR
UT WOS:000744170900031
PM 33965573
DA 2026-05-09
ER

PT J
AU Muniraj, T
Aslanian, HR
AF Muniraj, Thiruvengadam
Aslanian, Harry R.
TI Interventional EUS: Big Bang or Little Fizzle?
SO PRACTICAL GASTROENTEROLOGY
LA English
DT Article
ID GUIDED BILIARY DRAINAGE; CELIAC PLEXUS NEUROLYSIS; FORWARD-VIEWING ECHOENDOSCOPE; PANCREATIC CYSTIC LESIONS; GROUP DOCUMENT EVALUATION; ENDOSCOPIC-ULTRASOUND; TRANSMURAL DRAINAGE; SINGLE-STEP; GANGLIA NEUROLYSIS; FLUID COLLECTIONS
C1 [Muniraj, Thiruvengadam] MRCP, London, England.
[Aslanian, Harry R.] Yale Univ, Sect Digest Dis, New Haven, CT 06520 USA.
C3 Yale University
RP Muniraj, T (corresponding author), MRCP, London, England.
CR Ahn JY, 2010, GUT LIVER, V4, P524, DOI 10.5009/gnl.2010.4.4.524
Antillon MR, 2006, GASTROINTEST ENDOSC, V63, P797, DOI 10.1016/j.gie.2005.10.025
Ardengh JC, 2008, DIGEST DIS, V26, P370, DOI 10.1159/000177024
Artifon ELA, 2011, GASTROINTEST ENDOSC, V73, P1317, DOI 10.1016/j.gie.2010.10.041
Ascunce G, 2011, GASTROINTEST ENDOSC, V73, P267, DOI 10.1016/j.gie.2010.10.029
Ashida R, 2006, ENDOSCOPY, V38, P190, DOI 10.1055/s-2005-870404
Aslanian H, 2005, GASTROINTEST ENDOSC, V62, P723, DOI 10.1016/j.gie.2005.06.048
Attasaranya S, 2012, GASTROENT RES PRACT, V2012, DOI 10.1155/2012/680753
Binmoeller KF, 2011, GASTROINTEST ENDOSC, V74, P1019, DOI 10.1016/j.gie.2011.06.030
Bories E, 2007, ENDOSCOPY, V39, P287, DOI 10.1055/s-2007-966212
Brauer BC, 2009, GASTROINTEST ENDOSC, V70, P471, DOI 10.1016/j.gie.2008.12.233
Burmester E, 2003, GASTROINTEST ENDOSC, V57, P246, DOI 10.1067/mge.2003.85

Carrara S, 2013, WORLD J GASTRO ENDOS, V5, P141, DOI 10.4253/wjge.v5.i4.141
Catalano MF, 2005, GASTROINTEST ENDOSC, V61, pAB273, DOI 10.1016/S0016-5107(05)01417-3
Chang KJ, 2008, NAT CLIN PRACT GASTR, V5, P107, DOI 10.1038/ncpgasthep1033
Chang KJ, 2009, GASTROINTEST ENDOSC, V69, pS54, DOI 10.1016/j.gie.2008.10.057
CHANG KJ, 1994, GASTROINTEST ENDOSC, V40, P694, DOI 10.1016/S0016-5107(94)70112-1
De Lusong MA, 2008, GASTROINTEST ENDOSC, V68, P988, DOI 10.1016/j.gie.2008.05.028
de Paulo GA, 2006, GASTROINTEST ENDOSC, V63, P396, DOI 10.1016/j.gie.2005.10.039
Deprez PH, 2008, ACTA GASTRO-ENT BELG, V71, P333
DeWitt J, 2010, GASTROINTEST ENDOSC, V72, P862, DOI 10.1016/j.gie.2010.02.039
DeWitt J, 2009, GASTROINTEST ENDOSC, V70, P710, DOI 10.1016/j.gie.2009.03.1173
Di Matteo F, 2011, GASTROINTEST ENDOSC, V73, P632, DOI 10.1016/j.gie.2010.08.019
Diehl DL, 2012, GASTROINTEST ENDOSC, V75, P428, DOI 10.1016/j.gie.2011.09.041
DIMAGNO EP, 1980, LANCET, V1, P629
DiMaio CJ, 2011, PANCREAS, V40, P664, DOI 10.1097/MPA.0b013e3182128d06
Doi S, 2013, ENDOSCOPY, V45, P362, DOI 10.1055/s-0032-1326225
Easler J. J., 2013, GASTROINTEST ENDOSC, V77
Ergun M, 2011, ENDOSCOPY, V43, P518, DOI 10.1055/s-0030-1256333
ETZKORN KP, 1995, ENDOSCOPY, V27, P329, DOI 10.1055/s-2007-1005702
Fabbri C, 2011, ENDOSCOPY, V43, P438, DOI 10.1055/s-0030-1256097
Fockens P, 1997, GASTROINTEST ENDOSC, V46, P412, DOI 10.1016/S0016-5107(97)70033-6
Fockens P, 1996, GASTROINTEST ENDOSC, V44, P58, DOI 10.1016/S0016-5107(96)70230-4
Fockens P, 2002, GASTROINTEST ENDOSC, V56, pS93, DOI 10.1067/mge.2002.127756
Gaidhane M, 2012, GASTROENT RES PRACT, V2012, DOI 10.1155/2012/431451
Gan SI, 2005, GASTROINTEST ENDOSC, V61, P746, DOI 10.1016/S0016-5107(05)00320-2
Gerolami R, 1997, ENDOSCOPY, V29, P106, DOI 10.1055/s-2007-1004083
Giovannini M, 2001, ENDOSCOPY, V33, P898, DOI 10.1055/s-2001-17324
Giovannini M, 2001, ENDOSCOPY, V33, P473, DOI 10.1055/s-2001-14967
Gleeson FC, 2007, ENDOSCOPY, V39, P620, DOI 10.1055/s-2007-966337
Gluck M, 2010, CLIN GASTROENTEROL H, V8, P1083, DOI 10.1016/j.cgh.2010.09.010
Gonzalez JM, 2012, ENDOSCOPY, V44, P539, DOI 10.1055/s-0031-1291609
Gress FG, 2002, GASTROINTEST ENDOSC, V55, P594, DOI 10.1067/mge.2002.122580
Gunaratnam NT, 2001, GASTROINTEST ENDOSC, V54, P316, DOI 10.1067/mge.2001.117515
Gupta K., 2013, J CLIN GASTROENTEROL
Ha Tae-In, 2008, Korean Journal of Internal Medicine, V23, P5, DOI 10.3904/kjim.2008.23.1.5
Hara K, 2013, ENDOSCOPY, V45, P392, DOI 10.1055/s-0032-1326076
Hara K, 2011, AM J GASTROENTEROL, V106, P1239, DOI 10.1038/ajg.2011.84
Hecht JR, 2003, CLIN CANCER RES, V9, P555
Heinzow HS, 2011, SCAND J GASTROENTERO, V46, P1004, DOI 10.3109/00365521.2011.571709
Henry W. A., 2012, GASTROINTEST ENDOSC, V76, pe1061
Henry WA, 2012, GASTROINTEST ENDOSC, V76, P1065, DOI 10.1016/j.gie.2011.09.046
Herman JM, 2013, J CLIN ONCOL, V31, P886, DOI 10.1200/JCO.2012.44.7516
Hlavsa J, 2011, Klin Onkol, V24, P209

Hoffman BJ, 1997, GASTROINTEST ENDOSC, V45, P77, DOI 10.1016/S0016-5107(97)70306-7
Horaguchi J, 2009, DIGEST ENDOSC, V21, P239, DOI 10.1111/j.1443-1661.2009.00899.x
Ito K, 2010, DIGEST ENDOSC, V22, pS132, DOI 10.1111/j.1443-1661.2010.00971.x
Itoi T, 2012, GASTROINTEST ENDOSC, V75, P870, DOI 10.1016/j.gie.2011.10.020
Itoi T, 2010, J HEPATO-BIL-PAN SCI, V17, P611, DOI 10.1007/s00534-009-0196-1
Iwashita Takuji, 2012, Gastrointest Endosc Clin N Am, V22, P249, DOI 10.1016/j.giec.2012.04.018
Iwashita T, 2012, J GASTROEN HEPATOL, V27, P362, DOI 10.1111/j.1440-1746.2011.06923.x
Iwata K, 2011, DIGEST ENDOSC, V23, P140, DOI 10.1111/j.1443-1661.2010.01046.x
Jin Z, 2008, ENDOSCOPY, V40, P314, DOI 10.1055/s-2007-995476
Jin Zhendong, 2012, Gastrointest Endosc Clin N Am, V22, P325, DOI 10.1016/j.giec.2012.04.012
Jürgensen C, 2006, GASTROINTEST ENDOSC, V63, P1059, DOI 10.1016/j.gie.2005.10.034
Kahaleh M, 2006, GASTROINTEST ENDOSC, V64, P52, DOI 10.1016/j.gie.2006.01.063
Kahaleh M, 2007, GASTROINTEST ENDOSC, V65, P224, DOI 10.1016/j.gie.2006.05.008
Kahaleh M, 2013, WORLD J GASTROENTERO, V19, P1372, DOI 10.3748/wjg.v19.i9.1372
Kaufman M, 2010, J CLIN GASTROENTEROL, V44, P127, DOI 10.1097/MCG.0b013e3181bb854d
Keter Daniel, 2008, Acta Gastroenterologica Latinoamericana, V38, P146
Khashab MA, 2012, GASTROINTEST ENDOSC, V76, P209, DOI 10.1016/j.gie.2012.03.170
Kim YS, 2010, ENDOSCOPY, V42, P496, DOI 10.1055/s-0029-1244082
Klapman Jason B, 2005, Gastrointest Endosc Clin N Am, V15, P169, DOI 10.1016/j.giec.2004.07.009
Komaki T, 2011, PANCREATOLOGY, V11, P47, DOI 10.1159/000323508
Lahoti S, 2000, GASTROINTEST ENDOSC, V51, P331, DOI 10.1016/S0016-5107(00)70363-4
Lai R, 2005, ENDOSCOPY, V37, P487, DOI 10.1055/s-2005-861250
Lakhtakia S, 2011, INDIAN J GASTROENTER, V30, P277, DOI 10.1007/s12664-011-0149-x
Lankisch PG, 2001, PANCREATOLOGY, V1, P3, DOI 10.1159/000055786
Larghi A, 2009, GASTROINTEST ENDOSC, V69, P356, DOI 10.1016/j.gie.2008.08.038
Larghi A, 2010, GASTROINTEST ENDOSC, V72, P213, DOI 10.1016/j.gie.2009.11.003
LeBlanc JK, 2011, GASTROINTEST ENDOSC, V74, P1300, DOI 10.1016/j.gie.2011.07.073
Levy M, 2006, AM J GASTROENTEROL, V101, P1787, DOI 10.1111/j.1572-0241.2006.00685.x
Levy MJ, 2008, GASTROINTEST ENDOSC, V67, P572, DOI 10.1016/j.gie.2007.06.063
Levy MJ, 2008, AM J GASTROENTEROL, V103, P352, DOI 10.1111/j.1572-0241.2007.01616.x
Levy MJ, 2008, AM J GASTROENTEROL, V103, P98, DOI 10.1111/j.1572-0241.2007.01607.x
Levy Michael J, 2012, Gastrointest Endosc Clin N Am, V22, P231, DOI 10.1016/j.giec.2012.04.003
Levy MJ, 2012, GASTROINTEST ENDOSC, V75, P200, DOI 10.1016/j.gie.2011.09.019
Lowe A. S., 2005, GASTROINTEST ENDOSC, V15, pxi
Lowe Andrew S, 2005, Gastrointest Endosc Clin N Am, V15, P209, DOI 10.1016/j.giec.2004.07.011
Mallery S, 2004, GASTROINTEST ENDOSC, V59, P100, DOI 10.1016/S0016-5107(03)02300-9
Maranki J, 2009, ENDOSCOPY, V41, P532, DOI 10.1055/s-0029-1214712
Moore DC, 2004, REGION ANESTH PAIN M, V29, P377, DOI 10.1016/j.rapm.2004.02.001
Nakaji S, 2012, Endoscopy, V44 Suppl 2 UCTN, pE380, DOI 10.1055/s-0032-1309918
Navarro-Martinez J, 2003, REGION ANESTH PAIN M, V28, P528, DOI 10.1016/S1098-7339(03)00232-3
Nguyen-Tang T, 2010, ENDOSCOPY, V42, P232, DOI 10.1055/s-0029-1243858
Nguyen-Tang T, 2010, GASTROINTEST ENDOSC, V72, P606, DOI 10.1016/j.gie.2010.03.1126

O'Toole TM, 2009, ENDOSCOPY, V41, P593, DOI 10.1055/s-0029-1214868
Oh HC, 2008, GASTROINTEST ENDOSC, V67, P636, DOI 10.1016/j.gie.2007.09.038
Oh HC, 2012, DIGEST DIS SCI, V57, P1965, DOI 10.1007/s10620-012-2103-x
Oh HC, 2011, J DIGEST DIS, V12, P497, DOI 10.1111/j.1751-2980.2011.00538.x
Oh HC, 2011, GASTROENTEROLOGY, V140, P172, DOI 10.1053/j.gastro.2010.10.001
Owens DJ, 2009, GASTROINTEST ENDOSC, V69, P972, DOI 10.1016/j.gie.2008.05.052
Panamonta N, 2012, EUR J GASTROEN HEPAT, V24, P1355, DOI 10.1097/MEG.0b013e32835871eb
Park DH, 2009, ENDOSCOPY, V41, P842, DOI 10.1055/s-0029-1215133
Park DH, 2011, GASTROINTEST ENDOSC, V74, P1276, DOI 10.1016/j.gie.2011.07.054
Park WG, 2010, GASTROINTEST ENDOSC, V71, P513, DOI 10.1016/j.gie.2009.10.030
Penn DE, 2012, GASTROINTEST ENDOSC, V76, P679, DOI 10.1016/j.gie.2012.04.457
Pishvaian AC, 2006, GASTROINTEST ENDOSC, V64, P412, DOI 10.1016/j.gie.2006.01.048
Puli SR, 2009, DIGEST DIS SCI, V54, P2330, DOI 10.1007/s10620-008-0651-x
Püspök A, 2005, AM J GASTROENTEROL, V100, P1743, DOI 10.1111/j.1572-0241.2005.41806.x
Ramesh J, 2012, ENDOSCOPY, V44, pE327, DOI 10.1055/s-0032-1309855
Ramírez-Luna MA, 2011, ENDOSCOPY, V43, P826, DOI 10.1055/s-0030-1256406
Romero-Castro R, 2007, GASTROINTEST ENDOSC, V66, P402, DOI 10.1016/j.gie.2007.03.008
Sahai AV, 2009, AM J GASTROENTEROL, V104, P326, DOI 10.1038/ajg.2008.64
Sahakian A. B., 2013, J CLIN GASTROENTEROL
SAHEL J, 1987, PANCREAS, V2, P447, DOI 10.1097/00006676-198707000-00012
SAHEL J, 1988, GASTROEN CLIN BIOL, V12, P431
Sakamoto H, 2010, AM J GASTROENTEROL, V105, P2599, DOI 10.1038/ajg.2010.339
Sanders MK, 2010, GASTROINTEST ENDOSC, V71, P1178, DOI 10.1016/j.gie.2009.12.020
Schiano TD, 1996, GASTROINTEST ENDOSC, V44, P151, DOI 10.1016/S0016-5107(96)70132-3
Schnack Christina, 2012, Ugeskr Laeger, V174, P501
Siddiqui A. A., 2013, GASTROINTESTINAL END
Talreja JP, 2008, GASTROINTEST ENDOSC, V68, P1199, DOI 10.1016/j.gie.2008.06.015
Tarantino I, 2008, ENDOSCOPY, V40, P336, DOI 10.1055/s-2007-995455
Tessier G, 2007, GASTROINTEST ENDOSC, V65, P233, DOI 10.1016/j.gie.2006.06.029
Trevino JM, 2009, GASTROINTEST ENDOSC, V69, P361, DOI 10.1016/j.gie.2008.09.021
Uchida N, 2010, GASTROINTEST ENDOSC, V72, P1321, DOI 10.1016/j.gie.2010.03.1068
Varadarajulu S, 2004, GASTROINTEST ENDOSC, V60, P631, DOI 10.1016/S0016-5107(04)01891-7
Varadarajulu S, 2010, ENDOSCOPY, V42, P423, DOI 10.1055/s-0029-1243989
Varadarajulu S, 2008, GASTROINTEST ENDOSC, V68, P649, DOI 10.1016/j.gie.2008.02.057
Varadarajulu S, 2007, GASTROINTEST ENDOSC, V66, P1107, DOI 10.1016/j.gie.2007.03.1027
Varadarajulu S, 2011, GASTROINTEST ENDOSC, V74, P74, DOI 10.1016/j.gie.2011.03.1122
Varadarajulu S, 2009, GASTROINTEST ENDOSC, V69, pS32, DOI 10.1016/j.gie.2008.11.005
VENTAFRIDDA GV, 1990, EUR J SURG ONCOL, V16, P1
VILMANN P, 1992, GASTROINTEST ENDOSC, V38, P172, DOI 10.1016/S0016-5107(92)70385-X
Voermans RP, 2007, GASTROINTEST ENDOSC, V66, P1013, DOI 10.1016/j.gie.2007.02.057
Wiersema MJ, 1996, GASTROINTEST ENDOSC, V44, P656, DOI 10.1016/S0016-5107(96)70047-0
Wyse JM, 2011, J CLIN ONCOL, V29, P3541, DOI 10.1200/JCO.2010.32.2750

Yamao K, 2008, ENDOSCOPY, V40, P340, DOI 10.1055/s-2007-995485
Yamao K, 2006, GASTROINTEST ENDOSC, V64, P663, DOI 10.1016/j.gie.2006.07.003
Yan BM, 2007, AM J GASTROENTEROL, V102, P430, DOI 10.1111/j.1572-0241.2006.00967.x
Yang J, 2009, GASTROINTEST ENDOSC, V70, P579, DOI 10.1016/j.gie.2009.03.001
Yoon Won Jae, 2012, Gastrointest Endosc Clin N Am, V22, P359, DOI 10.1016/j.giec.2012.04.017
NR 143
TC 0
Z9 0
U1 0
U2 0
PU SHUGAR PUBLISHING INC
PI WESTHAMPTON BEACH
PA 32 MILL ROAD, WESTHAMPTON BEACH, NY 11978 USA
SN 0277-4208
J9 PRACT GASTROENTEROL
JI Pract. Gasterontol.
PD AUG
PY 2013
VL 37
IS 8
BP 11
EP 24
PG 11
WC Gastroenterology & Hepatology
WE Emerging Sources Citation Index (ESCI)
SC Gastroenterology & Hepatology
GA V8W6D
UT WOS:000422033400001
DA 2026-05-09
ER

PT J
AU Tyberg, A
Jha, K
Shah, S
Kedia, P
Gaidhane, M
Kahaleh, M
AF Tyberg, Amy
Jha, Kopal
Shah, Shawn
Kedia, Prashant

Gaidhane, Monica
Kahaleh, Michel
TI EUS-guided gallbladder drainage: a learning curve modified by technical progress
SO ENDOSCOPY INTERNATIONAL OPEN
LA English
DT Article
ID APPOSING METAL STENT; ACUTE CHOLECYSTITIS; PERCUTANEOUS CHOLECYSTOSTOMY; FEASIBILITY; PLACEMENT; ACCESS; ERCP
AB Introduction Endoscopic ultrasound-guided gallbladder drainage (EUS-GBD) is an efficacious and safe option for patients who cannot undergo cholecystectomy. It is a technically challenging procedure, requiring skills in EUS, and ERCP. The aim of this study was to define the learning curve for EUS-GBD. Patients and methods Consecutive patients undergoing EUS-GBD by a single operator were included from a prospective registry over 5 years. Demographics, procedure information, post-procedure follow-up data, and information on adverse events were collected. Non-linear regression and CUSUM analyses were conducted for the learning curve. Clinical success was defined as resolution of cholecystitis post-procedure. Results Forty-eight patients were included (58 % male, mean age 76 years). Twenty patients (42 %) had malignant cholecystitis. Most patients had lumen-apposing metal stents (LAMS) (15 mm, n = 29, 60 %; 10 mm, n = 8, 7 %). The remaining patients had FCSEMS (n = 9, 19 %) or plastic stents alone (n = 2, 4 %). Clinical success was achieved in 36 (86 %) of patients. Of the remaining 12, 7 were lost to follow-up and 5 had persistent cholecystitis. 9 patients (19 %) had adverse events including bleeding (n = 4), liver abscesses (n = 2), and hypotension. Two patients passed away post-procedure. Median procedure time was 41 minutes (range 16 - 121 min), with the 41-minute time occurring during the 19th procedure. Procedure durations further reduced, with the last 10 procedures being 20 minutes or under (nonlinear regression p value P < 0.0001). Conclusion Endoscopists experienced in EUS-GBD are expected to achieve a reduction in procedure time over successive cases, with efficiency reached at 41 minutes and a learning rate of 19 cases. Continued improvement is demonstrated with additional experience
C1 [Tyberg, Amy; Gaidhane, Monica; Kahaleh, Michel] Rutgers Robert Wood Johnson Med Ctr, Div Gastroenterol, New Brunswick, NJ USA.
[Jha, Kopal] Cornell Univ, Ithaca, NY USA.
[Shah, Shawn] Weill Cornell Med, W, New York, NY USA.
[Kedia, Prashant] Methodist Hlth Syst, Dallas, TX USA.
C3 Rutgers University System; Rutgers University New Brunswick; Rutgers University Biomedical & Health Sciences; Cornell University; Cornell University; Weill Cornell Medicine
RP Kahaleh, M (corresponding author), Rutgers State Univ, Robert Wood Johnson Univ Hosp, Gastroenterol, 1 RWJ Pl,MEB 464, New Brunswick, NJ 08901 USA.
EM mkahaleh@gmail.com
RI Kahaleh, Michel/A-9408-2011; Gaidhane, Monica/B-9414-2009
OI Gaidhane, Monica/0000-0001-6773-7080
CR Adler DG, 2019, GASTROINTEST ENDOSC, V89, P299, DOI 10.1016/j.gie.2018.09.010
Akhan O, 2002, EUR J RADIOL, V43, P229, DOI 10.1016/S0720-048X(02)00158-4
Anderloni A, 2016, SURG ENDOSC, V30, P5200, DOI 10.1007/s00464-016-4894-x
Súbtil JC, 2010, WORLD J GASTRO ENDOS, V2, P203, DOI 10.4253/wjge.v2.i6.203

de la Serna-Higuera C, 2013, GASTROINTEST ENDOSC, V77, P303, DOI 10.1016/j.gie.2012.09.021
Duncan CB, 2012, J GASTROINTEST SURG, V16, P2011, DOI 10.1007/s11605-012-2024-1
Hatzidakis AA, 2002, EUR RADIOL, V12, P1778, DOI 10.1007/s00330-001-1247-4
Irani S, 2017, CLIN GASTROENTEROL H, V15, P738, DOI 10.1016/j.cgh.2016.12.021
Irani S, 2015, GASTROINTEST ENDOSC, V82, P1110, DOI 10.1016/j.gie.2015.05.045
Itoi T, 2012, GASTROINTEST ENDOSC, V75, P870, DOI 10.1016/j.gie.2011.10.020
Jang JW, 2011, GASTROINTEST ENDOSC, V74, P176, DOI 10.1016/j.gie.2011.03.1120
Minaga K, 2019, DIGEST ENDOSC, V31, P180, DOI 10.1111/den.13242
Moon JH, 2014, GASTROINTEST ENDOSC, V79, P990, DOI 10.1016/j.gie.2014.02.015
Morse BC, 2010, AM SURGEON, V76, P708
Oh D, 2017, THER ADV GASTROENTER, V10, P42, DOI 10.1177/1756283X16671671
Poincloux L, 2015, ENDOSCOPY, V47, P794, DOI [10.1055/s-0035-1545399, 10.1055/s-0034-1391988]
Saumoy M, 2019, J CLIN GASTROENTEROL, V53, P231, DOI 10.1097/MCG.0000000000001036
Song TJ, 2010, GASTROINTEST ENDOSC, V71, P634, DOI 10.1016/j.gie.2009.11.024
Teoh AYB, 2017, ENDOSCOPY, V49, P130, DOI 10.1055/s-0042-119036
Teoh AYB, 2014, GASTROINTEST ENDOSC, V80, P1171, DOI 10.1016/j.gie.2014.03.038
Tyberg A, 2018, J CLIN GASTROENTEROL, V52, P79, DOI 10.1097/MCG.0000000000000786
Tyberg A, 2017, ENDOSCOPY, V49, P1007, DOI 10.1055/s-0043-113551
Tyberg A, 2016, GASTROINTEST ENDOSC, V83, P254, DOI 10.1016/j.gie.2015.08.003
Widmer J, 2014, DIGEST ENDOSC, V26, P599, DOI 10.1111/den.12163
Yamashita Y, 2007, J HEPATO-BILIARY-PAN, V14, P91, DOI 10.1007/s00534-006-1161-x
NR 25
TC 32
Z9 35
U1 0
U2 3
PU GEORG THIEME VERLAG KG
PI STUTTGART
PA RUDIGERSTR 14, D-70469 STUTTGART, GERMANY
SN 2364-3722
EI 2196-9736
J9 ENDOSC INT OPEN
JI Endosc. Int. Open
PD JAN
PY 2020
VL 8
IS 1
BP E92
EP E96
DI 10.1055/a-1005-6602
PG 5
WC Gastroenterology & Hepatology; Surgery

WE Emerging Sources Citation Index (ESCI)
SC Gastroenterology & Hepatology; Surgery
GA KB4DE
UT WOS:000506447500015
PM 31921991
OA Green Submitted, gold
DA 2026-05-09
ER

PT J
AU Matsubara, S
Isayama, H
Nakai, Y
Kawakubo, K
Yamamoto, N
Saito, K
Saito, T
Takahara, N
Mizuno, S
Kogure, H
Ishizawa, T
Arita, J
Hasegawa, K
Koike, K
AF Matsubara, Saburo
Isayama, Hiroyuki
Nakai, Yousuke
Kawakubo, Kazumichi
Yamamoto, Natsuyo
Saito, Kei
Saito, Tomotaka
Takahara, Naminatsu
Mizuno, Suguru
Kogure, Hirofumi
Ishizawa, Takeaki
Arita, Junichi
Hasegawa, Kiyoshi
Koike, Kazuhiko
TI Endoscopic ultrasound-guided gallbladder drainage with a combined internal and external drainage tubes for acute cholecystitis
SO JOURNAL OF GASTROENTEROLOGY AND HEPATOLOGY
LA English

DT Article
DE acute cholecystitis; drainage; endoscopic ultrasound; gallbladder; stent
ID APPOSING METAL STENT; BILIARY; CHOLECYSTECTOMY; CHOLECYSTOSTOMY; MANAGEMENT
AB Background and Aim Lumen-apposing metal stent is widely used for endoscopic ultrasound-guided gallbladder drainage (EUS-GBD) nowadays but not approved in many countries and might be unsuitable for elective laparoscopic cholecystectomy (LC) because of its large enterocholecysto fistula. A combination of double pigtail plastic stent (DPPS) and naso-cystic tube (NCT) could overcome these problems. The aim of this study was to estimate the efficacy and safety of this method in patients with acute cholecystitis unfit for urgent cholecystectomy both as bridge to surgery and palliation.

Methods This was a prospective, single-center feasibility study. EUS-GBD was performed with a 7Fr DPPS followed by an NCT placement. NCT was removed after 1 week. LC was performed 2 or 3 months after EUS-GBD in eligible patients. In patients who did not underwent cholecystectomy, DPPS was left in place.

Results Twenty-three patients were enrolled. Both technical and clinical success rates were 96% (22/23). Early adverse events rate was 13% (3/23), including one bile peritonitis, one intraperitoneal abscess, and one melena. LC was attempted in 12 patients, and conversion to open cholecystectomy was required in three (25%). Neither recurrence of cholecystitis nor late adverse event occurred during 6 months of follow up in 10 patients who did not undergo cholecystectomy.

Conclusion EUS-GBD with a combination of DPPS and NCT is considered an effective and safe technique both as bridge to surgery and palliation.
C1 [Matsubara, Saburo; Isayama, Hiroyuki; Nakai, Yousuke; Kawakubo, Kazumichi; Yamamoto, Natsuyo; Saito, Kei; Saito, Tomotaka; Takahara, Naminatsu; Mizuno, Suguru; Kogure, Hirofumi; Koike, Kazuhiko] Univ Tokyo, Grad Sch Med, Dept Gastroenterol, Tokyo, Japan.

[Ishizawa, Takeaki; Arita, Junichi; Hasegawa, Kiyoshi] Univ Tokyo, Grad Sch Med, Hepatobiliary Pancreat Surg Div, Dept Surg, Tokyo, Japan.

[Ishizawa, Takeaki; Arita, Junichi; Hasegawa, Kiyoshi] Univ Tokyo, Grad Sch Med, Artificial Organ & Transplantat Div, Dept Surg, Tokyo, Japan.

[Isayama, Hiroyuki] Juntendo Univ, Grad Sch Med, Dept Gastroenterol, Tokyo, Japan.

[Yamamoto, Natsuyo] Fratern Mem Hosp, Dept Gastroenterol, Tokyo, Japan.

[Matsubara, Saburo] Saitama Med Univ, Dept Gastroenterol & Hepatol, Saitama Med Ctr, Kawagoe, Saitama, Japan.

[Kawakubo, Kazumichi] Hokkaido Univ, Grad Sch Med, Dept Gastroenterol, Sapporo, Hokkaido, Japan.

C3 University of Tokyo; University of Tokyo; University of Tokyo; Juntendo University; Saitama Medical University; Hokkaido University

RP Isayama, H (corresponding author), Juntendo Univ, Grad Sch Med, Dept Gastroenterol, Bunkyo Ku, 3-1-3 Hongo, Tokyo 1138431, Japan.

EM h-isayama@juntendo.ac.jp

RI Arita, Junichi/ABB-8635-2020; Mizuno, Suguru/AAF-8867-2019; Takahara, Naminatsu/K-8497-2019; Isayama, Hiroyuki/AER-9916-2022; Kawakubo, Kazumichi/F-2937-2011; Nakai, Yousuke/H-2964-2019

OI Nakai, Yousuke/0000-0001-7411-1385; Matsubara, Saburo/0000-0001-8182-3524
CR Anderloni A, 2016, SURG ENDOSC, V30, P5200, DOI 10.1007/s00464-016-4894-x

Baron TH, 2007, GASTROINTEST ENDOSC, V65, P735, DOI 10.1016/j.gie.2006.07.041

Baron TH, 2015, NEW ENGL J MED, V373, P357, DOI 10.1056/NEJMra1411372

Chantarojanasiri T, 2017, SAUDI J GASTROENTERO, V23, P218, DOI 10.4103/sjg.SJG_115_17
Choi JH, 2014, ENDOSCOPY, V46, P656, DOI 10.1055/s-0034-1365720
Cotton PB, 2010, GASTROINTEST ENDOSC, V71, P446, DOI 10.1016/j.gie.2009.10.027
de la Serna-Higuera C, 2013, GASTROINTEST ENDOSC, V77, P303, DOI 10.1016/j.gie.2012.09.021
Dhir V, 2017, DIGEST ENDOSC, V29, P472, DOI 10.1111/den.12818
Doi S, 2018, DIGEST ENDOSC, V30, P501, DOI 10.1111/den.13028
Dollhopf M, 2017, GASTROINTEST ENDOSC, V86, P636, DOI 10.1016/j.gie.2017.02.027
Hirota M, 2007, J HEPATO-BILIARY-PAN, V14, P78, DOI 10.1007/s00534-006-1159-4
Irani S, 2017, CLIN GASTROENTEROL H, V15, P738, DOI 10.1016/j.cgh.2016.12.021
Irani S, 2015, GASTROINTEST ENDOSC, V82, P1110, DOI 10.1016/j.gie.2015.05.045
Itoi T, 2011, J HEPATO-BIL-PAN SCI, V18, P282, DOI 10.1007/s00534-010-0310-4
Jang JW, 2012, GASTROENTEROLOGY, V142, P805, DOI 10.1053/j.gastro.2011.12.051
Kahaleh M, 2016, DIGEST LIVER DIS, V48, P1054, DOI 10.1016/j.dld.2016.05.021
Kamata K, 2010, ENDOSCOPY, V42, pE331, DOI 10.1055/s-0030-1255941
Kamata K, 2009, ENDOSCOPY, V41, pE315, DOI 10.1055/s-0029-1215258
Kim JJ, 2019, J CLIN GASTROENTEROL, V53, P392, DOI 10.1097/MCG.0000000000000890
Kwan V, 2007, GASTROINTEST ENDOSC, V66, P582, DOI 10.1016/j.gie.2007.02.065
Lee SS, 2007, GASTROINTEST ENDOSC, V66, P1008, DOI 10.1016/j.gie.2007.03.1080
McKay A, 2012, SURG ENDOSC, V26, P1343, DOI 10.1007/s00464-011-2035-0
Nakai Y, 2017, DIGEST ENDOSC, V29, P218, DOI 10.1111/den.12752
Ogura T, 2019, ENDOSC INT OPEN, V7, pE1652, DOI 10.1055/a-0961-7890
Okamoto K, 2018, J HEPATO-BIL-PAN SCI, V25, P55, DOI 10.1002/jhbp.516
Ryozawa S, 2017, DIGEST ENDOSC, V29, P559, DOI 10.1111/den.12872
Seerden TC, 2016, ENDOSCOPY, V48, pE179, DOI 10.1055/s-0042-107073
Song TJ, 2010, GASTROINTEST ENDOSC, V71, P634, DOI 10.1016/j.gie.2009.11.024
Takasawa O, 2009, DIGEST ENDOSC, V21, P43, DOI 10.1111/j.1443-1661.2008.00822.x
Teoh AYB, 2017, ENDOSCOPY, V49, P130, DOI 10.1055/s-0042-119036
Walter D, 2016, GUT, V65, P6, DOI 10.1136/gutjnl-2015-309925
NR 31
TC 14
Z9 15
U1 0
U2 3
PU WILEY
PI HOBOKEN
PA 111 RIVER ST, HOBOKEN 07030-5774, NJ USA
SN 0815-9319
EI 1440-1746
J9 J GASTROEN HEPATOL
JI J. Gastroenterol. Hepatol.
PD OCT
PY 2020

VL 35
IS 10
BP 1821
EP 1827
DI 10.1111/jgh.15065
EA APR 2020
PG 7
WC Gastroenterology & Hepatology
WE Science Citation Index Expanded (SCI-EXPANDED)
SC Gastroenterology & Hepatology
GA NY8GL
UT WOS:000529054700001
PM 32267555
DA 2026-05-09
ER

PT J
AU Canakis, A
Kahaleh, M
AF Canakis, Andrew
Kahaleh, Michel
TI Endoscopic palliation of malignant biliary obstruction
SO WORLD JOURNAL OF GASTROINTESTINAL ENDOSCOPY
LA English
DT Review
DE Biliary obstruction; Endoscopic retrograde cholangiopancreatography;
Endoscopic ultrasonography; Stenting; Ablation therapy
ID EXPANDABLE METAL STENTS; BILE-DUCT OBSTRUCTION; EUS-GUIDED
CHOLEDOCHODUODENOSTOMY; RADIOFREQUENCY ABLATION; PHOTODYNAMIC THERAPY;
FAILED ERCP; UNRESECTABLE CHOLANGIOCARCINOMA; RANDOMIZED-TRIAL;
NONRESECTABLE CHOLANGIOCARCINOMA; INTERNATIONAL MULTICENTER
AB Malignant biliary obstruction often presents with challenges requiring the endoscopist to assess the location of the lesion, the staging of the disease, the eventual resectability and patient preferences in term of biliary decompression. This review will focus on the different modalities available in order to offer the most appropriate palliation, such as conventional endoscopic retrograde cholangiopancreatography, endoscopic ultrasound guided biliary drainage as well as ablative therapies including photodynamic therapy or radiofrequency ablation.
C1 [Canakis, Andrew] Univ Maryland, Dept Gastroenterol & Hepatol, Sch Med, Baltimore, MD 21201 USA.
[Kahaleh, Michel] Robert Wood Johnson Med Ctr, Dept Gastroenterol & Hepatol, New Brunswick, NJ 08901 USA.
[Kahaleh, Michel] Robert Wood Johnson Med Ctr, Dept Gastroenterol & Hepatol, 1 Robert Wood Johnson Pl, New Brunswick, NJ 08901 USA.
C3 University System of Maryland; University of Maryland Baltimore; Rutgers
University System; Rutgers University New Brunswick; Rutgers University

Biomedical & Health Sciences; Rutgers University System; Rutgers
University New Brunswick; Rutgers University Biomedical & Health
Sciences
RP Kahaleh, M (corresponding author), Robert Wood Johnson Med Ctr, Dept Gastroenterol & Hepatol, 1 Robert Wood Johnson Pl, New Brunswick, NJ 08901 USA.
EM mkahaleh@gmail.com
RI Kahaleh, Michel/A-9408-2011
FU Boston Scientific; Apollo; Olympus; Cook; Microtech; Fuji
FX Dr. Kahaleh is a consultant for Boston Scientific, Medtronic and Abvvie,
he has received research grant from Boston Scientific, Apollo, Olympus,
Cook, Microtech and Fuji; Dr Canakis has no disclose.
CR Abraham NS, 2002, GASTROINTEST ENDOSC, V56, P835, DOI 10.1067/mge.2002.129868
Almadi MA, 2017, AM J GASTROENTEROL, V112, P260, DOI 10.1038/ajg.2016.512
de Lima SLA, 2015, INT J HEPATOBILIARY, V5, P35, DOI 10.5348/ijhpd-2015-32-CR-7
Amano M, 2017, J GASTROEN HEPATOL, V32, P716, DOI 10.1111/jgh.13489
Amato A, 2021, ENDOSCOPY, V53, P1037, DOI 10.1055/a-1324-7919
Ardengh JC, 2018, WORLD J GASTRO ENDOS, V10, P99, DOI 10.4253/wjge.v10.i5.99
Artifon ELA, 2015, GASTROINTEST ENDOSC, V81, P950, DOI 10.1016/j.gie.2014.09.047
ASGE Technology Committee, 2017, VideoGIE, V2, P252, DOI [10.1016/j.vgie.2017.06.002, 10.1016/j.vgie.2017.06.002]
Attasaranya S, 2012, GASTROENT RES PRACT, V2012, DOI 10.1155/2012/680753
BISMUTH H, 1982, WORLD J SURG, V6, P3, DOI 10.1007/BF01656368
BISMUTH H, 1975, SURG GYNECOL OBSTET, V140, P170
Boskoski I, 2021, J PERS MED, V11, DOI 10.3390/jpm11020078
Boulay Brian R, 2018, Gastrointest Endosc Clin N Am, V28, P171, DOI [10.1016/j.giec.2017.11.005, 10.1016/j.giec.2017.11.005]
Burmester E, 2003, GASTROINTEST ENDOSC, V57, P246, DOI 10.1067/mge.2003.85
Canakis A, 2022, J HEPATO-BIL-PAN SCI, V29, P941, DOI 10.1002/jhbp.1055
Canakis A, 2020, BMJ OPEN GASTROENTER, V7, DOI 10.1136/bmjgast-2020-000428
Canakis A, 2020, GASTROINTEST ENDOSC, V91, P520, DOI 10.1016/j.gie.2019.09.037
Chang G, 2017, ABDOM RADIOL, V42, P2745, DOI 10.1007/s00261-017-1174-8
Chang WH, 1998, GASTROINTEST ENDOSC, V47, P354, DOI 10.1016/S0016-5107(98)70218-4
Cho DH, 2017, GASTROINTEST ENDOSC, V85, P1067, DOI 10.1016/j.gie.2016.09.010
Choi J, 2012, HEPATOB PANCREAT DIS, V11, P636, DOI 10.1016/S1499-3872(12)60237-9
Conio M, 2018, GASTROINTEST ENDOSC, V88, P283, DOI 10.1016/j.gie.2018.03.029
Sanz MD, 2021, SURG ENDOSC, V35, P6754, DOI 10.1007/s00464-020-08179-y
Dhar J, 2022, CLIN J GASTROENTEROL, V15, P11, DOI 10.1007/s12328-021-01559-4
Dhir V, 2015, GASTROINTEST ENDOSC, V81, P913, DOI 10.1016/j.gie.2014.09.054
Distler M, 2010, J PANCREAS, V11, P568
Dumonceau JM, 2018, ENDOSCOPY, V50, P910, DOI 10.1055/a-0659-9864
Dutta AK, 2017, EXPERT REV GASTROENT, V11, P779, DOI 10.1080/17474124.2017.1314784
Figueroa-Barojas P, 2013, J ONCOL, V2013, DOI 10.1155/2013/910897

Fugazza A, 2022, GASTROINTEST ENDOSC, V95, P896, DOI 10.1016/j.gie.2021.12.032
Gao DJ, 2021, GASTROINTEST ENDOSC, V94, P91, DOI 10.1016/j.gie.2020.12.016
Giovannini M, 2001, ENDOSCOPY, V33, P898, DOI 10.1055/s-2001-17324
Gonzalez-Carmona MA, 2019, ALIMENT PHARM THER, V49, P437, DOI 10.1111/apt.15050
Guo, MULTIINSTITUTION CON
Guo JT, 2016, GASTROENT RES PRACT, V2016, DOI 10.1155/2016/9469472
Gupta K, 2014, J CLIN GASTROENTEROL, V48, P80, DOI 10.1097/MCG.0b013e31828c6822
Han SJ, 2017, GASTROINTEST ENDOSC, V85, pAB88, DOI 10.1016/j.gie.2017.04.037
Hathorn KE, 2022, GASTROINTEST ENDOSC, V95, P443, DOI 10.1016/j.gie.2021.10.013
Hidalgo M, 2010, NEW ENGL J MED, V362, P1605, DOI 10.1056/NEJMra0901557
Holt BA, 2016, GASTROINTEST ENDOSC, V83, P160, DOI 10.1016/j.gie.2015.06.019
Hong WD, 2014, CLINICS, V69, P647, DOI 10.6061/clinics/2014(09)12
Huh G, 2022, GASTROINTEST ENDOSC, V95, P452, DOI 10.1016/j.gie.2021.11.016
Hwang JC, 2011, SCAND J GASTROENTERO, V46, P326, DOI 10.3109/00365521.2010.536253
Inamdar S, 2016, JAMA ONCOL, V2, P112, DOI 10.1001/jamaoncol.2015.3670
Inoue T, 2022, DIGEST ENDOSC, V34, P345, DOI 10.1111/den.14059
Inoue T, 2020, CLIN TRANSL GASTROEN, V11, DOI 10.14309/ctg.0000000000000250
Inoue T, 2018, DIGEST ENDOSC, V30, P395, DOI 10.1111/den.13016
Isayama H, 2004, GUT, V53, P729, DOI 10.1136/gut.2003.018945
Isayama H, 2015, DIGEST ENDOSC, V27, P259, DOI 10.1111/den.12379
Isayama H, 2011, DIGEST ENDOSC, V23, P310, DOI 10.1111/j.1443-1661.2011.01124.x
Ishigaki K, 2020, DIGEST DIS SCI, V65, P3710, DOI 10.1007/s10620-020-06155-z
Jacques J, 2019, ENDOSCOPY, V51, P540, DOI 10.1055/a-0735-9137
Jang SI, 2019, ENDOSCOPY, V51, P843, DOI 10.1055/a-0754-5763
Jang SI, 2017, J GASTROEN HEPATOL, V32, P932, DOI 10.1111/jgh.13602
John ES, 2021, TRANSL GASTROENT HEP, V6, DOI 10.21037/tgh.2020.02.03
Kahaleh M, 2008, CLIN GASTROENTEROL H, V6, P290, DOI 10.1016/j.cgh.2007.12.004
Kallis Y, 2015, DIGEST DIS SCI, V60, P3449, DOI 10.1007/s10620-015-3731-8
Kang H, 2021, SURG ENDOSC, V35, P63, DOI 10.1007/s00464-020-07689-z
Kawakubo K, 2014, J HEPATO-BIL-PAN SCI, V21, P328, DOI 10.1002/jhbp.27
Keswani RN, 2017, CLIN GASTROENTEROL H, V15, P1866, DOI 10.1016/j.cgh.2017.06.002
Khashab MA, 2016, ENDOSC INT OPEN, V4, pE175, DOI 10.1055/s-0041-109083
Kim KM, 2012, HEPATO-GASTROENTEROL, V59, P341, DOI 10.5754/hge11533
Kim TH, 2012, WORLD J GASTROENTERO, V18, P2526, DOI 10.3748/wjg.v18.i20.2526
Kitano M, 2013, AM J GASTROENTEROL, V108, P1713, DOI 10.1038/ajg.2013.305
Kloek JJ, 2010, J GASTROINTEST SURG, V14, P119, DOI 10.1007/s11605-009-1009-1
Kogure H, 2011, SURG ENDOSC, V25, P463, DOI 10.1007/s00464-010-1194-8
Kullman E, 2010, GASTROINTEST ENDOSC, V72, P915, DOI 10.1016/j.gie.2010.07.036
Laleman W, 2017, ENDOSCOPY, V49, P977, DOI 10.1055/s-0043-113559
Lee DW, 2022, DIGEST DIS SCI, V67, P1635, DOI 10.1007/s10620-022-07394-y
Lee JH, 2013, GASTROINTEST ENDOSC, V78, P312, DOI 10.1016/j.gie.2013.02.032
Lee JH, 2007, GASTROINTEST ENDOSC, V66, P364, DOI 10.1016/j.gie.2006.12.061

Lee SH, 2007, WORLD J GASTROENTERO, V13, P3948, DOI 10.3748/wjg.v13.i29.3948
Lee SJ, 2014, J VASC INTERV RADIOL, V25, P1912, DOI 10.1016/j.jvir.2014.05.021
Lee TH, 2020, DIGEST ENDOSC, V32, P275, DOI 10.1111/den.13549
Lee TH, 2016, CLIN GASTROENTEROL H, V14, P1011, DOI 10.1016/j.cgh.2015.12.032
Lee TY, 2012, WORLD J GASTROENTERO, V18, P5589, DOI 10.3748/wjg.v18.i39.5589
Lee YN, 2019, J GASTROEN HEPATOL, V34, P1454, DOI 10.1111/jgh.14657
Leggett CL, 2012, PHOTODIAGN PHOTODYN, V9, P189, DOI 10.1016/j.pdpdt.2012.03.002
Li JJ, 2016, PLOS ONE, V11, DOI 10.1371/journal.pone.0149066
Lu Y, 2015, CLIN RES HEPATOL GAS, V39, P718, DOI 10.1016/j.clinre.2014.10.015
Lyu YX, 2021, DIGEST LIVER DIS, V53, P1247, DOI 10.1016/j.dld.2021.03.029
Meybodi MA, 2020, ENDOSC INT OPEN, V8, pE281, DOI 10.1055/a-1067-4326
Minaga K, 2019, DIGEST ENDOSC, V31, P575, DOI 10.1111/den.13406
Mohan BP, 2022, J CLIN GASTROENTEROL, V56, pE153, DOI 10.1097/MCG.0000000000001524
Mohan BP, 2021, J CLIN GASTROENTEROL, V55, P271, DOI 10.1097/MCG.0000000000001377
Moole H, 2017, WORLD J GASTROENTERO, V23, P1278, DOI 10.3748/wjg.v23.i7.1278
Moole H, 2016, CAN J GASTROENTEROL, V2016, DOI 10.1155/2016/4726078
Mukai S, 2019, ENDOSC ULTRASOUND, V8, pS7, DOI 10.4103/eus.eus_46_19
Mukai S, 2017, J HEPATO-BIL-PAN SCI, V24, P537, DOI 10.1002/jhbp.496
Naitoh I, 2009, J GASTROEN HEPATOL, V24, P552, DOI 10.1111/j.1440-1746.2008.05750.x
Nakai Y, 2020, J GASTROEN HEPATOL, V35, P967, DOI 10.1111/jgh.14955
Nakai Y, 2019, ENDOSC ULTRASOUND, V8, P111, DOI 10.4103/eus.eus_17_18
Nunes N, 2021, GE PORT J GASTROENT, V28, P179, DOI 10.1159/000510026
Ogura T, 2016, ENDOSCOPY, V48, P156, DOI 10.1055/s-0034-1392859
Oh D, 2017, THER ADV GASTROENTER, V10, P42, DOI 10.1177/1756283X16671671
On W, 2022, GASTROINTEST ENDOSC, V95, P432, DOI 10.1016/j.gie.2021.09.040
Ortner MEJ, 2003, GASTROENTEROLOGY, V125, P1355, DOI 10.1016/j.gastro.2003.07.015
Paik WH, 2009, GASTROINTEST ENDOSC, V69, P55, DOI 10.1016/j.gie.2008.04.005
Park DH, 2015, J GASTROEN HEPATOL, V30, P1461, DOI 10.1111/jgh.13027
Park DH, 2014, EUR J CANCER, V50, P1259, DOI 10.1016/j.ejca.2014.01.008
Park SJ, 2013, GASTROINTEST ENDOSC, V78, P374, DOI 10.1016/j.gie.2013.04.183
Patel J, 2015, BEST PRACT RES CL GA, V29, P309, DOI 10.1016/j.bpg.2015.02.008
Pawa R, 2021, WORLD J GASTRO ENDOS, V13, P302, DOI 10.4253/wjge.v13.i8.302
Perez-Miranda M, 2021, ENDOSCOPY, V53, P63, DOI 10.1055/a-1296-8207
Poincloux L, 2015, ENDOSCOPY, V47, P794, DOI [10.1055/s-0035-1545399, 10.1055/s-0034-1391988]
Prachayakul V, 2013, WORLD J GASTROENTERO, V19, P4758, DOI 10.3748/wjg.v19.i29.4758
Prat F, 1998, GASTROINTEST ENDOSC, V47, P1, DOI 10.1016/S0016-5107(98)70291-3
Rerknimitr R, 2013, J GASTROEN HEPATOL, V28, P593, DOI 10.1111/jgh.12128
Sakai Y, 2021, SCAND J GASTROENTERO, V56, P1229, DOI 10.1080/00365521.2021.1938207
Sanders DJ, 2021, WORLD J GASTRO ENDOS, V13, P260, DOI 10.4253/wjge.v13.i8.260
Sawas T, 2022, ENDOSC ULTRASOUND, V11, P223, DOI 10.4103/EUS-D-21-00031
Schmidt A, 2016, UNITED EUR GASTROENT, V4, P570, DOI 10.1177/2050640615621235
Schmidt A, 2015, ENDOSCOPY, V47, P430, DOI 10.1055/s-0034-1391232

Sharaiha RZ, 2017, GASTROINTEST ENDOSC, V85, P904, DOI 10.1016/j.gie.2016.12.023
Sharaiha RZ, 2014, DIGEST DIS SCI, V59, P3099, DOI 10.1007/s10620-014-3264-6
Sofi AA, 2018, GASTROINTEST ENDOSC, V87, P944, DOI 10.1016/j.gie.2017.10.029
Staub J, 2020, ANN GASTROENTEROL, V33, P202, DOI 10.20524/aog.2020.0451
Steel AW, 2011, GASTROINTEST ENDOSC, V73, P149, DOI 10.1016/j.gie.2010.09.031
Strand DS, 2014, GASTROINTEST ENDOSC, V80, P794, DOI 10.1016/j.gie.2014.02.1030
Takahashi E, 2015, WORLD J GASTROENTERO, V21, P4946, DOI 10.3748/wjg.v21.i16.4946
Talreja JP, 2013, CLIN ENDOSC, V46, P390, DOI 10.5946/ce.2013.46.4.390
Talreja JP, 2010, GUT LIVER, V4, pS62, DOI 10.5009/gnl.2010.4.S1.S62
Telford JJ, 2010, GASTROINTEST ENDOSC, V72, P907, DOI 10.1016/j.gie.2010.08.021
Teoh AYB, 2018, GUT, V67, P1209, DOI 10.1136/gutjnl-2017-314341
Tringali A, 2018, ENDOSCOPY, V50, P631, DOI 10.1055/s-0043-125062
Tyberg A, 2022, ENDOSC ULTRASOUND, V11, P38, DOI 10.4103/EUS-D-21-00006
Tyberg A, 2020, ENDOSC ULTRASOUND, V9, P392, DOI 10.4103/eus.eus_42_20
Tyberg A, 2016, GASTROINTEST ENDOSC, V84, P941, DOI 10.1016/j.gie.2016.05.035
Uemura RS, 2018, J CLIN GASTROENTEROL, V52, P123, DOI 10.1097/MCG.0000000000000948
van Wanrooij RLJ, 2022, ENDOSCOPY, V54, P310, DOI 10.1055/a-1738-6780
Vienne A, 2010, GASTROINTEST ENDOSC, V72, P728, DOI 10.1016/j.gie.2010.06.040
Viesca MFY, 2019, CLIN EXP GASTROENTER, V12, P415, DOI 10.2147/CEG.S195714
Wang CC, 2020, MEDICINA-LITHUANIA, V56, DOI 10.3390/medicina56030114
Wang L, 2019, WORLD J SURG ONCOL, V17, DOI 10.1186/s12957-019-1656-y
Weismüller TJ, 2021, VISC MED, V37, P39, DOI 10.1159/000513970
Wentrup R, 2016, GUT LIVER, V10, P470, DOI 10.5009/gnl15175
Williams EJ, 2012, ENDOSCOPY, V44, P674, DOI 10.1055/s-0032-1309345
Yamao K, 2018, GASTROINTEST ENDOSC, V88, P66, DOI 10.1016/j.gie.2018.01.021
Yang JF, 2020, GASTROINTEST ENDOSC, V92, P1204, DOI 10.1016/j.gie.2020.04.075
Yang JF, 2018, ENDOSCOPY, V50, P751, DOI 10.1055/s-0043-124870
Yang MJ, 2015, SCAND J GASTROENTERO, V50, P1490, DOI 10.3109/00365521.2015.1057219
Zheng X, 2016, J DIGEST DIS, V17, P716, DOI 10.1111/1751-2980.12429
Zoepf T, 2005, AM J GASTROENTEROL, V100, P2426, DOI 10.1111/j.1572-0241.2005.00318.x
NR 143
TC 10
Z9 13
U1 0
U2 2
PU BAISHIDENG PUBLISHING GROUP INC
PI PLEASANTON
PA 7041 Koll Center Parkway, Suite 160, PLEASANTON, CA, UNITED STATES
SN 1948-5190
J9 WORLD J GASTRO ENDOS
JI World J. Gastrointest. Endosc.
PD OCT 16

PY 2022
VL 14
IS 10
BP 581
EP 596
DI 10.4253/wjge.v14.i10.581
PG 16
WC Gastroenterology & Hepatology
WE Emerging Sources Citation Index (ESCI)
SC Gastroenterology & Hepatology
GA 5U4OJ
UT WOS:000876527900001
PM 36303806
OA Green Accepted, gold
DA 2026-05-09
ER

PT J
AU Súbtil, JC
Betes, M
Muñoz-Navas, M
AF Carlos Subtil, Jose
Betes, Maite
Munoz-Navas, Miguel
TI Gallbladder drainage guided by endoscopic ultrasound
SO WORLD JOURNAL OF GASTROINTESTINAL ENDOSCOPY
LA English
DT Article
DE Gallbladder drainage; Endoscopic ultrasound-guided;
Cholecystenterostomy; Cholecystogastrostomy; Cholecystoduodenostomy;
Percutaneous cholecystostomy; Endoscopic transpapillary gallbladder
drainage; Acute cholecystitis
AB The gold-standard management of acute cholecystitis is cholecystectomy. Surgical intervention may be contraindicated due to permanent causes. To date, the classical approach is percutaneous cholecystostomy in patients unresponsive to medical therapy. However, with this treatment some patients may experience discomfort, complications and a decrease in their quality of life. In these cases, endoscopic ultrasound (EUS)-guided gallbladder drainage may represent an effective minimally invasive alternative. Our objective is to describe in detail this new and not well-known technique: EUS-guided cho-lecystenterostomy. We will describe how the patient should be prepared, what accessories are needed and how the technique is performed. We will also discuss the possible indications for this technique and will pro-vide a brief review based on published reports and our own experience. (C) 2010 Baishideng. All rights reserved.
C1 [Carlos Subtil, Jose; Betes, Maite; Munoz-Navas, Miguel] Univ Clin Navarra, Gastroenterol Dept, C Pio 12 36, Pamplona 31008, Spain.

C3 University of Navarra
RP Súbtil, JC (corresponding author), Univ Clin Navarra, Gastroenterol Dept, C Pio 12 36, Pamplona 31008, Spain.
EM jcsubtil@unav.es
RI Betés, Maite/O-2809-2015
CR Bakkaloglu H, 2006, WORLD J GASTROENTERO, V12, P7179, DOI 10.3748/wjg.v12.i44.7179
Baron TH, 2007, GASTROINTEST ENDOSC, V65, P735, DOI 10.1016/j.gie.2006.07.041
Bories E, 2007, ENDOSCOPY, V39, P287, DOI 10.1055/s-2007-966212
François E, 2002, GASTROINTEST ENDOSC, V56, P128, DOI 10.1067/mge.2002.125547
Hamy A, 1997, SURGERY, V121, P398, DOI 10.1016/S0039-6060(97)90309-3
Ito K, 2004, AM J ROENTGENOL, V183, P193, DOI 10.2214/ajr.183.1.1830193
Kwan V, 2007, GASTROINTEST ENDOSC, V66, P582, DOI 10.1016/j.gie.2007.02.065
Lee SS, 2007, GASTROINTEST ENDOSC, V66, P1008, DOI 10.1016/j.gie.2007.03.1080
Lopes CV, 2007, SCAND J GASTROENTERO, V42, P524, DOI 10.1080/00365520601065093
Savides TJ, 2009, GASTROINTEST ENDOSC, V69, pS3, DOI 10.1016/j.gie.2008.10.060
Seewald S, 2005, GASTROINTEST ENDOSC, V61, P495, DOI 10.1016/S0016-5107(04)02848-2
Seewald S, 2009, ENDOSCOPY, V41, P166, DOI 10.1055/s-0028-1119501
Spira RM, 2002, AM J SURG, V183, P62, DOI 10.1016/S0002-9610(01)00849-2
Toyota N, 2006, J HEPATO-BILIARY-PAN, V13, P80, DOI 10.1007/s00534-005-1062-4
vanOverhagen H, 1996, CARDIOVASC INTER RAD, V19, P72
Varadarajulu S, 2007, GASTROINTEST ENDOSC, V66, P372, DOI 10.1016/j.gie.2007.02.054
Wehrmann T, 2005, GASTROINTEST ENDOSC, V62, P344, DOI 10.1016/j.gie.2005.03.001
NR 17
TC 26
Z9 34
U1 0
U2 1
PU BAISHIDENG PUBLISHING GROUP INC
PI PLEASANTON
PA 8226 REGENCY DR, PLEASANTON, CA 94588 USA
SN 1948-5190
J9 WORLD J GASTRO ENDOS
JI World J. Gastrointest. Endosc.
PD JUN 16
PY 2010
VL 2
IS 6
BP 203
EP 209
DI 10.4253/wjge.v2.i6.203
PG 7
WC Gastroenterology & Hepatology
WE Emerging Sources Citation Index (ESCI)

SC Gastroenterology & Hepatology
GA V5B9I
UT WOS:000219467700003
PM 21160934
OA Green Submitted, Bronze
DA 2026-05-09
ER

PT J
AU Mangiavillano, B
Moon, JH
Facciorusso, A
Vargas-Madrigal, J
Di Matteo, F
Rizzatti, G
De Luca, L
Forti, E
Mutignani, M
Al-Lehibi, A
Paduano, D
Bulajic, M
Decembrino, F
Auriemma, F
Franchellucci, G
De Marco, A
Gentile, C
Shin, I
Rea, R
Massidda, M
Calabrese, F
Mirante, VG
Ofosu, A
Crinò, SF
Hassan, C
Repici, A
Larghi, A
AF Mangiavillano, Benedetto
Moon, Jong Ho
Facciorusso, Antonio
Vargas-Madrigal, Jorge
Di Matteo, Francesco
Rizzatti, Gianenrico

De Luca, Luca
Forti, Edoardo
Mutignani, Massimiliano
Al-Lehibi, Abed
Paduano, Danilo
Bulajic, Milutin
Decembrino, Francesco
Auriemma, Francesco
Franchellucci, Gianluca
De Marco, Alessandro
Gentile, Carmine
Shin, Il Sang
Rea, Roberta
Massidda, Marco
Calabrese, Federica
Mirante, Vincenzo Giorgio
Ofosu, Andrew
Crino, Stefano Francesco
Hassan, Cesare
Repici, Alessandro
Larghi, Alberto
TI Endoscopic ultrasound-guided gallbladder drainage as a first approach for jaundice palliation in unresectable malignant distal biliary obstruction: Prospective study
SO DIGESTIVE ENDOSCOPY
LA English
DT Article
DE drainage; EUS-GBD; EC-LAMS; Hot-Spaxus; malignant biliary obstruction (MBO); interventional EUS
ID APPOSING METAL STENT; RESCUE TREATMENT; ADVERSE EVENTS
AB Objectives: Endoscopic retrograde cholangiopancreatography (ERCP) represents the gold standard for jaundice palliation in patients with distal malignant biliary obstruction (DMBO). Biliary drainage using electrocautery lumen apposing metal stent (EC-LAMS) is currently a well-established procedure when ERCP fails. In a palliative setting the endoscopic ultrasoundguided gallbladder drainage (EUS-GBD) could represent an easy and valid option. We performed a prospective study with a new EC-LAMS with the primary aim to assess the clinical success rate of EUS-GBD as a first-line approach to the palliation of DMBO.

Methods: In all, 37 consecutive patients undergoing EUS-GBD with a new EC-LAMS were prospectively enrolled. Clinical success was defined as bilirubin level decrease >15% within 24 h and >50% within 14 days after EC-LAMS placement.

Results: The mean age was 73.5 +/- 10.8 years; there were 17 male patients (45.9%). EC-LAMS placement was technically feasible in all patients (100%) and the clinical success rate was 100%. Four patients (10.8%) experienced adverse events, one bleeding, one food impaction, and two cystic duct obstructions because of disease progression. No

stent-related deaths were observed. The mean hospitalization was 7.7 +/- 3.4 days. Median overall survival was 4 months (95% confidence interval 1-8).

Conclusion: Endoscopic ultrasound-guided gallbladder drainage with the new EC-LAMS is a valid option in palliative endoscopic biliary drainage as a first-step approach in low survival patients with malignant jaundice unfit for surgery. A smaller diameter EC-LAMS should be preferred, particularly if the drainage is performed through the stomach, to avoid potential food impaction, which could result in stent dysfunction.
C1 [Mangiavillano, Benedetto; Paduano, Danilo; Auriemma, Francesco; Franchellucci, Gianluca; De Marco, Alessandro; Gentile, Carmine; Calabrese, Federica] Human Mater Domini, Gastrointestinal Endoscopy Unit, Varese, Italy.

[Mangiavillano, Benedetto; Hassan, Cesare; Repici, Alessandro] Humanitas Univ, Dept Biomed Sci, Milan, Italy.

[De Luca, Luca] ASST St Paolo & Carlo, Endoscop Unit, Milan, Italy.

[Forti, Edoardo; Mutignani, Massimiliano] Osped Niguarda Ca Granda, Digest & Intervent Endoscopy Unit, ASST Niguarda, Milan, Italy.

[Franchellucci, Gianluca; De Marco, Alessandro; Hassan, Cesare; Repici, Alessandro] IRCCS, Human Clin & Res Ctr, Milan, Italy.

[Facciorusso, Antonio] Univ Foggia, Dept Med & Surg Sci, Gastroenterol Unit, Foggia, Italy.

[Di Matteo, Francesco] Campus Bio Med Univ, Digest Endoscopy, Rome, Italy.

[Rizzatti, Gianenrico; Rea, Roberta; Larghi, Alberto] Fdn Policlin Univ A Gemelli IRCCS, Digest Endoscopy Unit, Rome, Italy.

[Bulajic, Milutin] Fatebenefratelli Isola Tiberina Gemelli Isola, Digest Endoscopy, Rome, Italy.

[Di Matteo, Francesco] Ente Ecclesiast Osped Gen Regionale F Miulli, UOC Gastroenterol Endoscopia Digest, Bari, Italy.

[Massidda, Marco] Mater Olbia Hosp, Digest Endoscopy, Olbia, Italy.

[Mirante, Vincenzo Giorgio] Azienda USL, Gastroenterol & Digest Endoscopy Unit, IRCCS Reggio Emilia, Reggio Emilia, Italy.

[Crino, Stefano Francesco] GB Rossi Univ Hosp, Pancreas Inst, Gastroenterol & Digest Endoscopy Unit, Verona, Italy.

[Moon, Jong Ho] SoonChunHyang Univ, Digest Dis Ctr, Dept Internal Med, Sch Med, Bucheon, South Korea.

[Moon, Jong Ho] SoonChunHyang Univ, Res Inst, Dept Internal Med, Sch Med, Bucheon, South Korea.

[Vargas-Madrigal, Jorge] Hosp Enr Baltodano Briceno, Dept Gastroenterol & Endoscopy, Liberia, Costa Rica.

[Al-Lehibi, Abed] King Saud bin Abdulaziz Univ Hlth Sci, Fac Med, King Fahad Med City, Riyadh, Saudi Arabia.

[Ofosu, Andrew] Univ Cincinnati, Div Digest Dis, Cincinnati, OH USA.

C3 Humanitas University; Ospedale Niguarda Ca' Granda; IRCCS Ca Granda Ospedale Maggiore Policlinico; University of Foggia; University Campus Bio-Medico - Rome Italy; Catholic University of the Sacred Heart; IRCCS Policlinico Gemelli; University of Verona; Azienda Ospedaliera Universitaria Integrata Verona; Soonchunhyang University; Soonchunhyang University; King Saud Bin Abdulaziz University for Health Sciences; University System of Ohio; University of Cincinnati

RP Mangiavillano, B (corresponding author), Human Mater Domini, Gastrointestinal Endoscopy Unit, Via Gerenzano N2, I-21053 Varese, Italy.

EM bennymangiavillano@gmail.com

RI ; Shin, Il Sang/HTL-6962-2023; Mirante, Vincenzo Giorgio/AAU-9045-2020; Repici, Alessandro/HFH-8162-2022; Vargas Madrigal, Jorge/LQJ-0413-2024;

hassan, cesare/H-2844-2012; Gentile, Carmine/G-6446-2017; Larghi, Alberto/AAC-7448-2022; Rizzatti, Gianenrico/B-1406-2019; Di Matteo, Francesco Maria/AAC-1076-2022; Bulajic, Milutin/GSE-3379-2022; Facciorusso, Antonio/AAA-5966-2019
OI Mangiavillano, Benedetto/0000-0003-0611-7448; Shin, Il Sang/0000-0003-2199-1942; Mirante, Vincenzo Giorgio/0000-0002-2924-4717; Repici, Alessandro/0000-0002-1621-6450; Vargas Madrigal, Jorge/0009-0007-9623-1389; hassan, cesare/0000-0001-7167-1459; Massidda, Marco/0000-0003-4558-1980;
CR Bang JY, 2019, DIGEST ENDOSC, V31, P619, DOI 10.1111/den.13428
Cotton PB, 2010, GASTROINTEST ENDOSC, V71, P446, DOI 10.1016/j.gie.2009.10.027
Dumonceau JM, 2018, ENDOSCOPY, V50, P910, DOI 10.1055/a-0659-9864
Enochsson L, 2010, GASTROINTEST ENDOSC, V72, P1175, DOI 10.1016/j.gie.2010.07.047
Facciorusso A, 2022, CANCERS, V14, DOI 10.3390/cancers14133291
Giovannini M, 2001, ENDOSCOPY, V33, P898, DOI 10.1055/s-2001-17324
Imai H, 2016, GASTROINTEST ENDOSC, V84, P147, DOI 10.1016/j.gie.2015.12.024
Issa D, 2021, ENDOSCOPY, V53, P827, DOI 10.1055/a-1259-0349
Mangiavillano B, 2022, DIGEST DIS, V40, P526, DOI 10.1159/000518539
Mangiavillano B, 2022, ENDOSC INT OPEN, V10, pE998, DOI 10.1055/a-1838-2683
Mangiavillano B, 2022, GASTROINTEST ENDOSC, V95, P115, DOI 10.1016/j.gie.2021.07.021
Mangiavillano B, 2020, EUR J GASTROEN HEPAT, V32, P1279, DOI 10.1097/MEG.0000000000001752
Minaga K, 2020, WORLD J GASTROENTERO, V26, P947, DOI 10.3748/wjg.v26.i9.947
Nakai Y, 2016, GASTROINTEST ENDOSC, V83, P1302, DOI 10.1016/j.gie.2016.01.015
Nass KJ, 2022, GASTROINTEST ENDOSC, V95, P1078, DOI 10.1016/j.gie.2021.11.038
Paduano D, 2023, CANCERS, V15, DOI 10.3390/cancers15020490
Podboy A, 2021, GASTROINTEST ENDOSC, V93, P797, DOI 10.1016/j.gie.2020.09.040
Teoh AYB, 2023, GASTROENTEROLOGY, V165, P473, DOI 10.1053/j.gastro.2023.04.016
van der Merwe SW, 2022, ENDOSCOPY, V54, P185, DOI 10.1055/a-1717-1391
VOEGELI DR, 1985, AM J SURG, V150, P243, DOI 10.1016/0002-9610(85)90129-1
Winick A B, 2001, Tech Vasc Interv Radiol, V4, P200, DOI 10.1016/S1089-2516(01)90026-5
NR 21
TC 25
Z9 32
U1 0
U2 3
PU WILEY
PI HOBOKEN
PA 111 RIVER ST, HOBOKEN 07030-5774, NJ USA
SN 0915-5635
EI 1443-1661
J9 DIGEST ENDOSC
JI Dig. Endosc.

PD MAR
PY 2024
VL 36
IS 3
BP 351
EP 358
DI 10.1111/den.14606
EA JUL 2023
PG 8
WC Gastroenterology & Hepatology; Surgery
WE Science Citation Index Expanded (SCI-EXPANDED)
SC Gastroenterology & Hepatology; Surgery
GA KJ8O0
UT WOS:001020878000001
PM 37253185
OA Bronze
DA 2026-05-09
ER

PT J
AU Artifon, ELA
Perez-Miranda, M
AF Artifon, Everson L. A.
Perez-Miranda, Manuel
TI EUS-guided choledochoduodenostomy for malignant distal biliary
obstruction palliation: an article review
SO ENDOSCOPIC ULTRASOUND
LA English
DT Review
DE biliary drainage; endoscopic ultrasound-guided; choledocho
ID ENDOSCOPIC ULTRASOUND; DRAINAGE; CHOLANGIOGRAPHY; PLACEMENT
AB The BUS-guided biliary drainage is a new tool for the palliation of distal obstructive biliary lesions. The EUS-guided access, which creates a fistulization between the duodenal bulb and distal common biliary duct, is an effective method to relieve jaundice and has low morbidity and mortality, in patients with distal biliary obstruction (pancreatic mass or papillary cancer). This technique is called choledochoduodenostomy and is presented promptly in this article. The BUS-guided biliary drainage should be made within protocol conditions and done by very experienced endosonographers.
C1 [Artifon, Everson L. A.; Perez-Miranda, Manuel] Univ Sao Paulo, Sao Paulo, Brazil.
C3 Universidade de Sao Paulo
RP Artifon, ELA (corresponding author), Univ Sao Paulo, Rua Guimaraes Passos 260-121, Sao Paulo, Brazil.
EM eartifon@hotmail.com
CR Ang TL, 2007, J PANCREAS, V8, P438
Brauer BC, 2009, GASTROINTEST ENDOSC, V70, P471, DOI 10.1016/j.gie.2008.12.233

Burmester E, 2003, GASTROINTEST ENDOSC, V57, P246, DOI 10.1067/mge.2003.85
Fogel EL, 2001, ENDOSCOPY, V33, P31, DOI 10.1055/s-2001-11186
Fujita N, 2007, WORLD J GASTROENTERO, V13, P5512, DOI 10.3748/wjg.v13.i41.5512
Giovannini M, 2001, ENDOSCOPY, V33, P898, DOI 10.1055/s-2001-17324
Gupta K, 2007, REV GASTROENTEROL DI, V7, P22
Hanada K, 2009, DIGEST ENDOSC, V21, pS75, DOI 10.1111/j.1443-1661.2009.00855.x
Horaguchi J, 2009, DIGEST ENDOSC, V21, P239, DOI 10.1111/j.1443-1661.2009.00899.x
Itoi T, 2008, WORLD J GASTROENTERO, V14, P6078, DOI 10.3748/wjg.14.6078
Itoi T, 2010, J HEPATO-BIL-PAN SCI, V17, P611, DOI 10.1007/s00534-009-0196-1
Itoi T, 2009, GASTROINTEST ENDOSC, V69, pS8, DOI 10.1016/j.gie.2008.11.003
Iwamuro M, 2010, DIGEST ENDOSC, V22, P236, DOI 10.1111/j.1443-1661.2010.00997.x
Kahaleh M, 2006, GASTROINTEST ENDOSC, V64, P52, DOI 10.1016/j.gie.2006.01.063
Kim YS, 2010, ENDOSCOPY, V42, P496, DOI 10.1055/s-0029-1244082
LAI ECS, 1992, NEW ENGL J MED, V326, P1582, DOI 10.1056/NEJM199206113262401
Maranki J, 2009, ENDOSCOPY, V41, P532, DOI 10.1055/s-0029-1214712
Nguyen-Tang T, 2010, ENDOSCOPY, V42, P232, DOI 10.1055/s-0029-1243858
Park DH, 2009, AM J GASTROENTEROL, V104, P2168, DOI 10.1038/ajg.2009.254
Perez-Miranda M, 2010, WORLD J GASTRO ENDOS, V2, P212, DOI 10.4253/wjge.v2.i6.212
Püspök A, 2005, AM J GASTROENTEROL, V100, P1743, DOI 10.1111/j.1572-0241.2005.41806.x
SMITH AC, 1994, LANCET, V344, P1655, DOI 10.1016/S0140-6736(94)90455-3
Talreja JP, 2008, GASTROINTEST ENDOSC, V68, P1199, DOI 10.1016/j.gie.2008.06.015
Tarantino I, 2008, ENDOSCOPY, V40, P336, DOI 10.1055/s-2007-995455
Wiersema MJ, 1996, GASTROINTEST ENDOSC, V43, P102, DOI 10.1016/S0016-5107(06)80108-2
Wilson JA, 2010, GASTROINTEST ENDOSC, V72, P947, DOI 10.1016/j.gie.2010.07.016
Yamao K, 2008, ENDOSCOPY, V40, P340, DOI 10.1055/s-2007-995485
Yamao K, 2006, GASTROINTEST ENDOSC, V64, P663, DOI 10.1016/j.gie.2006.07.003
NR 28
TC 7
Z9 9
U1 0
U2 2
PU WOLTERS KLUWER MEDKNOW PUBLICATIONS
PI MUMBAI
PA WOLTERS KLUWER INDIA PVT LTD , A-202, 2ND FLR, QUBE, C T S NO 1498A-2
VILLAGE MAROL, ANDHERI EAST, MUMBAI, 400059, INDIA
SN 2303-9027
EI 2226-7190
J9 ENDOSC ULTRASOUND
JI Endosc. Ultrasound
PD MAY
PY 2012
VL 1

IS 1
BP 2
EP 7
DI 10.7178/eus.01.002
PG 6
WC Gastroenterology & Hepatology
WE Science Citation Index Expanded (SCI-EXPANDED)
SC Gastroenterology & Hepatology
GA V31PF
UT WOS:000208894800002
PM 24949329
OA hybrid
DA 2026-05-09
ER

PT J
AU Minaga, K
Takenaka, M
Yamao, K
Kamata, K
Omoto, S
Nakai, A
Yamazaki, T
Okamoto, A
Ishikawa, R
Yoshikawa, T
Chiba, Y
Watanabe, T
Kudo, M
AF Minaga, Kosuke
Takenaka, Mamoru
Yamao, Kentaro
Kamata, Ken
Omoto, Shunsuke
Nakai, Atsushi
Yamazaki, Tomohiro
Okamoto, Ayana
Ishikawa, Rei
Yoshikawa, Tomoe
Chiba, Yasutaka
Watanabe, Tomohiro
Kudo, Masatoshi

TI Clinical utility of treatment method conversion during single-session endoscopic ultrasound-guided biliary drainage

SO WORLD JOURNAL OF GASTROENTEROLOGY
LA English
DT Article
DE Endoscopic ultrasound; Endoscopic ultrasound-guided biliary drainage; Interventional endoscopic ultrasound; Biliary drainage; Biliary obstruction
ID RETROGRADE CHOLANGIOPANCREATOGRAPHY; ADVERSE EVENTS; FAILED ERCP; GUIDELINES; CHOLEDOCHODUODENOSTOMY; ULTRASONOGRAPHY; COMPLICATIONS; MULTICENTER; CHOLANGITIS; ALGORITHM

AB BACKGROUND

Although several techniques for endoscopic ultrasound-guided biliary drainage (EUS-BD) are available at present, an optimal treatment algorithm of EUS-BD has not yet been established.

AIM

To evaluate the clinical utility of treatment method conversion during single endoscopic sessions for difficult cases in initially planned EUS-BD.

METHODS

This was a single-center retrospective analysis using a prospectively accumulated database. Patients with biliary obstruction undergoing EUS-BD between May 2008 and April 2016 were included. The primary outcome was to evaluate the improvement in EUS-BD success rates by converting the treatment methods during a single endoscopic session. Secondary outcomes were clarification of the factors leading to the conversion from the initial EUS-BD and the assessment of efficacy and safety of the conversion as judged by technical success, clinical success, and adverse events (AEs).

RESULTS

A total of 208 patients underwent EUS-BD during the study period. For 18.8% (39/208) of the patients, the treatment methods were converted to another EUS-BD technique from the initial plan. Biliary obstruction was caused by pancreatobiliary malignancies, other malignant lesions, biliary stones, and other benign lesions in 22, 11, 4, and 2 patients, respectively. The reasons for the difficulty with the initial EUS-BD were classified into the following 3 procedures: Target puncture (n = 13), guidewire manipulation (n = 18), and puncture tract dilation (n = 8). Technical success was achieved in 97.4% (38/39) of the cases and clinical success was achieved in 89.5% of patients (34/38). AEs occurred in 10.3% of patients, including bile leakage (n = 2), bleeding (n = 1), and cholecystitis (n = 1). The puncture target and drainage technique were altered in subsequent EUS-BD procedures in 25 and 14 patients, respectively. The final technical success rate with 95%CI for all 208 cases was 97.1% (95%CI: 93.8%-98.9%), while that of the initially planned EUS-BD was 78.8% (95%CI: 72.6%-84.2%).

CONCLUSION

Among multi-step procedures in EUS-BD, guidewire manipulation appeared to be the most technically challenging. When initially planned EUS-BD is technically difficult, treatment method conversion in a single endoscopic session may result in successful EUS-BD without leading to severe AEs.

C1 [Minaga, Kosuke; Takenaka, Mamoru; Yamao, Kentaro; Kamata, Ken; Omoto, Shunsuke; Nakai, Atsushi; Yamazaki, Tomohiro; Okamoto, Ayana; Ishikawa, Rei; Yoshikawa, Tomoe; Watanabe, Tomohiro; Kudo, Masatoshi] Kindai Univ, Fac Med, Dept Gastroenterol & Hepatol, 377-2 Ohno Higashi, Osaka 5898511, Japan.

[Chiba, Yasutaka] Kindai Univ Hosp, Clin Res Ctr, Osaka 5898511, Japan.

C3 Kindai University (Kinki University)
RP Minaga, K (corresponding author), Kindai Univ, Fac Med, Dept Gastroenterol & Hepatol, 377-2 Ohno Higashi, Osaka 5898511, Japan.
EM kousukeminaga@med.kindai.ac.jp
RI Kudo, Masatoshi/AAA-9744-2019; Watanabe, Tomohiro/ABA-4712-2021; Minaga, Kosuke/AAD-2107-2020
OI Watanabe, Tomohiro/0000-0002-6175-8064;
CR Alvarez-Sanchez MV, 2014, SURG ENDOSC, V28, P712, DOI 10.1007/s00464-013-3260-5
Amano M, 2017, J GASTROEN HEPATOL, V32, P716, DOI 10.1111/jgh.13489
Anderloni A, 2019, GASTROINTEST ENDOSC, V89, P69, DOI [10.1016/j.gie.2018.04.1746, 10.1016/j.gie.2018.08.047]
Coté GA, 2012, CLIN GASTROENTEROL H, V10, P920, DOI 10.1016/j.cgh.2012.02.019
Cotton PB, 2010, GASTROINTEST ENDOSC, V71, P446, DOI 10.1016/j.gie.2009.10.027
Dhir V, 2014, DIGEST ENDOSC, V26, P430, DOI 10.1111/den.12153
Dumonceau JM, 2018, ENDOSCOPY, V50, P910, DOI 10.1055/a-0659-9864
El Chafic AH, 2019, DIGEST DIS SCI, V64, P3321, DOI 10.1007/s10620-019-05688-2
Giovannini M, 2001, ENDOSCOPY, V33, P898, DOI 10.1055/s-2001-17324
GUNTHER RW, 1988, CARDIOVASC INTER RAD, V11, P65, DOI 10.1007/BF02577061
Hathorn KE, 2019, ENDOSC INT OPEN, V7, pE1432, DOI 10.1055/a-0990-9488
Hedjoudje A, 2019, UNITED EUR GASTROENT, V7, P60, DOI 10.1177/2050640618808147
Imai H, 2016, GASTROINTEST ENDOSC, V84, P147, DOI 10.1016/j.gie.2015.12.024
Isayama H, 2019, J HEPATO-BIL-PAN SCI, V26, P249, DOI 10.1002/jhbp.631
Itoi T, 2011, J HEPATO-BIL-PAN SCI, V18, P664, DOI 10.1007/s00534-011-0410-9
Iwashita T, 2016, GASTROINTEST ENDOSC, V83, P394, DOI 10.1016/j.gie.2015.04.043
Iwashita T, 2014, CLIN J GASTROENTEROL, V7, P94, DOI 10.1007/s12328-014-0467-5
Kanno Y, 2017, WORLD J GASTRO ENDOS, V9, P304, DOI 10.4253/wjge.v9.i7.304
Khan MA, 2016, DIGEST DIS SCI, V61, P684, DOI 10.1007/s10620-015-3933-0
Kunda R, 2016, SURG ENDOSC, V30, P5002, DOI 10.1007/s00464-016-4845-6
Logiudice FP, 2019, WORLD J GASTRO ENDOS, V11, P281, DOI 10.4253/wjge.v11.i4.281
Minaga K, 2019, DIGEST ENDOSC, V31, P575, DOI 10.1111/den.13406
Minaga K, 2018, DIGEST ENDOSC, V30, P38, DOI 10.1111/den.12910
Minaga K, 2016, WORLD J GASTROENTERO, V22, P4264, DOI 10.3748/wjg.v22.i16.4264
Mukai S, 2017, J HEPATO-BIL-PAN SCI, V24, P537, DOI 10.1002/jhbp.496
Nennstiel S, 2015, J CLIN GASTROENTEROL, V49, P764, DOI 10.1097/MCG.0000000000000275
Ogura T, 2019, ENDOSC INT OPEN, V7, pE1652, DOI 10.1055/a-0961-7890
Oh HC, 2007, ENDOSCOPY, V39, P731, DOI 10.1055/s-2007-966577
Park DH, 2011, GASTROINTEST ENDOSC, V74, P1276, DOI 10.1016/j.gie.2011.07.054
Teoh AYB, 2018, GUT, V67, P1209, DOI 10.1136/gutjnl-2017-314341
Tsuchiya T, 2016, DIGEST ENDOSC, V28, P96, DOI 10.1111/den.12611
Tyberg A, 2016, GASTROINTEST ENDOSC, V84, P941, DOI 10.1016/j.gie.2016.05.035
Uemura RS, 2018, J CLIN GASTROENTEROL, V52, P123, DOI 10.1097/MCG.0000000000000948
Wang KX, 2016, GASTROINTEST ENDOSC, V83, P1218, DOI 10.1016/j.gie.2015.10.033

NR 34
TC 5
Z9 8
U1 0
U2 3
PU BAISHIDENG PUBLISHING GROUP INC
PI PLEASANTON
PA 7041 Koll Center Parkway, Suite 160, PLEASANTON, CA, UNITED STATES
SN 1007-9327
EI 2219-2840
J9 WORLD J GASTROENTERO
JI World J. Gastroenterol.
PD MAR 7
PY 2020
VL 26
IS 9
BP 947
EP 959
DI 10.3748/wjg.v26.i9.947
PG 13
WC Gastroenterology & Hepatology
WE Science Citation Index Expanded (SCI-EXPANDED)
SC Gastroenterology & Hepatology
GA LS2ZZ
UT WOS:000536258700005
PM 32206005
OA Green Accepted, hybrid
DA 2026-05-09
ER

PT J
AU Jacques, J
Privat, J
Pinard, F
Fumex, F
Valats, JC
Chaoui, A
Cholet, F
Godard, B
Grandval, P
Legros, R
Kerever, S

Napoleon, B
AF Jacques, Jeremie
Privat, Jocelyn
Pinard, Fabien
Fumex, Fabien
Valats, Jean-Christophe
Chaoui, Azzedine
Cholet, Franck
Godard, Bruno
Grandval, Philippe
Legros, Romain
Kerever, Sebastien
Napoleon, Bertrand
TI Endoscopic ultrasound-guided choledochoduodenostomy with electrocautery-enhanced lumen-apposing stents: a retrospective analysis
SO ENDOSCOPY
LA English
DT Article
ID BILIARY DRAINAGE; HEPATICOGASTROSTOMY; OBSTRUCTION; ERCP
AB Background Endoscopic ultrasound-guided biliary drainage is an alternative to percutaneous biliary drainage in cases of malignant biliary obstruction and failure of classic endoscopic drainage by endoscopic retrograde cholangio-pancreatography (ERCP). Recently, a new electrocautery-enhanced lumen-apposing metal stent (ECE-LAMS) that allows for endoscopic anastomosis (apposition stent) has become available for use in EUS-choledochoduodenostomy (EUS-CDS) and facilitates the procedure.

Methods This was a retrospective study of all EUS-CDS procedures performed in France between April 2016 and August 2017. The primary end point was the technical and clinical success rates of EUS-CDS using an ECE-LAMS.

Results 52 consecutive patients were included in the study. The etiology of distal bile duct obstruction was distal pancreatic adenocarcinoma in 43 patients (82.7%). The technical success rate was 88.5% (4652 patients), and the clinical success rate was 100% (46/46 patients). The mean duration of the procedure was 10.2 minutes (range 1-90). Two patients (3.8%) presented with short-term complications after EUS-CDS and before discharge from hospital. In univariate analyses, a small diameter of the common bile duct and not following the recommended procedure technique were significant risk factors for technical failure. Over a mean follow-up of 157 days, the median survival time without biliary complications was 135 days.

Conclusion EUS-CDS with an ECE-LAMS is efficacious and safe in distal malignant obstruction of the common bile duct and could be proposed as the first option in cases of ERCP failure.
C1 [Jacques, Jeremie; Legros, Romain] CHU Dupuytren, Serv Hepatogastroenterol, 2 Ave Martin Luther King, F-87042 Limoges, France.
[Privat, Jocelyn] CH Jacques Lacarin, Serv Hepatogastroenterol, Vichy, France.
[Pinard, Fabien] Hop Cournouaille, Serv Hepatogastroenterol, Quimper, France.
[Fumex, Fabien; Napoleon, Bertrand] Hop Prive Jean Mermoz, Serv Hepatogastroenterol, Lyon, France.
[Valats, Jean-Christophe] CHU St Eloi, Serv Hepatogastroenterol, Montpellier, France.
[Chaoui, Azzedine] CH Moulins, Serv Hepatogastroenterol, Moulins, France.

[Cholet, Franck] CHU Brest, Serv Hepatogastroenterol, Brest, France.
[Godard, Bruno] CHU Trousseau, Serv Hepatogastroenterol, Tours, France.
[Grandval, Philippe] Hop La Timone, AP HM, Serv Hepatogastroenterol, Marseille, France.
[Kerever, Sebastien] Hop St Louis, AP HP, Serv Biostat & Informat Med, Paris, France.
C3 CHU Limoges; Universite de Montpellier; CHU de Montpellier; Universite
de Bretagne Occidentale; CHU Brest; CHU Tours; Aix-Marseille Universite;
Assistance Publique-Hopitaux de Marseille; Assistance Publique Hopitaux
Paris (APHP); Universite Paris Cite; Hopital Universitaire Saint-Louis -
APHP
RP Jacques, J (corresponding author), CHU Dupuytren, Serv Hepatogastroenterol, 2 Ave Martin Luther King, F-87042 Limoges, France.
EM jeremiejacques@gmail.com
RI Kerever, Sébastien/S-1620-2019
OI Kerever, Sébastien/0000-0003-0764-4370
CR Cotton PB, 2010, GASTROINTEST ENDOSC, V71, P446, DOI 10.1016/j.gie.2009.10.027
Khan MA, 2016, DIGEST DIS SCI, V61, P684, DOI 10.1007/s10620-015-3933-0
Kunda R, 2016, SURG ENDOSC, V30, P5002, DOI 10.1007/s00464-016-4845-6
Park JK, 2018, GASTROINTEST ENDOSC, V88, P277, DOI 10.1016/j.gie.2018.03.015
Rimbas Mihai, 2017, Gastrointest Endosc Clin N Am, V27, P681, DOI [10.1016/j.giec.2017.06.006, 10.1016/j.giec.2017.06.006]
Sharaiha RZ, 2017, GASTROINTEST ENDOSC, V85, P904, DOI 10.1016/j.gie.2016.12.023
Sportes A, 2017, THER ADV GASTROENTER, V10, P483, DOI 10.1177/1756283X17702096
Téllez-Avila FI, 2018, SURG LAPARO ENDO PER, V28, P183, DOI 10.1097/SLE.0000000000000528
Tsuchiya T, 2018, GASTROINTEST ENDOSC, V87, P1138, DOI 10.1016/j.gie.2017.08.017
Tyberg A, 2016, GASTROINTEST ENDOSC, V84, P941, DOI 10.1016/j.gie.2016.05.035
Uemura RS, 2018, J CLIN GASTROENTEROL, V52, P123, DOI 10.1097/MCG.0000000000000948
van der Gaag NA, 2010, NEW ENGL J MED, V362, P129, DOI 10.1056/NEJMoa0903230
NR 12
TC 80
Z9 88
U1 0
U2 4
PU GEORG THIEME VERLAG KG
PI STUTTGART
PA RUDIGERSTR 14, D-70469 STUTTGART, GERMANY
SN 0013-726X
EI 1438-8812
J9 ENDOSCOPY
JI Endoscopy
PD JUN
PY 2019
VL 51

IS 6
BP 540
EP 547
DI 10.1055/a-0735-9137
PG 8
WC Gastroenterology & Hepatology; Surgery
WE Science Citation Index Expanded (SCI-EXPANDED)
SC Gastroenterology & Hepatology; Surgery
GA IC3UJ
UT WOS:000470887100024
PM 30347424
DA 2026-05-09
ER

PT J
AU Troncone, E
Fugazza, A
Cappello, A
Blanco, GD
Monteleone, G
Repici, A
Teoh, AYB
Anderloni, A
AF Troncone, Edoardo
Fugazza, Alessandro
Cappello, Annalisa
Blanco, Giovanna Del Vecchio
Monteleone, Giovanni
Repici, Alessandro
Teoh, Anthony Yuen Bun
Anderloni, Andrea
TI Malignant gastric outlet obstruction: Which is the best therapeutic
option?
SO WORLD JOURNAL OF GASTROENTEROLOGY
LA English
DT Review
DE Gastrojejunostomy; Self-expanding metal stent; Enteral stent;
Interventional endoscopic ultrasonography; Endoscopic ultrasound-guided
gastroenterostomy; Pancreatic cancer; Gastric cancer; Duodenal stricture
ID EXPANDABLE METAL STENTS; EUS-GUIDED GASTROENTEROSTOMY;
STOMACH-PARTITIONING GASTROJEJUNOSTOMY; PALLIATIVE TREATMENT;
CLINICAL-OUTCOMES; LAPAROSCOPIC GASTROJEJUNOSTOMY; GASTRODUODENAL

OBSTRUCTIONS; PANCREATIC-CANCER; DUODENAL STENT; ENTERAL STENT

AB Malignant gastric outlet obstruction (MGOO) is a clinical condition characterized by the mechanical obstruction of the pylorus or the duodenum due to tumor compression/infiltration, with consequent reduction or impossibility of an adequate oral intake. MGOO is mainly secondary to advanced pancreatic or gastric cancers, and significantly impacts on patients' survival and quality of life. Patients suffering from this condition often present with intractable vomiting and severe malnutrition, which further compromise therapeutic chances. Currently, palliative strategies are based primarily on surgical gastrojejunostomy and endoscopic enteral stenting with self-expanding metal stents. Several studies have shown that surgical approach has the advantage of a more durable relief of symptoms and the need of fewer re-interventions, at the cost of higher procedure-related risks and longer hospital stay. On the other hand, enteral stenting provides rapid clinical improvement, but have the limit of higher stent dysfunction rate due to tumor ingrowth and a subsequent need of frequent re-interventions. Recently, a third way has come from interventional endoscopic ultrasound, through the development of endoscopic ultrasound-guided gastroenterostomy technique with lumen-apposing metal stent. This new technique may ideally encompass the minimal invasiveness of an endoscopic procedure and the long-lasting effect of the surgical gastrojejunostomy, and brought encouraging results so far, even if prospective comparative trial are still lacking. In this Review, we described technical aspects and clinical outcomes of the above-cited therapeutic approaches, and discussed the open questions about the current management of MGOO.

C1 [Troncone, Edoardo; Blanco, Giovanna Del Vecchio; Monteleone, Giovanni] Univ Roma Tor Vergata, Dept Syst Med, I-00133 Rome, Italy.

[Fugazza, Alessandro; Cappello, Annalisa; Repici, Alessandro; Anderloni, Andrea] Humanitas Clin & Res Ctr IRCCS, Digest Endoscopy Unit, Via Manzoni 56, I-20089 Rozzano, Mi, Italy.

[Repici, Alessandro] Humanitas Univ, Dept Biomed Sci, Via Rita Levi Montalcini 4, I-20090 Milan, Italy.

[Teoh, Anthony Yuen Bun] Chinese Univ Hong Kong, Prince Wales Hosp, Dept Surg, Hong Kong 999077, Peoples R China.

C3 University of Rome Tor Vergata; Humanitas University; Prince of Wales

Hospital Hong Kong; Chinese University of Hong Kong

RP Anderloni, A (corresponding author), Humanitas Clin & Res Ctr IRCCS, Digest Endoscopy Unit, Via Manzoni 56, I-20089 Rozzano, Mi, Italy.

EM andrea.anderloni@humanitas.it

RI Repici, Alessandro/HFH-8162-2022; Fugazza, Alessandro/ABG-9381-2021;

Troncone, Edoardo/AHD-9276-2022; Anderloni, Andrea/ABE-9139-2020; Teoh,

Anthony/L-4796-2016; Monteleone, Giovanni/AAE-1418-2021

OI Repici, Alessandro/0000-0002-1621-6450; DEL VECCHIO BLANCO,

Giovanna/0000-0002-6327-563X; Fugazza, Alessandro/0000-0003-0485-4903;

Troncone, Edoardo/0000-0003-4520-6865; Anderloni,

Andrea/0000-0002-1021-0031; Cappello, Annalisa/0000-0002-9248-9549;

CR Adler DG, 2002, AM J GASTROENTEROL, V97, P72

Anderloni A, 2019, WORLD J GASTROENTERO, V25, P3857, DOI 10.3748/wjg.v25.i29.3857

Anderloni A, 2018, ANN GASTROENTEROL, V31, P735, DOI 10.20524/aog.2018.0299

Anderloni A, 2019, GASTROINTEST ENDOSC, V89, P69, DOI [10.1016/j.gie.2018.04.1746, 10.1016/j.gie.2018.08.047]

Anderloni A, 2018, AM J GASTROENTEROL, V113, P644, DOI 10.1038/s41395-018-0040-9

Anderloni A, 2016, ENDOSCOPY, V48, pE321, DOI 10.1055/s-0042-117425

Arigami T, 2016, ANTICANCER RES, V36, P5431, DOI 10.21873/anticanres.11121
Baron TH, 2001, NEW ENGL J MED, V344, P1681, DOI 10.1056/NEJM200105313442206
Binmoeller KF, 2012, ENDOSCOPY, V44, P499, DOI 10.1055/s-0032-1309382
Brimhall Bryan, 2011, Gastrointest Endosc Clin N Am, V21, P389, DOI 10.1016/j.giec.2011.04.002
Carbajo AY, 2020, J CLIN GASTROENTEROL, V54, P1, DOI 10.1097/MCG.0000000000001262
Chen YI, 2018, GASTROINTEST ENDOSC, V87, P1215, DOI 10.1016/j.gie.2017.07.030
Chen YI, 2017, SURG ENDOSC, V31, P2946, DOI 10.1007/s00464-016-5311-1
Costamagna G, 2012, DIGEST LIVER DIS, V44, P37, DOI 10.1016/j.dld.2011.08.012
Dollhopf M, 2017, GASTROINTEST ENDOSC, V86, P636, DOI 10.1016/j.gie.2017.02.027
Dormann A, 2004, ENDOSCOPY, V36, P543, DOI 10.1055/s-2004-814434
Fiori E, 2004, ANTICANCER RES, V24, P265
Ge PS, 2019, SURG ENDOSC, V33, P3404, DOI 10.1007/s00464-018-06636-3
Grunwald D, 2016, THER ADV GASTROENTER, V9, P815, DOI 10.1177/1756283X16667893
Hamada T, 2017, DIGEST ENDOSC, V29, P259, DOI 10.1111/den.12786
Hori Y, 2019, SURG ENDOSC, V33, P4026, DOI 10.1007/s00464-019-06694-1
Hori Y, 2015, J GASTROEN HEPATOL, V30, P1246, DOI 10.1111/jgh.12933
Itoi T, 2017, DIGEST ENDOSC, V29, P495, DOI 10.1111/den.12794
Itoi T, 2016, GASTROINTEST ENDOSC, V83, P461, DOI 10.1016/j.gie.2015.08.030
Itoi T, 2016, GUT, V65, P193, DOI 10.1136/gutjnl-2015-310348
Itoi T, 2013, GASTROINTEST ENDOSC, V78, P934, DOI 10.1016/j.gie.2013.09.025
Jang S, 2019, CLIN GASTROENTEROL H, V17, P1295, DOI 10.1016/j.cgh.2018.10.042
Jeon HH, 2014, SURG ENDOSC, V28, P988, DOI 10.1007/s00464-013-3268-x
Jeurnink SM, 2010, J GASTROENTEROL, V45, P537, DOI 10.1007/s00535-009-0181-0
Jeurnink SM, 2007, BMC GASTROENTEROL, V7, DOI 10.1186/1471-230X-7-18
Jeurnink SM, 2010, GASTROINTEST ENDOSC, V71, P490, DOI 10.1016/j.gie.2009.09.042
Jeurnink SM, 2009, SURG ENDOSC, V23, P562, DOI 10.1007/s00464-008-9880-5
Jung GS, 2000, RADIOLOGY, V216, P758, DOI 10.1148/radiology.216.3.r00au05758
Jung GS, 2002, J VASC INTERV RADIOL, V13, P275, DOI 10.1016/S1051-0443(07)61720-5
Kaw M, 2003, SURG ENDOSC, V17, P457, DOI 10.1007/s00464-002-8541-3
Khashab M, 2013, SURG ENDOSC, V27, P2068, DOI 10.1007/s00464-012-2712-7
Khashab MA, 2017, ENDOSC INT OPEN, V5, pE275, DOI 10.1055/s-0043-101695
Khashab MA, 2015, GASTROINTEST ENDOSC, V82, P932, DOI 10.1016/j.gie.2015.06.017
Khashab MA, 2015, GASTROINTEST ENDOSC, V81, P1234, DOI 10.1016/j.gie.2014.12.053
Kim CG, 2012, ENDOSCOPY, V44, P807, DOI 10.1055/s-0032-1309893
Kim ID, 2010, SCAND J GASTROENTERO, V45, P100, DOI 10.3109/00365520903410554
Kim JH, 2007, GASTROINTEST ENDOSC, V66, P256, DOI 10.1016/j.gie.2006.12.017
Kozarek RA, 1999, DIGEST DIS, V17, P14, DOI 10.1159/000016899
Larssen L, 2009, BMC GASTROENTEROL, V9, DOI 10.1186/1471-230X-9-45
Lee H, 2015, AM J GASTROENTEROL, V110, P1440, DOI 10.1038/ajg.2015.286
Lee KM, 2009, SCAND J GASTROENTERO, V44, P846, DOI 10.1080/00365520902929849
Liu SY, 2012, DIAGN INTERV RADIOL, V18, P360, DOI 10.4261/1305-3825.DIR.5045-11.1
Lorusso D, 2019, J GASTROINTEST ONCOL, V10, P283, DOI 10.21037/jgo.2018.10.10

Ly J, 2010, SURG ENDOSC, V24, P290, DOI 10.1007/s00464-009-0577-1
Maetani I, 1996, GASTROINTEST ENDOSC, V44, P468, DOI 10.1016/S0016-5107(96)70102-5
Maetani I, 2010, DIGEST ENDOSC, V22, P83, DOI 10.1111/j.1443-1661.2010.00942.x
Maetani I, 2009, GASTROINTEST ENDOSC, V69, P806, DOI 10.1016/j.gie.2008.06.009
Mehta S, 2006, SURG ENDOSC, V20, P239, DOI 10.1007/s00464-005-0130-9
Mintziras I, 2019, SURG ENDOSC, V33, P3153, DOI 10.1007/s00464-019-06955-z
Mussetto A, 2018, ANN GASTROENTEROL, V31, P535, DOI 10.20524/aog.2018.0287
Mutignani M, 2007, ENDOSCOPY, V39, P440, DOI 10.1055/s-2007-966327
Nevitt AW, 1998, GASTROINTEST ENDOSC, V47, P271, DOI 10.1016/S0016-5107(98)70326-8
Pais-Cunha I, 2018, EUR J GASTROEN HEPAT, V30, P1033, DOI 10.1097/MEG.0000000000001178
Pan YM, 2014, BMC GASTROENTEROL, V14, DOI 10.1186/1471-230X-14-170
Park CH, 2015, GASTROINTEST ENDOSC, V81, P321, DOI 10.1016/j.gie.2014.06.024
Park JM, 2015, DIGEST DIS SCI, V60, P524, DOI 10.1007/s10620-014-3343-8
Park JC, 2012, DIGEST LIVER DIS, V44, P999, DOI 10.1016/j.dld.2012.06.019
Perez-Miranda M, 2017, J CLIN GASTROENTEROL, V51, P896, DOI 10.1097/MCG.0000000000000887
Rademacher C, 2016, WORLD J GASTROENTERO, V22, P9554, DOI 10.3748/wjg.v22.i43.9554
Ratone JP, 2020, DIGEST LIVER DIS, V52, P51, DOI 10.1016/j.dld.2019.06.025
Roy A, 2012, SURG ENDOSC, V26, P3114, DOI 10.1007/s00464-012-2301-9
Sasaki T, 2015, DIGEST ENDOSC, V27, P37, DOI 10.1111/den.12321
Sasaki T, 2012, DIGEST ENDOSC, V24, P226, DOI 10.1111/j.1443-1661.2011.01208.x
Siddiqui UD, 2018, GASTROENTEROLOGY, V154, P1911, DOI 10.1053/j.gastro.2017.12.046
Staub J, 2018, GASTROINTEST ENDOSC, V87, P1499, DOI 10.1016/j.gie.2018.01.040
Tringali A, 2014, GASTROINTEST ENDOSC, V79, P66, DOI 10.1016/j.gie.2013.06.032
Tyberg A, 2016, ENDOSC INT OPEN, V4, pE276, DOI 10.1055/s-0042-101789
van den Berg MW, 2013, ENDOSCOPY, V45, P174, DOI 10.1055/s-0032-1326077
van Halsema EE, 2015, WORLD J GASTROENTERO, V21, P12468, DOI 10.3748/wjg.v21.i43.12468
van Hooft J, 2007, ENDOSCOPY, V39, P434, DOI 10.1055/s-2007-966338
van Hooft JE, 2011, ENDOSCOPY, V43, P671, DOI 10.1055/s-0030-1256383
van Hooft JE, 2009, GASTROINTEST ENDOSC, V69, P1059, DOI 10.1016/j.gie.2008.07.026
Varadarajulu S, 2011, GASTROINTEST ENDOSC, V74, P455, DOI 10.1016/j.gie.2011.04.011
Venu RP, 1998, ENDOSCOPY, V30, P553, DOI 10.1055/s-2007-1001343
Walter D, 2016, GUT, V65, P6, DOI 10.1136/gutjnl-2015-309925
Walter D, 2015, ENDOSCOPY, V47, P63, DOI 10.1055/s-0034-1378113
WATANAPA P, 1992, BRIT J SURG, V79, P8, DOI 10.1002/bjs.1800790105
WILSON RG, 1992, BRIT J SURG, V79, P1348, DOI 10.1002/bjs.1800791235
Yamao K, 2018, GASTROINTEST ENDOSC, V88, P66, DOI 10.1016/j.gie.2018.01.021
Yamao K, 2016, GASTROINTEST ENDOSC, V84, P757, DOI 10.1016/j.gie.2016.03.1498
Yates MR, 1998, ENDOSCOPY, V30, P266, DOI 10.1055/s-2007-1001253
NR 86
TC 86
Z9 107
U1 0

U2 3
PU BAISHIDENG PUBLISHING GROUP INC
PI PLEASANTON
PA 7041 Koll Center Parkway, Suite 160, PLEASANTON, CA, UNITED STATES
SN 1007-9327
EI 2219-2840
J9 WORLD J GASTROENTERO
JI World J. Gastroenterol.
PD APR 28
PY 2020
VL 26
IS 16
BP 1847
EP 1860
DI 10.3748/wjg.v26.i16.1847
PG 14
WC Gastroenterology & Hepatology
WE Science Citation Index Expanded (SCI-EXPANDED)
SC Gastroenterology & Hepatology
GA LS7HT
UT WOS:000536552400001
PM 32390697
OA Green Submitted, Green Accepted, hybrid
DA 2026-05-09
ER

PT J
AU Nakai, Y
Kogure, H
Isayama, H
Koike, K
AF Nakai, Yousuke
Kogure, Hirofumi
Isayama, Hiroyuki
Koike, Kazuhiko
TI Endoscopic Ultrasound-Guided Biliary Drainage for Unresectable Hilar
Malignant Biliary Obstruction
SO CLINICAL ENDOSCOPY
LA English
DT Review
DE Biliary drainage; Endosonography; Hilar biliary obstruction; Neoplasms
ID COVERED METAL STENT; SIDE-BY-SIDE; REVISION EFFICACY;

HEPATICOGASTROSTOMY; STRICTURES; PLACEMENT; INTERVENTION; MULTICENTER; CONVERSION; RESCUE

AB Endoscopic transpapillary biliary drainage is the current standard of care for unresectable hilar malignant biliary obstruction (MBO) and bilateral metal stent placement is shown to have longer patency. However, technical and clinical failure is possible and percutaneous transhepatic biliary drainage (PTBD) is sometimes necessary. Endoscopic ultrasound-guided biliary drainage (EUS-BD) is increasingly being reported as an alternative rescue procedure to PTBD. EUS-BD has a potential advantage of not traversing the biliary stricture and internal drainage can be completed in a single session. Some approaches to bilateral biliary drainage for hilar MBO under EUS-guidance include a bridging method, hepaticoduodenostomy, and a combination of BUS-BD and transpapillary biliary drainage. The aim of this review is to summarize data on EUS-BD for hilar MBO and to clarify its advantages over the conventional approaches such as endoscopic transpapillary biliary drainage and PTBD.

C1 [Nakai, Yousuke; Kogure, Hirofumi; Isayama, Hiroyuki; Koike, Kazuhiko] Univ Tokyo, Grad Sch Med, Dept Gastroenterol, Tokyo, Japan.

[Isayama, Hiroyuki] Juntendo Univ, Grad Sch Med, Dept Gastroenterol, Tokyo, Japan.

C3 University of Tokyo; Juntendo University

RP Nakai, Y (corresponding author), Univ Tokyo, Grad Sch Med, Dept Gastroenterol, Tokyo, Japan.; Nakai, Y (corresponding author), Univ Tokyo, Grad Sch Med, Dept Gastroenterol, Bunkyo Ku, 7-3-1 Hongo, Tokyo 1138655, Japan.

EM ynakai-tky@umin.ac.jp

RI Nakai, Yousuke/H-2964-2019; Isayama, Hiroyuki/AER-9916-2022

CR Bories E, 2007, ENDOSCOPY, V39, P287, DOI 10.1055/s-2007-966212

De Palma GD, 2001, GASTROINTEST ENDOSC, V53, P547, DOI 10.1067/mge.2001.113381

Dhir V, 2014, DIGEST ENDOSC, V26, P430, DOI 10.1111/den.12153

Han SJ, 2017, GASTROINTEST ENDOSC, V85, pAB88, DOI 10.1016/j.gie.2017.04.037

Kanno Y, 2017, INTERNAL MED, V56, P3145, DOI 10.2169/internalmedicine.9001-17

Kogure H, 2014, DIGEST ENDOSC, V26, P93, DOI 10.1111/den.12055

Lee TH, 2013, ENDOSCOPY, V45, P106, DOI 10.1055/s-0032-1325928

Lee TH, 2016, ENDOSCOPY, V48, P1129, DOI 10.1055/s-0042-112574

Lee TH, 2013, DIGEST DIS SCI, V58, P547, DOI 10.1007/s10620-012-2346-6

Minaga K, 2018, DIGEST ENDOSC, V30, P38, DOI 10.1111/den.12910

Minaga K, 2017, SURG ENDOSC, V31, P4764, DOI 10.1007/s00464-017-5553-6

Moole H, 2016, CAN J GASTROENTEROL, V2016, DOI 10.1155/2016/4726078

Moon JH, 2015, J HEPATO-BIL-PAN SCI, V22, P650, DOI 10.1002/jhbp.270

Moryoussef F, 2017, THER ADV GASTROENTER, V10, P537, DOI 10.1177/1756283X17702614

Mukai S, 2017, GASTROINTEST ENDOSC, V86, P203, DOI [10.1016/j.gie.2016.07.056, 10.1016/j.gie.2016.11.025, 10.1016/j.gie.2016.07.056]

Mukai T, 2013, J HEPATO-BIL-PAN SCI, V20, P214, DOI 10.1007/s00534-012-0508-8

Nakai Y, 2017, ENDOSC ULTRASOUND, V6, P205, DOI 10.4103/2303-9027.208172

Nakai Y, 2017, DIGEST ENDOSC, V29, P218, DOI 10.1111/den.12752

Nakai Y, 2016, ENDOSCOPY, V48, P1125, DOI 10.1055/s-0042-116595

Ogura T, 2017, J GASTROEN HEPATOL, V32, P163, DOI 10.1111/jgh.13437

Ogura T, 2015, ENDOSCOPY, V47, P72, DOI 10.1055/s-0034-1378111

Ogura T, 2014, ENDOSCOPY, V46, pE32, DOI 10.1055/s-0033-1359133

Paik WH, 2017, ENDOSCOPY, V49, P544, DOI 10.1055/s-0043-102388
Park DH, 2015, J HEPATO-BIL-PAN SCI, V22, P664, DOI 10.1002/jhbp.271
Park DH, 2010, GASTROINTEST ENDOSC, V71, P413, DOI 10.1016/j.gie.2009.10.015
Park SJ, 2013, GASTROINTEST ENDOSC, V78, P374, DOI 10.1016/j.gie.2013.04.183
Prachayakul V, 2015, WORLD J GASTROENTERO, V21, P10045, DOI 10.3748/wjg.v21.i34.10045
Sangchan A, 2012, GASTROINTEST ENDOSC, V76, P93, DOI 10.1016/j.gie.2012.02.048
Takahashi E, 2015, WORLD J GASTROENTERO, V21, P4946, DOI 10.3748/wjg.v21.i16.4946
Vienne A, 2010, GASTROINTEST ENDOSC, V72, P728, DOI 10.1016/j.gie.2010.06.040
Wang KX, 2016, GASTROINTEST ENDOSC, V83, P1218, DOI 10.1016/j.gie.2015.10.033
Yasuda I, 2013, DIGEST ENDOSC, V25, P81, DOI 10.1111/den.12060
NR 32
TC 47
Z9 54
U1 0
U2 2
PU KOREAN SOC GASTROINTESTINAL ENDOSCOPY
PI SEOUL
PA 2003 LG PALACE, 165-8 DONGGYO-DONG, MAPO-GU, SEOUL, 121-754, SOUTH KOREA
SN 2234-2400
EI 2234-2443
J9 CLIN ENDOSC
JI Clin. Endosc.
PD MAY
PY 2019
VL 52
IS 3
BP 220
EP 225
DI 10.5946/ce.2018.094
PG 6
WC Gastroenterology & Hepatology
WE Emerging Sources Citation Index (ESCI)
SC Gastroenterology & Hepatology
GA IA8LA
UT WOS:000469809100006
PM 30472818
OA Green Submitted, gold
DA 2026-05-09
ER

PT J
AU Sagami, R

Hayasaka, K
Nishikiori, H
Harada, H
Amano, Y
AF Sagami, Ryota
Hayasaka, Kenji
Nishikiori, Hidefumi
Harada, Hideaki
Amano, Yuji
TI Current Status in the Treatment of Acute Cholecystitis Patients
Receiving Antithrombotic Therapy: Is Endoscopic Drainage Feasible?- A
Systematic Review
SO CLINICAL ENDOSCOPY
LA English
DT Review
DE Acute cholecystitis; Antithrombotic therapy; Endoscopic transpapillary
gallbladder drainage; Endoscopic ultrasound-guided gallbladder drainage;
Percutaneous transhepatic gallbladder drainage
ID GUIDED GALLBLADDER DRAINAGE; APPOSING METAL STENT; LONG-TERM OUTCOMES;
HIGH-RISK PATIENTS; ELECTIVE LAPAROSCOPIC CHOLECYSTECTOMY; PERCUTANEOUS
CHOLECYSTOSTOMY; MANAGEMENT; EFFICACY; PLACEMENT; SAFETY
AB The bleeding complication risk of surgery or percutaneous transhepatic gallbladder drainage (PTGBD) may increase in patients with acute cholecystitis receiving antithrombotic therapy (ATT). Endoscopic gallbladder drainage (EGBD) may be recommended for such patients. English articles published between 1991 and 2018 in peer-reviewed journals that discuss cholecystectomy, PTGBD, and EGBD in patients with ATT or coagulopathy were reviewed to assess the safety of the procedures, especially in terms of the bleeding complication. There were 8 studies on cholecystectomy, 3 on PTGBD, and 1 on endoscopic transpapillary gallbladder drainage (ETGBD) in patients receiving ATT. With respect to EGBD, 28 studies on ETGBD (including 1 study already mentioned above) and 26 studies on endoscopic ultrasound-guided gallbladder drainage (EUS-GBD) were also analyzed. The overall bleeding complication rate in patients with ATT who underwent cholecystectomy was significantly higher than that in patients without ATT (6.5% [23/354] vs. 1.2% [26/2,224], p<0.001). However, the bleeding risk of cholecystectomy and PTGBD in patients receiving ATT was controversial. The overall technical success, clinical success, and bleeding complication rates of ETGBD vs. EUS-GBD were 84% vs. 96% (p<0.001), 92% vs. 97% (p<0.001), and 0.65% vs. 2.1% (p=0.005), respectively. One patient treated with ETGBD experienced bleeding complication among 191 patients with bleeding tendency. ETGBD may be an ideal drainage procedure for patients receiving ATT from the viewpoint of bleeding, although EUS-GBD is also efficacious.
C1 [Sagami, Ryota; Hayasaka, Kenji; Harada, Hideaki] New Tokyo Hosp, Dept Gastroenterol, 1271 Wanagaya, Matsudo, Chiba 2702232, Japan.
[Nishikiori, Hidefumi] Oita San Ai Med Ctr, Dept Gastroenterol, Oita, Japan.
[Amano, Yuji] New Tokyo Hosp, Dept Endoscopy, Chiba, Japan.
RP Sagami, R (corresponding author), New Tokyo Hosp, Dept Gastroenterol, 1271 Wanagaya, Matsudo, Chiba 2702232, Japan.
EM sagami1985@yahoo.co.jp

OI Sagami, Ryota/0000-0003-4961-3521

CR Ahmed O, 2018, SAUDI J GASTROENTERO, V24, P183, DOI 10.4103/sjg.SJG_506_17
Akahoshi K, 2017, AM SURGEON, V83, P486
Anderson K, 2014, AM J SURG, V208, P926, DOI 10.1016/j.amjsurg.2014.08.001
Baron TH, 2007, GASTROINTEST ENDOSC, V65, P735, DOI 10.1016/j.gie.2006.07.041
Cho DH, 2019, SURG ENDOSC, V33, P2135, DOI 10.1007/s00464-018-6485-5
Choi JH, 2017, GASTROINTEST ENDOSC, V85, P357, DOI 10.1016/j.gie.2016.07.067
Choi JH, 2014, ENDOSCOPY, V46, P656, DOI 10.1055/s-0034-1365720
Conway JD, 2005, GASTROINTEST ENDOSC, V61, P32, DOI 10.1016/S0016-5107(04)02445-9
de la Serna-Higuera C, 2013, GASTROINTEST ENDOSC, V77, P303, DOI 10.1016/j.gie.2012.09.021
Dewhurst C, 2012, AM J ROENTGENOL, V199, pW753, DOI 10.2214/AJR.11.8445
Dollhopf M, 2017, GASTROINTEST ENDOSC, V86, P636, DOI 10.1016/j.gie.2017.02.027
Ercan M, 2010, LANGENBECK ARCH SURG, V395, P247, DOI 10.1007/s00423-009-0483-y
FERETIS C, 1993, ENDOSCOPY, V25, P392, DOI 10.1055/s-2007-1010347
Fujikawa T, 2018, WORLD J CLIN CASES, V6, P767, DOI 10.12998/wjcc.v6.i14.767
Ge N, 2016, BMC GASTROENTEROL, V16, DOI 10.1186/s12876-016-0420-9
Hamada T, 2015, RADIOLOGY, V274, P605, DOI 10.1148/radiol.14140293
Hanan EL, 1999, SURGERY, V125, P223
Hatanaka T, 2016, INTERNAL MED, V55, P1411, DOI 10.2169/internalmedicine.55.5820
Iino C, 2018, ENDOSC INT OPEN, V6, pE594, DOI 10.1055/s-0044-102091
Imai H, 2016, GASTROINTEST ENDOSC, V84, P147, DOI 10.1016/j.gie.2015.12.024
Ingraham AM, 2010, J AM COLL SURGEONS, V211, P176, DOI 10.1016/j.jamcollsurg.2010.04.003
Inoue T, 2016, GASTROINTEST ENDOSC, V83, P905, DOI 10.1016/j.gie.2015.08.072
Irani S, 2017, CLIN GASTROENTEROL H, V15, P738, DOI 10.1016/j.cgh.2016.12.021
Irani S, 2015, GASTROINTEST ENDOSC, V82, P1110, DOI 10.1016/j.gie.2015.05.045
Itoi T, 2008, GASTROINTEST ENDOSC, V68, P455, DOI 10.1016/j.gie.2008.02.052
Itoi T, 2017, J HEPATO-BIL-PAN SCI, V24, P362, DOI 10.1002/jhbp.454
Itoi T, 2015, GASTROINTEST ENDOSC, V81, P111, DOI 10.1016/j.gie.2014.09.046
Itoi T, 2012, GASTROINTEST ENDOSC, V75, P870, DOI 10.1016/j.gie.2011.10.020
Itoi T, 2010, GASTROINTEST ENDOSC, V71, P1038, DOI 10.1016/j.gie.2010.01.026
Jang JW, 2012, GASTROENTEROLOGY, V142, P805, DOI 10.1053/j.gastro.2011.12.051
Jang JW, 2011, GASTROINTEST ENDOSC, V74, P176, DOI 10.1016/j.gie.2011.03.1120
JOHLIN FC, 1993, GASTROINTEST ENDOSC, V39, P645, DOI 10.1016/S0016-5107(93)70216-3
Joseph B, 2015, AM J SURG, V209, P689, DOI 10.1016/j.amjsurg.2014.04.014
Kahaleh M, 2016, DIGEST LIVER DIS, V48, P1054, DOI 10.1016/j.dld.2016.05.021
Kamata K, 2017, WORLD J GASTROENTERO, V23, P661, DOI 10.3748/wjg.v23.i4.661
Kawamoto Y, 2018, J HEPATO-BIL-PAN SCI, V25, P518, DOI 10.1002/jhbp.588
Kedia P, 2015, GASTROINTEST ENDOSC, V82, P1031, DOI 10.1016/j.gie.2015.03.1912
Khan MA, 2017, GASTROINTEST ENDOSC, V85, P76, DOI 10.1016/j.gie.2016.06.032
Kjaer DW, 2007, ENDOSCOPY, V39, P304, DOI 10.1055/s-2007-966335
Korte W, 2011, THROMB HAEMOSTASIS, V105, P743, DOI 10.1160/TH10-04-0217
Lee SS, 2007, GASTROINTEST ENDOSC, V66, P1008, DOI 10.1016/j.gie.2007.03.1080

Lee TH, 2011, ENDOSCOPY, V43, P702, DOI 10.1055/s-0030-1256226

Maekawa S, 2013, BMC GASTROENTEROL, V13, DOI 10.1186/1471-230X-13-65

Manta R, 2017, ENDOSC INT OPEN, V5, pE1111, DOI 10.1055/s-0043-118659

McCarthy ST, 2015, DIGEST DIS SCI, V60, P2516, DOI 10.1007/s10620-014-3371-4

McMahon AJ, 2000, LANCET, V356, P1632, DOI 10.1016/S0140-6736(00)03156-1

Mori Y, 2018, J HEPATO-BIL-PAN SCI, V25, P87, DOI 10.1002/jhbp.504

Mutignani M, 2009, ENDOSCOPY, V41, P539, DOI 10.1055/s-0029-1214727

Nakatsu T., 1997, J HEPATOBILIARY PANC, V4, P31, DOI DOI 10.1007/BF01211341

Noda T, 2014, HEPATO-GASTROENTEROL, V61, P1501, DOI 10.5754/hge13830

Ogawa O, 2008, CAN J GASTROENTEROL, V22, P681, DOI 10.1155/2008/702516

Oh D, 2019, GASTROINTEST ENDOSC, V89, P289, DOI 10.1016/j.gie.2018.08.052

Ono K, 2013, SURG LAPARO ENDO PER, V23, P97, DOI 10.1097/SLE.0b013e318278cdf8

Ono S, 2015, DIGEST ENDOSC, V27, P649, DOI 10.1111/den.12498

Pannala R, 2008, Minerva Gastroenterol Dietol, V54, P107

Patel IJ, 2012, J VASC INTERV RADIOL, V23, P727, DOI 10.1016/j.jvir.2012.02.012

Patel M, 2000, AM SURGEON, V66, P33

Schlenker C, 2006, AM J GASTROENTEROL, V101, P278, DOI 10.1111/j.1572-0241.2006.00403.x

Shibasaki S, 2014, J HEPATO-BIL-PAN SCI, V21, P335, DOI 10.1002/jhbp.28

Shrestha R, 1999, LIVER TRANSPLANT SUR, V5, P275, DOI 10.1002/lt.500050402

Siddiqui A, 2019, SURG ENDOSC, V33, P1260, DOI 10.1007/s00464-018-6406-7

Song TJ, 2010, GASTROINTEST ENDOSC, V71, P634, DOI 10.1016/j.gie.2009.11.024

Takagi W, 2016, THER ADV GASTROENTER, V9, P19, DOI 10.1177/1756283X15609285

TAMADA K, 1991, ENDOSCOPY, V23, P2, DOI 10.1055/s-2007-1010596

Teoh AYB, 2017, ENDOSCOPY, V49, P130, DOI 10.1055/s-0042-119036

Toyota N, 2006, J HEPATO-BILIARY-PAN, V13, P80, DOI 10.1007/s00534-005-1062-4

Tujios SR, 2015, J CLIN GASTROENTEROL, V49, P771, DOI 10.1097/MCG.0000000000000269

Tyberg A, 2018, J CLIN GASTROENTEROL, V52, P79, DOI 10.1097/MCG.0000000000000786

Walter D, 2016, GUT, V65, P6, DOI 10.1136/gutjnl-2015-309925

Widmer J, 2015, CLIN ENDOSC, V48, P411, DOI 10.5946/ce.2015.48.5.411

Yane K, 2015, GUT LIVER, V9, P239, DOI 10.5009/gnl14070

Yang MJ, 2016, SCAND J GASTROENTERO, V51, P472, DOI 10.3109/00365521.2015.1115116

Yokoe M, 2018, J HEPATO-BIL-PAN SCI, V25, P41, DOI 10.1002/jhbp.515

Yun JH, 2017, ANN SURG TREAT RES, V92, P143, DOI 10.4174/astr.2017.92.3.143

NR 74

TC 17

Z9 18

U1 0

U2 0

PU KOREAN SOC GASTROINTESTINAL ENDOSCOPY

PI SEOUL

PA 2003 LG PALACE, 165-8 DONGGYO-DONG, MAPO-GU, SEOUL, 121-754, SOUTH KOREA

SN 2234-2400

EI 2234-2443
J9 CLIN ENDOSC
JI Clin. Endosc.
PD MAR
PY 2020
VL 53
IS 2
BP 176
EP 188
DI 10.5946/ce.2019.177
PG 13
WC Gastroenterology & Hepatology
WE Emerging Sources Citation Index (ESCI)
SC Gastroenterology & Hepatology
GA KY6KR
UT WOS:000522682300010
PM 31914723
OA Green Submitted, Green Accepted, gold
DA 2026-05-09
ER

PT J
AU Tamura, T
Kitano, M
AF Tamura, Takashi
Kitano, Masayuki
TI Role of CH-EUS as guidance for EUS-biliary drainage malignant
obstruction
SO MINERVA GASTROENTEROLOGY
LA English
DT Article
DE Biliary tract; Drainage; Endosonography; Bile duct neoplasms;
Choledochostomy
ID HARMONIC ENDOSCOPIC ULTRASONOGRAPHY; DIFFERENTIAL-DIAGNOSIS; ERCP
AB Endoscopic ultrasound (EUS)-guided biliary drainage is the usual method of choice for patients with biliary obstructions difficult to treat by endoscopic retrograde cholangiopancreatography. In some patients, however, the bile duct is difficult to detect during EUS-guided biliary drainage. Contrast-enhanced harmonic endoscopic ultrasound has reportedly been useful for interventional EUS procedures. This study describes five patients who underwent EUS-guided biliary drainage with contrast-enhanced harmonic imaging between April 2017 and March 2020 for malignant biliary obstruction due to bile duct cancer. The procedure was performed through an intrahepatic bile duct approach in three patients and through an extrahepatic bile duct approach in the other two. Although fundamental B-mode EUS was unable to detect the target intrahepatic bile duct because the duct was filled with debris, contrast-enhanced harmonic imaging was able to detect

the bile duct by clarifying its border with the hepatic parenchyma. Similarly, in patients who underwent extrahepatic bile duct approach, fundamental B-mode EUS could not distinguish between debris and tumor, whereas contrast-enhanced harmonic EUS was able to differentiate between them. The procedure was technically and clinically successful in all five patients. One patient experienced bleeding as an adverse event. In conclusion, EUS-guided biliary drainage with contrast-enhanced harmonic imaging is useful in detecting the fluid space in bile ducts filled with debris or filled with debris and tumor invasion. (Cite this article as: Tamura T, Kitano M. Role of CH-EUS as guidance for EUS-biliary drainage malignant obstruction. Minerva Gastroenterol 2022;68:210-5. DOI: 10.23736/S2724-5985.21.02859-X)
C1 [Tamura, Takashi; Kitano, Masayuki] Wakayama Med Univ, Dept Internal Med 2, Kimiidera811-1, Wakayama 6418509, Japan.
C3 Wakayama Medical University
RP Kitano, M (corresponding author), Wakayama Med Univ, Dept Internal Med 2, Kimiidera811-1, Wakayama 6418509, Japan.
EM kitano@wakayama-med.ac.jp
RI Tamura, Takashi/ADO-2791-2022
CR ASGE, 1999, GASTROINTEST ENDOSC, V50, P910
Bailey AA, 2008, ENDOSCOPY, V40, P296, DOI 10.1055/s-2007-995566
Dhindsa BS, 2020, ENDOSC ULTRASOUND, V9, P101, DOI 10.4103/eus.eus_80_19
Fusaroli P, 2016, GASTROINTEST ENDOSC, V84, P587, DOI 10.1016/j.gie.2016.06.006
Fusaroli P, 2015, ENDOSC ULTRASOUND, V4, P336, DOI 10.4103/2303-9027.170427
Fusaroli P, 2014, PANCREAS, V43, P584, DOI 10.1097/MPA.0000000000000081
Fusaroli P, 2012, DIGEST DIS SCI, V57, P602, DOI 10.1007/s10620-011-1961-y
Giovannini M, 2001, ENDOSCOPY, V33, P898, DOI 10.1055/s-2001-17324
Guo JT, 2018, ENDOSC ULTRASOUND, V7, P356, DOI 10.4103/eus.eus_53_18
Kamata K, 2018, DIGEST ENDOSC, V30, P98, DOI 10.1111/den.12900
Kamata K, 2017, J GASTROEN HEPATOL, V32, P1686, DOI 10.1111/jgh.13766
Kitano M, 2019, J GASTROENTEROL, V54, P19, DOI 10.1007/s00535-018-1519-2
Minaga K, 2019, DIGEST ENDOSC, V31, P575, DOI 10.1111/den.13406
Minaga K, 2017, ENDOSCOPY, V49, pE281, DOI 10.1055/s-0043-117939
Minaga K, 2016, ENDOSCOPY, V48, pE228, DOI 10.1055/s-0042-109059
Minami Y, 2020, HEPATOMA RES, V2020, DOI [10.20517/2394-5079.2020.32, 10.20517/2394-5079.2020.32, DOI 10.20517/2394-5079.2020.32]
Sharaiha RZ, 2017, GASTROINTEST ENDOSC, V85, P904, DOI 10.1016/j.gie.2016.12.023
Tamura T, 2021, J HEPATO-BIL-PAN SCI, V28, pe1, DOI 10.1002/jhbp.834
Yamashita Y, 2019, DIGEST ENDOSC, V31, P125, DOI 10.1111/den.13290
NR 19
TC 5
Z9 6
U1 0
U2 3
PU EDIZIONI MINERVA MEDICA
PI TURIN
PA CORSO BRAMANTE 83-85 INT JOURNALS DEPT., 10126 TURIN, ITALY

SN 2724-5985
EI 2724-5365
J9 MINERVA GASTROENTERO
JI Minerva Gastroenterol.
PD JUN
PY 2022
VL 68
IS 2
BP 210
EP 215
DI 10.23736/S2724-5985.21.02859-X
PG 6
WC Gastroenterology & Hepatology
WE Science Citation Index Expanded (SCI-EXPANDED)
SC Gastroenterology & Hepatology
GA 2P1CA
UT WOS:000819487200009
PM 33793159
DA 2026-05-09
ER

PT J
AU Ono, M
Ikeda, Y
Ohmori, G
Arihara, Y
Shibuya, R
Uesugi, A
Oiwa, S
Ito, R
Usami, M
Yamada, M
Abe, T
Maeda, M
AF Ono, Michihiro
Ikeda, Yuki
Ohmori, Ginji
Arihara, Yohei
Shibuya, Ryo
Uesugi, Atsushi
Oiwa, Shutaro
Ito, Ryo

Usami, Makoto
Yamada, Michiko
Abe, Tomoyuki
Maeda, Masahiro
TI Efficacy and safety of endoscopic ultrasound-guided gallbladder drainage without dilation by using a 0.035-inch stiff guidewire
SO DEN OPEN
LA English
DT Article
DE acute cholecystitis; endoscopy; dilation; drainage; stents
ID ACUTE CHOLECYSTITIS; STENT
AB Endoscopic ultrasound-guided gallbladder drainage for patients with cholecystitis and high surgical risk is commonly performed by dilating the fistula before inserting the delivery sheath; however, this carries an increased risk of peritonitis. To overcome this problem, we developed a new technique that did not require dilation, using a 0.035-inch stiff guidewire, and retrospectively evaluated the efficacy and safety of this technique. This retrospective case series report collected data on non-surgical patients who underwent endoscopic ultrasound-guided gallbladder drainage for various indications at Steel Memorial Muroran Hospital between November 2020 and October 2022. A total of 71 patients were included (mean age 83 +/- 7.6 years; 33 women and 38 men). Breakthrough of the delivery sheath without dilation of the fistula was successful in 97.2% (n = 69) of patients. The success rate of stent placement was 98.6% (n = 70), as was the clinical success rate. Complications occurred in 2.8% (n = 2) of patients. Early and late adverse events occurred in 2.8% (n = 2) and 12.7% (n = 9) of patients, respectively. The mean procedure time was 24.8 +/- 9.3 min. If a 0.035-inch stiff guidewire is used, the dilation procedure can be omitted in the endoscopic ultrasound-guided gallbladder drainage using self-expandable metal stents.
C1 [Ono, Michihiro] Steel Mem Muroran Hosp, Dept Pancreatobiliary Med, Hokkaido, Japan.
[Ikeda, Yuki; Ohmori, Ginji] Oji Gen Hosp, Dept Gastroenterol, Hokkaido, Japan.
[Arihara, Yohei; Shibuya, Ryo; Ito, Ryo] Sapporo Med Univ, Sch Med, Dept Med Oncol, Hokkaido, Japan.
[Shibuya, Ryo; Uesugi, Atsushi; Oiwa, Shutaro; Ito, Ryo; Usami, Makoto; Yamada, Michiko; Abe, Tomoyuki; Maeda, Masahiro] Steel Mem Muroran Hosp, Dept Gastroenterol, Hokkaido, Japan.
[Ono, Michihiro] Steel Mem Muroran Hosp, Dept Pancreatobiliary Med, 1-45 Chiribetsucho, Muroran city, Hokkaido 0500076, Japan.
C3 Sapporo Medical University
RP Ono, M (corresponding author), Steel Mem Muroran Hosp, Dept Pancreatobiliary Med, 1-45 Chiribetsucho, Muroran city, Hokkaido 0500076, Japan.
EM onomichihiroono34@gmail.com
OI ABE, TOMOYUKI/0009-0000-6816-6187; Ono, Michihiro/0009-0004-9346-6738;
Oiwa, Shutaro/0000-0003-4415-0214; Usami, Makoto/0009-0004-2570-1011;
Ikeda, Yuki/0000-0002-7371-8326
FU None
FX We thank Editage () for English language editing.
CR Anderloni A, 2018, ANN GASTROENTEROL, V31, P735, DOI 10.20524/aog.2018.0299
CHARLSON M, 1994, J CLIN EPIDEMIOL, V47, P1245, DOI 10.1016/0895-4356(94)90129-5
CHARLSON ME, 1987, J CHRON DIS, V40, P373, DOI 10.1016/0021-9681(87)90171-8

Cotton PB, 2010, GASTROINTEST ENDOSC, V71, P446, DOI 10.1016/j.gie.2009.10.027
de la Serna-Higuera C, 2013, GASTROINTEST ENDOSC, V77, P303, DOI 10.1016/j.gie.2012.09.021
Honjo M, 2018, ENDOSC ULTRASOUND, V7, P376, DOI 10.4103/eus.eus_2_18
Law RJ, 2021, GASTROINTEST ENDOSC, V94, P457, DOI 10.1016/j.gie.2021.05.020
Lisotti A, 2020, ENDOSCOPY, V52, P517, DOI [10.1055/a-1065-1678, 10.1055/a-1157-6615, 10.1055/a-1065-1678]
Ogura T, 2019, DIGEST ENDOSC, V31, P55, DOI 10.1111/den.13334
Okamoto K, 2018, J HEPATO-BIL-PAN SCI, V25, P55, DOI 10.1002/jhbp.516
Ono M, 2023, ENDOSCOPY, V55, pE450, DOI 10.1055/a-2015-2804
Sagami R, 2020, ANN GASTROENTEROL, V33, P391, DOI 10.20524/aog.2020.0496
Su JY, 2023, ENDOSCOPY, V55, pE42, DOI 10.1055/a-1929-9259
Teoh AYB, 2020, GUT, V69, P1085, DOI 10.1136/gutjnl-2019-319996
Teoh AYB, 2017, ENDOSCOPY, V49, P130, DOI 10.1055/s-0042-119036
Teoh AYB, 2014, GASTROINTEST ENDOSC, V80, P1171, DOI 10.1016/j.gie.2014.03.038
Tyberg A, 2018, J CLIN GASTROENTEROL, V52, P79, DOI 10.1097/MCG.0000000000000786
Walter D, 2016, GUT, V65, P6, DOI 10.1136/gutjnl-2015-309925
Yokoe M, 2018, J HEPATO-BIL-PAN SCI, V25, P41, DOI 10.1002/jhbp.515
Yoo HW, 2021, ENDOSCOPY, V53, P922, DOI 10.1055/a-1301-1526
NR 20
TC 0
Z9 0
U1 0
U2 1
PU WILEY
PI HOBOKEN
PA 111 RIVER ST, HOBOKEN 07030-5774, NJ USA
SN 2692-4609
J9 DEN OPEN
JI DEN Open
PD JAN 6
PY 2024
VL 4
IS 1
AR e327
DI 10.1002/deo2.327
PG 8
WC Gastroenterology & Hepatology
WE Emerging Sources Citation Index (ESCI)
SC Gastroenterology & Hepatology
GA EE1H3
UT WOS:001137145000001
PM 38188358
OA Green Submitted, gold

DA 2026-05-09
ER

PT J
AU Tan, SL
Zhong, CY
Ren, YT
Luo, XJ
Xu, J
Peng, Y
Fu, XS
Tang, XW
AF Tan, Shali
Zhong, Chunyu
Ren, Yutang
Luo, Xujuan
Xu, Jin
Peng, Yan
Fu, Xiangsheng
Tang, Xiaowei
TI Are Lumen-Apposing Metal Stents More Effective Than Plastic Stents for
the Management of Pancreatic Fluid Collections: An Updated Systematic
Review and Meta-analysis
SO GASTROENTEROLOGY RESEARCH AND PRACTICE
LA English
DT Review
ID WALLED-OFF NECROSIS; ULTRASOUND-GUIDED DRAINAGE; ENHANCED
DELIVERY-SYSTEM; DOUBLE-PIGTAIL STENTS; ENDOSCOPIC ULTRASOUND; EFFICACY;
SAFETY; NECROSECTOMY; OUTCOMES; THERAPY
AB Background and Aims. Recently, a new type of metal stent, named lumen-apposing metal stents (LAMS), has been designed to manage pancreatic fluid collections (PFC), and a few studies have reported its efficacy and safety. Therefore, we conducted this meta-analysis to investigate the role of LAMS for PFC. Methods. We searched the studies from PubMed, MEDLINE, Embase, and Cochrane databases from inception to May 2019. We extracted the data and analyzed the technical success, clinical success, and adverse events of LAMS to evaluate its efficacy and safety. Results. Twenty studies with 1534 patients were included. The pooled technical success, clinical success, and adverse event rates of LAMS for PFC were 96.2% (95% confidence interval (CI): 94.6%-97.4%), 86.8% (95% CI: 83.1%-89.8%), and 20.7% (95% CI: 16.1%-26.1%), respectively. Eight studies including 875 patients compared the clinical outcomes of LAMS with plastic stents. The pooled risk ratio (RR) of technical success and clinical success for LAMS and plastic stent was 1.01 (95% CI: 0.98-1.04, P=0.62) and 1.06 (95% CI: 1.01-1.12, P=0.03), respectively. As for the overall adverse events, the pooled RR was 1.51 (95% CI: 0.67-3.44, P=0.32). Conclusions. Our current study revealed that LAMS has advantages over plastic stents for PFC, with higher clinical success rate and lower complication rate of infection and occlusion.
C1 [Tan, Shali; Zhong, Chunyu; Luo, Xujuan; Xu, Jin; Peng, Yan; Tang, Xiaowei] Southwest Med Univ, Dept

Gastroenterol, Affiliated Hosp, Luzhou, Peoples R China.
[Ren, Yutang] Peoples Hosp Lianshui, Dept Gastroenterol, Huaian, Peoples R China.
[Fu, Xiangsheng] North Sichuan Med Coll, Dept Gastroenterol, Affiliated Hosp, Nanchong, Peoples R China.
C3 Southwest Medical University; North Sichuan Medical University
RP Tang, XW (corresponding author), Southwest Med Univ, Dept Gastroenterol, Affiliated Hosp, Luzhou, Peoples R China.; Fu, XS (corresponding author), North Sichuan Med Coll, Dept Gastroenterol, Affiliated Hosp, Nanchong, Peoples R China.
EM 957242961@qq.com; 447166418@qq.com; pumcren@126.com; 509255311@qq.com;
251905683@qq.com; 1806857826@qq.com; drfuxs@gmail.com;
solitude5834@hotmail.com
RI Fu, Xiangsheng/H-9411-2019; Tang, Xiaowei/JFS-6037-2023
OI Fu, Xiangsheng/0000-0001-8889-3314; Tang, Xiaowei/0000-0001-6064-0526
FU Medical science and technology plan projects of Zhejiang Province
[2017196257]; Youth Foundation of Southwest Medical University
[0903-00031099]; Doctoral research start-up funding project of the
Affiliated Hospital of Southwest Medical University [16229]
FX This study is an independent research funded by the following grants:
Medical science and technology plan projects of Zhejiang Province (No.
2017196257), Youth Foundation of Southwest Medical University (No.
0903-00031099), and Doctoral research start-up funding project of the
Affiliated Hospital of Southwest Medical University (No. 16229). Thanks
for the DDW 2019 for presenting our preliminary research results as an
abstract.
CR Aburajab M, 2018, GASTROINTEST ENDOSC, V87, P1248, DOI 10.1016/j.gie.2017.11.033
Adler DG, 2019, ENDOSC ULTRASOUND, V8, P36, DOI 10.4103/eus.eus_30_17
Anderloni A, 2018, ANN GASTROENTEROL, V31, P735, DOI 10.20524/aog.2018.0299
Bakker OJ, 2012, JAMA-J AM MED ASSOC, V307, P1053, DOI 10.1001/jama.2012.276
Bang JY, 2019, GUT, V68, P1200, DOI 10.1136/gutjnl-2017-315335
Bang JY, 2017, GUT, V66, P2054, DOI 10.1136/gutjnl-2016-312812
Bang JY, 2017, DIGEST ENDOSC, V29, P83, DOI 10.1111/den.12681
Banks PA, 2013, GUT, V62, P102, DOI 10.1136/gutjnl-2012-302779
Bapaye A, 2017, DIGEST ENDOSC, V29, P104, DOI 10.1111/den.12704
Bekkali NLH, 2017, ENDOSC INT OPEN, V5, pE1189, DOI 10.1055/s-0043-120831
Brimhall B, 2018, CLIN GASTROENTEROL H, V16, P1521, DOI 10.1016/j.cgh.2018.02.021
Chen YI, 2018, ENDOSC INT OPEN, V6, pE780, DOI 10.1055/a-0611-5082
Chen YI, 2018, GASTROINTEST ENDOSC, V88, P267, DOI 10.1016/j.gie.2018.03.021
Fotoohi M, 1999, RADIOLOGY, V213, P573, DOI 10.1148/radiology.213.2.r99nv19573
Ge N, 2017, J TRANSL INTERN MED, V5, P213, DOI 10.1515/jtim-2017-0036
Gornals JB, 2016, SURG ENDOSC, V30, P2592, DOI 10.1007/s00464-015-4505-2
GRIMM H, 1992, GASTROINTEST ENDOSC, V38, P170, DOI 10.1016/S0016-5107(92)70384-8
Hammad T, 2018, DIGEST DIS SCI, V63, P289, DOI 10.1007/s10620-017-4851-0
Jadad AR, 1996, CONTROL CLIN TRIALS, V17, P1, DOI 10.1016/0197-2456(95)00134-4

Khan MA, 2018, ENDOSC INT OPEN, V6, pE474, DOI 10.1055/s-0044-102299
Lakhtakia S, 2017, GASTROINTEST ENDOSC, V85, P1243, DOI 10.1016/j.gie.2016.10.037
Lang GD, 2018, GASTROINTEST ENDOSC, V87, P150, DOI 10.1016/j.gie.2017.06.029
Mukai S, 2015, ENDOSCOPY, V47, P47, DOI 10.1055/s-0034-1377966
Rinninella E, 2015, GASTROINTEST ENDOSC, V82, P1039, DOI 10.1016/j.gie.2015.04.006
Saumoy Monica, 2018, Gastrointest Endosc Clin N Am, V28, P197, DOI [10.1016/j.giec.2017.11.007, 10.1016/j.giec.2017.11.007]
Shah RJ, 2015, CLIN GASTROENTEROL H, V13, P747, DOI 10.1016/j.cgh.2014.09.047
Sharaiha RZ, 2016, CLIN GASTROENTEROL H, V14, P1797, DOI 10.1016/j.cgh.2016.05.011
Shin HC, 2019, CLIN ENDOSC, V52, P353
Siddiqui AA, 2017, GASTROINTEST ENDOSC, V85, P758, DOI 10.1016/j.gie.2016.08.014
Singhal S, 2013, CLIN ENDOSC, V46, P506, DOI 10.5946/ce.2013.46.5.506
Song TJ, 2019, GASTROINTEST ENDOSC, V90, P507, DOI 10.1016/j.gie.2019.05.033
Stang A, 2010, EUR J EPIDEMIOL, V25, P603, DOI 10.1007/s10654-010-9491-z
Varadarajulu S, 2011, J GASTROINTEST SURG, V15, P2080, DOI 10.1007/s11605-011-1621-8
Varadarajulu S, 2009, GASTROINTEST ENDOSC, V70, P1260, DOI 10.1016/j.gie.2009.06.007
Walter D, 2015, ENDOSCOPY, V47, P63, DOI 10.1055/s-0034-1378113
Wang ZJ, 2019, J GASTROEN HEPATOL, V34, P791, DOI 10.1111/jgh.14557
Wells GA., 2020, NEWCASTLE OTTAWA SCA
Wiersema MJ, 1996, GASTROINTEST ENDOSC, V44, P614, DOI 10.1016/S0016-5107(96)70022-6
Yang D, 2018, ENDOSC INT OPEN, V6, pE1267, DOI 10.1055/a-0732-502
Yoo J, 2017, ENDOSC ULTRASOUND, V6, P131, DOI 10.4103/2303-9027.204814
NR 40
TC 22
Z9 28
U1 1
U2 1
PU WILEY
PI HOBOKEN
PA 111 RIVER ST, HOBOKEN 07030-5774, NJ USA
SN 1687-6121
EI 1687-630X
J9 GASTROENT RES PRACT
JI Gastroenterol. Res. Pract.
PD APR 20
PY 2020
VL 2020
AR 4952721
DI 10.1155/2020/4952721
PG 13
WC Gastroenterology & Hepatology
WE Science Citation Index Expanded (SCI-EXPANDED)

SC Gastroenterology & Hepatology
GA LM5KO
UT WOS:000532287500002
PM 32382266
OA Green Submitted, gold
DA 2026-05-09
ER

PT J
AU Hama, K
Tanaka, R
Itoi, T
AF Hama, Kazuki
Tanaka, Reina
Itoi, Takao
TI Troubleshooting the migration of endoscopic ultrasound-guided pancreatic duct drainage stent to avoid repuncture
SO DIGESTIVE ENDOSCOPY
LA English
DT Article
AB a video of this article.
C1 [Hama, Kazuki; Tanaka, Reina; Itoi, Takao] Tokyo Med Univ, Dept Gastroenterol & Hepatol, 6-7-1 Nishihinjuku, Tokyo 1600023, Japan.
C3 Tokyo Medical University
RP Itoi, T (corresponding author), Tokyo Med Univ, Dept Gastroenterol & Hepatol, 6-7-1 Nishihinjuku, Tokyo 1600023, Japan.
EM itoi@tokyo-med.ac.jp
OI Hama, Kazuki/0000-0003-0149-2249
CR Chapman CG, 2016, CLIN ENDOSC, V49, P161, DOI 10.5946/ce.2016.011
Ergun M, 2011, ENDOSCOPY, V43, P518, DOI 10.1055/s-0030-1256333
Itoi T, 2015, ENDOSCOPY, V47, P462, DOI 10.1055/s-0034-1391083
Itoi T, 2013, DIGEST ENDOSC, V25, P241, DOI 10.1111/den.12048
NR 4
TC 0
Z9 0
U1 0
U2 0
PU WILEY
PI HOBOKEN
PA 111 RIVER ST, HOBOKEN 07030-5774, NJ USA
SN 0915-5635
EI 1443-1661

J9 DIGEST ENDOSC
JI Dig. Endosc.
PD NOV
PY 2024
VL 36
IS 11
BP 1288
EP 1289
DI 10.1111/den.14906
EA SEP 2024
PG 2
WC Gastroenterology & Hepatology; Surgery
WE Science Citation Index Expanded (SCI-EXPANDED)
SC Gastroenterology & Hepatology; Surgery
GA L6O4R
UT WOS:001308347100001
PM 39252687
OA hybrid
DA 2026-05-09
ER

PT J
AU Amano, M
Ogura, T
Onda, S
Takagi, W
Sano, T
Okuda, A
Miyano, A
Masuda, D
Higuchi, K
AF Amano, Mio
Ogura, Takeshi
Onda, Saori
Takagi, Wataru
Sano, Tastsushi
Okuda, Astushi
Miyano, Akira
Masuda, Daisuke
Higuchi, Kazuhide
TI Prospective clinical study of endoscopic ultrasound-guided biliary
drainage using novel balloon catheter (with video)

SO JOURNAL OF GASTROENTEROLOGY AND HEPATOLOGY
LA English
DT Article
DE EUS; EUS-guided biliary drainage; EUS-CDS; EUS-HGS; EUS-FNA
ID STENT; METAL
AB Background and Aim: On endoscopic ultrasound (EUS)-guided biliary drainage, fistula dilation may be accompanied by leakage of bile juice, potentially along with bacteria or air, so this procedure should be kept as simple and short as possible. To date, various techniques to dilate the fistula have been reported. This prospective, single-center, single-arm study evaluated the technical feasibility and safety of EUS-guided biliary drainage using novel balloon catheters.

Methods: Tip of novel balloon catheters is only 3 Fr and tapered, and the catheters also show favorable push ability. First, biliary tract was punctured using 19-G fine needle aspiration needle. Next, we immediately inserted the novel balloon catheter, and the biliary tract and intestinal wall were dilated. Then, we performed metallic stent placement without any dilation fistula.

Results: A total of 20 patients were prospectively enrolled in this study. Technical success rate was 100%. Eleven patients underwent EUS-guided choledochoduodenostomy, and nine patients underwent EUS-guided hepaticogastrostomy. Median procedure time was only 11 min (range, 8-16 min) for EUS-guided choledochoduodenostomy and 14 min (range, 11-18 min) for EUS-guided hepaticogastrostomy. Adverse events were seen in 15% (3/20; self-limited abdominal pain n = 2, peritonitis n = 1).

Conclusions: Although additional cases and a randomized controlled comparison with another dilation technique such as the graded dilation or cystotome dilation technique are needed, our technique may be helpful for EUS-guided biliary drainage.
C1 [Amano, Mio; Ogura, Takeshi; Onda, Saori; Takagi, Wataru; Sano, Tastsushi; Okuda, Astushi; Miyano, Akira; Masuda, Daisuke; Higuchi, Kazuhide] Osaka Med Coll, Dept Internal Med 2, 2-7 Daigakuchou, Osaka 5698686, Japan.
C3 Osaka Medical & Pharmaceutical University
RP Ogura, T (corresponding author), Osaka Med Coll, Dept Internal Med 2, 2-7 Daigakuchou, Osaka 5698686, Japan.
EM oguratakeshi0411@yahoo.co.jp
RI Ogura, Takeshi/AAU-5643-2021
CR Bang JY, 2015, DIGEST ENDOSC, V27, P486, DOI 10.1111/den.12418
Cotton PB, 2010, GASTROINTEST ENDOSC, V71, P446, DOI 10.1016/j.gie.2009.10.027
Hara K, 2011, AM J GASTROENTEROL, V106, P1239, DOI 10.1038/ajg.2011.84
Khashab MA, 2015, GASTROINTEST ENDOSC, V82, P993, DOI 10.1016/j.gie.2015.06.043
Mukai S, 2015, J GASTROENTEROL, V50, P211, DOI 10.1007/s00535-014-0957-8
Noh SH, 2010, GASTROINTEST ENDOSC, V71, P1314, DOI 10.1016/j.gie.2009.12.045
Ogura T, 2015, J GASTROEN HEPATOL, V30, P1748, DOI 10.1111/jgh.13021
Ogura T, 2016, DIGEST DIS SCI, V61, P303, DOI 10.1007/s10620-015-3841-3
Ogura T, 2015, ENDOSCOPY, V47, pE583, DOI 10.1055/s-0034-1393381
Ogura T, 2015, ENDOSCOPY, V47, pE573, DOI 10.1055/s-0034-1393380
Ogura T, 2015, ENDOSCOPY, V47, pE245, DOI 10.1055/s-0034-1391859
Ogura T, 2015, WORLD J GASTROENTERO, V21, P820, DOI 10.3748/wjg.v21.i3.820
Paik WH, 2014, WORLD J GASTROENTERO, V20, P5051, DOI 10.3748/wjg.v20.i17.5051
Park DH, 2015, J GASTROEN HEPATOL, V30, P1461, DOI 10.1111/jgh.13027
Park Do Hyun, 2012, Gastrointest Endosc Clin N Am, V22, P271, DOI 10.1016/j.giec.2012.04.009

Park DH, 2011, GASTROINTEST ENDOSC, V74, P1276, DOI 10.1016/j.gie.2011.07.054
Prachayakul V, 2015, WORLD J GASTROENTERO, V21, P2725, DOI 10.3748/wjg.v21.i9.2725
Song TJ, 2014, GASTROINTEST ENDOSC, V80, P707, DOI 10.1016/j.gie.2014.05.327
Song TJ, 2012, WORLD J GASTROENTERO, V18, P4435, DOI 10.3748/wjg.v18.i32.4435
Umeda J, 2015, GASTROINTEST ENDOSC, V82, P390, DOI 10.1016/j.gie.2015.02.041
NR 20
TC 51
Z9 54
U1 0
U2 2
PU WILEY
PI HOBOKEN
PA 111 RIVER ST, HOBOKEN 07030-5774, NJ USA
SN 0815-9319
EI 1440-1746
J9 J GASTROEN HEPATOL
JI J. Gastroenterol. Hepatol.
PD MAR
PY 2017
VL 32
IS 3
BP 716
EP 720
DI 10.1111/jgh.13489
PG 5
WC Gastroenterology & Hepatology
WE Science Citation Index Expanded (SCI-EXPANDED)
SC Gastroenterology & Hepatology
GA EP3JI
UT WOS:000397278200028
PM 27420770
DA 2026-05-09
ER

PT J
AU Rai, P
Harish, K
Majeed, A
Goel, A
AF Rai, Praveer
Harish, K.
Majeed, Abdul

Goel, Amit
TI EUS-guided drainage of pancreatic fluid collection, using a modified technique of cystotome alone without a FNA needle
SO SAUDI JOURNAL OF GASTROENTEROLOGY
LA English
DT Article
DE Cystotome; endoscopic ultrasound; fully covered self-expanding metal stent; lumen apposing metal stent; pancreatic fluid collection; pancreatic necrosis
ID EXPANDING METAL STENTS; WALLED-OFF NECROSIS; ENDOSCOPIC ULTRASOUND; TRANSMURAL DRAINAGE; SINGLE-STEP; PSEUDOCYST DRAINAGE
AB Background: Endoscopic ultrasound (EUS)-guided drainage for pancreatic fluid collection (PFC) involves puncture with a fine-needle aspiration (FNA) needle, followed by tract dilation involving exchange of multiple accessories, and finally deployment of stent. The procedure is time consuming and carries a risk of loss of wire access and hence technical failure. We used a modified technique with a 10-F cystotome alone instead of a FNA needle and dilators.

Methods: We retrospectively analysed records of consecutive patients who had undergone EUS-guided drainage of PFC using a modified technique, with puncture of PFC using a 10-Fcystotome, followed by passage of a guidewire through it into the PFC cavity and deployment of a biflanged, 2-cm-long, fully covered self-expanding metal stent over it. Technical and clinical success rates and procedure time were assessed.

Results: Forty-five patients underwent PFC drainage, median age was 35 (12-76), and 35 (77.8%) were males. The median (range) duration of symptoms was 125 (38-1080) days, while the median PFC size was 11.8 x 11 x 11 cm, and the follow-up period after stent removal was 111 +/- 72 (18-251) weeks. The procedure took 10 (8-12) min and had technical and clinical success rates of 100 and 97.8%, respectively. Minor complications occurred in six (13.3%) patients, while recurrence occurred in one.

Conclusion: EUS-guided drainage of PFC using a cystotome is a quick, effective and safe procedure. It may also be less expensive since it obviates the use of FNA needles and dilators, and is likely to be a useful alternative to the conventional technique.
C1 [Rai, Praveer; Harish, K.; Majeed, Abdul; Goel, Amit] Sanjay Gandhi Postgrad Inst Med Sci, Dept Gastroenterol, Lucknow 226014, Uttar Pradesh, India.
C3 Sanjay Gandhi Postgraduate Institute of Medical Sciences
RP Rai, P (corresponding author), Sanjay Gandhi Postgrad Inst Med Sci, Dept Gastroenterol, Lucknow 226014, Uttar Pradesh, India.
EM praveer_rai@yahoo.com
RI Goel, Amit/H-2822-2019
OI Goel, Amit/0000-0003-3525-9381
CR Ahlawat SK, 2006, J PANCREAS, V7, P616
Bang JY, 2017, DIGEST ENDOSC, V29, P83, DOI 10.1111/den.12681
Banks PA, 2013, GUT, V62, P102, DOI 10.1136/gutjnl-2012-302779
Baron TH, 2002, GASTROINTEST ENDOSC, V56, P7, DOI 10.1067/mge.2002.125106
BINMOELLER KF, 1995, GASTROINTEST ENDOSC, V42, P219, DOI 10.1016/S0016-5107(95)70095-1
Cahen D, 2005, ENDOSCOPY, V37, P977, DOI 10.1055/s-2005-870336
Chandran S, 2015, GASTROINTEST ENDOSC, V81, P127, DOI 10.1016/j.gie.2014.06.025

Gornals JB, 2013, SURG ENDOSC, V27, P1428, DOI 10.1007/s00464-012-2591-y
Heinzow HS, 2011, SCAND J GASTROENTERO, V46, P1004, DOI 10.3109/00365521.2011.571709
Lee BU, 2014, ENDOSCOPY, V46, P1078, DOI 10.1055/s-0034-1390871
Mangiavillano B, 2012, J DIGEST DIS, V13, P47, DOI 10.1111/j.1751-2980.2011.00547.x
Mukai S, 2015, ENDOSCOPY, V47, P47, DOI 10.1055/s-0034-1377966
Penn DE, 2012, GASTROINTEST ENDOSC, V76, P679, DOI 10.1016/j.gie.2012.04.457
Rinninella E, 2015, GASTROINTEST ENDOSC, V82, P1039, DOI 10.1016/j.gie.2015.04.006
Seewald S, 2009, GASTROINTEST ENDOSC, V69, pS13, DOI 10.1016/j.gie.2008.10.061
Siddiqui AA, 2017, GASTROINTEST ENDOSC, V85, P758, DOI 10.1016/j.gie.2016.08.014
Singhal S, 2013, CLIN ENDOSC, V46, P506, DOI 10.5946/ce.2013.46.5.506
Song TJ, 2014, CLIN ENDOSC, V47, P222, DOI 10.5946/ce.2014.47.3.222
Talreja JP, 2008, GASTROINTEST ENDOSC, V68, P1199, DOI 10.1016/j.gie.2008.06.015
Teoh AYB, 2016, WORLD J GASTRO ENDOS, V8, P310, DOI 10.4253/wjge.v8.i6.310
Teoh AYB, 2015, ENDOSC INT OPEN, V3, pE130, DOI 10.1055/s-0034-1390890
Varadarajulu Shyam, 2013, Gastrointest Endosc Clin N Am, V23, P863, DOI 10.1016/j.giec.2013.06.008
Varadarajulu S, 2011, J GASTROINTEST SURG, V15, P2080, DOI 10.1007/s11605-011-1621-8
NR 23
TC 2
Z9 2
U1 0
U2 1
PU WOLTERS KLUWER MEDKNOW PUBLICATIONS
PI MUMBAI
PA WOLTERS KLUWER INDIA PVT LTD , A-202, 2ND FLR, QUBE, C T S NO 1498A-2
VILLAGE MAROL, ANDHERI EAST, MUMBAI, Maharashtra, INDIA
SN 1319-3767
EI 1998-4049
J9 SAUDI J GASTROENTERO
JI Saudi J. Gastroenterol.
PD SEP-OCT
PY 2021
VL 27
IS 5
BP 283
EP 288
DI 10.4103/sjg.sjg_132_21
PG 6
WC Gastroenterology & Hepatology
WE Science Citation Index Expanded (SCI-EXPANDED)
SC Gastroenterology & Hepatology
GA WO8FV
UT WOS:000712684200004

PM 34677161
OA Green Submitted, gold
DA 2026-05-09
ER

PT J
AU Yasuda, I
Hanaoka, T
Takahashi, K
Araki, Y
Doi, S
Iwashita, T
Iwata, K
Mukai, T
AF Yasuda, Ichiro
Hanaoka, Tatsuyuki
Takahashi, Kosuke
Araki, Yasuhiro
Doi, Shinpei
Iwashita, Takuji
Iwata, Keisuke
Mukai, Tsuyoshi
TI Recent topics on endoscopic ultrasonography-guided celiac plexus
neurolysis
SO INTERNATIONAL JOURNAL OF GASTROINTESTINAL INTERVENTION
LA English
DT Review
DE Celiac plexus; Endoscopic ultrasonography; Neurolysis; Pancreatic cancer
ID ABDOMINAL-PAIN; GANGLIA NEUROLYSIS; PANCREATIC-CANCER; BLOCK;
GUIDELINES; MANAGEMENT; EFFICACY; INJECTION; RELIEF; SAFETY
AB Endoscopic ultrasonography-guided celiac plexus neurolysis (EUS-CPN) is a widely practiced technique. Three sets of guidelines have recently been published and this procedure has become a major EUS technique. However, there are still several unanswered questions. The purpose of this manuscript is to review the recent literature pertaining to EUS-CPN. Currently, the main indication of EUS-CPN is pancreatic cancer pain. It is also performed for patients with chronic pancreatitis, but the indication is controversial due to its limited efficacy and a high incidence of infectious complications. Various techniques, such as central and bilateral EUS-CPN, and EUS-guided direct celiac ganglia neurolysis (EUS-CGN) have been performed. However, the efficacies of these techniques remain controversial. Complications related to the procedures are generally not serious, but major adverse events, such as paraplegia and ischemic complications, have been reported. The impacts of EUS-CPN on survival have also been evaluated. Although increased survival was expected via improvements in the quality of life, data suggests that EUS-CPN related procedures, especially EUS-CGN, might reduce the survival time. However, precise mechanisms have not been elucidated. In addition to conventional techniques, new techniques, such as EUS-guided celiac ganglion radiofrequency ablation (EUS-RFA) and the use of highly viscous phenol-

glycerol, dexmedetomidine, and contrast-enhanced agents, have been introduced. However, these techniques are still in experimental stages. Additional studies need to be conducted to address these gaps in the literature. Copyright (C) 2020, Society of Gastrointestinal Intervention.
C1 [Yasuda, Ichiro; Hanaoka, Tatsuyuki; Takahashi, Kosuke; Araki, Yasuhiro] Univ Toyama, Dept Internal Med 3, 2630 Sugitani, Toyama 9300194, Japan.
[Doi, Shinpei] Teikyo Univ, Mizonokuchi Hosp, Dept Gastroenterol, Kawasaki, Kanagawa, Japan.
[Iwashita, Takuji] Gifu Univ, Dept Internal Med 1, Gifu, Japan.
[Iwata, Keisuke] Gifu Prefectural Med Ctr, Dept Gastroenterol, Gifu, Japan.
[Mukai, Tsuyoshi] Gifu Municipal Hosp, Dept Gastroenterol, Gifu, Japan.
C3 University of Toyama; Teikyo University; Gifu University; Gifu Municipal
Hospital
RP Yasuda, I (corresponding author), Univ Toyama, Dept Internal Med 3, 2630 Sugitani, Toyama 9300194, Japan.
EM yasudaic@med.u-toyama.ac.jp
RI araki, yasuhiro/Y-9452-2018; Yasuda, Ichiro/IUP-0131-2023
OI Yasuda, Ichiro/0000-0002-6888-0310; Iwata, Keisuke/0000-0003-0564-8669;
Mukai, Tsuyoshi/0000-0002-5198-9475; Takahashi,
Kosuke/0000-0003-2361-5043
CR Alhazzani W, 2015, WORLD J GASTRO ENDOS, V7, P563, DOI 10.4253/wjge.v7.i5.563
Ascunce G, 2011, GASTROINTEST ENDOSC, V73, P267, DOI 10.1016/j.gie.2010.10.029
Bang JY, 2019, GASTROINTEST ENDOSC, V89, P58, DOI 10.1016/j.gie.2018.08.005
Doi S, 2013, ENDOSCOPY, V45, P362, DOI 10.1055/s-0032-1326225
Facciorusso A, 2020, ENDOSC ULTRASOUND, V9, P238, DOI 10.4103/eus.eus_24_20
Facciorusso A, 2019, PANCREATOLOGY, V19, P866, DOI 10.1016/j.pan.2019.07.038
Facciorusso A, 2015, ENDOSC ULTRASOUND, V4, P342, DOI 10.4103/2303-9027.170428
Faigel DO, 1996, AM J GASTROENTEROL, V91, P1675
Fujii-Lau LL, 2015, GASTROINTEST ENDOSC, V82, P46, DOI 10.1016/j.gie.2014.12.036
Fusaroli P, 2016, ULTRASCHALL MED, V37, pE77, DOI 10.1055/s-0035-1553738
Ishiwatari H, 2015, GASTROINTEST ENDOSC, V81, P479, DOI 10.1016/j.gie.2014.10.031
Iwata K, 2011, DIGEST ENDOSC, V23, P140, DOI 10.1111/j.1443-1661.2010.01046.x
Kanno Y, 2020, GASTROINTEST ENDOSC, V92, P120, DOI 10.1016/j.gie.2020.01.011
Kappelle WFW, 2017, GASTROINTEST ENDOSC, V86, P655, DOI 10.1016/j.gie.2017.01.041
Kaufman M, 2010, J CLIN GASTROENTEROL, V44, P127, DOI 10.1097/MCG.0b013e3181bb854d
LeBlanc JK, 2011, GASTROINTEST ENDOSC, V74, P1300, DOI 10.1016/j.gie.2011.07.073
Levy MJ, 2008, AM J GASTROENTEROL, V103, P98, DOI 10.1111/j.1572-0241.2007.01607.x
Levy MJ, 2019, CLIN GASTROENTEROL H, V17, P728, DOI 10.1016/j.cgh.2018.08.040
Lu F, 2018, SUPPORT CARE CANCER, V26, P353, DOI 10.1007/s00520-017-3888-0
Manta R, 2016, SURG ENDOSC, V30, P1863, DOI 10.1007/s00464-015-4403-7
Minaga K, 2016, THER ADV GASTROENTER, V9, P483, DOI 10.1177/1756283X16644248
Minaga K, 2016, GASTROINTEST ENDOSC, V83, P1039, DOI 10.1016/j.gie.2015.10.044
Puli SR, 2009, DIGEST DIS SCI, V54, P2330, DOI 10.1007/s10620-008-0651-x
Sakamoto H, 2010, AM J GASTROENTEROL, V105, P2599, DOI 10.1038/ajg.2010.339
Saleh AAG, 2021, J GASTROINTEST CANC, V52, P682, DOI 10.1007/s12029-020-00449-1

Sey MSL, 2015, ENDOSC INT OPEN, V3, pE56, DOI 10.1055/s-0034-1377919
Téllez-Avila FI, 2013, ENDOSC ULTRASOUND, V2, P153, DOI [10.4103/2303-9027.117664, 10.7178/eus.06.007]
Teoh AYB, 2018, GUT, V67, P1209, DOI 10.1136/gutjnl-2017-314341
Wang L, 2020, EUR RADIOL, V30, P4514, DOI 10.1007/s00330-020-06705-z
Wiersema MJ, 1996, GASTROINTEST ENDOSC, V44, P656, DOI 10.1016/S0016-5107(96)70047-0
Wyse JM, 2017, ENDOSC ULTRASOUND, V6, P369, DOI 10.4103/eus.eus_97_17
Yasuda I, 2017, DIGEST ENDOSC, V29, P455, DOI 10.1111/den.12824
NR 32
TC 0
Z9 0
U1 0
U2 4
PU SOC GASTROINTESTINAL INTERVENTION
PI SEOUL
PA 12, HAKDONG-RO 31, GANGNAM-GU, SEOUL, 00000, SOUTH KOREA
SN 2636-0004
EI 2636-0012
J9 INT J GASTROINT INT
JI Int. J. Gastrointest. Interv.
PD OCT
PY 2020
VL 9
IS 4
BP 164
EP 169
DI 10.18528/ijgii200033
PG 6
WC Gastroenterology & Hepatology
WE Emerging Sources Citation Index (ESCI)
SC Gastroenterology & Hepatology
GA OJ3OQ
UT WOS:000583875100008
OA Green Submitted, gold
DA 2026-05-09
ER

PT J
AU Chantarojanasiri, T
Matsubara, S
Isayama, H
Nakai, Y
Takahara, N

Mizuno, S
Kogure, H
Hakuta, R
Ito, Y
Tada, M
Koike, K
AF Chantarojanasiri, Tanyaporn
Matsubara, Saburo
Isayama, Hiroyuki
Nakai, Yousuke
Takahara, Naminatsu
Mizuno, Suguru
Kogure, Hirofumi
Hakuta, Ryunosuke
Ito, Yukiko
Tada, Minoru
Koike, Kazuhiko
TI Feasibility of conversion of percutaneous cholecystostomy to internal
transmural endoscopic ultrasound-guided gallbladder drainage
SO SAUDI JOURNAL OF GASTROENTEROLOGY
LA English
DT Article
DE Cholecystitis; endoscopic ultrasound; gallbladder drainage; percutaneous
cholecystostomy
ID BILIARY OBSTRUCTION; ACUTE CHOLECYSTITIS; STENT PLACEMENT; METALLIC
STENT; CHOLECYSTECTOMY; VIDEOS; TRACT
AB Background/Aim: Percutaneous cholecystostomy [percutaneous transhepatic gallbladder drainage (PTGBD)] is the treatment of choice in surgically unfit patients with acute cholecystitis. However, PTGBD tube removal after symptoms resolution results in 41-46% recurrence. This study aims to demonstrate the feasibility of the conversion of PTGBD to transmural endoscopic ultrasound-guided gallbladder drainage (EUS-GBD) using plastic stents in patients unfit for cholecystectomy.

Patients and Methods: Patients who underwent internal transmural EUS-GBD as a conversion from PTGBD were reviewed. EUS-GBD was performed after the improvement of cholecystitis due to recurrent cholecystitis and PTGBD intolerance. One or two 7-Fr double pigtail plastic stent insertion with or without temporary endoscopic naso-gallbladder drainage (ENGBD) insertion was performed.

Results: Six patients (age 61-88), with three cases of acute cholecystitis after metallic biliary stenting and three cases of calculus cholecystitis, who underwent PTGBD were included. EUS-GBD was performed 10-63 days after PTGBD, using one plastic stent in five cases, two stents in one case, with temporary ENGBD in two cases. The technical success and clinical success were achieved and the PTGBD tubes were subsequently removed in all patients. All ENGBD tubes were removed within 5 days after insertion. Bile leak with peritonitis was demonstrated in one case, which was treated conservatively. No recurrent cholecystitis was seen during 3-26 months of follow-up.

Conclusion: The conversion of percutaneous cholecystostomy to internal transmural EUS-GBD with plastic stents is

feasible for patients unfit for cholecystectomy. However, more studies are still needed to confirm the results.

C1 [Chantarojanasiri, Tanyaporn; Matsubara, Saburo; Isayama, Hiroyuki; Nakai, Yousuke; Takahara, Naminatsu; Mizuno, Suguru; Kogure, Hirofumi; Tada, Minoru; Koike, Kazuhiko] Univ Tokyo, Grad Sch Med, Dept Gastroenterol, Tokyo, Japan.
[Matsubara, Saburo] Tokyo Metropolitan Police Hosp, Dept Gastroenterol, Tokyo, Japan.
[Hakuta, Ryunosuke; Ito, Yukiko] Japanese Red Cross Med Ctr, Dept Gastroenterol, Tokyo, Japan.
[Chantarojanasiri, Tanyaporn] Police Gen Hosp, Dept Internal Med, Bangkok, Thailand.

C3 University of Tokyo; University of Tokyo; Japanese Red Cross Medical
Center

RP Isayama, H (corresponding author), 7-3-1 Hongo,Bunkyo Ku, Tokyo 1138655, Japan.

EM isayama-tky@umin.ac.jp

RI Chantarojanasiri, Tanyaporn/AAM-8488-2020; Nakai, Yousuke/H-2964-2019;
Mizuno, Suguru/AAF-8867-2019; Isayama, Hiroyuki/AER-9916-2022; Takahara,
Naminatsu/K-8497-2019

CR DAGOSTINO HB, 1991, RADIOLOGY, V181, P675, DOI 10.1148/radiology.181.3.1947080
Ha JPY, 2008, HEPATO-GASTROENTEROL, V55, P1497
Hatjidakis AA, 1998, CARDIOVASC INTER RAD, V21, P36, DOI 10.1007/s002709900208
Isayama H, 2006, CLIN GASTROENTEROL H, V4, P1148, DOI 10.1016/j.cgh.2006.06.004
Itoi T, 2015, GASTROINTEST ENDOSC, V81, P111, DOI 10.1016/j.gie.2014.09.046
Itoi T, 2011, J HEPATO-BIL-PAN SCI, V18, P282, DOI 10.1007/s00534-010-0310-4
Jang JW, 2012, GASTROENTEROLOGY, V142, P805, DOI 10.1053/j.gastro.2011.12.051
Kwan V, 2007, GASTROINTEST ENDOSC, V66, P582, DOI 10.1016/j.gie.2007.02.065
Law R, 2016, CLIN GASTROENTEROL H, V14, P476, DOI 10.1016/j.cgh.2015.10.026
McKay A, 2012, SURG ENDOSC, V26, P1343, DOI 10.1007/s00464-011-2035-0
Miura F, 2013, J HEPATO-BIL-PAN SCI, V20, P47, DOI 10.1007/s00534-012-0563-1
Nakai Y, 2017, ENDOSCOPIC ULTRASOUN
Nakai Y, 2014, J GASTROEN HEPATOL, V29, P1557, DOI 10.1111/jgh.12582
Song TJ, 2010, GASTROINTEST ENDOSC, V71, P634, DOI 10.1016/j.gie.2009.11.024
Yamashita Y, 2013, J HEPATO-BIL-PAN SCI, V20, P89, DOI 10.1007/s00534-012-0567-x

NR 15

TC 12

Z9 12

U1 0

U2 2

PU MEDKNOW PUBLICATIONS & MEDIA PVT LTD

PI MUMBAI

PA B-9, KANARA BUSINESS CENTRE, OFF LINK RD, GHAKTOPAR-E, MUMBAI, 400075,
INDIA

SN 1319-3767

EI 1998-4049

J9 SAUDI J GASTROENTERO

JI Saudi J. Gastroenterol.

PD NOV-DEC

PY 2017
VL 23
IS 6
BP 218
EP 222
DI 10.4103/sjg.SJG_115_17
PG 5
WC Gastroenterology & Hepatology
WE Science Citation Index Expanded (SCI-EXPANDED)
SC Gastroenterology & Hepatology
GA FP3JE
UT WOS:000417513700003
OA Green Submitted, gold
DA 2026-05-09
ER

PT J
AU Minaga, K
Kitano, M
Sakamoto, H
Miyata, T
Imai, H
Yamao, K
Kamata, K
Omoto, S
Kadosaka, K
Sakurai, T
Nishida, N
Chiba, Y
Kudo, M
AF Minaga, Kosuke
Kitano, Masayuki
Sakamoto, Hiroki
Miyata, Takeshi
Imai, Hajime
Yamao, Kentaro
Kamata, Ken
Omoto, Shunsuke
Kadosaka, Kumpei
Sakurai, Toshiharu
Nishida, Naoshi
Chiba, Yasutaka

Kudo, Masatoshi
TI Predictors of pain response in patients undergoing endoscopic
ultrasound-guided neurolysis for abdominal pain caused by pancreatic
cancer
SO THERAPEUTIC ADVANCES IN GASTROENTEROLOGY
LA English
DT Article
DE cancer-associated pain; celiac plexus neurolysis; endoscopic ultrasound;
EUS-guided neurolysis; pancreatic cancer; predictor
ID CELIAC PLEXUS NEUROLYSIS; SPINAL-CORD INFARCTION; GANGLIA NEUROLYSIS;
BLOCK; ULTRASONOGRAPHY; EFFICACY; RELIEF; VISUALIZATION; MANAGEMENT;
CARCINOMA
AB Background: Interventional endoscopic ultrasound (EUS)-guided procedures such as EUS-guided celiac ganglia neurolysis (EUS-CGN) and EUS-guided broad plexus neurolysis (EUS-BPN) were developed to treat abdominal cancer-associated pain; however, these procedures are not always effective. The aim of this study was to explore predictors of pain response in EUS-guided neurolysis for pancreatic cancer-associated pain.

Methods: This was a retrospective analysis of prospectively collected data of 112 consecutive patients who underwent EUS-BPN in our institution. EUS-CGN was added in cases of visible celiac ganglia. The neurolytic-spread area was divided into six sections and evaluated by post-procedural computed tomography scanning. Pain intensity was assessed using a visual analog scale (VAS), and a decrease in VAS scores by 3 points after neurolysis was considered a good pain response. Univariable and multivariable logistic regression analyses were performed to explore predictors of pain response at 1 and 4 weeks, and complications.

Results: A good pain response was obtained in 77.7% and 67.9% of patients at 1 and 4 weeks, respectively. In the multivariable analysis of these patients, the combination method (EUS-BPN plus CGN) was a significant positive predictive factor at 1 week (odds ratio = 3.69, $p = 0.017$) and 4 weeks (odds ratio = 6.37, $p = 0.043$). The numbers of neurolytic/contrast spread areas (mean SD) were 4.98 +/- 1.08 and 4.15 +/- 1.12 in patients treated with the combination method and single method, respectively ($p < 0.001$). There was no significant predictor of complications.

Conclusions: EUS-BPN in combination with EUS-CGN was a predictor of a good pain response in EUS-guided neurolysis for pancreatic cancer-related pain. The larger number of neurolytic/contrast spread areas may lead to better outcomes in patients receiving combination treatment.
C1 [Minaga, Kosuke; Kitano, Masayuki; Sakamoto, Hiroki; Miyata, Takeshi; Imai, Hajime; Yamao, Kentaro; Kamata, Ken; Omoto, Shunsuke; Kadosaka, Kumpei; Sakurai, Toshiharu; Nishida, Naoshi; Kudo, Masatoshi] Kinki Univ, Dept Gastroenterol & Hepatol, Fac Med, 377-2 Ohno Higashi, Osaka 5898511, Japan.
[Chiba, Yasutaka] Kinki Univ Hosp, Clin Res Ctr, Osaka, Japan.
C3 Kindai University (Kinki University); Kindai University (Kinki
University)
RP Kitano, M (corresponding author), Kinki Univ, Dept Gastroenterol & Hepatol, Fac Med, 377-2 Ohno Higashi, Osaka 5898511, Japan.
EM m-kitano@med.kindai.ac.jp
RI Kudo, Masatoshi/AAA-9744-2019; Minaga, Kosuke/AAD-2107-2020; Nishida,
Naoshi/AFV-2598-2022
OI Kudo, Masatoshi/0000-0002-4102-3474;

FU Japan Society for the Promotion of Science [22590764, 25461035];
Grants-in-Aid for Scientific Research [25461035] Funding Source: KAKEN
FX The present study was supported by grants from the Japan Society for the
Promotion of Science (grant numbers 22590764 and 25461035).
CR Alvarez-Sanchez MV, 2014, SURG ENDOSC, V28, P712, DOI 10.1007/s00464-013-3260-5
Ascunce G, 2011, GASTROINTEST ENDOSC, V73, P267, DOI 10.1016/j.gie.2010.10.029
Collins D, 2006, ENDOSCOPY, V38, P935, DOI 10.1055/s-2006-944734
Doi S, 2013, ENDOSCOPY, V45, P362, DOI 10.1055/s-0032-1326225
Fujii L, 2012, ENDOSCOPY, V44, pE265, DOI 10.1055/s-0032-1309708
Gerke H, 2006, GASTROINTEST ENDOSC, V64, P35, DOI 10.1016/j.gie.2006.01.071
Gleeson FC, 2007, ENDOSCOPY, V39, P620, DOI 10.1055/s-2007-966337
Gunaratnam NT, 2001, GASTROINTEST ENDOSC, V54, P316, DOI 10.1067/mge.2001.117515
Ha Tae-In, 2008, Korean Journal of Internal Medicine, V23, P5, DOI 10.3904/kjim.2008.23.1.5
Iwata K, 2011, DIGEST ENDOSC, V23, P140, DOI 10.1111/j.1443-1661.2010.01046.x
Kaufman M, 2010, J CLIN GASTROENTEROL, V44, P127, DOI 10.1097/MCG.0b013e3181bb854d
Kitoh Takeshi, 2005, J Anesth, V19, P328, DOI 10.1007/s00540-005-0342-0
Leblanc Julia K, 2013, Diagn Ther Endosc, V2013, P327036, DOI 10.1155/2013/327036
LeBlanc JK, 2011, GASTROINTEST ENDOSC, V74, P1300, DOI 10.1016/j.gie.2011.07.073
Levy MJ, 2008, AM J GASTROENTEROL, V103, P98, DOI 10.1111/j.1572-0241.2007.01607.x
Luz LP, 2014, ENDOSC ULTRASOUND, V3, P213, DOI 10.4103/2303-9027.144515
Michaels AJ, 2007, WORLD J GASTROENTERO, V13, P3575, DOI 10.3748/wjg.v13.i26.3575
Mittal MK, 2012, NEUROLOGY, V78, pE57, DOI 10.1212/WNL.0b013e318248df51
O'Toole TM, 2009, ENDOSCOPY, V41, P593, DOI 10.1055/s-0029-1214868
Penman ID, 2009, GASTROINTEST ENDOSC, V69, pS28, DOI 10.1016/j.gie.2008.11.004
Puli SR, 2009, DIGEST DIS SCI, V54, P2330, DOI 10.1007/s10620-008-0651-x
Rykowski JJ, 2000, ANESTHESIOLOGY, V92, P347, DOI 10.1097/00000542-200002000-00014
Sahai AV, 2009, AM J GASTROENTEROL, V104, P326, DOI 10.1038/ajg.2008.64
Sakamoto H., 2006, DIGEST ENDOSC, V18, P206
Sakamoto H, 2011, PANCREATOLOGY, V11, P52, DOI 10.1159/000323513
Sakamoto H, 2010, AM J GASTROENTEROL, V105, P2599, DOI 10.1038/ajg.2010.339
Seicean A, 2014, WORLD J GASTROENTERO, V20, P110, DOI 10.3748/wjg.v20.i1.110
Seicean A, 2013, J GASTROINTEST LIVER, V22, P59
Soweid AM, 2010, WORLD J GASTRO ENDOS, V2, P228, DOI 10.4253/wjge.v2.i6.228
Wang KX, 2012, GASTROINTEST ENDOSC, V76, P945, DOI 10.1016/j.gie.2012.05.032
Wiersema MJ, 1996, GASTROINTEST ENDOSC, V44, P656, DOI 10.1016/S0016-5107(96)70047-0
Wyse JM, 2011, J CLIN ONCOL, V29, P3541, DOI 10.1200/JCO.2010.32.2750
Zou XP, 2012, PANCREAS, V41, P655, DOI 10.1097/MPA.0b013e31823d06a9
NR 33
TC 19
Z9 22
U1 0
U2 2

PU SAGE PUBLICATIONS LTD
PI LONDON
PA 1 OLIVERS YARD, 55 CITY ROAD, LONDON EC1Y 1SP, ENGLAND
SN 1756-283X
EI 1756-2848
J9 THER ADV GASTROENTER
JI Ther. Adv. Gastroenterol.
PD JUL
PY 2016
VL 9
IS 4
BP 483
EP 494
DI 10.1177/1756283X16644248
PG 12
WC Gastroenterology & Hepatology
WE Science Citation Index Expanded (SCI-EXPANDED)
SC Gastroenterology & Hepatology
GA DP9CG
UT WOS:000378793700007
PM 27366217
OA Green Submitted, Bronze
DA 2026-05-09
ER

PT J
AU Gökce, E
Devisscher, L
Rashidian, N
Palmeri, E
Hindryckx, P
AF Gokce, Emine
Devisscher, Lindsey
Rashidian, Niki
Palmeri, Enrico
Hindryckx, Pieter
TI Lumen-apposing metal stents for anastomosis creation throughout the
gastrointestinal tract: A large single-center experience
SO DEN OPEN
LA English
DT Article
DE EDGE; EDGI; gastric outlet obstruction; lumen-apposing metal stent;

Roux-en-Y gastric bypass

AB Objectives: The introduction of lumen-apposing metal stents (LAMSs) has revolutionized the field of therapeutic endoscopic ultrasound. This study aims to evaluate the efficacy and safety of LAMS in creating an endoscopic ultrasound-guided anastomosis between two segments of the gastrointestinal (GI) tract. Methods: Data from all consecutive LAMS procedures for anastomosis creation between two segments of the GI, conducted between October 2019 and February 2024, were retrospectively analyzed for technical success (defined as correct deployment of the LAMS in the target), clinical success (defined as achievement of the intended clinical goal), and adverse events. Results: A total of 145 LAMS procedures were performed in 136 patients. Indications for LAMS procedures included the need for endoscopic access to or reversal of surgically excluded segments of the GI tract (n = 73, 50.3%), and the alleviation of any GI outflow obstruction (n = 72, 49.7%). The overall technical and clinical success rates were very high (97.2% and 95.2%, respectively). Adverse events were observed in 20/145 (13.8%) cases, including 11 (7.6%) minor events (AGREE <3) and nine (6.2%) major events (AGREE >= 3). Major events included stent migration (n = 1), persisting fistula (n = 3), and bleeding (n = 4). All adverse events were successfully managed, and there were no procedure-related deaths. Loss of LAMS patency occurred in 4/145 (2.8%) cases and could be endoscopically managed in all cases. Conclusions: The creation of anastomoses with LAMS between two segments of the GI tract appears to be effective and safe, with a low reintervention rate due to loss of LAMS patency.

C1 [Gokce, Emine; Palmeri, Enrico; Hindryckx, Pieter] Ghent Univ Hosp, Dept Gastroenterol & Hepatol, Ghent, Belgium.

[Gokce, Emine; Devisscher, Lindsey] Univ Ghent, Dept Basic & Appl Med Sci, Gut Liver Immunopharmacol Unit, Ghent, Belgium.

[Gokce, Emine; Devisscher, Lindsey] Univ Ghent, Dept Liver Res Ctr Ghent, Ghent, Belgium.

[Rashidian, Niki] Ghent Univ Hosp, Dept HPB Surg & Liver Transplantat, Ghent, Belgium.

C3 Ghent University; Ghent University Hospital; Ghent University; Ghent University; Ghent University; Ghent University Hospital

RP Gökce, E (corresponding author), Univ Ghent, Univ Hosp Ghent, Dept Gastroenterol & Hepatol, Corneel Heymanslaan 10, B-9000 Ghent, Belgium.

EM Emine.gokce@ugent.be

RI Devisscher, Lindsey/AAD-5969-2021

OI Palmeri, Enrico/0009-0001-8300-2567

CR Bronswijk M, 2024, Dig Endosc. Epub

Carbajo AY, 2020, J CLIN GASTROENTEROL, V54, P1, DOI 10.1097/MCG.0000000000001262

Gutierrez OIB, 2018, ENDOSCOPY, V50, P891, DOI 10.1055/s-0044-102254

Hindryckx P, 2021, SURG ENDOSC, V35, P6013, DOI 10.1007/s00464-020-08090-6

Iqbal U, 2020, ENDOSC ULTRASOUND, V9, P16, DOI 10.4103/eus.eus_70_19

James TW, 2020, GASTROINTEST ENDOSC, V91, P537, DOI 10.1016/j.gie.2019.11.017

James TW, 2019, OBES SURG, V29, P451, DOI 10.1007/s11695-018-3531-2

Kastelijn JB, 2020, ENDOSC INT OPEN, V08, pE1194, DOI 10.1055/a-1214-5659

Kedia P, 2019, J CLIN GASTROENTEROL, V53, P304, DOI 10.1097/MCG.0000000000001037

McCarty Thomas R, 2019, Endosc Int Open, V7, pE1474, DOI [10.1055/a-0996-8178, 10.1055/a-0996-8178]

Miller C, 2023, ENDOSC INT OPEN, V11, pE660, DOI 10.1055/a-2098-2570

Nass KJ, 2022, GASTROINTEST ENDOSC, V95, P1078, DOI 10.1016/j.gie.2021.11.038

Ouazzani S, 2023, ENDOSCOPY, V55, pE414, DOI 10.1055/a-2007-1952

Perez-Miranda M, 2017, J CLIN GASTROENTEROL, V51, P896, DOI 10.1097/MCG.0000000000000887

Rimba M., 2023, Diagnostics, V13
Sharma P, 2020, WORLD J GASTROENTERO, V26, P2715, DOI 10.3748/wjg.v26.i21.2715
Stefanovic S, 2024, AM J GASTROENTEROL, V119, P671, DOI 10.14309/ajg.0000000000002571
Stefanovic S, 2023, J CLIN MED, V12, DOI 10.3390/jcm12031037
van der Merwe SW, 2022, ENDOSCOPY, V54, P185, DOI 10.1055/a-1717-1391
Wang TJ, 2021, SURG ENDOSC, V35, P4469, DOI 10.1007/s00464-020-07952-3
Wang TJ, 2019, SURG ENDOSC, V33, P2024, DOI 10.1007/s00464-019-06715-z
NR 21
TC 4
Z9 4
U1 0
U2 3
PU WILEY
PI HOBOKEN
PA 111 RIVER ST, HOBOKEN 07030-5774, NJ USA
SN 2692-4609
J9 DEN OPEN
JI DEN Open
PD OCT 12
PY 2024
VL 5
IS 1
AR e419
DI 10.1002/deo2.419
PG 7
WC Gastroenterology & Hepatology
WE Emerging Sources Citation Index (ESCI)
SC Gastroenterology & Hepatology
GA J3P2Z
UT WOS:001336210100001
PM 39398259
OA Green Submitted, gold
DA 2026-05-09
ER

PT J
AU Chin, JYL
Seleq, S
Weilert, F
AF Chin, Jerry Yung-Lun
Seleq, Samir
Weilert, Frank

TI Safety and outcomes of endoscopic ultrasound-guided drainage for malignant biliary obstruction using cautery-enabled lumen-apposing metal stent
SO ENDOSCOPY INTERNATIONAL OPEN
LA English
DT Article
ID DUODENAL OBSTRUCTION; CHOLEDOCHODUODENOSTOMY; MULTICENTER; COMPLICATIONS; PALLIATION; PLACEMENT; INSERTION; FAILS; ERCP
AB Background and study aims There is increasing evidence to suggest that EUS-guided biliary drainage (EUS-BD) is a safe and effective treatment alternative for patients with malignant biliary obstructions (MBOs) after failed endoscopic retrograde cholangiopancreatography. Patients and methods We performed a retrospective analysis of data prospectively collected from patients with MBO who underwent choledochoduodenostomy (CDS) or gallbladder drainage (GBD) between August 2016 and June 2020 using the electrocautery-enabled lumen-apposing metal stents (ECE-LAMS). The primary endpoint was technical and clinical success. Secondary endpoints were adverse events (AEs) and reinterventions.

Results A total of 60 patients were included in the study, with 56 CDS and 4 GBD. Median age was 76 years with 57 % male (34/60). The most common indication for EUS-BD was pancreatic cancer (78%). Technical success was achieved in 100% of cases, with a clinical success rate of 91.7%. Mean total bilirubin pre-procedure was 202umol/L (normal < 20 umol/L) and 63.8umol/L post procedure (P<.001). Twenty-one patients had bilirubin recorded at 2 weeks post EUS-BD with 20 of 21 patients demonstrating >50% reduction in bilirubin (mean bilirubin reduction 75%). AEs occurred in 12 of 60 patients (20%), all of which were mild. The reintervention rate was 11.7% (7/60). Stent occlusion occurred in 10 of 60 patients (16.7%) with a mean time to stent occlusion of 46.2 days (3-133). Stent patency of 83.3% was observed with a mean follow up of 7.9 months.

Conclusion EUS-CDS and GBD using ECE-LAMS are effective EUS-based techniques for managing patients with MBO. AEs are usually mild and resolved by reintervention.
C1 [Chin, Jerry Yung-Lun; Seleq, Samir; Weilert, Frank] Waikato Hosp, Dept Gastroenterol & Hepatol, Pembroke St, Hamilton 3240, New Zealand.
C3 Waikato Hospital
RP Weilert, F (corresponding author), Waikato Hosp, Dept Gastroenterol & Hepatol, Pembroke St, Hamilton 3240, New Zealand.
EM Frank.Weilert@waikatodhb.health.nz
OI Chin, Jerry Yung-Lun/0000-0003-4089-7345
CR Anderloni A, 2019, GASTROINTEST ENDOSC, V89, P69, DOI [10.1016/j.gie.2018.04.1746, 10.1016/j.gie.2018.08.047]
Anderloni A, 2018, AM J GASTROENTEROL, V113, P644, DOI 10.1038/s41395-018-0040-9
Anderloni A, 2016, ENDOSCOPY, V48, pE321, DOI 10.1055/s-0042-117425
Artifon ELA, 2006, AM J GASTROENTEROL, V101, P2031, DOI 10.1111/j.1572-0241.2006.00764.x
Bliss LA, 2016, HPB, V18, P671, DOI 10.1016/j.hpb.2016.05.008
El Chafic AH, 2019, DIGEST DIS SCI, V64, P3321, DOI 10.1007/s10620-019-05688-2
Fabbri C, 2019, DIGEST LIVER DIS, V51, pE126, DOI 10.1016/S1590-8658(19)30236-1
Giovannini M, 2001, ENDOSCOPY, V33, P898, DOI 10.1055/s-2001-17324
Han SY, 2019, SCI REP-UK, V9, DOI 10.1038/s41598-019-52993-x
Imai H, 2016, GASTROINTEST ENDOSC, V84, P147, DOI 10.1016/j.gie.2015.12.024

Itoi T, 2014, GASTROINTEST ENDOSC, V79, P715, DOI 10.1016/j.gie.2013.11.021
Jacques J, 2020, GASTROINTEST ENDOSC, V92, P134, DOI 10.1016/j.gie.2020.01.055
Jacques J, 2019, ENDOSCOPY, V51, P540, DOI 10.1055/a-0735-9137
Kadaba RS, 2017, ANN ROY COLL SURG, V99, P210, DOI 10.1308/rcsann.2016.0293
Khan MA, 2016, DIGEST DIS SCI, V61, P684, DOI 10.1007/s10620-015-3933-0
Khashab MA, 2014, ENDOSCOPY, V46, P251, DOI 10.1055/s-0033-1359214
Lee JJ, 2017, SCAND J GASTROENTERO, V52, P1258, DOI 10.1080/00365521.2017.1349833
Miller CS, 2019, ENDOSC INT OPEN, V7, pE1563, DOI 10.1055/a-0998-8129
Mutignani M, 2007, ENDOSCOPY, V39, P440, DOI 10.1055/s-2007-966327
Nam K, 2018, ENDOSC ULTRASOUND, V7, P48, DOI 10.4103/eus.eus_100_17
Nennstiel S, 2015, J CLIN GASTROENTEROL, V49, P764, DOI 10.1097/MCG.0000000000000275
Paik WH, 2018, AM J GASTROENTEROL, V113, P987, DOI 10.1038/s41395-018-0122-8
Sassatelli R, 2020, DIGEST LIVER DIS, V52, P57, DOI 10.1016/j.dld.2019.07.009
Sawas T, 2015, GASTROINTEST ENDOSC, V82, P256, DOI 10.1016/j.gie.2015.03.1980
Sharaiha RZ, 2017, GASTROINTEST ENDOSC, V85, P904, DOI 10.1016/j.gie.2016.12.023
Staub J, 2018, GASTROINTEST ENDOSC, V87, P1499, DOI 10.1016/j.gie.2018.01.040
Tsuchiya T, 2018, GASTROINTEST ENDOSC, V87, P1138, DOI 10.1016/j.gie.2017.08.017
Wang KX, 2016, GASTROINTEST ENDOSC, V83, P1218, DOI 10.1016/j.gie.2015.10.033
Zhao XQ, 2015, DIGEST ENDOSC, V27, P137, DOI 10.1111/den.12320
NR 29
TC 31
Z9 34
U1 0
U2 1
PU GEORG THIEME VERLAG KG
PI STUTTGART
PA RUDIGERSTR 14, D-70469 STUTTGART, GERMANY
SN 2364-3722
EI 2196-9736
J9 ENDOSC INT OPEN
JI Endosc. Int. Open
PD NOV
PY 2020
VL 08
IS 11
BP E1633
EP E1638
DI 10.1055/a-1236-3217
PG 6
WC Gastroenterology & Hepatology; Surgery
WE Emerging Sources Citation Index (ESCI)
SC Gastroenterology & Hepatology; Surgery

GA OF2FV
UT WOS:000581031400009
PM 33140019
OA Green Submitted, gold
DA 2026-05-09
ER

PT J
AU Siripun, A
Sripongpun, P
Ovartlarnporn, B
AF Siripun, Aroon
Sripongpun, Pimsiri
Ovartlarnporn, Bancha
TI Endoscopic ultrasound-guided biliary intervention in patients with
surgically altered anatomy
SO WORLD JOURNAL OF GASTROINTESTINAL ENDOSCOPY
LA English
DT Review
DE Endoscopic ultrasound-guided anterograde approach; Endoscopic
ultrasound-guided biliary drainage; Endoscopic ultrasound-guided
choledochoduodenostomy; Endoscopic ultrasound-guided
hepaticogastrostomy; Endoscopic ultrasound-rendezvous technique;
Surgically altered anatomy; Overtube-assisted enteroscopy-endoscopic
retrograde cholangiopancreatography
ID EN-Y GASTROJEJUNOSTOMY; FAILED ERCP; GASTRIC BYPASS; RETROGRADE
CHOLANGIOPANCREATOGRAPHY; TRANSHEPATIC CHOLANGIOGRAPHY; CASE SERIES;
DRAINAGE; EUS; OBSTRUCTION; EXPERIENCE
AB AIM: To evaluate the efficacy of endoscopic ultrasound guided biliary drainage (EUS-BD) in patients with surgically altered anatomies.

METHODS: We performed a search of the MEDLINE database for studies published between 2001 to July 2014 reporting on EUS-BD in patients with surgically altered anatomy using the terms "EUS drainage" and "altered anatomy". All relevant articles were accessed in full text. A manual search of the reference lists of relevant retrieved articles was also performed. Only full-text English papers were included. Data regarding age, gender, diagnosis, method of EUS-BD and intervention, type of altered anatomy, technical success, clinical success, and complications were extracted and collected. Anatomic alterations were categorized as: group 1, Billroth.; group 2, Billroth.; group 4, Roux-en-Y with gastric bypass; and group 3, all other types.

RESULTS: Twenty three articles identified in the literature search, three reports were from the same group with different numbers of cases. In total, 101 cases of EUS-BD in patients with altered anatomy were identified. Twenty-seven cases had no information and were excluded. Seventy four cases were included for analysis. Data of EUS-BD in patients categorized as group 1, 2 and 4 were limited with 2, 3 and 6 cases with EUS-BD done respectively. Thirty four cases with EUS-BD were reported in group 3. The pooled technical success, clinical success, and complication rates of all reports with

available data were 89.18%, 91.07% and 17.5%, respectively. The results are similar to the reported outcomes of EUS-BD in general, however, with limited data of EUS-BD in patients with altered anatomy rendered it difficult to draw a firm conclusion.

CONCLUSION: EUS-BD may be an option for patients with altered anatomy after a failed endoscopic-retrograde-cholangiography in centers with expertise in EUS-BD procedures in a research setting.
C1 [Siripun, Aroon; Ovartlarnporn, Bancha] Prince Songkla Univ, NKC Inst Gastroenterol & Hepatol, Fac Med, Hat Yai 90110, Songkhla, Thailand.

[Sripongpun, Pimsiri] Prince Songkla Univ, Dept Internal Med, Fac Med, Hat Yai 90110, Songkhla, Thailand.

C3 Prince of Songkla University; Prince of Songkla University

RP Ovartlarnporn, B (corresponding author), Prince Songkla Univ, NKC Inst Gastroenterol & Hepatol, Fac Med, 15 Karnjanavanich Rd, Hat Yai 90110, Songkhla, Thailand.

EM mrovart@ji-net.com

RI Sripongpun, Pimsiri/GMW-7585-2022
CR Artifon ELA, 2012, J CLIN GASTROENTEROL, V46, P768, DOI 10.1097/MCG.0b013e31825f264c

Artifon ELA, 2011, J PANCREAS, V12, P610

Attasaranya S, 2012, GASTROENT RES PRACT, V2012, DOI 10.1155/2012/680753

Bapaye A, 2013, UNITED EUR GASTROENT, V1, P285, DOI 10.1177/2050640613490928

Baron Todd H Sr, 2013, Gastrointest Endosc Clin N Am, V23, P295, DOI 10.1016/j.giec.2013.01.001

Bertin PM, 2011, SURG ENDOSC, V25, P2592, DOI 10.1007/s00464-011-1593-5

Binmoeller KF, 2011, J HEPATO-BIL-PAN SCI, V18, P319, DOI 10.1007/s00534-010-0358-1

Binmoeller KF, 2010, GASTROINTEST ENDOSC, V71, pAB349, DOI 10.1016/j.gie.2010.03.932

Bories E, 2007, ENDOSCOPY, V39, P287, DOI 10.1055/s-2007-966212

Burmester E, 2003, GASTROINTEST ENDOSC, V57, P246, DOI 10.1067/mge.2003.85

Chang KJ, 2011, GASTROINTEST ENDOSC, V73, pAB326

Costamagna G, 2013, ANN GASTROENTEROL, V26, P37

Desai S V., 2011, Gastrointest Endosc, V73, pAB122, DOI [10.1016/j.gie.2011.03.039, DOI 10.1016/J.GIE.2011.03.039]

Devière J, 2014, GASTROENTEROLOGY, V147, P385, DOI 10.1053/j.gastro.2014.04.043

Freeman ML, 2005, GASTROINTEST ENDOSC, V61, P112, DOI 10.1016/S0016-5107(04)02463-0

Fusaroli P, 2013, ENDOSC ULTRASOUND, V2, P64, DOI 10.4103/2303-9027.117689

Giovannini M, 2001, ENDOSCOPY, V33, P898, DOI 10.1055/s-2001-17324

Hara K, 2013, ENDOSCOPY, V45, P392, DOI 10.1055/s-0032-1326076

Hintze RE, 1997, ENDOSCOPY, V29, P74, DOI 10.1055/s-2007-1004078

Horaguchi J, 2009, DIGEST ENDOSC, V21, P239, DOI 10.1111/j.1443-1661.2009.00899.x

Imazu H, 2012, ENDOSCOPY, V44, P99, DOI 10.1055/s-0031-1291445

Iwashita T, 2012, ENDOSCOPY, V44, P60, DOI 10.1055/s-0030-1256871

Iwashita T, 2014, CLIN J GASTROENTEROL, V7, P94, DOI 10.1007/s12328-014-0467-5

Iwashita T, 2013, DIGEST DIS SCI, V58, P2417, DOI 10.1007/s10620-013-2645-6

Kahaleh M, 2006, GASTROINTEST ENDOSC, V64, P52, DOI 10.1016/j.gie.2006.01.063

Kahaleh M, 2005, GASTROINTEST ENDOSC, V61, P307, DOI 10.1016/S0016-5107(04)02585-4

Kahaleh M, 2013, WORLD J GASTROENTERO, V19, P1372, DOI 10.3748/wjg.v19.i9.1372

Kamiya S, 2004, ANN SURG, V239, P510, DOI 10.1097/01.sla.0000118594.23874.89

Kawakubo K, 2014, J HEPATO-BIL-PAN SCI, V21, P328, DOI 10.1002/jhbp.27
Kedia P, 2013, CLIN ENDOSC, V46, P543, DOI 10.5946/ce.2013.46.5.543
Khashab MA, 2015, DIGEST DIS SCI, V60, P557, DOI 10.1007/s10620-014-3300-6
Khashab MA, 2013, GASTROINTEST ENDOSC, V78, P734, DOI 10.1016/j.gie.2013.05.013
Kühn JP, 2010, AM J ROENTGENOL, V195, P851, DOI 10.2214/AJR.09.3461
Lee TH, 2014, WORLD J GASTROENTERO, V20, P5859, DOI 10.3748/wjg.v20.i19.5859
Lopes TL, 2009, GASTROINTEST ENDOSC, V70, P1254, DOI 10.1016/j.gie.2009.07.035
Ma J, 2011, ENDOSCOPY, V43, pE102, DOI 10.1055/s-0030-1256104
Maranki J, 2009, ENDOSCOPY, V41, P532, DOI 10.1055/s-0029-1214712
Nguyen-Tang T, 2010, ENDOSCOPY, V42, P232, DOI 10.1055/s-0029-1243858
Ogura T, 2014, ENDOSCOPY, V46, P416, DOI 10.1055/s-0034-1365020
Park DH, 2013, GASTROINTEST ENDOSC, V78, P91, DOI 10.1016/j.gie.2013.01.042
Park DH, 2012, GASTROINTEST ENDOSC, V75, P692, DOI 10.1016/j.gie.2011.04.013
Park DH, 2011, GASTROINTEST ENDOSC, V74, P1276, DOI 10.1016/j.gie.2011.07.054
Prachayakul V, 2013, WORLD J GASTROENTERO, V19, P4758, DOI 10.3748/wjg.v19.i29.4758
Püspök A, 2005, AM J GASTROENTEROL, V100, P1743, DOI 10.1111/j.1572-0241.2005.41806.x
Sarkaria S, 2013, GUT LIVER, V7, P129, DOI 10.5009/gnl.2013.7.2.129
Schreiner MA, 2012, GASTROINTEST ENDOSC, V75, P748, DOI 10.1016/j.gie.2011.11.019
Shah JN, 2012, GASTROINTEST ENDOSC, V75, P56, DOI 10.1016/j.gie.2011.08.032
Shah RJ, 2013, GASTROINTEST ENDOSC, V77, P593, DOI 10.1016/j.gie.2012.10.015
Skinner M, 2014, ENDOSCOPY, V46, P560, DOI 10.1055/s-0034-1365698
Vila JJ, 2012, GASTROINTEST ENDOSC, V76, P1133, DOI 10.1016/j.gie.2012.08.001
Weilert F, 2011, ENDOSCOPY, V43, P1105, DOI 10.1055/s-0030-1256961
Will U, 2007, ENDOSCOPY, V39, P292, DOI 10.1055/s-2007-966215
Wilson JA, 2010, GASTROINTEST ENDOSC, V72, P947, DOI 10.1016/j.gie.2010.07.016
Wright BE, 2002, GASTROINTEST ENDOSC, V56, P225, DOI 10.1067/mge.2002.126136
NR 54
TC 29
Z9 34
U1 0
U2 2
PU BAISHIDENG PUBLISHING GROUP INC
PI PLEASANTON
PA 8226 REGENCY DR, PLEASANTON, CA 94588 USA
SN 1948-5190
J9 WORLD J GASTRO ENDOS
JI World J. Gastrointest. Endosc.
PD MAR 16
PY 2015
VL 7
IS 3
BP 283

EP 289
DI 10.4253/wjge.v7.i3.283
PG 7
WC Gastroenterology & Hepatology
WE Emerging Sources Citation Index (ESCI)
SC Gastroenterology & Hepatology
GA CR0GQ
UT WOS:000360996900017
PM 25789101
OA Green Submitted, hybrid
DA 2026-05-09
ER

PT J
AU Capurso, G
Coluccio, C
Rizzo, GEM
Crinò, SF
Cucchetti, A
Facciorusso, A
Hassan, C
Amato, A
Auriemma, F
Bertanil, H
Binda, C
Cipolletta, F
Fortin, E
Fugazza, A
Lisotti, A
Maida, M
Sinagra, E
Sbrancia, M
Spadaccini, M
Tacelli, M
Vanella, G
Anderloni, A
Fabbri, C
Tarantino, I
AF Capurso, Gabriele
Coluccio, Chiara
Rizzo, Giacomo Emanuele Maria
Crino, Stefano Francesco

Cucchetti, Alessandro
Facciorusso, Antonio
Hassan, Cesare
Amato, Arnaldo
Auriemma, Francesco
Bertanil, Helga
Binda, Cecilia
Cipolletta, Fabio
Fortin, Edoardo
Fugazza, Alessandro
Lisotti, Andrea
Maida, Marcello
Sinagra, Emanuele
Sbrancia, Monica
Spadaccini, Marco
Tacelli, Matteo
Vanella, Giuseppe
Anderloni, Andrea
Fabbri, Carlo
Tarantino, Ilaria
CA i-EUS Working Grp
TI The 1st i-EUS consensus on the management of pancreatic fluid
collections - Part 2
SO DIGESTIVE AND LIVER DISEASE
LA English
DT Article
DE Pancreatic fluid collections; Walled-off necrosis; Pseudocysts; LAMS;
Endoscopy; EUS; Consensus; guidelines
ID EARLY ENTERAL NUTRITION; STEP-UP APPROACH; PARENTERAL-NUTRITION;
NECROTIZING PANCREATITIS; ENDOSCOPIC NECROSECTOMY; NASOJEJUNAL TUBE;
METAANALYSIS; MULTICENTER; MODERATE; OUTCOMES
AB Pancreatic fluid collections (PFCs), including pancreatic pseudocysts (PPs) and walled-off pancreatic necrosis (WON), are common complications of pancreatitis and pancreatic surgery. Historically, the treatment of these conditions has relied on surgical and radiological approaches. The treatment of patients with PFCs has already focused toward an endoscopy-based approach, and with the development of dedicated lumen-apposing metal stents (LAMS), it has almost totally shifted towards interventional Endoscopic Ultrasound (EUS)-guided procedures. However, there is still limited consensus on several aspects of PFCs treatment within the multidisciplinary management. The interventional endoscopy and ultrasound (i-EUS) group is an Italian network of clinicians and scientists with special interest in biliopancreatic interventional endoscopy, especially interventional EUS. This manuscript focuses on the second part of the results of a consensus conference organized by i-EUS, with the aim of providing evidence-based guidance on several intra- and post-procedural aspects of PFCs drainage, such as clinical management and follow-up. (c) 2024 Editrice Gastroenterologica Italiana S.r.l. Published by Elsevier Ltd. All rights are reserved, including those for text and data mining, AI training, and similar

technologies.
C1 [Capurso, Gabriele] Ist Sci San Raffaele, Pancreat Biliary Endoscopy & Endosonog Div, Pancreas Translat & Clin Res Ctr, Milan, Italy.

[Coluccio, Chiara; Binda, Cecilia; Sbrancia, Monica; Fabbri, Carlo] AUSL Romagna, Forli Cesena Hosp, Gastroenterol & Digest Endoscopy Unit, Forli Cesena, Italy.

[Rizzo, Giacomo Emanuele Maria; Tarantino, Ilaria] IRCCS, Dept Diagnost & Therapeut Serv, Endoscopy Serv, ISMETT, Palermo, Italy.

[Rizzo, Giacomo Emanuele Maria] Univ Palermo, Dept Precis Med Med Surg & Cit Care Me Pre CC, Palermo, Italy.

[Crino, Stefano Francesco] GB Rossi Univ Hosp, Pancreas Inst, Diagnost & Intervent Endoscopy Pancreas, I-37134 Verona, Italy.

[Cucchetti, Alessandro] Alma Mater Studiorum Univ Bologna, Dept Med & Surg Sci DIMEC, Bologna, Italy.

[Facciorusso, Antonio] Univ Foggia, Dept Med Sci, Gastroenterol Unit, Foggia, Italy.

[Hassan, Cesare; Spadaccini, Marco] Humanitas Univ, Dept Biomed Sci, Pieve Emanuele, Italy.

[Hassan, Cesare; Fugazza, Alessandro] IRCCS, Human Res Hosp, Div Gastroenterol & Digest Endoscopy, Milan, Italy.

[Amato, Arnaldo] ASST Lecco, Digest Endoscopy & Gastroenterol Dept, Lecce, Italy.

[Auriemma, Francesco] Human Mater Domini, Gastrointestinal Endoscopy Unit, I-21100 Castellanza, Italy.

[Bertanil, Helga] Azienda Osped Univ Policlin Modena, Gastroenterol Endoscopia Digestiva, Modena, Italy.

[Cipolletta, Fabio] Osped Mare, ASL Ctr NA1, Dept Gastroenterol, Naples, Italy.

[Fortin, Edoardo] ASST Niguarda, Niguarda Hosp, Milan, Italy.

[Lisotti, Andrea] Univ Bologna, Hosp Imola, Gastroenterol Unit, Imola, Italy.

[Maida, Marcello] Univ Enna Kore, Umberto Hosp 1, Dept Med & Surg, Gastroenterol Unit, Enna, Italy.

[Sinagra, Emanuele] Fdn Ist G Giglio, Gastroenterol & Endoscopy Unit, Cefalu, Italy.

[Tacelli, Matteo; Vanella, Giuseppe] IRCCS, Pancreato Biliary Endoscopy & EUS Div, San Raffaele Sci Inst, Milan, Italy.

[Anderloni, Andrea] IRCCS Fdn Policlin San Matteo, Gastroenterol & Digest Endoscopy Unit, Pavia, Italy.

C3 Vita-Salute San Raffaele University; IRCCS Ospedale San Raffaele; AUSL della Romagna; IRCCS Istituto Mediterraneo per i Trapianti e Terapie ad Alta Specializzazione (ISMETT); University of Palermo; University of Verona; Azienda Ospedaliera Universitaria Integrata Verona; University of Bologna; University of Foggia; Humanitas University; Universita di Modena e Reggio Emilia; Universita di Modena e Reggio Emilia Hospital; Ospedale Niguarda Ca' Granda; Azienda USL di Imola; University of Bologna; Universita Kore di ENNA; Vita-Salute San Raffaele University; IRCCS Ospedale San Raffaele; IRCCS Fondazione San Matteo

RP Rizzo, GEM (corresponding author), IRCCS, Dept Diagnost & Therapeut Serv, Endoscopy Serv, ISMETT, Palermo, Italy.

EM g.rizzo.gr@gmail.com

RI Lisotti, Andrea/L-6762-2019; Binda, Cecilia/ABH-1588-2020; Tacelli, Matteo/AAK-8808-2020; Spadaccini, Marco/HOH-7613-2023; Tarantino, Ilaria/AFS-0726-2022; Vanella, Giuseppe/LQI-8930-2024; Sinagra, Emanuele/C-9336-2014; Capurso, Gabriele/AAB-1389-2019; Bertani, Helga/AAF-9839-2019; Maida, Marcello/I-4118-2019; Facciorusso,

Antonio/AAA-5966-2019; Amato, Arnaldo/ABC-5688-2020; Fugazza, Alessandro/ABG-9381-2021; hassan, cesare/H-2844-2012; Rizzo, Giacomo Emanuele Maria/IAR-5913-2023; Crinó, Stefano Francesco/Q-3421-2019
OI Tacelli, Matteo/0000-0003-2642-6694; Capurso, Gabriele/0000-0002-0019-8753; Coluccio, Chiara/0000-0002-8791-7764; Amato, Arnaldo/0000-0002-4397-4142; Fugazza, Alessandro/0000-0003-0485-4903; hassan, cesare/0000-0001-7167-1459; Rizzo, Giacomo Emanuele Maria/0000-0001-9335-6740; Crinó, Stefano Francesco/0000-0003-4560-8741
CR Abdelhafez Mohamed, 2013, Surg Endosc, V27, P3911, DOI 10.1007/s00464-013-2948-x
Al-Omran M, 2010, COCHRANE DB SYST REV, DOI 10.1002/14651858.CD002837.pub2
Anderloni A, 2021, SURG ENDOSC, V35, P1741, DOI 10.1007/s00464-020-07567-8
Atkins D, 2004, BMC HEALTH SERV RES, V4, DOI 10.1186/1472-6963-4-38
Bakker OJ, 2014, NEW ENGL J MED, V371, P1983, DOI 10.1056/NEJMoa1404393
Bakker OJ, 2014, PANCREATOLOGY, V14, P340, DOI 10.1016/j.pan.2014.07.008
Bang JY, 2013, J GASTROEN HEPATOL, V28, P1725, DOI 10.1111/jgh.12328
Banks PA, 2013, GUT, V62, P102, DOI 10.1136/gutjnl-2012-302779
Baron TH, 2020, GASTROENTEROLOGY, V158, P67, DOI 10.1053/j.gastro.2019.07.064
Besselink M, 2013, PANCREATOLOGY, V13, pE1, DOI 10.1016/j.pan.2013.07.063
Binmoeller KF, 2011, ENDOSCOPY, V43, P337, DOI 10.1055/s-0030-1256127
Büchler MW, 2000, ANN SURG, V232, P619
Chang YS, 2013, CRIT CARE, V17, DOI 10.1186/cc12790
Chen YI, 2019, ENDOSC INT OPEN, V7, pE347, DOI 10.1055/a-0828-7630
Cho J, 2020, CLIN TRANSL GASTROEN, V11, DOI 10.14309/ctg.0000000000000251
Cornberg M, 2019, J HEPATOL, V70, P5, DOI 10.1016/j.jhep.2018.10.011
Dervenis Christos, 2003, J Hepatobiliary Pancreat Surg, V10, P415, DOI 10.1007/s00534-002-0727-5
Eatock FC, 2005, AM J GASTROENTEROL, V100, P432, DOI 10.1111/j.1572-0241.2005.40587.x
Eckerwall GE, 2006, ANN SURG, V244, P959, DOI 10.1097/01.sla.0000246866.01930.58
Facciorusso A, 2022, GASTROINTEST ENDOSC, V95, P1158, DOI 10.1016/j.gie.2021.12.006
Feng P, 2017, MEDICINE, V96, DOI 10.1097/MD.0000000000008648
Gao ZL, 2023, MEDICINE, V102, DOI 10.1097/MD.0000000000036454
Garret C, 2020, ANN INTENSIVE CARE, V10, DOI 10.1186/s13613-020-00698-0
Ge PS, 2020, PANCREAS, V49, P236, DOI 10.1097/MPA.0000000000001476
Gou SM, 2014, CRIT CARE, V18, DOI 10.1186/cc13809
Guo QH, 2022, HELIYON, V8, DOI 10.1016/j.heliyon.2022.e08852
Huang W, 2019, DIGEST DIS SCI, V64, P1985, DOI 10.1007/s10620-019-05568-9
Hyun JJ, 2019, GUT LIVER, V13, P215, DOI 10.5009/gnl18234
Iannuzzi JP, 2022, GASTROENTEROLOGY, V162, P122, DOI 10.1053/j.gastro.2021.09.043
Jablonska B, 2021, NUTRIENTS, V13, DOI 10.3390/nu13051498
Jagielski M, 2022, FRONT CELL INFECT MI, V12, DOI 10.3389/fcimb.2022.939138
Jin M, 2017, PANCREATOLOGY, V17, P651, DOI 10.1016/j.pan.2017.08.011
Jürgensen C, 2012, SURG ENDOSC, V26, P1359, DOI 10.1007/s00464-011-2039-9

Kahl S, 2014, J PANCREAS, V15, P165, DOI 10.6092/1590-8577/797
Kumar A, 2006, J CLIN GASTROENTEROL, V40, P431, DOI 10.1097/00004836-200605000-00013
Li W, 2018, J INT MED RES, V46, P3948, DOI 10.1177/0300060518782070
Li XP, 2014, MED SCI MONITOR, V20, P2327, DOI 10.12659/MSM.892770
Loosen SH, 2022, Z GASTROENTEROL, V60, P310, DOI 10.1055/a-1682-7621
Maharshi S, 2021, ENDOSC INT OPEN, V09, pE1108, DOI 10.1055/a-1480-7115
Marik PE, 2004, BMJ-BRIT MED J, V328, P1407, DOI 10.1136/bmj.38118.593900.55
McClave SA, 2016, JPEN-PARENTER ENTER, V40, P159, DOI [10.1177/0148607115621863, 10.1097/CCM.0000000000001525]
Messallam AA, 2021, AM J GASTROENTEROL, V116, P700, DOI 10.14309/ajg.0000000000000987
Migliori M, 2004, PANCREAS, V28, P359, DOI 10.1097/00006676-200405000-00001
Mohan BP, 2022, GASTROINTEST ENDOSC, V95, P1060, DOI 10.1016/j.gie.2022.01.018
Moka P, 2018, PANCREAS, V47, P489, DOI 10.1097/MPA.0000000000001019
Nakashima I, 2021, PANCREAS, V50, P371, DOI 10.1097/MPA.0000000000001768
Nally DM, 2014, BRIT J NUTR, V112, P1769, DOI 10.1017/S0007114514002566
Negm AA, 2013, GASTROINTEST ENDOSC, V78, P303, DOI 10.1016/j.gie.2013.03.001
Oh D, 2022, GASTROINTEST ENDOSC, V95, P735, DOI 10.1016/j.gie.2021.12.015
Patankar R V, 1995, HPB Surg, V8, P159
Petrov MS, 2008, ALIMENT PHARM THER, V28, P704, DOI 10.1111/j.1365-2036.2008.03786.x
Petrov MS, 2009, BRIT J SURG, V96, P1243, DOI 10.1002/bjs.6862
Petrov MS, 2010, BRIT J NUTR, V103, P1287, DOI 10.1017/S0007114510000887
Petrov MS, 2008, J PANCREAS, V9, P440
Petrov MS, 2009, BRIT J NUTR, V101, P787, DOI 10.1017/S0007114508123443
Pokras S, 2021, PANCREAS, V50, P330, DOI 10.1097/MPA.0000000000001757
Poropat G, 2015, COCHRANE DB SYST REV, DOI 10.1002/14651858.CD010605.pub2
Powers PC, 2019, ENDOSC ULTRASOUND, V8, P194, DOI DOI 10.4103/eus.eus_59_18
Qi DS, 2018, JPEN-PARENTER ENTER, V42, P1139, DOI 10.1002/jpen.1139
Qu C, 2021, PANCREATOLOGY, V21, P64, DOI 10.1016/j.pan.2020.11.010
Quan HM, 2011, GASTROENT RES PRACT, V2011, DOI 10.1155/2011/698248
Rai A, 2022, DIGEST DIS SCI, V67, P2535, DOI 10.1007/s10620-021-06992-6
Ramírez-Maldonado E, 2021, ANN SURG, V274, P255, DOI 10.1097/SLA.0000000000004596
Sahar N, 2018, EUR J CLIN MICROBIOL, V37, P1353, DOI 10.1007/s10096-018-3259-x
Seewald S, 2009, GASTROINTEST ENDOSC, V69, pS13, DOI 10.1016/j.gie.2008.10.061
Singh N, 2012, PANCREAS, V41, P153, DOI 10.1097/MPA.0b013e318221c4a8
Smit M, 2016, WORLD J SURG, V40, P1454, DOI 10.1007/s00268-015-3388-7
Song JB, 2018, MEDICINE, V97, DOI 10.1097/MD.0000000000011871
Stimac D, 2016, PANCREATOLOGY, V16, P523, DOI 10.1016/j.pan.2016.04.003
Tamura T, 2019, DIGEST ENDOSC, V31, P307, DOI 10.1111/den.13319
Thompson CC, 2016, PANCREATOLOGY, V16, P66, DOI 10.1016/j.pan.2015.12.001
Tiengou LE, 2006, JPEN-PARENTER ENTER, V30, P1, DOI 10.1177/014860710603000101
van Brunschot S, 2018, GUT, V67, P697, DOI 10.1136/gutjnl-2016-313341
van Brunschot S, 2018, LANCET, V391, P51, DOI 10.1016/S0140-6736(17)32404-2

van der Merwe SW, 2022, ENDOSCOPY, V54, P185, DOI 10.1055/a-1717-1391
van Minnen LP, 2007, J GASTROINTEST SURG, V11, P682, DOI 10.1007/s11605-007-0088-0
van Santvoort HC, 2010, NEW ENGL J MED, V362, P1491, DOI 10.1056/NEJMoa0908821
Wan YD, 2021, WORLD J GASTROENTERO, V27, P224, DOI 10.3748/wjg.v27.i2.224
Wittau M, 2011, SCAND J GASTROENTERO, V46, P261, DOI 10.3109/00365521.2010.531486
Wu LM, 2014, BJS-BRIT J SURG, V101, P1644, DOI 10.1002/bjs.9665
Wu P, 2018, BIOSCIENCE REP, V38, DOI [10.1042/BSR20181515, 10.1042/bsr20181515]
Wu XM, 2010, PANCREAS, V39, P248, DOI 10.1097/MPA.0b013e3181bd6370
Yao H, 2018, EUR J CLIN NUTR, V72, P66, DOI 10.1038/ejcn.2017.139
Yao Q, 2022, PANCREATOLOGY, V22, P175, DOI 10.1016/j.pan.2021.11.009
Yi FM, 2012, INTERNAL MED, V51, P523, DOI 10.2169/internalmedicine.51.6685
Zhao XL, 2015, NUTRITION, V31, P171, DOI 10.1016/j.nut.2014.07.002
Zhu YF, 2016, GASTROENT RES PRACT, V2016, DOI 10.1155/2016/6430632
NR 87
TC 12
Z9 12
U1 1
U2 3
PU ELSEVIER SCIENCE INC
PI NEW YORK
PA STE 800, 230 PARK AVE, NEW YORK, NY 10169 USA
SN 1590-8658
EI 1878-3562
J9 DIGEST LIVER DIS
JI Dig. Liver Dis.
PD NOV
PY 2024
VL 56
IS 11
BP 1819
EP 1827
DI 10.1016/j.dld.2024.06.004
EA OCT 2024
PG 9
WC Gastroenterology & Hepatology
WE Science Citation Index Expanded (SCI-EXPANDED)
SC Gastroenterology & Hepatology
GA L0K6D
UT WOS:001347700900001
PM 39030137
DA 2026-05-09
ER

PT J
AU Soga, K
AF Soga, Koichi
TI Single-pigtail plastic stent made from endoscopic nasobiliary drainage
tubes in endoscopic ultrasound-guided gallbladder drainage: a
retrospective case series
SO CLINICAL ENDOSCOPY
LA English
DT Article
DE Cholecystitis; Drainage; Endoscopy; Gallbladder
AB Technical failure of endoscopic ultrasound-guided gallbladder drainage (EUS-GBD) is often attributed to device failure. To rectify this problem, we developed a single-pigtail plastic stent (SPPS) for EUS-GBD. We retrospectively reviewed the cases of four patients who underwent EUS-GBD for acute cholecystitis. To prepare the SPPS, a 7.5-Fr endoscopic nasobiliary drainage tube was cut to an appropriate length. The use of SPPS during EUS-GBD was successful from both technical and clinical standpoints. The SPPS spontaneously detached 57 days after the procedure in patient 4 and 412 days after the procedure in patient 1. Patient 1 developed cholecystitis after 426 days and was managed with antibiotics. The other three patients did not develop any complications after surgery. In conclusion, we designed a new SPPS dedicated to EUS-GBD and established its technical feasibility and clinical effectiveness.
C1 [Soga, Koichi] Omihachiman Community Med Ctr, Dept Gastroenterol, 1379 Tsuchida cho, Omihachiman, Shiga 5230082, Japan.
RP Soga, K (corresponding author), Omihachiman Community Med Ctr, Dept Gastroenterol, 1379 Tsuchida cho, Omihachiman, Shiga 5230082, Japan.
EM sogatti@koto.kpu-m.ac.jp
OI SOGA, KOICHI/0000-0003-2916-2268
CR [Anonymous], 2020, Asa Physical Status Classification System
[Anonymous], 2022, Flexima Plus
CHARLSON ME, 1987, J CHRON DIS, V40, P373, DOI 10.1016/0021-9681(87)90171-8
James TW, 2019, ENDOSC ULTRASOUND, V8, pS28, DOI 10.4103/eus.eus_41_19
Kanda M, 2016, MEDICINE, V95, DOI 10.1097/MD.0000000000003781
Nishiguchi K, 2021, ENDOSC ULTRASOUND, V10, P448, DOI 10.4103/EUS-D-20-00130
Park SW, 2022, DIGEST ENDOSC, V34, P439, DOI 10.1111/den.14083
Rana SS, 2021, ANN GASTROENTEROL, V34, P142, DOI 10.20524/aog.2020.0568
van der Merwe SW, 2022, ENDOSCOPY, V54, P185, DOI 10.1055/a-1717-1391
Yang MJ, 2020, DIGEST DIS, V38, P542, DOI 10.1159/000505644
NR 10
TC 4
Z9 4
U1 0
U2 0
PU KOREAN SOC GASTROINTESTINAL ENDOSCOPY
PI SEOUL

PA 2003 LG PALACE, 165-8 DONGGYO-DONG, MAPO-GU, SEOUL, 121-754, SOUTH KOREA
SN 2234-2400
EI 2234-2443
J9 CLIN ENDOSC
JI Clin. Endosc.
PD MAR
PY 2024
VL 57
IS 2
BP 263
EP 267
DI 10.5946/ce.2022.213
PG 5
WC Gastroenterology & Hepatology
WE Emerging Sources Citation Index (ESCI)
SC Gastroenterology & Hepatology
GA OK9P9
UT WOS:001207287000006
PM 37011902
OA Green Submitted, gold
DA 2026-05-09
ER

PT J
AU Tyberg, A
Kats, D
Choi, A
Gaidhane, M
Nieto, J
Kahaleh, M
AF Tyberg, Amy
Kats, Daniel
Choi, Anthony
Gaidhane, Monica
Nieto, Jose
Kahaleh, Michel
TI Endoscopic Ultrasound Guided Gastroenterostomy What Is the Learning
Curve?
SO JOURNAL OF CLINICAL GASTROENTEROLOGY
LA English
DT Article
DE endoscopic ultrasound guided gastroenterostomy EUS-GE; lumen-apposing

metal stent; LAMS; CUSUM; learning curve

ID GASTRIC OUTLET OBSTRUCTION; APPOSING METAL STENT; SURGICAL GASTROJEJUNOSTOMY; CLINICAL-EXPERIENCE; MULTICENTER; PALLIATION; PLACEMENT

AB Introduction: Endoscopic ultrasound guided gastroenterostomy (EUS-GE) is a minimally invasive option for gastric outlet obstruction. It requires skills in endoscopic ultrasound, fluoroscopy, and lumen-apposing metal stent deployment. The aim of this study was to determine the learning curve for EUS-GE. Methods: Consecutive patients undergoing EUS-GE by a single operator were included from a prospective registry over 3 years. Demographics, procedure info, postprocedure follow-up data, and adverse events were collected. Nonlinear regression and cumulative sum analyses were conducted for the learning curve. Clinical success was defined as tolerating a diet postprocedure. Results: Twenty-three patients were included (39% male, mean age 65.8 y). Technical success was achieved in 22 (96%) patients. Clinical success was achieved in 21/22 (95%) patients. Average follow-up time 10.8 months (9.1 SD). Five patients had minor postprocedure complications; 1 patient had a periprocedural esophageal tear treated with clips. Four patients required repeat intervention for stent revision or removal if no longer needed. Median procedure time was 88 minutes (range: 45 to 140 min). Cumulative sum chart shows 88-minute procedure time was achieved at the seventh procedure indicating efficiency. Even with bridging of a misdeployed lumen-apposing metal stent, the procedure duration further reduced with consequent procedures indicating continued improvement with experience (nonlinear regression P<0.0001). Conclusions: Endoscopists experienced in EUS-GE achieve a reduction in procedure time over successive cases, with efficiency reached at 88 minutes and a learning rate of 7 cases. Misdeployed stents that require bridging add to the procedure time even after competency is achieved but do not affect the overall learning curve trend.

C1 [Tyberg, Amy; Kats, Daniel; Gaidhane, Monica; Kahaleh, Michel] Robert Wood Johnson Med Ctr, New Brunswick, NJ USA.

[Choi, Anthony] Weill Cornell Med, New York, NY USA.

[Nieto, Jose] Borland Groover Clin, Jacksonville, FL USA.

C3 Rutgers University System; Rutgers University New Brunswick; Rutgers University Biomedical & Health Sciences; Cornell University; Weill Cornell Medicine

RP Kahaleh, M (corresponding author), Robert Wood Johnson Univ Hosp, 1 RWJ Pl,MEB 491C, New Brunswick, NJ 08901 USA.

EM mkahaleh@gmail.com

RI Kahaleh, Michel/A-9408-2011; M, Gaidhane/B-9414-2009; Gaidhane, Monica/B-9414-2009

OI Gaidhane, Monica/0000-0001-6773-7080

CR Barthet M, 2015, GASTROINTEST ENDOSC, V81, P215, DOI 10.1016/j.gie.2014.09.039

Bartlett EK, 2014, J GASTROINTEST SURG, V18, P1292, DOI 10.1007/s11605-014-2502-8

Chen YI, 2018, GASTROINTEST ENDOSC, V87, P1215, DOI 10.1016/j.gie.2017.07.030

Chen YI, 2017, GASTROENTEROLOGY, V153, P15, DOI 10.1053/j.gastro.2017.04.045

Chen YI, 2017, SURG ENDOSC, V31, P2946, DOI 10.1007/s00464-016-5311-1

Cotton PB, 2010, GASTROINTEST ENDOSC, V71, P446, DOI 10.1016/j.gie.2009.10.027

Gonzalez JM, 2016, ENDOSC INT OPEN, V4, pE1286, DOI 10.1055/s-0042-118176

Jeurnink SM, 2007, J SURG ONCOL, V96, P389, DOI 10.1002/jso.20828

Jeurnink SM, 2010, GASTROINTEST ENDOSC, V71, P490, DOI 10.1016/j.gie.2009.09.042

Khashab M, 2013, SURG ENDOSC, V27, P2068, DOI 10.1007/s00464-012-2712-7
Khashab MA, 2017, ENDOSC INT OPEN, V5, pE275, DOI 10.1055/s-0043-101695
Khashab MA, 2015, GASTROINTEST ENDOSC, V82, P932, DOI 10.1016/j.gie.2015.06.017
Maetani I, 2004, ENDOSCOPY, V36, P73
Miyazaki Y, 2016, WORLD J GASTROINTEST, V8, P165, DOI 10.4253/wjge.v8.i3.165
Oh D, 2017, THER ADV GASTROENTER, V10, P42, DOI 10.1177/1756283X16671671
Perez-Miranda M, 2017, J CLIN GASTROENTEROL, V51, P896, DOI 10.1097/MCG.0000000000000887
Rodrigues-Pinto E, 2015, ENDOSCOPY, V47, pE594, DOI 10.1055/s-0034-1393650
Takeno A, 2013, ANN SURG ONCOL, V20, P3527, DOI 10.1245/s10434-013-3033-3
Tyberg A, 2017, ENDOSCOPY, V49, P1007, DOI 10.1055/s-0043-113551
Tyberg A, 2016, ENDOSC INT OPEN, V4, pE276, DOI 10.1055/s-0042-101789
NR 20
TC 18
Z9 23
U1 0
U2 1
PU LIPPINCOTT WILLIAMS & WILKINS
PI PHILADELPHIA
PA TWO COMMERCE SQ, 2001 MARKET ST, PHILADELPHIA, PA 19103 USA
SN 0192-0790
EI 1539-2031
J9 J CLIN GASTROENTEROL
JI J. Clin. Gastroenterol.
PD SEP
PY 2021
VL 55
IS 8
BP 691
EP 693
DI 10.1097/MCG.0000000000001400
PG 3
WC Gastroenterology & Hepatology
WE Science Citation Index Expanded (SCI-EXPANDED)
SC Gastroenterology & Hepatology
GA UA5UT
UT WOS:000685227700008
PM 32740096
DA 2026-05-09
ER

PT J
AU Ogura, T

Ueno, S
Hakoda, A
Okuda, A
Nishioka, N
Sakamoto, J
Yamamura, M
Hattori, N
Bessho, K
Nishikawa, H
Kanaoka, R
Kurose, Y
AF Ogura, Takeshi
Ueno, Saori
Hakoda, Akitoshi
Okuda, Atsushi
Nishioka, Nobu
Sakamoto, Jun
Yamamura, Masahiro
Hattori, Nobuhiro
Bessho, Kimi
Nishikawa, Hiroki
Kanaoka, Rie
Kurose, Youhei
TI Mechanical evaluation of the dilation force of dilation devices during interventional endoscopic ultrasound
SO ENDOSCOPY INTERNATIONAL OPEN
LA English
DT Article
DE Endoscopic ultrasonography; Intervention EUS; Biliary tract
ID GUIDED BILIARY DRAINAGE; ANTEGRADE DILATION; ADVERSE EVENTS; FEASIBILITY; STRICTURE; CONSENSUS; SAFETY
AB Background and study aims To insert the metal stent delivery system (8.5F) during interventional endoscopic ultrasound (I-EUS), several dilation steps are needed, which may be related to increased bile leakage from a fistula. There have been no definitive studies of dilation force. The aim of the present study was to evaluate dilation force during I-EUS using several dilation devices. Methods In the present study, seven dilation devices were evaluated including bougie dilators such as a straight-shaped dilator (the ES dilator, Soehendra dilator, a standard ERCP catheter) a screw-shaped dilator (Tornus ES, Soehendra stent retriever), and a 4-mm balloon catheter (REN biliary balloon catheter, Hurricane RX). The diameter of each dilator and dilation force were measured. Results Of the bougie dilators, the dilation force of the ES dilator was the highest (0.908 +/- 0.035 kg). Of the balloon catheters, the dilation force of the Hurricane RX (3.261 +/- 0.024 kg) was slightly higher than that of the REN (3.159 +/- 0.072 kg). Of the bougie dilators, although the diameter of the ES dilator was not larger than that of the Tornus ES, the dilation force was stronger. Similarly, the diameter of the Soehendra stent retriever was greater than that of the ERCP catheter or Soehendra dilator and the dilation force was lower. Conclusions Compared

with bougie dilators, balloon catheters have stronger dilation force according to our experimental study. The present results should be evaluated in clinical trials.
C1 [Ogura, Takeshi; Ueno, Saori; Hakoda, Akitoshi; Okuda, Atsushi; Nishioka, Nobu; Sakamoto, Jun; Yamamura, Masahiro; Hattori, Nobuhiro; Bessho, Kimi; Nishikawa, Hiroki] Osaka Med & Pharmaceut Univ, Dept Internal Med 2, Takatsuki, Japan.
[Kanaoka, Rie; Kurose, Youhei] Kaneka Medix Corp, Dept Business Planning, Osaka, Japan.
C3 Osaka Medical & Pharmaceutical University
RP Ogura, T (corresponding author), Osaka Med & Pharmaceut Univ, Dept Internal Med 2, Takatsuki, Japan.
EM oguratakeshi0411@yahoo.co.jp
CR Amano M, 2017, J GASTROEN HEPATOL, V32, P716, DOI 10.1111/jgh.13489
'Anna GD, 2022, BEST PRACT RES CL GA, V60-61, DOI 10.1016/j.bpg.2022.101810
Bessho K, 2023, THER ADV GASTROENTER, V16, DOI 10.1177/17562848231207004
Giovannini M, 2019, ENDOSC ULTRASOUND, V8, pS35, DOI 10.4103/eus.eus_47_19
Guo JT, 2018, ENDOSC ULTRASOUND, V7, P356, DOI 10.4103/eus.eus_53_18
Hattori N, 2023, GASTROINTEST ENDOSC, V97, P1153, DOI 10.1016/j.gie.2023.02.003
Honjo M, 2018, ENDOSC ULTRASOUND, V7, P376, DOI 10.4103/eus.eus_2_18
Kobori I, 2022, J CLIN MED, V11, DOI 10.3390/jcm11174986
Nakamura J, 2023, THER ADV GASTROENTER, V16, DOI 10.1177/17562848231188562
Ogawa T, 2023, DEN OPEN, V3, DOI 10.1002/deo2.170
Ogura T, 2023, ENDOSCOPY, V55, pE790, DOI 10.1055/a-2094-9374
Ogura T, 2023, ENDOSCOPY, V55, pE525, DOI 10.1055/a-2037-5749
Ogura T, 2021, DEN OPEN, V1, DOI 10.1002/deo2.8
Ogura T, 2021, GUT LIVER, V15, P196
Ogura T, 2020, CLIN ENDOSC, V53, P429
Park DH, 2011, GASTROINTEST ENDOSC, V74, P1276, DOI 10.1016/j.gie.2011.07.054
Prachayakul V, 2015, WORLD J GASTROENTERO, V21, P2725, DOI 10.3748/wjg.v21.i9.2725
Teoh AYB, 2018, GUT, V67, P1209, DOI 10.1136/gutjnl-2017-314341
Ueshima K, 2019, UNITED EUR GASTROENT, V7, P419, DOI 10.1177/2050640618823662
Yamamoto Y, 2020, ENDOSC ULTRASOUND, V9, P110, DOI 10.4103/eus.eus_68_19
NR 20
TC 3
Z9 4
U1 0
U2 1
PU GEORG THIEME VERLAG KG
PI STUTTGART
PA RUDIGERSTR 14, D-70469 STUTTGART, GERMANY
SN 2364-3722
EI 2196-9736
J9 ENDOSC INT OPEN
JI Endosc. Int. Open
PD AUG

PY 2024
VL 12
IS 08
BP E955
EP E961
DI 10.1055/a-2351-0647
PG 7
WC Gastroenterology & Hepatology; Surgery
WE Emerging Sources Citation Index (ESCI)
SC Gastroenterology & Hepatology; Surgery
GA C1A6Y
UT WOS:001286763600003
PM 39131731
OA Green Submitted, gold
DA 2026-05-09
ER

PT J
AU Isayama, H
Nakai, Y
Itoi, T
Yasuda, I
Kawakami, H
Ryozawa, S
Kitano, M
Irisawa, A
Katanuma, A
Hara, K
Iwashita, T
Fujita, N
Yamao, K
Yoshida, M
Inui, K
AF Isayama, Hiroyuki
Nakai, Yousuke
Itoi, Takao
Yasuda, Ichiro
Kawakami, Hiroshi
Ryozawa, Shomei
Kitano, Masayuki
Irisawa, Atsushi
Katanuma, Akio

Hara, Kazuo
Iwashita, Takuji
Fujita, Naotaka
Yamao, Kenji
Yoshida, Masahiro
Inui, Kazuo
TI Clinical practice guidelines for safe performance of endoscopic
ultrasound/ultrasonography-guided biliary drainage: 2018
SO JOURNAL OF HEPATO-BILIARY-PANCREATIC SCIENCES
LA English
DT Article
DE Biliary stricture; Endoscopic ultrasonography; EUS-guided biliary
drainage; Interventional EUS
ID COVERED METAL STENT; BILE-DUCT OBSTRUCTION; MALIGNANT BILIARY; FAILED
ERCP; DUODENAL STENT; ANTIBIOTIC-PROPHYLAXIS; CHOLEDOCHODUODENOSTOMY;
PLACEMENT; HEPATICOGASTROSTOMY; ACCESS
AB Endoscopic ultrasound/ultrasonography-guided biliary drainage (EUS-BD) is a relatively new modality for biliary drainage after failed or difficult transpapillary biliary cannulation. Despite its clinical utility, EUS-BD can be complicated by severe adverse events such as bleeding, perforation, and peritonitis. The aim of this paper is to provide practice guidelines for safe performance of EUS-BD as well as safe introduction of the procedure to non-expert centers. The guidelines comprised patient-intervention-comparison-outcome-formatted clinical questions (CQs) and questions (Qs), which are background statements to facilitate understanding of the CQs. A literature search was performed using the PubMed and Cochrane Library databases. Statement, evidence level, and strength of recommendation were created according to the GRADE system. Four committees were organized: guideline creation, expert panelist, evaluation, and external evaluation committees. We developed 13 CQs (methods, device selection, supportive treatment, management of adverse events, education and ethics) and six Qs (definition, indication, outcomes and adverse events) with statements, evidence levels, and strengths of recommendation. The guidelines explain the technical aspects, management of adverse events, and ethics of EUS-BD and its introduction to non-expert institutions.
C1 [Isayama, Hiroyuki] Juntendo Univ, Dept Gastroenterol, Grad Sch Med, Tokyo, Japan.
[Isayama, Hiroyuki; Nakai, Yousuke] Univ Tokyo, Dept Gastroenterol, Grad Sch Med, Tokyo, Japan.
[Itoi, Takao] Tokyo Med Univ, Dept Gastroenterol & Hepatol, Tokyo, Japan.
[Yasuda, Ichiro] Univ Toyama, Dept Internal Med 3, Toyama, Japan.
[Kawakami, Hiroshi] Univ Miyazaki, Fac Med, Dept Internal Med, Div Gastroenterol & Hepatol, Miyazaki, Japan.
[Ryozawa, Shomei] Saitama Med Univ, Dept Gastroenterol, Int Med Ctr, Saitama, Japan.
[Kitano, Masayuki] Wakayama Med Univ, Dept Internal Med 2, Wakayama, Japan.
[Irisawa, Atsushi] Dokkyo Med Univ, Dept Gastroenterol, Mibu, Tochigi, Japan.
[Katanuma, Akio] Teine Keijinkai Hosp, Ctr Gastroenterol, Sapporo, Hokkaido, Japan.
[Hara, Kazuo] Aichi Canc Ctr Hosp, Dept Gastroenterol, Nagoya, Aichi, Japan.
[Iwashita, Takuji] Gifu Univ Hosp, Dept Internal Med 1, Gifu, Japan.
[Fujita, Naotaka] Miyagi Hlth Check Up Plaza, Sendai, Miyagi, Japan.
[Yamao, Kenji] Narita Mem Hosp, Dept Gastroenterol, Nagoya, Aichi, Japan.
[Yoshida, Masahiro] Int Univ Hlth & Welf, Dept Hepatobiliary Pancreat & Gastrointestinal Su, Sch Med, Ichikawa,

Japan.
[Inui, Kazuo] Fujita Hlth Univ, Bantane Hosp, Dept Gastroenterol, Nagoya, Aichi, Japan.
C3 Juntendo University; University of Tokyo; Tokyo Medical University;
University of Toyama; University of Miyazaki; Saitama Medical
University; Wakayama Medical University; Dokkyo Medical University;
Teine Keijinkai Hospital; Aichi Cancer Center; Gifu University;
International University of Health & Welfare; Fujita Health University
RP Isayama, H (corresponding author), Juntendo Univ, Grad Sch Med, Dept Gastroenterol, Bunkyo Ku, 2-1-1 Hongo, Tokyo 1138421, Japan.
EM h-isayama@juntendo.ac.jp
RI Nakai, Yousuke/H-2964-2019; Isayama, Hiroyuki/AER-9916-2022; Yasuda,
Ichiro/IUP-0131-2023
OI Yasuda, Ichiro/0000-0002-6888-0310
FU Piolax Medical Devices, Inc. Funding Source: Medline; Boston Scientific
Japan K.K. Funding Source: Medline; Gadelius Medical K.K. Funding
Source: Medline; FUJIFILM Medical Co., Ltd Funding Source: Medline;
Japan Biliary Association Funding Source: Medline; Century Medical, Inc.
Funding Source: Medline
CR Allison MC, 2009, GUT, V58, P869, DOI 10.1136/gut.2007.136580
Ang TL, 2010, SINGAP MED J, V51, P762
Angsuwatcharakon P, 2012, J GASTROEN HEPATOL, V27, P356, DOI 10.1111/j.1440-1746.2011.06927.x
[Anonymous], 2019, PRINCIPLES BIOMEDICA
Artifon ELA, 2015, ENDOSC ULTRASOUND, V4, P235, DOI 10.4103/2303-9027.163010
Artifon ELA, 2015, GASTROINTEST ENDOSC, V81, P950, DOI 10.1016/j.gie.2014.09.047
Artifon ELA, 2012, J CLIN GASTROENTEROL, V46, P768, DOI 10.1097/MCG.0b013e31825f264c
Artifon ELA, 2011, J PANCREAS, V12, P610
Atkins D, 2004, BMC HEALTH SERV RES, V4, DOI 10.1186/1472-6963-4-38
Attasaranya S, 2012, GASTROENT RES PRACT, V2012, DOI 10.1155/2012/680753
Bang JY, 2018, GASTROINTEST ENDOSC, V88, P9, DOI 10.1016/j.gie.2018.03.012
Bapaye A, 2013, UNITED EUR GASTROENT, V1, P285, DOI 10.1177/2050640613490928
Bories E, 2007, ENDOSCOPY, V39, P287, DOI 10.1055/s-2007-966212
Brauer BC, 2009, GASTROINTEST ENDOSC, V70, P471, DOI 10.1016/j.gie.2008.12.233
Brückner S, 2015, ENDOSCOPY, V47, P858, DOI 10.1055/s-0034-1392104
Burmester E, 2003, GASTROINTEST ENDOSC, V57, P246, DOI 10.1067/mge.2003.85
Dhir V, 2017, ENDOSC INT OPEN, V5, pE1087, DOI 10.1055/s-0043-118097
Dhir Vinay, 2011, J Interv Gastroenterol, V1, P153
Dhir V, 2015, GASTROINTEST ENDOSC, V81, P913, DOI 10.1016/j.gie.2014.09.054
Dhir V, 2015, GASTROINTEST ENDOSC, V81, P440, DOI 10.1016/j.gie.2014.09.011
Dhir V, 2013, UNITED EUR GASTROENT, V1, P103, DOI 10.1177/2050640613480145
Dhir V, 2014, DIGEST ENDOSC, V26, P430, DOI 10.1111/den.12153
Dhir V, 2012, GASTROINTEST ENDOSC, V75, P354, DOI 10.1016/j.gie.2011.07.075
Eisen GA, 2002, GASTROINTEST ENDOSC, V55, P780, DOI 10.1016/S0016-5107(02)70403-3

Fabbri C, 2011, ENDOSCOPY, V43, P438, DOI 10.1055/s-0030-1256097
Fujimoto K, 2014, DIGEST ENDOSC, V26, P1, DOI 10.1111/den.12183
Fujita N, 2008, J GASTROENTEROL, V43, P637, DOI 10.1007/s00535-008-2204-7
Fujita N, 2007, WORLD J GASTROENTERO, V13, P5512, DOI 10.3748/wjg.v13.i41.5512
Giovannini M, 2001, ENDOSCOPY, V33, P898, DOI 10.1055/s-2001-17324
Glessing BR, 2015, GASTROINTEST ENDOSC, V81, P1019, DOI 10.1016/j.gie.2014.09.061
Gupta K, 2014, J CLIN GASTROENTEROL, V48, P80, DOI 10.1097/MCG.0b013e31828c6822
Guyatt GH, 2008, BMJ-BRIT MED J, V336, P995, DOI 10.1136/bmj.39490.551019.BE
Guyatt GH, 2008, BRIT MED J, V336, P924, DOI 10.1136/bmj.39489.470347.AD
Hamada T, 2015, RADIOLOGY, V274, P605, DOI 10.1148/radiol.14140293
Hamada T, 2014, DIGEST DIS SCI, V59, P1931, DOI 10.1007/s10620-014-3062-1
Hamada T, 2013, DIGEST ENDOSC, V25, P340, DOI 10.1111/den.12035
Hanada K, 2009, DIGEST ENDOSC, V21, pS75, DOI 10.1111/j.1443-1661.2009.00855.x
Hara K, 2013, ENDOSCOPY, V45, P392, DOI 10.1055/s-0032-1326076
Hara K, 2016, WORLD J GASTROENTERO, V22, P1297, DOI 10.3748/wjg.v22.i3.1297
Hara K, 2011, AM J GASTROENTEROL, V106, P1239, DOI 10.1038/ajg.2011.84
Holt BA, 2016, GASTROINTEST ENDOSC, V83, P160, DOI 10.1016/j.gie.2015.06.019
Horaguchi J, 2012, DIGEST ENDOSC, V24, P457, DOI 10.1111/j.1443-1661.2012.01316.x
Imai H, 2016, GASTROINTEST ENDOSC, V84, P147, DOI 10.1016/j.gie.2015.12.024
Ito K, 2010, DIAGN THER ENDOSC, V2010, DOI 1.1155/01/426534
Itoi T, 2014, GASTROINTEST ENDOSC, V79, P715, DOI 10.1016/j.gie.2013.11.021
Iwamuro M, 2010, DIGEST ENDOSC, V22, P236, DOI 10.1111/j.1443-1661.2010.00997.x
Iwashita T, 2012, ENDOSCOPY, V44, P60, DOI 10.1055/s-0030-1256871
Iwashita T, 2016, GASTROINTEST ENDOSC, V83, P394, DOI 10.1016/j.gie.2015.04.043
Iwashita T, 2013, DIGEST DIS SCI, V58, P2417, DOI 10.1007/s10620-013-2645-6
Kahaleh M, 2006, GASTROINTEST ENDOSC, V64, P52, DOI 10.1016/j.gie.2006.01.063
Kawakami H, 2016, GUT LIVER, V10, P318, DOI 10.5009/gnl15238
Kawakubo K, 2016, ENDOSCOPY, V48, P164, DOI 10.1055/s-0034-1393179
Kawakubo K, 2014, J HEPATO-BIL-PAN SCI, V21, P328, DOI 10.1002/jhbp.27
Kawakubo K, 2013, SURG ENDOSC, V27, P3437, DOI 10.1007/s00464-013-2896-5
Khan MA, 2016, DIGEST DIS SCI, V61, P684, DOI 10.1007/s10620-015-3933-0
Khashab MA, 2016, ENDOSC INT OPEN, V4, pE1322, DOI 10.1055/s-0042-110790
Khashab MA, 2016, ENDOSC INT OPEN, V4, pE175, DOI 10.1055/s-0041-109083
Khashab MA, 2015, GASTROINTEST ENDOSC, V81, P81, DOI 10.1016/j.gie.2014.08.008
Khashab MA, 2013, GASTROINTEST ENDOSC, V78, P734, DOI 10.1016/j.gie.2013.05.013
Khashab MA, 2012, GASTROINTEST ENDOSC, V76, P209, DOI 10.1016/j.gie.2012.03.170
Kida M, 2011, ENDOSCOPY, V43, P796, DOI 10.1055/s-0030-1256508
Kim TH, 2012, WORLD J GASTROENTERO, V18, P2526, DOI 10.3748/wjg.v18.i20.2526
Kim YS, 2010, ENDOSCOPY, V42, P496, DOI 10.1055/s-0029-1244082
Kunda R, 2016, SURG ENDOSC, V30, P5002, DOI 10.1007/s00464-016-4845-6
Lee TH, 2016, CLIN GASTROENTEROL H, V14, P1011, DOI 10.1016/j.cgh.2015.12.032
Lee TH, 2014, WORLD J GASTROENTERO, V20, P5859, DOI 10.3748/wjg.v20.i19.5859

Mallery S, 2004, GASTROINTEST ENDOSC, V59, P100, DOI 10.1016/S0016-5107(03)02300-9
Martins FP, 2010, ENDOSCOPY, V42, pE126, DOI 10.1055/s-0029-1243911
Miyano A, 2018, J GASTROINTEST SURG, V22, P1312, DOI 10.1007/s11605-018-3758-1
Nakai Y, 2016, ENDOSCOPY, V48, P1125, DOI 10.1055/s-0042-116595
Nguyen-Tang T, 2010, ENDOSCOPY, V42, P232, DOI 10.1055/s-0029-1243858
Ogura T, 2017, J GASTROEN HEPATOL, V32, P163, DOI 10.1111/jgh.13437
Ogura T, 2016, GASTROINTEST ENDOSC, V83, P834, DOI 10.1016/j.gie.2015.10.009
Ogura T, 2016, ENDOSCOPY, V48, P156, DOI 10.1055/s-0034-1392859
Ogura T, 2015, J GASTROEN HEPATOL, V30, P1748, DOI 10.1111/jgh.13021
Ogura T, 2015, ENDOSCOPY, V47, pE245, DOI 10.1055/s-0034-1391859
Ogura T, 2014, J GASTROEN HEPATOL, V29, P1815, DOI 10.1111/jgh.12598
Ogura T, 2013, AM J GASTROENTEROL, V108, P454, DOI 10.1038/ajg.2012.383
Oh D, 2017, THER ADV GASTROENTER, V10, P42, DOI 10.1177/1756283X16671671
Okuno N, 2015, ENDOSCOPY, V47, pE311, DOI 10.1055/s-0034-1392314
Paik WH, 2018, AM J GASTROENTEROL, V113, P987, DOI 10.1038/s41395-018-0122-8
Paik WH, 2014, WORLD J GASTROENTERO, V20, P5051, DOI 10.3748/wjg.v20.i17.5051
Park DH, 2015, J GASTROEN HEPATOL, V30, P1461, DOI 10.1111/jgh.13027
Park DH, 2013, GASTROINTEST ENDOSC, V78, P91, DOI 10.1016/j.gie.2013.01.042
Park DH, 2011, GASTROINTEST ENDOSC, V74, P1276, DOI 10.1016/j.gie.2011.07.054
Park DH, 2010, GASTROINTEST ENDOSC, V71, P413, DOI 10.1016/j.gie.2009.10.015
Park DH, 2009, AM J GASTROENTEROL, V104, P2168, DOI 10.1038/ajg.2009.254
Park JK, 2018, GASTROINTEST ENDOSC, V88, P277, DOI 10.1016/j.gie.2018.03.015
Park SJ, 2013, GASTROINTEST ENDOSC, V78, P374, DOI 10.1016/j.gie.2013.04.183
Peng CY, 2013, BMC GASTROENTEROL, V13, DOI 10.1186/1471-230X-13-147
Perez-Miranda M, 2014, GASTROINTEST ENDOSC, V80, P339, DOI 10.1016/j.gie.2014.04.030
Poincloux L, 2015, ENDOSCOPY, V47, P794, DOI [10.1055/s-0035-1545399, 10.1055/s-0034-1391988]
Polkowski M, 2012, ENDOSCOPY, V44, P190, DOI 10.1055/s-0031-1291543
Prachayakul V, 2013, ENDOSCOPY, V45, pE223, DOI 10.1055/s-0033-1344064
Prachayakul V, 2011, ENDOSCOPY, V43, pE213, DOI 10.1055/s-0030-1256395
Prachayakul V, 2015, WORLD J GASTROENTERO, V21, P2725, DOI 10.3748/wjg.v21.i9.2725
Prachayakul V, 2013, WORLD J GASTROENTERO, V19, P4758, DOI 10.3748/wjg.v19.i29.4758
Romagnuolo J, 2011, ENDOSCOPY, V43, pE356, DOI 10.1055/s-0030-1256859
Saxena P, 2015, GASTROINTEST ENDOSC, V81, P1010, DOI 10.1016/j.gie.2014.06.038
Shah JN, 2012, GASTROINTEST ENDOSC, V75, P56, DOI 10.1016/j.gie.2011.08.032
Sharaiha RZ, 2017, GASTROINTEST ENDOSC, V85, P904, DOI 10.1016/j.gie.2016.12.023
Siddiqui AA, 2011, SURG ENDOSC, V25, P549, DOI 10.1007/s00464-010-1216-6
Siripun A, 2015, WORLD J GASTRO ENDOS, V7, P283, DOI 10.4253/wjge.v7.i3.283
Song TJ, 2014, GASTROINTEST ENDOSC, V80, P707, DOI 10.1016/j.gie.2014.05.327
Song TJ, 2012, WORLD J GASTROENTERO, V18, P4435, DOI 10.3748/wjg.v18.i32.4435
Stanciu C, 2003, ENDOSCOPY, V35, P772, DOI 10.1055/s-2003-41590
The Comittee for Proposal of EUS BD, 2014, GASTROENTEROL ENDOSC, V56, P2254
Tsuchiya T, 2018, GASTROINTEST ENDOSC, V87, P1138, DOI 10.1016/j.gie.2017.08.017

Tsuchiya T, 2016, DIGEST ENDOSC, V28, P96, DOI 10.1111/den.12611

Uchida D, 2018, J MED ULTRASON, V45, P399, DOI 10.1007/s10396-017-0846-x

Uemura RS, 2018, J CLIN GASTROENTEROL, V52, P123, DOI 10.1097/MCG.0000000000000948

Umeda J, 2015, GASTROINTEST ENDOSC, V82, P390, DOI 10.1016/j.gie.2015.02.041

Vila JJ, 2012, GASTROINTEST ENDOSC, V76, P1133, DOI 10.1016/j.gie.2012.08.001

Vila JJ, 2010, AM J GASTROENTEROL, V105, P959, DOI 10.1038/ajg.2009.690

Vu CKF, 2006, GASTROINTEST ENDOSC, V63, P808, DOI 10.1016/j.gie.2005.09.033

Wang KX, 2016, GASTROINTEST ENDOSC, V83, P1218, DOI 10.1016/j.gie.2015.10.033

Weilert F, 2014, SURG ENDOSC, V28, P3193, DOI 10.1007/s00464-014-3588-5

Wiersema MJ, 1996, GASTROINTEST ENDOSC, V43, P102, DOI 10.1016/S0016-5107(06)80108-2

Will U, 2007, ENDOSCOPY, V39, P292, DOI 10.1055/s-2007-966215

Williams EJ, 2012, ENDOSCOPY, V44, P674, DOI 10.1055/s-0032-1309345

Yamao K, 2008, ENDOSCOPY, V40, P340, DOI 10.1055/s-2007-995485

Yamao K, 2007, DIGEST ENDOSC, V19, pS180, DOI 10.1111/j.1443-1661.2007.00742.x

Yamao K, 2006, GASTROINTEST ENDOSC, V64, P663, DOI 10.1016/j.gie.2006.07.003

NR 123

TC 136

Z9 148

U1 1

U2 6

PU WILEY

PI HOBOKEN

PA 111 RIVER ST, HOBOKEN 07030-5774, NJ USA

SN 1868-6974

EI 1868-6982

J9 J HEPATO-BIL-PAN SCI

JI J. Hepato-Biliary-Pancreat. Sci.

PD JUL

PY 2019

VL 26

IS 7

BP 249

EP 269

DI 10.1002/jhbp.631

EA JUN 2019

PG 21

WC Gastroenterology & Hepatology; Surgery

WE Science Citation Index Expanded (SCI-EXPANDED)

SC Gastroenterology & Hepatology; Surgery

GA IH1OC

UT WOS:000473726900001

PM 31025816

OA Green Submitted, hybrid
DA 2026-05-09
ER

PT J
AU On, W
Ahmed, W
Everett, S
Huggett, M
Paranandi, B
AF On, Wei
Ahmed, Wafaa
Everett, Simon
Huggett, Matthew
Paranandi, Bharat
TI Utility of interventional endoscopic ultrasound in pancreatic cancer
SO FRONTIERS IN ONCOLOGY
LA English
DT Review
DE pancreatic cancer; pancreatic ductal adenocarcinoma; endoscopic
ultrasound; interventional endoscopy; pancreatobiliary endoscopy
ID CELIAC PLEXUS NEUROLYSIS; GUIDED BILIARY DRAINAGE; APPOSING METAL STENT;
GASTRIC OUTLET OBSTRUCTION; FINE-NEEDLE INJECTION; QUALITY-OF-LIFE;
RADIOFREQUENCY ABLATION; LAPAROSCOPIC GASTROJEJUNOSTOMY; PHOTODYNAMIC
THERAPY; PRACTICE GUIDELINES
AB Endoscopic ultrasound (EUS) has an important role in the management algorithm of patients with pancreatic ductal adenocarcinoma (PDAC), typically for its diagnostic utilities. The past two decades have seen a rapid expansion of the therapeutic capabilities of EUS. Interventional EUS is now one of the more exciting developments within the field of endoscopy. The local effects of PDAC tend to be in anatomical areas which are difficult to target and endoscopy has cemented itself as a key role in managing the clinical sequelae of PDAC. Interventional EUS is increasingly utilized in situations whereby conventional endoscopy is either impossible to perform or unsuccessful. It also adds a different dimension to the host of oncological and surgical treatments for patients with PDAC. In this review, we aim to summarize the various ways in which interventional EUS could benefit patients with PDAC and aim to provide a balanced commentary on the current evidence of interventional EUS in the literature.
C1 [On, Wei; Ahmed, Wafaa; Everett, Simon; Huggett, Matthew; Paranandi, Bharat] Leeds Teaching Hosp NHS Trust, Dept Gastroenterol, Leeds, England.
C3 University of Leeds
RP On, W (corresponding author), Leeds Teaching Hosp NHS Trust, Dept Gastroenterol, Leeds, England.
EM aaron.on@nhs.net
FU The authors declare that no financial support was received for the
research, authorship, and/or publication of this article.
FX The authors declare that no financial support was received for the

research, authorship, and/or publication of this article.
CR Abbas A, 2022, ENDOSCOPY, V54, P671, DOI 10.1055/a-1708-0037
Adler DG, 2019, CLIN GASTROENTEROL H, V17, P1242, DOI 10.1016/j.cgh.2018.11.049
Akshintala VS, 2023, GASTROINTEST ENDOSC, V98, DOI 10.1016/j.gie.2023.03.023
Allemani C, 2018, LANCET, V391, P1023, DOI 10.1016/S0140-6736(17)33326-3
Alvarez-Sanchez MV, 2014, SURG ENDOSC, V28, P712, DOI 10.1007/s00464-013-3260-5
Anderloni A, 2019, GASTROINTEST ENDOSC, V89, P69, DOI [10.1016/j.gie.2018.04.1746, 10.1016/j.gie.2018.08.047]
Artifon ELA, 2015, GASTROINTEST ENDOSC, V81, P950, DOI 10.1016/j.gie.2014.09.047
Artifon ELA, 2012, J CLIN GASTROENTEROL, V46, P768, DOI 10.1097/MCG.0b013e31825f264c
Asif AA, 2021, J COMMUNITY HOSP INT, V11, P536, DOI 10.1080/20009666.2021.1929049
Baars JE, 2018, ENDOSC ULTRASOUND, V7, P4, DOI 10.4103/eus.eus_105_17
Bang JY, 2018, GASTROINTEST ENDOSC, V88, P9, DOI 10.1016/j.gie.2018.03.012
Baniya R, 2017, CLIN EXP GASTROENTER, V10, P67, DOI 10.2147/CEG.S132004
Barreto SG, 2012, PANCREATOLOGY, V12, P104, DOI 10.1016/j.pan.2012.02.010
Binda C, 2023, GASTROINTEST ENDOSC, V98, P765, DOI 10.1016/j.gie.2023.06.054
Binmoeller KF, 2012, ENDOSCOPY, V44, P499, DOI 10.1055/s-0032-1309382
Brimhall Bryan, 2011, Gastrointest Endosc Clin N Am, V21, P389, DOI 10.1016/j.giec.2011.04.002
Bronswijk Michiel, 2022, VideoGIE, V7, P466, DOI [10.1016/j.vgie.2022.08.021, 10.1016/j.vgie.2022.08.021]
Bronswijk M, 2021, GASTROINTEST ENDOSC, V94, P526, DOI 10.1016/j.gie.2021.04.006
Buwenge M, 2022, CURR ONCOL, V29, P2616, DOI 10.3390/curroncol29040214
Canakis A, 2020, BMJ OPEN GASTROENTER, V7, DOI 10.1136/bmjgast-2020-000428
Carrara S, 2021, ENDOSC ULTRASOUND, V10, P414, DOI 10.4103/EUS-D-20-00116
Castiglione D, 2020, ABDOM RADIOL, V45, P1162, DOI 10.1007/s00261-019-02136-7
Ceyhan GO, 2009, GASTROENTEROLOGY, V136, P177, DOI 10.1053/j.gastro.2008.09.029
Chan HH, 2004, GASTROINTEST ENDOSC, V59, P95, DOI 10.1016/S0016-5107(03)02361-7
Chan SM, 2023, DIGEST ENDOSC, V35, P512, DOI 10.1111/den.14472
Chandnani M, 2020, ENDOSC INT OPEN, V8, pE179, DOI 10.1055/a-1068-9128
Chang KJ, 2000, CANCER-AM CANCER SOC, V88, P1325, DOI 10.1002/(SICI)1097-0142(20000315)88:6<1325::AID-CNCR8>3.0.CO;2-T
Chen YI, 2017, SURG ENDOSC, V31, P2946, DOI 10.1007/s00464-016-5311-1
Cheung SLH, 2022, GUT LIVER, V16, P190
Chin JYL, 2020, ENDOSC INT OPEN, V08, pE1633, DOI 10.1055/a-1236-3217
Crinò SF, 2018, J GASTROINTEST LIVER, V27, P67, DOI 10.15403/jgld.2014.1121.271.eus
Artifon ELD, 2019, ENDOSC ULTRASOUND, V8, pS72, DOI 10.4103/eus.eus_62_19
Demir IE, 2013, PLOS ONE, V8, DOI 10.1371/journal.pone.0060529
DeWitt JM, 2019, GASTROINTEST ENDOSC, V89, P390, DOI 10.1016/j.gie.2018.09.007
Dhindsa BS, 2020, ENDOSC ULTRASOUND, V9, P101, DOI 10.4103/eus.eus_80_19
Di Mitri R, 2022, SURG ENDOSC, V36, P4553, DOI 10.1007/s00464-021-08808-0
di Mola FF, 2008, LANGENBECK ARCH SURG, V393, P919, DOI 10.1007/s00423-007-0277-z
Dietrich CF, 2022, ENDOSC ULTRASOUND, V11, P342, DOI 10.4103/EUS-D-21-00188
Ducreux M, 2015, ANN ONCOL, V26, pV56, DOI 10.1093/annonc/mdv295

Dumonceau JM, 2018, ENDOSCOPY, V50, P910, DOI 10.1055/a-0659-9864
Ekkelenkamp VE, 2015, ENDOSCOPY, V47, P504, DOI 10.1055/s-0034-1391231
El Chafic AH, 2019, DIGEST DIS SCI, V64, P3321, DOI 10.1007/s10620-019-05688-2
Fabbri C, 2019, J GASTROINTEST LIVER, V28, P125, DOI 10.15403/jgld.2014.1121.281.eus
Facciorusso A, 2022, CANCERS, V14, DOI 10.3390/cancers14133291
Fahmawi Y, 2022, TRANSL GASTROENT HEP, V7, DOI 10.21037/tgh-20-84
Figueiredo M, 2021, ENDOSC INT OPEN, V09, pE253, DOI 10.1055/a-1324-2892
Fiori E, 2004, ANTICANCER RES, V24, P269
Freelove R, 2006, AM FAM PHYSICIAN, V73, P485
Fugazza A, 2022, GASTROINTEST ENDOSC, V95, P896, DOI 10.1016/j.gie.2021.12.032
Ge PS, 2019, SURG ENDOSC, V33, P3404, DOI 10.1007/s00464-018-06636-3
Ghandour B, 2022, GASTROINTEST ENDOSC, V95, P80, DOI 10.1016/j.gie.2021.07.023
Giovannini M, 2001, ENDOSCOPY, V33, P898, DOI 10.1055/s-2001-17324
Girelli R, 2010, BRIT J SURG, V97, P220, DOI 10.1002/bjs.6800
Hall B, 2018, ANN SURG ONCOL, V25, pS123, DOI [10.18632/oncotarget.25036, 10.18632/oncotarget.25036]
Hanada Y, 2021, GASTROINTEST ENDOSC, V94, P179, DOI 10.1016/j.gie.2021.02.027
Herman JM, 2015, CANCER-AM CANCER SOC, V121, P1128, DOI 10.1002/cncr.29161
Herman JM, 2013, J CLIN ONCOL, V31, P886, DOI 10.1200/JCO.2012.44.7516
Hirooka Y, 2018, ONCOTARGET, V9, P2838, DOI 10.18632/oncotarget.22974
Iqbal U, 2020, ENDOSC ULTRASOUND, V9, P16, DOI 10.4103/eus.eus_70_19
Irani Shayan, 2020, VideoGIE, V5, P48, DOI [10.1016/j.vgie.2019.10.007, 10.1016/j.vgie.2019.10.007]
Jacques J, 2019, ENDOSCOPY, V51, P540, DOI 10.1055/a-0735-9137
Janet J, 2023, ANN SURG ONCOL, V30, P5036, DOI 10.1245/s10434-023-13466-8
Jaruvongvanich V, 2023, ENDOSC INT OPEN, V11, pE60, DOI 10.1055/a-1976-2279
Jeurnink SM, 2007, BMC GASTROENTEROL, V7, DOI 10.1186/1471-230X-7-18
Jeurnink SM, 2010, GASTROINTEST ENDOSC, V71, P490, DOI 10.1016/j.gie.2009.09.042
Jin Z, 2020, DIGEST ENDOSC, V32, P16, DOI 10.1111/den.13456
Jovani M, 2021, GASTROINTEST ENDOSC, V93, P1088, DOI 10.1016/j.gie.2020.09.041
Jue TL, 2021, GASTROINTEST ENDOSC, V93, P309, DOI 10.1016/j.gie.2020.07.063
Kaufman M, 2010, J CLIN GASTROENTEROL, V44, P127, DOI 10.1097/MCG.0b013e3181bb854d
Khamar J, 2023, SURG ENDOSC, V37, P4834, DOI 10.1007/s00464-022-09572-5
Khashab MA, 2017, ENDOSC INT OPEN, V5, pE275, DOI 10.1055/s-0043-101695
Khashab MA, 2012, GASTROINTEST ENDOSC, V76, P962, DOI 10.1016/j.gie.2012.07.006
Kothary N, 2009, J VASC INTERV RADIOL, V20, P235, DOI 10.1016/j.jvir.2008.09.026
Kouanda A, 2021, SURG ENDOSC, V35, P7058, DOI 10.1007/s00464-020-08221-z
Koulouris AI, 2021, PANCREATOLOGY, V21, P434, DOI 10.1016/j.pan.2020.12.016
Koulouris AI, 2017, DIGEST DIS SCI, V62, P861, DOI 10.1007/s10620-017-4488-z
Krishnamoorthi R, 2022, ENDOSC INT OPEN, V10, pE874, DOI 10.1055/a-1794-0635
Krishnamoorthi R, 2020, SURG ENDOSC, V34, P2866, DOI 10.1007/s00464-020-07484-w
Kristensen A, 2016, CRIT REV ONCOL HEMAT, V99, P286, DOI 10.1016/j.critrevonc.2016.01.006
Kunda R, 2016, SURG ENDOSC, V30, P5002, DOI 10.1007/s00464-016-4845-6
Latenstein AEJ, 2021, HPB, V23, P270, DOI 10.1016/j.hpb.2020.06.009

Lee PJ, 2018, HPB, V20, P477, DOI 10.1016/j.hpb.2017.12.007
Lee TH, 2016, CLIN GASTROENTEROL H, V14, P1011, DOI 10.1016/j.cgh.2015.12.032
Levy MJ, 2017, GASTROINTEST ENDOSC, V86, P161, DOI 10.1016/j.gie.2016.11.014
Lo W, 2022, CURR OPIN GASTROEN, V38, P521, DOI 10.1097/MOG.0000000000000874
Lu F, 2018, SUPPORT CARE CANCER, V26, P353, DOI 10.1007/s00520-017-3888-0
Ly J, 2010, SURG ENDOSC, V24, P290, DOI 10.1007/s00464-009-0577-1
Mangiavillano B, 2024, DIGEST ENDOSC, V36, P351, DOI 10.1111/den.14606
Mangiavillano B, 2022, GASTROINTEST ENDOSC, V95, P115, DOI 10.1016/j.gie.2021.07.021
Marx M, 2022, ENDOSC ULTRASOUND, V11, P495, DOI 10.4103/EUS-D-21-00108
Matthes K, 2007, GASTROINTEST ENDOSC, V65, P448, DOI 10.1016/j.gie.2006.06.030
McCarty Thomas R, 2019, Endosc Int Open, V7, pE1474, DOI [10.1055/a-0996-8178, 10.1055/a-0996-8178]
Mehta S, 2006, SURG ENDOSC, V20, P239, DOI 10.1007/s00464-005-0130-9
Michl P, 2021, UNITED EUR GASTROENT, V9, P860, DOI 10.1002/ueg2.12123
Minaga K, 2018, CANCERS, V10, DOI 10.3390/cancers10020050
Mizrahi JD, 2020, LANCET, V395, P2008
Moningi S, 2015, J ONCOL PRACT, V11, pE216, DOI 10.1200/JOP.2014.000976
Moole H, 2017, MEDICINE, V96, DOI 10.1097/MD.0000000000005154
Mussetto A, 2018, ANN GASTROENTEROL, V31, P535, DOI 10.20524/aog.2018.0287
Nehme F, 2022, DIGEST ENDOSC, V34, P428, DOI 10.1111/den.14081
Nennstiel S, 2015, J CLIN GASTROENTEROL, V49, P764, DOI 10.1097/MCG.0000000000000275
Obstein KL, 2010, WORLD J GASTROENTERO, V16, P728, DOI 10.3748/wjg.v16.i6.728
On W, 2022, GASTROINTEST ENDOSC, V95, P432, DOI 10.1016/j.gie.2021.09.040
Packard M, 2015, J MED IMAG RADIAT ON, V59, P499, DOI 10.1111/1754-9485.12294
Paik WH, 2020, Current Treatment Options in Gastroenterology, V18, P188, DOI [10.1007/s11938-020-00282-2, 10.1007/s11938-020-00282-2, DOI 10.1007/S11938-020-00282-2]
Paik WH, 2018, AM J GASTROENTEROL, V113, P987, DOI 10.1038/s41395-018-0122-8
Park JK, 2018, GASTROINTEST ENDOSC, V88, P277, DOI 10.1016/j.gie.2018.03.015
Park WG, 2010, GASTROINTEST ENDOSC, V71, P513, DOI 10.1016/j.gie.2009.10.030
Pawa R, 2024, DEN OPEN, V4, DOI 10.1002/deo2.248
Perez-Miranda M, 2017, J CLIN GASTROENTEROL, V51, P896, DOI 10.1097/MCG.0000000000000887
Prades J, 2020, ESMO OPEN, V5, DOI 10.1136/esmoopen-2020-001051
Puli SR, 2009, DIGEST DIS SCI, V54, P2330, DOI 10.1007/s10620-008-0651-x
Riemann JF, 2010, NAT REV GASTRO HEPAT, V7, P308, DOI 10.1038/nrgastro.2010.65
Sánchez-Aldehuelo R, 2022, GASTROINTEST ENDOSC, V96, P1012, DOI 10.1016/j.gie.2022.07.018
Saxena P, 2015, DIGEST ENDOSC, V27, P265, DOI 10.1111/den.12394
Schmitz D, 2022, PLOS ONE, V17, DOI 10.1371/journal.pone.0275029
Scopelliti F, 2018, SURG ENDOSC, V32, P4022, DOI 10.1007/s00464-018-6217-x
Sharaiha RZ, 2017, GASTROINTEST ENDOSC, V85, P904, DOI 10.1016/j.gie.2016.12.023
Sharaiha Reem Z, 2013, Gastrointest Endosc Clin N Am, V23, P917, DOI 10.1016/j.giec.2013.06.010
Shrikhande SV, 2012, HPB, V14, P658, DOI 10.1111/j.1477-2574.2012.00508.x
Song TJ, 2016, GASTROINTEST ENDOSC, V83, P440, DOI 10.1016/j.gie.2015.08.048
Subramani VN, 2022, J GASTROINTEST CANC, V53, P543, DOI 10.1007/s12029-021-00655-5

Swarm RA, 2019, J NATL COMPR CANC NE, V17, P977, DOI 10.6004/jnccn.2019.0038
Teoh AYB, 2018, GUT, V67, P1209, DOI 10.1136/gutjnl-2017-314341
Teoh AYB, 2023, GASTROENTEROLOGY, V165, P473, DOI 10.1053/j.gastro.2023.04.016
Troncone E, 2020, WORLD J GASTROENTERO, V26, P1847, DOI 10.3748/wjg.v26.i16.1847
Tsuchiya T, 2018, GASTROINTEST ENDOSC, V87, P1138, DOI 10.1016/j.gie.2017.08.017
Tyberg A, 2020, ENDOSC ULTRASOUND, V9, P392, DOI 10.4103/eus.eus_42_20
van der Horst A, 2014, INT J RADIAT ONCOL, V89, P641, DOI 10.1016/j.ijrobp.2014.03.029
van der Merwe SW, 2022, ENDOSCOPY, V54, P185, DOI 10.1055/a-1717-1391
van Wanrooij RLJ, 2022, ENDOSCOPY, V54, P1023, DOI 10.1055/a-1782-7568
van Wanrooij RLJ, 2022, ENDOSCOPY, V54, P310, DOI 10.1055/a-1738-6780
Vanella G, 2023, DIGEST ENDOSC, V35, P377, DOI 10.1111/den.14445
Vanella G, 2023, ENDOSC ULTRASOUND, V12, P16, DOI 10.4103/EUS-D-21-00177
Vincent A, 2011, LANCET, V378, P607, DOI 10.1016/S0140-6736(10)62307-0
Wang KX, 2016, GASTROINTEST ENDOSC, V83, P1218, DOI 10.1016/j.gie.2015.10.033
Wiersema MJ, 1996, GASTROINTEST ENDOSC, V44, P656, DOI 10.1016/S0016-5107(96)70047-0
Wyse JM, 2017, ENDOSC ULTRASOUND, V6, P369, DOI 10.4103/eus.eus_97_17
Wyse JM, 2011, J CLIN ONCOL, V29, P3541, DOI 10.1200/JCO.2010.32.2750
Yamamoto Y, 2020, ENDOSC ULTRASOUND, V9, P110, DOI 10.4103/eus.eus_68_19
Yasuda I, 2017, DIGEST ENDOSC, V29, P455, DOI 10.1111/den.12824
Zakaria A, 2022, CANCERS, V14, DOI 10.3390/cancers14061373
Zeyaullah M, 2012, PATHOL ONCOL RES, V18, P771, DOI 10.1007/s12253-012-9548-2
NR 143
TC 1
Z9 1
U1 1
U2 4
PU FRONTIERS MEDIA SA
PI LAUSANNE
PA AVENUE DU TRIBUNAL FEDERAL 34, LAUSANNE, CH-1015, SWITZERLAND
SN 2234-943X
J9 FRONT ONCOL
JI Front. Oncol.
PD SEP 15
PY 2023
VL 13
AR 1252824
DI 10.3389/fonc.2023.1252824
PG 15
WC Oncology
WE Science Citation Index Expanded (SCI-EXPANDED)
SC Oncology
GA S7RB3

UT WOS:001073094300001
PM 37781196
OA Green Submitted, gold
DA 2026-05-09
ER

PT J
AU Sundaram, S
Kale, A
AF Sundaram, Sridhar
Kale, Aditya
TI Endoscopic ultrasound guided biliary drainage in surgically altered
anatomy: A comprehensive review of various approaches
SO WORLD JOURNAL OF GASTROINTESTINAL ENDOSCOPY
LA English
DT Review
DE Endoscopic ultrasound guided biliary drainage; Surgically altered
anatomy; Endoscopic retrograde cholangiopancreatography; Endoscopic
ultrasound; Stents; Intervention
ID ENTEROSCOPY-ASSISTED ERCP; OBSTRUCTION; HEPATICOGASTROSTOMY;
MULTICENTER; BALLOON; STONE
AB Endoscopic retrograde cholangiopancreatography (ERCP) is the preferred modality for drainage of the obstructed biliary tree. In patients with surgically altered anatomy, ERCP using standard techniques may not be feasible. Enteroscope assisted ERCP is usually employed with variable success rate. With advent of endoscopic ultrasound (EUS), biliary drainage procedures in patients with biliary obstruction and surgically altered anatomy is safe and effective. In this narrative review, we discuss role of EUS guided biliary drainage in patients with altered anatomy and the various approaches used in patients with benign and malignant biliary obstruction.
C1 [Sundaram, Sridhar] Homi Bhabha Natl Inst Mumbai, Tata Mem Hosp, Dept Digest Dis & Clin Nutr, Mumbai 400012, Maharashtra, India.
[Kale, Aditya] Seth GS Med Coll & King Edward Mem Hosp, Dept Gastroenterol, Mumbai 400012, India.
[Sundaram, Sridhar] Homi Bhabha Natl Inst Mumbai, Tata Mem Hosp, Dept Digest Dis & Clin Nutr, Dr E Borges Rd, Mumbai 400012, Maharashtra, India.
C3 Tata Memorial Centre (TMC); Tata Memorial Hospital; Homi Bhabha National
Institute; Seth Gordhandas Sunderdas Medical College & King Edward
Memorial Hospital; Homi Bhabha National Institute; Tata Memorial Centre
(TMC); Tata Memorial Hospital
RP Sundaram, S (corresponding author), Homi Bhabha Natl Inst Mumbai, Tata Mem Hosp, Dept Digest Dis & Clin Nutr, Dr E Borges Rd, Mumbai 400012, Maharashtra, India.
EM drsridharsundaram@kem.edu
RI Kale, Aditya/KHW-7676-2024; S, Sridhar/R-6416-2017
CR Bukhari M, 2018, GASTROINTEST ENDOSC, V88, P486, DOI 10.1016/j.gie.2018.04.2356
Hosmer A, 2018, ENDOSC INT OPEN, V6, pE127, DOI 10.1055/s-0043-123188

Huang P, 2020, SURG LAPARO ENDO PER, V30, P454, DOI 10.1097/SLE.0000000000000813
Ichkhanian Y, 2020, GASTROINTEST ENDOSC, V91, P1188, DOI 10.1016/j.gie.2019.12.043
Itoi T, 2014, J HEPATO-BIL-PAN SCI, V21, pE86, DOI 10.1002/jhbp.165
Iwashita T, 2020, J HEPATO-BIL-PAN SCI, V27, P968, DOI 10.1002/jhbp.823
Iwashita T, 2016, J HEPATO-BIL-PAN SCI, V23, P227, DOI 10.1002/jhbp.329
Iwashita T, 2013, DIGEST DIS SCI, V58, P2417, DOI 10.1007/s10620-013-2645-6
James TW, 2018, GASTROINTEST ENDOSC, V88, P547, DOI 10.1016/j.gie.2018.04.2353
Jovani M, 2019, ENDOSC ULTRASOUND, V8, pS57, DOI 10.4103/eus.eus_53_19
Katanuma A, 2020, DIGEST ENDOSC, V32, P263, DOI 10.1111/den.13567
Kedia P, 2019, J CLIN GASTROENTEROL, V53, P304, DOI 10.1097/MCG.0000000000001037
Kedia P, 2015, ENDOSCOPY, V47, P159, DOI 10.1055/s-0034-1390771
Khara Harshit S, 2021, Curr Gastroenterol Rep, V23, P10, DOI [10.1007/s11894-021-00808-3, 10.1007/s11894-021-00808-3]
Khashab MA, 2016, ENDOSC INT OPEN, V4, pE1322, DOI 10.1055/s-0042-110790
Khashab MA, 2013, GASTROINTEST ENDOSC, V78, P734, DOI 10.1016/j.gie.2013.05.013
Law R, 2016, CURR OPIN GASTROEN, V32, P232, DOI 10.1097/MOG.0000000000000255
Lee Alexander, 2013, Gastrointest Endosc Clin N Am, V23, P483, DOI 10.1016/j.giec.2012.12.005
Martin A, 2016, ENDOSC ULTRASOUND, V5, P149, DOI 10.4103/2303-9027.183969
Minaga K, 2020, THER ADV GASTROENTER, V13, DOI 10.1177/1756284820930964
Miranda-García P, 2016, ENDOSC INT OPEN, V4, pE461, DOI 10.1055/s-0042-103241
Mukai S, 2019, GASTROINTEST ENDOSC, V89, P399, DOI 10.1016/j.gie.2018.07.030
Nakai Y, 2019, CLIN ENDOSC, V52, P212, DOI 10.5946/ce.2018.188
Park SJ, 2013, GASTROINTEST ENDOSC, V78, P374, DOI 10.1016/j.gie.2013.04.183
Schreiner MA, 2012, GASTROINTEST ENDOSC, V75, P748, DOI 10.1016/j.gie.2011.11.019
Shah RJ, 2013, GASTROINTEST ENDOSC, V77, P593, DOI 10.1016/j.gie.2012.10.015
Skinner M, 2014, ENDOSCOPY, V46, P560, DOI 10.1055/s-0034-1365698
Sundaram S., 2022, DIGEST DIS SCI
Weilert F, 2011, ENDOSCOPY, V43, P1105, DOI 10.1055/s-0030-1256961
NR 29
TC 12
Z9 13
U1 1
U2 4
PU BAISHIDENG PUBLISHING GROUP INC
PI PLEASANTON
PA 7041 Koll Center Parkway, Suite 160, PLEASANTON, CA, UNITED STATES
SN 1948-5190
J9 WORLD J GASTRO ENDOS
JI World J. Gastrointest. Endosc.
PD MAR 16
PY 2023
VL 15

IS 3
BP 122
EP 132
DI 10.4253/wjge.v15.i3.122
PG 11
WC Gastroenterology & Hepatology
WE Emerging Sources Citation Index (ESCI)
SC Gastroenterology & Hepatology
GA F4NW4
UT WOS:000982140900004
PM 37034975
OA Green Submitted, gold
DA 2026-05-09
ER

PT J
AU Kim, BK
Chun, JW
Lee, SH
Ryu, JK
Kim, YT
Paik, WH
AF Kim, Bo Kyung
Chun, Jung Won
Lee, Sang Hyub
Ryu, Ji Kon
Kim, Yong-Tae
Paik, Woo Hyun
TI A Remnant Choledochal Cyst after Choledochal. Cyst Excision Treated with
a Lumen-Apposing Metal Stent: A Case Report
SO CLINICAL ENDOSCOPY
LA English
DT Article
DE Choledochal cyst; Endosonography; Self-expandable metallic steins
ID PANCREATIC FLUID COLLECTIONS; MANAGEMENT; DRAINAGE; ADULTS
AB A lumen-apposing metal stent (LAMS) is a saddle-shaped stent with large flanges at both ends, thereby preventing stent migration and helping with approximation of the adjacent structures. We report the case of a 25-year-old female with remnant choledochal cyst which was successfully treated with LAMS after initial treatment failure with a plastic stent. Although complete excision of the cyst is the definite treatment of choledochal cysts, endoscopic ultrasonography-guided cystoduodenostomy can be considered in cases wherein surgery is not feasible and dysplasia is not present. LAMS may be preferred to plastic stents for effective resolution of remnant choledochal cyst and prevention of ascending infection.
C1 [Paik, Woo Hyun] Seoul Natl Univ, Seoul Natl Univ Hosp, Coll Med, Dept Internal Med, 101 Daehak Ro, Seoul 03080,

South Korea.
[Paik, Woo Hyun] Seoul Natl Univ, Seoul Natl Univ Hosp, Coll Med, Liver Res Inst, 101 Daehak Ro, Seoul 03080, South Korea.
C3 Seoul National University (SNU); Seoul National University Hospital;
Seoul National University (SNU); Seoul National University Hospital
RP Paik, WH (corresponding author), Seoul Natl Univ, Seoul Natl Univ Hosp, Coll Med, Dept Internal Med, 101 Daehak Ro, Seoul 03080, South Korea.; Paik, WH (corresponding author), Seoul Natl Univ, Seoul Natl Univ Hosp, Coll Med, Liver Res Inst, 101 Daehak Ro, Seoul 03080, South Korea.
EM whpaik@snuh.org
RI Paik, Woo Hyun/B-9003-2016
OI Paik, Woo Hyun/0000-0001-8708-3280; Lee, Sang Hyub/0000-0003-2174-9726;
Kim, Bokyung/0000-0002-9143-9654; Ryu, Ji Kon/0000-0001-8798-0491; Chun,
Jung Won/0000-0003-1964-7501
CR Bang JY, 2019, GUT, V68, P1200, DOI 10.1136/gutjnl-2017-315335
Bank JS, 2017, INT J GASTROINT INT, V6, P9, DOI 10.18528/gii160033
Brimhall B, 2018, CLIN GASTROENTEROL H, V16, P1521, DOI 10.1016/j.cgh.2018.02.021
DeSimone ML, 2017, WORLD J GASTRO ENDOS, V9, P456, DOI 10.4253/wjge.v9.i9.456
Guo JT, 2016, SURG ENDOSC, V30, P3114, DOI 10.1007/s00464-015-4557-3
Hammad T, 2018, DIGEST DIS SCI, V63, P289, DOI 10.1007/s10620-017-4851-0
Khashab MA, 2016, ENDOSC INT OPEN, V4, pE175, DOI 10.1055/s-0041-109083
Lee SE, 2011, ARCH SURG-CHICAGO, V146, P1178, DOI 10.1001/archsurg.2011.243
Mussetto A, 2018, ANN GASTROENTEROL, V31, P535, DOI 10.20524/aog.2018.0287
Singham J, 2010, CAN J SURG, V53, P51
Singham Janakie, 2007, HPB (Oxford), V9, P383, DOI 10.1080/13651820701646198
Soreide K, 2007, ANN SURG ONCOL, V14, P1200, DOI 10.1245/s10434-006-9294-3
Tan MY, 2018, HPB, V20, pS751, DOI 10.1016/j.hpb.2018.06.1536
TODANI T, 1979, CANCER-AM CANCER SOC, V44, P1134, DOI 10.1002/1097-0142(197909)44:3<1134::AID-CNCR2820440350>3.0.CO;2-T
Todani Takuji, 2003, J Hepatobiliary Pancreat Surg, V10, P340, DOI 10.1007/s00534-002-0733-7
Wang KX, 2016, GASTROINTEST ENDOSC, V83, P1218, DOI 10.1016/j.gie.2015.10.033
Xia HT, 2016, SURGERY, V159, P418, DOI 10.1016/j.surg.2015.04.042
Yirgin H, 2017, CAUSAPEDIA, V6, P170
NR 18
TC 1
Z9 1
U1 0
U2 0
PU KOREAN SOC GASTROINTESTINAL ENDOSCOPY
PI SEOUL
PA 2003 LG PALACE, 165-8 DONGGYO-DONG, MAPO-GU, SEOUL, 121-754, SOUTH KOREA
SN 2234-2400
EI 2234-2443

J9 CLIN ENDOSC
JI Clin. Endosc.
PD JUL
PY 2022
VL 55
IS 4
BP 564
EP 569
DI 10.5946/ce.2019.176
EA JAN 2020
PG 6
WC Gastroenterology & Hepatology
WE Emerging Sources Citation Index (ESCI)
SC Gastroenterology & Hepatology
GA 3L4OF
UT WOS:000805989600001
PM 31910611
OA Green Submitted, gold
DA 2026-05-09
ER

PT J
AU Yamao, K
Hara, K
Mizuno, N
Sawaki, A
Hijioka, S
Niwa, Y
Tajika, M
Kawai, H
Kondo, S
Shimizu, Y
Bhatia, V
AF Yamao, Kenji
Hara, Kazuo
Mizuno, Nobumasa
Sawaki, Akira
Hijioka, Susumu
Niwa, Yasumasa
Tajika, Masahiro
Kawai, Hiroki
Kondo, Shinya

Shimizu, Yasuhiro
Bhatia, Vikram
TI EUS-Guided Biliary Drainage
SO GUT AND LIVER
LA English
DT Review
DE Endoscopic ultrasonography; Endoscopic ultrasonography-guided biliary drainage; Rendezvous technique; Endoscopic ultrasonography-guided choledochoduodenostomy; Endoscopic ultrasonography-guided hepaticogastrostomy
ID ENDOSCOPIC ULTRASONOGRAPHY; TRANSHEPATIC CHOLANGIOGRAPHY; CASE SERIES; BILE-DUCT; OBSTRUCTION; CHOLEDOCHODUODENOSTOMY
AB Endoscopic ultrasonography (EUS) combines endoscopy and intraluminal ultrasonography, and allows imaging with a high-frequency transducer over a short distance to generate high-resolution ultrasonographic images. EUS is now a widely accepted modality for diagnosing pancreatobiliary diseases. EUS-guided fine-needle aspiration (EUS-FNA) using a curved linear-array echoendoscope was initially described more than 20 years ago, and since then many researchers have expanded its indications to sample diverse lesions and have also used it for various therapeutic purposes. EUS-guided biliary drainage (EUS-BD) is one of the therapeutic procedures that has been developed using a curved linear-array echoendoscope. Technically, EUS-BD includes rendezvous techniques via transesophageal, transgastric, and transduodenal routes, EUS-guided choledochoduodenostomy (EUS-CDS), and EUS-guided hepaticogastrostomy (EUS-HGS). Published data have demonstrated a high success rate, albeit with a comparatively high rate of nonfatal complications for EUS-CDS and EUS-HGS, and a comparatively low success rate with a low complication rate for the rendezvous technique. At present, these procedures represent an alternative to surgery or percutaneous transhepatic biliary drainage (PTBD) for patients with obstructive jaundice when endoscopic biliary drainage (EBD) has failed. However, these procedures should be performed in centers with extensive experience in linear EUS and therapeutic biliary ERCP. Large prospective studies are needed in the near future to establish standardized EUS-BD procedures as well as to perform controlled comparative trials between EUS-BD and PTBD, between rendezvous techniques and direct-access techniques (EUS-CDS and EUS-HGS), and between EBD and EUS-BD. (Gut Liver 2010;4(Suppl. 1):S67-75)
C1 [Yamao, Kenji] Aichi Canc Ctr Hosp, Dept Gastroenterol, Chikusa Ku, Nagoya, Aichi 4648681, Japan.
[Niwa, Yasumasa; Tajika, Masahiro; Kawai, Hiroki; Kondo, Shinya] Aichi Canc Ctr Hosp, Dept Endoscopy, Nagoya, Aichi 4648681, Japan.
[Shimizu, Yasuhiro] Aichi Canc Ctr Hosp, Dept Gastrointestinal Surg, Nagoya, Aichi 4648681, Japan.
[Bhatia, Vikram] Inst Liver & Biliary Sci, Dept Med Hepatol, Delhi, India.
C3 Aichi Cancer Center; Aichi Cancer Center; Aichi Cancer Center; Institute of Liver & Biliary Sciences (ILBS)
RP Yamao, K (corresponding author), Aichi Canc Ctr Hosp, Dept Gastroenterol, Chikusa Ku, 1-1 Kanokoden, Nagoya, Aichi 4648681, Japan.
EM kyamao@aichi-cc.jp
RI Hijioka, Susumu/I-1039-2019; Bhatia, Vikram/JUU-4166-2023
OI Hijioka, Susumu/0000-0002-0394-6876;
CR Ang TL, 2007, J PANCREAS, V8, P438
Artifon ELA, 2007, CLINICS, V62, P799, DOI 10.1590/S1807-59322007000600023

Bhutani MS, 1999, INTERVENTIONAL ENDOS, P65
Bories E, 2007, ENDOSCOPY, V39, P287, DOI 10.1055/s-2007-966212
Burmester E, 2003, GASTROINTEST ENDOSC, V57, P246, DOI 10.1067/mge.2003.85
Fujita N, 2007, WORLD J GASTROENTERO, V13, P5512, DOI 10.3748/wjg.v13.i41.5512
Giovannini M, 2001, ENDOSCOPY, V33, P898, DOI 10.1055/s-2001-17324
Hanada K, 2009, DIGEST ENDOSC, V21, pS75, DOI 10.1111/j.1443-1661.2009.00855.x
Hara K, 2010, DIGEST ENDOSC, V22, P147, DOI 10.1111/j.1443-1661.2010.00944.x
Itoi T, 2010, J HEPATO-BIL-PAN SCI, V17, P611, DOI 10.1007/s00534-009-0196-1
Kahaleh M, 2006, GASTROINTEST ENDOSC, V64, P52, DOI 10.1016/j.gie.2006.01.063
Kahaleh M, 2004, GASTROINTEST ENDOSC, V60, P138, DOI 10.1016/S0016-5107(04)01528-7
Kahaleh M, 2005, GASTROINTEST ENDOSC, V61, P307, DOI 10.1016/S0016-5107(04)02585-4
Kim YS, 2010, ENDOSCOPY, V42, P496, DOI 10.1055/s-0029-1244082
Lai R, 2005, ENDOSCOPY, V37, P487, DOI 10.1055/s-2005-861250
Mallery S, 2004, GASTROINTEST ENDOSC, V59, P100, DOI 10.1016/S0016-5107(03)02300-9
Maranki J, 2009, ENDOSCOPY, V41, P532, DOI 10.1055/s-0029-1214712
Park DH, 2009, AM J GASTROENTEROL, V104, P2168, DOI 10.1038/ajg.2009.254
Püspök A, 2005, AM J GASTROENTEROL, V100, P1743, DOI 10.1111/j.1572-0241.2005.41806.x
Tarantino I, 2008, ENDOSCOPY, V40, P336, DOI 10.1055/s-2007-995455
VILMANN P, 1992, GASTROINTEST ENDOSC, V38, P172, DOI 10.1016/S0016-5107(92)70385-X
Wiersema MJ, 1996, GASTROINTEST ENDOSC, V43, P102, DOI 10.1016/S0016-5107(06)80108-2
Will U, 2007, ENDOSCOPY, V39, P292, DOI 10.1055/s-2007-966215
Will U, 2007, J HEPATO-BILIARY-PAN, V14, P377, DOI 10.1007/s00534-006-1139-8
Yamao K, 2008, ENDOSCOPY, V40, P340, DOI 10.1055/s-2007-995485
YAMAO K, 2006, SUIZO, V21, P353
Yamao K, 2006, GASTROINTEST ENDOSC, V64, P663, DOI 10.1016/j.gie.2006.07.003
NR 27
TC 31
Z9 36
U1 0
U2 0
PU EDITORIAL OFFICE GUT & LIVER
PI SEOUL
PA 305 LOTTE GOLD ROSE II, 890-59, DAECHI 4-DONG, GANGNAM-GU, SEOUL,
135-839, SOUTH KOREA
SN 1976-2283
J9 GUT LIVER
JI Gut Liver
PD SEP 30
PY 2010
VL 4
SU 1
BP S67

EP S75
DI 10.5009/gnl.2010.4.S1.S67
PG 9
WC Gastroenterology & Hepatology
WE Science Citation Index Expanded (SCI-EXPANDED)
SC Gastroenterology & Hepatology
GA 651MV
UT WOS:000281932700011
PM 21103298
OA Green Submitted, gold
DA 2026-05-09
ER

PT J
AU She, YM
Ge, N
AF She, Yu Mo
Ge, Nan
TI The value of endoscopic ultrasonography for differential diagnosis in obstructive jaundice of the distal common bile duct
SO EXPERT REVIEW OF GASTROENTEROLOGY & HEPATOLOGY
LA English
DT Review
DE Computed tomography; distal obstructive jaundice; endoscopic ultrasonography; magnetic resonance imaging; ultrasound
ID FINE-NEEDLE-ASPIRATION; GUIDED BILIARY DRAINAGE; MAGNETIC-RESONANCE CHOLANGIOGRAPHY; SOLID PANCREATIC LESIONS; AUTOIMMUNE PANCREATITIS; EUS-FNA; COMPUTED-TOMOGRAPHY; RETROGRADE CHOLANGIOPANCREATOGRAPHY; TRANSABDOMINAL SONOGRAPHY; ULTRASOUND ELASTOGRAPHY
AB Introduction Obstructive jaundice is a common clinical disease of great significance; however, diagnosing it according to etiology, especially in patients with distal obstructive jaundice is difficult. The development of endoscopic ultrasonography has improved diagnostic methods. Endoscopic ultrasonography not only improves the accuracy of conventional endoscopic ultrasound technology in etiological diagnosis, but also offers several special endoscopic ultrasound technologies for diagnosing distal obstructive jaundice of the common bile duct. What's more, endoscopic ultrasonography can be used to treat distal obstructive jaundice of common bile duct. Areas covered This review discusses the diagnostic value and applications of endoscopic ultrasonography for obstructive jaundice of the distal common bile duct. Expert opinion This article summarizes the value of endoscopic ultrasonography in the etiological diagnosis, relevant treatment applications of distal obstructive jaundice and the limitations of endoscopic ultrasonography in some etiologies due to the lack of clear comparison with other imaging methods. We also provide new data for the future research direction of endoscopic ultrasonography in distal obstructive jaundice.
C1 [She, Yu Mo; Ge, Nan] China Med Univ, Shengjing Hosp, Dept Gastroenterol, Shenyang, Liaoning, Peoples R China.
C3 China Medical University

RP Ge, N (corresponding author), China Med Univ, Shengjing Hosp, Dept Gastroenterol, Shenyang, Liaoning, Peoples R China.
EM nge@cmu.edu.cn
RI Ge, Nan/AAM-2115-2020
CR Agarwal B, 2004, AM J GASTROENTEROL, V99, P844, DOI 10.1111/j.1572-0241.2004.04177.x
Ainsworth AP, 2003, ENDOSCOPY, V35, P1029, DOI 10.1055/s-2003-44603
Akahoshi K, 1998, BRIT J RADIOL, V71, P492, DOI 10.1259/bjr.71.845.9691893
AMOUYAL P, 1994, GASTROENTEROLOGY, V106, P1062, DOI 10.1016/0016-5085(94)90768-4
Arya N, 2020, ENDOSC ULTRASOUND, V9, P154, DOI 10.4103/eus.eus_28_20
Bang JY, 2017, DIGEST ENDOSC, V29, P338, DOI 10.1111/den.12769
Bohra AK, 2002, ULSTER MED J, V71, P121
Borbath I, 2005, PANCREATOLOGY, V5, P553, DOI 10.1159/000087497
Bunganic B, 2018, SCAND J GASTROENTERO, V53, P1411, DOI 10.1080/00365521.2018.1524023
Buxbaum J, 2020, CLIN GASTROENTEROL H, V18, P917, DOI 10.1016/j.cgh.2019.08.054
Cannon ME, 1999, GASTROINTEST ENDOSC, V50, P27, DOI 10.1016/S0016-5107(99)70340-8
Canto MIF, 1998, GASTROINTEST ENDOSC, V47, P439, DOI 10.1016/S0016-5107(98)70242-1
Cao L, 2018, SCAND J GASTROENTERO, V53, P1604, DOI 10.1080/00365521.2018.1534137
Chak A, 1999, GASTROINTEST ENDOSC, V49, P599, DOI 10.1016/S0016-5107(99)70388-3
Chen CH, 2001, J CLIN ULTRASOUND, V29, P313, DOI 10.1002/jcu.1041
Chen CH, 2009, J CLIN ULTRASOUND, V37, P18, DOI 10.1002/jcu.20523
Chen J, 2012, J CANCER RES CLIN, V138, P1433, DOI 10.1007/s00432-012-1268-1
Chhoda A, 2020, CLIN J GASTROENTEROL, V13, P669, DOI 10.1007/s12328-020-01153-0
Cho IR, 2021, GASTROINTEST ENDOSC, V94, P303, DOI 10.1016/j.gie.2021.01.018
Chu YL, 2013, EUR J GASTROEN HEPAT, V25, P455, DOI 10.1097/MEG.0b013e32835ca1d7
Crinò SF, 2018, HEPATOB PANCREAT DIS, V17, P70, DOI 10.1016/j.hbpd.2018.01.010
de Lédinghen V, 1999, GASTROINTEST ENDOSC, V49, P26, DOI 10.1016/S0016-5107(99)70441-4
DeWitt J, 2006, GASTROINTEST ENDOSC, V64, P325, DOI 10.1016/j.gie.2005.11.064
Dewitt J, 2006, CLIN GASTROENTEROL H, V4, P717, DOI 10.1016/j.cgh.2006.02.020
Dhindsa BS, 2020, ENDOSC ULTRASOUND, V9, P101, DOI 10.4103/eus.eus_80_19
Dickerson LD, 2019, WORLD J SURG, V43, P1604, DOI 10.1007/s00268-019-04928-w
Dietrich CF, 2009, ENDOSCOPY, V41, P718, DOI 10.1055/s-0029-1214866
Dietrich CF, 2022, ENDOSC ULTRASOUND, V11, P27, DOI 10.4103/EUS-D-21-00102
Dietrich CF, 2020, ENDOSC ULTRASOUND, V9, P284, DOI 10.4103/eus.eus_25_20
Ding H, 2019, ANN TRANSL MED, V7, DOI 10.21037/atm.2019.06.51
Du C, 2022, ENDOSC ULTRASOUND, V11, P44, DOI 10.4103/EUS-D-20-00231
Duan F, 2017, CANCER IMAGING, V17, DOI 10.1186/s40644-017-0129-1
Dubay D, 2010, CLIN GASTROENTEROL H, V8, pXXII, DOI 10.1016/j.cgh.2010.02.006
Eloubeidi MA, 2006, GASTROINTEST ENDOSC, V63, P622, DOI 10.1016/j.gie.2005.05.024
Eloubeidi MA, 2004, CLIN GASTROENTEROL H, V2, P209, DOI 10.1016/S1542-3565(04)00005-9
Fritscher-Ravens A, 2004, AM J GASTROENTEROL, V99, P45, DOI 10.1046/j.1572-0241.2003.04006.x
Fujimoto K, 2014, DIGEST ENDOSC, V26, P1, DOI 10.1111/den.12183
Giovannini M, 2009, WORLD J GASTROENTERO, V15, P1587, DOI 10.3748/wjg.15.1587

Guo JT, 2020, ENDOSC ULTRASOUND, V9, P319, DOI 10.4103/eus.eus_56_20
Guo T, 2021, ENDOSC ULTRASOUND, V10, P280, DOI 10.4103/EUS-D-20-00212
Hafezi-Nejad N, 2018, MAGN RESON IMAGING C, V26, P463, DOI 10.1016/j.mric.2018.03.008
Hayat U, 2022, ENDOSC ULTRASOUND, V11, P4, DOI 10.4103/EUS-D-21-00009
Hernandez LV, 2008, CURR OPIN GASTROEN, V24, P617, DOI 10.1097/MOG.0b013e3283088e12
Hewitt MJ, 2012, GASTROINTEST ENDOSC, V75, P319, DOI 10.1016/j.gie.2011.08.049
Hirche TO, 2008, ENDOSCOPY, V40, P910, DOI 10.1055/s-2008-1077726
Hocke M, 2006, WORLD J GASTROENTERO, V12, P246, DOI 10.3748/wjg.v12.i2.246
Moura DTH, 2018, ENDOSC INT OPEN, V6, pE769, DOI 10.1055/s-0043-123186
Hua YP, 2009, HEPATOB PANCREAT DIS, V8, P79
Imazu H, 2012, SCAND J GASTROENTERO, V47, P853, DOI 10.3109/00365521.2012.679686
Isayama H, 2019, J HEPATO-BIL-PAN SCI, V26, P249, DOI 10.1002/jhbp.631
Ishikawa T, 2020, ENDOSCOPY, V52, P978, DOI 10.1055/a-1183-3583
Ishikawa T, 2019, CAN J GASTROENTEROL, V2019, DOI 10.1155/2019/8581743
Kamata K, 2014, ENDOSCOPY, V46, P22, DOI 10.1055/s-0033-1353603
Kanno A, 2016, GASTROINTEST ENDOSC, V84, P797, DOI 10.1016/j.gie.2016.03.1511
Kanno A, 2012, GASTROINTEST ENDOSC, V76, P594, DOI 10.1016/j.gie.2012.05.014
Karakan T, 2009, GASTROINTEST ENDOSC, V69, P244, DOI 10.1016/j.gie.2008.05.023
Khashab MA, 2015, DIGEST DIS SCI, V60, P557, DOI 10.1007/s10620-014-3300-6
Kitano M, 2004, GUT, V53, P854, DOI 10.1136/gut.2003.029934
Kitano M, 2019, J GASTROENTEROL, V54, P19, DOI 10.1007/s00535-018-1519-2
Kubo H, 1999, BRIT J RADIOL, V72, P443, DOI 10.1259/bjr.72.857.10505006
LAING FC, 1986, RADIOLOGY, V160, P39, DOI 10.1148/radiology.160.1.3012631
Lee TH, 2016, CLIN GASTROENTEROL H, V14, P1011, DOI 10.1016/j.cgh.2015.12.032
Legmann P, 1998, AM J ROENTGENOL, V170, P1315, DOI 10.2214/ajr.170.5.9574609
Leng Jian-Jun, 2014, World J Surg Oncol, V12, P272
Lovecek M, 2012, Rozhl Chir, V91, P608
Lutz HH, 2012, ENDOSCOPY, V44, P934, DOI 10.1055/s-0032-1309896
Manta R, 2010, WORLD J GASTROENTERO, V16, P5592, DOI 10.3748/wjg.v16.i44.5592
Manu NK, 2011, HEPATO-GASTROENTEROL, V58, P1862, DOI 10.5754/hge10531
MCDERMOTT VG, 1995, AM J ROENTGENOL, V165, P301, DOI 10.2214/ajr.165.2.7618544
Meara RS, 2006, CYTOPATHOLOGY, V17, P42, DOI 10.1111/j.1365-2303.2006.00319.x
Mesenas S, 2006, GASTROINTEST ENDOSC, V63, P403, DOI 10.1016/j.gie.2005.10.040
Mohamadnejad M, 2011, GASTROINTEST ENDOSC, V73, P71, DOI 10.1016/j.gie.2010.08.050
Moon Sung-Hoon, 2013, Gastrointest Endosc Clin N Am, V23, P893, DOI 10.1016/j.giec.2013.06.005
Moon SH, 2010, GASTROINTEST ENDOSC, V71, P960, DOI 10.1016/j.gie.2009.12.004
Morishima T, 2016, GASTROINTEST ENDOSC, V84, P241, DOI 10.1016/j.gie.2016.01.016
MULLER MF, 1994, RADIOLOGY, V190, P745
Nakatsubo R, 2021, ENDOSC ULTRASOUND, V10, P207, DOI 10.4103/EUS-D-20-00174
Netinatsunton N, 2016, ENDOSC ULTRASOUND, V5, P165, DOI 10.4103/2303-9027.183971
Noguchi K, 2020, DIAGNOSTICS, V10, DOI 10.3390/diagnostics10110954
Notohara K, 2020, PANCREATOLOGY, V20, P834, DOI 10.1016/j.pan.2020.05.026

Ogura T, 2021, ENDOSC ULTRASOUND, V10, P305, DOI 10.4103/EUS-D-20-00194
Ogura T, 2019, DIGEST LIVER DIS, V51, P247, DOI 10.1016/j.dld.2018.09.015
Ogura T, 2012, ENDOSC ULTRASOUND, V1, P84, DOI 10.7178/eus.02.006
Okabe Y, 2012, J HEPATO-BIL-PAN SCI, V19, P266, DOI 10.1007/s00534-011-0392-7
Onoyama T, 2019, J CLIN MED, V8, DOI 10.3390/jcm8040417
Oppong K, 2010, J PANCREAS, V11, P560
PALAZZO L, 1995, GASTROINTEST ENDOSC, V42, P225, DOI 10.1016/S0016-5107(95)70096-X
Prachayakul V, 2014, BMC GASTROENTEROL, V14, DOI 10.1186/1471-230X-14-165
Prat F, 1996, LANCET, V347, P75, DOI 10.1016/S0140-6736(96)90208-1
Puli SR, 2013, PANCREAS, V42, P20, DOI 10.1097/MPA.0b013e3182546e79
Rahman MIO, 2020, SAUDI J GASTROENTERO, V26, P113, DOI 10.4103/sjg.SJG_39_20
RISCHIN D, 1988, AUST NZ J MED, V18, P736, DOI 10.1111/j.1445-5994.1988.tb00169.x
Rizzo A, 2020, IN VIVO, V34, P1701, DOI 10.21873/invivo.11964
Roberts KJ, 2013, HPB, V15, P18, DOI 10.1111/j.1477-2574.2012.00542.x
ROSCH T, 1992, GASTROENTEROLOGY, V102, P188
Rustemovic N, 2010, EUR J GASTROEN HEPAT, V22, P748, DOI 10.1097/MEG.0b013e32832d489f
Saftoiu A, 2008, GASTROINTEST ENDOSC, V68, P1086, DOI 10.1016/j.gie.2008.04.031
Sakamoto H, 2008, ULTRASOUND MED BIOL, V34, P525, DOI 10.1016/j.ultrasmedbio.2007.09.018
Seicean A, 2020, ENDOSCOPY, V52, P1084, DOI 10.1055/a-1193-4954
Seicean A, 2010, WORLD J GASTROENTERO, V16, P4253, DOI 10.3748/wjg.v16.i34.4253
Seifert H, 2008, Z GASTROENTEROL, V46, P909, DOI 10.1055/s-2008-1027569
Seifert H, 2021, ENDOSC ULTRASOUND, V10, P246, DOI 10.4103/EUS-D-20-00252
Sharaiha RZ, 2016, SURG ENDOSC, V30, P5500, DOI 10.1007/s00464-016-4913-y
Singh VK, 2019, JAMA-J AM MED ASSOC, V322, P2422, DOI 10.1001/jama.2019.19411
Skordilis Panagiotis, 2002, BMC Surg, V2, P1, DOI 10.1186/1471-2482-2-1
SNADY H, 1992, GASTROINTEST ENDOSC, V38, P27, DOI 10.1016/S0016-5107(92)70326-5
Sotoudehmanesh Rasoul, 2016, Middle East J Dig Dis, V8, P267, DOI 10.15171/mejdd.2016.35
Spadaccini M, 2019, AM J GASTROENTEROL, V114, P174, DOI 10.1038/s41395-018-0371-6
Strongin A, 2013, ENDOSC ULTRASOUND, V2, P71, DOI [10.4103/2303-9027.117690, 10.7178/eus.05.003]
Sugimoto M, 2017, J ULTRAS MED, V36, P2237, DOI 10.1002/jum.14265
Sugiyama M, 1997, GASTROINTEST ENDOSC, V45, P143, DOI 10.1016/S0016-5107(97)70237-2
Sugiyama M, 1997, ABDOM IMAGING, V22, P434, DOI 10.1007/s002619900227
Sugumar A, 2011, GUT, V60, P666, DOI 10.1136/gut.2010.207951
Tio TL, 1996, GASTROINTEST ENDOSC, V44, P706, DOI 10.1016/S0016-5107(96)70056-1
Tsukinaga S, 2007, WORLD J GASTROENTERO, V13, P3758
Udristoiu AL, 2021, PLOS ONE, V16, DOI 10.1371/journal.pone.0251701
Wang Long, 2014, Acta Anaesthesiol Taiwan, V52, P22, DOI 10.1016/j.aat.2014.03.002
Wang M, 2016, GASTROENT RES PRACT, V2016, DOI 10.1155/2016/6957235
Wang W, 2013, GASTROINTEST ENDOSC, V78, P73, DOI 10.1016/j.gie.2013.01.040
Weber C, 2008, ENDOSCOPY, V40, P739, DOI 10.1055/s-2008-1077509
Wiersema MJ, 1997, GASTROENTEROLOGY, V112, P1087, DOI 10.1016/S0016-5085(97)70164-1
Yamashita Y, 2019, DIGEST ENDOSC, V31, P125, DOI 10.1111/den.13290

Yang YT, 2022, ULTRASOUND Q, V38, P170, DOI 10.1097/RUQ.0000000000000553
Yoon SB, 2022, DIGEST DIS SCI, V67, P3284, DOI 10.1007/s10620-021-07138-4
Yoon SB, 2021, DIGEST ENDOSC, V33, P1024, DOI 10.1111/den.13866
Yoon WJ, 2022, FRONT ONCOL, V12, DOI 10.3389/fonc.2022.844083
Zaheer A, 2014, J COMPUT ASSIST TOMO, V38, P146, DOI 10.1097/RCT.0b013e3182a9a431
Zerem E, 2022, CLIN EXP HEPATOL, V8, P70, DOI 10.5114/ceh.2022.114190
Zhang BL, 2018, PANCREATOLOGY, V18, P833, DOI 10.1016/j.pan.2018.07.008
Zhang QL, 1996, CHINESE MED J-PEKING, V109, P622
Zhao XQ, 2015, DIGEST ENDOSC, V27, P137, DOI 10.1111/den.12320
Zidi SH, 1999, GUT, V44, P118, DOI 10.1136/gut.44.1.118
Zou D, 1995, Zhonghua Nei Ke Za Zhi, V34, P315
NR 133
TC 1
Z9 3
U1 0
U2 11
PU TAYLOR & FRANCIS LTD
PI ABINGDON
PA 2-4 PARK SQUARE, MILTON PARK, ABINGDON OR14 4RN, OXON, ENGLAND
SN 1747-4124
EI 1747-4132
J9 EXPERT REV GASTROENT
JI Expert Rev. Gastroenterol. Hepatol.
PD JUL 3
PY 2022
VL 16
IS 7
BP 653
EP 664
DI 10.1080/17474124.2022.2098111
EA JUL 2022
PG 12
WC Gastroenterology & Hepatology
WE Science Citation Index Expanded (SCI-EXPANDED)
SC Gastroenterology & Hepatology
GA 3B6AA
UT WOS:000822299900001
PM 35793397
DA 2026-05-09
ER

PT J

AU Wang, ZJ
Zhao, SB
Meng, QQ
Wang, SL
Chen, Y
Wang, FM
Li, Q
Yao, J
Du, YQ
Jin, ZD
Bai, Y
Li, ZS
Wang, D
AF Wang, Zhijie
Zhao, Shengbing
Meng, Qianqian
Wang, Shuling
Chen, Yan
Wang, Fuming
Li, Qiang
Yao, Jun
Du, Yiqi
Jin, Zhendong
Bai, Yu
Li, Zhaoshen
Wang, Dong
TI Comparison of three different stents for endoscopic ultrasound-guided
drainage of pancreatic fluid collection: A large retrospective study
SO JOURNAL OF GASTROENTEROLOGY AND HEPATOLOGY
LA English
DT Article
DE adverse event; bleeding; endoscopic drainage; lumen-apposing metal
stent; pancreatic fluid collection
ID APPOSING METAL STENTS; INFECTED NECROTIZING PANCREATITIS; TRANSMURAL
DRAINAGE; PLASTIC STENTS; NECROSIS; PSEUDOCYST; MIGRATION; EFFICACY;
OUTCOMES; LAMS
AB Background and Aim Endoscopic transmural drainage has been recognized as the first-line treatment for pancreatic fluid collection (PFC). Currently, three different types of stents have been extensively applied, including double pigtail plastic stent (PS), fully covered self-expanding metal stent (FCSEMS), and novel lumen-apposing metal stent (LAMS). Nonetheless, limited data are available about the comparison among them in terms of their clinical outcomes and safety for endoscopic ultrasound-guided drainage of PFC.

Methods The current retrospective study was carried on 160 PFC patients undergoing endoscopic ultrasound-guided

drainage from 2010 to 2018 at a single tertiary care center. Patients were divided into three groups based on different drainage ways: drainage using PS, FCSEMS, or LAMS.

Results A total of 160 PFC patients (104 male and 56 female) were analyzed in this retrospective study, including 62 patients drained with PS, 28 with FCSEMS, and 70 with LAMS. Typically, the technical success (93.5% vs 96.4% vs 94.3%, P = 1.000) and treatment success rates (84.6% vs 85.2% vs 89.2%, P = 0.763) were similar between PS, FCSEMS, and LAMS. With regard to major adverse events, four patients (FCSEMS: n = 2 and LAMS: n = 2) with pseudoaneurysms developed severe bleeding; among them, 75% (3/4) of patients were observed within 2 weeks after intervention, and two patients in LAMS group died.

Conclusions There are no significant differences in the clinical outcomes of PFC patients treated with PS, FCSEMS, or LAMS. Nonetheless, severe (or even fatal) bleeding may occur at the early stage after metal stent placement, which should be paid particular attention to.
C1 [Wang, Zhijie; Zhao, Shengbing; Meng, Qianqian; Wang, Shuling; Chen, Yan; Du, Yiqi; Jin, Zhendong; Bai, Yu; Li, Zhaoshen; Wang, Dong] Second Mil Med Univ, Dept Gastroenterol, Gongli Hosp, Shanghai, Peoples R China.

[Wang, Zhijie] Second Mil Med Univ, Gongli Hosp, Dept Gastroenterol, Shanghai, Peoples R China.

[Wang, Fuming] Second Mil Med Univ, Intervent Changhai Hosp, Shanghai, Peoples R China.

[Li, Qiang] Zaozhuang Min Grp, Zaozhuang Hosp, Dept Gastroenterol, Zaozhuang, Shandong, Peoples R China.

[Yao, Jun] Jinan Univ Second Clin Med Sci, Dept Gastroenterol, Shenzhen Municipal Peoples Hosp, Shenzhen, Guangdong, Peoples R China.

C3 Naval Medical University; Naval Medical University; Naval Medical University; Jinan University

RP Jin, ZD; Bai, Y; Li, ZS; Wang, D (corresponding author), Second Mil Med Univ, Dept Gastroenterol, Gongli Hosp, Shanghai, Peoples R China.; Jin, ZD; Bai, Y; Li, ZS; Wang, D (corresponding author), Second Mil Med Univ, Dept Gastroenterol, Changhai Hosp, Shanghai, Peoples R China.

EM zhendjin@126.com; baiyu1998@hotmail.com; li.zhaoshen@hotmail.com; dongwang0901@sina.com

RI 胜兵, 赵/IWV-4506-2023; Wang, Zhijie/ACL-5759-2022; bai, yu/HLW-5287-2023

OI Wang, Zhijie/0000-0001-8015-5413; jin, zhendong/0000-0002-9358-7351; Du, Yiqi/0000-0002-4261-6888;
FU National Key R&D Program of China [2017YFC1308800, 2018YFC1313103]; National Natural Science Foundation of China [81370589]; Three Engineering Training Funds in Shenzhen [SYLY201718]

FX Dr. Bai Yu is supported by the National Key R&D Program of China (2017YFC1308800, 2018YFC1313103), and National Natural Science Foundation of China (Grant No. 81670473 and 81873546) and Three Engineering Training Funds in Shenzhen (No. SYLY201718). Dr. Dong Wang was supported by the National Natural Science Foundation of China (grant no. 81370589).
CR Aburajab M, 2018, GASTROINTEST ENDOSC

Amin S, 2017, CLIN ENDOSC, V50, P388, DOI 10.5946/ce.2016.091

Ang TL, 2016, ENDOSC ULTRASOUND, V5, P320, DOI 10.4103/2303-9027.191659

Bakker OJ, 2012, JAMA-J AM MED ASSOC, V307, P1053, DOI 10.1001/jama.2012.276

Bang JY, 2019, GUT, V68, P1200, DOI 10.1136/gutjnl-2017-315335

Bang JY, 2017, GUT, V66, P2054, DOI 10.1136/gutjnl-2016-312812
Banks PA, 2013, GUT, V62, P102, DOI 10.1136/gutjnl-2012-302779
Bapaye A, 2017, DIGEST ENDOSC, V29, P104, DOI 10.1111/den.12704
Brimhall B, 2018, CLIN GASTROENTEROL H, V16, P1521, DOI 10.1016/j.cgh.2018.02.021
Chen PW, 2018, GASTROINTEST ENDOSC, V87, P904, DOI [10.1016/j.gie.2018.03.018, 10.1016/j.gie.2017.11.006]
Cotton PB, 2010, GASTROINTEST ENDOSC, V71, P446, DOI 10.1016/j.gie.2009.10.027
Elmunzer BJ, 2018, CLIN GASTROENTEROL H, V16, P1851, DOI 10.1016/j.cgh.2018.03.021
Itoi T, 2012, GASTROINTEST ENDOSC, V75, P870, DOI 10.1016/j.gie.2011.10.020
Garcia-Alonso FJ, 2018, ENDOSCOPY, V50, P386, DOI 10.1055/a-0581-9040
Lang GD, 2018, GASTROINTEST ENDOSC, V87, P150, DOI 10.1016/j.gie.2017.06.029
Law ST, 2018, SURG ENDOSC, V32, P2448, DOI 10.1007/s00464-017-5946-6
Muthusamy VR, 2016, GASTROINTEST ENDOSC, V83, P481, DOI 10.1016/j.gie.2015.11.027
Park JK, 2011, AM J GASTROENTEROL, V106, P1761, DOI 10.1038/ajg.2011.212
Penn DE, 2012, GASTROINTEST ENDOSC, V76, P679, DOI 10.1016/j.gie.2012.04.457
Rinninella E, 2015, GASTROINTEST ENDOSC, V82, P1039, DOI 10.1016/j.gie.2015.04.006
Sahar N, 2017, ENDOSC INT OPEN, V5, pE1052, DOI 10.1055/s-0043-111794
Sharaiha RZ, 2016, CLIN GASTROENTEROL H, V14, P1797, DOI 10.1016/j.cgh.2016.05.011
Siddiqui AA, 2017, GASTROINTEST ENDOSC, V85, P758, DOI 10.1016/j.gie.2016.08.014
Siddiqui AA, 2016, GASTROINTEST ENDOSC, V83, P699, DOI 10.1016/j.gie.2015.10.020
Stecher SS, 2017, GUT, V66, P1871, DOI 10.1136/gutjnl-2016-313562
Talreja JP, 2008, GASTROINTEST ENDOSC, V68, P1199, DOI 10.1016/j.gie.2008.06.015
Teoh AYB, 2018, GUT, V67, P1209, DOI 10.1136/gutjnl-2017-314341
van Brunschot S, 2018, LANCET, V391, P51, DOI 10.1016/S0140-6736(17)32404-2
van Santvoort HC, 2011, GASTROENTEROLOGY, V141, P1254, DOI 10.1053/j.gastro.2011.06.073
Varadarajulu S, 2013, GASTROENTEROLOGY, V145, P583, DOI 10.1053/j.gastro.2013.05.046
Vazquez-Sequeiros E, 2016, GASTROINTEST ENDOSC, V84, P450, DOI 10.1016/j.gie.2016.02.044
Venkatachalapathy SV, 2018, ENDOSC INT OPEN, V6, pE259, DOI 10.1055/s-0043-125362
Walter D, 2015, ENDOSCOPY, V47, P63, DOI 10.1055/s-0034-1378113
Zeissig S, 2019, GUT, V68, P25, DOI 10.1136/gutjnl-2018-316023
Zhu HY, 2017, GASTROINTEST ENDOSC, V86, P235, DOI 10.1016/j.gie.2016.11.009
NR 35
TC 31
Z9 35
U1 1
U2 4
PU WILEY
PI HOBOKEN
PA 111 RIVER ST, HOBOKEN 07030-5774, NJ USA
SN 0815-9319
EI 1440-1746
J9 J GASTROEN HEPATOL
JI J. Gastroenterol. Hepatol.

PD APR
PY 2019
VL 34
IS 4
BP 791
EP 798
DI 10.1111/jgh.14557
PG 8
WC Gastroenterology & Hepatology
WE Science Citation Index Expanded (SCI-EXPANDED)
SC Gastroenterology & Hepatology
GA HQ7OV
UT WOS:000462611300029
PM 30475420
DA 2026-05-09
ER

PT J
AU Giovannini, M
Pesenti, C
Rolland, AL
Moutardier, V
Delpero, JR
AF Giovannini, M
Pesenti, C
Rolland, AL
Moutardier, V
Delpero, JR
TI Endoscopic ultrasound-guided drainage of pancreatic pseudocysts or
pancreatic abscesses using a therapeutic echo endoscope
SO ENDOSCOPY
LA English
DT Article
ID TRANSMURAL DRAINAGE; ENDOSONOGRAPHY; CYSTS; CYSTODUODENOSTOMY
AB Study Aims: The purpose of this study is to evaluate a new drainage technique for pancreatic pseudocysts or pancreatic abscesses entirely guided by endoscopic ultrasound (EUS) and using an interventional echo endoscope with a linear curved array transducer.

Patients and Methods: Between July 1996 and September 1999, PUS-guided drainage of a pancreatic pseudocyst or pancreatic abscess was carried out in 35 patients (26 men, 9 women; mean age 56.7, range 29-69). The mean size of the 35 pancreatic cysts was 7.8cm (4-12 cm), Pancreatic pseudocysts were located in the head of the pancreas in two cases, in the body in six cases and in the tail in seven cases. On the other hand, the pancreatic abscesses were located in the tail of the pancreas in 17 cases and in the gastric wall in three cases. The EUS instrument used was the FG 38X endoscope

manufactured by Pentax-Hitachi.

Results: No major complication occurred except in one case of a pneumoperitoneum, which was managed medically, Placement of the 7-F nasocystic drain was successful in 18/20 cases of pancreatic abscess. Surgery was performed in the two other patients. Concerning the pancreatic pseudocysts, placement of an 8.5-French stent was successful in 10 patients and of a nasopancreatic drain in five patients, In one case, only a puncture-aspiration was performed. One recurrence among the 15 pancreatic pseudocysts and two relapses of the 18 pancreatic abscesses have been observed, over a mean follow-up of 27 months (6-48 months). EUS-guided drainage was successful in 31/35 patients (88.5%); only four patients with pancreatic abscesses underwent surgery. No bleeding occurred during the time of this study.

Conclusion: Internal drainage of pancreatic pseudocysts and abscesses exclusively performed with an echo endoscope is a safe and efficient method which should be evaluated further in larger studies.

C1 Inst J Paoli I Calmettes, Endoscop Unit, F-13273 Marseille 9, France.
C3 UNICANCER; Institut Paoli-Calmette (IPC)
RP Giovannini, M (corresponding author), Inst J Paoli I Calmettes, Endoscop Unit, 232 Bd St Marguerite, F-13273 Marseille 9, France.
OI DELPERO, JEAN ROBERT/0000-0002-0000-1332
CR Binmoeller K F, 1995, Gastrointest Endosc Clin N Am, V5, P805
BINMOELLER KF, 1994, GASTROINTEST ENDOSC, V40, P112, DOI 10.1016/S0016-5107(94)70031-1
CREMER M, 1989, GASTROINTEST ENDOSC, V35, P1, DOI 10.1016/S0016-5107(89)72677-8
Gerolami R, 1997, ENDOSCOPY, V29, P106, DOI 10.1055/s-2007-1004083
Giovannini M, 1998, GASTROINTEST ENDOSC, V48, P200, DOI 10.1016/S0016-5107(98)70165-8
GRIMM H, 1992, GASTROINTEST ENDOSC, V38, P170, DOI 10.1016/S0016-5107(92)70384-8
Howell DA, 1998, GASTROINTEST ENDOSC, V8, P142
Mönkemüller KE, 1998, GASTROINTEST ENDOSC, V48, P195, DOI 10.1016/S0016-5107(98)70164-6
Neuhaus H, 2000, ENDOSCOPY, V32, P217, DOI 10.1055/s-2000-94
SAHEL J, 1987, PANCREAS, V2, P447, DOI 10.1097/00006676-198707000-00012
SAHEL J, 1991, ENDOSCOPY, V23, P181, DOI 10.1055/s-2007-1010651
Seifert H, 2000, ENDOSCOPY, V32, P255, DOI 10.1055/s-2000-93
Vilmann P, 1998, ENDOSCOPY, V30, P730, DOI 10.1055/s-2007-1001399
Wiersema MJ, 1996, GASTROINTEST ENDOSC, V44, P614, DOI 10.1016/S0016-5107(96)70022-6
NR 14
TC 222
Z9 239
U1 0
U2 3
PU GEORG THIEME VERLAG KG
PI STUTTGART
PA RUDIGERSTR 14, D-70469 STUTTGART, GERMANY
SN 0013-726X
J9 ENDOSCOPY
JI Endoscopy
PD JUN
PY 2001

VL 33
IS 6
BP 473
EP 477
DI 10.1055/s-2001-14967
PG 5
WC Gastroenterology & Hepatology; Surgery
WE Science Citation Index Expanded (SCI-EXPANDED)
SC Gastroenterology & Hepatology; Surgery
GA 444DW
UT WOS:000169384600001
PM 11437038
DA 2026-05-09
ER

PT J
AU Prachayakul, V
Aswakul, P
AF Prachayakul, Varayu
Aswakul, Pitulak
TI Endoscopic ultrasound-guided biliary drainage as an alternative to
percutaneous drainage and surgical bypass
SO WORLD JOURNAL OF GASTROINTESTINAL ENDOSCOPY
LA English
DT Review
DE Endoscopic ultrasound; Endoscopic ultrasound; Biliary drainage;
Choledochoduodenostomy; Hepaticogastrostomy; Technique
ID POST-ERCP PANCREATITIS; RETROGRADE CHOLANGIOPANCREATOGRAPHY
PANCREATITIS; MALIGNANT BILIARY; METAL STENT; BILE-DUCT; CANNULATION
TECHNIQUE; EUROPEAN-SOCIETY; ULTRASONOGRAPHY; MULTICENTER; OBSTRUCTION
AB Endoscopic retrograde cholangiopancreatography had been a treatment modality of choice for both benign and malignant biliary tract obstruction for more than half century, with a very high clinical success rate and low complications. But in certain circumstances, such as advanced and locally advanced pancreatobiliary malignancies (pancreatic cancer, cholangiocarcinoma, ampullary tumor) and tight benign strictures, endoscopic retrograde cholangiopancreatography (ERCP) fails. Up to this point, the only alternative interventions for these conditions were percutaneous transhepatic biliary drainage or surgery. Endoscopic ultrasound guided interventions was introduced for a couple decades with the better visualization and achievement of the pancreatobiliary tract. And it's still in the process of ongoing development. The inventions of new techniques and accessories lead to more feasibility of high-ended procedures. Endoscopic ultrasound guided biliary drainage was a novel treatment modality for the patient who failed ERCP with the less invasive technique comparing to surgical bypass. The technical and clinical success was high with acceptable complications. Regarded the ability to drain the biliary tract internally without an exploratory laparotomy, this treatment modality became a very interesting procedures for many endosonographers, worldwide, in a short period. We have reviewed the literature and

suggest that endoscopic ultrasound-guided biliary drainage is also an option, and one with a high probability of success, for biliary drainage in the patients who failed conventional endoscopic drainage.

C1 [Prachayakul, Varayu] Mahidol Univ, Siriraj Hosp, Dept Internal Med, Siriraj GI Endoscopy Ctr,Div Gastroenterol,Fac Me, Bangkok 10700, Thailand.

[Aswakul, Pitulak] Samitivej Sukhumvit Hosp, Liver & Digest Inst, Bangkok 10120, Thailand.

C3 Mahidol University

RP Prachayakul, V (corresponding author), Mahidol Univ, Siriraj Hosp, Dept Internal Med, Siriraj GI Endoscopy Ctr,Div Gastroenterol, Bangkok 10700, Thailand.

EM kaiyjr@gmail.com

CR Altonbary AY, 2014, ENDOSC ULTRASOUND, V3, P137, DOI 10.4103/2303-9027.131043

Alvarez-Sanchez MV, 2014, SURG ENDOSC, V28, P712, DOI 10.1007/s00464-013-3260-5

[Anonymous], 2013, J INTERV GASTROENTER, DOI DOI 10.7178/JIG.128

Artifon E L A, 2011, Rev Gastroenterol Mex, V76, P270

Artifon Everson L.A, 2014, Rev. gastroenterol. Perú, V34, P53

Artifon ELA, 2013, ENDOSC ULTRASOUND, V2, P61, DOI 10.4103/2303-9027.117687

Artifon ELA, 2012, J CLIN GASTROENTEROL, V46, P768, DOI 10.1097/MCG.0b013e31825f264c

Artifon ELA, 2012, KOREAN J RADIOL, V13, pS74, DOI 10.3348/kjr.2012.13.S1.S74

Artifon ELA, 2011, J PANCREAS, V12, P610

Artifon ELA, 2010, J PANCREAS, V11, P597

Audisio R A, 1993, HPB Surg, V6, P287, DOI 10.1155/1993/17078

Belletrutti PJ, 2010, J PANCREAS, V11, P234

Burmester E, 2003, GASTROINTEST ENDOSC, V57, P246, DOI 10.1067/mge.2003.85

CATALANO D, 1961, Rass Int Clin Ter, V41, P255

Choudhary A, 2014, WORLD J GASTROENTERO, V20, P4093, DOI 10.3748/wjg.v20.i14.4093

DEMLING L, 1970, DEUT MED WOCHENSCHR, V95, P1427, DOI 10.1055/s-0028-1108660

Dhir V, 2014, DIGEST ENDOSC, V26, P430, DOI 10.1111/den.12153

Dumonceau JM, 2007, ENDOSCOPY, V39, P124, DOI 10.1055/s-2006-945096

Dumonceau JM, 2014, ENDOSCOPY, V46, P798, DOI 10.1055/s-0034-1377875

Eum J, 2010, GASTROINTEST ENDOSC, V72, P1279, DOI 10.1016/j.gie.2010.07.026

Fabbri C, 2014, WORLD J GASTROENTERO, V20, P8424, DOI 10.3748/wjg.v20.i26.8424

Galasso D, 2013, ENDOSCOPY, V45, pE233, DOI 10.1055/s-0033-1344322

Garcarek J, 2012, ADV CLIN EXP MED, V21, P621

Giovannini M, 2001, ENDOSCOPY, V33, P898, DOI 10.1055/s-2001-17324

Glazer ES, 2014, J PAIN SYMPTOM MANAG, V47, P307, DOI 10.1016/j.jpainsymman.2013.03.013

Gupta K, 2014, J CLIN GASTROENTEROL, V48, P80, DOI 10.1097/MCG.0b013e31828c6822

Hamada T, 2014, DIGEST DIS SCI, V59, P1931, DOI 10.1007/s10620-014-3062-1

Ho CS, 2012, KOREAN J RADIOL, V13, pS56, DOI 10.3348/kjr.2012.13.S1.S56

Iqbal S, 2013, GASTROENT RES PRACT, V2013, DOI 10.1155/2013/869214

Ito K, 2014, DIGEST ENDOSC, V26, P442, DOI 10.1111/den.12158

Iwashita T, 2012, ENDOSCOPY, V44, P60, DOI 10.1055/s-0030-1256871

Iwashita T, 2014, CLIN J GASTROENTEROL, V7, P94, DOI 10.1007/s12328-014-0467-5

Jürgensen C, 2013, ENDOSCOPY, V45, pE40, DOI [10.1055/s0032-1325891, 10.1055/s-0032-1325891]

Kahaleh M, 2013, WORLD J GASTROENTERO, V19, P1372, DOI 10.3748/wjg.v19.i9.1372
Kawakubo K, 2014, J HEPATO-BIL-PAN SCI, V21, P328, DOI 10.1002/jhbp.27
Kawakubo K, 2013, SURG ENDOSC, V27, P3437, DOI 10.1007/s00464-013-2896-5
Khashab MA, 2014, DIG DIS SCI
Khashab MA, 2013, GASTROINTEST ENDOSC, V78, P675, DOI 10.1016/j.gie.2013.07.017
Khashab MA, 2013, GASTROINTEST ENDOSC, V78, P102, DOI 10.1016/j.gie.2013.03.004
Khashab MA, 2012, GASTROINTEST ENDOSC, V76, P921, DOI 10.1016/j.gie.2012.05.018
Kobayashi G, 2013, DIGEST ENDOSC, V25, P295, DOI 10.1111/j.1443-1661.2012.01372.x
Kubiliun Nisa M, 2013, Gastrointest Endosc Clin N Am, V23, P769, DOI 10.1016/j.giec.2013.06.003
Kumta Nikhil A, 2014, Curr Treat Options Gastroenterol, V12, P154, DOI 10.1007/s11938-014-0011-1
Lai LH, 2010, GASTROINTEST ENDOSC, V72, P186, DOI 10.1016/j.gie.2010.01.052
Leng Jian-Jun, 2014, World J Surg Oncol, V12, P272
Luz LP, 2014, WORLD J GASTROENTERO, V20, P7808, DOI 10.3748/wjg.v20.i24.7808
Mallery S, 2004, GASTROINTEST ENDOSC, V59, P100, DOI 10.1016/S0016-5107(03)02300-9
Myung DS, 2014, ENDOSCOPY, V46, P352, DOI 10.1055/s-0034-1365060
Okabe Y, 2014, DIGEST ENDOSC, V26, P122, DOI 10.1111/den.12274
Panpimanmas Sukij, 2011, J Med Assoc Thai, V94 Suppl 2, pS129
Park Do Hyun, 2012, Gastrointest Endosc Clin N Am, V22, P271, DOI 10.1016/j.giec.2012.04.009
Park DH, 2011, GASTROINTEST ENDOSC, V74, P1276, DOI 10.1016/j.gie.2011.07.054
Park DH, 2010, GASTROINTEST ENDOSC, V71, P413, DOI 10.1016/j.gie.2009.10.015
Park SJ, 2013, GASTROINTEST ENDOSC, V78, P374, DOI 10.1016/j.gie.2013.04.183
Perez-Miranda Manuel, 2013, Curr Gastroenterol Rep, V15, P349, DOI 10.1007/s11894-013-0349-x
Polkowski M, 2012, ENDOSCOPY, V44, P190, DOI 10.1055/s-0031-1291543
Prachayakul V, 2011, ENDOSCOPY, V43, pE109, DOI 10.1055/s-0030-1256140
Prachayakul V, 2013, WORLD J GASTROENTERO, V19, P4758, DOI 10.3748/wjg.v19.i29.4758
Püspök A, 2005, AM J GASTROENTEROL, V100, P1743, DOI 10.1111/j.1572-0241.2005.41806.x
Qian Y, 2014, WORLD J GASTROENTERO, V20, P10217, DOI 10.3748/wjg.v20.i29.10217
Sarkaria S, 2013, GUT LIVER, V7, P129, DOI 10.5009/gnl.2013.7.2.129
Sarkaria Savreet, 2013, Gastrointest Endosc Clin N Am, V23, P435, DOI 10.1016/j.giec.2012.12.013
Saxena P, 2014, ENDOSCOPY, V46, pE140, DOI 10.1055/s-0033-1359240
Shami VM, 2010, DIGEST LIVER DIS, V42, P419, DOI 10.1016/j.dld.2009.09.009
Sharaiha RZ, 2012, GASTROINTEST ENDOSC, V76, P227, DOI 10.1016/j.gie.2012.01.044
Skinner M, 2014, ENDOSCOPY, V46, P560, DOI 10.1055/s-0034-1365698
Song BJ, 2014, CLIN ENDOSC, V47, P217, DOI 10.5946/ce.2014.47.3.217
STANLEY J, 1986, RADIOLOGY, V158, P195, DOI 10.1148/radiology.158.1.2416006
Tonozuka R, 2013, DIGEST ENDOSC, V25, P100, DOI 10.1111/den.12063
Tse F, 2013, ENDOSCOPY, V45, P605, DOI 10.1055/s-0032-1326640
Varadarajulu S, 2013, GASTROINTEST ENDOSC, V78, P742, DOI 10.1016/j.gie.2013.06.009
Vila JJ, 2010, AM J GASTROENTEROL, V105, P959, DOI 10.1038/ajg.2009.690
Wiersema MJ, 1996, GASTROINTEST ENDOSC, V43, P102, DOI 10.1016/S0016-5107(06)80108-2
Will U, 2014, ULTRASCHALL MED
Zang JF, 2014, SURG LAPARO ENDO PER, V24, P429, DOI 10.1097/SLE.0000000000000062

NR 75
TC 13
Z9 16
U1 0
U2 4
PU BAISHIDENG PUBLISHING GROUP INC
PI PLEASANTON
PA 8226 REGENCY DR, PLEASANTON, CA 94588 USA
SN 1948-5190
J9 WORLD J GASTRO ENDOS
JI World J. Gastrointest. Endosc.
PD JAN 16
PY 2015
VL 7
IS 1
BP 37
EP 44
DI 10.4253/wjge.v7.i1.37
PG 8
WC Gastroenterology & Hepatology
WE Emerging Sources Citation Index (ESCI)
SC Gastroenterology & Hepatology
GA CR0GL
UT WOS:000360996400003
PM 25610532
OA Green Submitted, hybrid
DA 2026-05-09
ER

PT J
AU Katanuma, A
Maguchi, H
Osanai, M
Takahashi, K
AF Katanuma, Akio
Maguchi, Hiroyuki
Osanai, Manabu
Takahashi, Kuniyuki
TI ENDOSCOPIC ULTRASOUND-GUIDED BILIARY DRAINAGE PERFORMED FOR REFRACTORY
BILE DUCT STENOSIS DUE TO CHRONIC PANCREATITIS: A CASE REPORT
SO DIGESTIVE ENDOSCOPY
LA English

DT Article
DE benign bile duct stenosis; chronic pancreatitis; endoscopic
ultrasound-guided biliary drainage
ID OBSTRUCTION; CHOLEDOCHODUODENOSTOMY; STRICTURES; STENTS; TRACT
AB We report a case of the patient who underwent endoscopic ultrasound-guided biliary drainage (EUS-BD) for refractory bile duct stenosis due to chronic pancreatitis. The patient had repeatedly undergone endoscopic biliary stenting for bile duct stenosis due to chronic pancreatitis. Because of repeated relapses of cholangitis and jaundice, transpapillary treatment was judged to have reached its limits. Surgical bypass was attempted but had to be abandoned due to adhesions. Thus, EUS-BD was performed. The procedure was successful, and placement of a covered expandable metallic stent (C-EMS) relieved cholangitis. Two months after placement, the C-EMS was removed, and the patient became stent-free but closure of the fistula subsequently occurred.
C1 [Katanuma, Akio] Teine Keijinkai Hosp, Ctr Gastroenterol, Teine Ku, Sapporo, Hokkaido 0068555, Japan.
C3 Teine Keijinkai Hospital
RP Katanuma, A (corresponding author), Teine Keijinkai Hosp, Ctr Gastroenterol, Teine Ku, 1-40 Maeda 1Jyo, Sapporo, Hokkaido 0068555, Japan.
EM akio-ka@ta2.so-net.ne.jp
CR Costamagna G, 2001, GASTROINTEST ENDOSC, V54, P162, DOI 10.1016/S0016-5107(01)70100-9
Giovannini M, 2003, ENDOSCOPY, V35, P1076, DOI 10.1055/s-2003-44596
Giovannini M, 2001, ENDOSCOPY, V33, P898, DOI 10.1055/s-2001-17324
Hammel P, 2001, NEW ENGL J MED, V344, P418, DOI 10.1056/NEJM200102083440604
Hara K, 2011, AM J GASTROENTEROL, V106, P1239, DOI 10.1038/ajg.2011.84
Judah JR, 2007, WORLD J GASTROENTERO, V13, P3531, DOI 10.3748/wjg.v13.i26.3531
LESUR G, 1993, HEPATOLOGY, V18, P1078
Mukai T., 2003, DIGEST ENDOSC, V16, P12
SILVIS SE, 1994, GASTROINTEST ENDOSC, V40, P17, DOI 10.1016/S0016-5107(94)70004-4
Smits ME, 1996, BRIT J SURG, V83, P764, DOI 10.1002/bjs.1800830612
van Boeckel PGA, 2009, BMC GASTROENTEROL, V9, DOI 10.1186/1471-230X-9-96
WILSON C, 1989, GUT, V30, P520, DOI 10.1136/gut.30.4.520
Yamaguchi T, 2006, WORLD J GASTROENTERO, V12, P426, DOI 10.3748/wjg.v12.i3.426
Yamao K, 2008, ENDOSCOPY, V40, P340, DOI 10.1055/s-2007-995485
Yamao K, 2006, GASTROINTEST ENDOSC, V64, P663, DOI 10.1016/j.gie.2006.07.003
NR 15
TC 6
Z9 10
U1 0
U2 2
PU WILEY-BLACKWELL
PI MALDEN
PA COMMERCE PLACE, 350 MAIN ST, MALDEN 02148, MA USA
SN 0915-5635
J9 DIGEST ENDOSC
JI Dig. Endosc.

PD MAY
PY 2012
VL 24
SU 1
SI SI
BP 34
EP 37
DI 10.1111/j.1443-1661.2012.01256.x
PG 4
WC Gastroenterology & Hepatology; Surgery
WE Science Citation Index Expanded (SCI-EXPANDED)
SC Gastroenterology & Hepatology; Surgery
GA 931CZ
UT WOS:000303193500007
PM 22533749
OA Bronze
DA 2026-05-09
ER

PT J
AU Kedia, P
Gaidhane, M
Kahaleh, M
AF Kedia, Prashant
Gaidhane, Monica
Kahaleh, Michel
TI Endoscopic Guided Biliary Drainage: How Can We Achieve Efficient Biliary
Drainage?
SO CLINICAL ENDOSCOPY
LA English
DT Review
DE Endosonography; Cholangiopancreatography; endoscopic retrograde;
Endoscopic ultrasound guided biliary drainage; Biliary stricture
AB Currently, endoscopic retrograde cholangiopancreatography (ERCP) is the preferred procedure for biliary drainage for various pancreatico-biliary disorders. ERCP is successful in 90% of the cases, but is unsuccessful in cases with altered anatomy or with tumors obstructing access to the duodenum. Due to the morbidity and mortality associated with surgical or percutaneous approaches in unsuccessful ERCP cases, biliary endoscopists have been using endoscopic ultrasound-guided biliary drainage (EUS-BD) more frequently within the last decade in different countries. As with any novel advanced endoscopic procedure that incorporates various approaches, advanced endoscopists all over the world have innovated and adopted diverse EUS-BD techniques. Indications for EUS-BD include failed conventional ERCP, altered anatomy, tumor preventing access into the biliary tree and contraindication to percutaneous access (i.e., ascites, etc.). EUS-BD utilizing EUS-guided rendezvous technique is conducted by creating a tract from either the stomach or the duodenum into the bile

duct. Although EUS-BD has rapidly been gaining attraction and popularity in the endoscopic world, the indications and methods have yet to be standardized. There are several access routes and techniques that are employed by advanced endoscopists throughout the world for BD. This article reviews the indications and currently practiced EUS-BD techniques, including indications, technical details (intrahepatic or extrahepatic approach), equipment, patient selection, complications, and overall advantages and limitations.
C1 [Kedia, Prashant; Gaidhane, Monica; Kahaleh, Michel] New York Presbyterian Hosp, Weill Cornell Med Coll, Div Gastroenterol & Hepatol, 525 E 68th St, New York, NY 10021 USA.
C3 Cornell University; Weill Cornell Medicine; NewYork-Presbyterian
Hospital
RP Kahaleh, M (corresponding author), New York Presbyterian Hosp, Weill Cornell Med Coll, Div Gastroenterol & Hepatol, 525 E 68th St, New York, NY 10021 USA.
EM mkahaleh@gmail.com
RI Gaidhane, Monica/B-9414-2009; Kahaleh, Michel/A-9408-2011; M,
Gaidhane/B-9414-2009
OI Gaidhane, Monica/0000-0001-6773-7080; Kahaleh,
Michel/0000-0003-0836-6114;
FU Boston Scientific, Fujinon, EMcison, Xlumena Inc., MaunaKea, W.L
FX Dr. Michel Kahaleh has received grant support from Boston Scientific,
Fujinon, EMcison, Xlumena Inc., MaunaKea, W.L. Gore, Cook Endoscopy,
Aspire Bariatrics, GI Dynamics and MI Tech. He is a consultant for
Xlumena Inc. and Boston Scientific.
CR Ang TL, 2007, J PANCREAS, V8, P438
Artifon ELA, 2012, J CLIN GASTROENTEROL, V46, P768, DOI 10.1097/MCG.0b013e31825f264c
Artifon ELA, 2010, J PANCREAS, V11, P597
Attasaranya S, 2012, GASTROENT RES PRACT, V2012, DOI 10.1155/2012/680753
Baron TH, 2006, GASTROINTEST ENDOSC, V63, pS29, DOI 10.1016/j.gie.2006.02.019
Belletrutti PJ, 2010, J PANCREAS, V11, P234
Bories E, 2007, ENDOSCOPY, V39, P287, DOI 10.1055/s-2007-966212
Brauer BC, 2009, GASTROINTEST ENDOSC, V70, P471, DOI 10.1016/j.gie.2008.12.233
Burmester E, 2003, GASTROINTEST ENDOSC, V57, P246, DOI 10.1067/mge.2003.85
DAVIDS PHP, 1992, LANCET, V340, P1488, DOI 10.1016/0140-6736(92)92752-2
Dhir V, 2012, GASTROINTEST ENDOSC, V75, P354, DOI 10.1016/j.gie.2011.07.075
Fabbri C, 2011, ENDOSCOPY, V43, P438, DOI 10.1055/s-0030-1256097
FERRUCCI JT, 1980, RADIOLOGY, V135, P1
Fujita N, 2007, WORLD J GASTROENTERO, V13, P5512, DOI 10.3748/wjg.v13.i41.5512
Giovannini M, 2003, ENDOSCOPY, V35, P1076, DOI 10.1055/s-2003-44596
Giovannini M, 2001, ENDOSCOPY, V33, P898, DOI 10.1055/s-2001-17324
Gupta K, 2014, J CLIN GASTROENTEROL, V48, P80, DOI 10.1097/MCG.0b013e31828c6822
Hanada K, 2009, DIGEST ENDOSC, V21, pS75, DOI 10.1111/j.1443-1661.2009.00855.x
Hara K, 2013, ENDOSCOPY, V45, P392, DOI 10.1055/s-0032-1326076
Hara K, 2011, AM J GASTROENTEROL, V106, P1239, DOI 10.1038/ajg.2011.84
HARBIN WP, 1980, RADIOLOGY, V135, P15, DOI 10.1148/radiology.135.1.6987704

Horaguchi J, 2009, DIGEST ENDOSC, V21, P239, DOI 10.1111/j.1443-1661.2009.00899.x
Itoi T, 2008, WORLD J GASTROENTERO, V14, P6078, DOI 10.3748/wjg.14.6078
Itoi T, 2013, DIGEST ENDOSC, V25, P142, DOI 10.1111/den.12065
Iwamuro M, 2010, DIGEST ENDOSC, V22, P236, DOI 10.1111/j.1443-1661.2010.00997.x
Iwashita T, 2013, DIGEST DIS SCI, V58, P2417, DOI 10.1007/s10620-013-2645-6
Kaassis M, 2003, GASTROINTEST ENDOSC, V57, P178, DOI 10.1067/mge.2003.66
Kahaleh M, 2006, GASTROINTEST ENDOSC, V64, P52, DOI 10.1016/j.gie.2006.01.063
Kahaleh M, 2013, GASTROINTEST ENDOS S, V77, pAB127, DOI DOI 10.1016/J.GIE.2013.04.021
Kahaleh M, 2013, WORLD J GASTROENTERO, V19, P1372, DOI 10.3748/wjg.v19.i9.1372
Katanuma A, 2012, DIGEST ENDOSC, V24, P34, DOI 10.1111/j.1443-1661.2012.01256.x
Kawakubo K, 2012, GUT LIVER, V6, P399, DOI 10.5009/gnl.2012.6.3.399
Khashab MA, 2012, GASTROINTEST ENDOSC, V76, P209, DOI 10.1016/j.gie.2012.03.170
Kim TH, 2012, WORLD J GASTROENTERO, V18, P2526, DOI 10.3748/wjg.v18.i20.2526
Kim YS, 2010, ENDOSCOPY, V42, P496, DOI 10.1055/s-0029-1244082
Komaki T, 2011, PANCREATOLOGY, V11, P47, DOI 10.1159/000323508
Lai R, 2005, ENDOSCOPY, V37, P487, DOI 10.1055/s-2005-861250
Mallery S, 2004, GASTROINTEST ENDOSC, V59, P100, DOI 10.1016/S0016-5107(03)02300-9
Maranki J, 2009, ENDOSCOPY, V41, P532, DOI 10.1055/s-0029-1214712
Nguyen-Tang T, 2010, ENDOSCOPY, V42, P232, DOI 10.1055/s-0029-1243858
Park DH, 2013, GASTROINTEST ENDOSC, V78, P91, DOI 10.1016/j.gie.2013.01.042
Park DH, 2011, GASTROINTEST ENDOSC, V74, P1276, DOI 10.1016/j.gie.2011.07.054
Prachayakul V, 2011, ENDOSCOPY, V43, pE109, DOI 10.1055/s-0030-1256140
Püspök A, 2005, AM J GASTROENTEROL, V100, P1743, DOI 10.1111/j.1572-0241.2005.41806.x
Ramírez-Luna MA, 2011, ENDOSCOPY, V43, P826, DOI 10.1055/s-0030-1256406
Sarkaria S, 2013, GUT LIVER, V7, P129, DOI 10.5009/gnl.2013.7.2.129
Siddiqui AA, 2011, SURG ENDOSC, V25, P549, DOI 10.1007/s00464-010-1216-6
SMITH AC, 1994, LANCET, V344, P1655, DOI 10.1016/S0140-6736(94)90455-3
Song TJ, 2012, WORLD J GASTROENTERO, V18, P4435, DOI 10.3748/wjg.v18.i32.4435
Tarantino I, 2008, ENDOSCOPY, V40, P336, DOI 10.1055/s-2007-995455
Wiersema MJ, 1996, GASTROINTEST ENDOSC, V43, P102, DOI 10.1016/S0016-5107(06)80108-2
Will U, 2007, ENDOSCOPY, V39, P292, DOI 10.1055/s-2007-966215
Yamao K, 2008, ENDOSCOPY, V40, P340, DOI 10.1055/s-2007-995485
Yamao K, 2006, GASTROINTEST ENDOSC, V64, P663, DOI 10.1016/j.gie.2006.07.003
NR 54
TC 34
Z9 43
U1 0
U2 3
PU KOREAN SOC GASTROINTESTINAL ENDOSCOPY
PI SEOUL
PA 2003 LG PALACE, 165-8 DONGGYO-DONG, MAPO-GU, SEOUL, 121-754, SOUTH KOREA
SN 2234-2400

EI 2234-2443
J9 CLIN ENDOSC
JI Clin. Endosc.
PD SEP
PY 2013
VL 46
IS 5
SI SI
BP 543
EP 551
DI 10.5946/ce.2013.46.5.543
PG 9
WC Gastroenterology & Hepatology
WE Emerging Sources Citation Index (ESCI)
SC Gastroenterology & Hepatology
GA VA4VX
UT WOS:000409983500023
PM 24143319
OA gold
DA 2026-05-09
ER

PT J
AU Jin, Z
Wei, YP
Lin, HP
Yang, J
Jin, HB
Shen, SS
Zhang, XF
AF Jin, Zheng
Wei, Yaping
Lin, Huapeng
Yang, Jing
Jin, Hangbin
Shen, Sisi
Zhang, Xiaofeng
TI Endoscopic ultrasound-guided versus endoscopic retrograde cholangiopancreatography-guided biliary drainage for primary treatment of distal malignant biliary obstruction: A systematic review and meta-analysis
SO DIGESTIVE ENDOSCOPY

LA English
DT Review
DE choledochoduodenostomy; endoscopic ultrasound-guided biliary drainage;
hepaticogastrostomy; malignant biliary obstruction
ID EXPANDABLE METAL STENTS; ERCP; PALLIATION; TRIALS
AB Objectives Current evidence supporting the utility of endoscopic ultrasound-guided biliary drainage (EUS-BD) as primary treatment for distal malignant biliary obstruction (MBO) is limited. We conducted a meta-analysis to compare the performance of EUS-BD and endoscopic retrograde cholangiopancreatography-guided biliary drainage (ERCP-BD) as primary palliation of distal MBO. Methods We searched several databases for comparative studies evaluating EUS-BD vs. ERCP-BD in primary drainage of distal MBO up to 28 February 2019. Primary outcomes were technical success and clinical success. Secondary outcomes included adverse events, stent patency, stent dysfunction, tumor in/overgrowth, reinterventions, procedure duration, and overall survival. Results Four studies involving 302 patients were qualified for the final analysis. There was no difference in technical success (risk ratio [RR] 1.00; 95% confidence interval [95% CI] 0.93-1.08), clinical success (RR 1.00; 95% CI 0.94-1.06) and total adverse events (RR 0.68; 95% CI: 0.31-1.48) between the two procedures. EUS-BD was associated with lower rates of post-procedure pancreatitis (RR 0.12; 95% CI 0.02-0.62), stent dysfunction (RR 0.54; 95% CI 0.32-0.91), and tumor in/overgrowth (RR 0.22; 95% CI 0.07-0.76). No differences were noted in reinterventions (RR 0.59; 95% CI 0.21-1.69), procedure duration (weighted mean difference -2.11; 95% CI -9.51 to 5.29), stent patency (hazard ratio [HR] 0.61; 95% CI 0.34-1.11), and overall survival (HR 1.00; 95% CI 0.66-1.51). Conclusions With adequate endoscopy expertise, EUS-BD could show similar efficacy and safety when compared with ERCP-BD for primary palliation of distal MBO and exhibits several clinical advantages.
C1 [Jin, Zheng; Wei, Yaping; Lin, Huapeng; Yang, Jing; Jin, Hangbin; Zhang, Xiaofeng] Zhejiang Univ, Sch Med, Affiliated Hangzhou Peoples Hosp 1, Hangzhou, Zhejiang, Peoples R China.
[Shen, Sisi] Zhejiang Univ, Sch Med, Affiliated Hosp 2, Hangzhou, Zhejiang, Peoples R China.
C3 Zhejiang University; Zhejiang University
RP Zhang, XF (corresponding author), Zhejiang Univ, Sch Med, Affiliated Hangzhou Peoples Hosp 1, Dept Gastroenterol, 261 Huansha Rd, Hangzhou 310006, Zhejiang, Peoples R China.
EM zxf837@tom.com
RI Jin, Zheng/AAD-3141-2019
CR Almadi MA, 2017, AM J GASTROENTEROL, V112, P260, DOI 10.1038/ajg.2016.512
Baars JE, 2018, ENDOSC ULTRASOUND, V7, P4, DOI 10.4103/eus.eus_105_17
Bang JY, 2018, GASTROINTEST ENDOSC, V88, P9, DOI 10.1016/j.gie.2018.03.012
Castaño R, 2010, SURG ENDOSC, V24, P2092, DOI 10.1007/s00464-010-0903-7
Das A, 2000, Cancer Control, V7, P452
Dhir V, 2017, DIGEST ENDOSC, V29, P472, DOI 10.1111/den.12818
Dhir V, 2015, GASTROINTEST ENDOSC, V81, P913, DOI 10.1016/j.gie.2014.09.054
Egger M, 1997, BMJ-BRIT MED J, V315, P629, DOI 10.1136/bmj.315.7109.629
Giovannini M, 2001, ENDOSCOPY, V33, P898, DOI 10.1055/s-2001-17324
Higgins JPT, 2002, STAT MED, V21, P1539, DOI [10.1002/sim.1186, 10.1016/j.bandl.2008.10.005]
Higgins JPT, 2011, BMJ-BRIT MED J, V343, DOI 10.1136/bmj.d5928
Hozo S., 2005, BMC MED RES METHODOL, V5, P13, DOI [10.1186/1471-2288-5-13, DOI 10.1186/1471-2288-5-13]
Kawakubo K, 2016, ENDOSCOPY, V48, P164, DOI 10.1055/s-0034-1393179
Kawakubo K, 2014, J HEPATO-BIL-PAN SCI, V21, P328, DOI 10.1002/jhbp.27

Khan MA, 2016, DIGEST DIS SCI, V61, P684, DOI 10.1007/s10620-015-3933-0
Macdonald Geraldine, 2012, Cochrane Database Syst Rev, pCD001930, DOI 10.1002/14651858.CD001930.pub3
Moher D, 2009, BMJ-BRIT MED J, V339, DOI 10.1136/bmj.b2535
Moole H, 2016, CAN J GASTROENTEROL, V2016, DOI 10.1155/2016/4726078
Moss AC, 2007, EUR J GASTROEN HEPAT, V19, P1119, DOI 10.1097/MEG.0b013e3282f16206
Nakai Y, 2017, DIGEST ENDOSC, V29, P218, DOI 10.1111/den.12752
Paik WH, 2018, AM J GASTROENTEROL, V113, P987, DOI 10.1038/s41395-018-0122-8
Park JK, 2018, GASTROINTEST ENDOSC, V88, P277, DOI 10.1016/j.gie.2018.03.015
Parmar MKB, 1998, STAT MED, V17, P2815, DOI 10.1002/(SICI)1097-0258(19981230)17:24<2815::AID-SIM110>3.0.CO;2-8
Poincloux L, 2015, ENDOSCOPY, V47, P794, DOI [10.1055/s-0035-1545399, 10.1055/s-0034-1391988]
Sharaiha RZ, 2017, GASTROINTEST ENDOSC, V85, P904, DOI 10.1016/j.gie.2016.12.023
Shrier I, 2007, AM J EPIDEMIOL, V166, P1203, DOI 10.1093/aje/kwm189
Tierney JF, 2007, TRIALS, V8, DOI 10.1186/1745-6215-8-16
Tol JAMG, 2016, GUT, V65, P1981, DOI 10.1136/gutjnl-2014-308762
UEMURA RS, 2017, J CLIN GASTROENTEROL, V52, P1
Wang KX, 2016, GASTROINTEST ENDOSC, V83, P1218, DOI 10.1016/j.gie.2015.10.033
WELLS GA, 2014, NEWCASTLE OTTAWA SCA
NR 31
TC 57
Z9 71
U1 0
U2 7
PU WILEY
PI HOBOKEN
PA 111 RIVER ST, HOBOKEN 07030-5774, NJ USA
SN 0915-5635
EI 1443-1661
J9 DIGEST ENDOSC
JI Dig. Endosc.
PD JAN
PY 2020
VL 32
IS 1
BP 16
EP 26
DI 10.1111/den.13456
EA JUL 2019
PG 11
WC Gastroenterology & Hepatology; Surgery
WE Science Citation Index Expanded (SCI-EXPANDED)
SC Gastroenterology & Hepatology; Surgery

GA KA4LY
UT WOS:000474255700001
PM 31165527
OA Bronze
DA 2026-05-09
ER

PT J
AU Sassatelli, R
Cecinato, P
Lupo, M
Azzolini, F
Decembrino, F
Iori, V
Sereni, G
Tioli, C
Cavina, M
Zecchini, R
Parmeggiani, F
Grillo, S
Camellini, L
AF Sassatelli, Romano
Cecinato, Paolo
Lupo, Marinella
Azzolini, Francesco
Decembrino, Francesco
Iori, Veronica
Sereni, Giuliana
Tioli, Cristiana
Cavina, Maurizio
Zecchini, Ramona
Parmeggiani, Francesca
Grillo, Simone
Camellini, Lorenzo
TI Endoscopic ultrasound-guided biliary drainage for malignant biliary obstruction after failed ERCP in low performance status patients
SO DIGESTIVE AND LIVER DISEASE
LA English
DT Article
DE EUS guided biliary drainage; Obstructive jaundice; Malignant biliary obstruction; Metal stent; Plastic stent
ID ADVERSE EVENTS; METAL STENT; EUS; HEPATICOGASTROSTOMY;

CHOLEDOCHODUODENOSTOMY; COMPLICATIONS; MULTICENTER; EFFICACY; ACCESS

AB Background: Endoscopic retrograde cholangiopancreatography (ERCP) with the placement of a biliary stent is the treatment of choice for palliation of malignant obstructive jaundice. In 5-10% of cases ERCP fails. In these cases an effective alternative is endoscopic ultrasonography-guided biliary drainage (EUSBD).

Aim: Evaluation of the principal clinical outcomes of direct transluminal EUS-BD.

Patients and methods: This study is a retrospective analysis. All consecutive patients with malignant obstructive jaundice, in whom ERCP had failed, were enrolled. The primary outcome was the technical success of EUS-BD defined as the correct placement of the metal or plastic stent across the stomach or duodenum to the biliary tree. The most important secondary outcomes were early and late clinical success, both linked to the decrease of bilirubin haematic level.

Results: Between January 2011 and November 2017 thirty-six patients were included. Technical success was obtained in 91.6%. A clinical success, early or late was obtained in 75.8%. The ECOG performance status of less than 3 was correlated with clinical success. Adverse events occurred in 30.3% of patients.

Conclusions: EUS-BD is an effective and safe procedure. (c) 2019 Editrice Gastroenterologica Italiana S.r.l. Published by Elsevier Ltd. All rights reserved.

C1 [Sassatelli, Romano; Cecinato, Paolo; Lupo, Marinella; Decembrino, Francesco; Iori, Veronica; Sereni, Giuliana; Tioli, Cristiana; Cavina, Maurizio; Zecchini, Ramona; Parmeggiani, Francesca; Grillo, Simone] Azienda USL IRCCS Reggio Emilia, Unit Gastroenterol & Digest Endoscopy, Reggio Emilia, Italy.

[Azzolini, Francesco] Univ Vita Salute San Raffaele, Div Gastroenterol & Gastrointestinal Endoscopy, Milan, Italy.

[Camellini, Lorenzo] ASL 5 Spezzino, Gastroenterol Unit, La Spezia, Italy.

C3 Vita-Salute San Raffaele University

RP Cecinato, P (corresponding author), Arcispedale Santa Maria Nuova IRCCS, Unit Gastroenterol & Digest Endoscopy, I-42123 Reggio Emilia, Italy.

EM paolo.cecinato@ausl.re.it

RI Sereni, Giuliana/AAB-1141-2021; Grillo, Simone/AAA-4332-2021; Cecinato, Paolo/AAB-1079-2021; Iori, Veronica/HSG-7098-2023; Sassatelli, Romano/AAC-2934-2020; Azzolini, Francesco/IAR-6915-2023

OI Sereni, Giuliana/0000-0002-5611-3640; Grillo, Simone/0000-0002-8770-7329; Cecinato, Paolo/0000-0002-1870-5952; Iori, Veronica/0000-0002-7865-1511;

CR Amin MB, 2017, AJCC CANCER STAGING

Anderloni A, 2019, GASTROINTEST ENDOSC, V89, P69, DOI [10.1016/j.gie.2018.04.1746, 10.1016/j.gie.2018.08.047]

Artifon ELA, 2015, GASTROINTEST ENDOSC, V81, P950, DOI 10.1016/j.gie.2014.09.047

Artifon ELA, 2012, J PANCREAS, V13, P7

Baars JE, 2018, ENDOSC ULTRASOUND, V7, P4, DOI 10.4103/eus.eus_105_17

Baron TH, 2006, GASTROINTEST ENDOSC, V63, pS29, DOI 10.1016/j.gie.2006.02.019

Cotton PB, 2010, GASTROINTEST ENDOSC, V71, P446, DOI 10.1016/j.gie.2009.10.027

Dhir V, 2017, DIGEST ENDOSC, V29, P472, DOI 10.1111/den.12818

Dhir V, 2014, DIGEST ENDOSC, V26, P430, DOI 10.1111/den.12153

Ekkelenkamp VE, 2015, ENDOSCOPY, V47, P504, DOI 10.1055/s-0034-1391231

Enochsson L, 2010, GASTROINTEST ENDOSC, V72, P1175, DOI 10.1016/j.gie.2010.07.047

Giovannini M, 2006, ENDOSCOPY, V38, pS93, DOI 10.1055/s-2006-946665

Giovannini M, 2001, ENDOSCOPY, V33, P898, DOI 10.1055/s-2001-17324
Gupta K, 2014, J CLIN GASTROENTEROL, V48, P80, DOI 10.1097/MCG.0b013e31828c6822
HARBIN WP, 1980, RADIOLOGY, V135, P15, DOI 10.1148/radiology.135.1.6987704
Hedjoudje A, 2019, UNITED EUR GASTROENT, V7, P60, DOI 10.1177/2050640618808147
Huibregtse KKM, 1995, TEXTBOOK OF GASTROEN
Jacques J, 2019, ENDOSCOPY, V51, P540, DOI 10.1055/a-0735-9137
Khan MA, 2016, DIGEST DIS SCI, V61, P684, DOI 10.1007/s10620-015-3933-0
Khashab MA, 2016, ENDOSC INT OPEN, V4, pE175, DOI 10.1055/s-0041-109083
Li MW, 2015, CARDIOVASC INTER RAD, V38, P709, DOI 10.1007/s00270-014-0992-0
Martins FP, 2010, ENDOSCOPY, V42, pE126, DOI 10.1055/s-0029-1243911
Ogura T, 2016, ENDOSCOPY, V48, P156, DOI 10.1055/s-0034-1392859
OKEN MM, 1982, AM J CLIN ONCOL-CANC, V5, P649, DOI 10.1097/00000421-198212000-00014
Park DH, 2011, GASTROINTEST ENDOSC, V74, P1276, DOI 10.1016/j.gie.2011.07.054
Poincloux L, 2015, ENDOSCOPY, V47, P794, DOI [10.1055/s-0035-1545399, 10.1055/s-0034-1391988]
Sharaiha RZ, 2017, GASTROINTEST ENDOSC, V85, P904, DOI 10.1016/j.gie.2016.12.023
Tyberg A, 2016, GASTROINTEST ENDOSC, V84, P941, DOI 10.1016/j.gie.2016.05.035
Wang KX, 2016, GASTROINTEST ENDOSC, V83, P1218, DOI 10.1016/j.gie.2015.10.033
NR 29
TC 10
Z9 11
U1 0
U2 5
PU ELSEVIER SCIENCE INC
PI NEW YORK
PA STE 800, 230 PARK AVE, NEW YORK, NY 10169 USA
SN 1590-8658
EI 1878-3562
J9 DIGEST LIVER DIS
JI Dig. Liver Dis.
PD JAN
PY 2020
VL 52
IS 1
BP 57
EP 63
DI 10.1016/j.dld.2019.07.009
PG 7
WC Gastroenterology & Hepatology
WE Science Citation Index Expanded (SCI-EXPANDED)
SC Gastroenterology & Hepatology
GA JY9UN
UT WOS:000504751100009

PM 31409577
DA 2026-05-09
ER

PT J
AU Prachayakul, V
Aswakul, P
AF Prachayakul, Varayu
Aswakul, Pitulak
TI Feasibility and safety of using Soehendra stent retriever as a new
technique for biliary access in endoscopic ultrasound-guided biliary
drainage
SO WORLD JOURNAL OF GASTROENTEROLOGY
LA English
DT Article
DE Soehendra stent retriever; Endoscopic ultrasound-guided
choledochoduodenostomy; Neotract creation; Endoscopic ultrasound-guided
biliary drainage; Endoscopic ultrasound-guided hepaticogastrostomy
ID HEPATICOGASTROSTOMY
AB AIM: To assess the feasibility and safety of the use of soehendra stent retriever as a new technique for biliary access in endoscopic ultrasound-guided biliary drainage.
METHODS: The medical records and endoscopic reports of the patients who underwent endoscopic ultrasound-guided biliary drainage (EUS-BD) owing to failed endoscopic retrograde cholangiopancreatography in our institute between June 2011 and January 2014 were collected and reviewed. All the procedures were performed in the endoscopic suite under intravenous sedation with propofol and full anaesthetic monitoring. Then we used the Soehendra stent retriever as new equipment for neo-tract creation and dilation when performing EUS-BD procedures. The patients were observed in the recovery room for 1-2 h and transferred to the regular ward, patients' clinical data were reviewed and analysed, clinical outcomes were defined by using several different criteria. Data were analysed by using SPSS 13 and presented as percentages, means, and medians.
RESULTS: A total of 12 patients were enrolled. The most common indications for EUS-BD in this series were failed common bile duct cannulation, duodenal obstruction, failed selective intrahepatic duct cannulation, and surgical altered anatomy for 50%, 25%, 16.7%, and 8.3%, respectively. Seven patients underwent EUS-guided hepaticogastrostomy (58.3%), and 5 underwent EUS-guided choledochoduodenostomy (41.7%). The technical success rate was 100%, while the clinical success rate was 91.7%. Major and minor complications occurred in 16.6% and 33.3% of patients, respectively, but there were no procedurerelated death.
CONCLUSION: Soehendra stent retriever could be used as an alternative instrument for biliary access in endoscopic ultrasound guided biliary drainage.
C1 [Prachayakul, Varayu] Mahidol Univ, Siriraj Hosp, Fac Med, Siriraj GI Endoscopy Ctr,Div Gastroenterol,Dept I, Bangkok 10700, Thailand.
[Aswakul, Pitulak] Samitivej Sukhumvit Hosp, Liver & Digest Inst, Bangkok 10120, Thailand.
C3 Mahidol University
RP Prachayakul, V (corresponding author), Mahidol Univ, Siriraj Hosp, Fac Med, Siriraj GI Endoscopy Ctr,Div

Gastroenterol,Dept I, Bangkok 10700, Thailand.
EM kaiyjr@gmail.com
CR Attasaranya S, 2012, GASTROENT RES PRACT, V2012, DOI 10.1155/2012/680753
Giovannini M, 2012, GASTROENT RES PRACT, V2012, DOI 10.1155/2012/348719
Irisawa A, 2009, J HEPATO-BILIARY-PAN, V16, P598, DOI 10.1007/s00534-009-0131-5
Itoi T, 2010, J HEPATO-BIL-PAN SCI, V17, P611, DOI 10.1007/s00534-009-0196-1
Kato H, 2013, HEPATO-GASTROENTEROL, V60, P286, DOI 10.5754/hge11325
Kim TH, 2012, WORLD J GASTROENTERO, V18, P2526, DOI 10.3748/wjg.v18.i20.2526
Levy Michael J, 2010, Curr Gastroenterol Rep, V12, P141, DOI 10.1007/s11894-010-0090-7
Park DH, 2011, GASTROINTEST ENDOSC, V74, P1276, DOI 10.1016/j.gie.2011.07.054
Prachayakul V, 2013, ENDOSCOPY, V45, pE223, DOI 10.1055/s-0033-1344064
Prachayakul V, 2011, ENDOSCOPY, V43, pE213, DOI 10.1055/s-0030-1256395
Prachayakul V, 2013, WORLD J GASTROENTERO, V19, P4758, DOI 10.3748/wjg.v19.i29.4758
Tarantino I, 2012, WORLD J GASTRO ENDOS, V4, P306, DOI 10.4253/wjge.v4.i7.306
Vila JJ, 2010, AM J GASTROENTEROL, V105, P959, DOI 10.1038/ajg.2009.690
Yamaguchi T, 2004, ENDOSCOPY, V36, P755, DOI 10.1055/s-2004-825666
NR 14
TC 12
Z9 13
U1 0
U2 2
PU BAISHIDENG PUBLISHING GROUP INC
PI PLEASANTON
PA 8226 REGENCY DR, PLEASANTON, CA 94588 USA
SN 1007-9327
EI 2219-2840
J9 WORLD J GASTROENTERO
JI World J. Gastroenterol.
PD MAR 7
PY 2015
VL 21
IS 9
BP 2725
EP 2730
DI 10.3748/wjg.v21.i9.2725
PG 6
WC Gastroenterology & Hepatology
WE Science Citation Index Expanded (SCI-EXPANDED)
SC Gastroenterology & Hepatology
GA CC9KJ
UT WOS:000350688400019
PM 25759542

OA hybrid
DA 2026-05-09
ER

PT J
AU Lyu, YX
Li, T
Cheng, YX
Wang, B
Cao, YC
Wang, Y
AF Lyu, Yunxiao
Li, Ting
Cheng, Yunxiao
Wang, Bin
Cao, Yuchen
Wang, Yuan
TI Endoscopic ultrasound-guided vs ERCP-guided biliary drainage for malignant biliary obstruction: A up-to-date meta-analysis and systematic review
SO DIGESTIVE AND LIVER DISEASE
LA English
DT Review
DE Endoscopic ultrasound-guided biliary; drainage; Malignant biliary; Obstruction
ID METAL STENTS; CHOLEDOCHODUODENOSTOMY; PLACEMENT
AB Endoscopic ultrasound-guided biliary drainage (EUS-BD) is being used increasingly as an alternative treatment for malignant biliary obstruction (MBO). However, few studies have compared EUS-BD and endoscopic retrograde cholangiopancreatography biliary drainage (ERCP-BD). We searched the PubMed, Embase, Web of Science, Cochrane Central Register of Controlled Trials, and ClinicalTrials.gov databases until 1 November 2020 for studies comparing EUS-BD versus ERCP-BD. The primary outcomes of interest in this study were technical and clinical success. Nine studies involving 634 patients were included in this meta-analysis. Regarding technical and clinical success, there were no significant differences between EUS-BD and ERCP-BD (odds ratio [OR], 0.76; 95% CI: 0.30-1.91; OR, 1.45, 95% confidence interval [CI], 0.66-3.16, respectively). EUS-BD was associated with significantly less reintervention vs ERCP-BD (OR, 0.36, 95% CI, 0.15-0.86). Regarding adverse events, the rates were similar for EUS-BD and ERCP-BD (OR: 0.75, 95% CI, 0.45-1.24). There were no significant differences in the types of adverse events (stent occlusion, stent migration, stent dysfunction, and duration of stent patency) between the two techniques. EUS-BD was associated with lower reintervention rates compared with ERCP-BD, with comparable safety and efficacy outcomes. However, more high-quality randomized trials are required. (c) 2021 Editrice Gastroenterologica Italiana S.r.l. Published by Elsevier Ltd. All rights reserved.
C1 [Lyu, Yunxiao; Cheng, Yunxiao; Wang, Bin] Wenzhou Med Univ, Dongyang Hosp, Dongyang Peoples Hosp, Dept Hepatobiliary Surg, 60 West Wuning Rd, Dongyang 322100, Zhejiang, Peoples R China.

[Li, Ting] Wenzhou Med Univ, Dongyang Hosp, Dept Personnel Off, Dongyang 322100, Zhejiang, Peoples R China.
[Cao, Yuchen] Zhejiang Chinese Med Univ, Dept Clin Med, Hangzhou 310000, Zhejiang, Peoples R China.
[Wang, Yuan] Dongyang Hosp Tradit Chinses Med, Dept Pharm, Dongyang 322100, Zhejiang, Peoples R China.
C3 Wenzhou Medical University; Wenzhou Medical University; Zhejiang Chinese Medical University
RP Lyu, YX (corresponding author), Wenzhou Med Univ, Dongyang Hosp, Dongyang Peoples Hosp, Dept Hepatobiliary Surg, 60 West Wuning Rd, Dongyang 322100, Zhejiang, Peoples R China.
EM yunxiao.lyu@hotmail.com
CR Artifon ELA, 2015, ENDOSC ULTRASOUND, V4, P235, DOI 10.4103/2303-9027.163010
Bang JY, 2018, GASTROINTEST ENDOSC, V88, P9, DOI 10.1016/j.gie.2018.03.012
Conio M, 2001, CRIT REV ONCOL HEMAT, V37, P127, DOI 10.1016/S1040-8428(00)00108-6
Dhir V, 2015, GASTROINTEST ENDOSC, V81, P913, DOI 10.1016/j.gie.2014.09.054
Donnan E, 2016, J SURG ONCOL, V113, P647, DOI 10.1002/jso.24191
Enochsson L, 2010, GASTROINTEST ENDOSC, V72, P1175, DOI 10.1016/j.gie.2010.07.047
Giovannini M, 2001, ENDOSCOPY, V33, P898, DOI 10.1055/s-2001-17324
Hamada T, 2018, SCAND J GASTROENTERO, V53, P46, DOI 10.1080/00365521.2017.1382567
Han SY, 2019, SCI REP-UK, V9, DOI 10.1038/s41598-019-52993-x
Higgins JPT, 2003, BMJ-BRIT MED J, V327, P557, DOI 10.1136/bmj.327.7414.557
Higgins JPT, 2011, BMJ-BRIT MED J, V343, DOI 10.1136/bmj.d5928
Isayama H, 2006, CLIN GASTROENTEROL H, V4, P1148, DOI 10.1016/j.cgh.2006.06.004
Jin Z, 2020, DIGEST ENDOSC, V32, P16, DOI 10.1111/den.13456
Kawakubo K, 2016, ENDOSCOPY, V48, P164, DOI 10.1055/s-0034-1393179
Li DF, 2019, REV ESP ENFERM DIG, V111, P953, DOI 10.17235/reed.2019.6125/2018
Moher D, 2010, INT J SURG, V8, P336, DOI 10.1016/j.ijsu.2010.02.007
Moss AC, 2007, EUR J GASTROEN HEPAT, V19, P1119, DOI 10.1097/MEG.0b013e3282f16206
Nakai Y, 2019, ENDOSC ULTRASOUND, V8, P111, DOI 10.4103/eus.eus_17_18
Paik WH, 2018, AM J GASTROENTEROL, V113, P987, DOI 10.1038/s41395-018-0122-8
Park JK, 2018, GASTROINTEST ENDOSC, V88, P277, DOI 10.1016/j.gie.2018.03.015
Peterson J, 2015, NEWCASTLE OTTAWA SCA
Puga M, 2019, REV ESP ENFERM DIG, V111, P683, DOI 10.17235/reed.2019.6225/2019
Ridtitid W, 2016, J GASTROEN HEPATOL, V31, P272
Sharaiha RZ, 2017, GASTROINTEST ENDOSC, V85, P904, DOI 10.1016/j.gie.2016.12.023
Téllez-Avila FI, 2021, SURG ENDOSC, V35, P2531, DOI 10.1007/s00464-020-07667-5
Tol JAMG, 2016, GUT, V65, P1981, DOI 10.1136/gutjnl-2014-308762
NR 26
TC 27
Z9 35
U1 0
U2 7
PU ELSEVIER SCIENCE INC
PI NEW YORK
PA STE 800, 230 PARK AVE, NEW YORK, NY 10169 USA

SN 1590-8658
EI 1878-3562
J9 DIGEST LIVER DIS
JI Dig. Liver Dis.
PD OCT
PY 2021
VL 53
IS 10
BP 1247
EP 1253
DI 10.1016/j.dld.2021.03.029
EA SEP 2021
PG 7
WC Gastroenterology & Hepatology
WE Science Citation Index Expanded (SCI-EXPANDED)
SC Gastroenterology & Hepatology
GA WH0QU
UT WOS:000707394700006
PM 33926814
DA 2026-05-09
ER

PT J
AU Itoi, T
Itokawa, F
Kurihara, T
AF Itoi, Takao
Itokawa, Fumihide
Kurihara, Toshio
TI Endoscopic ultrasonography-guided gallbladder drainage: actual technical
presentations and review of the literature (with videos)
SO JOURNAL OF HEPATO-BILIARY-PANCREATIC SCIENCES
LA English
DT Review
DE Endoscopic ultrasonography; Gallbladder drainage; Interventional EUS
ID HIGH-RISK PATIENTS; ACUTE CHOLECYSTITIS; PERCUTANEOUS CHOLECYSTOSTOMY;
CHOLECYSTECTOMY; STENT; MANAGEMENT; ASPIRATION; DISEASE
AB Endoscopic ultrasonography-guided gallbladder drainage (EUS-GBD) has been developed as an alternative drainage method in patients with acute cholangitis. Here, we describe two successful EUS-GBD cases and review the literature on this topic.
EUS-GBD was conducted using a curved linear array echoendoscope and a 19-gauge needle.
A 7-Fr double pigtail stent was successfully placed transgastrically in one patient and transduodenally in the other

patient, without any serious early adverse events in either patient. No late complications or relapse of acute cholecystitis have been seen during the 3- to 6-month follow-up period.

EUS-GBD holds high potential as an alternative gallbladder decompression procedure. However, because current experience is limited, multicenter trials for the accurate evaluation of this procedure appear to be necessary in the near future.

C1 [Itoi, Takao; Itokawa, Fumihide; Kurihara, Toshio] Tokyo Med Univ, Dept Gastroenterol & Hepatol, Shinjuku Ku, Tokyo, Japan.

C3 Tokyo Medical University

RP Itoi, T (corresponding author), Tokyo Med Univ, Dept Gastroenterol & Hepatol, Shinjuku Ku, 6-7-1 Nishishinjuku, Tokyo, Japan.

EM itoi@tokyo-med.ac.jp

CR Baron TH, 2007, GASTROINTEST ENDOSC, V65, P735, DOI 10.1016/j.gie.2006.07.041

Binmoeller KF, 2009, GASTROINTEST ENDOSC, V69, pAB322, DOI 10.1016/j.gie.2009.03.923

Chopra S, 2001, AM J ROENTGENOL, V176, P1025, DOI 10.2214/ajr.176.4.1761025

Conway JD, 2005, GASTROINTEST ENDOSC, V61, P32, DOI 10.1016/S0016-5107(04)02445-9

FRAZEE RC, 1989, MAYO CLIN PROC, V64, P163, DOI 10.1016/S0025-6196(12)65670-5

Hirota M, 2007, J HEPATO-BILIARY-PAN, V14, P78, DOI 10.1007/s00534-006-1159-4

HOUGHTON PWJ, 1985, BRIT J SURG, V72, P220, DOI 10.1002/bjs.1800720327

Ito K, 2004, AM J ROENTGENOL, V183, P193, DOI 10.2214/ajr.183.1.1830193

Itoi T, 2009, J HEPATOBILIARY PANC

Itoi T, 2008, GASTROINTEST ENDOSC, V68, P455, DOI 10.1016/j.gie.2008.02.052

Itoi T, 2010, GASTROINTEST ENDOSC, V71, P1038, DOI 10.1016/j.gie.2010.01.026

Kahaleh M, 2004, GASTROINTEST ENDOSC, V60, P640, DOI 10.1016/S0016-5107(04)01959-5

KALLOO AN, 1994, GASTROINTEST ENDOSC, V40, P608, DOI 10.1016/S0016-5107(94)70263-2

Kwan V, 2007, GASTROINTEST ENDOSC, V66, P582, DOI 10.1016/j.gie.2007.02.065

Lee SS, 2007, GASTROINTEST ENDOSC, V66, P1008, DOI 10.1016/j.gie.2007.03.1080

NAHRWOLD DL, 1997, ACUTE CHOLECYSTITIS, P1126

Pannala R, 2008, Minerva Gastroenterol Dietol, V54, P107

Patterson EJ, 1996, SURG ENDOSC, V10, P1185, DOI 10.1007/s004649900275

Song TJ, 2010, GASTROINTEST ENDOSC, V71, P634, DOI 10.1016/j.gie.2009.11.024

Takasawa O, 2009, DIGEST ENDOSC, V21, P43, DOI 10.1111/j.1443-1661.2008.00822.x

NR 20

TC 44

Z9 48

U1 0

U2 2

PU SPRINGER TOKYO

PI TOKYO

PA 1-11-11 KUDAN-KITA, CHIYODA-KU, TOKYO, 102-0073, JAPAN

SN 1868-6974

J9 J HEPATO-BIL-PAN SCI

JI J. Hepato-Biliary-Pancreat. Sci.

PD MAR
PY 2011
VL 18
IS 2
BP 282
EP 286
DI 10.1007/s00534-010-0310-4
PG 5
WC Gastroenterology & Hepatology; Surgery
WE Science Citation Index Expanded (SCI-EXPANDED)
SC Gastroenterology & Hepatology; Surgery
GA 737HQ
UT WOS:000288560200022
PM 20652716
DA 2026-05-09
ER

PT J
AU Seicean, A
Pojoga, C
Mostean, O
Bolboaca, SD
Ilie, M
Rimbas, M
Gheorghiu, M
Lucaciu, L
Bartos, A
Al Hajjar, N
Sandru, V
Constantinescu, G
Seicean, R
AF Seicean, Andrada
Pojoga, Cristina
Mostean, Ofelia
Bolboaca, Sorana D.
Ilie, Madalina
Rimbas, Mihai
Gheorghiu, Marcel
Lucaciu, Laura
Bartos, Adrian
Al Hajjar, Nadim
Sandru, Vasile

Constantinescu, Gabriel
Seicean, Radu

TI What is the Impact of the Proportion of Solid Necrotic Content on the Number of Necrosectomies during EUS-Guided Drainage using Lumen-Apposing Metallic Stents of Pancreatic Walled-off Necrosis?

SO JOURNAL OF GASTROINTESTINAL AND LIVER DISEASES

LA English

DT Article

DE endosonography; drainage; pancreatic necrosis; metallic stent; acute pancreatitis

ID FLUID COLLECTIONS; ENDOSCOPIC ULTRASOUND; MANAGEMENT; INTERVENTION; CT

AB Background & Aims: The fully-covered, lumen apposing metal stents are designed for one step placement, facilitating the direct endoscopic necrosectomy into the walled-off pancreatic necrosis. However, the prediction of the number of necrosectomy sessions in these patients is not known. This study evaluated the association between the proportion of solid necrotic material inside walled-off necrosis, as assessed during the endosonography placement of a lumen apposing metal stent, and the number of necrosectomies subsequently required.

Methods: Patients from three tertiary medical centers with symptomatic walled off pancreatic necrosis (pain, infection, gastric/biliary obstruction) at more than 4 weeks after onset of acute pancreatitis were retrospectively analysed. Proportion of solid necrotic debris was estimated during endosonography procedure of lumen apposing metal stents placement. Necrosectomy was performed when obstruction or inflammation occurred subsequently. Lumen apposing metal stents were removed after clearance of necrotic content.

Results: In 46 patients with successful lumen apposing metal stents placement, necrosectomy was performed in 39 patients (72.78%). Performance of 3 or more necrosectomies was significantly associated with more than 50% pancreatic necrosis (p=0.032), but not with walled-off pancreatic necrosis size or location. Necrotic infection during lumen apposing metal stents stenting was associated with hypoalbuminemia, but not with necrosectomy requirement. Clinical success after a median follow-up of 13.37 months was 87%.

Conclusions: Walled-off pancreatic necrosis with more than 50% solid necrotic content were associated with more necrosectomy procedures, requiering longer endoscopy time, intravenous sedations, and higher costs.

C1 [Seicean, Andrada; Mostean, Ofelia; Bolboaca, Sorana D.; Gheorghiu, Marcel; Lucaciu, Laura; Bartos, Adrian; Al Hajjar, Nadim; Seicean, Radu] Iuliu Hatieganu Univ Med & Pharm, Cluj Napoca, Romania.

[Seicean, Andrada; Pojoga, Cristina; Mostean, Ofelia; Bartos, Adrian; Al Hajjar, Nadim] Reg Inst Gastroenterol & Hepatol, 19-23 Croitorilor St, Cluj Napoca 400192, Romania.

[Pojoga, Cristina] Babes Bolyai Univ Cluj Napoca, Clin Psychol & Psychotherapy Dept, Cluj Napoca, Romania.

[Bolboaca, Sorana D.] Dept Med Informat & Biostat, Bucharest, Romania.

[Ilie, Madalina; Rimbas, Mihai; Sandru, Vasile; Constantinescu, Gabriel] Carol Davila Univ Med & Pharm, Bucharest, Romania.

[Ilie, Madalina; Sandru, Vasile; Constantinescu, Gabriel] Floreasca Emergency Hosp, Bucharest, Romania.

[Rimbas, Mihai] Colentina Clin Hosp, Bucharest, Romania.

[Seicean, Radu] First Surg Clin, Cluj Napoca, Romania.

C3 Iuliu Hatieganu University of Medicine & Pharmacy; Regional Institute of Gastroenterology & Hepatology; Babes Bolyai University from Cluj; Carol Davila University of Medicine & Pharmacy

RP Seicean, A (corresponding author), Reg Inst Gastroenterol & Hepatol, 19-23 Croitorilor St, Cluj Napoca 400192, Romania.
EM andradaseicean@gmail.com
RI ; Al Hajjar, Nadim/I-3147-2017; Rimbas, Mihai/AAF-5521-2020; Ilie, Madalina/NVM-1777-2025; Sandru, Vasile/C-4861-2019; Pojoga, Cristina/JPK-8791-2023; Seicean, Anada/C-4057-2011; Seicean, Radu/IUP-6041-2023; Bolboacă, Sorana D/E-4457-2010
OI GHEORGHIU, Marcel/0000-0003-1886-8791; Al Hajjar, Nadim/0000-0001-5986-1233; Seicean, Anada/0000-0002-5809-1334; Bolboacă, Sorana D/0000-0002-2342-4311
CR Alali A, 2017, CLIN ENDOSC, V50, P117, DOI 10.5946/ce.2017.045
Arvanitakis M, 2018, ENDOSCOPY, V50, P524, DOI 10.1055/a-0588-5365
Bang JY, 2019, GASTROENTEROLOGY, V156, P1027, DOI 10.1053/j.gastro.2018.11.031
Baron TH, 2020, GASTROENTEROLOGY, V158, P67, DOI 10.1053/j.gastro.2019.07.064
Brimhall B, 2018, CLIN GASTROENTEROL H, V16, P1521, DOI 10.1016/j.cgh.2018.02.021
Chen YI, 2019, ENDOSC INT OPEN, V7, pE347, DOI 10.1055/a-0828-7630
Dhaka N, 2015, WORLD J GASTROENTERO, V21, P13403, DOI 10.3748/wjg.v21.i48.13403
Gornals JB, 2016, SURG ENDOSC, V30, P2592, DOI 10.1007/s00464-015-4505-2
Guo JT, 2020, SURG ENDOSC, V34, P1177, DOI 10.1007/s00464-019-06870-3
Guo JT, 2017, ENDOSC ULTRASOUND, V6, P285, DOI 10.4103/eus.eus_85_17
Hammad T, 2018, DIGEST DIS SCI, V63, P289, DOI 10.1007/s10620-017-4851-0
Garcia-Alonso FJ, 2018, ENDOSCOPY, V50, P386, DOI 10.1055/a-0581-9040
Kamal A, 2015, ABDOM IMAGING, V40, P1608, DOI 10.1007/s00261-014-0303-x
Kumta NA, 2019, DIGEST LIVER DIS, V51, P1557, DOI 10.1016/j.dld.2019.05.033
Manrai M, 2018, ANN SURG, V267, P357, DOI 10.1097/SLA.0000000000002065
Medarapalem JB, 2014, Gastrointest. Endosc, V79, pAB445, DOI [10.1016/j.gie.2014, DOI 10.1016/J.GIE.2014.02.637]]
Morgan DE, 1997, RADIOLOGY, V203, P773, DOI 10.1148/radiology.203.3.9169703
Patra PS, 2014, BRIT J SURG, V101, P1721, DOI 10.1002/bjs.9666
Porter KK, 2019, J AM COLL RADIOL, V16, pS316, DOI [DOI 10.1016/J.JACR.2019.05.017, 10.1016/j.jacr.2019.05.017]
Rana SS, 2016, GASTROENTEROL REP, V4, P50, DOI 10.1093/gastro/gou088
Rana SS, 2014, ENDOSC ULTRASOUND, V3, P118, DOI 10.4103/2303-9027.131039
Sharaiha RZ, 2016, CLIN GASTROENTEROL H, V14, P1797, DOI 10.1016/j.cgh.2016.05.011
Siddiqui AA, 2016, GASTROINTEST ENDOSC, V83, P699, DOI 10.1016/j.gie.2015.10.020
Tarantino I, 2017, ENDOSC INT OPEN, V5, pE784, DOI 10.1055/s-0043-112494
Teoh AY, 2016, GASTROINTEST ENDOSC, V84, P62, DOI 10.1016/j.gie.2015.11.041
Teoh AYB, 2020, DIGEST ENDOSC, V32, P391, DOI 10.1111/den.13494
van Brunschot S, 2018, LANCET, V391, P51, DOI 10.1016/S0140-6736(17)32404-2
van Grinsven J, 2019, TRIALS, V20, DOI 10.1186/s13063-019-3315-6
van Santvoort HC, 2011, GASTROENTEROLOGY, V141, P1254, DOI 10.1053/j.gastro.2011.06.073
Varadarajulu S, 2011, J GASTROINTEST SURG, V15, P2080, DOI 10.1007/s11605-011-1621-8

Yan L, 2019, ENDOSC ULTRASOUND, V8, P172, DOI 10.4103/eus.eus_108_17
Yoon SB, 2018, UNITED EUR GASTROENT, V6, P729, DOI 10.1177/2050640618761702
NR 32
TC 19
Z9 25
U1 0
U2 7
PU MEDICAL UNIV PRESS
PI CLUJ-NAPOCA
PA 3RD MEDICAL CLINIC, STR CROITORILOR NO 19-21, CLUJ-NAPOCA, 400162, ROMANIA
SN 1841-8724
EI 1842-1121
J9 J GASTROINTEST LIVER
JI J. Gastrointest. Liver Dis.
PD DEC
PY 2020
VL 29
IS 4
BP 623
EP 628
DI 10.15403/jgld-3128
PG 6
WC Gastroenterology & Hepatology
WE Science Citation Index Expanded (SCI-EXPANDED)
SC Gastroenterology & Hepatology
GA PE6PK
UT WOS:000598486100023
PM 33331355
OA gold
DA 2026-05-09
ER

PT J
AU Kaplan, J
Khalid, A
Cosgrove, N
Soomro, A
Mazhar, SM
Siddiqui, AA
AF Kaplan, Jeremy
Khalid, Amaara

Cosgrove, Natalie
Soomro, Ayesha
Mazhar, Syed Mohsin
Siddiqui, Ali A.
TI Endoscopic ultrasound-fine needle injection for oncological therapy
SO WORLD JOURNAL OF GASTROINTESTINAL ONCOLOGY
LA English
DT Review
DE Endoscopic ultrasound-fine needle injection; Endoscopic
ultrasound-guided ablation; Photodynamic therapy; Radiofrequency
ablation; Cryothermal ablation; Endoscopic ultrasound-guided
brachytherapy; Fiducial markers; Endoscopic ultrasound-guided antitumor
agent injection
AB The minimal invasiveness and precision of endoscopic ultrasound (EUS) has lead to both its widespread use as a diagnostic and staging modality for gastrointestinal and pancreaticobiliary malignancies, and to its expanding role as a therapeutic modality. EUS-guided celiac plexus neurolysis is now a well-accepted modality for palliation of pain in patients with pancreatic cancer. EUS-guided ablation, brachytherapy, fiducial marker placement, and antitumor agent injection have been described as methods of performing minimally invasive oncological therapy. EUS-fine needle injection may be performed as adjunctive, alternative, or palliative treatment. This review summarizes the studies to date that have described these methods. A literature search using the PubMed/MEDLINE databases was performed. While most published studies to date are limited with disappointing outcomes, the concept of a role of EUS in oncological therapy seems promising.
C1 [Kaplan, Jeremy; Khalid, Amaara; Cosgrove, Natalie; Soomro, Ayesha; Mazhar, Syed Mohsin; Siddiqui, Ali A.] Thomas Jefferson Univ Hosp, Div Gastroenterol & Hepatol, Dept Internal Med, Philadelphia, PA 19107 USA.
C3 Thomas Jefferson University
RP Siddiqui, AA (corresponding author), Thomas Jefferson Univ Hosp, Div Gastroenterol & Hepatol, Dept Internal Med, Med, 132 S 10th St,Suite 585, Philadelphia, PA 19107 USA.
EM ali.siddiqui@jefferson.edu
CR Agostinis P, 2011, CA-CANCER J CLIN, V61, P250, DOI 10.3322/caac.20114
[Anonymous], GASTROINTESTINAL END
Arcidiacono PG, 2012, GASTROINTEST ENDOSC, V76, P1142, DOI 10.1016/j.gie.2012.08.006
Artifon ELA, 2007, GASTROINTEST ENDOSC, V66, P1201, DOI 10.1016/j.gie.2007.05.051
Banchereau J, 2003, ANN NY ACAD SCI, V987, P180, DOI 10.1111/j.1749-6632.2003.tb06047.x
Barclay RL, 2002, GASTROINTEST ENDOSC, V55, P266, DOI 10.1067/mge.2002.120784
Bhutani MS, 2003, ENDOSCOPY, V35, pS54
Bown SG, 2002, GUT, V50, P549, DOI 10.1136/gut.50.4.549
Chan HH, 2004, GASTROINTEST ENDOSC, V59, P95, DOI 10.1016/S0016-5107(03)02361-7
Chang KJ, 2012, GASTROINTEST ENDOSC, V75, P1139, DOI 10.1016/j.gie.2012.01.042
Chang KJ, 2000, CANCER-AM CANCER SOC, V88, P1325, DOI 10.1002/(SICI)1097-0142(20000315)88:6<1325::AID-CNCR8>3.0.CO;2-T
Chen MH, 2005, WORLD J GASTROENTERO, V11, P6395, DOI 10.3748/wjg.v11.i40.6395
DeWitt J, 2010, GASTROINTEST ENDOSC, V72, P862, DOI 10.1016/j.gie.2010.02.039
Di Matteo F, 2010, GASTROINTEST ENDOSC, V72, P358, DOI 10.1016/j.gie.2010.02.027

Endo H, 2012, J HEPATO-BIL-PAN SCI, V19, P465, DOI 10.1007/s00534-011-0457-7
Fajardo RD, 2014, GASTROINTEST ENDOSC, V79, P851, DOI 10.1016/j.gie.2013.12.027
Fuccio L, 2014, GASTROINTEST ENDOSC, V80, P533, DOI 10.1016/j.gie.2014.03.028
Gan SI, 2005, GASTROINTEST ENDOSC, V61, P746, DOI 10.1016/S0016-5107(05)00320-2
Gomez V, 2015, GASTROINTEST ENDOSC, V81, pAB113, DOI 10.1016/j.gie.2015.03.009
Günter E, 2003, GASTROINTEST ENDOSC, V57, P113, DOI 10.1067/mge.2003.39
Hecht JR, 2012, GASTROINTEST ENDOSC, V75, P332, DOI 10.1016/j.gie.2011.10.007
Hecht JR, 2003, CLIN CANCER RES, V9, P555
Herman JM, 2013, J CLIN ONCOL, V31, P886, DOI 10.1200/JCO.2012.44.7516
Hirooka Y, 2009, PANCREAS, V38, pE69, DOI 10.1097/MPA.0b013e318197a9e3
Huggett MT, 2014, BRIT J CANCER, V110, P1698, DOI 10.1038/bjc.2014.95
Irisawa A, 2007, PANCREAS, V35, P189, DOI 10.1097/01.mpa.0000250141.25639.e9
Jin Z, 2008, ENDOSCOPY, V40, P314, DOI 10.1055/s-2007-995476
Jürgensen C, 2006, GASTROINTEST ENDOSC, V63, P1059, DOI 10.1016/j.gie.2005.10.034
Kanazawa Masashi, 2004, Gan To Kagaku Ryoho, V31, P1773
Lah JJ, 2005, GASTROINTEST ENDOSC, V62, P805, DOI 10.1016/j.gie.2005.07.019
Leslie T, 2012, BRIT J RADIOL, V85, P1363, DOI 10.1259/bjr/56737365
Li T, 2015, GASTROINTEST ENDOSC, V81, P1243, DOI 10.1016/j.gie.2014.12.019
Machiels M, 2015, GASTROINTEST ENDOSC, V82, P641, DOI 10.1016/j.gie.2015.03.1972
Maier W, 1999, J LARYNGOL OTOL, V113, P41, DOI 10.1017/S0022215100143117
Minsky B D, 1988, J Pain Symptom Manage, V3, P199, DOI 10.1016/0885-3924(88)90031-0
Moningi S, 2015, GASTROINTEST ENDOSC, V81, P765, DOI 10.1016/j.gie.2014.11.004
Moyer MT, 2015, GASTROINTEST ENDOSC, V81, pAB114, DOI 10.1016/j.gie.2015.03.010
Oh HC, 2008, GASTROINTEST ENDOSC, V67, P636, DOI 10.1016/j.gie.2007.09.038
Oh HC, 2013, GASTROINTEST ENDOSC, V77, P526, DOI 10.1016/j.gie.2012.10.033
Ohara K, 2013, MOL CLIN ONCOL, V1, P231, DOI 10.3892/mco.2012.47
OVERHOLT B, 1993, GASTROINTEST ENDOSC, V39, P73, DOI 10.1016/S0016-5107(93)70017-6
Pai M, 2015, WORLD J GASTRO SURG, V7, P52, DOI 10.4240/wjgs.v7.i4.52
Park WG, 2010, GASTROINTEST ENDOSC, V71, P513, DOI 10.1016/j.gie.2009.10.030
Pishvaian AC, 2006, GASTROINTEST ENDOSC, V64, P412, DOI 10.1016/j.gie.2006.01.048
Rasmussen H, 2002, CANCER GENE THER, V9, P951, DOI 10.1038/sj.cgt.7700518
REGULA J, 1995, GUT, V36, P67, DOI 10.1136/gut.36.1.67
Ries Stefan J, 2005, Future Oncol, V1, P763, DOI 10.2217/14796694.1.6.763
Schnurr M, 2001, CANCER RES, V61, P6445
Steel AW, 2011, GASTROINTEST ENDOSC, V73, P149, DOI 10.1016/j.gie.2010.09.031
Sun S, 2006, ENDOSCOPY, V38, P399, DOI 10.1055/s-2006-925253
Suzuki Rei, 2013, Diagn Ther Endosc, V2013, P157581, DOI 10.1155/2013/157581
Tang Zhe, 2008, Zhonghua Yi Xue Za Zhi, V88, P391
Varadarajulu S, 2010, ENDOSCOPY, V42, P423, DOI 10.1055/s-0029-1243989
Verna Elizabeth C, 2008, Therap Adv Gastroenterol, V1, P103, DOI 10.1177/1756283X08093887
VILMANN P, 1992, GASTROINTEST ENDOSC, V38, P172, DOI 10.1016/S0016-5107(92)70385-X
Wu YL, 2006, J SURG ONCOL, V94, P392, DOI 10.1002/jso.20580

Xu ZF, 2014, J CLIN ULTRASOUND, V42, P321, DOI 10.1002/jcu.22141
Yang JL, 2011, GASTROINTEST ENDOSC, V73, P1302, DOI 10.1016/j.gie.2011.01.045
Yang XJ, 2009, GASTROINTEST ENDOSC, V69, pS263, DOI 10.1016/j.gie.2008.12.220
Yusuf TE, 2008, GASTROINTEST ENDOSC, V67, P957, DOI 10.1016/j.gie.2007.08.020
NR 60
TC 6
Z9 10
U1 0
U2 3
PU BAISHIDENG PUBLISHING GROUP INC
PI PLEASANTON
PA 8226 REGENCY DR, PLEASANTON, CA 94588 USA
SN 1948-5204
J9 WORLD J GASTRO ONCOL
JI World J. Gastrointest. Oncol.
PD DEC 15
PY 2015
VL 7
IS 12
BP 466
EP 472
DI 10.4251/wjgo.v7.i12.466
PG 7
WC Oncology; Gastroenterology & Hepatology
WE Science Citation Index Expanded (SCI-EXPANDED)
SC Oncology; Gastroenterology & Hepatology
GA V9A6Z
UT WOS:000422139600010
PM 26691224
OA Green Submitted, hybrid
DA 2026-05-09
ER

PT J
AU Hammad, T
Khan, MA
Alastal, Y
Lee, W
Nawras, A
Ismail, MK
Kahaleh, M
AF Hammad, Tariq

Khan, Muhammad Ali
Alastal, Yaseen
Lee, Wade
Nawras, Ali
Ismail, Mohammad Kashif
Kahaleh, Michel
TI Efficacy and Safety of Lumen-Apposing Metal Stents in Management of Pancreatic Fluid Collections: Are They Better Than Plastic Stents? A Systematic Review and Meta-Analysis
SO DIGESTIVE DISEASES AND SCIENCES
LA English
DT Review
DE Lumen-apposing metal stents; Pancreatic fluid collections; Plastic stents; EUS; Walled off necrosis; Pancreatic pseudocysts
ID ULTRASOUND-GUIDED DRAINAGE; WALLED-OFF NECROSIS; ENDOSCOPIC ULTRASOUND; CLINICAL-EVALUATION; NECROSECTOMY; MULTICENTER; PSEUDOCYST; EXPERIENCE; OUTCOMES; THERAPY
AB Endoscopic ultrasound (EUS)-guided transmural drainage has been increasingly utilized as a first-line therapeutic modality for drainage of pancreatic fluid collections (PFC). Recently, lumen-apposing metal stents (LAMS) have been utilized for management of PFCs. We conducted a systematic review and meta-analysis to evaluate the cumulative efficacy and safety of LAMS in the management of PFC (primary outcome). We also compared the efficacy and safety of LAMS with multiple plastic stents (MPS) in the management of PFC (secondary outcome).

We searched Medline, Embase and Cochrane databases from inception to November 5, 2016, to identify studies (with ae10 patients) reporting technical success, clinical success, and adverse events (AE) of EUS-guided transmural drainage of PFC using LAMS. Weighted pooled rates (WPR) were calculated for technical success, clinical success and AE. Risk ratios (RR) were calculated and pooled to compare LAMS with MPS in terms of technical success, clinical success, and AE. Pooled mean difference (MD) was calculated to compare the number of endoscopic sessions required by each type of stent to achieve clinical success. All analyses were done using random effects model.

Eleven studies with 688 patients were included in this meta-analysis. WPR for technical success of LAMS in PFC management was 98% (96, 99%), (I (2) = 15%). WPR for clinical success was 93% (89, 96%) with moderate heterogeneity (I (2) = 50%). There was no difference in clinical success for pseudocysts (PP) versus walled-off pancreatic necrosis (WON) (P = 0.51). WPR for AE was 13% (9, 20%), (I (2) = 64%). AE were 10% more in WON as compared to PP (P = 0.009). Most common AE requiring intervention was stent migration (4.2%), followed by infection (3.8%), bleeding (2.4%), and stent occlusion (1.9%). Six studies with 504 patients compared the performance of LAMS with MPS. Pooled RR for technical success was 1.71 (0.38, 7.37). Pooled RR for clinical success was 0.37 (0.20, 0.67) in favor of LAMS. Pooled RR for AE was 0.39 (0.18, 0.84), (I (2) = 50%). Pooled MD for number of endoscopic sessions was - 0.84 (- 1.69, 0.01).

LAMS seem to have excellent efficacy and safety in the management of PFCs. They may be preferred over plastic stents as they are associated with better clinical success and lesser adverse events.
C1 [Hammad, Tariq; Alastal, Yaseen; Nawras, Ali] Univ Toledo, Div Gastroenterol & Hepatol, 2801 W Bancroft St, Toledo, OH 43606 USA.
[Hammad, Tariq] Baylor Coll Med, Div Gastroenterol & Hepatol, Houston, TX 77030 USA.
[Khan, Muhammad Ali; Ismail, Mohammad Kashif] Univ Tennessee, Ctr Hlth Sci, Div Gastroenterol & Hepatol,

Memphis, TN 38163 USA.
[Lee, Wade] Univ Toledo, Carlson Lib, 2801 W Bancroft St, Toledo, OH 43606 USA.
[Lee, Wade] Univ Toledo, Mulford Lib, 2801 W Bancroft St, Toledo, OH 43606 USA.
[Kahaleh, Michel] Weill Cornell Med Coll, Dept Gastroenterol & Hepatol, 1305 York Ave,4th Floor, New York, NY 10021 USA.
C3 University System of Ohio; University of Toledo; Baylor College of
Medicine; University of Tennessee System; University of Tennessee Health
Science Center; University System of Ohio; University of Toledo;
University System of Ohio; University of Toledo; Cornell University;
Weill Cornell Medicine
RP Kahaleh, M (corresponding author), Weill Cornell Med Coll, Dept Gastroenterol & Hepatol, 1305 York Ave,4th Floor, New York, NY 10021 USA.
EM mkahaleh@gmail.com
RI Lee-Smith, Wade/D-3361-2009; Kahaleh, Michel/A-9408-2011
OI Lee-Smith, Wade/0000-0003-1744-8525; Kahaleh, Michel/0000-0003-0836-6114
FU Boston Scientific; Olympus; Gore
FX Dr. Michel Kahaleh has received Grant support from Boston Scientific,
Olympus and Gore. He is a consultant for Boston Scientific and Olympus.
CR Akshintala VS, 2014, GASTROINTEST ENDOSC, V79, P921, DOI 10.1016/j.gie.2013.10.032
Ang TL, 2016, ENDOSC ULTRASOUND, V5, P320, DOI 10.4103/2303-9027.191659
[Anonymous], 2009, BMJ-BRIT MED J, V21, P7, DOI [DOI 10.1136/bmj.b2535, DOI 10.1136/BMJ.B2700, 10.7326/0003-4819-151-4-200908180-00135, 10.1371/journal.pmed.1000097, DOI 10.1371/JOURNAL.PMED.1000097]
[Anonymous], GUT
[Anonymous], DIG ENDOSC
[Anonymous], DIG ENDOSC
[Anonymous], QUAL ASS TOOL STUD N
Bakker OJ, 2012, JAMA-J AM MED ASSOC, V307, P1053, DOI 10.1001/jama.2012.276
Bapaye A, 2015, DIGEST ENDOSC, V27, P499, DOI 10.1111/den.12421
Baron TH, 1996, GASTROENTEROLOGY, V111, P755, DOI 10.1053/gast.1996.v111.pm8780582
Chandran S, 2015, GASTROINTEST ENDOSC, V81, P127, DOI 10.1016/j.gie.2014.06.025
Connor S, 2005, SURGERY, V137, P499, DOI 10.1016/j.surg.2005.01.003
DERSIMONIAN R, 1986, CONTROL CLIN TRIALS, V7, P177, DOI 10.1016/0197-2456(86)90046-2
Forsmark CE, 2007, GASTROENTEROLOGY, V132, P2022, DOI 10.1053/j.gastro.2007.03.065
Gornals JB, 2016, SURG ENDOSC, V30, P2592, DOI 10.1007/s00464-015-4505-2
Gornals JB, 2013, SURG ENDOSC, V27, P1428, DOI 10.1007/s00464-012-2591-y
Guyatt GH, 2008, BRIT MED J, V336, P924, DOI 10.1136/bmj.39489.470347.AD
Hookey LC, 2006, GASTROINTEST ENDOSC, V63, P635, DOI 10.1016/j.gie.2005.06.028
Itoi T, 2012, GASTROINTEST ENDOSC, V75, P870, DOI 10.1016/j.gie.2011.10.020
Jefferson T, 2007, COCHRANE DB SYST REV, DOI 10.1002/14651858.CD000975.pub2
Lakhtakia S, 2016, GASTROINTEST ENDOSC, V83, pAB343, DOI 10.1016/j.gie.2016.03.875
Mukai S, 2015, ENDOSC ULTRASOUND, V4, P120, DOI 10.4103/2303-9027.156738
Mukai S, 2015, J GASTROENTEROL, V50, P211, DOI 10.1007/s00535-014-0957-8

Mukai S, 2015, ENDOSCOPY, V47, P47, DOI 10.1055/s-0034-1377966
Poornachandra KS, 2011, J CLIN GASTROENTEROL, V45, P159, DOI 10.1097/MCG.0b013e3181dd9d14
Raijman I, 2015, ENDOSC ULTRASOUND, V4, P213, DOI 10.4103/2303-9027.163000
Rinninella E, 2015, GASTROINTEST ENDOSC, V82, P1039, DOI 10.1016/j.gie.2015.04.006
Seifert H, 2009, GUT, V58, P1260, DOI 10.1136/gut.2008.163733
Shah RJ, 2015, CLIN GASTROENTEROL H, V13, P747, DOI 10.1016/j.cgh.2014.09.047
Sharaiha RZ, 2016, CLIN GASTROENTEROL H, V14, P1797, DOI 10.1016/j.cgh.2016.05.011
Sheu Y, 2012, EMERG RADIOL, V19, P237, DOI 10.1007/s10140-011-1001-4
Siddiqui AA, 2017, GASTROINTEST ENDOSC, V85, P758, DOI 10.1016/j.gie.2016.08.014
Siddiqui AA, 2016, GASTROINTEST ENDOSC, V83, P699, DOI 10.1016/j.gie.2015.10.020
Singhal S, 2013, CLIN ENDOSC, V46, P506, DOI 10.5946/ce.2013.46.5.506
TADDIO A, 1994, CAN MED ASSOC J, V150, P1611
van Santvoort HC, 2010, NEW ENGL J MED, V362, P1491, DOI 10.1056/NEJMoa0908821
Varadarajulu S, 2011, J GASTROINTEST SURG, V15, P2080, DOI 10.1007/s11605-011-1621-8
Walter D, 2015, ENDOSCOPY, V47, P63, DOI 10.1055/s-0034-1378113
NR 38
TC 89
Z9 102
U1 1
U2 7
PU SPRINGER
PI DORDRECHT
PA VAN GODEWIJCKSTRAAT 30, 3311 GZ DORDRECHT, NETHERLANDS
SN 0163-2116
EI 1573-2568
J9 DIGEST DIS SCI
JI Dig. Dis. Sci.
PD FEB
PY 2018
VL 63
IS 2
BP 289
EP 301
DI 10.1007/s10620-017-4851-0
PG 13
WC Gastroenterology & Hepatology
WE Science Citation Index Expanded (SCI-EXPANDED)
SC Gastroenterology & Hepatology
GA FU9TA
UT WOS:000424198800005
PM 29282638
DA 2026-05-09

ER

PT J
AU Krafft, MR
Nasr, JY
AF Krafft, Matthew R.
Nasr, John Y.
TI Anterograde Endoscopic Ultrasound-Guided Pancreatic Duct Drainage: A Technical Review
SO DIGESTIVE DISEASES AND SCIENCES
LA English
DT Review
DE Anterograde endoscopic ultrasound-guided pancreatic duct drainage; Pancreaticogastrostomy; Endoscopic ultrasound; Chronic pancreatitis; Pancreaticojejunostomy; Disconnected pancreatic duct syndrome
ID RETROGRADE CHOLANGIOPANCREATOGRAPHY; HEAD RESECTION; FAILED ERCP; MULTICENTER; THERAPY; PAIN; PANCREATICODUODENECTOMY; PANCREATOGRAPHY; LITHOTRIPSY; OBSTRUCTION
AB The advancement of pancreatic endotherapy has increased the availability of minimally invasive endoscopic pancreatic ductal drainage techniques. In this regard, familiarity with endoscopic ultrasound-guided pancreatic duct drainage (EUS-PDD) is critical for treatment of obstructed pancreatic ductal systems, especially in nonsurgical candidates and in patients desiring a minimally invasive approach. Two distinct forms of EUS-PDD exist, viz. rendezvous-assisted endoscopic retrograde pancreatography (rendezvous-assisted ERP) and anterograde EUS-PDD. Anterograde EUS-PDD refers to transmural anterograde passage of a pancreatic drainage catheter or stent directly into the main pancreatic duct, through either the gastric or enteral wall. Rendezvous-assisted ERP should be attempted after failed conventional ERP, and anterograde EUS-PDD should be considered if rendezvous-assisted ERP fails or is not technically feasible. Common clinical scenarios that fulfil these conditions are chronic pancreatitis with high-grade main pancreatic duct obstruction, surgically altered anatomy with ductal/anastomotic obstruction, pancreas divisum, and disconnected pancreatic duct syndrome. The focus of this review article is anterograde EUS-PDD and its indications, technique, and outcomes. It also provides a summary of our own experience with this procedure, and a video demonstration of the technique.
C1 [Krafft, Matthew R.; Nasr, John Y.] West Virginia Univ Med, Sect Digest Dis, POB 9161,One Med Ctr Dr, Morgantown, WV 26506 USA.
RP Nasr, JY (corresponding author), West Virginia Univ Med, Sect Digest Dis, POB 9161,One Med Ctr Dr, Morgantown, WV 26506 USA.
EM mrkrafft@hsc.wvu.edu; jynasr@hsc.wvu.edu
CR Ahmed Ali U, 2015, COCHRANE DB SYST REV
Anderson MA, 2016, PANCREATOLOGY, V16, P83, DOI 10.1016/j.pan.2015.10.015
BEGER HG, 1980, CHIRURG, V51, P303
Brauer BC, 2009, GASTROINTEST ENDOSC, V70, P471, DOI 10.1016/j.gie.2008.12.233
Chahal P, 2006, ENDOSCOPY, V38, P1241, DOI 10.1055/s-2006-945003
Chandrasekhara V, 2015, GASTROINTEST ENDOSC, V82, P203, DOI 10.1016/j.gie.2015.04.022
Chen YI, 2017, GASTROINTEST ENDOSC, V85, P170, DOI 10.1016/j.gie.2016.07.031

Delhaye M, 2004, CLIN GASTROENTEROL H, V2, P1096, DOI 10.1016/S1542-3565(04)00544-0
Demirjian AN, 2010, HPB, V12, P482, DOI 10.1111/j.1477-2574.2010.00214.x
Diener MK, 2017, LANCET, V390, P1027, DOI 10.1016/S0140-6736(17)31960-8
Dumonceau JM, 2012, ENDOSCOPY, V44, P784, DOI 10.1055/s-0032-1309840
Dumonceau JM, 2007, GUT, V56, P545, DOI 10.1136/gut.2006.096883
Dumonceau JM, 1996, GASTROINTEST ENDOSC, V43, P547, DOI 10.1016/S0016-5107(96)70189-X
Ergun M, 2011, ENDOSCOPY, V43, P518, DOI 10.1055/s-0030-1256333
Farnbacher MJ, 2006, SCAND J GASTROENTERO, V41, P111, DOI 10.1080/00365520510024098
François E, 2002, GASTROINTEST ENDOSC, V56, P128, DOI 10.1067/mge.2002.125547
FREY CF, 1987, PANCREAS, V2, P701, DOI 10.1097/00006676-198711000-00014
Fujii LL, 2013, GASTROINTEST ENDOSC, V78, P854, DOI 10.1016/j.gie.2013.05.016
Fujii-Lau LL, 2015, J HEPATO-BIL-PAN SCI, V22, P51, DOI 10.1002/jhbp.187
Hobbs Paul M, 2016, World J Gastrointest Pharmacol Ther, V7, P370, DOI [10.4292/wjgpt.v7.i3.370, 10.4292/wjgpt.v7.i3.370]
Itoi T, 2013, DIGEST ENDOSC, V25, P241, DOI 10.1111/den.12048
Kahaleh M, 2007, GASTROINTEST ENDOSC, V65, P224, DOI 10.1016/j.gie.2006.05.008
KARANJIA ND, 1992, DIGEST DIS SCI, V37, P268, DOI 10.1007/BF01308182
Kurihara T, 2013, DIGEST ENDOSC, V25, P109, DOI 10.1111/den.12100
Löhr JM, 2017, UNITED EUR GASTROENT, V5, P153, DOI 10.1177/2050640616684695
Mizandari M, 2017, CARDIOVASC INTER RAD, V40, P1911, DOI 10.1007/s00270-017-1727-9
Morgan KA, 2010, HPB, V12, P211, DOI 10.1111/j.1477-2574.2009.00154.x
Oh D, 2016, GASTROINTEST ENDOSC, V83, P366, DOI 10.1016/j.gie.2015.07.015
PUESTOW CB, 1958, ARCH SURG-CHICAGO, V76, P898
REBER HA, 1992, GASTROENTEROLOGY, V103, P652, DOI 10.1016/0016-5085(92)90861-R
Reid-Lombardo KM, 2007, J GASTROINTEST SURG, V11, P1704, DOI 10.1007/s11605-007-0369-7
Renou C, 2000, INT J PANCREATOL, V27, P143, DOI 10.1385/IJGC:27:2:143
Rösch T, 2002, ENDOSCOPY, V34, P765, DOI 10.1055/s-2002-34256
Shah JN, 2012, GASTROINTEST ENDOSC, V75, P56, DOI 10.1016/j.gie.2011.08.032
Shimamura Y, 2017, CLIN ENDOSC, V50, P112, DOI 10.5946/ce.2017.046
Siddiqui UD, 2018, GASTROENTEROLOGY, V154, P1911, DOI 10.1053/j.gastro.2017.12.046
Tadenuma H, 2005, CLIN GASTROENTEROL H, V3, P1128, DOI 10.1016/S1542-3565(05)00530-6
Tessier G, 2007, GASTROINTEST ENDOSC, V65, P233, DOI 10.1016/j.gie.2006.06.029
TRAVERSO LW, 1978, SURG GYNECOL OBSTET, V146, P959
Tyberg A, 2017, GASTROINTEST ENDOSC, V85, P164, DOI 10.1016/j.gie.2016.07.030
Vila JJ, 2012, GASTROINTEST ENDOSC, V76, P1133, DOI 10.1016/j.gie.2012.08.001
Wilcox CM, 2015, CLIN GASTROENTEROL H, V13, P552, DOI 10.1016/j.cgh.2014.10.015
Will U, 2015, WORLD J GASTROENTERO, V21, P13140, DOI 10.3748/wjg.v21.i46.13140
NR 43
TC 39
Z9 48
U1 1
U2 4

PU SPRINGER
PI DORDRECHT
PA VAN GODEWIJCKSTRAAT 30, 3311 GZ DORDRECHT, NETHERLANDS
SN 0163-2116
EI 1573-2568
J9 DIGEST DIS SCI
JI Dig. Dis. Sci.
PD JUL
PY 2019
VL 64
IS 7
BP 1770
EP 1781
DI 10.1007/s10620-019-05495-9
PG 12
WC Gastroenterology & Hepatology
WE Science Citation Index Expanded (SCI-EXPANDED)
SC Gastroenterology & Hepatology
GA IE1TS
UT WOS:000472169300010
PM 30734236
DA 2026-05-09
ER

PT J
AU Vanella, G
Dell'Anna, G
Loria, A
Petrone, MC
Del Vecchio, A
Arcidiacono, PG
AF Vanella, Giuseppe
Dell'Anna, Giuseppe
Loria, Alessandro
Petrone, Maria Chiara
Del Vecchio, Antonella
Arcidiacono, Paolo Giorgio
TI Radiation exposure during modern therapeutic endoscopic ultrasound procedures and standard alternatives
SO ENDOSCOPY INTERNATIONAL OPEN
LA English
DT Article

ID EUROPEAN-SOCIETY

AB Background and study aims Therapeutic EUS (t-EUS) is increasingly being adopted in clinical practice in tertiary referral centers; however, little is known about radiation exposure (RE) metrics and diagnostic reference limits for it.

Methods Kerma-area product (KAP [Gy.cm(2)]), Air Kerma and fluoroscopy time were retrospectively evaluated for all consecutive t-EUS procedures performed in San Raffaele Institute between 2019 and 2021. For EUS-guided choledochoduodenostomies (EUS-CDS) and gastroenterostomies (EUS-GE), an equal number of endoscopic retrograde cholangiopancreatographies (ERCPs) plus metal stenting and duodenal stents were included respectively for comparison.

Results Data from 141 t-EUS procedures were retrieved (49% pancreatic cancer, 38% peripancreatic fluid collections). EUS-CDS (N =44) were mainly performed fluoroless, while ERCPs required a significantly higher RE (KAP =25 [17-55], P<0.0001). Fluid collection drainage (EUS-FCD) with lumen apposing metal stents (LAMS, N =26) were performed fluoroless, while EUS-FCD with double-pigtail plastic stents (DPPS, N=28) required higher RE (KAP=23 [13-45]). EUS-guided gallbladder drainage (EUS-GBD, N=6) required scarce RE (KAP =9 [3-21]) for coaxial DPPS placement. EUS-GE (N =27) required higher RE than duodenal stenting (KAP=44 [28-88] versus 29 [19-46], P=0.03). EUS-guided hepaticogastrostomies (EUS-HGS, N =10) had the highest RE among t-EUS procedures (KAP =81 [49-123]). Procedure complexity or intervening complications were evaluated and resulted in higher RE within each procedure.

Conclusions t-EUS procedures have different RE (P< 0.000001). EUS-CDS, EUS-GBD, and EUS-FCD with LAMS can be performed with no-to-mild radioscopy, unlike standard alternatives. However, radioscopy remains essential in case of technical difficulties or complications. EUS-GE and EUS-HGS involve a high RE. Endoscopists involved in t-EUS might experience RE higher than category standards, which indicates a need for increased awareness and personalized preventive measures.

C1 [Vanella, Giuseppe; Dell'Anna, Giuseppe; Petrone, Maria Chiara; Arcidiacono, Paolo Giorgio] IRCSS San Raffaele Sci Inst, Pancreatobil Endoscopy & Endosonog Div, Pancreas Translat & Clin Res Ctr, Milan, Italy.

[Loria, Alessandro; Del Vecchio, Antonella] IRCCS San Raffaele Sci Inst, Dept Med Phys, Milan, Italy.

C3 Vita-Salute San Raffaele University; IRCCS Ospedale San Raffaele;

Vita-Salute San Raffaele University; IRCCS Ospedale San Raffaele

RP Vanella, G (corresponding author), Univ Vita Salute San Raffaele, IRCCS San Raffaele Sci Inst, Pancreas Translat & Clin Res Ctr, Pancreatobil Endoscopy & Endosonog Div, Via Olgettina 60, I-20132 Milan, Italy.

EM g.e.vanella@gmail.com

RI Vanella, Giuseppe/LQI-8930-2024; Dell Anna, Giuseppe/IQT-0773-2023;

Petrone, Maria/K-8853-2016; Arcidiacono, Paolo Giorgio/K-2122-2018

OI Vanella, Giuseppe/0000-0001-7280-1761; Arcidiacono, Paolo

Giorgio/0000-0001-6692-7720

CR Bang JY, 2020, AM J GASTROENTEROL, V115, P555, DOI 10.14309/ajg.0000000000000565

Bang JY, 2019, GUT, V68, P1200, DOI 10.1136/gutjnl-2017-315335

Bronswijk Michiel, 2020, VideoGIE, V5, P442, DOI [10.1016/j.vgie.2020.06.012, 10.1016/j.vgie.2020.06.012]

Dumonceau JM, 2012, ENDOSCOPY, V44, P408, DOI 10.1055/s-0031-1291791

Hayashi S, 2022, LANCET REG HEALTH-W, V20, DOI 10.1016/j.lanwpc.2021.100376

Jin Z, 2020, DIGEST ENDOSC, V32, P16, DOI 10.1111/den.13456

Papanastasiou E, 2021, PHYS MEDICA, V89, P63, DOI 10.1016/j.ejmp.2021.07.029

Takenaka M, 2022, DIGEST ENDOSC, V34, P579, DOI 10.1111/den.14060

Teoh AYB, 2020, GUT, V69, P1085, DOI 10.1136/gutjnl-2019-319996

Testoni PA, 2016, ENDOSCOPY, V48, P657, DOI 10.1055/s-0042-108641

van der Merwe SW, 2022, ENDOSCOPY, V54, P185, DOI 10.1055/a-1717-1391
van Wanrooij RLJ, 2022, ENDOSCOPY, V54, P1023, DOI 10.1055/a-1782-7568
van Wanrooij RLJ, 2022, ENDOSCOPY, V54, P310, DOI 10.1055/a-1738-6780
Vanella G., 2022, ENDOSCOPY, DOI [10.1055/A-1792-2755, DOI 10.1055/A]
Vanella G, 2022, CLIN GASTROENTEROL H, V20, pE1507, DOI 10.1016/j.cgh.2021.07.002
Vanella Giuseppe, 2022, VideoGIE, V7, P120, DOI [10.1016/j.vgie.2021.10.002, 10.1016/j.vgie.2021.10.002]
Vanella G, 2020, ENDOSC INT OPEN, V08, pE1782, DOI 10.1055/a-1264-7511
Vano E, 2017, ANN ICRP, V46, p1 to 144
NR 18
TC 12
Z9 12
U1 0
U2 0
PU GEORG THIEME VERLAG KG
PI STUTTGART
PA RUDIGERSTR 14, D-70469 STUTTGART, GERMANY
SN 2364-3722
EI 2196-9736
J9 ENDOSC INT OPEN
JI Endosc. Int. Open
PD AUG
PY 2022
VL 10
IS 08
BP E1105
EP E1111
DI 10.1055/a-1853-0451
PG 7
WC Gastroenterology & Hepatology; Surgery
WE Emerging Sources Citation Index (ESCI)
SC Gastroenterology & Hepatology; Surgery
GA 3T7RP
UT WOS:000840469300011
PM 36247073
OA Green Submitted, gold
DA 2026-05-09
ER

PT J
AU Martinez-Moreno, B
López-Roldán, G
Martínez-Sempere, J

de-Madaria, E
Jover, R
Aparicio, JR
AF Martinez-Moreno, Belen
Lopez-Roldan, Gonzalo
Martinez-Sempere, Juan
de-Madaria, Enrique
Jover, Rodrigo
Aparicio, Jose Ramon
TI Long-term results after EUS gallbladder drainage in high-surgical-risk
patients with acute cholecystitis: A 3-year follow-up registry
SO ENDOSCOPY INTERNATIONAL OPEN
LA English
DT Article
DE Endoscopic ultrasonography; Intervention EUS; Biliary tract
ID APPOSING METAL STENT
AB Background and study aims Endoscopic ultrasound-guided gallbladder drainage (EUS-GBD) has become the favorite drainage option for high surgical-risk patients with acute cholecystitis. However, data on long-term outcomes regarding efficacy and security over 1 year are scarce.Patients and methods We performed a retrospective review of a prospectively maintained database to analyze the 3-year long-term outcomes of EUS-GBD with lumen apposing metal stents (LAMS) in high-surgical-risk patients with acute cholecystitis.Results Fifty patients with acute cholecystitis who underwent EUS-GBD with LAMS and 3-year follow-up or until death were included in this study. No endoscopic revisions were scheduled unless an adverse event (AE) or suspected LAMS dysfunction occurred. AEs occurred in 18%, 20%, and 26% of patients in the first, second, and third years, respectively. Thirteen patients developed at least one AE, and six presented with a second AE during follow-up. Recurrence of cholecystitis occurred in two patients (4%). Seven stent migrations (14%) occurred but all were asymptomatic. Symptomatic LAMS-related AEs (LAMS-RAEs) (37.5%) were related to gastric location of the stent compared with duodenal location (66.7% vs. 12.5%, P = 0.03). No stent-related bleeding or stent-related mortality was observed.Conclusions EUS-GBD with LAMS without scheduled removal is an effective and safe long-term treatment in high-surgical-risk patients with acute cholecystitis. Late LAMS-RAEs tend to be more asymptomatic over time. Symptomatic LAMS-RAEs are associated with gastric location, and overall, AEs tend to recur.
C1 [Martinez-Moreno, Belen; Lopez-Roldan, Gonzalo; Martinez-Sempere, Juan] Hosp Gen Univ Alicante, Endoscopy Unit, Alicante, Spain.
[de-Madaria, Enrique; Jover, Rodrigo; Aparicio, Jose Ramon] Hosp Gen Univ Alicante, Gastroenterol, Alicante, Spain.
C3 General University Hospital of Alicante; General University Hospital of
Alicante
RP Martinez-Moreno, B (corresponding author), Hosp Gen Univ Alicante, Endoscopy Unit, Alicante, Spain.
EM belenmm27@gmail.com
RI Jover, Rodrigo/ABC-3258-2021; de-Madaria, Enrique/F-5225-2013
OI Martinez-Moreno, Belen/0000-0002-4025-6735; de-Madaria,
Enrique/0000-0002-2412-9541; Aparicio Tormo, Jose
Ramón/0000-0003-2245-4006
CR Bang JY, 2020, GUT, V69, P1379, DOI 10.1136/gutjnl-2019-320539

Chan SM, 2017, GASTROINTEST ENDOSC, V85, P1225, DOI 10.1016/j.gie.2016.10.014
Choi JH, 2014, ENDOSCOPY, V46, P656, DOI 10.1055/s-0034-1365720
Cotton PB, 2010, GASTROINTEST ENDOSC, V71, P446, DOI 10.1016/j.gie.2009.10.027
Handler C, 2023, EUR J TRAUMA EMERG S, V49, P1157, DOI 10.1007/s00068-022-02106-7
Irani SS, 2023, ANN SURG, V278, pE556, DOI 10.1097/SLA.0000000000005784
Krishnamoorthi R, 2020, SURG ENDOSC, V34, P1904, DOI 10.1007/s00464-020-07409-7
Luk SWY, 2019, ENDOSCOPY, V51, P722, DOI 10.1055/a-0929-6603
Lyu YX, 2021, J LAPAROENDOSC ADV S, V31, P1232, DOI 10.1089/lap.2020.0786
Mohan BP, 2020, ENDOSCOPY, V52, P96, DOI 10.1055/a-1020-3932
Okamoto K, 2018, J HEPATO-BIL-PAN SCI, V25, P55, DOI 10.1002/jhbp.516
Teoh AYB, 2020, GUT, V69, P1085, DOI 10.1136/gutjnl-2019-319996
Teoh AYB, 2021, GASTROINTEST ENDOSC, V93, P577, DOI 10.1016/j.gie.2020.06.066
Yuste RT, 2019, ANN GASTROENTEROL, V32, P514, DOI 10.20524/aog.2019.0395
van der Merwe SW, 2022, ENDOSCOPY, V54, P185, DOI 10.1055/a-1717-1391
Walter D, 2016, GUT, V65, P6, DOI 10.1136/gutjnl-2015-309925
Yokoe M, 2018, J HEPATO-BIL-PAN SCI, V25, P41, DOI 10.1002/jhbp.515
NR 17
TC 20
Z9 24
U1 0
U2 1
PU GEORG THIEME VERLAG KG
PI STUTTGART
PA RUDIGERSTR 14, D-70469 STUTTGART, GERMANY
SN 2364-3722
EI 2196-9736
J9 ENDOSC INT OPEN
JI Endosc. Int. Open
PD NOV
PY 2023
VL 11
IS 11
BP E1063
EP E1068
DI 10.1055/a-2180-9817
PG 6
WC Gastroenterology & Hepatology; Surgery
WE Emerging Sources Citation Index (ESCI)
SC Gastroenterology & Hepatology; Surgery
GA Y2DA6
UT WOS:001103413100004
PM 37954111

OA Green Submitted, gold
DA 2026-05-09
ER

PT J
AU Abu Dayyeh, BK
Chandrasekhara, V
Shah, RJ
Easler, JJ
Storm, AC
Topazian, M
Levy, MJ
Martin, JA
Petersen, BT
Takahashi, N
Edmundowicz, S
Hammad, H
Wagh, MS
Wani, S
DeWitt, J
Bick, B
Gromski, M
Al Haddad, M
Sherman, S
Merchant, AA
Peetermans, JA
Gjata, O
McMullen, E
Willingham, FF
AF Abu Dayyeh, Barham K.
Chandrasekhara, Vinay
Shah, Raj J.
Easler, Jeffrey J.
Storm, Andrew C.
Topazian, Mark
Levy, Michael J.
Martin, John A.
Petersen, Bret T.
Takahashi, Naoki
Edmundowicz, Steven
Hammad, Hazem
Wagh, Mihir S.

Wani, Sachin
DeWitt, John
Bick, Benjamin
Gromski, Mark
Al Haddad, Mohammad
Sherman, Stuart
Merchant, Ambreen A.
Peetermans, Joyce A.
Gjata, Ornela
McMullen, Edmund
Willingham, Field F.
TI Combined Drainage and Protocolized Necrosectomy Through a Coaxial
Lumen-apposing Metal Stent for Pancreatic Walled-off Necrosis A
Prospective Multicenter Trial
SO ANNALS OF SURGERY
LA English
DT Article
DE drainage; metal stents; pancreatic fluid collection; pancreatitis;
plastic stents
ID INFECTED NECROTIZING PANCREATITIS; GUIDED TRANSMURAL DRAINAGE; FLUID
COLLECTIONS; UP APPROACH; MANAGEMENT; INTERVENTION; NEED
AB Objective: We evaluated a protocolized endoscopic necrosectomy approach with a lumen-apposing metal stent (LAMS) in patients with large symptomatic walled-off pancreatic necrosis (WON) comprising significant necrotic content, with or without infection.

Summary Background Data: Randomized trials have shown similar efficacy of endoscopic treatment compared with surgery for infected WON.

Design: We conducted a regulatory, prospective, multicenter single-arm clinical trial examining the efficacy and safety of endoscopic ultrasound-guided LAMS with protocolized necrosectomy to treat symptomatic WON >= 6 cm in diameter with >30% solid necrosis. After LAMS placement, protocolized WON assessment was conducted and endoscopic necrosectomy was performed for insufficient WON size reduction and persistent symptoms. Patients with radiographic WON resolution to <= 3 cm and/or 60-day LAMS indwell had LAMS removal, then 6-month follow-up. Primary endpoints were probability of radiographic resolution by 60 days and procedure-related serious adverse events.

Results: Forty consecutive patients were enrolled September 2018 to March 2020, of whom 27 (67.5%) were inpatients and 19 (47.5%) had clinical evidence of infection at their index procedure. Mean WON size was 15.0 +/- 5.6 cm with mean 53.2% +/- 16.7% solid necrosis. Radiographic WON resolution was seen in 97.5% (95% CI, 86.8%, 99.9%) by 60 days, without recurrence in 34 patients with 6-month follow-up data. Mean time to radiographic WON resolution was 34.1 +/- 16.8 days. Serious adverse events occurred in 3 patients (7.5%), including sepsis, vancomycin-resistant enterococcal bacteremia and shock, and upper gastrointestinal bleeding. There were no procedure-related deaths.

Conclusions: Endoscopic ultrasound-guided drainage with protocolized endoscopic necrosectomy to treat large symptomatic or infected walled-off necrotic pancreatic collections was highly effective and safe.
C1 [Abu Dayyeh, Barham K.; Chandrasekhara, Vinay; Storm, Andrew C.; Topazian, Mark; Levy, Michael J.; Martin, John A.; Petersen, Bret T.; Takahashi, Naoki] Mayo Clin, Div Gastroenterol & Hepatol, Rochester, MN 55905 USA.

[Shah, Raj J.; Edmundowicz, Steven; Hammad, Hazem; Wagh, Mihir S.; Wani, Sachin; DeWitt, John; Bick, Benjamin] Univ Colorado Anschutz Med Campus, Div Gastroenterol & Hepatol, Aurora, CO USA.
[Easler, Jeffrey J.; Gromski, Mark; Al Haddad, Mohammad; Sherman, Stuart] Indiana Univ Sch Med, Div Gastroenterol & Hepatol, Indianapolis, IN USA.
[Merchant, Ambreen A.; Willingham, Field F.] Emory Univ, Dept Med, Div Digest Dis, Atlanta, GA USA.
[Peetermans, Joyce A.; Gjata, Ornela; McMullen, Edmund] Boston Sci Corp, Endoscopy Div, Marl Borough, MA, Brazil.
C3 Mayo Clinic; University of Colorado System; University of Colorado
Anschutz; Indiana University System; Indiana University Bloomington;
Emory University
RP Abu Dayyeh, BK (corresponding author), Mayo Clin, Div Gastroenterol & Hepatol, Rochester, MN 55905 USA.
EM abudayyeh.barham@mayo.edu
RI ; Gromski, Mark/HLH-8123-2023
OI Takahashi, Naoki/0000-0002-7946-6078;
FU Boston Scientific Corporation; National Institute of Diabetes and
Digestive and Kidney Diseases; National Cancer Institute [U01DK108323]
Funding Source: NIH RePORTER
FX Boston Scientific Corporation.
CR Abu Dayyeh BK, 2018, GASTROINTEST ENDOSC, V87, P141, DOI 10.1016/j.gie.2017.04.032
Bakker OJ, 2012, JAMA-J AM MED ASSOC, V307, P1053, DOI 10.1001/jama.2012.276
Bang JY, 2020, DIGEST ENDOSC, V32, P298, DOI 10.1111/den.13470
Bang JY, 2019, GASTROENTEROLOGY, V156, P1027, DOI 10.1053/j.gastro.2018.11.031
Bang JY, 2019, GUT, V68, P1200, DOI 10.1136/gutjnl-2017-315335
Banks PA, 2013, GUT, V62, P102, DOI 10.1136/gutjnl-2012-302779
Bapaye A, 2017, DIGEST ENDOSC, V29, P104, DOI 10.1111/den.12704
Baron TH, 2020, GASTROENTEROLOGY, V158, P67, DOI 10.1053/j.gastro.2019.07.064
Chandrasekhara V, 2021, CLIN GASTROENTEROL H, V19, P2192, DOI 10.1016/j.cgh.2021.05.005
Cosgrove N, 2022, J CLIN GASTROENTEROL, V56, P457, DOI 10.1097/MCG.0000000000001549
Gapp J, 2019, PANCREAS, V48, P548, DOI [10.1097/MPA.0000000000001275, 10.1097/mpa.0000000000001275]
Gardner TB, 2009, GASTROINTEST ENDOSC, V69, P1085, DOI 10.1016/j.gie.2008.06.061
Garg SK, 2019, J CLIN GASTROENTEROL, V53, P328
Haney CM, 2020, SURG ENDOSC, V34, P2429, DOI 10.1007/s00464-020-07469-9
Hollemans RA, 2019, GASTROENTEROLOGY, V156, P1016, DOI 10.1053/j.gastro.2018.10.045
Lakhtakia S, 2017, GASTROINTEST ENDOSC, V85, P1243, DOI 10.1016/j.gie.2016.10.037
Law RJ, 2021, GASTROINTEST ENDOSC, V94, P457, DOI 10.1016/j.gie.2021.05.020
Manrai M, 2018, ANN SURG, V267, P357, DOI 10.1097/SLA.0000000000002065
Neermark S, 2019, PANCREATOLOGY, V19, P828, DOI 10.1016/j.pan.2019.07.042
Oblizajek N, 2020, GASTROINTEST ENDOSC, V91, P1303, DOI 10.1016/j.gie.2020.01.017
Papachristou GI, 2007, ANN SURG, V245, P943, DOI 10.1097/01.sla.0000254366.19366.69
Patra PS, 2014, BRIT J SURG, V101, P1721, DOI 10.1002/bjs.9666
Puli SR, 2014, CAN J GASTROENTEROL, V28, P50, DOI 10.1155/2014/539783
Rana SS, 2017, PANCREATOLOGY, V17, P203, DOI 10.1016/j.pan.2017.02.007

Rana SS, 2019, DIGEST LIVER DIS, V51, P730, DOI 10.1016/j.dld.2018.10.010
Schmidt PN, 2015, SCAND J GASTROENTERO, V50, P611, DOI 10.3109/00365521.2014.946078
Smoczynski M, 2015, VIDEOSURGERY MINIINV, V10, P237, DOI 10.5114/wiitm.2015.52058
Stamatakos M, 2010, WORLD J GASTROENTERO, V16, P1707, DOI 10.3748/wjg.v16.i14.1707
van Brunschot S, 2018, LANCET, V391, P51, DOI 10.1016/S0140-6736(17)32404-2
van Santvoort HC, 2010, NEW ENGL J MED, V362, P1491, DOI 10.1056/NEJMoa0908821
Varadarajulu S, 2011, AM SURGEON, V77, P1650
Varadarajulu S, 2011, GASTROINTEST ENDOSC, V74, P74, DOI 10.1016/j.gie.2011.03.1122
Wang L, 2021, ENDOSCOPY, V53, P603, DOI 10.1055/a-1213-1489
NR 33
TC 6
Z9 6
U1 0
U2 2
PU LIPPINCOTT WILLIAMS & WILKINS
PI PHILADELPHIA
PA TWO COMMERCE SQ, 2001 MARKET ST, PHILADELPHIA, PA 19103 USA
SN 0003-4932
EI 1528-1140
J9 ANN SURG
JI Ann. Surg.
PD MAY
PY 2023
VL 277
IS 5
BP E1072
EP E1080
DI 10.1097/SLA.0000000000005274
PG 9
WC Surgery
WE Science Citation Index Expanded (SCI-EXPANDED)
SC Surgery
GA G4WT4
UT WOS:000989182800035
PM 35129503
OA hybrid
DA 2026-05-09
ER

PT J
AU Dhir, V
Udawat, P

Shah, R
Alahari, A
AF Dhir, Vinay
Udawat, Priyanka
Shah, Rahul
Alahari, Aruna
TI Evaluation of an all-in-one hybrid model (EUS Magic Box) for stepwise teaching and training in multiple interventional EUS procedures
SO ENDOSCOPY INTERNATIONAL OPEN
LA English
DT Article
ID GUIDED BILIARY DRAINAGE; HANDS-ON
AB Background and study aims While multiple interventional endoscopic ultrasound (EUS)-guided procedures have evolved over the past two decades, there is no model that allows for training in all of these procedures. We aimed to develop and validate an all-in-one hybrid model for step-wise learning in multiple EUS interventions.

Methods A hybrid model was created utilizing a pig esophagus and stomach, a silicon-based duodenum and pancreato-biliary system, a pseudocyst, and biopsy targets. This model was designed to provide hands-on training in multiple interventional EUS procedures, such as EUS-guided fine-needle biopsy (EUS-FNB), biliary drainage (BD), pancreatic duct drainage (PD), pseudocyst drainage (PSD), and gastro-enterostomy (GE). Thirty-six trainees underwent training on this model over 6 days, in two batches. Lumen apposing metal stents were used for PSD and GE. Trainees were assessed for objective criteria of technical difficulties. Subjective assessment by trainees was done via a questionnaire.

Results All the trainees were able to complete the requisite steps for all the procedures under supervision. On subjective assessment, 30 trainees (83%) graded the model as good or excellent. A total of 107 technical difficulties were noted (scope position 55, duct puncture 27, guidewire-related problems 25). Time taken to complete the requisite steps of biopsy, PSD, and GE (10.5 minutes; range 3.5 to 22) was significantly less than that for BD and PD (28 minutes; range 17 to 40) ($P<0.001$).

Conclusions The hybrid model provided training for multiple EUS interventions with good acceptance by trainees. Stepwise mentoring with the possibility of performing multiple procedures in a single model with or without X-ray could prove useful in conference as well as institutional settings.
C1 [Dhir, Vinay; Udawat, Priyanka; Shah, Rahul; Alahari, Aruna] SL Raheja Hosp, Sch EUS, Inst Digest & Liver Care, Dept Gastroenterol, Mumbai, Maharashtra, India.
RP Dhir, V (corresponding author), SL Raheja Hosp, Inst Digest & Liver Care, Mumbai 400016, Maharashtra, India.
EM vinaydhir@gmail.com
CR Alcaide N, 2013, GASTROINTEST ENDOSC, V77, pAB294
Baron TH, 2015, ENDOSC ULTRASOUND, V4, P34, DOI 10.4103/2303-9027.151326
Barthet M, 2007, ENDOSCOPY, V39, P535, DOI 10.1055/s-2007-966336
Bhutani MS, 1998, ENDOSCOPY, V30, P605, DOI 10.1055/s-2007-1001364
Binmoeller KF, 2012, ENDOSC ULTRASOUND, V1, P137, DOI 10.7178/eus.03.004
Dhir V., 2017, DIGEST ENDOSC, V29, P485
Dhir V, 2017, ENDOSC INT OPEN, V5, pE1087, DOI 10.1055/s-0043-118097
Dhir V, 2015, GASTROINTEST ENDOSC, V81, P440, DOI 10.1016/j.gie.2014.09.011
FORNAGE BD, 1989, J ULTRAS MED, V8, P701

Holt BA, 2016, GASTROINTEST ENDOSC, V83, P160, DOI 10.1016/j.gie.2015.06.019
Maple JT, 2017, GASTROINTEST ENDOSC, V85, P465, DOI 10.1016/j.gie.2016.11.021
Tonozuka R, 2016, GASTROINTEST ENDOSC, V84, P206, DOI 10.1016/j.gie.2016.03.020
NR 12
TC 8
Z9 8
U1 0
U2 1
PU GEORG THIEME VERLAG KG
PI STUTTGART
PA RUDIGERSTR 14, D-70469 STUTTGART, GERMANY
SN 2364-3722
EI 2196-9736
J9 ENDOSC INT OPEN
JI Endosc. Int. Open
PD MAY
PY 2022
VL 10
IS 05
BP E634
EP E643
DI 10.1055/a-1784-3978
PG 10
WC Gastroenterology & Hepatology; Surgery
WE Emerging Sources Citation Index (ESCI)
SC Gastroenterology & Hepatology; Surgery
GA 1F2AX
UT WOS:000794976400013
PM 35571462
OA Green Submitted, gold
DA 2026-05-09
ER

PT J
AU Nieto, J
Mekaroonkamol, P
Shah, RJ
Khashab, MA
Loren, DE
Waxman, I
Edmundowicz, SA
Willingham, FF

AF Nieto, Jose
Mekaroonkamol, Parit
Shah, Raj
Khashab, Mouen A.
Loren, David E.
Waxman, Irving
Edmundowicz, Steven A.
Willingham, Field F.
TI Electrocautery-enhanced Lumen-apposing Metal Stents in the Management of Symptomatic Pancreatic Fluid Collections Results From the Multicenter Prospective Pivotal Trial
SO JOURNAL OF CLINICAL GASTROENTEROLOGY
LA English
DT Article
DE pseudocyst; cystgastrostomy; lumen-apposing stent; electrocautery enhanced
ID ULTRASOUND-GUIDED DRAINAGE; WALLED-OFF NECROSIS; ENDOSCOPIC ULTRASOUND; DELIVERY-SYSTEM; GALLBLADDER DRAINAGE; TRANSMURAL DRAINAGE; NECROSECTOMY; PSEUDOCYSTS; EFFICACY; OUTCOMES
AB Background and Aims:Endoscopic decompression is considered a first-line treatment for symptomatic pancreatic fluid collections (PFCs). A lumen-apposing metal stent (LAMS) with an electrocautery-enhanced delivery system [electrocautery-enhanced lumen-apposing metal stent (ELAMS)] may facilitate this procedure. This study evaluated the safety and efficacy of ELAMS in the management of symptomatic PFCs. Patients and Methods:A multicenter, nonblinded, prospective, Food and Drug Administration (FDA)-approved, investigational device exemption clinical trial was conducted. Enrollment criteria included symptomatic PFCs >= 6 cm with >= 70% fluid fraction. Subjects were followed prospectively for safety, efficacy, and resolution of the collections. Primary endpoint success was defined as >= 50% reduction in PFC size. Clinical outcomes were compared with our previously published series of LAMS without the cautery-enhanced delivery system. Results:The target enrollment of 30 patients was achieved in 7 US tertiary care centers. All patients underwent successful placement of the ELAMS. The mean procedure duration, stent placement time, and fluoroscopy exposures were 28.1 +/- 12.5, 5.8 +/- 2.6, and 1.8 +/- 1.6 minutes, respectively. Eight patients had no fluoroscopy. The primary endpoint was achieved in 83.3% of patients. Two adverse events were attributed to the ELAMS: 1 bleeding upon stent removal and 1 stent migration. Relative to the comparator noncautery LAMS multicenter trial (N=33, 8 tertiary centers), there was a significantly shorter procedure duration [36 min (P<0.001)] with similar technical and clinical outcomes in the ELAMS cohort. Conclusion:LAMS placed using an electrocautery delivery system significantly reduce procedure duration and were safe and effective in the management of symptomatic PFCs.
C1 [Nieto, Jose] Borland Groover Clin, Jacksonville, FL USA.
[Mekaroonkamol, Parit; Willingham, Field F.] Emory Univ, Sch Med, Atlanta, GA USA.
[Shah, Raj; Edmundowicz, Steven A.] Univ Colorado, Sch Med, Aurora, CO USA.
[Khashab, Mouen A.] Johns Hopkins Univ, Sch Med, Baltimore, MD USA.
[Loren, David E.] Thomas Jefferson Univ, Sidney Kimmel Med Coll, Philadelphia, PA USA.
[Waxman, Irving] Univ Chicago, Pritzker Sch Med, Chicago, IL USA.
[Mekaroonkamol, Parit] Chulalongkorn Univ, Fac Med, Dept Med, Div Gastroenterol, Bangkok, Thailand.

[Mekaroonkamol, Parit] King Chulalongkorn Mem Hosp, Thai Red Cross, Bangkok, Thailand.
[Willingham, Field F.] Dept Med, Div Digest Dis, 1365 Clifton Rd,NE,Bldg B Suite 1200, Atlanta, GA 30322 USA.
C3 Emory University; University of Colorado System; University of Colorado
Anschutz; Johns Hopkins University; Thomas Jefferson University;
University of Chicago; Chulalongkorn University; Thai Red Cross Society;
Chulalongkorn University
RP Willingham, FF (corresponding author), Dept Med, Div Digest Dis, 1365 Clifton Rd,NE,Bldg B Suite 1200, Atlanta, GA 30322 USA.
EM drjnieto@yahoo.com; parit_m@hotmail.com; raj.shah@ucdenver.edu;
mkhasha1@jhmi.edu; david.loren@jefferson.edu;
iwaxman@medicine.bsd.uchicago.edu; steven.edmundowicz@ucdenver.edu;
field.willingham@emory.edu
RI khashab, mouen/F-6643-2017
FU DSMC
FX ACKNOWLEDGMENTS The authors acknowledge Drs Brian Brauer, Qiang Cai,
Scott Cooper, Jeffrey Easler, Steven Keilin, Jason Korenblit, Thomas
Kowalski, Vladimir Kushnir, Daniel Mullady, Faris Murad, Ali Siddiqui,
Uzma Siddiqui, and Kevin Woods who participated as sub-investigators and
or provided assistance in subject recruitment, clinical care, or study
activities. They also acknowledge Dr Amrita Sethi, a chairperson of the
DSMC, Drs Marc Bessler and Jonathan Susman who participated as the DSMC
committee members. Many thanks to Sarah Cristofaro, MPH, for
administrative, technical, and research support. The authors
particularly thank and acknowledge Tom DeSimio, RN, who provided
superior in-person technical and clinical case support to the
investigators.
CR Akshintala VS, 2014, GASTROINTEST ENDOSC, V79, P921, DOI 10.1016/j.gie.2013.10.032
Baillie J, 2004, GASTROINTEST ENDOSC, V60, P105, DOI 10.1016/S0016-5107(04)01532-9
Bakker OJ, 2012, JAMA-J AM MED ASSOC, V307, P1053, DOI 10.1001/jama.2012.276
Bang JY, 2017, GUT, V66, P2054, DOI 10.1136/gutjnl-2016-312812
Bang JY, 2016, DIGEST ENDOSC, V28, P103, DOI 10.1111/den.12557
Banks PA, 2013, GUT, V62, P102, DOI 10.1136/gutjnl-2012-302779
Cahen D, 2005, ENDOSCOPY, V37, P977, DOI 10.1055/s-2005-870336
Chandran S, 2015, GASTROINTEST ENDOSC, V81, P127, DOI 10.1016/j.gie.2014.06.025
Chantarojanasiri T, 2015, WORLD J GASTRO ENDOS, V7, P960, DOI 10.4253/wjge.v7.i10.960
Cosgrove ND, 2018, GASTROENTEROLOGY, V154, pS940
Fabbri C, 2014, WORLD J GASTROENTERO, V20, P8424, DOI 10.3748/wjg.v20.i26.8424
Faisal MF, 2018, GASTROINTEST ENDOSC, V87, pAB430, DOI 10.1016/j.gie.2018.04.1936
Galasso D, 2015, ENDOSCOPY, V47, pE68, DOI 10.1055/s-0034-1391244
Gornals JB, 2016, SURG ENDOSC, V30, P2592, DOI 10.1007/s00464-015-4505-2
Gornals JB, 2013, SURG ENDOSC, V27, P1428, DOI 10.1007/s00464-012-2591-y
Hookey LC, 2006, GASTROINTEST ENDOSC, V63, P635, DOI 10.1016/j.gie.2005.06.028

Itoi T, 2012, GASTROINTEST ENDOSC, V75, P870, DOI 10.1016/j.gie.2011.10.020
Jacobson BC, 2005, GASTROINTEST ENDOSC, V61, P363, DOI 10.1016/S0016-5107(04)02779-8
Messallam Ahmed A, 2019, VideoGIE, V4, P353, DOI [10.1016/j.vgie.2018.10.013, 10.1016/j.vgie.2018.10.013]
Mönkemüller KE, 1998, GASTROINTEST ENDOSC, V48, P195, DOI 10.1016/S0016-5107(98)70164-6
Mukai S, 2015, ENDOSCOPY, V47, P47, DOI 10.1055/s-0034-1377966
Olaiya B, 2017, AM J GASTROENTEROL, V112, pS437, DOI 10.14309/00000434-201710001-00783
Park DH, 2009, ENDOSCOPY, V41, P842, DOI 10.1055/s-0029-1215133
Rinninella E, 2015, GASTROINTEST ENDOSC, V82, P1039, DOI 10.1016/j.gie.2015.04.006
Saxena P, 2014, GASTROINTEST ENDOSC, V80, P319, DOI 10.1016/j.gie.2014.04.041
Shah RJ, 2015, CLIN GASTROENTEROL H, V13, P747, DOI 10.1016/j.cgh.2014.09.047
Sharaiha RZ, 2016, CLIN GASTROENTEROL H, V14, P1797, DOI 10.1016/j.cgh.2016.05.011
Siddiqui AA, 2017, GASTROINTEST ENDOSC, V85, P758, DOI 10.1016/j.gie.2016.08.014
Siddiqui AA, 2016, GASTROINTEST ENDOSC, V83, P699, DOI 10.1016/j.gie.2015.10.020
Singhal S, 2013, CLIN ENDOSC, V46, P506, DOI 10.5946/ce.2013.46.5.506
Teoh AYB, 2014, GASTROINTEST ENDOSC, V80, P1171, DOI 10.1016/j.gie.2014.03.038
Varadarajulu S, 2013, GASTROENTEROLOGY, V145, P583, DOI 10.1053/j.gastro.2013.05.046
Varadarajulu S, 2009, GASTROINTEST ENDOSC, V69, pS200, DOI 10.1016/j.gie.2008.12.032
Walter D, 2015, ENDOSCOPY, V47, P63, DOI 10.1055/s-0034-1378113
Yoo J, 2017, ENDOSC ULTRASOUND, V6, P131, DOI 10.4103/2303-9027.204814
NR 35
TC 6
Z9 6
U1 0
U2 0
PU LIPPINCOTT WILLIAMS & WILKINS
PI PHILADELPHIA
PA TWO COMMERCE SQ, 2001 MARKET ST, PHILADELPHIA, PA 19103 USA
SN 0192-0790
EI 1539-2031
J9 J CLIN GASTROENTEROL
JI J. Clin. Gastroenterol.
PD FEB
PY 2023
VL 57
IS 2
BP 218
EP 226
DI 10.1097/MCG.0000000000001545
PG 9
WC Gastroenterology & Hepatology
WE Science Citation Index Expanded (SCI-EXPANDED)
SC Gastroenterology & Hepatology

GA 7N9FC
UT WOS:000907640000016
PM 33899781
DA 2026-05-09
ER

PT J
AU Hara, K
Yamao, K
Mizuno, N
Hijioka, S
Sawaki, A
Tajika, M
Kawai, H
Kondo, S
Shimizu, Y
Niwa, Y
AF Hara, Kazuo
Yamao, Kenji
Mizuno, Nobumasa
Hijioka, Susumu
Sawaki, Akira
Tajika, Masahiro
Kawai, Hiroki
Kondo, Shinya
Shimizu, Yasuhiro
Niwa, Yasumasa
TI Interventional endoscopic ultrasonography for pancreatic cancer
SO WORLD JOURNAL OF CLINICAL ONCOLOGY
LA English
DT Article
DE Endoscopic ultrasonography-biliary drainage; Endoscopic ultrasonography-choledochoduodenostomy; Endoscopic ultrasonography-fine needle aspiration; Endoscopic ultrasonography-guided biliary drainage; Interventional endoscopic ultrasonography
ID NEEDLE-ASPIRATION BIOPSY; GUIDED BILIARY DRAINAGE; CELIAC PLEXUS NEUROLYSIS; RADIOFREQUENCY ABLATION; ETHANOL INJECTION; BILE-DUCT; EUS; OBSTRUCTION; CHOLANGIOGRAPHY; FNA
AB Endoscopic ultrasonography (EUS) represents the combination of endoscopy and intraluminal ultrasonography. This allows use of a high-frequency transducer (5-20 MHz) that, due to the short distance to the target lesion, provides ultrasonographic images of higher resolution than those obtained from other imaging modalities, including multiple-detector-row-computed tomography, magnetic resonance imaging, and positron emission tomography. EUS is now a widely

accepted modality for diagnosing pancreatic diseases. However, the most important limitation of EUS has been the lack of specificity in differentiating between benign and malignant changes. In 1992, EUS-guided fine needle aspiration (FNA) of lesions in the pancreas head was introduced into clinical practice, using a curved linear-array echoendoscope. Since then, EUS has evolved from EUS imaging to EUS-FNA and wider applications. Interventional EUS for pancreatic cancer includes EUS-FNA, EUS-guided fine needle injection, EUS-guided biliary drainage and anastomosis, EUS-guided celiac neurolysis, radiofrequency ablation, brachytherapy, and delivery of a growing number of anti-tumor agents. This review focuses on interventional EUS, including EUS-FNA and therapeutic EUS for pancreatic cancer. (C) 2011 Baishideng. All rights reserved.
C1 [Hara, Kazuo; Yamao, Kenji; Mizuno, Nobumasa; Hijioka, Susumu; Sawaki, Akira; Tajika, Masahiro; Kawai, Hiroki; Kondo, Shinya; Niwa, Yasumasa] Aichi Canc Ctr Hosp, Dept Gastroenterol, Nagoya, Aichi 4648681, Japan.

[Shimizu, Yasuhiro] Aichi Canc Ctr Hosp, Dept Gastrointestinal Surg, Nagoya, Aichi 4648681, Japan.

C3 Aichi Cancer Center; Aichi Cancer Center

RP Hara, K (corresponding author), Aichi Canc Ctr Hosp, Dept Gastroenterol, Nagoya, Aichi 4648681, Japan.

EM khara@aichi-cc.jp

RI Hijioka, Susumu/I-1039-2019

OI Hijioka, Susumu/0000-0002-0394-6876

CR Al-Haddad M, 2010, J PANCREAS, V11, P1

Ang TL, 2007, J PANCREAS, V8, P438

Ashida R, 2006, ENDOSCOPY, V38, P190, DOI 10.1055/s-2005-870404

Ashida R, 2004, GATROINTEST ENDOSC, V59, pA212

Aslanian H, 2005, GASTROINTEST ENDOSC, V62, P723, DOI 10.1016/j.gie.2005.06.048

Bhutani MS, 1998, ENDOSCOPY, V30, P605, DOI 10.1055/s-2007-1001364

Bhutani MS, 1999, INTERVENTIONAL ENDOS, P65

Bories E, 2007, ENDOSCOPY, V39, P287, DOI 10.1055/s-2007-966212

Burmester E, 2003, GASTROINTEST ENDOSC, V57, P246, DOI 10.1067/mge.2003.85

Carrara S, 2008, ENDOSCOPY, V40, P759, DOI 10.1055/s-2008-1077520

Chang KJ, 2000, CANCER-AM CANCER SOC, V88, P1325, DOI 10.1002/(SICI)1097-0142(20000315)88:6<1325::AID-CNCR8>3.0.CO;2-T

CHANG KJ, 2004, GASTROINTEST ENDOSC, V59

DeWitt J, 2006, INTERVENTIONAL ENDOS, P177

Eloubeidi MA, 2006, GASTROINTEST ENDOSC, V63, P622, DOI 10.1016/j.gie.2005.05.024

Erickson RA, 2004, GASTROINTEST ENDOSC, V60, P267, DOI 10.1016/S0016-5107(04)01529-9

Giovannini M, 2001, ENDOSCOPY, V33, P898, DOI 10.1055/s-2001-17324

Goldberg SN, 1999, GASTROINTEST ENDOSC, V50, P392, DOI 10.1053/ge.1999.v50.98847

Gress F, 2001, AM J GASTROENTEROL, V96, P409

Gress FG, 2002, GASTROINTEST ENDOSC, V55, P594, DOI 10.1067/mge.2002.122580

GRIMM H, 1992, GASTROINTEST ENDOSC, V38, P170, DOI 10.1016/S0016-5107(92)70384-8

Gunaratnam NT, 2001, GASTROINTEST ENDOSC, V54, P316, DOI 10.1067/mge.2001.117515

Hara K, 2010, DIGEST ENDOSC, V22, P147, DOI 10.1111/j.1443-1661.2010.00944.x

Hecht JR, 2003, CLIN CANCER RES, V9, P555

Hirooka Y, 2003, J GASTROEN HEPATOL, V18, P1323, DOI 10.1046/j.1440-1746.2003.03040.x

Itoi T, 2010, J HEPATO-BIL-PAN SCI, V17, P611, DOI 10.1007/s00534-009-0196-1

Kahaleh M, 2006, GASTROINTEST ENDOSC, V64, P52, DOI 10.1016/j.gie.2006.01.063
Kahaleh M, 2005, GASTROINTEST ENDOSC, V61, P307, DOI 10.1016/S0016-5107(04)02585-4
Kasuya H, 2005, CANCER GENE THER, V12, P725, DOI 10.1038/sj.cgt.7700830
Kim YS, 2010, ENDOSCOPY, V42, P496, DOI 10.1055/s-0029-1244082
Kouzu T, 2006, GUIDELINES GASTROINT, P170
Lai R, 2005, ENDOSCOPY, V37, P487, DOI 10.1055/s-2005-861250
Levy MJ, 2008, AM J GASTROENTEROL, V103, P98, DOI 10.1111/j.1572-0241.2007.01607.x
Levy MJ, 2003, GASTROINTEST ENDOSC, V57, P923, DOI 10.1016/S0016-5107(03)70036-4
Maier W, 1999, J LARYNGOL OTOL, V113, P41, DOI 10.1017/S0022215100143117
Mallery S, 2004, GASTROINTEST ENDOSC, V59, P100, DOI 10.1016/S0016-5107(03)02300-9
MALLERY S, 1999, INTERVENTIONAL ENDOS, P1
Maranki J, 2009, ENDOSCOPY, V41, P532, DOI 10.1055/s-0029-1214712
Matsumoto K, 2003, GASTROINTEST ENDOSC, V57, P269, DOI 10.1067/mge.2003.79
Matsumoto K, 2008, J GASTROEN HEPATOL, V23, pE1, DOI 10.1111/j.1440-1746.2007.04918.x
Park DH, 2009, AM J GASTROENTEROL, V104, P2168, DOI 10.1038/ajg.2009.254
Sakamoto H, 2009, J GASTROEN HEPATOL, V24, P384, DOI 10.1111/j.1440-1746.2008.05636.x
Tarantino I, 2008, ENDOSCOPY, V40, P336, DOI 10.1055/s-2007-995455
Varadarajulu S, 2009, GASTROINTEST ENDOSC, V69, pS176, DOI 10.1016/j.gie.2008.12.024
VILMANN P, 1992, GASTROINTEST ENDOSC, V38, P172, DOI 10.1016/S0016-5107(92)70385-X
Wiersema MJ, 1996, GASTROINTEST ENDOSC, V44, P656, DOI 10.1016/S0016-5107(96)70047-0
Wiersema MJ, 1996, GASTROINTEST ENDOSC, V43, P102, DOI 10.1016/S0016-5107(06)80108-2
Will U, 2007, ENDOSCOPY, V39, P292, DOI 10.1055/s-2007-966215
Will U, 2007, J HEPATO-BILIARY-PAN, V14, P377, DOI 10.1007/s00534-006-1139-8
Yamao K, 2008, ENDOSCOPY, V40, P340, DOI 10.1055/s-2007-995485
Yamao K, 2005, J GASTROENTEROL, V40, P1013, DOI 10.1007/s00535-005-1717-6
Yamao K, 2002, GUIDELINES GASTROINT, P327
Yamao K, 2006, GASTROINTEST ENDOSC, V64, P663, DOI 10.1016/j.gie.2006.07.003
NR 52
TC 9
Z9 10
U1 0
U2 0
PU BAISHIDENG PUBLISHING GROUP INC
PI PLEASANTON
PA 8226 REGENCY DR, PLEASANTON, CA 94588 USA
SN 2218-4333
J9 WORLD J CLIN ONCOL
JI World J. Clin. Oncol.
PD FEB 10
PY 2011
VL 2
IS 2

BP 108
EP 114
DI 10.5306/wjco.v2.i2.108
PG 7
WC Oncology
WE Emerging Sources Citation Index (ESCI)
SC Oncology
GA VE3BP
UT WOS:000439053200004
PM 21603319
OA Green Submitted, hybrid
DA 2026-05-09
ER

PT J
AU Wang, K
Jin, Z
Du, Y
Chen, J
Zhan, X
Wang, L
Li, Z
Zou, D
Liu, Y
AF Wang, K.
Jin, Z.
Du, Y.
Chen, J.
Zhan, X.
Wang, L.
Li, Z.
Zou, D.
Liu, Y.
TI Evaluation of endoscopic-ultrasound-guided celiac ganglion irradiation
with iodine-125 seeds: a pilot study in a porcine model
SO ENDOSCOPY
LA English
DT Article
ID GAMMA-KNIFE RADIOSURGERY; TRIGEMINAL NEURALGIA; CARCINOMA; CANCER; BLOCK
AB Background and study aims: Implantation of iodine-125 (I-125) seeds under the guidance of endoscopic ultrasonography (EUS) has been proved to be a safe alternative treatment option for advanced pancreatic cancer. The main purpose of this study was to evaluate the feasibility and safety of EUS-guided radiation of the celiac ganglia with I-125

seeds in a porcine model.

Methods: Twelve domestic pigs were randomly divided into three groups. The pigs in group A had blank seeds implanted, and groups B and C had 0.4 mCi and 0.8 mCi I-125 seeds implanted, respectively. Enhanced CT scan and 3-D vascular remodeling were used to check the location of the seeds. All animals were killed and celiac ganglia were checked at 14 and at 60 days after procedure. Necrosis and apoptosis in celiac ganglia and adjacent vessels and organs were also observed.

Results: EUS-guided implantations of I-125 seeds in celiac plexus were successful at first procedure in 10 animals. The remaining two animals were implanted successfully the next day. No severe complications occurred in any animal and no damage was found in the surrounding organs. At day 60 after operation, the apoptotic index in the ganglia was 0.53 in the 0.4 mCi group and 0.94 in the 0.8 mCi group. No apoptotic cells were found in the control group implanted with blank seeds. Damage to vessels and surrounding organs by radioactive seeds was observed in only one pig at each time point.

Conclusion: EUS-guided implantation of I-125 seeds beside the celiac ganglia is a safe procedure and can induce apoptosis of local neurons. This new method may lead to an alternative treatment for pain accompanying pancreatic diseases in human.
C1 [Wang, K.; Jin, Z.; Du, Y.; Chen, J.; Zhan, X.; Wang, L.; Li, Z.; Zou, D.; Liu, Y.] Second Mil Med Univ, Changhai Hosp, Dept Gastroenterol, Shanghai 200433, Peoples R China.
C3 Naval Medical University
RP Li, Z (corresponding author), Second Mil Med Univ, Changhai Hosp, Dept Gastroenterol, 174 Changhai Rd, Shanghai 200433, Peoples R China.
EM zhsli@81890.net
OI jin, zhendong/0000-0002-9358-7351; Wang, Luowei/0000-0002-6647-786X
CR Akhan O, 2004, AM J ROENTGENOL, V182, P891, DOI 10.2214/ajr.182.4.1820891
Bajrovic A, 2004, RADIOTHER ONCOL, V71, P297, DOI 10.1016/j.radonc.2004.03.005
HAO C, 2005, CHIN J MINIM INVASIV, V10, P3
Itoh K, 2001, ACTA NEUROPATHOL, V101, P92
Jin Z, 2008, ENDOSCOPY, V40, P314, DOI 10.1055/s-2007-995476
Kondziolka D, 1998, ARCH NEUROL-CHICAGO, V55, P1524, DOI 10.1001/archneur.55.12.1524
Lin YS, 2002, CANCER, V95, P404, DOI 10.1002/cncr.10668
Liutkiene G, 2007, MEDICINA-LITHUANIA, V43, P390, DOI 10.3390/medicina43050048
NECHUSHKIN M I, 1988, Meditsinskaya Radiologiya, V33, P45
Nicol B, 2000, J NEUROSURG, V93, P152, DOI 10.3171/jns.2000.93.supplement_3.0152
Tashchereau R, 2002, MED PHYS, V29, P1397
王俊杰, 2004, [中华普通外科杂志, Chinese Journal of General Surgery], V19, P512
Wiersema MJ, 1996, GASTROINTEST ENDOSC, V44, P656, DOI 10.1016/S0016-5107(96)70047-0
XIA SX, 1998, RADIOBIOLOGY, P237
Yan BM, 2007, AM J GASTROENTEROL, V102, P430, DOI 10.1111/j.1572-0241.2006.00967.x
NR 15
TC 15
Z9 19
U1 0
U2 8
PU GEORG THIEME VERLAG KG

PI STUTTGART
PA RUDIGERSTR 14, D-70469 STUTTGART, GERMANY
SN 0013-726X
EI 1438-8812
J9 ENDOSCOPY
JI Endoscopy
PD APR
PY 2009
VL 41
IS 4
BP 346
EP 351
DI 10.1055/s-0028-1119588
PG 6
WC Gastroenterology & Hepatology; Surgery
WE Science Citation Index Expanded (SCI-EXPANDED)
SC Gastroenterology & Hepatology; Surgery
GA 438AG
UT WOS:000265527700011
PM 19340740
DA 2026-05-09
ER

PT J
AU Canakis, A
Baron, TH
AF Canakis, Andrew
Baron, Todd H.
TI Relief of biliary obstruction: choosing between endoscopic ultrasound
and endoscopic retrograde cholangiopancreatography
SO BMJ OPEN GASTROENTEROLOGY
LA English
DT Review
DE diagnostic and therapeutic endoscopy; biliary obstruction; endoscopic
retrograde pancreatography; endoscopic ultrasonography; stents
ID EUS-GUIDED CHOLEDOCHODUODENOSTOMY; SURGICALLY ALTERED ANATOMY; BILE-DUCT
STONE; EXPANDABLE METAL STENT; LONG-TERM OUTCOMES; MALIGNANT BILIARY;
PERCUTANEOUS DRAINAGE; FAILED ERCP; INTERNATIONAL MULTICENTER; ANTEGRADE
TREATMENT
AB Endoscopic ultrasound (EUS) was originally devised as a novel diagnostic technique to enable endoscopists to stage malignancies and acquire tissue. However, it rapidly advanced toward therapeutic applications and has provided gastroenterologists with the ability to effectively treat and manage advanced diseases in a minimally invasive manner. EUS-

guided biliary drainage (EUS-BD) has gained considerable attention as an approach to provide relief in malignant and benign biliary obstruction for patients when endoscopic retrograde cholangiopancreatography (ERCP) fails or is not feasible. Such instances occur in those with surgically altered anatomy, gastroduodenal obstruction, periampullary diverticulum or prior transampullary duodenal stenting. While ERCP remains the gold standard, a multitude of studies are showing that EUS-BD can be used as an alternative modality even in patients who could successfully undergo ERCP. This review will shed light on recent EUS-guided advancements and techniques in malignant and benign biliary obstruction.
C1 [Canakis, Andrew] Boston Univ, Med Ctr, Dept Med, Boston, MA USA.

[Baron, Todd H.] Univ N Carolina, Div Gastroenterol & Hepatol, Adv Therapeut Endoscopy, Chapel Hill, NC 27515 USA.

C3 Boston University; University of North Carolina; University of North Carolina Chapel Hill

RP Baron, TH (corresponding author), Univ N Carolina, Div Gastroenterol & Hepatol, Adv Therapeut Endoscopy, Chapel Hill, NC 27515 USA.

EM todd_baron@med.unc.edu

OI Canakis, Andrew/0000-0002-6646-6693
CR Amano M, 2017, J GASTROEN HEPATOL, V32, P716, DOI 10.1111/jgh.13489

Anderloni A, 2019, GASTROINTEST ENDOSC, V89, P69, DOI [10.1016/j.gie.2018.04.1746, 10.1016/j.gie.2018.08.047]

Ardengh JC, 2018, WORLD J GASTRO ENDOS, V10, P99, DOI 10.4253/wjge.v10.i5.99

Artifon ELA, 2015, GASTROINTEST ENDOSC, V81, P950, DOI 10.1016/j.gie.2014.09.047

Artifon ELA, 2011, J PANCREAS, V12, P610

Attasaranya S, 2012, GASTROENT RES PRACT, V2012, DOI 10.1155/2012/680753

Bang JY, 2018, GASTROINTEST ENDOSC, V88, P9, DOI 10.1016/j.gie.2018.03.012

Baniya R, 2017, CLIN EXP GASTROENTER, V10, P67, DOI 10.2147/CEG.S132004

Binmoeller KF, 2011, ENDOSCOPY, V43, P337, DOI 10.1055/s-0030-1256127

Cho DH, 2017, GASTROINTEST ENDOSC, V85, P1067, DOI 10.1016/j.gie.2016.09.010

Dhir V, 2015, GASTROINTEST ENDOSC, V81, P913, DOI 10.1016/j.gie.2014.09.054

Dhir V, 2014, DIGEST ENDOSC, V26, P430, DOI 10.1111/den.12153

Dumonceau JM, 2018, ENDOSCOPY, V50, P910, DOI 10.1055/a-0659-9864

Fabbri C, 2014, WORLD J GASTROENTERO, V20, P8424, DOI 10.3748/wjg.v20.i26.8424

Giovannini M, 2001, ENDOSCOPY, V33, P898, DOI 10.1055/s-2001-17324

Guo J., 2016, Discrete Dynamics in Nature and Society Volume, V2016, P1, DOI [DOI 10.1016/J.PR0CIR.2016.01.163, 10.1155/2016/1516271, DOI 10.1155/2016/1516271]

Hamada T, 2018, SCAND J GASTROENTERO, V53, P46, DOI 10.1080/00365521.2017.1382567

Hamada T, 2014, DIGEST DIS SCI, V59, P1931, DOI 10.1007/s10620-014-3062-1

Han SY, 2019, SCI REP-UK, V9, DOI 10.1038/s41598-019-52993-x

Hara K, 2016, WORLD J GASTROENTERO, V22, P1297, DOI 10.3748/wjg.v22.i3.1297

Holt BA, 2016, GASTROINTEST ENDOSC, V83, P160, DOI 10.1016/j.gie.2015.06.019

Horaguchi J, 2009, DIGEST ENDOSC, V21, P239, DOI 10.1111/j.1443-1661.2009.00899.x

Hosmer A, 2018, ENDOSC INT OPEN, V6, pE127, DOI 10.1055/s-0043-123188

Isayama H, 2013, J HEPATO-BIL-PAN SCI, V20, P413, DOI 10.1007/s00534-012-0577-8

Itoi T, 2008, WORLD J GASTROENTERO, V14, P6078, DOI 10.3748/wjg.14.6078

Itoi T, 2014, J HEPATO-BIL-PAN SCI, V21, pE86, DOI 10.1002/jhbp.165

Itoi T, 2013, J HEPATO-BIL-PAN SCI, V20, P71, DOI 10.1007/s00534-012-0569-8

Iwashita T, 2016, J HEPATO-BIL-PAN SCI, V23, P227, DOI 10.1002/jhbp.329

Iwashita T, 2014, CLIN J GASTROENTEROL, V7, P94, DOI 10.1007/s12328-014-0467-5

Iwashita T, 2013, DIGEST DIS SCI, V58, P2417, DOI 10.1007/s10620-013-2645-6

Jacques J, 2020, GASTROINTEST ENDOSC, V92, P134, DOI 10.1016/j.gie.2020.01.055

Jacques J, 2019, ENDOSCOPY, V51, P540, DOI 10.1055/a-0735-9137

Jain D, 2018, DIGESTION, V98, P1, DOI 10.1159/000487185

James TW, 2018, GASTROINTEST ENDOSC, V88, P547, DOI 10.1016/j.gie.2018.04.2353

Kahaleh M, 2006, GASTROINTEST ENDOSC, V64, P52, DOI 10.1016/j.gie.2006.01.063

Kawakami H, 2015, ENDOSCOPY, V47, pE340, DOI 10.1055/s-0034-1392507

Kawakubo K, 2016, ENDOSCOPY, V48, P164, DOI 10.1055/s-0034-1393179

Kawakubo K, 2014, J HEPATO-BIL-PAN SCI, V21, P328, DOI 10.1002/jhbp.27

Kedia P, 2014, GASTROENTEROLOGY, V147, P566, DOI 10.1053/j.gastro.2014.05.045

Khan MA, 2016, DIGEST DIS SCI, V61, P684, DOI 10.1007/s10620-015-3933-0

Khashab MA, 2016, ENDOSC INT OPEN, V4, pE487, DOI 10.1055/s-0042-102648

Khashab MA, 2016, ENDOSC INT OPEN, V4, pE175, DOI 10.1055/s-0041-109083

Khashab MA, 2015, DIGEST DIS SCI, V60, P557, DOI 10.1007/s10620-014-3300-6

Ki ELL, 2019, ENDOSC ULTRASOUND, V8, pS17, DOI 10.4103/eus.eus_50_19

Ki ELL, 2019, WORLD J GASTRO ENDOS, V11, P345, DOI 10.4253/wjge.v11.i5.345

Kim TH, 2012, WORLD J GASTROENTERO, V18, P2526, DOI 10.3748/wjg.v18.i20.2526

Krafft MR, 2019, ENDOSC INT OPEN, V7, pE1231, DOI 10.1055/a-0915-2192

Krishnamoorthi R, 2020, SURG ENDOSC, V34, P2866, DOI 10.1007/s00464-020-07484-w

Kunda R, 2016, SURG ENDOSC, V30, P5002, DOI 10.1007/s00464-016-4845-6

Lee TH, 2016, CLIN GASTROENTEROL H, V14, P1011, DOI 10.1016/j.cgh.2015.12.032

Li DF, 2019, REV ESP ENFERM DIG, V111, P953, DOI 10.17235/reed.2019.6125/2018

Logiudice FP, 2019, WORLD J GASTRO ENDOS, V11, P281, DOI 10.4253/wjge.v11.i4.281

Lu LL, 2017, COMPLEXITY, DOI 10.1155/2017/7628537

Miller CS, 2019, ENDOSC INT OPEN, V7, pE1563, DOI 10.1055/a-0998-8129

Mine T, 2017, J GASTROENTEROL, V52, P1013, DOI 10.1007/s00535-017-1359-5

Miranda-García P, 2016, ENDOSC INT OPEN, V4, pE461, DOI 10.1055/s-0042-103241

Moole H, 2017, MEDICINE, V96, DOI 10.1097/MD.0000000000005154

Mukai S, 2019, GASTROINTEST ENDOSC, V89, P399, DOI 10.1016/j.gie.2018.07.030

Mukai S, 2017, J HEPATO-BIL-PAN SCI, V24, P537, DOI 10.1002/jhbp.496

Nakai Y, 2019, CLIN ENDOSC, V52, P212, DOI 10.5946/ce.2018.188

Nakai Y, 2019, ENDOSC ULTRASOUND, V8, P111, DOI 10.4103/eus.eus_17_18

Nakai Y, 2018, DIGEST ENDOSC, V30, P125, DOI 10.1111/den.12965

Nam K, 2018, ENDOSC ULTRASOUND, V7, P48, DOI 10.4103/eus.eus_100_17

Nguyen-Tang T, 2010, ENDOSCOPY, V42, P232, DOI 10.1055/s-0029-1243858

Ogura T, 2019, ENDOSC ULTRASOUND, V8, P398, DOI 10.4103/eus.eus_45_19

Ogura T, 2016, WORLD J GASTROENTERO, V22, P3945, DOI 10.3748/wjg.v22.i15.3945

Ogura T, 2016, ENDOSCOPY, V48, P156, DOI 10.1055/s-0034-1392859

Oh D, 2017, THER ADV GASTROENTER, V10, P42, DOI 10.1177/1756283X16671671
Okuno N, 2018, J GASTROEN HEPATOL, V33, P1413, DOI 10.1111/jgh.14112
Okuno N, 2017, INTERNAL MED, V56, P3135, DOI 10.2169/internalmedicine.8677-16
Paik WH, 2018, AM J GASTROENTEROL, V113, P987, DOI 10.1038/s41395-018-0122-8
Park DH, 2015, J GASTROEN HEPATOL, V30, P1461, DOI 10.1111/jgh.13027
Park DH, 2013, GASTROINTEST ENDOSC, V78, P91, DOI 10.1016/j.gie.2013.01.042
Park DH, 2012, GASTROINTEST ENDOSC, V75, P692, DOI 10.1016/j.gie.2011.04.013
Park DH, 2011, GASTROINTEST ENDOSC, V74, P1276, DOI 10.1016/j.gie.2011.07.054
Park DH, 2009, AM J GASTROENTEROL, V104, P2168, DOI 10.1038/ajg.2009.254
Park JK, 2018, GASTROINTEST ENDOSC, V88, P277, DOI 10.1016/j.gie.2018.03.015
Pizzicannella M, 2020, ENDOSC ULTRASOUND, V9, P45, DOI 10.4103/eus.eus_55_19
Poincloux L, 2015, ENDOSCOPY, V47, P794, DOI [10.1055/s-0035-1545399, 10.1055/s-0034-1391988]
Prachayakul V, 2015, WORLD J GASTRO ENDOS, V7, P37, DOI 10.4253/wjge.v7.i1.37
Prachayakul V, 2013, WORLD J GASTROENTERO, V19, P4758, DOI 10.3748/wjg.v19.i29.4758
Saad WEA, 2010, J VASC INTERV RADIOL, V21, P789, DOI 10.1016/j.jvir.2010.01.012
Saleem A, 2010, ENDOSCOPY, V42, P656, DOI 10.1055/s-0030-1255557
Shah JN, 2012, GASTROINTEST ENDOSC, V75, P56, DOI 10.1016/j.gie.2011.08.032
Shah SL, 2019, GASTROINTEST ENDOSC, V89, pAB606, DOI 10.1016/j.gie.2019.03.1049
Shah SL, 2018, J CLIN GASTROENTEROL, V52, P765, DOI 10.1097/MCG.0000000000001104
Sharaiha RZ, 2017, GASTROINTEST ENDOSC, V85, P904, DOI 10.1016/j.gie.2016.12.023
Song TJ, 2014, GASTROINTEST ENDOSC, V80, P707, DOI 10.1016/j.gie.2014.05.327
Tarantino I, 2012, WORLD J GASTRO ENDOS, V4, P306, DOI 10.4253/wjge.v4.i7.306
Teoh AYB, 2018, GUT, V67, P1209, DOI 10.1136/gutjnl-2017-314341
Tonozuka R, 2013, DIGEST ENDOSC, V25, P100, DOI 10.1111/den.12063
Tsuchiya T, 2018, GASTROINTEST ENDOSC, V87, P1138, DOI 10.1016/j.gie.2017.08.017
Uemura RS, 2018, J CLIN GASTROENTEROL, V52, P123, DOI 10.1097/MCG.0000000000000948
Wang GX, 2019, J TRANSL INTERN MED, V7, P93, DOI 10.2478/jtim-2019-0021
Wang KX, 2016, GASTROINTEST ENDOSC, V83, P1218, DOI 10.1016/j.gie.2015.10.033
Yamamoto K, 2018, ENDOSC ULTRASOUND, V7, P404, DOI 10.4103/eus.eus_51_18
Yamao K, 2018, GASTROINTEST ENDOSC, V88, P66, DOI 10.1016/j.gie.2018.01.021
NR 97
TC 27
Z9 33
U1 0
U2 5
PU BMJ PUBLISHING GROUP
PI LONDON
PA BRITISH MED ASSOC HOUSE, TAVISTOCK SQUARE, LONDON WC1H 9JR, ENGLAND
SN 2054-4774
J9 BMJ OPEN GASTROENTER
JI BMJ Open Gastroenterol.
PY 2020

VL 7
IS 1
AR e000428
DI 10.1136/bmjgast-2020-000428
PG 9
WC Gastroenterology & Hepatology
WE Emerging Sources Citation Index (ESCI)
SC Gastroenterology & Hepatology
GA ND2KV
UT WOS:000561735000001
PM 32727716
OA Green Submitted, gold
DA 2026-05-09
ER

PT J
AU Ferreira, FG
Ribeiro, MA
Abreu, P
Ferreira, R
Assef, MS
Park, JH
Szutan, LA
AF Ferreira, Fabio Goncalves
Ribeiro, Mauricio Alves
Abreu, Phillipe
Ferreira, Raphaella
Assef, Mauricio Saab
Park, Jong Hun
Szutan, Luiz Arnaldo
TI Endoscopic Ultrasound-Guided Ethanol Injection Associated with
Trans-arterial Embolization of a Giant Intra-abdominal Cavernous
Hemangioma: Case Report and New Therapeutic Option
SO JOURNAL OF GASTROINTESTINAL CANCER
LA English
DT Article
DE Cavernous hemangioma; Hematemesis; Endoscopic ultrasound; Angiography;
Malnutrition; Absolute Alcohol; Stomach
ID MALFORMATIONS
AB Background Cavernous hemangiomas are congenital hamartomatous lesions that originate from mesodermal tissue composed of dilated blood vessels. Abdominal pain and palpable mass are the most common presenting symptoms. The different types of treatment for symptomatic patients remain controversial. However, surgical resection is always the most

preferred method when possible. To date, there are no reports of endoscopic ultrasound-guided (EUS-guided) absolute ethanol injection as a treatment for such disease when surgery is not an option.

Case Presentation A 19-year-old girl with giant cavernous intra-abdominal hemangioma extending to the hepatic hilum, also affecting the gastric wall and occupying the entire supra-mesocolic cavity, initially presents with upper gastrointestinal bleeding and loss of 20 kg in 1 year (BMI = 18 kg/m(2)). Percutaneous angiography identified a mass with arterial blood supply by the left gastric artery that was embolized. After re-bleeding, an alternative treatment with EUS-guided injection of alcohol was proposed once resection was not feasible without major risks to a young patient. This procedure was repeated 15 and 45 days after the initial treatment, with the ethanol injection of 25 cc and 15 cc, respectively. On the second and third procedure dates, there was evident regression of the hemangioma. On the third procedure, it was possible to identify all anatomic structures that were not clear on the first EUS. After 45 days of last injection, abdominal CT and EUS showed notorious regression of the lesion. Eight months later, abdominal CT showed only a remnant lesion in the hepatogastric ligament with 129 cm(3) on volumetry (87% lower in comparison to the initial image), and the patient remains asymptomatic with BMI of 26. In the most recent follow-up CT, 4 years and 2 months after first treatment, the patient presents with a slight increase in the hemangioma-now with 183 cm(3) on volumetry.

Conclusion Transgastric EUS-guided ethanol injection in the treatment of giant intra-abdominal cavernous hemangioma can provide good outcomes without major complications and can be repeated if necessary.

C1 [Ferreira, Fabio Goncalves; Ribeiro, Mauricio Alves; Abreu, Phillipe; Ferreira, Raphaella; Szutan, Luiz Arnaldo] Santa Casa Sao Paulo Sch Med Sci, Div Liver Surg & Portal Hypertens, Dept Surg, Sao Paulo, Brazil.

[Abreu, Phillipe] Univ Miami, Miami Transplant Inst, Jackson Mem Hosp, Dept Surg, 1801 NW 9th Ave,7th Floor, Miami, FL 33136 USA.

[Assef, Mauricio Saab] Santa Casa Sao Paulo Sch Med Sci, Div Endoscopy Surg, Dept Surg, Sao Paulo, Brazil.

[Park, Jong Hun] Santa Casa Sao Paulo Sch Med Sci, Div Endovasc Surg, Dept Surg, Sao Paulo, Brazil.

C3 University of Miami

RP Abreu, P (corresponding author), Santa Casa Sao Paulo Sch Med Sci, Div Liver Surg & Portal Hypertens, Dept Surg, Sao Paulo, Brazil.

EM Dr.PhillipeAbreu@gmail.com

RI Ribeiro, Mauricio/I-1465-2012; Abreu, Phillipe/U-2719-2019; Ferreira, Fabio/AAX-6834-2020

OI Ferreira, Raphaella/0000-0003-4990-4839; Abreu, Phillipe/0000-0001-6340-7738; Ferreira, Fabio/0000-0001-8714-2615

CR Bamanikar A A, 2004, Indian J Gastroenterol, V23, P113

Choi YH, 2002, J VASC INTERV RADIOL, V13, P475

Chung JJ, 2000, ABDOM IMAGING, V25, P542, DOI 10.1007/s002610000087

Fabbri C, 2014, WORLD J GASTROENTERO, V20, P8424, DOI 10.3748/wjg.v20.i26.8424

Gitelman J M, 1976, N Y State Dent J, V42, P420

ISO Y, 1988, ENDOSCOPY, V20, P285, DOI 10.1055/s-2007-1018198

Kinoshita T, 1997, AM J ROENTGENOL, V169, P600, DOI 10.2214/ajr.169.2.9242791

MULLIKEN JB, 1982, PLAST RECONSTR SURG, V69, P412, DOI 10.1097/00006534-198203000-00002

Robinson M, 2007, CARDIOVASC INTER RAD, V30, P515, DOI 10.1007/s00270-006-0081-0

Romero-Castro R, 2007, GASTROINTEST ENDOSC, V66, P402, DOI 10.1016/j.gie.2007.03.008

Schettini ST, 2007, J PEDIATR SURG, V42, P717, DOI 10.1016/j.jpedsurg.2006.12.044

Seo HI, 2009, WORLD J GASTROENTERO, V15, P3437, DOI 10.3748/wjg.15.3437

Sreenarasimhaiah J, 2009, AM J MED SCI, V338, P319, DOI 10.1097/MAJ.0b013e3181a44405
Takamura M, 2000, Radiat Med, V18, P67
Zhou JX, 2013, WORLD J GASTROENTERO, V19, P2974, DOI 10.3748/wjg.v19.i19.2974
NR 15
TC 3
Z9 4
U1 0
U2 0
PU SPRINGER
PI NEW YORK
PA ONE NEW YORK PLAZA, SUITE 4600, NEW YORK, NY, UNITED STATES
SN 1941-6628
EI 1941-6636
J9 J GASTROINTEST CANC
JI J. Gastrointest. Cancer
PD MAR
PY 2021
VL 52
IS 1
BP 381
EP 385
DI 10.1007/s12029-020-00568-9
EA JAN 2021
PG 5
WC Oncology; Gastroenterology & Hepatology
WE Emerging Sources Citation Index (ESCI)
SC Oncology; Gastroenterology & Hepatology
GA QK8ND
UT WOS:000605900900001
PM 33411258
DA 2026-05-09
ER

PT J
AU Lyu, YX
Li, T
Wang, B
Cheng, YX
Chen, L
Zhao, SC
AF Lyu, Yunxiao
Li, Ting

Wang, Bin
Cheng, Yunxiao
Chen, Liang
Zhao, Sicong
TI Endoscopic Ultrasound-Guided Gallbladder Drainage Versus Percutaneous
Transhepatic Gallbladder Drainage for Acute Cholecystitis with High
Surgical Risk: An Up-to-Date Meta-Analysis and Systematic Review
SO JOURNAL OF LAPAROENDOSCOPIC & ADVANCED SURGICAL TECHNIQUES
LA English
DT Review
DE acute cholecystitis; drainage; endosonography; gallbladder; systematic
review
ID METAL STENT; MANAGEMENT; CHOLECYSTOSTOMY; TIME
AB Background: To compare the safety and effectiveness of endoscopic ultrasound-guided gallbladder drainage (EUSGBD) with percutaneous transhepatic gallbladder drainage (PTGBD) for acute cholecystitis with high surgical risk.

Methods: An electronic search was performed of the major databases, namely PubMed, Embase, Web of Science, the Cochrane Central Register of Controlled Trials, and ClinicalTrials.gov until July 1, 2020. Studies comparing EUSGBD with PTGBD were included.

Results: We identified 8 studies involving 801 patients, and patients were divided into two groups (EUSGBD group = 338 and PTGBD = 463). EUSGBD was associated with less reintervention (odds ratio [OR] = 0.15; 95% confidence interval [CI]: 0.07-0.32; P < .00001) and readmission (OR = 0.24; 95% CI: 0.08-0.67; P = 7). With lumen-apposing metal stents (LAMS), EUSGBD was associated with fewer adverse events (OR = 0.35; 95% CI: 0.13-0.93; P = .03), recurrent cholecystitis (OR = 0.27; 95% CI: 0.10-0.71; P = .008) and readmission (OR = 0.10; 95% CI: 0.03-0.32; P = .0001). There were no significant differences between the groups regarding clinical success (OR = 1.47; 95% CI: 0.75-2.90; P = .26). Technical success with PTGBD was higher than that with EUSGBD (OR = 0.32; 95% CI: 0.13-0.83; P = .02).

Conclusions: EUSGBD was comparable with PTGBD regarding clinical success, with less reintervention and readmission, for acute cholecystitis with high surgical risk. The cholecystitis recurrence rate was lower with EUSGBD with LAMS.
C1 [Lyu, Yunxiao; Wang, Bin; Cheng, Yunxiao; Chen, Liang; Zhao, Sicong] Wenzhou Med Univ, Dept Hepatobiliary Surg, Affiliated Dongyang Hosp, 60 West Wuning Rd, Dongyang 322100, Zhejiang, Peoples R China.
[Li, Ting] Wenzhou Med Univ, Dept Personnel Off, Affiliated Dongyang Hosp, Dongyang, Peoples R China.
C3 Wenzhou Medical University; Wenzhou Medical University
RP Lyu, YX (corresponding author), Wenzhou Med Univ, Dept Hepatobiliary Surg, Affiliated Dongyang Hosp, 60 West Wuning Rd, Dongyang 322100, Zhejiang, Peoples R China.
EM lvyunxiao1986@gmail.com
CR Ahmed O, 2018, SURG ENDOSC, V32, P1627, DOI 10.1007/s00464-018-6041-3
Anderloni A, 2016, SURG ENDOSC, V30, P5200, DOI 10.1007/s00464-016-4894-x
Baron TH, 2007, GASTROINTEST ENDOSC, V65, P735, DOI 10.1016/j.gie.2006.07.041
Chen YI, 2018, GASTROINTEST ENDOSC, V88, P267, DOI 10.1016/j.gie.2018.03.021
Irani S, 2017, CLIN GASTROENTEROL H, V15, P738, DOI 10.1016/j.cgh.2016.12.021
Itoi T, 2008, GASTROINTEST ENDOSC, V68, P455, DOI 10.1016/j.gie.2008.02.052
Jadad AR, 1996, CONTROL CLIN TRIALS, V17, P1, DOI 10.1016/0197-2456(95)00134-4

Jang JW, 2012, GASTROENTEROLOGY, V142, P805, DOI 10.1053/j.gastro.2011.12.051
Khan MA, 2017, GASTROINTEST ENDOSC, V85, P76, DOI 10.1016/j.gie.2016.06.032
Kiewiet JJS, 2012, RADIOLOGY, V264, P708, DOI 10.1148/radiol.12111561
Kozakai F, 2018, GASTROINTEST ENDOSC, V87, pAB333, DOI 10.1016/j.gie.2018.04.1715
Kunda R, 2016, SURG ENDOSC, V30, P5002, DOI 10.1007/s00464-016-4845-6
Lee SS, 2007, GASTROINTEST ENDOSC, V66, P1008, DOI 10.1016/j.gie.2007.03.1080
Loozen CS, 2017, SURG ENDOSC, V31, P504, DOI 10.1007/s00464-016-5011-x
Mohan BP, 2020, ENDOSCOPY, V52, P96, DOI 10.1055/a-1020-3932
Mori Y, 2018, J HEPATO-BIL-PAN SCI, V25, P87, DOI 10.1002/jhbp.504
Okamoto K, 2018, J HEPATO-BIL-PAN SCI, V25, P55, DOI 10.1002/jhbp.516
Pucher PH, 2014, BRIT J SURG, V101, P1499, DOI 10.1002/bjs.9615
Rerknimitr R, 2020, CLIN ENDOSC, V53, P678, DOI 10.5946/ce.2019.186
Saumoy M, 2019, J CLIN GASTROENTEROL, V53, P231, DOI 10.1097/MCG.0000000000001036
Siddiqui A, 2019, SURG ENDOSC, V33, P1260, DOI 10.1007/s00464-018-6406-7
Teoh AY, 2019, ENDOSC INT OPEN, V7, pE964, DOI 10.1055/a-0915-2098
Teoh AYB, 2020, GUT, V69, P1085, DOI 10.1136/gutjnl-2019-319996
Teoh AYB, 2017, ENDOSCOPY, V49, P130, DOI 10.1055/s-0042-119036
Tyberg A, 2018, J CLIN GASTROENTEROL, V52, P79, DOI 10.1097/MCG.0000000000000786
Wells GA., 2015, The Newcastle- Ottawa Scale (NOS) for assessing the quality if nonrandomized studies in metaanalyses
Yokoe M, 2018, J HEPATO-BIL-PAN SCI, V25, P41, DOI 10.1002/jhbp.515
Zafar SN, 2015, JAMA SURG, V150, P129, DOI 10.1001/jamasurg.2014.2339
NR 28
TC 14
Z9 14
U1 0
U2 4
PU MARY ANN LIEBERT, INC
PI NEW ROCHELLE
PA 140 HUGUENOT STREET, 3RD FL, NEW ROCHELLE, NY 10801 USA
SN 1092-6429
EI 1557-9034
J9 J LAPAROENDOSC ADV S
JI J. Laparoendosc. Adv. Surg. Tech.
PD NOV
PY 2021
VL 31
IS 11
BP 1232
EP 1240
DI 10.1089/lap.2020.0786
EA JAN 2021

PG 9
WC Surgery
WE Science Citation Index Expanded (SCI-EXPANDED)
SC Surgery
GA YY9PT
UT WOS:000606513000001
PM 33400595
DA 2026-05-09
ER

PT J
AU Fujita, N
Sugawara, T
Noda, Y
Kobayashi, G
Ito, K
Obana, T
Horaguchi, J
Takasawa, O
AF Fujita, Naotaka
Sugawara, Toshiki
Noda, Yutaka
Kobayashi, Go
Ito, Kei
Obana, Takashi
Horaguchi, Jun
Takasawa, Osamu
TI SNARE-OVER-THE-WIRE TECHNIQUE FOR SAFE EXCHANGE OF A STENT FOLLOWING ENDOSONOGRAPHY-GUIDED BILIARY DRAINAGE
SO DIGESTIVE ENDOSCOPY
LA English
DT Article
DE biliary stenting; endoscopic biliary drainage; endosonography; interventional endoscopic ultrasound; obstructive jaundice
ID PANCREATIC-DUCTS; OBSTRUCTION; MANAGEMENT; THERAPY
AB Endosonography-guided biliary drainage (ESBD) is a new option that allows establishment of biliary drainage. Due to the diameter of the working channel of an echoendoscope, it is necessary to replace a small caliber stent with a larger one to lessen the risk of stent occlusion. However, insertion of a guidewire into the bile duct via the hole of the sinus tract following direct removal of a previously placed stent is not always possible, resulting in guidewire passage outside the fistula and bile leakage. Cannulation of the previously deployed stent, guidewire insertion into the bile duct via the cannula and the stent, and removal of the stent with the snare over the guidewire leaving the guidewire in place (the snare-over-the-wire technique [SOW]) for stent exchange following ESBD was attempted. Four patients who required stent exchange

following ESBD were included in the present study to evaluate the feasibility and usefulness of SOW. SOW was successful in all the cases. A new stent was also successfully deployed over the guidewire in all the cases. No complications were encountered. The snare-over-the-wire technique is feasible and useful in stent exchange following ESBD for the reduction of the risk of guidewire migration.
C1 [Fujita, Naotaka] Sendai City Med Ctr, Dept Gastroenterol, Miyagino Ku, Sendai, Miyagi 9830824, Japan.
RP Fujita, N (corresponding author), Sendai City Med Ctr, Dept Gastroenterol, Miyagino Ku, 5-22-1 Tsurugaya, Sendai, Miyagi 9830824, Japan.
EM naotaka.fujita@japan.email.ne.jp
CR BOHORFOUSH AG, 1993, GASTROINTEST ENDOSC, V39, P799, DOI 10.1016/S0016-5107(93)70268-0
Burmester E, 2003, GASTROINTEST ENDOSC, V57, P246, DOI 10.1067/mge.2003.85
Fujita N, 1998, GASTROINTEST ENDOSC, V48, P517, DOI 10.1016/S0016-5107(98)70096-3
Fujita N, 2008, DIGEST ENDOSC, V20, P55, DOI 10.1111/j.1443-1661.2008.00782.x
Fujita N, 2007, WORLD J GASTROENTERO, V13, P5512, DOI 10.3748/wjg.v13.i41.5512
Giovannini M, 2001, ENDOSCOPY, V33, P898, DOI 10.1055/s-2001-17324
Kahaleh M, 2006, GASTROINTEST ENDOSC, V64, P52, DOI 10.1016/j.gie.2006.01.063
Mallery S, 2004, GASTROINTEST ENDOSC, V59, P100, DOI 10.1016/S0016-5107(03)02300-9
Noon WJ, 2006, GASTROINTEST ENDOSC, V63, P996, DOI 10.1016/j.gie.2005.11.054
Park DH, 2006, CLIN GASTROENTEROL H, V4, P790, DOI 10.1016/j.cgh.2006.03.032
PESSA ME, 1987, ANN SURG, V205, P389, DOI 10.1097/00000658-198704000-00008
Püspök A, 2005, AM J GASTROENTEROL, V100, P1743, DOI 10.1111/j.1572-0241.2005.41806.x
SHERMAN S, 1993, GASTROINTEST ENDOSC, V39, P794, DOI 10.1016/S0016-5107(93)70267-9
TAKADA T, 1976, Journal of Surgical Oncology, V8, P83, DOI 10.1002/jso.2930080113
TAKADA T, 1995, HEPATO-GASTROENTEROL, V42, P317
Yamao K, 2006, GASTROINTEST ENDOSC, V64, P663, DOI 10.1016/j.gie.2006.07.003
NR 16
TC 18
Z9 18
U1 0
U2 0
PU WILEY-BLACKWELL PUBLISHING, INC
PI MALDEN
PA COMMERCE PLACE, 350 MAIN ST, MALDEN 02148, MA USA
SN 0915-5635
J9 DIGEST ENDOSC
JI Dig. Endosc.
PD JAN
PY 2009
VL 21
IS 1
BP 48
EP 52
DI 10.1111/j.1443-1661.2008.00821.x

PG 5
WC Gastroenterology & Hepatology; Surgery
WE Science Citation Index Expanded (SCI-EXPANDED)
SC Gastroenterology & Hepatology; Surgery
GA 387OZ
UT WOS:000261962800011
PM 19691803
DA 2026-05-09
ER

PT J
AU Mukai, S
Itoi, T
AF Mukai, Shuntaro
Itoi, Takao
TI Preoperative endoscopic ultrasound-guided biliary drainage for primary drainage in obstructive jaundice
SO EXPERT REVIEW OF GASTROENTEROLOGY & HEPATOLOGY
LA English
DT Review
DE Preoperative biliary drainage; endoscopic transpapillary biliary drainage; ERCP; EUS-guided biliary drainage; EUS-guided rendezvous technique; EUS-guided choledochoduodenostomy; EUS-guided hepaticogastrostomy; EUS-guided antegrade stenting
ID APPOSING METAL STENT; LONG-TERM OUTCOMES; MALIGNANT BILIARY; BILE-DUCT; BALLOON ENTEROSCOPY; PLASTIC STENT; CHOLEDOCHODUODENOSTOMY; HEPATICOGASTROSTOMY; MULTICENTER; CHOLANGIOPANCREATOGRAPHY
AB IntroductionEndoscopic transpapillary approach by endoscopic retrograde cholangiopancreatography (ERCP) is the established technique for preoperative biliary drainage (PBD). Recently, endoscopic ultrasound-guided biliary drainage (EUS-BD) has been reported to be a useful alternative technique after ERCP fail. However, the optimal strategy remain controversial.Area coveredThis review summarizes the literature on EUS-BD techniques for PBD with a literature search using PubMed, Web of Science, and the Cochrane Central Register of Controlled Trials database between 2000 and 2023 using keywords for 'preoperative biliary drainage' and all types of EUS-BD techniques.Expert opinionAs there is no consensus on the optimal EUS-BD technique for PBD, selection of the EUS-BD approach depends on the patient's condition, the biliary obstruction site, the anastomosis after surgical intervention, and the preference of the endoscopist. However, we consider that EUS-HGS using a dedicated plastic stent may have some advantages in the adverse impact of surgical procedure because the location where the fistula is created by EUS-HGS is away from the site of the surgical procedure. Although there remain many issues that require further investigation, EUS-BD can be a feasible and safe alternative method of PBD for malignant biliary obstruction after ERCP fail.
C1 [Mukai, Shuntaro; Itoi, Takao] Tokyo Med Univ, Dept Gastroenterol & Hepatol, 6-7-1 Nishishinjuku,Shinjuku Ku, Tokyo 1600023, Japan.
C3 Tokyo Medical University

RP Itoi, T (corresponding author), Tokyo Med Univ, Dept Gastroenterol & Hepatol, 6-7-1 Nishishinjuku,Shinjuku Ku, Tokyo 1600023, Japan.
EM itoi@tokyo-med.ac.jp
RI Mukai, Shuntaro/AAD-4278-2020
CR Aabakken L, 2007, ENDOSCOPY, V39, P1068, DOI 10.1055/s-2007-966841
Akashi M, 2020, J HEPATO-BIL-PAN SCI, V27, P640, DOI 10.1002/jhbp.783
Bang JY, 2018, GASTROINTEST ENDOSC, V88, P9, DOI 10.1016/j.gie.2018.03.012
Burmester E, 2003, GASTROINTEST ENDOSC, V57, P246, DOI 10.1067/mge.2003.85
COTTON PB, 1972, GUT, V13, P851
Dhir V, 2012, GASTROINTEST ENDOSC, V75, P354, DOI 10.1016/j.gie.2011.07.075
Dumonceau JM, 2018, ENDOSCOPY, V50, P910, DOI 10.1055/a-0659-9864
Fabbri C, 2019, J GASTROINTEST LIVER, V28, P125, DOI 10.15403/jgld.2014.1121.281.eus
Garcea G, 2010, PANCREAS, V39, P119, DOI 10.1097/MPA.0b013e3181bd65de
Gaujoux S, 2021, HPB, V23, P154, DOI 10.1016/j.hpb.2020.06.001
Giovannini M, 2001, ENDOSCOPY, V33, P898, DOI 10.1055/s-2001-17324
Giri S, 2023, GASTROINTEST ENDOSC, V98, DOI 10.1016/j.gie.2023.06.055
GLENN F, 1962, ANN SURG, V156, P451, DOI 10.1097/00000658-196209000-00012
Glessing BR, 2015, GASTROINTEST ENDOSC, V81, P1019, DOI 10.1016/j.gie.2014.09.061
Gupta K, 2014, J CLIN GASTROENTEROL, V48, P80, DOI 10.1097/MCG.0b013e31828c6822
Holt BA, 2016, GASTROINTEST ENDOSC, V83, P160, DOI 10.1016/j.gie.2015.06.019
Imai H, 2017, ONCOLOGY-BASEL, V93, P69, DOI 10.1159/000481233
Inoue T, 2021, ENDOSC INT OPEN, V09, pE417, DOI 10.1055/a-1326-1611
Itoi T, 2008, WORLD J GASTROENTERO, V14, P6078, DOI 10.3748/wjg.14.6078
Itoi T, 2013, DIGEST ENDOSC, V25, P137, DOI 10.1111/den.12084
Itoi T, 2011, J HEPATO-BIL-PAN SCI, V18, P664, DOI 10.1007/s00534-011-0410-9
Itoi T, 2010, AM J GASTROENTEROL, V105, P93, DOI 10.1038/ajg.2009.559
Iwashita T, 2023, DIGEST ENDOSC, V35, P264, DOI 10.1111/den.14393
Iwashita T, 2014, CLIN J GASTROENTEROL, V7, P94, DOI 10.1007/s12328-014-0467-5
Jacques J, 2019, ENDOSCOPY, V51, P540, DOI 10.1055/a-0735-9137
Jinkins LJ, 2013, SURGERY, V154, P179, DOI 10.1016/j.surg.2013.03.016
Kakked G, 2020, ENDOSC ULTRASOUND, V9, P298, DOI 10.4103/eus.eus_10_20
Kato H, 2022, DEN OPEN, V2, DOI 10.1002/deo2.33
Kawakubo K, 2014, J HEPATO-BIL-PAN SCI, V21, P328, DOI 10.1002/jhbp.27
Kawashima H, 2013, ANN SURG, V257, P121, DOI 10.1097/SLA.0b013e318262b2e9
Khan MA, 2016, DIGEST DIS SCI, V61, P684, DOI 10.1007/s10620-015-3933-0
Khashab MA, 2016, ENDOSC INT OPEN, V4, pE175, DOI 10.1055/s-0041-109083
Kloek JJ, 2010, J CLIN GASTROENTEROL, V44, P646, DOI 10.1097/MCG.0b013e3181ce5b36
Kongkam P, 2021, ENDOSCOPY, V53, P55, DOI 10.1055/a-1195-8197
Koutlas NJ, 2022, CUREUS J MED SCIENCE, V14, DOI 10.7759/cureus.23209
Ma KW, 2020, J GASTROEN HEPATOL, V35, P1753, DOI 10.1111/jgh.15089
Mallery S, 2004, GASTROINTEST ENDOSC, V59, P100, DOI 10.1016/S0016-5107(03)02300-9
Motoi F, 2019, JPN J CLIN ONCOL, V49, P190, DOI 10.1093/jjco/hyy190

Mukai S, 2023, J HEPATO-BIL-PAN SCI, V30, P983, DOI 10.1002/jhbp.1292
Mukai S, 2017, J HEPATO-BIL-PAN SCI, V24, P537, DOI 10.1002/jhbp.496
Mukai S, 2017, GASTROINTEST ENDOSC, V86, P203, DOI [10.1016/j.gie.2016.07.056, 10.1016/j.gie.2016.11.025, 10.1016/j.gie.2016.07.056]
Mukai S, 2016, EXPERT REV GASTROENT, V10, P709, DOI 10.1586/17474124.2016.1143774
Nagakawa Y, 2019, ANN SURG ONCOL, V26, P1629, DOI 10.1245/s10434-018-07131-8
Nagakawa Y, 2013, PANCREAS, V42, P701, DOI 10.1097/MPA.0b013e31826d3a41
Nakai Y, 2020, GASTROINTEST ENDOSC, V92, P623, DOI 10.1016/j.gie.2020.03.3856
Nakai Y, 2019, CLIN ENDOSC, V52, P220
Nara S, 2021, EXPERT REV GASTROENT, V15, P537, DOI 10.1080/17474124.2021.1911645
Nehme F, 2022, DIGEST ENDOSC, V34, P428, DOI 10.1111/den.14081
Nguyen-Tang T, 2010, ENDOSCOPY, V42, P232, DOI 10.1055/s-0029-1243858
Ogura T, 2021, DEN OPEN, V1, DOI 10.1002/deo2.8
Ogura T, 2018, DIGEST ENDOSC, V30, P252, DOI 10.1111/den.12976
Ogura T, 2015, WORLD J GASTROENTERO, V21, P820, DOI 10.3748/wjg.v21.i3.820
Ogura T, 2015, ENDOSCOPY, V47, P72, DOI 10.1055/s-0034-1378111
Paik WH, 2018, AM J GASTROENTEROL, V113, P987, DOI 10.1038/s41395-018-0122-8
Park JK, 2018, GASTROINTEST ENDOSC, V88, P277, DOI 10.1016/j.gie.2018.03.015
Sauvanet A, 2015, J AM COLL SURGEONS, V221, P380, DOI 10.1016/j.jamcollsurg.2015.03.058
Sundaram S, 2023, DIGEST DIS SCI, V68, P1551, DOI 10.1007/s10620-022-07655-w
Teoh A., 2021, ENDOSCOPY, V53, pS39
Tol JAMG, 2016, GUT, V65, P1981, DOI 10.1136/gutjnl-2014-308762
Tonozuka R, 2016, GASTROINTEST ENDOSC, V84, P206, DOI 10.1016/j.gie.2016.03.020
Tsuchiya T, 2018, GASTROINTEST ENDOSC, V87, P1138, DOI 10.1016/j.gie.2017.08.017
Tsuchiya T, 2016, DIGEST ENDOSC, V28, P96, DOI 10.1111/den.12611
Tyberg Amy, 2023, J Clin Gastroenterol, V57, P962, DOI 10.1097/MCG.0000000000001795
Umeda J, 2015, J GASTROENTEROL, V50, P940, DOI 10.1007/s00535-015-1096-6
Umeda J, 2015, GASTROINTEST ENDOSC, V82, P390, DOI 10.1016/j.gie.2015.02.041
van der Gaag NA, 2010, NEW ENGL J MED, V362, P129, DOI 10.1056/NEJMoa0903230
van Wanrooij RLJ, 2022, ENDOSCOPY, V54, P310, DOI 10.1055/a-1738-6780
Vila JJ, 2012, GASTROINTEST ENDOSC, V76, P1133, DOI 10.1016/j.gie.2012.08.001
Whipple AO, 1935, ANN SURG, V102, P763, DOI 10.1097/00000658-193510000-00023
Wiersema MJ, 1996, GASTROINTEST ENDOSC, V43, P102, DOI 10.1016/S0016-5107(06)80108-2
Yamamoto K, 2018, ENDOSC ULTRASOUND, V7, P404, DOI 10.4103/eus.eus_51_18
Yamao K, 2008, ENDOSCOPY, V40, P340, DOI 10.1055/s-2007-995485
NR 72
TC 6
Z9 11
U1 0
U2 2
PU TAYLOR & FRANCIS LTD
PI ABINGDON

PA 2-4 PARK SQUARE, MILTON PARK, ABINGDON OR14 4RN, OXON, ENGLAND
SN 1747-4124
EI 1747-4132
J9 EXPERT REV GASTROENT
JI Expert Rev. Gastroenterol. Hepatol.
PD DEC 2
PY 2023
VL 17
IS 12
BP 1197
EP 1204
DI 10.1080/17474124.2023.2293813
EA DEC 2023
PG 8
WC Gastroenterology & Hepatology
WE Science Citation Index Expanded (SCI-EXPANDED)
SC Gastroenterology & Hepatology
GA FD4R8
UT WOS:001129500100001
PM 38124621
DA 2026-05-09
ER

PT J
AU Xu, RM
Zhang, K
Guo, JT
Sun, SY
AF Xu, Rongmin
Zhang, Kai
Guo, Jintao
Sun, Siyu
TI A review of endoscopic ultrasound-guided gallbladder drainage and gastroenterostomy: assisted approaches and comparison with alternative techniques
SO THERAPEUTIC ADVANCES IN GASTROENTEROLOGY
LA English
DT Review
DE endoscopic enteral stenting; EUS-GBD; EUS-GE; laparoscopic cholecystectomy; peroral cholecystoscopy; PT-GBD
ID GASTRIC OUTLET OBSTRUCTION; CONFOCAL LASER ENDOMICROSCOPY; MALIGNANT BILIARY OBSTRUCTION; APPOSING METAL STENT; SURGICAL GASTROJEJUNOSTOMY;

PERCUTANEOUS CHOLECYSTOSTOMY; ACUTE CHOLECYSTITIS; EUS; MANAGEMENT;
TRIAL
AB Over the last 40 years, the role of endoscopic ultrasound (EUS) has evolved from being diagnostic to therapeutic. EUS-guided gallbladder drainage (EUS-GBD) and EUS-guided gastroenterostomy (EUS-GE) are emerging techniques in recent years; however, there are limited studies and inconsistent results regarding these techniques. In addition, EUS has become a more common alternative to traditional interventions due to its super minimally invasive nature, but the mobility of both the gallbladder and intestine makes it challenging to introduce stents. An increasing number of researchers are dedicating themselves to solving this problem, leading to the development of various assisted technologies. Consequently, this review focused on the comparison of EUS-GBD and EUS-GE with other alternative approaches and explored the various assisted techniques employed for EUS-GBD and EUS-GE.
C1 [Xu, Rongmin; Zhang, Kai; Guo, Jintao; Sun, Siyu] China Med Univ, Dept Gastroenterol, Shengjing Hosp, 36 Sanhao St, Shenyang 110004, Liaoning, Peoples R China.
C3 China Medical University
RP Sun, SY (corresponding author), China Med Univ, Dept Gastroenterol, Shengjing Hosp, 36 Sanhao St, Shenyang 110004, Liaoning, Peoples R China.
EM sun-siyu@163.com
RI sun, siyu/AAD-4972-2019
OI Xu, Rongmin/0009-0002-9457-0807
FU Scientific Research Fund of Liaoning Province Education Department
[JYTQN 2023025]; Young and middle-aged science and technology innovation
talent Program [RC220482]; The 345 Talent Project
FX The authors disclosed receipt of the following financial support for the
research, authorship, and/or publication of this article: This work was
supported by the Scientific Research Fund of Liaoning Province Education
Department (Grant No. JYTQN 2023025), Young and middle-aged science and
technology innovation talent Program (Grant No. RC220482), and 345
Talent Project.
CR Abbas A, 2022, ENDOSCOPY, V54, P671, DOI 10.1055/a-1708-0037
Abel WF, 2024, SURG ENDOSC, V38, P3849, DOI 10.1007/s00464-024-10897-6
Asghar M, 2024, THER ADV GASTROENTER, V17, DOI 10.1177/17562848241248219
Bazaga S, 2024, J GASTROEN HEPATOL, V39, P360, DOI 10.1111/jgh.16392
Bellocchi MCC, 2024, CANCERS, V16, DOI 10.3390/cancers16040724
Boghossian MB, 2021, LANGENBECK ARCH SURG, V406, P1803, DOI 10.1007/s00423-021-02215-8
Bomman S, 2022, ENDOSC INT OPEN, V10, pE361, DOI 10.1055/a-1783-8949
Boregowda U, 2023, DIAGNOSTICS, V13, DOI 10.3390/diagnostics13040657
Bronswijk M, 2023, DIGEST ENDOSC, V35, P255, DOI 10.1111/den.14381
Bronswijk M, 2021, GASTROINTEST ENDOSC, V94, P526, DOI 10.1016/j.gie.2021.04.006
Canakis A, 2023, GASTROINTEST ENDOSC, V98, P348, DOI 10.1016/j.gie.2023.03.022
Carbajo AY, 2020, J CLIN GASTROENTEROL, V54, P1, DOI 10.1097/MCG.0000000000001262
Chan SM, 2023, DIGEST ENDOSC, V35, P512, DOI 10.1111/den.14472
Chan SM, 2017, GASTROINTEST ENDOSC, V85, P1225, DOI 10.1016/j.gie.2016.10.014
Chandan S, 2021, ENDOSC INT OPEN, V09, pE496, DOI 10.1055/a-1341-0788

Chang JI, 2019, ENDOSC INT OPEN, V7, pE655, DOI 10.1055/a-0826-4309
Chavarría C, 2023, ENDOSC ULTRASOUND, V12, P305, DOI 10.4103/EUS-D-22-00178
Chen YI, 2018, ENDOSC INT OPEN, V6, pE363, DOI 10.1055/s-0043-123468
Chen YI, 2017, SURG ENDOSC, V31, P2946, DOI 10.1007/s00464-016-5311-1
Cho SH, 2023, DIGEST ENDOSC, V35, P658, DOI 10.1111/den.14485
Cho SH, 2020, GASTROINTEST ENDOSC, V91, P543, DOI 10.1016/j.gie.2019.09.042
Cotton PB, 2010, GASTROINTEST ENDOSC, V71, P446, DOI 10.1016/j.gie.2009.10.027
Cucchetti A, 2022, GASTROINTEST ENDOSC, V95, P399, DOI 10.1016/j.gie.2021.09.028
Dindo D, 2004, ANN SURG, V240, P205, DOI [10.17116/hirurgia2018090162, 10.1097/01.sla.0000133083.54934.ae]
Dong ZY, 2023, FRONT MICROBIOL, V14, DOI 10.3389/fmicb.2023.1194419
Fabbri C, 2022, SURG ENDOSC, V36, P7974, DOI 10.1007/s00464-022-09339-y
Fan WT, 2022, MINIM INVASIV THER, V31, P159, DOI 10.1080/13645706.2020.1792500
Fischer H, 2022, ENDOSC INT OPEN, V10, pE1358, DOI 10.1055/a-1907-5393
Flynn D, 2021, ENDOSC ULTRASOUND, V10, P381, DOI 10.4103/EUS-D-21-00040
Fuentes-Valenzuela E, 2023, ENDOSC ULTRASOUND, V12, P164, DOI 10.4103/EUS-D-21-00209
Gallaher JR, 2022, JAMA-J AM MED ASSOC, V327, P965, DOI 10.1001/jama.2022.2350
Gangwani MK, 2024, ENDOSC ULTRASOUND, V13, P16, DOI 10.1097/eus.0000000000000032
Garcia-Alonso FJ, 2023, GASTROINTEST ENDOSC, V98, P28, DOI 10.1016/j.gie.2023.02.015
Ge N, 2020, ENDOSC ULTRASOUND, V9, P162, DOI 10.4103/eus.eus_36_20
Ge N, 2016, BMC GASTROENTEROL, V16, DOI 10.1186/s12876-016-0420-9
Ge N, 2014, BMC GASTROENTEROL, V14, DOI 10.1186/1471-230X-14-164
Ge PS, 2019, SURG ENDOSC, V33, P3404, DOI 10.1007/s00464-018-06636-3
Hayat U, 2024, GASTROINTEST ENDOSC, V99, DOI 10.1016/j.gie.2023.10.043
He XJ, 2022, BMC GASTROENTEROL, V22, DOI 10.1186/s12876-022-02269-y
Hemerly MC, 2023, SURG ENDOSC, V37, P2421, DOI 10.1007/s00464-022-09712-x
Hu JL, 2019, DIGEST ENDOSC, V31, pE11, DOI 10.1111/den.13276
Irani Shayan, 2020, VideoGIE, V5, P125, DOI [10.1016/j.vgie.2019.12.005, 10.1016/j.vgie.2019.12.005]
Irani S, 2017, CLIN GASTROENTEROL H, V15, P738, DOI 10.1016/j.cgh.2016.12.021
James TW, 2020, GASTROINTEST ENDOSC, V91, P537, DOI 10.1016/j.gie.2019.11.017
Jaruvongvanich V, 2023, ENDOSC INT OPEN, V11, pE60, DOI 10.1055/a-1976-2279
Kahaleh Michel, 2024, J Clin Gastroenterol, V58, P570, DOI 10.1097/MCG.0000000000001903
Kalva NR, 2018, CAN J GASTROENTEROL, V2018, DOI 10.1155/2018/7070961
Kamal F, 2023, ENDOSC ULTRASOUND, V12, DOI 10.4103/EUS-D-22-00077
Kamal F, 2023, ENDOSC ULTRASOUND, V12, P8, DOI 10.4103/EUS-D-21-00206
Kastelijn JB, 2023, TRIALS, V24, DOI 10.1186/s13063-023-07522-7
Khashab MA, 2017, ENDOSC INT OPEN, V5, pE275, DOI 10.1055/s-0043-101695
Koizumi K, 2023, ENDOSC ULTRASOUND, V12, P342, DOI 10.1097/eus.0000000000000005
Kouanda A, 2021, SURG ENDOSC, V35, P7058, DOI 10.1007/s00464-020-08221-z
Koutlas NJ, 2023, DIAGNOSTICS, V13, DOI 10.3390/diagnostics13111933
Kumar A, 2022, ENDOSC INT OPEN, V10, pE448, DOI 10.1055/a-1765-4035
Kurihara H., 1647, Medicina (Kaunas, Lithuania), V58
Larghi A, 2023, ENDOSC ULTRASOUND, V12, P386, DOI 10.1097/eus.0000000000000015

Larghi A, 2022, ENDOSC ULTRASOUND, V11, P84, DOI 10.4103/EUS-D-21-00033
Law R, 2016, CLIN GASTROENTEROL H, V14, P476, DOI 10.1016/j.cgh.2015.10.026
Li JS, 2023, MINIM INVASIV THER, V32, P285, DOI 10.1080/13645706.2023.2221336
Li P, 2023, ENDOSC ULTRASOUND, V12, P259, DOI 10.4103/EUS-D-22-00058
Luk SWY, 2019, ENDOSCOPY, V51, P722, DOI 10.1055/a-0929-6603
Mangiavillano B, 2024, DIGEST ENDOSC, V36, P351, DOI 10.1111/den.14606
Mangiavillano B, 2023, ENDOSC ULTRASOUND, V12, P277, DOI 10.4103/EUS-D-22-00002
Marino A, 2022, ENDOSCOPY, V54, P170, DOI 10.1055/a-1392-4546
Marrache MK, 2021, GASTROINTEST ENDOSC, V93, P34, DOI 10.1016/j.gie.2020.06.057
Martinet E, 2024, LANGENBECK ARCH SURG, V409, DOI 10.1007/s00423-024-03365-1
Matsubara S, 2023, J CLIN MED, V12, DOI 10.3390/jcm12103524
Matsumi A, 2024, GASTROINTEST ENDOSC, V100, P76, DOI 10.1016/j.gie.2024.02.019
Matsunami Y, 2023, ENDOSC ULTRASOUND, V12, P251, DOI 10.4103/EUS-D-22-00059
Miller C, 2023, ENDOSC INT OPEN, V11, pE660, DOI 10.1055/a-2098-2570
Mohan BP, 2020, ENDOSCOPY, V52, P96, DOI 10.1055/a-1020-3932
Monino L, 2023, ENDOSCOPY, V55, P991, DOI 10.1055/a-2119-7529
Moond V, 2024, ENDOSC ULTRASOUND, V13, P171, DOI 10.1097/eus.0000000000000055
Ogura T, 2023, ENDOSC ULTRASOUND, V12, P384, DOI 10.1097/eus.0000000000000012
Ogura T, 2023, ENDOSC ULTRASOUND, V12, P345, DOI 10.1097/eus.0000000000000013
Ogura T, 2022, GASTROINTEST ENDOSC, V96, P262, DOI 10.1016/j.gie.2022.03.013
Okamoto K, 2018, J HEPATO-BIL-PAN SCI, V25, P55, DOI 10.1002/jhbp.516
On W, 2023, SURG ENDOSC, V37, P1749, DOI 10.1007/s00464-022-09692-y
Park SW, 2022, DIGEST ENDOSC, V34, P439, DOI 10.1111/den.14083
Perez-Cuadrado-Robles E, 2022, CANCERS, V14, DOI 10.3390/cancers14225516
Perez-Miranda M, 2018, ENDOSC ULTRASOUND, V7, P79, DOI 10.4103/eus.eus_5_18
Perez-Miranda M, 2017, J CLIN GASTROENTEROL, V51, P896, DOI 10.1097/MCG.0000000000000887
Puigcerver-Mas M, 2023, ENDOSC ULTRASOUND, V12, P388, DOI 10.1097/eus.0000000000000019
Robles-Medranda C, 2023, GASTROINTEST ENDOSC, V97, P445, DOI 10.1016/j.gie.2022.10.037
Saumoy M, 2021, GASTROINTEST ENDOSC, V94, P671, DOI 10.1016/j.gie.2021.05.031
Seitz N, 2024, SURG ENDOSC, V38, P2260, DOI 10.1007/s00464-024-10745-7
Shen YH, 2020, J DIGEST DIS, V21, P422, DOI 10.1111/1751-2980.12865
Shen YH, 2020, MEDICINE, V99, DOI 10.1097/MD.0000000000022903
Shin I, 2024, GASTROINTEST ENDOSC, V100, DOI 10.1016/j.gie.2024.04.2910
Shiomi H, 2023, ENDOSCOPY, V55, pE362, DOI 10.1055/a-1997-9382
Snchez-Aldehuelo R., 2022, Gastrointest Endosc, V96
Suehiro H, 2023, ENDOSC ULTRASOUND, V12, P143, DOI 10.4103/EUS-D-21-00258
Sun SY, 2018, ENDOSC ULTRASOUND, V7, P73, DOI 10.4103/eus.eus_14_18
Takinami M, 2020, J HEPATO-BIL-PAN SCI, V27, P470, DOI 10.1002/jhbp.767
Tang BF, 2023, DIGEST DIS, V41, P325, DOI 10.1159/000526678
Teoh AYB, 2020, GUT, V69, P1085, DOI 10.1136/gutjnl-2019-319996
Teoh AYB, 2014, ENDOSCOPY, V46, pE13, DOI 10.1055/s-0033-1359139
Teoh AYB, 2024, LANCET GASTROENTEROL, V9, P124, DOI 10.1016/S2468-1253(23)00242-X

Teoh AYB, 2023, GASTROINTEST ENDOSC, V97, P454, DOI 10.1016/j.gie.2022.11.016
Teoh AYB, 2021, DIGEST ENDOSC, V33, P1139, DOI 10.1111/den.13911
Teoh AYB, 2021, GASTROINTEST ENDOSC, V93, P577, DOI 10.1016/j.gie.2020.06.066
Teoh AYB, 2017, ENDOSCOPY, V49, P130, DOI 10.1055/s-0042-119036
Teshima CW, 2010, ENDOSCOPY, V42, P944, DOI 10.1055/s-0030-1255795
Tonozuka R, 2020, CLIN ENDOSC, V53, P510, DOI 10.5946/ce.2020.151
Troncone E, 2024, MEDICINA-LITHUANIA, V60, DOI 10.3390/medicina60040633
Tyberg A, 2023, CLIN TRANSL GASTROEN, V14, DOI 10.14309/ctg.0000000000000593
Tyberg A, 2018, J CLIN GASTROENTEROL, V52, P79, DOI 10.1097/MCG.0000000000000786
van der Merwe SW, 2022, ENDOSCOPY, V54, P185, DOI 10.1055/a-1717-1391
van Wanrooij RLJ, 2022, ENDOSCOPY, V54, P1023, DOI 10.1055/a-1782-7568
van Wanrooij RLJ, 2022, ENDOSCOPY, V54, P310, DOI 10.1055/a-1738-6780
Vanella G, 2023, GASTROINTEST ENDOSC, V98, DOI 10.1016/j.gie.2023.04.2072
Vanella G, 2023, ENDOSC ULTRASOUND, V12, P16, DOI 10.4103/EUS-D-21-00177
Vanella Giuseppe, 2022, VideoGIE, V7, P120, DOI [10.1016/j.vgie.2021.10.002, 10.1016/j.vgie.2021.10.002]
Voiosu T, 2023, GASTROINTEST ENDOSC, V98, P466, DOI 10.1016/j.gie.2023.03.028
Wang GX, 2020, WORLD J GASTROENTERO, V26, P3603, DOI 10.3748/wjg.v26.i25.3603
Wang ZJ, 2023, ENDOSC ULTRASOUND, V12, P29, DOI 10.4103/EUS-D-21-00272
Xu N, 2024, ENDOSC ULTRASOUND, V13, P40, DOI 10.1097/eus.0000000000000039
Xu YY, 2022, FRONT MICROBIOL, V13, DOI 10.3389/fmicb.2022.800604
Yoo HW, 2022, ENDOSCOPY, V54, P384, DOI 10.1055/a-1518-7039
Zaftis J, 2023, ENDOSC ULTRASOUND, V12, P150, DOI 10.4103/EUS-D-21-00099
Zhang K, 2018, GASTROINTEST ENDOSC, V88, P957, DOI 10.1016/j.gie.2018.07.019
Zhang Y, 2018, PLOS ONE, V13, DOI 10.1371/journal.pone.0204142
NR 123
TC 1
Z9 1
U1 1
U2 2
PU SAGE PUBLICATIONS LTD
PI LONDON
PA 1 OLIVERS YARD, 55 CITY ROAD, LONDON EC1Y 1SP, ENGLAND
EI 1756-2848
J9 THER ADV GASTROENTER
JI Ther. Adv. Gastroenterol.
PY 2024
VL 17
AR 17562848241299755
DI 10.1177/17562848241299755
PG 15
WC Gastroenterology & Hepatology
WE Science Citation Index Expanded (SCI-EXPANDED)

SC Gastroenterology & Hepatology
GA O2I0D
UT WOS:001369418000001
PM 39635228
OA Green Submitted, gold
DA 2026-05-09
ER

PT J
AU Baniya, R
Upadhaya, S
Madala, S
Subedi, SC
Mohammed, TS
Bachuwa, G
AF Baniya, Ramkaji
Upadhaya, Sunil
Madala, Seetharamprasad
Subedi, Subash Chandra
Mohammed, Tabrez Shaik
Bachuwa, Ghassan
TI Endoscopic ultrasound-guided biliary drainage versus percutaneous
transhepatic biliary drainage after failed endoscopic retrograde
cholangiopancreatography: a meta-analysis
SO CLINICAL AND EXPERIMENTAL GASTROENTEROLOGY
LA English
DT Article
DE failed endoscopic retrograde cholangiopancreatography; percutaneous
transhepatic biliary drainage; endoscopic ultrasound-guided biliary
drainage; technical success; clinical success
ID SINGLE-STEP PLACEMENT; GALLBLADDER DRAINAGE; PANCREATIC-CANCER; METAL
STENTS; MALIGNANT OBSTRUCTION; DUCT OBSTRUCTION; ERCP;
CHOLEDOCHODUODENOSTOMY; COMPLICATIONS; OUTCOMES
AB The failure rate of endoscopic retrograde cholangiopancreatography for biliary cannulation is approximately 6%-7% in cases of obstructive jaundice. Percutaneous transhepatic biliary drainage (PTBD) is the procedure of choice in such cases. Endoscopic ultrasound-guided biliary drainage (EGBD) is a novel technique that allows biliary drainage by echoendoscopy and fluoroscopy using a stent from the biliary tree to the gastrointestinal tract. Information in PubMed, Scopus, clinicaltrials.gov and Cochrane review were analyzed to obtain studies comparing EGBD and PTBD. Six studies fulfilled the inclusion criteria. Technical (odds ratio (OR): 0.34; confidence interval (CI) 0.10-1.14; p=0.05) and clinical (OR: 1.48; CI 0.46-4.79; p=0.51) success rates were not statistically significant between the EGBD and PTBD groups. Mild adverse events were nonsignificantly different (OR: 0.36; CI 0.10-1.24; p=0.11) but not the moderate-to-severe adverse events (OR: 0.16; CI 0.08-0.32; p <= 0.00001) and total adverse events (OR: 0.34; CI 0.20-0.59; p <= 0.0001). EGBD is equally

effective but safer than PTBD.

C1 [Baniya, Ramkaji; Upadhaya, Sunil; Madala, Seetharamprasad; Subedi, Subash Chandra; Mohammed, Tabrez Shaik; Bachuwa, Ghassan] Michigan State Univ, Hurley Med Ctr, Flint, MI USA.

C3 Michigan State University

RP Baniya, R (corresponding author), Michigan State Univ, Hurley Med Ctr, Dept Internal Med, One Hurley Plaza,Suite 212, Flint, MI 48503 USA.

EM mail2ramkazi@gmail.com

RI ; Madala, Seetharamprasad/JMC-7068-2023; Upadhaya, Sunil/MIK-2395-2025

OI Baniya, Ramkaji/0000-0003-4654-3169; Madala,
Seetharamprasad/0000-0001-8985-0965;

CR Artifon ELA, 2006, AM J GASTROENTEROL, V101, P2031, DOI 10.1111/j.1572-0241.2006.00764.x
Artifon ELA, 2012, J CLIN GASTROENTEROL, V46, P768, DOI 10.1097/MCG.0b013e31825f264c
Bahra M., 2008, V177, P111
Bapaye A, 2013, UNITED EUR GASTROENT, V1, P285, DOI 10.1177/2050640613490928
Súbtil JC, 2010, WORLD J GASTRO ENDOS, V2, P203, DOI 10.4253/wjge.v2.i6.203
Choi JH, 2017, GASTROINTEST ENDOSC, V85, P357, DOI 10.1016/j.gie.2016.07.067
Choi JH, 2014, ENDOSCOPY, V46, P656, DOI 10.1055/s-0034-1365720
Coté GA, 2012, CLIN GASTROENTEROL H, V10, P920, DOI 10.1016/j.cgh.2012.02.019
Dhir V, 2015, GASTROINTEST ENDOSC, V81, P913, DOI 10.1016/j.gie.2014.09.054
Enochsson L, 2010, GASTROINTEST ENDOSC, V72, P1175, DOI 10.1016/j.gie.2010.07.047
FERRUCCI JT, 1980, RADIOLOGY, V135, P1
Giovannini M, 2001, ENDOSCOPY, V33, P898, DOI 10.1055/s-2001-17324
Giovannini M, 2015, GASTROINTEST ENDOSC, V81, pAB174, DOI 10.1016/j.gie.2015.03.1926
Gupta K, 2007, REV GASTROENTEROL DI, V7, P22
HARBIN WP, 1980, RADIOLOGY, V135, P15, DOI 10.1148/radiology.135.1.6987704
Holt BA, 2016, GASTROINTEST ENDOSC, V83, P160, DOI 10.1016/j.gie.2015.06.019
Irani S, 2015, GASTROINTEST ENDOSC, V82, P1110, DOI 10.1016/j.gie.2015.05.045
Itoi T, 2008, WORLD J GASTROENTERO, V14, P6078, DOI 10.3748/wjg.14.6078
Itoi T, 2016, GASTROINTEST ENDOSC, V84, P947, DOI 10.1016/j.gie.2016.06.043
James PD, 2016, BEST PRACT RES CL GA, V30, P421, DOI 10.1016/j.bpg.2016.05.003
Jang JW, 2011, GASTROINTEST ENDOSC, V74, P176, DOI 10.1016/j.gie.2011.03.1120
Kahaleh M, 2013, WORLD J GASTROENTERO, V19, P1372, DOI 10.3748/wjg.v19.i9.1372
Kawakubo K, 2016, ENDOSCOPY, V48, P164, DOI 10.1055/s-0034-1393179
Kawakubo K, 2014, J HEPATO-BIL-PAN SCI, V21, P328, DOI 10.1002/jhbp.27
Khajanchee YS, 2012, J GASTROINTEST SURG, V16, P801, DOI 10.1007/s11605-011-1768-3
Khashab MA, 2015, DIGEST DIS SCI, V60, P557, DOI 10.1007/s10620-014-3300-6
Lakhtakia S, 2016, BEST PRACT RES CL GA, V30, P807, DOI 10.1016/j.bpg.2016.10.008
LAMERIS JS, 1991, RADIOLOGY, V179, P703, DOI 10.1148/radiology.179.3.2027978
Lee TH, 2016, CLIN GASTROENTEROL H, V14, P1011, DOI 10.1016/j.cgh.2015.12.032
Luu C, 2013, AM SURGEON, V79, P1054
Nakai Y, 2016, ENDOSCOPY, V48, P1125, DOI 10.1055/s-0042-116595
Nennstiel S, 2015, J CLIN GASTROENTEROL, V49, P764, DOI 10.1097/MCG.0000000000000275

Piñol V, 2002, RADIOLOGY, V225, P27, DOI 10.1148/radiol.2243011517
Püspök A, 2005, AM J GASTROENTEROL, V100, P1743, DOI 10.1111/j.1572-0241.2005.41806.x
Sharaiha RZ, 2016, SURG ENDOSC, V30, P5500, DOI 10.1007/s00464-016-4913-y
SMITH AC, 1994, LANCET, V344, P1655, DOI 10.1016/S0140-6736(94)90455-3
Song TJ, 2010, GASTROINTEST ENDOSC, V71, P634, DOI 10.1016/j.gie.2009.11.024
Spanheimer PM, 2014, J SURG ONCOL, V109, P697, DOI 10.1002/jso.23560
Teoh AYB, 2014, GASTROINTEST ENDOSC, V80, P1171, DOI 10.1016/j.gie.2014.03.038
Torres-Ruiz MF, 2016, GASTROINTEST ENDOSC, V83, pAB356, DOI 10.1016/j.gie.2016.03.910
Tyberg Amy., 2016, J CLIN GASTROENTEROL
Varadarajulu S, 2006, GASTROINTEST ENDOSC, V64, P338, DOI 10.1016/j.gie.2005.05.016
Wang KX, 2016, GASTROINTEST ENDOSC, V83, P1218, DOI 10.1016/j.gie.2015.10.033
Widmer J, 2014, DIGEST ENDOSC, V26, P599, DOI 10.1111/den.12163
NR 44
TC 39
Z9 50
U1 0
U2 4
PU DOVE MEDICAL PRESS LTD
PI ALBANY
PA PO BOX 300-008, ALBANY, AUCKLAND 0752, NEW ZEALAND
SN 1178-7023
J9 CLIN EXP GASTROENTER
JI Clin. Exp. Gastroenterol.
PY 2017
VL 10
BP 67
EP 74
DI 10.2147/CEG.S132004
PG 8
WC Gastroenterology & Hepatology
WE Emerging Sources Citation Index (ESCI)
SC Gastroenterology & Hepatology
GA EQ4EX
UT WOS:000398028700001
PM 28408850
OA Green Submitted, gold
DA 2026-05-09
ER

PT J
AU Iwamuro, M
Kawamoto, H

Harada, R
Kato, H
Hirao, K
Mizuno, O
Ishida, E
Ogawa, T
Okada, H
Yamamoto, K
AF Iwamuro, Masaya
Kawamoto, Hirofumi
Harada, Ryo
Kato, Hironari
Hirao, Ken
Mizuno, Osamu
Ishida, Etsuji
Ogawa, Tsuneyoshi
Okada, Hiroyuki
Yamamoto, Kazuhide
TI COMBINED DUODENAL STENT PLACEMENT AND ENDOSCOPIC ULTRASONOGRAPHY-GUIDED BILIARY DRAINAGE FOR MALIGNANT DUODENAL OBSTRUCTION WITH BILIARY STRICTURE
SO DIGESTIVE ENDOSCOPY
LA English
DT Article
DE biliary drainage; duodenal stent; endoscopic ultrasonography; pancreatic cancer
ID GROUP DOCUMENT EVALUATION; CHOLANGIOPANCREATOGRAPHY; CHOLEDOCHODUODENOSTOMY
AB Patients with malignant pancreatobiliary neoplasm sometimes manifest duodenal obstruction and biliary stricture synchronously or metachronously. In this paper, we reviewed our experience with and technique for combined endoscopic duodenal stent placement and endoscopic ultrasonography (EUS)-guided biliary drainage. Between May 2007 and September 2009, this combined technique was performed on seven patients with distal biliary strictures and duodenal obstructions. The clinical success rate of the procedure, complications, patency periods of duodenal stents and patency periods of biliary stents were retrospectively evaluated. Clinical success was achieved in all seven cases for both procedures. Complications related to EUS-biliary drainage, namely localized peritonitis due to bile leakage, occurred in two cases. Both patients recovered without additional interventions. Occlusion of a duodenal stent was observed in one patient, but additional intervention could not be performed due to sepsis. Occlusion of both a duodenal stent and a biliary stent was also observed in one patient, and this was resolved with the insertion of an additional duodenal stent and a biliary stent exchange. In conclusion, combined duodenal stent placement and EUS-guided biliary drainage is a therapeutic option in case of failed endoscopic retrograde cannulation of malignant strictures with a malignant duodenal obstruction.
C1 [Kawamoto, Hirofumi] Okayama Univ, Grad Sch Med Dent & Pharmaceut Sci, Dept Gastroenterol & Hepatol, Kita Ku, Okayama 7008558, Japan.

C3 Okayama University
RP Kawamoto, H (corresponding author), Okayama Univ, Grad Sch Med Dent & Pharmaceut Sci, Dept Gastroenterol & Hepatol, Kita Ku, 2-5-1 Shikata Cho, Okayama 7008558, Japan.
EM h-kawamo@md.okayama-u.ac.jp
OI Iwamuro, Masaya/0000-0002-1757-5754
CR Beissert M, 2002, Z GASTROENTEROL, V40, P503, DOI 10.1055/s-2002-32806
Bories E, 2007, ENDOSCOPY, V39, P287, DOI 10.1055/s-2007-966212
Burmester E, 2003, GASTROINTEST ENDOSC, V57, P246, DOI 10.1067/mge.2003.85
Fogel EL, 2001, ENDOSCOPY, V33, P31, DOI 10.1055/s-2001-11186
Giovannini M, 2001, ENDOSCOPY, V33, P898, DOI 10.1055/s-2001-17324
Harada R, 2007, ENDOSCOPY, V39, pE284, DOI 10.1055/s-2007-966707
Itoi T, 2009, GASTROINTEST ENDOSC, V69, pS8, DOI 10.1016/j.gie.2008.11.003
Jeurnink SM, 2007, BMC GASTROENTEROL, V7, DOI 10.1186/1471-230X-7-18
Kahaleh M, 2006, GASTROINTEST ENDOSC, V64, P52, DOI 10.1016/j.gie.2006.01.063
Kama NA, 1999, HEPATO-GASTROENTEROL, V46, P103
LAMERIS JS, 1991, RADIOLOGY, V179, P703, DOI 10.1148/radiology.179.3.2027978
Mallery S, 2004, GASTROINTEST ENDOSC, V59, P100, DOI 10.1016/S0016-5107(03)02300-9
PERRY LJ, 1995, RADIOLOGY, V195, P163, DOI 10.1148/radiology.195.1.7892460
Püspök A, 2005, AM J GASTROENTEROL, V100, P1743, DOI 10.1111/j.1572-0241.2005.41806.x
Savides TJ, 2009, GASTROINTEST ENDOSC, V69, pS3, DOI 10.1016/j.gie.2008.10.060
Schöfl R, 2001, ENDOSCOPY, V33, P147
Wiersema MJ, 1996, GASTROINTEST ENDOSC, V43, P102, DOI 10.1016/S0016-5107(06)80108-2
NR 17
TC 44
Z9 49
U1 0
U2 1
PU WILEY-BLACKWELL
PI MALDEN
PA COMMERCE PLACE, 350 MAIN ST, MALDEN 02148, MA USA
SN 0915-5635
J9 DIGEST ENDOSC
JI Dig. Endosc.
PD JUL
PY 2010
VL 22
IS 3
BP 236
EP 240
DI 10.1111/j.1443-1661.2010.00997.x
PG 5
WC Gastroenterology & Hepatology; Surgery

WE Science Citation Index Expanded (SCI-EXPANDED)
SC Gastroenterology & Hepatology; Surgery
GA 613YM
UT WOS:000279021300014
PM 20642617
DA 2026-05-09
ER

PT J
AU Rimbas, M
Larghi, A
Costamagna, G
AF Rimbas, Mihai
Larghi, Alberto
Costamagna, Guido
TI Endoscopic ultrasound-guided gastroenterostomy: Are we ready for prime time?
SO ENDOSCOPIC ULTRASOUND
LA English
DT Review
DE Endoscopic ultrasonography; endoscopic ultrasonography-guided gastroenterostomy; endoscopic ultrasonography-guided gastroduodenostomy; endoscopic ultrasonography-guided gastrojejunostomy; gastric outlet obstruction
ID APPOSING METAL STENT; GASTRIC OUTLET OBSTRUCTION; OCCLUDED GASTROJEJUNOSTOMY BYPASS; CLINICAL-EXPERIENCE; VIDEOS; MULTICENTER
AB Interventional endoscopic ultrasonography (EUS) is currently becoming the less invasive therapeutic approach for the drainage of pancreatic fluid collections, of acute cholecystitis in patients unfit for surgery and for biliary drainage after failed endoscopic retrograde cholangiopancreatography. In addition, EUS-guided gastroenterostomy (EUS-GE) has recently emerged as a feasible procedure to treat patients with gastric outlet obstruction, as an alternative to surgery or to standard endoscopy when endoscopic stent placement is not possible. Prior animal studies have shown that the procedure is safe and can create a stable anastomosis. However, the major challenge in translating the results of the animal studies into clinical practice is represented by the proper identification of the distal duodenal or proximal jejunal loop to be accessed in order to create the anastomosis. Currently, there are three EUS-GE techniques available: the direct EUS-GE technique, assisted EUS-GE technique, and its variant called the EUS-guided double-balloon-occluded gastrojejunostomy bypass. The present review describes the current EUS-GE techniques, depicts the different procedural aspects of the procedure, and presents the clinical evidences available so far, with a focus on the future perspectives of this EUS-guided technique.
C1 [Rimbas, Mihai; Larghi, Alberto; Costamagna, Guido] Catholic Univ, Digest Endoscopy Unit, Rome, Italy.
[Rimbas, Mihai] Carol Davila Univ Med, Colentina Clin Hosp, Dept Gastroenterol & Internal Med, Bucharest, Romania.
[Costamagna, Guido] Univ Strasbourg, IHU USIAS, Strasbourg, France.
C3 Catholic University of the Sacred Heart; IRCCS Policlinico Gemelli;

Carol Davila University of Medicine & Pharmacy; Universites de Strasbourg Etablissements Associes; Universite de Strasbourg

RP Costamagna, G (corresponding author), Univ Cattolica Sacro Cuore, Digest Endoscopy Unit, Largo A Gemelli 8, I-00168 Rome, Italy.

EM guido.costamagna@unicatt.it

RI Larghi, Alberto/AAC-7448-2022; Rimbas, Mihai/AAF-5521-2020

OI Larghi, Alberto/0000-0001-7131-3686;

CR Barthet M, 2015, GASTROINTEST ENDOSC, V81, P215, DOI 10.1016/j.gie.2014.09.039

Binmoeller KF, 2012, ENDOSCOPY, V44, P499, DOI 10.1055/s-0032-1309382

Chen YI, 2017, SURG ENDOSC, V31, P2946, DOI 10.1007/s00464-016-5311-1

Fritscher-Ravens A, 2002, GASTROINTEST ENDOSC, V56, P737, DOI 10.1067/mge.2002.129084

Fuccio Lorenzo, 2014, Curr Treat Options Gastroenterol, V12, P183, DOI 10.1007/s11938-014-0015-x

Itoi T, 2017, DIGEST ENDOSC, V29, P495, DOI 10.1111/den.12794

Itoi T, 2016, GASTROINTEST ENDOSC, V83, P461, DOI 10.1016/j.gie.2015.08.030

Itoi T, 2016, GUT, V65, P193, DOI 10.1136/gutjnl-2015-310348

Itoi T, 2013, GASTROINTEST ENDOSC, V78, P934, DOI 10.1016/j.gie.2013.09.025

Itoi T, 2012, GASTROINTEST ENDOSC, V75, P870, DOI 10.1016/j.gie.2011.10.020

Khashab MA, 2017, ENDOSC INT OPEN, V5, pE275, DOI 10.1055/s-0043-101695

Khashab MA, 2015, GASTROINTEST ENDOSC, V82, P932, DOI 10.1016/j.gie.2015.06.017

Majmudar K, 2016, GASTROINTEST ENDOSC, V84, P853, DOI 10.1016/j.gie.2016.06.007

Tyberg A, 2016, ENDOSC INT OPEN, V4, pE276, DOI 10.1055/s-0042-101789

NR 14

TC 30

Z9 37

U1 0

U2 2

PU MEDKNOW PUBLICATIONS & MEDIA PVT LTD

PI MUMBAI

PA B-9, KANARA BUSINESS CENTRE, OFF LINK RD, GHAKTOPAR-E, MUMBAI, 400075, INDIA

SN 2303-9027

EI 2226-7190

J9 ENDOSC ULTRASOUND

JI Endosc. Ultrasound

PD JUL-AUG

PY 2017

VL 6

IS 4

BP 235

EP 240

DI 10.4103/eus.eus_47_17

PG 6

WC Gastroenterology & Hepatology
WE Science Citation Index Expanded (SCI-EXPANDED)
SC Gastroenterology & Hepatology
GA FD8PD
UT WOS:000407785700004
PM 28820145
OA Green Submitted
DA 2026-05-09
ER

PT J
AU Sansak, I
Itoi, T
Moriyasu, F
AF Sansak, Inchaya
Itoi, Takao
Moriyasu, Fuminori
TI Endoscopic ultrasonography-guided transhepatic antegrade stone removal
in a patient with Roux-en-Y anastomosis (with video)
SO JOURNAL OF HEPATO-BILIARY-PANCREATIC SCIENCES
LA English
DT Article
DE Bile duct stone; Endoscopic papillary balloon dilation; Endoscopic
ultrasonography; Endoscopic ultrasonography-guided biliary drainage
AB Recently, endoscopic ultrasonography (EUS)-guided biliopancreatic access techniques for cholangiopancreatography and biliopancreatic drainage have been introduced in patients with surgically altered anatomy [1-3]. Furthermore, EUS-guided transhepatic antegrade papillary balloon dilation for the treatment of common bile duct (CBD) stones has emerged [4, 5].
C1 [Sansak, Inchaya; Itoi, Takao; Moriyasu, Fuminori] Tokyo Med Univ, Dept Gastroenterol & Hepatol, Shinjuku Ku, Tokyo 1600023, Japan.
[Sansak, Inchaya] Udonthan Hosp, Dept Gastroenterol & Hepatol, Udon Thani, Thailand.
C3 Tokyo Medical University
RP Itoi, T (corresponding author), Tokyo Med Univ, Dept Gastroenterol & Hepatol, Shinjuku Ku, 6-7-1 Nishishinjuku, Tokyo 1600023, Japan.
EM itoi@tokyo-med.ac.jp
CR Itoi T, 2014, J HEPATO-BIL-PAN SCI, V21, pE4, DOI 10.1002/jhbp.43
Itoi T, 2013, J HEPATO-BIL-PAN SCI, V20, P71, DOI 10.1007/s00534-012-0569-8
Iwashita T, 2013, DIGEST ENDOSC, V25, P89, DOI 10.1111/j.1443-1661.2012.01381.x
Park DH, 2011, GASTROINTEST ENDOSC, V74, P1276, DOI 10.1016/j.gie.2011.07.054
Weilert F, 2011, ENDOSCOPY, V43, P1105, DOI 10.1055/s-0030-1256961
NR 5
TC 5

Z9 7
U1 0
U2 2
PU WILEY
PI HOBOKEN
PA 111 RIVER ST, HOBOKEN 07030-5774, NJ USA
SN 1868-6974
EI 1868-6982
J9 J HEPATO-BIL-PAN SCI
JI J. Hepato-Biliary-Pancreat. Sci.
PD SEP
PY 2014
VL 21
IS 9
BP 719
EP 720
DI 10.1002/jhbp.133
PG 2
WC Gastroenterology & Hepatology; Surgery
WE Science Citation Index Expanded (SCI-EXPANDED)
SC Gastroenterology & Hepatology; Surgery
GA AS4OL
UT WOS:000344255500015
PM 24975269
OA Bronze
DA 2026-05-09
ER

PT J
AU Guzmán-Calderón, E
Chacaltana, A
Díaz, R
Li, B
Martinez-Moreno, B
Aparicio, JR
AF Guzman-Calderon, Edson
Chacaltana, Alfonso
Diaz, Ramiro
Li, Bruno
Martinez-Moreno, Belen
Aparicio, Jose Ramon
TI Head-to-head comparison between endoscopic ultrasound guided lumen

apposing metal stent and plastic stents for the treatment of pancreatic fluid collections: A systematic review and meta-analysis

SO JOURNAL OF HEPATO-BILIARY-PANCREATIC SCIENCES
LA English
DT Review
DE endoscopic ultrasound; lumen apposing metal stent; pancreatic fluid collection; plastic stents
ID WALLED-OFF NECROSIS; INFECTED NECROTIZING PANCREATITIS; DOUBLE-PIGTAIL STENTS; PSEUDOCYST DRAINAGE; TRANSMURAL DRAINAGE; CLINICAL-OUTCOMES; PUBLICATION BIAS; MANAGEMENT; EFFICACY; MULTICENTER

AB Background/Aims Peripancreatic fluid collections (PFCs) result from acute or chronic pancreatic inflammation that suffers a rupture of its ducts. Currently, there exists three options for drainage or debridement of pancreatic pseudocysts and walled-off necrosis (WON). The traditional procedure is drainage by placing double pigtail plastic stents (DPPS); lumen-apposing metal stent (LAMS) has a biflanged design with a wide lumen that avoids occlusion with necrotic tissue, which is more common with DPPS and reduces the possibility of migration. We performed a systematic review and meta-analyses head-to-head, including only studies that compare the two main techniques to drainage of PFCs: LAMS vs DPPS. Methods We conducted a systematic review in different databases, such as PubMed, OVID, Medline, and Cochrane Databases. This meta-analysis considers studies published from 2014 to 2020, including only studies that compare the two main techniques to drainage of PFCs: LAMS vs DPPS. Results Thirteen studies were included in the meta-analyses. Only one of all studies was a randomized controlled trial. These studies comprise 1584 patients; 68.2% were male, and 31.8% were female. Six hundred sixty-three patients (41.9%) were treated with LAMS, and 921 (58.1%) were treated with DPPS. Six studies included only WON in their analysis, two included only pancreatic pseudocysts, and five studies included both pancreatic pseudocysts and WON. The technical success was similar in patients treated with LAMS and DPPS (97.6% vs 97.5%, respectively, P = .986, RR = 1.00 [95% CI 0.93-1.08]). The clinical success was similar in both groups (LAMS: 90.1% vs DPPS: 84.2%, P = .139, RR = 1.063 [95% CI 0.98-1.15]). Patients treated with LAMS had a lower complication rate than the DPPS groups, with a significant statistical difference (LAMS: 16.0% vs DPPS: 20.2%, P = .009, RR = 0.746 [95% CI 0.60-0.93]). Bleeding was the most common complication in the LAMS group (33 patients, [5.0%]), whereas infection was the most common complication in the DPPS group (56 patients, [6.1%]). The LAMS migration rate was lower than in the DPPS (0.9% vs 2.2%, respectively, P = .05). The mortality rate was similar in both groups, 0.6% in the LAMS group (four patients) and 0.4% in the DPPS group (four patients; P = .640). Conclusion The PFCs drainage is an indication when persistent symptoms or PFCs-related complications exist. EUS guided drainage with LAMS has similar technical and clinical success to DPPS drainage for the management of PFCs. The technical and clinical success rates are high in both groups. However, LAMS drainage has a lower adverse events rate than DPPS drainage. More randomized controlled trials are needed to confirm the real advantage of LAMS drainage over DPPS drainage.

C1 [Guzman-Calderon, Edson; Chacaltana, Alfonso; Diaz, Ramiro; Li, Bruno] Hosp Nacl Edgardo Rebagliati Martins, Gastroenterol Unit, Lima, Peru.
[Guzman-Calderon, Edson] Univ Peruana Ciencias Aplicadas UPC, Lima, Peru.
[Guzman-Calderon, Edson] Angloamer Clin, Gastroenterol Unit, Lima, Peru.
[Martinez-Moreno, Belen; Aparicio, Jose Ramon] Hosp Gen Univ Alicante, Gastroenterol Unit, Alicante, Spain.
C3 Seguro Social de Salud del Peru; Universidad Peruana de Ciencias Aplicadas (UPC); General University Hospital of Alicante
RP Guzmán-Calderón, E (corresponding author), Av Edgardo Rebagliati S-N, Lima, Peru.

EM edson_guzman@hotmail.com
RI Díaz, Ramiro/L-1155-2018
OI Guzman Calderon, Edson/0000-0002-1673-3669; Martinez-Moreno, Belen/0000-0002-4025-6735; Aparicio Tormo, Jose Ramón/0000-0003-2245-4006
CR Abu Dayyeh BK, 2018, GASTROINTEST ENDOSC, V87, P141, DOI 10.1016/j.gie.2017.04.032
Aghdassi Alexander A, 2006, HPB (Oxford), V8, P432, DOI 10.1080/13651820600748012
Ang TL, 2016, ENDOSC ULTRASOUND, V5, P320, DOI 10.4103/2303-9027.191659
Arvanitakis M, 2018, ENDOSCOPY, V50, P524, DOI 10.1055/a-0588-5365
Bakker OJ, 2012, JAMA-J AM MED ASSOC, V307, P1053, DOI 10.1001/jama.2012.276
Bang JY, 2019, GUT, V68, P1200, DOI 10.1136/gutjnl-2017-315335
Bang JY, 2017, DIGEST ENDOSC, V29, P83, DOI 10.1111/den.12681
Bapaye A, 2017, DIGEST ENDOSC, V29, P104, DOI 10.1111/den.12704
Bazerbachi F, 2018, GASTROINTEST ENDOSC, V87, P30, DOI 10.1016/j.gie.2017.08.025
Brimhall B, 2018, CLIN GASTROENTEROL H, V16, P1521, DOI 10.1016/j.cgh.2018.02.021
Chandrasekhara V, 2020, ENDOSC INT OPEN, V08, pE1639, DOI 10.1055/a-1243-0092
Chen YI, 2019, ENDOSC INT OPEN, V7, pE347, DOI 10.1055/a-0828-7630
Duval S, 2000, BIOMETRICS, V56, P455, DOI 10.1111/j.0006-341X.2000.00455.x
EASTERBROOK PJ, 1991, LANCET, V337, P867, DOI 10.1016/0140-6736(91)90201-Y
Ge N, 2017, J TRANSL INTERN MED, V5, P213, DOI 10.1515/jtim-2017-0036
Gliem N, 2021, DIGESTION, V102, P503, DOI 10.1159/000506830
Gornals JB, 2019, TRIALS, V20, DOI 10.1186/s13063-019-3988-x
Hammad T, 2018, DIGEST DIS SCI, V63, P289, DOI 10.1007/s10620-017-4851-0
Lakhtakia Sundeep, 2018, VideoGIE, V3, P284, DOI [10.1016/j.vgie.2018.07.001, 10.1016/j.vgie.2018.07.001]
Lang GD, 2018, GASTROINTEST ENDOSC, V87, P150, DOI 10.1016/j.gie.2017.06.029
Mukai S, 2015, ENDOSCOPY, V47, P47, DOI 10.1055/s-0034-1377966
Muniraj T, 2017, J CLIN GASTROENTEROL, V51, P19, DOI 10.1097/MCG.0000000000000644
Penn DE, 2012, GASTROINTEST ENDOSC, V76, P679, DOI 10.1016/j.gie.2012.04.457
Rana SS, 2020, ANN GASTROENTEROL, V33, P426, DOI 10.20524/aog.2020.0499
Saunders R, 2019, SURG ENDOSC, V33, P1412, DOI 10.1007/s00464-018-6416-5
Sharaiha RZ, 2015, GASTROINTEST ENDOSC, V82, P822, DOI 10.1016/j.gie.2015.02.035
Shin HC, 2019, CLIN ENDOSC, V52, P353
Siddiqui AA, 2017, GASTROINTEST ENDOSC, V85, P758, DOI 10.1016/j.gie.2016.08.014
Tan SL, 2020, GASTROENT RES PRACT, V2020, DOI 10.1155/2020/4952721
van Brunschot S, 2018, LANCET, V391, P51, DOI 10.1016/S0140-6736(17)32404-2
Varadarajulu S, 2013, GASTROENTEROLOGY, V145, P583, DOI 10.1053/j.gastro.2013.05.046
Wang ZJ, 2019, J GASTROEN HEPATOL, V34, P791, DOI 10.1111/jgh.14557
Yang JL, 2019, ENDOSCOPY, V51, P1035, DOI 10.1055/a-0759-1353
Zaheer A, 2013, ABDOM IMAGING, V38, P125, DOI 10.1007/s00261-012-9908-0
Zhu HY, 2018, TRIALS, V19, DOI 10.1186/s13063-018-2901-3
NR 35
TC 60

Z9 71
U1 1
U2 5
PU WILEY
PI HOBOKEN
PA 111 RIVER ST, HOBOKEN 07030-5774, NJ USA
SN 1868-6974
EI 1868-6982
J9 J HEPATO-BIL-PAN SCI
JI J. Hepato-Biliary-Pancreat. Sci.
PD FEB
PY 2022
VL 29
IS 2
BP 198
EP 211
DI 10.1002/jhbp.1008
EA JUN 2021
PG 14
WC Gastroenterology & Hepatology; Surgery
WE Science Citation Index Expanded (SCI-EXPANDED)
SC Gastroenterology & Hepatology; Surgery
GA ZL2VV
UT WOS:000667161000001
PM 34107170
OA Green Submitted
DA 2026-05-09
ER

PT J
AU Huang, P
Zhang, H
Zhang, XF
Lv, W
Lou, SM
AF Huang, Ping
Zhang, Hao
Zhang, Xiao-Feng
Lv, Wen
Lou, Songmei
TI Comparison of Endoscopic Ultrasonography Guided Biliary Drainage and
Percutaneous Transhepatic Biliary Drainage in the Management of

Malignant Obstructive Jaundice After Failed ERCP
SO SURGICAL LAPAROSCOPY ENDOSCOPY & PERCUTANEOUS TECHNIQUES
LA English
DT Article
DE malignant obstructive jaundice; endoscopic ultrasonography guided
biliary drainage; percutaneous transhepatic biliary drainage
ID BILE-DUCT OBSTRUCTION; PANCREATIC-DUCTS; CHOLEDOCHODUODENOSTOMY;
CHOLANGIOPANCREATOGRAPHY; HEPATICOGASTROSTOMY; ACCESS; INTERVENTIONS;
PREDICTORS
AB Aims:The aim of this study is to compare the efficacy and safety of endoscopic ultrasonography guided biliary drainage and percutaneous transhepatic biliary drainage in the management of malignant obstructive jaundice after failed ERCP.Methods:We retrospectively analyzed the data of 66 consecutive patients with malignant obstructive jaundice admitted to our hospital between January 2014 and January 2016. Patients were performed endoscopic ultrasonography-guided biliary drainage in 36 cases (group A) and percutaneous transhepatic biliary drainage in 30 cases (group B) according to the results of the draw. Data on the following variables were compared between the 2 groups: the technical success rate, the clinical success rate, complications, length of hospital stay, and hospital costs.Results:There was statistically significant difference in the clinical success rate (88.89% vs. 66.67%; (2)=4.84), complications (5.56% vs. 23.33%; (2)=4.39), length of hospital stay (11.543.73d vs. 15.68 +/- 6.56d; t=8.17) and hospital costs (23.52 +/- 8.44 thousand yuan vs. 32.81 +/- 6.06 thousand yuan; t=16.28) (P<0.05) between group A and group B. The technical success rate was higher in groups A than that in group B, although the difference did not reach statistical significance (94.44% vs. 86.67%; (2)=1.20; P>0.05).Conclusions:In the treatment of malignant obstructive jaundice, endoscopic ultrasonography guided biliary drainage is safer and more effective than percutaneous transhepatic biliary drainage when performed by experienced practitioners after failed ERCP. Its more widespread use is recommended.
C1 [Huang, Ping; Zhang, Xiao-Feng; Lv, Wen; Lou, Songmei] Nanjing Med Univ, Hangzhou Peoples Hosp 1, Dept Gastroenterol, Hangzhou 310006, Zhejiang, Peoples R China.
[Zhang, Hao] Hangzhou Xixi Hosp, Dept Gastroenterol, Hangzhou, Zhejiang, Peoples R China.
C3 Nanjing Medical University
RP Zhang, XF (corresponding author), Nanjing Med Univ, Hangzhou Peoples Hosp 1, Dept Gastroenterol, Hangzhou 310006, Zhejiang, Peoples R China.
EM zxf.837.@tom.com
RI Liu, Siming/HKE-1097-2023
FU Science and Technology Development Program of Hangzhou [20140733Q11]
FX Supported by Science and Technology Development Program of Hangzhou
(20140733Q11). The authors declare no conflicts of interest.
CR Artifon ELA, 2015, GASTROINTEST ENDOSC, V81, P950, DOI 10.1016/j.gie.2014.09.047
Barkay O, 2010, GASTROINTEST ENDOSC, V71, P1166, DOI 10.1016/j.gie.2009.10.048
Brauer BC, 2009, GASTROINTEST ENDOSC, V70, P471, DOI 10.1016/j.gie.2008.12.233
Dhir V, 2014, DIGEST ENDOSC, V26, P430, DOI 10.1111/den.12153
Dhir V, 2012, GASTROINTEST ENDOSC, V75, P354, DOI 10.1016/j.gie.2011.07.075
Hara K, 2013, ENDOSCOPY, V45, P392, DOI 10.1055/s-0032-1326076
Imamine R, 2015, J VASC INTERV RADIOL, V26, P1852, DOI 10.1016/j.jvir.2015.07.029
Iqbal S, 2013, GASTROENT RES PRACT, V3, P1

Iwashita T, 2012, ENDOSCOPY, V44, P60, DOI 10.1055/s-0030-1256871
Kahaleh M, 2015, WORLD J GASTROENTERO, V21, P726, DOI 10.3748/wjg.v21.i3.726
Kahaleh M, 2013, WORLD J GASTROENTERO, V19, P1372, DOI 10.3748/wjg.v19.i9.1372
Kawakubo K, 2016, ENDOSCOPY, V48, P164, DOI 10.1055/s-0034-1393179
Kawakubo K, 2013, SURG ENDOSC, V27, P3437, DOI 10.1007/s00464-013-2896-5
Khan MA, 2016, DIGEST DIS SCI, V61, P684, DOI 10.1007/s10620-015-3933-0
Khashab MA, 2016, ENDOSC INT OPEN, V4, pE487, DOI 10.1055/s-0042-102648
Khashab MA, 2016, ENDOSC INT OPEN, V4, pE175, DOI 10.1055/s-0041-109083
Khashab MA, 2015, DIGEST DIS SCI, V60, P557, DOI 10.1007/s10620-014-3300-6
Knudsen MH, 2015, UGESKRIFT LAEGER, V177, P18
Lee TH, 2016, CLIN GASTROENTEROL H, V14, P1011, DOI 10.1016/j.cgh.2015.12.032
Mallery S, 2004, GASTROINTEST ENDOSC, V59, P100, DOI 10.1016/S0016-5107(03)02300-9
Minaga K, 2016, WORLD J GASTROENTERO, V22, P4264, DOI 10.3748/wjg.v22.i16.4264
Ogura T, 2016, WORLD J GASTROENTERO, V22, P3945, DOI 10.3748/wjg.v22.i15.3945
Ogura T, 2016, ENDOSCOPY, V48, P156, DOI 10.1055/s-0034-1392859
Ogura T, 2015, J GASTROEN HEPATOL, V30, P1748, DOI 10.1111/jgh.13021
Park DH, 2011, GASTROINTEST ENDOSC, V74, P1276, DOI 10.1016/j.gie.2011.07.054
Perez-Miranda M, 2010, WORLD J GASTRO ENDOS, V2, P212, DOI 10.4253/wjge.v2.i6.212
Prachayakul V, 2016, WORLD J GASTRO ENDOS, V8, P104, DOI 10.4253/wjge.v8.i2.104
Prachayakul V, 2015, WORLD J GASTRO ENDOS, V7, P37, DOI 10.4253/wjge.v7.i1.37
Sarkaria S, 2013, GUT LIVER, V7, P129, DOI 10.5009/gnl.2013.7.2.129
Shah JN, 2012, GASTROINTEST ENDOSC, V75, P56, DOI 10.1016/j.gie.2011.08.032
Sharaiha RZ, 2016, SURG ENDOSC, V30, P5500, DOI 10.1007/s00464-016-4913-y
Shimizu H, 2015, WORLD J GASTROENTERO, V21, P12628, DOI 10.3748/wjg.v21.i44.12628
Shu JC, 2011, MED PRIN PRACT, V20, P47, DOI 10.1159/000322073
Siripun A, 2015, WORLD J GASTRO ENDOS, V7, P283, DOI 10.4253/wjge.v7.i3.283
van Delden OM, 2008, EUR RADIOL, V18, P448, DOI 10.1007/s00330-007-0796-6
Wiersema MJ, 1996, GASTROINTEST ENDOSC, V43, P102, DOI 10.1016/S0016-5107(06)80108-2
Will U, 2007, J HEPATO-BILIARY-PAN, V14, P377, DOI 10.1007/s00534-006-1139-8
Xu Chuan, 2015, Asian Pac J Cancer Prev, V16, P2543, DOI 10.7314/apjcp.2015.16.6.2543
Zhou LY, 2012, EUR RADIOL, V22, P371, DOI 10.1007/s00330-011-2265-5
NR 39
TC 17
Z9 20
U1 0
U2 6
PU LIPPINCOTT WILLIAMS & WILKINS
PI PHILADELPHIA
PA TWO COMMERCE SQ, 2001 MARKET ST, PHILADELPHIA, PA 19103 USA
SN 1530-4515
EI 1534-4908
J9 SURG LAPARO ENDO PER

JI Surg. Laparosc. Endosc. Pct. Tech.
PD DEC
PY 2017
VL 27
IS 6
BP E127
EP E131
DI 10.1097/SLE.0000000000000485
PG 5
WC Surgery
WE Science Citation Index Expanded (SCI-EXPANDED)
SC Surgery
GA FQ1VC
UT WOS:000418144700002
PM 29206804
DA 2026-05-09
ER

PT J
AU Tarrio, I
Moreira, M
Araújo, T
Lopes, L
AF Tarrio, Isabel
Moreira, Marta
Araujo, Tarcisio
Lopes, Luis
TI EUS-Guided Choledochoduodenostomy after Failed Endoscopic Retrograde Cholangiopancreatography in Distal Malignant Biliary Obstruction
SO GE PORTUGUESE JOURNAL OF GASTROENTEROLOGY
LA English
DT Article
DE Obstructive jaundice; EUS-guided biliary drainage; Choledochoduodenostomy; Lumen-apposing metal stent; Endoscopic ultrasonography
ID DRAINAGE; PALLIATION
AB Introduction: Malignant biliary obstruction drainage is essential, since jaundice is associated with morbidity and mortality. Endoscopic retrograde cholangiopancreatography (ERCP) is the recommended procedure for biliary drainage, with percutaneous biliary drainage being the classic alternative in cases of unsuccessful ERCP. Recently, endoscopic ultrasound-guided biliary drainage has been emerged as a new option, with EUS-guided choledochoduodenostomy (EUS-CDS) being considered an effective and safe method in the drainage of distal obstructions of the common bile duct. Aim: The aim of the study was to evaluate the efficacy and safety of EUS-CDS performed in patients with distal malignant biliary

obstructions, after failed ERCP. Methods: Single-center retrospective cohort study between July 2017 and June 2022 including all consecutive patients submitted to EUS-CDS in our center. The primary outcomes were "technical success" and "clinical success," defined as "resolution of jaundice or improvement in total serum bilirubin level above 50% at 7th day and above 75% at 30th day after the procedure." Secondary outcomes were procedure-related adverse events, endoscopic reintervention, and survival time. Results: EUS-CDS was performed in 20 patients (65.0% male; median age 76 years). The most frequent etiology for the biliary obstruction was pancreatic adenocarcinoma (n = 17; 85.0%), and most patients presented at advanced stages of cancer (12/60% in stages III or IV). ERCP failure was mainly due to the presence of obstruction in the duodenal lumen (n = 11; 55.0%). Fully covered metallic stents were used in all patients, mostly HotAxios (TM) (n = 15; 75.0%). The technical success rate was 100%, and the clinical success rate was 89.5% (n = 17/19) at 7th day and 93.3% (n = 14/15) at 30th day. Four patients (20.0%) developed cholangitis within the first 30 days after the procedure; there were no late complications, and no patient died as a complication of the procedure. In 2 patients (10.0%), endoscopic reintervention was necessary due to stent migration, incidentally detected. Median survival was 93 days (minimum 5-maximum 751). Conclusion: EUS-CDS was effective in biliary decompression of malignant obstructions of the common bile duct, with high clinical success and a favorable safety profile. (c) 2023 The Author(s). Published by S. Karger AG, Basel
C1 [Tarrio, Isabel; Moreira, Marta; Araujo, Tarcisio; Lopes, Luis] ULS Alto Minho, Hosp Santa Luzia, Gastroenterol Dept, Viana Do Castelo, Portugal.
[Lopes, Luis] Univ Minho, Life & Hlth Sci Res Inst ICVS, Sch Med, Braga, Portugal.
[Lopes, Luis] ICVS 3Bs PT Govt Associate Lab, Braga, Portugal.
C3 Universidade do Minho; Laboratorio Associado ICVS 3B's
RP Tarrio, I (corresponding author), ULS Alto Minho, Hosp Santa Luzia, Gastroenterol Dept, Viana Do Castelo, Portugal.
EM isatarrio@gmail.com
RI ; Lopes, Luís/D-6238-2016
OI Araújo, Tarcísio/0000-0002-4901-8581; Lopes, Luís/0000-0002-8835-0923;
Moreira, Marta/0000-0002-3907-3165
CR Anderloni A, 2019, GASTROINTEST ENDOSC, V89, P69, DOI [10.1016/j.gie.2018.04.1746, 10.1016/j.gie.2018.08.047]
Canena J, 2021, SCI REP-UK, V11, DOI 10.1038/s41598-021-96142-9
Cotton PB, 2010, GASTROINTEST ENDOSC, V71, P446, DOI 10.1016/j.gie.2009.10.027
Garcia-Sumalla Albert, 2021, VideoGIE, V6, P263, DOI [10.1016/j.vgie.2021.03.002, 10.1016/j.vgie.2021.03.002]
Giovannini M, 2001, ENDOSCOPY, V33, P898, DOI 10.1055/s-2001-17324
Hathorn KE, 2019, ENDOSC INT OPEN, V7, pE1432, DOI 10.1055/a-0990-9488
Khan MA, 2016, DIGEST DIS SCI, V61, P684, DOI 10.1007/s10620-015-3933-0
Khashab MA, 2015, DIGEST DIS SCI, V60, P557, DOI 10.1007/s10620-014-3300-6
Lesmana CRA, 2021, WORLD J GASTRO SURG, V13, P537, DOI 10.4240/wjgs.v13.i6.537
Li JS, 2022, FRONT SURG, V9, DOI 10.3389/fsurg.2022.811005
Matsumoto S, 2020, DIGEST ENDOSC, V32, P957, DOI 10.1111/den.13620
Nennstiel S, 2015, J CLIN GASTROENTEROL, V49, P764, DOI 10.1097/MCG.0000000000000275
Ogura T, 2021, DEN OPEN, V1, DOI 10.1002/deo2.8
Peng ZX, 2021, CAN J GASTROENTEROL, V2021, DOI 10.1155/2021/6696950
Rai P, 2018, ENDOSC INT OPEN, V6, pE67, DOI 10.1055/s-0043-120664
Sassatelli R, 2020, DIGEST LIVER DIS, V52, P57, DOI 10.1016/j.dld.2019.07.009

Sharaiha RZ, 2017, GASTROINTEST ENDOSC, V85, P904, DOI 10.1016/j.gie.2016.12.023
Stark A, 2015, SEMIN ONCOL, V42, P163, DOI 10.1053/j.seminoncol.2014.12.014
Tanisaka Y, 2021, J CLIN MED, V10, DOI 10.3390/jcm10194619
van der Merwe SW, 2022, ENDOSCOPY, V54, P185, DOI 10.1055/a-1717-1391
VANDENBOSCH RP, 1994, ANN SURG, V219, P18, DOI 10.1097/00000658-199401000-00004
Viesca MFY, 2019, CLIN EXP GASTROENTER, V12, P415, DOI 10.2147/CEG.S195714
Wang KX, 2016, GASTROINTEST ENDOSC, V83, P1218, DOI 10.1016/j.gie.2015.10.033
World Med Assoc, 2013, JAMA-J AM MED ASSOC, V310, P2191, DOI 10.1001/jama.2013.281053
NR 24
TC 2
Z9 2
U1 1
U2 2
PU KARGER
PI BASEL
PA ALLSCHWILERSTRASSE 10, CH-4009 BASEL, SWITZERLAND
SN 2341-4545
EI 2387-1954
J9 GE PORT J GASTROENT
JI GE Port. J. Gastroenterol.
PD SEP
PY 2023
VL 30
IS SUPPL 1
SU 1
BP 65
EP 73
DI 10.1159/000528808
EA MAR 2023
PG 9
WC Gastroenterology & Hepatology
WE Emerging Sources Citation Index (ESCI)
SC Gastroenterology & Hepatology
GA R6TT3
UT WOS:000949205100001
PM 37818398
OA Green Submitted, gold
DA 2026-05-09
ER

PT J
AU Irani, SS

Sharma, NR
Storm, AC
Shah, RJ
Chahal, P
Willingham, FF
Swanstrom, L
Baron, TH
Shlomovitz, E
Kozarek, RA
Peetermans, JA
McMullen, E
Ho, EVLY
van der Merwe, SW
AF Irani, Shayan S.
Sharma, Neil R.
Storm, Andrew C.
Shah, Raj J.
Chahal, Prabhleen
Willingham, Field F.
Swanstrom, Lee
Baron, Todd H.
Shlomovitz, Eran
Kozarek, Richard A.
Peetermans, Joyce A.
McMullen, Edmund
Ho, Evelyne
van der Merwe, Schalk W.
TI Endoscopic Ultrasound-guided Transluminal Gallbladder Drainage in
Patients With Acute Cholecystitis A Prospective Multicenter Trial
SO ANNALS OF SURGERY
LA English
DT Article
DE acute cholecystitis; clinical study; drainage; gallbladder; metal stents
ID APPOSING METAL STENT; PERCUTANEOUS CHOLECYSTOSTOMY; CHOLANGIOSCOPY;
MANAGEMENT; TIME
AB Objective:To evaluate the safety and efficacy of endoscopic ultrasound-guided gallbladder drainage (EUS-GBD) using a lumen-apposing metal stent (LAMS). Background:For patients with acute cholecystitis who are poor surgical candidates, EUS-GBD using a LAMS is an important treatment alternative to percutaneous gallbladder drainage. Methods:We conducted a regulatory-compliant, prospective multicenter trial at 7 tertiary referral centers in the United States of America and Belgium. Thirty consecutive patients with mild or moderate acute cholecystitis who were not candidates for cholecystectomy were enrolled between September 2019 and August 2021. Eligible patients had a LAMS placed transmurally with 30 to 60-day indwell if removal was clinically indicated, and 30-day follow-up post-LAMS removal.

Endpoints included days until acute cholecystitis resolution, reintervention rate, acute cholecystitis recurrence rate, and procedure-related adverse events (AEs). Results:Technical success was 93.3% (28/30) for LAMS placement and 100% for LAMS removal in 19 patients for whom removal was attempted. Five (16.7%) patients required reintervention. Mean time to acute cholecystitis resolution was 1.6 & PLUSMN;1.5 days. Acute cholecystitis symptoms recurred in 10.0% (3/30) after LAMS removal. Five (16.7%) patients died from unrelated causes. Procedure-related AEs were reported to the FDA in 30.0% (9/30) of patients, including one fatal event 21 days after LAMS removal; however, no AEs were causally related to the LAMS. Conclusions:For selected patients with acute cholecystitis who are at elevated surgical risk, EUS-GBD with LAMS is an alternative to percutaneous gallbladder drainage. It has high technical and clinical success, with low recurrence and an acceptable AE rate. Clinicaltrials.gov, Number: NCT03767881.
C1 [Irani, Shayan S.; Kozarek, Richard A.] Virginia Mason Med Ctr, Div Gastroenterol, Seattle, WA 98101 USA.
[Sharma, Neil R.] Parkview Canc Inst, Div Intervent Oncol & Surg Endoscopy IOSE, Ft Wayne, IN USA.
[Storm, Andrew C.] Mayo Clin, Div Gastroenterol & Hepatol, Rochester, MN USA.
[Shah, Raj J.] Univ Colorado, Div Gastroenterol & Hepatol, Anschutz Med Campus, Aurora, CO USA.
[Chahal, Prabhleen] Cleveland Clin, Div Gastroenterol Hepatol & Nutr, Cleveland, OH USA.
[Willingham, Field F.] Emory Univ, Dept Med, Div Digest Dis, Atlanta, GA USA.
[Swanstrom, Lee] IHU Strasbourg, Inst Image Guided Surg, Strasbourg, France.
[Baron, Todd H.] Univ N Carolina, Div Gastroenterol & Hepatol, Adv Therapeut Endoscopy, Chapel Hill, NC USA.
[Shlomovitz, Eran] Univ Toronto, Dept Intervent Radiol, Toronto, ON, Canada.
[Peetermans, Joyce A.; McMullen, Edmund; Ho, Evelyne] Boston Sci Corp, Endoscopy Div, Marlborough, MA USA.
[van der Merwe, Schalk W.] UZ Leuven, Div Gastroenterol & Hepatol, Leuven, Belgium.
C3 Virginia Mason Medical Center; Mayo Clinic; University of Colorado System; University of Colorado Anschutz; Cleveland Clinic Foundation; Emory University; Universites de Strasbourg Etablissements Associes; Universite de Strasbourg; University of North Carolina; University of North Carolina Chapel Hill; University of Toronto; Boston Scientific; KU Leuven; University Hospital Leuven
RP Irani, SS (corresponding author), Virginia Mason Med Ctr, Div Gastroenterol, Seattle, WA 98101 USA.
EM shayan.irani@virginiamason.org; nrsharma219@yahoo.com; Storm.Andrew@mayo.edu; raj.shah@ucdenver.edu; chahalp@ccf.org; field.willingham@emory.edu; lswanstrom@gmail.com; todd_baron@med.unc.edu; eran.shlomovitz@uhn.ca; richard.kozarek@virginiamason.org; Joyce.Peetermans@bsci.com; Edmund.McMullen@bsci.com; Evelyne.Ho@bsci.com; schalk.vandermerwe@uzleuven.be
RI Kozarek, Richard/AAF-2695-2020
OI Kozarek, Richard/0000-0002-9745-7538; Van der Merwe, Schalk/0000-0002-9891-2686
FU Boston Scientific Corporation
FX Grant supported by Boston Scientific Corporation.
CR Barakat MT, 2018, GASTROINTEST ENDOSC, V87, P584, DOI 10.1016/j.gie.2017.07.042
Baron TH, 2007, GASTROINTEST ENDOSC, V65, P735, DOI 10.1016/j.gie.2006.07.041
ELYADERANI M, 1979, RADIOLOGY, V130, P601, DOI 10.1148/130.3.601

FRIEDMAN GD, 1993, AM J SURG, V165, P399, DOI 10.1016/S0002-9610(05)80930-4
GLENN F, 1977, ANN SURG, V185, P185, DOI 10.1097/00000658-197702000-00009
Inoue T, 2021, J CLIN MED, V10, DOI 10.3390/jcm10214842
Irani S, 2017, CLIN GASTROENTEROL H, V15, P738, DOI 10.1016/j.cgh.2016.12.021
Itoi T, 2012, GASTROINTEST ENDOSC, V75, P870, DOI 10.1016/j.gie.2011.10.020
Itoi T, 2010, GASTROINTEST ENDOSC, V71, P1038, DOI 10.1016/j.gie.2010.01.026
Jain D, 2018, CLIN ENDOSC, V51, P450, DOI 10.5946/ce.2018.024
Kaura K, 2020, HPB, V22, P996, DOI 10.1016/j.hpb.2019.10.1530
Kedia P, 2015, GASTROINTEST ENDOSC, V82, P1031, DOI 10.1016/j.gie.2015.03.1912
KOZAREK RA, 1984, J CLIN GASTROENTEROL, V6, P37
Kwan V, 2007, GASTROINTEST ENDOSC, V66, P582, DOI 10.1016/j.gie.2007.02.065
Litynski G S, 1999, JSLS, V3, P163
MENDEZ A, 1994, SEMIN INTERVENT RAD, V11, P283, DOI 10.1055/s-2008-1074766
Mohan BP, 2020, ENDOSCOPY, V52, P96, DOI 10.1055/a-1020-3932
Ostapenko A, 2022, J GASTROINTEST SURG, V26, P602, DOI 10.1007/s11605-021-04996-1
Podboy A, 2021, GASTROINTEST ENDOSC, V93, P797, DOI 10.1016/j.gie.2020.09.040
Posner H, 2020, TRANSL GASTROENT HEP, V5, DOI 10.21037/tgh.2019.12.20
Pucher PH, 2018, SURG ENDOSC, V32, P2175, DOI 10.1007/s00464-017-5974-2
Sejpal DV, 2019, ENDOSC INT OPEN, V7, pE608, DOI 10.1055/a-0842-6450
Siddiqui A, 2019, SURG ENDOSC, V33, P1260, DOI 10.1007/s00464-018-6406-7
Sobani ZA, 2021, DIGEST DIS SCI, V66, P2154, DOI 10.1007/s10620-020-06520-y
Storm Andrew C, 2021, Gastrointest Endosc, V94, P742, DOI 10.1016/j.gie.2021.03.025
Teoh AYB, 2020, GUT, V69, P1085, DOI 10.1136/gutjnl-2019-319996
Teoh AYB, 2017, ENDOSCOPY, V49, P130, DOI 10.1055/s-0042-119036
Teoh AYB, 2014, GASTROINTEST ENDOSC, V80, P1171, DOI 10.1016/j.gie.2014.03.038
Tyberg A, 2020, ENDOSC INT OPEN, V8, pE92, DOI 10.1055/a-1005-6602
Tyberg A, 2018, J CLIN GASTROENTEROL, V52, P79, DOI 10.1097/MCG.0000000000000786
van Wanrooij RLJ, 2022, ENDOSCOPY, V54, P310, DOI 10.1055/a-1738-6780
Walter D, 2016, GUT, V65, P6, DOI 10.1136/gutjnl-2015-309925
Yang JJ, 2019, BMC GASTROENTEROL, V19, DOI 10.1186/s12876-019-1045-6
Yokoe M, 2013, J HEPATO-BIL-PAN SCI, V20, P35, DOI 10.1007/s00534-012-0568-9
NR 34
TC 29
Z9 34
U1 0
U2 3
PU LIPPINCOTT WILLIAMS & WILKINS
PI PHILADELPHIA
PA TWO COMMERCE SQ, 2001 MARKET ST, PHILADELPHIA, PA 19103 USA
SN 0003-4932
EI 1528-1140
J9 ANN SURG

JI Ann. Surg.
PD SEP
PY 2023
VL 278
IS 3
BP E556
EP E562
DI 10.1097/SLA.0000000000005784
PG 7
WC Surgery
WE Science Citation Index Expanded (SCI-EXPANDED)
SC Surgery
GA O9TW8
UT WOS:001047180100037
PM 36537290
OA Green Submitted, hybrid
DA 2026-05-09
ER

PT J
AU Kahaleh, M
Artifon, ELA
Perez-Miranda, M
Gupta, K
Itoi, T
Binmoeller, KF
Giovannini, M
AF Kahaleh, Michel
Artifon, Everson L. A.
Perez-Miranda, Manuel
Gupta, Kapil
Itoi, Takao
Binmoeller, Kenneth F.
Giovannini, Marc
TI Endoscopic ultrasonography guided biliary drainage: Summary of consortium meeting, May 7th, 2011, Chicago
SO WORLD JOURNAL OF GASTROENTEROLOGY
LA English
DT Review
DE Endoscopic ultrasound; Biliary drainage; Endosonography-guided cholangiopancreatography; Endoscopic ultrasound guided; Pancreatic drainage; Endoscopic retrograde cholangiopancreatography

ID PANCREATIC-DUCT DRAINAGE; OF-THE-ART; TRANSHEPATIC CHOLANGIOGRAPHY; CASE SERIES; BILE-DUCT; EUS; ULTRASOUND; OBSTRUCTION; CHOLEDOCHODUODENOSTOMY; ERCP

AB Endoscopic retrograde cholangiopancreatography (ERCP) has become the preferred procedure for biliary or pancreatic drainage in various pancreatico-biliary disorders. With a success rate of more than 90%, ERCP may not achieve biliary or pancreatic drainage in cases with altered anatomy or with tumors obstructing access to the duodenum. In the past those failures were typically managed exclusively by percutaneous approaches by interventional radiologists or surgical intervention. The morbidity associated was significant especially in those patients with advanced malignancy, seeking minimally invasive interventions and improved quality of life. With the advent of biliary drainage via endoscopic ultrasound (EUS) guidance, EUS guided biliary drainage has been used more frequently within the last decade in different countries. As with any novel advanced endoscopic procedure that encompasses various approaches, advanced endoscopists all over the world have innovated and adopted diverse EUS guided biliary and pancreatic drainage techniques. This diversity has resulted in variations and improvements in EUS Guided biliary and pancreatic drainage; and over the years has led to an extensive nomenclature. The diversity of techniques, nomenclature and recent progress in our intrumentation has led to a dedicated meeting on May 7th, 2011 during Digestive Disease Week 2011. More than 40 advanced endoscopists from United States, Brazil, Mexico, Venezuela, Colombia, Italy, France, Austria, Germany, Spain, Japan, China, South Korea and India attended this pivotal meeting. The meeting covered improved EUS guided biliary access and drainage procedures, terminology, nomenclature, training and credentialing; as well as emerging devices for EUS guided biliary drainage. This paper summarizes the meeting's agenda and the conclusions generated by the creation of this consortium group. (C) 2013 Baishideng. All rights reserved.

C1 [Kahaleh, Michel] Weill Cornell Med Coll, Div Gastroenterol & Hepatol, New York, NY 10021 USA.
[Artifon, Everson L. A.] Univ Sao Paulo, Dept Gastroenterol, Sch Med, BR-05403000 Sao Paulo, Brazil.
[Perez-Miranda, Manuel] Hosp Univ Rio Hortega, Endoscopy Unit, Valladolid 47012, Spain.
[Gupta, Kapil] Cedars Sinai Med Ctr, Pancreat Biliary Dept, Los Angeles, CA 90048 USA.
[Itoi, Takao] Tokyo Med Univ, Dept Gastroenterol & Hepatol, Tokyo 1600023, Japan.
[Binmoeller, Kenneth F.] Calif Pacific Med Ctr, San Francisco, CA 94115 USA.
[Giovannini, Marc] Paolicalmettes Inst, Dept Gastroenterol, F-13273 Marseille, France.
C3 Cornell University; Weill Cornell Medicine; Universidade de Sao Paulo; Hospital del Rio Hortega; Cedars Sinai Medical Center; Tokyo Medical University; California Pacific Medical Center
RP Kahaleh, M (corresponding author), Weill Cornell Med Coll, Div Gastroenterol & Hepatol, 1305 York Ave,4th Floor, New York, NY 10021 USA.
EM mkahaleh@gmail.com
RI Gupta, Kapil/OHU-4277-2025; Kahaleh, Michel/A-9408-2011
OI Kahaleh, Michel/0000-0003-0836-6114

CR Ang TL, 2007, J PANCREAS, V8, P438
[Anonymous], 2006, GASTROINTEST ENDOSC, V63, P361, DOI 10.1016/j.gie.2006.01.010
Artifon ELA, 2007, CLINICS, V62, P799, DOI 10.1590/S1807-59322007000600023
Bataille L, 2002, GASTROINTEST ENDOSC, V55, P740, DOI 10.1067/mge.2002.123621
Binmoeller KF, 2011, ENDOSCOPY, V43, P337, DOI 10.1055/s-0030-1256127
Binmoeller KF, 2010, GASTROINTEST ENDOSC, V71, pAB349, DOI 10.1016/j.gie.2010.03.932
Bories E, 2007, ENDOSCOPY, V39, P287, DOI 10.1055/s-2007-966212

Brauer BC, 2009, GASTROINTEST ENDOSC, V70, P471, DOI 10.1016/j.gie.2008.12.233
Burmester E, 2003, GASTROINTEST ENDOSC, V57, P246, DOI 10.1067/mge.2003.85
Chang KJ, 2011, GASTROINTEST ENDOSC, V73, pAB326
DeWitt J, 2004, GASTROINTEST ENDOSC, V59, P133, DOI 10.1016/S0016-5107(03)02278-8
Eisen GM, 2001, GASTROINTEST ENDOSC, V54, P811, DOI 10.1016/S0016-5107(01)70082-X
François E, 2002, GASTROINTEST ENDOSC, V56, P128, DOI 10.1067/mge.2002.125547
Fujita N, 2007, WORLD J GASTROENTERO, V13, P5512, DOI 10.3748/wjg.v13.i41.5512
Giovannini M, 2003, ENDOSCOPY, V35, P1076, DOI 10.1055/s-2003-44596
Giovannini M, 2001, ENDOSCOPY, V33, P898, DOI 10.1055/s-2001-17324
Gupta K, 2007, REV GASTROENTEROL DI, V7, P22
Itoi T, 2008, WORLD J GASTROENTERO, V14, P6078, DOI 10.3748/wjg.14.6078
Itoi T, 2011, DIGEST ENDOSC, V23, P17, DOI 10.1111/j.1443-1661.2011.01132.x
Jamidar P, 2009, GASTROINTEST ENDOSC, V69, P1333, DOI 10.1016/j.gie.2008.09.061
Kahaleh M, 2006, GASTROINTEST ENDOSC, V64, P52, DOI 10.1016/j.gie.2006.01.063
Kahaleh M, 2004, GASTROINTEST ENDOSC, V60, P138, DOI 10.1016/S0016-5107(04)01528-7
Kahaleh M, 2005, GASTROINTEST ENDOSC, V61, P307, DOI 10.1016/S0016-5107(04)02585-4
Kahaleh M, 2003, GASTROINTEST ENDOSC, V58, P919, DOI 10.1016/S0016-5107(03)02297-1
Kim YS, 2010, ENDOSCOPY, V42, P496, DOI 10.1055/s-0029-1244082
Lai R, 2005, ENDOSCOPY, V37, P487, DOI 10.1055/s-2005-861250
Littenberg G, 2009, CODING PRIMER GUIDE, P1
Mallery S, 2004, GASTROINTEST ENDOSC, V59, P100, DOI 10.1016/S0016-5107(03)02300-9
Maranki J, 2009, ENDOSCOPY, V41, P532, DOI 10.1055/s-0029-1214712
Nguyen-Tang T, 2010, ENDOSCOPY, V42, P232, DOI 10.1055/s-0029-1243858
Palazzo L, 2009, GASTROINTEST ENDOSC, V69, pS220, DOI 10.1016/j.gie.2008.12.034
Perez-Miranda M, 2010, WORLD J GASTRO ENDOS, V2, P212, DOI 10.4253/wjge.v2.i6.212
Püspök A, 2005, AM J GASTROENTEROL, V100, P1743, DOI 10.1111/j.1572-0241.2005.41806.x
Talreja JP, 2008, GASTROINTEST ENDOSC, V68, P1199, DOI 10.1016/j.gie.2008.06.015
Tarantino I, 2008, ENDOSCOPY, V40, P336, DOI 10.1055/s-2007-995455
Will U, 2007, ENDOSCOPY, V39, P292, DOI 10.1055/s-2007-966215
Will U, 2005, ENDOSCOPY, V37, P171, DOI 10.1055/s-2004-826151
Yamao K, 2008, ENDOSCOPY, V40, P340, DOI 10.1055/s-2007-995485
Yamao K, 2006, GASTROINTEST ENDOSC, V64, P663, DOI 10.1016/j.gie.2006.07.003
Yasuda K, 2009, GASTROINTEST ENDOSC, V69, pS226, DOI 10.1016/j.gie.2008.12.033
NR 40
TC 60
Z9 84
U1 0
U2 3
PU BAISHIDENG PUBLISHING GROUP INC
PI PLEASANTON
PA 8226 REGENCY DR, PLEASANTON, CA 94588 USA
SN 1007-9327

EI 2219-2840
J9 WORLD J GASTROENTERO
JI World J. Gastroenterol.
PD MAR 7
PY 2013
VL 19
IS 9
BP 1372
EP 1379
DI 10.3748/wjg.v19.i9.1372
PG 8
WC Gastroenterology & Hepatology
WE Science Citation Index Expanded (SCI-EXPANDED)
SC Gastroenterology & Hepatology
GA 104NT
UT WOS:000316001700007
PM 23538784
OA hybrid
DA 2026-05-09
ER

PT J
AU Lu, L
Jin, HB
Yang, JF
Zhang, XF
AF Lu, Lei
Jin, Hang-Bin
Yang, Jian-Feng
Zhang, Xiao-Feng
TI Endoscopic ultrasound-guided pancreaticogastrostomy for symptomatic
pancreatic duct obstruction caused by migrated pancreatic stent
SO WORLD JOURNAL OF GASTROINTESTINAL ENDOSCOPY
LA English
DT Article
DE Endoscopic retrograde cholangiopancreatography; Pancreatic stent; Stent
migration; Pancreatic duct obstruction; Endoscopic ultrasound-guided
pancreatic duct drainage
ID DRAINAGE; EUS; INTERVENTION; OUTCOMES; BILIARY
AB Endoscopic pancreatic stenting has been widely used in various pancreatic conditions. With the increasing use of pancreatic stents, many complications have been recognized. Especially, proximal stent migration presents a serious condition because of subsequent pancreatic duct obstruction, impaired drainage, ductal dilation, and pancreatic pain.

Although endoscopic retrieval is the preferred treatment for proximally migrated pancreatic stents, it is not always successful, resulting in conversion to surgery. To date, endoscopic ultrasound-guided pancreatic duct drainage (EUS-PD) has never been reported for treatment of pancreatic duct obstruction caused by proximally migrated pancreatic stent. We herein describe a case of pancreatic duct rupture and obstruction caused by proximally migrated pancreatic stent that was successfully treated by EUS-guided pancreaticogastrostomy while keeping the former stent in situ after failed endoscopic retrograde cholangiopancreatography. We believe that this report adds to the increasing evidence of symptomatic pancreatic duct obstruction being successfully treated by EUS-PD.
C1 [Lu, Lei; Jin, Hang-Bin; Yang, Jian-Feng; Zhang, Xiao-Feng] Nanjing Med Univ, Hangzhou Peoples Hosp 1, Dept Gastroenterol, Hangzhou 310000, Zhejiang, Peoples R China.
C3 Nanjing Medical University
RP Zhang, XF (corresponding author), Nanjing Med Univ, Hangzhou Peoples Hosp 1, Dept Gastroenterol, Hangzhou 310000, Zhejiang, Peoples R China.
EM zxf837@tom.com
CR Bataille L, 2002, GASTROINTEST ENDOSC, V55, P740, DOI 10.1067/mge.2002.123621
Binmoeller KF, 1995, ENDOSCOPY, V27, P638, DOI 10.1055/s-2007-1005780
Dhir V, 2017, DIGEST ENDOSC, V29, P472, DOI 10.1111/den.12818
Ergun M, 2011, ENDOSCOPY, V43, P518, DOI 10.1055/s-0030-1256333
Freeman ML, 2004, GASTROINTEST ENDOSC, V59, P845, DOI 10.1016/S0016-5107(04)00353-0
Fujii LL, 2013, GASTROINTEST ENDOSC, V78, P854, DOI 10.1016/j.gie.2013.05.016
Giovannini M, 2013, GASTROINTEST ENDOSC, V78, P865, DOI 10.1016/j.gie.2013.10.019
Itoi T, 2014, J HEPATO-BIL-PAN SCI, V21, pE4, DOI 10.1002/jhbp.43
JOHANSON JF, 1992, GASTROINTEST ENDOSC, V38, P341, DOI 10.1016/S0016-5107(92)70429-5
Kahaleh M, 2015, WORLD J GASTROENTERO, V21, P726, DOI 10.3748/wjg.v21.i3.726
Matsumoto K, 2014, J HEPATO-BIL-PAN SCI, V21, pE34, DOI 10.1002/jhbp.94
Oh D, 2016, GASTROINTEST ENDOSC, V83, P366, DOI 10.1016/j.gie.2015.07.015
Tessier G, 2007, GASTROINTEST ENDOSC, V65, P233, DOI 10.1016/j.gie.2006.06.029
NR 13
TC 2
Z9 2
U1 0
U2 5
PU BAISHIDENG PUBLISHING GROUP INC
PI PLEASANTON
PA 8226 REGENCY DR, PLEASANTON, CA 94588 USA
SN 1948-5190
J9 WORLD J GASTRO ENDOS
JI World J. Gastrointest. Endosc.
PD OCT 16
PY 2017
VL 9
IS 10
BP 535

EP 539
DI 10.4253/wjge.v9.i10.535
PG 5
WC Gastroenterology & Hepatology
WE Emerging Sources Citation Index (ESCI)
SC Gastroenterology & Hepatology
GA FK2WT
UT WOS:000413344500006
PM 29085565
OA hybrid
DA 2026-05-09
ER

PT J
AU Poincloux, L
Rouquette, O
Buc, E
Privat, J
Pezet, D
Dapoigny, M
Bommelaer, G
Abergel, A
AF Poincloux, Laurent
Rouquette, Olivier
Buc, Emmanuel
Privat, Jocelyn
Pezet, Denis
Dapoigny, Michel
Bommelaer, Gilles
Abergel, Armando
TI Endoscopic ultrasound-guided biliary drainage after failed ERCP:
cumulative experience of 101 procedures at a single center
SO ENDOSCOPY
LA English
DT Article
ID INTERVENTIONAL EUS; OBSTRUCTION; CHOLANGIOPANCREATOGRAPHY;
CHOLANGIOGRAPHY; PLACEMENT
AB Background and study aim: Endoscopic ultrasound (EUS)-guided biliary access is an alternative to percutaneous access after failed endoscopic retrograde cholangiopancreatography (ERCP). This report presents 7 years' cumulative experience of EUS-guided biliary drainage for obstructive jaundice in patients with failed ERCP.

Patients and methods: Between February 2006 and February 2013, 101 patients (malignant=98, benign=3) with previous failed ERCP underwent an EUS intra-or extrahepatic approach with transluminal stenting or an EUS-guided rendezvous

procedure with transpapillary stent placement. A single endoscopist performed all procedures.

Results: A total of 71 patients underwent the intrahepatic approach (66 hepatogastrostomies and 5 EUS-guided rendezvous), and 30 underwent the extrahepatic approach (26 choledochoduodenostomies, 1 choledochojejunostomy, 1 choledochoantrostomy, and 2 EUS-guided cholangiographies). Technical and clinical success rates were 98.0% and 92.1 %, respectively. There was no difference in efficacy between hepatogastrostomies and choledochoduodenostomies (94% vs. 90%; P=0.69) or in major complications (10.6% vs. 6.7 %; P=1). Adverse events occurred in 12 patients (11.9 %): 10 in the hepatogastrostomy group (2 limited pneumoperitoneum, 1 hepatic hematoma, 5 bile leakage, 2 sepsis), and 2 in the choledochoduodenostomy group (1 arteriobiliary fistula and 1 sepsis). There were six procedurerelated deaths, five among the first 50 patients and one among the last 51 patients. Hepatogastrostomy vs. choledochoduodenostomy, plastic vs. metal stenting, stent-in-stent vs. 1 stent, nasobiliary drain, or postoperative octreotide infusion were not prognostic of bile leakage.

Conclusion: EUS-guided biliary drainage is an efficient technique, but is associated with significant morbidity that seems to decrease with the learning curve. It should be performed in tertiary care centers in selected patients. Prospective randomized studies are needed to compare EUS-guided biliary drainage with percutaneous transhepatic cholangiography drainage.
C1 [Poincloux, Laurent; Rouquette, Olivier; Privat, Jocelyn; Dapoigny, Michel; Bommelaer, Gilles; Abergel, Armando] CHU Estaing Clermont Ferrand, Dept Digest & Hepatobiliary Dis, F-63003 Clermont Ferrand, France.

[Poincloux, Laurent; Abergel, Armando] Univ Auvergne, UMR CNRS 6284, Image Sci Innovat Tech, Clermont Ferrand, France.

[Buc, Emmanuel; Pezet, Denis] CHU Estaing Clermont Ferrand, Dept Digest Surg, F-63003 Clermont Ferrand, France.

C3 Universite Clermont Auvergne (UCA); CHU Clermont Ferrand; Universite
Clermont Auvergne (UCA); Centre National de la Recherche Scientifique
(CNRS); Universite Clermont Auvergne (UCA); CHU Clermont Ferrand

RP Poincloux, L (corresponding author), CHU Estaing Clermont Ferrand, Dept Digest & Hepatobiliary Dis, 1 Pl Lucie & Raymond Aubrac, F-63003 Clermont Ferrand, France.

EM lpoincloux@chu-clermontferrand.fr

RI DAPOIGNY, Michel/M-1285-2015; Pezet, Denis/I-4423-2017; Buc,
Emmanuel/JEP-6444-2023
CR Bories E, 2007, ENDOSCOPY, V39, P287, DOI 10.1055/s-2007-966212
Fogel EL, 1998, GASTROINTEST ENDOSC, V47, P396, DOI 10.1016/S0016-5107(98)70226-3
Giovannini M, 2006, ENDOSCOPY, V38, pS93, DOI 10.1055/s-2006-946665
Giovannini M, 2003, ENDOSCOPY, V35, P1076, DOI 10.1055/s-2003-44596
Giovannini M, 2001, ENDOSCOPY, V33, P898, DOI 10.1055/s-2001-17324
Giovannini M, 2012, GASTROENT RES PRACT, V2012
Iwashita T, 2012, ENDOSCOPY, V44, P60, DOI 10.1055/s-0030-1256871
Iwashita T, 2014, CLIN J GASTROENTEROL, V7, P94, DOI 10.1007/s12328-014-0467-5
Kahaleh M, 2006, GASTROINTEST ENDOSC, V64, P52, DOI 10.1016/j.gie.2006.01.063
Kawakubo K, 2014, J HEPATO-BIL-PAN SCI, V21, P328, DOI 10.1002/jhbp.27
Khashab MA, 2013, GASTROINTEST ENDOSC, V78, P734, DOI 10.1016/j.gie.2013.05.013
Khashab MA, 2012, GASTROINTEST ENDOSC, V76, P921, DOI 10.1016/j.gie.2012.05.018
Kim YS, 2010, ENDOSCOPY, V42, P496, DOI 10.1055/s-0029-1244082
LAI ECS, 1992, SURGERY, V112, P891
Mallery S, 2004, GASTROINTEST ENDOSC, V59, P100, DOI 10.1016/S0016-5107(03)02300-9

Maranki J, 2009, ENDOSCOPY, V41, P532, DOI 10.1055/s-0029-1214712
Moss AC, 2006, COCHRANE DB SYST REV, DOI 10.1002/14651858.CD004200.pub4
Nguyen-Tang T, 2010, ENDOSCOPY, V42, P232, DOI 10.1055/s-0029-1243858
Park DH, 2013, GASTROINTEST ENDOSC, V78, P91, DOI 10.1016/j.gie.2013.01.042
Park DH, 2011, GASTROINTEST ENDOSC, V74, P1276, DOI 10.1016/j.gie.2011.07.054
Püspök A, 2005, AM J GASTROENTEROL, V100, P1743, DOI 10.1111/j.1572-0241.2005.41806.x
Seifert H, 2001, ENDOSCOPY, V33, P1022, DOI 10.1055/s-2001-18927
Shah JN, 2012, GASTROINTEST ENDOSC, V75, P56, DOI 10.1016/j.gie.2011.08.032
Tessier G, 2007, GASTROINTEST ENDOSC, V65, P233, DOI 10.1016/j.gie.2006.06.029
Vila JJ, 2012, GASTROINTEST ENDOSC, V76, P1133, DOI 10.1016/j.gie.2012.08.001
Wiersema MJ, 1996, GASTROINTEST ENDOSC, V43, P102, DOI 10.1016/S0016-5107(06)80108-2
Will U, 2007, ENDOSCOPY, V39, P292, DOI 10.1055/s-2007-966215
Will U, 2015, ULTRASCHALL MED, V36, P276, DOI 10.1055/s-0034-1366557
Winick A B, 2001, Tech Vasc Interv Radiol, V4, P200, DOI 10.1016/S1089-2516(01)90026-5
Wright BE, 2002, GASTROINTEST ENDOSC, V56, P225, DOI 10.1067/mge.2002.126136
NR 30
TC 163
Z9 179
U1 0
U2 5
PU GEORG THIEME VERLAG KG
PI STUTTGART
PA RUDIGERSTR 14, D-70469 STUTTGART, GERMANY
SN 0013-726X
EI 1438-8812
J9 ENDOSCOPY
JI Endoscopy
PD SEP
PY 2015
VL 47
IS 9
BP 794
EP 801
DI 10.1055/s-0034-1391988
PG 8
WC Gastroenterology & Hepatology; Surgery
WE Science Citation Index Expanded (SCI-EXPANDED)
SC Gastroenterology & Hepatology; Surgery
GA CQ0GM
UT WOS:000360273400005
PM 25961443
DA 2026-05-09

ER

PT J
AU Chan, JHY
Teoh, AYB
AF Chan, Joey Ho Yi
Teoh, Anthony Yuen Bun
TI Current Status of Endoscopic Gallbladder Drainage
SO CLINICAL ENDOSCOPY
LA English
DT Review
DE Endoscopic gallbladder drainage; Endoscopic ultrasound-guided
gallbladder drainage; Percutaneous cholecystostomy; Cholecystitis;
acute; Interventional endoscopic ultrasonography
ID APPOSING METAL STENT; CONFOCAL LASER ENDOMICROSCOPY; ACUTE
CHOLECYSTITIS; PERCUTANEOUS CHOLECYSTOSTOMY; CHOLECYSTECTOMY; VIDEO;
FEASIBILITY; DISEASES; OUTCOMES; SAFETY
AB The gold standard for treatment of acute cholecystitis is laparoscopic cholecystectomy. However, cholecystectomy is often not suitable for surgically unfit patients who are too frail due to various co-morbidities. As such, several less invasive endoscopic treatment modalities have been developed to control sepsis, either as a definitive treatment or as a temporizing modality until the patient is stable enough to undergo cholecystectomy at a later stage. Recent developments in endoscopic ultrasound-guided gallbladder drainage (EUS-GBD) with EUS-specific stents having lumen-apposing properties have demonstrated potential as a definitive treatment modality. Furthermore, advanced gallbladder procedures can be performed using the stents as a portal. With similar effectiveness as percutaneous transhepatic cholecystostomy and lower rates of adverse events reported in some studies, EUS-GBD has opened exciting possibilities in becoming the next best alternative in treating acute cholecystitis in surgically unfit patients. The aim of this review article is to provide a summary of the various methods of GBD with particular focus on EUS-GBD and the many new prospects it allows.
C1 [Chan, Joey Ho Yi; Teoh, Anthony Yuen Bun] Chinese Univ Hong Kong, Prince Wales Hosp, Dept Surg, Shatin, Hong Kong, Peoples R China.
C3 Chinese University of Hong Kong; Prince of Wales Hospital Hong Kong
RP Teoh, AYB (corresponding author), Chinese Univ Hong Kong, Prince Wales Hosp, Dept Surg, Div Upper Gastrointestinal & Metab Surg, 4F Clin Sci Bldg, Shatin, Hong Kong, Peoples R China.
EM anthonyteoh@surgery.cuhk.edu.hk
RI Teoh, Anthony/L-4796-2016
CR Chan SM, 2017, GASTROINTEST ENDOSC, V85, P1225, DOI 10.1016/j.gie.2016.10.014
Choi JH, 2014, ENDOSCOPY, V46, P656, DOI 10.1055/s-0034-1365720
de la Serna-Higuera C, 2013, GASTROINTEST ENDOSC, V77, P303, DOI 10.1016/j.gie.2012.09.021
Dollhopf M, 2017, GASTROINTEST ENDOSC, V86, P636, DOI 10.1016/j.gie.2017.02.027
FERETIS CB, 1990, GASTROINTEST ENDOSC, V36, P523, DOI 10.1016/S0016-5107(90)71134-0
Irani S, 2017, CLIN GASTROENTEROL H, V15, P738, DOI 10.1016/j.cgh.2016.12.021
Itoi T, 2008, GASTROINTEST ENDOSC, V68, P455, DOI 10.1016/j.gie.2008.02.052
Itoi T, 2017, J HEPATO-BIL-PAN SCI, V24, P362, DOI 10.1002/jhbp.454

Itoi T, 2010, GASTROINTEST ENDOSC, V71, P1038, DOI 10.1016/j.gie.2010.01.026
Jang JW, 2011, GASTROINTEST ENDOSC, V74, P176, DOI 10.1016/j.gie.2011.03.1120
Khan MA, 2017, GASTROINTEST ENDOSC, V85, P76, DOI 10.1016/j.gie.2016.06.032
Kjaer DW, 2007, ENDOSCOPY, V39, P304, DOI 10.1055/s-2007-966335
Kortram K, 2011, SURG ENDOSC, V25, P3642, DOI 10.1007/s00464-011-1771-5
Lee TH, 2011, ENDOSCOPY, V43, P702, DOI 10.1055/s-0030-1256226
MCGAHAN JP, 1989, RADIOLOGY, V173, P481, DOI 10.1148/radiology.173.2.2678261
Mutignani M, 2009, ENDOSCOPY, V41, P539, DOI 10.1055/s-0029-1214727
Nakai Y, 2014, DIGEST ENDOSC, V26, P86, DOI 10.1111/den.12152
Nakatsu T., 1997, J HEPATOBILIARY PANC, V4, P31, DOI DOI 10.1007/BF01211341
Sugiyama M, 1998, WORLD J SURG, V22, P459, DOI 10.1007/s002689900416
SWAIN CP, 1991, GASTROINTEST ENDOSC, V37, P628, DOI 10.1016/S0016-5107(91)70870-5
Teoh AYB, 2007, BRIT J SURG, V94, P1128, DOI 10.1002/bjs.5777
Teoh AYB, 2014, ENDOSCOPY, V46, pE13, DOI 10.1055/s-0033-1359139
Teoh AY, 2016, GASTROINTEST ENDOSC, V84, P62, DOI 10.1016/j.gie.2015.11.041
Teoh AYB, 2017, ENDOSCOPY, V49, P130, DOI 10.1055/s-0042-119036
Teoh AYB, 2014, GASTROINTEST ENDOSC, V80, P1171, DOI 10.1016/j.gie.2014.03.038
Tyberg A, 2018, J CLIN GASTROENTEROL, V52, P79, DOI 10.1097/MCG.0000000000000786
Walter D, 2016, GUT, V65, P6, DOI 10.1136/gutjnl-2015-309925
Winbladh A, 2009, HPB, V11, P183, DOI 10.1111/j.1477-2574.2009.00052.x
Yeung Baldwin, 2016, Curr Gastroenterol Rep, V18, P42, DOI [10.1007/s11894-016-0518-9, 10.1007/s11894-016-0518-9]
NR 29
TC 21
Z9 22
U1 0
U2 6
PU KOREAN SOC GASTROINTESTINAL ENDOSCOPY
PI SEOUL
PA 2003 LG PALACE, 165-8 DONGGYO-DONG, MAPO-GU, SEOUL, 121-754, SOUTH KOREA
SN 2234-2400
EI 2234-2443
J9 CLIN ENDOSC
JI Clin. Endosc.
PD MAR
PY 2018
VL 51
IS 2
BP 150
EP 155
DI 10.5946/ce.2017.125
PG 6

WC Gastroenterology & Hepatology
WE Emerging Sources Citation Index (ESCI)
SC Gastroenterology & Hepatology
GA GF3MM
UT WOS:000431860100009
PM 29145349
OA Green Submitted, gold
DA 2026-05-09
ER

PT J
AU Bang, JY
Wilcox, CM
Navaneethan, U
Hawes, R
Varadarajulu, S
AF Bang, Ji Young
Wilcox, Charles Mel
Navaneethan, Udayakumar
Hawes, Robert
Varadarajulu, Shyam
TI Impact of endoprosthesis type on inflammatory response in patients
undergoing endoscopic drainage of pancreatic fluid collections
SO DIGESTIVE ENDOSCOPY
LA English
DT Article
DE inflammatory response; lumen-apposing metal stent; pancreatic fluid
collection; plastic stent; walled-off necrosis
ID INFECTED NECROTIZING PANCREATITIS; ORGAN FAILURE; DEFINITIONS; NECROSIS;
SEPSIS
AB ObjectiveTo study the impact of endoprosthesis type on inflammatory response in patients undergoing endoscopic ultrasound (EUS)-guided drainage of pancreatic fluid collections (PFC). MethodsPatients undergoing EUS-guided PFC drainage and treated using lumen-apposing metal stents (LAMS) or plastic endoprostheses constituted the study cohort. The primary outcome was the presence of systemic inflammatory response syndrome (SIRS) after index intervention. Secondary outcomes were persistent organ failure, new onset organ failure, duration of hospitalization, and treatment success. ResultsIn all, 303 patients were treated using LAMS (n = 247) or plastic stents (n = 56). At 48 h postintervention, the presence of SIRS (25.0 vs. 14.2%, P = 0.047), new onset SIRS (10.0 vs. 1.8%, P = 0.017), and new organ failure (5.4 vs. 0.4%, P = 0.003) were significantly higher in the plastic stent cohort compared to LAMS. On multivariable logistic regression analysis, the use of plastic stents (odds ratio [OR] 2.7, 95% confidence interval [CI] 1.2-6.0, P = 0.014), patients receiving high-acuity care (OR 5.1, 95% CI 2.5-10.4, P < 0.001) and the presence of >= 33% of necrosis (OR 4.5, 95% CI 2.0-10.0, P < 0.001) were significantly associated with the presence of SIRS or new organ failure. While there was no significant difference in treatment success (96.4 vs. 95.5%, P = 0.77), duration of hospitalization was significantly longer

for the plastic stent cohort (mean [standard deviation] 12.5 [17.8] vs. 7.9 [10.1] days, P = 0.009). ConclusionsUse of plastic stents as compared to LAMS was associated with a higher proportion of SIRS and new organ failure that prolonged hospital stay. Therefore, placement of LAMS is recommended in sick patients and those with >= 33% necrosis to minimize inflammatory response.
C1 [Bang, Ji Young; Wilcox, Charles Mel; Navaneethan, Udayakumar; Hawes, Robert; Varadarajulu, Shyam] Digest Hlth Inst, Orlando Hlth, Orlando, FL 32806 USA.

[Varadarajulu, Shyam] Orlando Hlth, Digest Hlth Inst, 1335 Sligh Blvd, Orlando, FL 32806 USA.

C3 Orlando Health; Orlando Health

RP Varadarajulu, S (corresponding author), Orlando Hlth, Digest Hlth Inst, 1335 Sligh Blvd, Orlando, FL 32806 USA.

EM svaradarajulu@yahoo.com
CR Austin PC, 2011, MULTIVAR BEHAV RES, V46, P399, DOI 10.1080/00273171.2011.568786

Bakker OJ, 2012, JAMA-J AM MED ASSOC, V307, P1053, DOI 10.1001/jama.2012.276

Bang JY, 2022, DIGEST ENDOSC, V34, P612, DOI 10.1111/den.14099

Bang JY, 2021, CLIN GASTROENTEROL H, V19, P1275, DOI 10.1016/j.cgh.2020.07.022

Bang JY, 2020, DIGEST ENDOSC, V32, P298, DOI 10.1111/den.13470

Bang JY, 2019, GASTROENTEROLOGY, V156, P1027, DOI 10.1053/j.gastro.2018.11.031

Bang JY, 2019, GUT, V68, P1200, DOI 10.1136/gutjnl-2017-315335

Bang JY, 2018, ANN SURG, V267, P561, DOI 10.1097/SLA.0000000000002082

Bang JY, 2017, DIGEST ENDOSC, V29, P83, DOI 10.1111/den.12681

Banks PA, 2013, GUT, V62, P102, DOI 10.1136/gutjnl-2012-302779

BONE RC, 1992, CHEST, V101, P1644, DOI 10.1378/chest.101.6.1644

Boxhoorn L, 2023, GUT, V72, P66, DOI 10.1136/gutjnl-2021-325632

Petrov MS, 2010, GASTROENTEROLOGY, V139, P813, DOI 10.1053/j.gastro.2010.06.010

Seymour CW, 2016, JAMA-J AM MED ASSOC, V315, P762, DOI 10.1001/jama.2016.0288

Strom T, 2010, LANCET, V375, P475, DOI 10.1016/S0140-6736(09)62072-9

van Brunschot S, 2018, LANCET, V391, P51, DOI 10.1016/S0140-6736(17)32404-2

Varadarajulu S, 2013, GASTROENTEROLOGY, V145, P583, DOI 10.1053/j.gastro.2013.05.046

Velasquez-Rodriguez JG, 2022, GASTROINTEST ENDOSC, V95, pAB491

Yang JY, 2019, GASTROINTEST ENDOSC, V90, P360, DOI 10.1016/j.gie.2019.04.236
NR 19

TC 9

Z9 10

U1 0

U2 1

PU WILEY

PI HOBOKEN

PA 111 RIVER ST, HOBOKEN 07030-5774, NJ USA

SN 0915-5635

EI 1443-1661

J9 DIGEST ENDOSC

JI Dig. Endosc.

PD FEB

PY 2024
VL 36
IS 2
BP 195
EP 202
DI 10.1111/den.14565
EA MAY 2023
PG 8
WC Gastroenterology & Hepatology; Surgery
WE Science Citation Index Expanded (SCI-EXPANDED)
SC Gastroenterology & Hepatology; Surgery
GA GX3I9
UT WOS:000985175800001
PM 37039707
DA 2026-05-09
ER

PT J
AU Haddad, JD
Tielleman, T
Fuller, A
Tavakkoli, A
Vanderveldt, D
Goldschmiedt, M
Kubiliun, N
Sawas, T
AF Haddad, James D.
Tielleman, Thomas
Fuller, Andrew
Tavakkoli, Anna
Vanderveldt, Dutch
Goldschmiedt, Markus
Kubiliun, Nisa
Sawas, Tarek
TI Safety and Efficacy of Lumen-Apposing Metal Stents With and Without
Coaxial Plastic Stents for Pancreatic Fluid Collections
SO TECHNIQUES AND INNOVATIONS IN GASTROINTESTINAL ENDOSCOPY
LA English
DT Article
DE LAMS; Cystgastrostomy; Pancreatic fluid collections; Walled-off necrosis
ID GUIDED DRAINAGE; MANAGEMENT; OUTCOMES
AB BACKGROUND AND AIMS: Pancreatic fluid collections (PFCs) are common complications of acute pancreatitis and

can cause pain, difficulty feeding, and infection. Endoscopic ultrasound-guided drain-age has become the standard of care, with lumen-apposing metal stents (LAMSs) replacing double pig-tail plastic stents (DPPSs) as the preferred device. Coaxial placement of DPPSs through LAMSs is hypothesized to lower the risks of adverse events related to LAMSs. We conducted a retrospective study to evaluate the safety and efficacy of this strategy.METHODS: We conducted a retrospective study of consecutive patients with PFCs undergoing endo-scopic cystgastrostomy with LAMSs and DPPSs or LAMSs alone at two U.S. academic tertiary care cen-ters from January 2016 until November 2022. Propensity scoring and an adjusted logistic regression model were used for analysis. RESULTS: We included 68 patients with an average follow-up of 189 days. The most common etiology of pancreatitis was gallstones (35.3%), most PFCs were walled-off necrosis (61.8%), and the mean size was 14.7 cm (SD </n> 5.9 cm). Overall clinical success was 88.2%, without significant differences between LAMSs and DPPSs vs LAMSs alone (95.7% vs 84.4%; P = 0.18; aOR = 4.6; 95% CI, 0.5-41.4). We found no statistically significant differences in rates of LAMS occlusion (aOR = 0.47; 95% CI, 0.09-2.5), infection (aOR = 1.03; 95% CI, 0.17-6.2), bleeding (aOR = 0.4; 95% CI, 0.03-5), or stent migration (aOR = 0.42; 95% CI, 0.04-4.1) between the 2 groups.CONCLUSION: This retrospective cohort study found no statistically significant differences in the safety or efficacy of cystgastrostomy with LAMSs and DPPSs vs LAMSs alone. Larger, prospective trials comparing these strategies are needed.
C1 [Haddad, James D.; Tielleman, Thomas; Fuller, Andrew; Tavakkoli, Anna; Vanderveldt, Dutch; Goldschmiedt, Markus; Kubiliun, Nisa; Sawas, Tarek] Univ Texas Southwestern Med Ctr, Div Digest & Liver Dis, Dallas, TX USA.

[Sawas, Tarek] Univ Texas Southwestern Med Ctr, Div Digest & Liver Dis, 5323 Harry Hines Blvd, Dallas, TX 75390 USA.

C3 University of Texas System; University of Texas Southwestern Medical Center; University of Texas System; University of Texas Southwestern Medical Center

RP Sawas, T (corresponding author), Univ Texas Southwestern Med Ctr, Div Digest & Liver Dis, 5323 Harry Hines Blvd, Dallas, TX 75390 USA.

EM tarek.sawas@utsouthwestern.edu

RI Sawas, Tarek/GXV-4455-2022

OI Haddad, James/0000-0002-6031-7232
CR Aburajab M, 2018, GASTROINTEST ENDOSC, V87, P1248, DOI 10.1016/j.gie.2017.11.033

Ahmad W, 2020, SCAND J GASTROENTERO, V55, P242, DOI 10.1080/00365521.2019.1710246

Ali SE, 2019, ANN GASTROENTEROL, V32, P620, DOI 10.20524/aog.2019.0414

Bang JY, 2017, GUT, V66, P2054, DOI 10.1136/gutjnl-2016-312812

Chen YI, 2019, ENDOSC INT OPEN, V7, pE347, DOI 10.1055/a-0828-7630

Cui ML, 2014, DIGEST DIS SCI, V59, P1055, DOI 10.1007/s10620-013-2967-4

DeSimone ML, 2017, WORLD J GASTRO ENDOS, V9, P456, DOI 10.4253/wjge.v9.i9.456

Dorrell R, 2021, J CLIN MED, V10, DOI 10.3390/jcm10020284

Fugazza A, 2020, GASTROINTEST ENDOSC, V91, P574, DOI 10.1016/j.gie.2019.11.021

Ge PS, 2020, PANCREAS, V49, P236, DOI 10.1097/MPA.0000000000001476

Hammad T, 2018, DIGEST DIS SCI, V63, P289, DOI 10.1007/s10620-017-4851-0

Lang GD, 2018, GASTROINTEST ENDOSC, V87, P150, DOI 10.1016/j.gie.2017.06.029

Li Jing, 2020, Chronic Dis Transl Med, V6, P158, DOI [10.1016/j.cdtm.2020.03.001, 10.1016/j.cdtm.2020.03.001]

Lyu YX, 2021, PANCREAS, V50, P571, DOI 10.1097/MPA.0000000000001798

Puga M, 2018, ENDOSCOPY, V50, P1022, DOI 10.1055/a-0582-9127

Shamah SP, 2022, ENDOSC ULTRASOUND, V11, P53, DOI 10.4103/EUS-D-21-00030

Siddiqui AA, 2017, GASTROINTEST ENDOSC, V85, P758, DOI 10.1016/j.gie.2016.08.014
Tan SL, 2020, GASTROENT RES PRACT, V2020, DOI 10.1155/2020/4952721
NR 18
TC 4
Z9 4
U1 0
U2 0
PU ELSEVIER SCIENCE INC
PI NEW YORK
PA STE 800, 230 PARK AVE, NEW YORK, NY 10169 USA
SN 2666-5107
EI 2590-0307
J9 TECH INNOVAT GASTROI
JI Tech. Innovation Gastrointes. Endoscopy
PY 2023
VL 25
IS 2
BP 113
EP 118
DI 10.1016/j.tige.2022.12.001
EA APR 2023
PG 6
WC Gastroenterology & Hepatology
WE Emerging Sources Citation Index (ESCI)
SC Gastroenterology & Hepatology
GA P8XO1
UT WOS:001053451100001
DA 2026-05-09
ER

PT J
AU Dhar, J
Samanta, J
AF Dhar, Jahnvi
Samanta, Jayanta
TI Role of therapeutic endoscopic ultrasound in gastrointestinal
malignancy- current evidence and future directions
SO CLINICAL JOURNAL OF GASTROENTEROLOGY
LA English
DT Review
DE Biliary drainage; Gastrojejunostomy; Celiac plexus neurolysis; Tumor
ablation; Immunotherapy

ID GUIDED BILIARY DRAINAGE; FINE-NEEDLE INJECTION; ADVANCED PANCREATIC-CANCER; APPOSING METAL STENT; RETROGRADE CHOLANGIOPANCREATOGRAPHY; GALLBLADDER DRAINAGE; FIDUCIAL PLACEMENT; RADIATION-THERAPY; INTERSTITIAL BRACHYTHERAPY; INTERNATIONAL MULTICENTER
AB Endoscopic ultrasound (EUS) has come a long way from a mere diagnostic tool to an advanced therapeutic modality. With the advent of better technologies and accessories, EUS has found ground in the management of gastrointestinal (GI) malignancies, not only for diagnosis but also for therapeutic purposes. EUS can tackle a host of conditions, including hepato-pancreatico-biliary malignancies. Advances and experience in various EUS-guided biliary drainage techniques have enabled the endosonologist to tackle biliary obstruction when conventional techniques of endoscopic retrograde cholangiopancreatography (ERCP) and/or percutaneous transhepatic biliary drainage (PTBD) fails. More and more emerging data not only establishes the safety of EUS-BD but also demonstrates superior efficacy over PTBD and sometimes even ERCP. Malignant gastric outlet obstruction can now be safely managed with EUS-guided gastroenterostomy. Starting from pain management in malignant tumors through celiac plexus neurolysis to various tumor ablative therapies, EUS has forged ahead over percutaneous treatment or surgical options in the management of GI malignancies. Additional data is now coming up on the prospects of EUS-guided immunotherapy and biological therapy for tumor management. The future of EUS therapeutics in the field of GI malignancies is bright. With increasing evidence, this modality becoming a key player in management of a host of complex clinical conditions arising out of GI malignancies is in the offing. This review focuses on elucidating the role of therapeutic EUS in the management of GI malignancies, a synopsis of various techniques, data on its safety and efficacy as well as future advancements in this domain.
C1 [Dhar, Jahnvi; Samanta, Jayanta] Postgrad Inst Med Educ & Res, Dept Gastroenterol, Chandigarh 160012, India.
C3 Post Graduate Institute of Medical Education & Research (PGIMER), Chandigarh
RP Samanta, J (corresponding author), Postgrad Inst Med Educ & Res, Dept Gastroenterol, Chandigarh 160012, India.
EM jahnvi3012@gmail.com; dj_samanta@yahoo.co.in
OI Dhar, Jahnvi/0000-0002-6929-4276
CR Alvarez-Sanchez MV, 2014, SURG ENDOSC, V28, P712, DOI 10.1007/s00464-013-3260-5
Amin Sunil, 2017, Gastrointest Endosc Clin N Am, V27, P707, DOI [10.1016/j.giec.2017.06.009, 10.1016/j.giec.2017.06.009]
[Anonymous], 2013, J Interv Gastroenterol, DOI [DOI 10.7178/JIG.102, 10.7178/jig.102]
Arcidiacono PG, 2012, GASTROINTEST ENDOSC, V76, P1142, DOI 10.1016/j.gie.2012.08.006
Artifon ELA, 2012, J CLIN GASTROENTEROL, V46, P768, DOI 10.1097/MCG.0b013e31825f264c
Bang JY, 2019, GASTROINTEST ENDOSC, V89, P58, DOI 10.1016/j.gie.2018.08.005
Bang JY, 2018, GASTROINTEST ENDOSC, V88, P9, DOI 10.1016/j.gie.2018.03.012
Bapaye A, 2013, UNITED EUR GASTROENT, V1, P285, DOI 10.1177/2050640613490928
Baron TH, 2007, GASTROINTEST ENDOSC, V65, P735, DOI 10.1016/j.gie.2006.07.041
Bhutani Manoop S, 2019, VideoGIE, V4, P223, DOI [10.1016/j.vgie.2019.02.009, 10.1016/j.vgie.2019.02.009]
Boulay Brian R, 2018, Gastrointest Endosc Clin N Am, V28, P171, DOI [10.1016/j.giec.2017.11.005, 10.1016/j.giec.2017.11.005]
Bowles MJ, 2001, BRIT MED J, V323, P1413, DOI 10.1136/bmj.323.7326.1413
Chandan S, 2021, ENDOSC INT OPEN, V09, pE496, DOI 10.1055/a-1341-0788
Chang KJ, 2000, CANCER-AM CANCER SOC, V88, P1325, DOI 10.1002/(SICI)1097-0142(20000315)88:6<1325::AID-CNCR8>3.0.CO;2-T

CHANG KJ, 1995, AM J GASTROENTEROL, V90, P148
Chen YI, 2019, TRIALS, V20, DOI 10.1186/s13063-019-3918-y
Chen YI, 2017, SURG ENDOSC, V31, P2946, DOI 10.1007/s00464-016-5311-1
Chin MA, 2018, ENDOSC ULTRASOUND, V7, P223, DOI 10.4103/eus.eus_31_18
Choi JH, 2015, ENDOSCOPY, V47, P1035, DOI 10.1055/s-0034-1392150
Choi JH, 2014, GUT LIVER, V8, P88, DOI 10.5009/gnl.2014.8.1.88
Dawod E, 2018, TRANSL GASTROENT HEP, V3, DOI 10.21037/tgh.2018.11.03
DeWitt J, 2007, CLIN GASTROENTEROL H, V5, P609, DOI 10.1016/j.cgh.2006.11.021
DeWitt JM, 2019, GASTROINTEST ENDOSC, V89, P390, DOI 10.1016/j.gie.2018.09.007
Dhaliwal A, 2020, ENDOSC INT OPEN, V08, pE1243, DOI 10.1055/a-1221-5012
Dhindsa BS, 2020, ENDOSC ULTRASOUND, V9, P101, DOI 10.4103/eus.eus_80_19
Dhir V, 2015, GASTROINTEST ENDOSC, V81, P913, DOI 10.1016/j.gie.2014.09.054
Dhir V, 2013, UNITED EUR GASTROENT, V1, P103, DOI 10.1177/2050640613480145
Dhir V, 2014, DIGEST ENDOSC, V26, P430, DOI 10.1111/den.12153
Di Matteo F, 2014, ENDOSCOPY, V46, pE380, DOI 10.1055/s-0034-1377376
DiMaio CJ, 2010, GASTROINTEST ENDOSC, V71, P1204, DOI 10.1016/j.gie.2010.01.003
Emmanuel J, 2020, JGH OPEN, V4, P1059, DOI 10.1002/jgh3.12386
European Assoc Study Liver, 2018, J HEPATOL, V69, P406, DOI 10.1016/j.jhep.2018.03.024
Faigel DO, 1996, AM J GASTROENTEROL, V91, P1675
Fujii-Lau LL, 2015, GASTROINTEST ENDOSC, V82, P46, DOI 10.1016/j.gie.2014.12.036
Giovannini M, 2001, ENDOSCOPY, V33, P898, DOI 10.1055/s-2001-17324
Glazer ES, 2014, J PAIN SYMPTOM MANAG, V47, P307, DOI 10.1016/j.jpainsymman.2013.03.013
Gupta K, 2014, J CLIN GASTROENTEROL, V48, P80, DOI 10.1097/MCG.0b013e31828c6822
Han SY, 2019, SCI REP-UK, V9, DOI 10.1038/s41598-019-52993-x
Hanada Y, 2021, GASTROINTEST ENDOSC, V94, P179, DOI 10.1016/j.gie.2021.02.027
Hanna N, 2012, CANCER GENE THER, V19, P374, DOI 10.1038/cgt.2012.10
Hecht JR, 2012, GASTROINTEST ENDOSC, V75, P332, DOI 10.1016/j.gie.2011.10.007
Hecht JR, 2003, CLIN CANCER RES, V9, P555
Hirooka Y, 2018, BMC CANCER, V18, DOI 10.1186/s12885-018-4453-z
Hirooka Y, 2009, PANCREAS, V38, pE69, DOI 10.1097/MPA.0b013e318197a9e3
Holt BA, 2016, GASTROINTEST ENDOSC, V83, P160, DOI 10.1016/j.gie.2015.06.019
Imai H, 2016, GASTROINTEST ENDOSC, V84, P147, DOI 10.1016/j.gie.2015.12.024
Irisawa A, 2007, PANCREAS, V35, P189, DOI 10.1097/01.mpa.0000250141.25639.e9
Isayama H, 2019, J HEPATO-BIL-PAN SCI, V26, P249, DOI 10.1002/jhbp.631
Itoi T, 2016, GUT, V65, P193, DOI 10.1136/gutjnl-2015-310348
Jeurnink SM, 2010, GASTROINTEST ENDOSC, V71, P490, DOI 10.1016/j.gie.2009.09.042
Jin Z, 2008, ENDOSCOPY, V40, P314, DOI 10.1055/s-2007-995476
Jovani M, 2021, GASTROINTEST ENDOSC, V93, P1088, DOI 10.1016/j.gie.2020.09.041
Karaca C, 2011, GASTROINTEST ENDOSC, V73, P603, DOI 10.1016/j.gie.2010.11.003
Kaushik N, 2006, GASTROINTEST ENDOSC, V64, P908, DOI 10.1016/j.gie.2005.11.058
Kawakubo K, 2014, J HEPATO-BIL-PAN SCI, V21, P328, DOI 10.1002/jhbp.27
Khan MA, 2016, DIGEST DIS SCI, V61, P684, DOI 10.1007/s10620-015-3933-0

Khashab MA, 2017, ENDOSC INT OPEN, V5, pE275, DOI 10.1055/s-0043-101695
Khashab MA, 2016, ENDOSC INT OPEN, V4, pE487, DOI 10.1055/s-0042-102648
Khashab MA, 2015, DIGEST DIS SCI, V60, P557, DOI 10.1007/s10620-014-3300-6
Khashab MA, 2013, GASTROINTEST ENDOSC, V78, P734, DOI 10.1016/j.gie.2013.05.013
Köck R, 2018, ENDOSC INT OPEN, V6, pE902, DOI 10.1055/a-0605-3587
Kongkam P, 2021, ENDOSCOPY, V53, P55, DOI 10.1055/a-1195-8197
Lee TH, 2016, CLIN GASTROENTEROL H, V14, P1011, DOI 10.1016/j.cgh.2015.12.032
Lee YT, 2005, GUT, V54, P1541, DOI 10.1136/gut.2004.055772
Levy MJ, 2017, GASTROINTEST ENDOSC, V86, P161, DOI 10.1016/j.gie.2016.11.014
Levy MJ, 2015, GASTROINTEST ENDOSC, V81, P1215, DOI 10.1016/j.gie.2014.10.028
Li T, 2015, GASTROINTEST ENDOSC, V81, P1243, DOI 10.1016/j.gie.2014.12.019
Ma KW, 2020, J GASTROEN HEPATOL, V35, P1753, DOI 10.1111/jgh.15089
Marino A, 2022, ENDOSCOPY, V54, P170, DOI 10.1055/a-1392-4546
Matthes K, 2007, GASTROINTEST ENDOSC, V65, P448, DOI 10.1016/j.gie.2006.06.030
Moole H, 2016, CAN J GASTROENTEROL, V2016, DOI 10.1155/2016/4726078
Mukai S, 2017, J HEPATO-BIL-PAN SCI, V24, P537, DOI 10.1002/jhbp.496
Nakai Y, 2019, ENDOSC ULTRASOUND, V8, P111, DOI 10.4103/eus.eus_17_18
Nguyen NQ, 2020, GASTROINTEST ENDOSC, V91, pAB93, DOI 10.1016/j.gie.2020.03.859
Nguyen PT, 2001, GASTROINTEST ENDOSC, V54, P336, DOI 10.1067/mge.2001.117544
Nishimura M, 2018, GASTROINTEST ENDOSC, V87, P1126, DOI 10.1016/j.gie.2017.10.030
Nussbaum Jeremy S, 2019, Gastrointest Endosc Clin N Am, V29, P277, DOI [10.1016/j.giec.2018.11.005, 10.1016/j.giec.2018.11.005]
Ogura T, 2021, DIGEST DIS, V39, P150, DOI 10.1159/000509215
Oh D, 2017, THER ADV GASTROENTER, V10, P42, DOI 10.1177/1756283X16671671
Paik WH, 2018, AM J GASTROENTEROL, V113, P987, DOI 10.1038/s41395-018-0122-8
Park JK, 2018, GASTROINTEST ENDOSC, V88, P277, DOI 10.1016/j.gie.2018.03.015
Park SJ, 2013, GASTROINTEST ENDOSC, V78, P374, DOI 10.1016/j.gie.2013.04.183
Petrone MC, 2017, UNITED EUROPEAN GAST, V3, pA377
Pishvaian AC, 2006, GASTROINTEST ENDOSC, V64, P412, DOI 10.1016/j.gie.2006.01.048
Podboy A, 2021, GASTROINTEST ENDOSC, V93, P797, DOI 10.1016/j.gie.2020.09.040
Rai P, 2020, INDIAN J GASTROENTER, V39, P171, DOI 10.1007/s12664-019-01003-z
Romero-Castro R., EUS GUIDED DRAINAGE
Romero-Castro R, 2017, ENDOSC INT OPEN, V5, pE1096, DOI 10.1055/s-0043-118746
Saleh AAG, 2021, J GASTROINTEST CANC, V52, P682, DOI 10.1007/s12029-020-00449-1
Sharaiha RZ, 2016, SURG ENDOSC, V30, P5500, DOI 10.1007/s00464-016-4913-y
Sportes A, 2017, THER ADV GASTROENTER, V10, P483, DOI 10.1177/1756283X17702096
Sun S, 2006, ENDOSCOPY, V38, P399, DOI 10.1055/s-2006-925253
Sun SY, 2005, GASTROINTEST ENDOSC, V62, P775, DOI 10.1016/j.gie.2005.06.050
Suzuki R, 2014, WORLD J GASTRO ENDOS, V6, P55, DOI 10.4253/wjge.v6.i2.55
Tarantino I, 2021, ENDOSC INT OPEN, V09, pE110, DOI 10.1055/a-1313-6850
Teoh AYB, 2020, GUT, V69, P1085, DOI 10.1136/gutjnl-2019-319996
Teoh AYB, 2021, GASTROINTEST ENDOSC, V93, P577, DOI 10.1016/j.gie.2020.06.066

Teoh AYB, 2014, GASTROINTEST ENDOSC, V80, P1171, DOI 10.1016/j.gie.2014.03.038
Tsuchiya T, 2018, GASTROINTEST ENDOSC, V87, P1138, DOI 10.1016/j.gie.2017.08.017
Umeda J, 2015, GASTROINTEST ENDOSC, V82, P390, DOI 10.1016/j.gie.2015.02.041
Varadarajulu S, 2010, ENDOSCOPY, V42, P423, DOI 10.1055/s-0029-1243989
Varadarajulu S, 2008, GASTROINTEST ENDOSC, V67, P758, DOI 10.1016/j.gie.2007.08.025
Wang KX, 2012, GASTROINTEST ENDOSC, V76, P945, DOI 10.1016/j.gie.2012.05.032
Wang KX, 2016, GASTROINTEST ENDOSC, V83, P1218, DOI 10.1016/j.gie.2015.10.033
Wardeh R, 2011, CANCER CYTOPATHOL, V119, P27, DOI 10.1002/cncy.20123
Wiersema MJ, 1996, GASTROINTEST ENDOSC, V44, P656, DOI 10.1016/S0016-5107(96)70047-0
Winick A B, 2001, Tech Vasc Interv Radiol, V4, P200, DOI 10.1016/S1089-2516(01)90026-5
Wyse JM, 2017, ENDOSC ULTRASOUND, V6, P369, DOI 10.4103/eus.eus_97_17
Wyse JM, 2011, J CLIN ONCOL, V29, P3541, DOI 10.1200/JCO.2010.32.2750
Yang J, 2020, J PAIN RES, V13, P411, DOI 10.2147/JPR.S244173
Zhao YC, 2021, ANN PALLIAT MED, V10, P1755, DOI 10.21037/apm-20-1158
NR 111
TC 6
Z9 6
U1 1
U2 4
PU SPRINGER JAPAN KK
PI TOKYO
PA SHIROYAMA TRUST TOWER 5F, 4-3-1 TORANOMON, MINATO-KU, TOKYO, 105-6005, JAPAN
SN 1865-7257
EI 1865-7265
J9 CLIN J GASTROENTEROL
JI Clin. J. Gastroenterol.
PD FEB
PY 2022
VL 15
IS 1
BP 11
EP 29
DI 10.1007/s12328-021-01559-4
EA JAN 2022
PG 19
WC Gastroenterology & Hepatology
WE Emerging Sources Citation Index (ESCI)
SC Gastroenterology & Hepatology
GA ZD0ZX
UT WOS:000742287200001
PM 35028906

DA 2026-05-09
ER

PT J
AU van der Merwe, SW
Omoshoro-Jones, J
Sanyika, C
AF van der Merwe, Schalk Willem
Omoshoro-Jones, Jones
Sanyika, Charles
TI Endocoil placement after endoscopic ultrasound-guided biliary drainage
may prevent a bile leak
SO WORLD JOURNAL OF GASTROINTESTINAL ENDOSCOPY
LA English
DT Article
DE Endoscopic ultrasound; Biliary drainage; Transhepatic; Endocoil
AB AIM: To further reduce the risk of bleeding or bile leakage.

METHODS: We performed endoscopic ultrasound guided biliary drainage in 6 patients in whom endoscopic retrograde cholangiopancreatography (ERCP) had failed. Biliary access of a dilated segment 2 or 3 duct was achieved from the stomach using a 19G needle. After radiologically confirming access a guide wire was placed, a transhepatic tract created using a 6 Fr cystotome followed by balloon dilation of the stricture and antegrade metallic stent placement across the malignant obstruction. This was followed by placement of an endocoil in the transhepatic tract.

RESULTS: Dilated segmental ducts were observed in all patients with the linear endoscopic ultrasound scope from the proximal stomach. Transgastric biliary access was obtained using a 19G needle in all patients. Bili- ary drainage was achieved in all patients. Placement of an endocoil was possible in 5/6 patients. All patients responded to biliary drainage and no complications occurred.

CONCLUSION: We show that placing endocoils at the time of endoscopic ultrasound guided biliary stenting is feasible and may reduce the risk of bleeding or bile leakage. (C) 2013 Baishideng. All rights reserved.
C1 [van der Merwe, Schalk Willem] Univ Pretoria, Dept Immunol, Hepatol & GI Res Lab, ZA-0002 Pretoria, South Africa.
[van der Merwe, Schalk Willem] Pretoria East Hosp, Intervent Endoscopy Unit, Garsfontein Rd, ZA-0044 Pretoria, South Africa.
[Omoshoro-Jones, Jones] Univ Witwatersrand, Chris Hani Baragwanath Hosp, Dept Surg, ZA-2013 Johannesburg, South Africa.
[Omoshoro-Jones, Jones] Univ Witwatersrand, Fac Hlth Sci, ZA-2013 Johannesburg, South Africa.
[Sanyika, Charles] WITS Donald Gordon Hosp, Dept Intervent radiol, ZA-2193 Johannesburg, South Africa.
C3 University of Pretoria; University of Witwatersrand; University of
Witwatersrand
RP van der Merwe, SW (corresponding author), Pretoria East Hosp, Intervent Endoscopy Unit, Garsfontein Rd, ZA-0044 Pretoria, South Africa.
EM schalk.vandermerwe@uzleuven.be
RI OMOSHORO-JONES, Jones A. O/AAV-1341-2020
OI OMOSHORO-JONES, Jones A. O/0000-0002-1071-298X; Van der Merwe,

Schalk/0000-0002-9891-2686; Sanyika, Charles/0000-0002-7803-8290

CR Bories E, 2007, ENDOSCOPY, V39, P287, DOI 10.1055/s-2007-966212
Burmester E, 2003, GASTROINTEST ENDOSC, V57, P246, DOI 10.1067/mge.2003.85
Giovannini M, 2006, ENDOSCOPY, V38, pS93, DOI 10.1055/s-2006-946665
Giovannini M, 2003, ENDOSCOPY, V35, P1076, DOI 10.1055/s-2003-44596
Hara K, 2011, AM J GASTROENTEROL, V106, P1239, DOI 10.1038/ajg.2011.84
Lyon SM, 2006, CARDIOVASC INTER RAD, V29, P1011, DOI 10.1007/s00270-005-0183-0
Nguyen-Tang T, 2010, ENDOSCOPY, V42, P232, DOI 10.1055/s-0029-1243858
Park DH, 2010, GASTROINTEST ENDOSC, V71, P413, DOI 10.1016/j.gie.2009.10.015
Raymond J, 2008, AM J NEURORADIOL, V29, P745, DOI 10.3174/ajnr.A0902
Saad WEA, 2010, J VASC INTERV RADIOL, V21, P789, DOI 10.1016/j.jvir.2010.01.012
SMITH AC, 1994, LANCET, V344, P1655, DOI 10.1016/S0140-6736(94)90455-3
Stoker J, 1993, J Vasc Interv Radiol, V4, P767, DOI 10.1016/S1051-0443(93)71970-3
STOKER J, 1993, BJS-BRIT J SURG, V80, P1185, DOI 10.1002/bjs.1800800941
Sut M, 2010, J PALLIAT MED, V13, P1311, DOI 10.1089/jpm.2010.0205
Vu DN, 2006, J VASC INTERV RADIOL, V17, P63, DOI 10.1097/01.RVI.0000195399.68445.DD

NR 15
TC 0
Z9 1
U1 0
U2 0
PU BAISHIDENG PUBLISHING GROUP INC
PI PLEASANTON
PA 8226 REGENCY DR, PLEASANTON, CA 94588 USA
SN 1948-5190
J9 WORLD J GASTRO ENDOS
JI World J. Gastrointest. Endosc.
PD MAY 16
PY 2013
VL 5
IS 5
BP 246
EP 250
DI 10.4253/wjge.v5.i5.246
PG 5
WC Gastroenterology & Hepatology
WE Emerging Sources Citation Index (ESCI)
SC Gastroenterology & Hepatology
GA V5D8X
UT WOS:000219518600007
PM 23678378
OA hybrid

DA 2026-05-09
ER

PT J
AU Lesmana, C
Gani, R
Hasan, I
Sulaiman, A
Ho, K
Dhir, V
Lesmana, L
AF Lesmana, C. RinaldiA.
Gani, RinoA.
Hasan, Irsan
Sulaiman, AndriSanityoso
Ho, KhekYu
Dhir, Vinay
Lesmana, LaurentiusA.
TI Palliative Endoscopic Ultrasound Biliary Drainage for Advanced Malignant
Biliary Obstruction: Should It Replace the Percutaneous Approach?
SO CASE REPORTS IN GASTROENTEROLOGY
LA English
DT Article
DE Endoscopic ultrasound-guided biliary drainage; Endoscopic
ultrasound-guided choledochoduodenostomy; Endoscopic retrograde
cholangiopancreatography; Malignant biliary obstruction; Percutaneous
transhepatic biliary drainage
ID FAILED ERCP; GUIDED-CHOLEDOCHODUODENOSTOMY; ADVERSE EVENTS; PREDICTORS;
MANAGEMENT; ACCESS
AB Endoscopic retrograde cholangiopancreatography (ERCP) and percutaneous transhepatic biliary drainage (PTBD) are the standard of care in malignant biliary obstruction cases. Recently, endoscopic ultrasound-guided biliary drainage (EUS-BD) has been widely used after unsuccessful ERCP. However, the patient's clinical impact of EUS-BD over PTBD is still not obvious. Therefore, this case series study aims to evaluate the clinical outcomes of patients with advanced malignant biliary obstruction who underwent EUS-BD after failed ERCP. A retrospective database study was performed between January 2016 and June 2018 in patients with advanced malignant biliary obstruction. Patients were consecutively enrolled without randomization. Treatment options consisted of ERCP and PTBD or EUS-BD if ERCP failed. Based on 144 biliary obstruction cases, 38 patients were enrolled; 24 (63.2%) were men. The patients' mean age was 66.8 +/- 12.36 years. The most common cause of malignant biliary obstruction was pancreatic cancer (44.7%). Biliary drainage was achieved by ERCP (39.5%), PTBD (39.5%), and EUS-BD (21.1%). The technical success rate was 86.7% by PTBD and 87.5% by EUS-BD (p = 1.000), while the clinical success rate was 93.3% by PTBD and 62.5% by EUS-BD (p = 0.500). The median survival in patients who underwent PTBD versus those wo underwent EUS-BD was 11 versus 3 months (log-rank p = 0.455). In conclusion, there is no significant advantage of EUS-BD when compared to PTBD in terms of clinical success

and survival benefit in advanced malignant biliary obstruction. (C) 2019 The Author(s) Published by S. Karger AG, Basel

C1 [Lesmana, C. RinaldiA.; Lesmana, LaurentiusA.] Medistra Hosp, Digest Dis & Gastrointestinal Oncol Ctr, Jl Jend Gatot Subroto Kav 59, Jakarta 12950, Indonesia.

[Lesmana, C. RinaldiA.; Gani, RinoA.; Hasan, Irsan; Sulaiman, AndriSanityoso] Univ Indonesia, Dr Cipto Mangunkusumo Natl Gen Hosp, Fac Med, Hepatobil Div,Dept Internal Med, Jakarta, Indonesia.

[Ho, KhekYu] Natl Univ Singapore Hosp, Div Gastroenterol, Dept Med, Singapore, Singapore.

[Dhir, Vinay] SL Raheja Hosp Fortis Assoc Mahim, Dept Gastroenterol & Endoscopy, Mumbai, Maharashtra, India.

[Dhir, Vinay] Somaiya Ayurvihar, Asian Canc Inst, Dept Gastroenterol & Endoscopy, Mumbai, Maharashtra, India.

C3 Medistra Hospital; University of Indonesia; National University of
Singapore

RP Lesmana, C (corresponding author), Medistra Hosp, Digest Dis & Gastrointestinal Oncol Ctr, Jl Jend Gatot Subroto Kav 59, Jakarta 12950, Indonesia.

EM medicaldr2001id@yahoo.com

OI Hasan, Irsan/0000-0001-6702-2659; Gani, Rino A/0000-0003-4009-7938

CR Artifon ELA, 2012, J CLIN GASTROENTEROL, V46, P768, DOI 10.1097/MCG.0b013e31825f264c

Baniya R, 2017, CLIN EXP GASTROENTER, V10, P67, DOI 10.2147/CEG.S132004

Bill JG, 2016, ENDOSC INT OPEN, V4, pE980, DOI 10.1055/s-0042-112584

Boulay BR, 2016, WORLD J GASTRO ONCOL, V8, P498, DOI 10.4251/wjgo.v8.i6.498

Dhir V, 2012, GASTROINTEST ENDOSC, V75, P354, DOI 10.1016/j.gie.2011.07.075

Hara K, 2011, AM J GASTROENTEROL, V106, P1239, DOI 10.1038/ajg.2011.84

Huang P, 2017, SURG LAPARO ENDO PER, V27, pE127, DOI 10.1097/SLE.0000000000000485

Ikeuchi N., 2015, GASTROINTEST INTERV, V4, P31, DOI [10.1016/j.gii.2015.04.002, 10.1016/j.gii.2015.04.002, DOI 10.1016/J.GII.2015.04.002]

Inamdar S, 2016, JAMA ONCOL, V2, P112, DOI 10.1001/jamaoncol.2015.3670

Iwashita T, 2012, ENDOSCOPY, V44, P60, DOI 10.1055/s-0030-1256871

Kim YS, 2010, ENDOSCOPY, V42, P496, DOI 10.1055/s-0029-1244082

Kosmidis C, 2016, ANN TRANSL MED, V4, DOI 10.21037/atm.2016.05.11

Kurniawan J, 2016, ACTA MED INDONES, V48, P282

Lee TH, 2016, CLIN GASTROENTEROL H, V14, P1011, DOI 10.1016/j.cgh.2015.12.032

Lesmana CR, 2017, EMJ GASTROENTEROL, V6, P69

Lesmana CR., 2018, J GASTROENTEROL HEPA, P1

Lorenz JM, 2016, SEMIN INTERVENT RAD, V33, P259, DOI 10.1055/s-0036-1592330

Makmun Dadang, 2017, Diagn Ther Endosc, V2017, P4856276, DOI [10.1155/2017/4856276, 10.1155/2017/4856276]

Moole H, 2017, MEDICINE, V96, DOI 10.1097/MD.0000000000005154

Park DH, 2011, GASTROINTEST ENDOSC, V74, P1276, DOI 10.1016/j.gie.2011.07.054

Poincloux L, 2015, ENDOSCOPY, V47, P794, DOI [10.1055/s-0035-1545399, 10.1055/s-0034-1391988]

Sabarudin A., 2016, INDONES J GASTROENTE, V17, P93

Sarkaria Savreet, 2013, Gastrointest Endosc Clin N Am, V23, P435, DOI 10.1016/j.giec.2012.12.013

Sharaiha RZ, 2016, SURG ENDOSC, V30, P5500, DOI 10.1007/s00464-016-4913-y

Téllez-Avila FI, 2018, SURG LAPARO ENDO PER, V28, P183, DOI 10.1097/SLE.0000000000000528

NR 25

TC 10

Z9 12
U1 0
U2 0
PU KARGER
PI BASEL
PA ALLSCHWILERSTRASSE 10, CH-4009 BASEL, SWITZERLAND
EI 1662-0631
J9 CASE REP GASTROENTER
JI Case Rep. Gastroenterol.
PD SEP-DEC
PY 2019
VL 13
IS 3
BP 385
EP 397
DI 10.1159/000502835
PG 13
WC Gastroenterology & Hepatology
WE Emerging Sources Citation Index (ESCI)
SC Gastroenterology & Hepatology
GA VK7DV
UT WOS:000744670000004
PM 31616233
OA Green Submitted, gold
DA 2026-05-09
ER

PT J
AU So, H
Oh, D
Takenaka, M
Minaga, K
Uemura, S
Iwashita, T
Saito, T
Nakai, Y
Kim, SO
Park, D
AF So, Hoonsub
Oh, Dongwook
Takenaka, Mamoru
Minaga, Kosuke

Uemura, Shinya
Iwashita, Takuji
Saito, Tomotaka
Nakai, Yousuke
Kim, Seon Ok
Park, Do Hyun
TI Initial experience of endoscopic ultrasound-guided antegrade covered stent placement with long duodenal extension for malignant distal biliary obstruction (with video)
SO JOURNAL OF HEPATO-BILIARY-PANCREATIC SCIENCES
LA English
DT Article
DE duodenal obstruction; Endoscopic ultrasound; EUS-guided biliary drainage; malignant biliary obstruction; surgically altered anatomy
ID DRAINAGE; HEPATICOGASTROSTOMY
AB Background/Purpose This study aimed to evaluate the feasibility of endoscopic ultrasound (EUS)-guided antegrade covered stent placement with long duodenal extension (EASL) for malignant distal biliary obstruction (MDBO) with duodenal obstruction (DO) or surgically altered anatomy (SAA) after failed endoscopic retrograde cholangiopancreatography (ERCP). Methods Outcomes were technical and clinical success, reintervention rate, adverse events, stent patency, and overall survival. Inverse probability of treatment weighting (IPTW) and competing-risk analysis were performed to compare with conventional EUS-BD. Results Twenty-five patients (DO, n = 18; SAA, n = 7) were included. The technical and clinical success rates were 96% and 84%, respectively. Reintervention occurred in two patients (8.3%). Adverse events occurred in six patients (24%; two cholangitis, 16%; four mild postprocedural pancreatitis [24% (n = 4/17) in patients with non-pancreatic cancers]). The median patency was 9.4 months, and the overall survival was 2.73 months. After IPTW adjustment, the median patency in the EASL (n = 25) and conventional EUS-BD (n = 29) were 10.1 and 6.5 months, respectively (P = .018). Conclusions EASL has acceptable clinical outcomes with a low reintervention rate but higher rate of postprocedural pancreatitis in patients with non-pancreatic cancers. Randomized trials comparing EASL and conventional EUS-BD for MDBO with pancreatic cancers and DO/SAA after failed ERCP are needed to validate our findings.
C1 [So, Hoonsub; Park, Do Hyun] Univ Ulsan Coll Med, Ulsan Univ Hosp, Dept Internal Med, Ulsan, South Korea.
[Oh, Dongwook; Park, Do Hyun] Univ Ulsan Coll Med, Asan Med Ctr, Dept Internal Med, Divis Gastroenterol, 88,Olympic ro 43-gil,Songpa gu, Seoul 05505, South Korea.
[Takenaka, Mamoru; Minaga, Kosuke; Park, Do Hyun] Kindai Univ, Fac Med, Dept Gastroenterol & Hepatol, Osaka, Japan.
[Uemura, Shinya; Iwashita, Takuji; Park, Do Hyun] Gifu Univ Hosp, Dept Internal Med, Gifu, Japan.
[Saito, Tomotaka; Nakai, Yousuke; Park, Do Hyun] Univ Tokyo, Grad Sch Med, Department Gastroenterol, Tokyo, Japan.
[Nakai, Yousuke; Park, Do Hyun] Univ Tokyo, Grad Sch Med, Dept Endoscopy & Endoscop Surg, Tokyo, Japan.
[Kim, Seon Ok; Park, Do Hyun] Univ Ulsan Coll Med, Asan Med Ctr, Dept Clin Epidemiol & Biostat, Seoul, South Korea.
C3 University of Ulsan; Ulsan University Hospital; University of Ulsan; Asan Medical Center; Kindai University (Kinki University); Gifu

University; University of Tokyo; University of Tokyo; University of
Ulsan; Asan Medical Center
RP Park, D (corresponding author), Univ Ulsan Coll Med, Ulsan Univ Hosp, Dept Internal Med, Ulsan, South Korea.; Park, D (corresponding author), Univ Ulsan Coll Med, Asan Med Ctr, Dept Internal Med, Divis Gastroenterol, 88,Olympic ro 43-gil,Songpa gu, Seoul 05505, South Korea.; Park, D (corresponding author), Kindai Univ, Fac Med, Dept Gastroenterol & Hepatol, Osaka, Japan.; Park, D (corresponding author), Gifu Univ Hosp, Dept Internal Med, Gifu, Japan.; Park, D (corresponding author), Univ Tokyo, Grad Sch Med, Department Gastroenterol, Tokyo, Japan.; Park, D (corresponding author), Univ Tokyo, Grad Sch Med, Dept Endoscopy & Endoscop Surg, Tokyo, Japan.; Park, D (corresponding author), Univ Ulsan Coll Med, Asan Med Ctr, Dept Clin Epidemiol & Biostat, Seoul, South Korea.; Park, D (corresponding author), Univ Ulsan Coll Med, Asan Med Ctr, Dept Internal Med, Div Gastroenterol, 88,Olympic ro 43-gil,Songpa gu, Seoul, South Korea.
EM dhpark@amc.seoul.kr
RI Minaga, Kosuke/AAD-2107-2020; Nakai, Yousuke/H-2964-2019
OI Takenaka, Mamoru/0000-0001-7308-4311; So, Hoonsub/0000-0001-8092-2811;
Nakai, Yousuke/0000-0001-7411-1385
FU Korea Medical Device Development Fund grant - Korea government (the
Ministry of Science and ICT) [KMDF_PR_20200901_0266, 999100693]; Korea
Medical Device Development Fund grant - Korea government (Ministry of
Trade, Industry and Energy) [KMDF_PR_20200901_0266, 999100693]; Korea
Medical Device Development Fund grant - Korea government (Ministry of
Health & Welfare, Republic of Korea) [KMDF_PR_20200901_0266, 999100693];
Korea Medical Device Development Fund grant - Korea government (Ministry
of Food and Drug Safety) [KMDF_PR_20200901_0266, 999100693]
FX This work was supported by the Korea Medical Device Development Fund
grant funded by the Korea government (the Ministry of Science and ICT,
the Ministry of Trade, Industry and Energy, the Ministry of Health &
Welfare, Republic of Korea, the Ministry of Food and Drug Safety)
(project number: KMDF_PR_20200901_0266, 999100693)
CR Bang BW, 2012, DIGEST DIS SCI, V57, P1056, DOI 10.1007/s10620-011-1958-6
Cotton PB, 2010, GASTROINTEST ENDOSC, V71, P446, DOI 10.1016/j.gie.2009.10.027
Dumonceau JM, 2018, ENDOSCOPY, V50, P910, DOI 10.1055/a-0659-9864
Gwon DI, 2018, KOREAN J RADIOL, V19, P230, DOI 10.3348/kjr.2018.19.2.230
Isayama H, 2019, J HEPATO-BIL-PAN SCI, V26, P249, DOI 10.1002/jhbp.631
Khashab MA, 2015, GASTROINTEST ENDOSC, V82, P993, DOI 10.1016/j.gie.2015.06.043
Lee TH, 2019, GASTROINTEST ENDOSC, V90, P663, DOI 10.1016/j.gie.2019.06.007
Misra SP, 2009, GASTROINTEST ENDOSC, V70, P317, DOI 10.1016/j.gie.2008.12.054
Nakai Y, 2020, GASTROINTEST ENDOSC, V92, P623, DOI 10.1016/j.gie.2020.03.3856
Nennstiel S, 2015, J CLIN GASTROENTEROL, V49, P764, DOI 10.1097/MCG.0000000000000275
Oh D, 2017, THER ADV GASTROENTER, V10, P42, DOI 10.1177/1756283X16671671
Paik WH, 2018, AM J GASTROENTEROL, V113, P987, DOI 10.1038/s41395-018-0122-8
Park DH, 2013, GASTROINTEST ENDOSC, V78, P91, DOI 10.1016/j.gie.2013.01.042
Sharaiha RZ, 2017, GASTROINTEST ENDOSC, V85, P904, DOI 10.1016/j.gie.2016.12.023

Shin SH, 2020, J GASTROEN HEPATOL, V35, P1201, DOI 10.1111/jgh.14957
Yamada M, 2019, J GASTROINTEST SURG, V23, P192, DOI 10.1007/s11605-018-3914-7
Yamamoto K, 2018, ENDOSC ULTRASOUND, V7, P404, DOI 10.4103/eus.eus_51_18
NR 17
TC 12
Z9 13
U1 0
U2 1
PU WILEY
PI HOBOKEN
PA 111 RIVER ST, HOBOKEN 07030-5774, NJ USA
SN 1868-6974
EI 1868-6982
J9 J HEPATO-BIL-PAN SCI
JI J. Hepato-Biliary-Pancreat. Sci.
PD DEC
PY 2021
VL 28
IS 12
BP 1130
EP 1137
DI 10.1002/jhbp.1011
EA JUL 2021
PG 8
WC Gastroenterology & Hepatology; Surgery
WE Science Citation Index Expanded (SCI-EXPANDED)
SC Gastroenterology & Hepatology; Surgery
GA XT9CK
UT WOS:000670743000001
PM 34118136
OA Green Submitted, hybrid
DA 2026-05-09
ER

PT J
AU Sarkaria, S
Lee, HS
Gaidhane, M
Kahaleh, M
AF Sarkaria, Savreet
Lee, Ho-Su
Gaidhane, Monica

Kahaleh, Michel
TI Advances in Endoscopic Ultrasound-Guided Biliary Drainage: A
Comprehensive Review
SO GUT AND LIVER
LA English
DT Review
DE Endoscopic retrograde cholangiopancreatography; Endoscopic ultrasound;
Self expanding metal stent
ID CASE SERIES; BILE-DUCT; OBSTRUCTION; ERCP; CHOLEDOCHODUODENOSTOMY;
CHOLANGIOGRAPHY; STENT; CHOLANGIOPANCREATOGRAPHY; SUCCESS
AB Endoscopic retrograde cholangiopancreatography (ERCP) has become the first-line therapy for bile duct drainage. In the hands of experienced endoscopists, conventional ERCP results in a failed cannulation rate of 3% to 5%. This failure can occur more commonly in the setting of altered anatomy or technically difficult cases due to either duodenal or biliary obstruction. In cases of ERCP failure, patients have traditionally been referred for either percutaneous transhepatic biliary drainage (PTBD) or surgery. However, both PTBD and surgery have higher than desirable complication rates. Within the last decade, endoscopic ultrasound-guided biliary drainage (EUS-BD) has become an attractive alternative to PTBD after failed ERCP. Many groups have reported on the feasibility, efficacy and safety of this technique. This article reviews the indications for ERCP and the currently practiced EUS-BD techniques, including EUS-guided rendezvous, EUS-guided choledochoduodenostomy and EUS-guided hepaticogastrostomy. (Gut Liver 2013;7:129-136)
C1 [Sarkaria, Savreet; Lee, Ho-Su; Gaidhane, Monica; Kahaleh, Michel] Weill Cornell Med Coll, Dept Med, Div Gastroenterol & Hepatol, New York, NY 10021 USA.
C3 Cornell University; Weill Cornell Medicine
RP Kahaleh, M (corresponding author), Weill Cornell Med Coll, Dept Med, Div Gastroenterol & Hepatol, 1305 York Ave,4th Floor, New York, NY 10021 USA.
EM mkahaleh@gmail.com
RI ; Kahaleh, Michel/A-9408-2011; Gaidhane, Monica/B-9414-2009; M,
Gaidhane/B-9414-2009
OI Lee, Ho-Su/0000-0003-3711-2180; Kahaleh, Michel/0000-0003-0836-6114;
Gaidhane, Monica/0000-0001-6773-7080;
CR Artifon ELA, 2006, AM J GASTROENTEROL, V101, P2031, DOI 10.1111/j.1572-0241.2006.00764.x
Baron TH, 2006, GASTROINTEST ENDOSC, V63, pS29, DOI 10.1016/j.gie.2006.02.019
Bories E, 2007, ENDOSCOPY, V39, P287, DOI 10.1055/s-2007-966212
Brauer BC, 2009, GASTROINTEST ENDOSC, V70, P471, DOI 10.1016/j.gie.2008.12.233
Burmester E, 2003, GASTROINTEST ENDOSC, V57, P246, DOI 10.1067/mge.2003.85
Choudari CP, 2000, GASTROINTEST ENDOSC, V52, P478, DOI 10.1067/mge.2000.108972
Fabbri C, 2011, ENDOSCOPY, V43, P438, DOI 10.1055/s-0030-1256097
FERRUCCI JT, 1980, RADIOLOGY, V135, P1
Fujita N, 2008, J GASTROENTEROL, V43, P637, DOI 10.1007/s00535-008-2204-7
Fujita N, 2009, DIGEST ENDOSC, V21, P48, DOI 10.1111/j.1443-1661.2008.00821.x
Giovannini M, 2001, ENDOSCOPY, V33, P898, DOI 10.1055/s-2001-17324
Hanada K, 2009, DIGEST ENDOSC, V21, pS75, DOI 10.1111/j.1443-1661.2009.00855.x
Hara K, 2011, AM J GASTROENTEROL, V106, P1239, DOI 10.1038/ajg.2011.84

HARBIN WP, 1980, RADIOLOGY, V135, P15, DOI 10.1148/radiology.135.1.6987704
Horaguchi J, 2009, DIGEST ENDOSC, V21, P239, DOI 10.1111/j.1443-1661.2009.00899.x
Ito K, 2010, DIGEST ENDOSC, V22, pS132, DOI 10.1111/j.1443-1661.2010.00971.x
Itoi T, 2010, J HEPATO-BIL-PAN SCI, V17, P611, DOI 10.1007/s00534-009-0196-1
Kahaleh M, 2006, GASTROINTEST ENDOSC, V64, P52, DOI 10.1016/j.gie.2006.01.063
Kim YS, 2010, ENDOSCOPY, V42, P496, DOI 10.1055/s-0029-1244082
KUMAR S, 1995, GASTROINTEST ENDOSC, V41, P445, DOI 10.1016/S0016-5107(05)80001-X
Lai R, 2005, ENDOSCOPY, V37, P487, DOI 10.1055/s-2005-861250
Mallery S, 2004, GASTROINTEST ENDOSC, V59, P100, DOI 10.1016/S0016-5107(03)02300-9
Maranki J, 2009, ENDOSCOPY, V41, P532, DOI 10.1055/s-0029-1214712
Nguyen-Tang T, 2010, ENDOSCOPY, V42, P232, DOI 10.1055/s-0029-1243858
Park DH, 2011, GASTROINTEST ENDOSC, V74, P1276, DOI 10.1016/j.gie.2011.07.054
Püspök A, 2005, AM J GASTROENTEROL, V100, P1743, DOI 10.1111/j.1572-0241.2005.41806.x
Ramírez-Luna MA, 2011, ENDOSCOPY, V43, P826, DOI 10.1055/s-0030-1256406
SMITH AC, 1994, LANCET, V344, P1655, DOI 10.1016/S0140-6736(94)90455-3
Tarantino I, 2008, ENDOSCOPY, V40, P336, DOI 10.1055/s-2007-995455
Weber A, 2009, EUR J RADIOL, V72, P412, DOI 10.1016/j.ejrad.2008.08.012
Wiersema MJ, 1996, GASTROINTEST ENDOSC, V43, P102, DOI 10.1016/S0016-5107(06)80108-2
Will U, 2007, ENDOSCOPY, V39, P292, DOI 10.1055/s-2007-966215
Yamao K, 2008, ENDOSCOPY, V40, P340, DOI 10.1055/s-2007-995485
NR 33
TC 35
Z9 45
U1 0
U2 5
PU EDITORIAL OFFICE GUT & LIVER
PI SEOUL
PA 305 LOTTE GOLD ROSE II, 890-59, DAECHI 4-DONG, GANGNAM-GU, SEOUL,
135-839, SOUTH KOREA
SN 1976-2283
J9 GUT LIVER
JI Gut Liver
PD MAR 15
PY 2013
VL 7
IS 2
BP 129
EP 136
DI 10.5009/gnl.2013.7.2.129
PG 8
WC Gastroenterology & Hepatology
WE Science Citation Index Expanded (SCI-EXPANDED)

SC Gastroenterology & Hepatology
GA 108OD
UT WOS:000316304300001
PM 23560147
OA Green Submitted, gold
DA 2026-05-09
ER

PT J
AU Alshati, A
Srinivasan, I
Chuang, KY
AF Alshati, Ali
Srinivasan, Indu
Chuang, Keng-Yu
TI Spontaneous Closure of a Large Transmural Gastric Defect After Removal
of a Migrated AXIOS Stent
SO ACG CASE REPORTS JOURNAL
LA English
DT Article
ID PANCREATIC FLUID COLLECTIONS; APPOSING METAL STENT;
ENDOSCOPIC-ULTRASOUND; GUIDED DRAINAGE; PSEUDOCYST
AB Pancreatic fluid collections occur in 5%-15% of acute complicated pancreatitis cases. Endoscopic drainage using lumen-apposing metal stents has become the treatment of choice with less incidence of complications, shorter hospital stays, and less cost. The AXIOS stent has proven to be safe and effective in several studies. Despite a low complication rate, bleeding, perforation, and stent migration were still reported. In this case, a patient presented for delayed AXIOS stent removal and was found to have stent migration with embedment in the gastric wall. The transmural defect that resulted after endoscopic stent removal was observed to close spontaneously without further need for intervention.
C1 [Alshati, Ali] Creighton Univ, Maricopa Integrated Hlth Syst, Dept Internal Med, 2601 E Roosevelt St, Phoenix, AZ 85008 USA.
[Srinivasan, Indu; Chuang, Keng-Yu] Creighton Univ, Maricopa Integrated Hlth Syst, Dept Gastroenterol, Phoenix, AZ USA.
[Chuang, Keng-Yu] Creighton Univ, St Josephs Hosp & Med Ctr, Dept Gastroenterol, Phoenix, AZ USA.
C3 Maricopa County General Hospital; Maricopa Integrated Health System;
Creighton University; Maricopa County General Hospital; Maricopa
Integrated Health System; Creighton University; Creighton University;
St. Joseph's Hospital and Medical Center
RP Alshati, A (corresponding author), Creighton Univ, Maricopa Integrated Hlth Syst, Dept Internal Med, 2601 E Roosevelt St, Phoenix, AZ 85008 USA.
EM ali.alshati@mihs.org
CR Akshintala VS, 2014, GASTROINTEST ENDOSC, V79, P921, DOI 10.1016/j.gie.2013.10.032
Gornals JB, 2013, SURG ENDOSC, V27, P1428, DOI 10.1007/s00464-012-2591-y

Itoi T, 2012, GASTROINTEST ENDOSC, V75, P870, DOI 10.1016/j.gie.2011.10.020
Lopes CV, 2007, SCAND J GASTROENTERO, V42, P524, DOI 10.1080/00365520601065093
Patil R, 2016, ANN GASTROENTEROL, V29, P168, DOI 10.20524/aog.2016.0008
Poornachandra KS, 2011, J CLIN GASTROENTEROL, V45, P159, DOI 10.1097/MCG.0b013e3181dd9d14
Raijman Isaac, 2003, Gastrointest Endosc Clin N Am, V13, P561
Singhal S, 2013, CLIN ENDOSC, V46, P506, DOI 10.5946/ce.2013.46.5.506
Tyberg A, 2016, WORLD J GASTROENTERO, V22, P2256, DOI 10.3748/wjg.v22.i7.2256
Varadarajulu S, 2013, GASTROENTEROLOGY, V145, P583, DOI 10.1053/j.gastro.2013.05.046
Varadarajulu S, 2011, J GASTROINTEST SURG, V15, P2080, DOI 10.1007/s11605-011-1621-8
Varadarajulu S, 2011, J GASTROEN HEPATOL, V26, P1504, DOI 10.1111/j.1440-1746.2011.06771.x
Walter D, 2015, ENDOSCOPY, V47, P63, DOI 10.1055/s-0034-1378113
NR 13
TC 3
Z9 4
U1 0
U2 0
PU LIPPINCOTT WILLIAMS & WILKINS
PI PHILADELPHIA
PA TWO COMMERCE SQ, 2001 MARKET ST, PHILADELPHIA, PA 19103 USA
SN 2326-3253
J9 ACG CASE REP J
JI ACG Case Rep. J.
PD MAR
PY 2019
VL 6
IS 3
DI 10.14309/crj.0000000000000033
PG 3
WC Gastroenterology & Hepatology
WE Emerging Sources Citation Index (ESCI)
SC Gastroenterology & Hepatology
GA IK4OH
UT WOS:000476565700008
PM 31620499
OA Green Submitted, gold
DA 2026-05-09
ER

PT J
AU Saftoiu, A
Vilmann, A
Vilmann, P

AF Saftoiu, Adrian
Vilmann, Andreas
Vilmann, Peter
TI Endoscopic ultrasound-guided drainage of pancreatic pseudocysts
SO ENDOSCOPIC ULTRASOUND
LA English
DT Review
DE Endoscopic ultrasound (EUS); necrosectomy; pancreatic pseudocysts
ID WALLED-OFF NECROSIS; PERIPANCREATIC FLUID COLLECTIONS;
FINE-NEEDLE-ASPIRATION; EXPANDING METAL STENTS; DOUBLE-WIRE TECHNIQUE;
TRANSMURAL DRAINAGE; ONE-STEP; ECHO ENDOSCOPE; RANDOMIZED-TRIAL; CYSTIC
LESIONS
AB Pancreatic pseudocysts are fluid collections in the peripancreatic tissues associated with acute or chronic pancreatitis. Endoscopic ultrasound (EUS)-guided drainage has become an established indication, having better results as compared to percutaneous drainage, nonguided endoscopic drainage, or surgical drainage. The aim of this review is to assess critically the current literature concerning EUS-guided pseudocyst drainage and to review the place of the procedure in the clinical decision management algorithms of these patients.
C1 [Saftoiu, Adrian; Vilmann, Andreas; Vilmann, Peter] Copenhagen Univ Hosp, Gastro Unit, Dept Endoscopy, Herlev, Denmark.
[Saftoiu, Adrian] Univ Med & Pharm, Res Ctr Gastroenterol & Hepatol Craiova, Dept Gastroenterol, Craiova, Romania.
C3 University of Copenhagen; Copenhagen University Hospital; University of
Medicine & Pharmacy of Craiova
RP Saftoiu, A (corresponding author), 2 Petru Rares St, Dolj 200349, Craiova, Romania.
EM adrian.saftoiu@umfcv.ro
RI Vilmann, Peter/AAJ-8401-2020; Saftoiu, Adrian/C-2792-2011
OI Saftoiu, Adrian/0000-0001-7993-8269
CR Ahlawat SK, 2006, J PANCREAS, V7, P616
Andren-Sandberg Ake, 2004, JOP, V5, P64
Andren-Sandberg Ake, 2004, JOP, V5, P8
Ang TL, 2008, GASTROINTEST ENDOSC, V68, P192, DOI 10.1016/j.gie.2007.11.018
Antillon MR, 2006, GASTROINTEST ENDOSC, V63, P797, DOI 10.1016/j.gie.2005.10.025
Azar RR, 2006, GASTROINTEST ENDOSC, V63, P688, DOI 10.1016/j.gie.2005.10.032
Bang JY, 2014, BRIT J SURG, V101, P1729, DOI 10.1002/bjs.9664
Bang JY, 2014, CLIN ENDOSC, V47, P429, DOI 10.5946/ce.2014.47.5.429
Banks PA, 2013, GUT, V62, P102, DOI 10.1136/gutjnl-2012-302779
Baron TH, 2000, GASTROINTEST ENDOSC, V52, P545, DOI 10.1067/mge.2000.108928
Binmoeller KF, 2013, SURG ENDOSC, V27, P1835, DOI 10.1007/s00464-012-2682-9
Brugge WR, 2001, PANCREATOLOGY, V1, P637, DOI 10.1159/000055874
Costache MI, 2013, ENDOSC ULTRASOUND, V2, P77, DOI [10.7178/eus.05.004, 10.4103/2303-9027.117691]
Fabbri C, 2012, ENDOSCOPY, V44, P429, DOI 10.1055/s-0031-1291624
Fabbri C, 2014, WORLD J GASTROENTERO, V20, P8424, DOI 10.3748/wjg.v20.i26.8424
Fockens P, 2002, GASTROINTEST ENDOSC, V56, pS93, DOI 10.1067/mge.2002.127756

Fusaroli P, 2011, J GASTROINTEST LIVER, V20, P216
Giovannini M, 1998, GASTROINTEST ENDOSC, V48, P200, DOI 10.1016/S0016-5107(98)70165-8
Giovannini M, 2001, ENDOSCOPY, V33, P473, DOI 10.1055/s-2001-14967
Giovannini M, 2007, TECH GASTROINTEST EN, V9, P32, DOI 10.1016/j.tgie.2006.11.013
Itoi T, 2013, J HEPATO-BIL-PAN SCI, V20, P403, DOI 10.1007/s00534-012-0551-5
Itoi T, 2012, GASTROINTEST ENDOSC, V75, P870, DOI 10.1016/j.gie.2011.10.020
Jacobson BC, 2005, GASTROINTEST ENDOSC, V61, P363, DOI 10.1016/S0016-5107(04)02779-8
Kahaleh M, 2006, ENDOSCOPY, V38, P355, DOI 10.1055/s-2006-925249
Khashab MA, 2012, SURG ENDOSC, V26, P3320, DOI 10.1007/s00464-012-2297-1
Krüger M, 2006, GASTROINTEST ENDOSC, V63, P409, DOI 10.1016/j.gie.2005.11.047
Lopes CV, 2007, SCAND J GASTROENTERO, V42, P524, DOI 10.1080/00365520601065093
Mönkemüller KE, 1998, GASTROINTEST ENDOSC, V48, P195, DOI 10.1016/S0016-5107(98)70164-6
Mukai S, 2015, J GASTROENTEROL, V50, P211, DOI 10.1007/s00535-014-0957-8
Mukai S, 2015, ENDOSCOPY, V47, P47, DOI 10.1055/s-0034-1377966
Ng PY, 2013, ENDOSC ULTRASOUND, V2, P199, DOI 10.4103/2303-9027.121245
Panamonta N, 2012, EUR J GASTROEN HEPAT, V24, P1355, DOI 10.1097/MEG.0b013e32835871eb
Penn DE, 2012, GASTROINTEST ENDOSC, V76, P679, DOI 10.1016/j.gie.2012.04.457
Piraka C, 2009, GASTROINTEST ENDOSC, V70, P786, DOI 10.1016/j.gie.2009.04.049
Puli SR, 2014, CAN J GASTROENTEROL, V28, P50, DOI 10.1155/2014/539783
Rische S, 2013, SCAND J GASTROENTERO, V48, P231, DOI 10.3109/00365521.2012.752029
Saftoiu A, 2006, ENDOSCOPY, V38, P538, DOI 10.1055/s-2006-925229
Saftoiu A, 2013, BMC GASTROENTEROL, V13, DOI 10.1186/1471-230X-13-93
Seewald S, 2005, GASTROINTEST ENDOSC, V62, P92, DOI 10.1016/S0016-5107(05)00541-9
Seewald S, 2006, GASTROINTEST ENDOSC, V64, P805, DOI 10.1016/j.gie.2006.07.049
Seifert H, 2000, ENDOSCOPY, V32, P255, DOI 10.1055/s-2000-93
Singhal S, 2013, CLIN ENDOSC, V46, P506, DOI 10.5946/ce.2013.46.5.506
Sriram PVJ, 2005, ENDOSCOPY, V37, P231, DOI 10.1055/s-2005-860997
Trevino JM, 2009, GASTROINTEST ENDOSC, V70, P793, DOI 10.1016/j.gie.2009.05.023
Varadarajulu S, 2008, GASTROINTEST ENDOSC, V68, P1102, DOI 10.1016/j.gie.2008.04.028
Varadarajulu S, 2008, GASTROINTEST ENDOSC, V68, P656, DOI 10.1016/j.gie.2008.03.1091
Varadarajulu S, 2008, GASTROINTEST ENDOSC, V68, P649, DOI 10.1016/j.gie.2008.02.057
Varadarajulu S, 2007, GASTROINTEST ENDOSC, V66, P1107, DOI 10.1016/j.gie.2007.03.1027
Varadarajulu S, 2013, GASTROENTEROLOGY, V145, P583, DOI 10.1053/j.gastro.2013.05.046
Vilmann P, 1998, ENDOSCOPY, V30, P730, DOI 10.1055/s-2007-1001399
VILMANN P, 1992, GASTROINTEST ENDOSC, V38, P172, DOI 10.1016/S0016-5107(92)70385-X
Voermans RP, 2007, GASTROINTEST ENDOSC, V66, P1013, DOI 10.1016/j.gie.2007.02.057
Wiersema MJ, 2001, GASTROINTEST ENDOSC, V53, P811, DOI 10.1067/mge.2001.113272
NR 53
TC 18
Z9 23
U1 0
U2 4

PU MEDKNOW PUBLICATIONS & MEDIA PVT LTD
PI MUMBAI
PA B-9, KANARA BUSINESS CENTRE, OFF LINK RD, GHAKTOPAR-E, MUMBAI, 400075,
INDIA
SN 2303-9027
EI 2226-7190
J9 ENDOSC ULTRASOUND
JI Endosc. Ultrasound
PD OCT-DEC
PY 2015
VL 4
IS 4
BP 319
EP 323
DI 10.4103/2303-9027.170424
PG 5
WC Gastroenterology & Hepatology
WE Science Citation Index Expanded (SCI-EXPANDED)
SC Gastroenterology & Hepatology
GA CX5WB
UT WOS:000365772100009
PM 26643700
OA Green Submitted
DA 2026-05-09
ER

PT J
AU Teoh, AYB
AF Teoh, Anthony Yuen Bun
TI Endoscopic ultrasound-guided anastomosis: Is it ready for prime time?
SO JOURNAL OF GASTROENTEROLOGY AND HEPATOLOGY
LA English
DT Review
DE Endoscopic ultrasound; EUS-guided anastomosis; Lumen apposing metallic
stents
ID APPOSING METAL STENTS; TRANSHEPATIC GALLBLADDER DRAINAGE; BILIARY
DRAINAGE; PANCREATIC PSEUDOCYSTS; TRANSMURAL DRAINAGE; EUS;
GASTROJEJUNOSTOMY; MULTICENTER; GASTROENTEROSTOMY; INTERVENTIONS
AB Significant advancements in endoscopic ultrasound (EUS)-guided drainage procedures and EUS-guided anastomosis have recently been described. The described procedures include EUS-guided drainage of pancreatic fluid collections, biliary drainage, pancreatic duct drainage, gallbladder drainage, and gastroenterostomy. These procedures have been made possible with novel devices that can reduce the difficulties of the procedures and potentially reducing the risk of adverse events. The

procedures are also becoming standardized, which is essential for dissemination of the techniques. Furthermore, results from randomized studies are becoming available showing definite benefits associated with these procedures. In this paper, we will provide a review on EUS-guided anastomotic procedures.

C1 [Teoh, Anthony Yuen Bun] Chinese Univ Hong Kong, Prince Wales Hosp, Dept Surg, Shatin, Hong Kong, Peoples R China.
C3 Chinese University of Hong Kong; Prince of Wales Hospital Hong Kong
RP Teoh, AYB (corresponding author), Chinese Univ Hong Kong, Prince Wales Hosp, Dept Surg, Shatin, Hong Kong, Peoples R China.
EM anthonyteoh@surgery.cuhk.edu.hk
RI Teoh, Anthony/L-4796-2016
OI Teoh, Anthony/0000-0002-1885-8035
CR Bang JY, 2019, GASTROENTEROLOGY, V156, P1027, DOI 10.1053/j.gastro.2018.11.031
Bang JY, 2019, GUT, V68, P1200, DOI 10.1136/gutjnl-2017-315335
Bang JY, 2017, GUT, V66, P2054, DOI 10.1136/gutjnl-2016-312812
Baron TH, 2007, GASTROINTEST ENDOSC, V65, P735, DOI 10.1016/j.gie.2006.07.041
Binmoeller KF, 2011, ENDOSCOPY, V43, P337, DOI 10.1055/s-0030-1256127
Brimhall B, 2018, CLIN GASTROENTEROL H, V16, P1521, DOI 10.1016/j.cgh.2018.02.021
Bukhari M, 2018, GASTROINTEST ENDOSC, V88, P486, DOI 10.1016/j.gie.2018.04.2356
Chan SM, 2017, GASTROINTEST ENDOSC, V85, P1225, DOI 10.1016/j.gie.2016.10.014
Chen YI, 2017, SURG ENDOSC, V31, P2946, DOI 10.1007/s00464-016-5311-1
Dhir V, 2015, GASTROINTEST ENDOSC, V82, P650, DOI 10.1016/j.gie.2015.01.061
François E, 2002, GASTROINTEST ENDOSC, V56, P128, DOI 10.1067/mge.2002.125547
Ge PS, 2019, SURG ENDOSC, V33, P3404, DOI 10.1007/s00464-018-06636-3
Giovannini M, 2001, ENDOSCOPY, V33, P898, DOI 10.1055/s-2001-17324
GRIMM H, 1992, GASTROINTEST ENDOSC, V38, P170, DOI 10.1016/S0016-5107(92)70384-8
Gutierrez OIB, 2018, ENDOSCOPY, V50, P891, DOI 10.1055/s-0044-102254
Hedjoudje A, 2019, UNITED EUR GASTROENT, V7, P60, DOI 10.1177/2050640618808147
Irani S, 2017, CLIN GASTROENTEROL H, V15, P738, DOI 10.1016/j.cgh.2016.12.021
Itoi T, 2016, GUT, V65, P193, DOI 10.1136/gutjnl-2015-310348
James TW, 2020, GASTROINTEST ENDOSC, V91, P537, DOI 10.1016/j.gie.2019.11.017
Jang JW, 2012, GASTROENTEROLOGY, V142, P805, DOI 10.1053/j.gastro.2011.12.051
Jin Z, 2020, DIGEST ENDOSC, V32, P16, DOI 10.1111/den.13456
Kedia P, 2015, GASTROINTEST ENDOSC, V82, P560, DOI 10.1016/j.gie.2015.03.1913
Khan MA, 2016, DIGEST DIS SCI, V61, P684, DOI 10.1007/s10620-015-3933-0
Khashab MA, 2017, ENDOSC INT OPEN, V5, pE275, DOI 10.1055/s-0043-101695
Krishnamoorthi R, 2020, SURG ENDOSC, V34, P1904, DOI 10.1007/s00464-020-07409-7
Logiudice FP, 2019, WORLD J GASTRO ENDOS, V11, P281, DOI 10.4253/wjge.v11.i4.281
Lou X, 2019, MINERVA MED, V110, P564, DOI 10.23736/S0026-4806.19.05981-0
Luk SWY, 2019, ENDOSCOPY, V51, P722, DOI 10.1055/a-0929-6603
Miller CS, 2019, ENDOSC INT OPEN, V7, pE1563, DOI 10.1055/a-0998-8129
Park DH, 2009, ENDOSCOPY, V41, P842, DOI 10.1055/s-0029-1215133
Park DH, 2015, J GASTROEN HEPATOL, V30, P1461, DOI 10.1111/jgh.13027

Parsa N, 2020, ENDOSCOPY, V52, P211, DOI 10.1055/a-1096-3299
Perez-Miranda M, 2017, J CLIN GASTROENTEROL, V51, P896, DOI 10.1097/MCG.0000000000000887
Perez-Miranda M, 2016, GASTROINTEST ENDOSC, V83, pAB175, DOI 10.1016/j.gie.2016.03.155
Sharaiha RZ, 2016, CLIN GASTROENTEROL H, V14, P1797, DOI 10.1016/j.cgh.2016.05.011
Song TJ, 2019, GASTROINTEST ENDOSC, V90, P507, DOI 10.1016/j.gie.2019.05.033
Teoh AY, 2016, GASTROINTEST ENDOSC, V84, P62, DOI 10.1016/j.gie.2015.11.041
Teoh AYB, 2020, DIGEST ENDOSC, V32, P391, DOI 10.1111/den.13494
Teoh AYB, 2017, ENDOSCOPY, V49, P130, DOI 10.1055/s-0042-119036
Teoh AYB, 2016, WORLD J GASTRO ENDOS, V8, P310, DOI 10.4253/wjge.v8.i6.310
Teoh AYB, 2014, GASTROINTEST ENDOSC, V80, P1171, DOI 10.1016/j.gie.2014.03.038
TEOH AYB, 2018, GUT
Teoh AYB, 2020, GUT
Tyberg A, 2018, J CLIN GASTROENTEROL, V52, P79, DOI 10.1097/MCG.0000000000000786
van Brunschot S, 2018, LANCET, V391, P51, DOI 10.1016/S0140-6736(17)32404-2
Varadarajulu S, 2008, GASTROINTEST ENDOSC, V68, P1102, DOI 10.1016/j.gie.2008.04.028
Varadarajulu S, 2013, GASTROENTEROLOGY, V145, P583, DOI 10.1053/j.gastro.2013.05.046
NR 47
TC 6
Z9 6
U1 0
U2 3
PU WILEY
PI HOBOKEN
PA 111 RIVER ST, HOBOKEN 07030-5774, NJ USA
SN 0815-9319
EI 1440-1746
J9 J GASTROEN HEPATOL
JI J. Gastroenterol. Hepatol.
PD AUG
PY 2020
VL 35
IS 8
BP 1288
EP 1293
DI 10.1111/jgh.15099
EA MAY 2020
PG 6
WC Gastroenterology & Hepatology
WE Science Citation Index Expanded (SCI-EXPANDED)
SC Gastroenterology & Hepatology
GA MT4YS
UT WOS:000535306900001

PM 32415861
DA 2026-05-09
ER

PT J
AU Binda, C
Sbrancia, M
La Marca, M
Colussi, D
Vizzuso, A
Tomasoni, M
Agnoletti, V
Giampalma, E
Ansaloni, L
Fabbri, C
AF Binda, Cecilia
Sbrancia, Monica
La Marca, Marina
Colussi, Dora
Vizzuso, Antonio
Tomasoni, Matteo
Agnoletti, Vanni
Giampalma, Emanuela
Ansaloni, Luca
Fabbri, Carlo
TI EUS-guided drainage using lumen apposing metal stent and percutaneous
endoscopic necrosectomy as dual approach for the management of complex
walled-off necrosis: a case report and a review of the literature
SO WORLD JOURNAL OF EMERGENCY SURGERY
LA English
DT Review
DE Necrotizing pancreatitis; Percutaneous endoscopic necrosectomy;
Walled-off pancreatic necrosis; Endoscopic necrosectomy; Lumen-apposing
metal stent
ID SINUS TRACT ENDOSCOPY; PANCREATIC NECROSIS; NECROTIZING PANCREATITIS; UP
APPROACH; SINGLE
AB Background Endoscopic ultrasound-guided drainage is suggested as the first approach in the management of symptomatic and complex walled-off pancreatic necrosis. Dual approach with percutaneous drainage could be the best choice when the necrosis is deep extended till the pelvic paracolic gutter; however, the available catheter could not be large enough to drain solid necrosis neither to perform necrosectomy, entailing a higher need for surgery. Therefore, percutaneous endoscopic necrosectomy through a large bore percutaneous self-expandable metal stent has been proposed. Case presentation In this study, we present the case of a 61-year-old man admitted to our hospital with a history of sepsis and

persistent multiorgan failure secondary to walled-off pancreatic necrosis due to acute necrotizing pancreatitis. Firstly, the patient underwent transgastric endoscopic ultrasound-guided drainage using a lumen-apposing metal stent and three sessions of direct endoscopic necrosectomy. Because of recurrence of multiorgan failure and the presence of the necrosis deeper to the pelvic paracolic gutter at computed tomography scan, we decided to perform percutaneous endoscopic necrosectomy using an esophageal self-expandable metal stent. After four sessions of necrosectomy, the collection was resolved without complications. Therefore, we perform a revision of the literature, in order to provide the state-of-art on this technique. The available data are, to date, derived by case reports and case series, which showed high rates both of technical and clinical success. However, a not negligible rate of adverse events has been reported, mainly represented by fistulas and abdominal pain. Conclusion Dual approach, using lumen apposing metal stent and percutaneous self-expandable metal stent, is a compelling option of treatment for patients affected by symptomatic, complex walled-off pancreatic necrosis, allowing to directly remove large amounts of necrosis avoiding surgery. Percutaneous endoscopic necrosectomy seems a promising technique that could be part of the step-up-approach, before emergency surgery. However, to date, it should be reserved in referral centers, where a multidisciplinary team is disposable.
C1 [Binda, Cecilia; Sbrancia, Monica; Colussi, Dora; Fabbri, Carlo] AUSL Romagna, Forli Cesena Hosp, Gastroenterol & Digest Endoscopy Unit, Forli Cesena, Italy.

[La Marca, Marina] St Orsola Marcello Malpighi Hosp, Dept Med & Surg Sci, Gastroenterol Unit, Bologna, Italy.

[Vizzuso, Antonio] AUSL Romagna, Morgagni Pierantoni Hosp, Radiol Unit, Forli, Italy.

[Tomasoni, Matteo; Ansaloni, Luca] Osped Gen Provinciale M Bufalini, Gen Emergency & Trauma Surg Dept, Cesena, Italy.

[Agnoletti, Vanni] AUSL Romagna, M Bufalini Hosp, Anesthesia & Intens Care Unit, Cesena, Italy.

[Giampalma, Emanuela] AUSL Romagna, M Bufalini Hosp, Radiol Unit, Cesena, Italy.

C3 AUSL della Romagna; IRCCS Azienda Ospedaliero-Universitaria di Bologna;
AUSL della Romagna; AUSL della Romagna; AUSL della Romagna

RP Binda, C (corresponding author), AUSL Romagna, Forli Cesena Hosp, Gastroenterol & Digest Endoscopy Unit, Forli Cesena, Italy.

EM cecilia.binda@gmail.com

RI ; Fabbri, Carlo/AAC-4467-2022; Agnoletti, Vanni/AAC-9854-2022; Binda, Cecilia/ABH-1588-2020; Tomasoni, Matteo/AAC-3732-2022

OI Ansaloni, Luca/0000-0001-6427-0307; Fabbri, Carlo/0000-0003-0670-9910; Agnoletti, Vanni/0000-0002-7093-2749; La Marca, Marina/0000-0003-1040-0529; Vizzuso, Antonio/0000-0001-9823-3336;

CR Arvanitakis M, 2018, ENDOSCOPY, V50, P524, DOI 10.1055/a-0588-5365

Bakken JC., 2011, Gastrointest Endosc, V73, pAB103, DOI [10.1016/j.gie.2011.03.1217, DOI 10.1016/J.GIE.2011.03.1217]

Bakken JC., 2011, Endoscopy, V43, pA69

Bang JY, 2020, DIGEST ENDOSC, V32, P298, DOI 10.1111/den.13470

Bang JY, 2019, GASTROENTEROLOGY, V156, P1027, DOI 10.1053/j.gastro.2018.11.031

Banks PA, 2013, GUT, V62, P102, DOI 10.1136/gutjnl-2012-302779

Baron TH, 2020, GASTROENTEROLOGY, V158, P67, DOI 10.1053/j.gastro.2019.07.064

Carter CR, 2000, ANN SURG, V232, P175, DOI 10.1097/00000658-200008000-00004

Cerecedo-Rodriguez J, 2014, GASTROINTEST ENDOSC, V80, P165, DOI 10.1016/j.gie.2014.04.009

D'Souza LS, 2017, ENDOSCOPY, V49, pE242, DOI 10.1055/s-0043-114407

Dhingra R, 2015, GASTROINTEST ENDOSC, V81, P351, DOI 10.1016/j.gie.2014.07.060
Fugazza A, 2020, GASTROINTEST ENDOSC, V91, P574, DOI 10.1016/j.gie.2019.11.021
Goenka MK, 2018, CLIN ENDOSC, V51, P279, DOI 10.5946/ce.2017.066
Jain S, 2020, DIGEST DIS SCI, V65, P615, DOI 10.1007/s10620-019-05696-2
Ke L, 2019, WORLD J SURG, V43, P1121, DOI 10.1007/s00268-018-04878-9
Kedia P, 2015, GASTROINTEST ENDOSC, V81, P1252, DOI 10.1016/j.gie.2014.07.043
Mathers B, 2016, ENDOSC INT OPEN, V4, pE102, DOI 10.1055/s-0041-107802
Moyer Matthew T, 2019, VideoGIE, V4, P389, DOI [10.1016/j.vgie.2019.04.005, 10.1016/j.vgie.2019.04.005]
Mui LM, 2005, SURG ENDOSC, V19, P393, DOI 10.1007/s00464-004-9120-6
Navarrete C, 2011, GASTROINTEST ENDOSC, V73, P609, DOI 10.1016/j.gie.2010.08.015
Pérez-Cuadrado-Robles E, 2020, ENDOSCOPY, V52, pE88, DOI 10.1055/a-1011-3555
Ross AS, 2014, GASTROINTEST ENDOSC, V79, P929, DOI 10.1016/j.gie.2013.10.014
Sato Satoshi, 2016, Semin Arthritis Rheum, V46, pe13, DOI [10.1016/j.semarthrit.2016.05.003, 10.1016/j.semarthrit.2016.05.003]
Saumoy M, 2018, J CLIN GASTROENTEROL, V52, P458, DOI 10.1097/MCG.0000000000000895
Thorsen A, 2018, DIGEST DIS SCI, V63, P2456, DOI 10.1007/s10620-018-5131-3
Tringali A, 2018, ENDOSC INT OPEN, V6, pE274, DOI 10.1055/s-0043-125313
van Brunschot S, 2018, LANCET, V391, P51, DOI 10.1016/S0140-6736(17)32404-2
van Dijk SM, 2017, GUT, V66, P2024, DOI 10.1136/gutjnl-2016-313595
van Santvoort HC, 2010, NEW ENGL J MED, V362, P1491, DOI 10.1056/NEJMoa0908821
Yamamoto N, 2013, ENDOSCOPY, V45, pE44, DOI [10.1055/s0032-1309927, 10.1055/s-0032-1309927]
Zuber-Jerger Ina, 2007, Clin Gastroenterol Hepatol, V5, pe45, DOI 10.1016/j.cgh.2007.09.006
NR 31
TC 15
Z9 18
U1 0
U2 6
PU BMC
PI LONDON
PA CAMPUS, 4 CRINAN ST, LONDON N1 9XW, ENGLAND
SN 1749-7922
J9 WORLD J EMERG SURG
JI World J. Emerg. Surg.
PD JUN 2
PY 2021
VL 16
IS 1
AR 28
DI 10.1186/s13017-021-00367-y
PG 8
WC Emergency Medicine; Surgery
WE Science Citation Index Expanded (SCI-EXPANDED)

SC Emergency Medicine; Surgery
GA SM0MI
UT WOS:000657306600001
PM 34078409
OA Green Submitted, gold
DA 2026-05-09
ER

PT J
AU Chang, LK
Chen, KC
Cheng, MF
Lin, CC
Wang, HP
Sung, CT
Chen, JH
Yen, RF
Hsu, CL
Shih, SR
AF Chang, Ling-Kai
Chen, Kuan-Chih
Cheng, Mei-Fang
Lin, Chia-Chi
Wang, Hsiu-Po
Sung, Chia-Tung
Chen, Jou-Ho
Yen, Ruoh-Fang
Hsu, Chia-Lin
Shih, Shyang-Rong
TI Endoscopic ultrasound ablation in a patient with multiple metastatic pancreatic tumors from adrenocorticotropic hormone-producing thymic neuroendocrine neoplasm
SO DIGESTIVE ENDOSCOPY
LA English
DT Article
DE Ga-68-DOTATOC scan; Cushing's syndrome; neuroendocrine neoplasm; pancreas; somatostatin receptor; thymus
AB Adrenocorticotropic hormone (ACTH)-producing neuroendocrine neoplasm (NEN) of the thymus is rare. Lymph nodes and bones are the most common metastatic sites. Most cases present with florid Cushing's syndrome (CS). Here, we reported a 58-year-old woman, who presented with intermittent flush and weight loss. Imaging studies revealed tumors in the mediastinum, pancreas, and bones. Contrast-enhanced harmonic endoscopic ultrasound (EUS) of the pancreatic tumors showed heterogeneous and hyperenhancing characteristics. EUS elastography revealed a heterogeneous stiff pattern. EUS-

fine needle biopsy to the pancreatic lesion confirmed the NEN nature. Serum ACTH and cortisol levels were abnormally high. Immunohistochemical staining of the thymic and pancreatic specimens was positive for ACTH. However, the patient did not have obvious CS appearance. The patient underwent surgery, radiation, EUS-guided ethanol injection, and anti-cancer medications, but the disease still progressed. The patient died from infection 16 months after NEN was diagnosed. In conclusion, the pancreas can be a metastatic site for ACTH-producing thymic NEN. EUS-associated procedures can help in the diagnosis and treatment of pancreatic metastatic NEN.
C1 [Chang, Ling-Kai; Wang, Hsiu-Po; Chen, Jou-Ho; Hsu, Chia-Lin; Shih, Shyang-Rong] Natl Taiwan Univ Hosp, Dept Internal Med, Taipei, Taiwan.

[Cheng, Mei-Fang; Yen, Ruoh-Fang] Natl Taiwan Univ Hosp, Dept Nucl Med, Taipei, Taiwan.

[Lin, Chia-Chi] Natl Taiwan Univ Hosp, Dept Oncol, Taipei, Taiwan.

[Sung, Chia-Tung] Natl Taiwan Univ Hosp, Dept Pathol, Taipei, Taiwan.

[Shih, Shyang-Rong] Natl Taiwan Univ Hosp, Ctr Antiaging & Hlth Consultat, Taipei, Taiwan.

[Lin, Chia-Chi] Natl Taiwan Univ, Coll Med, Grad Inst Clin Med, Taipei, Taiwan.

[Shih, Shyang-Rong] Natl Taiwan Univ, Coll Med, Dept Internal Med, Taipei, Taiwan.

[Sung, Chia-Tung] Natl Taiwan Univ, Med Ctr, Inst Forens Med, Taipei, Taiwan.

[Chen, Kuan-Chih] Far Eastern Mem Hosp, Dept Internal Med, Div Gastroenterol & Hepatol, New Taipei, Taiwan.

C3 National Taiwan University; National Taiwan University Hospital;

National Taiwan University; National Taiwan University Hospital;

National Taiwan University; National Taiwan University Hospital;

National Taiwan University; National Taiwan University Hospital;

National Taiwan University; National Taiwan University Hospital;

National Taiwan University; National Taiwan University; National Taiwan University; Far Eastern Memorial Hospital

RP Hsu, CL; Shih, SR (corresponding author), Natl Taiwan Univ Hosp, 7 Chung Shan South Rd, Taipei, Taiwan.

EM clhsu7@ntu.edu.tw; srshih@ntu.edu.tw

RI Shiao, Chih-Chung/AAM-2716-2020; Liao, Chun-Hsing/HTQ-5271-2023; CHENG, MEI-FANG/AAV-8665-2021

OI Chang, Ling-Kai/0000-0002-6112-2322; YEN, RUOH-FANG/0000-0003-1648-6482; SHIH, SHYANG-RONG/0000-0001-7424-1358; CHENG, MEI-FANG/0000-0002-9359-0606
CR Bohnenberger H, 2017, J THORAC DIS, V9, pS1448, DOI 10.21037/jtd.2017.02.02

Buxbaum J, 2020, CLIN GASTROENTEROL H, V18, P917, DOI 10.1016/j.cgh.2019.08.054

Dietrich CF, 2019, CLIN ENDOSC, V52, P533

Krenning EP, 1999, ITAL J GASTROENTEROL, V31, pS219

Modlin IM, 2017, J THORAC DIS, V9, pS1458, DOI 10.21037/jtd.2017.03.82

Neary NM, 2012, J CLIN ENDOCR METAB, V97, P2223, DOI 10.1210/jc.2011-3355

Oronsky B, 2017, NEOPLASIA, V19, P991, DOI 10.1016/j.neo.2017.09.002

Rimbas M, 2020, DIGEST ENDOSC, V32, P1031, DOI 10.1111/den.13635

Tseng IC, 2015, AM J SURG PATHOL, V39, P850, DOI 10.1097/PAS.0000000000000435

Weissferdt A, 2013, MODERN PATHOL, V26, P1554, DOI 10.1038/modpathol.2013.111
NR 10

TC 3

Z9 3
U1 0
U2 5
PU WILEY
PI HOBOKEN
PA 111 RIVER ST, HOBOKEN 07030-5774, NJ USA
SN 0915-5635
EI 1443-1661
J9 DIGEST ENDOSC
JI Dig. Endosc.
PD MAR
PY 2021
VL 33
IS 3
BP 458
EP 463
DI 10.1111/den.13752
EA AUG 2020
PG 6
WC Gastroenterology & Hepatology; Surgery
WE Science Citation Index Expanded (SCI-EXPANDED)
SC Gastroenterology & Hepatology; Surgery
GA QR9HK
UT WOS:000560231400001
PM 32462727
DA 2026-05-09
ER

PT J
AU Mack, S
Galasso, D
Marx, M
Robert, M
Romailler, E
Oumrani, SH
Aslan, N
Moschouri, E
Schoepfer, A
Godat, S
AF Mack, Sahar
Galasso, Domenico
Marx, Mariola

Robert, Maxime
Romailler, Elodie
Oumrani, Sarra Hadjer
Aslan, Nurullah
Moschouri, Eleni
Schoepfer, Alain
Godat, Sebastien
TI Efficacy and safety of endoscopic ultrasound guided drainage of pseudocysts and walled-off necrosis after failure of percutaneous drainage
SO SURGICAL ENDOSCOPY AND OTHER INTERVENTIONAL TECHNIQUES
LA English
DT Article
DE Endoscopic ultrasound; Endoscopic ultrasound guided drainage; Percutaneous drainage; Pseudocyst; Pancreatic collections; Walled of necrosis
ID PANCREATIC FLUID COLLECTIONS; APPOSING METAL STENTS; MANAGEMENT; EUS; CONSENSUS
AB Background Endoscopic Ultrasound (EUS) represents the gold standard for initial drainage of pancreatic fluid collections (PFC) due to various etiologies. However, data concerning salvage EUS drainage after initial percutaneous drainage are limited. The purpose of our study was to evaluate the clinical outcomes and safety of EUS-guided drainage of pancreatic collections after failure of percutaneous drainage.

Methods This retrospective study was conducted in a single, tertiary university center from August 2013 to January 2020. Indication was pancreatic collection after acute pancreatitis with PFC requiring EUS-guided drainage after failure of percutaneous drainage.

Results Twenty-two patients with PFC after acute pancreatitis were included (mean age 64.1 +/- 11.3 years) of which 4/22 (18.2%) had pancreatic pseudocyst and 18/22 (81.8%) presented with a walled-off necrosis. Seventy-six interventions were performed among the 22 patients. Lumen-Apposing Metal Stent (LAMS) were used in 5/22 (22.7%) and double pigtail plastic stents in 17/22 (77.3%) of interventions with a median number intervention of 3 per patient (range 1 to 7). Technical success rate was 98.7% (75/76) with an overall clinical success of 81.8% (18/22). Procedure related adverse events rate was 9.1% (2/22) including one bleeding and one pancreatic fistula. Two non-procedure related deaths were observed.

Conclusion EUS-guided pancreatic collection drainage is clinically effective and safe after clinical/technical failure of radiological percutaneous management.

[GRAPHICS]

.
C1 [Mack, Sahar; Marx, Mariola; Robert, Maxime; Romailler, Elodie; Oumrani, Sarra Hadjer; Aslan, Nurullah; Moschouri, Eleni; Schoepfer, Alain; Godat, Sebastien] CHU Vaudois, Dept Med Specialties, Div Gastroenterol & Hepatol, Univ Hosp Lausanne, Rue Bugnon 46, CH-1011 Lausanne, Switzerland.

[Galasso, Domenico] Hop Riviera Chablais, HRC, Div Gastroenterol, Rennaz, Switzerland.
C3 University of Lausanne; Centre Hospitalier Universitaire Vaudois (CHUV)
RP Godat, S (corresponding author), CHU Vaudois, Dept Med Specialties, Div Gastroenterol & Hepatol, Univ Hosp

Lausanne, Rue Bugnon 46, CH-1011 Lausanne, Switzerland.
EM sebastien.godat@chuv.ch
CR Akshintala VS, 2014, GASTROINTEST ENDOSC, V79, P921, DOI 10.1016/j.gie.2013.10.032
Arvanitakis M, 2018, ENDOSCOPY, V50, P524, DOI 10.1055/a-0588-5365
Bang JY, 2019, GASTROENTEROLOGY, V156, P1027, DOI 10.1053/j.gastro.2018.11.031
Banks PA, 2013, GUT, V62, P102, DOI 10.1136/gutjnl-2012-302779
Brimhall B, 2018, CLIN GASTROENTEROL H, V16, P1521, DOI 10.1016/j.cgh.2018.02.021
Chandrasekhara V, 2017, GASTROINTEST ENDOSC, V85, P32, DOI 10.1016/j.gie.2016.06.051
Cotton PB, 2010, GASTROINTEST ENDOSC, V71, P446, DOI 10.1016/j.gie.2009.10.027
Dhir V, 2015, GASTROINTEST ENDOSC, V82, P650, DOI 10.1016/j.gie.2015.01.061
Dumonceau JM, 2019, ENDOSCOPY, V51, P179, DOI 10.1055/a-0822-0832
Guo JT, 2017, ENDOSC ULTRASOUND, V6, P285, DOI 10.4103/eus.eus_85_17
Horvath K, 2010, ARCH SURG-CHICAGO, V145, P817, DOI 10.1001/archsurg.2010.178
Isayama H, 2016, J GASTROEN HEPATOL, V31, P1555, DOI 10.1111/jgh.13398
Itoi T, 2012, GASTROINTEST ENDOSC, V75, P870, DOI 10.1016/j.gie.2011.10.020
Johnson MD, 2009, J CLIN GASTROENTEROL, V43, P586, DOI 10.1097/MCG.0b013e31817440be
Keane MG, 2016, SURG ENDOSC, V30, P3730, DOI 10.1007/s00464-015-4668-x
Khan MA, 2018, ENDOSC INT OPEN, V6, pE474, DOI 10.1055/s-0044-102299
Kwon YM, 2013, SURG ENDOSC, V27, P2422, DOI 10.1007/s00464-012-2752-z
Lang GD, 2018, GASTROINTEST ENDOSC, V87, P150, DOI 10.1016/j.gie.2017.06.029
Rinninella E, 2015, GASTROINTEST ENDOSC, V82, P1039, DOI 10.1016/j.gie.2015.04.006
Shah RJ, 2015, CLIN GASTROENTEROL H, V13, P747, DOI 10.1016/j.cgh.2014.09.047
Sharaiha RZ, 2016, CLIN GASTROENTEROL H, V14, P1797, DOI 10.1016/j.cgh.2016.05.011
van Santvoort HC, 2010, NEW ENGL J MED, V362, P1491, DOI 10.1056/NEJMoa0908821
Varadarajulu S, 2008, GASTROINTEST ENDOSC, V68, P649, DOI 10.1016/j.gie.2008.02.057
Xie LT, 2019, MED SCI MONITOR, V25, P5785, DOI 10.12659/MSM.915193
NR 24
TC 2
Z9 2
U1 0
U2 3
PU SPRINGER
PI NEW YORK
PA ONE NEW YORK PLAZA, SUITE 4600, NEW YORK, NY, UNITED STATES
SN 0930-2794
EI 1432-2218
J9 SURG ENDOSC
JI Surg. Endosc.
PD APR
PY 2023
VL 37
IS 4

BP 2626
EP 2632
DI 10.1007/s00464-022-09741-6
EA NOV 2022
PG 7
WC Surgery
WE Science Citation Index Expanded (SCI-EXPANDED)
SC Surgery
GA D2UK8
UT WOS:000881878900001
PM 36369409
DA 2026-05-09
ER

PT J
AU Doi, S
Yasuda, I
Kawakami, H
Hayashi, T
Hisai, H
Irisawa, A
Mukai, T
Katanuma, A
Kubota, K
Ohnishi, T
Ryozawa, S
Hara, K
Itoi, T
Hanada, K
Yamao, K
AF Doi, S.
Yasuda, I.
Kawakami, H.
Hayashi, T.
Hisai, H.
Irisawa, A.
Mukai, T.
Katanuma, A.
Kubota, K.
Ohnishi, T.
Ryozawa, S.
Hara, K.

Itoi, T.
Hanada, K.
Yamao, K.
TI Endoscopic ultrasound-guided celiac ganglia neurolysis vs. celiac plexus
neurolysis: a randomized multicenter trial
SO ENDOSCOPY
LA English
DT Article
ID PAIN RELIEF; CANCER PAIN; BLOCK; VISUALIZATION
AB Background and study aims: No prospective comparison of endoscopic ultrasonography-guided direct celiac ganglia neurolysis (EUS-CGN) vs. EUS-guided celiac plexus neurolysis (EUS-CPN) has been reported. The aim of the current study was to compare the effectiveness of EUS-CGN and EUS-CPN in providing pain relief from upper abdominal cancer pain in a multicenter randomized controlled trial.

Patients and methods: Patients with upper abdominal cancer pain were randomly assigned to treatment using either EUS-CGN or EUS-CPN. Evaluation was performed at Day 7 postoperatively using a pain scale of 0 to 10. Patients for whom pain decreased to <= 3 were considered to have a positive response, and those experiencing a decrease in pain to <= 1 were considered to be completely responsive. Comparison between the two groups was performed using intention-to-treat analysis. The primary endpoint was the difference in treatment response rates between EUS-CGN and EUS-CPN at postoperative Day 7. Secondary endpoints included differences in complete response rates, pain scores, duration of pain relief, and incidence of adverse effects.

Results: A total of 34 patients were assigned to each group. Visualization of ganglia was possible in 30 cases (88%) in the EUS-CGN group. The positive response rate was significantly higher in the EUS-CGN group (73.5%) than in the EUS-CPN group (45.5%; P=0.026). The complete response rate was also significantly higher in the EUS-CGN group (50.0%) than in the EUS-CPN group (18.2%; P=0.010). There was no difference in adverse events or duration of pain relief between the two groups.

Conclusions: EUS-CGN is significantly superior to conventional EUS-CPN in cancer pain relief. Clinical trial registration: http://www.umin.ac.jp/ctr/index.htm (ID: UMIN-000002536)
C1 [Doi, S.; Yasuda, I.] Gifu Univ Hosp, Dept Internal Med 1, Gifu 5011194, Japan.
[Kawakami, H.] Hokkaido Univ, Dept Gastroenterol & Hepatol, Grad Sch Med, Sapporo, Hokkaido, Japan.
[Hayashi, T.] Sapporo Med Univ, Sch Med, Dept Internal Med 4, Sapporo, Hokkaido, Japan.
[Hisai, H.] Japan Red Cross Date Gen Hosp, Dept Gastroenterol, Date, Japan.
[Irisawa, A.] Fukushima Med Univ Hosp, Dept Gastroenterol, Fukushima, Japan.
[Mukai, T.] Gifu Municipal Hosp, Dept Gastroenterol, Gifu, Japan.
[Katanuma, A.] Teine Keijinkai Hosp, Ctr Gastroenterol, Sapporo, Hokkaido, Japan.
[Kubota, K.] Yokohama City Univ, Div Gastroenterol, Grad Sch Med, Yokohama, Kanagawa 232, Japan.
[Ohnishi, T.] Gifu Prefectural Gen Med Ctr, Dept Gastroenterol, Gifu, Japan.
[Ryozawa, S.] Showa Univ, Northern Yokohama Hosp, Ctr Digest Dis, Yokohama, Kanagawa, Japan.
[Hara, K.; Yamao, K.] Aichi Canc Ctr Hosp, Dept Gastroenterol, Nagoya, Aichi 464, Japan.
[Itoi, T.] Tokyo Med Univ, Dept Gastroenterol & Hepatol, Tokyo 1608402, Japan.
[Hanada, K.] Onomichi Gen Hosp, Ctr Gastroendoscopy, Onomichi, Japan.
C3 Gifu University; Hokkaido University; Sapporo Medical University;
Fukushima Medical University; Gifu Municipal Hospital; Teine Keijinkai

Hospital; Yokohama City University; Showa Medical University; Aichi
Cancer Center; Tokyo Medical University
RP Yasuda, I (corresponding author), Gifu Univ Hosp, Dept Internal Med 1, 1-1 Yanagido, Gifu 5011194, Japan.
EM YASUDAIC@aol.com
RI Kawakami, Hiroshi/F-7297-2012
FU Japanese Foundation for Research and Promotion of Endoscopy (JFE)
FX This work was supported by a grant from The Japanese Foundation for
Research and Promotion of Endoscopy (JFE).
CR Ascunce G, 2011, GASTROINTEST ENDOSC, V73, P267, DOI 10.1016/j.gie.2010.10.029
De Cicco M, 2001, ANESTHESIOLOGY, V94, P561, DOI 10.1097/00000542-200104000-00006
EISENBERG E, 1995, ANESTH ANALG, V80, P290, DOI 10.1097/00000539-199502000-00015
Faigel DO, 1996, AM J GASTROENTEROL, V91, P1675
Gerke H, 2006, GASTROINTEST ENDOSC, V64, P35, DOI 10.1016/j.gie.2006.01.071
Gleeson FC, 2007, ENDOSCOPY, V39, P620, DOI 10.1055/s-2007-966337
Gunaratnam NT, 2001, GASTROINTEST ENDOSC, V54, P316, DOI 10.1067/mge.2001.117515
HAAGA JR, 1984, AM J ROENTGENOL, V142, P1201
Harada N, 1997, Gastrointest Endosc Clin N Am, V7, P237
HARADA S, 1980, AM J HUM GENET, V32, P8
Iwata K, 2011, DIGEST ENDOSC, V23, P140, DOI 10.1111/j.1443-1661.2010.01046.x
Kappis M, 1914, Verh. Dtsch. Ges. Chir, V43, P87
Levy M, 2006, AM J GASTROENTEROL, V101, P1787, DOI 10.1111/j.1572-0241.2006.00685.x
Levy MJ, 2008, AM J GASTROENTEROL, V103, P98, DOI 10.1111/j.1572-0241.2007.01607.x
OKEN MM, 1982, AM J CLIN ONCOL-CANC, V5, P649, DOI 10.1097/00000421-198212000-00014
Puli SR, 2009, DIGEST DIS SCI, V54, P2330, DOI 10.1007/s10620-008-0651-x
Sahai AV, 2009, AM J GASTROENTEROL, V104, P326, DOI 10.1038/ajg.2008.64
Sakamoto H, 2010, AM J GASTROENTEROL, V105, P2599, DOI 10.1038/ajg.2010.339
Tran QN, 2006, J SUPPORT ONCOL, V4, P463
Tran Quy N H, 2006, J Support Oncol, V4, P460
VENTAFRIDDA V, 1987, CANCER-AM CANCER SOC, V59, P850, DOI 10.1002/1097-
0142(19870215)59:4<850::AID-CNCR2820590432>3.0.CO;2-1
Wiersema MJ, 1996, GASTROINTEST ENDOSC, V44, P656, DOI 10.1016/S0016-5107(96)70047-0
NR 22
TC 89
Z9 115
U1 0
U2 17
PU GEORG THIEME VERLAG KG
PI STUTTGART
PA RUDIGERSTR 14, D-70469 STUTTGART, GERMANY
SN 0013-726X
EI 1438-8812
J9 ENDOSCOPY

JI Endoscopy
PD MAY
PY 2013
VL 45
IS 5
BP 362
EP 369
DI 10.1055/s-0032-1326225
PG 8
WC Gastroenterology & Hepatology; Surgery
WE Science Citation Index Expanded (SCI-EXPANDED)
SC Gastroenterology & Hepatology; Surgery
GA 131VY
UT WOS:000318026600006
PM 23616126
DA 2026-05-09
ER

PT J
AU Dietrich, CF
Braden, B
Jenssen, C
AF Dietrich, Christoph F.
Braden, Barbara
Jenssen, Christian
TI Interventional endoscopic ultrasound
SO CURRENT OPINION IN GASTROENTEROLOGY
LA English
DT Review
DE biliary drainage; cancer; endoscopy; guideline; ultrasound
ID GUIDED BILIARY DRAINAGE; APPOSING METAL STENTS; LONG-TERM OUTCOMES;
EFSUMB GUIDELINES; GALLBLADDER DRAINAGE; GASTRIC VARICES; PLASTIC
STENTS; RETROGRADE CHOLANGIOPANCREATOGRAPHY; MALIGNANT OBSTRUCTION;
ACUTE CHOLECYSTITIS
AB Purpose of review
To update on recent advances in interventional endoscopic ultrasound (INVEUS) techniques.
Recent findings
The introduction of linear echoendoscopes with larger instrument channels and the combined development of new tools and devices have enabled various new applications of minimally invasive endoscopic ultrasound (EUS)-guided transluminal interventions of the pancreas, biliary system and peri-gastrointestinal structures. In this review, EUS-guided interventions are discussed and evaluated: drainage of peripancreatic fluid collections, access and drainage of bile ducts, gallbladder and pancreatic duct, treatment of gastrointestinal haemorrhage, coeliac plexus block and coeliac plexus neurolysis, fiducial

placement, solid and cystic tumour ablation, drug delivery and brachytherapy, gastroenterostomy, angiotherapy and other EUS-guided applications. The EUS-guided interventions are classified based on the available evidence as established or experimental, standardized or nonstandardized procedures in common or rare diseases with well or little known complications and their established or nonestablished treatment.

Summary

Some EUS-guided interventions have sparse published evidence with only single-centre studies, case series or individual case reports, others like drainage of peripancreatic fluid collections have become widely accepted practice. INVEUS has been accepted as an alternative to several surgical approaches, EUSguided techniques result in faster recovery times, shorter hospital stay and lower costs.

C1 [Dietrich, Christoph F.] Kliniken Hirslanden Beau Site Salem & Permanence, Dept Allgemeine Innere Med DAIM, CH-3036 Bern, Switzerland.

[Braden, Barbara] Oxford Univ Hosp NHS Fdn Trust, Translat Gastroenterol Unit, John Radcliffe Hosp, Oxford, England.

[Jenssen, Christian] Krankenhaus Maerkisch Oderland, Dept Internal Med, D-15344 Strausberg, Germany.

[Jenssen, Christian] Med Univ Brandenburg, Brandenburg Inst Clin Ultrasound, Neuruppin, Germany.

C3 Oxford University Hospitals NHS Foundation Trust; University of Oxford

RP Dietrich, CF (corresponding author), Kliniken Hirslanden Beau Site Salem & Permanence, Dept Allgemeine Innere Med DAIM, CH-3036 Bern, Switzerland.

EM c.f.dietrich@googlemail.com

RI Dietrich, Christoph Frank/AFQ-4722-2022; Braden, Barbara/AAH-8693-2021

OI Dietrich, Christoph Frank/0000-0001-6015-6347;

CR Adoor Dayyan, 2020, VideoGIE, V5, P542, DOI [10.1016/j.vgie.2020.05.032, 10.1016/j.vgie.2020.05.032]

Ahmed A, 2020, INDIAN J PALLIAT CAR, V26, P198, DOI 10.4103/0973-1075.285695

Ahmed O, 2018, SURG ENDOSC, V32, P1627, DOI 10.1007/s00464-018-6041-3

Alvarez-Sanchez MV, 2014, SURG ENDOSC, V28, P712, DOI 10.1007/s00464-013-3260-5

Amato A, 2021, ENDOSCOPY, V53, P1037, DOI 10.1055/a-1324-7919

Attila T, 2019, EUR J GASTROEN HEPAT, V31, P1, DOI 10.1097/MEG.0000000000001297

Bang JY, 2019, GASTROINTEST ENDOSC, V89, P58, DOI 10.1016/j.gie.2018.08.005

Bang JY, 2019, GUT, V68, P1200, DOI 10.1136/gutjnl-2017-315335

Bang JY, 2018, GASTROINTEST ENDOSC, V88, P9, DOI 10.1016/j.gie.2018.03.012

Baniya R, 2017, CLIN EXP GASTROENTER, V10, P67, DOI 10.2147/CEG.S132004

Banks PA, 2013, GUT, V62, P102, DOI 10.1136/gutjnl-2012-302779

Baron TH, 2020, GASTROENTEROLOGY, V158, P67, DOI 10.1053/j.gastro.2019.07.064

Bazarbashi AN, 2020, ENDOSC INT OPEN, V8, pE221, DOI 10.1055/a-1027-6708

Bazerbachi F, 2018, GASTROINTEST ENDOSC, V87, P30, DOI 10.1016/j.gie.2017.08.025

Braden B, 2019, ENDOSC ULTRASOUND, V8, P370, DOI 10.4103/eus.eus_39_19

Bukhari M, 2018, GASTROINTEST ENDOSC, V88, P486, DOI 10.1016/j.gie.2018.04.2356

Canakis A, 2020, BMJ OPEN GASTROENTER, V7, DOI 10.1136/bmjgast-2020-000428

Chandan S, 2020, ENDOSC INT OPEN, V08, pE1664, DOI 10.1055/a-1236-3350

Chandnani M, 2020, ENDOSC INT OPEN, V8, pE179, DOI 10.1055/a-1068-9128

Chandrasekhara V, 2020, ENDOSC INT OPEN, V08, pE1639, DOI 10.1055/a-1243-0092

Chang JI, 2019, ENDOSC INT OPEN, V7, pE655, DOI 10.1055/a-0826-4309

Chen SA, 2019, HEPATOB PANCREAT DIS, V18, P296, DOI 10.1016/j.hbpd.2018.11.004
Chen YI, 2017, GASTROINTEST ENDOSC, V85, P170, DOI 10.1016/j.gie.2016.07.031
Chin JYL, 2020, ENDOSC INT OPEN, V08, pE1633, DOI 10.1055/a-1236-3217
Coronel E, 2019, GASTROINTEST ENDOSC, V89, P659, DOI 10.1016/j.gie.2018.10.047
Dalal A, 2020, DIGEST ENDOSC, V32, P409, DOI 10.1111/den.13504
De Angelis CG, 2020, J CLIN MED, V9, DOI 10.3390/jcm9030866
Dhaliwal A, 2020, ENDOSC INT OPEN, V08, pE1243, DOI 10.1055/a-1221-5012
Dietrich CF, 2016, ULTRASCHALL MED, V37, P27, DOI 10.1055/s-0035-1553965
Dietrich CF, 2016, ULTRASCHALL MED, V37, pE1, DOI 10.1055/s-0035-1553917
Dietrich CF, 2015, ULTRASCHALL MED, V36, P460, DOI 10.1055/s-0035-1553462
Dietrich CF., 2020, Gastrointestinal and pancreatico-biliary diseases: advanced diagnostic and therapeutic endoscopy, DOI [10.1007/978-3-030-29964-4_95-1, DOI 10.1007/978-3-030-29964-4_95-1]
Facciorusso A, 2017, J GASTROEN HEPATOL, V32, P439, DOI 10.1111/jgh.13478
Fan WT, 2022, MINIM INVASIV THER, V31, P159, DOI 10.1080/13645706.2020.1792500
Fueldner F, 2020, Z GASTROENTEROL, V58, P1081, DOI 10.1055/a-1250-8834
Fujii-Lau LL, 2016, SURG ENDOSC, V30, P1396, DOI 10.1007/s00464-015-4342-3
Fusaroli P, 2016, ULTRASCHALL MED, V37, P408, DOI 10.1055/s-0035-1553742
Fusaroli P, 2016, ULTRASCHALL MED, V37, pE77, DOI 10.1055/s-0035-1553738
de Paredes AGG, 2020, ENDOSC INT OPEN, V8, pE6, DOI 10.1055/a-1031-9295
Ge N, 2020, ENDOSC ULTRASOUND, V9, P162, DOI 10.4103/eus.eus_36_20
Godat S, 2019, SWISS MED WKLY, p11S
Guo JT, 2017, ENDOSC ULTRASOUND, V6, P285, DOI 10.4103/eus.eus_85_17
Gutierrez OIB, 2018, ENDOSCOPY, V50, P891, DOI 10.1055/s-0044-102254
Hammad T, 2018, DIGEST DIS SCI, V63, P289, DOI 10.1007/s10620-017-4851-0
Han SY, 2019, SCI REP-UK, V9, DOI 10.1038/s41598-019-52993-x
Hathorn KE, 2019, ENDOSC INT OPEN, V7, pE1432, DOI 10.1055/a-0990-9488
Imai H, 2016, GASTROINTEST ENDOSC, V84, P147, DOI 10.1016/j.gie.2015.12.024
Imoto A, 2020, CLIN ENDOSC, V53, P525, DOI 10.5946/ce.2020.173
Imperatore N, 2020, ENDOSC INT OPEN, V08, pE1759, DOI 10.1055/a-1261-9605
Iqbal U, 2020, ENDOSC ULTRASOUND, V9, P16, DOI 10.4103/eus.eus_70_19
Issa D, 2021, ENDOSCOPY, V53, P827, DOI 10.1055/a-1259-0349
Jain D, 2018, CLIN ENDOSC, V51, P450, DOI 10.5946/ce.2018.024
James Theodore W, 2020, VideoGIE, V5, P428, DOI 10.1016/j.vgie.2020.05.004
Jenssen C, 2016, ULTRASCHALL MED, V37, P473, DOI 10.1055/s-0035-1553450
Jenssen C, 2016, ULTRASCHALL MED, V37, P157, DOI 10.1055/s-0035-1553788
Jenssen C, 2016, ULTRASCHALL MED, V37, pE33, DOI 10.1055/s-0035-1553785
Jin Z, 2020, DIGEST ENDOSC, V32, P16, DOI 10.1111/den.13456
Kakked G, 2020, ENDOSC ULTRASOUND, V9, P298, DOI 10.4103/eus.eus_10_20
Kalva NR, 2018, CAN J GASTROENTEROL, V2018, DOI 10.1155/2018/7070961
Kastelijn JB, 2020, ENDOSC INT OPEN, V08, pE1194, DOI 10.1055/a-1214-5659
Kedia P, 2019, J CLIN GASTROENTEROL, V53, P304, DOI 10.1097/MCG.0000000000001037
Kerdsirichairat T, 2020, CURR OPIN GASTROEN, V36, P402, DOI 10.1097/MOG.0000000000000662

Khashab MA, 2015, GASTROINTEST ENDOSC, V81, P81, DOI 10.1016/j.gie.2014.08.008
Khoury T, 2019, HEPATOL COMMUN, V3, P207, DOI 10.1002/hep4.1289
Ki ELL, 2019, ENDOSC ULTRASOUND, V8, pS17, DOI 10.4103/eus.eus_50_19
Kochhar Gursimran S, 2020, Endosc Int Open, V8, pE877, DOI [10.1055/a-1164-6282, 10.1055/a-1164-6282]
Krafft MR, 2021, DIGEST DIS SCI, V66, P2776, DOI 10.1007/s10620-020-06551-5
Krafft MR, 2019, DIGEST DIS SCI, V64, P1770, DOI 10.1007/s10620-019-05495-9
Krishnamoorthi R, 2020, SURG ENDOSC, V34, P1904, DOI 10.1007/s00464-020-07409-7
Levy MJ, 2019, CLIN GASTROENTEROL H, V17, P728, DOI 10.1016/j.cgh.2018.08.040
Levy MJ, 2013, GASTROINTEST ENDOSC, V78, P722, DOI 10.1016/j.gie.2013.07.004
Li DF, 2019, REV ESP ENFERM DIG, V111, P953, DOI 10.17235/reed.2019.6125/2018
Logiudice FP, 2019, WORLD J GASTRO ENDOS, V11, P281, DOI 10.4253/wjge.v11.i4.281
Lu F, 2018, SUPPORT CARE CANCER, V26, P353, DOI 10.1007/s00520-017-3888-0
Luk SWY, 2019, ENDOSCOPY, V51, P722, DOI 10.1055/a-0929-6603
Machicado JD, 2019, CLIN GASTROENTEROL H, V17, P2749, DOI 10.1016/j.cgh.2019.04.046
Mai HD, 2018, CLIN J GASTROENTEROL, V11, P282, DOI 10.1007/s12328-018-0837-5
Manta R, 2018, EUR J GASTROEN HEPAT, V30, P695, DOI 10.1097/MEG.0000000000001112
Marrache MK, 2021, GASTROINTEST ENDOSC, V93, P34, DOI 10.1016/j.gie.2020.06.057
McCarty TR, 2020, ENDOSC ULTRASOUND, V9, P6, DOI 10.4103/eus.eus_37_19
Miller CS, 2019, ENDOSC INT OPEN, V7, pE1563, DOI 10.1055/a-0998-8129
Mohan BP, 2020, ENDOSCOPY, V52, P259, DOI 10.1055/a-1098-1817
Mohan BP, 2020, ENDOSCOPY, V52, P96, DOI 10.1055/a-1020-3932
Mohan BP, 2019, ENDOSC ULTRASOUND, V8, P82, DOI 10.4103/eus.eus_7_19
Moole H, 2017, MEDICINE, V96, DOI 10.1097/MD.0000000000005154
Mori Y, 2018, J HEPATO-BIL-PAN SCI, V25, P87, DOI 10.1002/jhbp.504
Moyer MT, 2017, GASTROENTEROLOGY, V153, P1295, DOI 10.1053/j.gastro.2017.08.009
Muthusamy VR, 2016, GASTROINTEST ENDOSC, V83, P481, DOI 10.1016/j.gie.2015.11.027
Ogura T, 2019, ENDOSC ULTRASOUND, V8, P398, DOI 10.4103/eus.eus_45_19
Olsen GA, 2020, DAN MED J, V67
Paik WH, 2018, AM J GASTROENTEROL, V113, P987, DOI 10.1038/s41395-018-0122-8
Park JK, 2018, GASTROINTEST ENDOSC, V88, P277, DOI 10.1016/j.gie.2018.03.015
Patel JB, 2020, WORLD J GASTRO ENDOS, V12, DOI 10.4253/wjge.v12.i8.231
Podboy A, 2021, GASTROINTEST ENDOSC, V93, P797, DOI 10.1016/j.gie.2020.09.040
Poincloux L, 2017, ENDOSCOPY, V49, P484, DOI 10.1055/s-0042-122011
Rai P, 2018, ENDOSC INT OPEN, V6, pE821, DOI 10.1055/a-0608-4402
Rai P, 2017, ENDOSCOPY, V49, pE179, DOI 10.1055/s-0043-109790
Rigter LS, 2019, ENDOSC INT OPEN, V7, pE1357, DOI 10.1055/a-0958-2148
Rimbas M, 2020, ENDOSC ULTRASOUND, V9, P291, DOI 10.4103/eus.eus_44_20
Robles-Medranda C, 2020, ENDOSCOPY, V52, P268, DOI 10.1055/a-1123-9054
Rosa F, 2021, SURG ENDOSC, V35, P486, DOI 10.1007/s00464-020-07996-5
Sagami R, 2020, ANN GASTROENTEROL, V33, P391, DOI 10.20524/aog.2020.0496
Sagami R, 2020, CLIN ENDOSC, V53, P176, DOI 10.5946/ce.2019.177
Saumoy M, 2018, ENDOSC ULTRASOUND, V7, P97, DOI 10.4103/eus.eus_9_18

Saunders R, 2019, SURG ENDOSC, V33, P1412, DOI 10.1007/s00464-018-6416-5
Schreiber-Dietrich D, 2019, APPL SCI-BASEL, V9, DOI 10.3390/app9235036
Schulman AR, 2017, GASTROINTEST ENDOSC, V85, P243, DOI 10.1016/j.gie.2016.07.041
Sharaiha RZ, 2017, GASTROINTEST ENDOSC, V85, P904, DOI 10.1016/j.gie.2016.12.023
Shen YH, 2020, MEDICINE, V99, DOI 10.1097/MD.0000000000022903
Tan SL, 2020, GASTROENT RES PRACT, V2020, DOI 10.1155/2020/4952721
Teoh AY, 2019, ENDOSC INT OPEN, V7, pE964, DOI 10.1055/a-0915-2098
Teoh AYB, 2020, GUT, V69, P1085, DOI 10.1136/gutjnl-2019-319996
Teoh AYB, 2018, GUT, V67, P1209, DOI 10.1136/gutjnl-2017-314341
Teoh AYB, 2021, DIGEST ENDOSC, V33, P1139, DOI 10.1111/den.13911
Testoni SGG, 2020, ENDOSC ULTRASOUND, V9, P83, DOI 10.4103/eus.eus_74_19
Tsuchiya T, 2018, GASTROINTEST ENDOSC, V87, P1138, DOI 10.1016/j.gie.2017.08.017
Tyberg A, 2017, GASTROINTEST ENDOSC, V85, P164, DOI 10.1016/j.gie.2016.07.030
Wang TJ, 2021, SURG ENDOSC, V35, P4469, DOI 10.1007/s00464-020-07952-3
Will U, 2015, WORLD J GASTROENTERO, V21, P13140, DOI 10.3748/wjg.v21.i46.13140
Wyse Jonathan M, 2018, Curr Treat Options Gastroenterol, V16, P417, DOI [10.1007/s11938-018-0193-z, 10.1007/s11938-018-0193-z]
Wyse JM, 2017, ENDOSC ULTRASOUND, V6, P369, DOI 10.4103/eus.eus_97_17
Xu GF, 2020, ENDOSC INT OPEN, V08, pE1690, DOI 10.1055/a-1221-9656
Yan L, 2019, ENDOSC ULTRASOUND, V8, P172, DOI 10.4103/eus.eus_108_17
Yoon SB, 2018, UNITED EUR GASTROENT, V6, P729, DOI 10.1177/2050640618761702
Yoon WJ, 2020, J CLIN MED, V9, DOI 10.3390/jcm9061666
Zhang L, 2020, SCAND J GASTROENTERO, V55, P1121, DOI 10.1080/00365521.2020.1797870
NR 126
TC 9
Z9 9
U1 0
U2 0
PU LIPPINCOTT WILLIAMS & WILKINS
PI PHILADELPHIA
PA TWO COMMERCE SQ, 2001 MARKET ST, PHILADELPHIA, PA 19103 USA
SN 0267-1379
EI 1531-7056
J9 CURR OPIN GASTROEN
JI Curr. Opin. Gastroenterol.
PD SEP
PY 2021
VL 37
IS 5
BP 449
EP 461
DI 10.1097/MOG.0000000000000760

PG 13
WC Gastroenterology & Hepatology
WE Science Citation Index Expanded (SCI-EXPANDED)
SC Gastroenterology & Hepatology
GA TQ9AA
UT WOS:000678567500008
PM 34091534
DA 2026-05-09
ER

PT J
AU Puga, M
Consiglieri, CF
Busquets, J
Pallarès, N
Secanella, L
Peláez, N
Fabregat, J
Castellote, J
Gornals, JB
AF Puga, Manuel
Consiglieri, Claudia F.
Busquets, Juli
Pallares, Natalia
Secanella, Lluis
Pelaez, Nuria
Fabregat, Joan
Castellote, Jose
Gornals, Joan B.
TI Safety of lumen-apposing stent with or without coaxial plastic stent for endoscopic ultrasound-guided drainage of pancreatic fluid collections: a retrospective study
SO ENDOSCOPY
LA English
DT Article
ID METAL STENT; NECROSIS
AB Background The aim of this study was to evaluate whether the placement of a coaxial double-pigtail plastic stent (DPS) within a lumen-apposing metal stent (LAMS) may improve the safety of endoscopic ultrasound (EUS)-guided drainage of pancreatic fluid collections (PFCs).

Methods This was a retrospective cohort study including patients with PFCs and an indication for transmural drainage. Two strategies (LAMS alone or LAMS plus DPS) were used at the endoscopist's discretion.

Results A total of 41 patients were treated (21 LAMS alone; 20 LAMS plus DPS). The characteristics of the PFCs, and

the technical and clinical success rates did not differ between groups. The LAMS alone group had a significantly higher rate of adverse events than the LAMS plus DPS group (42.9% vs. 10.0%; P =0.04). Bleeding was the most frequent adverse event observed.

Conclusions The addition of a coaxial DPS to LAMS was associated with a lower rate of adverse events in EUS-guided drainage of PFCs.
C1 [Puga, Manuel] Complexo Hosp Univ Ourense, Dept Digest Dis, Orense, Spain.

[Puga, Manuel; Consiglieri, Claudia F.; Pelaez, Nuria; Gornals, Joan B.] Univ Barcelona, Endoscopy Unit, Dept Digest Dis, Hosp Univ Bellvitge,Bellvitge Biomed Res Inst IDI, Barcelona, Spain.

[Busquets, Juli; Secanella, Lluis; Pelaez, Nuria; Fabregat, Joan] Univ Barcelona, Dept Surg, Hosp Univ Bellvitge, Bellvitge Biomed Res Inst IDIBELL, Barcelona, Spain.

[Pallares, Natalia] Bellvitge Biomed Res Inst IDIBELL, Stat Advisory Serv, Barcelona, Spain.

[Castellote, Jose] Univ Barcelona, Hepatol Unit, Dept Digest Dis, Hosp Univ Bellvitge,Bellvitge Biomed Res Inst IDI, Barcelona, Spain.

C3 Complexo Hospitalario Universitario de Ourense, Verin e O Barco de
Valdeorras; Institut d'Investigacio Biomedica de Bellvitge (IDIBELL);
Bellvitge University Hospital; University of Barcelona; University of
Barcelona; Institut d'Investigacio Biomedica de Bellvitge (IDIBELL);
Bellvitge University Hospital; Institut d'Investigacio Biomedica de
Bellvitge (IDIBELL); University of Barcelona; Institut d'Investigacio
Biomedica de Bellvitge (IDIBELL); Bellvitge University Hospital

RP Gornals, JB (corresponding author), Hosp Univ Bellvitge, Endoscopy Unit, Dept Digest Dis, IDIBELL Bellvitge Biomed Res Inst, Feixa Llarga S-N, Barcelona 08907, Spain.

EM jgornals@bellvitgehospital.cat

RI ; Pallares, Natalia/AFQ-8910-2022; Busquets, Juli/AAD-3774-2019

OI CASTELLOTE, JOSE/0000-0002-8528-3112; gornals, joan
b/0000-0001-8857-3556; Busquets, Juli/0000-0001-6685-3135
CR Bang JY, 2017, GUT, V66, P2054, DOI 10.1136/gutjnl-2016-312812
Bang JY, 2015, DIGEST ENDOSC, V27, P486, DOI 10.1111/den.12418
Banks PA, 2013, GUT, V62, P102, DOI 10.1136/gutjnl-2012-302779
Besselink M, 2013, PANCREATOLOGY, V13, pE1, DOI 10.1016/j.pan.2013.07.063
Cotton PB, 2010, GASTROINTEST ENDOSC, V71, P446, DOI 10.1016/j.gie.2009.10.027
Fabbri C, 2015, GASTROINTEST ENDOSC, V82, P585, DOI 10.1016/j.gie.2015.04.035
Gornals JB, 2016, SURG ENDOSC, V30, P2592, DOI 10.1007/s00464-015-4505-2
Gornals JB, 2013, SURG ENDOSC, V27, P1428, DOI 10.1007/s00464-012-2591-y
Itoi T, 2012, GASTROINTEST ENDOSC, V75, P870, DOI 10.1016/j.gie.2011.10.020
Perez-Miranda M, 2015, THESIS
Siddiqui AA, 2017, GASTROINTEST ENDOSC, V85, P758, DOI 10.1016/j.gie.2016.08.014
Siddiqui AA, 2016, GASTROINTEST ENDOSC, V83, P699, DOI 10.1016/j.gie.2015.10.020
Stecher SS, 2017, GUT, V66, P1871, DOI 10.1136/gutjnl-2016-313562
Vazquez-Sequeiros E, 2016, GASTROINTEST ENDOSC, V84, P450, DOI 10.1016/j.gie.2016.02.044
Walter D, 2015, ENDOSCOPY, V47, P63, DOI 10.1055/s-0034-1378113
NR 15

TC 70
Z9 86
U1 1
U2 2
PU GEORG THIEME VERLAG KG
PI STUTTGART
PA RUDIGERSTR 14, D-70469 STUTTGART, GERMANY
SN 0013-726X
EI 1438-8812
J9 ENDOSCOPY
JI Endoscopy
PD OCT
PY 2018
VL 50
IS 10
BP 1022
EP 1026
DI 10.1055/a-0582-9127
PG 5
WC Gastroenterology & Hepatology; Surgery
WE Science Citation Index Expanded (SCI-EXPANDED)
SC Gastroenterology & Hepatology; Surgery
GA GV1BO
UT WOS:000445802100027
PM 29590668
DA 2026-05-09
ER

PT J
AU Meng, FS
Zhang, ZH
Ji, F
AF Meng, Fan-Sheng
Zhang, Zhao-Hong
Ji, Feng
TI Therapeutic role of endoscopic ultrasound in pancreaticobiliary disease: A comprehensive review
SO WORLD JOURNAL OF GASTROENTEROLOGY
LA English
DT Review
DE Endoscopic ultrasound; Therapeutic; Biliary drainage; Peripancreatic fluid collection; Celiac plexus neurolysis

ID CELIAC PLEXUS NEUROLYSIS; GUIDED BILIARY DRAINAGE; PERIPANCREATIC FLUID COLLECTIONS; SPINAL-CORD INFARCTION; TRIAL COMPARING EUS; TRANSMURAL DRAINAGE; STEREOTACTIC RADIOSURGERY; FIDUCIAL PLACEMENT; RADIATION-THERAPY; PAIN RELIEF

AB With the development of technology and accessories, the role of endoscopic ultrasound (EUS) has evolved from diagnostics to therapeutics. In order to characterise the therapeutic role of EUS, we searched Web of Knowledge database and reviewed articles associated with therapeutic EUS. There are two modalities for the therapeutic purpose: drainage and fine-needle injection. EUS-guided drainage is a promising procedure for the treatment of peripancreatic fluid collection and biliary obstruction; EUS-guided fine-needle injections such as celiac plexus neurolysis, for the purpose of pain relief for pancreatic cancer and chronic pancreatitis, has emerged as a promising procedure. The aim of the study was to perform a comprehensive and conscientious review on the techniques, complications and clinical outcomes of those EUS-based procedures.

C1 [Meng, Fan-Sheng; Ji, Feng] Zhejiang Univ, Affiliated Hosp 1, Sch Med, Dept Gastroenterol, Hangzhou 310000, Zhejiang, Peoples R China.

[Zhang, Zhao-Hong] Linyi Peoples Hosp, Dept Hematol, Linyi 276300, Shandong, Peoples R China.

C3 Zhejiang University

RP Ji, F (corresponding author), Zhejiang Univ, Affiliated Hosp 1, Sch Med, Dept Gastroenterol, Qingchun Rd 79, Hangzhou 310000, Zhejiang, Peoples R China.

EM jifeng1126@sina.com

CR Ahn JY, 2010, GUT LIVER, V4, P524, DOI 10.5009/gnl.2010.4.4.524

Altonbary AY, 2014, ENDOSC ULTRASOUND, V3, P137, DOI 10.4103/2303-9027.131043

Ammar T, 2010, GASTROINTEST ENDOSC, V71, P630, DOI 10.1016/j.gie.2009.11.023

Andren-Sandberg Ake, 2004, JOP, V5, P64

Ang TL, 2010, SINGAP MED J, V51, P762

[Anonymous], 2013, J Interv Gastroenterol, DOI [DOI 10.7178/JIG.102, 10.7178/jig.102]

Artifon ELA, 2012, J CLIN GASTROENTEROL, V46, P768, DOI 10.1097/MCG.0b013e31825f264c

Baron Todd H, 2003, Gastrointest Endosc Clin N Am, V13, P743, DOI 10.1016/S1052-5157(03)00100-4

Bhasin DK, 2009, J GASTROEN HEPATOL, V24, P720, DOI 10.1111/j.1440-1746.2009.05809.x

Bollen FL, 2008, BRIT J SURG, V95, P6, DOI 10.1002/bjs.6010

Carrara S, 2013, WORLD J GASTRO ENDOS, V5, P141, DOI 10.4253/wjge.v5.i4.141

Chavalitdhamrong D, 2012, WORLD J GASTROENTERO, V18, P491, DOI 10.3748/wjg.v18.i6.491

DeWitt J, 2009, GASTROINTEST ENDOSC, V70, P710, DOI 10.1016/j.gie.2009.03.1173

Dhir V, 2012, GASTROINTEST ENDOSC, V75, P354, DOI 10.1016/j.gie.2011.07.075

Didolkar MS, 2010, J GASTROINTEST SURG, V14, P1547, DOI 10.1007/s11605-010-1323-7

DiMaio CJ, 2010, GASTROINTEST ENDOSC, V71, P1204, DOI 10.1016/j.gie.2010.01.003

Doi S, 2013, ENDOSCOPY, V45, P362, DOI 10.1055/s-0032-1326225

Fujii L, 2012, ENDOSCOPY, V44, pE265, DOI 10.1055/s-0032-1309708

Gimeno-García AZ, 2012, ENDOSCOPY, V44, pE267, DOI 10.1055/s-0032-1309709

Gunaratnam NT, 2001, GASTROINTEST ENDOSC, V54, P316, DOI 10.1067/mge.2001.117515

Guo XZ, 2013, ENDOSC ULTRASOUND, V2, P181, DOI 10.4103/2303-9027.121238

Gupta K, 2014, J CLIN GASTROENTEROL, V48, P80, DOI 10.1097/MCG.0b013e31828c6822

Hadithi M, 2014, WORLD J GASTRO ENDOS, V6, P373, DOI 10.4253/wjge.v6.i8.373

Hara K, 2013, ENDOSCOPY, V45, P392, DOI 10.1055/s-0032-1326076
Hookey LC, 2006, GASTROINTEST ENDOSC, V63, P635, DOI 10.1016/j.gie.2005.06.028
Iwashita T, 2012, ENDOSCOPY, V44, P60, DOI 10.1055/s-0030-1256871
Iwata K, 2011, DIGEST ENDOSC, V23, P140, DOI 10.1111/j.1443-1661.2010.01046.x
Jang HY, 2013, CLIN ENDOSC, V46, P306, DOI 10.5946/ce.2013.46.3.306
Jin Z, 2008, ENDOSCOPY, V40, P314, DOI 10.1055/s-2007-995476
Jooste V, 2013, INT J CANCER, V132, P2370, DOI 10.1002/ijc.27857
Kahaleh M, 2006, ENDOSCOPY, V38, P355, DOI 10.1055/s-2006-925249
Kahaleh M, 2013, WORLD J GASTROENTERO, V19, P1372, DOI 10.3748/wjg.v19.i9.1372
Kawakami H, 2014, GUT LIVER, V8, P341, DOI 10.5009/gnl.2014.8.4.341
Kawakubo K, 2014, J HEPATO-BIL-PAN SCI, V21, P328, DOI 10.1002/jhbp.27
Kedia P, 2013, CLIN ENDOSC, V46, P543, DOI 10.5946/ce.2013.46.5.543
Koong AC, 2005, INT J RADIAT ONCOL, V63, P320, DOI 10.1016/j.ijrobp.2005.07.002
Koong AC, 2004, INT J RADIAT ONCOL, V58, P1017, DOI 10.1016/j.ijrobp.2003.11.004
Kumta Nikhil A, 2014, Curr Treat Options Gastroenterol, V12, P154, DOI 10.1007/s11938-014-0011-1
LeBlanc JK, 2011, GASTROINTEST ENDOSC, V74, P1300, DOI 10.1016/j.gie.2011.07.073
Loeve US, 2013, GASTROINTEST ENDOSC, V77, P151, DOI 10.1016/j.gie.2012.03.005
Lopes CV, 2007, SCAND J GASTROENTERO, V42, P524, DOI 10.1080/00365520601065093
Mittal MK, 2012, NEUROLOGY, V78, pE57, DOI 10.1212/WNL.0b013e318248df51
Muscatiello N, 2008, ENDOSCOPY, V40, pE83, DOI 10.1055/s-2007-995540
Navaneethan U, 2008, AM J GASTROENTEROL, V103, pS83, DOI 10.14309/00000434-200809001-00215
Ng PY, 2013, ENDOSC ULTRASOUND, V2, P199, DOI 10.4103/2303-9027.121245
Oh HC, 2008, GASTROINTEST ENDOSC, V67, P636, DOI 10.1016/j.gie.2007.09.038
Park DH, 2009, ENDOSCOPY, V41, P842, DOI 10.1055/s-0029-1215133
Park WG, 2010, GASTROINTEST ENDOSC, V71, P513, DOI 10.1016/j.gie.2009.10.030
Pishvaian AC, 2006, GASTROINTEST ENDOSC, V64, P412, DOI 10.1016/j.gie.2006.01.048
Puli SR, 2009, DIGEST DIS SCI, V54, P2330, DOI 10.1007/s10620-008-0651-x
Rana SS, 2012, J DIG ENDOSC, V3, P40, DOI 10.4103/0976-5042.95030
Rana SS, 2010, J GASTROEN HEPATOL, V25, P1087, DOI 10.1111/j.1440-1746.2009.06172.x
Rotman SR, 2012, ENDOSC ULTRASOUND, V1, P61, DOI 10.7178/eus.02.002
Rwigema JCM, 2011, AM J CLIN ONCOL-CANC, V34, P63, DOI 10.1097/COC.0b013e3181d270b4
Sahai AV, 2009, AM J GASTROENTEROL, V104, P326, DOI 10.1038/ajg.2008.64
Sanders MK, 2010, GASTROINTEST ENDOSC, V71, P1178, DOI 10.1016/j.gie.2009.12.020
Seewald S, 2009, DIGEST ENDOSC, V21, pS61, DOI 10.1111/j.1443-1661.2009.00860.x
Seicean A, 2014, WORLD J GASTROENTERO, V20, P110, DOI 10.3748/wjg.v20.i1.110
Singhal S, 2014, THER ADV GASTROENTER, V7, P93, DOI 10.1177/1756283X13506178
Singhal S, 2013, CLIN ENDOSC, V46, P506, DOI 10.5946/ce.2013.46.5.506
Song TJ, 2014, CLIN ENDOSC, V47, P222, DOI 10.5946/ce.2014.47.3.222
Teshima CW, 2014, WORLD J GASTROENTERO, V20, P9976, DOI 10.3748/wjg.v20.i29.9976
Varadarajulu S, 2005, GASTROINTEST ENDOSC, V61, P568, DOI 10.1016/S0016-5107(04)02832-9
Varadarajulu S, 2008, GASTROINTEST ENDOSC, V68, P1102, DOI 10.1016/j.gie.2008.04.028
Varadarajulu S, 2008, GASTROINTEST ENDOSC, V68, P656, DOI 10.1016/j.gie.2008.03.1091

Varadarajulu S, 2007, GASTROINTEST ENDOSC, V66, P1107, DOI 10.1016/j.gie.2007.03.1027
Varadarajulu S, 2012, J DIG ENDOSC, V3, P58, DOI 10.4103/0976-5042.95035
Varadarajulu S, 2013, GASTROENTEROLOGY, V145, P583, DOI 10.1053/j.gastro.2013.05.046
Varadarajulu S, 2011, J GASTROINTEST SURG, V15, P2080, DOI 10.1007/s11605-011-1621-8
Varadarajulu S, 2010, GASTROINTEST ENDOSC, V71, pAB116, DOI 10.1016/j.gie.2010.03.047
Widmer J, 2014, DIGEST ENDOSC, V26, P525, DOI 10.1111/den.12221
Wong GY, 2004, JAMA-J AM MED ASSOC, V291, P1092, DOI 10.1001/jama.291.9.1092
Wyse JM, 2011, J CLIN ONCOL, V29, P3541, DOI 10.1200/JCO.2010.32.2750
Yoon WJ, 2012, ENDOSC ULTRASOUND, V1, P75, DOI 10.7178/eus.02.004
Yusuf TE, 2006, GASTROINTEST ENDOSC, V63, P223, DOI 10.1016/j.gie.2005.09.034
Zhang WY, 2013, WORLD J GASTROENTERO, V19, P3397, DOI 10.3748/wjg.v19.i22.3397
NR 76
TC 3
Z9 4
U1 0
U2 7
PU BAISHIDENG PUBLISHING GROUP INC
PI PLEASANTON
PA 8226 REGENCY DR, PLEASANTON, CA 94588 USA
SN 1007-9327
EI 2219-2840
J9 WORLD J GASTROENTERO
JI World J. Gastroenterol.
PD DEC 14
PY 2015
VL 21
IS 46
BP 12996
EP 13003
DI 10.3748/wjg.v21.i46.12996
PG 8
WC Gastroenterology & Hepatology
WE Science Citation Index Expanded (SCI-EXPANDED)
SC Gastroenterology & Hepatology
GA CY1WY
UT WOS:000366200100002
PM 26675538
OA Green Submitted, hybrid
DA 2026-05-09
ER

PT J

AU Auriemma, F
De Marco, A
Repici, A
Mangiavillano, B
AF Auriemma, Francesco
De Marco, Alessandro
Repici, Alessandro
Mangiavillano, Benedetto
TI ENDOSCOPIC ULTRASOUND GASTRO-ENTERO ANASTOMOSIS FOR BENIGN GASTRIC
OUTLET OBSTRUCTION DUE TO LARGE DUODENAL DIVERTICULA
SO DIGESTIVE DISEASES
LA English
DT Article
ID GASTROJEJUNOSTOMY; MULTICENTER
AB Gastric outlet obstruction (GOO) is a condition characterized by inadequate gastric emptying due to benign or malignant conditions inducing an inadequate oral intake. In recent years a novel therapeutic treatment for this condition is given by the use of electrocautery lumen-apposing metal stents (EC-LAMS). In this case report we want to present an example of a benign condition treated by EUS-guided gastroenterostomy (EUS-GE) with the bi-flanged Spaxus (TM) stent (Taewoong Medical Co.) mounted on electrocautery catheter.
C1 [Auriemma, Francesco; Mangiavillano, Benedetto] Humanitas Mater Domini, Gastrointestinal Endoscopy Unit, Via Gerenzano 2, I-21053 Castellanza, VA, Italy.
[De Marco, Alessandro; Repici, Alessandro; Mangiavillano, Benedetto] Humanitas Univ, Dept Biomed Sci, Milan, Italy.
[Repici, Alessandro] Humanitas Clin & Res Ctr IRCCS, Rozzano, MI, Italy.
C3 Humanitas University
RP Mangiavillano, B (corresponding author), Humanitas Mater Domini, Gastrointestinal Endoscopy Unit, Via Gerenzano 2, I-21053 Castellanza, VA, Italy.
EM bennymangiavillano@gmail.com
RI Repici, Alessandro/HFH-8162-2022
OI Repici, Alessandro/0000-0002-1621-6450
CR [Anonymous], 2018, SURG ENDOSC
Antonelli G, 2020, DIGEST LIVER DIS, V52, P1294, DOI 10.1016/j.dld.2020.04.021
Jeurnink SM, 2010, GASTROINTEST ENDOSC, V71, P490, DOI 10.1016/j.gie.2009.09.042
Jue TL, 2021, GASTROINTEST ENDOSC, V93, P309, DOI 10.1016/j.gie.2020.07.063
Khashab MA, 2017, ENDOSC INT OPEN, V5, pE275, DOI 10.1055/s-0043-101695
Mangiavillano B, 2022, DIGEST DIS, V40, P526, DOI 10.1159/000518539
Tyberg A, 2016, ENDOSC INT OPEN, V4, pE276, DOI 10.1055/s-0042-101789
NR 7
TC 1
Z9 1
U1 0
U2 1
PU KARGER

PI BASEL
PA ALLSCHWILERSTRASSE 10, CH-4009 BASEL, SWITZERLAND
SN 0257-2753
EI 1421-9875
J9 DIGEST DIS
JI Dig. Dis.
PD SEP
PY 2023
VL 41
IS 5
BP 819
EP 821
DI 10.1159/000528885
EA JAN 2023
PG 3
WC Gastroenterology & Hepatology
WE Science Citation Index Expanded (SCI-EXPANDED)
SC Gastroenterology & Hepatology
GA T0CY9
UT WOS:000910539100001
PM 36596281
DA 2026-05-09
ER

PT J
AU Budhram, DR
Nasirzadeh, R
Hookey, L
AF Budhram, Dalton R.
Nasirzadeh, Reza
Hookey, Lawrence
TI An Unusual Case of Lumen-Apposing Metal Stent-Related Splenic Capsule
Hemorrhage
SO JOURNAL OF THE CANADIAN ASSOCIATION OF GASTROENTEROLOGY
LA English
DT Article
C1 [Budhram, Dalton R.] Queens Univ, Dept Med, Kingston, ON, Canada.
[Nasirzadeh, Reza] Queens Univ, Dept Radiol, Kingston, ON, Canada.
[Hookey, Lawrence] Queens Univ, Dept Med, Gastrointestinal Dis Res Unit, Kingston, ON, Canada.
C3 Queens University - Canada; Queens University - Canada; Queens
University - Canada
RP Hookey, L (corresponding author), Queens Univ, Dept Med, Gastrointestinal Dis Res Unit, Kingston, ON, Canada.

EM Lawrence.Hookey@kingstonhsc.ca
NR 0
TC 0
Z9 0
U1 0
U2 0
PU OXFORD UNIV PRESS
PI OXFORD
PA GREAT CLARENDON ST, OXFORD OX2 6DP, ENGLAND
SN 2515-2084
EI 2515-2092
J9 J CAN ASSOC GASTROEN
JI J. Can. Assoc. Gastroenterol.
PD DEC 14
PY 2020
VL 5
IS 1
BP 1
EP 2
DI 10.1093/jcag/gwaa038
PG 2
WC Gastroenterology & Hepatology
WE Emerging Sources Citation Index (ESCI)
SC Gastroenterology & Hepatology
GA M0D8U
UT WOS:001354345100001
PM 35118220
OA Green Submitted, gold
DA 2026-05-09
ER

PT J
AU Lisotti, A
Linguerri, R
Bacchilega, I
Cominardi, A
Marocchi, G
Fusaroli, P
AF Lisotti, Andrea
Linguerri, Romano
Bacchilega, Igor
Cominardi, Anna

Marocchi, Gianmarco
Fusaroli, Pietro
TI EUS-guided gallbladder drainage in high-risk surgical patients with
acute cholecystitis-procedure outcomes and evaluation of mortality
predictors
SO SURGICAL ENDOSCOPY AND OTHER INTERVENTIONAL TECHNIQUES
LA English
DT Article
DE LAMS; Hot-axios; AKI; PT-GBD; ERCP; Acute kidney injury; Charlson
Comorbidity Index
AB Background Recent evidences suggest that gallbladder drainage is the treatment of choice in elderly or high-risk surgical patients with acute cholecystitis (AC). Despite better outcomes compared to other approaches, endoscopic ultrasound-guided gallbladder drainage (EUS-GBD) is burdened by high mortality. The aim of the study was to evaluate predictive factors for mortality in high-risk surgical patients who underwent EUS-GBD for AC. Methods A retrospective analysis of a prospectively maintained database was performed. Electrocautery-enhanced lumen-apposing metal stents were used; all recorded variables were evaluated as potential predictive factors for mortality. Results Thirty-four patients underwent EUS for suspected AC and 25 (44% male, age 78) were finally included. Technical, clinical success rate and adverse events rate were 92%, 88%, and 16%, respectively. 30-day and 1-year mortality were 12% and 32%. On univariate analysis, age-adjusted Charlson Comorbidity Index (CCI) (OR 20.8[4-68.2]), acute kidney injury (AKI) (OR 21.4[2.6-52.1]) and clinical success (OR 8.9[1.2-11.6]) were related to 30-day mortality. On multivariate analysis, CCI and AKI were independently related to long-term mortality. Kaplan-Meier curves showed an increased long-term mortality in patients with CCI > 6 (hazard ratio 7.6[1.7-34.6]) and AKI (hazard ratio 11.3[1.4-91.5]). Conclusions Severe comorbidities and AKI were independent predictive factors confirming of long-term mortality after EUS-GBD. Outcomes of EUS-GBD appear more influenced by patients' conditions rather than by procedure success.
C1 [Lisotti, Andrea; Cominardi, Anna; Marocchi, Gianmarco; Fusaroli, Pietro] Univ Bologna, Dept Med & Surg Sci, Gastroenterol Unit, Hosp Imola, Via Montericco 4, I-40026 Imola, BO, Italy.
[Linguerri, Romano] Hosp Imola, Surg Unit, Imola, Italy.
[Bacchilega, Igor] Hosp Imola, Intens Care Unit, Imola, Italy.
C3 University of Bologna; Azienda USL di Imola; Azienda USL di Imola;
Azienda USL di Imola
RP Lisotti, A (corresponding author), Univ Bologna, Dept Med & Surg Sci, Gastroenterol Unit, Hosp Imola, Via Montericco 4, I-40026 Imola, BO, Italy.
EM lisotti.andrea@gmail.com
RI Lisotti, Andrea/L-6762-2019; Fusaroli, Pietro/AAC-2182-2020
OI Lisotti, Andrea/0000-0002-7724-7402; Marocchi,
Gianmarco/0000-0001-9925-3486; Fusaroli, Pietro/0000-0002-4397-9314
CR [Anonymous], 2012, Kidney Int Suppl (2011), V2, P1, DOI DOI 10.1038/KISUP.2012.1
Brighi N, 2020, ONCOLOGIST, V25, P259, DOI 10.1634/theoncologist.2019-0403
Brighi N, 2019, DIGEST LIVER DIS, V51, P689, DOI 10.1016/j.dld.2018.09.013
Buscarini E, 2014, DIGEST LIVER DIS, V46, P579, DOI 10.1016/j.dld.2014.02.019
CHARLSON M, 1994, J CLIN EPIDEMIOL, V47, P1245, DOI 10.1016/0895-4356(94)90129-5
CHARLSON ME, 1987, J CHRON DIS, V40, P373, DOI 10.1016/0021-9681(87)90171-8

Cotton PB, 2010, GASTROINTEST ENDOSC, V71, P446, DOI 10.1016/j.gie.2009.10.027
Fabbri C, 2014, WORLD J GASTROENTERO, V20, P8424, DOI 10.3748/wjg.v20.i26.8424
Fusaroli, 2019, ENDOSCOPY, V52, P517
Fusaroli P, 2016, ULTRASCHALL MED, V37, P408, DOI 10.1055/s-0035-1553742
Fusaroli P, 2018, ENDOSCOPY, V50, pE299, DOI 10.1055/a-0655-1881
Fusaroli P, 2015, ENDOSC ULTRASOUND, V4, P336, DOI 10.4103/2303-9027.170427
Fusaroli P, 2012, DIGEST DIS SCI, V57, P602, DOI 10.1007/s10620-011-1961-y
Fusaroli P, 2011, J CLIN GASTROENTEROL, V45, P400, DOI 10.1097/MCG.0b013e3181fbde42
Huang SZ, 2021, UPDATES SURG, V73, P481, DOI 10.1007/s13304-020-00894-4
Keus F, 2010, COCHRANE DB SYST REV, DOI 10.1002/14651858.CD008318
Krishnamoorthi R, 2020, SURG ENDOSC, V34, P1904, DOI 10.1007/s00464-020-07409-7
Lammert F, 2016, J HEPATOL, V65, P146, DOI [10.1016/j.jhep.2016.03.005, 10.1016/j.jhep.2016.03.005]
Lisotti A, 2020, DIGEST LIVER DIS, V52, P613, DOI 10.1016/j.dld.2020.03.022
Lisotti A, 2020, ENDOSCOPY, V52, pE263, DOI 10.1055/a-1158-9102
Loozen CS, 2018, BMJ-BRIT MED J, V363, DOI 10.1136/bmj.k3965
Luk SWY, 2019, ENDOSCOPY, V51, P722, DOI 10.1055/a-0929-6603
Mohan BP, 2020, ENDOSCOPY, V52, P96, DOI 10.1055/a-1020-3932
Mori Y, 2018, J HEPATO-BIL-PAN SCI, V25, P87, DOI 10.1002/jhbp.504
Okamoto K, 2018, J HEPATO-BIL-PAN SCI, V25, P55, DOI 10.1002/jhbp.516
Teoh AYB, 2020, GUT, V69, P1085, DOI 10.1136/gutjnl-2019-319996
Wakabayashi G, 2018, J HEPATO-BIL-PAN SCI, V25, P73, DOI 10.1002/jhbp.517
Yokoe M, 2018, J HEPATO-BIL-PAN SCI, V25, P41, DOI 10.1002/jhbp.515
NR 28
TC 36
Z9 37
U1 0
U2 1
PU SPRINGER
PI NEW YORK
PA ONE NEW YORK PLAZA, SUITE 4600, NEW YORK, NY, UNITED STATES
SN 0930-2794
EI 1432-2218
J9 SURG ENDOSC
JI Surg. Endosc.
PD JAN
PY 2022
VL 36
IS 1
BP 569
EP 578
DI 10.1007/s00464-021-08318-z
EA JAN 2021

PG 10
WC Surgery
WE Science Citation Index Expanded (SCI-EXPANDED)
SC Surgery
GA YD3WN
UT WOS:000612601800003
PM 33507383
OA Green Submitted, Bronze
DA 2026-05-09
ER

PT J
AU Bispo, M
Caldeira, A
Leite, S
Marques, S
Moreira, T
Moutinho-Ribeiro, P
Nunes, N
AF Bispo, Miguel
Caldeira, Ana
Leite, Silvia
Marques, Susana
Moreira, Teresa
Moutinho-Ribeiro, Pedro
Nunes, Nuno
CA Portuguese Grp Ultrasound Gastroen
TI Endoscopic Ultrasound-Guided Ablation of Focal Pancreatic Lesions: The
GRUPUGE Perspective
SO GE PORTUGUESE JOURNAL OF GASTROENTEROLOGY
LA English
DT Article
DE Chemoablation; Endoscopic ultrasound-guided ablation; Pancreatic
mucinous neoplasm; Pancreatic neuroendocrine tumour; Pancreatic cyst;
Radiofrequency ablation
ID CYSTIC NEOPLASMS; MANAGEMENT; DIAGNOSIS
AB Focal pancreatic lesions include a heterogeneous group of solid and cystic lesions, with different natures and variable clinical, imagiological, and pathological characteristics. Several endoscopic ultrasound (EUS)-guided ablative techniques have been tested during the last decade for the treatment of these pancreatic lesions, mostly consisting of the injection of ablative agents and, more recently, radiofrequency ablation. The most encouraging EUS-guided ablation outcomes are being reached in the treatment of some pancreatic cystic neoplasms and small (<= 2 cm) pancreatic neuroendocrine tumours (pNETs). Data supporting a potential role of ablative therapies in the treatment of pancreatic ductal adenocarcinoma is still

lacking. In this article, GRUPUGE presents an updated perspective of the potential role of EUS-guided ablation for the treatment of pancreatic cystic neoplasms and pNETs, addressing the selection criteria and technical issues of different techniques and analysing recent data on their safety and efficacy.
C1 [Bispo, Miguel; Marques, Susana] Champalimaud Fdn, Dept Gastroenterol & Digest Endoscopy, Av Brasilia, P-1400038 Lisbon, Portugal.

[Caldeira, Ana] Hosp Amato Lusitano, Dept Gastroenterol, Castelo Branco, Portugal.

[Leite, Silvia] Hosp Senhora da Oliveira, Dept Gastroenterol, Guimaraes, Portugal.

[Moreira, Teresa] Ctr Hosp Univ Porto, Hosp Santo Antonio, Dept Gastroenterol, Porto, Portugal.

[Moutinho-Ribeiro, Pedro] Ctr Hosp & Univ Sao Joao, Dept Gastroenterol, Porto, Portugal.

[Nunes, Nuno] Hosp Divino Espirito Santo, Dept Gastroenterol, Ponta Delgada, Sao Miguel, Portugal.

C3 Fundacao Champalimaud; Universidade do Porto

RP Bispo, M (corresponding author), Champalimaud Fdn, Dept Gastroenterol & Digest Endoscopy, Av Brasilia, P-1400038 Lisbon, Portugal.

EM miguel.bispo@fundacaochampalimaud.pt

RI ; Marques, Susana/E-9291-2010; Nunes, Nuno/GXH-4259-2022

OI Bispo, Miguel/0000-0003-0488-3253;
CR Barthet M, 2019, ENDOSCOPY, V51, P836, DOI 10.1055/a-0824-7067

Bartolini I, 2018, GASTROENT RES PRACT, V2018, DOI 10.1155/2018/9647247

Choi JH, 2017, ENDOSCOPY, V49, P866, DOI 10.1055/s-0043-110030

Dasari A, 2017, JAMA ONCOL, V3, P1335, DOI 10.1001/jamaoncol.2017.0589

Del Chiaro M, 2018, GUT, V67, P789, DOI 10.1136/gutjnl-2018-316027

Elta GH, 2018, AM J GASTROENTEROL, V113, P464, DOI 10.1038/ajg.2018.14

Farrell JJ, 2015, GUT LIVER, V9, P571, DOI 10.5009/gnl15063

Gómez V, 2016, GASTROINTEST ENDOSC, V83, P914, DOI 10.1016/j.gie.2015.08.069

Howe JR, 2020, PANCREAS, V49, P1, DOI 10.1097/MPA.0000000000001454

Lakhtakia S, 2017, DIGEST ENDOSC, V29, P486, DOI 10.1111/den.12833

Lakhtakia S, 2016, GASTROINTEST ENDOSC, V83, P234, DOI 10.1016/j.gie.2015.08.085

Lee DW, 2017, CLIN ENDOSC, V50, P537, DOI 10.5946/ce.2017.131

Moutinho-Ribeiro P, 2019, CLIN RES HEPATOL GAS, V43, P513, DOI 10.1016/j.clinre.2019.02.014

Moyer MT, 2017, GASTROENTEROLOGY, V153, P1295, DOI 10.1053/j.gastro.2017.08.009

Moyer MT, 2016, ENDOSC INT OPEN, V4, pE603, DOI 10.1055/s-0042-105431

Muthusamy VR, 2016, GASTROINTEST ENDOSC, V84, P1, DOI 10.1016/j.gie.2016.04.014

Sachs T, 2009, J GASTROINTEST SURG, V13, P405, DOI 10.1007/s11605-008-0788-0

Slovak R, 2017, J IMMUNOTHER CANCER, V5, DOI 10.1186/s40425-017-0284-8

Tanaka M, 2017, PANCREATOLOGY, V17, P738, DOI 10.1016/j.pan.2017.07.007

Teoh AYB, 2019, ENDOSC INT OPEN, V7, pE1064, DOI 10.1055/a-0959-5870

Wu BU, 2014, AM J GASTROENTEROL, V109, P121, DOI 10.1038/ajg.2013.334

Yachimski P, 2017, GASTROENTEROLOGY, V153, P1183, DOI 10.1053/j.gastro.2017.09.028
NR 22

TC 6

Z9 6

U1 0

U2 2
PU KARGER
PI BASEL
PA ALLSCHWILERSTRASSE 10, CH-4009 BASEL, SWITZERLAND
SN 2341-4545
EI 2387-1954
J9 GE PORT J GASTROENT
JI GE Port. J. Gastroenterol.
PD NOV
PY 2020
VL 27
IS 6
BP 410
EP 416
DI 10.1159/000507895
PG 7
WC Gastroenterology & Hepatology
WE Emerging Sources Citation Index (ESCI)
SC Gastroenterology & Hepatology
GA OT7TV
UT WOS:000591045300006
PM 33251290
OA Green Submitted, gold
DA 2026-05-09
ER

PT J
AU Park, DH
AF Park, Do Hyun
TI Endoscopic ultrasound-guided biliary drainage of hilar biliary
obstruction
SO JOURNAL OF HEPATO-BILIARY-PANCREATIC SCIENCES
LA English
DT Article
DE Biliary obstruction; Endoscopic retrograde cholangiopancreatography;
Endoscopic ultrasound-guided biliary drainage; Endoscopic
ultrasound-guided hepaticogastrostomy; Percutaneous transhepatic biliary
drainage
ID COVERED METAL STENT; FAILED ERCP; DUCT OBSTRUCTION; BILE-DUCT;
HEPATICOGASTROSTOMY; VIDEOS
AB Only 20-30% of patients with hilar cholangiocarcinoma (CC) are candidates for potentially curative resection. However, even after curative (R0) resection, these patients have a disease recurrence rate of up to 76%. The prognosis of hilar

cholangiocarcinoma (CC) is limited by tumor spread along the biliary tree leading to obstructive jaundice, cholangitis, and liver failure. Therefore, palliative biliary drainage may be a major goal for patients with hilar CC. Endoscopic retrograde cholangiopancreatography (ERCP) with stent placement is an established method for palliation of patients with malignant biliary obstruction. However, there are patients for whom endoscopic stent placement is not possible because of failed biliary cannulation or tumor infiltration that limits transpapillary access. In this situation, percutaneous transhepatic biliary drainage (PTBD) is an alternative method. However, PTBD has a relatively high rate of complications and is frequently associated with patient discomfort related to external drainage. Endoscopic ultrasound-guided biliary drainage has therefore been introduced as an alternative to PTBD in cases of biliary obstruction when ERCP is unsuccessful. In this review, the indications, technical tips, outcomes, and the future role of EUS-guided intrahepatic biliary drainage, such as hepaticogastrostomy or hepaticoduodenostomy, for hilar biliary obstruction will be summarized.
C1 Univ Ulsan, Coll Med, Dept Internal Med, Div Gastroenterol,Asan Med Ctr, Seoul 138736, South Korea.
C3 University of Ulsan
RP Park, D (corresponding author), Univ Ulsan, Coll Med, Dept Internal Med, Div Gastroenterol,Asan Med Ctr, 88 Olymp Ro 43 Gil, Seoul 138736, South Korea.
EM dhpark@amc.seoul.kr
CR Artifon ELA, 2007, CLINICS, V62, P799, DOI 10.1590/S1807-59322007000600023
Bories E, 2007, ENDOSCOPY, V39, P287, DOI 10.1055/s-2007-966212
Burmester E, 2003, GASTROINTEST ENDOSC, V57, P246, DOI 10.1067/mge.2003.85
Eum J, 2010, GASTROINTEST ENDOSC, V72, P1279, DOI 10.1016/j.gie.2010.07.026
Itoi T, 2014, J HEPATO-BIL-PAN SCI, V21, pE4, DOI 10.1002/jhbp.43
Kahaleh M, 2006, GASTROINTEST ENDOSC, V64, P52, DOI 10.1016/j.gie.2006.01.063
Lee TH, 2014, WORLD J GASTROENTERO, V20, P5859, DOI 10.3748/wjg.v20.i19.5859
Martins FP, 2010, ENDOSCOPY, V42, pE126, DOI 10.1055/s-0029-1243911
Ogura T, 2015, ENDOSCOPY, V47, P72, DOI 10.1055/s-0034-1378111
Paik WH, 2014, WORLD J GASTROENTERO, V20, P5051, DOI 10.3748/wjg.v20.i17.5051
Park DH, 2013, GASTROINTEST ENDOSC, V78, P91, DOI 10.1016/j.gie.2013.01.042
Park Do Hyun, 2012, Gastrointest Endosc Clin N Am, V22, P271, DOI 10.1016/j.giec.2012.04.009
Park DH, 2012, GASTROINTEST ENDOSC, V75, P692, DOI 10.1016/j.gie.2011.04.013
Park DH, 2011, GASTROINTEST ENDOSC, V74, P1276, DOI 10.1016/j.gie.2011.07.054
Park DH, 2010, GASTROINTEST ENDOSC, V71, P413, DOI 10.1016/j.gie.2009.10.015
Park DH, 2009, AM J GASTROENTEROL, V104, P2168, DOI 10.1038/ajg.2009.254
Park SJ, 2013, GASTROINTEST ENDOSC, V78, P374, DOI 10.1016/j.gie.2013.04.183
Ramírez-Luna MA, 2011, ENDOSCOPY, V43, P826, DOI 10.1055/s-0030-1256406
Savides TJ, 2009, GASTROINTEST ENDOSC, V69, pS3, DOI 10.1016/j.gie.2008.10.060
Song TJ, 2014, GASTROINTEST ENDOSC, V80, P707, DOI 10.1016/j.gie.2014.05.327
Will U, 2007, ENDOSCOPY, V39, P292, DOI 10.1055/s-2007-966215
Will U, 2007, SCAND J GASTROENTERO, V42, P412, DOI 10.1080/00365520600881136
Yamao K, 2010, GUT LIVER, V4, pS67, DOI 10.5009/gnl.2010.4.S1.S67
NR 23
TC 33
Z9 37
U1 0

U2 4
PU WILEY
PI HOBOKEN
PA 111 RIVER ST, HOBOKEN 07030-5774, NJ USA
SN 1868-6974
EI 1868-6982
J9 J HEPATO-BIL-PAN SCI
JI J. Hepato-Biliary-Pancreat. Sci.
PD SEP
PY 2015
VL 22
IS 9
BP 664
EP 668
DI 10.1002/jhbp.271
PG 5
WC Gastroenterology & Hepatology; Surgery
WE Science Citation Index Expanded (SCI-EXPANDED)
SC Gastroenterology & Hepatology; Surgery
GA CP9HS
UT WOS:000360205400004
PM 26178753
OA Bronze
DA 2026-05-09
ER

PT J
AU Rana, SS
Shah, J
Kang, M
Gupta, R
AF Rana, Surinder S.
Shah, Jimil
Kang, Mandeep
Gupta, Rajesh
TI Complications of endoscopic ultrasound-guided transmural drainage of pancreatic fluid collections and their management
SO ANNALS OF GASTROENTEROLOGY
LA English
DT Review
DE Endosonography; bleeding; pseudoaneurysm; perforation; embolism; stents
ID APPOSING METAL STENT; NECROTIZING PANCREATITIS; UP APPROACH;

MORPHOLOGICAL FEATURES; PLASTIC STENTS; RISK-FACTORS; NECROSECTOMY; NECROSIS; MULTICENTER; EUS

AB The development of endoscopic ultrasound (EUS)-guided drainage techniques and lumen-apposing metal stents (LAMS) has markedly reduced the complication rate of endoscopic transmural drainage of pancreatic collections and made these procedures safer and more effective. Despite its improved safety profile, various types of complications, some even life-threatening, can occur after EUS-guided drainage of pancreatic fluid collections. Stent maldeployment/migration, bleeding, gastrointestinal perforation, and air embolism are important complications of EUS-guided drainage of pancreatic collections. Delayed complications weeks after the procedure, such as bleeding and buried LAMS due to the presence of prolonged indwelling transmural stents, have also been described. Careful patient selection, with proper assessment of the size, solid necrotic content and location of the collection, as well as an in-depth understanding of various risk factors that predict complications, are important for a safer and more effective endoscopic transmural drainage. For a better clinical outcome, it is important for the endoscopist to know about various complications of EUS-guided drainage of pancreatic collections, as well as their appropriate management strategies.

C1 [Rana, Surinder S.; Shah, Jimil] Postgrad Inst Med Educ & Res PGIMER, Dept Gastroenterol, Chandigarh, India.
[Kang, Mandeep] Postgrad Inst Med Educ & Res PGIMER, Dept Radiodiag, Chandigarh, India.
[Gupta, Rajesh] Postgrad Inst Med Educ & Res PGIMER, Dept Surg, Chandigarh, India.
C3 Post Graduate Institute of Medical Education & Research (PGIMER), Chandigarh; Post Graduate Institute of Medical Education & Research (PGIMER), Chandigarh; Post Graduate Institute of Medical Education & Research (PGIMER), Chandigarh
RP Rana, SS (corresponding author), PGIMER, Dept Gastroenterol, Chandigarh 160012, India.
EM drsurinderrana@gmail.com

CR Abu Dayyeh BK, 2018, GASTROINTEST ENDOSC, V87, P141, DOI 10.1016/j.gie.2017.04.032
Adler DG, 2019, ENDOSC ULTRASOUND, V8, P36, DOI 10.4103/eus.eus_30_17
[Anonymous], 2018, SCI REP UK
Bang JY, 2019, GUT, V68, P1200, DOI 10.1136/gutjnl-2017-315335
Bang JY, 2017, DIGEST ENDOSC, V29, P83, DOI 10.1111/den.12681
Banks PA, 2013, GUT, V62, P102, DOI 10.1136/gutjnl-2012-302779
Bapaye A, 2017, DIGEST ENDOSC, V29, P104, DOI 10.1111/den.12704
Bonnot B, 2014, AM J GASTROENTEROL, V109, P607, DOI 10.1038/ajg.2013.473
Brimhall B, 2018, CLIN GASTROENTEROL H, V16, P1521, DOI 10.1016/j.cgh.2018.02.021
Chandran S, 2015, GASTROINTEST ENDOSC, V81, P127, DOI 10.1016/j.gie.2014.06.025
Chavan Radhika, 2019, VideoGIE, V4, P27, DOI [10.1016/j.vgie.2018.09.010, 10.1016/j.vgie.2018.09.010]
Chung Il Hyung, 2011, J Interv Gastroenterol, V1, P142
Cui ML, 2014, DIGEST DIS SCI, V59, P1055, DOI 10.1007/s10620-013-2967-4
Elmunzer BJ, 2018, CLIN GASTROENTEROL H, V16, P1851, DOI 10.1016/j.cgh.2018.03.021
Fabbri C, 2015, GASTROINTEST ENDOSC, V82, P585, DOI 10.1016/j.gie.2015.04.035
Fasullo M, 2018, CLIN EXP GASTROENTER, V11, P249, DOI 10.2147/CEG.S167736
Guo JT, 2017, ENDOSC ULTRASOUND, V6, P285, DOI 10.4103/eus.eus_85_17
Guo JT, 2016, SURG ENDOSC, V30, P3114, DOI 10.1007/s00464-015-4557-3
Irani Shayan, 2016, VideoGIE, V1, P25, DOI [10.1016/j.vgie.2016.07.007, 10.1016/j.vgie.2016.07.007]
Iwashita T, 2012, GASTROINTEST ENDOSC, V76, P214, DOI 10.1016/j.gie.2011.06.038

Lakhtakia S, 2017, GASTROINTEST ENDOSC, V85, P1243, DOI 10.1016/j.gie.2016.10.037
Lakhtakia S, 2016, BEST PRACT RES CL GA, V30, P807, DOI 10.1016/j.bpg.2016.10.008
Lakhtakia S, 2016, ENDOSCOPY, V48, pE180, DOI 10.1055/s-0042-106966
Lang GD, 2018, GASTROINTEST ENDOSC, V87, P150, DOI 10.1016/j.gie.2017.06.029
Mehta N, 2018, ENDOSCOPY, V50, pE286, DOI 10.1055/a-0640-2786
Ng PY, 2013, ENDOSC ULTRASOUND, V2, P199, DOI 10.4103/2303-9027.121245
Panamonta N, 2012, EUR J GASTROEN HEPAT, V24, P1355, DOI 10.1097/MEG.0b013e32835871eb
Petrone MC, 2018, UNITED EUR GASTROENT, V6, P1363, DOI 10.1177/2050640618785078
Puri R, 2012, J GASTROEN HEPATOL, V27, P722, DOI 10.1111/j.1440-1746.2012.07089.x
Rana SS, 2019, DIGEST LIVER DIS, V51
Rana SS, 2019, JGH OPEN, P3
Rana SS, 2017, PANCREATOLOGY, V17, P203, DOI 10.1016/j.pan.2017.02.007
Rana SS, 2014, ANN GASTROENTEROL, V27, P258
Rana SS, 2019, EXPERT REV GASTROENT, V13, P331, DOI 10.1080/17474124.2019.1574568
Rana SS, 2018, PANCREATOLOGY, V18, P868, DOI 10.1016/j.pan.2018.10.001
Rana SS, 2018, DIGEST LIVER DIS, V50, P1095, DOI 10.1016/j.dld.2018.05.002
Rana SS, 2014, ENDOSC ULTRASOUND, V3, P118, DOI 10.4103/2303-9027.131039
Rinninella E, 2015, GASTROINTEST ENDOSC, V82, P1039, DOI 10.1016/j.gie.2015.04.006
Rodrigues-Pinto E, 2016, GASTROINTEST ENDOSC, V83, P853, DOI 10.1016/j.gie.2015.11.017
Saftoiu A, 2013, BMC GASTROENTEROL, V13, DOI 10.1186/1471-230X-13-93
Seerden TC, 2016, ENDOSCOPY, V48, pE179, DOI 10.1055/s-0042-107073
Seifert H, 2009, GUT, V58, P1260, DOI 10.1136/gut.2008.163733
Sharaiha RZ, 2016, CLIN GASTROENTEROL H, V14, P1797, DOI 10.1016/j.cgh.2016.05.011
Siddiqui AA, 2016, GASTROINTEST ENDOSC, V83, P699, DOI 10.1016/j.gie.2015.10.020
Tarantino I, 2014, ENDOSCOPY, V46, pE71, DOI 10.1055/s-0033-1359164
van Brunschot S, 2018, LANCET, V391, P51, DOI 10.1016/S0140-6736(17)32404-2
van Brunschot S, 2014, SURG ENDOSC, V28, P1425, DOI 10.1007/s00464-013-3382-9
van Santvoort HC, 2010, NEW ENGL J MED, V362, P1491, DOI 10.1056/NEJMoa0908821
Varadarajulu S, 2007, ENDOSCOPY, V39, pE18, DOI 10.1055/s-2006-944916
Varadarajulu S, 2008, GASTROINTEST ENDOSC, V68, P1102, DOI 10.1016/j.gie.2008.04.028
Varadarajulu S, 2011, GASTROINTEST ENDOSC, V74, P1408, DOI 10.1016/j.gie.2011.07.049
Varadarajulu S, 2011, J GASTROINTEST SURG, V15, P2080, DOI 10.1007/s11605-011-1621-8
Varadarajulu S, 2011, J GASTROEN HEPATOL, V26, P1504, DOI 10.1111/j.1440-1746.2011.06771.x
Vazquez-Sequeiros E, 2016, GASTROINTEST ENDOSC, V84, P450, DOI 10.1016/j.gie.2016.02.044
Venkatachalapathy SV, 2018, ENDOSC INT OPEN, V6, pE259, DOI 10.1055/s-0043-125362
Voermans RP, 2015, J HEPATO-BIL-PAN SCI, V22, P20, DOI 10.1002/jhbp.180
Walter D, 2015, ENDOSCOPY, V47, P63, DOI 10.1055/s-0034-1378113
Wang BH, 2018, WORLD J CLIN CASES, V6, P459, DOI 10.12998/wjcc.v6.i11.459
Wang ZJ, 2019, J GASTROEN HEPATOL, V34, P791, DOI 10.1111/jgh.14557
Yamauchi H, 2019, DIGEST DIS SCI, V64, P1976, DOI 10.1007/s10620-019-05508-7
Yan L, 2019, ENDOSC ULTRASOUND, V8, P172, DOI 10.4103/eus.eus_108_17
Yang D, 2018, ENDOSC INT OPEN, V6, pE1267, DOI 10.1055/a-0732-502

Yasuda I, 2013, ENDOSCOPY, V45, P627, DOI 10.1055/s-0033-1344027
NR 63
TC 40
Z9 47
U1 0
U2 0
PU HELLENIC SOC GASTROENTEROLOGY
PI ATHENS
PA DEMOKRATIAS AVE 67, ATHENS, 15451, GREECE
SN 1108-7471
EI 1792-7463
J9 ANN GASTROENTEROL
JI Ann. Gastroenterol.
PY 2019
VL 32
IS 5
BP 441
EP 450
DI 10.20524/aog.2019.0404
PG 10
WC Gastroenterology & Hepatology
WE Emerging Sources Citation Index (ESCI)
SC Gastroenterology & Hepatology
GA IU1BT
UT WOS:000483312500003
PM 31474789
OA gold
DA 2026-05-09
ER

PT J
AU Kitamura, H
Hijioka, S
Nagashio, Y
Sugawara, S
Nara, S
Sone, M
Esaki, M
Arai, Y
Okusaka, T
Nakajima, A
AF Kitamura, Hidetoshi

Hijioka, Susumu
Nagashio, Yoshikuni
Sugawara, Shunsuke
Nara, Satoshi
Sone, Miyuki
Esaki, Minoru
Arai, Yasuaki
Okusaka, Takuji
Nakajima, Atsushi
TI Use of endoscopic ultrasound-guided biliary drainage as a rescue of re-intervention after the placement of multiple metallic stents for malignant hilar biliary obstruction
SO JOURNAL OF HEPATO-BILIARY-PANCREATIC SCIENCES
LA English
DT Article
DE bile ducts; biliary tract cancer; endoscopic retrograde cholangiopancreatography; endosonography; interventional ultrasonography
ID STRICTURES
AB Background/Purpose Endoscopic transpapillary re-intervention (ETP-RI) after multiple self-expandable metallic stent (SEMS) placement for unresectable malignant hilar biliary obstruction (MHBO) is challenging, but endoscopic ultrasound-guided biliary drainage (EUS-BD) could be an alternative following failed ETP-RI. We investigated appropriate re-intervention (RI) methods in MHBO after multiple SEMS placement and evaluated RI with EUS-BD (EUS-RI) benefits following ETP-RI failure. Methods Patients requiring RI after multiple SEMS placement for MHBO between October 2017 and April 2021 were enrolled. Patients' characteristics, metallic-stent type, stent-placement configuration, overall survival, stent patency, re-intervention results, and re-intervention technical and clinical success rates were reviewed. Results Forty-nine patients underwent ETP-RI for MHBO. ETP-RI's technical success rate was 69.4%. Of 15 failed ETP-RI cases, all underwent EUS-RI, achieving technical and clinical success rates of 86.7% and 100%, respectively. Time to recurrent biliary obstruction (TRBO) after RI was significantly different between EUS-RI and ETP-RI (212 vs 84 days; P = .01). On multivariate analysis, EUS-RI was the only factor associated with TRBO (odds ratio: 4.48; 95% confidence interval: 1.01-19.91; P = .04). Acute pancreatitis and bile peritonitis were present in 6.1% of ETP-RI and 13.3% of EUS-RI cases, respectively; both improved conservatively. Conclusions EUS-RI was effective and safe in difficult ETP-RI cases after multiple SEMS placement for MHBO.
C1 [Kitamura, Hidetoshi; Hijioka, Susumu; Nagashio, Yoshikuni; Okusaka, Takuji] Natl Canc Ctr, Dept Hepatobiliary & Pancreat Oncol, Chuo Ku, 5-1-1 Tsukiji, Tokyo 1040045, Japan.
[Kitamura, Hidetoshi; Nakajima, Atsushi] Yokohama City Univ Med, Dept Gastroenterol & Hepatol, Yokohama, Kanagawa, Japan.
[Sugawara, Shunsuke; Sone, Miyuki; Arai, Yasuaki] Natl Canc Ctr, Dept Diagnost Radiol, Chuo Ku, Tokyo, Japan.
[Nara, Satoshi; Esaki, Minoru] Natl Canc Ctr, Dept Hepatobiliary & Pancreat Surg, Chuo Ku, Tokyo, Japan.
C3 National Cancer Center - Japan; Yokohama City University; National Cancer Center - Japan; National Cancer Center - Japan
RP Hijioka, S (corresponding author), Natl Canc Ctr, Dept Hepatobiliary & Pancreat Oncol, Chuo Ku, 5-1-1 Tsukiji, Tokyo 1040045, Japan.

EM shijioka@ncc.go.jp
RI Hijioka, Susumu/I-1039-2019; takuji, okusaka/ADI-3231-2022; Nara, Satoshi/G-1238-2011
OI Hijioka, Susumu/0000-0002-0394-6876; Nara, Satoshi/0000-0002-3516-8765; Sone, Miyuki/0000-0002-4597-1500; Kitamura, Hidetoshi/0000-0002-6278-1450
FU National Cancer Center Research and Development Fund [31-A-13]; Japanese Foundation for Research and Promotion of Endoscopy
FX The National Cancer Center Research and Development Fund, Grant/Award Number: 31-A-13; The Japanese Foundation for Research and Promotion of Endoscopy
CR Ashat M, 2019, WORLD J GASTROENTERO, V25, P5210, DOI 10.3748/wjg.v25.i34.5210
Chennat J, 2010, GASTROINTEST ENDOSC, V72, P632, DOI 10.1016/j.gie.2010.04.037
Duan F, 2017, CANCER IMAGING, V17, DOI 10.1186/s40644-017-0129-1
Dumonceau JM, 2018, ENDOSCOPY, V50, P910, DOI 10.1055/a-0659-9864
Fujii M, 2013, HEPATO-GASTROENTEROL, V60, P447, DOI 10.5754/hge12679
Fujita T, 2019, GASTROENT RES PRACT, V2019, DOI 10.1155/2019/5928040
Hong JB, 2017, MEDICINE, V96, DOI 10.1097/MD.0000000000008867
Inoue T, 2016, DIGEST ENDOSC, V28, P731, DOI 10.1111/den.12657
Isayama H, 2015, DIGEST ENDOSC, V27, P259, DOI 10.1111/den.12379
Kanno Y, 2017, INTERNAL MED, V56, P3145, DOI 10.2169/internalmedicine.9001-17
Kitamura K, 2019, THER ADV GASTROINTES, V12, DOI 10.1177/2631774519846345
Law R, 2013, DIGEST DIS SCI, V58, P2667, DOI 10.1007/s10620-013-2671-4
Lee T H, 2014, Minerva Gastroenterol Dietol, V60, P135
Maruki Y, 2020, GASTROINTEST ENDOSC, V92, P763, DOI 10.1016/j.gie.2020.03.003
Maruyama H, 2017, HEPATOB PANCREAT DIS, V16, P659, DOI 10.1016/S1499-3872(17)60072-9
Minaga K, 2017, SURG ENDOSC, V31, P4764, DOI 10.1007/s00464-017-5553-6
Moole H, 2016, CAN J GASTROENTEROL, V2016, DOI 10.1155/2016/4726078
Nakai Y, 2019, CLIN ENDOSC, V52, P220
Ogura T, 2017, J GASTROEN HEPATOL, V32, P163, DOI 10.1111/jgh.13437
Okuno M, 2019, J HEPATO-BIL-PAN SCI, V26, P211, DOI 10.1002/jhbp.626
Park DH, 2010, GASTROINTEST ENDOSC, V71, P413, DOI 10.1016/j.gie.2009.10.015
Park JM, 2016, HEPATOB PANCREAT DIS, V15, P633, DOI 10.1016/S1499-3872(16)60107-8
Park SJ, 2013, GASTROINTEST ENDOSC, V78, P374, DOI 10.1016/j.gie.2013.04.183
Ridtitid W, 2010, SURG ENDOSC, V24, P2216, DOI 10.1007/s00464-010-0931-3
Sharaiha RZ, 2016, SURG ENDOSC, V30, P5500, DOI 10.1007/s00464-016-4913-y
Son JH, 2018, HEPATOB PANCREAT DIS, V17, P437, DOI 10.1016/j.hbpd.2018.07.007
Uppal DS, 2015, WORLD J GASTRO ENDOS, V7, P675, DOI 10.4253/wjge.v7.i7.675
Vienne A, 2010, GASTROINTEST ENDOSC, V72, P728, DOI 10.1016/j.gie.2010.06.040
Xia MX, 2020, GASTROINTEST ENDOSC, V91, P1117, DOI 10.1016/j.gie.2019.12.023
Yang MJ, 2018, GUT LIVER, V12, P722, DOI 10.5009/gnl17468
NR 30

TC 11
Z9 11
U1 0
U2 7
PU WILEY
PI HOBOKEN
PA 111 RIVER ST, HOBOKEN 07030-5774, NJ USA
SN 1868-6974
EI 1868-6982
J9 J HEPATO-BIL-PAN SCI
JI J. Hepato-Biliary-Pancreat. Sci.
PD MAR
PY 2022
VL 29
IS 3
BP 404
EP 414
DI 10.1002/jhbp.1059
EA NOV 2021
PG 11
WC Gastroenterology & Hepatology; Surgery
WE Science Citation Index Expanded (SCI-EXPANDED)
SC Gastroenterology & Hepatology; Surgery
GA 0G4JZ
UT WOS:000720007700001
PM 34661973
DA 2026-05-09
ER

PT J
AU Puri, R
Manrai, M
Thandassery, RB
Alfadda, AA
AF Puri, Rajesh
Manrai, Manish
Thandassery, Ragesh Babu
Alfadda, Abdulrahman A.
TI Endoscopic ultrasound in the diagnosis and management of carcinoma
pancreas
SO WORLD JOURNAL OF GASTROINTESTINAL ENDOSCOPY
LA English

DT Article
DE Carcinoma pancreas; Endoscopic ultrasound; Treatment
ID FINE-NEEDLE-ASPIRATION; CELIAC PLEXUS NEUROLYSIS; EUS-GUIDED FNA;
IN-SITU HYBRIDIZATION; FIDUCIAL PLACEMENT; RETROGRADE PANCREATOGRAPHY;
DIFFERENTIAL-DIAGNOSIS; COMPUTED-TOMOGRAPHY; FOCAL PANCREATITIS;
RADIATION-THERAPY
AB Endoscopic ultrasound (EUS) has become an important component in the diagnosis and treatment of carcinoma pancreas. With the advent of advanced imaging techniques and tissue acquisition methods the role of EUS is becoming increasingly important. Small pancreatic tumors can be reliably diagnosed with EUS. EUS guided fine needle aspiration establishes diagnosis in some cases. EUS plays an important role in staging of carcinoma pancreas and in some important therapeutic methods that include celiac plexus neurolysis, EUS guided biliary drainage and drug delivery. In this review we attempt to review the role of EUS in diagnosis and management of carcinoma pancreas.
C1 [Puri, Rajesh] Medanta, Inst Digest & Hepatobiliary Sci, Gurgaon 122001, Haryana, India.
[Manrai, Manish] Postgrad Inst Med Educ & Res, Dept Gastroenterol, Chandigarh 160012, India.
[Thandassery, Ragesh Babu] Hamad Gen Hosp, Div Gastroenterol, Dept Med, 2 South 2, Doha 3050, Qatar.
[Alfadda, Abdulrahman A.] King Faisal Specialist Hosp & Res Ctr, Div Gastroenterol, Dept Med, Riyadh 12713, Saudi Arabia.
C3 Post Graduate Institute of Medical Education & Research (PGIMER), Chandigarh; Hamad Medical Corporation; Hamad General Hospital; King Faisal Specialist Hospital & Research Center
RP Thandassery, RB (corresponding author), Hamad Gen Hosp, Div Gastroenterol, Dept Med, 2 South 2, Doha 3050, Qatar.
EM doc.ragesh@gmail.com
RI Manrai, Manish/M-8095-2019; Thandassery, Ragesh/GPX-8591-2022
CR Agarwal B, 2004, AM J GASTROENTEROL, V99, P844, DOI 10.1111/j.1572-0241.2004.04177.x
Aljebreen AM, 2007, WORLD J GASTROENTERO, V13, P3962, DOI 10.3748/wjg.v13.i29.3962
[Anonymous], VIDEO J ENCY GI ENDO
Arcidiacono PG, 2011, COCHRANE DB SYST REV, DOI 10.1002/14651858.CD007519.pub2
Arcidiacono PG, 2012, GASTROINTEST ENDOSC, V76, P1142, DOI 10.1016/j.gie.2012.08.006
Ardengh JC, 2007, WORLD J GASTROENTERO, V13, P3112, DOI 10.3748/wjg.v13.i22.3112
Arslan A, 2001, EUR J RADIOL, V38, P151, DOI 10.1016/S0720-048X(00)00280-1
Becker D, 2001, GASTROINTEST ENDOSC, V53, P784, DOI 10.1067/mge.2001.115007
Bhutani MS, 1997, ENDOSCOPY, V29, P635, DOI 10.1055/s-2007-1004270
Bhutani MS, 2004, ENDOSCOPY, V36, P385, DOI 10.1055/s-2004-814320
Bhutani MS, 1997, ENDOSCOPY, V29, P854, DOI 10.1055/s-2007-1004321
Bronstein YL, 2004, AM J ROENTGENOL, V182, P619, DOI 10.2214/ajr.182.3.1820619
Catalano MF, 1998, GASTROINTEST ENDOSC, V48, P11, DOI 10.1016/S0016-5107(98)70122-1
Chang KJ, 2000, CANCER-AM CANCER SOC, V88, P1325, DOI 10.1002/(SICI)1097-0142(20000315)88:6<1325::AID-CNCR8>3.0.CO;2-T
Chen J, 2012, J CANCER RES CLIN, V138, P1433, DOI 10.1007/s00432-012-1268-1
Dhir V, 2015, GASTROINTEST ENDOSC, V81, P913, DOI 10.1016/j.gie.2014.09.054
DiMaio CJ, 2010, GASTROINTEST ENDOSC, V71, P1204, DOI 10.1016/j.gie.2010.01.003
Doi S, 2013, ENDOSCOPY, V45, P362, DOI 10.1055/s-0032-1326225

Egorov VI, 2013, WORLD J GASTRO SURG, V5, P83, DOI 10.4240/wjgs.v5.i4.83
Eloubeidi MA, 2003, CANCER CYTOPATHOL, V99, P285, DOI 10.1002/cncr.11643
Fabbri C, 2011, DIGEST LIVER DIS, V43, P647, DOI 10.1016/j.dld.2011.04.005
Faigel DO, 1997, J CLIN ONCOL, V15, P1439, DOI 10.1200/JCO.1997.15.4.1439
Fuccio L, 2013, GASTROINTEST ENDOSC, V78, P596, DOI 10.1016/j.gie.2013.04.162
Fusaroli P, 2010, CLIN GASTROENTEROL H, V8, P629, DOI 10.1016/j.cgh.2010.04.012
Giovannini M, 2006, ENDOSCOPY, V38, P344, DOI 10.1055/s-2006-925158
Giovannini M, 2007, THESCIENTIFICWORLDJO, V7, P341, DOI 10.1100/tsw.2007.28
Giovannini M, 2009, WORLD J GASTROENTERO, V15, P1587, DOI 10.3748/wjg.15.1587
Gress F, 2001, ANN INTERN MED, V134, P459, DOI 10.7326/0003-4819-134-6-200103200-00010
Harewood GC, 2002, AM J GASTROENTEROL, V97, P1386, DOI 10.1016/S0002-9270(02)04133-3
Hébert-Magee S, 2013, CYTOPATHOLOGY, V24, P159, DOI 10.1111/cyt.12071
Hecht JR, 2012, GASTROINTEST ENDOSC, V75, P332, DOI 10.1016/j.gie.2011.10.007
Hecht JR, 2003, CLIN CANCER RES, V9, P555
Herman JM, 2013, J CLIN ONCOL, V31, P886, DOI 10.1200/JCO.2012.44.7516
Hirooka Y, 1998, AM J GASTROENTEROL, V93, P632
Hirooka Y, 2009, PANCREAS, V38, pE69, DOI 10.1097/MPA.0b013e318197a9e3
Hocke M, 2006, WORLD J GASTROENTERO, V12, P246, DOI 10.3748/wjg.v12.i2.246
Iglesias-Garcia J, 2010, GASTROENTEROLOGY, V139, P1172, DOI 10.1053/j.gastro.2010.06.059
Iglesias-Garcia J, 2009, GASTROINTEST ENDOSC, V70, P1101, DOI 10.1016/j.gie.2009.05.011
Irisawa A, 2007, PANCREAS, V35, P189, DOI 10.1097/01.mpa.0000250141.25639.e9
Iwashita T, 2014, CLIN J GASTROENTEROL, V7, P94, DOI 10.1007/s12328-014-0467-5
Jhala NC, 2007, ANN DIAGN PATHOL, V11, P176, DOI 10.1016/j.anndiagpath.2006.03.005
Jin Z, 2008, ENDOSCOPY, V40, P314, DOI 10.1055/s-2007-995476
Kaufman M, 2010, J CLIN GASTROENTEROL, V44, P127, DOI 10.1097/MCG.0b013e3181bb854d
Khashab MA, 2012, GASTROINTEST ENDOSC, V76, P962, DOI 10.1016/j.gie.2012.07.006
Kitano M, 2015, DIGEST ENDOSC, V27, P60, DOI 10.1111/den.12454
Kitano M, 2012, AM J GASTROENTEROL, V107, P303, DOI 10.1038/ajg.2011.354
Klapman JB, 2003, AM J GASTROENTEROL, V98, P1289, DOI 10.1016/S0002-9270(03)00246-6
Koelblinger C, 2011, RADIOLOGY, V259, P757, DOI 10.1148/radiol.11101189
Kubiliun N, 2011, GASTROINTEST ENDOSC, V74, P541, DOI 10.1016/j.gie.2011.04.043
Law JK, 2013, SURG ENDOSC, V27, P3921, DOI 10.1007/s00464-013-2975-7
Layfield LJ, 2014, DIAGN CYTOPATHOL, V42, P351, DOI 10.1002/dc.23093
LeBlanc JK, 2004, GASTROINTEST ENDOSC, V59, P475, DOI 10.1016/S0016-5107(03)02863-3
LeBlanc JK, 2011, GASTROINTEST ENDOSC, V74, P1300, DOI 10.1016/j.gie.2011.07.073
Lee TY, 2013, GUT LIVER, V7, P599, DOI 10.5009/gnl.2013.7.5.599
Mishra G, 2006, GASTROINTEST ENDOSC, V63, P648, DOI 10.1016/j.gie.2005.11.056
Möller K, 2009, GASTROINTEST ENDOSC, V70, P60, DOI 10.1016/j.gie.2008.10.008
Napoleon B, 2010, ENDOSCOPY, V42, P564, DOI 10.1055/s-0030-1255537
NATTERMANN C, 1993, ENDOSCOPY, V25, P565, DOI 10.1055/s-2007-1010406
Ngamruengphong S, 2010, GASTROINTEST ENDOSC, V72, P78, DOI 10.1016/j.gie.2010.01.072
Oh HC, 2008, GASTROINTEST ENDOSC, V67, P636, DOI 10.1016/j.gie.2007.09.038

Oh HC, 2011, GASTROENTEROLOGY, V140, P172, DOI 10.1053/j.gastro.2010.10.001
Othman MO, 2011, DIGEST LIVER DIS, V43, P587, DOI 10.1016/j.dld.2011.05.014
Pai M, 2015, WORLD J GASTRO SURG, V7, P52, DOI 10.4240/wjgs.v7.i4.52
Park JS, 2014, WORLD J GASTROENTERO, V20, P518, DOI 10.3748/wjg.v20.i2.518
Park WG, 2010, GASTROINTEST ENDOSC, V71, P513, DOI 10.1016/j.gie.2009.10.030
Penman ID, 2009, GASTROINTEST ENDOSC, V69, pS28, DOI 10.1016/j.gie.2008.11.004
Pishvaian AC, 2006, GASTROINTEST ENDOSC, V64, P412, DOI 10.1016/j.gie.2006.01.048
Poley JW, 2009, AM J GASTROENTEROL, V104, P2175, DOI 10.1038/ajg.2009.276
Puli SR, 2013, PANCREAS, V42, P20, DOI 10.1097/MPA.0b013e3182546e79
Puli SR, 2009, DIGEST DIS SCI, V54, P2330, DOI 10.1007/s10620-008-0651-x
Reicher S, 2011, PANCREAS, V40, P1057, DOI 10.1097/MPA.0b013e3182200201
ROSCH T, 1991, GASTROINTEST ENDOSC, V37, P347, DOI 10.1016/S0016-5107(91)70729-3
Saftoiu A, 2011, ENDOSCOPY, V43, P596, DOI 10.1055/s-0030-1256314
Saif MW, 2011, J PANCREAS, V12, P316
Sakamoto H, 2009, J GASTROEN HEPATOL, V24, P384, DOI 10.1111/j.1440-1746.2008.05636.x
Sanders MK, 2010, GASTROINTEST ENDOSC, V71, P1178, DOI 10.1016/j.gie.2009.12.020
Schrader H, 2012, Ultraschall Med, V33, pE196, DOI 10.1055/s-0031-1273256
Seicean A, 2010, ULTRASCHALL MED, V31, P571, DOI 10.1055/s-0029-1245833
Siddiqui AA, 2011, GASTROINTEST ENDOSC, V74, P535, DOI 10.1016/j.gie.2011.04.039
Soriano A, 2004, AM J GASTROENTEROL, V99, P492, DOI 10.1111/j.1572-0241.2004.04087.x
Surveillance Research Program, SEER STAT FACT SHEET
Téllez-Avila FI, 2013, ENDOSC ULTRASOUND, V2, P153, DOI [10.4103/2303-9027.117664, 10.7178/eus.06.007]
Tuca Albert, 2012, Cancer Manag Res, V4, P159, DOI 10.2147/CMAR.S29297
Varadarajulu S, 2005, GASTROINTEST ENDOSC, V62, P728, DOI 10.1016/j.gie.2005.06.051
Volmar KE, 2005, GASTROINTEST ENDOSC, V61, P854, DOI 10.1016/S0016-5107(05)00364-0
Weilert F, 2014, SURG ENDOSC, V28, P3193, DOI 10.1007/s00464-014-3588-5
Wiersema MJ, 1997, GASTROENTEROLOGY, V112, P1087, DOI 10.1016/S0016-5085(97)70164-1
Wyse JM, 2011, J CLIN ONCOL, V29, P3541, DOI 10.1200/JCO.2010.32.2750
Yang D, 2014, PANCREAS, V43, P922, DOI 10.1097/MPA.0000000000000157
NR 89
TC 3
Z9 5
U1 0
U2 3
PU BAISHIDENG PUBLISHING GROUP INC
PI PLEASANTON
PA 8226 REGENCY DR, PLEASANTON, CA 94588 USA
SN 1948-5190
J9 WORLD J GASTRO ENDOS
JI World J. Gastrointest. Endosc.
PD JAN 25
PY 2016

VL 8
IS 2
BP 67
EP 76
DI 10.4253/wjge.v8.i2.67
PG 10
WC Gastroenterology & Hepatology
WE Emerging Sources Citation Index (ESCI)
SC Gastroenterology & Hepatology
GA DD1UM
UT WOS:000369707500004
PM 26839647
OA Green Submitted, hybrid
DA 2026-05-09
ER

PT J
AU Armellini, E
Metelli, F
Anderloni, A
Cominardi, A
Aragona, G
Marini, M
Pace, F
AF Armellini, Elia
Metelli, Flavio
Anderloni, Andrea
Cominardi, Anna
Aragona, Giovanni
Marini, Michele
Pace, Fabio
TI Lumen-apposing-metal stent misdeployment in endoscopic ultrasound-guided drainages: A systematic review focusing on issues and rescue management
SO WORLD JOURNAL OF GASTROENTEROLOGY
LA English
DT Review
DE Lams misdeployment; Endoscopic ultrasound-guided drainage; Lams maldeployment; Biliary drainage; Gallbladder drainage; Pancreatic fluid collections; Lumen-apposing metal stents
ID PANCREATIC FLUID COLLECTIONS; DISTAL BILIARY OBSTRUCTION; TRANSMURAL GALLBLADDER DRAINAGE; PERCUTANEOUS CHOLECYSTOSTOMY; TRANSLUMINAL DRAINAGE; MALIGNANT BILIARY; CHOLEDOCHODUODENOSTOMY; MULTICENTER;

SAFETY; EFFICACY

AB BACKGROUNDThe introduction of lumen-apposing metal stents (LAMS) for endoscopic ultrasound (EUS)-guided drainages has marked a turning point in the field of interventional ultrasound and it is gathering worldwide diffusion in different clinical settings. Nevertheless, the procedure may conceal unexpected pitfalls. LAMS misdeployment is the most frequent cause of technical failure and it can be considered a procedure-related adverse event when it hampers the conclusion of the planned procedure or results in significant clinical consequences. Stent misdeployment can be managed successfully by endoscopic rescue maneuvers to allow the completion of the procedure. To date, no standardized indication is available to guide an appropriate rescue strategy depending on the type of procedure or of misdeployment.AIMTo evaluate the incidence of LAMS misdeployment during EUS-guided choledochoduodenostomy (EUS-CDS), gallbladder drainage (EUS-GBD) and pancreatic fluid collections drainage (EUS-PFC) and to describe the endoscopic rescue strategies adopted under the circumstance.METHODSWe conducted a systematic review of the literature on PubMed by searching for studies published up to October 2022. The search was carried out using the exploded medical subject heading terms "lumen apposing metal stent", "LAMS", "endoscopic ultrasound" and "choledochoduodenostomy" or "gallbladder" or "pancreatic fluid collections". We included in the review on-label EUS-guided procedures namely EUS-CDS, EUS-GBD and EUS-PFC. Only those publications reporting EUS-guided LAMS positioning were considered. The studies reporting a technical success rate of 100% and other procedure-related adverse events were considered to calculate the overall rate of LAMS misdeployment, while studies not reporting the causes of technical failure were excluded. Case reports were considered only for the extraction of data regarding the issues of misdeployment and rescue techniques. The following data were collected from each study: Author, year of publication, study design, study population, clinical indication, technical success, reported number of misdeployment, stent type and size, flange misdeployed and type of rescue strategy.RESULTSThe overall technical success rate of EUS-CDS, EUS-GBD and EUS-PFC was 93.7%, 96.1%, and 98.1% respectively. Significant rates of LAMS misdeployment have been reported for EUS-CDS, EUS-GBD and EUS-PFC drainage, respectively 5.8%, 3.4%, and 2.0%. Endoscopic rescue treatment was feasible in 86.8%, 80%, and 96.8% of cases. Non endoscopic rescue strategies were required only in 10.3%, 16% and 3.2% for EUS-CDS, EUS-GBD, and EUS-PFC. The endoscopic rescue techniques described were over-the-wire deployment of a new stent through the created fistula tract in 44.1%, 8% and 64.5% and stent-in-stent in 23.5%, 60%, and 12.9%, respectively for EUS-CDS, EUS-GBD, and EUS-PFC. Further therapeutic option were endoscopic rendezvous in 11.8% of EUS-CDS and repeated procedure of EUS-guided drainage in 16.1% of EUS-PFC.CONCLUSIONLAMS misdeployment is a relatively common adverse event in EUS-guided drainages. There is no consensus on the best rescue approach in these cases and the choice is often made by the endoscopist relying upon the clinical scenario, anatomical characteristics, and local expertise.

C1 [Armellini, Elia; Metelli, Flavio; Pace, Fabio] ASST Bergamoest, Dept Gastroenterol, I-24068 Bergamo, Italy.
[Anderloni, Andrea] Fdn IRCCS Policlin San Matteo, Gastroenterol & Digest Endoscopy Unit, I-27100 Pavia, Italy.
[Cominardi, Anna; Aragona, Giovanni] Guglielmo da Saliceto Hosp, Dept Gastroenterol, I-29121 Piacenza, Italy.
[Marini, Michele] ASST Bergamoest, Dept Gen Surg, I-24068 Seriate, Italy.
[Armellini, Elia] ASST Bergamoest, Dept Gastroenterol, Via Paderno 21, I-24068 Bergamo, Italy.

C3 IRCCS Fondazione San Matteo; Guglielmo da Saliceto Hospital

RP Armellini, E (corresponding author), ASST Bergamoest, Dept Gastroenterol, Via Paderno 21, I-24068 Bergamo, Italy.

EM elia_armellini@hotmail.com

RI Armellini, Elia/V-1705-2019

CR Adler DG, 2019, ENDOSC ULTRASOUND, V8, P136, DOI 10.4103/eus.eus_58_18
Adler DG, 2018, ENDOSC ULTRASOUND, V7, P422, DOI 10.4103/eus.eus_39_18
Adler DG, 2019, ENDOSC ULTRASOUND, V8, P36, DOI 10.4103/eus.eus_30_17

Amato A, 2021, ENDOSCOPY, V53, P1037, DOI 10.1055/a-1324-7919
Anderloni A, 2021, SURG ENDOSC, V35, P1741, DOI 10.1007/s00464-020-07567-8
Anderloni A, 2019, WORLD J GASTROENTERO, V25, P3857, DOI 10.3748/wjg.v25.i29.3857
Anderloni A, 2018, ANN GASTROENTEROL, V31, P735, DOI 10.20524/aog.2018.0299
Anderloni A, 2019, GASTROINTEST ENDOSC, V89, P69, DOI [10.1016/j.gie.2018.04.1746, 10.1016/j.gie.2018.08.047]
Anderloni A, 2017, ENDOSC INT OPEN, V5, pE1100, DOI 10.1055/s-0043-118828
Anderloni A, 2016, ENDOSCOPY, V48, pE321, DOI 10.1055/s-0042-117425
Armellini E, 2023, ENDOSCOPY, V55, pE37, DOI 10.1055/a-1929-9148
Armellini E, 2015, ENDOSCOPY, V47, pE600, DOI 10.1055/s-0034-1393677
Arvanitakis M, 2018, ENDOSCOPY, V50, P524, DOI 10.1055/a-0588-5365
Binmoeller KF, 2011, ENDOSCOPY, V43, P337, DOI 10.1055/s-0030-1256127
Brückner S, 2015, ENDOSCOPY, V47, P858, DOI 10.1055/s-0034-1392104
Chan JHY, 2018, CLIN ENDOSC, V51, P150, DOI 10.5946/ce.2017.125
Chang JI, 2019, ENDOSC INT OPEN, V7, pE655, DOI 10.1055/a-0826-4309
Chin JYL, 2020, ENDOSC INT OPEN, V08, pE1633, DOI 10.1055/a-1236-3217
Cho DH, 2019, SURG ENDOSC, V33, P2135, DOI 10.1007/s00464-018-6485-5
Cho SH, 2020, GASTROINTEST ENDOSC, V91, P543, DOI 10.1016/j.gie.2019.09.042
Cotton PB, 2010, GASTROINTEST ENDOSC, V71, P446, DOI 10.1016/j.gie.2009.10.027
Curieses-Luengo M, 2019, REV ESP ENFERM DIG, V111, P243, DOI 10.17235/reed.2019.5924/2018
Sanz MD, 2021, SURG ENDOSC, V35, P6754, DOI 10.1007/s00464-020-08179-y
de la Serna-Higuera C, 2013, GASTROINTEST ENDOSC, V77, P303, DOI 10.1016/j.gie.2012.09.021
Despott EJ, 2020, ENDOSCOPY, V52, pE300, DOI 10.1055/a-1085-9343
Dhindsa BS, 2020, ENDOSC ULTRASOUND, V9, P101, DOI 10.4103/eus.eus_80_19
Di Mitri R, 2022, SURG ENDOSC, V36, P4553, DOI 10.1007/s00464-021-08808-0
Dollhopf M, 2017, GASTROINTEST ENDOSC, V86, P636, DOI 10.1016/j.gie.2017.02.027
El Chafic AH, 2019, DIGEST DIS SCI, V64, P3321, DOI 10.1007/s10620-019-05688-2
Fabbri C, 2022, WORLD J EMERG SURG, V17, DOI 10.1186/s13017-022-00409-z
Fugazza A, 2022, GASTROINTEST ENDOSC, V95, P896, DOI 10.1016/j.gie.2021.12.032
Fugazza A, 2020, ENDOSCOPY, V52, pE430, DOI 10.1055/a-1158-8749
Fugazza A, 2020, GASTROINTEST ENDOSC, V91, P574, DOI 10.1016/j.gie.2019.11.021
de Paredes AGG, 2020, ENDOSC INT OPEN, V8, pE6, DOI 10.1055/a-1031-9295
Garcia-Sumalla A, 2021, SURG ENDOSC, V35, P4873, DOI 10.1007/s00464-021-08435-9
Garg R, 2020, CLIN ENDOSC, V53, P480, DOI 10.5946/ce.2019.116
Garg Rajat, 2018, Korean J Gastroenterol, V72, P83, DOI [10.4166/kjg.2018.72.2.83, 10.4166/kjg.2018.72.2.83]
Giovannini M, 2001, ENDOSCOPY, V33, P898, DOI 10.1055/s-2001-17324
Graves Miles, 2021, VideoGIE, V6, P234, DOI [10.1016/j.vgie.2021.01.010, 10.1016/j.vgie.2021.01.010]
Guzmán-Calderón E, 2022, J HEPATO-BIL-PAN SCI, V29, P198, DOI 10.1002/jhbp.1008
Han SY, 2019, SCI REP-UK, V9, DOI 10.1038/s41598-019-52993-x
Hathorn KE, 2019, ENDOSC INT OPEN, V7, pE1432, DOI 10.1055/a-0990-9488
Higa JT, 2019, GASTROINTEST ENDOSC, V90, P483, DOI 10.1016/j.gie.2019.04.238
Hindryckx P, 2021, SURG ENDOSC, V35, P6013, DOI 10.1007/s00464-020-08090-6

Holt BA, 2016, GASTROINTEST ENDOSC, V83, P160, DOI 10.1016/j.gie.2015.06.019
Irani S, 2017, CLIN GASTROENTEROL H, V15, P738, DOI 10.1016/j.cgh.2016.12.021
Irani S, 2015, GASTROINTEST ENDOSC, V82, P1110, DOI 10.1016/j.gie.2015.05.045
Issa D, 2021, ENDOSCOPY, V53, P827, DOI 10.1055/a-1259-0349
Itoi T, 2014, GASTROINTEST ENDOSC, V79, P715, DOI 10.1016/j.gie.2013.11.021
Itoi T, 2012, GASTROINTEST ENDOSC, V75, P870, DOI 10.1016/j.gie.2011.10.020
Jacques J, 2020, GASTROINTEST ENDOSC, V92, P134, DOI 10.1016/j.gie.2020.01.055
Jacques J, 2019, ENDOSCOPY, V51, P540, DOI 10.1055/a-0735-9137
Jain D, 2018, CLIN ENDOSC, V51, P450, DOI 10.5946/ce.2018.024
James TW, 2019, ENDOSC INT OPEN, V7, pE1099, DOI 10.1055/a-0965-6662
Kawakami H, 2014, GUT LIVER, V8, P341, DOI 10.5009/gnl.2014.8.4.341
Khan S, 2021, J GASTROEN HEPATOL, V36, P3395, DOI 10.1111/jgh.15658
Kunda R, 2016, SURG ENDOSC, V30, P5002, DOI 10.1007/s00464-016-4845-6
Ladd AM, 2020, WORLD J GASTRO ENDOS, V12, P149, DOI 10.4253/wjge.v12.i5.149
Larghi A, 2022, ENDOSC ULTRASOUND, V11, P84, DOI 10.4103/EUS-D-21-00033
Law R, 2016, CLIN GASTROENTEROL H, V14, P476, DOI 10.1016/j.cgh.2015.10.026
Law ST, 2018, SURG ENDOSC, V32, P2448, DOI 10.1007/s00464-017-5946-6
Li Jing, 2021, Chronic Dis Transl Med, V7, P157, DOI [10.1016/j.cdtm.2021.07.001, 10.1016/j.cdtm.2021.07.001]
Ligresti D, 2018, ENDOSCOPY, V50, pE190, DOI 10.1055/a-0605-3198
Luk SWY, 2019, ENDOSCOPY, V51, P722, DOI 10.1055/a-0929-6603
Mangiavillano B, 2022, GASTROINTEST ENDOSC, V95, P115, DOI 10.1016/j.gie.2021.07.021
Mayumi T, 2018, J HEPATO-BIL-PAN SCI, V25, P96, DOI 10.1002/jhbp.519
McCarty TR, 2021, SURG ENDOSC, V35, P4964, DOI 10.1007/s00464-020-07758-3
Melita G, 2021, J CLIN MED, V10, DOI 10.3390/jcm10122638
Mohan BP, 2019, ENDOSC ULTRASOUND, V8, P241, DOI 10.4103/eus.eus_63_18
Moon JH, 2014, GASTROINTEST ENDOSC, V79, P990, DOI 10.1016/j.gie.2014.02.015
Mussetto A, 2018, ANN GASTROENTEROL, V31, P535, DOI 10.20524/aog.2018.0287
Ngamruengphong S, 2015, GASTROINTEST ENDOSC, V82, P1124, DOI 10.1016/j.gie.2015.06.009
Nieto J, 2023, J CLIN GASTROENTEROL, V57, P218, DOI 10.1097/MCG.0000000000001545
Nishiguchi K, 2021, ENDOSC ULTRASOUND, V10, P448, DOI 10.4103/EUS-D-20-00130
Olaiya B, 2020, GASTROENTEROL REP, V8, P425, DOI 10.1093/gastro/goaa020
On W, 2022, GASTROINTEST ENDOSC, V95, P432, DOI 10.1016/j.gie.2021.09.040
Pisano M, 2020, WORLD J EMERG SURG, V15, DOI 10.1186/s13017-020-00336-x
Rajadurai A, 2022, J GASTROEN HEPATOL, V37, P179, DOI 10.1111/jgh.15688
Rana SS, 2021, ANN GASTROENTEROL, V34, P142, DOI 10.20524/aog.2020.0568
Rimbas M, 2021, ENDOSC INT OPEN, V09, pE1801, DOI 10.1055/a-1526-1208
Rinninella E, 2015, GASTROINTEST ENDOSC, V82, P1039, DOI 10.1016/j.gie.2015.04.006
Sanchez-Ocana R, 2022, ENDOSCOPY, V54, pE65, DOI 10.1055/a-1368-3985
Sarkaria Savreet, 2013, Gastrointest Endosc Clin N Am, V23, P435, DOI 10.1016/j.giec.2012.12.013
Saumoy M, 2019, J CLIN GASTROENTEROL, V53, P231, DOI 10.1097/MCG.0000000000001036
Sawas T, 2022, ENDOSC ULTRASOUND, V11, P223, DOI 10.4103/EUS-D-21-00031
Shah RJ, 2015, CLIN GASTROENTEROL H, V13, P747, DOI 10.1016/j.cgh.2014.09.047

Sharaiha RZ, 2017, GASTROINTEST ENDOSC, V85, P904, DOI 10.1016/j.gie.2016.12.023
Sharaiha RZ, 2015, GASTROINTEST ENDOSC, V81, pAB425, DOI 10.1016/j.gie.2015.03.1618
Sharaiha RZ, 2016, CLIN GASTROENTEROL H, V14, P1797, DOI 10.1016/j.cgh.2016.05.011
Siddiqui AA, 2016, GASTROINTEST ENDOSC, V83, P699, DOI 10.1016/j.gie.2015.10.020
Sobani ZA, 2021, DIGEST DIS SCI, V66, P2154, DOI 10.1007/s10620-020-06520-y
Song TJ, 2019, GASTROINTEST ENDOSC, V90, P507, DOI 10.1016/j.gie.2019.05.033
Stier Matthew W, 2018, Gastrointest Endosc Clin N Am, V28, P207, DOI [10.1016/j.giec.2017.11.008, 10.1016/j.giec.2017.11.008]
Teoh AY, 2019, ENDOSC INT OPEN, V7, pE964, DOI 10.1055/a-0915-2098
Teoh AYB, 2020, GUT, V69, P1085, DOI 10.1136/gutjnl-2019-319996
Teoh AYB, 2021, DIGEST ENDOSC, V33, P1139, DOI 10.1111/den.13911
Teoh AYB, 2020, DIGEST ENDOSC, V32, P391, DOI 10.1111/den.13494
Teoh AYB, 2017, ENDOSCOPY, V49, P130, DOI 10.1055/s-0042-119036
Yuste RT, 2020, DIGEST ENDOSC, V32, P608, DOI 10.1111/den.13562
Tsuchiya T, 2018, GASTROINTEST ENDOSC, V87, P1138, DOI 10.1016/j.gie.2017.08.017
van der Merwe SW, 2022, ENDOSCOPY, V54, P185, DOI 10.1055/a-1717-1391
Vanella G, 2023, DIGEST ENDOSC, V35, P377, DOI 10.1111/den.14445
Vanella G, 2023, ENDOSC ULTRASOUND, V12, P16, DOI 10.4103/EUS-D-21-00177
Venkatachalapathy SV, 2018, ENDOSC INT OPEN, V6, pE259, DOI 10.1055/s-0043-125362
Walter D, 2016, GUT, V65, P6, DOI 10.1136/gutjnl-2015-309925
Walter D, 2015, ENDOSCOPY, V47, P63, DOI 10.1055/s-0034-1378113
Yang JL, 2019, ENDOSCOPY, V51, P1035, DOI 10.1055/a-0759-1353
Yasuda I, 2021, DIGEST ENDOSC, V33, P335, DOI 10.1111/den.13699
Yoo HW, 2021, ENDOSCOPY, V53, P922, DOI 10.1055/a-1301-1526
Yoo J, 2017, ENDOSC ULTRASOUND, V6, P131, DOI 10.4103/2303-9027.204814
Zhang LY, 2022, ENDOSCOPY, V54, P706, DOI 10.1055/a-1682-7095
NR 111
TC 22
Z9 27
U1 0
U2 0
PU BAISHIDENG PUBLISHING GROUP INC
PI PLEASANTON
PA 7041 Koll Center Parkway, Suite 160, PLEASANTON, CA, UNITED STATES
SN 1007-9327
EI 2219-2840
J9 WORLD J GASTROENTERO
JI World J. Gastroenterol.
PD JUN 7
PY 2023
VL 29
IS 21

BP 3341
EP 3361
DI 10.3748/wjg.v29.i21.3341
PG 21
WC Gastroenterology & Hepatology
WE Science Citation Index Expanded (SCI-EXPANDED)
SC Gastroenterology & Hepatology
GA L0VB4
UT WOS:001020507000010
PM 37377584
OA Green Submitted, hybrid
DA 2026-05-09
ER

PT J
AU Chantarojanasiri, T
Ratanachu-Ek, T
Pausawasdi, N
AF Chantarojanasiri, Tanyaporn
Ratanachu-Ek, Thawee
Pausawasdi, Nonthalee
TI What You Need to Know Before Performing Endoscopic Ultrasound-guided
Hepaticogastrostomy
SO CLINICAL ENDOSCOPY
LA English
DT Review
DE Bile duct obstruction; Biliary fistula; Drainage; Endoscopic
ultrasonography
ID COVERED METAL STENT; LONG-TERM OUTCOMES; BILIARY DRAINAGE;
ELECTROCAUTERY DILATOR; ADVERSE EVENTS; FAILED ERCP; ULTRASONOGRAPHY;
MIGRATION; PATIENT; SAFETY
AB Endoscopic retrograde cholangiopancreatography (ERCP) is the primary treatment modality for bile duct obstruction. When ERCP is unsuccessful, percutaneous transhepatic biliary drainage can be an alternative method. Endoscopic ultrasound-guided biliary drainage (EUS-BD) has emerged as a treatment option for biliary obstruction, especially after ERCP failure. EUS-BD offers transluminal intrahepatic and extrahepatic drainage through a transgastric and transduodenal approach. EUS-guided hepaticogastrostomy (EUS-HGS) is an excellent choice for patients with hilar strictures or those with a surgically altered anatomy. The optimal steps in EUS-HGS are case selection, bile duct visualization, puncture-site selection, wire insertion and manipulation, tract dilation, and stent placement. Caution should be taken at each step to prevent complications. Dedicated devices for EUS-HGS have been developed to improve the technical success rate and reduce complications. This technical review focuses on the essential practical points at each step of EUS-HGS.
C1 [Chantarojanasiri, Tanyaporn] Rajavithi Hosp, Dept Internal Med, Bangkok, Thailand.
[Ratanachu-Ek, Thawee] Rajavithi Hosp, Dept Surg, Bangkok, Thailand.

[Pausawasdi, Nonthalee] Mahidol Univ, Fac Med, Dept Med, Div Gastroenterol,Siriraj Hosp, Bangkok, Thailand.
C3 Rajavithi Hospital; Rajavithi Hospital; Mahidol University
RP Pausawasdi, N (corresponding author), Mahidol Univ, Fac Med, Dept Internal Med, Div Gastroenterol,Siriraj Hosp, Bangkok 10700, Thailand.
EM nonthaleep7@gmail.com
RI Chantarojanasiri, Tanyaporn/AAM-8488-2020
CR Adler DG, 2018, GASTROINTEST ENDOSC, V87, P617, DOI 10.1016/j.gie.2017.08.007
Amano M, 2017, J GASTROEN HEPATOL, V32, P716, DOI 10.1111/jgh.13489
ASGE Technology Committee, 2017, VideoGIE, V2, P35, DOI [10.1016/j.vgie.2016.12.001, 10.1016/j.vgie.2016.12.001]
Burmester E, 2003, GASTROINTEST ENDOSC, V57, P246, DOI 10.1067/mge.2003.85
Chantarojanasiri T, 2015, WORLD J GASTRO ENDOS, V7, P960, DOI 10.4253/wjge.v7.i10.960
Cho DH, 2017, GASTROINTEST ENDOSC, V85, P1067, DOI 10.1016/j.gie.2016.09.010
De Cassan C, 2017, ENDOSC ULTRASOUND, V6, P329, DOI 10.4103/2303-9027.209869
Fujisawa T, 2019, DIGEST ENDOSC, V31, pE74, DOI 10.1111/den.13368
Hamada T, 2013, DIGEST ENDOSC, V25, P340, DOI 10.1111/den.12035
Han SY, 2019, SCI REP-UK, V9, DOI 10.1038/s41598-019-52993-x
Honjo M, 2018, ENDOSC ULTRASOUND, V7, P376, DOI 10.4103/eus.eus_2_18
Isayama H, 2019, J HEPATO-BIL-PAN SCI, V26, P249, DOI 10.1002/jhbp.631
Kamata K, 2017, ARAB J GASTROENTEROL, V18, P120, DOI 10.1016/j.ajg.2017.05.016
Kanno Yoshihide, 2020, VideoGIE, V5, P355, DOI [10.1016/j.vgie.2020.04.003, 10.1016/j.vgie.2020.04.003]
Kanno Y, 2017, WORLD J GASTRO ENDOS, V9, P304, DOI 10.4253/wjge.v9.i7.304
Kawakami H, 2020, J HEPATO-BIL-PAN SCI, V27, pE11, DOI 10.1002/jhbp.676
Kedia P, 2013, CLIN ENDOSC, V46, P543, DOI 10.5946/ce.2013.46.5.543
Khashab MA, 2012, GASTROINTEST ENDOSC, V76, P921, DOI 10.1016/j.gie.2012.05.018
Lakhtakia Sundeep, 2020, VideoGIE, V5, P359, DOI [10.1016/j.vgie.2020.04.023, 10.1016/j.vgie.2020.04.023]
Lee NJ, 2018, DIAGN INTERV IMAG, V99, P717, DOI 10.1016/j.diii.2018.06.006
Maehara K, 2020, ENDOSC INT OPEN, V08, pE1034, DOI 10.1055/a-1169-3749
Martínez B, 2019, ENDOSC INT OPEN, V7, pE1038, DOI 10.1055/a-0918-5931
Martins FP, 2010, ENDOSCOPY, V42, pE126, DOI 10.1055/s-0029-1243911
Minaga K, 2018, DIGEST ENDOSC, V30, P38, DOI 10.1111/den.12910
Miyano A, 2018, J GASTROINTEST SURG, V22, P1312, DOI 10.1007/s11605-018-3758-1
Nakai Y, 2020, GASTROINTEST ENDOSC, V92, P623, DOI 10.1016/j.gie.2020.03.3856
Nakai Y, 2019, CLIN ENDOSC, V52, P212, DOI 10.5946/ce.2018.188
Nakai Y, 2019, CLIN ENDOSC, V52, P220
Nakai Y, 2017, DIGEST ENDOSC, V29, P218, DOI 10.1111/den.12752
Nakai Y, 2016, ENDOSCOPY, V48, P1125, DOI 10.1055/s-0042-116595
Ogura T, 2021, ENDOSCOPY, V53, P369, DOI 10.1055/a-1199-5418
Ogura T, 2021, GUT LIVER, V15, P196
Ogura T, 2019, ENDOSC INT OPEN, V7, pE1652, DOI 10.1055/a-0961-7890
Ogura T, 2019, ENDOSC ULTRASOUND, V8, P398, DOI 10.4103/eus.eus_45_19
Ogura T, 2019, ENDOSC ULTRASOUND, V8, P133, DOI 10.4103/eus.eus_68_18

Ogura T, 2016, WORLD J GASTROENTERO, V22, P3945, DOI 10.3748/wjg.v22.i15.3945
Ogura T, 2015, J GASTROEN HEPATOL, V30, P1748, DOI 10.1111/jgh.13021
Ogura T, 2015, ENDOSCOPY, V47, pE583, DOI 10.1055/s-0034-1393381
Ogura T, 2014, J GASTROEN HEPATOL, V29, P1815, DOI 10.1111/jgh.12598
Oh D, 2017, THER ADV GASTROENTER, V10, P42, DOI 10.1177/1756283X16671671
Okuno N, 2018, INT J GASTROINT INT, V7, P40, DOI 10.18528/gii180006
Okuno N, 2017, INT J GASTROINT INT, V6, P82, DOI 10.18528/gii170008
Okuno N, 2018, J GASTROEN HEPATOL, V33, P1413, DOI 10.1111/jgh.14112
Paik WH, 2019, ENDOSC ULTRASOUND, V8, pS44, DOI 10.4103/eus.eus_51_19
Paik WH, 2014, WORLD J GASTROENTERO, V20, P5051, DOI 10.3748/wjg.v20.i17.5051
Park DH, 2015, J GASTROEN HEPATOL, V30, P1461, DOI 10.1111/jgh.13027
Park DH, 2011, GASTROINTEST ENDOSC, V74, P1276, DOI 10.1016/j.gie.2011.07.054
Park SJ, 2013, GASTROINTEST ENDOSC, V78, P374, DOI 10.1016/j.gie.2013.04.183
Patel V, 2019, ABDOM RADIOL, V44, P1901, DOI 10.1007/s00261-019-01916-5
Prachayakul V, 2013, ENDOSCOPY, V45, pE223, DOI 10.1055/s-0033-1344064
Prachayakul V, 2015, WORLD J GASTROENTERO, V21, P2725, DOI 10.3748/wjg.v21.i9.2725
Prachayakul V, 2013, WORLD J GASTROENTERO, V19, P4758, DOI 10.3748/wjg.v19.i29.4758
Ryou M, 2020, GASTROINTEST ENDOSC, V91, P178, DOI 10.1016/j.gie.2019.07.035
Sato Tatsuya, 2019, VideoGIE, V4, P420, DOI [10.1016/j.vgie.2019.04.017, 10.1016/j.vgie.2019.04.017]
Shima Y, 2014, ENDOSCOPY, V46, pE563, DOI 10.1055/s-0034-1377945
Shiomi H, 2019, DIGEST ENDOSC, V31, pe50, DOI 10.1111/den.13328
Teoh AYB, 2018, GUT, V67, P1209, DOI 10.1136/gutjnl-2017-314341
Tsujino T, 2018, ENDOSC ULTRASOUND, V7, P246, DOI 10.4103/eus.eus_34_18
Tyberg A, 2016, GASTROINTEST ENDOSC, V84, P941, DOI 10.1016/j.gie.2016.05.035
Umeda J, 2015, GASTROINTEST ENDOSC, V82, P390, DOI 10.1016/j.gie.2015.02.041
Wang S, 2016, ENDOSCOPY, V48
Yamamoto Y, 2020, ENDOSC ULTRASOUND, V9, P110, DOI 10.4103/eus.eus_68_19
NR 62
TC 13
Z9 15
U1 0
U2 1
PU KOREAN SOC GASTROINTESTINAL ENDOSCOPY
PI SEOUL
PA 2003 LG PALACE, 165-8 DONGGYO-DONG, MAPO-GU, SEOUL, 121-754, SOUTH KOREA
SN 2234-2400
EI 2234-2443
J9 CLIN ENDOSC
JI Clin. Endosc.
PD MAY
PY 2021
VL 54

IS 3
BP 301
EP 308
DI 10.5946/ce.2021.103
PG 8
WC Gastroenterology & Hepatology
WE Emerging Sources Citation Index (ESCI)
SC Gastroenterology & Hepatology
GA SL9VM
UT WOS:000657262500004
PM 34082486
OA Green Submitted, gold
DA 2026-05-09
ER

PT J
AU Oh, D
Park, DH
Song, TJ
Lee, SS
Seo, DW
Lee, SK
Kim, MH
AF Oh, Dongwook
Park, Do Hyun
Song, Tae Jun
Lee, Sang Soo
Seo, Dong-Wan
Lee, Sung Koo
Kim, Myung-Hwan
TI Long-term outcome of endoscopic ultrasound-guided pancreatic duct
drainage using a fully covered self-expandable metal stent for
pancreaticojejunal anastomosis stricture
SO JOURNAL OF GASTROENTEROLOGY AND HEPATOLOGY
LA English
DT Article
DE endoscopic ultrasound-guided pancreatic duct drainage; fully covered
self-expandable metal stents; long-term outcomes; pancreatic ductal
obstruction; Whipple procedures
ID BILIARY DRAINAGE; ADVERSE EVENTS; FAILED ERCP; CHOLANGIOPANCREATOGRAPHY;
PAIN; PANCREATICOGASTROSTOMY; FEASIBILITY; MULTICENTER; SAFETY
AB Background and Aim Endoscopic ultrasound-guided pancreatic duct drainage (EUS-PD) has been proposed for

pancreatic duct obstruction after failure of endoscopic retrograde pancreatography. We evaluate the long-term outcomes of EUS-PD using a fully covered self-expandable metal stent (FCSEMS) for pancreaticojejunal anastomosis (PJA) strictures following Whipple procedures. Methods Twenty-three patients with PJA strictures underwent EUS-PD according to the findings of EUS-guided pancreatogram and the passage of the guidewire through PJA stricture (complete vs partial stricture) after failure of endoscopic retrograde pancreatography. Technical and clinical success, adverse events (AEs), and long-term outcomes were assessed. Results Technical and clinical success was achieved in all patients. The complete and partial strictures were 11 and 12, respectively. The direct transanastomotic and transmural plastic stenting in partial PJA stricture was successful in only three patients (13%). Therefore, 20 patients underwent EUS-guided transmural FCSEMS placement during the initial attempt. Early AEs, including abdominal pain (n = 3) and peripancreatic fluid collection (n = 1), occurred in four patients (17.4%). During the follow-up periods (median, 27.2 months; interquartile range [IQR], 18.7-40.6), five patients (21.7%) developed late AEs, including asymptomatic stent fracture at the gastric end (n = 3), asymptomatic stent migration (n = 1), and stent occlusion (n = 1). The total duration of stent placement was 27.2 months (IQR, 18.7-40.6), and the median number of stent revision was 2 (IQR, 1-2). Conclusions In terms of safety and efficacy, EUS-PD with an FCSEMS showed favorable success and acceptable AEs rate and durable long-term outcomes.
C1 [Oh, Dongwook; Park, Do Hyun; Song, Tae Jun; Lee, Sang Soo; Seo, Dong-Wan; Lee, Sung Koo; Kim, Myung-Hwan] Univ Ulsan, Asan Med Ctr, Dept Internal Med, Div Gastroenterol,Coll Med, 88 Olymp Ro 43 Gil, Seoul 05505, South Korea.
C3 University of Ulsan; Asan Medical Center
RP Park, D (corresponding author), Univ Ulsan, Asan Med Ctr, Dept Internal Med, Div Gastroenterol,Coll Med, 88 Olymp Ro 43 Gil, Seoul 05505, South Korea.
EM dhpark@amc.seoul.kr
OI Oh, Dongwook/0000-0002-6084-0138
CR Amano H, 1998, HEPATO-GASTROENTEROL, V45, P2382
Blondet JJ, 2007, SURG CLIN N AM, V87, P1477, DOI 10.1016/j.suc.2007.08.014
Chen YI, 2017, GASTROINTEST ENDOSC, V85, P170, DOI 10.1016/j.gie.2016.07.031
Cotton PB, 2010, GASTROINTEST ENDOSC, V71, P446, DOI 10.1016/j.gie.2009.10.027
Demirjian AN, 2010, HPB, V12, P482, DOI 10.1111/j.1477-2574.2010.00214.x
Devière J, 2017, GASTROINTEST ENDOSC, V85, P178, DOI 10.1016/j.gie.2016.08.041
Enestvedt BK, 2016, GASTROINTEST ENDOSC, V83, P1061, DOI 10.1016/j.gie.2016.03.018
Ergun M, 2011, ENDOSCOPY, V43, P518, DOI 10.1055/s-0030-1256333
Fujii-Lau LL, 2015, J HEPATO-BIL-PAN SCI, V22, P51, DOI 10.1002/jhbp.187
Fukukura Y, 2002, RADIOLOGY, V222, P674, DOI 10.1148/radiol.2223010684
Giovannini M, 2013, GASTROINTEST ENDOSC, V78, P865, DOI 10.1016/j.gie.2013.10.019
Itoi T, 2014, J HEPATO-BIL-PAN SCI, V21, pE4, DOI 10.1002/jhbp.43
JENSEN MP, 1986, PAIN, V27, P117, DOI 10.1016/0304-3959(86)90228-9
Kahaleh M, 2007, GASTROINTEST ENDOSC, V65, P224, DOI 10.1016/j.gie.2006.05.008
Kethu SR, 2010, GASTROINTEST ENDOSC, V71, P435, DOI 10.1016/j.gie.2009.07.038
Khashab MA, 2013, GASTROINTEST ENDOSC, V78, P734, DOI 10.1016/j.gie.2013.05.013
Law JK, 2016, GASTROINTEST ENDOSC, V83, P374, DOI 10.1016/j.gie.2015.08.033
Matsunami Y, 2018, ENDOSC INT OPEN, V6, pE505, DOI 10.1055/s-0044-101753
Moreels TG, 2014, WORLD J GASTRO ENDOS, V6, P345, DOI 10.4253/wjge.v6.i8.345
Oh D, 2016, GASTROINTEST ENDOSC, V83, P366, DOI 10.1016/j.gie.2015.07.015

Park DH, 2011, GASTROINTEST ENDOSC, V74, P1276, DOI 10.1016/j.gie.2011.07.054
Park DH, 2008, GASTROINTEST ENDOSC, V68, P1182, DOI 10.1016/j.gie.2008.07.027
Russell RCG, 2003, WORLD J SURG, V27, P1203, DOI 10.1007/s00268-003-7239-6
Tessier G, 2007, GASTROINTEST ENDOSC, V65, P233, DOI 10.1016/j.gie.2006.06.029
Tyberg A, 2017, GASTROINTEST ENDOSC, V85, P164, DOI 10.1016/j.gie.2016.07.030
Vila JJ, 2012, GASTROINTEST ENDOSC, V76, P1133, DOI 10.1016/j.gie.2012.08.001
Widmer Jessica, 2013, Gastrointest Endosc Clin N Am, V23, P847, DOI 10.1016/j.giec.2013.06.011
NR 27
TC 28
Z9 29
U1 0
U2 2
PU WILEY
PI HOBOKEN
PA 111 RIVER ST, HOBOKEN 07030-5774, NJ USA
SN 0815-9319
EI 1440-1746
J9 J GASTROEN HEPATOL
JI J. Gastroenterol. Hepatol.
PD JUN
PY 2020
VL 35
IS 6
BP 994
EP 1001
DI 10.1111/jgh.14897
EA DEC 2019
PG 8
WC Gastroenterology & Hepatology
WE Science Citation Index Expanded (SCI-EXPANDED)
SC Gastroenterology & Hepatology
GA LX5WY
UT WOS:000501700100001
PM 31677201
DA 2026-05-09
ER

PT J
AU Noda, Y
Fujita, N
Kobayashi, G
Ito, K

Obana, T
Horaguchi, J
Koshita, S
Kanno, Y
Sawai, T
AF Noda, Yutaka
Fujita, Naotaka
Kobayashi, Go
Ito, Kei
Obana, Takashi
Horaguchi, Jun
Koshita, Shinsuke
Kanno, Yoshihide
Sawai, Takashi
TI TRANSGASTRIC TRANSHEPATIC ENDOSONOGRAPHY-GUIDED BILIARY DRAINAGE: HISTOLOGICAL ASSESSMENT OF ITS SINUS TRACT IN AN AUTOPSY CASE
SO DIGESTIVE ENDOSCOPY
LA English
DT Article
DE biliary drainage; endoscopic ultrasound; endoscopic ultrasound-guided fine-needle aspiration (EUS-FNA); endosonography; malignant biliary obstruction
ID OBSTRUCTION
AB Endosonography-guided biliary drainage (ESBD) is gaining attention as a promising drainage technique for obstructive jaundice. However, histological changes resulting from ESBD have not been well understood. We had an opportunity to histologically investigate the influence of ESBD, established between the left hepatic duct and the stomach, on the relevant organs in an autopsy case with bile duct cancer extending from the pancreatic head to the hepatic hilum with duodenal invasion. Localized fibrous connective tissues were present around and along the sinus tract, including the connection between the surfaces of the left lobe of the liver and the gastric serosa, without hemorrhage, inflammatory changes, or cancer invasion. The inside of the ESBD stent was slightly stenotic at the intramural portion of the stomach due to proliferation of granulation tissue. No bile stasis or abscess was observed in the left lobe. These results are quite suggestive of the high safety and efficacy of ESBD with adequate performance.
C1 [Noda, Yutaka; Fujita, Naotaka; Kobayashi, Go; Ito, Kei; Obana, Takashi; Horaguchi, Jun; Koshita, Shinsuke; Kanno, Yoshihide] Sendai City Med Ctr, Dept Gastroenterol, Sendai, Miyagi 9830824, Japan.
[Sawai, Takashi] Iwate Med Univ, Dept Pathol 1, Morioka, Iwate 020, Japan.
C3 Iwate Medical University
RP Noda, Y (corresponding author), Sendai City Med Ctr, Dept Gastroenterol, Sendai, Miyagi 9830824, Japan.
EM noda@openhp.or.jp
OI Kanno, Yoshihide/0000-0002-2044-5483
CR Bories E, 2007, ENDOSCOPY, V39, P287, DOI 10.1055/s-2007-966212
Fujita N, 2008, DIGEST ENDOSC, V20, P55, DOI 10.1111/j.1443-1661.2008.00782.x
Fujita N, 2007, WORLD J GASTROENTERO, V13, P5512, DOI 10.3748/wjg.v13.i41.5512

Giovannini M, 2003, ENDOSCOPY, V35, P1076, DOI 10.1055/s-2003-44596
Giovannini M, 2001, ENDOSCOPY, V33, P898, DOI 10.1055/s-2001-17324
HORAGUCHI J, 2008, GASTROENTEROL ENDOSC, V50, P1332
Kahaleh M, 2006, GASTROINTEST ENDOSC, V64, P52, DOI 10.1016/j.gie.2006.01.063
Püspök A, 2005, AM J GASTROENTEROL, V100, P1743, DOI 10.1111/j.1572-0241.2005.41806.x
TAKASAWA O, 2008, J JPN BILIARY ASS, V22, P536
Will U, 2007, ENDOSCOPY, V39, P292, DOI 10.1055/s-2007-966215
NR 10
TC 2
Z9 2
U1 0
U2 1
PU WILEY-BLACKWELL
PI MALDEN
PA COMMERCE PLACE, 350 MAIN ST, MALDEN 02148, MA USA
SN 0915-5635
J9 DIGEST ENDOSC
JI Dig. Endosc.
PD OCT
PY 2010
VL 22
IS 4
BP 341
EP 344
DI 10.1111/j.1443-1661.2010.01027.x
PG 4
WC Gastroenterology & Hepatology; Surgery
WE Science Citation Index Expanded (SCI-EXPANDED)
SC Gastroenterology & Hepatology; Surgery
GA 656IN
UT WOS:000282323100018
PM 21175493
DA 2026-05-09
ER

PT J
AU Sato, R
Matsumoto, K
Matsumi, A
Morimoto, K
Terasawa, H
Fujii, Y

Yamazaki, T
Tsutsumi, K
Horiguchi, S
Kato, H
AF Sato, Ryosuke
Matsumoto, Kazuyuki
Matsumi, Akihiro
Morimoto, Kosaku
Terasawa, Hiroyuki
Fujii, Yuki
Yamazaki, Tatsuhiro
Tsutsumi, Koichiro
Horiguchi, Shigeru
Kato, Hironari
TI A case of endoscopic retrograde cholangiopancreatography-related main
pancreatic duct perforation salvaged by endoscopic
ultrasonography-guided pancreatic duct drainage
SO CLINICAL JOURNAL OF GASTROENTEROLOGY
LA English
DT Article
DE EUS-guided pancreatic duct drainage; Pancreatic duct perforation;
ERCP-related perforation
ID MANAGEMENT
AB We herein report a 78-year-old man who underwent endoscopic retrograde cholangiopancreatography (ERCP) to examine main pancreatic duct (MPD) stenosis. During ERCP, MPD perforation occurred due to the cytology brush maneuver. Endoscopic pancreatic stenting to bridge the perforated site failed because the MPD was bent and formed a loop. Thus, we placed the stent at the proximal perforated side. The patient developed retroperitoneal perforation and pancreatic fistula with infection, showing a worsening condition. Pancreatic duct drainage was not effective, so we performed endoscopic ultrasonography-guided pancreatic duct drainage. Subsequently, he gradually improved and was discharged 3 months after initial ERCP.
C1 [Sato, Ryosuke; Matsumoto, Kazuyuki; Matsumi, Akihiro; Morimoto, Kosaku; Terasawa, Hiroyuki; Fujii, Yuki; Yamazaki, Tatsuhiro; Tsutsumi, Koichiro; Horiguchi, Shigeru; Kato, Hironari] Okayama Univ Hosp, Dept Gastroenterol & Hepatol, Kita Ku, 2-5-1 Shikata Cho, Okayama, Okayama 7008558, Japan.
C3 Okayama University
RP Matsumoto, K (corresponding author), Okayama Univ Hosp, Dept Gastroenterol & Hepatol, Kita Ku, 2-5-1 Shikata Cho, Okayama, Okayama 7008558, Japan.
EM matsumoto.k@okayama-u.ac.jp
RI ; Tsutsumi, Koichiro/MGU-6876-2025
OI Matsumoto, Kazuyuki/0000-0002-5102-7452; Sato,
Ryosuke/0000-0002-5368-6347;
CR Bill JG, 2018, SURG ENDOSC, V32, P4841, DOI 10.1007/s00464-018-6235-8
Dalal A, 2020, DIGEST ENDOSC, V32, P409, DOI 10.1111/den.13504

Dumonceau JM, 2020, ENDOSCOPY, V52, P127, DOI 10.1055/a-1075-4080
Enns R, 2002, ENDOSCOPY, V34, P293, DOI 10.1055/s-2002-23650
Fatima J, 2007, ARCH SURG-CHICAGO, V142, P448, DOI 10.1001/archsurg.142.5.448
Fujita N, 2007, WORLD J GASTROENTERO, V13, P5512, DOI 10.3748/wjg.v13.i41.5512
Glomsaker T, 2013, BRIT J SURG, V100, P373, DOI 10.1002/bjs.8992
Howard TJ, 1999, SURGERY, V126, P658, DOI 10.1016/S0039-6060(99)70119-4
Inoue T, 2022, J GASTROEN HEPATOL, V37, P780, DOI 10.1111/jgh.15724
Ito Y, 2012, WORLD J EMERG SURG, V7, DOI 10.1186/1749-7922-7-21
Kim HS, 2001, GASTROINTEST ENDOSC, V54, P49, DOI 10.1067/mge.2001.115733
Kwon W, 2012, J KOREAN SURG SOC, V83, P218, DOI 10.4174/jkss.2012.83.4.218
Miller R, 2013, AM J SURG, V206, P180, DOI 10.1016/j.amjsurg.2012.07.050
Polydorou A, 2011, J GASTROINTEST SURG, V15, P2211, DOI 10.1007/s11605-011-1723-3
Ponchon T, 2000, ENDOSCOPY, V32, P200, DOI 10.1055/s-2000-95
Reddymasu S, 2011, J PANCREAS, V12, P489
Shimizu T., 2016, NIHON FUKUBU KYUKYU, V36, P79
Sonoda Y, 2003, GASTROINTEST ENDOSC, V58, P305, DOI 10.1067/mge.2003.350
Stapfer M, 2000, ANN SURG, V232, P191, DOI 10.1097/00000658-200008000-00007
NR 19
TC 1
Z9 1
U1 1
U2 2
PU SPRINGER JAPAN KK
PI TOKYO
PA SHIROYAMA TRUST TOWER 5F, 4-3-1 TORANOMON, MINATO-KU, TOKYO, 105-6005,
JAPAN
SN 1865-7257
EI 1865-7265
J9 CLIN J GASTROENTEROL
JI Clin. J. Gastroenterol.
PD DEC
PY 2022
VL 15
IS 6
BP 1179
EP 1184
DI 10.1007/s12328-022-01699-1
EA SEP 2022
PG 6
WC Gastroenterology & Hepatology
WE Emerging Sources Citation Index (ESCI)
SC Gastroenterology & Hepatology

GA 6M3EH
UT WOS:000852906700001
PM 36094734
DA 2026-05-09
ER

PT J
AU Fukuda, S
Hijioka, S
Nagashio, Y
Maruki, Y
Ohba, A
Agarie, D
Hagiwara, Y
Hara, H
Okamoto, K
Yamashige, D
Yagi, S
Kuwada, M
Chatto, M
Kondo, S
Morizane, C
Ueno, H
Saito, Y
Okusaka, T
AF Fukuda, Soma
Hijioka, Susumu
Nagashio, Yoshikuni
Maruki, Yuta
Ohba, Akihiro
Agarie, Daiki
Hagiwara, Yuya
Hara, Hidenobu
Okamoto, Kohei
Yamashige, Daiki
Yagi, Shin
Kuwada, Masaru
Chatto, Mark
Kondo, Shunsuke
Morizane, Chigusa
Ueno, Hideki
Saito, Yutaka

Okusaka, Takuji
TI Feasibility and safety of a novel plastic stent designed specifically
for endoscopic ultrasound-guided pancreatic duct drainage
SO ENDOSCOPY INTERNATIONAL OPEN
LA English
DT Article
DE Pancreatobiliary (ERCP/PTCD); Strictures; Endoscopic ultrasonography;
Pancreas; Intervention EUS
ID FAILED ERCP; OUTCOMES; MULTICENTER
AB Background and study aims Endoscopic ultrasound-guided pancreatic duct drainage (EUS-PD) is emerging as an effective alternative treatment for obstructive pancreatitis after unsuccessful endoscopic retrograde pancreatography (ERP). However, the high incidence of adverse events associated with EUS-PD (approximately 20%) remains an issue. Recently, we developed a novel plastic stent for EUS-PD, with a radiopaque marker positioned at approximately one-third of the length from the distal end of the stent and side holes positioned exclusively distal to the marker. This study aimed to evaluate the feasibility and safety of using this stent in EUS-PD. Patients and methods We retrospectively reviewed data from 10 patients who underwent EUS-PD with the novel plastic stent at the National Cancer Center Hospital between March 2021 and October 2023. Technical and clinical success, procedure times, adverse events (AEs), recurrent pancreatic duct obstruction (RPO), and time to RPO were assessed. Results Of the 10 patients, five had postoperative benign pancreaticojejunal anastomotic strictures and five had malignant pancreatic duct obstruction. The technical and clinical success rates were both 100% (10/10). An AE (self-limited abdominal pain) occurred in one patient (10.0%). Two patients (20.0%) died of their primary disease during the follow-up period (median, 44 days; range, 25-272 days). The incidence of RPO was 10.0% (1/10), and the 3-month non-RPO rate was 83.3%. Conclusions The novel plastic stent shows potential as a useful and safe tool in EUS-PD.
C1 [Fukuda, Soma; Hijioka, Susumu; Nagashio, Yoshikuni; Maruki, Yuta; Ohba, Akihiro; Agarie, Daiki; Hagiwara, Yuya; Hara, Hidenobu; Okamoto, Kohei; Yamashige, Daiki; Yagi, Shin; Kuwada, Masaru; Chatto, Mark; Kondo, Shunsuke; Morizane, Chigusa; Ueno, Hideki; Okusaka, Takuji] Natl Canc Ctr, Dept Hepatobiliary & Pancreat Oncol, 5-1-1 Tsukiji Chuo Ku, Tokyo 1040045, Japan.
[Chatto, Mark] Makati Med Ctr, Dept Med, Makati, Philippines.
[Saito, Yutaka] Natl Canc Ctr, Endoscopy Div, Tokyo, Japan.
C3 National Cancer Center - Japan; Makati Medical Center; National Cancer
Center - Japan
RP Hijioka, S (corresponding author), Natl Canc Ctr, Dept Hepatobiliary & Pancreat Oncol, 5-1-1 Tsukiji Chuo Ku, Tokyo 1040045, Japan.
EM shijioka@ncc.go.jp
RI ; Yamashige, Daiki/ONJ-2124-2025; takuji, okusaka/ADI-3231-2022; Kondo,
Shunsuke/AAO-4777-2021; Maruki, Yuta/LKJ-3855-2024; Hijioka,
Susumu/I-1039-2019; yuya, hagiwara/PTE-5006-2026
OI Fukuda, Soma/0000-0003-2907-7776; Yamashige, Daiki/0000-0002-6152-8920;
Hagiwara, Yuya/0009-0009-3856-0795; Chatto, Mark
Paolo/0000-0001-5512-9336; Hara, Hidenobu/0009-0001-4331-6799; Maruki,
Yuta/0000-0002-5045-8082; Hijioka, Susumu/0000-0002-0394-6876;
CR Bhurwal A, 2021, PANCREATOLOGY, V21, P990, DOI 10.1016/j.pan.2021.03.021

Bor R, 2015, WORLD J GASTRO ENDOS, V7, P1023, DOI 10.4253/wjge.v7.i11.1023
Chandan S, 2020, ENDOSC INT OPEN, V08, pE1664, DOI 10.1055/a-1236-3350
Chen YI, 2017, GASTROINTEST ENDOSC, V85, P170, DOI 10.1016/j.gie.2016.07.031
Cotton PB, 2010, GASTROINTEST ENDOSC, V71, P446, DOI 10.1016/j.gie.2009.10.027
Dalal A, 2020, DIGEST ENDOSC, V32, P409, DOI 10.1111/den.13504
Ergun M, 2011, ENDOSCOPY, V43, P518, DOI 10.1055/s-0030-1256333
François E, 2002, GASTROINTEST ENDOSC, V56, P128, DOI 10.1067/mge.2002.125547
Fujii LL, 2013, GASTROINTEST ENDOSC, V78, P854, DOI 10.1016/j.gie.2013.05.016
Hijioka S, 2023, ENDOSCOPY, V55, pE24, DOI 10.1055/a-1930-6319
Isayama H, 2015, DIGEST ENDOSC, V27, P259, DOI 10.1111/den.12379
Kahaleh M, 2007, GASTROINTEST ENDOSC, V65, P224, DOI 10.1016/j.gie.2006.05.008
Kurihara T, 2013, DIGEST ENDOSC, V25, P109, DOI 10.1111/den.12100
Matsunami Y, 2018, ENDOSC INT OPEN, V6, pE505, DOI 10.1055/s-0044-101753
Oh D, 2016, GASTROINTEST ENDOSC, V83, P366, DOI 10.1016/j.gie.2015.07.015
Sakai T, 2022, PANCREATOLOGY, V22, P58, DOI 10.1016/j.pan.2021.10.006
Sanders DJ, 2021, WORLD J GASTRO ENDOS, V13, P260, DOI 10.4253/wjge.v13.i8.260
Tessier G, 2007, GASTROINTEST ENDOSC, V65, P233, DOI 10.1016/j.gie.2006.06.029
Tyberg A, 2017, GASTROINTEST ENDOSC, V85, P164, DOI 10.1016/j.gie.2016.07.030
Uchida D, 2018, CAN J GASTROENTEROL, V2018, DOI 10.1155/2018/8216109
NR 20
TC 3
Z9 3
U1 0
U2 0
PU GEORG THIEME VERLAG KG
PI STUTTGART
PA RUDIGERSTR 14, D-70469 STUTTGART, GERMANY
SN 2364-3722
EI 2196-9736
J9 ENDOSC INT OPEN
JI Endosc. Int. Open
PD JUN
PY 2024
VL 12
IS 06
BP E715
EP E722
DI 10.1055/a-2294-8517
PG 8
WC Gastroenterology & Hepatology; Surgery
WE Emerging Sources Citation Index (ESCI)
SC Gastroenterology & Hepatology; Surgery

GA WI8C4
UT WOS:001254323100001
PM 38841434
OA Green Submitted, gold
DA 2026-05-09
ER

PT J
AU Waldthaler, A
Rutkowski, W
Valente, R
Arnelo, U
Löhr, JM
AF Waldthaler, Alexander
Rutkowski, Wiktor
Valente, Roberto
Arnelo, Urban
Lohr, J. -Matthias
TI Palliative therapy in pancreatic cancer-interventional treatment with
stents
SO TRANSLATIONAL GASTROENTEROLOGY AND HEPATOLOGY
LA English
DT Review
DE Pancreatic cancer; endosonography (EUS); endoscopic retrograde
cholangiopancreatography (ERCP); self-expanding metal stents (SEMS);
Lumen apposing metal stent (LAMS)
ID GUIDED BILIARY DRAINAGE; ENDOSCOPIC RETROGRADE CHOLANGIOPANCREATOGRAPHY;
EXPANDABLE METAL STENTS; BILE-DUCT OBSTRUCTION; SURGICAL
GASTROJEJUNOSTOMY; HEPATICOGASTROSTOMY; CHOLECYSTITIS; MANAGEMENT;
PLACEMENT; GASTROENTEROSTOMY
AB Interventional treatment with stents in pancreatic cancer is a topic that developed during recent years and new fields of palliative stent therapy have evolved. The increasing life expectancy of patients with unresectable pancreatic cancer increases the need for clinical and cost effective therapeutic interventions. Current literature, guidelines, practice and evidence were reviewed. Besides the most obvious biliary stenting via endoscopic retrograde cholangiopancreatography (ERCP), pancreatic and gastroduodenal stenting as well as percutaneous transhepatic cholangiography (PTC) and the rapidly growing field of endosonographic stent implantation in the palliative care of patients with pancreatic cancer are being discussed from several points of view in this review.
C1 Karolinska Univ Hosp, Dept Upper Abdominal Dis, Stockholm, Sweden.
Karolinska Univ Hosp, Clin Sci Intervent & Technol CLINTEC, Stockholm, Sweden.
C3 Karolinska Institutet; Karolinska University Hospital; Karolinska
Institutet; Karolinska University Hospital
RP Waldthaler, A (corresponding author), Karolinska Univ Hosp, Halsovagen 11, S-14186 Stockholm, Sweden.

EM Alexander.Waldthaler@sll.se
RI Valente, Roberto/AAO-2912-2020; Löhr, Matthias/A-3692-2012
OI Valente, Roberto/0000-0002-6785-2326; Löhr, Matthias/0000-0002-7647-198X
CR Almadi MA, 2013, CLIN GASTROENTEROL H, V11, P27, DOI 10.1016/j.cgh.2012.10.019
Ardengh JC, 2018, WORLD J GASTRO ENDOS, V10, P99, DOI 10.4253/wjge.v10.i5.99
Bang JY, 2018, GASTROINTEST ENDOSC, V88, P9, DOI 10.1016/j.gie.2018.03.012
Baniya R, 2017, CLIN EXP GASTROENTER, V10, P67, DOI 10.2147/CEG.S132004
Bulajic M, 2012, HEPATOB PANCREAT DIS, V11, P209, DOI 10.1016/S1499-3872(12)60150-7
Burmester E, 2003, GASTROINTEST ENDOSC, V57, P246, DOI 10.1067/mge.2003.85
Cho DH, 2017, GASTROINTEST ENDOSC, V85, P1067, DOI 10.1016/j.gie.2016.09.010
Chopita N, 2005, ENDOSCOPY, V37, P313, DOI 10.1055/s-2005-861358
Costamagna G, 2004, DIGEST LIVER DIS, V36, P635, DOI 10.1016/j.dld.2004.05.001
Costamagna G, 2011, ENDOSCOPY, V43, P317, DOI 10.1055/s-0030-1256294
COSTAMAGNA G, 1993, GASTROINTEST ENDOSC, V39, P774, DOI 10.1016/S0016-5107(93)70263-1
Timea,, 2016, ORVOSI HETILAP, V157, P268, DOI 10.1556/650.2016.30365
Dawod E, 2018, CLIN ENDOSC, V51, P156, DOI 10.5946/ce.2017.085
De Cassan C, 2017, ENDOSC ULTRASOUND, V6, P329, DOI 10.4103/2303-9027.209869
Dhir V, 2012, GASTROINTEST ENDOSC, V75, P354, DOI 10.1016/j.gie.2011.07.075
Dumonceau JM, 2018, ENDOSCOPY, V50, P910, DOI 10.1055/a-0659-9864
Eloubeidi MA, 2016, GASTROINTEST ENDOSC, V83, P17, DOI 10.1016/j.gie.2015.09.009
Enochsson L, 2010, GASTROINTEST ENDOSC, V72, P1175, DOI 10.1016/j.gie.2010.07.047
Fritscher-Ravens A, 2002, GASTROINTEST ENDOSC, V56, P737, DOI 10.1067/mge.2002.129084
Fujii-Lau LL, 2015, J HEPATO-BIL-PAN SCI, V22, P51, DOI 10.1002/jhbp.187
Giovannini M, 2003, ENDOSCOPY, V35, P1076, DOI 10.1055/s-2003-44596
Giovannini M, 2001, ENDOSCOPY, V33, P898, DOI 10.1055/s-2001-17324
González N, 2013, ENDOSCOPY, V45, pE254, DOI 10.1055/s-0033-1344414
Holt BA, 2016, GASTROINTEST ENDOSC, V83, P160, DOI 10.1016/j.gie.2015.06.019
Jang S, 2018, GASTROINTEST ENDOSC, V87, P1061, DOI 10.1016/j.gie.2017.08.024
Kaassis M, 2003, GASTROINTEST ENDOSC, V57, P178, DOI 10.1067/mge.2003.66
Kamata K, 2017, ONCOLOGY-BASEL, V93, P87, DOI 10.1159/000481238
Khan MA, 2016, DIGEST DIS SCI, V61, P684, DOI 10.1007/s10620-015-3933-0
Khashab MA, 2017, ENDOSC INT OPEN, V5, pE275, DOI 10.1055/s-0043-101695
Lamarca A, 2016, WORLD J GASTROENTERO, V22, P6065, DOI 10.3748/wjg.v22.i26.6065
Li JJ, 2016, PLOS ONE, V11, DOI 10.1371/journal.pone.0149066
Linder S, 2006, J GASTROINTEST SURG, V10, P672, DOI 10.1016/j.gassur.2005.10.005
Löhr JM, 2017, UNITED EUR GASTROENT, V5, P153, DOI 10.1177/2050640616684695
Martinez JM, 2017, J GASTROINTEST CANC, V48, P58, DOI 10.1007/s12029-016-9907-4
Noon WJ, 2006, GASTROINTEST ENDOSC, V63, P996, DOI 10.1016/j.gie.2005.11.054
Ogura T, 2016, WORLD J GASTROENTERO, V22, P3945, DOI 10.3748/wjg.v22.i15.3945
Ogura T, 2016, ENDOSCOPY, V48, P156, DOI 10.1055/s-0034-1392859
Oh D, 2017, THER ADV GASTROENTER, V10, P42, DOI 10.1177/1756283X16671671
Paik WH, 2018, AM J GASTROENTEROL, V113, P987, DOI 10.1038/s41395-018-0122-8

Park JK, 2018, GASTROINTEST ENDOSC, V88, P277, DOI 10.1016/j.gie.2018.03.015
Perez-Miranda M, 2017, J CLIN GASTROENTEROL, V51, P896, DOI 10.1097/MCG.0000000000000887
Poincloux L, 2015, ENDOSCOPY, V47, P794, DOI [10.1055/s-0035-1545399, 10.1055/s-0034-1391988]
Rimbas M, 2017, ENDOSC ULTRASOUND, V6, P235, DOI 10.4103/eus.eus_47_17
Saleem A, 2011, GASTROINTEST ENDOSC, V74, P321, DOI 10.1016/j.gie.2011.03.1249
Shah SL, 2018, J CLIN GASTROENTEROL, V52, P765, DOI 10.1097/MCG.0000000000001104
Shimizu S, 2013, J GASTROEN HEPATOL, V28, P68, DOI 10.1111/j.1440-1746.2012.07283.x
SOEHENDRA N, 1979, DEUT MED WOCHENSCHR, V104, P206, DOI 10.1055/s-0028-1103870
Sogabe Y, 2018, DIGEST ENDOSC, V30, P380, DOI 10.1111/den.12991
SPEER AG, 1987, LANCET, V2, P57, DOI 10.1016/S0140-6736(87)92733-4
Staub J, 2018, GASTROINTEST ENDOSC, V87, P1499, DOI 10.1016/j.gie.2018.01.040
Stier Matthew W, 2018, Gastrointest Endosc Clin N Am, V28, P207, DOI [10.1016/j.giec.2017.11.008, 10.1016/j.giec.2017.11.008]
Tham TCK, 2000, AM J GASTROENTEROL, V95, P956
Tonozuka R, 2016, GASTROINTEST ENDOSC, V84, P206, DOI 10.1016/j.gie.2016.03.020
Tsauo J, 2016, ABDOM RADIOL, V41, P2233, DOI 10.1007/s00261-016-0810-z
Tsuchiya T, 2011, DIGEST DIS SCI, V56, P1889, DOI 10.1007/s10620-011-1581-6
Uemura S, 2018, PANCREATOLOGY
Vienne A, 2010, GASTROINTEST ENDOSC, V72, P728, DOI 10.1016/j.gie.2010.06.040
Walter D, 2015, GASTROENTEROLOGY, V149, P130, DOI 10.1053/j.gastro.2015.03.012
Wang AY, 2015, GASTROINTEST ENDOSC, V81, P367, DOI 10.1016/j.gie.2014.11.027
Wang KX, 2016, GASTROINTEST ENDOSC, V83, P1218, DOI 10.1016/j.gie.2015.10.033
Wehrmann T, 2005, EUR J GASTROEN HEPAT, V17, P1395, DOI 10.1097/00042737-200512000-00020
Yamao K, 2018, GASTROINTEST ENDOSC, V88, P66, DOI 10.1016/j.gie.2018.01.021
Yang MJ, 2015, SCAND J GASTROENTERO, V50, P1490, DOI 10.3109/00365521.2015.1057219
Yoon WJ, 2009, GASTROINTEST ENDOSC, V70, P284, DOI 10.1016/j.gie.2008.12.241
Yoshida Y, 2017, PANCREATOLOGY, V17, P983, DOI 10.1016/j.pan.2017.09.011
NR 65
TC 5
Z9 5
U1 0
U2 3
PU AME PUBLISHING COMPANY
PI SHATIN
PA FLAT-RM C 16F, KINGS WING PLAZA 1, NO 3 KWAN ST, SHATIN, HONG KONG
00000, PEOPLES R CHINA
EI 2415-1289
J9 TRANSL GASTROENT HEP
JI Transl. Gastroenterol. Hepatol.
PD JAN
PY 2019
VL 4

AR 7
DI 10.21037/tgh.2019.01.05
PG 9
WC Gastroenterology & Hepatology
WE Emerging Sources Citation Index (ESCI)
SC Gastroenterology & Hepatology
GA HM8YN
UT WOS:000459769400007
PM 30854494
OA Green Submitted, gold
DA 2026-05-09
ER

PT J
AU Chen, YI
Sahai, A
Donatelli, G
Lam, E
Forbes, N
Mosko, J
Paquin, SC
Donnellan, F
Chatterjee, A
Telford, J
Miller, C
Desilets, E
Sandha, G
Kenshil, S
Mohamed, R
May, G
Gan, I
Barkun, J
Calo, N
Nawawi, A
Friedman, G
Cohen, A
Maniere, T
Chaudhury, P
Metrakos, P
Zogopoulos, G
Bessissow, A
Abou Khalil, J

Baffis, V

Waschke, K

Parent, J

Soulellis, C

Khashab, M

Kunda, R

Geraci, O

Martel, M

Schwartzman, K

Fiore, JFF

Rahme, E

Barkun, A

AF Chen, Yen-, I

Sahai, Anand

Donatelli, Gianfranco

Lam, Eric

Forbes, Nauzer

Mosko, Jeffrey

Paquin, Sarto C.

Donnellan, Fergal

Chatterjee, Avijit

Telford, Jennifer

Miller, Corey

Desilets, Etienne

Sandha, Gurpal

Kenshil, Sana

Mohamed, Rachid

May, Gary

Gan, Ian

Barkun, Jeffrey

Calo, Natalia

Nawawi, Abrar

Friedman, Gad

Cohen, Albert

Maniere, Thibaut

Chaudhury, Prosanto

Metrakos, Peter

Zogopoulos, George

Bessissow, Ali

Abou Khalil, Jad

Baffis, Vicky

Waschke, Kevin

Parent, Josee
Soulellis, Constantine
Khashab, Mouen
Kunda, Rastislav
Geraci, Olivia
Martel, Myriam
Schwartzman, Kevin
Fiore Jr, Julio F.
Rahme, Elham
Barkun, Alan
TI Endoscopic Ultrasound-Guided Biliary Drainage of First Intent With a Lumen-Apposing Metal Stent vs Endoscopic Retrograde Cholangiopancreatography in Malignant Distal Biliary Obstruction: A Multicenter Randomized Controlled Study (ELEMENT Trial)
SO GASTROENTEROLOGY
LA English
DT Article
DE Endoscopic Ultrasonography; Stent; ERCP; Pancre-atic Cancer; Biliary Obstruction
ID PANCREATIC ADENOCARCINOMA; COST-EFFECTIVENESS; CANNULATION; ERCP; GEMCITABINE; PALLIATION
AB Background & aims: Endoscopic ultrasound-guided choledochoduodenostomy with a lumen-apposing metal stent (EUS-CDS) is a promising modality for management of malignant distal biliary obstruction (MDBO) with potential for better stent patency. We compared its outcomes with endoscopic retrograde cholangiopancreatography with metal stenting (ERCP-M).Methods: In this multicenter randomized controlled trial, we recruited patients with MDBO secondary to borderline resectable, locally advanced, or unresectable peri-ampullary cancers across 10 Canadian institutions and 1 French institution. This was a superiority trial with a noninferiority assessment of technical success. Patients were randomized to EUS-CDS or ERCP-M. The primary end point was the rate of stent dysfunction at 1 year, considering competing risks of death, clinical failure, and surgical resection. Analyses were performed according to intention-to-treat principles.Results: From February 2019 to February 2022, 144 patients were recruited; 73 were randomized to EUS-CDS and 71 were randomized to ERCP-M. The mean (SD) procedure time was 14.0 (11.4) minutes for EUS-CDS and 23.1 (15.6) minutes for ERCP-M ($P < .01$); 40% of the former was performed without fluoroscopy. Technical success was achieved in 90.4% (95% CI, 81.5% to 95.3%) of EUS-CDS and 83.1% (95% CI, 72.7% to 90.1%) of ERCP-M with a risk difference of 7.3% (95% CI, -4.0% to 18.8%) indicating noninferiority. Stent dysfunction occurred in 9.6% vs 9.9% of EUS-CDS and ERCP-M cases, respectively ($P = .96$). No differences in adverse events, pancreaticoduodenectomy and oncologic outcomes, or quality of life were noted.Conclusions: Although not superior in stent function, EUS-CDS is an efficient and safe alternative to ERCP-M in patients with MDBO. These findings provide evidence for greater adoption of EUS-CDS in clinical practice as a complementary and exchangeable first-line modality to ERCP in patients with MDBO.
C1 [Chen, Yen-, I; Baffis, Vicky; Waschke, Kevin; Parent, Josee; Soulellis, Constantine; Barkun, Alan] McGill Univ, Hlth Ctr, Div Gastroenterol & Hepatol, 1001 Decarie Blvd, Montreal, PQ H4A 3J1, Canada.
[Sahai, Anand; Paquin, Sarto C.] Ctr Hosp Univ Montreal, Serv Gastroenterol, Montreal, PQ, Canada.
[Donatelli, Gianfranco] Hop Prive Peupliers, Unite Endoscopie Intervent, Radiotherapy Dept, Paris, France.

[Lam, Eric; Telford, Jennifer] St Paul Hosp, Div Gastroenterol & Hepatol, Vancouver, BC, Canada.
[Forbes, Nauzer; Mohamed, Rachid] Univ Calgary, Div Gastroenterol & Hepatol, Calgary, AB, Canada.
[Mosko, Jeffrey; May, Gary] Univ Toronto, St Michaels Hosp, Div Gastroenterol, Toronto, ON, Canada.
[Donnellan, Fergal; Calo, Natalia] Vancouver Gen Hosp, Div Gastroenterol & Hepatol, Vancouver, BC, Canada.
[Chatterjee, Avijit; Abou Khalil, Jad] Ottawa Hosp, Div Gastroenterol & Hepatol, Ottawa, ON, Canada.
[Miller, Corey; Friedman, Gad; Cohen, Albert] Jewish Gen Hosp, Div Gastroenterol, Montreal, PQ, Canada.
[Desilets, Etienne; Maniere, Thibaut] Hop Charles Le Moyne, Div Gastroenterol, Longeuil, PQ, Canada.
[Sandha, Gurpal] Univ Alberta Hosp, Div Gastroenterol & Hepatol, Edmonton, AB, Canada.
[Barkun, Jeffrey; Nawawi, Abrar; Chaudhury, Prosanto; Metrakos, Peter; Zogopoulos, George; Fiore Jr, Julio F.] McGill Univ, Hlth Ctr, Dept Surg, Montreal, PQ, Canada.
[Bessissow, Ali] McGill Univ, Hlth Ctr, Dept Radiol, Montreal, PQ, Canada.
[Khashab, Mouen] Johns Hopkins Univ Hosp, Div Gastroenterol & Hepatol, Baltimore, MD USA.
[Kunda, Rastislav] Vrije Univ Brussel, Univ Ziekenhuis Brussel, Dept Surg, Dept Gastroenterol Hepatol, Brussels, Belgium.
[Geraci, Olivia; Martel, Myriam] McGill Univ, Res Inst, Hlth Ctr, Montreal, PQ, Canada.
[Schwartzman, Kevin] McGill Univ, Hlth Ctr, Resp Div, Montreal, PQ, Canada.
[Barkun, Alan] McGill Univ, Dept Med, Div Clin Epidemiol, Montreal, PQ, Canada.
C3 McGill University; Universite de Montreal; University of Calgary; University of Toronto; Saint Michaels Hospital Toronto; University of British Columbia; University of Ottawa; Ottawa Hospital Research Institute; Jewish General Hospital - Montreal; University of Alberta; McGill University; McGill University; Johns Hopkins University; Johns Hopkins Medicine; Vrije Universiteit Brussel; University Hospital Brussels; McGill University; McGill University; McGill University
RP Chen, YI (corresponding author), McGill Univ, Hlth Ctr, Div Gastroenterol & Hepatol, 1001 Decarie Blvd, Montreal, PQ H4A 3J1, Canada.
EM cyen33@gmail.com
RI ; khashab, mouen/F-6643-2017; Kunda, Rastislav/N-1775-2019; metrakos, peter/D-3726-2013; Martel, Myriam/ABB-3862-2020; Barkun, Alan N/JDV-6782-2023; Sandha, Gurpal/JAX-7044-2023
OI Telford, Jennifer/0000-0002-6884-6147; Mosko, Jeffrey/0000-0003-2983-524X; Donatelli, Gianfranco/0000-0003-0645-1710;
FU Canadian Institute of Health Research; Fonds de Recherche Sante Quebec career grant; American Society for Gastrointestinal Endoscopy Research Award; Boston Scientific
FX This study was supported by a Canadian Institute of Health Research project grant, Fonds de Recherche Sante Quebec career grant, American Society for Gastrointestinal Endoscopy Research Award, and arm's-length funding from Boston Scientific.
CR Abraham NS, 2002, GASTROINTEST ENDOSC, V56, P835, DOI 10.1067/mge.2002.129868
Almadi MA, 2017, AM J GASTROENTEROL, V112, P260, DOI 10.1038/ajg.2016.512
Almadi MA, 2016, GASTROINTEST ENDOSC, V83, P852, DOI 10.1016/j.gie.2015.10.035

Almadi Majid A, 2015, Gastrointest Endosc Clin N Am, V25, P691, DOI [10.1016/j.giec.2015.06.002, 10.1016/j.giec.2015.06.002]

Anderloni A, 2019, ENDOSCOPY, V51, pE239, DOI 10.1055/a-0889-7051

Anderloni A, 2019, GASTROINTEST ENDOSC, V89, P69, DOI [10.1016/j.gie.2018.04.1746, 10.1016/j.gie.2018.08.047]

Bailey AA, 2008, ENDOSCOPY, V40, P296, DOI 10.1055/s-2007-995566

Bang JY, 2018, GASTROINTEST ENDOSC, V88, P9, DOI 10.1016/j.gie.2018.03.012

Barkun AN, 2015, CAN J GASTROENTEROL, V29, P377, DOI 10.1155/2015/743417

Bishay K, 2019, ENDOSCOPY, V51, P950, DOI 10.1055/a-0901-7343

Chen YI, 2019, TRIALS, V20, DOI 10.1186/s13063-019-3918-y

Chen YI, 2018, GASTROINTEST ENDOSC, V87, P1215, DOI 10.1016/j.gie.2017.07.030

Conio M, 2018, GASTROINTEST ENDOSC, V88, P283, DOI 10.1016/j.gie.2018.03.029

Conroy T, 2011, NEW ENGL J MED, V364, P1817, DOI 10.1056/NEJMoa1011923

Cotton PB, 2010, GASTROINTEST ENDOSC, V71, P446, DOI 10.1016/j.gie.2009.10.027

Ekkelenkamp VE, 2015, ENDOSCOPY, V47, P504, DOI 10.1055/s-0034-1391231

Fine JP, 1999, J AM STAT ASSOC, V94, P496, DOI 10.2307/2670170

Freeman ML, 1996, NEW ENGL J MED, V335, P909, DOI 10.1056/NEJM199609263351301

Gardner TB, 2016, GASTROINTEST ENDOSC, V84, P460, DOI 10.1016/j.gie.2016.02.047

Giovannini M, 2001, ENDOSCOPY, V33, P898, DOI 10.1055/s-2001-17324

Herrmann R, 2007, J CLIN ONCOL, V25, P2212, DOI 10.1200/JCO.2006.09.0886

Jacques J, 2019, ENDOSCOPY, V51, P540, DOI 10.1055/a-0735-9137

Janet J, 2023, ANN SURG ONCOL, V30, P5036, DOI 10.1245/s10434-023-13466-8

Kiriyama S, 2018, J HEPATO-BIL-PAN SCI, V25, P17, DOI 10.1002/jhbp.512

Kochar B, 2015, GASTROINTEST ENDOSC, V81, P143, DOI 10.1016/j.gie.2014.06.045

Kunda R, 2016, SURG ENDOSC, V30, P5002, DOI 10.1007/s00464-016-4845-6

Lee JH, 2013, GASTROINTEST ENDOSC, V78, P312, DOI 10.1016/j.gie.2013.02.032

Lee YS, 2019, SCI REP-UK, V9, DOI 10.1038/s41598-019-52167-9

Li JJ, 2016, PLOS ONE, V11, DOI 10.1371/journal.pone.0149066

Liao WC, 2013, LANCET ONCOL, V14, P1095, DOI 10.1016/S1470-2045(13)70388-7

Macedo FI, 2019, ANN SURG, V270, P400, DOI 10.1097/SLA.0000000000003468

Miller CS, 2019, ENDOSC INT OPEN, V7, pE1563, DOI 10.1055/a-0998-8129

Nambu T, 2011, SCAND J GASTROENTERO, V46, P109, DOI 10.3109/00365521.2010.521889

Paik WH, 2018, AM J GASTROENTEROL, V113, P987, DOI 10.1038/s41395-018-0122-8

Palmieri V, 2023, ENDOSC ULTRASOUND, V12, P104, DOI 10.4103/EUS-D-21-00137

Porta Miquel, 2005, Clin Transl Oncol, V7, P189, DOI 10.1007/BF02712816

Rangelova E, 2021, ANN SURG, V273, P579, DOI 10.1097/SLA.0000000000003301

Stoop TF, 2022, HPB, V24, P1261, DOI 10.1016/j.hpb.2021.12.018

Suker M, 2016, LANCET ONCOL, V17, P801, DOI 10.1016/S1470-2045(16)00172-8

Teoh AYB, 2023, GASTROENTEROLOGY, V165, P473, DOI 10.1053/j.gastro.2023.04.016

Testoni PA, 2016, ENDOSCOPY, V48, P657, DOI 10.1055/s-0042-108641

Tse F, 2013, ENDOSCOPY, V45, P605, DOI 10.1055/s-0032-1326640

Tyberg Amy, 2023, J Clin Gastroenterol, V57, P962, DOI 10.1097/MCG.0000000000001795

Yan JJ, 2024, EUR J HEALTH ECON, V25, P147, DOI 10.1007/s10198-023-01570-1
NR 44
TC 95
Z9 107
U1 2
U2 12
PU W B SAUNDERS CO-ELSEVIER INC
PI PHILADELPHIA
PA 1600 JOHN F KENNEDY BOULEVARD, STE 1800, PHILADELPHIA, PA 19103-2899 USA
SN 0016-5085
EI 1528-0012
J9 GASTROENTEROLOGY
JI Gastroenterology
PD NOV
PY 2023
VL 165
IS 5
BP 1249
EP +
DI 10.1053/j.gastro.2023.07.024
EA OCT 2023
PG 18
WC Gastroenterology & Hepatology
WE Science Citation Index Expanded (SCI-EXPANDED)
SC Gastroenterology & Hepatology
GA X9XQ9
UT WOS:001101908500001
PM 37549753
OA hybrid
DA 2026-05-09
ER

PT J
AU Lewis, JJ
Kowalski, TE
AF Lewis, Jason J.
Kowalski, Thomas E.
TI Endoscopic Ultrasound and Fine Needle Aspiration in Pancreatic Cancer
SO CANCER JOURNAL
LA English
DT Review
DE Pancreatic cancer; endoscopic ultrasound (EUS); disease stage

ID EUS-GUIDED FNA; COMPUTED-TOMOGRAPHY; INTERSTITIAL BRACHYTHERAPY; INJECTION; ULTRASONOGRAPHY; TRIAL; GEMCITABINE; DRAINAGE; THERAPY

AB Pancreatic cancer is the fourth leading cause of cancer mortality for both men and women in the United States. Overall 5-year survival from time of diagnosis is less than 5%. The only meaningful chance for cure results from early detection while the tumor remains resectable without locoregional extension or metastasis. Endoscopic ultrasound (EUS) is uniquely suited to the examination and treatment of the pancreas. Endoscopic ultrasound can provide important information with regard to disease stage including involvement of the mesenteric vasculature, adjacent organ invasion, and regional lymph node metastasis. In addition, EUS is considered the procedure of choice for tissue diagnosis of pancreatic cancer. More recently, EUS has developed therapeutic roles in an array of other clinical situations including EUS-guided fine needle injection, EUS-guided biliary drainage, celiac plexus neurolysis, brachytherapy, fiducial placement, and emerging antitumor agents delivered directly into the tumor.

C1 [Kowalski, Thomas E.] Thomas Jefferson Univ, Div Gastroenterol & Hepatol, Dept Internal Med, Philadelphia, PA 19107 USA.
[Lewis, Jason J.] Carolinas Med Ctr, Div Gastroenterol, Dept Internal Med, Charlotte, NC 28203 USA.
C3 Thomas Jefferson University; Carolinas Medical Center
RP Kowalski, TE (corresponding author), Thomas Jefferson Univ, Div Gastroenterol & Hepatol, Dept Internal Med, 132 S 10th St,Suite 480 Main, Philadelphia, PA 19107 USA.
EM Thomas.Kowalski@jefferson.edu

CR Ang TL, 2007, J PANCREAS, V8, P438
Bilimoria KY, 2007, CANCER-AM CANCER SOC, V110, P738, DOI 10.1002/cncr.22852
CESPEDES I, 1993, ULTRASONIC IMAGING, V15, P73, DOI 10.1006/uimg.1993.1007
Chan HH, 2004, GASTROINTEST ENDOSC, V59, P95, DOI 10.1016/S0016-5107(03)02361-7
Chang KJ, 2000, CANCER-AM CANCER SOC, V88, P1325, DOI 10.1002/(SICI)1097-0142(20000315)88:6<1325::AID-CNCR8>3.0.CO;2-T
Cochlin DL, 2002, CLIN RADIOL, V57, P1014, DOI 10.1053/crad.2002.0989
DeWitt J, 2004, ANN INTERN MED, V141, P753, DOI 10.7326/0003-4819-141-10-200411160-00006
Eisen GM, 2001, GASTROINTEST ENDOSC, V54, P811, DOI 10.1016/S0016-5107(01)70082-X
Eloubeidi MA, 2005, GASTROINTEST ENDOSC, V61, P700, DOI 10.1016/S0016-5107(05)00363-9
Enochsson L, 2010, GASTROINTEST ENDOSC, V72, P1175, DOI 10.1016/j.gie.2010.07.047
Goldberg SN, 1999, GASTROINTEST ENDOSC, V50, P392, DOI 10.1053/ge.1999.v50.98847
Greene FL, 2002, CANC STAGING HDB AM, P179
Gress F, 1999, AM J GASTROENTEROL, V94, P900
Gress F, 2001, ANN INTERN MED, V134, P459, DOI 10.7326/0003-4819-134-6-200103200-00010
Hecht JR, 2012, GASTROINTEST ENDOSC, V75, P332, DOI 10.1016/j.gie.2011.10.007
Hecht JR, 2003, CLIN CANCER RES, V9, P555
Hewitt MJ, 2012, GASTROINTEST ENDOSC, V75, P319, DOI 10.1016/j.gie.2011.08.049
Horwhat JD, 2006, GASTROINTEST ENDOSC, V63, P966, DOI 10.1016/j.gie.2005.09.028
Howlader N., SEER CANC STAT REV 1
Irisawa A, 2007, PANCREAS, V35, P189, DOI 10.1097/01.mpa.0000250141.25639.e9
Irisawa A, 2009, J HEPATO-BILIARY-PAN, V16, P598, DOI 10.1007/s00534-009-0131-5
Jemal A, 2006, CA-CANCER J CLIN, V56, P106, DOI 10.3322/canjclin.56.2.106
Jin Z, 2008, ENDOSCOPY, V40, P314, DOI 10.1055/s-2007-995476

Kahaleh M, 2006, GASTROINTEST ENDOSC, V64, P52, DOI 10.1016/j.gie.2006.01.063
KALSER MH, 1985, CANCER-AM CANCER SOC, V56, P397, DOI 10.1002/1097-0142(19850715)56:2<397::AID-CNCR2820560232>3.0.CO;2-I
Kappis M, 1914, Verh. Dtsch. Ges. Chir, V43, P87
Klapman JB, 2003, AM J GASTROENTEROL, V98, P1289, DOI 10.1016/S0002-9270(03)00246-6
Kothary N, 2009, J VASC INTERV RADIOL, V20, P235, DOI 10.1016/j.jvir.2008.09.026
Matthes K, 2007, GASTROINTEST ENDOSC, V65, P448, DOI 10.1016/j.gie.2006.06.030
Puli SR, 2009, DIGEST DIS SCI, V54, P2330, DOI 10.1007/s10620-008-0651-x
Sanders MK, 2010, GASTROINTEST ENDOSC, V71, P1178, DOI 10.1016/j.gie.2009.12.020
Savides TJ, 2009, GASTROINTEST ENDOSC, V69, pS3, DOI 10.1016/j.gie.2008.10.060
Siddiqui AA, 2011, DIGEST DIS SCI, V56, P3370, DOI 10.1007/s10620-011-1782-z
Sreenarasimhaiah J, 2009, AM J MED SCI, V338, P319, DOI 10.1097/MAJ.0b013e3181a44405
Sun S, 2006, ENDOSCOPY, V38, P399, DOI 10.1055/s-2006-925253
Sun SY, 2005, GASTROINTEST ENDOSC, V62, P775, DOI 10.1016/j.gie.2005.06.050
Takao I, 2009, GASTROINTEST ENDOSC, V69, pS8
Tellez-Avila FI, 2012, PANCREAS, V41, P636, DOI 10.1097/MPA.0b013e31823e3632
Timmerman RD, 2007, J CLIN ONCOL, V25, P947, DOI 10.1200/JCO.2006.09.7469
Varadarajulu S, 2009, GASTROINTEST ENDOSC, V70, P372, DOI 10.1016/j.gie.2009.03.008
Volmar KE, 2005, GASTROINTEST ENDOSC, V61, P854, DOI 10.1016/S0016-5107(05)00364-0
WARD EM, 1979, ANESTH ANALG, V58, P461
WARSHAW AL, 1992, NEW ENGL J MED, V326, P455, DOI 10.1056/NEJM199202133260706
Wiersema MJ, 1996, GASTROINTEST ENDOSC, V43, P102, DOI 10.1016/S0016-5107(06)80108-2
Wild AT, 2012, J CLIN ONCOL, V30
Wyse JM, 2011, J CLIN ONCOL, V29, P3541, DOI 10.1200/JCO.2010.32.2750
NR 46
TC 7
Z9 9
U1 0
U2 13
PU LIPPINCOTT WILLIAMS & WILKINS
PI PHILADELPHIA
PA TWO COMMERCE SQ, 2001 MARKET ST, PHILADELPHIA, PA 19103 USA
SN 1528-9117
EI 1540-336X
J9 CANCER J
JI Cancer J.
PD NOV-DEC
PY 2012
VL 18
IS 6
BP 523
EP 529

DI 10.1097/PPO.0b013e318277008e
PG 7
WC Oncology
WE Science Citation Index Expanded (SCI-EXPANDED)
SC Oncology
GA 047IN
UT WOS:000311833400007
PM 23187838
DA 2026-05-09
ER

PT J
AU Imai, M
Takahashi, Y
Sato, T
Maruyama, M
Isokawa, O
AF Imai, Michitaka
Takahashi, Yoshifumi
Sato, Toshihiro
Maruyama, Masaki
Isokawa, Osamu
TI Long-term Outcomes of Elective EUS-guided Gallbladder Drainage After
Percutaneous Transhepatic Gallbladder Drainage
SO TECHNIQUES AND INNOVATIONS IN GASTROINTESTINAL ENDOSCOPY
LA English
DT Article
DE Acute cholecystitis; Endoscopic ultrasound-guided gallbladder drainage;
Percutaneous transhepatic gallbladder drainage
ID APPOSING METAL STENT; CRITICALLY ILL PATIENTS; HIGH-RISK PATIENTS; ACUTE
CHOLECYSTITIS; LAPAROSCOPIC CHOLECYSTECTOMY; CHOLECYSTOSTOMY;
FEASIBILITY; ASPIRATION; CONVERSION; PLACEMENT
AB BACKGROUND: Endoscopic ultrasound (EUS)-guided gallbladder drainage (GBD) has gained increasing popularity as an alternative treatment approach for acute cholecystitis in high-risk patients. We report the procedural and long-term outcomes of elective EUS-GBD using self-expandable metallic stents (SEMSs) after percutaneous transhepatic gallbladder drainage (PTGBD) for acute cholecystitis. METHODS: Between April 2015 and January 2017, 15 patients at the study institution underwent elective EUS-GBD after PTGBD. SEMSs were placed in all patients, and the external gallbladder catheter was removed one week after the procedure. Procedural and long-term outcomes were measured retrospectively. RESULTS: Both the technical and clinical success rates were 93.3% (14/15). Two patients (13.3%) developed adverse events, with distal stent migration in one patient (6.7%) and asymptomatic pneumoperitoneum in the other patient (6.7%). Cholecystitis recurred only in patients who experienced stent migration. None of the patients underwent elective cholecystectomy. The median duration of stent patency was 544 days (18-1006 days), without any deaths. The overall stent

patency rate was 86.7% (13/15). CONCLUSION: Thus, elective EUS-GBD using SEMSs after PTGBD exhibited favorable long-term outcomes, indicating that this approach is effective for patients with severe comorbidities that preclude
C1 [Imai, Michitaka; Takahashi, Yoshifumi; Sato, Toshihiro; Maruyama, Masaki; Isokawa, Osamu] Kashiwazaki Gen Hosp & Med Ctr, Dept Gastroenterol, 2-11-3 Kitahanda, Kashiwazaki, Niigata, Japan.
RP Imai, M (corresponding author), Kashiwazaki Gen Hosp & Med Ctr, Dept Gastroenterol, 2-11-3 Kitahanda, Kashiwazaki, Niigata, Japan.
EM michitaka7supernova1979@gmail.com
CR Akhan O, 2002, EUR J RADIOL, V43, P229, DOI 10.1016/S0720-048X(02)00158-4
Anderloni A, 2016, SURG ENDOSC, V30, P5200, DOI 10.1007/s00464-016-4894-x
Chantarojanasiri T, 2017, SAUDI J GASTROENTERO, V23, P218, DOI 10.4103/sjg.SJG_115_17
Choi JH, 2014, ENDOSCOPY, V46, P656, DOI 10.1055/s-0034-1365720
Chopra S, 2001, AM J ROENTGENOL, V176, P1025, DOI 10.2214/ajr.176.4.1761025
de la Serna-Higuera C, 2013, GASTROINTEST ENDOSC, V77, P303, DOI 10.1016/j.gie.2012.09.021
Dollhopf M, 2017, GASTROINTEST ENDOSC, V86, P636, DOI 10.1016/j.gie.2017.02.027
Irani S, 2015, GASTROINTEST ENDOSC, V82, P1110, DOI 10.1016/j.gie.2015.05.045
Ito K, 2004, AM J ROENTGENOL, V183, P193, DOI 10.2214/ajr.183.1.1830193
Itoi T, 2012, GASTROINTEST ENDOSC, V75, P870, DOI 10.1016/j.gie.2011.10.020
Itoi T, 2011, J HEPATO-BIL-PAN SCI, V18, P282, DOI 10.1007/s00534-010-0310-4
Jang JW, 2012, GASTROENTEROLOGY, V142, P805, DOI 10.1053/j.gastro.2011.12.051
Jang JW, 2011, GASTROINTEST ENDOSC, V74, P176, DOI 10.1016/j.gie.2011.03.1120
Kamata K, 2017, WORLD J GASTROENTERO, V23, P661, DOI 10.3748/wjg.v23.i4.661
Kimura Y, 2013, J HEPATO-BIL-PAN SCI, V20, P8, DOI 10.1007/s00534-012-0564-0
Law R, 2016, CLIN GASTROENTEROL H, V14, P476, DOI 10.1016/j.cgh.2015.10.026
Lee SS, 2007, GASTROINTEST ENDOSC, V66, P1008, DOI 10.1016/j.gie.2007.03.1080
Lee TH, 2011, ENDOSCOPY, V43, P702, DOI 10.1055/s-0030-1256226
MCGAHAN JP, 1989, RADIOLOGY, V173, P481, DOI 10.1148/radiology.173.2.2678261
McKay A, 2012, SURG ENDOSC, V26, P1343, DOI 10.1007/s00464-011-2035-0
Mutignani M, 2009, ENDOSCOPY, V41, P539, DOI 10.1055/s-0029-1214727
Raju RP, 2011, DIGEST DIS SCI, V56, P1557, DOI 10.1007/s10620-010-1550-5
Song TJ, 2010, GASTROINTEST ENDOSC, V71, P634, DOI 10.1016/j.gie.2009.11.024
Spira RM, 2002, AM J SURG, V183, P62, DOI 10.1016/S0002-9610(01)00849-2
Walter D, 2016, GUT, V65, P6, DOI 10.1136/gutjnl-2015-309925
Yi NJ, 2006, MINIM INVASIV THER, V15, P159, DOI 10.1080/13645700600760044
Yoon WJ, 2009, GASTROINTEST ENDOSC, V70, P284, DOI 10.1016/j.gie.2008.12.241
NR 27
TC 4
Z9 4
U1 0
U2 0
PU ELSEVIER SCIENCE INC
PI NEW YORK
PA STE 800, 230 PARK AVE, NEW YORK, NY 10169 USA

SN 2666-5107
EI 2590-0307
J9 TECH INNOVAT GASTROI
JI Tech. Innovation Gastrointes. Endoscopy
PY 2021
VL 23
IS 1
SI SI
BP 1
EP 7
DI 10.1016/j.tige.2020.09.002
PG 7
WC Gastroenterology & Hepatology
WE Emerging Sources Citation Index (ESCI)
SC Gastroenterology & Hepatology
GA ZT0AL
UT WOS:000768819900001
DA 2026-05-09
ER

PT J
AU Emmanuel, J
Omar, H
See, LT
AF Emmanuel, James
Omar, Haniza
See, Lee T.
TI Endoscopic ultrasound-guided hepaticogastrostomy using a partially
covered metal stent in patients with malignant biliary obstruction after
failedEndoscopic retrograde cholangiopancreatography
SO JGH OPEN
LA English
DT Article
DE endoscopic ultrasound-guided biliary drainage; malignant biliary
obstruction; partially covered metal stent
ID DRAINAGE
AB Background and Aim The advent of endoscopic ultrasound-guided biliary drainage (EUS-BD) has provided an inimitable alternative for gaining biliary access in patients who fail conventional endoscopic drainage. The antimigratory features of the partially covered metal stent (PCMS), namely, the flange head and uncovered portion of the stent, makes it a valuable option in patients undergoing EUS-guided hepaticogastrostomy (EUS-HGS). The aim of the study is to evaluate the clinical outcome of EUS-BD via the hepaticogastrostomy approach using PCMS in patients with malignant biliary obstruction after failed ERCP. Methods This is a single-center retrospective observational study of patients with malignant

biliary obstruction undergoing EUS-HGS after failed ERCP between January 2018 and May 2019. The end-point of the study was to assess the technical and clinical success rate, as well as the stent- and procedure-related complications. Results There were 20 subjects in this study. The average age was 71.8 +/- 7.6 years. Most patients were male, 16 (80%). Inaccessible papillae was the most common indication for this procedure, 16 (80%). Technical success was achieved in all patients. The average procedural time was 39.9 +/- 1.3 min. Mean preprocedural bilirubin levels were 348.6 +/- 28.8 and subsequently decreased to 108.94 +/- 37.1 mu mol/L at 2 weeks postprocedure. The clinical success rate was 95% (19/20), with one patient requiring percutaneous transhepatic biliary drainage (PTBD). There were no stent- or procedure-related complications reported in this study. Conclusion EUS-HGS with PCMS is a feasible, effective, and safe alternative for biliary decompression in patients with failed endoscopic retrograde cholangiopancreatography (ERCP).
C1 [Emmanuel, James; Omar, Haniza; See, Lee T.] Selayang Hosp, Dept Gastroenterol & Hepatol, Selangor, Malaysia.
RP Emmanuel, J (corresponding author), Selayang Hosp, Dept Gastroenterol & Hepatol, Selangor, Malaysia.
EM sentulian83@gmail.com
OI EMMANUEL, JAMES/0000-0001-7154-461X
CR Amano M, 2016, GASTROINTEST ENDOSC, V83, P633
[Anonymous], DIAGN THER ENDOSC
Baars JE, 2018, ENDOSC ULTRASOUND, V7, P4, DOI 10.4103/eus.eus_105_17
Cho DH, 2016, GASTROINTEST ENDOSC, V85, P1067
Artifon ELD, 2019, ENDOSC ULTRASOUND, V8, pS72, DOI 10.4103/eus.eus_62_19
De Cassan C, 2017, ENDOSC ULTRASOUND, V6, P329, DOI 10.4103/2303-9027.209869
Dhir V, 2015, GASTROINTEST ENDOSC, V81, P913, DOI 10.1016/j.gie.2014.09.054
Giovannini M, 2019, ENDOSC ULTRASOUND, V8, pS35, DOI 10.4103/eus.eus_47_19
Gornals JB, 2016, GASTROENT HEPAT-BARC, V39, P627, DOI 10.1016/j.gastrohep.2015.12.008
Honjo M, 2018, ENDOSC ULTRASOUND, V7, P376, DOI 10.4103/eus.eus_2_18
Hosmer A, 2018, ENDOSC INT OPEN, V6, pE127, DOI 10.1055/s-0043-123188
Ikeuchi N., 2015, GASTROINTEST INTERV, V4, P31, DOI [10.1016/j.gii.2015.04.002, 10.1016/j.gii.2015.04.002, DOI 10.1016/J.GII.2015.04.002]
Itoi T, 2010, J HEPATO-BIL-PAN SCI, V17, P611, DOI 10.1007/s00534-009-0196-1
Jovani M, 2019, ENDOSC ULTRASOUND, V8, pS57, DOI 10.4103/eus.eus_53_19
Kahaleh M, 2006, GASTROINTEST ENDOSC, V64, P52, DOI 10.1016/j.gie.2006.01.063
Kamata K, 2017, ARAB J GASTROENTEROL, V18, P120, DOI 10.1016/j.ajg.2017.05.016
Ki ELL, 2019, WORLD J GASTRO ENDOS, V11, P345, DOI 10.4253/wjge.v11.i5.345
Leung ELK., 2019, ENDOSC ULTRASOUND, V8, P17
Lu LL, 2017, COMPLEXITY, DOI 10.1155/2017/7628537
Minaga K, 2017, SURG ENDOSC, V31, P4764, DOI 10.1007/s00464-017-5553-6
Miyano A, 2018, J GASTROINTEST SURG, V22, P1312, DOI 10.1007/s11605-018-3758-1
Moryoussef F, 2017, THER ADV GASTROENTER, V10, P537, DOI 10.1177/1756283X17702614
Nennstiel S, 2015, J CLIN GASTROENTEROL, V49, P764, DOI 10.1097/MCG.0000000000000275
Ogura T, 2016, WORLD J GASTROENTERO, V22, P3945, DOI 10.3748/wjg.v22.i15.3945
Okuno N, 2018, J GASTROEN HEPATOL, V33, P1413, DOI 10.1111/jgh.14112
Rimba M, 2017, ENDOSC ULTRASOUND, V6, P122
Samarasena Jason B, 2012, J Interv Gastroenterol, V2, P78
Savides TJ, 2009, GASTROINTEST ENDOSC, V69, pS3, DOI 10.1016/j.gie.2008.10.060

Sportes A, 2017, THER ADV GASTROENTER, V10, P483, DOI 10.1177/1756283X17702096
Yamao K, 2010, GUT LIVER, V4, pS67, DOI 10.5009/gnl.2010.4.S1.S67
NR 30
TC 7
Z9 7
U1 0
U2 0
PU WILEY
PI HOBOKEN
PA 111 RIVER ST, HOBOKEN 07030-5774, NJ USA
SN 2397-9070
J9 JGH OPEN
JI JGH Open
PD DEC
PY 2020
VL 4
IS 6
BP 1059
EP 1064
DI 10.1002/jgh3.12386
EA JUL 2020
PG 6
WC Gastroenterology & Hepatology
WE Emerging Sources Citation Index (ESCI)
SC Gastroenterology & Hepatology
GA PD0XE
UT WOS:000547568700001
PM 33319037
OA Green Submitted, gold
DA 2026-05-09
ER

PT J
AU Okuno, N
Hara, K
Mizuno, N
Haba, S
Kuwahara, T
Koda, H
Tajika, M
Tanaka, T
Onishi, S

Yamada, K
Miyano, A
Fumihara, D
Elshair, M
AF Okuno, Nozomi
Hara, Kazuo
Mizuno, Nobumasa
Haba, Shin
Kuwahara, Takamichi
Koda, Hiroki
Tajika, Masahiro
Tanaka, Tsutomu
Onishi, Sachiyo
Yamada, Keisaku
Miyano, Akira
Fumihara, Daiki
Elshair, Moaz
TI Outcomes of Endoscopic Ultrasound-Guided Biliary Drainage in Patients
Undergoing Antithrombotic Therapy
SO CLINICAL ENDOSCOPY
LA English
DT Article
DE Anticoagulant agents; Antiplatelet agents; Antithrombotic agents;
Bleeding; FUS-guided biliary drainage
ID SPHINCTEROTOMY; GUIDELINES; EVENTS; FNA
AB Background/Aims: The Japan Gastroenterological Endoscopy Society (JGES) has published guidelines for gastroenterological endoscopy in patients undergoing antithrombotic treatment. These guidelines classify endoscopic ultrasound-guided biliary drainage (EUS-BD) as a high-risk procedure. Nevertheless, the bleeding risk of EUS-BD in patients undergoing antithrombotic therapy is uncertain. Therefore, this study aimed to assess the bleeding risk in patients undergoing antithrombotic therapy.

Methods: This single-center retrospective study included 220 consecutive patients who underwent EUS-BD between January 2013 and December 2018. We managed the withdrawal and continuation of antithrombotic agents according to the JGES guidelines. We compared the bleeding event rates among patients who received and those who did not receive antithrombotic agents.

Results: A total of 18 patients (8.1%) received antithrombotic agents and 202 patients (91.8%) did not. Three patients experienced bleeding events, with an overall bleeding event rate of 1.3% (3/220): one patient was in the antithrombotic group (5.5%) and two patients were in the non-antithrombotic group (0.9%) (p=0.10). All cases were moderate. The sole thromboembolic event (0.4%) was a cerebral infarction in a patient in the non-antithrombotic group.

Conclusions: The rate of EUS-BD-related bleeding events was low. Even in patients receiving antithrombotic therapy, the bleeding event rates were not significantly different from those in patients not receiving antithrombotic therapy.
C1 [Okuno, Nozomi; Hara, Kazuo; Mizuno, Nobumasa; Haba, Shin; Kuwahara, Takamichi; Koda, Hiroki; Miyano, Akira; Fumihara, Daiki; Elshair, Moaz] Aichi Canc Ctr Hosp, Dept Gastroenterol, Nagoya, Aichi, Japan.

[Tajika, Masahiro; Tanaka, Tsutomu; Onishi, Sachiyo; Yamada, Keisaku] Aichi Canc Ctr Hosp, Dept Endoscopy, Nagoya, Aichi, Japan.
C3 Aichi Cancer Center; Aichi Cancer Center
RP Hara, K (corresponding author), Aichi Canc Ctr Hosp, Dept Gastroenterol, Chikusa Ku, 1-1 Kanokoden, Nagoya, Aichi 4648681, Japan.
EM khara@aichi-cc.jp
RI Elshair, Moaz/HLP-7842-2023; Okuno, Nozomi/AHE-2009-2022
OI Sachiyo, Onishi/0000-0001-8633-1019; Koda, Hiroki/0000-0003-4000-6899;
Okuno, Nozomi/0000-0001-6376-687X; Mizuno, Nobumasa/0000-0001-9704-1885;
Haba, Shin/0000-0002-7578-5610; HARA, KAZUO/0000-0002-4699-6136; Tajika, Masahiro/0000-0002-3761-6971; Kuwahara, Takamichi/0000-0001-8348-8926
CR Anderson MA, 2009, GASTROINTEST ENDOSC, V70, P1060, DOI 10.1016/j.gie.2009.09.040
Baron TH, 2013, NEW ENGL J MED, V369, P1079, DOI 10.1056/NEJMc1308259
Boustière C, 2011, ENDOSCOPY, V43, P445, DOI 10.1055/s-0030-1256317
Cotton PB, 2010, GASTROINTEST ENDOSC, V71, P446, DOI 10.1016/j.gie.2009.10.027
Douketis JD, 2015, NEW ENGL J MED, V373, P823, DOI 10.1056/NEJMoa1501035
Fujimoto K, 2014, DIGEST ENDOSC, V26, P1, DOI 10.1111/den.12183
Hamada T, 2015, ENDOSCOPY, V47, P997, DOI 10.1055/s-0034-1392408
Ikarashi S, 2017, J GASTROENTEROL, V52, P1258, DOI 10.1007/s00535-017-1347-9
Inoue T, 2017, DIGEST ENDOSC, V29, P91, DOI 10.1111/den.12687
Isayama H, 2019, J HEPATO-BIL-PAN SCI, V26, P249, DOI 10.1002/jhbp.631
Kato M, 2018, DIGEST ENDOSC, V30, P433, DOI 10.1111/den.13184
Kawakubo K, 2018, GUT LIVER, V12, P353, DOI 10.5009/gnl17293
Li HK, 2011, PACE, V34, P868, DOI 10.1111/j.1540-8159.2011.03049.x
Matsumoto S, 2020, DIGEST ENDOSC, V32, P957, DOI 10.1111/den.13620
Okuno N, 2017, INT J GASTROINT INT, V6, P82, DOI 10.18528/gii170008
Okuno N, 2018, J GASTROEN HEPATOL, V33, P1413, DOI 10.1111/jgh.14112
Polmanee P, 2019, ENDOSC INT OPEN, V7, pE15, DOI 10.1055/a-0735-9107
Veitch AM, 2008, GUT, V57, P1322, DOI 10.1136/gut.2007.142497
Vu CKF, 2006, GASTROINTEST ENDOSC, V63, P808, DOI 10.1016/j.gie.2005.09.033
NR 19
TC 11
Z9 12
U1 0
U2 1
PU KOREAN SOC GASTROINTESTINAL ENDOSCOPY
PI SEOUL
PA 2003 LG PALACE, 165-8 DONGGYO-DONG, MAPO-GU, SEOUL, 121-754, SOUTH KOREA
SN 2234-2400
EI 2234-2443
J9 CLIN ENDOSC
JI Clin. Endosc.

PD JUL
PY 2021
VL 54
IS 4
BP 596
EP 602
DI 10.5946/ce.2020.194
PG 7
WC Gastroenterology & Hepatology
WE Emerging Sources Citation Index (ESCI)
SC Gastroenterology & Hepatology
GA TU2CY
UT WOS:000680848600021
PM 33596639
OA Green Submitted, gold
DA 2026-05-09
ER

PT J
AU Ogura, T
Nishioka, N
Yamada, M
Yamada, T
Ueno, S
Matsuno, J
Ueshima, K
Yamamoto, Y
Okuda, A
Higuchi, K
AF Ogura, Takeshi
Nishioka, Nobu
Yamada, Masanori
Yamada, Tadahiro
Ueno, Saori
Matsuno, Jyun
Ueshima, Kazuya
Yamamoto, Yoshitaro
Okuda, Atsushi
Higuchi, Kazuhide
TI Novel transluminal treatment protocol for hepaticojejunostomy stricture
using covered self-expandable metal stent
SO SURGICAL ENDOSCOPY AND OTHER INTERVENTIONAL TECHNIQUES

LA English

DT Article

DE EUS; EUS-HGS; Hepaticojejunostomy stricture; Endoscopic
ultrasound-guided biliary drainage; ERCP

ID GUIDED BILIARY DRAINAGE; ANATOMY

AB Background Hepaticojejunostomy anastomotic stricture (HJS) is a rare complication after pancreatoduodenostomy. However, the rate of HJS may be increased with the expansion of operative indications, such as intraductal papillary mucinous neoplasm. Recently, the indications for EUS-guided biliary drainage to treat benign biliary disease have expanded. Recently, novel transluminal treatment protocol has been established in our hospital. The aim of this study was thus to evaluate the technical feasibility and safety of our treatment protocol. Patients and method Consecutive patients with complications of HJS between January and December 2018 were enrolled in this study. EUS-guided hepaticogastrostomy (HGS) is firstly performed. After 7 days to create the fistula, HGS stent is removed. HJS is transluminally evaluated by a cholangioscope, and antegrade balloon dilation is attempted. After 3 months, if HJS is still presence, antegrade stent deployment is performed using a covered metal stent. Also, after 1 month, antegrade stent removal is transluminally performed. Results Among total 29 patients, 14 patients were underwent antegrade metal stent deployment. The technical success rate of antegrade stent deployment was 92.9%. Median period of stent placement was 30.5 days (range 28-38 days), and transluminal stent removal was successfully performed in all patients. During follow-up (median 278 days; range 171-505 days), recurrence of HJS was seen in 2 patients. Severe adverse events were not seen in any patients during follow-up period. Conclusion Transluminal stent deployment for HJS under EUS-guidance appears feasible and safe, although further study with a larger sample size and longer follow-up is warranted.

C1 [Ogura, Takeshi; Nishioka, Nobu; Yamada, Masanori; Yamada, Tadahiro; Ueno, Saori; Matsuno, Jyun; Ueshima, Kazuya; Yamamoto, Yoshitaro; Okuda, Atsushi; Higuchi, Kazuhide] Osaka Med Coll, Dept Internal Med 2, 2-7 Daigakuchou, Takatsuki, Osaka 5698686, Japan.

C3 Osaka Medical & Pharmaceutical University

RP Ogura, T (corresponding author), Osaka Med Coll, Dept Internal Med 2, 2-7 Daigakuchou, Takatsuki, Osaka 5698686, Japan.

EM oguratakeshi0411@yahoo.co.jp

RI Ogura, Takeshi/AAU-5643-2021; yamamoto, yoshitaro/OML-4665-2025

CR Baars JE, 2018, ENDOSC ULTRASOUND, V7, P4, DOI 10.4103/eus.eus_105_17
Balcom JH, 2001, ARCH SURG-CHICAGO, V136, P391, DOI 10.1001/archsurg.136.4.391
Bonnel DH, 2012, AM J SURG, V203, P675, DOI 10.1016/j.amjsurg.2012.02.001
Bukhari M, 2018, GASTROINTEST ENDOSC, V88, P486, DOI 10.1016/j.gie.2018.04.2356
Cotton PB, 2010, GASTROINTEST ENDOSC, V71, P446, DOI 10.1016/j.gie.2009.10.027
Glas L, 2008, J VASC INTERV RADIOL, V19, P1336, DOI 10.1016/j.jvir.2008.05.019
House MG, 2006, ANN SURG, V243, P571, DOI 10.1097/01.sla.0000216285.07069.fc
Khashab MA, 2016, ENDOSC INT OPEN, V4, pE1322, DOI 10.1055/s-0042-110790
Khashab MA, 2015, GASTROINTEST ENDOSC, V82, P993, DOI 10.1016/j.gie.2015.06.043
Mizukawa S, 2018, BMC GASTROENTEROL, V18, DOI 10.1186/s12876-018-0742-x
MOLNAR W, 1974, AM J ROENTGENOL, V122, P356, DOI 10.2214/ajr.122.2.356
Mukai S, 2019, GASTROINTEST ENDOSC, V89, P399, DOI 10.1016/j.gie.2018.07.030
Nakai Y, 2019, CLIN ENDOSC, V52, P212, DOI 10.5946/ce.2018.188
Reid-Lombardo KM, 2007, J GASTROINTEST SURG, V11, P1704, DOI 10.1007/s11605-007-0369-7

Sano I, 2019, J GASTROEN HEPATOL, V34, P612, DOI 10.1111/jgh.14605
Shao XD, 2017, SAUDI J GASTROENTERO, V23, P150, DOI 10.4103/1319-3767.207713
Shimatani M, 2009, ENDOSCOPY, V41, P849, DOI 10.1055/s-0029-1215108
Shimatani M, 2017, J GASTROEN HEPATOL, V32, P1348, DOI 10.1111/jgh.13713
Tanisaka Y, 2019, DIGEST ENDOSC, V31, P682, DOI 10.1111/den.13414
Yamada A, 2019, DIGEST ENDOSC, V31, P86, DOI 10.1111/den.13261
NR 20
TC 32
Z9 34
U1 0
U2 6
PU SPRINGER
PI NEW YORK
PA ONE NEW YORK PLAZA, SUITE 4600, NEW YORK, NY, UNITED STATES
SN 0930-2794
EI 1432-2218
J9 SURG ENDOSC
JI Surg. Endosc.
PD JAN
PY 2021
VL 35
IS 1
BP 209
EP 215
DI 10.1007/s00464-020-07381-2
PG 7
WC Surgery
WE Science Citation Index Expanded (SCI-EXPANDED)
SC Surgery
GA PG9JZ
UT WOS:000600044000021
PM 31932928
DA 2026-05-09
ER

PT J
AU Mori, Y
Itoi, T
Baron, TH
Takada, T
Strasberg, SM
Pitt, HA

Ukai, T
Shikata, S
Noguchi, Y
Teoh, AYB
Kim, MH
Asbun, HJ
Endo, I
Yokoe, M
Miura, F
Okamoto, K
Suzuki, K
Umezawa, A
Iwashita, Y
Hibi, T
Wakabayashi, G
Han, HS
Yoon, YS
Choi, IS
Hwang, TL
Chen, MF
Garden, OJ
Singh, H
Liau, KH
Huang, WSW
Gouma, DJ
Belli, G
Dervenis, C
de Santibañes, E
Giménez, ME
Windsor, JA
Lau, WY
Cherqui, D
Jagannath, P
Supe, AN
Liu, KH
Su, CH
Deziel, DJ
Chen, XP
Fan, ST
Ker, CG
Jonas, E
Padbury, R

Mukai, S
Honda, G
Sugioka, A
Asai, K
Higuchi, R
Wada, K
Yoshida, M
Mayumi, T
Hirata, K
Sumiyama, Y
Inui, K
Yamamoto, M
AF Mori, Yasuhisa
Itoi, Takao
Baron, Todd H.
Takada, Tadahiro
Strasberg, Steven M.
Pitt, Henry A.
Ukai, Tomohiko
Shikata, Satoru
Noguchi, Yoshinori
Teoh, Anthony Yuen Bun
Kim, Myung-Hwan
Asbun, Horacio J.
Endo, Itaru
Yokoe, Masamichi
Miura, Fumihiko
Okamoto, Kohji
Suzuki, Kenji
Umezawa, Akiko
Iwashita, Yukio
Hibi, Taizo
Wakabayashi, Go
Han, Ho-Seong
Yoon, Yoo-Seok
Choi, In-Seok
Hwang, Tsann-Long
Chen, Miin-Fu
Garden, O. James
Singh, Harjit
Liau, Kui-Hin
Huang, Wayne Shih-Wei

Gouma, Dirk J.
Belli, Giulio
Dervenis, Christos
de Santibanes, Eduardo
Eduardo Gimenez, Mariano
Windsor, John A.
Lau, Wan Yee
Cherqui, Daniel
Jagannath, Palepu
Supe, Avinash Nivritti
Liu, Keng-Hao
Su, Cheng-Hsi
Deziel, Daniel J.
Chen, Xiao-Ping
Fan, Sheung Tat
Ker, Chen-Guo
Jonas, Eduard
Padbury, Robert
Mukai, Shuntaro
Honda, Goro
Sugioka, Atsushi
Asai, Koji
Higuchi, Ryota
Wada, Keita
Yoshida, Masahiro
Mayumi, Toshihiko
Hirata, Koichi
Sumiyama, Yoshinobu
Inui, Kazuo
Yamamoto, Masakazu
TI Tokyo Guidelines 2018: management strategies for gallbladder drainage in patients with acute cholecystitis (with videos)
SO JOURNAL OF HEPATO-BILIARY-PANCREATIC SCIENCES
LA English
DT Article
DE Acute cholecystitis; Endoscopic transpapillary gallbladder drainage; Endoscopic ultrasound-guided gallbladder drainage; Gallbladder drainage; Percutaneous transhepatic gallbladder drainage
ID APPOSING METAL STENT; CRITICALLY ILL PATIENTS; HIGH-RISK PATIENTS; PERCUTANEOUS CHOLECYSTOSTOMY; LAPAROSCOPIC CHOLECYSTECTOMY; JAPAN; TAIWAN; ASPIRATION; PLACEMENT; SINGLE
AB Since the publication of the Tokyo Guidelines in 2007 and their revision in 2013, appropriate management for acute

cholecystitis has been more clearly established. Since the last revision, several manuscripts, especially for alternative endoscopic techniques, have been reported; therefore, additional evaluation and refinement of the 2013 Guidelines is required. We describe a standard drainage method for surgically high-risk patients with acute cholecystitis and the latest developed endoscopic gallbladder drainage techniques described in the updated Tokyo Guidelines 2018 (TG18). Our study confirmed that percutaneous transhepatic gallbladder drainage should be considered the first alternative to surgical intervention in surgically high-risk patients with acute cholecystitis. Also, endoscopic transpapillary gallbladder drainage or endoscopic ultrasound-guided gallbladder drainage can be considered in high-volume institutes by skilled endoscopists. In the endoscopic transpapillary approach, either endoscopic naso-gallbladder drainage or gallbladder stenting can be considered for gallbladder drainage. We also introduce special techniques and the latest outcomes of endoscopic ultrasound-guided gallbladder drainage studies. Free full articles and mobile app of TG18 are available at: . Related clinical questions and references are also included.
C1 [Mori, Yasuhisa] Kyushu Univ, Grad Sch Med Sci, Dept Surg & Oncol, Fukuoka, Japan.
[Itoi, Takao; Mukai, Shuntaro] Tokyo Med Univ Hosp, Dept Gastroenterol & Hepatol, Tokyo, Japan.
[Baron, Todd H.] Univ North Carolina Chapel Hill, Div Gastroenterol & Hepatol, Chapel Hill, NC USA.
[Takada, Tadahiro; Miura, Fumihiko; Wada, Keita] Teikyo Univ, Dept Surg, Sch Med, Tokyo, Japan.
[Strasberg, Steven M.] Washington Univ, Sect Hepatopancreatobiliary Surg, St Louis, MO USA.
[Pitt, Henry A.] Temple Univ, Lewis Katz Sch Med, Philadelphia, PA 19122 USA.
[Ukai, Tomohiko] Mie Prefectural Ichishi Hosp, Dept Family Med, Tsu, Mie, Japan.
[Shikata, Satoru] Mie Prefectural Ichishi Hosp, Tsu, Mie, Japan.
[Noguchi, Yoshinori; Yokoe, Masamichi] Japanese Red Cross Nagoya Daini Hosp, Dept Gen Internal Med, Nagoya, Aichi, Japan.
[Teoh, Anthony Yuen Bun] Chinese Univ Hong Kong, Dept Surg, Shatin, Hong Kong, Peoples R China.
[Kim, Myung-Hwan] Univ Ulsan, Dept Gastroenterol, Coll Med, Seoul, South Korea.
[Asbun, Horacio J.] Mayo Clin, Coll Med, Dept Surg, Jacksonville, FL 32224 USA.
[Endo, Itaru] Yokohama City Univ, Dept Surg Gastroenterol, Grad Sch Med, Yokohama, Kanagawa, Japan.
[Okamoto, Kohji] Kitakyushu City Yahata Hosp, Ctr Gastroenterol & Liver Dis, Dept Surg, Fukuoka, Japan.
[Suzuki, Kenji] Fujinomiya City Gen Hosp, Dept Surg, Shizuoka, Japan.
[Umezawa, Akiko] Yotsuya Med Cube, Minimally Invas Surg Ctr, Tokyo, Japan.
[Iwashita, Yukio] Oita Univ, Dept Gastroenterol & Pediat Surg, Fac Med, Oita, Japan.
[Hibi, Taizo] Keio Univ, Dept Surg, Sch Med, Tokyo, Japan.
[Wakabayashi, Go] Ageo Cent Gen Hosp, Dept Surg, Saitama, Japan.
[Han, Ho-Seong; Yoon, Yoo-Seok] Seoul Natl Univ, Coll Med, Dept Surg, Bundang Hosp, Seoul, South Korea.
[Choi, In-Seok] Konyang Univ Hosp, Dept Surg, Daejeon, South Korea.
[Hwang, Tsann-Long; Chen, Miin-Fu; Liu, Keng-Hao] Linkou Chang Gung Mem Hosp, Div Gen Surg, Taoyuan, Taiwan.
[Garden, O. James] Univ Edinburgh, Surg Clin, Edinburgh, Midlothian, Scotland.
[Singh, Harjit] Hosp Selayang, Dept Hepatopancreatobiliary Surg, Selangor, Malaysia.
[Liau, Kui-Hin] Mt Elizabeth Novena Hosp, Liau KH Consulting PL, Singapore, Singapore.
[Liau, Kui-Hin] Natl Univ Singapore, Yong Loo Lin Sch Med, Singapore, Singapore.
[Huang, Wayne Shih-Wei] Show Chwan Mem Hosp, Dept Surg, Changhua, Taiwan.
[Gouma, Dirk J.] Acad Med Ctr, Dept Surg, Amsterdam, Netherlands.
[Belli, Giulio] Loreto Nuovo Hosp, Dept Gen & HPB Surg, Naples, Italy.

[Dervenis, Christos] Agia Olga Hosp, Dept Surg 1, Athens, Greece.
[de Santibanes, Eduardo] Univ Buenos Aires, Hosp Italiano, Dept Surg, Buenos Aires, DF, Argentina.
[Eduardo Gimenez, Mariano] Univ Buenos Aires, DAICIM Fdn, Chair Gen Surg & Minimal Invas Surg Taquini, Buenos Aires, DF, Argentina.
[Windsor, John A.] Univ Auckland, Dept Surg, Auckland, New Zealand.
[Lau, Wan Yee] Chinese Univ Hong Kong, Fac Med, Shatin, Hong Kong, Peoples R China.
[Cherqui, Daniel] Hop Paul Brousse, Hepatobiliary Ctr, Villejuif, France.
[Jagannath, Palepu] Lilavati Hosp & Res Ctr, Dept Surg Oncol, Bombay, Maharashtra, India.
[Supe, Avinash Nivritti] Seth GS Med Coll & KEM Hosp, Dept Surg Gastroenterol, Bombay, Maharashtra, India.
[Su, Cheng-Hsi] Cheng Hsin Gen Hosp, Dept Surg, Taipei, Taiwan.
[Deziel, Daniel J.] Rush Univ, Med Ctr, Dept Surg, Chicago, IL 60612 USA.
[Chen, Xiao-Ping] Huazhong Univ Sci & Technol, Tongji Hosp, Tongji Med Coll, Hepat Surg Ctr,Dept Surg, Wuhan, Hubei, Peoples R China.
[Fan, Sheung Tat] Hong Kong Sanat & Hosp, Liver Surg Ctr, Hong Kong, Hong Kong, Peoples R China.
[Ker, Chen-Guo] Yuans Gen Hosp, Dept Surg, Kaohsiung, Taiwan.
[Jonas, Eduard] Univ Cape Town, Surg Gastroenterol Hepatopancreatobiliary Unit, Cape Town, South Africa.
[Jonas, Eduard] Groote Schuur Hosp, Cape Town, South Africa.
[Padbury, Robert] Flinders Med Ctr, Div Surg & Specialty Serv, Adelaide, SA, Australia.
[Honda, Goro] Tokyo Metropolitan Komagome Hosp, Dept Surg, Tokyo, Japan.
[Sugioka, Atsushi] Fujita Hlth Univ, Dept Surg, Sch Med, Toyoake, Aichi, Japan.
[Asai, Koji] Toho Univ, Dept Surg, Ohashi Med Ctr, Tokyo, Japan.
[Higuchi, Ryota; Yamamoto, Masakazu] Tokyo Womens Med Univ, Dept Surg, Inst Gastroenterol, Tokyo, Japan.
[Yoshida, Masahiro] Int Univ Hlth & Welf, Ichikawa Hosp, Dept Hemodialysis & Surg, Chiba, Japan.
[Yoshida, Masahiro] Japan Council Qual Hlth Care, Dept EBM & Guidelines, Tokyo, Japan.
[Mayumi, Toshihiko] Univ Occupat & Environm Hlth, Sch Med, Dept Emergency Med, Fukuoka, Japan.
[Hirata, Koichi] JR Sapporo Hosp, Dept Surg, Sapporo, Hokkaido, Japan.
[Sumiyama, Yoshinobu] Toho Univ, Tokyo, Japan.
[Inui, Kazuo] Fujita Hlth Univ, Teaching Hosp 2, Dept Gastroenterol, Toyoake, Aichi, Japan.
C3 Kyushu University; Tokyo Medical University; University of North Carolina; University of North Carolina Chapel Hill; University of North Carolina School of Medicine; Teikyo University; Washington University (WUSTL); Pennsylvania Commonwealth System of Higher Education (PCSHE); Temple University; Japanese Red Cross Nagoya Daini Hospital; Chinese University of Hong Kong; University of Ulsan; Mayo Clinic; Yokohama City University; Oita University; Keio University; Seoul National University (SNU); Seoul National University Hospital; Konyang University; Konyang University Hospital; Chang Gung Memorial Hospital; University of Edinburgh; National University of Singapore; Show Chwan Memorial Hospital; University of Amsterdam; Academic Medical Center Amsterdam; General Hospital of N. Ionia Agia Olga; University of Buenos Aires; University of Buenos Aires Hospital; Hospital Italiano de Buenos Aires; University of Buenos Aires; University of Auckland; Chinese University

of Hong Kong; Assistance Publique Hopitaux Paris (APHP); Hopital Universitaire Paul-Brousse - APHP; Seth Gordhandas Sunderdas Medical College & King Edward Memorial Hospital; Cheng Hsin General Hospital; Rush University; Huazhong University of Science & Technology; University of Cape Town; University of Cape Town; Flinders Medical Centre; Tokyo Metropolitan Cancer & Infectious Diseases Center Komagome Hospital; Fujita Health University; Toho University; Tokyo Women's Medical University; International University of Health & Welfare; University of Occupational & Environmental Health - Japan; Toho University; Fujita Health University
RP Takada, T (corresponding author), Teikyo Univ, Sch Med, Dept Surg, Itabashi Ku, 2-11-1 Kaga, Tokyo 1738605, Japan.
EM t-takada@jshbps.jp
RI Honda, Goro/AAZ-8447-2020; Teoh, Anthony/L-4796-2016; Ker, Chen-Guo/D-5508-2009; Miura, Fumihiko/I-4631-2019; Yoon, Yoo-Seok/AAD-4820-2020; Mayumi, Toshihiko/AAC-7418-2019; Cherqui, Daniel/T-6831-2018; Higuchi, Ryota/AAF-4351-2020; Mukai, Shuntaro/AAD-4278-2020; Supe, Avinash/ABA-6934-2020; Jonas, Eduard/ABF-4888-2022; Harrison, Ewen/LZH-1527-2025; Okamoto, Kohji/AAF-5980-2020
OI Supe, Avinash/0000-0002-8325-1918; Jonas, Eduard/0000-0003-0123-256X; Windsor, John/0000-0001-5995-5432; Ukai, Tomohiko/0000-0002-0764-0110; Shikata, Satoru/0000-0002-9888-3344
CR Akhan O, 2002, EUR J RADIOL, V43, P229, DOI 10.1016/S0720-048X(02)00158-4
Anderloni A, 2016, SURG ENDOSC, V30, P5200, DOI 10.1007/s00464-016-4894-x
Asai K, 2017, ASIAN J ENDOSC SURG, V10, P166, DOI 10.1111/ases.12353
BABB RR, 1992, J CLIN GASTROENTEROL, V15, P238, DOI 10.1097/00004836-199210000-00014
Choi JH, 2015, DIGEST ENDOSC, V27, P1, DOI 10.1111/den.12386
Choi JH, 2014, ENDOSCOPY, V46, P656, DOI 10.1055/s-0034-1365720
Chopra S, 2001, AM J ROENTGENOL, V176, P1025, DOI 10.2214/ajr.176.4.1761025
Davis CA, 1999, ARCH SURG-CHICAGO, V134, P727, DOI 10.1001/archsurg.134.7.727
Dimou FM, 2017, J AM COLL SURGEONS, V224, pe1
Dollhopf M, 2017, GASTROINTEST ENDOSC, V86, P636, DOI 10.1016/j.gie.2017.02.027
DONALD JJ, 1994, GUT, V35, P692, DOI 10.1136/gut.35.5.692
Endo I, 2017, J HEPATO-BIL-PAN SCI, V24, P346, DOI 10.1002/jhbp.456
Hamada T, 2015, RADIOLOGY, V274, P605, DOI 10.1148/radiol.14140293
Hibi T, 2017, J HEPATO-BIL-PAN SCI, V24, P24, DOI 10.1002/jhbp.417
Hultman CS, 1996, AM SURGEON, V62, P263
Irani S, 2017, CLIN GASTROENTEROL H, V15, P738, DOI 10.1016/j.cgh.2016.12.021
Ito K, 2004, AM J ROENTGENOL, V183, P193, DOI 10.2214/ajr.183.1.1830193
Itoi T, 2008, WORLD J GASTROENTERO, V14, P6078, DOI 10.3748/wjg.14.6078
Itoi T, 2017, J HEPATO-BIL-PAN SCI, V24, P362, DOI 10.1002/jhbp.454
Itoi T, 2017, GASTROINTEST ENDOSC, V85, P88, DOI 10.1016/j.gie.2016.07.039

Itoi T, 2015, GASTROINTEST ENDOSC, V81, P111, DOI 10.1016/j.gie.2014.09.046
Itoi T, 2012, GASTROINTEST ENDOSC, V75, P870, DOI 10.1016/j.gie.2011.10.020
Itoi T, 2010, GASTROINTEST ENDOSC, V71, P1038, DOI 10.1016/j.gie.2010.01.026
Iwashita Y, 2017, J HEPATO-BIL-PAN SCI, V24, P191, DOI 10.1002/jhbp.440
Jang JW, 2012, GASTROENTEROLOGY, V142, P805, DOI 10.1053/j.gastro.2011.12.051
Jang JW, 2011, GASTROINTEST ENDOSC, V74, P176, DOI 10.1016/j.gie.2011.03.1120
Kahaleh M, 2016, DIGEST LIVER DIS, V48, P1054, DOI 10.1016/j.dld.2016.05.021
Kedia P, 2015, GASTROINTEST ENDOSC, V82, P1031, DOI 10.1016/j.gie.2015.03.1912
Khan MA, 2017, GASTROINTEST ENDOSC, V85, P76
Kiviniemi H, 1998, INT SURG, V83, P299
Komatsu S, 2016, J HEPATO-BIL-PAN SCI, V23, P708, DOI 10.1002/jhbp.394
Kwan V, 2007, GASTROINTEST ENDOSC, V66, P582, DOI 10.1016/j.gie.2007.02.065
Lillemoe KD, 2000, AM SURGEON, V66, P138
Loozen CS, 2017, DIGEST SURG, V34, P371, DOI 10.1159/000455241
Maekawa S, 2013, BMC GASTROENTEROL, V13, DOI 10.1186/1471-230X-13-65
MELIN MM, 1995, BRIT J SURG, V82, P1274, DOI 10.1002/bjs.1800820939
Moon JH, 2014, GASTROINTEST ENDOSC, V79, P990, DOI 10.1016/j.gie.2014.02.015
Mukai S, 2017, GASTROINTEST ENDOSC, V86, P203, DOI [10.1016/j.gie.2016.07.056, 10.1016/j.gie.2016.11.025, 10.1016/j.gie.2016.07.056]
Mutignani M, 2009, ENDOSCOPY, V41, P539, DOI 10.1055/s-0029-1214727
Pannala R, 2008, Minerva Gastroenterol Dietol, V54, P107
Patel IJ, 2012, J VASC INTERV RADIOL, V23, P727, DOI 10.1016/j.jvir.2012.02.012
Peñas-Herrero I, 2015, J HEPATO-BIL-PAN SCI, V22, P35, DOI 10.1002/jhbp.182
Shibasaki S, 2014, J HEPATO-BIL-PAN SCI, V21, P335, DOI 10.1002/jhbp.28
Song TJ, 2010, GASTROINTEST ENDOSC, V71, P634, DOI 10.1016/j.gie.2009.11.024
Sugiyama M, 1998, WORLD J SURG, V22, P459, DOI 10.1007/s002689900416
Takada T, 2013, J HEPATO-BIL-PAN SCI, V20, P1, DOI 10.1007/s00534-012-0566-y
Teoh AYB, 2017, ENDOSCOPY, V49, P130, DOI 10.1055/s-0042-119036
Tsutsui K, 2007, J GASTROENTEROL, V42, P583, DOI 10.1007/s00535-007-2061-9
Tsuyuguchi T, 2007, J HEPATO-BILIARY-PAN, V14, P46, DOI 10.1007/s00534-006-1155-8
Tsuyuguchi T, 2013, J HEPATO-BIL-PAN SCI, V20, P81, DOI 10.1007/s00534-012-0570-2
Tyberg A, 2018, J CLIN GASTROENTEROL, V52, P79, DOI 10.1097/MCG.0000000000000786
Walter D, 2016, GUT, V65, P6, DOI 10.1136/gutjnl-2015-309925
Widmer J, 2014, DIGEST ENDOSC, V26, P525, DOI 10.1111/den.12221
Yang MJ, 2016, SCAND J GASTROENTERO, V51, P472, DOI 10.3109/00365521.2015.1115116
Yokoe M, 2017, J HEPATO-BIL-PAN SCI, V24, P338, DOI 10.1002/jhbp.457
Yokoe M, 2017, J HEPATO-BIL-PAN SCI, V24, P319, DOI 10.1002/jhbp.450
NR 56
TC 232
Z9 265
U1 2
U2 38

PU WILEY
PI HOBOKEN
PA 111 RIVER ST, HOBOKEN 07030-5774, NJ USA
SN 1868-6974
EI 1868-6982
J9 J HEPATO-BIL-PAN SCI
JI J. Hepato-Biliary-Pancreat. Sci.
PD JAN
PY 2018
VL 25
IS 1
BP 87
EP 95
DI 10.1002/jhbp.504
PG 9
WC Gastroenterology & Hepatology; Surgery
WE Science Citation Index Expanded (SCI-EXPANDED)
SC Gastroenterology & Hepatology; Surgery
GA FS7ON
UT WOS:000419987400008
PM 28888080
OA Green Accepted, Green Submitted, Bronze
DA 2026-05-09
ER

PT J
AU de Paredes, AGG
Martin, JAG
Olcina, JRF
Lucas, DJ
Panizo, FG
Duran, SL
Sanchez, AM
Sanjuanbenito, A
Caminoa, A
Albillos, A
Vazquez-Sequeiros, E
AF Garcia de Paredes, Ana Garcia
Gonzalez Martin, Juan Angel
Foruny Olcina, Jose Ramon
Juzgado Lucas, Diego
Gonzalez Panizo, Fernando

Lopez Duran, Sergio
Martinez Sanchez, Alba
Sanjuanbenito, Alfonso
Caminoa, Alejandra
Albillos, Agustin
Vazquez-Sequeiros, Enrique

TI Lumen-apposing metal stents versus biliary fully-covered metal stents for EUS-guided drainage of pancreatic fluid collections: a case control study Meeting presentations: European Society of Gastrointestinal Endoscopy ESGE Days 2018

SO ENDOSCOPY INTERNATIONAL OPEN

LA English

DT Article

ID WALLED-OFF NECROSIS; TRANSMURAL DRAINAGE; NECROTIZING PANCREATITIS; PERCUTANEOUS DRAINAGE; DELIVERY-SYSTEM; NECROSECTOMY; PSEUDOCYSTS; OUTCOMES; EXPERIENCE; EFFICACY

AB Background and study aims Endoscopic ultrasound (EUS)-guided drainage has become first-line treatment for pancreatic fluid collections (PFC). The aim of this study was to compare the effectiveness and safety of biliary fully-covered self-expandable metal stents (BFCSEMS) and lumen-apposing metal stents with electrocautery (EC-LAMS). Patients and methods From April 2008 to March 2017, consecutive patients with symptomatic PFC drained under EUS-guidance with metal stents were included. Patients drained with EC-LAMS were considered the study group and those drained with BFCSEMS the control group. Two primary endpoints were evaluated: effectiveness (defined as reduction of >= 50 % of PFC size in cross-sectional imaging and improvement of symptoms 6 months after the transmural drainage) and safety. Results Thirty patients were drained with EC-LAMS and 60 patients with BFCSEMS. Patients and PFC baseline characteristics in both groups were similar. Use of a coaxial double pigtail plastic stent and a nasocystic lavage catheter was significantly less frequent in patients drained with EC-LAMS (33 % vs. 100 %, and 13 % vs. 58 %, respectively; P < 0.0001). Technical success was 100 % in both groups. Procedure time was < 30 minutes in all patients drained with EC-LAMS and over 30 minutes in all patients drained with BFCSEMS (P = 0.0001). Clinical success was higher with a tendency to significance in patients drained with EC-LAMS (96 % vs. 82 %, P = 0.055) and the adverse event rate was lower (4 % vs. 18 %, P = 0.04). No case of procedure-related mortality was recorded. Conclusions EC-LAMS and BFCSEMS are both effective for EUS-guided drainage of PFC. However, EC-LAMS requires less time to be performed and appears to be safer.

C1 [Garcia de Paredes, Ana Garcia; Gonzalez Martin, Juan Angel; Foruny Olcina, Jose Ramon; Lopez Duran, Sergio; Martinez Sanchez, Alba; Albillos, Agustin; Vazquez-Sequeiros, Enrique] Univ Alcala, Hosp Univ Ramon y Cajal, IRYCIS, Gastroenterol & Hepatol Dept, Madrid, Spain.
[Juzgado Lucas, Diego; Gonzalez Panizo, Fernando] Hosp Univ Quiron Madrid, Dept Gastroenterol, Madrid, Spain.
[Sanjuanbenito, Alfonso] Univ Alcala, IRYCIS, Hosp Univ Ramon y Cajal, Surg Dept, Madrid, Spain.
[Caminoa, Alejandra] Univ Alcala, IRYCIS, Hosp Univ Ramon y Cajal, Pathol Dept, Madrid, Spain.

C3 Universidad de Alcala; Hospital Universitario Ramon y Cajal; Universidad de Alcala; Hospital Universitario Ramon y Cajal; Universidad de Alcala; Hospital Universitario Ramon y Cajal

RP Vazquez-Sequeiros, E (corresponding author), Univ Alcala, IRYCIS, Hosp Univ Ramon y Cajal, Endoscopy

Unit,Gastroenterol & Hepatol Dept, Madrid, Spain.
EM evazquezse@gmail.com
OI VAZQUEZ-SEQUEIROS, ENRIQUE/0000-0003-4372-3893; Garcia Garcia de Paredes, Ana/0000-0001-5498-6280
CR Akshintala VS, 2014, GASTROINTEST ENDOSC, V79, P921, DOI 10.1016/j.gie.2013.10.032
Ang TL, 2017, DIGEST ENDOSC, V29, P463, DOI 10.1111/den.12797
Arvanitakis M, 2007, GASTROINTEST ENDOSC, V65, P609, DOI 10.1016/j.gie.2006.06.083
Bakker OJ, 2012, JAMA-J AM MED ASSOC, V307, P1053, DOI 10.1001/jama.2012.276
Bang JY, 2017, GUT, V66, P2054, DOI 10.1136/gutjnl-2016-312812
Bang JY, 2017, DIGEST ENDOSC, V29, P83, DOI 10.1111/den.12681
Bang JY, 2013, CLIN ENDOSC, V46, P500, DOI 10.5946/ce.2013.46.5.500
Banks PA, 2013, GUT, V62, P102, DOI 10.1136/gutjnl-2012-302779
Chen YI, 2018, GASTROINTEST ENDOSC, V88, P267, DOI 10.1016/j.gie.2018.03.021
Cosgrove ND, 2017, GASTROINTEST ENDOSC, V85, pAB101, DOI 10.1016/j.gie.2017.03.147
Cotton PB, 2010, GASTROINTEST ENDOSC, V71, P446, DOI 10.1016/j.gie.2009.10.027
Cui ML, 2014, DIGEST DIS SCI, V59, P1055, DOI 10.1007/s10620-013-2967-4
Gluck M, 2010, CLIN GASTROENTEROL H, V8, P1083, DOI 10.1016/j.cgh.2010.09.010
Keane MG, 2016, SURG ENDOSC, V30, P3730, DOI 10.1007/s00464-015-4668-x
Lopes CV, 2007, SCAND J GASTROENTERO, V42, P524, DOI 10.1080/00365520601065093
Mortelé KJ, 2009, AM J ROENTGENOL, V192, P110, DOI 10.2214/AJR.08.1116
Mukai S, 2015, ENDOSCOPY, V47, P47, DOI 10.1055/s-0034-1377966
Muthusamy VR, 2016, GASTROINTEST ENDOSC, V83, P481, DOI 10.1016/j.gie.2015.11.027
Patra PS, 2014, BRIT J SURG, V101, P1721, DOI 10.1002/bjs.9666
Penn DE, 2012, GASTROINTEST ENDOSC, V76, P679, DOI 10.1016/j.gie.2012.04.457
Rinninella E, 2015, GASTROINTEST ENDOSC, V82, P1039, DOI 10.1016/j.gie.2015.04.006
SAHEL J, 1987, PANCREAS, V2, P447, DOI 10.1097/00006676-198707000-00012
Sanchez-Yague A, 2015, GASTROINTEST ENDOSC, V81, pAB424, DOI 10.1016/j.gie.2015.03.1616
Shah RJ, 2015, CLIN GASTROENTEROL H, V13, P747, DOI 10.1016/j.cgh.2014.09.047
Siddiqui AA, 2017, GASTROINTEST ENDOSC, V85, P758, DOI 10.1016/j.gie.2016.08.014
Siddiqui AA, 2016, GASTROINTEST ENDOSC, V83, P699, DOI 10.1016/j.gie.2015.10.020
Siddiqui AA, 2013, GASTROINTEST ENDOSC, V78, P589, DOI 10.1016/j.gie.2013.03.1337
Song TJ, 2014, CLIN ENDOSC, V47, P222, DOI 10.5946/ce.2014.47.3.222
Teoh AYB, 2016, WORLD J GASTRO ENDOS, V8, P310, DOI 10.4253/wjge.v8.i6.310
van Brunschot S, 2014, SURG ENDOSC, V28, P1425, DOI 10.1007/s00464-013-3382-9
Varadarajulu S, 2008, GASTROINTEST ENDOSC, V68, P1102, DOI 10.1016/j.gie.2008.04.028
Varadarajulu S, 2013, GASTROENTEROLOGY, V145, P583, DOI 10.1053/j.gastro.2013.05.046
Vazquez-Sequeiros E, 2016, GASTROINTEST ENDOSC, V84, P450, DOI 10.1016/j.gie.2016.02.044
NR 33
TC 11
Z9 11
U1 0
U2 1

PU GEORG THIEME VERLAG KG
PI STUTTGART
PA RUDIGERSTR 14, D-70469 STUTTGART, GERMANY
SN 2364-3722
EI 2196-9736
J9 ENDOSC INT OPEN
JI Endosc. Int. Open
PD JAN
PY 2020
VL 8
IS 1
BP E6
EP E12
DI 10.1055/a-1031-9295
PG 7
WC Gastroenterology & Hepatology; Surgery
WE Emerging Sources Citation Index (ESCI)
SC Gastroenterology & Hepatology; Surgery
GA KB4DE
UT WOS:000506447500002
PM 31921978
OA Green Submitted, gold
DA 2026-05-09
ER

PT J
AU Storm, AC
Mahmoud, T
Akiki, K
Law, RJ
AF Storm, Andrew C.
Mahmoud, Tala
Akiki, Karl
Law, Ryan J.
TI Endoscopic Ultrasound-Guided Gastrojejunostomy for Superior Mesenteric
Artery Syndrome Secondary to Rapid Weight Loss
SO ACG CASE REPORTS JOURNAL
LA English
DT Article
ID MANAGEMENT
AB A 19-year-old man diagnosed with diffuse large B-cell lymphoma undergoing chemotherapy presented for recurrent emesis and weight loss. Imaging studies of the abdomen demonstrated features of superior mesenteric artery syndrome. The

patient deferred conservative treatment options and was deemed not to be a surgical candidate. Endoscopic ultrasound-guided gastroenterostomy using a lumen-apposing metal stent was performed to bypass the obstruction. Subsequently, the patient's oral intake and weight significantly improved. The stent was removed 6 months after placement with resolution of superior mesenteric artery syndrome symptoms.
C1 [Storm, Andrew C.; Mahmoud, Tala; Akiki, Karl; Law, Ryan J.] Mayo Clin, Rochester, MN 55902 USA.
C3 Mayo Clinic
RP Storm, AC (corresponding author), Mayo Clin, Rochester, MN 55902 USA.
EM Storm.Andrew@mayo.edu
RI Akiki, Karl/HPI-1002-2023
CR Bhenswala P, 2021, J CLIN GASTROENTEROL, V55, P641, DOI 10.1097/MCG.0000000000001566
Bronswijk M, 2021, ENDOSCOPY, V53, P204, DOI 10.1055/a-1190-3228
Chen YI, 2018, ENDOSC INT OPEN, V6, pE363, DOI 10.1055/s-0043-123468
Dawod E, 2018, TRANSL GASTROENT HEP, V3, DOI 10.21037/tgh.2018.11.03
Jue TL, 2021, GASTROINTEST ENDOSC, V93, P309, DOI 10.1016/j.gie.2020.07.063
Kouanda Abdul, 2021, VideoGIE, V6, P14, DOI [10.1016/j.vgie.2020.09.008, 10.1016/j.vgie.2020.09.008]
Mussetto A, 2018, ANN GASTROENTEROL, V31, P535, DOI 10.20524/aog.2018.0287
Perez-Miranda M, 2017, J CLIN GASTROENTEROL, V51, P896, DOI 10.1097/MCG.0000000000000887
Sinagra E, 2018, GASTROENT RES PRACT, V2018, DOI 10.1155/2018/1937416
Sobani ZA, 2020, AM J GASTROENTEROL, V115, P634, DOI 10.14309/ajg.0000000000000567
Welsch T, 2007, DIGEST SURG, V24, P149, DOI 10.1159/000102097
Xu MM, 2020, CLIN ENDOSC, V53, P94, DOI 10.5946/ce.2018.196
NR 12
TC 3
Z9 4
U1 0
U2 1
PU LIPPINCOTT WILLIAMS & WILKINS
PI PHILADELPHIA
PA TWO COMMERCE SQ, 2001 MARKET ST, PHILADELPHIA, PA 19103 USA
EI 2326-3253
J9 ACG CASE REP J
JI ACG Case Rep. J.
PD OCT
PY 2022
VL 9
IS 10
AR e00868
DI 10.14309/crj.0000000000000868
PG 3
WC Gastroenterology & Hepatology
WE Emerging Sources Citation Index (ESCI)
SC Gastroenterology & Hepatology

GA 5B7FA
UT WOS:000863733000004
PM 36212239
OA Green Submitted, gold
DA 2026-05-09
ER

PT J
AU Morita, S
Sugawara, S
Suda, T
Hoshi, T
Abe, S
Yagi, K
Terai, S
AF Morita, Shinichi
Sugawara, Shunsuke
Suda, Takeshi
Hoshi, Takahiro
Abe, Satoshi
Yagi, Kazuyoshi
Terai, Shuji
TI Conversion of percutaneous transhepatic biliary drainage to endoscopic ultrasound-guided biliary drainage
SO DEN OPEN
LA English
DT Article
DE adverse event; endoscopic ultrasound-guided biliary drainage; malignant biliary obstruction; percutaneous transhepatic biliary drainage; recurrent biliary obstruction; re-intervention
AB Introduction: Percutaneous transhepatic biliary drainage (PTBD) is a useful alternative treatment for malignant biliary obstruction (MBO) when patients have difficulty with endoscopic transpapillary drainage. We examined the feasibility of conversion of PTBD to endoscopic ultrasound-guided biliary drainage (EUS-BD) in patients with MBO unsuited for endoscopic transpapillary biliary drainage.

Methods: This retrospective study included patients who underwent conversion of PTBD to EUS-BD between March 2017 and December 2019. Eligible patients had unresectable MBO, required palliative biliary drainage, and were not suited for endoscopic transpapillary drainage. Initial PTBD had been performed for acute cholangitis or obstructive jaundice in all patients. EUS-BD was performed following improvements in cholangitis. Sixteen patients underwent conversion of PTBD to EUS-BD. We evaluated technical success, procedure time, clinical success (defined as subsequent external catheter removal), adverse events (AEs), time to recurrent biliary obstruction (TRBO), and re-intervention rates.

Results: Technical success was achieved in all patients (100%). The median procedure time was 45.0 minutes (interquartile range [IQR] 30.0-50.0 minutes). Clinical success was achieved in all patients (100%). There were mild early

AEs in two patients (12.5%) (acute cholangitis: 1, bile peritonitis: 1), which improved with antibiotic administration alone. Recurrent biliary obstruction (RBO) occurred in six patients (37.5%). Kaplan-Meier analysis revealed a 50% TRBO of 95 days (IQR 41-246 days). Endoscopic treatment was possible in all RBO cases, and repeat PTBD was not required.

Conclusions: Conversion of PTBD to EUS-BD for the management of MBO is both feasible and safe. This approach is expected to be widely practiced at centers with little experience in EUS-BD.
C1 [Morita, Shinichi; Suda, Takeshi; Hoshi, Takahiro; Abe, Satoshi; Yagi, Kazuyoshi] Niigata Univ Hosp, Dept Gastroenterol & Hepatol, Uonuma Inst Community Med, Niigata, Japan.

[Sugawara, Shunsuke] Natl Canc Ctr, Dept Diagnost Radiol, Tokyo, Japan.

[Terai, Shuji] Niigata Univ, Div Gastroenterol & Hepatol, Grad Sch Med & Dent Sci, Niigata, Japan.

C3 Niigata University; National Cancer Center - Japan; Niigata University

RP Morita, S (corresponding author), Niigata Univ Hosp, Uonuma Inst Community Med, Dept Gastroenterol & Hepatol, 4132 Urasa, Minamiuonuma City, Niigata 9497302, Japan.

EM m0riz0u@extra.ocn.ne.jp

OI Morita, Shinichi/0000-0003-1608-3827
CR Artifon ELA, 2012, J CLIN GASTROENTEROL, V46, P768, DOI 10.1097/MCG.0b013e31825f264c

Bapaye A, 2013, UNITED EUR GASTROENT, V1, P285, DOI 10.1177/2050640613490928

Baron TH, 2006, AM J GASTROENTEROL, V101, P892, DOI 10.1111/j.1572-0241.2006.00675.x

Chopin-Laly X, 2009, SURGERY, V145, P123, DOI 10.1016/j.surg.2008.07.019

Cotton PB, 2010, GASTROINTEST ENDOSC, V71, P446, DOI 10.1016/j.gie.2009.10.027

Fogel EL, 2005, ENDOSCOPY, V37, P139, DOI 10.1055/s-2004-826146

Hamada T, 2015, RADIOLOGY, V274, P605, DOI 10.1148/radiol.14140293

Hara K, 2016, WORLD J GASTROENTERO, V22, P1297, DOI 10.3748/wjg.v22.i3.1297

Inamdar S, 2016, JAMA ONCOL, V2, P112, DOI 10.1001/jamaoncol.2015.3670

Isayama H, 2015, Gastrointestinal Intervention, V4, P46, DOI 10.1016/j.gii.2015.03.002

Itonaga M, 2019, DIGEST ENDOSC, V31, P291, DOI 10.1111/den.13300

Khan MA, 2016, DIGEST DIS SCI, V61, P684, DOI 10.1007/s10620-015-3933-0

Lee TH, 2016, CLIN GASTROENTEROL H, V14, P1011, DOI 10.1016/j.cgh.2015.12.032

Minaga K, 2019, DIGEST ENDOSC, V31, P180, DOI 10.1111/den.13242

Minaga K, 2018, DIGEST ENDOSC, V30, P38, DOI 10.1111/den.12910

Moole H, 2017, MEDICINE, V96, DOI 10.1097/MD.0000000000005154

Morita S, 2019, DIGEST ENDOSC, V31, pE60, DOI 10.1111/den.13347

Nakai Y, 2017, ENDOSC ULTRASOUND, V6, P323, DOI 10.4103/2303-9027.199763

Ogura T, 2016, WORLD J GASTROENTERO, V22, P3945, DOI 10.3748/wjg.v22.i15.3945

Oh D, 2017, THER ADV GASTROENTER, V10, P42, DOI 10.1177/1756283X16671671

Paik WH, 2018, AM J GASTROENTEROL, V113, P987, DOI 10.1038/s41395-018-0122-8

Paik WH, 2017, ENDOSCOPY, V49, P544, DOI 10.1055/s-0043-102388

Sakakihara I, 2015, DIGEST ENDOSC, V27, P146, DOI 10.1111/den.12332

Sharaiha RZ, 2017, GASTROINTEST ENDOSC, V85, P904, DOI 10.1016/j.gie.2016.12.023

Sharaiha RZ, 2016, SURG ENDOSC, V30, P5500, DOI 10.1007/s00464-016-4913-y

SHEPHERD HA, 1988, BRIT J SURG, V75, P1166, DOI 10.1002/bjs.1800751207

Sugawara S, 2020, RADIOGRAPHICS, V40, P667, DOI 10.1148/rg.2020190158

van Delden OM, 2008, EUR RADIOL, V18, P448, DOI 10.1007/s00330-007-0796-6

Vila JJ, 2012, GASTROINTEST ENDOSC, V76, P1133, DOI 10.1016/j.gie.2012.08.001
Wang KX, 2016, GASTROINTEST ENDOSC, V83, P1218, DOI 10.1016/j.gie.2015.10.033
Zhao XQ, 2015, DIGEST ENDOSC, V27, P137, DOI 10.1111/den.12320
NR 31
TC 5
Z9 5
U1 0
U2 1
PU WILEY
PI HOBOKEN
PA 111 RIVER ST, HOBOKEN 07030-5774, NJ USA
SN 2692-4609
J9 DEN OPEN
JI DEN Open
PD APR
PY 2021
VL 1
IS 1
AR e6
DI 10.1002/deo2.6
PG 8
WC Gastroenterology & Hepatology
WE Emerging Sources Citation Index (ESCI)
SC Gastroenterology & Hepatology
GA VM8AO
UT WOS:001047018700005
PM 35310153
OA Green Submitted, Green Published, gold
DA 2026-05-09
ER

PT J
AU Amato, A
Sinagra, E
Celsa, C
Enea, M
Buda, A
Vieceli, F
Scaramella, L
Belletrutti, P
Fugazza, A
Cammà, C

Radaelli, F
Repici, A
Anderloni, A
AF Amato, Arnaldo
Sinagra, Emanuele
Celsa, Ciro
Enea, Marco
Buda, Andrea
Vieceli, Filippo
Scaramella, Lucia
Belletrutti, Paul
Fugazza, Alessandro
Camma, Calogero
Radaelli, Franco
Repici, Alessandro
Anderloni, Andrea
TI Efficacy of lumen-apposing metal stents or self-expandable metal stents
for endoscopic ultrasound-guided choledochoduodenostomy: a systematic
review and meta-analysis
SO ENDOSCOPY
LA English
DT Review
ID DISTAL BILIARY OBSTRUCTION; LONG-TERM OUTCOMES; COVERED METAL; ADVERSE
EVENTS; DRAINAGE; PLACEMENT; QUALITY; MULTICENTER; GRADE
AB Background Endoscopic ultrasound (EUS)-guided biliary drainage is becoming an option for palliation of malignant biliary obstruction. Lumen-apposing metal stents (LAMS) are replacing self-expandable metal stents (SEMS). The aim of this meta-analysis was to evaluate the efficacy and safety of LAMS and SEMS for EUS-guided choledochoduodenostomy (EUS-CDS).

Methods A meta-analysis was performed using PRISMA protocols. Electronic databases were searched for studies on EUS-CDS. The primary outcome was clinical success. Secondary outcomes were technical success, reintervention, and adverse events. We used the random effects model with the DerSimonian-Laird estimation, and the results were depicted using forest plots. Subgroup analyses were also performed with data stratified by selected variable.

Results Overall, 31 studies (820 patients) were included. The pooled rates of clinical and technical success were 93.6% (95% confidence interval [CI] 88.6%-96.5%) and 94.8% (95%CI 90.2%-97.3%) for LAMS, and 91.7% (95%CI 88.1%-94.2%) and 92.7% (95%CI 89.9%-94.9%) for SEMS, respectively. The pooled rates of adverse events were 17.1% (95%CI 12.5%-22.8%) for LAMS and 18.3% (95%CI 14.3%-23.0%) for SEMS. The pooled rates of reintervention were 10.9% (95%CI 7.7%-15.3%) for LAMS and 13.9% (95%CI 9.6%-19.7%) for SEMS.Subgroup analyses confirmed these results.

Conclusions This meta-analysis showed that LAMS and SEMS are comparable in terms of efficacy for EUS-CDS. Clinical and technical success, post-procedure adverse events, and reintervention rates were similar between LAMS and SEMS use; however, adverse events require further investigation.
C1 [Amato, Arnaldo; Scaramella, Lucia; Radaelli, Franco] Valduce Hosp, Div Digest Endoscopy & Gastroenterol, Via Dante 11, I-22100 Como, Italy.

[Sinagra, Emanuele] Fdn Ist G Giglio, Gastroenterol & Endoscopy Unit, Cefalu, Italy.
[Sinagra, Emanuele] Euro Mediterranean Inst Sci & Technol IEMEST, Sect Nutr, Palermo, Italy.
[Celsa, Ciro; Camma, Calogero] Univ Palermo, Sect Gastroenterol & Hepatol, Dept Hlth Promot Mother & Child Care Internal Med, PROMISE, Palermo, Italy.
[Celsa, Ciro] Univ Palermo, Doctoral Programme Expt Oncol & Surg, Dept Surg Oncol & Oral Sci, Palermo, Italy.
[Enea, Marco] Univ Palermo, Dept Hlth Promot Mother & Child Care Internal Med, PROMISE, Palermo, Italy.
[Buda, Andrea] Santa Maria del Prato Hosp, Gastroenterol Unit, Feltre, Italy.
[Vieceli, Filippo] Univ Verona, Gastroenterol & Endoscopy Unit, Pancreas Ctr, Verona, Italy.
[Scaramella, Lucia] Univ Milan, Dept Pathophysiol & Transplantat, Milan, Italy.
[Belletrutti, Paul; Repici, Alessandro] Humanitas Univ, Dept Biomed Sci, Milan, Italy.
[Fugazza, Alessandro; Repici, Alessandro; Anderloni, Andrea] Humanitas Clin & Res Ctr IRCCS, Digest Endoscopy Unit, Dept Gastroenterol, Rozzano, MI, Italy.
C3 University of Palermo; University of Palermo; University of Palermo;
University of Verona; University of Milan; Humanitas University
RP Amato, A (corresponding author), Valduce Hosp, Div Digest Endoscopy & Gastroenterol, Via Dante 11, I-22100 Como, Italy.
EM arnamato@gmail.com
RI Fugazza, Alessandro/ABG-9381-2021; ENEA, Marco/AAY-3373-2020; Amato, Arnaldo/ABC-5688-2020; Buda, Anea/O-4341-2019; Scaramella, Lucia/AGG-8396-2022; Repici, Alessandro/HFH-8162-2022; Anderloni, Andrea/ABE-9139-2020; Celsa, Ciro/ACF-6633-2022; Sinagra, Emanuele/C-9336-2014
OI Fugazza, Alessandro/0000-0003-0485-4903; ENEA, Marco/0000-0002-9281-5746; Amato, Arnaldo/0000-0002-4397-4142; Buda, Anea/0000-0002-3099-8612; Scaramella, Lucia/0000-0002-9460-3296; Repici, Alessandro/0000-0002-1621-6450;
CR Amano M, 2017, J GASTROEN HEPATOL, V32, P716, DOI 10.1111/jgh.13489
Anderloni A, 2019, GASTROINTEST ENDOSC, V89, P69, DOI [10.1016/j.gie.2018.04.1746, 10.1016/j.gie.2018.08.047]
Artifon ELA, 2015, ENDOSC ULTRASOUND, V4, P235, DOI 10.4103/2303-9027.163010
Artifon ELA, 2012, J CLIN GASTROENTEROL, V46, P768, DOI 10.1097/MCG.0b013e31825f264c
Bang JY, 2018, GASTROINTEST ENDOSC, V88, P9, DOI 10.1016/j.gie.2018.03.012
Binmoeller KF, 2011, ENDOSCOPY, V43, P337, DOI 10.1055/s-0030-1256127
Cho DH, 2017, GASTROINTEST ENDOSC, V85, P1067, DOI 10.1016/j.gie.2016.09.010
Cotton PB, 2010, GASTROINTEST ENDOSC, V71, P446, DOI 10.1016/j.gie.2009.10.027
DERSIMONIAN R, 1986, CONTROL CLIN TRIALS, V7, P177, DOI 10.1016/0197-2456(86)90046-2
El Chafic AH, 2019, DIGEST DIS SCI, V64, P3321, DOI 10.1007/s10620-019-05688-2
Fabbri C, 2011, ENDOSCOPY, V43, P438, DOI 10.1055/s-0030-1256097
Giovannini M, 2001, ENDOSCOPY, V33, P898, DOI 10.1055/s-2001-17324
Guo JT, 2016, GASTROENT RES PRACT, V2016, DOI 10.1155/2016/9469472
Guyatt GH, 2008, BRIT MED J, V336, P924, DOI 10.1136/bmj.39489.470347.AD
Guyatt GH, 2011, J CLIN EPIDEMIOL, V64, P1294, DOI 10.1016/j.jclinepi.2011.03.017

Hara K, 2013, ENDOSCOPY, V45, P392, DOI 10.1055/s-0032-1326076
Higgins JPT, 2003, BMJ-BRIT MED J, V327, P557, DOI 10.1136/bmj.327.7414.557
Higgins JPT, 2009, J R STAT SOC A STAT, V172, P137, DOI 10.1111/j.1467-985X.2008.00552.x
Huang P, 2017, SURG LAPARO ENDO PER, V27, pE127, DOI 10.1097/SLE.0000000000000485
IntHout J, 2016, BMJ OPEN, V6, DOI 10.1136/bmjopen-2015-010247
Itonaga M, 2019, DIGEST ENDOSC, V31, P291, DOI 10.1111/den.13300
Jacques J, 2020, GASTROINTEST ENDOSC, V92, P134, DOI 10.1016/j.gie.2020.01.055
Jacques J, 2019, ENDOSCOPY, V51, P540, DOI 10.1055/a-0735-9137
Kanwal F, 2012, CLIN GASTROENTEROL H, V10, P1184, DOI 10.1016/j.cgh.2012.09.019
Khashab MA, 2016, ENDOSC INT OPEN, V4, pE487, DOI 10.1055/s-0042-102648
Khashab MA, 2013, GASTROINTEST ENDOSC, V78, P734, DOI 10.1016/j.gie.2013.05.013
Kim TH, 2012, WORLD J GASTROENTERO, V18, P2526, DOI 10.3748/wjg.v18.i20.2526
Kunda R, 2016, SURG ENDOSC, V30, P5002, DOI 10.1007/s00464-016-4845-6
Lee TH, 2016, CLIN GASTROENTEROL H, V14, P1011, DOI 10.1016/j.cgh.2015.12.032
Lu L, 2017, GASTROENT RES PRACT, V2017, DOI 10.1155/2017/6284094
Minaga K, 2019, DIGEST ENDOSC, V31, P575, DOI 10.1111/den.13406
Mohan BP, 2019, J CLIN GASTROENTEROL, V53, P243, DOI 10.1097/MCG.0000000000001167
Moher D, 2009, BMJ-BRIT MED J, V339, DOI 10.1136/bmj.b2535
Nakai Y, 2019, CLIN ENDOSC, V52, P220
Nennstiel S, 2015, J CLIN GASTROENTEROL, V49, P764, DOI 10.1097/MCG.0000000000000275
Ogura T, 2016, ENDOSCOPY, V48, P156, DOI 10.1055/s-0034-1392859
Paik WH, 2018, AM J GASTROENTEROL, V113, P987, DOI 10.1038/s41395-018-0122-8
Park DH, 2011, GASTROINTEST ENDOSC, V74, P1276, DOI 10.1016/j.gie.2011.07.054
Park DH, 2009, AM J GASTROENTEROL, V104, P2168, DOI 10.1038/ajg.2009.254
Park JK, 2018, GASTROINTEST ENDOSC, V88, P277, DOI 10.1016/j.gie.2018.03.015
Poincloux L, 2015, ENDOSCOPY, V47, P794, DOI [10.1055/s-0035-1545399, 10.1055/s-0034-1391988]
Rai P, 2018, ENDOSC INT OPEN, V6, pE67, DOI 10.1055/s-0043-120664
Riley Richard D., 2011, BMJ-BRIT MED J, V342, DOI 10.1136/bmj.d549
Soderlund C, 2006, GASTROINTEST ENDOSC, V63, P986, DOI 10.1016/j.gie.2005.11.052
Song TJ, 2012, WORLD J GASTROENTERO, V18, P4435, DOI 10.3748/wjg.v18.i32.4435
Stang A, 2010, EUR J EPIDEMIOL, V25, P603, DOI 10.1007/s10654-010-9491-z
Tsuchiya T, 2018, GASTROINTEST ENDOSC, V87, P1138, DOI 10.1016/j.gie.2017.08.017
Uemura RS, 2018, J CLIN GASTROENTEROL, V52, P123, DOI 10.1097/MCG.0000000000000948
Alvarez-Sánchez MV, 2018, J GASTROINTEST SURG, V22, P1213, DOI 10.1007/s11605-018-3731-z
Weber A, 2009, PANCREAS, V38, pE7, DOI 10.1097/MPA.0b013e3181870ab8
NR 50
TC 64
Z9 72
U1 0
U2 2
PU GEORG THIEME VERLAG KG
PI STUTTGART

PA Oswald-Hesse-Strasse 50, D-70469 STUTTGART, GERMANY
SN 0013-726X
EI 1438-8812
J9 ENDOSCOPY
JI Endoscopy
PD OCT
PY 2021
VL 53
IS 10
BP 1037
EP 1047
DI 10.1055/a-1324-7919
EA MAR 2021
PG 11
WC Gastroenterology & Hepatology; Surgery
WE Science Citation Index Expanded (SCI-EXPANDED)
SC Gastroenterology & Hepatology; Surgery
GA UW4LT
UT WOS:000624291700001
PM 33246343
DA 2026-05-09
ER

PT J
AU Huang, P
Zhang, H
Zhang, XF
Lv, W
Fan, Z
AF Huang, Ping
Zhang, Hao
Zhang, Xiao-Feng
Lv, Wen
Fan, Zhen
TI Application and Value of Endoscopic Ultrasonography Guided Biliary
Interventional Therapy in Patients With Biliary Obstruction and
Surgically Altered Anatomy
SO SURGICAL LAPAROSCOPY ENDOSCOPY & PERCUTANEOUS TECHNIQUES
LA English
DT Article
DE biliary obstruction; surgically altered anatomies; endoscopic
ultrasound-guided biliary drainage; percutaneous transhepatic biliary

drainage (PTBD)
ID ERCP; DRAINAGE; BALLOON
AB Aim: The aim of this study was to evaluate the efficacy and safety of endoscopic ultrasound-guided biliary drainage (EUS-BD) in patients with biliary obstruction and surgically altered anatomy. Patients and Methods: This was a retrospective study. We collected data from 60 patients with biliary obstruction and surgically altered anatomies who undergone EUS-BD (group A, 33 cases) and percutaneous transhepatic biliary drainage (PTBD) (group B, 27 cases) after unsuccessful endoscopic retrograde cholangiopancreatography from January 2016 to January 2018. The operation success rate, the clinical success rate, complications, hospital stay were observed. Results: In group A, 31 cases of 33 patients were successfully operated and placed stents or drainage tube by endoscopic ultrasound puncture [endoscopic ultrasonography (EUS) guided rendezvous 8, EUS-guided hepaticogastrostomy 14, EUS-guided choledochoduodenostomy 11], 28 had a significant decrease in jaundice. The complications rate was 9.1% (biliary bleeding 2; acute cholangitis 1). There was statistically significant difference in the complications [9.09% (3/33) vs. 33.33% (9/27), chi(2)=5.45,P<0.05] and length of hospital stay (14.45 +/- 5.78 vs. 18.52 +/- 8.64 d,t=10.03,P<0.01) between group A and group B. There was not statistical significance in the technical and clinical success rate between 2 groups [93.94% (31/33) vs. 92.60% (25/27), 84.85% (28/33) vs. 85.19% (23/27), chi(2)(1)=0.10, chi(2)(2)=0.11,P>0.05]. Conclusion: EUS-BD may be the first choice for patients with biliary obstruction and surgically altered anatomy after a failed endoscopic retrograde cholangiography in centers with expertise in EUS-BD procedures.
C1 [Huang, Ping; Zhang, Xiao-Feng; Lv, Wen; Fan, Zhen] Zhejiang Univ, Affiliated HangZhou Peoples Hosp 1, Dept Gastroenterol, Sch Med, Hangzhou, Peoples R China.
[Zhang, Hao] Hangzhou Xixi Hosp, Dept Gastroenterol, Hangzhou, Zhejiang, Peoples R China.
C3 Zhejiang University
RP Zhang, XF (corresponding author), 261 Huan Sha Rd, Hangzhou 310006, Zhejiang, Peoples R China.
EM zxf.837@tom.com
RI Zhang, Hao/AAW-6783-2020
FU Science and Technology Development Program of Hangzhou [20140733Q11]
FX Supported by Science and Technology Development Program of Hangzhou
(20140733Q11).
CR Artifon ELA, 2012, J CLIN GASTROENTEROL, V46, P768, DOI 10.1097/MCG.0b013e31825f264c
Attasaranya S, 2012, GASTROENT RES PRACT, V2012, P680
Bapaye A, 2013, UNITED EUR GASTROENT, V1, P285, DOI 10.1177/2050640613490928
Baron Todd H Sr, 2013, Gastrointest Endosc Clin N Am, V23, P295, DOI 10.1016/j.giec.2013.01.001
Bertin PM, 2011, SURG ENDOSC, V25, P2592, DOI 10.1007/s00464-011-1593-5
Costamagna G, 2013, ANN GASTROENTEROL, V26, P37
Desai S V., 2011, Gastrointest Endosc, V73, pAB122, DOI [10.1016/j.gie.2011.03.039, DOI 10.1016/J.GIE.2011.03.039]
Devière J, 2014, GASTROENTEROLOGY, V147, P385, DOI 10.1053/j.gastro.2014.04.043
Giovannini M, 2001, ENDOSCOPY, V33, P898, DOI 10.1055/s-2001-17324
Hintze RE, 1997, ENDOSCOPY, V29, P74, DOI 10.1055/s-2007-1004078
Imazu H, 2012, ENDOSCOPY, V44, P99, DOI 10.1055/s-0031-1291445
Iwashita T, 2014, CLIN J GASTROENTEROL, V7, P94, DOI 10.1007/s12328-014-0467-5
Iwashita T, 2013, DIGEST DIS SCI, V58, P2417, DOI 10.1007/s10620-013-2645-6
Kamiya S, 2004, ANN SURG, V239, P510, DOI 10.1097/01.sla.0000118594.23874.89

Khashab MA, 2015, DIGEST DIS SCI, V60, P557, DOI 10.1007/s10620-014-3300-6
Kühn JP, 2010, AM J ROENTGENOL, V195, P851, DOI 10.2214/AJR.09.3461
Lopes TL, 2009, GASTROINTEST ENDOSC, V70, P1254, DOI 10.1016/j.gie.2009.07.035
Park DH, 2013, GASTROINTEST ENDOSC, V78, P91, DOI 10.1016/j.gie.2013.01.042
Ping H, 2016, CHINESE J HEPATOBILI, V22, P464
Schreiner MA, 2012, GASTROINTEST ENDOSC, V75, P748, DOI 10.1016/j.gie.2011.11.019
Shah RJ, 2013, GASTROINTEST ENDOSC, V77, P593, DOI 10.1016/j.gie.2012.10.015
Skinner M, 2014, ENDOSCOPY, V46, P560, DOI 10.1055/s-0034-1365698
Wright BE, 2002, GASTROINTEST ENDOSC, V56, P225, DOI 10.1067/mge.2002.126136
NR 23
TC 3
Z9 3
U1 0
U2 1
PU LIPPINCOTT WILLIAMS & WILKINS
PI PHILADELPHIA
PA TWO COMMERCE SQ, 2001 MARKET ST, PHILADELPHIA, PA 19103 USA
SN 1530-4515
EI 1534-4908
J9 SURG LAPARO ENDO PER
JI Surg. Laparosc. Endosc. Pct. Tech.
PD OCT
PY 2020
VL 30
IS 5
BP 454
EP 458
DI 10.1097/SLE.0000000000000813
PG 5
WC Surgery
WE Science Citation Index Expanded (SCI-EXPANDED)
SC Surgery
GA OA9IS
UT WOS:000578093700014
PM 32487860
DA 2026-05-09
ER

PT J
AU Rotman, SR
Kahaleh, M
AF Rotman, Stephen R.

Kahaleh, Michel
TI Pancreatic Fluid Collection Drainage by Endoscopic Ultrasound: New Perspectives
SO ENDOSCOPIC ULTRASOUND
LA English
DT Review
DE endoscopic ultrasound; pancreatic fluid collection; pancreatic pseudocyst; non-bulging pancreatic fluid collection
ID TRANSMURAL DRAINAGE; NATURAL-HISTORY; PSEUDOCYSTS; MANAGEMENT; EUS; LESIONS; NECROSECTOMY; EXPERIENCE; ALGORITHM; DIAGNOSIS
AB Since the introduction of endoscopic ultrasonography (BUS), many centers have utilized this imaging modality for transmural pancreatic fluid collection (PFC) drainage. The expanded use of BUS has resulted in increased safety and efficacy of endoscopic PFC drainage. The major procedural steps include BUS-guided transgastric or transduodenal fistula creation into the PFC, and stent placement or nasocystic drain deployment to decompress the collection. In this and other applications, BUS has become a major therapeutic advancement in the field of endoscopy and has figured in myriad diagnostic applications. Recent research indicates a number of situations in which BUS-guided PFC drainage is appropriate. These include unusual location of the collection, small window of entry, non-bulging collections, coagulopathy, intervening varices, or failed conventional transmural drainage. In this study, we discuss the BUS-guided technique and review current literatures.
C1 [Rotman, Stephen R.; Kahaleh, Michel] Presbyterian Hosp, Div Gastroenterol & Hepatol, Weill Cornell Med Coll, New York, NY 10021 USA.
C3 Cornell University; Weill Cornell Medicine; NewYork-Presbyterian Hospital
RP Kahaleh, M (corresponding author), Presbyterian Hosp, Div Gastroenterol & Hepatol, Weill Cornell Med Coll, New York, NY 10021 USA.
EM mkahaleh@gmail.com
RI Kahaleh, Michel/A-9408-2011
OI Kahaleh, Michel/0000-0003-0836-6114
CR ADAMS DB, 1990, ARCH SURG-CHICAGO, V125, P1554
AHEARNE PM, 1992, AM J SURG, V163, P111, DOI 10.1016/0002-9610(92)90262-P
Andren-Sandberg Ake, 2004, JOP, V5, P64
Antillon MR, 2009, GASTROINTEST ENDOSC, V69, P178, DOI 10.1016/j.gie.2008.03.1066
Antillon MR, 2006, GASTROINTEST ENDOSC, V63, P797, DOI 10.1016/j.gie.2005.10.025
Arvanitakis M, 2005, GASTROINTEST ENDOSC, V62, P143, DOI 10.1016/S0016-5107(05)01627-5
Arvanitakis M, 2007, GASTROINTEST ENDOSC, V65, P609, DOI 10.1016/j.gie.2006.06.083
Azar RR, 2006, GASTROINTEST ENDOSC, V63, P688, DOI 10.1016/j.gie.2005.10.032
Baillie J, 2004, GASTROINTEST ENDOSC, V59, P873, DOI 10.1016/S0016-5107(04)00354-2
Bakker OJ, 2012, JAMA-J AM MED ASSOC, V307, P1053, DOI 10.1001/jama.2012.276
Baron TH, 1996, GASTROENTEROLOGY, V111, P755, DOI 10.1053/gast.1996.v111.pm8780582
Baron TH, 2002, GASTROINTEST ENDOSC, V56, P7, DOI 10.1067/mge.2002.125106
Baron TH, 1997, AM J MED, V102, P555
Baron TH, 2008, J GASTROINTEST SURG, V12, P369, DOI 10.1007/s11605-007-0334-5

Barthet M, 2008, GASTROINTEST ENDOSC, V67, P245, DOI 10.1016/j.gie.2007.06.014
Binmoeller K F, 1995, Gastrointest Endosc Clin N Am, V5, P805
BINMOELLER KF, 1995, GASTROINTEST ENDOSC, V42, P219, DOI 10.1016/S0016-5107(95)70095-1
Boerma D, 1999, DIGEST SURG, V16, P501, DOI 10.1159/000018776
BRADLEY EL, 1993, ARCH SURG-CHICAGO, V128, P586
BRADLEY EL, 1993, SURG GYNECOL OBSTET, V177, P215
BRADLEY EL, 1979, AM J SURG, V137, P135, DOI 10.1016/0002-9610(79)90024-2
Cahen D, 2005, ENDOSCOPY, V37, P977, DOI 10.1055/s-2005-870336
Cortes ES, 2002, GASTROINTEST ENDOSC, V56, P380, DOI 10.1067/mge.2002.126883
Delhaye Myriam, 2003, Gastrointest Endosc Clin N Am, V13, P717, DOI 10.1016/S1052-5157(03)00070-9
Giovannini M, 1998, GASTROINTEST ENDOSC, V48, P200, DOI 10.1016/S0016-5107(98)70165-8
Giovannini M, 2001, ENDOSCOPY, V33, P473, DOI 10.1055/s-2001-14967
Giovannini M, 2007, TECH GASTROINTEST EN, V9, P32, DOI 10.1016/j.tgie.2006.11.013
Gouyon B, 1997, GUT, V41, P821, DOI 10.1136/gut.41.6.821
Hookey LC, 2006, GASTROINTEST ENDOSC, V63, P635, DOI 10.1016/j.gie.2005.06.028
HOWELL DA, 1993, GASTROINTEST ENDOSC, V39, P693, DOI 10.1016/S0016-5107(93)70225-4
Jacobson BC, 2005, GASTROINTEST ENDOSC, V61, P363, DOI 10.1016/S0016-5107(04)02779-8
Kahaleh M, 2006, ENDOSCOPY, V38, P355, DOI 10.1055/s-2006-925249
Klöppel G, 2000, SEMIN DIAGN PATHOL, V17, P7
Krüger M, 2006, GASTROINTEST ENDOSC, V63, P409, DOI 10.1016/j.gie.2005.11.047
Lopes CV, 2007, SCAND J GASTROENTERO, V42, P524, DOI 10.1080/00365520601065093
Neff R, 2001, SURG CLIN N AM, V81, P399, DOI 10.1016/S0039-6109(05)70127-4
Puri R, 2012, J GASTROEN HEPATOL, V2012, DOI 10.1111/j.14401746.2012.07089.x
Seifert H, 2000, ENDOSCOPY, V32, P255, DOI 10.1055/s-2000-93
Seifert H, 2001, ENDOSCOPY, V33, P1022, DOI 10.1055/s-2001-18927
Talreja JP, 2008, GASTROINTEST ENDOSC, V68, P1199, DOI 10.1016/j.gie.2008.06.015
VANSONNENBERG E, 1989, RADIOLOGY, V170, P757, DOI 10.1148/radiology.170.3.2644662
Varadarajulu S, 2008, GASTROINTEST ENDOSC, V68, P1102, DOI 10.1016/j.gie.2008.04.028
Varadarajulu S, 2007, GASTROINTEST ENDOSC, V66, P1107, DOI 10.1016/j.gie.2007.03.1027
Varadarajulu S, 2011, J GASTROINTEST SURG, V15, P2080, DOI 10.1007/s11605-011-1621-8
Varadarajulu S, 2011, J GASTROEN HEPATOL, V26, P1504, DOI 10.1111/j.1440-1746.2011.06771.x
WARSHAW AL, 1985, ANN SURG, V202, P720, DOI 10.1097/00000658-198512000-00010
Wiersema MJ, 1996, GASTROINTEST ENDOSC, V44, P614, DOI 10.1016/S0016-5107(96)70022-6
Will U, 2011, ULTRASCHALL MED, V32, P176, DOI 10.1055/s-0031-1273347
Yeo CJ, 1997, ANN SURG, V226, P248, DOI 10.1097/00000658-199709000-00004
YEO CJ, 1990, SURG GYNECOL OBSTET, V170, P411
Zheng MW, 2011, HEPATO-GASTROENTEROL, V58, P1106, DOI 10.5754/hge11059
NR 51
TC 8
Z9 10
U1 0
U2 3

PU WOLTERS KLUWER MEDKNOW PUBLICATIONS
PI MUMBAI
PA WOLTERS KLUWER INDIA PVT LTD , A-202, 2ND FLR, QUBE, C T S NO 1498A-2
VILLAGE MAROL, ANDHERI EAST, MUMBAI, 400059, INDIA
SN 2303-9027
EI 2226-7190
J9 ENDOSC ULTRASOUND
JI Endosc. Ultrasound
PD AUG
PY 2012
VL 1
IS 2
BP 61
EP 68
DI 10.7178/eus.02.002
PG 8
WC Gastroenterology & Hepatology
WE Science Citation Index Expanded (SCI-EXPANDED)
SC Gastroenterology & Hepatology
GA V31PG
UT WOS:000208894900002
PM 24949339
OA hybrid
DA 2026-05-09
ER

PT J
AU Ramesh, J
Bang, JY
Trevino, J
Varadarajulu, S
AF Ramesh, Jayapal
Bang, Ji Y.
Trevino, Jessica
Varadarajulu, Shyam
TI Endoscopic Ultrasound-guided Drainage of Pancreatic Fluid Collections in
Children
SO JOURNAL OF PEDIATRIC GASTROENTEROLOGY AND NUTRITION
LA English
DT Article
DE cystogastrostomy; endoscopic drainage; endoscopic ultrasound;
endosonography; pancreatic fluid collections; pseudocyst drainage

ID TERM-FOLLOW-UP; PROSPECTIVE RANDOMIZED-TRIAL; TRANSMURAL DRAINAGE; PSEUDOCYST DRAINAGE; ECHO ENDOSCOPE; COMPARING EUS; MANAGEMENT; PREDICTORS; ABSCESSES; EFFICACY

AB Background and Objective: Although endoscopic ultrasound (EUS)-guided drainage of pancreatic fluid collections (PFCs) has evolved as the standard of care in adults, its role as a single-step drainage modality in children is unclear. The aim of the present study was to evaluate the efficacy and safety of single-step EUS-guided drainage of PFCs in children.

Methods: This is a retrospective study of all of the children who underwent single-step EUS-guided drainage of PFCs during a 4-year period at 1 institution. An endoscopic retrograde cholangiopancreatography was attempted before EUS-guided drainage to evaluate the pancreatic duct and bridge any ductal disruption.

Results: A total of 7 children (4 boys; mean age 8.4 years [standard deviation 2.1]) underwent EUS-guided drainage of PFCs. The etiology was blunt abdominal trauma in 5, hereditary pancreatitis in 1, and idiopathic pancreatitis in 1. Both technical and treatment success rates were 100% with median procedural duration of 12 minutes (interquartile range 12-20 minutes). Two patients underwent repeat EUS-guided drainage due to lack of adequate resolution of PFC on follow-up computed tomography. There were no immediate or delayed complications. At a median follow-up of 1033 days (interquartile range 193-1167 days), all of the children were doing well with no PFC recurrence.

Conclusions: Single-step EUS-guided drainage of PFC in children is technically feasible, safe, clinically effective, and when available, should be the first-line treatment modality.

C1 [Ramesh, Jayapal; Bang, Ji Y.; Trevino, Jessica; Varadarajulu, Shyam] Univ Alabama Birmingham, Div Gastroenterol & Hepatol, Birmingham, AL 35294 USA.

C3 University of Alabama System; University of Alabama Birmingham

RP Ramesh, J (corresponding author), Univ Alabama Birmingham, Div Gastroenterol & Hepatol, BDB 389,1808 7th Ave S, Birmingham, AL 35294 USA.

EM j1ramesh@gmail.com

CR Antillon MR, 2006, GASTROINTEST ENDOSC, V63, P797, DOI 10.1016/j.gie.2005.10.025
Baron TH, 2000, GASTROINTEST ENDOSC, V52, P545, DOI 10.1067/mge.2000.108928
BEJANIN H, 1993, GASTROEN CLIN BIOL, V17, P804
Benifla M, 2003, J CLIN GASTROENTEROL, V37, P169, DOI 10.1097/00004836-200308000-00015
BURNWEIT C, 1990, J TRAUMA, V30, P1273, DOI 10.1097/00005373-199010000-00013
Buxbaum JL, 2011, J PEDIATR GASTR NUTR, V52, P482, DOI 10.1097/MPG.0b013e3181fdef4f
CREMER M, 1989, GASTROINTEST ENDOSC, V35, P1, DOI 10.1016/S0016-5107(89)72677-8
FORD EG, 1990, AM SURGEON, V56, P384
Giovannini M, 2001, ENDOSCOPY, V33, P473, DOI 10.1055/s-2001-14967
Jazrawi SF, 2011, DIGEST DIS SCI, V56, P902, DOI 10.1007/s10620-010-1350-y
Kahaleh M, 2006, ENDOSCOPY, V38, P355, DOI 10.1055/s-2006-925249
Kimble RM, 1999, J PEDIATR SURG, V34, P1518, DOI 10.1016/S0022-3468(99)90116-1
Krüger M, 2006, GASTROINTEST ENDOSC, V63, P409, DOI 10.1016/j.gie.2005.11.047
Makin E, 2012, J PEDIAT GASTROENTER
Mattix KD, 2007, J PEDIATR SURG, V42, P340, DOI 10.1016/j.jpedsurg.2006.10.006
Mönkemüller KE, 1998, GASTROINTEST ENDOSC, V48, P195, DOI 10.1016/S0016-5107(98)70164-6
Park DH, 2009, ENDOSCOPY, V41, P842, DOI 10.1055/s-0029-1215133
Ratan SK, 2006, PEDIATR SURG INT, V22, P532, DOI 10.1007/s00383-006-1674-z
SAHEL J, 1987, PANCREAS, V2, P447, DOI 10.1097/00006676-198707000-00012

Seifert H, 2001, ENDOSCOPY, V33, P1022, DOI 10.1055/s-2001-18927
Sharma SS, 2008, J PEDIATR SURG, V43, P1636, DOI 10.1016/j.jpedsurg.2008.01.026
Sharma SS, 2002, ENDOSCOPY, V34, P203, DOI 10.1055/s-2002-20292
SMITS ME, 1995, GASTROINTEST ENDOSC, V42, P202, DOI 10.1016/S0016-5107(95)70092-7
Sriram PVJ, 2005, ENDOSCOPY, V37, P231, DOI 10.1055/s-2005-860997
Teh SH, 2006, J PEDIATR SURG, V41, P1889, DOI 10.1016/j.jpedsurg.2006.06.017
Trevino JM, 2010, J GASTROEN HEPATOL, V25, P526, DOI 10.1111/j.1440-1746.2009.06109.x
Varadarajulu S, 2005, GASTROINTEST ENDOSC, V61, P568, DOI 10.1016/S0016-5107(04)02832-9
Varadarajulu S, 2008, GASTROINTEST ENDOSC, V68, P1102, DOI 10.1016/j.gie.2008.04.028
Varadarajulu S, 2007, GASTROINTEST ENDOSC, V66, P1107, DOI 10.1016/j.gie.2007.03.1027
Varadarajulu S, 2011, AM SURGEON, V77, P1650
Varadarajulu S, 2011, J GASTROINTEST SURG, V15, P2080, DOI 10.1007/s11605-011-1621-8
Varadarajulu S, 2010, GASTROINTEST ENDOSC, V71, pAB116, DOI 10.1016/j.gie.2010.03.047
Yoder SM, 2009, J LAPAROENDOSC ADV S, V19, pS37, DOI 10.1089/lap.2008.0124.supp
NR 33
TC 27
Z9 35
U1 1
U2 1
PU LIPPINCOTT WILLIAMS & WILKINS
PI PHILADELPHIA
PA TWO COMMERCE SQ, 2001 MARKET ST, PHILADELPHIA, PA 19103 USA
SN 0277-2116
EI 1536-4801
J9 J PEDIATR GASTR NUTR
JI J. Pediatr. Gastroenterol. Nutr.
PD JAN
PY 2013
VL 56
IS 1
BP 30
EP 35
DI 10.1097/MPG.0b013e318267c113
PG 6
WC Gastroenterology & Hepatology; Nutrition & Dietetics; Pediatrics
WE Science Citation Index Expanded (SCI-EXPANDED)
SC Gastroenterology & Hepatology; Nutrition & Dietetics; Pediatrics
GA 063UV
UT WOS:000313028400014
PM 22785412
DA 2026-05-09
ER

PT J

AU Jo, HG
Amarbat, B
Jeong, JW
Song, HY
Song, SR
Kim, TH
AF Jo, Hoon-Gil
Amarbat, Baatarnum
Jeong, Jin-Woo
Song, Hyo-Yeop
Song, Seung-Ryel
Kim, Tae-Hyeon

TI Could Transgastric Endoscopic Ultrasound-Guided Aspiration Alone Be
Effective for the Treatment of Pancreatic Abscesses?

SO CLINICAL ENDOSCOPY
LA English
DT Article
DE Endoscopic drainage; Endoscopic ultrasound-guided fine needle
aspiration; Pancreatic abscess

AB Drainage of pancreatic abscesses is required for effective control of sepsis. Endoscopic ultrasound (EUS)-guided endoscopic drainage is less invasive than surgery and prevents local complications related to percutaneous drainage. Endoscopic drainage with stent placement in the uncinate process of the pancreas is a technically difficult procedure. We report a case of pancreatic abscess treated by repeated EUS-guided aspiration and intravenous antibiotics without an indwelling drainage catheter or surgical intervention.

C1 [Jo, Hoon-Gil; Jeong, Jin-Woo; Song, Hyo-Yeop; Song, Seung-Ryel; Kim, Tae-Hyeon] Wonkwang Univ, Dept Internal Med, Sch Med, 895 Muwang Ro, Iksan 570974, South Korea.
[Amarbat, Baatarnum] Natl Canc Ctr Mongolia, GI Endoscopy Ctr, Ulaanbaatar, Mongolia.
C3 Wonkwang University
RP Kim, TH (corresponding author), Wonkwang Univ, Dept Internal Med, Sch Med, 895 Muwang Ro, Iksan 570974, South Korea.
EM kth@wonkwang.ac.kr
RI Kim, TaeHyeon/JOK-7558-2023

FU Wonkwang University
FX This paper was supported by 2013 research funds from Wonkwang
University.

CR Baillie J, 2004, GASTROINTEST ENDOSC, V59, P873, DOI 10.1016/S0016-5107(04)00354-2
Jenssen C, 2009, BEST PRACT RES CL GA, V23, P743, DOI 10.1016/j.bpg.2009.05.006
Piraka C, 2009, GASTROINTEST ENDOSC, V70, P786, DOI 10.1016/j.gie.2009.04.049
Varadarajulu S, 2008, GASTROINTEST ENDOSC, V68, P649, DOI 10.1016/j.gie.2008.02.057
Varadarajulu S, 2011, J GASTROEN HEPATOL, V26, P1504, DOI 10.1111/j.1440-1746.2011.06771.x

NR 5
TC 1
Z9 2
U1 0
U2 0
PU KOREAN SOC GASTROINTESTINAL ENDOSCOPY
PI SEOUL
PA 2003 LG PALACE, 165-8 DONGGYO-DONG, MAPO-GU, SEOUL, 121-754, SOUTH KOREA
SN 2234-2400
EI 2234-2443
J9 CLIN ENDOSC
JI Clin. Endosc.
PD JUL
PY 2015
VL 48
IS 4
BP 345
EP 347
DI 10.5946/ce.2015.48.4.345
PG 3
WC Gastroenterology & Hepatology
WE Emerging Sources Citation Index (ESCI)
SC Gastroenterology & Hepatology
GA VA5MB
UT WOS:000410074000015
PM 26240812
OA gold
DA 2026-05-09
ER

PT J
AU Brugge, WR
AF Brugge, WR
TI Approaches to the drainage of pancreatic pseudocysts
SO CURRENT OPINION IN GASTROENTEROLOGY
LA English
DT Article
DE pancreatic pseudocyst; cyst-gastrostomy; external drainage; endoscopic
cyst-gastrostomy; pancreatitis
ID TERM FOLLOW-UP; TRANSMURAL DRAINAGE; ENDOSCOPIC DRAINAGE; ECHO
ENDOSCOPE; MANAGEMENT; PERFORMANCE; LESIONS
AB Purpose of review

Review the current methods for the drainage of pancreatic pseudocysts, focusing on the recent developments in the new methods of endoscopic drainage.
Recent findings
Pancreatic pseudocysts are collections of inflammatory fluid associated with acute and chronic pancreatitis. A leak in the pancreatic ductal system is most commonly the source of the fluid accumulation in these inflammatory cavities adjacent to the pancreas. Although most pseudocysts are not symptomatic and many resolve spontaneously over time, drainage of pseudocysts is occasionally required. There are several different approaches to the drainage: surgical, radiologic, and endoscopic. Surgical drainage is accomplished using the creation of a large anastomosis between the gastrointestinal tract and the pseudocyst cavity. Radiologically, pseudocysts are drained externally using a percutaneous, transabdominal drainage catheter. Endoscopy has offered the most recent advance, using the placement of transgastric or transduodenal stents. Most recently, therapeutic endoscopic ultrasound scopes have been used to introduce large stents that provide drainage into the upper gastrointestinal tract.
Summary
Of the three methods for the drainage of a pancreatic pseudocyst, only the endoscopic approach can provide minimally invasive internal drainage.
C1 Massachusetts Gen Hosp, Gastrointestinal Unit, Boston, MA 02114 USA.
C3 Harvard University; Harvard University Medical Affiliates; Massachusetts
General Hospital
RP Brugge, WR (corresponding author), Massachusetts Gen Hosp, Gastrointestinal Unit, 55 Fruit St, Boston, MA 02114 USA.
EM WBRUGGE@PARTNERS.Org
CR Bhattacharya D, 2003, SURG LAPARO ENDO PER, V13, P141, DOI 10.1097/00129689-200306000-00001
Boerma D, 2000, BRIT J SURG, V87, P1506, DOI 10.1046/j.1365-2168.2000.01573.x
Brugge WR, 2000, TECH GASTROINTEST EN, V2, P149
Cantasdemir M, 2003, SOUTH MED J, V96, P136, DOI 10.1097/01.SMJ.0000050682.65270.38
Cohen-Scali F, 2003, RADIOLOGY, V228, P727, DOI 10.1148/radiol.2283020973
De Palma GD, 2002, HEPATO-GASTROENTEROL, V49, P1113
Fockens P, 1997, GASTROINTEST ENDOSC, V46, P412, DOI 10.1016/S0016-5107(97)70033-6
FROESCHLE G, 1993, ACTA CHIR BELG, P102
Frossard JL, 2003, AM J GASTROENTEROL, V98, P1516, DOI 10.1016/S0002-9270(03)00359-9
Giovannini M, 2001, ENDOSCOPY, V33, P473, DOI 10.1055/s-2001-14967
Harewood GC, 2003, GASTROINTEST ENDOSC, V58, P230, DOI 10.1067/mge.2003.346
Mithofer K, 1997, WORLD J SURG, V21, P162, DOI 10.1007/s002689900209
Norton ID, 2001, MAYO CLIN PROC, V76, P794
Parks RW, 2000, ANN ROY COLL SURG, V82, P383
Seifert H, 2000, LANCET, V356, P653, DOI 10.1016/S0140-6736(00)02611-8
Seifert H, 2000, ENDOSCOPY, V32, P255, DOI 10.1055/s-2000-93
Sharma SS, 2002, ENDOSCOPY, V34, P203, DOI 10.1055/s-2002-20292
NR 17
TC 25
Z9 31
U1 0

U2 0
PU LIPPINCOTT WILLIAMS & WILKINS
PI PHILADELPHIA
PA TWO COMMERCE SQ, 2001 MARKET ST, PHILADELPHIA, PA 19103 USA
SN 0267-1379
EI 1531-7056
J9 CURR OPIN GASTROEN
JI Curr. Opin. Gastroenterol.
PD SEP
PY 2004
VL 20
IS 5
BP 488
EP 492
DI 10.1097/00001574-200409000-00012
PG 5
WC Gastroenterology & Hepatology
WE Science Citation Index Expanded (SCI-EXPANDED)
SC Gastroenterology & Hepatology
GA 848GF
UT WOS:000223454900011
PM 15689684
DA 2026-05-09
ER

PT J
AU Hindryckx, P
Degroote, H
Tate, DJ
Deprez, PH
AF Hindryckx, Pieter
Degroote, Helena
Tate, David J.
Deprez, Pierre H.
TI Endoscopic ultrasound-guided drainage of the biliary system: Techniques,
indications and future perspectives
SO WORLD JOURNAL OF GASTROINTESTINAL ENDOSCOPY
LA English
DT Review
DE Endoscopic ultrasound; Endoscopic retrograde cholangiopancreaticography;
Biliary drainage; Rendez-vous; Hepaticogastrostomy;
Choledochoduodenostomy

ID COVERED METAL STENT; BILE-DUCT OBSTRUCTION; FAILED ERCP; CHOLEDOCHODUODENOSTOMY; HEPATICOGASTROSTOMY; PLACEMENT; MULTICENTER; ACCESS; LONG; COMPLICATIONS
[truncated: 7,571,242 more chars]
