# Supplementary material for: Interventional Endoscopic Ultrasound in Gastroenterology: A Comprehensive Bibliometric Analysis (2001–2024)
Source: Clin Pract. 2026 Aug 20;16(8):153. doi: 10.3390/clinpract16080153 (PMC13511046; doi:10.3390/clinpract16080153)
Supplement: Supplementary file 1 [file clinpract-16-00153-s001.zip › Supplementary File S2.pdf]

Supplementary File S2. Custom thesaurus used for keyword unification

VOSviewer thesaurus file (standard two-column "label / replace by" format). Each entry maps an orthographic, spacing, hyphenation, or abbreviational variant onto a single canonical term; mappings are between variants of one concept, not between distinct clinical entities. Terms are given in lower case, consistent with the VOSviewer export.

label	replace by
endoscopic ultrasonography	eus
endosonography	eus
endoscopic ultrasound (eus)	eus
endoscopic ultrasonography (eus)	eus
endoscopic retrograde cholangiopancreatography	ercp
retrograde cholangiopancreatography	ercp
cholangiopancreatography	ercp
endoscopic retrograde	ercp
lumen-apposing metal stent	lams
lumen-apposing metal stents	lams
lumen apposing metal stent	lams
lumen apposing metal stents	lams
apposing metal stent	lams
apposing metal stents	lams
eus-guided biliary drainage	eus-bd
endoscopic ultrasound-guided biliary drainage	eus-bd
guided biliary drainage	eus-bd
eus-guided hepaticogastrostomy	eus-hgs
endoscopic ultrasound-guided hepaticogastrostomy	eus-hgs
hepaticogastrostomy	eus-hgs
eus-guided choledochoduodenostomy	eus-cds
endoscopic ultrasound-guided choledochoduodenostomy	eus-cds
choledochoduodenostomy	eus-cds
eus-guided gastroenterostomy	eus-ge
endoscopic ultrasound-guided gastroenterostomy	eus-ge
gastroenterostomy	eus-ge
gastrojejunostomy	eus-ge
endoscopic ultrasound-guided gallbladder drainage	eus-gbd
guided gallbladder drainage	eus-gbd
gallbladder drainage	eus-gbd
guided radiofrequency ablation	eus-rfa
radiofrequency ablation	eus-rfa
eus-guided fna	eus-fna
fine-needle-aspiration	eus-fna
fna	eus-fna
ganglia neurolysis	celiac plexus neurolysis
neurolysis	celiac plexus neurolysis
celiac plexus block	celiac plexus neurolysis
pancreatic fluid collections	pancreatic fluid collection
fluid collections	pancreatic fluid collection
peripancreatic fluid collections	pancreatic fluid collection
pseudocysts	pseudocyst
pancreatic pseudocyst	pseudocyst
pancreatic pseudocysts	pseudocyst
pseudocyst drainage	pseudocyst
walled-off pancreatic necrosis	walled-off necrosis
acute necrotizing pancreatitis	necrotizing pancreatitis
infected necrotizing pancreatitis	necrotizing pancreatitis
pancreatic-cancer	pancreatic cancer

pancreatic neoplasms	pancreatic cancer
unresectable pancreatic-cancer	pancreatic cancer
metal stents	metal stent
expandable metal stents	metal stent
expandable metal stent	metal stent
expanding metal stents	metal stent
interventional endoscopic ultrasound	interventional eus
therapeutic endoscopic ultrasound	interventional eus
intervention eus	interventional eus
metaanalysis	meta-analysis
complication	complications
adverse events	complications
acute cholecystitis	cholecystitis
common bile-duct	bile duct
stents	stent
plastic stent	plastic stents
endoscopic necrosectomy	necrosectomy
prospective randomized-trial	randomized-trial
guided drainage	eus-guided drainage
duct drainage	eus-guided drainage
strictures	stricture
choledocholithiasis	bile-duct stones
endoscopic ultrasound	eus
percutaneous cholecystostomy	cholecystostomy
hepatocellular-carcinoma	hepatocellular carcinoma
pancreatic-duct drainage	eus-pd
neuroendocrine tumors	neuroendocrine tumor
cystic neoplasms	cystic neoplasm
walled off necrosis	walled-off necrosis
ultrasound-guided drainage	drainage
obstructive jaundice	biliary obstruction
distal biliary obstruction	biliary obstruction
malignant biliary	biliary obstruction
malignant biliary obstruction	biliary obstruction
percutaneous transhepatic biliary drainage	percutaneous drainage
acute pancreatitis	pancreatitis
ethanol-lavage	ethanol ablation
duodenal obstruction	gastric outlet obstruction
common bile duct	bile duct
bile-duct obstruction	biliary obstruction
rv	eus-rv
rendezvous	eus-rv
choledochojejunostomy	eus-hjs
hepaticojejunostomy	eus-hjs
eus-guided gallbladder drainage	eus-gbd
injection	fine-needle injection
antegrade	eus-ag
endoscopic ultrasound-guided antegrade	eus-ag
eus-guided antegrade	eus-ag
pancreatic duct drainage	eus-pd
pancreatography	eus-pd
pancreaticogastrostomy	eus-pd
fine needle aspiration	eus-fna
fine-needle aspiration	eus-fna
pancreatic-fluid collections	pancreatic fluid collection
pancreatic cyst	pancreatic cysts
gastric fundal varices	gastric varices
