# Supplementary material for: Interventional Endoscopic Ultrasound in Gastroenterology: A Comprehensive Bibliometric Analysis (2001–2024)
Source: Clin Pract. 2026 Aug 20;16(8):153. doi: 10.3390/clinpract16080153 (PMC13511046; doi:10.3390/clinpract16080153)
Supplement: Supplementary file 1 [file clinpract-16-00153-s001.zip › Supplementary File S3.pdf]

Supplementary File S3. Full list of excluded terms, annotated by exclusion category

Of the 165 candidate keywords meeting the minimum co-occurrence threshold of 15, 123 were excluded prior to network construction and 42 were retained. Exclusion was performed independently by two gastroenterologists (K.T. and T.I.), each blinded to the other's assignments; observed inter-rater agreement was 96.4% (159/165 terms), and the six discordant terms were resolved by adjudication with a third investigator (H.K.). Terms denoting a specific anatomical target, disease entity, device, or procedure were retained irrespective of frequency.

Categories were prespecified and applied in the order listed below. Where a term satisfied more than one category, it was assigned to the lowest-numbered applicable category.

Category I — Terms intrinsic to the search strategy

Rationale: These terms are present in a substantial proportion of the corpus by construction, since they appear in the Boolean query itself or denote the modality under study. They therefore carry no discriminative value for distinguishing thematic sub-domains and would appear as artefactually dominant hub nodes obscuring genuine cluster structure. The term "ablation" is assigned to this category because the search string includes the proximity construct "guided ablat*"; the procedure-specific term "eus-rfa" was retained in its place.

n = 27

Term	Term	Term
eus	ercp	endoscopy
ultrasound	ultrasonography	echo endoscope
interventional eus	drainage	transmural drainage
biliary drainage	endoscopic drainage	guided transmural drainage
endoscopic ultrasound-guided drainage	percutaneous drainage	stent
stent placement	placement	access
cannulation	endoscopic sphincterotomy	sphincterotomy
cholangiography	cystogastrostomy	cholecystostomy
intervention	endoscopic management	ablation

Category II — Study-design and publication-type descriptors

Rationale: These terms denote research methodology or publication format rather than clinical or technical subject matter. Their inclusion would generate a spurious cluster organised around study design that cuts across, and therefore obscures, the thematic structure the analysis is intended to recover.

n = 14

Term	Term	Term
multicenter	international multicenter	randomized-trial
trial	meta-analysis	case series
consensus	guidelines	european-society
classification	feasibility	learning-curve
natural-history	group document evaluation	

Category III — Generic outcome and non-specific clinical descriptors

Rationale: These terms are applied indiscriminately across all sub-domains of the corpus and do not localise a research theme. They function as high-frequency, low-specificity nodes that inflate network density without contributing interpretable structure.

n = 33

Term	Term	Term
management	outcomes	clinical-outcomes
treatment outcomes	long-term outcomes	safety
efficacy	complications	therapy
risk	impact	quality
quality-of-life	survival	mortality
diagnosis	differential-diagnosis	follow-up
term-follow-up	disease	palliation
palliative treatment	high-risk patients	benign
lesions	tumors	neoplasms
cysts	carcinoma	adenocarcinoma
cancer	obstruction	biopsy

Category IV — Anatomical and organ-level terms lacking procedural specificity

Rationale: These terms denote broad anatomical regions or structures that are common to multiple clusters and therefore do not discriminate between them. Anatomically specific terms tied to a distinct procedure or disease entity (e.g., "walled-off necrosis," "gastric outlet obstruction," "pancreatic fluid collection") were retained.

n = 21

Term	Term	Term
pancreas	pancreatic necrosis	necrosis
necrotizing pancreatitis	chronic pancreatitis	liver
gallbladder	bile duct	bile-duct
biliary	biliary tract	biliary obstruction
biliary stricture	stricture	duct
stones	abscesses	jaundice
pain	block	cystic neoplasm

Category V — Terms denoting non-EUS comparator or adjacent procedures

Rationale: These terms designate surgical, oncological, or bariatric interventions that appear in the corpus principally as comparators within outcome studies rather than as I-EUS research themes in their own right. Retaining them would introduce nodes representing the control arm of the literature rather than its subject.

n = 18

Term	Term	Term
surgery	cholecystectomy	laparoscopic cholecystectomy

Term	Term	Term
pancreaticoduodenectomy	resection	surgical gastrojejunostomy
laparoscopic gastrojejunostomy	bypass	gastric bypass
y gastric bypass	roux-en-y gastric bypass	bariatric surgery
radiotherapy	radiation-therapy	photodynamic therapy
gemcitabine	ethanol	ethanol ablation

Category VI — Truncated, malformed, or topically unrelated index artefacts

Rationale: These entries arise from indexing errors, hyphenation-dependent string truncation, or homonymy with unrelated fields. They do not correspond to coherent clinical concepts within this corpus.

n = 10

Term	Nature of the artefact
long	Truncation of "long-term follow-up"
edge	Truncation, non-interpretable in isolation
up approach	Truncation of "step-up approach" (the complete term was retained)
videos	From the phrase "(with videos)" in article titles
ct	Computed tomography, an imaging comparator outside the analytic scope
computed-tomography	As above
spinal-cord infarction	Rare adverse-event report; not an I-EUS research theme
sump syndrome	Post-surgical entity unrelated to interventional EUS
enteroscopy-assisted ercp	Non-EUS technique captured incidentally
surgically altered anatomy	Patient-population descriptor common to multiple clusters

Note on adjudicated terms retained in the network

Of the six discordant terms, two were retained in the network on adjudication and therefore form no part of the 123 excluded terms: "gastric varices," initially flagged by one rater as topically remote from interventional EUS but retained as a specific disease entity for which EUS-guided therapy is an established indication; and "pancreatic cysts," distinguished from the excluded generic terms "cysts" and "cystic neoplasm" as a specific pancreatic entity constituting a recognised indication for EUS-guided intervention. Both satisfy the retention rule stated above and appear as nodes in Figures 3–5. The remaining four discordant terms were confirmed as excluded and appear in their categories above.

The remaining four discordant terms were confirmed as excluded and appear in their adjudicated categories above.

Summary

Category	Terms excluded
I. Intrinsic to the search strategy	27
II. Study-design and publication-type descriptors	14
III. Generic outcome and non-specific clinical descriptors	33
IV. Anatomical terms lacking procedural specificity	21
V. Non-EUS comparator or adjacent procedures	18

**Category**	**Terms excluded**
VI. Truncated, malformed, or topically unrelated artefacts	10
Total excluded	123
Candidate keywords meeting threshold	165
Keywords retained for network construction	42
